# *In vivo* CRISPR/Cas9 Screening Reveals that UBE2L3 Modulates Autophagic Flux through TSC2 Ubiquitination and Potentiates PD-1 Blockade in Triple-Negative Breast Cancer

**DOI:** 10.7150/ijbs.124937

**Published:** 2026-02-18

**Authors:** Jian Xu, Ling Cheng, Sien Ma, Chen Gan, Jiaying Chai, Xinyi Zheng, Longyu Hu, Meiwen Ling, Mingjun Zhang, Bao Zhao, Huaidong Cheng

**Affiliations:** 1 Department of Oncology, Shenzhen Hospital of Southern Medical University, Shenzhen, Guangdong, China.; 2 Shenzhen Clinical Medical School of Southern Medical University, China.; 3 Department of Surgical Oncology, The First Affiliated Hospital of Bengbu Medical University, Bengbu, Anhui, China.; 4 Department of Oncology, Second Affiliated Hospital of Anhui Medical University, Hefei, Anhui, China.; 5 Institute of Health and Medicine, Hefei Comprehensive National Science Center, Hefei, Anhui, China.; 6 Department of Traditional Chinese Medicine, The People's Hospital of Pingshan Shenzhen (Pingshan Hospital of Southern Medical University), Shenzhen, Guangdong, China.

**Keywords:** autophagy, CRISPR/Cas9, PD-1, TNBC, ubiquitination

## Abstract

Triple-negative breast cancer (TNBC), a distinct breast cancer subtype, poses significant challenges to conventional therapeutic approaches, and effective targeted therapies are limited. CRISPR/Cas9 library screening has demonstrated unprecedented efficiency and revolutionary potential in the identification of therapeutic targets. In this study, we performed *In vivo* CRISPR/Cas9 library screening and identified the E2 ubiquitin-conjugating enzyme UBE2L3 as a critical regulatory factor in the progression of TNBC. Loss of UBE2L3 restricted tumor cell growth by modulating autophagy in TNBC cells. Mechanistically, UBE2L3 downregulation led to increased tuberous sclerosis complex 2 (TSC2) expression, suppressing mTOR activity and altering autophagic processes in tumor cells. This regulation was mediated through the interaction between UBE2L3 and the E3 ubiquitin ligase SMURF2, which together control TSC2 protein ubiquitination and degradation. Autophagy and the tumor microenvironment are closely associated, and we observed that UBE2L3 knockdown in TNBC tumors significantly increased CD8+ T lymphocyte infiltration and enhanced tumor sensitivity to anti-PD-1 therapy. Collectively, our findings provide a theoretical foundation for considering UBE2L3 as a potential therapeutic target in TNBC.

## Introduction

Nearly 2.3 million new cases of breast cancer were diagnosed globally in 2022. Breast cancer is the most common malignancy among women, accounting for 11.6% of all newly diagnosed cancers worldwide. With continuous advancements in diagnostic, therapeutic, and screening methods, the mortality rate has been steadily declining. Nevertheless, breast cancer remains the fourth leading cause of cancer-related deaths globally, responsible for 6.9% of all cancer deaths 1. Among the various subtypes, triple-negative breast cancer (TNBC) is one of the most challenging forms of breast cancer to treat and represents 15-20% of all breast cancer cases. TNBC is characterized by the absence of key actionable drivers found in other subtypes, such as estrogen receptor positivity or HER2 overexpression. As a result, TNBC is associated with lower survival rates, earlier development of distant metastases, and higher recurrence rates than other subtypes, indicating the limited efficacy of conventional treatment approaches 2-4. Furthermore, the pronounced heterogeneity of this aggressive tumor complicates therapeutic strategies. With the evolution of treatment paradigms, the identification of effective therapeutic targets for precision medicine is providing new avenues for TNBC therapy 5. Therefore, elucidating the key regulatory molecules involved in TNBC progression is a prerequisite for the development of TNBC-specific targeted therapies.

Targeted gene editing technologies have demonstrated remarkable potential in biological research. Among these, CRISPR/Cas9 library screening—based on functional selection and high-throughput sequencing analysis—has played an increasingly important role in life sciences, particularly in the identification of novel tumor targets 6. Notably, *In vivo* CRISPR/Cas9 screening enables accurate modeling of the interactions between tumors and their hosts, providing a closer approximation of tumor growth dynamics and the surrounding microenvironment *In vivo*. This approach facilitates the identification of critical regulatory factors involved in tumor progression 7.

Autophagy is a crucial intracellular degradation pathway that is highly conserved across species. This process maintains cellular homeostasis through protein degradation and the removal of damaged organelles 8, 9. The role of autophagy in cancer remains controversial, as it can exert either tumor-promoting or tumor-suppressing effects, depending on the stage of tumor development 10. Nevertheless, it is well established that alterations in autophagy are closely associated with tumorigenesis and tumor progression 11. Breast cancer was one of the first malignancies to be linked to autophagy in scientific research. Beclin 1, an essential autophagy gene, significantly suppresses breast cancer initiation, and monoallelic deletion of Beclin 1 is observed in 40-75% of breast cancer patients 12. Conversely, some studies have demonstrated that autophagy can promote tumor cell survival, with late-stage tumors often exhibiting elevated autophagic activity 13, 14. The relationship between TNBC and autophagy has garnered increasing attention. Modulating the autophagic process—either by inhibition or activation—may represent a promising novel strategy for treating TNBC.

Protein ubiquitination is catalyzed by a series of enzymes, primarily including E1 ubiquitin-activating enzymes, E2 ubiquitin-conjugating enzymes, and E3 ubiquitin ligases 15. UBE2L3, which encodes a protein containing 153 amino acids, is one of the most abundant E2 enzymes in mammalian cell lines and is highly conserved across species 16, 17. UBE2L3 helps to modulate cell function by regulating protein stability, and its relevance to tumorigenesis has been increasingly recognized. In hepatocellular carcinoma, UBE2L3 expression influences the stability of GSK3β through ubiquitination, modulating p65 phosphorylation and ultimately regulating the growth of liver cancer cells 18. In lung cancer, UBE2L3 knockdown suppresses the growth of non-small-cell lung cancer (NSCLC) cells. UBE2L3 interacts with Skp2 to promote the ubiquitination and proteasomal degradation of p27^kip1^, and the combined expression of UBE2L3 and p27^kip1^ may serve as an effective prognostic indicator for NSCLC 19. However, the role of UBE2L3 in TNBC remains unclear.

Tuberous sclerosis complex 2 (TSC2) is a well-established tumor suppressor gene and the causative gene for the autosomal dominant disorder tuberous sclerosis complex. In addition, TSC2 regulates cellular metabolism and autophagy by inhibiting the mechanistic target of rapamycin (mTOR) signaling pathway. mTOR activation is dependent on Rheb-GTP. TSC2 loss or inactivation blocks the conversion of Rheb-GTP to Rheb-GDP, resulting in elevated Rheb-GTP levels and consequent activation of mTORC1 20, 21. As a critical tumor suppressor, TSC2 is mutated in a variety of tissues 22-24. One study found that among 5040 breast cancer patients, the prevalence of TSC2 germline mutations was 0.6% for pathogenic variants and 0.5% for variants of uncertain significance 25. In TNBC, DAPK1 phosphorylates TSC2 at Ser939, inhibiting TSC1/TSC2 complex formation and modulating mTOR signaling to regulate tumor growth 26.

In this study, we used an *In vivo* genome-wide CRISPR/Cas9 screening approach to identify essential genes required for the growth and development of 4T1 cells. Our screening and validation experiments demonstrated that UBE2L3 plays a pivotal role in the proliferation of 4T1 cells by modulating autophagic flux. Mechanistically, UBE2L3 interacts with SMURF2 and controls the ubiquitination of TSC2, thereby influencing mTOR activity and regulating autophagic processes. Furthermore, UBE2L3 knockdown in TNBC cells markedly enhanced sensitivity to anti-PD-1 therapy. Collectively, our findings suggest that UBE2L3 represents a potential therapeutic target for refractory TNBC, offering new strategies for clinical treatment.

## Materials and Methods

### CRISPR/Cas9 Library Screening and Data Analysis

**Figure 1:**
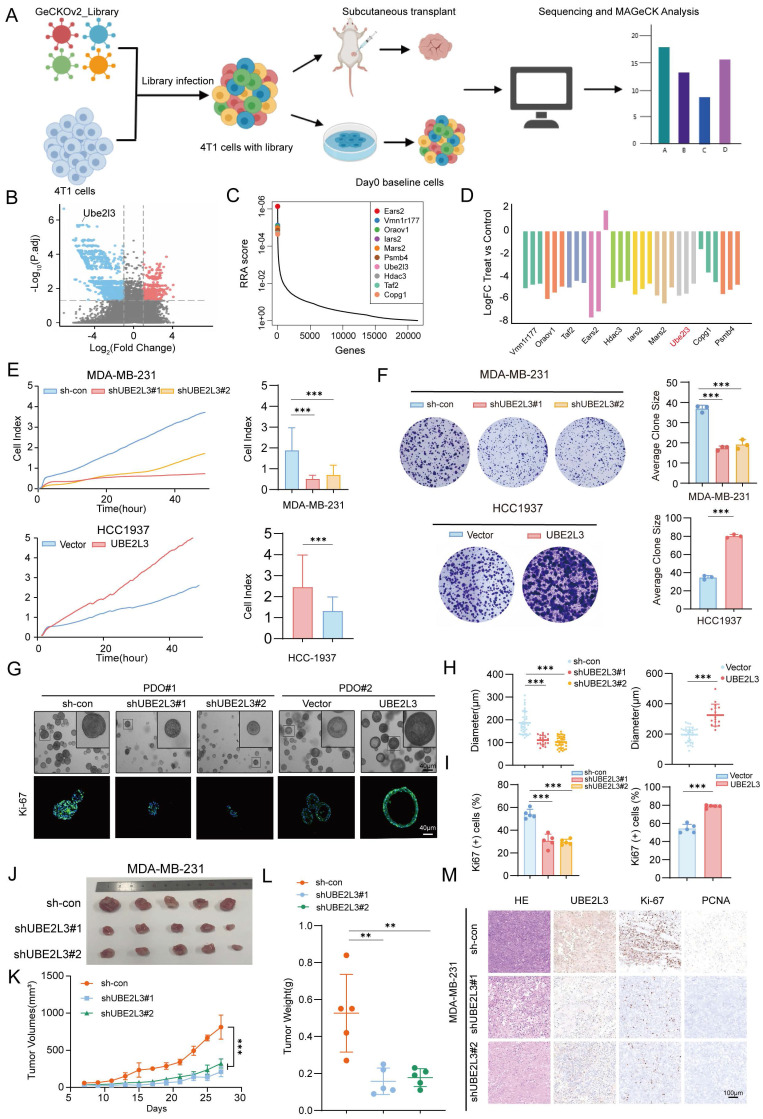
*In vivo* genome-wide CRISPR/Cas9 screening identifies UBE2L3 as a key regulator of TNBC cell growth. (**A**) Schematic diagram of the *In vivo* genome-wide CRISPR/Cas9 screening strategy. (**B**) Volcano plot of positive and negative selection hits. (**C, D**) Visualization of differentially expressed genes. (**E**) Real-time cell analysis (RTCA) of cell proliferation rates in triple-negative breast cancer (TNBC) cells. (**F**) Effect of UBE2L3 knockdown and overexpression on colony formation ability, as determined by colony formation assays. (**G-I**) Representative images of organoid diameter and Ki-67 immunofluorescence intensity following UBE2L3 knockdown or overexpression. Scale bar, 40 μm. Subcutaneous injection of UBE2L3-knockdown MDA-MB-231 cells into nude mice to evaluate tumor growth *In vivo*: (**J**) Representative images of tumors, (**K**) Tumor volume, (**L**) Tumor weight, and (**M**) Representative H&E staining and IHC images showing UBE2L3, Ki-67, and PCNA expression in xenograft tissues. Scale bar, 100 μm. Error bars represent mean ± SD. ***p < 0.001, **p < 0.01.

The Mouse_GeCKOv2_Library_A, containing 67,405 sgRNAs targeting 20,611 protein-coding genes, was obtained from Suzhou Jinweizhi Biotechnology Co., Ltd. Fig. 1A illustrates the workflow of the CRISPR/Cas9 library screening process. 4T1 cells were infected with the CRISPR library at a multiplicity of infection of 0.3. Based on preliminary experiments, an infection scale of 5 × 10^7^ cells was used to ensure 500-fold library coverage. Forty-eight hours after infection, cells were subjected to selection with 9 µg/mL puromycin for 7 days to eliminate uninfected cells. The surviving cells were then divided into four groups: one group was collected immediately to represent the baseline (day 0) sample for gDNA extraction, while the remaining three groups were subcutaneously injected into the mammary fat pads of 6-week-old female BALB/c mice to establish transplantation tumor models. After 3 weeks of growth, subcutaneous tumors were harvested, and tumor DNA was extracted. gDNA extraction and sequencing were performed by Suzhou Jinweizhi Biotechnology Co., Ltd. Data analysis was conducted using the RRA algorithm implemented in MAGeCK.

### Cell Lines and Cell Culture

All cell lines were maintained in a humidified incubator at 37°C with 5% CO_2_. The human embryonic kidney (HEK) cell line 293T and the mouse breast cancer cell line 4T1 were provided by the Institute of Health Sciences, Hefei Comprehensive National Science Center (Hefei, China). The human breast cancer cell lines MDA-MB-231 and HCC-1937 were purchased from Shanghai Zhong Qiao Xin Zhou Biotechnology Co., Ltd. (Shanghai, China). All cell lines were authenticated by short tandem repeat DNA profiling and underwent routine quality control assessments, including morphological evaluation, cell viability analysis, and mycoplasma testing. 4T1 and HCC-1937 cells were cultured in RPMI 1640 medium (KeyGEN, Nanjing, China) supplemented with 10% fetal bovine serum (FBS; Gibco). Other cell lines were cultured in DMEM (KeyGEN, Nanjing, China) with the same supplementation. All culture media were supplemented with 100 U/mL penicillin and 100 µg/mL streptomycin (KeyGEN, Nanjing, China).

### Animal Experiments

All animal experiments in this study were approved by the Institutional Animal Care and Use Committee of the Institute of Health Sciences, Hefei Comprehensive National Science Center, and were conducted in accordance with established guidelines (Approval No. IHM-AP-2025-052). Female BALB/c nude mice and BALB/c mice, aged 6-8 weeks, were purchased from Jiangsu Jicui Yaokang Biotechnology Co., Ltd. All animals were housed under specific pathogen-free conditions.

During CRISPR/Cas9 screening, 4T1 cells infected with the sgRNA library were subcutaneously injected into the mammary fat pads of BALB/c mice at a dose of 5 × 10^6^ cells per mouse. Tumor growth was monitored every 3 days. Two weeks after injection, the mice were euthanized, and tumors were excised for further sequencing analysis.

For single-gene validation, gene-edited MDA-MB-231 cells and corresponding control cells were subcutaneously injected into the mammary fat pads of BALB/c nude mice at a dose of 1 × 10^6^ cells per mouse. Tumor growth was assessed every 3 days. After 4 weeks, the mice were sacrificed, and tumors were dissected, weighed, photographed, and subjected to IHC staining.

In therapeutic experiments, BALB/c mice were randomly divided into four groups. Gene-edited 4T1 cells and control cells were subcutaneously injected into the mammary fat pads of mice in each group at a dose of 5 × 10^5^ cells per mouse. Seven days after cell inoculation, each group received intraperitoneal injections of either 100 μg anti-PD-1 antibody (BioXCell, BE0146) or IgG control (BioXCell, BE0089) every 3 days for a total of three doses, according to the experimental protocol. The mice were monitored daily. Two days after the final injection, the animals were euthanized, and tumors were collected for further analysis.

### Lentivirus and Plasmid Infection

Lentiviral vectors encoding target genes, short hairpin RNAs, and empty vectors were obtained from Tsingke Biotech (Beijing, China). To establish stable UBE2L3- or TSC2-knockdown cell lines, target cells were infected with lentiviral particles, and the culture medium was replaced with fresh medium 48 h after infection. Cells were then selected through incubation in puromycin-containing medium for 7 days to obtain stable cell lines. Western blot analysis was used to validate the efficiency of gene knockdown. The procedure for establishing stable UBE2L3-overexpressing cell lines was performed similarly.

All relevant plasmids were obtained from MiaolingBio (Wuhan, China). Plasmid transfection into 293T cells was performed using Lipofectamine® 3000 (Thermo Fisher Scientific), and western blot analysis was used to validate transfection efficiency.

### Western Blotting

Radioimmunoprecipitation assay buffer was used to extract cellular proteins. The BCA Protein Assay Kit (Yeasen Biotechnology) was used to determine protein concentrations. Equal amounts of protein were separated using SDS-polyacrylamide gel electrophoresis (SDS-PAGE) and transferred onto polyvinylidene difluoride (PVDF) membranes. Then, the membranes were blocked with non-fat milk at room temperature for 2 h and then incubated overnight at 4°C with the appropriate primary antibodies. After three washes with TBST, the membranes were incubated with secondary antibodies at room temperature for 2 h. Finally, the PVDF membranes were exposed to chemiluminescent substrate, and signals were detected using an Amersham Imager 600 (Cytiva).

### RTCA

RTCA was performed using the xCELLigence system (Roche, Basel, Switzerland) to monitor cell proliferation. Gene-edited and control cells were seeded at a density of 5 × 10^3^ cells per well in 16-well E-plates. Cell index values, reflecting cell viability in each well, were automatically monitored and recorded every 5 min using the system's dedicated software, with continuous monitoring conducted for 48 h.

### Colony Formation Assay

Cells were seeded in triplicate at a density of 1000 cells per 6 cm dish and cultured for 2 weeks. After removing the culture medium, the cells were washed with phosphate-buffered saline (PBS) and fixed with 4% paraformaldehyde for 15 min. Subsequently, colonies were stained with 0.1% crystal violet (Beyotime) for 30 min, rinsed, air-dried, and photographed. ImageJ software was used to quantify colony numbers.

### Organoid Culture

Fresh tumor tissues from TNBC patients were washed three times with ice-cold PBS and then minced on ice into fragments approximately 3 mm^3^ in size. Tissue fragments were enzymatically digested with trypsin (Bio-risezi, Shanghai, China) for approximately 30 min. Once the mixture became turbid, it was passed through a 100 µm cell strainer and centrifuged at 200 × *g* for 5 min to collect cell clusters. The cell clusters were then washed three times with PBS, centrifuging for 5 min each time to remove residual digestive enzymes. After the final wash, the cell clusters were embedded in Matrigel (Corning). Once solidified, the Matrigel-cell mixture was overlaid with organoid culture medium (Bio-risezi, Shanghai, China) and incubated at 37 °C in a CO_2_ incubator. For lentiviral infection, organoids were first dissociated into cell clusters, which were then suspended in medium containing lentiviral particles. The suspension was centrifuged at 700 × *g* and 25 °C for 90 min (spinfection) and then incubated at 37 °C for 4 h. Subsequently, the cell clusters were centrifuged at 300 × *g* for 5 min and embedded in Matrigel. Western blot analysis was used to validate infection efficiency.

### ROS Detection

After gene-edited MDA-MB-231 cells and their corresponding control cells were cultured in 6-well plates for 48 hours, the old culture medium was discarded. The cells were washed twice with PBS, followed by the addition of 1 mL of DCFH-DA probe working solution (Beyotime Biotechnology, S1105S) to each well. The plates were then incubated for 20 minutes at 37 °C in a humidified incubator with 5% CO₂ in the dark. After incubation, the probe working solution was removed, and the cells were washed three times with pre-chilled PBS. Subsequently, the cells were detached with trypsin, gently pipetted to form a single-cell suspension, and transferred into 1.5 mL centrifuge tubes. The cell suspensions were centrifuged at 350 × g for 5 minutes at 4 °C. The supernatant was discarded, and the cell pellets were resuspended in PBS buffer. The intracellular ROS levels were analyzed using a NovoExpress flow cytometer (Agilent Technologies) and FlowJo software (Version 10).

### Apoptosis Assay

Gene-edited MDA-MB-231 cells and control cells were cultured in six-well plates for 48 h, after which apoptosis was assessed using the Annexin V-APC Apoptosis Detection Kit (Yeasen Biotechnology, 40304ES50). Both floating and adherent cells were collected and washed three times with PBS. The cells were then stained with annexin V and propidium iodide (PI) in binding buffer for 30 min in the dark. Apoptosis was analyzed using a NovoExpress flow cytometer (Agilent) and FlowJo software (v10).

### TEM

Gene-edited cells and control cells were seeded in 6 cm culture dishes and cultured to 70%-80% confluence. The cells were rinsed three times with pre-chilled PBS, then scraped off the dishes using a cell scraper. The resulting cell suspension was centrifuged at 800 × g for 5 min at 4 °C to harvest cell pellets, which were subsequently fixed in 2.5% glutaraldehyde at 4 °C for 2 h. After that, the samples were subjected to embedding, sectioning and staining procedures. Imaging was performed using a transmission electron microscope (TEM), and all images were acquired at an accelerating voltage of 200 kV. Low-magnification images (×10000) were captured to observe the overall morphology of the samples, while high-magnification images (×25000) were taken to visualize detailed ultrastructural characteristics. TEM imaging was performed by Xinle Biotechnology Co., Ltd. (Hefei, China).

### mCherry-EGFP-LC3 Assay

Cells were transduced with mCherry-EGFP-LC3 lentivirus (Beyotime Biotechnology, C3002). Autophagic flux was assessed using a confocal fluorescence microscope (Leica, Stellaris 5, Germany). Yellow fluorescence (mCherry+/EGFP+) indicated LC3B accumulation on autophagosome membranes, while red fluorescence (mmCherry+/EGFP-) indicated the fusion of autophagosomes with lysosomes.

### Co-IP, IP/MS, and Proteomic Assays

Co-IP was performed using a commercial kit (Beyotime Biotechnology, P2179M) according to the manufacturer's instructions. Cells were lysed in buffer containing protease inhibitors and incubated on ice for 30 min, followed by centrifugation at 4 °C for 5 min. The cell lysates were incubated overnight at 4 °C with protein A/G agarose beads pre-conjugated with the relevant primary antibody. The agarose beads were then washed three times with lysis buffer and resuspended in SDS sample buffer, followed by boiling at 95 °C for 5 min. The immunoprecipitates were subsequently analyzed using western blotting.

The procedure for IP/MS analysis was similar to that described earlier. Briefly, cells were lysed in buffer containing protease inhibitors, and lysates were incubated overnight with protein A/G agarose beads pre-conjugated with the appropriate antibody. After washing, the beads were sent for liquid chromatography-mass spectrometry analysis. Mass spectrometry analysis was performed by Bioprofile Biotechnology Co., Ltd. (Shanghai, China).

For proteomic assays, gene-edited and control cells were seeded in 6 cm dishes, collected using a cell scraper, rapidly frozen in liquid nitrogen, and stored at -80 °C until proteomic analysis. Proteomic assays were conducted by Biotree Biotechnology Co., Ltd. (Shanghai, China).

### Immunofluorescence Assay

Cells were seeded onto confocal dishes and incubated overnight. After incubation, the cells were fixed with 4% paraformaldehyde at room temperature for 15 min, followed by permeabilization with 0.1% Triton X-100. Blocking was performed with 5% bovine serum albumin (BSA) for 1 h. The cells were then incubated overnight at 4 °C with the appropriate primary antibody. After washing with PBST, the cells were incubated with fluorophore-conjugated secondary antibodies at room temperature for 1 h, followed by DAPI staining (Thermo Scientific, 62248) for 15 min. Coverslips were mounted using an anti-fade mounting medium, and images were captured using a confocal microscope (Stellaris 5, Leica, Germany).

### BiFC

HEK293T cells were transfected to overexpress HA-tagged VN173-SMURF2 fusion protein and FLAG-tagged VC155-UBE2L3 or VC155-TSC2 fusion proteins. The relevant plasmids were obtained from MiaolingBio (Wuhan, China). Twenty-four hours after transfection, the cells were fixed with 4% paraformaldehyde for 15 min. The cytoskeleton was stained with phalloidin (Beyotime Biotechnology, C2205S), and nuclei were stained with DAPI (Thermo Scientific, 62248). Images were captured using a confocal microscope (Leica, Stellaris 5, Germany).

### Ubiquitination Assay

Transfected cells were treated with 10 μM MG132 (Sigma) for 8 h. The cells were then lysed in buffer containing protease inhibitors. The lysates were incubated overnight with protein A/G agarose beads pre-conjugated with the relevant primary antibody. After the agarose beads were washed, they were resuspended in SDS sample buffer and boiled at 95 °C for 5 min. The ubiquitination levels of target proteins were subsequently analyzed using western blotting.

### Flow Cytometry

For flow cytometry analysis of *In vivo* experiments, mouse tumors were rapidly excised and minced into 1-3 mm^3^ pieces in sterile PBS. The tissue fragments were digested in PBS containing collagenase IV and DNase at 37 °C with agitation for 60 min. Following mechanical dissociation, the cell suspension was filtered through a 70 μm cell strainer, and red blood cells were lysed using red blood cell lysis buffer for 3 min. Immune cells were collected by centrifugation at 1500 rpm for 5 min. After blocking with anti-CD16/32 antibody (clone 93; BioLegend) for 30 min, the cells were stained with fluorochrome-conjugated surface antibodies for 30 min. Data acquisition was performed using a NovoExpress flow cytometer (Agilent), and analysis was conducted with FlowJo software (v10).

### Intracellular Cytokine Staining of Tumor-Infiltrating Lymphocytes

For the detection of intracellular cytokines in tumor-infiltrating lymphocytes, the cells were incubated in RPMI medium containing GolgiPlug and GolgiStop (BD Biosciences), with the addition of PMA (50 ng/mL, Sigma-Aldrich) and ionomycin (500 ng/mL, Sigma-Aldrich) for 4 h. Subsequently, the cells were fixed and permeabilized using BD Cytofix/Cytoperm (BD Biosciences), followed by staining with cytokine-specific antibodies. Data acquisition was performed on a NovoExpress flow cytometer (Agilent), and data analysis was conducted using FlowJo software (v10).

### H&E Staining and IHC Assays

Tumor tissues were fixed overnight in 4% paraformaldehyde, dehydrated, and embedded in paraffin. Then, the prepared tissue sections were subjected to H&E staining and IHC analysis. Primary antibodies used for IHC analysis included anti-UBE2L3 (1:100 dilution), anti-TSC2 (1:100), anti-Ki-67 (1:100), and anti-PCNA (1:100). Modified histological scoring was used to evaluate IHC staining for UBE2L3, TSC2, and PCNA. The H-score was calculated as follows: [percentage of weakly stained cells × 1] + [percentage of moderately stained cells × 2] + [percentage of strongly stained cells × 3], with scores for each staining ranging from 0 to 300.

### Bioinformatics Analysis

mRNA expression data and corresponding clinical data of triple-negative breast cancer (TNBC) were downloaded from The Cancer Genome Atlas (TCGA) database (https://portal.gdc.cancer.gov). Survival distributions were analyzed using the log-rank test and Kaplan-Meier (K-M) survival analysis. In the TCGA-TNBC cohort, patients were divided into high-expression and low-expression groups based on the median value of UBE2L3. A dataset containing single-cell RNA sequencing (scRNA-seq) data of TNBC (GSE176078) was retrieved from the Gene Expression Omnibus (GEO) database (http://www.ncbi.nlm.nih.gov/geo). The combined dataset included samples from 9 TNBC patients. After quality control (QC), a total of 35,179 high-quality single cells were retained for subsequent analysis.

### Statistical Analysis

Statistical analyses were performed using GraphPad Prism 10.0 (CA, USA). Statistical significance was determined using paired or unpaired Student's *t*-tests or one-way analysis of variance (ANOVA), as appropriate. Pearson correlation analysis was used to assess correlations between two groups of data. All experiments were conducted at least three times. Differences were considered statistically significant at P < 0.05.

## Results

### Critical Role of UBE2L3 in TNBC Growth

We performed *In vivo* genome-wide CRISPR/Cas9 screening of the murine TNBC cell line 4T1 to simulate tumor growth *In vivo* and efficiently identify key genes involved in TNBC cell proliferation. The library consists of 67,405 sgRNAs targeting 20,611 genes (Table S1). We successfully constructed a mouse TNBC cell line 4T1 with stable Cas9 expression using this library. After constructing the *In vitro* library, we transplanted the library-transduced 4T1-Cas9 cells into BALB/c mice to investigate the effect of genetic alterations on tumor cells *In vivo*. Fourteen days later, the mice were euthanized, and tumors were collected for high-throughput sgRNA library sequencing. By comparing genomic DNA (gDNA) collected from tumors grown in mice with that from *In vitro* cultured tumor cells, we identified genes critical for the growth of TNBC cells, based on the enrichment or depletion of specific gDNA in mouse tumors (Fig. 1A). To minimize bias and improve screening accuracy, we conducted three independent replicate sequencing experiments. Whereas the single guide RNA (sgRNA) distribution in pre-transplant tumor cells (day 0) and the tumor masses followed a log-normal distribution, we observed marked changes in sgRNA representation in tumor cells harvested from BALB/c mice after transplantation (Fig. S1A). Comparison of sgRNA abundance between mouse tumor samples and day 0 samples revealed that 3102 genes were depleted in mouse tumors relative to those in the pre-transplant library (Fig. 1B and Table S2). We ranked the differentially expressed genes using the robust rank aggregation (RRA) algorithm in MAGeCK, with lower RRA scores indicating greater essentiality to cell growth. Ultimately, we identified the top 10 most significantly depleted genes (Fig. 1C) and compared the corresponding changes in their gRNA abundance (Fig. 1D). Tumor cells carrying sgRNA targeting these genes were markedly depleted during tumor growth in mice. For example, histone deacetylase 3 (Hdac3) is known to play a critical role in maintaining chromatin architecture and genomic stability in tumor cells 27, supporting the reliability of our screening approach. To better understand the molecular events associated with tumor growth, we analyzed the negatively selected genes and found significant enrichment in pathways related to proteasome and protein degradation (Fig. S1B and C). After converting the top 10 candidate genes into their corresponding human orthologs, we performed a comprehensive analysis of gene expression correlation and clinical relevance to TNBC based on the TCGA database. We selected UBE2L3 as the candidate gene for further investigation (Fig. S1D and E). Since the regulatory role of UBE2L3 in TNBC has not been previously reported, we further validated the authenticity of the library screening results. We constructed a UBE2L3 stably knockdown 4T1 cell line. To better mimic the library sequencing results, we directly performed *In vivo* experiments to verify the effect of UBE2L3 knockdown on tumor growth. *In vivo* experimental results showed that the tumor growth rate of mice transplanted with the UBE2L3 knockdown cell line was significantly lower than that of mice transplanted with the parental 4T1 cell line, and the final tumor weight and volume were also much smaller than those of the latter (Fig. S1F-H). These findings suggest that UBE2L3 plays an important regulatory role in the progression of TNBC.

Western blotting was performed to detect the protein levels of UBE2L3 inTNBC cell lines and a normal mammary epithelial cell line. Compared with MCF-10A cells, the TNBC cell lines exhibited significantly higher UBE2L3 expression levels. Based on the differential expression profiles of UBE2L3, MDA-MB-231 and HCC1937 cell lines were ultimately selected for subsequent experiments (Fig. S1I). To assess the direct effect of UBE2L3 on tumor cell proliferation, we performed *In vitro* proliferation assays comparing the growth rates of gene-edited and control cells. Short-term proliferation was evaluated using real-time cellular analysis (RTCA), while long-term proliferation was assessed using colony formation assays. UBE2L3 knockdown reduced MDA-MB-231 cell proliferation, whereas UBE2L3 overexpression enhanced HCC1937 cell proliferation (Fig. 1E and F).

Additionally, we established two independent patient-derived organoids (PDOs) from TNBC samples to further investigate the role of UBE2L3 in tumor growth (Fig. 1G). In organoids with high UBE2L3 expression (PDO#1), UBE2L3 knockdown resulted in reduced growth. Conversely, in organoids with low UBE2L3 expression (PDO#2), UBE2L3 overexpression significantly increased proliferative capacity (Fig. 1G and H). Ki-67 staining analysis demonstrated that the proportion of Ki-67-positive cells decreased in organoids with reduced UBE2L3 protein levels (Fig. 1I). TNBC organoids faithfully recapitulate the features of primary tissues in 2 cases of TNBC tissues (Fig. S1J).

Finally, we conducted xenograft experiments to validate the *In vivo* function of UBE2L3. UBE2L3 depletion markedly inhibited tumor growth, as evidenced by smaller tumor volumes and weights in the UBE2L3 knockdown group (Fig. 1J-L). In contrast, the UBE2L3 overexpression group exhibited significantly larger tumors (Fig. S1K-N). Furthermore, IHC staining revealed that reduced UBE2L3 expression led to significantly lower levels of Ki-67 (a proliferation marker) and proliferating cell nuclear antigen in tumor tissues (Fig. 1M). Collectively, these findings indicate that UBE2L3 loss impairs TNBC cell growth both *In vitro* and *In vivo*.

### Decreased UBE2L3 Impairs Autophagy and Regulates TNBC Tumor Cell Growth

**Figure 2:**
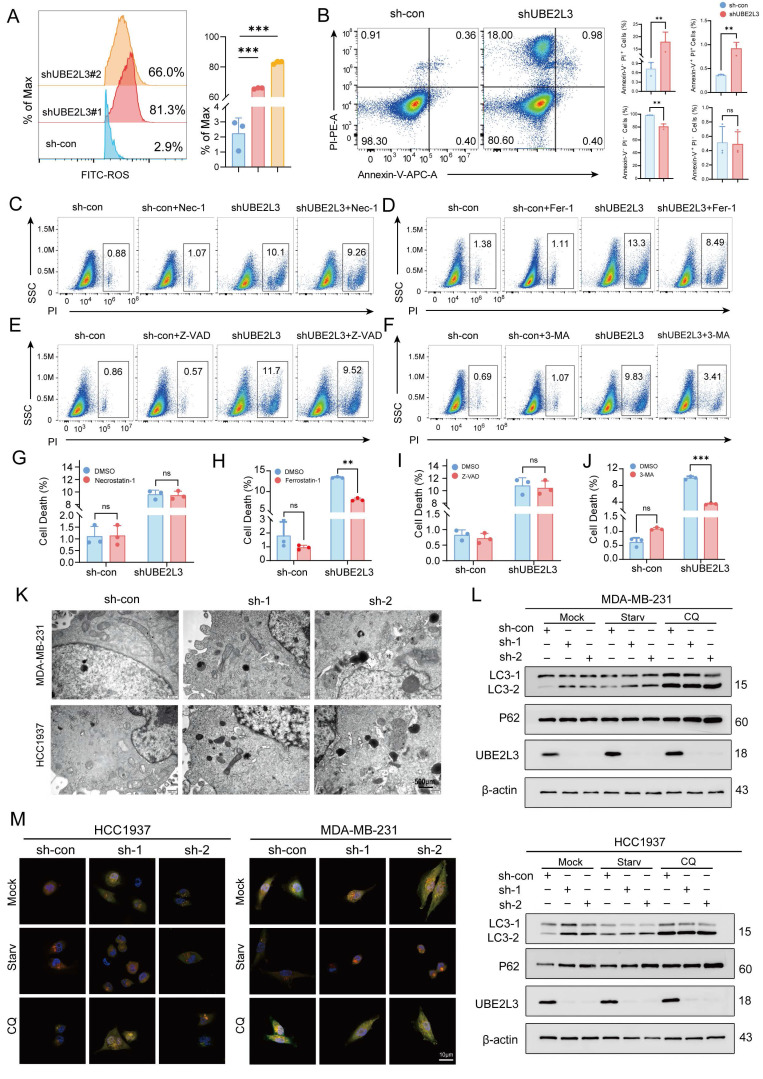
UBE2L3 deficiency promotes the accumulation of autophagosomes in TNBC cells. (**A**) Flow cytometric analysis of intracellular ROS levels in TNBC cells following UBE2L3 knockdown. (**B**) Annexin V-APC/PI staining and flow cytometry analysis of cell populations in UBE2L3-knockdown and control cells after 48 h of culture. PI staining was used to assess the effects of various inhibitors on UBE2L3 knockdown-induced TNBC cell death: (**C, G**) Nec-1, (**D, H**) Fer-1, (**E, I**) Z-VAD, (**F, J**) 3-MA. (**K**) Representative transmission electron microscopy (TEM) images showing autophagosomes in the indicated cells. Scale bar, 500 nm. (**L**) Western blot analysis of LC3 and p62 expression in control and UBE2L3-knockdown cells under starvation or chloroquine (CQ) treatment. (**M**) Detection of autophagic flux using the mCherry-EGFP-LC3B reporter in the indicated cells. Yellow puncta indicate autophagosomes (mCherry+/EGFP+), and red puncta indicate autolysosomes (mCherry+/EGFP-). Scale bar, 10 μm. Error bars represent mean ± SD. ***p < 0.001; **p < 0.01; not significant (NS), p ≥ 0.05.

To determine through which pathway UBE2L3 regulates tumor cell growth, flow cytometry was used to evaluate alterations in the cell cycle (Fig. S2A) and cellular injury (Fig. 2A). Upon reduction of UBE2L3 expression, no significant changes in the various phases of the tumor cell cycle were evident; however, a marked alteration in reactive oxygen species levels was noted. Hence, we hypothesized that UBE2L3 may attenuate tumor growth primarily by inducing cellular damage. To further investigate whether UBE2L3-mediated cellular damage affects tumor cell apoptosis, annexin V-FITC/PI staining was performed. Flow cytometry analysis revealed that the quantity of apoptotic cells in the experimental group did not increase significantly compared with that in the control group, whereas the quantity of cells undergoing alternative forms of death was markedly elevated (Fig. 2B). However, no change was observed when UBE2L3 was overexpressed (Fig. S2B). To delineate the precise mechanism underlying cell death induced by UBE2L3 deficiency, tumor cells were treated with inhibitors targeting autophagy (3-MA), ferroptosis (Fer-1), apoptosis (Z-VAD), and necroptosis (Nec-1), followed by rescue experiments. 3-MA significantly reversed UBE2L3-induced cell death, while Fer-1 partially mitigated this effect (Fig. 2C-J).

To corroborate the relationship between UBE2L3 and autophagy, transmission electron microscopy (TEM) was used to observe ultrastructural changes. The quantity of autophagosomes increased noticeably in TNBC cells following UBE2L3 knockdown (Fig. 2K). The formation of intracellular autophagosomes was inhibited when UBE2L3 was overexpressed (Fig. S2C). First, we detected the basal expression levels of LC3, TSC2, and mTOR in the MDA-MB-231 and HCC1937 cell lines (Fig. S2F). Subsequently, the involvement of UBE2L3 in autophagy initiation was verified. Markedly increased levels of LC3-II, a marker of autophagic activity, were observed upon UBE2L3 knockdown. Furthermore, under starvation and chloroquine (CQ) treatment, the conversion of LC3-I to LC3-II was further enhanced. As an indicator of autophagic flux, p62 reflects the rate of autophagosome transport to lysosomes; p62 accumulation was detected in cells with reduced UBE2L3 expression, implying that UBE2L3 knockdown impedes this process (Fig. 2L). In contrast, UBE2L3 overexpression reduced LC3-II accumulation during cell starvation (Fig. S2D).

Next, mCherry-EGFP-LC3B assays were conducted to monitor autophagic flux changes in TNBC cells. Under normal conditions, LC3B exhibits a diffuse yellow fluorescence in the cytoplasm. Upon autophagy induction, LC3B aggregates on autophagosome membranes, presenting as yellow puncta. Following fusion of autophagosomes with lysosomes, partial quenching of EGFP fluorescence results in red puncta. Notably, cells with UBE2L3 knockdown displayed an increase in yellow puncta without a proportional rise in red puncta, indicating enhanced autophagosome formation coupled with impaired autophagosome-lysosome fusion. Under starvation, accelerated autophagosome-lysosome fusion led to an increase in red puncta, whereas CQ, acting as a late-stage autophagy inhibitor, blocked p62 transport and augmented yellow puncta accumulation (Fig. 2M). In contrast, the inhibition of autophagosome formation was observed in cells with UBE2L3 overexpression (Fig. S2E).

Collectively, the experiments demonstrated that UBE2L3 loss promotes autophagosome formation while obstructing their transport to lysosomes, thereby disrupting autophagic flux in TNBC cells and ultimately inhibiting tumor cell proliferation.

### UBE2L3 Regulates Autophagy in TNBC Cells by Modulating TSC2 Ubiquitination and Affecting the mTOR Pathway

**Figure 3:**
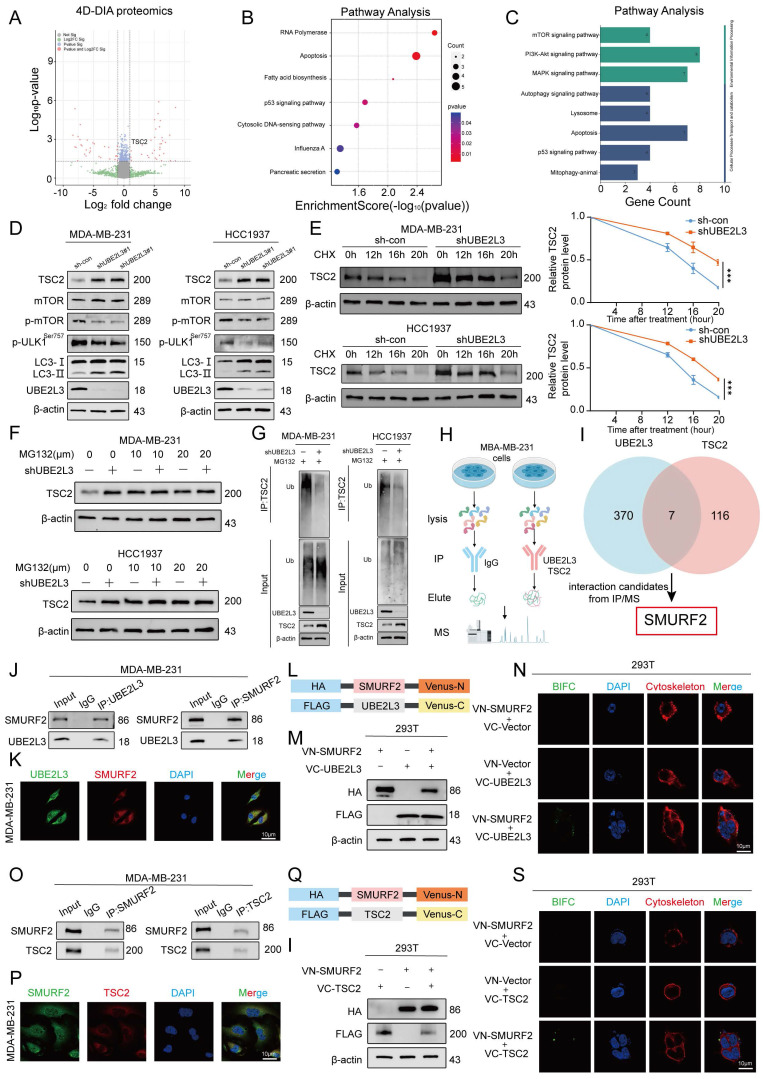
UBE2L3 regulates TSC2 expression via ubiquitination. (**A**) Volcano plot showing differentially expressed proteins between shUBE2L3 MDA-MB-231 cells and control cells (|log₂FC| > 1 and p < 0.05). (**B, C**) GO and KEGG analyses showing enrichment of differentially expressed proteins in autophagy-related pathways. (**D**) Western blot analysis of the impact of UBE2L3 deficiency on proteins involved in the mTOR-regulated autophagy pathway in TNBC cells. (**E**) Western blot analysis of TSC2 protein stability in UBE2L3-deficient TNBC cells following treatment with the protein synthesis inhibitor CHX (20 μM). (**F**) Western blot analysis of TSC2 protein levels in UBE2L3-knockdown and control TNBC cells after treatment with MG132 for 6 hours. (**G**) Western blot analysis of the effect of UBE2L3 deficiency on TSC2 ubiquitination in TNBC cells. (**H, I**) Schematic diagrams illustrating the identification of target E3 ubiquitin ligases by IP/MS. (**J**) Co-immunoprecipitation (Co-IP) experiments reveal the endogenous interaction between UBE2L3 and SMURF2 in MDA-MB-231 cells. (**K**) Representative immunofluorescence images showing co-localization of UBE2L3 and SMURF2 in MDA-MB-231 cells. Scale bar, 10 μm. (**L, Q**) Schematic diagrams of recombinant proteins used for bimolecular fluorescence complementation (BiFC) assays. (**M, R**) Western blot analysis of recombinant protein expression in HEK293T cells. (**N, S**) Representative immunofluorescence images showing protein-protein interactions in BiFC assays. Scale bar, 10 μm. (**O**) Co-IP experiments reveal the endogenous interaction between TSC2 and SMURF2 in MDA-MB-231 cells. (**P**) Representative immunofluorescence images showing co-localization of TSC2 and SMURF2 in MDA-MB-231 cells. Scale bar, 10 μm. Error bars represent mean ± SD. ***p < 0.001.

UBE2L3, recognized as a crucial E2 ubiquitin-conjugating enzyme, regulates the degradation of multiple substrate proteins and plays a pivotal role in modulating various biological processes. Proteomic sequencing was used to elucidate the underlying mechanisms by which UBE2L3 influences autophagy in tumor cells, with the aim of identifying potential substrate proteins regulated by UBE2L3 within TNBC cells. UBE2L3-knockdown MDA-MB-231 cells were compared with control cells, and 270 differentially expressed proteins were identified, of which 110 were upregulated and 160 were downregulated (Fig. 3A and Table S3). Notably, TSC2 exhibited upregulation. Subsequent Kyoto Encyclopedia of Genes and Genomes pathway enrichment analysis of these differentially expressed proteins revealed significant alterations, predominantly in the apoptosis and p53 signaling pathways (Fig. 3B). Further analysis of the differentially expressed proteins within these pathways showed that UBE2L3 suppression markedly affected autophagy-related signaling cascades, including the mTOR, PI3K-AKT, and MAPK pathways (Fig. 3C). Corroborated by previous rescue experiments, western blot validation of the enriched pathways confirmed that UBE2L3 regulates tumor cell autophagy primarily via the mTOR pathway. Specifically, upon reduction of UBE2L3 expression, an evident increase in the expression of TSC2, an mTOR inhibitor, was observed, concomitant with decreased phosphorylation levels of mTOR and the Ser757 site of ULK1. Additionally, expression of the autophagy marker LC3-II was elevated (Fig. 3D). Conversely, overexpression of UBE2L3 resulted in decreased TSC2 expression and increased phosphorylation levels of mTOR and ULK1 at the Ser757 site in TNBC cells (Fig. S3A).

The effect of UBE2L3 on TSC2 mRNA levels was assessed to determine whether UBE2L3 modulates TSC2 protein abundance and stability through ubiquitination. Knockdown of UBE2L3 did not alter TSC2 mRNA levels in TNBC cells (Fig. S3B). Subsequently, treatment of TNBC cells with the protein synthesis inhibitor cycloheximide demonstrated that the TSC2 protein undergoes time-dependent degradation. UBE2L3 downregulation prolonged the half-life of the TSC2 protein in both MDA-MB-231 and HCC1937 cells (Fig. 3E). To delineate the degradation pathway of TSC2, TNBC cells were treated with the proteasome inhibitor MG132 and the lysosomal inhibitor CQ. After 6 h of MG132 treatment, TSC2 levels increased in both MDA-MB-231 and HCC-1937 cells. However, UBE2L3 knockdown attenuated the MG132-induced elevation of TSC2 (Fig. 3F), whereas CQ treatment yielded no significant change (Fig. S3C). These findings indicate that the ubiquitin-proteasome system regulates TSC2 degradation and that UBE2L3 inhibition affects the TSC2 degradation rate. Finally, ubiquitination assays performed in the two cell lines confirmed that UBE2L3 knockdown significantly reduced the ubiquitination level of TSC2 following MG132 treatment (Fig. 3G).

Taken together, these results demonstrate that decreased UBE2L3 levels diminish TSC2 ubiquitination, extending its half-life and elevating protein levels. This suppresses mTOR pathway activity and modulates autophagy in TNBC cells.

### UBE2L3 Interacts with TSC2 in the Cytoplasm via SMURF2

Immunoprecipitation/mass spectrometry (IP/MS) was performed in MDA-MB-231 cells to elucidate the specific molecular mechanism by which UBE2L3 regulates TSC2 ubiquitination. Immunoprecipitation was conducted separately for UBE2L3 and TSC2, followed by mass spectrometry analysis (Fig. 3H). By comparing proteins interacting with UBE2L3 and those interacting with TSC2, SMURF2 was identified not only as a common interacting partner (Fig. S3D and E) but also as a key E3 ubiquitin ligase involved in ubiquitination regulation (Fig. 3I). To validate the protein-protein interactions among UBE2L3, SMURF2, and TSC2, Co-IP assays were conducted. The results demonstrated endogenous interactions between UBE2L3 and SMURF2 and between SMURF2 and TSC2 in MDA-MB-231 cells (Fig. 3J and O). Furthermore, to confirm the endogenous interactions *In situ*, immunofluorescence experiments were performed to assess colocalization within MDA-MB-231 cells. UBE2L3 and SMURF2, along with SMURF2 and TSC2, predominantly colocalized in the cytoplasm of tumor cells (Fig. 3K and P). To pinpoint the precise subcellular localization of these complexes, bimolecular fluorescence complementation (BiFC) assays were conducted. The split Venus protein fragments VN173 and VC155, widely used for BiFC detection, reassemble and emit fluorescence upon proximity in cells 28. SMURF2 was fused to the C-terminus of VC155, while UBE2L3 and TSC2 were fused to the N-terminus of VN173 (Fig. 3L and Q). These constructs were co-transfected into HEK293T cells according to their interaction groupings, and transfection efficiencies were verified by western blotting (Fig. 3M and I). Fluorescence was observed exclusively in cells co-transfected with both VN173 and VC155 fusion constructs, with no fluorescence detected in other groups; notably, the fluorescence localization was cytoplasmic (Fig. 3N and S). Finally, molecular docking models were constructed to predict specific amino acid residues mediating the binding interfaces between UBE2L3 and SMURF2 and between SMURF2 and TSC2 (Fig. S3F and G). We also constructed a SMURF2 stably knockdown cell line to investigate whether SMURF2 is involved in the regulation of TNBC growth (Fig. S3H and I).

### UBE2L3 Regulates TSC2 Ubiquitination via SMURF2

**Figure 4:**
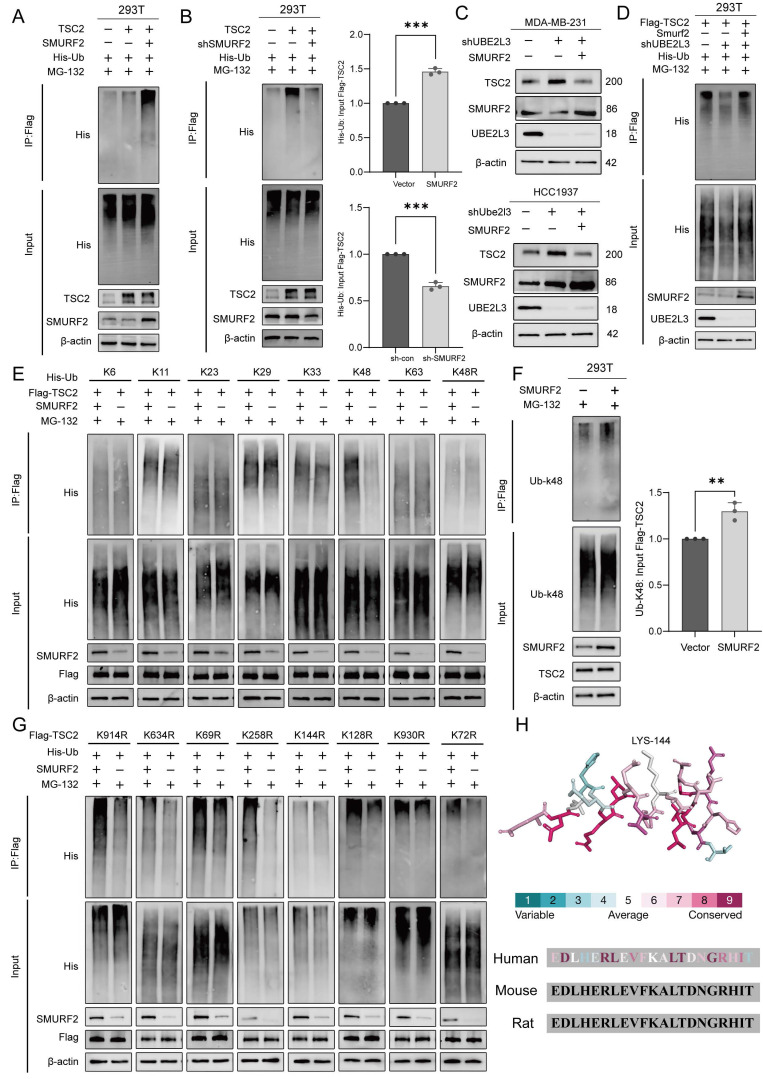
SMURF2 mediates K48-linked ubiquitination of TSC2 at lysine 144. (**A, B**) Western blot analysis of the effect of SMURF2 on TSC2 ubiquitination. HEK293T cells were co-transfected with Flag-TSC2, His-Ub, and SMURF2 or sh-SMURF2 plasmids for 24 hours, followed by incubation with 10 µM MG132 for 6 hours. Ubiquitinated proteins were pulled down using anti-Flag beads. (**C**) Western blot analysis of the effect of SMURF2 overexpression on TSC2 expression in UBE2L3-knockdown TNBC cells. (**D**) Western blot analysis of the effect of SMURF2 overexpression on TSC2 ubiquitination in UBE2L3-knockdown cells. (**E**) Western blot analysis of TSC2 ubiquitin chain types regulated by SMURF2. HEK293T cells were transfected with the indicated constructs for 24 hours, followed by incubation with 10 µM MG132 for 6 hours, and ubiquitinated proteins were pulled down using anti-Flag beads. The amounts of different ubiquitin constructs used for transfection were adjusted to equalize the input levels of TSC2 ubiquitination between groups. (**F**) Endogenous TSC2 K48-linked ubiquitination detected by anti-Ub-K48 antibody after 24-hour transfection of SMURF2 plasmid into HEK293T cells and 6-hour incubation with 10 µM MG132. (**G**) Western blot analysis of SMURF2-mediated ubiquitination of various TSC2 K-to-R mutants. HEK293T cells were co-transfected with different Flag-TSC2 K-to-R mutants and His-Ub for 24 hours, followed by incubation with 10 µM MG132 for 6 hours. Ubiquitinated proteins were pulled down using anti-Flag beads. (**H**) Conserved protein sequence of the K144 motif. Error bars represent mean ± SD. ***p < 0.001; **p < 0.01.

After establishing the interactions among UBE2L3, SMURF2, and TSC2, we hypothesized that SMURF2 serves as a critical mediator by which UBE2L3 regulates TSC2 ubiquitination. To explore this hypothesis, we investigated the ability of SMURF2 to ubiquitinate TSC2. Co-expression of SMURF2, Flag-tagged TSC2, and His-tagged ubiquitin (His-Ub) in HEK293T cells resulted in a significant increase in TSC2 ubiquitination compared with that in the control. Conversely, knockdown of SMURF2 markedly reduced TSC2 ubiquitination levels (Fig. 4A and B). These findings also support that changes in SMURF2 are involved in the regulation of TNBC growth.

To determine whether SMURF2 and UBE2L3 modulate TSC2 via the same ubiquitination pathway, SMURF2 was overexpressed in UBE2L3-knockdown TNBC cells. Western blot analysis revealed that the elevated expression of SMURF2 could rescue the effects of UBE2L3 knockdown on TSC2 (Fig. 4C). Additionally, transfection of SMURF2 plasmid into UBE2L3-deficient HEK293T cells was performed to examine changes in TSC2 ubiquitination; increased SMURF2 expression compensated for the reduction in TSC2 ubiquitination caused by UBE2L3 depletion (Fig. 4D). Collectively, these findings indicate that UBE2L3 regulates TSC2 ubiquitination through SMURF2 mediation.

### SMURF2 Mediates K48-Linked Ubiquitination of TSC2 at Lysine 144

Ubiquitin contains seven lysine residues (K6, K11, K27, K29, K33, K48, and K63), through which ubiquitin moieties can be interlinked, forming polyubiquitin chains with highly diverse topologies 29. Among these, K48- and K63-linked polyubiquitinations are most closely associated with protein degradation 30. To determine which ubiquitin linkage is assembled by SMURF2, we co-transfected ubiquitin mutants harboring only a single lysine residue, along with SMURF2, into HEK293T cells stably expressing Flag-tagged TSC2. SMURF2 predominantly facilitated the assembly of K48-linked ubiquitin chains on TSC2 (Fig. 4E). To validate this finding, His-Ub with a K48R mutation (His-Ub-K48R) was transfected into HEK293T cells; under these conditions, SMURF2 failed to further promote TSC ubiquitination via K48R mutant ubiquitin chains (Fig. 4E). After SMURF2 was overexpressed in HEK293T cells without exogenous ubiquitin expression, a marked increase in K48-linked ubiquitination of TSC2 was observed (Fig. 4F). Collectively, these results indicate that SMURF2 assembles K48-linked ubiquitin chains on TSC2.

The GPS-Uber online tool was used to analyze the TSC2 protein sequence and identify putative ubiquitination sites (Fig. S4A and B). The eight lysine residues with the highest scores were selected for individual lysine-to-arginine (K-to-R) mutagenesis. These mutants were co-transfected with SMURF2 into HEK293T cells. Mutation of lysine 144 (Lys144) to arginine (K144R) abolished the ability of SMURF2 to enhance TSC2 ubiquitination (Fig. 4G). This evidence strongly suggests that Lys144 is the critical site for SMURF2-mediated ubiquitination of TSC2. Finally, the conservation of residue K144 and its flanking amino acid sequence within TSC2 was verified across different species, underscoring its evolutionary importance (Fig. 4H).

### TSC2 Deletion in TNBC Cells Rescues the Effects Induced by UBE2L3 Depletion

**Figure 5:**
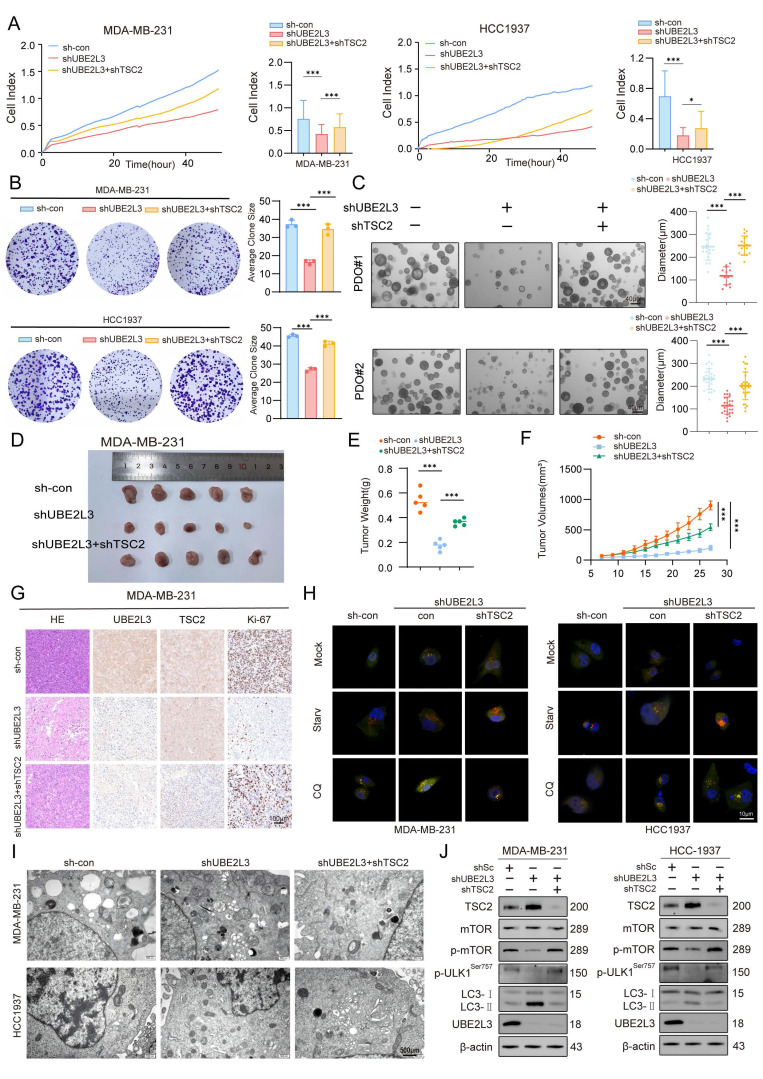
The regulatory effects of UBE2L3 deficiency on TNBC cells are mediated by TSC2. (**A**) Real-time cell analysis (RTCA) of proliferation rates in the indicated cells. (**B**) Colony formation assay measuring the impact of TSC2 knockdown on colony-forming ability in UBE2L3-deficient cells. (**C**) Representative images of organoid diameter and Ki-67 immunofluorescence intensity in UBE2L3-deficient organoids following TSC2 knockdown. Scale bar, 40 μm. The indicated cells were subcutaneously injected into nude mice, and tumor growth was monitored: (**D**) Representative images of tumors, (**E**) Tumor weight, (**F**) Tumor volume, and (**G**) Representative H&E staining and IHC images showing UBE2L3, TSC2, and Ki-67 expression in xenograft tissues. (**H**) Representative images of autophagic flux detected by the mCherry-EGFP-LC3B reporter in the indicated cells. Scale bar, 10 μm. (**I**) Representative transmission electron microscopy images showing the ultrastructure of autophagosomes and/or lysosomes in the indicated cells. Scale bar, 500 nm. (**J**) Western blot analysis of the effect of TSC2 knockdown on mTOR-regulated autophagy pathway proteins in UBE2L3-deficient TNBC cells. Error bars represent mean ± SD. ***p < 0.001; **p < 0.01.

To investigate whether TSC2 participates in the regulatory effects of UBE2L3, TSC2 was knocked down in UBE2L3-deficient MDA-MB-231 and HCC1937 cells. RTCA and colony formation assays revealed that TSC2 loss reversed the inhibitory effect of UBE2L3 knockdown on TNBC cell proliferation (Fig. 5A and B). In a parallel experiment, we overexpressed TSC2 in UBE2L3-overexpressing TNBC cells. The experimental results showed that overexpression of TSC2 inhibited the cell proliferation accelerated by UBE2L3 overexpression in TNBC cells (Fig. S5A and B). In the PDO model of TNBC, UBE2L3 depletion markedly suppressed organoid growth; however, concurrent TSC2 knockdown partially rescued the growth inhibition caused by UBE2L3 deficiency (Fig. 5C). Conversely, overexpression of TSC2 in UBE2L3-overexpressing organoid models resulted in inhibition of model growth (Fig. S5G).

Next, subcutaneous xenograft tumor models were established to elucidate the role of TSC2 in mediating the effects of UBE2L3 depletion *In vivo*. Consistent with the *In vitro* findings, TSC2 deletion abrogated the suppressive effect of UBE2L3 knockdown on tumor growth, as evidenced by tumor growth curves, tumor weight measurements, hematoxylin and eosin (H&E) staining, and immunohistochemical (IHC) analyses of tumor tissues from tumor-bearing mice (Fig. 5D-G). Meanwhile, *In vivo* experiments also verified that overexpression of TSC2 could rescue the accelerated tumor growth caused by UBE2L3 overexpression. This rescue effect was evident in gross observation of tumor size, hematoxylin-eosin (HE) staining of tissues, and measurement of tumor weight (Fig. S5C-F). These data suggest that the inhibitory effect of UBE2L3 depletion on TNBC cell growth is likely mediated through upregulation of TSC2 expression.

Finally, rescue experiments were conducted to determine whether TSC2 mediates the influence of UBE2L3 on autophagy in TNBC cells. TSC2 knockdown significantly reversed the increased autophagosome accumulation induced by UBE2L3 depletion (Fig. 5H and I). Western blot analyses confirmed that TSC2 reduction restored the alterations of key proteins within autophagy-related pathways caused by UBE2L3 knockdown (Fig. 5J). Additionally, Western blotting analysis of UBE2L3-overexpressing cells revealed that overexpression of TSC2 could still affect the autophagic pathway and promote the accumulation of LC3B (Fig. S5H).

Collectively, these findings substantiate the hypothesis that TSC2 functions as a critical downstream target through which UBE2L3 regulates TNBC cell behavior.

### Inhibition of UBE2L3 Remodels the Tumor Microenvironment of TNBC Cells and Enhances Sensitivity to Anti-PD-1 Therapy

To validate the clinical relevance of our study, data of TNBC patients were extracted from The Cancer Genome Atlas database based on pathological clinical parameters. A comparative analysis of UBE2L3 expression between normal and tumor tissues was then performed (Fig. S1E). The results demonstrated a trend of elevated UBE2L3 expression in tumor tissues relative to that in adjacent normal tissues in TNBC patients, corroborating our previous findings.

**Figure 6:**
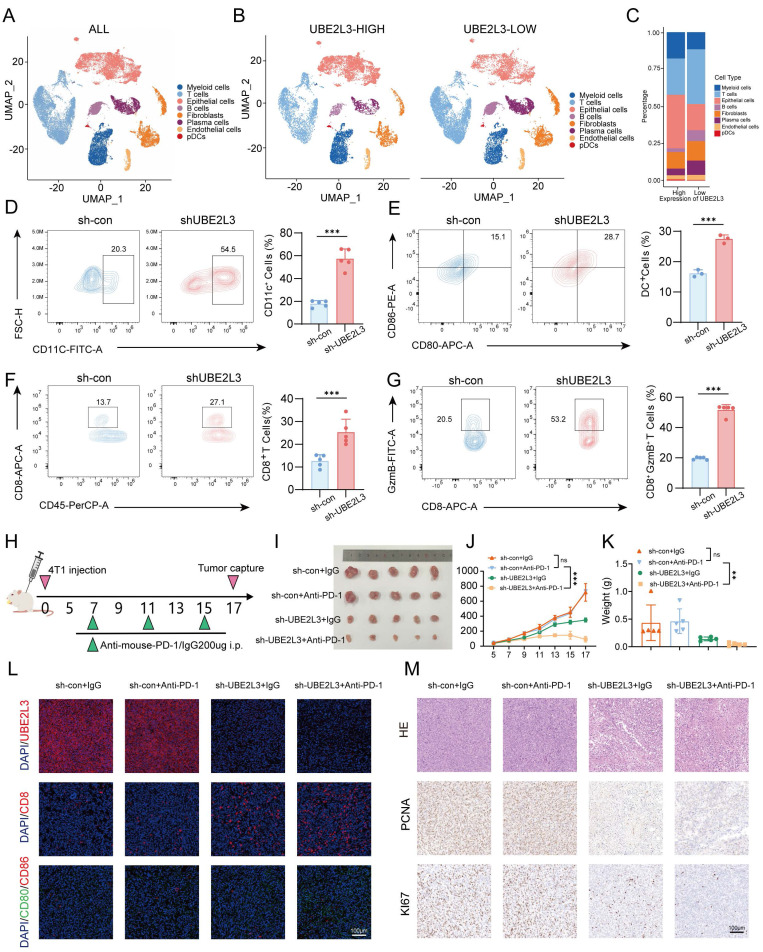
UBE2L3 deficiency enhances the sensitivity of TNBC to immunotherapy. (**A-C**) UMAP plots and stacked bar graphs showing the composition of tumor-infiltrating immune cells in TNBC. Cells and clusters are color-coded according to major identified cell types. Flow cytometric analysis of (**D**) CD11C+ cells among CD45+ cells, (**E**) dendritic cells among CD11C+ cells, (**F**) CD8+ T cells among CD3+ T cells, and (**G**) granzyme B+ (GZMB+) cells among CD8+ T cells in control and UBE2L3-knockdown 4T1 tumors harvested from BALB/c mice. (**H**) Schematic of the *In vivo* experiment: Control or UBE2L3-knockdown 4T1 cells were subcutaneously injected into BALB/c mice, and 7 days later, mice were treated three times with anti-PD-1 antibody or isotype control IgG. (**I**) Representative images of tumors, (**J**) tumor volume, (**K**) tumor weight, and (**L, M**) representative images of immunofluorescence (IF), H&E staining, and IHC in xenograft tissues. Scale bar, 100 μm. Error bars represent mean ± SD. ***p < 0.001; not significant (NS), p ≥ 0.05.

Autophagy dynamically adapts in response to various signals within the tumor microenvironment. Autophagic pathways in tumor cells are modulated accordingly; however, autophagy plays an indispensable role in regulating tumor immunity 31 and can endow tumors with novel immunogenicity in certain contexts. Previous studies have demonstrated that autophagy pathway blockade using anti-TIM4 monoclonal antibodies enhances tumor antigen-specific CD8^+^ T cell infiltration, thereby potentiating the antitumor efficacy of chemotherapeutic agents 32. We investigated whether autophagic alterations induced by UBE2L3 deficiency bear functional consequences on tumor immunity. Using data (GSE176078) from the Gene Expression Omnibus database, we analyzed tumor microenvironment profiles of TNBC patients. Single-cell sequencing results revealed that, compared with patients with high UBE2L3 expression, those exhibiting low UBE2L3 expression displayed enhanced tumor immune infiltration, predominantly characterized by increased T lymphocyte infiltration into the tumor microenvironment (Fig. 6A-C). To validate these findings, flow cytometry analysis was performed to quantify immune cell infiltration in tumor samples derived from BALB/c mice bearing orthotopically transplanted 4T1 cells, either with UBE2L3 knockdown or control. The results demonstrated a significant increase in the proportions of dendritic cells and CD8^+^ T lymphocytes in single-cell suspensions from UBE2L3-low tumors, while NK cells, as another common antitumor tumor-infiltrating immune cells (TIICs), and the major protumor TIICs regulatory T cells and myeloid-derived suppressor cells (MDSCs) remained unchanged (Fig. S6A-C). Moreover, elevated levels of cytotoxic mediators, including granzyme B, were observed (Fig. 6D-G). However, the expression of exhaustion markers (PD-1 and TIM-3) in single-cell suspensions showed no significant changes, and the level of another major cytotoxic factor, interferon-γ (IFN-γ), also remained unchanged (Fig. S6D-E).

Based on these results, we hypothesized that UBE2L3 may influence the efficacy of anti-PD-1 immunotherapy. To investigate the effect of UBE2L3 deficiency on anti-PD-1 treatment efficacy, four experimental groups were designed (Fig. 6H). Compared with controls, administration of an equivalent dose of anti-PD-1 antibody in mice bearing UBE2L3-knockdown tumors yielded superior antitumor effects, evidenced by reductions in final tumor size, tumor growth rate, and tumor weight (Fig. 6I-K). In mice bearing tumors without UBE2L3 knockdown, anti-PD-1 antibody treatment failed to produce significant differences between the experimental and control groups, consistent with previous reports that 4T1 tumors, characterized as immunologically “cold,” are resistant to anti-PD-1 therapy 33. Immunofluorescence analyses of tumor tissues corroborated the flow cytometry findings, revealing enhanced lymphocyte infiltration into UBE2L3-deficient tumors (Fig. 6K). IHC staining indicated that, relative to controls, UBE2L3-knockdown tumors treated with anti-PD-1 antibody exhibited reduced malignancy (Fig. 6L).

Collectively, these data suggest that loss of UBE2L3 increases lymphocyte infiltration into the tumor microenvironment, converting 4T1 tumors from an immunologically “cold” phenotype to a “hot” one and thereby enhancing their sensitivity to anti-PD-1 immunotherapy.

## Discussion

Owing to the high efficiency, precision, and design flexibility of CRISPR/Cas9 technology, it is used to explore cancer-related gene functions, elucidate immune cell-tumor cell interaction mechanisms, and identify novel drug targets 34. Conventional chemotherapy produces a limited clinical benefit in certain subsets of TNBC patients. Hence, the need to develop more effective and beneficial therapeutic strategies persists. Recent studies have proposed targeted therapies based on molecular and signaling pathway aberrations specific to TNBC, while immune checkpoint inhibitors are also being explored clinically 35. We used *In vivo* genome-wide CRISPR/Cas9 screening to search for effective therapeutic targets and identified UBE2L3 as a potential candidate.

UBE2L3, also known as UbcH7, is a conserved protein harboring a ubiquitin-conjugating domain capable of interacting with both E1-activating enzymes and E3 ligases 36. It plays critical roles in the pathogenesis of various diseases, including Parkinson's disease and multiple autoimmune disorders 37-39. Numerous cancer-related studies have focused on UBE2L3. For example, in hepatocellular carcinoma, loss of UBE2L3 enhances the stability of the GSK3β protein, promoting its expression and activation and ultimately inhibiting hepatocellular carcinoma cell proliferation. A study involving 125 gastric cancer patients revealed that UBE2L3 expression is upregulated in gastric cancer and that its high expression correlates significantly with poor differentiation and reduced overall survival 40.

Here, we identified UBE2L3 as a key regulator of TNBC growth. UBE2L3 deficiency exerted tumor-suppressive effects by increasing the accumulation of autophagosomes in tumor cells. This was substantiated by rescue experiments using early-stage autophagy inhibitors, confirming that UBE2L3 modulated tumor progression by regulating autophagy. However, reduction of UBE2L3 expression led to an increase in p62 levels, implying impaired autophagosome-lysosome fusion. Similar observations have been reported in Parkinson's disease models, where ubiquitination and p62 accumulation persist in mitochondria following UBE2L3 knockout but are markedly diminished upon concurrent knockout of multiple UBE2 family members 41. Hence, determining whether members of the UBE2 family synergistically regulate p62 represents a compelling direction for future investigations.

Mechanistically, UBE2L3 depletion attenuates TSC2 ubiquitination, prolongs its half-life, and increases its expression, suppressing mTOR activity. This suppression accelerates the conversion of LC3-I to LC3-II, promoting autophagosome accumulation and ultimately inhibiting TNBC growth. Subsequently, we identified SMURF2 as the ubiquitin ligase interacting with UBE2L3. Unlike most E2 enzymes that transfer ubiquitin to RING-type E3 ligases, UBE2L3 typically transfers ubiquitin to homologous to E6AP carboxyl terminus (HECT)-type E3 ligases 42. SMURF2, a HECT domain-containing E3 ubiquitin ligase 43, mediates K48-linked ubiquitination of TSC2 at Lys144.

As a tumor suppressor gene, TSC2 serves as a critical convergence node within a complex signaling network, integrating virtually all upstream stimuli capable of modulating mTORC1 activity 44. The TSC2-mTORC1-autophagy axis plays a pivotal regulatory role in numerous diseases. For example, in studies related to DNA damage, RhoB has been shown to mediate lysosomal translocation of TSC2, leading to mTORC1 inactivation and subsequent induction of autophagy 45. In mouse embryonic fibroblasts, TSC2 deficiency stimulates mTORC1 activation, thereby inhibiting autophagy and increasing sensitivity to various cell death stimuli 46. In the present study, UBE2L3 was shown to directly regulate TSC2 protein expression. Ubiquitination assays further revealed that UBE2L3, a key enzyme in the ubiquitination process, modulated TSC2 ubiquitination through the E3 ligase SMURF2. Elevated TSC2 expression suppresses mTORC1 activity, and activated mTOR phosphorylates ULK1 at Ser757, which disrupts phosphorylation at Ser317 and Ser777, thereby inhibiting ULK1 activation and suppressing autophagy 47. Consistently, our data showed that increased TSC2 expression reduces mTOR phosphorylation and leads to decreased phosphorylation at ULK1 Ser757, which relieves autophagy inhibition and results in LC3-II accumulation.

Autophagic modulation in tumor cells exerts a dual role in tumor immune evasion and immunotherapy. A deeper understanding of the functions of autophagy in the field of tumor immunology may facilitate the development of novel therapeutic strategies 48. Given the critical role of autophagy in regulating immune responses, several clinical trials exploring autophagy targeting for the activation of anti-tumor immunity have been initiated 49. Moreover, the regulation of tumor immunity by autophagy is multi-faceted. For instance, a study demonstrated that autophagic degradation of PD-L1 in small cell lung cancer could enhance anti-tumor immunity, augment T cell cytotoxicity, and suppress immune evasion 50. In another study, CXCL1 was found to promote immune evasion by enhancing autophagy-mediated MHC-I degradation in colorectal cancer 51. Additionally, in cervical cancer, IFN-γ stimulation upregulates IDO-1 expression and induces autophagy, which further promotes macrophage activation 52.

The tumor microenvironment governs tumor growth, metastasis, and immune responses. The reciprocal interactions between tumor cells and the tumor microenvironment manifest in immune microenvironmental regulation that can inhibit tumor cell proliferation via various microenvironmental constituents. Conversely, tumor cells can promote tumor progression by modulating tumor immunogenicity and selecting for clones better adapted to the microenvironment 53.

Due to the plasticity and heterogeneity inherent to the tumor microenvironment, immunotherapy has emerged as one of the most promising treatment modalities for cancer patients. TNBC exhibits a relatively high response rate to immune checkpoint inhibitors (ICIs); however, the efficacy of ICIs as monotherapy remains limited. Through quantitative assessment of tumor-infiltrating lymphocytes (TILs), TNBC tumors can be stratified into immunologically “hot” (high TIL) or “cold” (low TIL) phenotypes, which closely correlate with responsiveness to ICIs. Phenotypic characterization of TILs has deepened our understanding of immune cell populations, revealing that elevated levels of CD8^+^ T cells or increased CD8/FOXP3 ratios may predict more favorable responses 54. Accordingly, we investigated the effect of UBE2L3 modulation on the TNBC tumor microenvironment and the potential significance of targeting this gene. Our results demonstrated that UBE2L3 reshapes components of the TNBC tumor microenvironment, facilitating enhanced lymphocyte infiltration. Although UBE2L3 depletion does not directly regulate PD-L1 expression in tumor cells (Fig. S7I and J), its influence on T cells renders TNBC cells more sensitive to anti-PD-1 therapy.

Furthermore, we extended our analysis to the correlation between UBE2L3 and TSC2 across various other cancer types using TCGA data (Fig. S7A-F). The results revealed that the correlation between UBE2L3 and TSC2 is not universal, and we further validated this finding in relevant cancer types (Fig. S7G-H). These results suggest that the regulatory effect of UBE2L3 on TSC2 may be specific to TNBC. We hypothesize that the regulation of TSC2 stability by UBE2L3 may be tissue context-dependent, and its mechanism of action may be associated with the E3 ligases expressed in different cancer types. In addition, further investigations into different subtypes of breast cancer have not been conducted in the current study, particularly in hormone receptor-positive (HR+) breast cancer. This exploration is crucial as it determines whether the UBE2L3-SMURF2-TSC2 signaling axis exhibits breast cancer subtype specificity. TNBC is characterized by the loss of estrogen receptor (ER), progesterone receptor (PR), and human epidermal growth factor receptor 2 (HER2) expression, which prevents it from relying on classical hormone signaling pathways for proliferation. Instead, TNBC is highly dependent on alternative pathways such as autophagy, PI3K/Akt/mTOR, and immune checkpoints to maintain survival and progression. In contrast, the constitutively activated ER signaling pathway in HR+ cells may exert an “overriding effect” on the regulatory efficacy of the UBE2L3-SMURF2-TSC2 axis. Therefore, our research group is currently conducting further explorations on the role of UBE2L3 in various cancers and different types of breast cancer.

## Conclusions

**Figure 7:**
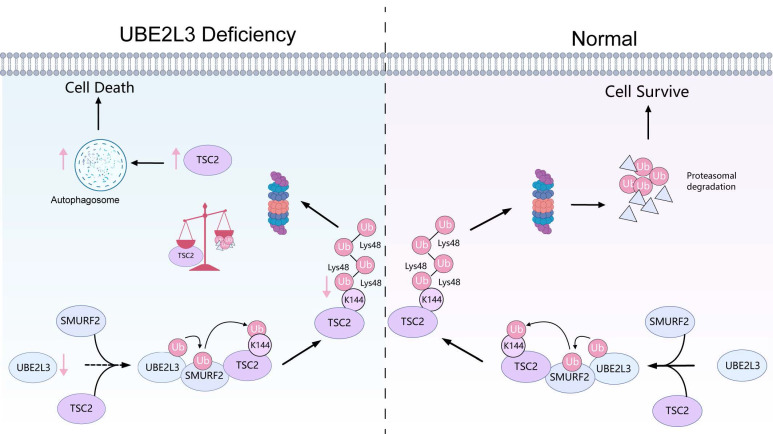
The mechanism of UBE2L3 in the growth process of TNBC. In TNBC, UBE2L3 regulates the protein level of TSC2 by influencing SMURF2-mediated ubiquitination of lysine 144 on TSC2, thereby affecting changes in autophagy of TNBC cells to regulate TNBC growth. (This figure was created with Medpeer).

In summary, our study demonstrated that UBE2L3 knockdown in TNBC inhibits ubiquitin-mediated TSC2 degradation, thereby suppressing mTOR activity, promoting LC3-II expression, and increasing autophagosome formation in tumor cells, ultimately inhibiting tumor growth (Fig. 7). Reduction of UBE2L3 expression enhanced the efficacy of immunotherapy. Although the underlying mechanisms require further investigation, these findings collectively indicate that targeting UBE2L3 represents a highly promising therapeutic strategy for TNBC.

## Supplementary Material

Supplementary figures and tables 4-5.

Supplementary table 1.

Supplementary table 2.

Supplementary table 3.

## Data Availability

Upon reasonable request, relevant materials can be provided. All data generated or analyzed in this study are available in the supplementary materials or from the corresponding author. The results presented herein are based on data generated from The Cancer Genome Atlas (TCGA) and Gene Expression Omnibus (GEO) cohorts, which are publicly available in the repositories of the GDC Data Portal (https://portal.gdc.cancer.gov/) and GEO database (http://www.ncbi.nlm.nih.gov/geo).

## Abbreviations

TNBC: Triple-negative breast cancer
UBE2L3: Ubiquitin-conjugating enzyme E2 L3
TSC2: TSC complex subunit 2
SMURF2: E3 ubiquitin-protein ligase SMURF2
CD8: Cluster of differentiation 8
PD-1: Programmed cell death 1
HER2: Receptor tyrosine-protein kinase erbB-2
GSK3β: GSK3B-interacting protein
p65: RelA
NSCLC: Non-small-cell lung cancer
Skp2: S-phase kinase-associated protein 2
p27^kip1^: Cyclin-dependent kinase inhibitor 1B
mTOR: Mammalian target of rapamycin
Rheb-GTP: GTP-binding protein Rheb homolog
DAPK1: Death-associated protein kinase 1
TSC1: Tuberous sclerosis 1 protein
sgRNA: Single guide RNA
RRA: Robust rank aggregation
IgG: Immunoglobulin G
SDS-PAGE: SDS-polyacrylamide gel electrophoresis
PVDF: polyvinylidene difluoride
TBST: Tris buffered Saline Tween
RTCA: Real-time cellular analysis
PI: Propidium iodide
TEM: Transmission electron microscopy
LC3: Microtubule-associated protein 1 light chain 3
BSA: Bovine serum albumin
DAPI: 4',6-diamidino-2-phenylindole
MG132: Z-Leu-Leu-Leu-al
HE: Hematoxylin and eosin
IHC: Immunohistochemistry
IP: Immunoprecipitation
IF: Immunofluorescence
gDNA: Genomic DNA
Hdac3: Histone deacetylase 3
PDOs: Patient-derived organoids
ROS: Reactive oxygen species
3-MA: 3-Methyladenine
Fer-1: Ferrostatin-1
Z-VAD: Z-Val-Ala-Asp (OMe)-Fluoromethylketone
Nec-1: Necrostatin-1
ULK1: Unc-51 like autophagy activating kinase 1
CQ: Chloroquine
MS: Mass spectrometry
BiFC: Bimolecular fluorescence complementation
ICIs: Immune checkpoint inhibitors
TILs: Tumor-infiltrating lymphocytes
FOXP3: Forkhead box P3
