# Supplementary material for: In vivo CRISPR/Cas9 Screening Reveals that UBE2L3 Modulates Autophagic Flux through TSC2 Ubiquitination and Potentiates PD-1 Blockade in Triple-Negative Breast Cancer
Source: Int J Biol Sci. 2026 Feb 18;22(6):2950–69. doi: 10.7150/ijbs.124937 (PMC13050453; doi:10.7150/ijbs.124937)
Supplement: Supplementary file 1 — Supplementary figures and tables 4-5. [file ijbsv22p2950s1.pdf]

In Vivo CRISPR/Cas9 Screening Reveals that UBE2L3 Modulates Autophagic Flux Through TSC2 Ubiquitination and Potentiates PD-1 Blockade in Triple-Negative Breast Cancer

Jian Xu[3,†], Ling Cheng[6,†], Sien Ma[5], Chen Gan[1,2], Jiaying Chai[1,2], Xinyi Zheng[1,2], Longyu Hu[1,2], Meiwen Ling[1,2], Mingjun Zhang[4*], Bao Zhao[5*], Huaidong Cheng[1,2*]

[1]Department of Oncology, Shenzhen Hospital of Southern Medical University, Shenzhen, Guangdong, China

[2]Shenzhen Clinical Medical School of Southern Medical University.

[3]Department of Surgical Oncology, The First Affiliated Hospital of Bengbu Medical University, Bengbu, Anhui, China

[4]Department of Oncology, Second Affiliated Hospital of Anhui Medical University, Hefei, Anhui, China

[5]Institute of Health and Medicine, Hefei Comprehensive National Science Center, Hefei, Anhui, China

[6]Department of Traditional Chinese Medicine, The People's Hospital of Pingshan Shenzhen (Pingshan Hospital of Southern Medical University), Shenzhen, Guangdong, China

[†]Jian Xu and Ling Cheng contributed equally.

* Corresponding author: Prof Huaidong Cheng, Department of Oncology, Shenzhen Hospital of Southern Medical University, Shenzhen, Guangdong 518000, China chd1975ay@126.com or Prof Bao Zhao, Institute of Health and Medicine, Hefei Comprehensive National Science Center, Hefei, Anhui, 230071, China, zhaobao@ihm.ac.cn or Mingjun Zhang, Department of Oncology, The Second Hospital of Anhui Medical University, Hefei, Anhui 230601, China, 13865952007@163.com

The PDF file includes: Figs. S1 to S7
Legends for tables S1 to S5
Tables S1 to S5

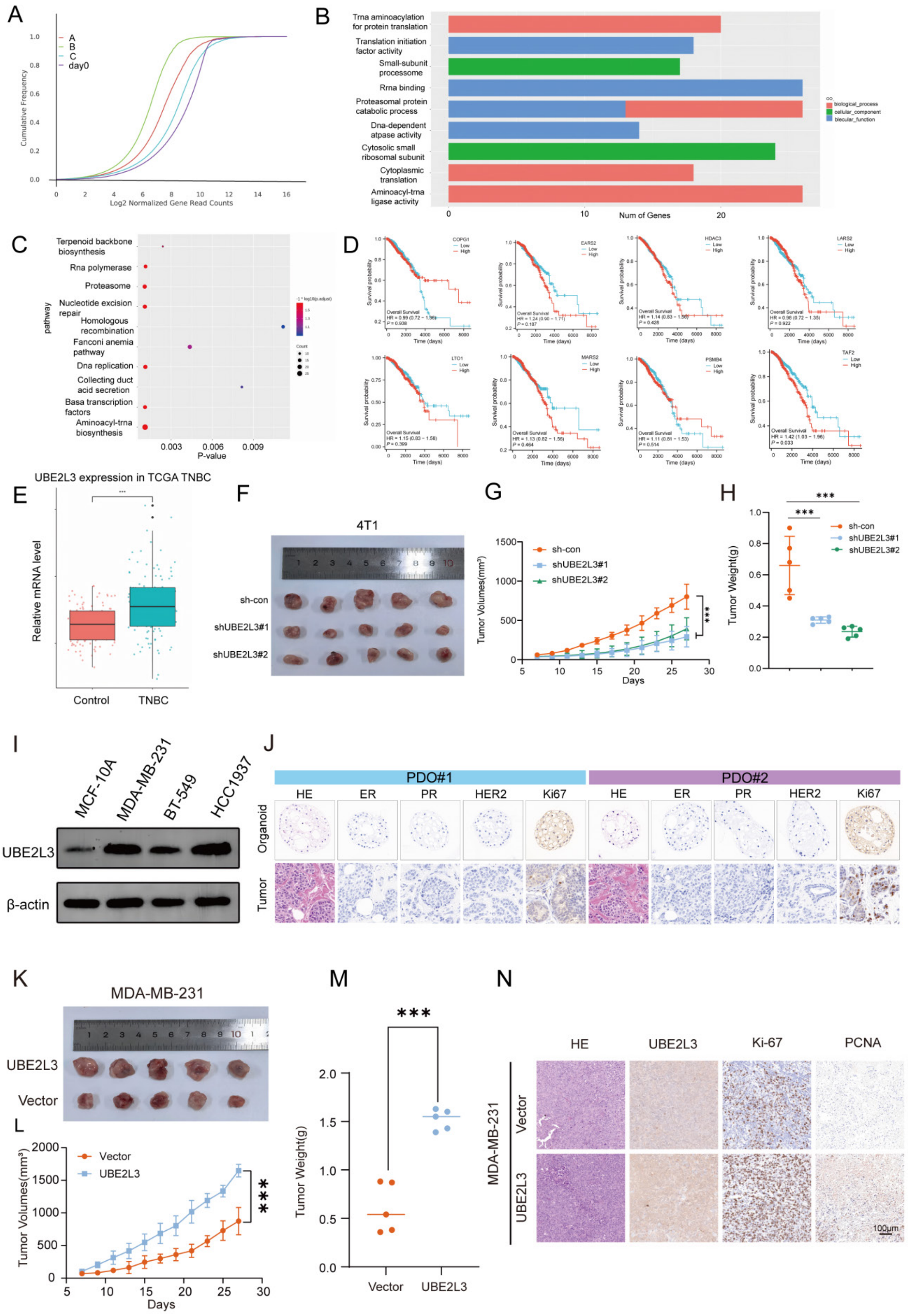

Figure S1. (**A**) The detected gRNA reads were counted and statistically analyzed according to their corresponding genes, followed by normalization and cumulative

distribution analysis of the statistical values to show the enrichment or loss of essential cellular genes in the samples. (**B**) Visualization of GO (cellular component, molecular function, biological process, respectively) classification of the top 10 genes ranked by differential screening results (pos|rank/neg|rank). (**C**) Visualization of KEGG pathways of the top 10 genes ranked by differential screening results (pos|rank/neg|rank). (**D**) Kaplan-Meier (K-M) curves of overall survival (OS) of breast cancer patients for the top 10 genes ranked by differential screening results in the TCGA dataset. (**E**) Analysis of UBE2L3 expression in triple-negative breast cancer and adjacent normal tissues using the TCGA database. 4T1 cells with UBE2L3 knockdown were subcutaneously inoculated into BALB/c mice to observe tumor growth. (**F**) Tumor images, (**G**) tumor volume, (**H**) tumor weight. (**I**) protein expression of UBE2L3 in MCF-10A,MDA-MB-231, BT-459, and HCC1937 cells. (**J**) Hematoxylin-Eosin (H&E) staining and immunostaining for ER, PR, HER2 and Ki-67 in organoids and control tissues; scale bars: 100 μm. MDA-MB-231 cells with UBE2L3 overexpression were subcutaneously inoculated into nude mice to observe tumor growth. (**C**) Tumor images, (**D**) tumor volume, (**E**) tumor weight, (**F**) Hematoxylin-Eosin (H&E) staining of xenograft tissues and representative IHC images of UBE2L3, Ki-67 and PCNA in tissues; scale bar: 100 μm.. Error bars represent mean ± standard deviation (SD). ***$p < 0.001$.

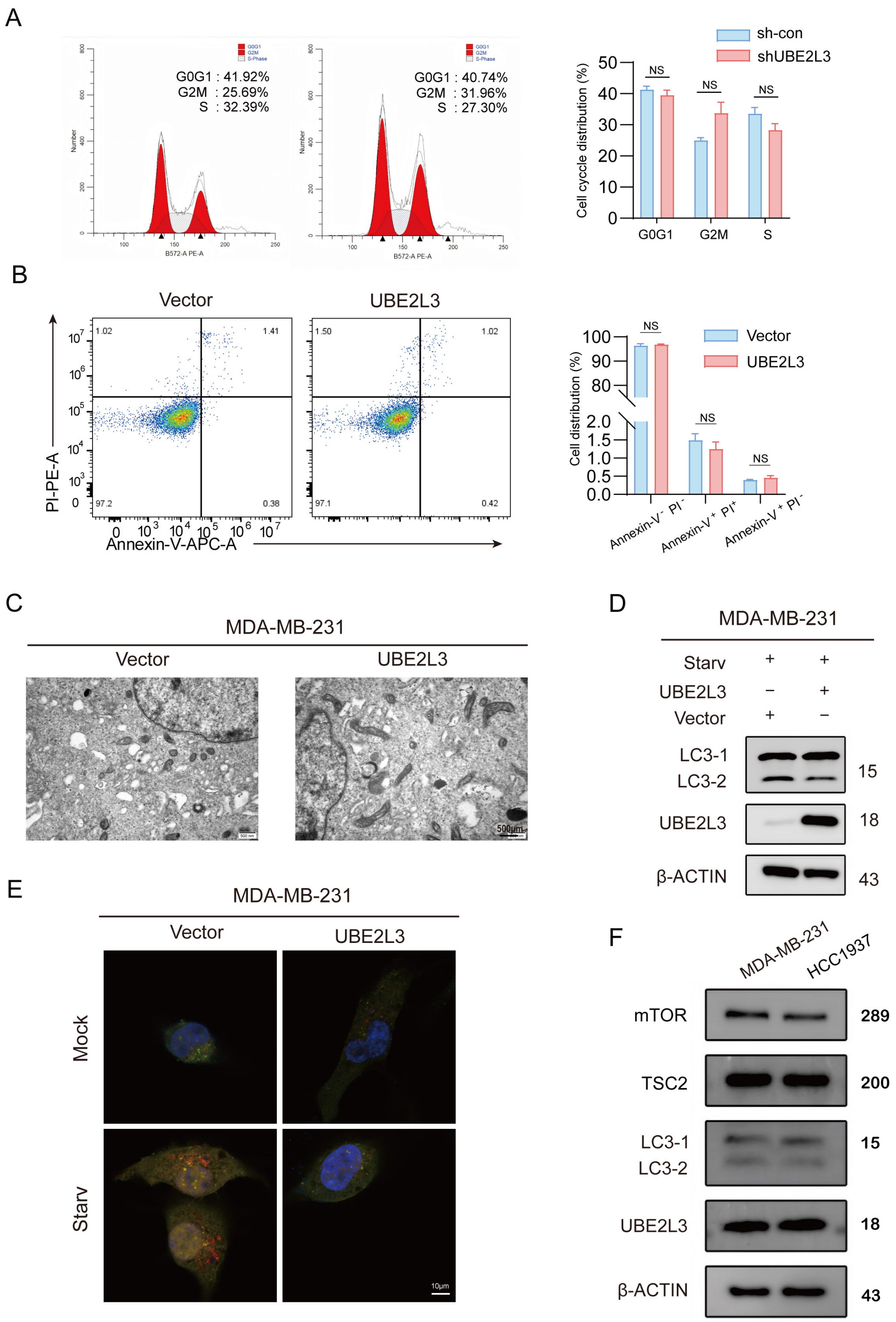

Figure S2. (**A**) Flow cytometry analysis of cell cycle distribution in MDA-MB-231 cells after UBE2L3 knockdown. (**B**) Annexin V-APC/PI staining and flow cytometry analysis of UBE2L3-overexpressing and control cells after 48 h of culture. (**C**) Representative transmission electron microscopy (TEM) images of autophagosomes in control and UBE2L3-overexpressing cells. Scale bar: 500 nm. (**D**) Western blot analysis of LC3

expression in control and UBE2L3-overexpressing cells under normal, starvation, and CQ-treated conditions. (**E**) Autophagic flux in control and UBE2L3-overexpressing cells detected by mCherry-EGFP-LC3B reporter analysis. Yellow puncta represent autophagosomes (mCherry+/EGFP+), and red puncta represent autolysosomes (mCherry+/EGFP−). Scale bar: 10 μm. (**F**) Western blot analysis was performed to determine the basal expression levels of the relevant proteins in the two cell lines. Error bars represent mean ± SD. not significant (NS), $p \geq 0.05$.

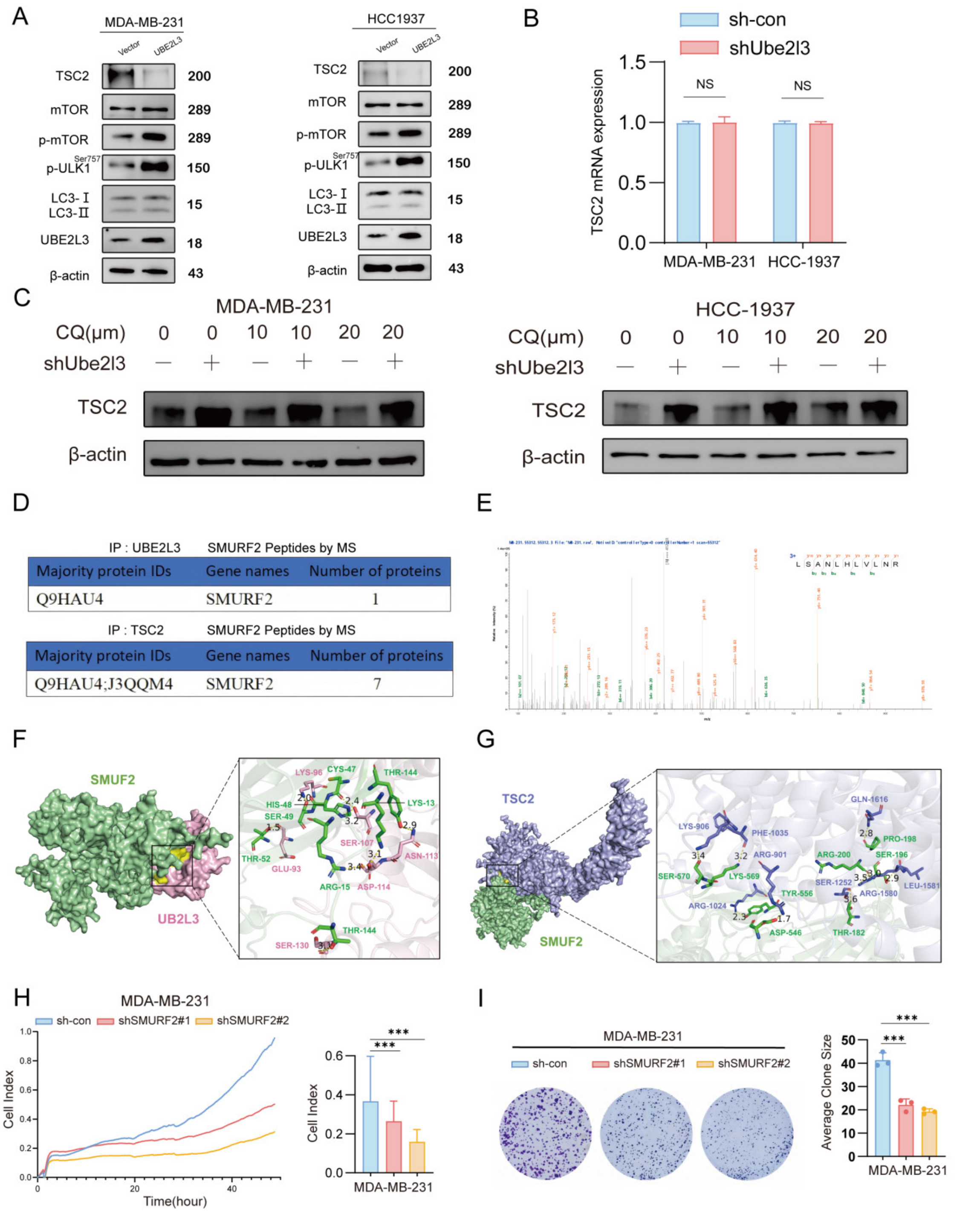

Figure S3. (**A**) Western blotting analysis of the effect of UBE2L3 overexpression on proteins related to the mTOR-regulated autophagic pathway in TNBC cells. (**B**) Quantitative real-time PCR (qRT-PCR) to detect the effect of UBE2L3 on TSC2 mRNA level. (**C**) Western blotting evaluation of changes in TSC2 protein levels in UBE2L3 knockdown and control TNBC cells treated with different concentrations of chloroquine (CQ) for 6 hours, respectively. (**D**) Co-immunoprecipitation (IP) of UBE2L3 and TSC2 followed by mass spectrometry (MS) analysis to identify SMURF2 as the target E3 ligase. (**E**) Secondary mass spectrometry images of unique peptides of SMURF2 from

co-immunoprecipitation experiments. (**F, G**) Molecular docking models of the interaction between UBE2L3 and SMURF2, and between TSC2 and SMURF2. (**H**) RTCA assay for measuring the growth rate of triple-negative breast cancer (TNBC) cells with SMURF2 knockdown. (**I**) Colony formation assay to detect the effect of SMURF2 knockdown on colony-forming ability. Error bars represent mean ± standard deviation (SD). ***p < 0.001; not significant (NS) p ≥ 0.05.

A

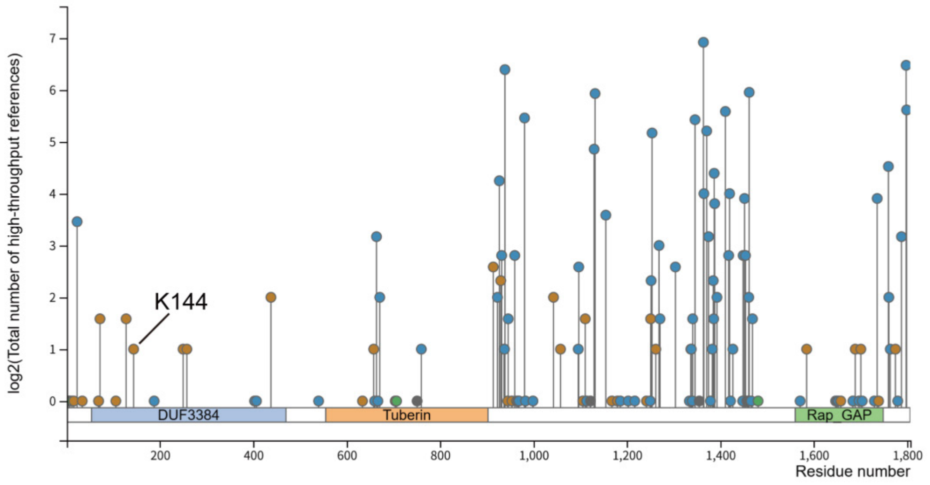

B

Ranking of Predicted Ubiquitination Sites in TSC2

Position	Code	Peptide	Score	Cutoff
634	K	DSLHRLGLPNKDGVVRFSPYC	0.7468	0.2655
144	K	EDLHERLEVFKALTDNGRHIT	0.7391	0.2655
69	K	RMIGQICEVAKTKKFEEHAVE	0.7323	0.2655
258	K	NVKELCEPCWKLMRNLLGTHL	0.6990	0.2655
250	K	IVTLCRTINVKELCEPCWKLM	0.6989	0.2655
72	K	GOICEVAKTKKFEEHAVEALW	0.6856	0.2655
914	K	FRKDEVPFITKGLRSNVLLSF	0.6068	0.2655
128	K	VLRALFFKVIKDYPSNEDLHE	0.5727	0.2655
930	K	VLLSFDDTPEKDSFRARSTSL	0.5679	0.2655
106	K	EARHAVLALLKAIVQGOGERL	0.5515	0.2655

Figure S4. (**A, B**) Predicted potential ubiquitination sites on TSC2 catalyzed by SMURF2.

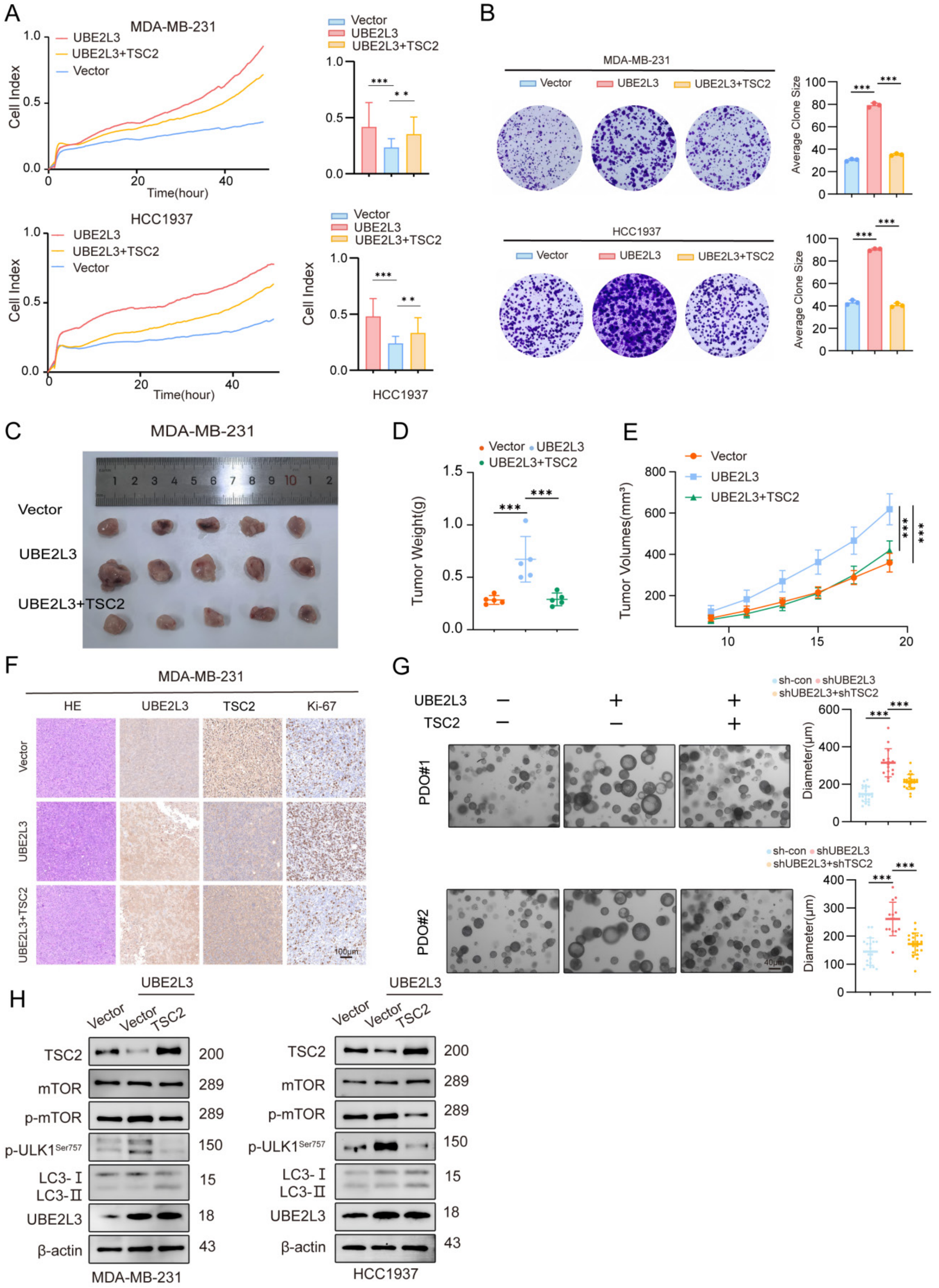

Figure S5. (A) RTCA assay for measuring the growth rate of the indicated cells. **(B)** Colony formation assay to detect the effect of TSC2 overexpression on colony-forming ability in UBE2L3-overexpressing cells. The indicated cells were subcutaneously inoculated into nude mice, and then tumor growth was monitored. **(C)** Tumor images, **(D)** tumor weight, **(E)** tumor volume, **(F)** Hematoxylin-Eosin (H&E) staining of xenograft

tissues and representative IHC images of UBE2L3, TSC2 and Ki-67 in tissues. **(G)** Representative images of organoid diameter and Ki-67 immunofluorescence intensity after TSC2 overexpression in UBE2L3-overexpressing organoids; scale bar: 40 μm. **(H)** Western blotting detection of the effect of TSC2 overexpression on proteins related to the mTOR-regulated autophagic pathway in UBE2L3-overexpressing TNBC cells. Error bars represent mean ± standard deviation (SD). ***p < 0.001; **p < 0.01.

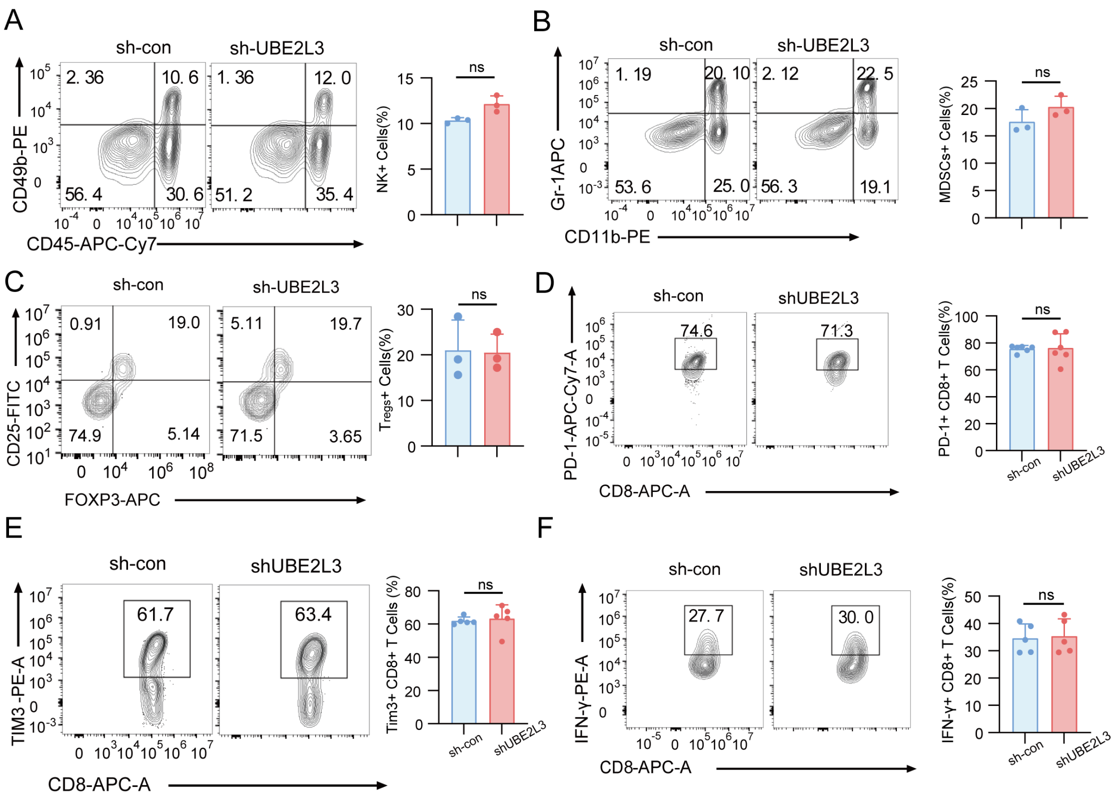

Figure S6. Control and UBE2L3-knockdown 4T1 tumors were collected from BALB/c mice for flow cytometry analysis: (**A**) NK$^+$ cell, (**B**) MDSCs cell, (**C**) Tregs cells, (**D**) PD-1$^+$ cells among CD8$^+$ T cells, (**E**) TIM-3$^+$ cells among CD8$^+$ T cells, (**F**) IFN-γ$^+$ cells among CD8$^+$ T cells. Error bars represent mean ± SD. not significant (NS), p ≥ 0.05.

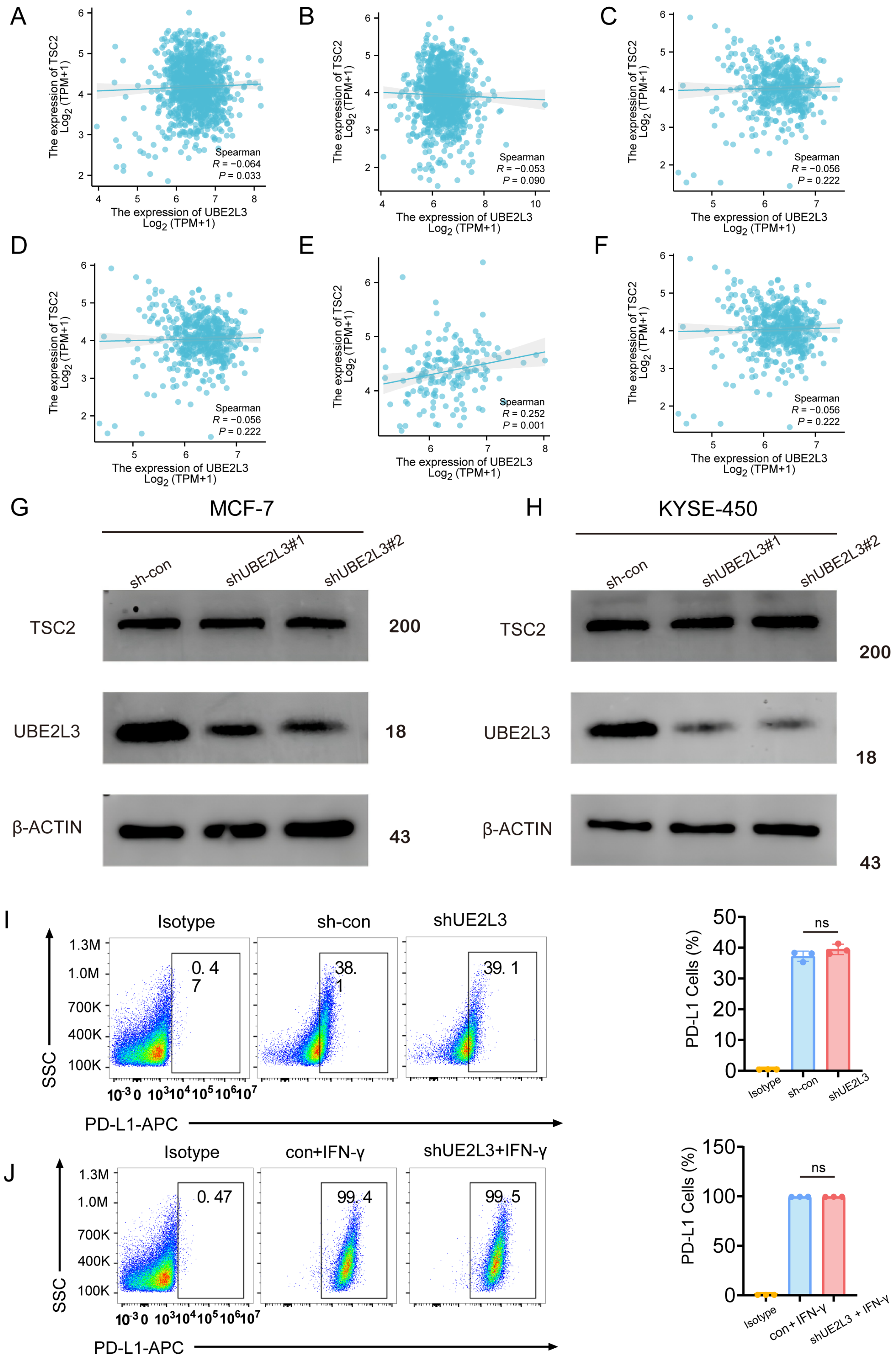

Figure S7. Correlation between UBE2L3 and TSC2 expression in different tumor types from the TCGA dataset: **(A)** Breast cancer, **(B)** Lung cancer, **(C)** Hepatocellular carcinoma, **(D)** Colon cancer, **(E)** Esophageal cancer, **(F)** Head and neck squamous cell carcinoma. Expression levels of UBE2L3 and TSC2 in different tumor types were

analyzed by Western blot: **(G)** Breast cancer, **(H)** Esophageal cancer. **(I)** Flow cytometry analysis of PD-L1 expression on the surface of 4T1 cells in different groups. **(J)** Flow cytometry analysis of PD-L1 expression on the surface of 4T1 cells in different groups following IFN-γ stimulation. Error bars represent mean ± SD. not significant (NS), $p \geq 0.05$.

Table S1 . sgRNA library annotation.
Table S2 . Rank of genes in sgRNA library.
Table S3 . Differentially expressed proteins in proteomic sequencing.
Table S4 . Sequences of shRNA used in this study.
Table S5 . Sequences of primer used in this study.

Table S4 Sequences of shRNA used in this study

Definition	Sequences
shUBE2L3#1	gatcgCCAGCAGAGTACCCATTCAAACTCGAGTTTGAATGGGTACTCTGCTGGTTTTTT
shUBE2L3#2	gatcgCCACCGAAGATCACATTTAAACTCGAGTTTAAATGTGATCTTCGGTGGTTTTT
shTSC2#1	gatcgGAGGGTAAACAGACGGAGTTTCTCGAGAAACTCCGTCTGTTTACCCTCTTTTTT
shTSC2#2	gatcgCAATGAGTCACAGTCCTTTGACTCGAGTCAAAGGACTGTGACTCATTGTTTTTT
shTSC2#3	gatcgCAGCATTAATCTCTTACCATACTCGAGTATGGTAAGAGATTAATGCTGTTTTTT

Table S5 Sequences of primer used in this study

Gene	Primer sequences
UBE2L3	F: ACGAACTCGgaattcgccaccatggcggccagcagga
	R: cgctgccGCTAGCTCTAGAgtccacaggtcgcttttcc
TSC2	F: ACGAACTCGgaattcgccaccatggaatgtggcctcaacaatcg
	R: cgctgccGCTAGCTCTAGAcacaaactcggtgaagtcctcca
SMURF2	F: ACGAACTCGgaattcgccaccATGTCTAACCCCGGAGGCC
	R: cgctgccGCTAGCTCTAGACTCGAGTTCCACAGCAAATCCACA
Ubiquitin	F: ACGAACTCGgaattcgccaccATGCAGATCTTCGTCAAGACGTTA
	R: cgctgccGCTAGCTCTAGAACCACCTCTGAGACGGAGGA
