# Supplementary material for: In vivo CRISPR/Cas9 Screening Reveals that UBE2L3 Modulates Autophagic Flux through TSC2 Ubiquitination and Potentiates PD-1 Blockade in Triple-Negative Breast Cancer
Source: Int J Biol Sci. 2026 Feb 18;22(6):2950–69. doi: 10.7150/ijbs.124937 (PMC13050453; doi:10.7150/ijbs.124937)
Supplement: Supplementary file 4 — Supplementary table 3. [file ijbsv22p2950s4.docx]

| **Supplementary Table S3.** Differentially expressed proteins in proteomic sequencing | | |
| --- | --- | --- |
| **Gene symbol** | **lfc** | **p-value** |
| ST8SIA6 | 8.506338028 | 3.52709E-06 |
| COL6A2 | 8.475198167 | 0.046493786 |
| OGN | 7.673114038 | 0.045794586 |
| FMOD | 7.567403437 | 0.000481424 |
| SPTB | 6.85090398 | 0.001742903 |
| CELA3A | 6.565553626 | 0.012665032 |
| OLAH | 6.401790812 | 0.001313662 |
| CYP2J2 | 6.287910805 | 0.000747258 |
| CTSH | 6.204613644 | 0.045054607 |
| SIGIRR | 6.187072252 | 0.002210201 |
| AMZ2 | 6.00168216 | 3.5833E-05 |
| SLC35C1 | 5.959061071 | 0.002297574 |
| PTGFRN | 5.935352657 | 0.004154052 |
| TNS1 | 5.91849947 | 0.006144573 |
| PUS10 | 5.860041991 | 0.017624855 |
| OAS3 | 5.859084983 | 0.000106443 |
| SYBU | 5.751625217 | 1.24731E-06 |
| IFIH1 | 5.633533778 | 1.02114E-05 |
| APOL2 | 5.399501792 | 0.035505139 |
| WBP1L | 5.246992864 | 0.000161074 |
| MANBA | 5.170200647 | 0.028417806 |
| SOCS2 | 5.07386091 | 0.000707586 |
| DGCR6L | 5.052385365 | 3.07064E-05 |
| ABCG2 | 5.024416815 | 0.00731633 |
| CAMTA2 | 4.714280151 | 0.011792179 |
| PABIR2 | 4.459365635 | 0.001188524 |
| SESTD1 | 4.110697871 | 0.025898592 |
| NCF2 | 3.910992725 | 0.018082906 |
| KIZ | 3.05710508 | 0.002797192 |
| BATF3 | 2.911816449 | 0.014952163 |
| OASL | 1.230676716 | 0.042704655 |
| C1orf131 | 1.199679852 | 0.00783048 |
| ASNS | 1.148931885 | 0.024313264 |
| TSC2 | 0.994525349 | 0.003892229 |
| UBE2G2 | 0.982648284 | 0.034915626 |
| RND3 | 0.976329129 | 0.015380783 |
| C18orf32 | 0.958076869 | 0.0158141 |
| KLHL18 | 0.916533689 | 0.024825345 |
| CYP1A1 | 0.913918852 | 0.036092407 |
| EIF5A2 | 0.906714788 | 0.021044781 |
| IFIT2 | 0.823736589 | 0.013546935 |
| NNMT | 0.823050392 | 0.031120994 |
| KIAA0319L | 0.818195296 | 0.017030678 |
| GAS2L1 | 0.765447782 | 0.028981605 |
| IFIT1 | 0.731339056 | 0.032403809 |
| DRAP1 | 0.704603503 | 9.86931E-05 |
| NOCT | 0.696098582 | 0.005224135 |
| ATP2A1 | 0.677313731 | 0.041246148 |
| ZRANB2 | 0.673712072 | 0.004643815 |
| FAIM | 0.672096872 | 0.043521193 |
| YAF2 | 0.657947043 | 0.049437167 |
| ARMC5 | 0.641383445 | 0.031748959 |
| IFIT3 | 0.640981953 | 0.007547555 |
| SLC2A3 | 0.630960542 | 0.04982466 |
| POLR3H | 0.613601376 | 0.033105106 |
| HMGA2 | 0.607199103 | 0.017805155 |
| DMAC2L | 0.595426668 | 0.008087018 |
| TCIRG1 | 0.566513887 | 0.008793273 |
| EIF1AD | 0.557773007 | 0.008321956 |
| IFRD2 | 0.540658515 | 0.039992931 |
| CHFR | 0.535298439 | 0.014907096 |
| POLR3G | 0.53507903 | 0.049497348 |
| TIMM10B | 0.528124059 | 0.026554838 |
| SCO2 | 0.522012091 | 0.043459878 |
| DNAJB2 | 0.505076194 | 0.003394227 |
| DAXX | 0.473326563 | 0.004588469 |
| RPLP1 | 0.470663389 | 0.002750064 |
| IQCB1 | 0.46538685 | 0.042675161 |
| RAB27B | 0.459179204 | 0.035902244 |
| TRIP12 | 0.456024663 | 0.010028192 |
| C19orf25 | 0.449738563 | 0.040246736 |
| MTM1 | 0.446749591 | 0.043600628 |
| TMEM219 | 0.438712281 | 0.036825794 |
| TRAPPC2 | 0.436968354 | 0.046150583 |
| SLC19A2 | 0.435525632 | 0.010926757 |
| PNISR | 0.428961313 | 0.010661973 |
| ZBTB43 | 0.425124942 | 0.034438265 |
| C9orf72 | 0.424729888 | 0.021551896 |
| HNRNPA1L2 | 0.41421778 | 0.042527132 |
| EDRF1 | 0.413539719 | 0.017785163 |
| RTN3 | 0.412724849 | 0.038337061 |
| DOCK6 | 0.408997759 | 0.009029397 |
| KPNA5 | 0.402835613 | 0.005743791 |
| CCDC32 | 0.401830627 | 0.029132841 |
| TOR2A | 0.399663639 | 0.035890595 |
| MPDU1 | 0.393871055 | 0.044295283 |
| YJU2 | 0.384878788 | 0.010792471 |
| TNFRSF10A | 0.384846768 | 0.038427128 |
| TUT1 | 0.377671717 | 0.017688083 |
| GET3 | 0.373874545 | 0.041841899 |
| ARID4A | 0.371815487 | 0.027457509 |
| LDLRAD3 | 0.367323614 | 0.022738793 |
| TRIR | 0.365514004 | 0.036696919 |
| CCDC51 | 0.36024138 | 0.030604241 |
| DYRK1A | 0.358736211 | 0.041867614 |
| GSE1 | 0.356482251 | 0.007675774 |
| PRKRIP1 | 0.353463127 | 0.006352887 |
| DCAF16 | 0.348659738 | 0.044436499 |
| POLR2I | 0.347550668 | 0.001151711 |
| CZIB | 0.345607666 | 0.018333969 |
| GCFC2 | 0.321877373 | 0.01211654 |
| PNMA2 | 0.31231018 | 0.008169233 |
| CCNE2 | 0.299186986 | 0.004002037 |
| KMT2C | 0.295449994 | 0.041691511 |
| TMEM115 | 0.29452699 | 0.01417861 |
| MOSPD2 | 0.2929111 | 0.015877862 |
| RAB11FIP1 | 0.28782999 | 0.041117852 |
| HECTD1 | 0.281846755 | 0.01240381 |
| ZNF689 | 0.278782567 | 0.002645273 |
| HDDC3 | 0.276258381 | 0.042856912 |
| CIBAR1 | 0.275383823 | 0.038345926 |
| KIF18A | 0.273328034 | 0.045222849 |
| CEP135 | 0.269665724 | 0.047182184 |
| GPAM | 0.251075894 | 0.032222657 |
| PRDM10 | 0.250174848 | 0.032394912 |
| ALG8 | 0.248282529 | 0.017100423 |
| NECAP1 | 0.241805506 | 0.028872577 |
| ACSL1 | 0.241374893 | 0.018010173 |
| TATDN1 | 0.227120524 | 0.047649038 |
| MRPS34 | 0.223377572 | 0.000592686 |
| NXN | 0.223107457 | 0.000517554 |
| CCNL1 | 0.22278435 | 0.03470821 |
| CNOT7 | 0.217188477 | 0.021610971 |
| NOP58 | 0.213831873 | 0.031142732 |
| SLC30A6 | 0.213568822 | 0.0390383 |
| NOP16 | 0.208357281 | 0.012280586 |
| TCEAL3 | 0.199499091 | 0.016523291 |
| VPS36 | 0.196579984 | 0.033010174 |
| TXNDC9 | 0.195909613 | 0.02911165 |
| EMC8 | 0.195191568 | 0.013249116 |
| SUPT6H | 0.189157191 | 0.030505727 |
| DDX51 | 0.188255589 | 0.015302494 |
| OGFOD1 | 0.185441765 | 0.025604404 |
| VRK2 | 0.175594363 | 0.03024359 |
| NDUFA8 | 0.168257095 | 0.00042898 |
| TRMT61A | 0.166621503 | 0.007742839 |
| TMEM68 | 0.162291007 | 0.02633359 |
| RABGEF1 | 0.161373931 | 0.031615373 |
| DCXR | 0.159936783 | 0.049108605 |
| CYB5A | 0.158926647 | 0.00930528 |
| CWC27 | 0.151329887 | 0.042141064 |
| TSG101 | 0.149530658 | 0.048642336 |
| GTF2F2 | 0.149433937 | 0.032001396 |
| PWP1 | 0.149430745 | 0.019720723 |
| HMG20A | 0.140126891 | 0.000967473 |
| GEMIN4 | 0.136193737 | 0.047541517 |
| KNL1 | 0.13424888 | 0.002853114 |
| SF3B2 | 0.133915153 | 0.004222705 |
| RIPK1 | 0.128559479 | 0.018838289 |
| GOSR1 | 0.125325029 | 0.036626553 |
| PARP2 | 0.124820025 | 0.003977146 |
| MRPS35 | 0.124487146 | 0.048624985 |
| INO80E | 0.113847738 | 0.035454011 |
| ZC3H15 | 0.113048766 | 0.037103513 |
| NUP42 | 0.108516873 | 0.001140421 |
| ZNF598 | 0.102648139 | 0.016443338 |
| CLASRP | 0.101515097 | 0.04579909 |
| COPS6 | 0.099909967 | 0.017741141 |
| TAOK2 | 0.09925694 | 0.015615981 |
| PPWD1 | 0.095409049 | 0.036103185 |
| SERPINH1 | 0.094879017 | 0.000620686 |
| BUD31 | 0.087767596 | 0.031317073 |
| PRPF6 | 0.071599585 | 0.005715711 |
| RREB1 | 0.07093919 | 0.044097451 |
| TPRKB | 0.063090678 | 0.015762476 |
| ILVBL | 0.055885388 | 0.026712098 |
| LTV1 | 0.052414871 | 0.047696002 |
| DDX52 | 0.045995883 | 0.009267902 |
| SENP8 | 0.029352036 | 0.03039292 |
| POLR1A | -0.060659554 | 0.036240891 |
| ZBTB21 | -0.070868411 | 0.046669085 |
| METTL3 | -0.076264425 | 0.027560112 |
| MECOM | -0.092717877 | 0.022361208 |
| AP4M1 | -0.094371825 | 0.004320049 |
| ZMYND8 | -0.096154653 | 0.036063716 |
| TRA2B | -0.10084912 | 0.028111039 |
| CNOT10 | -0.110273706 | 0.046111705 |
| PSME3IP1 | -0.112976046 | 0.00443074 |
| KATNAL1 | -0.11485644 | 0.008394551 |
| FTSJ1 | -0.122236696 | 0.044481819 |
| PRKAA2 | -0.123580446 | 0.006968149 |
| DCPS | -0.124696761 | 0.044790962 |
| RNF213 | -0.127523448 | 0.020307018 |
| LRRC47 | -0.128601517 | 0.020103611 |
| PHPT1 | -0.134892286 | 0.003013261 |
| DOCK7 | -0.142730873 | 0.036296033 |
| DDX39B | -0.144417472 | 0.018000624 |
| ABHD10 | -0.144768174 | 0.018203832 |
| METTL13 | -0.14638001 | 0.007390458 |
| TANC1 | -0.147620032 | 0.032302917 |
| GBF1 | -0.148172823 | 0.008036671 |
| PFDN5 | -0.148871161 | 0.027934603 |
| OGFRL1 | -0.149021873 | 0.042740053 |
| SPTLC1 | -0.151990962 | 0.038294515 |
| BRD4 | -0.152318782 | 0.039314332 |
| UBE2Z | -0.161754349 | 0.03747766 |
| NUP50 | -0.162101132 | 0.029958698 |
| MRE11 | -0.179573178 | 0.035334893 |
| CASP2 | -0.185528173 | 0.007680001 |
| PGP | -0.18557847 | 0.001532615 |
| POGLUT2 | -0.186851373 | 0.049676546 |
| DNAAF5 | -0.186969147 | 0.002836878 |
| BCR | -0.191884507 | 0.006581029 |
| PLEKHG3 | -0.192213118 | 0.035111124 |
| ATPAF2 | -0.194751026 | 0.031156345 |
| KIF22 | -0.196016938 | 0.012900977 |
| SEMA7A | -0.197488627 | 0.013827291 |
| HNRNPLL | -0.199216125 | 0.019893631 |
| RBM10 | -0.200975213 | 0.011977673 |
| DOCK1 | -0.206588611 | 0.046382835 |
| RIPOR1 | -0.210246701 | 0.029094392 |
| EPM2AIP1 | -0.210398365 | 0.002645714 |
| UBR3 | -0.210730333 | 0.006283515 |
| MCM7 | -0.212372456 | 0.009252383 |
| APOBEC3C | -0.219206971 | 0.024368608 |
| OSBPL10 | -0.220511732 | 0.045159414 |
| CCP110 | -0.22452524 | 0.037621423 |
| FRYL | -0.225167439 | 0.001006477 |
| METTL15 | -0.227343646 | 0.026181881 |
| RNASEH1 | -0.230671328 | 0.033418629 |
| EVI5L | -0.235466321 | 0.010323554 |
| COQ6 | -0.235806049 | 0.040783032 |
| TRIM26 | -0.237117309 | 0.02302755 |
| ACAD9 | -0.241945585 | 0.032949479 |
| MCM5 | -0.246199765 | 0.020431359 |
| G3BP1 | -0.249978512 | 0.012382069 |
| PPIG | -0.251201512 | 0.027490518 |
| SEPTIN10 | -0.251620898 | 0.017202512 |
| SMCHD1 | -0.260522439 | 0.031129378 |
| LIMD1 | -0.261998865 | 0.033288797 |
| SRR | -0.262464988 | 0.026029903 |
| MRPL28 | -0.265197735 | 0.012181351 |
| MBNL2 | -0.268895342 | 0.021803957 |
| CTPS2 | -0.27946707 | 0.0401022 |
| CGGBP1 | -0.280466745 | 0.045488235 |
| MPLKIP | -0.281036037 | 0.039155274 |
| CDC25C | -0.28137857 | 0.044910203 |
| TWF1 | -0.285034715 | 0.000859196 |
| PM20D2 | -0.287700653 | 0.032500565 |
| ARPC1B | -0.288021623 | 0.029048439 |
| SLC12A4 | -0.288747278 | 0.042947756 |
| MAGI1 | -0.288870874 | 0.017069489 |
| CERT1 | -0.291479408 | 0.034766655 |
| DOCK5 | -0.294272063 | 0.03140824 |
| TBC1D1 | -0.29520297 | 0.039867159 |
| THOC3 | -0.296318708 | 0.032683851 |
| MB21D2 | -0.296623797 | 0.007834742 |
| SLC7A11 | -0.297052882 | 0.023270672 |
| SIMC1 | -0.301978622 | 0.023605096 |
| NHSL3 | -0.302326843 | 0.029613377 |
| AASS | -0.302502124 | 0.007902305 |
| INPP1 | -0.304028602 | 0.023629135 |
| PTOV1 | -0.307590019 | 0.000920652 |
| ABI2 | -0.309156266 | 0.0081186 |
| HAUS3 | -0.31039743 | 0.040944732 |
| TENT2 | -0.31167001 | 0.016211038 |
| USP48 | -0.313090031 | 0.034025637 |
| ACVR1 | -0.314116435 | 0.019239656 |
| TNFAIP8 | -0.316213661 | 0.031673352 |
| SP2 | -0.322643102 | 0.049215193 |
| GORASP1 | -0.324521589 | 0.010838776 |
| MTMR2 | -0.32643024 | 0.003850715 |
| ORC4 | -0.326615045 | 0.001192026 |
| KLHDC10 | -0.33032843 | 0.023411621 |
| ASTE1 | -0.333390218 | 0.003883278 |
| PSMD10 | -0.337596232 | 0.038547917 |
| WDR33 | -0.342980294 | 0.028861833 |
| FAM120A | -0.344194318 | 0.036393018 |
| COX16 | -0.350476446 | 0.006747247 |
| ABCC10 | -0.350802975 | 0.002888015 |
| PAK4 | -0.350903998 | 0.014658297 |
| TUBGCP2 | -0.351043974 | 0.040663548 |
| C5orf24 | -0.35341765 | 0.049521263 |
| PSMD1 | -0.353602365 | 0.014080462 |
| ARMC10 | -0.354259384 | 0.026044542 |
| HNRNPA1 | -0.354900868 | 0.03088273 |
| ITM2B | -0.356781441 | 0.033907972 |
| CIAO1 | -0.358776162 | 0.015104001 |
| DBN1 | -0.359071192 | 0.011803768 |
| ATXN7L3 | -0.363115715 | 0.046719167 |
| ABHD12 | -0.36618742 | 0.027228272 |
| PXK | -0.368708288 | 0.03818287 |
| RAP2B | -0.36926055 | 0.020124996 |
| PIGS | -0.369365105 | 0.022920471 |
| SNTB1 | -0.369973767 | 0.01939218 |
| STARD7 | -0.370524739 | 0.04058555 |
| CDC27 | -0.371316041 | 0.023961852 |
| GULP1 | -0.373390837 | 0.006487966 |
| FDPS | -0.375167331 | 0.039785415 |
| NOA1 | -0.375546376 | 0.007184089 |
| EML3 | -0.381001406 | 0.02108907 |
| CCDC25 | -0.381149398 | 0.025852743 |
| STEAP1 | -0.38348543 | 0.0004316 |
| TP53 | -0.388617665 | 0.01530996 |
| PCGF6 | -0.395122562 | 0.031659473 |
| STRIP1 | -0.395595159 | 0.01340116 |
| PCNX3 | -0.399289921 | 0.041893464 |
| ACTN4 | -0.400201584 | 0.019219471 |
| NKAP | -0.400986374 | 0.002868952 |
| AGFG2 | -0.404011317 | 0.011037289 |
| TFB1M | -0.408661613 | 0.045850814 |
| FLRT2 | -0.408806458 | 0.028846448 |
| SSH1 | -0.413601649 | 0.04685761 |
| PLEKHA6 | -0.416702673 | 0.038910794 |
| SMURF2 | -0.418203932 | 0.008468974 |
| NSDHL | -0.418228931 | 0.039461434 |
| RBMS2 | -0.421369471 | 0.004095361 |
| PCLAF | -0.421653488 | 0.003587212 |
| ARMCX2 | -0.422596296 | 0.002426755 |
| SH3D19 | -0.423423294 | 0.006868821 |
| ZNF143 | -0.427043601 | 0.036160282 |
| ELF1 | -0.4271601 | 0.027574892 |
| SMARCD1 | -0.430367857 | 0.00724037 |
| TBC1D5 | -0.436025652 | 0.027036307 |
| TARS3 | -0.439031092 | 0.024567463 |
| DPYD | -0.440266224 | 0.037325851 |
| ACACA | -0.440390545 | 0.043736048 |
| TUBGCP6 | -0.445537993 | 0.014958945 |
| TPCN1 | -0.446254378 | 0.019963055 |
| GARRE1 | -0.450990815 | 0.049207714 |
| SVIL | -0.451630237 | 0.01339598 |
| FADS2 | -0.454414902 | 0.04222148 |
| ABCC1 | -0.455651852 | 0.041828126 |
| DHCR24 | -0.456564083 | 0.012541171 |
| ITGB5 | -0.458547267 | 0.002102178 |
| TDRKH | -0.459459579 | 0.047798347 |
| TTC28 | -0.45972566 | 0.033505483 |
| KIAA0513 | -0.465425878 | 0.025384492 |
| PXN | -0.465578546 | 0.047693264 |
| TRMT1L | -0.467067329 | 0.04426642 |
| GATAD1 | -0.470210434 | 0.024561196 |
| KDM1B | -0.471039007 | 0.044172065 |
| WDCP | -0.47548345 | 0.045432456 |
| WWTR1 | -0.479520296 | 0.000968277 |
| POLK | -0.480447862 | 0.034072496 |
| IBTK | -0.496866189 | 0.038365118 |
| TRIM8 | -0.512512782 | 0.006702533 |
| CLCN4 | -0.522421677 | 0.017294813 |
| PC | -0.528667394 | 0.027241784 |
| NES | -0.529179064 | 0.00659998 |
| HDAC4 | -0.529210814 | 0.040466214 |
| JPT2 | -0.530576517 | 0.024199958 |
| CAMK2G | -0.54165788 | 0.022326459 |
| ACTN2 | -0.544047997 | 0.021747129 |
| ATP11C | -0.551741281 | 0.014853424 |
| STC1 | -0.564130629 | 0.032441846 |
| MFSD14B | -0.580605926 | 0.045658338 |
| GINS3 | -0.581688519 | 0.03602603 |
| INSYN2B | -0.581973135 | 0.000602616 |
| PTBP2 | -0.582374311 | 0.011139562 |
| TMEM237 | -0.603886507 | 0.015344986 |
| PDLIM2 | -0.617442881 | 0.044493726 |
| SHKBP1 | -0.622763944 | 0.03401263 |
| ALDH3A2 | -0.647415181 | 0.041358779 |
| HSF2 | -0.651599928 | 0.020369866 |
| SHROOM2 | -0.653032316 | 0.048483579 |
| TMED7 | -0.667719618 | 0.027918587 |
| NHSL1 | -0.683018734 | 0.030024876 |
| GEMIN6 | -0.695273051 | 0.014465658 |
| FBXO3 | -0.729240918 | 0.037325495 |
| UBE2E1 | -0.743094857 | 0.00388338 |
| ZNF184 | -0.814986681 | 0.006899158 |
| STK17B | -0.857256862 | 0.012197476 |
| NAT14 | -0.865713524 | 0.012070379 |
| SLC37A2 | -0.896038703 | 0.031604437 |
| PCDHA12 | -0.94545818 | 0.002034679 |
| TBP | -0.965561927 | 0.001979015 |
| CREB1 | -0.994823239 | 0.012098777 |
| MOCS2 | -1.004067119 | 0.010327415 |
| CRIM1 | -1.025402678 | 0.041512356 |
| TIMP3 | -1.069057551 | 0.003376359 |
| UNKL | -1.075420815 | 0.020567675 |
| LOXL2 | -1.131869786 | 0.006442559 |
| BCL10 | -1.288756742 | 0.018843923 |
| GPR17 | -1.330858714 | 0.020203485 |
| NEFM | -1.466698353 | 0.036354308 |
| UBE2L3 | -2.325046319 | 0.033408595 |
| SLF1 | -2.502308975 | 0.004185774 |
| TUBB8 | -3.175003943 | 0.003676957 |
| PTPN21 | -3.818859795 | 0.026057824 |
| CYP27A1 | -4.108042882 | 0.021787827 |
| WDR25 | -4.868272244 | 0.002236725 |
| LY6H | -5.099093603 | 0.001887044 |
| BTG3 | -5.540383558 | 0.008192845 |
| HPSE | -5.543491801 | 0.004800808 |
| GRM3 | -5.65760321 | 0.011002562 |
| LRP6 | -6.033606969 | 0.001151792 |
| NLK | -6.192312108 | 0.006694436 |
| SNAP25 | -6.231724347 | 0.019572412 |
| PRKCB | -6.462738694 | 0.014151504 |
| SHC4 | -6.906465507 | 0.003205926 |
| TK2 | -7.059667381 | 0.002665725 |
| CD163L1 | -7.179524111 | 7.35072E-05 |
| LOX | -7.231591172 | 0.001059882 |
| MTMR10 | -7.543458981 | 0.000609905 |
| TGM1 | -7.602968533 | 0.045892522 |
| PIP | -7.878215574 | 0.004261311 |
| DUSP6 | -8.057717534 | 4.30686E-06 |
| KPRP | -8.062680657 | 0.003874482 |
| CNTNAP2 | -8.394365431 | 0.000488628 |
