# Supplementary material for: In vivo CRISPR/Cas9 Screening Reveals that UBE2L3 Modulates Autophagic Flux through TSC2 Ubiquitination and Potentiates PD-1 Blockade in Triple-Negative Breast Cancer
Source: Int J Biol Sci. 2026 Feb 18;22(6):2950–69. doi: 10.7150/ijbs.124937 (PMC13050453; doi:10.7150/ijbs.124937)
Supplement: Supplementary file 3 — Supplementary table 2. [file ijbsv22p2950s3.docx]

| **Supplementary Table S2. rank** of genes in sgRNA library. | | | | |
| --- | --- | --- | --- | --- |
| **Gene symbol** | **neg\|score** | **neg\|lfc** | **neg\|rank** | |
| Ears2 | 7.2919e-07 | -7.164 | 1 | |
| Vmn1r177 | 7.5674e-06 | -4.809 | 2 | |
| Oraov1 | 8.8534e-06 | -5.4974 | 3 | |
| Iars2 | 1.0224e-05 | -5.1902 | 4 | |
| Mars2 | 1.0925e-05 | -5.7626 | 5 | |
| Psmb4 | 1.4026e-05 | -5.2536 | 6 | |
| Ube2l3 | 2.0065e-05 | -5.6037 | 7 | |
| Hdac3 | 2.0386e-05 | -4.5725 | 8 | |
| Taf2 | 2.1492e-05 | -4.6605 | 9 | |
| Copg1 | 2.2758e-05 | -3.7424 | 10 | |
| Ccnh | 2.4701e-05 | -5.7514 | 11 | |
| Rps5 | 2.7941e-05 | -4.6331 | 12 | |
| Rpl7a | 3.3965e-05 | -5.0817 | 13 | |
| Thoc3 | 3.7673e-05 | -4.0853 | 14 | |
| Lsm10 | 4.1181e-05 | -5.9269 | 15 | |
| Ddx10 | 4.145e-05 | -5.2061 | 16 | |
| Eftud2 | 4.1915e-05 | -4.5613 | 17 | |
| Eef1a1 | 4.3051e-05 | -4.5613 | 18 | |
| Rpl35a | 4.386e-05 | -5.9197 | 19 | |
| Upf1 | 4.7821e-05 | -6.1976 | 20 | |
| 1810026J23Rik | 5.7776e-05 | -4.4069 | 21 | |
| Dbr1 | 5.7776e-05 | -4.9765 | 22 | |
| Prim1 | 6.1563e-05 | -4.6605 | 23 | |
| Kars | 6.2454e-05 | -4.3256 | 24 | |
| Polr2d | 6.6965e-05 | -4.425 | 25 | |
| Aldoa | 6.6965e-05 | -5.1018 | 26 | |
| Ssrp1 | 6.6965e-05 | -4.2378 | 27 | |
| Hmgcs1 | 6.8021e-05 | -4.6875 | 28 | |
| Strip1 | 6.8089e-05 | -6.029 | 29 | |
| Gm4975 | 6.8273e-05 | 0.0013896 | 30 | |
| Emg1 | 7.1179e-05 | -5.2892 | 31 | |
| Ddx47 | 7.2081e-05 | -4.6051 | 32 | |
| Chaf1b | 7.5728e-05 | -4.2554 | 33 | |
| Tuba1b | 7.8032e-05 | -4.1619 | 34 | |
| Birc5 | 8.2607e-05 | -4.219 | 35 | |
| Arf1 | 8.4171e-05 | -5.1902 | 36 | |
| Naa20 | 8.7896e-05 | -4.1767 | 37 | |
| Ect2 | 8.9526e-05 | -4.6592 | 38 | |
| D2Wsu81e | 9.2892e-05 | -4.2853 | 39 | |
| Snrpd2 | 9.7977e-05 | -4.3926 | 40 | |
| Gpn1 | 9.7977e-05 | -3.9344 | 41 | |
| Eif3a | 0.00010445 | -4.5766 | 42 | |
| Racgap1 | 0.00010633 | -4.1432 | 43 | |
| Vmn1r73 | 0.00011379 | -1.9037 | 44 | |
| Eif2b5 | 0.00011423 | -3.8416 | 45 | |
| Sharpin | 0.00011655 | -4.4667 | 46 | |
| Cds2 | 0.00011753 | -4.3428 | 47 | |
| Ppp4c | 0.00012275 | -5.6321 | 48 | |
| Slc25a26 | 0.00013078 | -5.5895 | 49 | |
| Rps15 | 0.00013528 | -4.9095 | 50 | |
| Rps25 | 0.00013631 | -4.4025 | 51 | |
| Med14 | 0.00013767 | -5.9083 | 52 | |
| Pomp | 0.00013767 | -5.9083 | 53 | |
| Gbf1 | 0.00014523 | -4.2462 | 54 | |
| Znrd1 | 0.00014671 | -5.8853 | 55 | |
| Tprkb | 0.00014707 | -3.9606 | 56 | |
| Psmb6 | 0.00014707 | -4.3164 | 57 | |
| Spin2d | 0.0001563 | -5.1993 | 58 | |
| Cops3 | 0.0001593 | -1.0698 | 59 | |
| Cct8 | 0.0001618 | -3.6359 | 60 | |
| Psma4 | 0.0001618 | -3.6733 | 61 | |
| Nop58 | 0.00016671 | -4.291 | 62 | |
| Polr1b | 0.00017075 | -5.838 | 63 | |
| Rangap1 | 0.00017075 | -5.838 | 64 | |
| Esf1 | 0.00018575 | -5.8015 | 65 | |
| Rps27rt | 0.00018575 | -5.8015 | 66 | |
| Phb2 | 0.00019051 | -4.291 | 67 | |
| Rps28 | 0.00019492 | -4.7398 | 68 | |
| A530032D15Rik | 0.000199 | -3.9199 | 69 | |
| Hscb | 0.00020436 | -5.4616 | 70 | |
| Hist3h2bb-ps | 0.00020481 | -1.2017 | 71 | |
| Wdr3 | 0.00020519 | -5.7126 | 72 | |
| Atp5b | 0.00021534 | -5.3061 | 73 | |
| Mrpl21 | 0.0002266 | -5.5266 | 74 | |
| Sec61a2 | 0.00022976 | -3.4831 | 75 | |
| Espl1 | 0.00023365 | -3.7697 | 76 | |
| Rpl18a | 0.00023365 | -3.9787 | 77 | |
| Tsg101 | 0.00023365 | -5.4393 | 78 | |
| Pabpn1 | 0.00023365 | -4.2894 | 79 | |
| Erdr1 | 0.00023677 | -3.6666 | 80 | |
| Ruvbl1 | 0.00024684 | -3.3959 | 81 | |
| Mtg1 | 0.00024918 | -5.2916 | 82 | |
| Gm16381 | 0.00025032 | -3.4597 | 83 | |
| Cdipt | 0.00025691 | -4.2554 | 84 | |
| Taf11 | 0.00026135 | -3.9423 | 85 | |
| Nuf2 | 0.00026444 | -4.9095 | 86 | |
| Elac2 | 0.00026556 | -4.9519 | 87 | |
| Tyms | 0.00027239 | -3.9762 | 88 | |
| Rragc | 0.00027623 | -4.3164 | 89 | |
| Gm5150 | 0.00027624 | -5.3356 | 90 | |
| Gm2913 | 0.00029017 | -3.3291 | 91 | |
| Cenpn | 0.00029583 | -4.557 | 92 | |
| Pop1 | 0.00029746 | -5.6178 | 93 | |
| Dolk | 0.00029746 | -5.6178 | 94 | |
| Smc6 | 0.00030933 | -3.3918 | 95 | |
| Tll1 | 0.00031112 | -5.6037 | 96 | |
| Rpl38 | 0.0003133 | -3.4345 | 97 | |
| Psma5 | 0.00032349 | -4.5475 | 98 | |
| Rps19 | 0.0003387 | -5.1018 | 99 | |
| Tubgcp2 | 0.00034134 | 0.015502 | 100 | |
| Sept7 | 0.0003414 | -3.9344 | 101 | |
| Pold2 | 0.00034541 | -3.8416 | 102 | |
| Ly96 | 0.00034952 | -5.3668 | 103 | |
| Adar | 0.00035263 | -3.3733 | 104 | |
| Olfr744 | 0.00035491 | -3.212 | 105 | |
| Ddx21 | 0.00036458 | -4.6339 | 106 | |
| Rnf31 | 0.00036901 | -5.5164 | 107 | |
| Eif3i | 0.0003698 | -5.5461 | 108 | |
| Prpf4 | 0.00037888 | -4.291 | 109 | |
| Polr2a | 0.00037989 | -3.0935 | 110 | |
| Wdr36 | 0.00038468 | -4.4566 | 111 | |
| Olfr142 | 0.00038684 | -2.4579 | 112 | |
| Cit | 0.00038855 | -3.3723 | 113 | |
| Kansl2 | 0.00039116 | -5.5164 | 114 | |
| Pop5 | 0.00040416 | -5.5013 | 115 | |
| Zfp259 | 0.00040416 | -5.5013 | 116 | |
| Rpl34-ps1 | 0.00042755 | -5.486 | 117 | |
| Tdrd12 | 0.00043235 | -4.109 | 118 | |
| Rps24 | 0.00043788 | -4.7933 | 119 | |
| Tfb2m | 0.00044223 | -5.341 | 120 | |
| Rad51 | 0.00046498 | -5.4393 | 121 | |
| Pabpc1 | 0.00046507 | -3.3285 | 122 | |
| Spc24 | 0.00047119 | -5.2372 | 123 | |
| Ppp2r1a | 0.0004747 | -4.9567 | 124 | |
| Gtf3a | 0.00047785 | -1.1944 | 125 | |
| Nup205 | 0.00049279 | -2.9307 | 126 | |
| Rpl31 | 0.00049749 | -3.3959 | 127 | |
| Rab7 | 0.00050207 | -3.5051 | 128 | |
| Npm1 | 0.00050984 | -5.4073 | 129 | |
| Atp2a2 | 0.00051089 | -4.0633 | 130 | |
| Kif23 | 0.00051089 | -4.3256 | 131 | |
| Eif2s3x | 0.00051089 | -4.425 | 132 | |
| mmu-mir-5118 | 0.00051089 | -3.1674 | 133 | |
| mmu-mir-3473a | 0.0005203 | -3.8396 | 134 | |
| Rrp12 | 0.00052226 | -5.0923 | 135 | |
| Polr2j | 0.00052226 | -5.0923 | 136 | |
| Tcp11l1 | 0.00052335 | -0.60594 | 137 | |
| Mastl | 0.00054027 | -5.3578 | 138 | |
| Mrpl12 | 0.00054027 | -5.391 | 139 | |
| Pyroxd1 | 0.00055948 | -3.258 | 140 | |
| Anapc10 | 0.00056118 | -3.2064 | 141 | |
| Clns1a | 0.00056335 | -4.6605 | 142 | |
| Ikbkg | 0.00056491 | -1.993 | 143 | |
| Npat | 0.00056785 | -5.3745 | 144 | |
| Nelfcd | 0.00057052 | -3.01 | 145 | |
| mmu-mir-466d | 0.00057366 | -2.8825 | 146 | |
| Lrr1 | 0.00057618 | -4.1101 | 147 | |
| Cdc16 | 0.00058725 | -5.3578 | 148 | |
| 1700010D01Rik | 0.00059357 | -4.1816 | 149 | |
| Gtpbp10 | 0.00061404 | -4.9216 | 150 | |
| Mcm4 | 0.00061434 | -3.4737 | 151 | |
| Ankef1 | 0.00061435 | -0.95513 | 152 | |
| Pmpcb | 0.00062111 | -4.9273 | 153 | |
| Cdca8 | 0.00062293 | -3.653 | 154 | |
| Wdr55 | 0.000627 | -5.3239 | 155 | |
| Xrn1 | 0.00063419 | -5.0122 | 156 | |
| Erh | 0.00065655 | -3.9344 | 157 | |
| Tyw5 | 0.00065985 | -1.3974 | 158 | |
| Mrps16 | 0.00066588 | -2.9294 | 159 | |
| Uqcrb | 0.00066796 | -5.2715 | 160 | |
| Haus3 | 0.00067372 | -3.6646 | 161 | |
| Thoc1 | 0.00068657 | -4.425 | 162 | |
| Nsmaf | 0.00069439 | -3.2513 | 163 | |
| Tsr1 | 0.00070002 | -5.2715 | 164 | |
| Noc4l | 0.00070002 | -5.2715 | 165 | |
| Sars2 | 0.00070534 | -0.92021 | 166 | |
| BC049715 | 0.00072738 | -2.8769 | 167 | |
| Qrsl1 | 0.00074164 | -5.2239 | 168 | |
| Ccdc40 | 0.00074262 | -5.2172 | 169 | |
| Olfr473 | 0.00075084 | -0.828 | 170 | |
| Bank1 | 0.00075543 | -5.2355 | 171 | |
| Gnl2 | 0.00076116 | -4.9273 | 172 | |
| Arfrp1 | 0.0007633 | -2.7745 | 173 | |
| Dhps | 0.00077195 | -5.2355 | 174 | |
| Olfr769 | 0.00077195 | -5.2355 | 175 | |
| Hipk1 | 0.00077847 | -4.8739 | 176 | |
| Gm15455 | 0.00079633 | 0.37155 | 177 | |
| Ddx23 | 0.00080037 | -5.2172 | 178 | |
| Ak2 | 0.00080726 | -4.0413 | 179 | |
| Kif4 | 0.00081737 | -3.5795 | 180 | |
| 1110037F02Rik | 0.00081896 | -2.8768 | 181 | |
| Dcaf13 | 0.00082055 | -2.8922 | 182 | |
| Pak1ip1 | 0.00083455 | -5.1986 | 183 | |
| mmu-mir-1970 | 0.00083455 | -3.7408 | 184 | |
| Psmd4 | 0.00083945 | -2.7804 | 185 | |
| Wdhd1 | 0.00084182 | 0.28838 | 186 | |
| Ddx54 | 0.00085925 | -4.3256 | 187 | |
| Olfr918 | 0.0008675 | -3.1883 | 188 | |
| Cenpu | 0.0008725 | -5.1797 | 189 | |
| Mrps31 | 0.00088019 | -5.1608 | 190 | |
| Incenp | 0.00088327 | -2.6429 | 191 | |
| Oplah | 0.00088731 | -1.2049 | 192 | |
| Kdm3a | 0.00089956 | -4.9009 | 193 | |
| Bccip | 0.00090211 | -3.3959 | 194 | |
| Atp5a1 | 0.00091449 | -4.0469 | 195 | |
| Heatr1 | 0.00091599 | -4.8864 | 196 | |
| Mrp63 | 0.00091756 | -4.8465 | 197 | |
| Dars2 | 0.00092624 | -4.8372 | 198 | |
| Fam83a | 0.0009328 | -1.9357 | 199 | |
| Olfr1126 | 0.00094725 | -4.662 | 200 | |
| Nup93 | 0.0009479 | -2.7475 | 201 | |
| Uba52 | 0.00096026 | -4.331 | 202 | |
| Rpp21 | 0.00096026 | -2.9389 | 203 | |
| Hnrnpk | 0.00096026 | -3.3673 | 204 | |
| Rpl37a | 0.00096026 | -3.5795 | 205 | |
| Fbrs | 0.0009716 | -2.6798 | 206 | |
| Psg28 | 0.00097829 | -1.5657 | 207 | |
| Wdr92 | 0.0010036 | -5.1018 | 208 | |
| Brix1 | 0.0010052 | -4.0401 | 209 | |
| Hnrnpu | 0.0010062 | -3.259 | 210 | |
| Psmc3 | 0.0010427 | -4.277 | 211 | |
| Frs2 | 0.0010427 | -5.0817 | 212 | |
| Mga | 0.0010427 | -5.0817 | 213 | |
| Spin2-ps1 | 0.0010488 | -4.6598 | 214 | |
| Cyp7b1 | 0.0010507 | -3.5406 | 215 | |
| Rev3l | 0.0010748 | -3.2582 | 216 | |
| Ldlr | 0.0010927 | -5.0612 | 217 | |
| Znhit6 | 0.0011147 | -4.2757 | 218 | |
| Cul3 | 0.0011168 | -4.7311 | 219 | |
| Bicd1 | 0.0011184 | -2.5978 | 220 | |
| Mrpl20 | 0.0011241 | -3.7144 | 221 | |
| Cirh1a | 0.0011246 | -4.9447 | 222 | |
| Etf1 | 0.0011246 | -5.0405 | 223 | |
| Plcz1 | 0.0011419 | -2.532 | 224 | |
| Mrpl41 | 0.0011727 | -3.2341 | 225 | |
| Cct4 | 0.0011959 | -2.8463 | 226 | |
| Ctage5 | 0.0011998 | -4.7933 | 227 | |
| C130050O18Rik | 0.0012003 | -1.2803 | 228 | |
| Mrps14 | 0.0012057 | -1.9997 | 229 | |
| Prelid1 | 0.0012115 | -4.9981 | 230 | |
| Cdt1 | 0.0012115 | -4.9981 | 231 | |
| Tpt1 | 0.0012259 | -4.6801 | 232 | |
| Eif2s1 | 0.0012296 | -4.6463 | 233 | |
| Krr1 | 0.0012319 | -3.411 | 234 | |
| Nup155 | 0.0012319 | -2.6196 | 235 | |
| B2m | 0.0012419 | -3.1285 | 236 | |
| Chrna7 | 0.001246 | -2.086 | 237 | |
| Atf7 | 0.0012496 | -4.8988 | 238 | |
| Hbs1l | 0.0012512 | -3.7424 | 239 | |
| Gm2927 | 0.0012529 | -4.2575 | 240 | |
| Tpr | 0.0012651 | -2.724 | 241 | |
| Ube2i | 0.0012662 | -4.9765 | 242 | |
| Klhl15 | 0.0012662 | -4.7608 | 243 | |
| Stx5a | 0.0012903 | -2.6089 | 244 | |
| Pik3c3 | 0.0012967 | -0.96819 | 245 | |
| Akirin2 | 0.0013052 | -3.0072 | 246 | |
| Spcs2 | 0.0013146 | -4.6904 | 247 | |
| Rpl5 | 0.0013384 | -3.1078 | 248 | |
| Slmo2 | 0.0013406 | -2.8946 | 249 | |
| R3hcc1l | 0.0013421 | -1.0001 | 250 | |
| Polr2h | 0.0013615 | -4.291 | 251 | |
| Kat8 | 0.0013629 | -3.3291 | 252 | |
| Nsun4 | 0.0013679 | -2.7039 | 253 | |
| Cdc42 | 0.001401 | -3.9728 | 254 | |
| Cope | 0.0014102 | -4.9095 | 255 | |
| Gtf2b | 0.0014259 | -3.9787 | 256 | |
| Ubtf | 0.0014346 | -4.9095 | 257 | |
| Cct3 | 0.0014346 | -4.9095 | 258 | |
| Vmn1r90 | 0.0014462 | -3.994 | 259 | |
| Herpud2 | 0.0014691 | -4.689 | 260 | |
| Myc | 0.0014796 | -4.0277 | 261 | |
| Gtf3c4 | 0.001501 | -2.5438 | 262 | |
| Rfc5 | 0.0015091 | -4.8864 | 263 | |
| Slc35b1 | 0.0015091 | -4.8864 | 264 | |
| Ccdc55 | 0.0015172 | -1.0774 | 265 | |
| Nubp1 | 0.0015335 | -4.6605 | 266 | |
| Nars | 0.0015389 | -2.3922 | 267 | |
| Trappc3 | 0.0015394 | -4.6487 | 268 | |
| mmu-mir-1231 | 0.0015467 | 1.0626 | 269 | |
| Bcl2l1 | 0.0015584 | -2.7773 | 270 | |
| Nus1 | 0.0015608 | -4.6525 | 271 | |
| Itgb1 | 0.0015608 | -4.6525 | 272 | |
| Kcnj15 | 0.0015608 | -4.6525 | 273 | |
| Pkn2 | 0.0015688 | -2.8414 | 274 | |
| Adat2 | 0.0015695 | -1.5811 | 275 | |
| Gm7609 | 0.0015728 | -2.565 | 276 | |
| Sf3b5 | 0.0015802 | -4.863 | 277 | |
| Mup4 | 0.0015802 | -4.863 | 278 | |
| Cenpe | 0.001596 | -2.7475 | 279 | |
| Tbcb | 0.001611 | -3.1918 | 280 | |
| A530054K11Rik | 0.001615 | -0.51418 | 281 | |
| Prpf19 | 0.0016457 | -4.8392 | 282 | |
| Grid1 | 0.0016604 | -0.6615 | 283 | |
| Zbtb8os | 0.0016826 | -4.815 | 284 | |
| Hars | 0.0016843 | -2.9389 | 285 | |
| Rpl6 | 0.0016843 | -4.6331 | 286 | |
| Itfg2 | 0.0017008 | -3.2582 | 287 | |
| Ssxb8 | 0.0017028 | -4.6056 | 288 | |
| Nup50 | 0.0017059 | -0.6286 | 289 | |
| Rab8b | 0.0017514 | -0.74708 | 290 | |
| Otud5 | 0.0017692 | -2.4408 | 291 | |
| mmu-mir-7241 | 0.0017871 | -1.8013 | 292 | |
| Wars2 | 0.0018167 | -4.3141 | 293 | |
| Imp3 | 0.0018175 | -4.1763 | 294 | |
| Ipo11 | 0.0018223 | -4.3164 | 295 | |
| Hdac10 | 0.0018423 | -0.45584 | 296 | |
| Dtymk | 0.0018621 | -4.7653 | 297 | |
| Tcof1 | 0.0018878 | 0.7176 | 298 | |
| Snrnp200 | 0.0018968 | -4.0216 | 299 | |
| Mta2 | 0.0019015 | -2.5151 | 300 | |
| Actr8 | 0.0019057 | -2.8929 | 301 | |
| Oosp3 | 0.0019106 | -2.3783 | 302 | |
| Higd1a | 0.0019225 | -2.8447 | 303 | |
| Gm20865 | 0.0019563 | -2.3597 | 304 | |
| Taf12 | 0.0019649 | -4.4194 | 305 | |
| Las1l | 0.0019787 | -3.3291 | 306 | |
| Atp6v0b | 0.0019902 | -3.7928 | 307 | |
| Ube2n | 0.0019909 | -3.9927 | 308 | |
| Melk | 0.0019988 | -2.4713 | 309 | |
| Rps4x | 0.0020012 | -3.9787 | 310 | |
| Rplp0 | 0.0020017 | -4.4069 | 311 | |
| mmu-mir-7688 | 0.0020122 | -1.762 | 312 | |
| Mptx1 | 0.0020168 | -4.444 | 313 | |
| Psg22 | 0.0020241 | -0.17424 | 314 | |
| mmu-mir-682 | 0.0020315 | -1.5626 | 315 | |
| Snapc1 | 0.002033 | -2.374 | 316 | |
| Vps11 | 0.0020432 | -2.378 | 317 | |
| Ddx19a | 0.0020447 | -4.7139 | 318 | |
| Mak16 | 0.0020447 | -4.7139 | 319 | |
| Ndufab1 | 0.0020447 | -3.9092 | 320 | |
| Hyou1 | 0.0020447 | -4.7139 | 321 | |
| Stat1 | 0.0020634 | -4.7984 | 322 | |
| Ptpmt1 | 0.0020696 | -3.416 | 323 | |
| Utp6 | 0.0020931 | -2.5268 | 324 | |
| Med8 | 0.0021082 | -4.3816 | 325 | |
| Icam1 | 0.002115 | 0.33574 | 326 | |
| mmu-mir-5101 | 0.0021253 | -4.0149 | 327 | |
| Gdi2 | 0.0021302 | -2.8985 | 328 | |
| Eif6 | 0.0021463 | -4.6875 | 329 | |
| Seh1l | 0.0021463 | -4.6875 | 330 | |
| Gm8300 | 0.0021505 | -3.2248 | 331 | |
| Ftsj3 | 0.0021506 | -2.4695 | 332 | |
| Pggt1b | 0.0021582 | -2.714 | 333 | |
| Polr1e | 0.0021734 | -2.2895 | 334 | |
| Fdft1 | 0.0022059 | 0.33782 | 335 | |
| Gars | 0.0022113 | -4.6524 | 336 | |
| Ammecr1 | 0.0022188 | -2.4452 | 337 | |
| Cdk1 | 0.0022442 | -4.3943 | 338 | |
| Psmd8 | 0.0022509 | -3.5397 | 339 | |
| Taf1b | 0.0022714 | -2.9098 | 340 | |
| Mis18a | 0.0022741 | -0.85122 | 341 | |
| Ndufc2 | 0.0022741 | 0.36079 | 342 | |
| Tor1a | 0.0023074 | -2.4534 | 343 | |
| Snrnp25 | 0.0023106 | -3.3955 | 344 | |
| Tcea1 | 0.0023194 | -2.6021 | 345 | |
| mmu-mir-7667 | 0.002323 | -3.126 | 346 | |
| Dnlz | 0.002323 | -4.3296 | 347 | |
| Adam1b | 0.0023273 | -2.3023 | 348 | |
| Rpl11 | 0.0023313 | -2.2946 | 349 | |
| Rpl30 | 0.0023392 | -4.6125 | 350 | |
| Mrpl45 | 0.0023423 | -1.8044 | 351 | |
| Timm23 | 0.002349 | -2.2965 | 352 | |
| Bub1b | 0.0023514 | -2.8492 | 353 | |
| Tmx2 | 0.0023655 | -4.3164 | 354 | |
| Rpl14 | 0.0023809 | -2.2661 | 355 | |
| Scaper | 0.0023874 | -2.7484 | 356 | |
| Serinc1 | 0.0024104 | -1.6035 | 357 | |
| Banp | 0.0024104 | 1.2756 | 358 | |
| Zfyve9 | 0.0024435 | -1.7325 | 359 | |
| Wrn | 0.0024709 | -2.5667 | 360 | |
| Atp5l | 0.0024786 | -0.73767 | 361 | |
| Anapc1 | 0.0024842 | -2.3246 | 362 | |
| Olfr459 | 0.0024926 | -1.4888 | 363 | |
| Chchd4 | 0.0024951 | -4.6051 | 364 | |
| Vmn1r68 | 0.002498 | -2.4302 | 365 | |
| mmu-mir-466m | 0.0025152 | -4.2996 | 366 | |
| Tango6 | 0.0025468 | -4.5645 | 367 | |
| Mrpl23 | 0.0025512 | -3.8922 | 368 | |
| Polr2f | 0.0025695 | -4.3926 | 369 | |
| Ssbp1 | 0.0025964 | -4.5766 | 370 | |
| Gm9733 | 0.0026604 | 0.10588 | 371 | |
| Alg1 | 0.0026678 | -4.234 | 372 | |
| Nars2 | 0.0026864 | -4.6992 | 373 | |
| 2810004N23Rik | 0.0027058 | -1.8637 | 374 | |
| Mettl16 | 0.0027065 | -4.5475 | 375 | |
| Cltc | 0.0027307 | -3.7942 | 376 | |
| Fam50a | 0.0027414 | -4.2198 | 377 | |
| Mfap3 | 0.0027441 | -0.77446 | 378 | |
| D15Ertd621e | 0.0027512 | -1.8882 | 379 | |
| Denr | 0.0027714 | -2.2821 | 380 | |
| Rab6a | 0.0027752 | -4.3099 | 381 | |
| Pelo | 0.0027989 | -2.2606 | 382 | |
| Eif3f | 0.0028065 | -4.4858 | 383 | |
| Chmp5 | 0.0028215 | -4.5179 | 384 | |
| Zcchc9 | 0.0028302 | -2.2546 | 385 | |
| Tmtc2 | 0.0028421 | -0.18247 | 386 | |
| Rps14 | 0.0028764 | -2.6823 | 387 | |
| A1cf | 0.0029218 | -2.6578 | 388 | |
| Nip7 | 0.0029291 | -4.4876 | 389 | |
| mmu-mir-466l | 0.0029991 | -4.4566 | 390 | |
| Psg25 | 0.0030011 | -0.25692 | 391 | |
| Hspd1 | 0.0030089 | -4.1466 | 392 | |
| Slc22a8 | 0.003016 | -1.072 | 393 | |
| Tgs1 | 0.0030259 | -4.5406 | 394 | |
| Gm14496 | 0.0030367 | -4.418 | 395 | |
| Mrpl27 | 0.0030692 | -1.9193 | 396 | |
| mmu-mir-3473b | 0.0030784 | -4.4566 | 397 | |
| Snrnp35 | 0.0030784 | -4.4566 | 398 | |
| U2af2 | 0.0030784 | -4.4566 | 399 | |
| Nubp2 | 0.0031146 | -0.74032 | 400 | |
| Adam32 | 0.0031185 | -1.9788 | 401 | |
| Vmn2r63 | 0.0031351 | -2.4392 | 402 | |
| Cd3eap | 0.0031601 | -2.1047 | 403 | |
| mmu-mir-92b | 0.0031783 | -1.601 | 404 | |
| Exosc7 | 0.0032212 | -4.425 | 405 | |
| Wrb | 0.0032212 | -4.425 | 406 | |
| Gtf2h2 | 0.0032212 | -3.8005 | 407 | |
| Gtpbp4 | 0.0032212 | -4.425 | 408 | |
| Pcyt1a | 0.0032509 | -1.3367 | 409 | |
| Cd53 | 0.0032586 | -3.1063 | 410 | |
| Ddx25 | 0.0032963 | 0.10273 | 411 | |
| Rpl12 | 0.0032968 | -2.7475 | 412 | |
| D14Abb1e | 0.0033034 | -4.1877 | 413 | |
| Psmd11 | 0.0033079 | -2.9897 | 414 | |
| Slc16a10 | 0.0033285 | -4.262 | 415 | |
| mmu-mir-680-2 | 0.0033319 | -3.5779 | 416 | |
| Nol9 | 0.0033417 | -1.8685 | 417 | |
| Cachd1 | 0.0033508 | -1.3248 | 418 | |
| Prpf38a | 0.003375 | -3.0106 | 419 | |
| Zfp820 | 0.0033872 | -0.41111 | 420 | |
| 2700062C07Rik | 0.0033966 | -2.1132 | 421 | |
| Rps2 | 0.003406 | -4.3926 | 422 | |
| Rps18 | 0.003406 | -4.3926 | 423 | |
| mmu-mir-669a-3 | 0.0034243 | -1.4409 | 424 | |
| Prkra | 0.003428 | -2.3982 | 425 | |
| Uba2 | 0.0034326 | -3.6132 | 426 | |
| Btaf1 | 0.0034421 | -4.4156 | 427 | |
| Olfr1425 | 0.0034441 | -2.4042 | 428 | |
| Gp1ba | 0.0034451 | -2.3819 | 429 | |
| Mis12 | 0.0034557 | -4.1619 | 430 | |
| Plbd1 | 0.003478 | 0.17612 | 431 | |
| Snw1 | 0.0034783 | -1.9355 | 432 | |
| Olfr52 | 0.0034785 | -2.154 | 433 | |
| H2afz | 0.0034962 | -3.2228 | 434 | |
| Gpatch1 | 0.0034996 | -4.4947 | 435 | |
| Spata5 | 0.0035067 | -2.2996 | 436 | |
| Acp2 | 0.0035234 | 0.43847 | 437 | |
| Cflar | 0.0035526 | -2.8463 | 438 | |
| Fam63a | 0.0035688 | -0.63229 | 439 | |
| Apitd1 | 0.0035765 | -2.5132 | 440 | |
| D10Wsu102e | 0.0035872 | -2.9474 | 441 | |
| Vmn1r212 | 0.0035936 | -3.8691 | 442 | |
| Nedd8 | 0.0035989 | -4.3595 | 443 | |
| Zfp952 | 0.003615 | -2.4152 | 444 | |
| Orc5 | 0.0036204 | -2.1037 | 445 | |
| Hand2 | 0.0036344 | -2.2287 | 446 | |
| Pgs1 | 0.0036369 | -1.3042 | 447 | |
| mmu-mir-717 | 0.0036375 | -1.7094 | 448 | |
| Prpf38b | 0.0036429 | -3.2978 | 449 | |
| Rnasek | 0.0036608 | -4.0212 | 450 | |
| Tbca | 0.0036701 | -4.1224 | 451 | |
| Arrb1 | 0.0036906 | -2.0994 | 452 | |
| Mrpl55 | 0.0036971 | -3.3926 | 453 | |
| Smarca5 | 0.0037059 | -4.2842 | 454 | |
| Larp7 | 0.0037122 | -4.4542 | 455 | |
| Phf7 | 0.0037364 | -2.2911 | 456 | |
| Zfp407 | 0.0037466 | -4.0047 | 457 | |
| Ndufb11 | 0.0037474 | -2.0854 | 458 | |
| Zw10 | 0.0037504 | -2.1973 | 459 | |
| Spty2d1 | 0.0037504 | -1.1108 | 460 | |
| mmu-mir-708 | 0.0037521 | -1.567 | 461 | |
| mmu-mir-7058 | 0.0037701 | -1.1116 | 462 | |
| Nop14 | 0.003786 | -4.3256 | 463 | |
| Ttc27 | 0.003786 | -4.3256 | 464 | |
| Ecd | 0.003786 | -4.3256 | 465 | |
| Wdr76 | 0.0037959 | -3.6345 | 466 | |
| Gnl3 | 0.0038637 | -3.9881 | 467 | |
| Vps25 | 0.0038637 | -3.9881 | 468 | |
| Snrpg | 0.0038728 | -3.0258 | 469 | |
| Arpc2 | 0.003918 | -3.9712 | 470 | |
| Pdcd11 | 0.0039395 | -2.0874 | 471 | |
| Zscan12 | 0.0039417 | -2.495 | 472 | |
| Ctu2 | 0.0039474 | -2.0804 | 473 | |
| Btbd11 | 0.0039547 | 0.77155 | 474 | |
| Mcm3 | 0.0039547 | -1.6288 | 475 | |
| Fam13a | 0.0039547 | -0.33976 | 476 | |
| Ranbp1 | 0.0039547 | -2.0815 | 477 | |
| Ndufa11 | 0.0039702 | -2.3617 | 478 | |
| Rpl7l1 | 0.0039782 | -2.9687 | 479 | |
| Rngtt | 0.0039841 | -4.291 | 480 | |
| Lage3 | 0.0039841 | -4.291 | 481 | |
| Olfr206 | 0.0039994 | -3.2814 | 482 | |
| Gm6432 | 0.0040206 | -1.7178 | 483 | |
| mmu-mir-1906-1 | 0.0040524 | -1.7357 | 484 | |
| Acaa2 | 0.0040895 | -2.0536 | 485 | |
| Cacna1c | 0.0040994 | -2.7579 | 486 | |
| Tomm22 | 0.0041136 | -3.0868 | 487 | |
| Psmb2 | 0.0041375 | -4.2853 | 488 | |
| Lrrcc1 | 0.0041524 | -2.2055 | 489 | |
| mmu-mir-130b | 0.0041765 | -2.2714 | 490 | |
| Mgst2 | 0.0041809 | -2.0622 | 491 | |
| Rtf1 | 0.0041954 | -4.2554 | 492 | |
| Rps15a | 0.0041954 | -4.2554 | 493 | |
| Olfr857 | 0.0041954 | -4.2554 | 494 | |
| Naf1 | 0.0042183 | -4.095 | 495 | |
| Flt3 | 0.0042271 | 0.6905 | 496 | |
| Ptplad2 | 0.0042313 | -1.9948 | 497 | |
| Slc25a11 | 0.0042624 | -2.0421 | 498 | |
| Eif1 | 0.0042654 | -4.3599 | 499 | |
| mmu-mir-5103 | 0.0042717 | 0.40287 | 500 | |
| Lyrm5 | 0.004284 | -2.2015 | 501 | |
| Smo | 0.0042952 | 0.79056 | 502 | |
| Olfr316 | 0.0042984 | -2.0308 | 503 | |
| Nbr1 | 0.004319 | -0.76385 | 504 | |
| Vps35 | 0.0043237 | -3.2471 | 505 | |
| Olfr1280 | 0.0043258 | -3.9018 | 506 | |
| Sult2a5 | 0.0043258 | -3.9018 | 507 | |
| mmu-mir-669o | 0.0043309 | -4.1881 | 508 | |
| Lsm11 | 0.0043405 | -1.2897 | 509 | |
| Vmn1r176 | 0.0043859 | -0.88861 | 510 | |
| Mfn2 | 0.004388 | -2.296 | 511 | |
| Cldn26 | 0.0043934 | -4.175 | 512 | |
| Rpl26 | 0.0044031 | -3.259 | 513 | |
| Gabra6 | 0.0044222 | -3.8839 | 514 | |
| Otub2 | 0.0044346 | -2.7103 | 515 | |
| mmu-mir-18a | 0.0044514 | -1.7367 | 516 | |
| Mrpl11 | 0.004454 | -1.7464 | 517 | |
| Smad6 | 0.0044563 | -4.213 | 518 | |
| Gm3763 | 0.0044738 | -3.3686 | 519 | |
| Nfatc3 | 0.0044864 | -2.7962 | 520 | |
| Prex2 | 0.0045043 | -3.9676 | 521 | |
| Ppa1 | 0.0045163 | -3.8657 | 522 | |
| Baz1b | 0.0045448 | -1.5092 | 523 | |
| Prl7b1 | 0.0045448 | -2.695 | 524 | |
| Wdr61 | 0.0045698 | -4.1381 | 525 | |
| Olfr868 | 0.0045786 | -2.6296 | 526 | |
| Ms4a6c | 0.0046252 | -1.1602 | 527 | |
| Ddx56 | 0.0046355 | -2.6228 | 528 | |
| Zbed6 | 0.0046355 | -2.2583 | 529 | |
| Asb12 | 0.0046409 | -4.1155 | 530 | |
| Mrpl52 | 0.0046549 | -4.3241 | 531 | |
| Vmn1r169 | 0.0046654 | -4.1287 | 532 | |
| Vmn1r235 | 0.0047036 | -1.1175 | 533 | |
| Dmap1 | 0.0047053 | -3.814 | 534 | |
| Rbck1 | 0.0047534 | -2.2789 | 535 | |
| Alyref | 0.0047672 | -3.9223 | 536 | |
| 2610015P09Rik | 0.0047695 | -2.0742 | 537 | |
| 4930415O20Rik | 0.0047733 | -2.2981 | 538 | |
| Mon2 | 0.0047943 | -0.51905 | 539 | |
| Lrrc45 | 0.0047943 | -3.1783 | 540 | |
| Cyfip1 | 0.0048083 | -2.566 | 541 | |
| Olfr1112 | 0.0048132 | -4.1432 | 542 | |
| Rft1 | 0.0048551 | -3.2271 | 543 | |
| Haus2 | 0.0048558 | -4.2966 | 544 | |
| Mtap7d3 | 0.004863 | -3.8099 | 545 | |
| Chd4 | 0.0048851 | -1.2392 | 546 | |
| Defb21 | 0.0048937 | -2.1907 | 547 | |
| Vmn1r222 | 0.0049299 | -2.2288 | 548 | |
| Usp34 | 0.0049345 | -4.304 | 549 | |
| Tapt1 | 0.0049758 | -0.095697 | 550 | |
| Ccna2 | 0.0049957 | -0.27445 | 551 | |
| mmu-mir-875 | 0.0049974 | 0.42344 | 552 | |
| Pet112 | 0.0050212 | -1.9039 | 553 | |
| mmu-mir-7647 | 0.0050812 | -2.5643 | 554 | |
| Dhx40 | 0.0050857 | -2.4292 | 555 | |
| mmu-mir-224 | 0.0050862 | -2.8329 | 556 | |
| Cand1 | 0.0051046 | -2.8618 | 557 | |
| Rbbp4 | 0.0051113 | -2.2962 | 558 | |
| Derl2 | 0.0051506 | -2.0372 | 559 | |
| Fntb | 0.0051572 | -1.6495 | 560 | |
| 4932438A13Rik | 0.0051737 | -2.647 | 561 | |
| Ppip5k2 | 0.0051999 | -0.50328 | 562 | |
| Cyc1 | 0.0052147 | -3.1424 | 563 | |
| Fnbp4 | 0.0052253 | -2.1638 | 564 | |
| Nup43 | 0.0052284 | -2.7923 | 565 | |
| Trmt6 | 0.0052559 | -2.7264 | 566 | |
| Tceb1 | 0.0052579 | -2.2819 | 567 | |
| mmu-mir-8103 | 0.00527 | -3.4505 | 568 | |
| Tamm41 | 0.0052757 | -3.7321 | 569 | |
| Atp6v1b1 | 0.0052933 | -1.3813 | 570 | |
| Tfam | 0.0052942 | -4.0628 | 571 | |
| Psmb1 | 0.0053555 | -3.5208 | 572 | |
| Dpf2 | 0.0053669 | -3.8791 | 573 | |
| Psmc5 | 0.0053781 | -4.0007 | 574 | |
| Olfr381 | 0.0053781 | -4.0007 | 575 | |
| Mrps35 | 0.005384 | -0.97044 | 576 | |
| Oip5 | 0.0053912 | -4.0606 | 577 | |
| Tubgcp6 | 0.0053912 | -4.0606 | 578 | |
| Vmn1r123 | 0.0053914 | -3.5509 | 579 | |
| Rps29 | 0.0053932 | -3.6359 | 580 | |
| Dynlrb1 | 0.0054227 | -2.0398 | 581 | |
| Ciao1 | 0.0054269 | -4.0522 | 582 | |
| Csnk1g3 | 0.0054294 | -2.5049 | 583 | |
| Cks1b | 0.0054678 | -3.7852 | 584 | |
| mmu-mir-6390 | 0.0054712 | -2.7065 | 585 | |
| Hmgb1 | 0.005472 | -2.1969 | 586 | |
| Mrps6 | 0.0054747 | -2.5185 | 587 | |
| Hist2h3c2 | 0.0054999 | -6.029 | 588 | |
| Eef1e1 | 0.0055251 | -4.0216 | 589 | |
| Letm1 | 0.0055881 | -0.82398 | 590 | |
| Asb3 | 0.0055973 | -3.8352 | 591 | |
| Rplp1 | 0.0056146 | -2.0268 | 592 | |
| C1galt1c1 | 0.0056391 | -2.5401 | 593 | |
| Cdk12 | 0.0056471 | -4.1508 | 594 | |
| Tceb2 | 0.005697 | -2.653 | 595 | |
| mmu-mir-302b | 0.0057028 | -2.2766 | 596 | |
| Pfdn5 | 0.0057413 | -4.135 | 597 | |
| Rpap2 | 0.0057479 | -2.2621 | 598 | |
| Cdk6 | 0.0057567 | -1.9806 | 599 | |
| Gadd45gip1 | 0.0057684 | -2.5787 | 600 | |
| Wbscr16 | 0.0057695 | -1.8678 | 601 | |
| Mrpl17 | 0.0057695 | -3.2683 | 602 | |
| Mrpl47 | 0.0057781 | -3.9267 | 603 | |
| Banf1 | 0.0058247 | -3.9787 | 604 | |
| Tubg1 | 0.0058247 | -3.9787 | 605 | |
| Sfi1 | 0.0058247 | -3.9787 | 606 | |
| Clp1 | 0.0058247 | -3.5208 | 607 | |
| Uxt | 0.0058602 | -1.0931 | 608 | |
| mmu-mir-669k | 0.0058896 | -2.5174 | 609 | |
| 9830147E19Rik | 0.0059041 | -1.9098 | 610 | |
| Bcs1l | 0.0059354 | -3.7506 | 611 | |
| Cenpl | 0.0059439 | -3.6284 | 612 | |
| Ttk | 0.0059496 | -2.0614 | 613 | |
| Rpl13a | 0.0059496 | -3.0258 | 614 | |
| Rpl3 | 0.0059496 | -2.1157 | 615 | |
| Ran | 0.0059496 | -2.4023 | 616 | |
| Iars | 0.0059496 | -2.4718 | 617 | |
| Erap1 | 0.0059508 | 0.6145 | 618 | |
| Ncapd3 | 0.0059508 | -2.1314 | 619 | |
| Pmf1 | 0.0059638 | -2.5843 | 620 | |
| Rnf32 | 0.0059773 | -2.0725 | 621 | |
| Paf1 | 0.0060683 | -4.095 | 622 | |
| Slc6a11 | 0.0061095 | -0.54255 | 623 | |
| Wfdc13 | 0.0061095 | -0.29672 | 624 | |
| Znhit3 | 0.0061095 | -3.1153 | 625 | |
| Ttf1 | 0.00612 | -3.9473 | 626 | |
| Rfx4 | 0.0061246 | -2.2131 | 627 | |
| Nop2 | 0.0061799 | -3.1297 | 628 | |
| 3830403N18Rik | 0.0062057 | -1.8529 | 629 | |
| Dmtn | 0.0062455 | -0.72589 | 630 | |
| Tom1l1 | 0.006262 | -2.2065 | 631 | |
| Mphosph10 | 0.0062945 | -3.9199 | 632 | |
| Cdc73 | 0.0063 | -3.5208 | 633 | |
| Ccdc38 | 0.0063365 | -1.8551 | 634 | |
| Cnot10 | 0.0063694 | -3.9989 | 635 | |
| Mepce | 0.0063815 | -0.24398 | 636 | |
| Hpse | 0.006414 | -1.8565 | 637 | |
| Armc7 | 0.0064223 | -3.8919 | 638 | |
| Kdelr3 | 0.0064268 | -2.923 | 639 | |
| Nol11 | 0.0064448 | -3.8887 | 640 | |
| D130040H23Rik | 0.0064448 | -3.8887 | 641 | |
| Actr10 | 0.0064448 | -3.8887 | 642 | |
| Gm5796 | 0.0064558 | -5.931 | 643 | |
| Gm12942 | 0.0064565 | -1.9624 | 644 | |
| Arl2 | 0.0064714 | -2.5861 | 645 | |
| Atp5c1 | 0.0065021 | -3.8257 | 646 | |
| Clgn | 0.0065064 | -2.3088 | 647 | |
| Olfr331 | 0.0065254 | -2.0629 | 648 | |
| Dnajb9 | 0.0065429 | -3.1363 | 649 | |
| Olfr784 | 0.0065627 | -1.6781 | 650 | |
| Gm13051 | 0.0065627 | -0.32475 | 651 | |
| Vmn1r232 | 0.0065627 | -1.0368 | 652 | |
| 4930524N10Rik | 0.0065684 | -1.7845 | 653 | |
| Rab9b | 0.0066154 | -1.9368 | 654 | |
| Serpinb5 | 0.0066277 | -1.8066 | 655 | |
| Spn | 0.0066389 | -2.2661 | 656 | |
| Pafah1b1 | 0.0066444 | -3.958 | 657 | |
| Dcun1d1 | 0.0066534 | -1.7576 | 658 | |
| Cdc45 | 0.0066963 | -3.5166 | 659 | |
| Bax | 0.0067026 | -2.5826 | 660 | |
| Polr3k | 0.0067075 | -1.833 | 661 | |
| Luc7l3 | 0.0067442 | -3.4949 | 662 | |
| Brap | 0.0067619 | -3.9817 | 663 | |
| Aldh7a1 | 0.0067651 | -3.6349 | 664 | |
| Olfr456 | 0.0067667 | 0.7318 | 665 | |
| Tbcc | 0.0067781 | -2.9844 | 666 | |
| mmu-mir-92a-2 | 0.00681 | -0.82076 | 667 | |
| Klf16 | 0.0068337 | -3.0437 | 668 | |
| Psmg4 | 0.0068343 | -3.8416 | 669 | |
| Rpa2 | 0.0068343 | -3.8416 | 670 | |
| Uba1 | 0.0068343 | -3.8416 | 671 | |
| Spc25 | 0.0068343 | -3.8416 | 672 | |
| Ckap5 | 0.0068343 | -3.8416 | 673 | |
| Uso1 | 0.0068343 | -3.8416 | 674 | |
| Ing4 | 0.0068501 | -1.208 | 675 | |
| Map3k7 | 0.0068559 | -2.5188 | 676 | |
| Vrk1 | 0.0068799 | -0.68001 | 677 | |
| Man1a2 | 0.0068799 | -0.41752 | 678 | |
| Hist1h2bq | 0.0068882 | -5.8968 | 679 | |
| Oxt | 0.0068962 | -1.8172 | 680 | |
| Ceacam5 | 0.0069081 | -3.8822 | 681 | |
| 6820408C15Rik | 0.0069632 | -1.8398 | 682 | |
| Cry1 | 0.0069705 | -0.59576 | 683 | |
| Grtp1 | 0.0070158 | -1.4098 | 684 | |
| Ptcd3 | 0.007039 | -2.4174 | 685 | |
| Fdps | 0.0070609 | -3.9437 | 686 | |
| Pmpca | 0.0070624 | -2.245 | 687 | |
| mmu-mir-466c-1 | 0.0070778 | -3.0005 | 688 | |
| Naa25 | 0.0070838 | -2.2078 | 689 | |
| Mrpl3 | 0.0070838 | -1.5823 | 690 | |
| Eftud1 | 0.0070866 | -3.7419 | 691 | |
| Yme1l1 | 0.0070994 | -3.8695 | 692 | |
| Cntln | 0.0071166 | -2.7917 | 693 | |
| Atp6ap1 | 0.0071297 | -1.9591 | 694 | |
| Sox8 | 0.0071517 | -0.35406 | 695 | |
| Rptn | 0.0071677 | -1.3513 | 696 | |
| Smc4 | 0.0071846 | -3.6901 | 697 | |
| Rcc1 | 0.007196 | -3.7928 | 698 | |
| Gm21637 | 0.007196 | -3.7928 | 699 | |
| Polr2g | 0.007196 | -3.7928 | 700 | |
| Fam167a | 0.007197 | -0.44846 | 701 | |
| Lsm3 | 0.0072041 | -2.5615 | 702 | |
| Atp6v1f | 0.0072288 | -2.0552 | 703 | |
| Slc26a4 | 0.0072373 | -1.8059 | 704 | |
| Lipf | 0.007265 | -0.49778 | 705 | |
| Tdrd3 | 0.007265 | 0.54213 | 706 | |
| Imp4 | 0.0072962 | -2.5087 | 707 | |
| Tmem138 | 0.0073047 | -1.8018 | 708 | |
| Vmn2r90 | 0.0073324 | -1.326 | 709 | |
| Anp32b | 0.0073391 | -2.7279 | 710 | |
| Arf2 | 0.0074235 | 0.052632 | 711 | |
| Smim1 | 0.0074235 | -1.4244 | 712 | |
| Guca2a | 0.0074235 | -0.15343 | 713 | |
| Tmprss4 | 0.0074235 | -1.8587 | 714 | |
| Mars | 0.0074323 | -2.8613 | 715 | |
| mmu-mir-8092 | 0.0074439 | -0.43916 | 716 | |
| Ppp1r12a | 0.0074831 | -3.7988 | 717 | |
| Prkrip1 | 0.0075029 | -2.4479 | 718 | |
| Telo2 | 0.0075073 | -2.7119 | 719 | |
| Wdr4 | 0.0075175 | -1.8122 | 720 | |
| Rab2a | 0.0075315 | -2.1629 | 721 | |
| Mybl2 | 0.0075342 | -1.7885 | 722 | |
| Zbtb17 | 0.0075381 | -3.7803 | 723 | |
| Col19a1 | 0.0075594 | -1.3061 | 724 | |
| Cd3g | 0.0075701 | -1.7914 | 725 | |
| Rab11fip5 | 0.0075822 | -1.1145 | 726 | |
| mmu-mir-7038 | 0.0075931 | -2.7241 | 727 | |
| Syt16 | 0.0076281 | -2.3153 | 728 | |
| Mterfd2 | 0.0076317 | -1.8028 | 729 | |
| Eif2s2 | 0.0076353 | -3.7424 | 730 | |
| Pes1 | 0.0076353 | -3.7424 | 731 | |
| Exosc4 | 0.0076353 | -3.7424 | 732 | |
| Tmem184a | 0.0076726 | -1.1895 | 733 | |
| mmu-mir-7659 | 0.0077115 | -2.4856 | 734 | |
| Sdhc | 0.0077405 | -1.6026 | 735 | |
| Fam171a1 | 0.0078537 | -1.3297 | 736 | |
| Krtap4-2 | 0.0078537 | 0.36508 | 737 | |
| Tex14 | 0.0078537 | 0.58484 | 738 | |
| Klhl5 | 0.0078615 | -1.7734 | 739 | |
| Pid1 | 0.0078886 | -2.5684 | 740 | |
| Nadk | 0.0078922 | -1.4566 | 741 | |
| mmu-mir-488 | 0.0078947 | -2.3668 | 742 | |
| Rrp7a | 0.0079012 | -2.8869 | 743 | |
| Gbp10 | 0.007912 | -1.214 | 744 | |
| Snx17 | 0.0079138 | -1.931 | 745 | |
| Olfr776 | 0.0079397 | -2.4485 | 746 | |
| Nf1 | 0.0079482 | -1.769 | 747 | |
| Atp5f1 | 0.0079668 | -3.6266 | 748 | |
| Nob1 | 0.0079909 | -2.0367 | 749 | |
| Gmppb | 0.0080076 | -3.3441 | 750 | |
| Vmn2r105 | 0.00801 | -1.8904 | 751 | |
| Alox8 | 0.0080122 | -1.681 | 752 | |
| Ncapg | 0.008063 | -2.1775 | 753 | |
| Gins4 | 0.0080644 | -3.6901 | 754 | |
| Rplp2 | 0.0080644 | -3.6901 | 755 | |
| Ccdc125 | 0.0080877 | -1.7579 | 756 | |
| Cep85 | 0.0080923 | -1.7523 | 757 | |
| Slit1 | 0.0081254 | -1.6534 | 758 | |
| 2700060E02Rik | 0.0081933 | -1.8268 | 759 | |
| Trp53inp1 | 0.0082175 | -2.7771 | 760 | |
| Trim11 | 0.008226 | -1.754 | 761 | |
| Mtg2 | 0.0082267 | -3.8823 | 762 | |
| Fam64a | 0.0082437 | -1.7439 | 763 | |
| Ang5 | 0.0082461 | -3.7214 | 764 | |
| Cp | 0.0082474 | -3.7506 | 765 | |
| Kif16b | 0.0082566 | -3.5996 | 766 | |
| Trmt2b | 0.0083291 | -2.1556 | 767 | |
| Cdc5l | 0.0083537 | -2.2953 | 768 | |
| Olfr961 | 0.0083743 | 1.1244 | 769 | |
| 5530400C23Rik | 0.0083744 | -3.7357 | 770 | |
| Tsr2 | 0.0083883 | -3.71 | 771 | |
| Cd2bp2 | 0.0083969 | -2.0555 | 772 | |
| Ptgs1 | 0.0084196 | -1.5467 | 773 | |
| Lrrc34 | 0.0084516 | -1.7193 | 774 | |
| Pop4 | 0.0084535 | -2.379 | 775 | |
| mmu-mir-669h | 0.0085115 | -2.6213 | 776 | |
| Cyct | 0.0085131 | -1.7484 | 777 | |
| Olfr235 | 0.0085768 | -1.7032 | 778 | |
| Apex2 | 0.008578 | -2.3238 | 779 | |
| Lrrc20 | 0.008578 | -0.9233 | 780 | |
| Rpl23 | 0.0085842 | -3.6359 | 781 | |
| Twistnb | 0.0085842 | -3.6359 | 782 | |
| Vprbp | 0.0085842 | -3.6359 | 783 | |
| Tomm70a | 0.0085842 | -3.6359 | 784 | |
| Rac1 | 0.0085842 | -3.6359 | 785 | |
| Ubb | 0.0085842 | -2.7475 | 786 | |
| Rps21 | 0.0085842 | -3.6359 | 787 | |
| Tbp | 0.0085842 | -3.6359 | 788 | |
| Ufm1 | 0.0086208 | -1.7336 | 789 | |
| Ddx17 | 0.0086641 | -3.2296 | 790 | |
| Sugt1 | 0.0086783 | -3.6465 | 791 | |
| Rps3a1 | 0.0087538 | -3.2633 | 792 | |
| Gm648 | 0.0087633 | -3.8108 | 793 | |
| Spns2 | 0.0087728 | -1.7178 | 794 | |
| Ncl | 0.0088043 | -2.3683 | 795 | |
| Stat3 | 0.0088043 | 0.24821 | 796 | |
| Arl8b | 0.0088505 | -1.7225 | 797 | |
| Csgalnact1 | 0.0088739 | -2.4975 | 798 | |
| Timd2 | 0.008906 | -2.5908 | 799 | |
| Nsmce2 | 0.0089155 | -3.6892 | 800 | |
| Taf1c | 0.0089299 | -2.9674 | 801 | |
| mmu-mir-6967 | 0.0089339 | -1.4672 | 802 | |
| Kcnc2 | 0.0089581 | -1.7289 | 803 | |
| 4930422G04Rik | 0.00896 | -1.7174 | 804 | |
| 1700019G17Rik | 0.0089718 | -3.1238 | 805 | |
| C130026I21Rik | 0.0089895 | -5.5013 | 806 | |
| Thoc2 | 0.0090065 | -3.5795 | 807 | |
| Nanp | 0.0090072 | -1.8498 | 808 | |
| Dnm1l | 0.0090401 | -3.7602 | 809 | |
| Hist1h4m | 0.0090675 | -4.0372 | 810 | |
| Mad2l1 | 0.0090704 | -1.9858 | 811 | |
| Rpl29 | 0.0090764 | -1.715 | 812 | |
| Klk5 | 0.0090983 | -3.2338 | 813 | |
| Lrrtm1 | 0.0090983 | -0.22467 | 814 | |
| Ncapd2 | 0.0090983 | -1.0046 | 815 | |
| Prmt5 | 0.0091061 | -2.1517 | 816 | |
| Copz1 | 0.0091118 | -0.56198 | 817 | |
| Btbd10 | 0.009114 | -2.1616 | 818 | |
| Ercc3 | 0.009122 | -3.2069 | 819 | |
| Bms1 | 0.0091922 | -3.2069 | 820 | |
| Vrk2 | 0.0092237 | -1.7006 | 821 | |
| mmu-mir-7042 | 0.0092269 | -2.1249 | 822 | |
| Mmp12 | 0.0092337 | -2.2418 | 823 | |
| Ube4a | 0.0092567 | -0.70341 | 824 | |
| Rabggta | 0.0092567 | -2.1335 | 825 | |
| Skil | 0.0092723 | -3.6717 | 826 | |
| Ints7 | 0.0092772 | -2.9437 | 827 | |
| Cryz | 0.0092967 | -1.7504 | 828 | |
| Cep55 | 0.0093211 | -3.4717 | 829 | |
| Triap1 | 0.0093245 | -2.0213 | 830 | |
| Phf12 | 0.0093524 | -2.4399 | 831 | |
| Zbtb37 | 0.0093565 | -2.231 | 832 | |
| Uqcrc2 | 0.0093698 | -1.6284 | 833 | |
| Thoc5 | 0.0093797 | -3.1641 | 834 | |
| Anapc13 | 0.0093846 | -3.6543 | 835 | |
| Dimt1 | 0.0093888 | -1.8223 | 836 | |
| Ncaph2 | 0.0094091 | -3.3267 | 837 | |
| Ttc4 | 0.009414 | -0.63734 | 838 | |
| Hoxb9 | 0.0094262 | -3.4568 | 839 | |
| Mrpl4 | 0.0094376 | 0.71519 | 840 | |
| Vmn2r25 | 0.0094793 | -1.8649 | 841 | |
| Spock3 | 0.0094966 | -2.2183 | 842 | |
| Bpifa6 | 0.0095055 | -2.1821 | 843 | |
| Gfm1 | 0.009515 | -2.6037 | 844 | |
| Dyrk1a | 0.0095457 | -2.4065 | 845 | |
| Eprs | 0.0095517 | -3.4417 | 846 | |
| Oser1 | 0.0095539 | -2.8919 | 847 | |
| Zscan5b | 0.0095616 | -3.6991 | 848 | |
| Nfs1 | 0.0096111 | -3.5208 | 849 | |
| Chek1 | 0.0096111 | -3.5208 | 850 | |
| Plk1 | 0.0096111 | -3.5208 | 851 | |
| Kcna6 | 0.0096279 | -1.6788 | 852 | |
| Sfpq | 0.0096547 | -2.1652 | 853 | |
| BC028528 | 0.0096637 | -0.87049 | 854 | |
| Smc1a | 0.0096637 | 0.14477 | 855 | |
| Wee2 | 0.0096637 | -0.70008 | 856 | |
| Wdr75 | 0.0096637 | -3.4216 | 857 | |
| Olfr659 | 0.0096904 | -0.19947 | 858 | |
| Pcna | 0.0097333 | -2.6414 | 859 | |
| Ackr2 | 0.0097374 | -1.7691 | 860 | |
| Kat2b | 0.0097402 | -2.8768 | 861 | |
| Cyp19a1 | 0.0098001 | -0.54829 | 862 | |
| Rbmxl1 | 0.0098101 | -1.495 | 863 | |
| Aurkc | 0.0098201 | -1.3824 | 864 | |
| B3gnt1 | 0.009822 | -1.2773 | 865 | |
| Ddx55 | 0.0098276 | -3.5868 | 866 | |
| Txn1 | 0.0098551 | -3.1481 | 867 | |
| mmu-mir-138-2 | 0.0098833 | -1.3654 | 868 | |
| mmu-mir-328 | 0.0098906 | -2.6869 | 869 | |
| Terf2 | 0.0098981 | -1.0021 | 870 | |
| Olfr825 | 0.0099124 | -2.3512 | 871 | |
| Tonsl | 0.0099317 | -3.3291 | 872 | |
| Slit3 | 0.009936 | -0.56516 | 873 | |
| Prpf6 | 0.0099455 | -3.6345 | 874 | |
| Dctn2 | 0.0099506 | -3.5602 | 875 | |
| Ccdc7 | 0.0099549 | -2.8167 | 876 | |
| Myo3b | 0.0099577 | -0.42753 | 877 | |
| Hist1h3h | 0.0099591 | -3.1546 | 878 | |
| Mbd4 | 0.0099959 | -3.4677 | 879 | |
| 9030624G23Rik | 0.0099992 | -3.3599 | 880 | |
| Sap25 | 0.010003 | -0.24703 | 881 | |
| Spata5l1 | 0.010006 | -3.6093 | 882 | |
| Slc44a3 | 0.010021 | -1.2558 | 883 | |
| Rnf113a2 | 0.010025 | -0.69133 | 884 | |
| Vmn1r233 | 0.010067 | -3.6195 | 885 | |
| Aars2 | 0.010116 | -2.232 | 886 | |
| Car5b | 0.010117 | -3.3955 | 887 | |
| Cpsf3l | 0.010117 | -3.3955 | 888 | |
| Cetn1 | 0.010139 | -1.4533 | 889 | |
| Ftl1 | 0.010139 | -0.95826 | 890 | |
| Cops2 | 0.010166 | -3.1178 | 891 | |
| mmu-mir-5127 | 0.010196 | -1.39 | 892 | |
| Atpaf2 | 0.010201 | -2.3627 | 893 | |
| Polrmt | 0.010229 | -1.5365 | 894 | |
| Zc2hc1c | 0.010248 | -1.6539 | 895 | |
| Slc2a1 | 0.01027 | -1.6534 | 896 | |
| Gm829 | 0.010274 | -1.119 | 897 | |
| Rps11 | 0.010278 | -3.4597 | 898 | |
| Topbp1 | 0.010278 | -3.4597 | 899 | |
| Zmym4 | 0.01036 | -3.5876 | 900 | |
| Vmn2r73 | 0.010383 | -3.3638 | 901 | |
| Nxf3 | 0.010384 | -1.9286 | 902 | |
| Cyp2c39 | 0.010387 | -0.38484 | 903 | |
| Gm757 | 0.010387 | -2.8746 | 904 | |
| Tnfrsf26 | 0.010387 | -0.66551 | 905 | |
| Gm5885 | 0.010393 | -2.1359 | 906 | |
| Pfpl | 0.010406 | -1.5528 | 907 | |
| Traip | 0.010423 | -1.9078 | 908 | |
| Sart3 | 0.010449 | -1.932 | 909 | |
| Mcmbp | 0.010465 | -3.5708 | 910 | |
| Rnf130 | 0.010512 | -0.24885 | 911 | |
| Olfr113 | 0.010517 | -3.5814 | 912 | |
| Wdr91 | 0.010539 | -1.6292 | 913 | |
| Prc1 | 0.010569 | -3.6173 | 914 | |
| Sepsecs | 0.010572 | -2.1703 | 915 | |
| Ist1 | 0.010591 | -2.6759 | 916 | |
| Mrpl36 | 0.010594 | -2.9107 | 917 | |
| Srsf2 | 0.010602 | -3.3314 | 918 | |
| Tmem242 | 0.010618 | -3.3898 | 919 | |
| Tti2 | 0.010628 | -3.5238 | 920 | |
| Ddx46 | 0.010642 | -1.4761 | 921 | |
| Fam213a | 0.010647 | -3.0552 | 922 | |
| Bzw1 | 0.010664 | -2.5901 | 923 | |
| Rpl19 | 0.010693 | -2.9632 | 924 | |
| Crot | 0.010704 | -0.3249 | 925 | |
| Sptssa | 0.010713 | -2.666 | 926 | |
| mmu-mir-1264 | 0.010764 | -1.1856 | 927 | |
| Sbds | 0.010772 | -3.5541 | 928 | |
| Gtf2f2 | 0.010782 | -1.2364 | 929 | |
| Dpagt1 | 0.010783 | -2.1837 | 930 | |
| Rps13 | 0.010783 | -3.1854 | 931 | |
| Pde12 | 0.010816 | -2.6099 | 932 | |
| mmu-mir-6974 | 0.010819 | -1.0768 | 933 | |
| Speer4a | 0.010819 | -2.3432 | 934 | |
| Tiparp | 0.010907 | -3.0374 | 935 | |
| Pop7 | 0.010913 | -3.3959 | 936 | |
| Cct2 | 0.010913 | -3.3959 | 937 | |
| Nkain3 | 0.010935 | -2.8362 | 938 | |
| Mrps27 | 0.010952 | -2.1608 | 939 | |
| Rab18 | 0.010992 | -3.3956 | 940 | |
| Ear10 | 0.010996 | -1.8578 | 941 | |
| Serpina6 | 0.011001 | -1.6244 | 942 | |
| Slc16a8 | 0.011005 | -1.6219 | 943 | |
| Agtrap | 0.011032 | -1.7237 | 944 | |
| Olfr1136 | 0.011088 | -0.87476 | 945 | |
| Ddx1 | 0.011129 | -2.1596 | 946 | |
| mmu-mir-3471-2 | 0.011142 | -2.7962 | 947 | |
| Gltscr2 | 0.011204 | -3.384 | 948 | |
| Isg20l2 | 0.011236 | -2.6935 | 949 | |
| Kansl1l | 0.011249 | -2.0651 | 950 | |
| Cnot6l | 0.011252 | -1.9836 | 951 | |
| Pdrg1 | 0.011291 | -1.8198 | 952 | |
| Srrm1 | 0.011291 | -2.6969 | 953 | |
| Ear1 | 0.011291 | -1.0638 | 954 | |
| Mis18bp1 | 0.011295 | -2.1213 | 955 | |
| Zfp322a | 0.0113 | -2.2972 | 956 | |
| mmu-mir-5620 | 0.011303 | -2.1646 | 957 | |
| Emcn | 0.011319 | -1.7167 | 958 | |
| Ptgfr | 0.011346 | -1.6119 | 959 | |
| Plxnb3 | 0.011376 | -3.2644 | 960 | |
| Eif3m | 0.011392 | -3.4332 | 961 | |
| mmu-mir-7072 | 0.011421 | -0.3999 | 962 | |
| Cenpm | 0.011501 | -1.9989 | 963 | |
| Prss1 | 0.011574 | -2.0518 | 964 | |
| Ivd | 0.011575 | -3.0694 | 965 | |
| Srfbp1 | 0.011584 | -1.7912 | 966 | |
| Flna | 0.011584 | -1.7484 | 967 | |
| Olfr1222 | 0.011584 | 0.027146 | 968 | |
| Arih1 | 0.011584 | 1.0737 | 969 | |
| Klk1b11 | 0.011613 | -1.5046 | 970 | |
| Zmym5 | 0.011627 | -1.5632 | 971 | |
| Acpp | 0.011635 | -3.4562 | 972 | |
| Gps1 | 0.011651 | -3.1189 | 973 | |
| Ankle2 | 0.011702 | -3.3291 | 974 | |
| Rpl27 | 0.011702 | -3.3291 | 975 | |
| Phf5a | 0.011702 | -2.6414 | 976 | |
| Haus1 | 0.011702 | -3.3291 | 977 | |
| Dhx16 | 0.011727 | -2.1197 | 978 | |
| Rbx1 | 0.011788 | -1.1285 | 979 | |
| Zfp711 | 0.011788 | -0.73616 | 980 | |
| Igf2bp1 | 0.011788 | -1.9576 | 981 | |
| Slc4a1 | 0.011876 | -1.7981 | 982 | |
| Crnkl1 | 0.011891 | -2.2097 | 983 | |
| Ndufc1 | 0.011893 | -3.0682 | 984 | |
| Efcab10 | 0.011903 | -1.714 | 985 | |
| Izumo3 | 0.011946 | -2.8353 | 986 | |
| Olfr1312 | 0.011973 | -3.0943 | 987 | |
| Tbpl1 | 0.011987 | -3.212 | 988 | |
| mmu-mir-471 | 0.011993 | -1.2617 | 989 | |
| Pdc | 0.012036 | -2.109 | 990 | |
| Snrnp48 | 0.012056 | -3.2644 | 991 | |
| Vmn2r9 | 0.012067 | -1.4074 | 992 | |
| Krtap26-1 | 0.0121 | -2.7859 | 993 | |
| Rpl27a | 0.012117 | -3.0258 | 994 | |
| Ppa2 | 0.012129 | -1.5774 | 995 | |
| Zfp747 | 0.01215 | -2.2123 | 996 | |
| Defb10 | 0.012161 | -0.16046 | 997 | |
| Gtf2e2 | 0.012161 | -3.1941 | 998 | |
| Lmnb2 | 0.012187 | -1.8469 | 999 | |
| Heatr2 | 0.012189 | -2.8008 | 1000 | |
| Acsl4 | 0.012196 | -1.6754 | 1001 | |
| Dcp1b | 0.012217 | -2.1791 | 1002 | |
| Olfr367-ps | 0.012251 | -3.0615 | 1003 | |
| Gtf2a2 | 0.01233 | -3.401 | 1004 | |
| Mrps10 | 0.012334 | -2.3795 | 1005 | |
| Ddx20 | 0.012352 | -0.88816 | 1006 | |
| Shq1 | 0.012352 | -0.6719 | 1007 | |
| Tinf2 | 0.012352 | -2.5223 | 1008 | |
| Dyrk2 | 0.012354 | -1.6561 | 1009 | |
| Setx | 0.012378 | -1.6448 | 1010 | |
| Rttn | 0.012383 | -3.451 | 1011 | |
| Slco1a4 | 0.012405 | -1.4452 | 1012 | |
| Olfr1371 | 0.012455 | -2.3588 | 1013 | |
| Igsf10 | 0.012475 | -2.0261 | 1014 | |
| Mrgpra2b | 0.012481 | -3.3155 | 1015 | |
| Ust | 0.012487 | -0.22681 | 1016 | |
| Nae1 | 0.012488 | -2.6683 | 1017 | |
| Nova1 | 0.012495 | -3.3317 | 1018 | |
| Abhd14a | 0.012581 | -1.7246 | 1019 | |
| Sart1 | 0.012584 | -2.9166 | 1020 | |
| mmu-mir-6365 | 0.012596 | -2.1659 | 1021 | |
| Gtf3c3 | 0.012623 | -1.9057 | 1022 | |
| Bud31 | 0.012625 | -3.259 | 1023 | |
| Snrpd1 | 0.012625 | -3.259 | 1024 | |
| Vmn2r113 | 0.012625 | -3.259 | 1025 | |
| Rpl17 | 0.012625 | -3.259 | 1026 | |
| mmu-mir-7665 | 0.01263 | -1.9876 | 1027 | |
| Wls | 0.012664 | -1.5727 | 1028 | |
| Prkcz | 0.012668 | -1.1836 | 1029 | |
| Ndfip2 | 0.01268 | -2.2864 | 1030 | |
| mmu-mir-6987 | 0.012701 | -1.1222 | 1031 | |
| Dad1 | 0.01271 | -3.0895 | 1032 | |
| Nelfa | 0.012712 | -2.8119 | 1033 | |
| Lhfpl4 | 0.012713 | -1.7767 | 1034 | |
| Nup210 | 0.012738 | -1.2125 | 1035 | |
| Echdc3 | 0.01274 | -1.5684 | 1036 | |
| Gemin7 | 0.012761 | -3.3432 | 1037 | |
| Olfr711 | 0.012762 | -1.5672 | 1038 | |
| Mrps26 | 0.0128 | -3.1033 | 1039 | |
| Npas3 | 0.012803 | -1.1189 | 1040 | |
| Trpm1 | 0.012809 | -2.3221 | 1041 | |
| Gm14092 | 0.012839 | -1.9826 | 1042 | |
| Ints9 | 0.012851 | -1.6097 | 1043 | |
| mmu-mir-340 | 0.012864 | -0.076039 | 1044 | |
| Zfp667 | 0.012869 | -1.5437 | 1045 | |
| Vmn1r69 | 0.012883 | -3.139 | 1046 | |
| Narfl | 0.012883 | -3.139 | 1047 | |
| mmu-mir-6962 | 0.012885 | -0.95219 | 1048 | |
| Prpf31 | 0.012889 | -2.5988 | 1049 | |
| Brca1 | 0.012894 | -2.333 | 1050 | |
| Ift88 | 0.012907 | -1.6076 | 1051 | |
| 1110004E09Rik | 0.012917 | -3.3937 | 1052 | |
| Dhrs1 | 0.012939 | -0.95461 | 1053 | |
| Eef1g | 0.012939 | -2.3778 | 1054 | |
| Gm17296 | 0.012943 | -3.3989 | 1055 | |
| Elfn1 | 0.012963 | -3.1897 | 1056 | |
| Pip4k2b | 0.012977 | -2.685 | 1057 | |
| Adam20 | 0.013012 | -1.5549 | 1058 | |
| Crk | 0.013032 | -2.1837 | 1059 | |
| Mcmdc2 | 0.013053 | -1.6579 | 1060 | |
| Cxxc1 | 0.013057 | -2.4013 | 1061 | |
| Amn1 | 0.013074 | -0.6356 | 1062 | |
| Fdxacb1 | 0.013074 | -3.2271 | 1063 | |
| BC056474 | 0.013083 | -1.6579 | 1064 | |
| Pgm3 | 0.013103 | -1.8913 | 1065 | |
| Srp9 | 0.013111 | -3.1067 | 1066 | |
| Preb | 0.013114 | -0.85985 | 1067 | |
| Cox6b2 | 0.013118 | -1.9994 | 1068 | |
| Rpp14 | 0.013154 | -1.5547 | 1069 | |
| Cpsf3 | 0.013155 | -2.8492 | 1070 | |
| Rpl9 | 0.013155 | -2.8492 | 1071 | |
| Csprs | 0.013164 | -0.59007 | 1072 | |
| Mios | 0.013164 | -1.0951 | 1073 | |
| Tars2 | 0.013172 | -3.4 | 1074 | |
| 2010109I03Rik | 0.013235 | -2.0235 | 1075 | |
| Rras2 | 0.013255 | -0.274 | 1076 | |
| Ntng1 | 0.01327 | -2.9856 | 1077 | |
| Serpinb6a | 0.013273 | -1.5502 | 1078 | |
| Srd5a1 | 0.013276 | -3.3885 | 1079 | |
| Secisbp2l | 0.013287 | -3.249 | 1080 | |
| Nme8 | 0.0133 | -0.45466 | 1081 | |
| mmu-mir-344d-1 | 0.013305 | -2.5388 | 1082 | |
| Olfr293 | 0.013336 | -1.0068 | 1083 | |
| Afmid | 0.013345 | 1.4862 | 1084 | |
| Kif26a | 0.013533 | -1.0956 | 1085 | |
| Pcdhb9 | 0.01357 | -0.50801 | 1086 | |
| Tfrc | 0.01357 | -1.1815 | 1087 | |
| Hmha1 | 0.01357 | 0.0020439 | 1088 | |
| Nrd1 | 0.01357 | -2.1354 | 1089 | |
| Serpinb1a | 0.01357 | -1.5018 | 1090 | |
| Idh3a | 0.01357 | -0.90204 | 1091 | |
| Exosc8 | 0.013578 | -3.1854 | 1092 | |
| Rps8 | 0.013578 | -3.1854 | 1093 | |
| Anapc11 | 0.013578 | -3.1854 | 1094 | |
| Gfer | 0.013592 | -2.0659 | 1095 | |
| Icmt | 0.013631 | -1.8071 | 1096 | |
| Uspl1 | 0.013656 | -1.5372 | 1097 | |
| Olfr1314 | 0.013659 | -3.3014 | 1098 | |
| Magea2 | 0.013694 | -1.6011 | 1099 | |
| Cdk9 | 0.013712 | -2.6593 | 1100 | |
| Mutyh | 0.013751 | -1.7075 | 1101 | |
| mmu-mir-3473d | 0.013767 | -1.283 | 1102 | |
| Dbt | 0.013769 | -1.8693 | 1103 | |
| 4933427D06Rik | 0.013809 | -1.9496 | 1104 | |
| Wars | 0.013812 | -2.8929 | 1105 | |
| Ldha | 0.013841 | -1.5993 | 1106 | |
| Rbm39 | 0.013841 | -1.9613 | 1107 | |
| 2010005H15Rik | 0.013841 | 0.28762 | 1108 | |
| Krtap10-10 | 0.013848 | -1.5298 | 1109 | |
| Tnfrsf23 | 0.013864 | -1.8366 | 1110 | |
| Arcn1 | 0.013865 | -3.0621 | 1111 | |
| Plk4 | 0.013887 | -2.8718 | 1112 | |
| Arpc4 | 0.013918 | -3.1494 | 1113 | |
| Olfr1284 | 0.013931 | 1.0013 | 1114 | |
| Gabarapl2 | 0.01394 | -1.6594 | 1115 | |
| Eif3d | 0.013965 | -3.3118 | 1116 | |
| mmu-mir-7079 | 0.01397 | -1.3741 | 1117 | |
| Ermn | 0.013972 | -1.527 | 1118 | |
| mmu-mir-1957b | 0.013976 | 0.17562 | 1119 | |
| Nr2c2ap | 0.013976 | -1.2372 | 1120 | |
| Dmc1 | 0.014011 | -1.552 | 1121 | |
| Mptx2 | 0.014037 | -3.0365 | 1122 | |
| Kpnb1 | 0.014043 | -1.5895 | 1123 | |
| Rsrc2 | 0.014062 | -1.6889 | 1124 | |
| Olfr749 | 0.014066 | -3.241 | 1125 | |
| Chmp2a | 0.014072 | -3.3241 | 1126 | |
| Mus81 | 0.014089 | -1.2739 | 1127 | |
| Brox | 0.014089 | 0.60019 | 1128 | |
| Slc2a5 | 0.014089 | -1.6931 | 1129 | |
| Mrpl54 | 0.01412 | -1.5594 | 1130 | |
| mmu-mir-3470b | 0.014156 | -1.4822 | 1131 | |
| Phax | 0.014173 | -3.2346 | 1132 | |
| Lztfl1 | 0.014179 | -2.7735 | 1133 | |
| Gm13154 | 0.014202 | -0.58748 | 1134 | |
| Anks4b | 0.014205 | -1.6299 | 1135 | |
| Morf4l1 | 0.014227 | -3.2822 | 1136 | |
| Ifngr2 | 0.014251 | -1.6944 | 1137 | |
| Dhx35 | 0.014292 | -1.4112 | 1138 | |
| Med22 | 0.014318 | -2.9799 | 1139 | |
| Ephb3 | 0.014326 | -1.7691 | 1140 | |
| Igbp1 | 0.014341 | -3.1078 | 1141 | |
| Gm6592 | 0.014371 | -1.5779 | 1142 | |
| Nefl | 0.014404 | -1.6925 | 1143 | |
| Gm5935 | 0.014414 | -5.0204 | 1144 | |
| Vmn1r10 | 0.014419 | -1.8989 | 1145 | |
| Vmn1r-ps103 | 0.01445 | -1.2326 | 1146 | |
| Uhrf1 | 0.01445 | -2.8362 | 1147 | |
| Sdr16c6 | 0.014491 | -3.0103 | 1148 | |
| mmu-mir-5116 | 0.014494 | -2.0747 | 1149 | |
| Dohh | 0.014496 | -2.9013 | 1150 | |
| H2-T23 | 0.014533 | -3.2464 | 1151 | |
| Lig1 | 0.014569 | -0.8282 | 1152 | |
| Fundc2 | 0.014637 | -1.5096 | 1153 | |
| Cables2 | 0.014652 | -0.40686 | 1154 | |
| Mrpl24 | 0.014653 | -1.7179 | 1155 | |
| Naa10 | 0.014659 | -1.9798 | 1156 | |
| mmu-mir-6391 | 0.014666 | -1.6674 | 1157 | |
| BC049730 | 0.014714 | -1.2066 | 1158 | |
| mmu-mir-125b-2 | 0.014732 | -0.8943 | 1159 | |
| Cfh | 0.014743 | 0.19983 | 1160 | |
| Olfm2 | 0.014755 | -1.7397 | 1161 | |
| Bcl2a1d | 0.014776 | -2.846 | 1162 | |
| Serpina3f | 0.014781 | -3.2687 | 1163 | |
| Col22a1 | 0.014788 | -0.41598 | 1164 | |
| Peo1 | 0.014799 | -1.0183 | 1165 | |
| mmu-mir-5133 | 0.014812 | -1.8062 | 1166 | |
| Nrbp1 | 0.014824 | -3.2284 | 1167 | |
| Vps28 | 0.014833 | -1.3137 | 1168 | |
| Slc3a2 | 0.014839 | -2.3568 | 1169 | |
| Pnpt1 | 0.014848 | -3.2432 | 1170 | |
| Polr3f | 0.014854 | -2.734 | 1171 | |
| Syt15 | 0.014866 | -1.2611 | 1172 | |
| Sapcd2 | 0.014878 | -1.5031 | 1173 | |
| Mylk3 | 0.014885 | -3.278 | 1174 | |
| Pecam1 | 0.014891 | -1.1924 | 1175 | |
| Mapk15 | 0.014911 | -1.8772 | 1176 | |
| Ewsr1 | 0.014974 | -1.7345 | 1177 | |
| Wbp2nl | 0.014988 | -1.4996 | 1178 | |
| Med10 | 0.015007 | -1.5125 | 1179 | |
| Med20 | 0.015036 | -1.8664 | 1180 | |
| Cstf1 | 0.015036 | -1.7043 | 1181 | |
| Rpf1 | 0.015036 | -1.9322 | 1182 | |
| Ddost | 0.01504 | -3.1432 | 1183 | |
| Vmn2r59 | 0.015046 | -0.60297 | 1184 | |
| Atp6ap2 | 0.01505 | -1.8389 | 1185 | |
| Pdcl3 | 0.015073 | -1.4976 | 1186 | |
| Strap | 0.015095 | -2.3886 | 1187 | |
| Dnttip2 | 0.015116 | -1.4905 | 1188 | |
| Olfr1046 | 0.015125 | -2.0219 | 1189 | |
| Mcm7 | 0.015136 | -3.2138 | 1190 | |
| Olfr704 | 0.015146 | -3.1556 | 1191 | |
| Dis3 | 0.015193 | -1.9493 | 1192 | |
| Mmadhc | 0.015197 | -1.4993 | 1193 | |
| Agk | 0.015219 | -3.2178 | 1194 | |
| Gm5506 | 0.015241 | -1.5331 | 1195 | |
| Lix1 | 0.015283 | -1.588 | 1196 | |
| Vma21 | 0.01529 | -0.023154 | 1197 | |
| Wsb1 | 0.015311 | -1.7447 | 1198 | |
| Lrp10 | 0.015323 | -1.487 | 1199 | |
| Ubr4 | 0.015342 | -3.1615 | 1200 | |
| Snx1 | 0.015345 | -1.4821 | 1201 | |
| Usp46 | 0.015355 | -1.5294 | 1202 | |
| Actl11 | 0.015373 | 0.20051 | 1203 | |
| Zscan4c | 0.015401 | -3.0098 | 1204 | |
| Ccnb3 | 0.015424 | -1.6109 | 1205 | |
| Entpd1 | 0.015427 | -1.4867 | 1206 | |
| Hspe1 | 0.015457 | -2.9599 | 1207 | |
| Dusp12 | 0.015458 | -1.5999 | 1208 | |
| Rprd1b | 0.015482 | -1.6733 | 1209 | |
| Lclat1 | 0.015503 | -1.4882 | 1210 | |
| Cacna1f | 0.015531 | -1.1251 | 1211 | |
| Mrps18a | 0.015531 | -1.2714 | 1212 | |
| S100a10 | 0.015531 | -0.69263 | 1213 | |
| Il6st | 0.015544 | -1.5766 | 1214 | |
| Csnk2a1 | 0.015555 | -1.6162 | 1215 | |
| Gm10094 | 0.015578 | -1.9979 | 1216 | |
| Tti1 | 0.015597 | -1.9808 | 1217 | |
| Polr3c | 0.015616 | -1.9238 | 1218 | |
| Gm5538 | 0.015619 | -1.0017 | 1219 | |
| Gm711 | 0.01565 | -1.3809 | 1220 | |
| Gm3383 | 0.01565 | -2.9078 | 1221 | |
| Slc7a6os | 0.015689 | -1.4028 | 1222 | |
| Gja1 | 0.0157 | -3.2201 | 1223 | |
| Olfr1155 | 0.01571 | -1.4024 | 1224 | |
| Ppil2 | 0.015712 | -3.053 | 1225 | |
| Med31 | 0.015725 | -1.512 | 1226 | |
| Cops6 | 0.015737 | -2.8532 | 1227 | |
| Ugcg | 0.015751 | -2.007 | 1228 | |
| Loxl2 | 0.015756 | -2.1632 | 1229 | |
| Vps13d | 0.015762 | -1.8045 | 1230 | |
| Vmn1r193 | 0.015762 | -3.2087 | 1231 | |
| Rad9a | 0.015846 | -1.365 | 1232 | |
| Gm12657 | 0.015846 | -1.7635 | 1233 | |
| Ap2b1 | 0.015914 | -1.4962 | 1234 | |
| Tsfm | 0.015938 | -3.183 | 1235 | |
| Sec16b | 0.015959 | 0.13472 | 1236 | |
| Gm7168 | 0.015962 | -2.2224 | 1237 | |
| Vmn2r17 | 0.015969 | -1.4642 | 1238 | |
| Ercc4 | 0.016006 | -1.4684 | 1239 | |
| Supt4a | 0.016017 | -1.7412 | 1240 | |
| Nek9 | 0.016026 | -2.2837 | 1241 | |
| Kntc1 | 0.016026 | -1.2225 | 1242 | |
| mmu-let-7e | 0.016055 | -2.2827 | 1243 | |
| Olfr1467 | 0.016156 | -3.1619 | 1244 | |
| Vmn2r115 | 0.016162 | 1.4082 | 1245 | |
| Cpt1c | 0.016162 | 0.35832 | 1246 | |
| Ddhd1 | 0.016166 | -2.3215 | 1247 | |
| Tspan33 | 0.016167 | -1.4112 | 1248 | |
| Mrpl44 | 0.016178 | -3.0581 | 1249 | |
| Pkmyt1 | 0.016186 | -1.3212 | 1250 | |
| Xkr4 | 0.01621 | -2.6518 | 1251 | |
| Pfdn1 | 0.016229 | -2.4313 | 1252 | |
| Kctd18 | 0.016259 | -1.5398 | 1253 | |
| Ppp3r2 | 0.016261 | -1.6743 | 1254 | |
| Sun3 | 0.016274 | -1.061 | 1255 | |
| Vmn2r6 | 0.016306 | -1.4708 | 1256 | |
| Itih3 | 0.016331 | -3.1457 | 1257 | |
| Skint1 | 0.016338 | -2.0207 | 1258 | |
| Sf3b4 | 0.016344 | -0.42832 | 1259 | |
| Anapc5 | 0.016356 | -2.3893 | 1260 | |
| Atp5j2 | 0.016409 | -0.5932 | 1261 | |
| Rbm14 | 0.016409 | -1.3026 | 1262 | |
| Sf3b2 | 0.016423 | -3.0258 | 1263 | |
| Ddx24 | 0.016423 | -3.0258 | 1264 | |
| Srsf1 | 0.016423 | -3.0258 | 1265 | |
| Tll2 | 0.016497 | -3.1777 | 1266 | |
| mmu-mir-5098 | 0.01651 | -2.1706 | 1267 | |
| B3gnt4 | 0.016518 | -1.4411 | 1268 | |
| Hist3h2a | 0.016523 | -2.9573 | 1269 | |
| Klk1b3 | 0.016544 | -0.10768 | 1270 | |
| Ddx51 | 0.016567 | -3.1636 | 1271 | |
| Tm4sf4 | 0.016606 | -3.1599 | 1272 | |
| Rps3 | 0.016612 | -2.7273 | 1273 | |
| Dhx36 | 0.016645 | -3.1132 | 1274 | |
| mmu-mir-7007 | 0.016675 | -0.15801 | 1275 | |
| Ccnd1 | 0.016692 | -1.8284 | 1276 | |
| Trim25 | 0.016702 | -1.6232 | 1277 | |
| Lct | 0.016702 | -2.835 | 1278 | |
| Krtap5-5 | 0.016702 | -0.69012 | 1279 | |
| Alg5 | 0.016704 | -1.4893 | 1280 | |
| Toe1 | 0.016748 | -3.1302 | 1281 | |
| Mbtps1 | 0.016814 | 0.93249 | 1282 | |
| Nsun5 | 0.016819 | -3.131 | 1283 | |
| mmu-mir-6537 | 0.016819 | -2.7087 | 1284 | |
| D430041D05Rik | 0.016845 | -1.3899 | 1285 | |
| Cdc25b | 0.016916 | -1.8441 | 1286 | |
| Ankrd50 | 0.016986 | -2.2725 | 1287 | |
| Rnf8 | 0.016994 | -0.87468 | 1288 | |
| Irf6 | 0.016994 | -2.3188 | 1289 | |
| Hbq1b | 0.017019 | -1.276 | 1290 | |
| mmu-mir-7018 | 0.017035 | -0.57243 | 1291 | |
| Wdr82 | 0.017071 | -3.0092 | 1292 | |
| Pabpc6 | 0.017156 | -0.59075 | 1293 | |
| Cst8 | 0.017165 | -1.4609 | 1294 | |
| Wdr73 | 0.017182 | -2.1225 | 1295 | |
| Speer2 | 0.017192 | -1.715 | 1296 | |
| Tmcc3 | 0.017208 | -3.1256 | 1297 | |
| Zbed4 | 0.017221 | -2.5438 | 1298 | |
| Cmtm7 | 0.01723 | -0.83305 | 1299 | |
| Prpf40a | 0.017242 | -1.5555 | 1300 | |
| Irs4 | 0.017264 | -0.43654 | 1301 | |
| Tsen2 | 0.017274 | -3.109 | 1302 | |
| Zbtb33 | 0.0173 | -1.6005 | 1303 | |
| mmu-mir-7230 | 0.017335 | -1.3245 | 1304 | |
| Spata31d1a | 0.017343 | -1.6573 | 1305 | |
| mmu-mir-709 | 0.017346 | -2.1289 | 1306 | |
| Gsta1 | 0.017385 | -2.277 | 1307 | |
| Tmem210 | 0.017404 | -1.4936 | 1308 | |
| Psma6 | 0.017418 | -0.56198 | 1309 | |
| Srgap1 | 0.017431 | -2.9463 | 1310 | |
| Star | 0.017438 | -1.4379 | 1311 | |
| Olfr792 | 0.017444 | 2.0426 | 1312 | |
| Ncaph | 0.017444 | -1.5302 | 1313 | |
| Ptprd | 0.017465 | -1.6092 | 1314 | |
| mmu-mir-30d | 0.017477 | -1.3783 | 1315 | |
| mmu-mir-1954 | 0.017514 | -0.87007 | 1316 | |
| Nr1h2 | 0.01753 | -1.7644 | 1317 | |
| Gpkow | 0.01753 | -2.8928 | 1318 | |
| Zmym2 | 0.017554 | -1.5801 | 1319 | |
| Qars | 0.017579 | -0.72944 | 1320 | |
| Olfr633 | 0.017589 | -1.1338 | 1321 | |
| Eda2r | 0.017596 | -3.0341 | 1322 | |
| Zc3h10 | 0.017596 | -1.4307 | 1323 | |
| Ccna1 | 0.017619 | -1.6301 | 1324 | |
| mmu-mir-6906 | 0.01765 | -1.6234 | 1325 | |
| Prelid2 | 0.017662 | -3.1196 | 1326 | |
| mmu-mir-7071 | 0.017664 | -1.4973 | 1327 | |
| mmu-mir-7044 | 0.017675 | -2.1453 | 1328 | |
| Cenpa | 0.017696 | -1.4297 | 1329 | |
| Pcnt | 0.017767 | -3.0133 | 1330 | |
| Ttc17 | 0.017782 | -1.3606 | 1331 | |
| Nsa2 | 0.01783 | -2.2661 | 1332 | |
| Cmc2 | 0.017887 | -2.9065 | 1333 | |
| Cyp2c69 | 0.017888 | -1.8983 | 1334 | |
| Mmp21 | 0.017923 | -0.40461 | 1335 | |
| Gosr2 | 0.018007 | -2.577 | 1336 | |
| Olfr243 | 0.018018 | -1.7183 | 1337 | |
| Isca1 | 0.018029 | -1.5089 | 1338 | |
| Speer3 | 0.018029 | -1.5177 | 1339 | |
| Zfp953 | 0.018029 | -2.2326 | 1340 | |
| Brf1 | 0.018029 | -0.90014 | 1341 | |
| Rpl35 | 0.018029 | -0.56198 | 1342 | |
| Cep104 | 0.018029 | -1.8264 | 1343 | |
| Pgk1 | 0.018047 | -3.0684 | 1344 | |
| E030019B06Rik | 0.01806 | -1.2974 | 1345 | |
| Rpl39 | 0.018151 | -2.9389 | 1346 | |
| Ints4 | 0.018151 | -2.9389 | 1347 | |
| Rps23 | 0.018151 | -2.9389 | 1348 | |
| Tnpo3 | 0.018151 | -2.9389 | 1349 | |
| Adamts3 | 0.018164 | -1.5775 | 1350 | |
| Rnf138rt1 | 0.018176 | -1.7754 | 1351 | |
| Nat10 | 0.018254 | -2.7705 | 1352 | |
| Ccdc83 | 0.018288 | -1.0465 | 1353 | |
| Mrps11 | 0.018344 | 2.1308 | 1354 | |
| Timm22 | 0.018402 | -2.8867 | 1355 | |
| mmu-mir-7074 | 0.018405 | -0.95221 | 1356 | |
| Gm6812 | 0.018409 | -2.8991 | 1357 | |
| Gpi1 | 0.018443 | -1.9757 | 1358 | |
| Osgep | 0.018479 | -2.5428 | 1359 | |
| Amd2 | 0.018479 | 0.93118 | 1360 | |
| Tas2r143 | 0.018479 | -0.27031 | 1361 | |
| Zfand2b | 0.018531 | -2.6528 | 1362 | |
| Kcna2 | 0.018571 | -1.4088 | 1363 | |
| Ankrd45 | 0.01862 | -1.4201 | 1364 | |
| Ccdc127 | 0.018627 | -1.4071 | 1365 | |
| 4732456N10Rik | 0.018636 | -1.1713 | 1366 | |
| Fbxo18 | 0.018646 | -1.6579 | 1367 | |
| Fdx1l | 0.018672 | -1.8765 | 1368 | |
| Srp14 | 0.018707 | -2.3529 | 1369 | |
| Sox6 | 0.018714 | -3.0392 | 1370 | |
| Matr3 | 0.018732 | -2.0071 | 1371 | |
| Gemin5 | 0.018768 | -3.0544 | 1372 | |
| Olfr630 | 0.018838 | -1.6039 | 1373 | |
| Pfn4 | 0.018838 | 0.61645 | 1374 | |
| Srsf6 | 0.018838 | -0.30067 | 1375 | |
| Dync1i2 | 0.018838 | -1.4772 | 1376 | |
| Cdc26 | 0.018877 | -2.9654 | 1377 | |
| Cd164 | 0.018893 | -1.4011 | 1378 | |
| Exosc1 | 0.018897 | -3.0391 | 1379 | |
| Adamts1 | 0.01896 | -1.4418 | 1380 | |
| Abce1 | 0.018973 | -2.6414 | 1381 | |
| Antxr2 | 0.018973 | -1.721 | 1382 | |
| Ikbkap | 0.019016 | -1.4664 | 1383 | |
| Cyp2a4 | 0.01912 | -1.9881 | 1384 | |
| Dctn6 | 0.01913 | -3.0238 | 1385 | |
| Cd2ap | 0.019137 | -2.7445 | 1386 | |
| Crx | 0.019153 | -1.7435 | 1387 | |
| Olfr239 | 0.019233 | -2.9435 | 1388 | |
| Abo | 0.019243 | -1.0324 | 1389 | |
| Ykt6 | 0.019277 | -1.3657 | 1390 | |
| mmu-mir-6349 | 0.019285 | -1.0815 | 1391 | |
| Olfr1181 | 0.019293 | -1.4424 | 1392 | |
| Hsp90aa1 | 0.019297 | -2.0072 | 1393 | |
| Lrrc40 | 0.019343 | -1.6549 | 1394 | |
| Vmn1r198 | 0.019385 | -1.307 | 1395 | |
| BC052040 | 0.019422 | -1.0867 | 1396 | |
| Olfr910 | 0.019431 | -1.3029 | 1397 | |
| mmu-mir-6410 | 0.019468 | -2.0099 | 1398 | |
| Pcdhb2 | 0.019471 | -2.8055 | 1399 | |
| Lyrm4 | 0.019523 | -2.8349 | 1400 | |
| Apba1 | 0.019543 | -1.3858 | 1401 | |
| Vars | 0.019565 | -2.5796 | 1402 | |
| Jak2 | 0.019664 | -2.3291 | 1403 | |
| Gm7361 | 0.019676 | -2.2622 | 1404 | |
| Tnfrsf13b | 0.019682 | -2.2893 | 1405 | |
| Snf8 | 0.01969 | -2.9012 | 1406 | |
| Cntfr | 0.019712 | -1.3811 | 1407 | |
| Gmps | 0.019737 | -1.3718 | 1408 | |
| Tbc1d19 | 0.019737 | -0.65857 | 1409 | |
| Mterfd1 | 0.019737 | -0.30982 | 1410 | |
| Rtcb | 0.019737 | -1.9501 | 1411 | |
| Tnrc6b | 0.019737 | -1.9275 | 1412 | |
| Terf1 | 0.019737 | -0.71329 | 1413 | |
| Olfr171 | 0.019759 | -3.0014 | 1414 | |
| Gm9376 | 0.019762 | -1.6183 | 1415 | |
| Tmco5b | 0.019772 | -1.6531 | 1416 | |
| Srd5a2 | 0.019885 | -1.4614 | 1417 | |
| Cog8 | 0.019941 | -2.6644 | 1418 | |
| Ccdc28a | 0.019976 | -2.7523 | 1419 | |
| Cldn11 | 0.020006 | -0.66579 | 1420 | |
| Arl14ep | 0.020051 | -2.1487 | 1421 | |
| Gpr143 | 0.020109 | -2.958 | 1422 | |
| Myh9 | 0.020154 | -2.6902 | 1423 | |
| Acly | 0.020157 | -1.6396 | 1424 | |
| C330018D20Rik | 0.020231 | 0.1758 | 1425 | |
| Hars2 | 0.020248 | -1.3669 | 1426 | |
| Adcyap1 | 0.020292 | -1.9386 | 1427 | |
| mmu-mir-7017 | 0.020303 | -1.7862 | 1428 | |
| Olfr679 | 0.020313 | -1.2293 | 1429 | |
| Tgm4 | 0.020321 | -2.9702 | 1430 | |
| Olfr12 | 0.020367 | -2.3329 | 1431 | |
| Nfatc4 | 0.02041 | 0.79269 | 1432 | |
| Wdr20 | 0.020441 | -2.9895 | 1433 | |
| Fam133b | 0.020466 | -1.6834 | 1434 | |
| Lce3c | 0.020496 | -1.8784 | 1435 | |
| Rpn1 | 0.020523 | -1.536 | 1436 | |
| C030046E11Rik | 0.020523 | -2.2889 | 1437 | |
| Vmn2r71 | 0.020534 | -1.3669 | 1438 | |
| Polr1c | 0.020536 | -2.8463 | 1439 | |
| Capzb | 0.020536 | -2.8463 | 1440 | |
| Snrpb | 0.020536 | -2.8463 | 1441 | |
| Nrf1 | 0.020536 | -2.8463 | 1442 | |
| Phb | 0.020536 | -2.8463 | 1443 | |
| Tpm3 | 0.020537 | -1.5311 | 1444 | |
| Gria4 | 0.020635 | -0.67591 | 1445 | |
| Atm | 0.020636 | -2.5401 | 1446 | |
| Sec1 | 0.020667 | -1.3601 | 1447 | |
| Ppie | 0.02068 | -1.4339 | 1448 | |
| Cdh11 | 0.020683 | -1.8323 | 1449 | |
| Clpb | 0.02076 | -1.4576 | 1450 | |
| Tra2b | 0.02076 | -2.9657 | 1451 | |
| Prl8a1 | 0.020768 | -2.9199 | 1452 | |
| Slc7a8 | 0.020796 | -1.358 | 1453 | |
| Rnh1 | 0.020808 | -1.6927 | 1454 | |
| Fndc3c1 | 0.020825 | -1.5338 | 1455 | |
| Oxsr1 | 0.020843 | -2.9538 | 1456 | |
| Pds5b | 0.020846 | -1.8182 | 1457 | |
| Lars2 | 0.020853 | -2.7812 | 1458 | |
| 1110001A16Rik | 0.020882 | -0.46575 | 1459 | |
| Ptpn4 | 0.020882 | -1.6774 | 1460 | |
| Coa6 | 0.020882 | -1.8036 | 1461 | |
| Acer3 | 0.020882 | -0.60449 | 1462 | |
| Ptar1 | 0.020932 | -1.5681 | 1463 | |
| Mcm5 | 0.020968 | -0.24858 | 1464 | |
| Olfr1008 | 0.020991 | -1.3925 | 1465 | |
| Olfr1167 | 0.021001 | -1.8616 | 1466 | |
| Mat2a | 0.021011 | -2.8939 | 1467 | |
| Ppm1m | 0.021027 | -1.5451 | 1468 | |
| mmu-mir-130a | 0.021064 | -2.3185 | 1469 | |
| mmu-mir-320 | 0.021072 | -1.2878 | 1470 | |
| Tpm4 | 0.021084 | -0.66645 | 1471 | |
| Sgol2 | 0.021111 | -2.6553 | 1472 | |
| Hps5 | 0.021113 | -2.4852 | 1473 | |
| C1qbp | 0.021165 | -1.6015 | 1474 | |
| Usp24 | 0.021172 | -1.878 | 1475 | |
| Lrrc42 | 0.021173 | -0.023315 | 1476 | |
| Dock7 | 0.021179 | -1.9231 | 1477 | |
| Urb1 | 0.021198 | -2.855 | 1478 | |
| Osgin2 | 0.021205 | -2.9314 | 1479 | |
| Kif18b | 0.021229 | -2.0755 | 1480 | |
| Slc28a3 | 0.021238 | -1.592 | 1481 | |
| Zbtb24 | 0.021248 | -1.3452 | 1482 | |
| Fzd6 | 0.021302 | -2.9364 | 1483 | |
| Ptcd1 | 0.021308 | -0.94436 | 1484 | |
| Vmn2r97 | 0.021308 | -1.0617 | 1485 | |
| Olfr1386 | 0.021308 | -0.97122 | 1486 | |
| Rab21 | 0.021308 | -1.84 | 1487 | |
| Zfp300 | 0.021308 | -1.1611 | 1488 | |
| Wbp2 | 0.02135 | -1.5049 | 1489 | |
| Ccl19 | 0.021356 | -1.6396 | 1490 | |
| Olfr514 | 0.021392 | -1.3317 | 1491 | |
| Srpk3 | 0.021399 | -1.3551 | 1492 | |
| Isca2 | 0.021413 | -1.5221 | 1493 | |
| Dars | 0.021443 | -2.2661 | 1494 | |
| Glrx3 | 0.02145 | -2.9214 | 1495 | |
| Rps6ka2 | 0.021487 | -1.5775 | 1496 | |
| Slc25a3 | 0.02153 | -2.9326 | 1497 | |
| Vmn1r75 | 0.021571 | -1.5315 | 1498 | |
| Atp1b3 | 0.021585 | -1.3367 | 1499 | |
| Med11 | 0.0216 | -0.66715 | 1500 | |
| Ngrn | 0.0216 | -2.1424 | 1501 | |
| Tsen15 | 0.021603 | -2.9004 | 1502 | |
| Supv3l1 | 0.021632 | -2.1625 | 1503 | |
| 2210018M11Rik | 0.021681 | -1.4149 | 1504 | |
| Wac | 0.021712 | -0.68156 | 1505 | |
| Zscan20 | 0.021763 | -2.8938 | 1506 | |
| Olfr1295 | 0.021769 | -1.5347 | 1507 | |
| Zc3h7a | 0.02177 | -1.4373 | 1508 | |
| Olfr1033 | 0.021785 | -1.312 | 1509 | |
| Glrx | 0.021801 | -1.6987 | 1510 | |
| Mtx1 | 0.021805 | -1.4203 | 1511 | |
| mmu-mir-7062 | 0.021819 | -0.32834 | 1512 | |
| Ift43 | 0.021821 | -1.3579 | 1513 | |
| Unc5b | 0.021844 | -1.3367 | 1514 | |
| Epm2aip1 | 0.021846 | 0.95736 | 1515 | |
| Odc1 | 0.021891 | -1.1192 | 1516 | |
| Vmn1r115 | 0.021916 | -2.7018 | 1517 | |
| Scml4 | 0.02196 | -2.7966 | 1518 | |
| Wdr72 | 0.021967 | -2.8977 | 1519 | |
| Olfr25 | 0.021989 | -1.0294 | 1520 | |
| Cops7b | 0.02204 | -1.8593 | 1521 | |
| Rad23b | 0.022044 | -2.0818 | 1522 | |
| Gm14499 | 0.02207 | -0.70374 | 1523 | |
| Vmn1r160 | 0.02207 | -1.4143 | 1524 | |
| Lipo1 | 0.02207 | -0.67344 | 1525 | |
| Mms19 | 0.02207 | -1.4594 | 1526 | |
| Rps6ka1 | 0.022084 | -2.1974 | 1527 | |
| Acss2 | 0.022099 | -2.9138 | 1528 | |
| Lyz2 | 0.022114 | -1.0382 | 1529 | |
| Mrpl34 | 0.022148 | -1.3326 | 1530 | |
| Gstz1 | 0.022173 | -2.3757 | 1531 | |
| Hmgxb4 | 0.022173 | -1.4944 | 1532 | |
| Hat1 | 0.02218 | -1.4734 | 1533 | |
| Med26 | 0.022191 | -1.6064 | 1534 | |
| Tmtc1 | 0.022277 | -1.325 | 1535 | |
| Rps27 | 0.022309 | -1.7552 | 1536 | |
| Phka2 | 0.02234 | -2.0242 | 1537 | |
| Nfasc | 0.022371 | -1.5926 | 1538 | |
| Mphosph8 | 0.022384 | -1.3631 | 1539 | |
| Tram1 | 0.0224 | -1.6306 | 1540 | |
| Lce1d | 0.022405 | -1.3335 | 1541 | |
| D630033O11Rik | 0.022407 | -1.3271 | 1542 | |
| mmu-mir-7119 | 0.022415 | -2.2332 | 1543 | |
| Rpl10 | 0.022424 | -1.5386 | 1544 | |
| Sf3a3 | 0.022424 | -2.7475 | 1545 | |
| Rasl12 | 0.022429 | 0.42206 | 1546 | |
| Cd177 | 0.022472 | -1.5033 | 1547 | |
| Scimp | 0.02249 | -2.165 | 1548 | |
| 4930555G01Rik | 0.02252 | -2.3253 | 1549 | |
| Olfr645 | 0.02257 | -2.3681 | 1550 | |
| Cd46 | 0.022581 | -1.4141 | 1551 | |
| mmu-mir-7066 | 0.022595 | 1.3313 | 1552 | |
| Rbm25 | 0.022601 | -2.6801 | 1553 | |
| Grk6 | 0.022653 | -0.64012 | 1554 | |
| Vmn1r101 | 0.022682 | -4.6709 | 1555 | |
| Gins3 | 0.022753 | -2.8727 | 1556 | |
| Wash | 0.022778 | -2.5804 | 1557 | |
| Acat3 | 0.022797 | -1.1825 | 1558 | |
| Utp15 | 0.022817 | -2.5217 | 1559 | |
| Csn3 | 0.022833 | -0.057788 | 1560 | |
| Nup88 | 0.022833 | -2.7178 | 1561 | |
| Rpp40 | 0.022833 | -1.7866 | 1562 | |
| Sfr1 | 0.022929 | -1.3309 | 1563 | |
| Trappc11 | 0.022954 | -1.705 | 1564 | |
| Tenm2 | 0.022984 | -1.7443 | 1565 | |
| Wbscr22 | 0.023003 | -2.7619 | 1566 | |
| Sobp | 0.023018 | -2.8511 | 1567 | |
| Snrpf | 0.023086 | -2.1065 | 1568 | |
| Dazap1 | 0.023102 | -2.7407 | 1569 | |
| mmu-mir-7116 | 0.023117 | -1.984 | 1570 | |
| 3110007F17Rik | 0.023137 | -2.2448 | 1571 | |
| Mst1 | 0.023137 | -1.309 | 1572 | |
| Nsl1 | 0.023172 | -1.4513 | 1573 | |
| Eps15l1 | 0.023191 | 0.60457 | 1574 | |
| P2ry6 | 0.023236 | -0.45412 | 1575 | |
| Ube2t | 0.023242 | -1.7869 | 1576 | |
| mmu-mir-1940 | 0.023263 | -0.98126 | 1577 | |
| Cars2 | 0.023264 | -1.3111 | 1578 | |
| Dicer1 | 0.023281 | -0.72787 | 1579 | |
| Rtn4ip1 | 0.023302 | -1.4505 | 1580 | |
| Eef1d | 0.023309 | -1.6125 | 1581 | |
| Tmprss11c | 0.023326 | -0.8903 | 1582 | |
| mmu-mir-1895 | 0.02343 | -0.5396 | 1583 | |
| Rbm8a | 0.023469 | -2.7364 | 1584 | |
| Wdr48 | 0.023482 | -2.4311 | 1585 | |
| Psmc4 | 0.023482 | -1.0861 | 1586 | |
| Rhd | 0.023482 | -0.39521 | 1587 | |
| Klhl1 | 0.023482 | -1.2076 | 1588 | |
| Eif4e | 0.023484 | -2.5813 | 1589 | |
| Csmd2os | 0.023507 | -1.7181 | 1590 | |
| Pfn1 | 0.023552 | -2.8361 | 1591 | |
| Fars2 | 0.023592 | -1.6758 | 1592 | |
| mmu-mir-3473f | 0.023598 | -1.6547 | 1593 | |
| C330021F23Rik | 0.023598 | -2.7475 | 1594 | |
| Cse1l | 0.023598 | -2.7475 | 1595 | |
| mmu-mir-8117 | 0.023598 | -1.6719 | 1596 | |
| Rab3il1 | 0.023615 | -1.3015 | 1597 | |
| U2af1 | 0.023629 | -2.1157 | 1598 | |
| Snx14 | 0.023651 | -2.6929 | 1599 | |
| Hnmt | 0.023662 | -1.1697 | 1600 | |
| mmu-mir-1927 | 0.023692 | -1.9207 | 1601 | |
| Abca2 | 0.023729 | -0.90295 | 1602 | |
| Vmn1r189 | 0.023767 | -2.2121 | 1603 | |
| Nufip2 | 0.023814 | -1.9345 | 1604 | |
| A730008H23Rik | 0.023833 | -2.2706 | 1605 | |
| Mrpl10 | 0.023855 | -2.2722 | 1606 | |
| Slc12a9 | 0.023886 | -1.4872 | 1607 | |
| Cops4 | 0.023908 | -2.344 | 1608 | |
| Olfr589 | 0.023909 | -1.1433 | 1609 | |
| Trnt1 | 0.02393 | 0.17315 | 1610 | |
| L3mbtl2 | 0.02393 | -0.73904 | 1611 | |
| Fbxw11 | 0.02393 | -2.7569 | 1612 | |
| Med15 | 0.02393 | 1.2153 | 1613 | |
| Asap2 | 0.02393 | 0.16324 | 1614 | |
| Zfp78 | 0.02393 | -1.0253 | 1615 | |
| Foxn3 | 0.02395 | -1.5584 | 1616 | |
| 4930549C01Rik | 0.023962 | -2.7213 | 1617 | |
| Senp2 | 0.023984 | -2.1013 | 1618 | |
| Pof1b | 0.024003 | -1.2757 | 1619 | |
| mmu-mir-138-1 | 0.024041 | -1.8499 | 1620 | |
| Hmgb3 | 0.024046 | -0.71873 | 1621 | |
| Ucn3 | 0.024107 | -1.7975 | 1622 | |
| Zdhhc9 | 0.024115 | -0.77165 | 1623 | |
| Cyp3a41b | 0.024116 | -4.815 | 1624 | |
| Gm21671 | 0.024116 | -4.0103 | 1625 | |
| Smad1 | 0.024121 | -1.3047 | 1626 | |
| Cgn | 0.024123 | -2.8557 | 1627 | |
| mmu-mir-6369 | 0.024139 | -1.9645 | 1628 | |
| Gm5072 | 0.02414 | -1.216 | 1629 | |
| Ercc2 | 0.024153 | -2.415 | 1630 | |
| Zfp964 | 0.024177 | -0.79325 | 1631 | |
| C1galt1 | 0.024181 | -1.2943 | 1632 | |
| Olfr697 | 0.024184 | -2.7989 | 1633 | |
| Ndufa9 | 0.024207 | -1.5972 | 1634 | |
| mmu-mir-3093 | 0.024243 | -1.5982 | 1635 | |
| Defb28 | 0.02433 | -2.8286 | 1636 | |
| Olfr492 | 0.024353 | -2.8297 | 1637 | |
| Olfr1380 | 0.024356 | -1.375 | 1638 | |
| Vmn2r78 | 0.024395 | -2.7061 | 1639 | |
| Cd300lb | 0.024445 | -0.78252 | 1640 | |
| Rpl31-ps12 | 0.024453 | -2.3726 | 1641 | |
| Lrrc58 | 0.024526 | -1.4002 | 1642 | |
| mmu-mir-7078 | 0.02458 | -1.3225 | 1643 | |
| Gemin2 | 0.024618 | -1.3411 | 1644 | |
| Taf10 | 0.024661 | -0.98721 | 1645 | |
| Pacsin1 | 0.024699 | -1.4578 | 1646 | |
| Ola1 | 0.024718 | -1.8299 | 1647 | |
| Gm16390 | 0.024734 | -2.7844 | 1648 | |
| Olfr920 | 0.024759 | -0.47539 | 1649 | |
| Ggh | 0.024759 | -1.9908 | 1650 | |
| Ulk2 | 0.024847 | -2.7815 | 1651 | |
| Clspn | 0.024854 | -2.8022 | 1652 | |
| Gapdhs | 0.024893 | -1.0271 | 1653 | |
| Rcor3 | 0.0249 | -1.7224 | 1654 | |
| Polr1a | 0.024901 | -2.5456 | 1655 | |
| Pwp2 | 0.024938 | -1.6485 | 1656 | |
| Trmt5 | 0.024954 | -2.1041 | 1657 | |
| Rasip1 | 0.025056 | -2.6146 | 1658 | |
| Fcgr1 | 0.025063 | -1.2782 | 1659 | |
| Top1 | 0.025093 | -2.0056 | 1660 | |
| Gpr18 | 0.02511 | -1.8115 | 1661 | |
| Gm527 | 0.025114 | -1.4223 | 1662 | |
| Rrp1 | 0.025124 | -2.465 | 1663 | |
| Oc90 | 0.025134 | -2.7917 | 1664 | |
| Papolb | 0.025138 | -1.6705 | 1665 | |
| Hmgcll1 | 0.025149 | -1.6489 | 1666 | |
| Nedd1 | 0.025165 | -2.8085 | 1667 | |
| Olfr315 | 0.025173 | -1.5212 | 1668 | |
| Hlf | 0.025185 | -1.2756 | 1669 | |
| Pkhd1l1 | 0.025189 | -2.8058 | 1670 | |
| Pptc7 | 0.025212 | -0.42946 | 1671 | |
| Iqcf5 | 0.025216 | -1.3861 | 1672 | |
| Trp53i11 | 0.02522 | -2.8073 | 1673 | |
| Hfm1 | 0.025231 | -2.0248 | 1674 | |
| Csnk2b | 0.025243 | -2.7943 | 1675 | |
| mmu-mir-669f | 0.025272 | -1.996 | 1676 | |
| Rgp1 | 0.025273 | -1.8736 | 1677 | |
| Fbxo5 | 0.025273 | -2.1906 | 1678 | |
| Tubb5 | 0.025273 | -1.08 | 1679 | |
| Atp6v0d1 | 0.025329 | -2.7391 | 1680 | |
| Psma7 | 0.025361 | -2.6506 | 1681 | |
| Cacna1s | 0.025376 | -2.7772 | 1682 | |
| Ano5 | 0.025475 | -1.2642 | 1683 | |
| Lancl3 | 0.025499 | -2.0324 | 1684 | |
| Gins1 | 0.025507 | -1.2663 | 1685 | |
| Aprt | 0.025519 | 0.49076 | 1686 | |
| Nde1 | 0.025553 | -2.7878 | 1687 | |
| Cpeb4 | 0.025564 | -0.69426 | 1688 | |
| Olfr933 | 0.02557 | -2.0588 | 1689 | |
| 4933405O20Rik | 0.025601 | -1.3776 | 1690 | |
| Fam69c | 0.025609 | 0.23 | 1691 | |
| Adhfe1 | 0.02562 | -2.7949 | 1692 | |
| Gm4937 | 0.025633 | -1.7104 | 1693 | |
| Abt1 | 0.025635 | -2.7579 | 1694 | |
| Tshz1 | 0.025637 | -1.3358 | 1695 | |
| Aif1l | 0.02568 | -1.2663 | 1696 | |
| Lhx2 | 0.025716 | -1.2664 | 1697 | |
| Metap2 | 0.025736 | -1.8721 | 1698 | |
| Gm2016 | 0.025785 | -3.1844 | 1699 | |
| Gm10406 | 0.025788 | -2.5376 | 1700 | |
| Psmd6 | 0.025788 | -0.56198 | 1701 | |
| Olfr497 | 0.025788 | -2.5438 | 1702 | |
| Eif3g | 0.025792 | -2.2061 | 1703 | |
| Lrrc41 | 0.025809 | -2.6077 | 1704 | |
| Mrps34 | 0.025832 | -2.7359 | 1705 | |
| Olfr523 | 0.025845 | -1.2578 | 1706 | |
| Dram1 | 0.025855 | -2.726 | 1707 | |
| mmu-mir-6984 | 0.025872 | -0.10473 | 1708 | |
| Aagab | 0.025873 | -1.8178 | 1709 | |
| Ncbp1 | 0.025903 | -2.5171 | 1710 | |
| Rnpc3 | 0.025903 | -2.5171 | 1711 | |
| Chchd6 | 0.025925 | -1.3694 | 1712 | |
| Ttc37 | 0.026006 | -2.2661 | 1713 | |
| Fbxo47 | 0.026022 | -2.5666 | 1714 | |
| Kdsr | 0.026046 | -1.658 | 1715 | |
| Zfp595 | 0.026105 | -1.7136 | 1716 | |
| Rbmx2 | 0.026165 | -2.71 | 1717 | |
| H2-Oa | 0.026199 | -1.2011 | 1718 | |
| Emp1 | 0.026215 | -1.3609 | 1719 | |
| Nle1 | 0.026219 | -1.2565 | 1720 | |
| Mlkl | 0.026227 | -1.191 | 1721 | |
| Elavl1 | 0.026235 | 0.98166 | 1722 | |
| mmu-mir-7043 | 0.026249 | -1.4233 | 1723 | |
| Taf7 | 0.0263 | -2.2429 | 1724 | |
| Fnta | 0.026316 | -2.2097 | 1725 | |
| Ubr3 | 0.026328 | -1.242 | 1726 | |
| Agtr1a | 0.02634 | -2.7491 | 1727 | |
| Ing3 | 0.026364 | -0.88896 | 1728 | |
| Asun | 0.026369 | -2.509 | 1729 | |
| mmu-mir-7084 | 0.026402 | -1.9457 | 1730 | |
| Olfr1215 | 0.026416 | -1.5558 | 1731 | |
| a | 0.026477 | -1.5771 | 1732 | |
| Gm10778 | 0.026593 | -2.2929 | 1733 | |
| Rpp30 | 0.026593 | -1.6579 | 1734 | |
| Hpgd | 0.026593 | -1.3232 | 1735 | |
| Nsmce1 | 0.026675 | -2.6226 | 1736 | |
| Tmem95 | 0.026684 | -1.8624 | 1737 | |
| 4930453N24Rik | 0.026715 | -2.7622 | 1738 | |
| Fnbp1l | 0.026724 | -1.6679 | 1739 | |
| mmu-mir-543 | 0.026779 | -1.8938 | 1740 | |
| Olfr129 | 0.0268 | -1.2773 | 1741 | |
| Tmem2 | 0.026839 | -0.065448 | 1742 | |
| 4930427A07Rik | 0.026848 | -2.7519 | 1743 | |
| Tmem30a | 0.026859 | -1.4603 | 1744 | |
| Styx | 0.026864 | -2.325 | 1745 | |
| Dctn5 | 0.0269 | -2.6414 | 1746 | |
| Rapsn | 0.02698 | -2.7519 | 1747 | |
| Cyp4f40 | 0.026986 | -1.0755 | 1748 | |
| Lsm14a | 0.026995 | -0.7288 | 1749 | |
| Pum2 | 0.027029 | -2.1863 | 1750 | |
| Letm2 | 0.02704 | -1.9287 | 1751 | |
| Wbp11 | 0.027059 | -1.2456 | 1752 | |
| Trpv6 | 0.027134 | -2.7461 | 1753 | |
| Eif2b1 | 0.027174 | -1.7328 | 1754 | |
| Dux | 0.027178 | -1.8356 | 1755 | |
| Slc25a40 | 0.027178 | -2.6805 | 1756 | |
| Cul9 | 0.027199 | -1.3873 | 1757 | |
| 2010106E10Rik | 0.027206 | -0.16656 | 1758 | |
| Fam175b | 0.027219 | -1.9325 | 1759 | |
| Taf9 | 0.027279 | -2.6414 | 1760 | |
| Gtf3c1 | 0.027279 | -2.6414 | 1761 | |
| Mettl8 | 0.027344 | -1.2654 | 1762 | |
| Gm10354 | 0.027375 | -0.095741 | 1763 | |
| Eif2b2 | 0.027375 | -0.99279 | 1764 | |
| Olfr1160 | 0.027375 | 0.00719 | 1765 | |
| Smarcb1 | 0.027376 | -2.7309 | 1766 | |
| Olfr780 | 0.02741 | -1.3722 | 1767 | |
| Tmem129 | 0.027474 | -1.0791 | 1768 | |
| Zfp457 | 0.027531 | 1.0121 | 1769 | |
| Kdm3b | 0.027555 | -1.8968 | 1770 | |
| Tgm3 | 0.027621 | -1.7961 | 1771 | |
| Vmn2r30 | 0.027624 | -2.9221 | 1772 | |
| Tfdp1 | 0.027637 | -2.6053 | 1773 | |
| Rab10 | 0.027671 | -1.9767 | 1774 | |
| Hjurp | 0.027677 | -3.2744 | 1775 | |
| Cpsf2 | 0.027709 | -1.8486 | 1776 | |
| Tm7sf2 | 0.027718 | -2.7061 | 1777 | |
| mmu-mir-6956 | 0.027719 | -1.868 | 1778 | |
| Cacna1a | 0.027759 | -1.1604 | 1779 | |
| Txnip | 0.027792 | -1.6428 | 1780 | |
| Pigs | 0.027836 | -2.7132 | 1781 | |
| Sema6c | 0.027918 | -1.488 | 1782 | |
| mmu-mir-6929 | 0.027931 | -1.7648 | 1783 | |
| Gm44 | 0.027964 | -1.6546 | 1784 | |
| Pus3 | 0.027985 | -1.9212 | 1785 | |
| Ptms | 0.027996 | -2.6352 | 1786 | |
| Dopey1 | 0.028027 | -1.4329 | 1787 | |
| Mcpt1 | 0.028037 | -2.7275 | 1788 | |
| Slc17a2 | 0.028043 | -1.6098 | 1789 | |
| Tctex1d4 | 0.028045 | -0.032345 | 1790 | |
| Cenpo | 0.028045 | -2.3268 | 1791 | |
| Olfr143 | 0.028045 | -1.6099 | 1792 | |
| Rcc2 | 0.028045 | -1.068 | 1793 | |
| Dhx8 | 0.028045 | -2.1468 | 1794 | |
| Rbm15b | 0.028078 | -1.3686 | 1795 | |
| mmu-mir-1306 | 0.028105 | -0.84466 | 1796 | |
| Prmt1 | 0.028111 | -1.2243 | 1797 | |
| Gm15114 | 0.02812 | -2.1787 | 1798 | |
| Sh3bp5 | 0.028153 | -1.5201 | 1799 | |
| Ier5l | 0.028161 | -1.2183 | 1800 | |
| Adam33 | 0.028212 | -1.1939 | 1801 | |
| Cmc1 | 0.028246 | -1.3932 | 1802 | |
| Drg2 | 0.028258 | -1.2511 | 1803 | |
| Krtap2-4 | 0.028289 | -4.719 | 1804 | |
| Atp5h | 0.028316 | -2.7042 | 1805 | |
| Fam83f | 0.028322 | -1.8486 | 1806 | |
| Gfra3 | 0.028335 | -0.14317 | 1807 | |
| Aifm2 | 0.028349 | -1.152 | 1808 | |
| Serpina9 | 0.028357 | -2.6898 | 1809 | |
| E130012A19Rik | 0.02838 | -1.5427 | 1810 | |
| Ptpn21 | 0.02839 | -2.7061 | 1811 | |
| Ppp1r8 | 0.028403 | -2.6979 | 1812 | |
| Rps17 | 0.02844 | -2.0722 | 1813 | |
| Olfr1364 | 0.028447 | -1.439 | 1814 | |
| mmu-mir-6934 | 0.028473 | -1.9976 | 1815 | |
| mmu-mir-5128 | 0.02849 | -1.7515 | 1816 | |
| Sgce | 0.028506 | -2.7127 | 1817 | |
| Olfr121 | 0.028517 | -2.1167 | 1818 | |
| Tnip3 | 0.028539 | -2.5973 | 1819 | |
| mmu-mir-5615-1 | 0.028559 | -0.29829 | 1820 | |
| Prkab2 | 0.028563 | -1.2123 | 1821 | |
| Psma1 | 0.028568 | -1.4045 | 1822 | |
| Dkc1 | 0.028602 | -2.0166 | 1823 | |
| Hsf5 | 0.028721 | -0.26083 | 1824 | |
| Cd70 | 0.028742 | -1.2005 | 1825 | |
| Zrsr1 | 0.028746 | -2.6869 | 1826 | |
| Rnf40 | 0.028782 | -2.0685 | 1827 | |
| Slc1a6 | 0.028837 | -0.61296 | 1828 | |
| Vmn1r67 | 0.028866 | -2.3445 | 1829 | |
| mmu-mir-6961 | 0.028903 | -1.8466 | 1830 | |
| Olfr267 | 0.028905 | -1.7257 | 1831 | |
| Bbox1 | 0.028962 | -2.651 | 1832 | |
| Skp1a | 0.028965 | -2.5146 | 1833 | |
| Ipp | 0.028978 | -1.0701 | 1834 | |
| Dnaja3 | 0.029027 | -1.7205 | 1835 | |
| Ahdc1 | 0.029027 | -1.8015 | 1836 | |
| Cdc23 | 0.029027 | -1.1684 | 1837 | |
| Il17b | 0.029062 | -1.6636 | 1838 | |
| Hist1h2ad | 0.029095 | -2.6535 | 1839 | |
| Cbr1 | 0.029104 | -2.6928 | 1840 | |
| Homez | 0.029163 | -1.5922 | 1841 | |
| Fermt2 | 0.029187 | -2.6716 | 1842 | |
| Iws1 | 0.02922 | -2.6763 | 1843 | |
| Mroh9 | 0.02929 | -1.2007 | 1844 | |
| mmu-mir-155 | 0.029303 | -1.6198 | 1845 | |
| Slc25a19 | 0.02931 | -1.2028 | 1846 | |
| Klk13 | 0.029312 | -2.6797 | 1847 | |
| Hk2 | 0.029317 | -0.7891 | 1848 | |
| Mfsd1 | 0.029353 | -1.5853 | 1849 | |
| Mrap | 0.029362 | -0.033795 | 1850 | |
| Ackr3 | 0.029379 | -1.1988 | 1851 | |
| Amica1 | 0.029379 | -2.6711 | 1852 | |
| Tnip2 | 0.029383 | -1.1756 | 1853 | |
| Vmn2r55 | 0.029413 | -2.6838 | 1854 | |
| Olfr1097 | 0.02943 | -1.536 | 1855 | |
| Ascl5 | 0.029444 | -1.1954 | 1856 | |
| Olfr1480 | 0.029451 | -2.6461 | 1857 | |
| Pef1 | 0.029451 | -0.15861 | 1858 | |
| Gm8267 | 0.029451 | -1.6465 | 1859 | |
| Slc5a2 | 0.029479 | -1.1937 | 1860 | |
| Ttc33 | 0.029496 | -2.087 | 1861 | |
| Pa2g4 | 0.029547 | -2.6833 | 1862 | |
| Akr1b10 | 0.029622 | -1.199 | 1863 | |
| Sec11a | 0.029656 | -2.6674 | 1864 | |
| Vezt | 0.029674 | -2.1768 | 1865 | |
| Zfand5 | 0.029674 | -1.3409 | 1866 | |
| Gemin6 | 0.029674 | -0.87255 | 1867 | |
| Hsd3b1 | 0.029741 | -2.5841 | 1868 | |
| Ppp2r5e | 0.029757 | -1.7638 | 1869 | |
| Ddit3 | 0.029774 | -2.6205 | 1870 | |
| Med17 | 0.029815 | -1.802 | 1871 | |
| Olfml3 | 0.029892 | -0.5604 | 1872 | |
| Cpb2 | 0.029897 | -0.6146 | 1873 | |
| Car11 | 0.030002 | -0.52874 | 1874 | |
| Atox1 | 0.030012 | -1.2577 | 1875 | |
| Mto1 | 0.030019 | -1.1843 | 1876 | |
| Depdc7 | 0.030045 | -0.10118 | 1877 | |
| Vgll3 | 0.030076 | 0.036054 | 1878 | |
| Olfr1140 | 0.03012 | -0.31824 | 1879 | |
| Akap13 | 0.030148 | -1.7103 | 1880 | |
| Sap30bp | 0.03015 | -2.397 | 1881 | |
| Heatr6 | 0.030261 | -2.1511 | 1882 | |
| Lonp1 | 0.030282 | -2.6578 | 1883 | |
| Jkamp | 0.03029 | -2.1456 | 1884 | |
| Zfp935 | 0.030299 | -2.0807 | 1885 | |
| Zfp281 | 0.030343 | 0.97849 | 1886 | |
| S100b | 0.030343 | 0.63996 | 1887 | |
| Rpn2 | 0.030409 | -2.4435 | 1888 | |
| Oas1d | 0.030452 | -1.0013 | 1889 | |
| Bop1 | 0.030537 | -0.41687 | 1890 | |
| Erlin1 | 0.03054 | -2.5249 | 1891 | |
| Cenpi | 0.030551 | -1.8677 | 1892 | |
| Elmsan1 | 0.030566 | -1.777 | 1893 | |
| Cd36 | 0.030569 | -1.1838 | 1894 | |
| Fbxw18 | 0.030623 | -2.0586 | 1895 | |
| Arc | 0.030655 | -0.25741 | 1896 | |
| Olfr205 | 0.030663 | -1.1757 | 1897 | |
| Olfr512 | 0.030748 | -1.1806 | 1898 | |
| Cul2 | 0.030776 | -1.5067 | 1899 | |
| Bambi | 0.030802 | -2.5765 | 1900 | |
| Zfp85 | 0.030812 | -1.1756 | 1901 | |
| Tufm | 0.030845 | -2.4806 | 1902 | |
| Klf17 | 0.030846 | -0.99996 | 1903 | |
| Cd79b | 0.030853 | -0.42067 | 1904 | |
| Smap1 | 0.030862 | -2.6292 | 1905 | |
| Gucy1b2 | 0.030945 | -0.048076 | 1906 | |
| Rfc2 | 0.030945 | -0.21199 | 1907 | |
| Copa | 0.030945 | -2.2326 | 1908 | |
| Cd209c | 0.030945 | -2.0588 | 1909 | |
| Ssu72 | 0.030945 | 0.53882 | 1910 | |
| mmu-mir-487b | 0.030965 | -0.94827 | 1911 | |
| Lin9 | 0.030967 | -2.5071 | 1912 | |
| Zfp11 | 0.030974 | -0.92515 | 1913 | |
| Olfr665 | 0.031048 | -1.1467 | 1914 | |
| Znfx1 | 0.031068 | -2.6223 | 1915 | |
| Rpf2 | 0.031077 | -2.4831 | 1916 | |
| Snx16 | 0.031147 | -1.2833 | 1917 | |
| Ccdc106 | 0.031183 | -1.8807 | 1918 | |
| Olfr1318 | 0.03119 | -1.026 | 1919 | |
| Olfr957 | 0.03119 | -1.4374 | 1920 | |
| Cycs | 0.03119 | -1.527 | 1921 | |
| Vmn1r179 | 0.031214 | -2.6241 | 1922 | |
| Gm14440 | 0.031224 | -4.591 | 1923 | |
| Msi2 | 0.031237 | -1.1721 | 1924 | |
| Taf8 | 0.03124 | -2.6174 | 1925 | |
| mmu-mir-452 | 0.031253 | -2.5928 | 1926 | |
| Zbtb34 | 0.031269 | -1.7245 | 1927 | |
| mmu-mir-34a | 0.031276 | -2.0162 | 1928 | |
| Wdr59 | 0.031279 | -1.4351 | 1929 | |
| Wwc2 | 0.031282 | -1.294 | 1930 | |
| mmu-mir-669e | 0.031307 | -0.40143 | 1931 | |
| Nelfb | 0.031318 | -2.629 | 1932 | |
| Olfr424 | 0.031324 | -0.12254 | 1933 | |
| Bace2 | 0.031326 | -2.0173 | 1934 | |
| Troap | 0.031335 | -0.62156 | 1935 | |
| Nudt5 | 0.031359 | -1.1707 | 1936 | |
| Cdk5rap3 | 0.031395 | -2.5735 | 1937 | |
| DXBay18 | 0.031447 | -1.4799 | 1938 | |
| C9 | 0.031473 | -2.5815 | 1939 | |
| Zfp142 | 0.031502 | -1.1357 | 1940 | |
| Olfr11 | 0.031508 | -1.703 | 1941 | |
| Alox5ap | 0.031559 | -1.1686 | 1942 | |
| Kmo | 0.031582 | -0.98438 | 1943 | |
| Clasp2 | 0.031586 | -2.2012 | 1944 | |
| Lrguk | 0.03162 | -0.37547 | 1945 | |
| 6720489N17Rik | 0.031636 | 0.45746 | 1946 | |
| Tomm20 | 0.031654 | -1.184 | 1947 | |
| Gm9839 | 0.03168 | 0.63844 | 1948 | |
| Slc25a24 | 0.031707 | -2.5964 | 1949 | |
| Cpe | 0.031732 | -1.2459 | 1950 | |
| Ank | 0.03175 | -1.4762 | 1951 | |
| Usp18 | 0.031759 | -1.2429 | 1952 | |
| Gabpb1 | 0.031814 | -1.0727 | 1953 | |
| Eral1 | 0.03182 | -2.609 | 1954 | |
| mmu-mir-8115 | 0.031865 | -1.9378 | 1955 | |
| Serpina3b | 0.031881 | 0.24226 | 1956 | |
| Lamtor1 | 0.031881 | -1.4764 | 1957 | |
| Rhob | 0.031896 | -1.16 | 1958 | |
| Cdc20 | 0.032011 | -2.5268 | 1959 | |
| Impact | 0.032037 | -1.1538 | 1960 | |
| Arhgdib | 0.03208 | -1.1292 | 1961 | |
| Fam92a | 0.032081 | -0.63289 | 1962 | |
| Olfr669 | 0.032215 | -0.079794 | 1963 | |
| Romo1 | 0.032273 | -1.5391 | 1964 | |
| Scgb1a1 | 0.032295 | -2.5942 | 1965 | |
| Dfnb59 | 0.032319 | -1.5544 | 1966 | |
| Tbkbp1 | 0.03237 | -1.6396 | 1967 | |
| Rpl34 | 0.032385 | -4.4566 | 1968 | |
| Sfmbt1 | 0.032438 | -0.33708 | 1969 | |
| Haus4 | 0.032438 | -0.50544 | 1970 | |
| Olfr148 | 0.032438 | -1.5148 | 1971 | |
| Utp23 | 0.032438 | -0.83485 | 1972 | |
| Ska1 | 0.032438 | -1.0626 | 1973 | |
| Fam173b | 0.032511 | -1.2124 | 1974 | |
| Samd8 | 0.032532 | -1.8964 | 1975 | |
| Gjb1 | 0.03258 | -0.49882 | 1976 | |
| 9130204L05Rik | 0.032583 | -2.3036 | 1977 | |
| Gabrg2 | 0.032593 | -1.2368 | 1978 | |
| Zkscan7 | 0.032648 | -1.1362 | 1979 | |
| Mcu | 0.03266 | -1.7736 | 1980 | |
| Nrtn | 0.032676 | -1.2828 | 1981 | |
| Sec62 | 0.032727 | -1.5268 | 1982 | |
| Klhl20 | 0.032737 | -1.232 | 1983 | |
| Dynll1 | 0.032741 | -1.4582 | 1984 | |
| Mrpl39 | 0.032749 | -2.3485 | 1985 | |
| Cntn5 | 0.032791 | -0.96752 | 1986 | |
| Cebpb | 0.032794 | -2.0639 | 1987 | |
| Ints5 | 0.032844 | -0.88208 | 1988 | |
| Spink8 | 0.032853 | -1.2009 | 1989 | |
| mmu-mir-7033 | 0.032856 | -1.7898 | 1990 | |
| Elp3 | 0.03286 | -0.41221 | 1991 | |
| Fam217a | 0.03286 | 0.37773 | 1992 | |
| Wdr1 | 0.032861 | -2.5712 | 1993 | |
| Olfr517 | 0.032886 | -1.5325 | 1994 | |
| Hist1h3a | 0.032888 | -2.5276 | 1995 | |
| Wdr24 | 0.032941 | -2.5941 | 1996 | |
| Olfr504 | 0.032961 | -1.9079 | 1997 | |
| Ift52 | 0.033003 | -2.5136 | 1998 | |
| Cyp4f16 | 0.03302 | -1.3972 | 1999 | |
| Rrp9 | 0.033047 | -2.5677 | 2000 | |
| Olfr1383 | 0.033061 | -0.31318 | 2001 | |
| Dym | 0.033061 | 0.13292 | 2002 | |
| 0610010F05Rik | 0.033061 | -0.71349 | 2003 | |
| Vmn1r220 | 0.033061 | -2.0562 | 2004 | |
| Mmp20 | 0.033073 | -2.2097 | 2005 | |
| Rpsa | 0.0331 | -2.5828 | 2006 | |
| Zfp800 | 0.033109 | -2.5807 | 2007 | |
| Maoa | 0.033116 | -2.1238 | 2008 | |
| Psmd5 | 0.033171 | -2.3151 | 2009 | |
| Olfr48 | 0.033194 | -1.6757 | 2010 | |
| Yars2 | 0.0332 | -1.5591 | 2011 | |
| Prss43 | 0.033206 | -0.1476 | 2012 | |
| Stk33 | 0.033214 | -1.3324 | 2013 | |
| Vmn2r33 | 0.033264 | -3.195 | 2014 | |
| Agr2 | 0.033283 | 2.9208 | 2015 | |
| Ddx3x | 0.033283 | -0.73405 | 2016 | |
| Hist2h2bb | 0.033283 | -0.43514 | 2017 | |
| Mtor | 0.033348 | -2.5762 | 2018 | |
| Parp4 | 0.033355 | -0.69451 | 2019 | |
| Dock9 | 0.033386 | -1.2459 | 2020 | |
| Pde8b | 0.033461 | -1.2909 | 2021 | |
| Olfr1049 | 0.033478 | -1.2815 | 2022 | |
| mmu-mir-3074-2 | 0.033503 | -2.948 | 2023 | |
| Pink1 | 0.033517 | -2.5753 | 2024 | |
| Ubxn4 | 0.033561 | -1.5721 | 2025 | |
| Olfr1151 | 0.033595 | -0.68227 | 2026 | |
| Tex13 | 0.033606 | -1.0706 | 2027 | |
| Cdkal1 | 0.033615 | -1.7346 | 2028 | |
| Retnla | 0.033642 | -1.4472 | 2029 | |
| Fgf15 | 0.033684 | -0.99224 | 2030 | |
| Ndufaf5 | 0.033696 | -1.874 | 2031 | |
| Mtpap | 0.033749 | -2.5237 | 2032 | |
| Hsd17b10 | 0.03375 | -1.4777 | 2033 | |
| Ncbp2 | 0.033772 | -1.7355 | 2034 | |
| Gm2083 | 0.033806 | -1.8845 | 2035 | |
| Ccdc69 | 0.033829 | -2.5605 | 2036 | |
| Gucy1a2 | 0.033847 | -1.2248 | 2037 | |
| Olfr811 | 0.033853 | -1.4367 | 2038 | |
| Tefm | 0.033862 | -0.6826 | 2039 | |
| Smc3 | 0.033896 | -0.5128 | 2040 | |
| Atp6v1a | 0.033906 | -0.01049 | 2041 | |
| Snip1 | 0.03395 | -1.0617 | 2042 | |
| Bub1 | 0.033972 | -1.1342 | 2043 | |
| Dsc3 | 0.033991 | -0.79919 | 2044 | |
| Olfr437 | 0.03403 | -1.1335 | 2045 | |
| Angptl1 | 0.034035 | -1.1205 | 2046 | |
| Rfc4 | 0.03408 | -2.5215 | 2047 | |
| Dctn4 | 0.034084 | -0.18765 | 2048 | |
| mmu-mir-3971 | 0.034107 | -1.4077 | 2049 | |
| Aspn | 0.034124 | -2.5499 | 2050 | |
| mmu-mir-6960 | 0.034131 | -2.2983 | 2051 | |
| A630073D07Rik | 0.034187 | -1.8534 | 2052 | |
| Rheb | 0.034197 | -1.4702 | 2053 | |
| Aim2 | 0.034218 | -1.1727 | 2054 | |
| Pih1d2 | 0.034218 | 1.7206 | 2055 | |
| 2700050L05Rik | 0.03425 | -1.1942 | 2056 | |
| Triobp | 0.034255 | -1.1325 | 2057 | |
| Spcs3 | 0.03426 | -1.3474 | 2058 | |
| Scn4b | 0.034264 | -1.2515 | 2059 | |
| Pilrb2 | 0.034269 | -1.1312 | 2060 | |
| Mrpl35 | 0.034273 | -2.5559 | 2061 | |
| Olfr707 | 0.034295 | -2.5389 | 2062 | |
| Olfr598 | 0.034349 | -1.1295 | 2063 | |
| Sdc4 | 0.034356 | -1.2706 | 2064 | |
| Wasf2 | 0.034418 | -1.4384 | 2065 | |
| Chst3 | 0.034442 | -1.1289 | 2066 | |
| Lgals2 | 0.034442 | -1.2456 | 2067 | |
| Gins2 | 0.034462 | -1.4586 | 2068 | |
| Utp11l | 0.034466 | -2.4422 | 2069 | |
| Fam126a | 0.034551 | -0.78449 | 2070 | |
| Scn3b | 0.034551 | -2.4793 | 2071 | |
| Vbp1 | 0.034551 | -1.1102 | 2072 | |
| Gpatch8 | 0.034551 | -0.85505 | 2073 | |
| Olfr832 | 0.034551 | -0.70697 | 2074 | |
| Timeless | 0.034615 | -2.5148 | 2075 | |
| Kin | 0.034616 | -2.1022 | 2076 | |
| Ralgapb | 0.034642 | -0.4669 | 2077 | |
| Dhx9 | 0.034645 | -1.6812 | 2078 | |
| Obox2 | 0.034665 | -0.53044 | 2079 | |
| mmu-mir-1983 | 0.03467 | -1.4013 | 2080 | |
| Mocs3 | 0.03471 | -2.5323 | 2081 | |
| mmu-mir-6337 | 0.034727 | -1.8473 | 2082 | |
| Cox6a1 | 0.034728 | -0.88975 | 2083 | |
| Olfr1110 | 0.034752 | -1.6111 | 2084 | |
| A630023A22Rik | 0.034796 | -0.98513 | 2085 | |
| Stard3 | 0.0348 | -2.0441 | 2086 | |
| mmu-mir-6922 | 0.034801 | -1.2311 | 2087 | |
| Chac1 | 0.034883 | -1.1121 | 2088 | |
| Fam122c | 0.034907 | -1.4712 | 2089 | |
| Ndufs5 | 0.034907 | 0.35978 | 2090 | |
| Igip | 0.034922 | -1.2413 | 2091 | |
| Sirpb1b | 0.034927 | -2.527 | 2092 | |
| Trappc1 | 0.034936 | -1.4492 | 2093 | |
| Olfr855 | 0.034946 | -2.1707 | 2094 | |
| Esco2 | 0.03495 | -2.1264 | 2095 | |
| Gart | 0.034961 | -1.6745 | 2096 | |
| Rbpms2 | 0.034972 | -2.53 | 2097 | |
| Zfp830 | 0.035004 | -2.3143 | 2098 | |
| Ints8 | 0.035018 | 0.83481 | 2099 | |
| Sos2 | 0.035029 | -1.2942 | 2100 | |
| Unc45a | 0.035034 | -1.5787 | 2101 | |
| Celsr2 | 0.035038 | -1.1174 | 2102 | |
| mmu-mir-872 | 0.035054 | -1.6137 | 2103 | |
| Zik1 | 0.035062 | -0.89715 | 2104 | |
| Gmds | 0.0351 | -2.5342 | 2105 | |
| Noc2l | 0.035107 | -2.5214 | 2106 | |
| Eif5b | 0.035146 | -1.5957 | 2107 | |
| Il31 | 0.035173 | -1.012 | 2108 | |
| Nxpe2 | 0.035209 | -0.58894 | 2109 | |
| Zzz3 | 0.035227 | -2.1197 | 2110 | |
| Rspry1 | 0.035245 | -1.7241 | 2111 | |
| Abhd14b | 0.035258 | -1.202 | 2112 | |
| Nphp1 | 0.035346 | -1.4475 | 2113 | |
| Ebag9 | 0.035373 | -0.11688 | 2114 | |
| Fcrlb | 0.035382 | -2.517 | 2115 | |
| Ubxn7 | 0.0354 | -1.5023 | 2116 | |
| mmu-mir-7679 | 0.035422 | -1.0257 | 2117 | |
| Pcdhac1 | 0.035441 | -1.3397 | 2118 | |
| Slc25a51 | 0.035489 | -1.1096 | 2119 | |
| Cdk13 | 0.035528 | -1.9443 | 2120 | |
| Hsd17b11 | 0.035529 | -2.0068 | 2121 | |
| Fam76b | 0.035529 | 2.3751 | 2122 | |
| Hbb-bh1 | 0.035538 | -1.6342 | 2123 | |
| Bhlhe23 | 0.035563 | -1.3943 | 2124 | |
| mmu-mir-26a-2 | 0.035616 | -2.1138 | 2125 | |
| mmu-mir-7094-1 | 0.035624 | -1.2539 | 2126 | |
| Plrg1 | 0.035684 | -1.0357 | 2127 | |
| mmu-mir-1907 | 0.035685 | -1.1629 | 2128 | |
| Syt4 | 0.035691 | -2.3591 | 2129 | |
| Fcer1a | 0.035729 | -1.5702 | 2130 | |
| Xkr9 | 0.035745 | -1.1107 | 2131 | |
| 4930579F01Rik | 0.035793 | -2.488 | 2132 | |
| Id2 | 0.035795 | -0.88804 | 2133 | |
| Hccs | 0.035795 | -0.78402 | 2134 | |
| Gm5458 | 0.035819 | -1.5644 | 2135 | |
| mmu-mir-423 | 0.035822 | -1.8316 | 2136 | |
| Tm9sf3 | 0.035867 | -2.3966 | 2137 | |
| Crip1 | 0.035912 | -1.3805 | 2138 | |
| Dppa1 | 0.035921 | -1.5366 | 2139 | |
| Psmb10 | 0.035973 | -1.5173 | 2140 | |
| E130309D02Rik | 0.035973 | -2.2369 | 2141 | |
| Gm5938 | 0.035973 | -1.227 | 2142 | |
| mmu-mir-680-1 | 0.035984 | -2.2764 | 2143 | |
| Naa60 | 0.036022 | -1.1949 | 2144 | |
| Olfr222 | 0.036037 | -0.70106 | 2145 | |
| mmu-mir-6986 | 0.036045 | -1.3386 | 2146 | |
| Olfr1382 | 0.036064 | -2.5096 | 2147 | |
| Il13ra1 | 0.036065 | -1.4334 | 2148 | |
| Tpgs2 | 0.036117 | -1.4521 | 2149 | |
| Panx3 | 0.036124 | -2.4959 | 2150 | |
| Xpnpep3 | 0.036151 | 0.85321 | 2151 | |
| Sod1 | 0.036225 | -2.4969 | 2152 | |
| Spata25 | 0.036225 | -2.4969 | 2153 | |
| Elp5 | 0.036262 | -0.72422 | 2154 | |
| Nucb1 | 0.036262 | -0.19528 | 2155 | |
| Arhgdia | 0.036286 | -2.1564 | 2156 | |
| Mipep | 0.036306 | -2.1855 | 2157 | |
| Cmtr1 | 0.036351 | -2.036 | 2158 | |
| E4f1 | 0.036416 | -1.5942 | 2159 | |
| Phtf2 | 0.036417 | -1.5292 | 2160 | |
| Mthfr | 0.036481 | -1.3588 | 2161 | |
| Exoc3 | 0.036528 | -1.1624 | 2162 | |
| Lsm8 | 0.036528 | -0.57107 | 2163 | |
| Olfr672 | 0.036617 | -2.4936 | 2164 | |
| Phrf1 | 0.036627 | -2.4921 | 2165 | |
| Gpx3 | 0.036672 | -1.5353 | 2166 | |
| Olfr482 | 0.036682 | -1.3695 | 2167 | |
| Ric8 | 0.036687 | -2.4936 | 2168 | |
| Ghrh | 0.036697 | -1.0714 | 2169 | |
| Rnf217 | 0.036706 | -1.4606 | 2170 | |
| Pan2 | 0.036715 | -2.4179 | 2171 | |
| Olfr963 | 0.036725 | -0.58066 | 2172 | |
| Ccl2 | 0.036734 | -2.5033 | 2173 | |
| Kir3dl1 | 0.03675 | -1.2631 | 2174 | |
| Nr2e3 | 0.036772 | -1.5655 | 2175 | |
| Hist1h2ag | 0.036772 | -1.5116 | 2176 | |
| Ctdspl2 | 0.036799 | -2.505 | 2177 | |
| Ankib1 | 0.036836 | -2.4513 | 2178 | |
| Smpx | 0.036858 | -1.8085 | 2179 | |
| Lpar4 | 0.036918 | -1.0379 | 2180 | |
| Grb14 | 0.036947 | -2.1157 | 2181 | |
| Cog3 | 0.036999 | -1.4925 | 2182 | |
| Orc4 | 0.037039 | 1.6332 | 2183 | |
| Atp6v1d | 0.037039 | 0.26876 | 2184 | |
| Ndufa3 | 0.037045 | -1.7144 | 2185 | |
| Rhobtb3 | 0.03709 | -1.1054 | 2186 | |
| Pttg1ip | 0.037131 | -1.115 | 2187 | |
| Olfr558 | 0.037136 | -1.4098 | 2188 | |
| Asb18 | 0.037166 | -1.2201 | 2189 | |
| Tcl1b4 | 0.037278 | -1.0546 | 2190 | |
| Mrps30 | 0.037339 | -2.4921 | 2191 | |
| Olfr1123 | 0.037372 | -0.94724 | 2192 | |
| Mdp1 | 0.037415 | -1.2479 | 2193 | |
| Dmrt1 | 0.037442 | -2.487 | 2194 | |
| Slc29a4 | 0.03746 | -0.55981 | 2195 | |
| mmu-mir-7656 | 0.037488 | -1.6251 | 2196 | |
| Camkv | 0.037505 | -1.0734 | 2197 | |
| Dnajc5 | 0.037554 | -0.97752 | 2198 | |
| Snx5 | 0.037568 | -2.4778 | 2199 | |
| Rhoh | 0.037578 | -1.0839 | 2200 | |
| Vars2 | 0.037592 | -2.492 | 2201 | |
| Olfr1441 | 0.037593 | -0.71976 | 2202 | |
| Car1 | 0.03764 | -1.4611 | 2203 | |
| Sec24b | 0.037704 | -2.2997 | 2204 | |
| Fam83c | 0.037711 | -1.9616 | 2205 | |
| Pglyrp1 | 0.037732 | -1.5196 | 2206 | |
| Dhx37 | 0.037779 | -1.127 | 2207 | |
| mmu-mir-5112 | 0.037786 | -1.29 | 2208 | |
| Surf6 | 0.037837 | 0.24967 | 2209 | |
| Dph6 | 0.037837 | -1.9611 | 2210 | |
| Wdr12 | 0.037837 | -0.91172 | 2211 | |
| Hus1b | 0.037837 | 0.24986 | 2212 | |
| Grwd1 | 0.037837 | -2.2686 | 2213 | |
| Rtfdc1 | 0.037837 | -2.3422 | 2214 | |
| Rhbdf1 | 0.03785 | -1.7569 | 2215 | |
| Vmn1r86 | 0.037869 | -2.3726 | 2216 | |
| Gm6086 | 0.037885 | -1.0729 | 2217 | |
| Gm597 | 0.037988 | -1.0819 | 2218 | |
| Ppp6c | 0.038014 | -1.313 | 2219 | |
| 4930452B06Rik | 0.038045 | -1.5505 | 2220 | |
| Eef2 | 0.0381 | -0.89784 | 2221 | |
| Srpx2 | 0.038126 | 0.26059 | 2222 | |
| Cul5 | 0.038132 | -1.4781 | 2223 | |
| Tex30 | 0.038137 | -2.449 | 2224 | |
| Tanc1 | 0.038203 | -2.4781 | 2225 | |
| D5Ertd577e | 0.038209 | -1.8012 | 2226 | |
| Rpl36a | 0.038214 | -0.66086 | 2227 | |
| Bcl11b | 0.038259 | -1.9745 | 2228 | |
| Tma16 | 0.038279 | -2.4617 | 2229 | |
| Ctbs | 0.038287 | -1.0943 | 2230 | |
| Olfr151 | 0.038326 | -2.4765 | 2231 | |
| mmu-mir-7090 | 0.038334 | -0.81035 | 2232 | |
| Ccne2 | 0.038348 | -0.45206 | 2233 | |
| Sra1 | 0.03836 | -1.6928 | 2234 | |
| Hax1 | 0.038365 | -1.3433 | 2235 | |
| Nudt21 | 0.038406 | -1.0781 | 2236 | |
| Rnf216 | 0.038422 | -1.0451 | 2237 | |
| Olfr1497 | 0.038427 | -1.4548 | 2238 | |
| F11 | 0.038481 | 0.52974 | 2239 | |
| mmu-mir-376b | 0.038514 | -2.0553 | 2240 | |
| mmu-mir-196a-1 | 0.038514 | -1.548 | 2241 | |
| Fam178b | 0.038569 | -0.86531 | 2242 | |
| mmu-mir-3108 | 0.03857 | -0.49068 | 2243 | |
| Szt2 | 0.038609 | -1.3358 | 2244 | |
| Kti12 | 0.038615 | -2.4555 | 2245 | |
| Krtap14 | 0.038615 | -2.4555 | 2246 | |
| mmu-mir-327 | 0.038634 | -1.4639 | 2247 | |
| Laptm4b | 0.038648 | -2.4681 | 2248 | |
| mmu-mir-7651 | 0.038656 | -1.6573 | 2249 | |
| Olfr292 | 0.038667 | -2.4288 | 2250 | |
| Gngt1 | 0.038729 | -0.89548 | 2251 | |
| Tmem135 | 0.038769 | 0.84812 | 2252 | |
| Polr3a | 0.038769 | -0.058059 | 2253 | |
| Slc22a4 | 0.038769 | -0.84769 | 2254 | |
| Ankrd27 | 0.038769 | -0.76841 | 2255 | |
| Fopnl | 0.03877 | -1.3281 | 2256 | |
| Inhbb | 0.038786 | -1.5437 | 2257 | |
| Tmem239 | 0.038791 | -2.0343 | 2258 | |
| Meiob | 0.038968 | -0.67421 | 2259 | |
| 1700030F18Rik | 0.039013 | -0.15515 | 2260 | |
| Ccnt2 | 0.039032 | -1.071 | 2261 | |
| Psmg3 | 0.039037 | -1.1058 | 2262 | |
| Zfp397 | 0.039057 | -1.3268 | 2263 | |
| Rpa1 | 0.0391 | -2.4023 | 2264 | |
| Rpl21 | 0.0391 | -2.4023 | 2265 | |
| Wdr5 | 0.0391 | -2.4023 | 2266 | |
| Napa | 0.0391 | -2.0722 | 2267 | |
| Prp2 | 0.039152 | -1.0609 | 2268 | |
| Fam26f | 0.039152 | -1.1425 | 2269 | |
| D030056L22Rik | 0.039178 | -1.0569 | 2270 | |
| Rad51b | 0.03922 | -1.8904 | 2271 | |
| Aebp1 | 0.039234 | 0.2784 | 2272 | |
| Dppa5a | 0.039234 | -1.0718 | 2273 | |
| Stxbp4 | 0.039283 | -1.118 | 2274 | |
| Eif3c | 0.039306 | -2.4528 | 2275 | |
| Nkap | 0.039315 | -1.1827 | 2276 | |
| Pphln1 | 0.039325 | -2.4606 | 2277 | |
| Irf1 | 0.039384 | -1.0815 | 2278 | |
| Olfr1281 | 0.039392 | -0.78111 | 2279 | |
| 2410141K09Rik | 0.039411 | -1.0651 | 2280 | |
| Lin7b | 0.039436 | -1.4605 | 2281 | |
| 1810033B17Rik | 0.039455 | -1.4773 | 2282 | |
| Cyp1a2 | 0.039456 | -1.0949 | 2283 | |
| Olfr1180 | 0.039463 | -1.1372 | 2284 | |
| C330027C09Rik | 0.039489 | -1.3061 | 2285 | |
| Tpst1 | 0.0395 | -0.70859 | 2286 | |
| Hist2h3c1 | 0.039519 | -1.8152 | 2287 | |
| Ppp2r5a | 0.039544 | 1.8013 | 2288 | |
| Gsc2 | 0.039589 | -0.72159 | 2289 | |
| Igsf8 | 0.039632 | -2.437 | 2290 | |
| Timm10 | 0.039655 | -1.5158 | 2291 | |
| Slco1a5 | 0.039655 | 0.36999 | 2292 | |
| Mrps21 | 0.039706 | -1.4287 | 2293 | |
| Zc3h14 | 0.039737 | -1.319 | 2294 | |
| Ticam1 | 0.03974 | -1.391 | 2295 | |
| Dnajc8 | 0.039785 | -2.198 | 2296 | |
| Zc2hc1a | 0.039854 | -1.2532 | 2297 | |
| Zfhx3 | 0.039882 | -0.73297 | 2298 | |
| Rlim | 0.039899 | -0.60037 | 2299 | |
| Mrs2 | 0.039899 | -1.3829 | 2300 | |
| Mrpl38 | 0.039915 | -2.4481 | 2301 | |
| Orc6 | 0.039929 | -1.5721 | 2302 | |
| Pde8a | 0.03993 | -2.4312 | 2303 | |
| 4930432K09Rik | 0.039939 | -1.1969 | 2304 | |
| Phgdh | 0.03995 | -1.7684 | 2305 | |
| Sugp1 | 0.039993 | -1.2318 | 2306 | |
| Ctsd | 0.039997 | -2.4427 | 2307 | |
| Rabggtb | 0.040024 | -2.1613 | 2308 | |
| Dsg1a | 0.040026 | -1.7576 | 2309 | |
| Gm16130 | 0.040038 | -1.5991 | 2310 | |
| Gbas | 0.040084 | -2.447 | 2311 | |
| Itpr3 | 0.04012 | -1.4866 | 2312 | |
| Gatad2b | 0.040132 | -1.7496 | 2313 | |
| Sept8 | 0.040142 | -0.59366 | 2314 | |
| Zfyve16 | 0.040168 | -1.5826 | 2315 | |
| Fam118b | 0.040184 | -1.0581 | 2316 | |
| Slc7a12 | 0.040185 | -1.1881 | 2317 | |
| Riok1 | 0.040187 | -1.86 | 2318 | |
| mmu-mir-7055 | 0.040292 | -0.66378 | 2319 | |
| Drc1 | 0.040318 | -1.0533 | 2320 | |
| Arhgap11a | 0.040335 | -1.9027 | 2321 | |
| Slc35a1 | 0.040364 | -2.029 | 2322 | |
| Adtrp | 0.040393 | -1.2278 | 2323 | |
| Olfr907 | 0.040413 | -2.4334 | 2324 | |
| Numa1 | 0.040415 | -2.0127 | 2325 | |
| Ptger2 | 0.04042 | -1.0529 | 2326 | |
| Ang2 | 0.04043 | -0.45684 | 2327 | |
| Tmem54 | 0.040432 | -2.427 | 2328 | |
| Tle4 | 0.040495 | -1.0472 | 2329 | |
| Gprc5c | 0.040533 | -1.0906 | 2330 | |
| Trpc1 | 0.040546 | -1.9415 | 2331 | |
| Prph2 | 0.040568 | -1.27 | 2332 | |
| Zmpste24 | 0.040597 | -2.4363 | 2333 | |
| Kirrel2 | 0.040607 | 0.12507 | 2334 | |
| Cnot11 | 0.040607 | -1.722 | 2335 | |
| Dhrs2 | 0.040608 | -1.0418 | 2336 | |
| Api5 | 0.040627 | -1.1447 | 2337 | |
| Caml | 0.040627 | -1.0483 | 2338 | |
| C1qtnf3 | 0.040655 | -2.4311 | 2339 | |
| Mrps28 | 0.040679 | -2.425 | 2340 | |
| Igf1r | 0.040696 | 0.23694 | 2341 | |
| Tcl1b2 | 0.040733 | -1.7827 | 2342 | |
| Fah | 0.040737 | -1.0511 | 2343 | |
| Ggt5 | 0.040759 | -1.0344 | 2344 | |
| Metap1 | 0.040791 | -1.5634 | 2345 | |
| Ssx9 | 0.040812 | -0.72821 | 2346 | |
| Rabif | 0.04083 | -2.0634 | 2347 | |
| D6Ertd527e | 0.04085 | -1.5953 | 2348 | |
| mmu-mir-466f-3 | 0.040859 | -3.9069 | 2349 | |
| Pelp1 | 0.040859 | -2.4287 | 2350 | |
| Copb2 | 0.040895 | -0.39942 | 2351 | |
| Fign | 0.040895 | 0.45239 | 2352 | |
| Mycbpap | 0.040895 | -1.0414 | 2353 | |
| Etohi1 | 0.040895 | -0.089915 | 2354 | |
| Pcnp | 0.040899 | -2.1431 | 2355 | |
| Nmnat1 | 0.04098 | -1.5991 | 2356 | |
| Pinx1 | 0.041018 | -1.1664 | 2357 | |
| Mrpl53 | 0.041023 | -0.9763 | 2358 | |
| Pprc1 | 0.041035 | -2.4202 | 2359 | |
| Slc44a2 | 0.041054 | -0.92241 | 2360 | |
| Olfr136 | 0.041094 | 0.57846 | 2361 | |
| Dhdds | 0.041103 | -2.4075 | 2362 | |
| Trim55 | 0.041139 | -0.069132 | 2363 | |
| Ngly1 | 0.041176 | -2.4153 | 2364 | |
| Dis3l | 0.04118 | -1.491 | 2365 | |
| Btbd16 | 0.041201 | -2.3969 | 2366 | |
| Sfswap | 0.041208 | -2.3127 | 2367 | |
| Atp1a1 | 0.041213 | -1.0577 | 2368 | |
| Casz1 | 0.04123 | -0.54994 | 2369 | |
| Serpinb10 | 0.04124 | -0.98306 | 2370 | |
| Armcx1 | 0.04124 | -1.0277 | 2371 | |
| Slc26a1 | 0.041259 | -0.29814 | 2372 | |
| 1700123I01Rik | 0.041259 | -1.0891 | 2373 | |
| Slc47a1 | 0.041271 | -0.44121 | 2374 | |
| Ikzf1 | 0.041284 | -1.0435 | 2375 | |
| Mfsd2a | 0.041289 | -0.77781 | 2376 | |
| Pgap1 | 0.041338 | -0.88233 | 2377 | |
| Lrch3 | 0.041338 | -1.1627 | 2378 | |
| Phf11a | 0.041354 | -1.5232 | 2379 | |
| Ndufb10 | 0.041373 | -1.8931 | 2380 | |
| Tbc1d24 | 0.041373 | -1.1194 | 2381 | |
| Rfpl4b | 0.041403 | -1.1508 | 2382 | |
| Igsf6 | 0.041416 | -2.4208 | 2383 | |
| Fmod | 0.041493 | -1.2923 | 2384 | |
| Bcl2l10 | 0.041514 | -2.3847 | 2385 | |
| Dclre1b | 0.04154 | -0.83125 | 2386 | |
| Atp6v1e1 | 0.041581 | -1.9194 | 2387 | |
| Tcp10c | 0.041581 | 0.031343 | 2388 | |
| Atg13 | 0.041581 | -0.46646 | 2389 | |
| Vmn2r31 | 0.0416 | -1.2933 | 2390 | |
| Epb4.1l4a | 0.041616 | -1.9949 | 2391 | |
| Mroh2a | 0.041693 | -1.2323 | 2392 | |
| Hpdl | 0.04172 | -2.4104 | 2393 | |
| Siah2 | 0.041753 | -1.0635 | 2394 | |
| Pstpip1 | 0.041769 | -1.9494 | 2395 | |
| Uba5 | 0.041828 | -0.49245 | 2396 | |
| Fam92b | 0.041838 | -2.4123 | 2397 | |
| Ndufb7 | 0.041863 | -0.83879 | 2398 | |
| Eif5 | 0.041927 | -0.089262 | 2399 | |
| Zfp825 | 0.041962 | -0.61604 | 2400 | |
| Epb4.1 | 0.042024 | -1.1519 | 2401 | |
| St8sia3 | 0.042055 | -0.96129 | 2402 | |
| Olfr1188 | 0.042163 | -2.2645 | 2403 | |
| Sirt3 | 0.042174 | -1.0274 | 2404 | |
| Phlpp2 | 0.042187 | -1.6978 | 2405 | |
| Slc25a36 | 0.0422 | -0.39198 | 2406 | |
| Top2a | 0.0422 | -1.6524 | 2407 | |
| Olfr263 | 0.0422 | 0.15408 | 2408 | |
| Lrrk2 | 0.0422 | 0.04881 | 2409 | |
| Ms4a13 | 0.0422 | -0.71452 | 2410 | |
| Gm20831 | 0.042248 | -1.1718 | 2411 | |
| 2810021J22Rik | 0.042262 | -0.7378 | 2412 | |
| Gckr | 0.042265 | -1.0706 | 2413 | |
| Angpt4 | 0.042331 | -0.74555 | 2414 | |
| Elp2 | 0.0424 | -2.403 | 2415 | |
| Usp35 | 0.042408 | -0.58439 | 2416 | |
| Fbxl15 | 0.04243 | -1.1047 | 2417 | |
| Eln | 0.042466 | -1.9395 | 2418 | |
| Coasy | 0.042497 | -1.4271 | 2419 | |
| BC018507 | 0.042509 | -2.4064 | 2420 | |
| mmu-mir-103-1 | 0.042545 | -0.50309 | 2421 | |
| 1700003M02Rik | 0.042552 | -0.9535 | 2422 | |
| Slc30a3 | 0.042586 | -1.1446 | 2423 | |
| Cdc7 | 0.042621 | -0.5101 | 2424 | |
| Adcy8 | 0.042621 | -0.64307 | 2425 | |
| mmu-mir-8101 | 0.042636 | -2.0993 | 2426 | |
| Cdk14 | 0.042718 | -2.3839 | 2427 | |
| Gm15104 | 0.042728 | -0.34206 | 2428 | |
| Fam81a | 0.042731 | -1.9957 | 2429 | |
| Defb2 | 0.042773 | -2.1209 | 2430 | |
| Hebp1 | 0.042775 | -0.25282 | 2431 | |
| Cobl | 0.0428 | -1.5291 | 2432 | |
| Clec4a4 | 0.042819 | -0.57615 | 2433 | |
| Wdr33 | 0.042827 | -0.7271 | 2434 | |
| Jak1 | 0.042847 | -2.3984 | 2435 | |
| Foxm1 | 0.042875 | -2.0669 | 2436 | |
| Pnkp | 0.042881 | -2.3079 | 2437 | |
| Gm13242 | 0.042892 | -1.7636 | 2438 | |
| Lif | 0.042897 | -2.3963 | 2439 | |
| BC048546 | 0.042907 | -2.1704 | 2440 | |
| Trpc4ap | 0.042908 | -1.6105 | 2441 | |
| Rdx | 0.042952 | -0.09396 | 2442 | |
| Naca | 0.042952 | -1.5799 | 2443 | |
| mmu-mir-7061 | 0.042967 | -1.8292 | 2444 | |
| Rab6b | 0.042992 | -1.5281 | 2445 | |
| Fam115a | 0.043006 | -2.3801 | 2446 | |
| Fkbp5 | 0.043037 | -1.019 | 2447 | |
| mmu-mir-302a | 0.043041 | -1.7386 | 2448 | |
| Gmnn | 0.043076 | -2.3644 | 2449 | |
| Nub1 | 0.043085 | -0.81163 | 2450 | |
| Zfp420 | 0.04311 | -1.4902 | 2451 | |
| Pkn3 | 0.043124 | -1.4348 | 2452 | |
| Cdk11b | 0.043156 | -2.3865 | 2453 | |
| Prkcq | 0.043195 | -0.60074 | 2454 | |
| Wdr13 | 0.043199 | -1.1301 | 2455 | |
| Gm14288 | 0.043221 | -2.5151 | 2456 | |
| Ubap1 | 0.04325 | -1.0272 | 2457 | |
| Vmn2r74 | 0.043261 | -0.59049 | 2458 | |
| Apol11b | 0.043306 | -1.8444 | 2459 | |
| Ctcf | 0.04332 | -2.3694 | 2460 | |
| Kif20a | 0.04335 | -2.3742 | 2461 | |
| Mas1 | 0.043469 | -1.193 | 2462 | |
| Rnf170 | 0.043491 | -1.129 | 2463 | |
| Procr | 0.043519 | -2.3141 | 2464 | |
| Olfr710 | 0.043545 | -0.037372 | 2465 | |
| Prdm12 | 0.043559 | -1.7325 | 2466 | |
| Stk36 | 0.04357 | -1.2154 | 2467 | |
| Kansl1 | 0.043637 | -2.0876 | 2468 | |
| Vmn1r41 | 0.043637 | 1.3956 | 2469 | |
| Sgk3 | 0.043658 | -1.5176 | 2470 | |
| Cenpc1 | 0.043686 | -1.6553 | 2471 | |
| Arf4 | 0.043716 | -2.2587 | 2472 | |
| Snrpe | 0.043717 | -1.4968 | 2473 | |
| Tctn2 | 0.043728 | -1.1039 | 2474 | |
| Slc5a10 | 0.043731 | -2.3627 | 2475 | |
| Bub3 | 0.043746 | -2.382 | 2476 | |
| Maml2 | 0.043796 | -1.7268 | 2477 | |
| 9930111J21Rik2 | 0.043797 | -2.2918 | 2478 | |
| mmu-mir-3088 | 0.043801 | -0.54882 | 2479 | |
| mmu-mir-1946b | 0.043821 | -1.2131 | 2480 | |
| Smpd1 | 0.043836 | -1.6902 | 2481 | |
| Phc3 | 0.043867 | -2.3653 | 2482 | |
| EU599041 | 0.04387 | -1.8084 | 2483 | |
| mmu-mir-7234 | 0.043876 | -1.3234 | 2484 | |
| Vmn2r27 | 0.043886 | -1.7065 | 2485 | |
| Sept14 | 0.043887 | -2.3772 | 2486 | |
| Krtap15 | 0.043897 | -2.2738 | 2487 | |
| Pik3r1 | 0.043902 | 0.59519 | 2488 | |
| Brk1 | 0.043902 | -1.15 | 2489 | |
| Ptx3 | 0.043932 | -1.2682 | 2490 | |
| Icosl | 0.043966 | -1.1246 | 2491 | |
| Dnajc21 | 0.043968 | 0.38631 | 2492 | |
| Birc2 | 0.043987 | -2.2385 | 2493 | |
| Fbxl20 | 0.043989 | -0.99351 | 2494 | |
| Sap18 | 0.044017 | -0.57676 | 2495 | |
| Larp4b | 0.044027 | -0.32671 | 2496 | |
| Rcn2 | 0.044035 | 0.73706 | 2497 | |
| Ccdc67 | 0.044035 | 0.48118 | 2498 | |
| Elof1 | 0.044054 | -1.5904 | 2499 | |
| mmu-mir-141 | 0.044087 | -1.4235 | 2500 | |
| Olfr109 | 0.044148 | -2.0874 | 2501 | |
| Ipo9 | 0.044167 | -1.3057 | 2502 | |
| St6galnac2 | 0.044184 | -1.0117 | 2503 | |
| Gm4567 | 0.044199 | -2.3539 | 2504 | |
| Ipo13 | 0.044278 | 0.62173 | 2505 | |
| Grm5 | 0.044278 | -1.3446 | 2506 | |
| Olfr768 | 0.044278 | 0.46404 | 2507 | |
| Peg12 | 0.044313 | -1.0119 | 2508 | |
| Rcbtb1 | 0.04434 | -2.3723 | 2509 | |
| mmu-mir-147 | 0.044368 | -1.1459 | 2510 | |
| Mnt | 0.04438 | -2.3716 | 2511 | |
| Sgk1 | 0.044392 | -1.3332 | 2512 | |
| Vcl | 0.044432 | -1.7969 | 2513 | |
| Fam115e | 0.044441 | -2.3699 | 2514 | |
| Dock2 | 0.044492 | -2.3299 | 2515 | |
| Vmn2r86 | 0.044512 | -1.1932 | 2516 | |
| Olah | 0.044512 | -1.2504 | 2517 | |
| Olfr509 | 0.04452 | -0.53239 | 2518 | |
| Ube2h | 0.04452 | -1.0425 | 2519 | |
| Tmem92 | 0.044521 | -1.7147 | 2520 | |
| Enpp5 | 0.044587 | 0.60735 | 2521 | |
| mmu-mir-7041 | 0.044599 | -1.6039 | 2522 | |
| Ppil3 | 0.044638 | -1.3308 | 2523 | |
| Tcta | 0.044687 | -1.0035 | 2524 | |
| Olfr1251 | 0.044719 | -2.3171 | 2525 | |
| mmu-mir-7655 | 0.044768 | -0.49533 | 2526 | |
| Olfr1082 | 0.044782 | -1.3523 | 2527 | |
| Klra3 | 0.044785 | -2.0619 | 2528 | |
| Kcnj4 | 0.044806 | -2.3596 | 2529 | |
| 1700013D24Rik | 0.044845 | -0.99439 | 2530 | |
| Olfr275 | 0.044877 | -2.2512 | 2531 | |
| Hist1h2bf | 0.044877 | -2.8561 | 2532 | |
| Camk1d | 0.044896 | -0.095391 | 2533 | |
| Fxyd6 | 0.04494 | 0.16512 | 2534 | |
| Prdm15 | 0.044943 | -0.98527 | 2535 | |
| Rab34 | 0.044954 | -1.2497 | 2536 | |
| Mkln1 | 0.04496 | -1.0644 | 2537 | |
| Hnrnpf | 0.044968 | -2.358 | 2538 | |
| Fez2 | 0.044984 | -0.37619 | 2539 | |
| Rnf139 | 0.045035 | -1.2839 | 2540 | |
| Cited4 | 0.045039 | -2.3596 | 2541 | |
| Ext1 | 0.045058 | -2.3386 | 2542 | |
| Fbll1 | 0.04506 | -2.1146 | 2543 | |
| Agbl1 | 0.045081 | -0.9972 | 2544 | |
| Rbm12b1 | 0.045127 | -1.0309 | 2545 | |
| Abcg3 | 0.045133 | -0.96203 | 2546 | |
| Per2 | 0.04515 | -2.0179 | 2547 | |
| Vmn1r45 | 0.045161 | -2.3369 | 2548 | |
| Ypel5 | 0.045162 | -2.0816 | 2549 | |
| Prl8a6 | 0.045202 | -0.53378 | 2550 | |
| Olfr397 | 0.045237 | -1.2035 | 2551 | |
| mmu-mir-6416 | 0.045278 | -1.2066 | 2552 | |
| Vmn1r3 | 0.04528 | -2.9972 | 2553 | |
| Cd96 | 0.045294 | -1.5767 | 2554 | |
| Haus6 | 0.045304 | -2.3513 | 2555 | |
| Gm14306 | 0.04534 | -1.935 | 2556 | |
| Gm7173 | 0.045359 | -0.69434 | 2557 | |
| Foxr1 | 0.045359 | -1.6549 | 2558 | |
| Il7 | 0.045365 | -1.1348 | 2559 | |
| Polr2l | 0.045443 | -0.56198 | 2560 | |
| Lgals3 | 0.045474 | -1.352 | 2561 | |
| Tmem132d | 0.045491 | -1.0414 | 2562 | |
| Zgpat | 0.045526 | -1.2027 | 2563 | |
| Commd8 | 0.045569 | -2.0107 | 2564 | |
| Myadml2 | 0.04559 | -1.0091 | 2565 | |
| Lce3a | 0.045631 | -2.3366 | 2566 | |
| Mmgt2 | 0.045651 | -0.55991 | 2567 | |
| Dcun1d5 | 0.045672 | -2.3496 | 2568 | |
| Ccdc174 | 0.045706 | -0.99647 | 2569 | |
| mmu-mir-669j | 0.045709 | -0.79425 | 2570 | |
| Diap3 | 0.045741 | -1.2608 | 2571 | |
| mmu-mir-7650 | 0.045751 | -1.4244 | 2572 | |
| Opa1 | 0.045754 | -2.2822 | 2573 | |
| Zfp507 | 0.045759 | -0.99132 | 2574 | |
| Pofut1 | 0.04577 | -1.2977 | 2575 | |
| 5430402E10Rik | 0.045805 | -2.3163 | 2576 | |
| Ing2 | 0.045877 | -2.2391 | 2577 | |
| Gm853 | 0.045887 | -1.1081 | 2578 | |
| Gm16367 | 0.045897 | -2.3369 | 2579 | |
| Leng1 | 0.045938 | -1.1441 | 2580 | |
| Clpsl2 | 0.045959 | -1.7671 | 2581 | |
| Gm5878 | 0.046004 | -1.0388 | 2582 | |
| Ndst4 | 0.046219 | -1.9262 | 2583 | |
| Bysl | 0.046219 | -0.29657 | 2584 | |
| Sp1 | 0.046219 | -1.9258 | 2585 | |
| Acp5 | 0.046219 | -1.1356 | 2586 | |
| Trappc2 | 0.046219 | -0.29293 | 2587 | |
| Thoc7 | 0.046219 | -0.74248 | 2588 | |
| Sfxn4 | 0.046219 | -0.6405 | 2589 | |
| Hes7 | 0.04622 | -1.3264 | 2590 | |
| Pkp2 | 0.046279 | -2.036 | 2591 | |
| Prss3 | 0.046298 | -2.308 | 2592 | |
| Shpk | 0.046452 | -2.3112 | 2593 | |
| Gtf2h3 | 0.046468 | -2.3226 | 2594 | |
| mmu-mir-1195 | 0.046563 | -1.4744 | 2595 | |
| Lipa | 0.046572 | -1.3753 | 2596 | |
| Fbxo28 | 0.046576 | -2.3141 | 2597 | |
| Armc3 | 0.046585 | -1.3448 | 2598 | |
| Psme4 | 0.046591 | -1.0831 | 2599 | |
| Azin1 | 0.046617 | -2.3326 | 2600 | |
| Tnni3 | 0.04662 | -1.0081 | 2601 | |
| Rhox4c | 0.046632 | -1.0539 | 2602 | |
| 1700030J22Rik | 0.046659 | -1.2938 | 2603 | |
| Glmn | 0.046695 | -2.3225 | 2604 | |
| Vmn2r37 | 0.046708 | -1.0579 | 2605 | |
| 4933402D24Rik | 0.046749 | -1.5114 | 2606 | |
| Adrb2 | 0.046783 | -1.4126 | 2607 | |
| Rp9 | 0.046837 | 1.0806 | 2608 | |
| Qpctl | 0.046844 | -1.0668 | 2609 | |
| Dab2ip | 0.046925 | -0.5622 | 2610 | |
| Tert | 0.046927 | -2.1255 | 2611 | |
| Narg2 | 0.046969 | -1.6137 | 2612 | |
| Ppil6 | 0.047011 | -2.3284 | 2613 | |
| Wipf2 | 0.047022 | -0.98976 | 2614 | |
| Mief2 | 0.047035 | -0.99838 | 2615 | |
| AW146154 | 0.047058 | -1.3122 | 2616 | |
| mmu-mir-7218 | 0.047098 | -1.8078 | 2617 | |
| Hint3 | 0.047111 | -2.115 | 2618 | |
| Fibp | 0.047141 | -1.1352 | 2619 | |
| Nup107 | 0.0472 | -1.0586 | 2620 | |
| Olfr810 | 0.047248 | -1.0905 | 2621 | |
| Ctnnd2 | 0.047249 | -1.374 | 2622 | |
| 1700011E24Rik | 0.047373 | -1.8177 | 2623 | |
| Taar6 | 0.047405 | -0.38879 | 2624 | |
| Abca9 | 0.047432 | -0.34321 | 2625 | |
| Aaed1 | 0.047432 | -0.16837 | 2626 | |
| Slc15a4 | 0.047435 | -2.0085 | 2627 | |
| Slc1a4 | 0.047462 | -0.98109 | 2628 | |
| Zfp623 | 0.047488 | -2.0741 | 2629 | |
| Tfec | 0.047504 | -2.0455 | 2630 | |
| Hist1h4d | 0.047552 | -1.2385 | 2631 | |
| Pxdn | 0.047582 | -0.37415 | 2632 | |
| Dapk1 | 0.047593 | -2.3169 | 2633 | |
| Tob1 | 0.047608 | -1.8702 | 2634 | |
| Ska3 | 0.047608 | -0.91957 | 2635 | |
| Ccdc18 | 0.047608 | -0.38193 | 2636 | |
| E330021D16Rik | 0.047645 | -2.3156 | 2637 | |
| Hhipl2 | 0.047671 | -0.96896 | 2638 | |
| Dgkg | 0.047719 | -1.0594 | 2639 | |
| Clmp | 0.047762 | 0.037841 | 2640 | |
| Crlf3 | 0.047895 | -2.2754 | 2641 | |
| Flot1 | 0.047938 | -2.0329 | 2642 | |
| Bckdk | 0.048043 | -1.9414 | 2643 | |
| Actr2 | 0.048049 | -1.3309 | 2644 | |
| Psma2 | 0.048073 | -1.5386 | 2645 | |
| Epb4.1l2 | 0.048094 | -2.2976 | 2646 | |
| mmu-mir-6388 | 0.048119 | -1.7467 | 2647 | |
| Pomgnt2 | 0.048206 | -1.5128 | 2648 | |
| Jtb | 0.048209 | -2.3015 | 2649 | |
| Bpifa5 | 0.04823 | -0.44944 | 2650 | |
| Cog2 | 0.048293 | -0.47331 | 2651 | |
| Mfap1a | 0.048296 | -1.1302 | 2652 | |
| Erc2 | 0.048381 | -1.2131 | 2653 | |
| Isg20 | 0.048399 | -0.95057 | 2654 | |
| Fcer2a | 0.048417 | -1.4024 | 2655 | |
| H2afb2-ps | 0.048438 | -0.96753 | 2656 | |
| Mdn1 | 0.048467 | -1.8807 | 2657 | |
| Usp7 | 0.048467 | -1.546 | 2658 | |
| Lmbr1 | 0.048467 | -0.59443 | 2659 | |
| Psmg2 | 0.048467 | -0.75721 | 2660 | |
| Myo3a | 0.048472 | -1.1985 | 2661 | |
| Pate2 | 0.048478 | -1.1217 | 2662 | |
| Atp6v1g1 | 0.048503 | -2.2556 | 2663 | |
| Abcg8 | 0.048514 | -1.2769 | 2664 | |
| E2f6 | 0.048535 | -1.6513 | 2665 | |
| Lrrc9 | 0.048535 | -1.2112 | 2666 | |
| Ifna1 | 0.048569 | -0.14808 | 2667 | |
| Mms22l | 0.048605 | -1.7268 | 2668 | |
| Skp2 | 0.048629 | -1.6076 | 2669 | |
| Atg12 | 0.048647 | -0.96198 | 2670 | |
| Atp8b5 | 0.048678 | -1.3404 | 2671 | |
| Rpl4 | 0.048729 | -2.2661 | 2672 | |
| Rpl23a | 0.048729 | -2.2661 | 2673 | |
| Olfr727 | 0.048751 | -1.2079 | 2674 | |
| Npy2r | 0.048757 | -1.0817 | 2675 | |
| Pcm1 | 0.048775 | -1.2992 | 2676 | |
| Mx1 | 0.048798 | -1.2117 | 2677 | |
| Glra2 | 0.048805 | -1.3581 | 2678 | |
| Gigyf2 | 0.048819 | -0.61485 | 2679 | |
| Bloc1s1 | 0.048872 | -1.6873 | 2680 | |
| Rexo4 | 0.048897 | -0.96367 | 2681 | |
| Cdk2 | 0.048921 | -1.2684 | 2682 | |
| Rnf222 | 0.048946 | -0.3611 | 2683 | |
| Adat1 | 0.049006 | -1.4023 | 2684 | |
| Olfr1361 | 0.049072 | -2.2915 | 2685 | |
| mmu-mir-1950 | 0.049078 | -0.5556 | 2686 | |
| 4931423N10Rik | 0.049105 | -1.0884 | 2687 | |
| Cul1 | 0.049115 | -2.2582 | 2688 | |
| Tnmd | 0.049171 | 0.77088 | 2689 | |
| Parp14 | 0.049183 | -2.2776 | 2690 | |
| Olfr95 | 0.049183 | -2.2776 | 2691 | |
| mmu-mir-297a-2 | 0.049199 | -2.1896 | 2692 | |
| Pin4 | 0.049215 | -0.65217 | 2693 | |
| Ccdc86 | 0.049257 | -1.4873 | 2694 | |
| Ndufa4 | 0.049263 | -1.5253 | 2695 | |
| Wdr45b | 0.049293 | -1.1469 | 2696 | |
| Olfr290 | 0.049305 | -1.1551 | 2697 | |
| Rraga | 0.049316 | -1.7145 | 2698 | |
| Trappc4 | 0.049348 | -1.1273 | 2699 | |
| Theg | 0.0494 | -2.2797 | 2700 | |
| Itih4 | 0.049411 | -0.27686 | 2701 | |
| Rgag1 | 0.049459 | -0.95761 | 2702 | |
| Hist4h4 | 0.049464 | -2.1366 | 2703 | |
| Olfr380 | 0.049479 | 0.91804 | 2704 | |
| Ccdc85b | 0.049479 | -1.95 | 2705 | |
| Ints1 | 0.049479 | 0.88824 | 2706 | |
| Vmn1r178 | 0.049489 | -2.128 | 2707 | |
| Gga3 | 0.049506 | -2.2543 | 2708 | |
| 1110059M19Rik | 0.049514 | -1.0094 | 2709 | |
| mmu-mir-6372 | 0.04955 | -1.0534 | 2710 | |
| Zfp341 | 0.049569 | -0.94964 | 2711 | |
| Coa4 | 0.04957 | -2.0838 | 2712 | |
| Adnp | 0.049631 | -1.2453 | 2713 | |
| Fcgr2b | 0.049643 | -1.0054 | 2714 | |
| Fyb | 0.049697 | -2.1878 | 2715 | |
| Col23a1 | 0.049719 | -2.2437 | 2716 | |
| Olfr1065 | 0.049719 | -2.2437 | 2717 | |
| Rab23 | 0.049743 | -1.3718 | 2718 | |
| Gab3 | 0.049784 | -0.93742 | 2719 | |
| Tspan6 | 0.049787 | -0.82546 | 2720 | |
| Lrif1 | 0.049815 | -0.94573 | 2721 | |
| Gm6880 | 0.049849 | -1.0415 | 2722 | |
| Tipin | 0.049875 | -0.76248 | 2723 | |
| Srbd1 | 0.049899 | -2.2589 | 2724 | |
| Glyat | 0.04991 | -1.0396 | 2725 | |
| Atad3a | 0.049941 | -2.1525 | 2726 | |
| Mcts1 | 0.049941 | -0.73365 | 2727 | |
| Ddx41 | 0.049976 | -1.1247 | 2728 | |
| Commd3 | 0.050033 | -2.1525 | 2729 | |
| Hgf | 0.050049 | -2.2758 | 2730 | |
| Zfp932 | 0.05005 | -1.8432 | 2731 | |
| mmu-mir-8094 | 0.050057 | -1.1526 | 2732 | |
| Nsfl1c | 0.050087 | -1.4657 | 2733 | |
| 1700011H14Rik | 0.050102 | -2.2693 | 2734 | |
| Gabrr2 | 0.050105 | -0.94161 | 2735 | |
| Gbx1 | 0.050117 | -1.4341 | 2736 | |
| Arhgef2 | 0.050145 | -0.1666 | 2737 | |
| Mcm6 | 0.050155 | -1.9985 | 2738 | |
| mmu-mir-7240 | 0.050203 | -1.6236 | 2739 | |
| Gmppa | 0.050204 | -2.114 | 2740 | |
| Gpr17 | 0.050211 | -2.034 | 2741 | |
| Pcp2 | 0.05026 | -1.9853 | 2742 | |
| Cd37 | 0.050271 | -0.63932 | 2743 | |
| Uchl5 | 0.050283 | -1.713 | 2744 | |
| Cnn3 | 0.050294 | -2.2578 | 2745 | |
| Gm14459 | 0.050315 | 1.9601 | 2746 | |
| Nrros | 0.050328 | -1.2013 | 2747 | |
| Bcl11a | 0.050359 | -1.0426 | 2748 | |
| Chmp1a | 0.05039 | -2.2603 | 2749 | |
| Mfhas1 | 0.050533 | -1.0896 | 2750 | |
| Ckap2 | 0.050567 | -2.2652 | 2751 | |
| Psmd14 | 0.050579 | -0.96768 | 2752 | |
| Eif3h | 0.050579 | -1.6147 | 2753 | |
| Riok2 | 0.050579 | 0.31736 | 2754 | |
| Srpr | 0.050583 | -1.1131 | 2755 | |
| Hdgfrp2 | 0.050655 | -1.0816 | 2756 | |
| Ywhag | 0.050664 | -1.4005 | 2757 | |
| Mrps9 | 0.050679 | -2.2475 | 2758 | |
| Hsd17b3 | 0.050701 | -2.2671 | 2759 | |
| mmu-mir-6546 | 0.05083 | -1.2323 | 2760 | |
| Fank1 | 0.050843 | -1.2887 | 2761 | |
| Scgb1b3 | 0.050926 | -2.1964 | 2762 | |
| Arhgef11 | 0.050931 | -1.3094 | 2763 | |
| Arpc3 | 0.050958 | -1.273 | 2764 | |
| mmu-mir-6336 | 0.050973 | -1.0666 | 2765 | |
| Zfp791 | 0.051008 | -1.14 | 2766 | |
| Bard1 | 0.051012 | -1.8456 | 2767 | |
| Gm10413 | 0.051024 | -2.2402 | 2768 | |
| Tas2r125 | 0.051027 | -1.8182 | 2769 | |
| Aasdhppt | 0.051041 | -1.7687 | 2770 | |
| Krt84 | 0.051055 | -1.016 | 2771 | |
| Hist1h4h | 0.051076 | -2.2613 | 2772 | |
| Eif4a2 | 0.051114 | -2.231 | 2773 | |
| Traf3ip1 | 0.051141 | -2.0335 | 2774 | |
| Chrnb3 | 0.051168 | -0.37334 | 2775 | |
| mmu-mir-30a | 0.051206 | -1.0502 | 2776 | |
| mmu-mir-7668 | 0.051293 | -1.1559 | 2777 | |
| Cdc27 | 0.051302 | -2.1681 | 2778 | |
| Kif11 | 0.051321 | -1.4532 | 2779 | |
| Enthd1 | 0.051337 | -1.6762 | 2780 | |
| Casp14 | 0.051347 | -0.9366 | 2781 | |
| Fpr2 | 0.051348 | 1.8678 | 2782 | |
| Rad52 | 0.051348 | -0.50555 | 2783 | |
| Eefsec | 0.051348 | 0.042972 | 2784 | |
| Pam16 | 0.051348 | -2.176 | 2785 | |
| mmu-mir-8111 | 0.051355 | 0.13358 | 2786 | |
| Defb36 | 0.051378 | -2.2567 | 2787 | |
| Gm14625 | 0.051378 | -1.2508 | 2788 | |
| Slc25a35 | 0.05141 | -2.2571 | 2789 | |
| Ttpa | 0.051472 | -1.2993 | 2790 | |
| Zfp280c | 0.051479 | -1.1351 | 2791 | |
| Them4 | 0.051486 | -2.2543 | 2792 | |
| Cyp3a44 | 0.051611 | -1.3895 | 2793 | |
| Prph | 0.051617 | -0.74638 | 2794 | |
| Pin1 | 0.051626 | -0.87362 | 2795 | |
| Apol8 | 0.051634 | 0.43017 | 2796 | |
| Gsdma | 0.051646 | -1.0741 | 2797 | |
| Camk4 | 0.051687 | -1.9617 | 2798 | |
| Six1 | 0.051699 | -0.99986 | 2799 | |
| Ciita | 0.051706 | -1.0021 | 2800 | |
| Eif3k | 0.051722 | -1.3129 | 2801 | |
| H60b | 0.051722 | -0.23135 | 2802 | |
| Sprn | 0.051737 | -1.0228 | 2803 | |
| Dnajc4 | 0.051751 | -2.2138 | 2804 | |
| 4930480E11Rik | 0.051751 | -1.8223 | 2805 | |
| Crem | 0.051778 | -2.2027 | 2806 | |
| Speer4e | 0.051784 | -1.6727 | 2807 | |
| Sry | 0.051839 | -1.2017 | 2808 | |
| D630045J12Rik | 0.051897 | 0.42261 | 2809 | |
| Olfr798 | 0.051941 | -1.2063 | 2810 | |
| Tmem79 | 0.051965 | -1.8541 | 2811 | |
| Cbln4 | 0.051972 | -0.91611 | 2812 | |
| Usp5 | 0.051972 | -2.2022 | 2813 | |
| Zscan4e | 0.052003 | -3.3701 | 2814 | |
| Sp6 | 0.052054 | -1.2272 | 2815 | |
| Stard3nl | 0.052079 | -1.0237 | 2816 | |
| As3mt | 0.052081 | -0.64945 | 2817 | |
| Sptssb | 0.052095 | -1.7607 | 2818 | |
| Defb40 | 0.052118 | -0.93274 | 2819 | |
| Dennd6a | 0.052127 | -0.93042 | 2820 | |
| Clasrp | 0.052143 | -0.63222 | 2821 | |
| Gm5800 | 0.052168 | -2.2408 | 2822 | |
| Pou5f2 | 0.052189 | -1.4682 | 2823 | |
| Ncf2 | 0.052194 | -0.84671 | 2824 | |
| Espnl | 0.052238 | -0.91427 | 2825 | |
| Msl2 | 0.052245 | -1.7665 | 2826 | |
| Gm14326 | 0.052249 | -0.96149 | 2827 | |
| Ctse | 0.05232 | -0.64028 | 2828 | |
| Obp2b | 0.052342 | -1.8171 | 2829 | |
| Htr3b | 0.052363 | -2.2452 | 2830 | |
| Krt80 | 0.052429 | -2.243 | 2831 | |
| 1700106J16Rik | 0.052458 | -1.1381 | 2832 | |
| Otog | 0.052468 | -0.18624 | 2833 | |
| Dnajc17 | 0.052468 | -0.43262 | 2834 | |
| Irf8 | 0.052468 | -0.90165 | 2835 | |
| Mmp27 | 0.052548 | -0.45263 | 2836 | |
| Anapc15 | 0.05257 | -1.3499 | 2837 | |
| Bptf | 0.052586 | -2.2293 | 2838 | |
| Dnpep | 0.052608 | -1.5085 | 2839 | |
| Atp2b2 | 0.052634 | -0.92134 | 2840 | |
| Atrip | 0.052646 | -2.1959 | 2841 | |
| Pola1 | 0.052691 | -0.92264 | 2842 | |
| Olfr1321 | 0.052707 | -1.2779 | 2843 | |
| Pcsk7 | 0.052732 | -1.0759 | 2844 | |
| Rapgef3 | 0.052742 | -1.3049 | 2845 | |
| Dynlrb2 | 0.052776 | -0.73693 | 2846 | |
| Ptpn6 | 0.052848 | -1.5349 | 2847 | |
| mmu-mir-3060 | 0.052888 | -0.86214 | 2848 | |
| Lmod2 | 0.052941 | -0.86342 | 2849 | |
| Olfr516 | 0.05297 | -1.2686 | 2850 | |
| Plscr5 | 0.052986 | -0.92468 | 2851 | |
| Impa2 | 0.053007 | -2.2353 | 2852 | |
| Ino80e | 0.053018 | -1.0316 | 2853 | |
| Tcl1b3 | 0.05305 | -1.3587 | 2854 | |
| Gimap5 | 0.053095 | -1.5892 | 2855 | |
| Ticrr | 0.053105 | 0.57037 | 2856 | |
| Exosc6 | 0.053105 | -1.6574 | 2857 | |
| Olfr919 | 0.053224 | -0.92357 | 2858 | |
| Gen1 | 0.05323 | -0.91552 | 2859 | |
| Scgb1c1 | 0.053269 | -0.9444 | 2860 | |
| Cox11 | 0.053275 | -1.0932 | 2861 | |
| mmu-mir-6942 | 0.053314 | -1.7543 | 2862 | |
| mmu-mir-7212 | 0.053328 | -1.5979 | 2863 | |
| Ppm1d | 0.053335 | -2.1958 | 2864 | |
| Vmn1r85 | 0.053346 | -1.962 | 2865 | |
| Olfr1200 | 0.053353 | -0.58686 | 2866 | |
| A630095E13Rik | 0.053359 | -0.91497 | 2867 | |
| BC048609 | 0.053368 | -0.36895 | 2868 | |
| Tceanc2 | 0.053368 | -0.14011 | 2869 | |
| mmu-mir-7029 | 0.053398 | -1.0696 | 2870 | |
| Luzp2 | 0.053434 | -2.2254 | 2871 | |
| Mrgpre | 0.05345 | -0.92013 | 2872 | |
| Alg3 | 0.05345 | -1.2424 | 2873 | |
| Sipa1l3 | 0.053482 | -1.2721 | 2874 | |
| Pax3 | 0.0535 | -0.20264 | 2875 | |
| Serpinb9e | 0.0535 | -0.76606 | 2876 | |
| Vmn1r229 | 0.0535 | -0.17635 | 2877 | |
| Calca | 0.053522 | -1.1279 | 2878 | |
| Hdx | 0.053582 | -0.97643 | 2879 | |
| Bean1 | 0.05361 | -0.28898 | 2880 | |
| Hoxc11 | 0.053631 | -1.763 | 2881 | |
| mmu-mir-675 | 0.053645 | -1.4843 | 2882 | |
| Fmo3 | 0.053648 | -2.1997 | 2883 | |
| Kif14 | 0.053653 | -1.6573 | 2884 | |
| Trpc5 | 0.05367 | -0.97855 | 2885 | |
| Dmrt2 | 0.053705 | -0.91137 | 2886 | |
| Pdk1 | 0.053708 | -2.1433 | 2887 | |
| Clta | 0.053741 | -0.10383 | 2888 | |
| Aqp8 | 0.053741 | -2.2208 | 2889 | |
| Gm10696 | 0.053785 | -1.0362 | 2890 | |
| Cinp | 0.053806 | -1.3008 | 2891 | |
| Arhgap10 | 0.053829 | -0.4571 | 2892 | |
| Cnep1r1 | 0.053851 | -2.2122 | 2893 | |
| Tmlhe | 0.053873 | -0.90535 | 2894 | |
| Fam98b | 0.053917 | -1.1787 | 2895 | |
| 1700019A02Rik | 0.053932 | -2.1076 | 2896 | |
| Rhox3h | 0.054007 | -1.535 | 2897 | |
| Defb47 | 0.05402 | -1.0946 | 2898 | |
| Rassf8 | 0.054066 | -0.90366 | 2899 | |
| Zfp551 | 0.054072 | -0.81777 | 2900 | |
| Cbwd1 | 0.054124 | -0.69197 | 2901 | |
| Dpy19l3 | 0.054137 | -1.3768 | 2902 | |
| Hdhd1a | 0.054138 | -0.39005 | 2903 | |
| Txndc15 | 0.054144 | -1.1453 | 2904 | |
| Pip | 0.05415 | -0.97974 | 2905 | |
| mmu-mir-6930 | 0.054152 | -0.8052 | 2906 | |
| Ftsj2 | 0.054189 | -1.3214 | 2907 | |
| Nudcd3 | 0.054202 | 0.23499 | 2908 | |
| Olfr1289 | 0.054202 | -0.12796 | 2909 | |
| Wdr74 | 0.054202 | -0.32499 | 2910 | |
| Dpcd | 0.054202 | -1.2221 | 2911 | |
| 4930467E23Rik | 0.054202 | -1.7751 | 2912 | |
| Gm10375 | 0.054218 | -3.2908 | 2913 | |
| Ghsr | 0.054228 | -1.6179 | 2914 | |
| Speer4b | 0.054237 | -2.1945 | 2915 | |
| Hspa4 | 0.054274 | -0.9116 | 2916 | |
| Hexdc | 0.0543 | -1.6971 | 2917 | |
| Fus | 0.054307 | -0.3731 | 2918 | |
| Rnf183 | 0.054326 | -2.201 | 2919 | |
| Diras2 | 0.054326 | -1.1185 | 2920 | |
| Fgl1 | 0.054437 | -0.95663 | 2921 | |
| Stoml3 | 0.05445 | -1.5676 | 2922 | |
| Prim2 | 0.054531 | -0.046516 | 2923 | |
| Sstr2 | 0.054552 | -2.2108 | 2924 | |
| N6amt1 | 0.054555 | -1.8921 | 2925 | |
| Tcp10b | 0.054572 | -1.6265 | 2926 | |
| Arl6 | 0.054598 | -0.94408 | 2927 | |
| Ngf | 0.054658 | -2.214 | 2928 | |
| Cd209f | 0.054706 | -1.3071 | 2929 | |
| Lipk | 0.054706 | -1.3411 | 2930 | |
| Krtap27-1 | 0.054794 | -1.4675 | 2931 | |
| Agbl2 | 0.054838 | -1.0687 | 2932 | |
| Ankrd49 | 0.054868 | -2.2036 | 2933 | |
| Otoa | 0.054913 | -1.9202 | 2934 | |
| Gm11992 | 0.054935 | -1.5056 | 2935 | |
| Csf2 | 0.054947 | -0.064981 | 2936 | |
| C3ar1 | 0.054949 | -1.1234 | 2937 | |
| Nt5dc1 | 0.054988 | -1.1624 | 2938 | |
| Atp6v0a1 | 0.055013 | -2.1283 | 2939 | |
| Vmn2r75 | 0.055035 | -2.1744 | 2940 | |
| Dph3 | 0.055041 | -1.0845 | 2941 | |
| mmu-mir-9-3 | 0.055054 | -0.20377 | 2942 | |
| Trmt13 | 0.055068 | -1.8853 | 2943 | |
| Arhgef17 | 0.055079 | -0.77348 | 2944 | |
| Scgb2b2 | 0.055174 | -2.2014 | 2945 | |
| Slc22a29 | 0.055213 | -2.1618 | 2946 | |
| Ppfibp1 | 0.055232 | -1.491 | 2947 | |
| Vmn1r72 | 0.055232 | -0.5478 | 2948 | |
| Tnfaip3 | 0.055232 | -0.69039 | 2949 | |
| Dut | 0.055232 | -1.3873 | 2950 | |
| Myo18a | 0.055258 | -2.205 | 2951 | |
| Gca | 0.055272 | -0.89449 | 2952 | |
| Vmn1r121 | 0.055276 | -2.8399 | 2953 | |
| Syngr4 | 0.055291 | -2.2068 | 2954 | |
| Kri1 | 0.055324 | -2.2021 | 2955 | |
| mmu-mir-680-3 | 0.055347 | -1.3839 | 2956 | |
| Mmrn1 | 0.055368 | -1.3502 | 2957 | |
| Olfr320 | 0.055388 | -1.9845 | 2958 | |
| Elf3 | 0.055437 | -1.0286 | 2959 | |
| Ago2 | 0.055458 | -2.2024 | 2960 | |
| Mettl13 | 0.055473 | -0.30959 | 2961 | |
| Zfand6 | 0.055473 | -0.66129 | 2962 | |
| mmu-mir-6926 | 0.055498 | -1.4562 | 2963 | |
| Arl4a | 0.055533 | -1.8088 | 2964 | |
| Nppa | 0.055626 | -0.13241 | 2965 | |
| Trib1 | 0.05567 | -0.88941 | 2966 | |
| Rad21 | 0.055693 | -2.1782 | 2967 | |
| Gm8693 | 0.05575 | -3.5795 | 2968 | |
| Rgag4 | 0.055785 | -1.2591 | 2969 | |
| Srprb | 0.055799 | -2.1659 | 2970 | |
| Slc27a3 | 0.055802 | -0.19575 | 2971 | |
| Zfyve1 | 0.055802 | -1.6196 | 2972 | |
| B3gnt2 | 0.055838 | -2.2014 | 2973 | |
| Cyp2d11 | 0.055849 | -2.1994 | 2974 | |
| mmu-mir-6240 | 0.055868 | -1.3179 | 2975 | |
| Tmf1 | 0.055883 | -2.1394 | 2976 | |
| mmu-mir-128-1 | 0.055942 | -0.42549 | 2977 | |
| Blm | 0.05595 | -1.4821 | 2978 | |
| Mx2 | 0.056017 | -1.6977 | 2979 | |
| Hist2h2ac | 0.056052 | -1.6992 | 2980 | |
| Car2 | 0.056084 | -2.1821 | 2981 | |
| Olfr1182 | 0.056086 | -1.0635 | 2982 | |
| Pik3ca | 0.056086 | -1.4063 | 2983 | |
| Zfyve20 | 0.056086 | -0.92615 | 2984 | |
| Slco6c1 | 0.056086 | -0.36929 | 2985 | |
| Mat1a | 0.056086 | 0.19251 | 2986 | |
| Ift74 | 0.056128 | -1.0763 | 2987 | |
| Htr2a | 0.056129 | -2.1934 | 2988 | |
| Mob1b | 0.056151 | -2.1971 | 2989 | |
| 1700007K13Rik | 0.056162 | -0.68224 | 2990 | |
| Vmn1r64 | 0.056174 | -0.18848 | 2991 | |
| Gpn2 | 0.056236 | -2.1872 | 2992 | |
| Catsper2 | 0.05624 | 0.45059 | 2993 | |
| Alg14 | 0.056309 | -0.70542 | 2994 | |
| mmu-mir-5125 | 0.056315 | -0.53688 | 2995 | |
| Cyp2j6 | 0.056331 | -0.75781 | 2996 | |
| Dmrtc2 | 0.056355 | -1.1705 | 2997 | |
| Rapgef2 | 0.056382 | -1.1582 | 2998 | |
| Plk1s1 | 0.056421 | -0.42882 | 2999 | |
| 4933413G19Rik | 0.056456 | -1.1382 | 3000 | |
| Zfp335 | 0.056469 | -1.0185 | 3001 | |
| Olfr915 | 0.056488 | -2.1909 | 3002 | |
| Rnmt | 0.056489 | -1.6423 | 3003 | |
| mmu-mir-3086 | 0.056505 | -1.4568 | 3004 | |
| 2610028H24Rik | 0.056522 | -2.1925 | 3005 | |
| Dhx33 | 0.056524 | 0.47352 | 3006 | |
| Snap23 | 0.056524 | -1.5282 | 3007 | |
| Fam3a | 0.056524 | 1.1762 | 3008 | |
| Polr2c | 0.056524 | -1.1544 | 3009 | |
| mmu-mir-6901 | 0.056536 | -1.6873 | 3010 | |
| Cmtm8 | 0.05661 | -1.0159 | 3011 | |
| Lhcgr | 0.05668 | -2.1801 | 3012 | |
| Bpifc | 0.056691 | -2.1701 | 3013 | |
| Dsn1 | 0.056758 | -0.27594 | 3014 | |
| BC030307 | 0.05677 | -2.1605 | 3015 | |
| Sphk1 | 0.056788 | -0.97842 | 3016 | |
| Ostc | 0.056792 | -0.60964 | 3017 | |
| Trip4 | 0.056809 | -0.70545 | 3018 | |
| Psmc1 | 0.056823 | -0.56198 | 3019 | |
| Adam28 | 0.056894 | -0.36192 | 3020 | |
| Olfr1166 | 0.056896 | -1.8576 | 3021 | |
| Barhl2 | 0.056896 | -0.068953 | 3022 | |
| Scgb2b20 | 0.056939 | -1.6183 | 3023 | |
| Pou2f1 | 0.05695 | -2.1855 | 3024 | |
| Krtcap3 | 0.056987 | -1.3412 | 3025 | |
| Trmt10c | 0.057006 | -1.1719 | 3026 | |
| Ehbp1 | 0.057063 | -2.1823 | 3027 | |
| 2410002F23Rik | 0.057071 | -1.3056 | 3028 | |
| Golga2 | 0.057088 | -0.88643 | 3029 | |
| Orai1 | 0.057095 | -0.35477 | 3030 | |
| Ap3b1 | 0.057137 | 0.24489 | 3031 | |
| Olfr399 | 0.057142 | -0.88807 | 3032 | |
| Eid1 | 0.057153 | -1.5959 | 3033 | |
| Etv5 | 0.057257 | -0.90417 | 3034 | |
| Farsb | 0.05727 | -1.0148 | 3035 | |
| Bivm | 0.0573 | -0.62093 | 3036 | |
| Gm5622 | 0.057334 | 0.21277 | 3037 | |
| Gapdh | 0.057334 | -0.88901 | 3038 | |
| Hist2h3b | 0.057334 | 0.45246 | 3039 | |
| Plekhg1 | 0.057345 | -0.87285 | 3040 | |
| Stx12 | 0.057351 | -0.88177 | 3041 | |
| Gtf2e1 | 0.057357 | -2.1223 | 3042 | |
| Tiprl | 0.057402 | -2.1782 | 3043 | |
| Wdr64 | 0.057453 | -1.0019 | 3044 | |
| Chrnb1 | 0.057473 | -1.9409 | 3045 | |
| Rasa2 | 0.057487 | -0.92491 | 3046 | |
| Nop9 | 0.057509 | -1.165 | 3047 | |
| Sla2 | 0.057515 | -1.3309 | 3048 | |
| Wdr95 | 0.057538 | -0.92241 | 3049 | |
| Tspan8 | 0.057553 | -0.28808 | 3050 | |
| Shf | 0.057597 | 1.235 | 3051 | |
| Ubxn1 | 0.057618 | -1.3627 | 3052 | |
| Dpm3 | 0.057629 | -2.1771 | 3053 | |
| Rab27b | 0.05764 | -1.0834 | 3054 | |
| 1300002K09Rik | 0.05764 | -0.35945 | 3055 | |
| Vmn1r53 | 0.057684 | 0.15907 | 3056 | |
| mmu-mir-6380 | 0.057755 | -1.2212 | 3057 | |
| mmu-mir-7083 | 0.057755 | -0.73331 | 3058 | |
| Hoxb7 | 0.057772 | -1.4899 | 3059 | |
| Eci1 | 0.057793 | -1.2866 | 3060 | |
| A630076J17Rik | 0.057799 | -1.1616 | 3061 | |
| Vmn2r91 | 0.057833 | -1.0689 | 3062 | |
| Tbck | 0.057879 | -2.0289 | 3063 | |
| Tnpo2 | 0.057903 | 0.19577 | 3064 | |
| Klf3 | 0.057942 | -1.04 | 3065 | |
| mmu-mir-410 | 0.057967 | -1.5102 | 3066 | |
| Spcs1 | 0.057968 | -1.2395 | 3067 | |
| Daam1 | 0.057968 | -1.2223 | 3068 | |
| Eif2b4 | 0.057981 | -1.321 | 3069 | |
| Gnai2 | 0.058004 | -0.99476 | 3070 | |
| Lrfn5 | 0.058017 | -0.87695 | 3071 | |
| Vmn1r230 | 0.058031 | -1.0657 | 3072 | |
| Rad54l2 | 0.058034 | 0.97617 | 3073 | |
| Mrpl22 | 0.058048 | -0.95705 | 3074 | |
| Pole3 | 0.058117 | -2.1705 | 3075 | |
| Tjp2 | 0.058127 | -0.82628 | 3076 | |
| Olfr905 | 0.058163 | -1.3573 | 3077 | |
| Zeb2 | 0.058181 | -1.6164 | 3078 | |
| mmu-mir-137 | 0.05821 | -1.3504 | 3079 | |
| Ddb1 | 0.05822 | -2.1703 | 3080 | |
| Cd99l2 | 0.05834 | 0.45492 | 3081 | |
| Nanos3 | 0.05834 | -1.4836 | 3082 | |
| Chchd2 | 0.05834 | -1.2331 | 3083 | |
| Efnb3 | 0.058435 | -1.1957 | 3084 | |
| mmu-mir-6339 | 0.058492 | -0.57235 | 3085 | |
| Zfp53 | 0.058551 | -1.1827 | 3086 | |
| 1700023E05Rik | 0.058559 | -0.659 | 3087 | |
| Gk2 | 0.058619 | -0.99446 | 3088 | |
| Sdpr | 0.058627 | -0.85682 | 3089 | |
| Stau2 | 0.05864 | -1.389 | 3090 | |
| Atp12a | 0.058646 | -0.86331 | 3091 | |
| Bex2 | 0.058709 | -0.87098 | 3092 | |
| Cacna2d4 | 0.058757 | -0.87907 | 3093 | |
| Usp13 | 0.058777 | -0.16671 | 3094 | |
| Hcfc1 | 0.058797 | -1.9191 | 3095 | |
| Btbd9 | 0.058825 | -0.3245 | 3096 | |
| Vmn1r183 | 0.058865 | -0.19798 | 3097 | |
| Serping1 | 0.058888 | -0.85622 | 3098 | |
| Smim8 | 0.058905 | -2.157 | 3099 | |
| mmu-mir-1839 | 0.058917 | -1.4797 | 3100 | |
| Mroh8 | 0.058929 | -1.7953 | 3101 | |
| Serp2 | 0.05895 | -1.1825 | 3102 | |
| Tox | 0.058952 | -1.2187 | 3103 | |
| Pla2g5 | 0.05904 | -2.1452 | 3104 | |
| 5031439G07Rik | 0.05906 | -1.4272 | 3105 | |
| Psg29 | 0.059215 | -2.125 | 3106 | |
| Gm17365 | 0.059223 | -0.87477 | 3107 | |
| Ogt | 0.059258 | -0.92105 | 3108 | |
| mmu-mir-3569 | 0.059291 | -1.4241 | 3109 | |
| Sar1a | 0.059295 | -2.0494 | 3110 | |
| mmu-mir-326 | 0.059298 | -0.85734 | 3111 | |
| Akr1b3 | 0.059302 | -0.85904 | 3112 | |
| Ddah1 | 0.059307 | -2.149 | 3113 | |
| Pcdhb18 | 0.059344 | -0.96546 | 3114 | |
| Gm15023 | 0.059399 | -3.1178 | 3115 | |
| Mnat1 | 0.059477 | -1.4085 | 3116 | |
| Snx18 | 0.059548 | -1.0498 | 3117 | |
| Ptx4 | 0.059601 | -1.5049 | 3118 | |
| Lypla2 | 0.059695 | -0.68635 | 3119 | |
| Ifna4 | 0.059709 | -2.1496 | 3120 | |
| Ace3 | 0.059719 | -1.1326 | 3121 | |
| Sardh | 0.059733 | -1.5117 | 3122 | |
| Olfr1340 | 0.059767 | -2.1381 | 3123 | |
| Epb4.1l1 | 0.059767 | -1.0126 | 3124 | |
| Olfr198 | 0.059767 | -1.3013 | 3125 | |
| Samt2 | 0.059768 | -2.6488 | 3126 | |
| Caap1 | 0.059788 | -1.3871 | 3127 | |
| Dalrd3 | 0.059813 | -2.1489 | 3128 | |
| Rtkn2 | 0.059826 | -1.0972 | 3129 | |
| mmu-mir-351 | 0.059834 | -1.5186 | 3130 | |
| Polr3d | 0.059836 | -2.1509 | 3131 | |
| Ilf3 | 0.060001 | -1.0197 | 3132 | |
| Rfc3 | 0.060004 | -1.733 | 3133 | |
| Klrb1f | 0.060066 | -0.8658 | 3134 | |
| Mrpl32 | 0.060109 | -1.2355 | 3135 | |
| Snx13 | 0.060132 | 0.40975 | 3136 | |
| Pigh | 0.060136 | -1.4943 | 3137 | |
| Exoc5 | 0.060197 | -0.28619 | 3138 | |
| Minpp1 | 0.060263 | -0.80252 | 3139 | |
| Ccdc24 | 0.060298 | -1.112 | 3140 | |
| Shroom1 | 0.060319 | -0.95846 | 3141 | |
| Abhd17b | 0.060326 | -1.2046 | 3142 | |
| Irgm2 | 0.060347 | -0.99588 | 3143 | |
| Cyb5 | 0.06035 | -0.7767 | 3144 | |
| Olfr155 | 0.060389 | -1.3032 | 3145 | |
| Atp10a | 0.060402 | -2.1455 | 3146 | |
| Ccdc112 | 0.060459 | -0.13651 | 3147 | |
| Zfp27 | 0.060459 | 0.29528 | 3148 | |
| Fut9 | 0.060483 | -2.1448 | 3149 | |
| Myo18b | 0.060536 | -0.97205 | 3150 | |
| Tceal6 | 0.060611 | -2.1365 | 3151 | |
| Vmn1r49 | 0.060669 | -0.30746 | 3152 | |
| Defb37 | 0.06068 | -2.1411 | 3153 | |
| Gzmc | 0.060726 | -1.4228 | 3154 | |
| AI182371 | 0.060775 | -2.0118 | 3155 | |
| Nol10 | 0.060787 | -1.5149 | 3156 | |
| Fcf1 | 0.060787 | -0.15972 | 3157 | |
| Trim54 | 0.060787 | 0.01909 | 3158 | |
| Olfr186 | 0.060787 | 1.5302 | 3159 | |
| Drosha | 0.060854 | -2.1395 | 3160 | |
| Mrgpra9 | 0.060901 | -2.1269 | 3161 | |
| Usp27x | 0.060923 | -1.1396 | 3162 | |
| Fgfr2 | 0.060924 | -2.1333 | 3163 | |
| Olfr728 | 0.060972 | -1.624 | 3164 | |
| mmu-mir-362 | 0.061029 | -1.5118 | 3165 | |
| Chl1 | 0.061078 | -1.3946 | 3166 | |
| Vmn1r30 | 0.061092 | -0.35444 | 3167 | |
| Axin2 | 0.061127 | -0.83249 | 3168 | |
| Stim1 | 0.061134 | -0.87284 | 3169 | |
| Calml3 | 0.061149 | -1.0657 | 3170 | |
| Muc15 | 0.061156 | -0.85149 | 3171 | |
| Gm10142 | 0.061163 | -1.0837 | 3172 | |
| Slco2a1 | 0.061168 | -2.1336 | 3173 | |
| Snrnp70 | 0.06118 | -0.5889 | 3174 | |
| Arl8a | 0.06118 | -1.1581 | 3175 | |
| Il1f9 | 0.061209 | -0.93342 | 3176 | |
| Ndufv1 | 0.061262 | -1.9151 | 3177 | |
| Mia | 0.061304 | -1.3506 | 3178 | |
| Cyp2c55 | 0.061311 | -1.4148 | 3179 | |
| Krt25 | 0.061368 | -1.908 | 3180 | |
| Brd8 | 0.061396 | -1.0647 | 3181 | |
| 4931431F19Rik | 0.061432 | -1.5154 | 3182 | |
| Stard13 | 0.061442 | -0.63916 | 3183 | |
| Ugp2 | 0.061485 | -1.7641 | 3184 | |
| Vmn1r34 | 0.061552 | -1.1266 | 3185 | |
| Rpl37 | 0.061577 | -1.9481 | 3186 | |
| Sly | 0.061606 | -2.5757 | 3187 | |
| Mzt2 | 0.061616 | -1.0805 | 3188 | |
| Olfr325 | 0.061616 | -0.77414 | 3189 | |
| Nppb | 0.06173 | -1.0793 | 3190 | |
| Dlk2 | 0.061751 | -1.3383 | 3191 | |
| Cenpb | 0.061758 | -0.98283 | 3192 | |
| 5430427O19Rik | 0.061808 | -1.1578 | 3193 | |
| Psg17 | 0.061865 | -0.77159 | 3194 | |
| Omd | 0.061893 | -1.6786 | 3195 | |
| Ppox | 0.061898 | -2.108 | 3196 | |
| Nbn | 0.061921 | 1.1887 | 3197 | |
| Ifi27l2a | 0.061936 | -0.8421 | 3198 | |
| Txndc16 | 0.061965 | -1.3399 | 3199 | |
| mmu-mir-8099-2 | 0.061971 | -0.75398 | 3200 | |
| mmu-mir-1193 | 0.06199 | -1.5312 | 3201 | |
| Sh3tc1 | 0.062033 | -2.1243 | 3202 | |
| Trim59 | 0.062044 | -1.9069 | 3203 | |
| Ugt2b34 | 0.062072 | -1.6953 | 3204 | |
| mmu-mir-6990 | 0.062082 | -1.4084 | 3205 | |
| Pddc1 | 0.062091 | -1.5793 | 3206 | |
| Syn2 | 0.062136 | -0.77695 | 3207 | |
| Sco2 | 0.062161 | -1.7274 | 3208 | |
| Gm12185 | 0.062161 | 0.8474 | 3209 | |
| Dnmt1 | 0.062227 | -0.55624 | 3210 | |
| Ttc14 | 0.062255 | -2.1226 | 3211 | |
| Ybey | 0.062267 | -2.1157 | 3212 | |
| Klhl14 | 0.062286 | -1.3641 | 3213 | |
| Galnt13 | 0.062314 | 0.71213 | 3214 | |
| Mpp1 | 0.062336 | -1.1855 | 3215 | |
| Zfp521 | 0.062384 | -1.3991 | 3216 | |
| Olfr891 | 0.062408 | -1.315 | 3217 | |
| Stat2 | 0.062423 | 0.20848 | 3218 | |
| Skint11 | 0.062423 | -0.7585 | 3219 | |
| Lrrc51 | 0.062423 | -0.48646 | 3220 | |
| Vmn1r188 | 0.062465 | -1.0865 | 3221 | |
| Hnf1b | 0.06249 | -1.3883 | 3222 | |
| Nacc1 | 0.062501 | -1.5526 | 3223 | |
| mmu-mir-6373 | 0.062535 | -0.96971 | 3224 | |
| Vstm2l | 0.062544 | -0.8245 | 3225 | |
| Ptpn5 | 0.062554 | -0.65991 | 3226 | |
| Spata24 | 0.06258 | -1.0071 | 3227 | |
| Sh2b2 | 0.062619 | -0.99314 | 3228 | |
| Tmem115 | 0.062619 | -2.118 | 3229 | |
| Csrp2 | 0.062714 | -2.1073 | 3230 | |
| Commd4 | 0.062799 | -1.5279 | 3231 | |
| Olfr180 | 0.06286 | -1.7631 | 3232 | |
| Gyk | 0.062867 | -1.0623 | 3233 | |
| Spice1 | 0.062926 | -0.26044 | 3234 | |
| mmu-mir-7657 | 0.062978 | -0.79801 | 3235 | |
| Ccdc58 | 0.062996 | -2.1092 | 3236 | |
| Tmem251 | 0.063033 | -1.1829 | 3237 | |
| Hist1h4a | 0.063033 | 0.63529 | 3238 | |
| Olfr394 | 0.063033 | -0.43405 | 3239 | |
| Rpl13 | 0.063033 | -1.2803 | 3240 | |
| Mtmr11 | 0.063033 | -1.9659 | 3241 | |
| Wdr7 | 0.063033 | -0.74774 | 3242 | |
| 1700019L03Rik | 0.063055 | -2.1128 | 3243 | |
| Olfr118 | 0.063144 | -2.1076 | 3244 | |
| Reep3 | 0.063206 | -1.0751 | 3245 | |
| Myl6 | 0.06322 | -0.83102 | 3246 | |
| mmu-mir-7094-2 | 0.063256 | -1.7882 | 3247 | |
| Mug2 | 0.0633 | -1.2567 | 3248 | |
| Prrg1 | 0.063356 | -2.0199 | 3249 | |
| Znf512b | 0.063394 | -0.91253 | 3250 | |
| Art2b | 0.063423 | -1.9909 | 3251 | |
| Cxcr1 | 0.063473 | -0.72036 | 3252 | |
| Fbxo45 | 0.063528 | -1.4953 | 3253 | |
| Plekha4 | 0.063563 | -2.1063 | 3254 | |
| Rps16 | 0.063623 | -2.1157 | 3255 | |
| Prep | 0.06364 | -1.6676 | 3256 | |
| Cpa5 | 0.063709 | -0.66446 | 3257 | |
| Gm5941 | 0.063796 | -1.7998 | 3258 | |
| AA986860 | 0.0638 | -0.8773 | 3259 | |
| Reep4 | 0.0638 | -2.0936 | 3260 | |
| H3f3b | 0.063836 | -2.1024 | 3261 | |
| Fam111a | 0.063839 | -1.4562 | 3262 | |
| Cttnbp2nl | 0.063848 | -2.1036 | 3263 | |
| Tmprss6 | 0.06386 | -2.0922 | 3264 | |
| Niacr1 | 0.063883 | -1.8883 | 3265 | |
| Fkbp2 | 0.063895 | -2.076 | 3266 | |
| Mrgbp | 0.063931 | -1.8901 | 3267 | |
| Stxbp3a | 0.063982 | -1.4209 | 3268 | |
| Sumo1 | 0.06399 | -2.0893 | 3269 | |
| Zfp69 | 0.064004 | -1.4915 | 3270 | |
| Hrnr | 0.064025 | -0.82905 | 3271 | |
| 1190005I06Rik | 0.064035 | -1.5422 | 3272 | |
| Hgs | 0.064047 | -0.9679 | 3273 | |
| Prtn3 | 0.064055 | -1.6266 | 3274 | |
| Alx3 | 0.064115 | -2.0795 | 3275 | |
| Ttc26 | 0.06415 | -2.0999 | 3276 | |
| Mrpl49 | 0.06415 | -2.0999 | 3277 | |
| Zfp808 | 0.064209 | -1.4641 | 3278 | |
| Nsf | 0.064209 | -0.897 | 3279 | |
| Casc4 | 0.064209 | -0.67614 | 3280 | |
| Pno1 | 0.064216 | -2.0652 | 3281 | |
| Dact3 | 0.064287 | -1.2099 | 3282 | |
| Ier3 | 0.064347 | -2.0955 | 3283 | |
| Wdr18 | 0.064382 | -1.8335 | 3284 | |
| Zbtb38 | 0.06439 | -1.4507 | 3285 | |
| Obox3 | 0.064442 | -0.8232 | 3286 | |
| Vmn2r87 | 0.064442 | -2.0983 | 3287 | |
| Slc4a9 | 0.064466 | -0.78876 | 3288 | |
| Tbc1d30 | 0.064478 | -0.66171 | 3289 | |
| Plekhd1 | 0.064513 | -2.0429 | 3290 | |
| Fgfr4 | 0.064537 | -0.81891 | 3291 | |
| Mbtps2 | 0.064573 | -1.7068 | 3292 | |
| Slc2a10 | 0.064621 | -2.0908 | 3293 | |
| Gskip | 0.064645 | 0.016706 | 3294 | |
| Zcwpw1 | 0.064645 | -1.4242 | 3295 | |
| Ncr1 | 0.064645 | -0.51891 | 3296 | |
| Hus1 | 0.064645 | 0.032297 | 3297 | |
| Msl3 | 0.064645 | 0.37599 | 3298 | |
| Pmvk | 0.064683 | -1.298 | 3299 | |
| Nt5dc3 | 0.064716 | -2.0919 | 3300 | |
| Zfc3h1 | 0.06472 | -1.8838 | 3301 | |
| Camk2n1 | 0.064727 | -1.4261 | 3302 | |
| Fam109b | 0.064728 | -2.0949 | 3303 | |
| Syna | 0.064764 | -1.1126 | 3304 | |
| Asns | 0.064771 | -1.1485 | 3305 | |
| Rae1 | 0.0648 | -2.089 | 3306 | |
| mmu-mir-6353 | 0.064812 | -1.8226 | 3307 | |
| 9130011E15Rik | 0.064837 | -0.92363 | 3308 | |
| Tcl1b5 | 0.064907 | -0.60457 | 3309 | |
| Camk2n2 | 0.064926 | -0.49857 | 3310 | |
| Nupr1l | 0.064955 | -0.82894 | 3311 | |
| Fam198b | 0.06497 | -1.3097 | 3312 | |
| Ddx59 | 0.065047 | -0.82325 | 3313 | |
| BC049635 | 0.065065 | -1.172 | 3314 | |
| Olfr667 | 0.06511 | -2.0892 | 3315 | |
| Olfr1212 | 0.065124 | -1.334 | 3316 | |
| Vmn2r96 | 0.065145 | -0.37086 | 3317 | |
| Zfp65 | 0.065189 | -0.95109 | 3318 | |
| Cgref1 | 0.065231 | -0.90955 | 3319 | |
| Sntb1 | 0.065232 | -0.27798 | 3320 | |
| C1qtnf5 | 0.065242 | -0.95644 | 3321 | |
| Gm2a | 0.065254 | -1.9997 | 3322 | |
| Fbxo16 | 0.065266 | -1.6456 | 3323 | |
| Hexb | 0.065279 | -1.0701 | 3324 | |
| Gm8439 | 0.065287 | -1.0432 | 3325 | |
| Ptp4a3 | 0.06529 | -2.0876 | 3326 | |
| Tmem123 | 0.065298 | -0.95666 | 3327 | |
| Zfp606 | 0.065309 | -0.40242 | 3328 | |
| Zfp580 | 0.065319 | -2.0359 | 3329 | |
| Ptf1a | 0.065331 | -0.38335 | 3330 | |
| Kctd10 | 0.065368 | -1.1339 | 3331 | |
| Gemin8 | 0.065406 | -1.1641 | 3332 | |
| Tmem107 | 0.065446 | -2.0687 | 3333 | |
| Prr12 | 0.065449 | -0.42775 | 3334 | |
| Tnfrsf14 | 0.065457 | -1.2268 | 3335 | |
| Aass | 0.065458 | -0.99776 | 3336 | |
| Gas6 | 0.065482 | -0.85283 | 3337 | |
| Pdk2 | 0.065494 | -2.0867 | 3338 | |
| Nup160 | 0.06553 | -1.1762 | 3339 | |
| Podxl2 | 0.065537 | -1.0571 | 3340 | |
| Lce1l | 0.065548 | -2.083 | 3341 | |
| Mvd | 0.065602 | -2.0857 | 3342 | |
| 4933428G20Rik | 0.065612 | -1.0772 | 3343 | |
| Tek | 0.06565 | -2.0619 | 3344 | |
| 4932416K20Rik | 0.065733 | 0.20133 | 3345 | |
| Zfp931 | 0.065733 | -0.16323 | 3346 | |
| Dmrtb1 | 0.065782 | -1.9704 | 3347 | |
| Vmn2r116 | 0.06579 | -0.80366 | 3348 | |
| Snrpd3 | 0.065794 | -1.9998 | 3349 | |
| Ddx42 | 0.065879 | -1.3885 | 3350 | |
| Tmem71 | 0.065885 | -1.1255 | 3351 | |
| Arhgap15 | 0.06589 | -2.0288 | 3352 | |
| Rdh1 | 0.065894 | -0.5245 | 3353 | |
| Ap2m1 | 0.065902 | -2.075 | 3354 | |
| Prr14l | 0.065916 | -1.5209 | 3355 | |
| Fitm2 | 0.065926 | -0.92316 | 3356 | |
| Olfr13 | 0.065928 | 0.27058 | 3357 | |
| Ilf2 | 0.065946 | -1.8153 | 3358 | |
| Gtl3 | 0.065962 | -2.0792 | 3359 | |
| Pak4 | 0.065975 | -1.2166 | 3360 | |
| Per1 | 0.066013 | -1.1858 | 3361 | |
| Exoc7 | 0.066015 | -0.61038 | 3362 | |
| Vpreb3 | 0.066015 | -1.7253 | 3363 | |
| Gm5113 | 0.06602 | -0.81398 | 3364 | |
| Elf5 | 0.066035 | -1.8155 | 3365 | |
| Coq2 | 0.06605 | -0.88344 | 3366 | |
| Mrps7 | 0.066057 | -1.133 | 3367 | |
| Sdcbp2 | 0.066065 | -1.0415 | 3368 | |
| Gprc5d | 0.066072 | -0.82971 | 3369 | |
| Rprd2 | 0.066276 | 0.16929 | 3370 | |
| Heatr3 | 0.066276 | -1.0069 | 3371 | |
| Fhad1 | 0.066276 | -1.9148 | 3372 | |
| mmu-mir-7649 | 0.066335 | -2.5189 | 3373 | |
| Rer1 | 0.066408 | -2.0592 | 3374 | |
| Fyco1 | 0.066422 | -1.2747 | 3375 | |
| Sumf1 | 0.066432 | -1.0734 | 3376 | |
| Tcn2 | 0.066437 | -0.90192 | 3377 | |
| Slc10a3 | 0.066468 | -0.7727 | 3378 | |
| mmu-mir-6971 | 0.066473 | -0.71199 | 3379 | |
| Madcam1 | 0.066504 | -2.0757 | 3380 | |
| 1110008F13Rik | 0.066512 | -0.81402 | 3381 | |
| Esp4 | 0.066516 | -1.3961 | 3382 | |
| Ect2l | 0.066528 | -2.0746 | 3383 | |
| 6430531B16Rik | 0.066538 | -1.1812 | 3384 | |
| Lsm1 | 0.066538 | -0.80027 | 3385 | |
| Tec | 0.066581 | -1.1409 | 3386 | |
| mmu-mir-153 | 0.066617 | -1.2902 | 3387 | |
| Rapgef4 | 0.066646 | -0.56397 | 3388 | |
| Aldob | 0.066646 | -1.1675 | 3389 | |
| Anapc4 | 0.066691 | -1.6953 | 3390 | |
| Fcrl6 | 0.066744 | -1.6036 | 3391 | |
| Olfr531 | 0.066776 | 0.15905 | 3392 | |
| Polr2m | 0.066776 | -0.1749 | 3393 | |
| Nlrp2 | 0.066794 | -2.0029 | 3394 | |
| Vamp7 | 0.066806 | -2.0645 | 3395 | |
| Vps26a | 0.066855 | -0.89954 | 3396 | |
| Lsm5 | 0.066864 | -1.3342 | 3397 | |
| Ropn1l | 0.066885 | -0.97506 | 3398 | |
| Gkn2 | 0.066961 | -1.0393 | 3399 | |
| Tom1l2 | 0.066976 | -1.3675 | 3400 | |
| Zc3hav1 | 0.066988 | -2.0702 | 3401 | |
| Gbp2 | 0.066994 | -1.6113 | 3402 | |
| Tmco1 | 0.067044 | -0.80003 | 3403 | |
| Clic3 | 0.067059 | -0.56144 | 3404 | |
| Snupn | 0.067059 | -1.6867 | 3405 | |
| Ccni | 0.06717 | -2.071 | 3406 | |
| Slc35g1 | 0.067206 | -2.0707 | 3407 | |
| Bsn | 0.067242 | -2.0699 | 3408 | |
| Cx3cr1 | 0.067288 | -0.9131 | 3409 | |
| Haus8 | 0.067288 | -0.8448 | 3410 | |
| Prpf3 | 0.067385 | -1.7099 | 3411 | |
| Ncapg2 | 0.067385 | -1.188 | 3412 | |
| Rrad | 0.06745 | -0.79863 | 3413 | |
| Selplg | 0.067473 | -0.61734 | 3414 | |
| 1700112E06Rik | 0.067488 | -1.5115 | 3415 | |
| Rnaseh2a | 0.067511 | -1.5302 | 3416 | |
| Cbfa2t2 | 0.067537 | -1.8135 | 3417 | |
| Epg5 | 0.067545 | -0.98582 | 3418 | |
| Khdc1a | 0.067639 | -0.99975 | 3419 | |
| Cyp2r1 | 0.067655 | -2.0564 | 3420 | |
| Cib4 | 0.067669 | -1.6145 | 3421 | |
| Krtap1-4 | 0.067783 | -0.55307 | 3422 | |
| Polr3b | 0.067797 | -1.4249 | 3423 | |
| Zfp354c | 0.067797 | -1.0678 | 3424 | |
| Urb2 | 0.067797 | -0.96666 | 3425 | |
| Lgi1 | 0.067797 | -1.7104 | 3426 | |
| Tars | 0.067813 | -1.272 | 3427 | |
| mmu-mir-369 | 0.067872 | -0.89961 | 3428 | |
| Mcam | 0.067911 | -2.0571 | 3429 | |
| Alpi | 0.06793 | -1.442 | 3430 | |
| Gm9047 | 0.068058 | -0.62433 | 3431 | |
| Kynu | 0.068078 | -0.96283 | 3432 | |
| Rps6ka4 | 0.068082 | -2.0626 | 3433 | |
| Iscu | 0.068101 | 0.60134 | 3434 | |
| Slc36a4 | 0.068106 | -1.9607 | 3435 | |
| Scn1b | 0.068109 | -0.94124 | 3436 | |
| Cnot3 | 0.068118 | -0.366 | 3437 | |
| Ppp6r2 | 0.068136 | -2.0622 | 3438 | |
| Cyp26c1 | 0.0682 | -0.78256 | 3439 | |
| mmu-mir-8093 | 0.06822 | -1.4306 | 3440 | |
| Sys1 | 0.068253 | -1.2293 | 3441 | |
| Hist1h2bn | 0.068253 | -0.6023 | 3442 | |
| Evi5l | 0.068265 | -1.6686 | 3443 | |
| Hes2 | 0.068299 | -1.7586 | 3444 | |
| Ogn | 0.068319 | 0.51769 | 3445 | |
| Itpripl1 | 0.068326 | -2.0501 | 3446 | |
| Rslcan18 | 0.068382 | -1.0619 | 3447 | |
| Hn1 | 0.068405 | 1.1325 | 3448 | |
| Shh | 0.068413 | -1.2806 | 3449 | |
| Anxa1 | 0.068436 | -2.0558 | 3450 | |
| Trpm7 | 0.068492 | -1.5471 | 3451 | |
| Celf2 | 0.068579 | -0.077053 | 3452 | |
| Ptger3 | 0.068618 | -0.78124 | 3453 | |
| Rhox4f | 0.068621 | -2.9297 | 3454 | |
| Grik2 | 0.068631 | -0.38939 | 3455 | |
| Pgap2 | 0.068657 | -0.82799 | 3456 | |
| Olfr1507 | 0.068662 | -2.0563 | 3457 | |
| Lsm4 | 0.068718 | -1.453 | 3458 | |
| Gm13023 | 0.068767 | -0.78904 | 3459 | |
| Lingo2 | 0.068794 | -0.74741 | 3460 | |
| Taf1 | 0.06884 | -1.4664 | 3461 | |
| 2210407C18Rik | 0.06884 | -1.4437 | 3462 | |
| mmu-mir-463 | 0.06885 | 2.6185 | 3463 | |
| mmu-mir-5134 | 0.06889 | -1.0233 | 3464 | |
| Tmtc3 | 0.068913 | -0.76484 | 3465 | |
| Hdac4 | 0.068993 | -0.77339 | 3466 | |
| Olfr1220 | 0.069024 | -1.6776 | 3467 | |
| Coro2a | 0.069036 | -2.0483 | 3468 | |
| Olfr247 | 0.06907 | -1.0887 | 3469 | |
| Olfr403 | 0.069085 | -1.1093 | 3470 | |
| Pfkl | 0.069134 | -1.993 | 3471 | |
| Morc3 | 0.069165 | -0.47315 | 3472 | |
| Il7r | 0.069195 | -0.42538 | 3473 | |
| Plin5 | 0.069231 | -0.90296 | 3474 | |
| Umps | 0.069246 | -0.75879 | 3475 | |
| 1-Mar | 0.069318 | -2.0514 | 3476 | |
| B4galt5 | 0.06933 | -2.0507 | 3477 | |
| Zfp583 | 0.069343 | -1.0129 | 3478 | |
| Tmed8 | 0.069355 | -0.38793 | 3479 | |
| Stard6 | 0.069376 | -0.82236 | 3480 | |
| Trmt12 | 0.069384 | -1.2644 | 3481 | |
| Foxp4 | 0.069392 | -2.0474 | 3482 | |
| Adm2 | 0.069404 | -1.1343 | 3483 | |
| Map3k7cl | 0.069415 | -1.3742 | 3484 | |
| Zfp330 | 0.069422 | -2.0468 | 3485 | |
| Defb8 | 0.069423 | -1.4534 | 3486 | |
| Neurod1 | 0.069447 | 2.231 | 3487 | |
| Htra2 | 0.069478 | -1.533 | 3488 | |
| Vmn2r18 | 0.0695 | -1.0016 | 3489 | |
| Arap3 | 0.069515 | -1.9429 | 3490 | |
| Sult1b1 | 0.069534 | -1.6859 | 3491 | |
| Osbp2 | 0.069552 | -0.56282 | 3492 | |
| Amt | 0.069564 | -2.0413 | 3493 | |
| Wipf1 | 0.069595 | -1.9643 | 3494 | |
| Wfdc2 | 0.069607 | -1.0929 | 3495 | |
| Olfr66 | 0.069613 | -2.0441 | 3496 | |
| Lhx6 | 0.06963 | -0.94884 | 3497 | |
| Rhoc | 0.069664 | -0.7674 | 3498 | |
| Defb45 | 0.069707 | -0.59049 | 3499 | |
| Myef2 | 0.069707 | -0.76566 | 3500 | |
| Olfr1339 | 0.069731 | -0.80573 | 3501 | |
| mmu-mir-1961 | 0.069736 | -1.0379 | 3502 | |
| Gm14474 | 0.069745 | -1.4324 | 3503 | |
| Lce1a1 | 0.069751 | -1.6627 | 3504 | |
| Adipor2 | 0.069773 | -2.0412 | 3505 | |
| Glrp1 | 0.069794 | 1.2943 | 3506 | |
| Smc5 | 0.06981 | -2.0417 | 3507 | |
| Usp36 | 0.069847 | -1.6595 | 3508 | |
| Zglp1 | 0.069869 | -1.1387 | 3509 | |
| Nolc1 | 0.069908 | -0.95926 | 3510 | |
| Csrp3 | 0.069971 | -2.0445 | 3511 | |
| Gm5862 | 0.069982 | -3.098 | 3512 | |
| Lrrc30 | 0.070063 | -1.0326 | 3513 | |
| Sh3d21 | 0.070082 | -2.0408 | 3514 | |
| Usp37 | 0.070098 | -0.22422 | 3515 | |
| mmu-mir-7670 | 0.070116 | 0.16509 | 3516 | |
| mmu-mir-344e | 0.070166 | -0.49399 | 3517 | |
| Sec14l1 | 0.070181 | -1.7889 | 3518 | |
| Tpmt | 0.070184 | -0.68789 | 3519 | |
| Defa21 | 0.070184 | 0.93574 | 3520 | |
| Ddx4 | 0.070184 | 0.37903 | 3521 | |
| Naip5 | 0.070241 | -0.90343 | 3522 | |
| Zmym1 | 0.070255 | -2.0353 | 3523 | |
| Slc46a2 | 0.070271 | 0.48529 | 3524 | |
| Olfr786 | 0.070326 | -1.2881 | 3525 | |
| Gpr179 | 0.070334 | -1.5292 | 3526 | |
| mmu-mir-339 | 0.070346 | -0.75741 | 3527 | |
| Mtmr4 | 0.070357 | -0.85808 | 3528 | |
| mmu-mir-361 | 0.070366 | -1.4361 | 3529 | |
| Has2 | 0.070388 | -0.81561 | 3530 | |
| Aph1b | 0.070435 | -1.2922 | 3531 | |
| mmu-mir-5627 | 0.070458 | -0.9854 | 3532 | |
| Ccdc93 | 0.070613 | -1.022 | 3533 | |
| Ints10 | 0.070618 | 0.45945 | 3534 | |
| Bex4 | 0.070618 | 0.042888 | 3535 | |
| Olfr1036 | 0.070618 | -0.83929 | 3536 | |
| Igfbp1 | 0.070618 | -0.48837 | 3537 | |
| mmu-mir-686 | 0.070618 | -0.052908 | 3538 | |
| Sbno1 | 0.070618 | -0.93902 | 3539 | |
| Actn3 | 0.070637 | -1.6227 | 3540 | |
| B3gnt5 | 0.070663 | -2.0319 | 3541 | |
| mmu-mir-6414 | 0.070667 | -1.8614 | 3542 | |
| Bcr | 0.070668 | -0.77748 | 3543 | |
| Fam83e | 0.07073 | -0.09552 | 3544 | |
| Specc1 | 0.070762 | -1.5929 | 3545 | |
| Olfr57 | 0.070775 | -1.6693 | 3546 | |
| Arsa | 0.070785 | -0.83925 | 3547 | |
| Slc35d2 | 0.070792 | -1.175 | 3548 | |
| Vmn1r39 | 0.070878 | -0.99721 | 3549 | |
| Bbx | 0.070878 | -0.76179 | 3550 | |
| mmu-mir-181b-1 | 0.070948 | -1.5346 | 3551 | |
| Gpr85 | 0.070956 | -1.1177 | 3552 | |
| Ccdc64b | 0.070961 | -0.19766 | 3553 | |
| Alx4 | 0.070965 | -0.67311 | 3554 | |
| Ap4b1 | 0.070987 | -1.9142 | 3555 | |
| Crip2 | 0.070992 | -2.0268 | 3556 | |
| Akr1a1 | 0.071051 | -1.9123 | 3557 | |
| Atp5sl | 0.071089 | -1.8841 | 3558 | |
| Smg6 | 0.071097 | -1.5313 | 3559 | |
| Prdm4 | 0.071108 | -0.77319 | 3560 | |
| Cog4 | 0.07116 | -1.5635 | 3561 | |
| Olfr45 | 0.07116 | -1.2548 | 3562 | |
| Cpsf4l | 0.071172 | -2.0302 | 3563 | |
| Hmga2 | 0.071184 | -2.0078 | 3564 | |
| Ripk1 | 0.071225 | -0.18226 | 3565 | |
| Sgcg | 0.071229 | -0.7762 | 3566 | |
| Wbscr27 | 0.071268 | 0.10206 | 3567 | |
| Asgr2 | 0.071271 | -2.0282 | 3568 | |
| Cln5 | 0.071311 | -1.5116 | 3569 | |
| Kncn | 0.071331 | -0.99778 | 3570 | |
| Gnb3 | 0.071358 | -2.0265 | 3571 | |
| Pcdhb20 | 0.071398 | -1.0899 | 3572 | |
| Vmn1r7 | 0.07141 | -1.1607 | 3573 | |
| Mgea5 | 0.071446 | -0.5211 | 3574 | |
| Vmn2r57 | 0.071485 | -1.8925 | 3575 | |
| Vps37c | 0.071528 | -1.198 | 3576 | |
| Wtap | 0.071593 | -1.8545 | 3577 | |
| Vmn1r184 | 0.071607 | -0.3396 | 3578 | |
| mmu-mir-129-1 | 0.071608 | -0.82747 | 3579 | |
| Sco1 | 0.071645 | -1.0071 | 3580 | |
| Olfr914 | 0.071701 | -0.29381 | 3581 | |
| Gjb4 | 0.071739 | -1.057 | 3582 | |
| Ncoa2 | 0.071766 | -0.030346 | 3583 | |
| Cetn4 | 0.071807 | -0.79207 | 3584 | |
| Nkx2-1 | 0.071831 | -0.71219 | 3585 | |
| Btbd7 | 0.071841 | -0.93513 | 3586 | |
| Tmem147 | 0.071873 | -0.88877 | 3587 | |
| 1700018C11Rik | 0.071904 | -0.75066 | 3588 | |
| Olfr1259 | 0.071912 | -0.81133 | 3589 | |
| mmu-mir-3471-1 | 0.072065 | -1.6839 | 3590 | |
| Atxn2 | 0.072069 | -1.4437 | 3591 | |
| Rhox13 | 0.072125 | -1.4796 | 3592 | |
| Aanat | 0.072134 | -1.4755 | 3593 | |
| Tm9sf2 | 0.072134 | -0.7896 | 3594 | |
| Tm2d2 | 0.072134 | 2.0021 | 3595 | |
| Olfr985 | 0.072134 | -1.4567 | 3596 | |
| Ptpn3 | 0.072134 | -1.5602 | 3597 | |
| Naglu | 0.072196 | -1.1805 | 3598 | |
| Cnih3 | 0.072294 | -0.66538 | 3599 | |
| Churc1 | 0.072307 | -2.017 | 3600 | |
| Scgb1b19 | 0.072351 | -2.0171 | 3601 | |
| Hoxc4 | 0.072369 | -0.80018 | 3602 | |
| Rhot1 | 0.072382 | -2.0178 | 3603 | |
| Slc4a2 | 0.072407 | -0.55169 | 3604 | |
| Mrps15 | 0.072425 | -0.7538 | 3605 | |
| Vmn1r125 | 0.072436 | -1.7691 | 3606 | |
| Tarsl2 | 0.072504 | -0.87297 | 3607 | |
| Gdf7 | 0.072524 | 2.6064 | 3608 | |
| Mesdc1 | 0.072545 | -1.7484 | 3609 | |
| Gm11559 | 0.072559 | -0.96303 | 3610 | |
| Fosl1 | 0.072595 | -0.75634 | 3611 | |
| Olfr120 | 0.072639 | -1.0347 | 3612 | |
| Slc22a18 | 0.072654 | -0.59595 | 3613 | |
| Dnaic1 | 0.072658 | -2.0151 | 3614 | |
| Lcmt1 | 0.072694 | -1.5779 | 3615 | |
| Dll4 | 0.072714 | -2.0053 | 3616 | |
| Nt5c2 | 0.072734 | -1.3283 | 3617 | |
| Wdr77 | 0.072752 | -1.9539 | 3618 | |
| Pigg | 0.072758 | -0.80452 | 3619 | |
| Wwtr1 | 0.072784 | -0.70855 | 3620 | |
| Tdrd5 | 0.072784 | -1.8062 | 3621 | |
| Isg15 | 0.072789 | -1.7727 | 3622 | |
| Art4 | 0.072796 | -2.002 | 3623 | |
| Kcnk2 | 0.072821 | -1.3319 | 3624 | |
| mmu-mir-6966 | 0.072831 | -1.2104 | 3625 | |
| Alg8 | 0.072845 | -1.2539 | 3626 | |
| Cacna2d3 | 0.0729 | -0.88336 | 3627 | |
| Tmem59l | 0.072913 | -1.6591 | 3628 | |
| Dync2h1 | 0.072984 | -2.0114 | 3629 | |
| Rfxank | 0.072992 | -0.93138 | 3630 | |
| Chtf18 | 0.073028 | -0.27031 | 3631 | |
| Olfr670 | 0.073043 | -0.26044 | 3632 | |
| Olfr765 | 0.073067 | -0.8371 | 3633 | |
| Stam2 | 0.073083 | -1.1936 | 3634 | |
| Olfr818 | 0.073087 | -0.8287 | 3635 | |
| Gpr89 | 0.073099 | -0.86723 | 3636 | |
| Sp2 | 0.073122 | -0.89749 | 3637 | |
| Ccdc73 | 0.07313 | -1.6238 | 3638 | |
| Klk1b16 | 0.073139 | -0.75821 | 3639 | |
| Tln1 | 0.073179 | -1.8842 | 3640 | |
| Ifi204 | 0.073195 | -1.3001 | 3641 | |
| Olfr538 | 0.07321 | -1.5339 | 3642 | |
| BC016579 | 0.073211 | -1.3128 | 3643 | |
| Arpc5l | 0.073219 | -0.73701 | 3644 | |
| Cdk15 | 0.073273 | -1.6744 | 3645 | |
| Gtsf1 | 0.073298 | -0.77527 | 3646 | |
| Il2 | 0.073338 | -1.9087 | 3647 | |
| Tmem41a | 0.073374 | -1.9885 | 3648 | |
| Olfr368 | 0.073394 | -0.66632 | 3649 | |
| Mapk1ip1l | 0.073412 | -0.19371 | 3650 | |
| Gm4944 | 0.073442 | -1.0815 | 3651 | |
| Wipf3 | 0.07349 | -1.2373 | 3652 | |
| 5730455P16Rik | 0.073498 | 0.25637 | 3653 | |
| Naa15 | 0.073498 | -0.22329 | 3654 | |
| Gm10324 | 0.073498 | -0.72127 | 3655 | |
| 4930412D23Rik | 0.073498 | -0.0459 | 3656 | |
| Pigt | 0.073512 | -2.0049 | 3657 | |
| Cct5 | 0.07353 | -0.72367 | 3658 | |
| Sprr2h | 0.073586 | -1.182 | 3659 | |
| Habp4 | 0.073588 | -1.8245 | 3660 | |
| Nccrp1 | 0.073602 | -0.77786 | 3661 | |
| Olfr1447 | 0.073701 | -2.0013 | 3662 | |
| 2310045N01Rik | 0.073738 | -0.73645 | 3663 | |
| mmu-mir-6988 | 0.073762 | -0.63367 | 3664 | |
| Dao | 0.073786 | -0.74877 | 3665 | |
| Synpo | 0.07379 | -2.0011 | 3666 | |
| Adamts10 | 0.073822 | -1.37 | 3667 | |
| Zcchc13 | 0.073866 | -0.23652 | 3668 | |
| Psmc2 | 0.073887 | -1.197 | 3669 | |
| Oosp2 | 0.073916 | -1.2265 | 3670 | |
| Taf5 | 0.073979 | -1.9981 | 3671 | |
| Rnase6 | 0.074005 | -1.8779 | 3672 | |
| Slc38a8 | 0.074026 | -1.1413 | 3673 | |
| Cltb | 0.074034 | -0.7236 | 3674 | |
| Casp12 | 0.074038 | -1.3305 | 3675 | |
| Atp6v0e | 0.074038 | -0.55777 | 3676 | |
| Dr1 | 0.074125 | -0.63052 | 3677 | |
| Cd27 | 0.074131 | -1.4438 | 3678 | |
| Tppp3 | 0.074179 | -0.82246 | 3679 | |
| Ryr3 | 0.074232 | -1.9835 | 3680 | |
| Cstf3 | 0.074254 | -1.2951 | 3681 | |
| Vmn2r36 | 0.07432 | -2.1394 | 3682 | |
| Ly6i | 0.074341 | 1.0034 | 3683 | |
| Ptprv | 0.074346 | -1.1844 | 3684 | |
| Ssxb3 | 0.07436 | -1.0661 | 3685 | |
| Pfdn4 | 0.074397 | -1.982 | 3686 | |
| Mex3d | 0.074435 | -1.9929 | 3687 | |
| Olfr1316 | 0.074447 | -1.9828 | 3688 | |
| Glipr1l2 | 0.074449 | -1.1486 | 3689 | |
| Gpr20 | 0.074449 | 0.3947 | 3690 | |
| Ccl17 | 0.074466 | -1.98 | 3691 | |
| Edn2 | 0.074514 | -0.82512 | 3692 | |
| Mpzl2 | 0.074517 | -0.33764 | 3693 | |
| Tpsab1 | 0.074573 | -1.3199 | 3694 | |
| 2700049A03Rik | 0.074574 | -0.357 | 3695 | |
| Parp12 | 0.074579 | 0.16724 | 3696 | |
| Tbc1d17 | 0.074587 | -1.9932 | 3697 | |
| Stat6 | 0.074597 | -1.696 | 3698 | |
| Anln | 0.074638 | -1.3785 | 3699 | |
| Ncoa7 | 0.074643 | -0.23021 | 3700 | |
| mmu-mir-7064 | 0.074647 | 0.021692 | 3701 | |
| Ercc6l2 | 0.07465 | -1.993 | 3702 | |
| Cdca3 | 0.074676 | -1.9927 | 3703 | |
| 2410018M08Rik | 0.074687 | -1.1323 | 3704 | |
| Fstl1 | 0.07473 | -0.63406 | 3705 | |
| Spata3 | 0.074765 | -1.3304 | 3706 | |
| mmu-mir-1191 | 0.074783 | -0.035458 | 3707 | |
| Edc3 | 0.074791 | -1.6995 | 3708 | |
| Pogk | 0.074836 | -0.74413 | 3709 | |
| Cramp1l | 0.07486 | -1.3139 | 3710 | |
| Prr13 | 0.074879 | -1.9769 | 3711 | |
| mmu-mir-6358 | 0.074988 | -0.96726 | 3712 | |
| Hspb6 | 0.075031 | -0.84635 | 3713 | |
| Xlr | 0.075064 | -2.7057 | 3714 | |
| Naprt1 | 0.075091 | -1.2559 | 3715 | |
| Swt1 | 0.075119 | 0.98693 | 3716 | |
| Gemin4 | 0.075119 | 0.30209 | 3717 | |
| 4930519F16Rik | 0.075119 | -1.2217 | 3718 | |
| Txnl1 | 0.075123 | -0.73514 | 3719 | |
| Bpifb5 | 0.075152 | -1.9823 | 3720 | |
| Leprot | 0.075171 | -1.9865 | 3721 | |
| Dennd5a | 0.075172 | -0.94491 | 3722 | |
| Cxcl1 | 0.07518 | -1.2696 | 3723 | |
| 1700052N19Rik | 0.075188 | -0.92987 | 3724 | |
| Lyn | 0.075222 | -1.9872 | 3725 | |
| Zfp143 | 0.075253 | -0.74071 | 3726 | |
| Gigyf1 | 0.075326 | -0.92997 | 3727 | |
| Kcnab2 | 0.075358 | -1.4154 | 3728 | |
| Rasl11b | 0.075383 | -0.72836 | 3729 | |
| Prepl | 0.075413 | -1.9863 | 3730 | |
| Mapk14 | 0.075423 | -1.8062 | 3731 | |
| Fam71b | 0.075432 | -1.9855 | 3732 | |
| Crebl2 | 0.075456 | -0.88823 | 3733 | |
| Olfr960 | 0.075464 | -1.8931 | 3734 | |
| Myh11 | 0.075477 | -1.9835 | 3735 | |
| Schip1 | 0.07548 | -0.44222 | 3736 | |
| Avp | 0.075488 | -1.2042 | 3737 | |
| Ntsr1 | 0.075586 | -1.6128 | 3738 | |
| Acyp2 | 0.07563 | -0.70567 | 3739 | |
| Mlst8 | 0.075635 | -1.335 | 3740 | |
| Atp5d | 0.075637 | -0.80598 | 3741 | |
| 2310003H01Rik | 0.075674 | -1.9837 | 3742 | |
| Psmd3 | 0.075692 | -1.0956 | 3743 | |
| Trib3 | 0.075724 | -0.94141 | 3744 | |
| Ndufa2 | 0.075732 | -1.9489 | 3745 | |
| Fbxo30 | 0.07577 | -1.9584 | 3746 | |
| Tiam2 | 0.075796 | -1.0708 | 3747 | |
| Sertad4 | 0.07581 | -0.5943 | 3748 | |
| Cox6b1 | 0.075846 | -1.4577 | 3749 | |
| Scgb2b12 | 0.075853 | -1.7069 | 3750 | |
| Gstt1 | 0.075898 | -1.7423 | 3751 | |
| Pgc | 0.075923 | -1.98 | 3752 | |
| Acacb | 0.076042 | -1.9109 | 3753 | |
| Gata1 | 0.07607 | -1.9761 | 3754 | |
| Heatr5b | 0.076128 | -0.72981 | 3755 | |
| Fam168b | 0.07614 | -1.0359 | 3756 | |
| Ept1 | 0.076155 | -1.3807 | 3757 | |
| Ccl28 | 0.076181 | -1.185 | 3758 | |
| Map3k12 | 0.076282 | -1.9766 | 3759 | |
| Ccdc110 | 0.076285 | -1.7535 | 3760 | |
| Ube2d3 | 0.076296 | -0.73257 | 3761 | |
| Enam | 0.076328 | -0.9604 | 3762 | |
| Ablim3 | 0.076346 | -1.3482 | 3763 | |
| Olfr99 | 0.07637 | -0.95645 | 3764 | |
| Acot7 | 0.076384 | -1.9713 | 3765 | |
| 1810046K07Rik | 0.076487 | -0.18421 | 3766 | |
| Tas2r120 | 0.076501 | -0.7342 | 3767 | |
| Casp7 | 0.076563 | -1.4638 | 3768 | |
| Olfr944 | 0.076587 | -0.27926 | 3769 | |
| Polr3e | 0.076616 | -1.2007 | 3770 | |
| Slc12a8 | 0.076628 | -1.3455 | 3771 | |
| Uba7 | 0.076717 | -0.66362 | 3772 | |
| Mrps2 | 0.076764 | -0.81859 | 3773 | |
| Atp6v0a4 | 0.076788 | -0.15267 | 3774 | |
| Cntnap1 | 0.076829 | -1.7618 | 3775 | |
| Lhx1 | 0.07685 | -1.2695 | 3776 | |
| Slc43a3 | 0.076871 | -1.97 | 3777 | |
| Rimklb | 0.07691 | -1.9708 | 3778 | |
| Gpn3 | 0.076954 | -0.99721 | 3779 | |
| Crkl | 0.076954 | -0.45022 | 3780 | |
| Prl5a1 | 0.076954 | 0.42342 | 3781 | |
| Tomm40 | 0.076954 | -1.4242 | 3782 | |
| Invs | 0.076954 | -0.44749 | 3783 | |
| Sntg1 | 0.076961 | -1.9588 | 3784 | |
| Olfr788 | 0.076969 | -1.4888 | 3785 | |
| Sp8 | 0.077013 | -0.78314 | 3786 | |
| Morc2a | 0.077257 | -1.9602 | 3787 | |
| Zfp764 | 0.077283 | -0.63238 | 3788 | |
| Lrrc8a | 0.077291 | -1.1888 | 3789 | |
| Anxa10 | 0.077296 | -0.6067 | 3790 | |
| Wbp1l | 0.077299 | -0.93969 | 3791 | |
| Zbtb8a | 0.077307 | -1.6109 | 3792 | |
| Olfr616 | 0.077332 | -0.92512 | 3793 | |
| Acvr2b | 0.077357 | -1.0485 | 3794 | |
| BC048671 | 0.077364 | -1.1471 | 3795 | |
| Elavl2 | 0.077556 | -0.97728 | 3796 | |
| Clec7a | 0.077566 | -1.6975 | 3797 | |
| Rnf157 | 0.077589 | -0.48478 | 3798 | |
| Tmem240 | 0.077605 | -0.75857 | 3799 | |
| Slc39a11 | 0.077623 | -1.835 | 3800 | |
| Oprl1 | 0.077623 | -1.4554 | 3801 | |
| Cfc1 | 0.077671 | -0.9154 | 3802 | |
| Ptprg | 0.077752 | -1.39 | 3803 | |
| Omg | 0.077773 | -1.9532 | 3804 | |
| Zfp534 | 0.077776 | -2.3013 | 3805 | |
| Fanci | 0.077795 | -0.7386 | 3806 | |
| Tubgcp4 | 0.077862 | -0.73345 | 3807 | |
| Ccdc63 | 0.077889 | -1.6672 | 3808 | |
| Syndig1 | 0.077904 | -0.9777 | 3809 | |
| Tmem117 | 0.077937 | -0.94859 | 3810 | |
| Gm5416 | 0.077945 | -0.92475 | 3811 | |
| Cd79a | 0.077946 | -0.24666 | 3812 | |
| Sf3a1 | 0.077954 | -1.9245 | 3813 | |
| Dio1 | 0.077958 | -0.88383 | 3814 | |
| Nrg4 | 0.077995 | -1.7454 | 3815 | |
| Klk1b5 | 0.078025 | -1.9446 | 3816 | |
| Vmn1r37 | 0.078092 | -0.80679 | 3817 | |
| Morn2 | 0.078103 | -0.80441 | 3818 | |
| Aox1 | 0.078118 | -0.90343 | 3819 | |
| Dcp2 | 0.078161 | -0.72704 | 3820 | |
| Celsr1 | 0.078199 | -1.9543 | 3821 | |
| Stoml1 | 0.078226 | -0.3955 | 3822 | |
| Gm20939 | 0.078269 | 0.24314 | 3823 | |
| Slc30a7 | 0.078312 | 0.79516 | 3824 | |
| Shb | 0.078328 | -1.7719 | 3825 | |
| Olfr1505 | 0.07834 | -1.1021 | 3826 | |
| Setd2 | 0.078356 | -1.0698 | 3827 | |
| Calr | 0.078395 | -0.47466 | 3828 | |
| Zfp474 | 0.078399 | -1.596 | 3829 | |
| Zfp445 | 0.078432 | -0.79918 | 3830 | |
| Palm | 0.078442 | -1.4938 | 3831 | |
| Olfr1384 | 0.078458 | -1.9535 | 3832 | |
| Amh | 0.07847 | -1.0431 | 3833 | |
| Mpdu1 | 0.078506 | -0.38759 | 3834 | |
| Alg11 | 0.07852 | -0.80802 | 3835 | |
| Chrna2 | 0.078528 | -0.70754 | 3836 | |
| Ccin | 0.078571 | -1.1139 | 3837 | |
| mmu-mir-6352 | 0.078614 | -1.2678 | 3838 | |
| Zfp14 | 0.078653 | -1.9095 | 3839 | |
| Tarm1 | 0.078653 | -0.94389 | 3840 | |
| Apobec1 | 0.078666 | -0.41636 | 3841 | |
| Cops5 | 0.078687 | -0.83168 | 3842 | |
| F2r | 0.078705 | -0.40995 | 3843 | |
| Nmd3 | 0.078718 | -1.7669 | 3844 | |
| Olfr1076 | 0.078729 | -0.72606 | 3845 | |
| Kat5 | 0.078808 | -0.68873 | 3846 | |
| Olfr279 | 0.078871 | -0.81013 | 3847 | |
| Kl | 0.078921 | -0.74336 | 3848 | |
| Cep70 | 0.078951 | -1.5823 | 3849 | |
| Scd2 | 0.07898 | -1.2294 | 3850 | |
| Ppp1r11 | 0.079013 | -0.7989 | 3851 | |
| Arvcf | 0.079038 | -0.8935 | 3852 | |
| Kcnq3 | 0.079068 | -1.9428 | 3853 | |
| Ralgapa1 | 0.079114 | -1.0601 | 3854 | |
| Gapt | 0.079131 | 1.0393 | 3855 | |
| Hsfy2 | 0.079198 | -1.8823 | 3856 | |
| Sptbn4 | 0.079235 | -1.4562 | 3857 | |
| Il5ra | 0.07925 | -1.6579 | 3858 | |
| Ankrd34c | 0.079261 | -1.3838 | 3859 | |
| Ccl1 | 0.079269 | -0.71719 | 3860 | |
| Serpinb12 | 0.079276 | -1.9388 | 3861 | |
| Sun2 | 0.079276 | -0.41261 | 3862 | |
| Slc37a4 | 0.079282 | -1.2534 | 3863 | |
| Zfp185 | 0.079302 | -1.9327 | 3864 | |
| Ceacam2 | 0.079315 | -1.5866 | 3865 | |
| S100a2 | 0.079315 | -1.9458 | 3866 | |
| Cyp2s1 | 0.079324 | -0.70134 | 3867 | |
| Klri1 | 0.079325 | 0.74028 | 3868 | |
| Cubn | 0.079349 | -1.2223 | 3869 | |
| Mrgprb3 | 0.079354 | -1.9366 | 3870 | |
| 1700125H20Rik | 0.07938 | -1.2211 | 3871 | |
| Hormad1 | 0.07939 | -0.70035 | 3872 | |
| Pyhin1 | 0.079391 | -1.6968 | 3873 | |
| Eef1a2 | 0.079406 | -1.9067 | 3874 | |
| Pml | 0.079433 | -0.39142 | 3875 | |
| Ndufs1 | 0.079458 | -0.74218 | 3876 | |
| Zyg11b | 0.079476 | -1.0454 | 3877 | |
| Cytl1 | 0.079498 | -1.9417 | 3878 | |
| Stx18 | 0.079519 | -0.28105 | 3879 | |
| Myof | 0.079534 | -1.0652 | 3880 | |
| Myrfl | 0.079726 | -1.9322 | 3881 | |
| Tmprss3 | 0.079734 | 2.1675 | 3882 | |
| Cpxcr1 | 0.079734 | -1.7749 | 3883 | |
| Olfr1360 | 0.079734 | -1.1033 | 3884 | |
| Ermard | 0.079734 | -0.21926 | 3885 | |
| Olfr804 | 0.079753 | -1.1149 | 3886 | |
| mmu-mir-1946a | 0.079758 | -1.249 | 3887 | |
| Slc33a1 | 0.079811 | -1.9373 | 3888 | |
| Cxcl12 | 0.079824 | -1.9416 | 3889 | |
| Hist1h3b | 0.079863 | -0.93875 | 3890 | |
| Vps39 | 0.079902 | -1.9152 | 3891 | |
| Tmeff2 | 0.079941 | -1.2495 | 3892 | |
| Slc7a10 | 0.079989 | -1.0008 | 3893 | |
| Tmem51 | 0.079993 | -1.8919 | 3894 | |
| Plscr1 | 0.080006 | -1.5383 | 3895 | |
| Btrc | 0.080036 | -0.71135 | 3896 | |
| Fam210a | 0.080057 | -0.89789 | 3897 | |
| Tut1 | 0.080078 | -1.9169 | 3898 | |
| Zfp712 | 0.080098 | -1.9384 | 3899 | |
| Epha3 | 0.080117 | -1.9209 | 3900 | |
| Cd5 | 0.08015 | -1.9387 | 3901 | |
| Angpt1 | 0.080165 | -0.15034 | 3902 | |
| Tbc1d22a | 0.080165 | -0.56587 | 3903 | |
| Pole | 0.080165 | -1.1674 | 3904 | |
| Zfp827 | 0.080192 | -0.97404 | 3905 | |
| Tlcd2 | 0.080243 | -1.4627 | 3906 | |
| Adra2a | 0.080268 | -1.9379 | 3907 | |
| C87977 | 0.080268 | -1.9379 | 3908 | |
| Trappc5 | 0.080268 | -1.9379 | 3909 | |
| Rgma | 0.080294 | -1.1457 | 3910 | |
| Olfr585 | 0.08032 | -0.33257 | 3911 | |
| Ctsh | 0.080359 | 1.4806 | 3912 | |
| Utp14a | 0.080408 | -1.1703 | 3913 | |
| Zscan29 | 0.080423 | -0.20759 | 3914 | |
| Vsig4 | 0.080466 | -1.0557 | 3915 | |
| Olfr218 | 0.08053 | -1.899 | 3916 | |
| Cd59a | 0.080552 | -1.0176 | 3917 | |
| Tmem261 | 0.080552 | -0.54497 | 3918 | |
| Fyn | 0.080557 | -1.0049 | 3919 | |
| Pnpla3 | 0.080616 | -0.70486 | 3920 | |
| Ahi1 | 0.080621 | -1.2822 | 3921 | |
| Trmt112 | 0.080635 | -1.9216 | 3922 | |
| Zfp939 | 0.080638 | -0.39026 | 3923 | |
| Darc | 0.080684 | -1.883 | 3924 | |
| B230219D22Rik | 0.080726 | -1.9336 | 3925 | |
| Dclre1c | 0.080727 | -0.69368 | 3926 | |
| Serpina1f | 0.080766 | -1.9333 | 3927 | |
| Ubr5 | 0.080779 | -1.9312 | 3928 | |
| 1810043H04Rik | 0.080792 | -0.68136 | 3929 | |
| Atxn2l | 0.080897 | -0.77428 | 3930 | |
| D630023F18Rik | 0.08091 | -0.28377 | 3931 | |
| Olfr1106 | 0.080922 | -0.9883 | 3932 | |
| Vwce | 0.081037 | -1.0174 | 3933 | |
| AU040320 | 0.081041 | -1.4249 | 3934 | |
| H2-M10.1 | 0.081059 | -1.1311 | 3935 | |
| Arrdc1 | 0.081076 | -0.87153 | 3936 | |
| Ivns1abp | 0.081101 | -0.67913 | 3937 | |
| Nom1 | 0.081112 | 0.8423 | 3938 | |
| Noc3l | 0.081112 | -1.328 | 3939 | |
| Cma2 | 0.081112 | -0.1996 | 3940 | |
| Kcnj3 | 0.081212 | -1.8377 | 3941 | |
| mmu-mir-344-2 | 0.081261 | -1.4087 | 3942 | |
| Tom1 | 0.081315 | -0.6953 | 3943 | |
| Slc52a3 | 0.081324 | -1.9299 | 3944 | |
| Ceacam12 | 0.081324 | -1.9299 | 3945 | |
| Scd4 | 0.081358 | -0.70115 | 3946 | |
| Olfr980 | 0.081423 | -1.9211 | 3947 | |
| Acbd6 | 0.081456 | -0.023421 | 3948 | |
| Ccdc15 | 0.081488 | -0.019949 | 3949 | |
| Mapk1 | 0.081515 | -1.9274 | 3950 | |
| Cab39l | 0.08152 | 0.16173 | 3951 | |
| Polg | 0.08152 | -1.2967 | 3952 | |
| Hsf4 | 0.081529 | -0.90264 | 3953 | |
| B230217C12Rik | 0.081597 | -1.103 | 3954 | |
| Ppt1 | 0.081606 | -1.7197 | 3955 | |
| Trim30a | 0.081631 | -1.0918 | 3956 | |
| Zpbp | 0.081671 | -0.57735 | 3957 | |
| Rnf133 | 0.081712 | -1.9253 | 3958 | |
| Tcp10a | 0.081739 | -1.9202 | 3959 | |
| Kif2a | 0.081757 | -0.22287 | 3960 | |
| Rwdd4a | 0.081765 | -1.9252 | 3961 | |
| Eif1ad | 0.081929 | -0.73189 | 3962 | |
| Olfr639 | 0.081931 | -1.0976 | 3963 | |
| Vps33a | 0.081949 | -1.2285 | 3964 | |
| Slc35f6 | 0.082026 | -1.3116 | 3965 | |
| Dpm1 | 0.082058 | -0.62343 | 3966 | |
| H2-M10.5 | 0.082101 | -0.32993 | 3967 | |
| Fbln2 | 0.082144 | -0.62171 | 3968 | |
| 4930433I11Rik | 0.082146 | -1.2544 | 3969 | |
| mmu-mir-7677 | 0.082153 | -0.55303 | 3970 | |
| Tsen54 | 0.082181 | -1.5073 | 3971 | |
| Frmd8 | 0.082258 | -0.68555 | 3972 | |
| Olfr1029 | 0.082273 | 0.29408 | 3973 | |
| Trim43c | 0.082317 | -2.9314 | 3974 | |
| D830030K20Rik | 0.082318 | -0.84572 | 3975 | |
| Calcr | 0.082337 | -0.20438 | 3976 | |
| Spin1 | 0.082337 | 0.43052 | 3977 | |
| Rbm38 | 0.082402 | 0.090076 | 3978 | |
| Tg | 0.082422 | -0.8536 | 3979 | |
| Gamt | 0.082445 | -0.92193 | 3980 | |
| Lrrc61 | 0.082448 | -0.73425 | 3981 | |
| Olfr1506 | 0.082461 | -2.9799 | 3982 | |
| Zfp2 | 0.082491 | -1.4631 | 3983 | |
| Srxn1 | 0.082518 | -0.84354 | 3984 | |
| Fam132b | 0.082545 | -0.26959 | 3985 | |
| Chrna10 | 0.082569 | -0.75176 | 3986 | |
| Myl1 | 0.082571 | -1.9172 | 3987 | |
| Ankar | 0.08266 | -0.65653 | 3988 | |
| Ddb2 | 0.08266 | -0.58699 | 3989 | |
| Ptgr1 | 0.082681 | -1.7512 | 3990 | |
| S100a6 | 0.082707 | -0.78863 | 3991 | |
| Olfr97 | 0.082733 | -1.6071 | 3992 | |
| Casd1 | 0.082853 | -1.7208 | 3993 | |
| Ppp1r3e | 0.082862 | -1.185 | 3994 | |
| Adsl | 0.082888 | -1.5887 | 3995 | |
| 1700003F12Rik | 0.082917 | -0.84947 | 3996 | |
| Hnrnpa2b1 | 0.082923 | -1.1559 | 3997 | |
| Rnf182 | 0.08294 | -0.79707 | 3998 | |
| Pcdhgc3 | 0.082974 | -0.9047 | 3999 | |
| Mcm2 | 0.083035 | -1.9139 | 4000 | |
| mmu-mir-541 | 0.083149 | 0.12249 | 4001 | |
| Kndc1 | 0.083165 | -1.3063 | 4002 | |
| Zfyve21 | 0.083181 | -1.9079 | 4003 | |
| Olfr347 | 0.083197 | 0.0094099 | 4004 | |
| Amz2 | 0.083197 | -0.92844 | 4005 | |
| Adc | 0.083197 | -1.66 | 4006 | |
| Tardbp | 0.083197 | -0.63595 | 4007 | |
| Bora | 0.083197 | -0.5184 | 4008 | |
| Vmn2r102 | 0.083207 | -0.77596 | 4009 | |
| Miip | 0.083244 | -1.3896 | 4010 | |
| Rrp36 | 0.083265 | -1.4413 | 4011 | |
| Sec61b | 0.083287 | -1.5769 | 4012 | |
| Fabp7 | 0.083354 | -1.905 | 4013 | |
| Sri | 0.083433 | -0.55122 | 4014 | |
| Gdf3 | 0.083433 | -1.0324 | 4015 | |
| Zfand1 | 0.083443 | -1.1566 | 4016 | |
| Thumpd3 | 0.083453 | -1.8366 | 4017 | |
| Bcas2 | 0.083453 | -1.8366 | 4018 | |
| Elp6 | 0.083476 | -1.1915 | 4019 | |
| Cnot6 | 0.083495 | -0.68826 | 4020 | |
| Ptpn14 | 0.083519 | -0.69362 | 4021 | |
| Vmn1r167 | 0.083522 | -0.79919 | 4022 | |
| Pdzd7 | 0.083556 | -0.92891 | 4023 | |
| Olfr643 | 0.083582 | -0.77341 | 4024 | |
| Spock1 | 0.08369 | -1.5568 | 4025 | |
| Pnkd | 0.083713 | -1.9063 | 4026 | |
| mmu-mir-7214 | 0.083717 | -0.055707 | 4027 | |
| Scap | 0.083733 | -0.44781 | 4028 | |
| Pir | 0.083776 | -0.52411 | 4029 | |
| Slc25a45 | 0.083819 | -1.6314 | 4030 | |
| Susd2 | 0.083846 | -1.887 | 4031 | |
| Trim23 | 0.083884 | -1.8759 | 4032 | |
| Apc | 0.083884 | -0.15868 | 4033 | |
| Nxf7 | 0.083935 | -1.4587 | 4034 | |
| Xpr1 | 0.083948 | 0.526 | 4035 | |
| Myo1f | 0.083992 | -0.80042 | 4036 | |
| Trpd52l3 | 0.084005 | -0.66998 | 4037 | |
| Zdhhc22 | 0.084013 | -1.2285 | 4038 | |
| Akr1b8 | 0.084019 | -0.68392 | 4039 | |
| G6pc | 0.084077 | -1.145 | 4040 | |
| Vmn1r214 | 0.084106 | -0.78257 | 4041 | |
| Adamts15 | 0.08412 | -1.8187 | 4042 | |
| Fthl17 | 0.084158 | -0.67298 | 4043 | |
| Krt77 | 0.08421 | -0.78168 | 4044 | |
| Gpx8 | 0.084246 | -0.78312 | 4045 | |
| Klra17 | 0.084273 | -1.8793 | 4046 | |
| Crispld2 | 0.084289 | -0.66331 | 4047 | |
| Pcdh8 | 0.084315 | -0.66497 | 4048 | |
| Hnrnpl | 0.084334 | -0.79377 | 4049 | |
| Zfp112 | 0.084399 | -0.65355 | 4050 | |
| Mettl7a2 | 0.084406 | -1.195 | 4051 | |
| Neto1 | 0.08442 | -1.8952 | 4052 | |
| Olfr506 | 0.08442 | -0.15946 | 4053 | |
| Cnpy2 | 0.084463 | 0.043118 | 4054 | |
| Ctdsp1 | 0.084487 | -1.7227 | 4055 | |
| Gbp7 | 0.084549 | 0.15492 | 4056 | |
| Ctdnep1 | 0.084549 | -1.1665 | 4057 | |
| Narf | 0.084561 | -1.1634 | 4058 | |
| Slc6a19os | 0.084567 | -0.011035 | 4059 | |
| Tfdp2 | 0.084635 | -0.087334 | 4060 | |
| Lama2 | 0.084723 | -1.6275 | 4061 | |
| Hs2st1 | 0.084794 | -1.0079 | 4062 | |
| Exosc9 | 0.084849 | -0.7986 | 4063 | |
| Olfr1271 | 0.084849 | -1.7898 | 4064 | |
| Vps37b | 0.084875 | -1.8251 | 4065 | |
| Prss48 | 0.084915 | -1.8994 | 4066 | |
| Olfr447 | 0.084928 | -1.179 | 4067 | |
| Vdac3 | 0.084938 | -1.1202 | 4068 | |
| mmu-mir-6935 | 0.084985 | -1.2886 | 4069 | |
| Twf2 | 0.085022 | -1.1755 | 4070 | |
| Blnk | 0.085035 | -0.99966 | 4071 | |
| Arl15 | 0.085056 | -1.0519 | 4072 | |
| Olfr971 | 0.085064 | -0.020328 | 4073 | |
| Zbtb3 | 0.08507 | -0.92571 | 4074 | |
| Zyg11a | 0.085129 | -1.8985 | 4075 | |
| Rab24 | 0.08515 | -0.19328 | 4076 | |
| Nudcd1 | 0.08515 | 0.0042648 | 4077 | |
| Arhgap22 | 0.08515 | -0.68764 | 4078 | |
| Vill | 0.085158 | -1.2434 | 4079 | |
| Slc9a6 | 0.08517 | -0.705 | 4080 | |
| Sult1d1 | 0.085235 | 0.083658 | 4081 | |
| Pibf1 | 0.085264 | -1.3168 | 4082 | |
| Slc22a15 | 0.08527 | -1.8954 | 4083 | |
| 4921517D22Rik | 0.085299 | -0.67242 | 4084 | |
| Hspa2 | 0.085308 | -0.80573 | 4085 | |
| Casr | 0.085344 | -0.79963 | 4086 | |
| Gabarap | 0.085351 | -0.069409 | 4087 | |
| Tprg | 0.085352 | -0.68512 | 4088 | |
| Mob4 | 0.085407 | -1.2337 | 4089 | |
| Vmn1r5 | 0.085431 | -1.8961 | 4090 | |
| Rad54l | 0.085476 | -0.65167 | 4091 | |
| Nup188 | 0.085493 | -0.5584 | 4092 | |
| Aste1 | 0.085505 | -0.61258 | 4093 | |
| Bai3 | 0.08552 | -1.8361 | 4094 | |
| Ppm1k | 0.085547 | -1.3011 | 4095 | |
| Slco6d1 | 0.085552 | -1.89 | 4096 | |
| Cpa3 | 0.085573 | -1.393 | 4097 | |
| Rpl22l1 | 0.085579 | -0.43212 | 4098 | |
| Tff3 | 0.085621 | -0.63697 | 4099 | |
| Psg19 | 0.085639 | -1.5772 | 4100 | |
| Ddx60 | 0.085653 | -1.6148 | 4101 | |
| Tmem245 | 0.085687 | -1.8917 | 4102 | |
| Gatc | 0.085707 | -1.8942 | 4103 | |
| Cenpt | 0.085707 | -0.26959 | 4104 | |
| Ggnbp2 | 0.085707 | -1.1269 | 4105 | |
| mmu-mir-1b | 0.085708 | -0.6177 | 4106 | |
| Rnf10 | 0.085836 | -1.1706 | 4107 | |
| Snx30 | 0.085847 | -1.8203 | 4108 | |
| Tacc3 | 0.085865 | -0.80492 | 4109 | |
| Itpr2 | 0.085879 | -0.68507 | 4110 | |
| Krtap9-5 | 0.085922 | -0.28872 | 4111 | |
| Maob | 0.085964 | -0.94841 | 4112 | |
| C77080 | 0.085989 | -1.212 | 4113 | |
| Defb30 | 0.086007 | 0.35376 | 4114 | |
| Hnrnpul2 | 0.086016 | -0.65663 | 4115 | |
| Lce1c | 0.08607 | -1.2749 | 4116 | |
| Cyp2d22 | 0.086078 | -0.706 | 4117 | |
| Dnajb13 | 0.086093 | 0.18793 | 4118 | |
| Clec3a | 0.086093 | -0.90669 | 4119 | |
| Atf6 | 0.086124 | -1.8912 | 4120 | |
| Wwox | 0.086149 | -0.81052 | 4121 | |
| Mettl2 | 0.086158 | -1.2341 | 4122 | |
| Adig | 0.086179 | -0.073106 | 4123 | |
| Fubp1 | 0.086202 | -0.6867 | 4124 | |
| Oprm1 | 0.086222 | -0.43778 | 4125 | |
| mmu-mir-1952 | 0.086244 | -1.3947 | 4126 | |
| Rmdn2 | 0.086259 | -1.0616 | 4127 | |
| Astn1 | 0.086282 | -0.71961 | 4128 | |
| Lin28a | 0.086318 | -0.82818 | 4129 | |
| Olfr996 | 0.086327 | -1.5455 | 4130 | |
| Olfr902 | 0.08635 | -0.95941 | 4131 | |
| Gm3646 | 0.086353 | -0.70251 | 4132 | |
| mmu-mir-664 | 0.086357 | 1.0555 | 4133 | |
| mmu-mir-3091 | 0.08638 | -1.6196 | 4134 | |
| Ccdc59 | 0.086425 | -0.8171 | 4135 | |
| Dnajb4 | 0.086448 | -1.8891 | 4136 | |
| Aicda | 0.086473 | -0.82988 | 4137 | |
| Add3 | 0.086487 | -0.66539 | 4138 | |
| Olfr767 | 0.086488 | -1.8714 | 4139 | |
| Hist1h2bj | 0.086522 | -0.56191 | 4140 | |
| Rrp15 | 0.086522 | -0.75488 | 4141 | |
| Vcp | 0.086522 | -0.62627 | 4142 | |
| 2210404O09Rik | 0.086522 | -0.78176 | 4143 | |
| Gltpd2 | 0.086549 | -0.36796 | 4144 | |
| Olfr885 | 0.086585 | -1.0262 | 4145 | |
| Rpl7 | 0.086603 | -1.9478 | 4146 | |
| Nutf2 | 0.086603 | -1.9478 | 4147 | |
| Rpl8 | 0.086603 | -1.9478 | 4148 | |
| Smim12 | 0.086693 | -1.1566 | 4149 | |
| Vmn2r62 | 0.086775 | -0.83351 | 4150 | |
| Hbb-y | 0.086822 | -1.0423 | 4151 | |
| Kif2b | 0.086834 | -0.49506 | 4152 | |
| Plac8 | 0.086886 | 0.9001 | 4153 | |
| Bfsp2 | 0.086886 | -1.0569 | 4154 | |
| Cyp3a25 | 0.086942 | -0.76526 | 4155 | |
| Crisp3 | 0.08695 | -0.48262 | 4156 | |
| Gsdmc4 | 0.086959 | -0.93652 | 4157 | |
| Chd3 | 0.086993 | -0.97122 | 4158 | |
| Olfr824 | 0.087022 | -0.82147 | 4159 | |
| Bdnf | 0.087036 | 1.2517 | 4160 | |
| Angptl3 | 0.087079 | -1.1056 | 4161 | |
| Gdpd3 | 0.087124 | -1.0169 | 4162 | |
| Rab42 | 0.087137 | -0.45593 | 4163 | |
| Nr5a1 | 0.087138 | -1.6587 | 4164 | |
| 2900092C05Rik | 0.087151 | -1.8799 | 4165 | |
| Zfp600 | 0.087179 | -2.1241 | 4166 | |
| Foxg1 | 0.087183 | -0.64667 | 4167 | |
| Sfxn1 | 0.087273 | -1.8852 | 4168 | |
| mmu-mir-6357 | 0.087317 | -1.1463 | 4169 | |
| Rnf20 | 0.087336 | -0.043014 | 4170 | |
| Olfr582 | 0.0874 | 0.84542 | 4171 | |
| Cdc6 | 0.087485 | -0.46374 | 4172 | |
| Timm9 | 0.087485 | -0.86624 | 4173 | |
| mmu-mir-8107 | 0.087517 | -0.85439 | 4174 | |
| Upf2 | 0.087533 | -0.64141 | 4175 | |
| Amelx | 0.08755 | -0.54322 | 4176 | |
| 4930503E14Rik | 0.087557 | -1.8832 | 4177 | |
| Ppp1cb | 0.087559 | -1.1975 | 4178 | |
| Maats1 | 0.087578 | -1.8823 | 4179 | |
| Ecm1 | 0.087586 | -0.65314 | 4180 | |
| Phf19 | 0.087612 | -1.8801 | 4181 | |
| Kcnc3 | 0.087625 | -1.8828 | 4182 | |
| Ccnf | 0.087639 | -1.8518 | 4183 | |
| Cuedc2 | 0.087707 | -0.6067 | 4184 | |
| Cish | 0.08772 | -1.8772 | 4185 | |
| Begain | 0.087764 | 0.38731 | 4186 | |
| Lcp2 | 0.08782 | -0.67616 | 4187 | |
| Rims2 | 0.087874 | -0.72262 | 4188 | |
| Ubl7 | 0.087883 | -1.2948 | 4189 | |
| Birc7 | 0.087892 | 0.20665 | 4190 | |
| 5-Mar | 0.087924 | -1.1859 | 4191 | |
| Setmar | 0.087935 | 0.031222 | 4192 | |
| Erbb3 | 0.087978 | -1.1316 | 4193 | |
| Slc38a10 | 0.088018 | -1.2046 | 4194 | |
| Fancf | 0.088019 | -1.8794 | 4195 | |
| Ubr1 | 0.088031 | -1.4422 | 4196 | |
| Aup1 | 0.088126 | -0.87669 | 4197 | |
| 2210404O07Rik | 0.088153 | -0.59143 | 4198 | |
| Chmp4b | 0.08825 | -0.43974 | 4199 | |
| Bves | 0.088299 | -0.50337 | 4200 | |
| Hist1h2bg | 0.088299 | -0.70077 | 4201 | |
| P2ry13 | 0.088316 | -0.75934 | 4202 | |
| Slitrk2 | 0.088421 | -1.8564 | 4203 | |
| Ube3a | 0.088478 | -0.36066 | 4204 | |
| Cnpy4 | 0.088505 | -0.6428 | 4205 | |
| Kctd7 | 0.088537 | -0.58157 | 4206 | |
| Foxn1 | 0.088573 | -0.64953 | 4207 | |
| Acp1 | 0.088636 | -0.91044 | 4208 | |
| Gm11837 | 0.08865 | -0.77751 | 4209 | |
| Hcrtr2 | 0.088855 | -1.5045 | 4210 | |
| Als2cl | 0.088876 | -1.5569 | 4211 | |
| Zfp239 | 0.088894 | -1.4869 | 4212 | |
| Vmn2r67 | 0.08898 | -1.8729 | 4213 | |
| Svop | 0.088981 | -0.99627 | 4214 | |
| Gm14781 | 0.089005 | -1.2896 | 4215 | |
| Ccdc116 | 0.08904 | -1.0158 | 4216 | |
| Pi4kb | 0.089058 | -0.65445 | 4217 | |
| Krit1 | 0.08909 | 0.15899 | 4218 | |
| Snx6 | 0.08911 | -0.81096 | 4219 | |
| H1fnt | 0.089154 | 0.11858 | 4220 | |
| Amhr2 | 0.089154 | -1.4289 | 4221 | |
| Dennd4a | 0.089203 | -1.7035 | 4222 | |
| Camk1 | 0.089206 | -0.75366 | 4223 | |
| Fam178a | 0.089226 | -1.8706 | 4224 | |
| Dffb | 0.089239 | -1.6517 | 4225 | |
| Gm21943 | 0.08925 | -2.8522 | 4226 | |
| Serpinb3c | 0.089261 | -1.1918 | 4227 | |
| Zdhhc7 | 0.089262 | -0.28851 | 4228 | |
| Apol10b | 0.089304 | 0.20787 | 4229 | |
| Olfr150 | 0.089315 | -1.868 | 4230 | |
| Rspo3 | 0.089358 | -1.6565 | 4231 | |
| mmu-mir-1951 | 0.089359 | -0.95708 | 4232 | |
| Psat1 | 0.08939 | -1.333 | 4233 | |
| 1810022K09Rik | 0.089394 | -0.85355 | 4234 | |
| Adh7 | 0.089475 | -0.56016 | 4235 | |
| Myh14 | 0.089479 | -1.6618 | 4236 | |
| Olfr193 | 0.089485 | -1.4905 | 4237 | |
| Wdr46 | 0.089494 | -0.68046 | 4238 | |
| Ppp1r26 | 0.089518 | -0.38076 | 4239 | |
| Tekt5 | 0.089576 | -0.64841 | 4240 | |
| Tmem173 | 0.089582 | 0.31587 | 4241 | |
| Eya4 | 0.089594 | -0.88437 | 4242 | |
| Efna1 | 0.089602 | -1.6161 | 4243 | |
| B3gntl1 | 0.089622 | -0.72533 | 4244 | |
| Il1f8 | 0.089623 | -1.8356 | 4245 | |
| Necab1 | 0.089631 | -1.6094 | 4246 | |
| Ubqln4 | 0.08964 | -1.3342 | 4247 | |
| Dlg2 | 0.089668 | -0.61494 | 4248 | |
| Sult2a3 | 0.089732 | -0.5676 | 4249 | |
| Pabpc5 | 0.089768 | -1.3563 | 4250 | |
| Sh2d1b1 | 0.089777 | -0.74323 | 4251 | |
| mmu-let-7i | 0.089782 | -0.92387 | 4252 | |
| Apol7a | 0.089804 | -0.82376 | 4253 | |
| Nudt4 | 0.089817 | -1.2815 | 4254 | |
| Fkrp | 0.089822 | -1.3051 | 4255 | |
| Atp2b1 | 0.089877 | -1.7519 | 4256 | |
| Rem2 | 0.08995 | -1.3743 | 4257 | |
| Myh1 | 0.090014 | -1.2943 | 4258 | |
| Zfp54 | 0.090028 | -1.7682 | 4259 | |
| Apoo | 0.090116 | -0.76524 | 4260 | |
| Srp54a | 0.090116 | -0.42064 | 4261 | |
| Bcl2l11 | 0.090116 | -1.2819 | 4262 | |
| Reck | 0.090161 | -1.4677 | 4263 | |
| Myh3 | 0.090206 | -0.91227 | 4264 | |
| Tas2r144 | 0.090225 | -1.087 | 4265 | |
| Ptplb | 0.090234 | -1.7811 | 4266 | |
| Cpped1 | 0.090261 | -0.63312 | 4267 | |
| Ccdc167 | 0.090307 | -0.94901 | 4268 | |
| Fcna | 0.090309 | -1.8624 | 4269 | |
| Col4a6 | 0.09033 | -0.65014 | 4270 | |
| Ugt2b35 | 0.090385 | -1.8595 | 4271 | |
| Fbln5 | 0.090398 | -1.862 | 4272 | |
| Srsf12 | 0.090412 | -1.2944 | 4273 | |
| Dsg1b | 0.090426 | -1.8625 | 4274 | |
| Taco1 | 0.090435 | -0.88404 | 4275 | |
| Cd300e | 0.090458 | -0.2281 | 4276 | |
| Gtf2a1l | 0.090467 | -1.3189 | 4277 | |
| H1f0 | 0.090481 | -0.99082 | 4278 | |
| mmu-mir-7050 | 0.090488 | -0.80164 | 4279 | |
| Car7 | 0.09049 | -0.72709 | 4280 | |
| Hist1h2ac | 0.090544 | -0.60525 | 4281 | |
| Cpsf6 | 0.09055 | -1.8451 | 4282 | |
| Zfp772 | 0.090564 | -0.3736 | 4283 | |
| Cacnb2 | 0.090591 | -1.8601 | 4284 | |
| Rbm47 | 0.090608 | 0.18885 | 4285 | |
| Gm13139 | 0.090646 | -2.8717 | 4286 | |
| Nts | 0.090715 | -0.4436 | 4287 | |
| Mettl21e | 0.090761 | -0.69434 | 4288 | |
| Bre | 0.090779 | -0.26547 | 4289 | |
| Wee1 | 0.090839 | -1.4756 | 4290 | |
| Gpr132 | 0.090843 | -1.2889 | 4291 | |
| Ccrl2 | 0.090849 | -1.4092 | 4292 | |
| Skap1 | 0.090853 | -1.7174 | 4293 | |
| Fchsd2 | 0.090913 | -1.079 | 4294 | |
| Nfyc | 0.090922 | -0.056194 | 4295 | |
| Olfr867 | 0.09097 | -1.8561 | 4296 | |
| Lrrc10b | 0.090978 | -1.3163 | 4297 | |
| Bcl2l13 | 0.091014 | -0.89619 | 4298 | |
| Olfr1109 | 0.091032 | -1.8474 | 4299 | |
| Vps29 | 0.091078 | -0.60813 | 4300 | |
| Tubb2a | 0.091134 | -0.607 | 4301 | |
| Spg20 | 0.09118 | -0.23651 | 4302 | |
| Brd3 | 0.091184 | -1.6475 | 4303 | |
| Olfr194 | 0.091199 | -0.62458 | 4304 | |
| Zfp59 | 0.091204 | -1.1733 | 4305 | |
| Dgkd | 0.091206 | 1.6339 | 4306 | |
| Nras | 0.091254 | -0.98115 | 4307 | |
| Saa2 | 0.091263 | -0.77683 | 4308 | |
| Tchp | 0.09127 | 0.3024 | 4309 | |
| Zfp709 | 0.0913 | -0.61168 | 4310 | |
| Ralgds | 0.091309 | -1.0044 | 4311 | |
| Kcnb2 | 0.091383 | -0.63541 | 4312 | |
| Acsbg1 | 0.091393 | -0.80621 | 4313 | |
| Ano9 | 0.091411 | -0.73468 | 4314 | |
| D130043K22Rik | 0.091441 | -1.2258 | 4315 | |
| Trmu | 0.091483 | -1.6692 | 4316 | |
| mmu-mir-3470a | 0.091526 | -0.56198 | 4317 | |
| Agfg2 | 0.091531 | -0.78919 | 4318 | |
| Slc7a1 | 0.09155 | -0.77047 | 4319 | |
| C2cd3 | 0.091559 | -0.62963 | 4320 | |
| Myod1 | 0.091577 | -0.71254 | 4321 | |
| Fbxo46 | 0.091598 | -0.77136 | 4322 | |
| Gm7616 | 0.091651 | -0.67525 | 4323 | |
| mmu-mir-1901 | 0.091677 | 0.78954 | 4324 | |
| mmu-mir-7652 | 0.091689 | -0.43794 | 4325 | |
| Atg5 | 0.091716 | -0.63977 | 4326 | |
| Slc25a22 | 0.091736 | -0.49553 | 4327 | |
| Rab37 | 0.091744 | -1.0539 | 4328 | |
| Fau | 0.091782 | -1.6834 | 4329 | |
| 0610009D07Rik | 0.091782 | 0.44738 | 4330 | |
| Pls3 | 0.091782 | -0.31084 | 4331 | |
| Zfp511 | 0.091782 | 1.0605 | 4332 | |
| Usp16 | 0.091792 | -1.8515 | 4333 | |
| Cx3cl1 | 0.091833 | -0.48653 | 4334 | |
| Lipm | 0.091916 | -0.11055 | 4335 | |
| Cyp17a1 | 0.091939 | -1.1241 | 4336 | |
| Cacng2 | 0.091948 | -1.7275 | 4337 | |
| Mrto4 | 0.092003 | -0.79371 | 4338 | |
| Engase | 0.092041 | -1.2222 | 4339 | |
| A4galt | 0.092055 | -0.55086 | 4340 | |
| mmu-mir-6241 | 0.092072 | -0.42287 | 4341 | |
| Hspb9 | 0.092081 | -0.54204 | 4342 | |
| Actl6a | 0.092081 | -1.5366 | 4343 | |
| Dand5 | 0.092096 | -0.32227 | 4344 | |
| Mtmr2 | 0.092106 | -0.77962 | 4345 | |
| Olfr846 | 0.092138 | -0.6286 | 4346 | |
| 2810403A07Rik | 0.092209 | -0.13924 | 4347 | |
| Tap2 | 0.092226 | -1.2443 | 4348 | |
| Upf3b | 0.092235 | -0.68026 | 4349 | |
| Cyp2c29 | 0.092252 | 0.15999 | 4350 | |
| Hist1h2bb | 0.092254 | -0.62169 | 4351 | |
| Ankrd54 | 0.092263 | -1.8351 | 4352 | |
| Gja4 | 0.092263 | -1.3565 | 4353 | |
| Isy1 | 0.092276 | -1.1368 | 4354 | |
| Sdhd | 0.09229 | -1.848 | 4355 | |
| Spef2 | 0.092328 | -1.2114 | 4356 | |
| Parp1 | 0.092375 | -0.68252 | 4357 | |
| Vmn1r17 | 0.092417 | -0.87416 | 4358 | |
| Vipr1 | 0.092529 | -1.0719 | 4359 | |
| Atg7 | 0.092582 | -1.8449 | 4360 | |
| Arl6ip5 | 0.092593 | -0.69427 | 4361 | |
| Cdkl4 | 0.092617 | -1.8438 | 4362 | |
| Hif1an | 0.092636 | -0.0085772 | 4363 | |
| Azgp1 | 0.092665 | -1.8404 | 4364 | |
| Gbp4 | 0.092678 | -1.1386 | 4365 | |
| Tmem53 | 0.092679 | -1.845 | 4366 | |
| Peg10 | 0.092707 | -1.8439 | 4367 | |
| Prmt6 | 0.092748 | -1.0199 | 4368 | |
| Tspan7 | 0.092764 | 2.9466 | 4369 | |
| Armc9 | 0.092764 | -1.26 | 4370 | |
| Ces5a | 0.092764 | -1.0519 | 4371 | |
| Clca2 | 0.09279 | -1.7189 | 4372 | |
| Adprhl2 | 0.092901 | -1.2875 | 4373 | |
| Mtif2 | 0.092913 | 1.3431 | 4374 | |
| Deptor | 0.092913 | 0.12365 | 4375 | |
| Tsku | 0.092925 | -0.63407 | 4376 | |
| Aurkb | 0.092998 | -0.98138 | 4377 | |
| Frmd7 | 0.092999 | -1.8408 | 4378 | |
| Pdpk1 | 0.093027 | -1.7738 | 4379 | |
| Plod1 | 0.093028 | -0.96943 | 4380 | |
| Ip6k1 | 0.093062 | -0.70491 | 4381 | |
| Lmbrd1 | 0.093068 | -1.8411 | 4382 | |
| Inpp4b | 0.093138 | -0.13398 | 4383 | |
| Tapbp | 0.09314 | -0.93775 | 4384 | |
| Gas1 | 0.093148 | -0.45105 | 4385 | |
| Gm15299 | 0.09315 | -2.8224 | 4386 | |
| Dclk1 | 0.09318 | -1.8396 | 4387 | |
| Gm5549 | 0.09328 | -0.61466 | 4388 | |
| Cdkl5 | 0.093308 | -0.8477 | 4389 | |
| Ttc28 | 0.093322 | -0.62006 | 4390 | |
| Taf1d | 0.093333 | -1.8379 | 4391 | |
| Klra7 | 0.093361 | 0.016909 | 4392 | |
| Ube2q1 | 0.093403 | -1.0882 | 4393 | |
| Gm13247 | 0.093458 | -0.60267 | 4394 | |
| Arhgap17 | 0.093486 | -0.60347 | 4395 | |
| Ifit3 | 0.093514 | -1.807 | 4396 | |
| Npcd | 0.093553 | -2.084 | 4397 | |
| Nlrp5 | 0.093612 | -1.6588 | 4398 | |
| Glrb | 0.093617 | -1.7197 | 4399 | |
| Gse1 | 0.093721 | -0.95097 | 4400 | |
| Tnfsf13 | 0.093735 | -1.3596 | 4401 | |
| Ddr2 | 0.093737 | -1.535 | 4402 | |
| Ubn1 | 0.093744 | -1.5646 | 4403 | |
| Alg13 | 0.093807 | -1.8378 | 4404 | |
| 4930544D05Rik | 0.093821 | -0.76101 | 4405 | |
| Wasl | 0.093835 | -1.8374 | 4406 | |
| Synpr | 0.093843 | -1.5049 | 4407 | |
| Adad1 | 0.093849 | -1.8344 | 4408 | |
| Fgg | 0.093881 | -1.2298 | 4409 | |
| Rfpl3s | 0.093909 | -1.512 | 4410 | |
| Agbl4 | 0.093961 | -1.0623 | 4411 | |
| Ugt8a | 0.093961 | -0.97116 | 4412 | |
| Gm6408 | 0.093989 | -1.8362 | 4413 | |
| Nckap1 | 0.094031 | -0.74034 | 4414 | |
| Daf2 | 0.094043 | 1.6253 | 4415 | |
| BC016423 | 0.094043 | 0.083923 | 4416 | |
| Rps6 | 0.094043 | 0.24575 | 4417 | |
| mmu-mir-1948 | 0.094046 | -0.084986 | 4418 | |
| Il1a | 0.094069 | -1.3189 | 4419 | |
| Nckap5l | 0.094088 | -1.5114 | 4420 | |
| Unc5cl | 0.094102 | -1.2457 | 4421 | |
| Setd5 | 0.094125 | -1.0986 | 4422 | |
| Hist1h2ba | 0.094135 | -0.722 | 4423 | |
| Dkk1 | 0.094184 | -1.835 | 4424 | |
| Olfr1303 | 0.094296 | -1.8132 | 4425 | |
| Gm6034 | 0.094297 | -2.2661 | 4426 | |
| Synj2bp | 0.094333 | -1.4872 | 4427 | |
| Serpinb13 | 0.094341 | 0.26231 | 4428 | |
| Txnrd1 | 0.094389 | -1.3294 | 4429 | |
| Prcp | 0.094394 | -1.8299 | 4430 | |
| Crisp2 | 0.094408 | -0.76484 | 4431 | |
| Cd300lf | 0.094427 | -0.60295 | 4432 | |
| Tex37 | 0.094429 | -1.831 | 4433 | |
| Erp29 | 0.094446 | -0.67961 | 4434 | |
| Hhip | 0.094469 | -0.88453 | 4435 | |
| Crebbp | 0.094469 | 0.76977 | 4436 | |
| Aamp | 0.094469 | -1.3708 | 4437 | |
| Zfp324 | 0.094478 | -1.8279 | 4438 | |
| Olfr518 | 0.094597 | 0.74185 | 4439 | |
| St13 | 0.094618 | -0.31517 | 4440 | |
| Bmp6 | 0.094625 | -0.69575 | 4441 | |
| Rasl10a | 0.094632 | -0.56616 | 4442 | |
| Prrx2 | 0.09466 | -1.3948 | 4443 | |
| Dapk3 | 0.094674 | -1.831 | 4444 | |
| Btbd2 | 0.094701 | -0.5576 | 4445 | |
| Prss8 | 0.094724 | -0.034647 | 4446 | |
| Hspb2 | 0.094724 | -0.64922 | 4447 | |
| Dnajb6 | 0.094745 | -1.8296 | 4448 | |
| 1700101E01Rik | 0.094831 | 0.15667 | 4449 | |
| Grin1 | 0.094831 | -0.92287 | 4450 | |
| Gm5409 | 0.094871 | -1.5341 | 4451 | |
| Olfr522 | 0.094885 | -1.7299 | 4452 | |
| Cdrt4 | 0.094937 | -0.60066 | 4453 | |
| Asphd2 | 0.094947 | -0.59462 | 4454 | |
| Ufl1 | 0.094955 | -1.8281 | 4455 | |
| Fabp2 | 0.094966 | -0.97444 | 4456 | |
| 1700042G07Rik | 0.095013 | -1.3286 | 4457 | |
| Nup98 | 0.095023 | -0.47987 | 4458 | |
| Crxos | 0.095051 | -0.95362 | 4459 | |
| Igf2r | 0.095067 | -1.8228 | 4460 | |
| Khdc3 | 0.09507 | -1.0815 | 4461 | |
| Fam185a | 0.095086 | -0.78484 | 4462 | |
| Prob1 | 0.095086 | 0.02963 | 4463 | |
| Alg6 | 0.095095 | -1.8295 | 4464 | |
| Morc1 | 0.095108 | -0.74499 | 4465 | |
| Clptm1 | 0.095109 | -0.89224 | 4466 | |
| Tmsb15a | 0.095152 | -1.7292 | 4467 | |
| Atf7ip | 0.095166 | -1.8274 | 4468 | |
| Gspt1 | 0.095193 | -1.293 | 4469 | |
| Trim72 | 0.095222 | -0.88255 | 4470 | |
| Vezf1 | 0.095231 | -0.8784 | 4471 | |
| Tuba1a | 0.095236 | -1.261 | 4472 | |
| Mboat1 | 0.095288 | -0.60257 | 4473 | |
| mmu-mir-3077 | 0.095299 | -1.4644 | 4474 | |
| 4933426M11Rik | 0.095322 | -1.1613 | 4475 | |
| mmu-mir-29b-2 | 0.095355 | -0.63697 | 4476 | |
| 1700008P02Rik | 0.095355 | -0.39851 | 4477 | |
| Eddm3b | 0.095362 | -1.8234 | 4478 | |
| Rbm27 | 0.095376 | -1.827 | 4479 | |
| Olfr68 | 0.095406 | -0.51002 | 4480 | |
| mmu-mir-146a | 0.095426 | -0.39833 | 4481 | |
| Atp8b3 | 0.09545 | -0.29539 | 4482 | |
| Stat4 | 0.095498 | -0.5801 | 4483 | |
| Dnajc2 | 0.095536 | -0.66517 | 4484 | |
| Foxj2 | 0.095564 | -0.61949 | 4485 | |
| Tnfrsf21 | 0.095574 | -1.2786 | 4486 | |
| 2010012O05Rik | 0.095576 | -1.2142 | 4487 | |
| Hist1h2bp | 0.095576 | -0.92078 | 4488 | |
| Slc10a5 | 0.095644 | -1.5799 | 4489 | |
| Socs2 | 0.095658 | -1.8249 | 4490 | |
| Gpr137c | 0.095672 | -1.8251 | 4491 | |
| Olfr836 | 0.095678 | -1.8115 | 4492 | |
| Capns2 | 0.095704 | -1.5218 | 4493 | |
| Gm3985 | 0.095742 | -0.9615 | 4494 | |
| Prss41 | 0.095746 | -0.31523 | 4495 | |
| Olfr869 | 0.09577 | -1.7787 | 4496 | |
| Nipal2 | 0.095789 | -0.76063 | 4497 | |
| Ebp | 0.095793 | -0.54609 | 4498 | |
| Fam83h | 0.095831 | -0.66781 | 4499 | |
| Rnf149 | 0.095832 | 0.17727 | 4500 | |
| mmu-mir-19a | 0.095843 | -1.0638 | 4501 | |
| Sag | 0.09585 | -0.58495 | 4502 | |
| Arhgap1 | 0.095859 | -0.49163 | 4503 | |
| Slc27a5 | 0.095917 | -0.66425 | 4504 | |
| Zscan4d | 0.095945 | -1.0752 | 4505 | |
| Fap | 0.096017 | -1.821 | 4506 | |
| Hpse2 | 0.096084 | -0.86527 | 4507 | |
| Recql4 | 0.096146 | -0.6369 | 4508 | |
| Fbxo15 | 0.096151 | -1.5913 | 4509 | |
| Olfr1038-ps | 0.096151 | -1.2877 | 4510 | |
| Btf3 | 0.096151 | -1.3374 | 4511 | |
| Spaca5 | 0.09625 | -1.0673 | 4512 | |
| Vmn2r54 | 0.09626 | -0.59308 | 4513 | |
| Olfr547 | 0.096285 | -1.8193 | 4514 | |
| Slc38a3 | 0.096394 | -0.60502 | 4515 | |
| AI848285 | 0.096404 | -0.63909 | 4516 | |
| Tlr7 | 0.09649 | -1.2081 | 4517 | |
| Necap1 | 0.096534 | 0.16663 | 4518 | |
| Elmo2 | 0.096538 | -1.427 | 4519 | |
| Ccl3 | 0.096597 | 0.25074 | 4520 | |
| Arglu1 | 0.096653 | -1.508 | 4521 | |
| Dctd | 0.096661 | 0.95391 | 4522 | |
| Amot | 0.096661 | -0.67616 | 4523 | |
| Parva | 0.096662 | -0.74648 | 4524 | |
| Snapc2 | 0.096695 | -1.7926 | 4525 | |
| Ednrb | 0.096739 | -0.99325 | 4526 | |
| Mns1 | 0.096746 | -0.12851 | 4527 | |
| Ndufv3 | 0.09681 | 1.6157 | 4528 | |
| Hoxd1 | 0.096829 | -1.8116 | 4529 | |
| Mob1a | 0.096853 | 0.42338 | 4530 | |
| Pgrmc1 | 0.096864 | -0.6049 | 4531 | |
| Ppih | 0.096885 | -0.5218 | 4532 | |
| Foxn4 | 0.096912 | -1.2945 | 4533 | |
| Olfr202 | 0.09697 | -0.60411 | 4534 | |
| Ly6c1 | 0.09698 | -0.26891 | 4535 | |
| Cactin | 0.096985 | -1.8155 | 4536 | |
| Iqcj | 0.097018 | -1.4021 | 4537 | |
| mmu-mir-1291 | 0.097089 | -1.6068 | 4538 | |
| Fam84a | 0.097108 | -1.7246 | 4539 | |
| Klf12 | 0.09715 | 0.57489 | 4540 | |
| Col28a1 | 0.097162 | -1.8065 | 4541 | |
| C7 | 0.097235 | -0.50569 | 4542 | |
| Olfr1101 | 0.097235 | -1.6042 | 4543 | |
| mmu-mir-763 | 0.097264 | -1.3773 | 4544 | |
| Tfpi | 0.097296 | -0.28358 | 4545 | |
| Cryaa | 0.097342 | -0.63806 | 4546 | |
| Setdb1 | 0.097395 | -1.1059 | 4547 | |
| Tbata | 0.097405 | -0.60696 | 4548 | |
| Cd86 | 0.097448 | -1.2523 | 4549 | |
| Ppib | 0.097509 | -1.8101 | 4550 | |
| Bcl7c | 0.097557 | -0.59721 | 4551 | |
| Olfr138 | 0.097575 | -0.82642 | 4552 | |
| D4Wsu53e | 0.097594 | -0.66447 | 4553 | |
| Kras | 0.09766 | 0.40651 | 4554 | |
| Rgl2 | 0.097687 | -0.60066 | 4555 | |
| Prkcb | 0.097706 | -1.0684 | 4556 | |
| Hmces | 0.097771 | -0.068004 | 4557 | |
| Myo19 | 0.097788 | -1.6752 | 4558 | |
| Rpe | 0.097798 | -0.65406 | 4559 | |
| Vat1 | 0.097821 | -1.8061 | 4560 | |
| Hgd | 0.09783 | 0.20873 | 4561 | |
| BC027231 | 0.09783 | -1.5663 | 4562 | |
| Carhsp1 | 0.09783 | -0.26056 | 4563 | |
| Erp27 | 0.09783 | -1.627 | 4564 | |
| mmu-mir-5121 | 0.097841 | 0.54989 | 4565 | |
| 4933436I01Rik | 0.097875 | -0.39005 | 4566 | |
| Slc2a2 | 0.097904 | -1.2894 | 4567 | |
| Bcl9l | 0.097956 | -1.8089 | 4568 | |
| Ccdc22 | 0.097958 | -1.2178 | 4569 | |
| Gclc | 0.097977 | -0.016645 | 4570 | |
| Tigd2 | 0.098085 | -0.32488 | 4571 | |
| Olfr341 | 0.09812 | -1.068 | 4572 | |
| BC026590 | 0.09817 | 0.044769 | 4573 | |
| Nanos2 | 0.098195 | -0.65785 | 4574 | |
| Sva | 0.098204 | -0.60112 | 4575 | |
| Msl1 | 0.098219 | -1.7849 | 4576 | |
| Cdh17 | 0.098298 | -1.2107 | 4577 | |
| Sema4f | 0.098361 | 0.97438 | 4578 | |
| Myo1d | 0.098361 | -0.48155 | 4579 | |
| Fam222b | 0.098376 | -1.8064 | 4580 | |
| Dmbt1 | 0.098408 | -1.7341 | 4581 | |
| Ddx49 | 0.098425 | -0.96198 | 4582 | |
| Nipbl | 0.098529 | -0.77251 | 4583 | |
| Gm13547 | 0.098547 | -1.8047 | 4584 | |
| Mageb3 | 0.098554 | -0.57991 | 4585 | |
| Ctxn2 | 0.098582 | -1.805 | 4586 | |
| Gpr44 | 0.098602 | -0.72197 | 4587 | |
| Eml6 | 0.09866 | -0.59988 | 4588 | |
| Akap4 | 0.098661 | -0.60964 | 4589 | |
| Ilvbl | 0.098718 | -1.8029 | 4590 | |
| Txndc5 | 0.098719 | -0.68136 | 4591 | |
| Apool | 0.098782 | -0.89911 | 4592 | |
| Haus7 | 0.098782 | -1.7882 | 4593 | |
| Pnrc2 | 0.098807 | -1.6011 | 4594 | |
| Cd151 | 0.098807 | -0.30206 | 4595 | |
| Txnl4b | 0.098807 | -1.399 | 4596 | |
| Xiap | 0.098807 | 0.089317 | 4597 | |
| Uts2b | 0.098807 | 0.094154 | 4598 | |
| Utp18 | 0.098807 | -0.66337 | 4599 | |
| Psmb3 | 0.098807 | -1.6005 | 4600 | |
| Mrpl37 | 0.098923 | -1.2722 | 4601 | |
| Dtd1 | 0.098943 | -0.73238 | 4602 | |
| Olfr556 | 0.098952 | -1.1666 | 4603 | |
| Pkm | 0.098989 | -1.8011 | 4604 | |
| Otx2 | 0.099001 | -0.83399 | 4605 | |
| Trmt2a | 0.099003 | -1.801 | 4606 | |
| Csmd2 | 0.099017 | -0.44035 | 4607 | |
| Arhgef26 | 0.09906 | -1.5583 | 4608 | |
| Adck3 | 0.099079 | -0.81137 | 4609 | |
| 4931408C20Rik | 0.099089 | -0.37985 | 4610 | |
| Gm10081 | 0.099103 | -0.97146 | 4611 | |
| Col5a3 | 0.099217 | -1.7953 | 4612 | |
| Arf6 | 0.099253 | -0.66628 | 4613 | |
| Erv3 | 0.099253 | -1.3403 | 4614 | |
| Pip5kl1 | 0.099264 | -0.59023 | 4615 | |
| Adam39 | 0.099359 | 0.24223 | 4616 | |
| Vsx1 | 0.099403 | -0.078135 | 4617 | |
| Tmem50a | 0.099411 | -0.53422 | 4618 | |
| Csnk2a2 | 0.09943 | -0.91191 | 4619 | |
| Vps26b | 0.09944 | -0.74712 | 4620 | |
| Klf6 | 0.099479 | -0.89715 | 4621 | |
| mmu-mir-5099 | 0.099479 | -1.1347 | 4622 | |
| Cyp3a59 | 0.099508 | 0.37072 | 4623 | |
| Rps6ka5 | 0.099538 | -0.79723 | 4624 | |
| Gm13283 | 0.099546 | -0.74209 | 4625 | |
| Tsc22d2 | 0.099626 | -0.98782 | 4626 | |
| Bmf | 0.099675 | -1.7971 | 4627 | |
| Osbpl6 | 0.099699 | -0.20379 | 4628 | |
| Pdzk1 | 0.099724 | -0.75947 | 4629 | |
| Pklr | 0.099741 | 0.20211 | 4630 | |
| Fgfr1op | 0.099753 | -0.87848 | 4631 | |
| Rapgef1 | 0.099761 | -1.7699 | 4632 | |
| mmu-mir-1187 | 0.099762 | -1.3899 | 4633 | |
| Tasp1 | 0.099775 | -0.22884 | 4634 | |
| mmu-mir-7016 | 0.099779 | 0.22713 | 4635 | |
| Cotl1 | 0.099851 | -0.7881 | 4636 | |
| Sertm1 | 0.099861 | -0.77984 | 4637 | |
| Zscan4b | 0.099869 | -1.2895 | 4638 | |
| Prl2a1 | 0.09988 | -1.3461 | 4639 | |
| Gm13212 | 0.099899 | -1.7148 | 4640 | |
| Olfr309 | 0.099919 | -0.62234 | 4641 | |
| Ska2 | 0.099929 | -0.79124 | 4642 | |
| Pmaip1 | 0.099969 | -0.58002 | 4643 | |
| Arhgap29 | 0.099975 | 1.6459 | 4644 | |
| Tbcd | 0.099975 | -1.0307 | 4645 | |
| 9130019O22Rik | 0.099975 | -1.4925 | 4646 | |
| Vmn1r199 | 0.099998 | -0.60443 | 4647 | |
| Nxnl1 | 0.10001 | -0.56459 | 4648 | |
| Cyp4f17 | 0.10002 | -1.0475 | 4649 | |
| Gdnf | 0.10003 | -1.7852 | 4650 | |
| Pgrmc2 | 0.10008 | -0.55558 | 4651 | |
| Slc25a21 | 0.1001 | -1.1983 | 4652 | |
| Il15 | 0.10012 | -1.0643 | 4653 | |
| Fas | 0.10017 | -1.2555 | 4654 | |
| Olfr586 | 0.10022 | -1.5612 | 4655 | |
| Akirin1 | 0.10025 | -0.64747 | 4656 | |
| Slc38a7 | 0.10026 | -0.92885 | 4657 | |
| Rarg | 0.10027 | 0.022482 | 4658 | |
| Impdh2 | 0.10027 | -1.6038 | 4659 | |
| 4933402E13Rik | 0.10035 | -1.0337 | 4660 | |
| Tex101 | 0.1004 | -0.77665 | 4661 | |
| Xlr3a | 0.10042 | -0.89647 | 4662 | |
| Pltp | 0.10042 | -0.42505 | 4663 | |
| Gpc1 | 0.10043 | -1.6513 | 4664 | |
| Fn1 | 0.10046 | -1.784 | 4665 | |
| Fam216a | 0.10058 | -1.6357 | 4666 | |
| Atp6v1g2 | 0.10059 | -1.2915 | 4667 | |
| Gsg1 | 0.10061 | -1.6145 | 4668 | |
| Cyp2b9 | 0.10069 | -0.2205 | 4669 | |
| Zfp365 | 0.10072 | -1.1917 | 4670 | |
| Adcyap1r1 | 0.1008 | -0.64933 | 4671 | |
| Dos | 0.1008 | -0.90129 | 4672 | |
| Wbp1 | 0.10081 | -1.2578 | 4673 | |
| Atp8b4 | 0.10083 | -0.82155 | 4674 | |
| Rps12 | 0.10084 | -0.79863 | 4675 | |
| Cndp1 | 0.10084 | -0.21336 | 4676 | |
| Adamts7 | 0.10085 | -1.7854 | 4677 | |
| Catsperg1 | 0.10089 | -1.1577 | 4678 | |
| Ccbe1 | 0.10092 | -0.57573 | 4679 | |
| mmu-mir-6413 | 0.10093 | -0.75834 | 4680 | |
| Mrpl15 | 0.10098 | -1.7879 | 4681 | |
| Ppp2r1b | 0.10098 | -0.54374 | 4682 | |
| Entpd4 | 0.10101 | -1.3453 | 4683 | |
| Fhl5 | 0.10101 | -0.98512 | 4684 | |
| Lilra6 | 0.10101 | -0.40325 | 4685 | |
| Olfr1256 | 0.10101 | -0.82791 | 4686 | |
| Ap1s2 | 0.10101 | -0.33097 | 4687 | |
| Rp2h | 0.10107 | -1.7876 | 4688 | |
| Bhlha15 | 0.10108 | -1.7875 | 4689 | |
| Taar8c | 0.1011 | -0.7416 | 4690 | |
| Vmn1r79 | 0.10111 | -1.7715 | 4691 | |
| Osr2 | 0.10117 | -0.71175 | 4692 | |
| Ifih1 | 0.10118 | 0.96573 | 4693 | |
| Ercc6 | 0.1012 | -1.7865 | 4694 | |
| Tmem179b | 0.10121 | -1.0792 | 4695 | |
| Cryge | 0.10123 | -1.1725 | 4696 | |
| Krtap19-3 | 0.10124 | -0.99982 | 4697 | |
| Olfr340 | 0.10134 | -1.7242 | 4698 | |
| D430019H16Rik | 0.10141 | -0.56995 | 4699 | |
| Gpr107 | 0.10143 | -0.62999 | 4700 | |
| Ndc1 | 0.10144 | -1.5967 | 4701 | |
| Dock3 | 0.1015 | -0.23011 | 4702 | |
| Eif4e2 | 0.1015 | -0.8516 | 4703 | |
| Yars | 0.1015 | -1.5209 | 4704 | |
| Olfr601 | 0.10153 | -1.4711 | 4705 | |
| Hsf1 | 0.10155 | -0.79286 | 4706 | |
| Slc6a4 | 0.10156 | -0.56292 | 4707 | |
| Atg9a | 0.10159 | -0.84825 | 4708 | |
| Rgs4 | 0.10161 | -0.71552 | 4709 | |
| Cenpk | 0.10165 | -1.1581 | 4710 | |
| Snx32 | 0.10171 | -1.7815 | 4711 | |
| Lamtor3 | 0.10173 | -1.3944 | 4712 | |
| Psrc1 | 0.10176 | -1.7091 | 4713 | |
| Havcr1 | 0.10184 | -1.6596 | 4714 | |
| Crybb1 | 0.10184 | -0.59027 | 4715 | |
| Atp1a2 | 0.10184 | -0.82978 | 4716 | |
| Sppl2a | 0.10188 | -1.7812 | 4717 | |
| Siae | 0.10188 | -0.60105 | 4718 | |
| Zfp472 | 0.10189 | -0.56233 | 4719 | |
| Igll1 | 0.10191 | -1.7732 | 4720 | |
| Raf1 | 0.10192 | -0.56044 | 4721 | |
| 9430007A20Rik | 0.10193 | -1.0951 | 4722 | |
| Gm4794 | 0.10194 | -1.0189 | 4723 | |
| Ndufb8 | 0.10195 | -1.3947 | 4724 | |
| Olfr790 | 0.10198 | -0.8052 | 4725 | |
| Mettl23 | 0.10198 | -0.46687 | 4726 | |
| mmu-mir-222 | 0.10202 | -1.502 | 4727 | |
| Rpl32 | 0.10203 | -1.7772 | 4728 | |
| Cacna1h | 0.10204 | -1.6121 | 4729 | |
| Ift27 | 0.10206 | -0.81547 | 4730 | |
| Cnpy3 | 0.10208 | -0.65728 | 4731 | |
| BC048943 | 0.1021 | -0.62744 | 4732 | |
| Gm13088 | 0.10211 | -1.7799 | 4733 | |
| Bsph1 | 0.10212 | 0.22551 | 4734 | |
| Ube3c | 0.10216 | -1.7786 | 4735 | |
| Dusp26 | 0.1022 | 0.79094 | 4736 | |
| Fgfr1 | 0.10221 | -0.30927 | 4737 | |
| mmu-let-7a-2 | 0.10225 | 0.3486 | 4738 | |
| Rab1b | 0.10227 | -0.55093 | 4739 | |
| Pdgfc | 0.10231 | -0.57225 | 4740 | |
| Lamtor2 | 0.10232 | -0.98799 | 4741 | |
| Slc25a14 | 0.10232 | -0.62805 | 4742 | |
| Snapc3 | 0.10235 | -1.0941 | 4743 | |
| D830031N03Rik | 0.10237 | -1.0093 | 4744 | |
| Tigd5 | 0.10241 | -1.1574 | 4745 | |
| mmu-let-7f-2 | 0.10244 | -1.1854 | 4746 | |
| Serpine2 | 0.10247 | -0.90966 | 4747 | |
| C030039L03Rik | 0.10248 | -0.5717 | 4748 | |
| Odf3 | 0.10248 | -0.75796 | 4749 | |
| Nrgn | 0.10251 | -1.7766 | 4750 | |
| Eif1b | 0.10255 | -0.18485 | 4751 | |
| Rhbg | 0.10257 | -0.84318 | 4752 | |
| Btla | 0.10258 | -1.5924 | 4753 | |
| Musk | 0.10258 | -1.0468 | 4754 | |
| Zp3r | 0.1026 | -1.2931 | 4755 | |
| Mast1 | 0.10261 | -0.87477 | 4756 | |
| Gon4l | 0.10263 | -1.0935 | 4757 | |
| mmu-mir-27b | 0.10264 | 1.1072 | 4758 | |
| Fam105a | 0.10265 | -1.2199 | 4759 | |
| Dennd1b | 0.10267 | -1.0117 | 4760 | |
| Npy | 0.10271 | -0.81186 | 4761 | |
| Rrm2b | 0.10274 | -1.7745 | 4762 | |
| Rimbp3 | 0.1028 | -1.0457 | 4763 | |
| Ugt2a3 | 0.10288 | -1.6355 | 4764 | |
| Rgs10 | 0.10291 | -1.7273 | 4765 | |
| Olfr731 | 0.10292 | -0.027343 | 4766 | |
| Lrriq1 | 0.10293 | -0.0053175 | 4767 | |
| Stxbp6 | 0.10295 | -0.5228 | 4768 | |
| Parp9 | 0.10296 | -1.116 | 4769 | |
| Hps4 | 0.103 | -1.6727 | 4770 | |
| Zkscan1 | 0.103 | -0.58063 | 4771 | |
| Lrrc25 | 0.10301 | -1.2543 | 4772 | |
| Sept2 | 0.10304 | -1.1746 | 4773 | |
| Fam154a | 0.10308 | -0.55894 | 4774 | |
| Olfr823 | 0.10309 | -0.48558 | 4775 | |
| R3hdm2 | 0.10311 | -0.86916 | 4776 | |
| 4921524J17Rik | 0.10315 | -0.82845 | 4777 | |
| Cecr2 | 0.10315 | -1.7502 | 4778 | |
| Fastkd5 | 0.10322 | -0.27653 | 4779 | |
| Tex16 | 0.10327 | -1.6916 | 4780 | |
| Ceacam13 | 0.10327 | -0.76397 | 4781 | |
| Hmgxb3 | 0.10328 | -1.7682 | 4782 | |
| Serpina1e | 0.1033 | -0.27004 | 4783 | |
| Cyp8b1 | 0.1033 | -1.0841 | 4784 | |
| mmu-mir-450a-1 | 0.10335 | -1.1155 | 4785 | |
| Mbd6 | 0.10337 | -1.7677 | 4786 | |
| Cyp2d40 | 0.10344 | -1.4582 | 4787 | |
| Tcap | 0.10349 | -1.5127 | 4788 | |
| Ppp6r3 | 0.1035 | -1.7695 | 4789 | |
| Polr3g | 0.10351 | 0.34373 | 4790 | |
| Slc39a6 | 0.10351 | -0.29831 | 4791 | |
| Usp40 | 0.10351 | -0.57882 | 4792 | |
| Oca2 | 0.10352 | -0.70442 | 4793 | |
| Nek4 | 0.10355 | -0.80212 | 4794 | |
| Notch4 | 0.10356 | -1.2218 | 4795 | |
| 2700089E24Rik | 0.10362 | -0.7338 | 4796 | |
| Olfr352 | 0.10367 | -1.1635 | 4797 | |
| Spata21 | 0.10372 | -1.7612 | 4798 | |
| Rasa4 | 0.1038 | -1.7437 | 4799 | |
| Mrps24 | 0.10385 | -0.77965 | 4800 | |
| Dbi | 0.10386 | -1.7674 | 4801 | |
| Pcdhb7 | 0.10389 | -0.60956 | 4802 | |
| Mrgprd | 0.10391 | -1.764 | 4803 | |
| Pld2 | 0.104 | -0.56302 | 4804 | |
| Gm2663 | 0.10401 | -0.9744 | 4805 | |
| Smc2 | 0.10404 | -0.97767 | 4806 | |
| Rps20 | 0.10404 | -0.96205 | 4807 | |
| Fem1b | 0.10404 | 1.631 | 4808 | |
| Tcp1 | 0.10404 | -1.2803 | 4809 | |
| Nup214 | 0.10404 | -0.00099942 | 4810 | |
| mmu-mir-1966 | 0.10409 | -0.17651 | 4811 | |
| Il21 | 0.10414 | -0.58459 | 4812 | |
| Ythdc2 | 0.10416 | -0.75574 | 4813 | |
| 1700001C02Rik | 0.10418 | -1.4115 | 4814 | |
| 4930503B20Rik | 0.10419 | -0.98083 | 4815 | |
| Ppil4 | 0.10424 | -1.6513 | 4816 | |
| mmu-mir-34b | 0.10435 | -0.61637 | 4817 | |
| Ddx18 | 0.10439 | -0.8482 | 4818 | |
| Ascc2 | 0.10444 | -0.87303 | 4819 | |
| Gnat1 | 0.10446 | -1.7617 | 4820 | |
| Ovgp1 | 0.10451 | -0.97005 | 4821 | |
| Svs2 | 0.10451 | -0.038554 | 4822 | |
| Klrc3 | 0.10451 | 0.40564 | 4823 | |
| Il31ra | 0.10453 | -0.79003 | 4824 | |
| Cysltr1 | 0.10456 | -0.68618 | 4825 | |
| Hist1h1c | 0.1046 | -0.567 | 4826 | |
| Ccdc173 | 0.10468 | -1.7611 | 4827 | |
| mmu-let-7c-2 | 0.10469 | -1.3097 | 4828 | |
| Olfr1124 | 0.1047 | -0.56911 | 4829 | |
| Rrm2 | 0.10472 | -0.96538 | 4830 | |
| Svep1 | 0.10476 | -0.80732 | 4831 | |
| Sept1 | 0.10477 | -1.7517 | 4832 | |
| Dusp15 | 0.10485 | -1.3173 | 4833 | |
| mmu-mir-693 | 0.10487 | -0.41678 | 4834 | |
| Tmprss15 | 0.10489 | -1.7438 | 4835 | |
| Faim2 | 0.10491 | 0.26791 | 4836 | |
| Abcb11 | 0.10491 | -0.88901 | 4837 | |
| Olfr631 | 0.10492 | -1.1683 | 4838 | |
| Iglon5 | 0.10493 | -1.7598 | 4839 | |
| Znhit2 | 0.10494 | -1.1712 | 4840 | |
| Zfp780b | 0.10495 | -0.95494 | 4841 | |
| Frmd4a | 0.105 | -1.4495 | 4842 | |
| Raph1 | 0.105 | -1.7205 | 4843 | |
| Sec22b | 0.10508 | -1.0685 | 4844 | |
| Olfr1028 | 0.10508 | -0.4062 | 4845 | |
| Pcnxl4 | 0.10508 | 0.23608 | 4846 | |
| Mtmr3 | 0.10513 | -1.7589 | 4847 | |
| Rph3al | 0.1052 | -0.39869 | 4848 | |
| Slc25a2 | 0.10522 | -1.002 | 4849 | |
| Adamts14 | 0.10523 | -1.7582 | 4850 | |
| Chchd7 | 0.10526 | -1.758 | 4851 | |
| Acot1 | 0.10529 | -0.3097 | 4852 | |
| Cdc40 | 0.1053 | -0.92175 | 4853 | |
| Olfr1189 | 0.10531 | -0.99279 | 4854 | |
| R3hcc1 | 0.10534 | -0.72165 | 4855 | |
| Zfp426 | 0.10535 | -0.89241 | 4856 | |
| Gimap9 | 0.10535 | -1.3655 | 4857 | |
| Mb | 0.10537 | -0.84794 | 4858 | |
| Eif4e3 | 0.10537 | -0.090098 | 4859 | |
| Xlr4a | 0.10545 | -1.3576 | 4860 | |
| Tmprss11g | 0.10549 | -1.5665 | 4861 | |
| Hnrnpc | 0.10556 | -0.9809 | 4862 | |
| Slc22a26 | 0.10556 | 0.26871 | 4863 | |
| Dpy19l1 | 0.10566 | -1.0937 | 4864 | |
| Fscn2 | 0.10567 | 0.27391 | 4865 | |
| Olfr1477 | 0.10567 | -1.7011 | 4866 | |
| mmu-mir-6386 | 0.10568 | -0.35065 | 4867 | |
| Atp2b3 | 0.10573 | -1.656 | 4868 | |
| Rars | 0.10573 | -0.66687 | 4869 | |
| Gpr149 | 0.10579 | -1.3384 | 4870 | |
| Ccl5 | 0.10586 | -1.5851 | 4871 | |
| Tnk1 | 0.10587 | -1.7521 | 4872 | |
| Olfr770 | 0.10591 | -0.066848 | 4873 | |
| Ubr7 | 0.10592 | -0.99928 | 4874 | |
| Glra4 | 0.10593 | -1.1741 | 4875 | |
| Olfr1116-ps | 0.10594 | -0.96327 | 4876 | |
| Vmp1 | 0.10595 | -0.8442 | 4877 | |
| Rad51ap1 | 0.10595 | -0.91473 | 4878 | |
| Gnpda1 | 0.10596 | -0.53161 | 4879 | |
| Ggcx | 0.10597 | -1.7155 | 4880 | |
| Tubb3 | 0.106 | -0.10966 | 4881 | |
| Zfp385a | 0.10603 | -0.53865 | 4882 | |
| Trps1 | 0.10604 | -1.645 | 4883 | |
| 6030498E09Rik | 0.10605 | 0.14822 | 4884 | |
| Alg12 | 0.10608 | -1.1235 | 4885 | |
| Prl2c5 | 0.1061 | -0.59885 | 4886 | |
| Fzd5 | 0.1061 | -0.51043 | 4887 | |
| Mrpl42 | 0.10611 | -0.92659 | 4888 | |
| Olfr906 | 0.10611 | -0.46776 | 4889 | |
| Sp3 | 0.10616 | -0.47919 | 4890 | |
| Gabpa | 0.10618 | -0.71505 | 4891 | |
| Frmpd3 | 0.10619 | -1.0918 | 4892 | |
| Cnbp | 0.10625 | -0.56026 | 4893 | |
| Dpf1 | 0.10626 | -0.54713 | 4894 | |
| Rac2 | 0.10627 | -0.8137 | 4895 | |
| Fbxo4 | 0.10629 | -1.2112 | 4896 | |
| Spam1 | 0.10632 | -1.7507 | 4897 | |
| Kcnj1 | 0.10635 | -1.0635 | 4898 | |
| Olfr592 | 0.10638 | -1.7509 | 4899 | |
| mmu-mir-20b | 0.1064 | -0.91042 | 4900 | |
| Gm4302 | 0.10644 | -0.66611 | 4901 | |
| Cic | 0.10645 | -0.83936 | 4902 | |
| Cnksr3 | 0.10648 | -0.8048 | 4903 | |
| Gnptab | 0.10649 | -0.72755 | 4904 | |
| Cnga3 | 0.10651 | -0.56953 | 4905 | |
| Fmr1nb | 0.10653 | -0.79918 | 4906 | |
| Fbxo3 | 0.10654 | -0.53899 | 4907 | |
| Axin1 | 0.10654 | -1.1857 | 4908 | |
| Acsbg2 | 0.10655 | -0.61173 | 4909 | |
| Olfr1260 | 0.10664 | -1.6282 | 4910 | |
| Zc4h2 | 0.10664 | -1.7491 | 4911 | |
| Plin4 | 0.10666 | -1.2573 | 4912 | |
| Yeats4 | 0.10666 | -1.3585 | 4913 | |
| Nvl | 0.10666 | 0.18116 | 4914 | |
| Defb33 | 0.10666 | -1.2773 | 4915 | |
| Ppp1cc | 0.10685 | -0.52797 | 4916 | |
| Krtap13-1 | 0.10694 | -1.7469 | 4917 | |
| Gm9513 | 0.10698 | -0.7685 | 4918 | |
| Mrps18b | 0.10698 | -1.7439 | 4919 | |
| Gtsf1l | 0.107 | -1.7457 | 4920 | |
| Dagla | 0.10702 | 1.1742 | 4921 | |
| Stk4 | 0.10707 | -1.7271 | 4922 | |
| Usmg5 | 0.10711 | -0.4149 | 4923 | |
| Arap1 | 0.10712 | -0.5759 | 4924 | |
| Stpg2 | 0.10714 | -0.57013 | 4925 | |
| Papln | 0.10715 | 0.20133 | 4926 | |
| Olfr775 | 0.10721 | -1.286 | 4927 | |
| Dscc1 | 0.10722 | -0.85912 | 4928 | |
| Olfr635 | 0.1073 | 0.34235 | 4929 | |
| Il10 | 0.1073 | -0.87436 | 4930 | |
| Fam103a1 | 0.10736 | 0.19764 | 4931 | |
| Olfr498 | 0.10739 | -1.5448 | 4932 | |
| Slc9b2 | 0.10741 | -0.70723 | 4933 | |
| Nxf1 | 0.10741 | -1.0695 | 4934 | |
| Foxf1 | 0.10744 | -1.1582 | 4935 | |
| Acaca | 0.10744 | 0.46861 | 4936 | |
| Slc9a4 | 0.10747 | -1.3741 | 4937 | |
| Gm6904 | 0.10747 | -1.0495 | 4938 | |
| Samsn1 | 0.1075 | -1.4208 | 4939 | |
| Dedd | 0.10754 | -1.0428 | 4940 | |
| Rexo2 | 0.10754 | -1.7432 | 4941 | |
| Tchhl1 | 0.10755 | -0.89351 | 4942 | |
| Dennd1c | 0.10761 | -0.89458 | 4943 | |
| Abi3bp | 0.10761 | -0.99343 | 4944 | |
| Gfy | 0.10762 | -1.6713 | 4945 | |
| Parm1 | 0.10766 | -0.67547 | 4946 | |
| Gm11570 | 0.10767 | 0.37876 | 4947 | |
| Sema3b | 0.10773 | -1.7409 | 4948 | |
| Ppp2r3d | 0.10774 | 2.0865 | 4949 | |
| Col11a1 | 0.10778 | -1.1627 | 4950 | |
| Tnfrsf8 | 0.10779 | -0.52803 | 4951 | |
| Adcy2 | 0.1078 | -0.89913 | 4952 | |
| Kcnv1 | 0.10782 | -0.20469 | 4953 | |
| Glcci1 | 0.10784 | -0.5676 | 4954 | |
| Fam71d | 0.10791 | -1.7405 | 4955 | |
| 1700008O03Rik | 0.10793 | 1.6828 | 4956 | |
| Reg3g | 0.10796 | -0.5354 | 4957 | |
| Prf1 | 0.10797 | -1.74 | 4958 | |
| mmu-mir-344f | 0.10797 | -1.097 | 4959 | |
| Smug1 | 0.10801 | 0.57191 | 4960 | |
| Trip10 | 0.10806 | -0.44532 | 4961 | |
| Sybu | 0.10807 | -1.0947 | 4962 | |
| 5430435G22Rik | 0.10807 | -1.7358 | 4963 | |
| Rps6kb1 | 0.10808 | 0.22908 | 4964 | |
| Thra | 0.10812 | -0.26114 | 4965 | |
| Fbxw16 | 0.10812 | -1.0691 | 4966 | |
| Gm5127 | 0.10817 | -0.52245 | 4967 | |
| Ky | 0.10822 | -0.74757 | 4968 | |
| Lad1 | 0.10824 | -1.0317 | 4969 | |
| Ly6d | 0.10829 | -0.67137 | 4970 | |
| 3110082J24Rik | 0.10834 | -0.53058 | 4971 | |
| Syt8 | 0.10837 | -0.63785 | 4972 | |
| Senp6 | 0.1084 | -1.7352 | 4973 | |
| Cenpp | 0.10846 | -1.727 | 4974 | |
| Bcl2l2 | 0.10848 | -0.086584 | 4975 | |
| Olfr923 | 0.10848 | -0.9144 | 4976 | |
| Olfr111 | 0.10848 | 0.038714 | 4977 | |
| Asph | 0.10848 | -0.20945 | 4978 | |
| Spata7 | 0.1085 | -0.61211 | 4979 | |
| Mlh1 | 0.1085 | -0.54329 | 4980 | |
| Nhp2l1 | 0.10856 | -0.56198 | 4981 | |
| Tceal1 | 0.10857 | -0.50719 | 4982 | |
| Gm16442 | 0.10858 | -0.85502 | 4983 | |
| Atp2b4 | 0.10874 | -1.6445 | 4984 | |
| Sync | 0.10879 | -0.39407 | 4985 | |
| Ergic3 | 0.1088 | -0.97668 | 4986 | |
| Ugt2b38 | 0.10883 | -1.7339 | 4987 | |
| Atxn7l3b | 0.1089 | -0.062379 | 4988 | |
| Scp2 | 0.1089 | -1.7336 | 4989 | |
| Cyb5r1 | 0.10891 | -1.2515 | 4990 | |
| Gtf2a1 | 0.10893 | -0.98465 | 4991 | |
| Dmrt3 | 0.10893 | -1.7188 | 4992 | |
| Gfpt2 | 0.10898 | -1.7329 | 4993 | |
| Sgol1 | 0.10898 | -0.70317 | 4994 | |
| Ier2 | 0.109 | -1.2096 | 4995 | |
| Anxa3 | 0.10907 | 1.1643 | 4996 | |
| Ahnak | 0.10907 | -1.7322 | 4997 | |
| mmu-mir-669i | 0.10909 | -0.74312 | 4998 | |
| mmu-mir-135a-1 | 0.10915 | -1.2489 | 4999 | |
| Btc | 0.10915 | -1.7314 | 5000 | |
| Krtap19-1 | 0.10917 | -1.7129 | 5001 | |
| mmu-mir-30c-1 | 0.10925 | -1.3126 | 5002 | |
| Cpa6 | 0.1093 | -0.88065 | 5003 | |
| mmu-mir-672 | 0.10936 | 0.083153 | 5004 | |
| Mybl1 | 0.10938 | -0.30413 | 5005 | |
| Dnajb1 | 0.10938 | -0.26857 | 5006 | |
| Ccdc122 | 0.10938 | 0.47668 | 5007 | |
| Stfa2 | 0.10938 | -0.072327 | 5008 | |
| Gpatch4 | 0.10939 | -0.61829 | 5009 | |
| Il1r2 | 0.1094 | -0.51558 | 5010 | |
| Klhl21 | 0.10945 | -0.75558 | 5011 | |
| Ankrd42 | 0.10948 | -1.7223 | 5012 | |
| Tor1aip1 | 0.10954 | -0.97079 | 5013 | |
| Epcam | 0.10955 | -1.7282 | 5014 | |
| Gpr26 | 0.10959 | -1.6677 | 5015 | |
| Camsap2 | 0.10962 | -0.88068 | 5016 | |
| mmu-mir-200b | 0.10966 | -1.0061 | 5017 | |
| Olfr613 | 0.10968 | -1.7286 | 5018 | |
| Atp6v0d2 | 0.10969 | -1.7287 | 5019 | |
| Fkbpl | 0.10974 | -1.3943 | 5020 | |
| Ccnk | 0.10976 | -0.59427 | 5021 | |
| Vmn1r89 | 0.10976 | -0.68954 | 5022 | |
| Cox5a | 0.10976 | -0.21563 | 5023 | |
| Defa-rs1 | 0.1098 | -1.4388 | 5024 | |
| Eef1b2 | 0.10995 | -0.73898 | 5025 | |
| mmu-mir-370 | 0.10999 | -1.2507 | 5026 | |
| Col9a2 | 0.11001 | 0.11907 | 5027 | |
| Lrrc55 | 0.11004 | -0.86988 | 5028 | |
| Pfdn2 | 0.11006 | -1.4469 | 5029 | |
| mmu-mir-1941 | 0.11012 | -1.3432 | 5030 | |
| Polm | 0.11012 | -1.7246 | 5031 | |
| Ica1 | 0.11018 | -1.6175 | 5032 | |
| Rbpj | 0.11018 | -0.84014 | 5033 | |
| Olfr569 | 0.11021 | -1.6428 | 5034 | |
| Car14 | 0.11022 | 0.12755 | 5035 | |
| Tmem184c | 0.11027 | -1.2923 | 5036 | |
| Ins2 | 0.1103 | -1.0487 | 5037 | |
| Poc5 | 0.11033 | -0.34648 | 5038 | |
| Reep5 | 0.11033 | -0.89648 | 5039 | |
| Lce1b | 0.11034 | -1.7147 | 5040 | |
| Rd3 | 0.11039 | -0.83116 | 5041 | |
| Ppp4r2 | 0.11041 | -1.6715 | 5042 | |
| Fbp2 | 0.11046 | -1.6617 | 5043 | |
| Zbtbd6 | 0.11052 | -0.52831 | 5044 | |
| Pax1 | 0.11056 | 0.49045 | 5045 | |
| Lta4h | 0.11057 | -1.0334 | 5046 | |
| BC049762 | 0.11061 | -0.64175 | 5047 | |
| Olfr204 | 0.11064 | -0.8302 | 5048 | |
| Epha2 | 0.11065 | -1.7205 | 5049 | |
| Lrrc56 | 0.11067 | -1.5157 | 5050 | |
| Scmh1 | 0.1107 | -0.49644 | 5051 | |
| Efcab4b | 0.11072 | -0.11213 | 5052 | |
| Lrrc4c | 0.11073 | -1.094 | 5053 | |
| Cypt2 | 0.11073 | -1.2375 | 5054 | |
| Primpol | 0.11084 | -0.55715 | 5055 | |
| Hsd17b6 | 0.11086 | -0.69648 | 5056 | |
| 1810041L15Rik | 0.11088 | -0.49533 | 5057 | |
| Olfr1355 | 0.1109 | -0.33678 | 5058 | |
| Cspp1 | 0.11091 | -0.50091 | 5059 | |
| Tor3a | 0.11094 | -0.71915 | 5060 | |
| Nrep | 0.11106 | -0.97318 | 5061 | |
| 4930564B18Rik | 0.11109 | -1.047 | 5062 | |
| Pyurf | 0.11111 | 0.58784 | 5063 | |
| Plcd4 | 0.11115 | 1.4706 | 5064 | |
| Slc26a10 | 0.11118 | -0.9322 | 5065 | |
| Rpusd4 | 0.11119 | -1.6214 | 5066 | |
| Sh2b1 | 0.11121 | -1.3064 | 5067 | |
| Slc7a11 | 0.11133 | -0.26611 | 5068 | |
| Ankrd11 | 0.11136 | -1.7182 | 5069 | |
| Lman1 | 0.11138 | -0.54731 | 5070 | |
| Scyl1 | 0.11144 | -0.68764 | 5071 | |
| Rhbdl3 | 0.11148 | -0.92382 | 5072 | |
| Hoxd8 | 0.11149 | -1.0891 | 5073 | |
| Pced1b | 0.11151 | -1.717 | 5074 | |
| Skor2 | 0.11155 | -0.30926 | 5075 | |
| Cela1 | 0.11156 | -0.61181 | 5076 | |
| Dnase2a | 0.11157 | -0.48775 | 5077 | |
| 9130023H24Rik | 0.11158 | -1.2119 | 5078 | |
| Alox12b | 0.11159 | -0.90877 | 5079 | |
| Lhfpl3 | 0.11162 | -1.2699 | 5080 | |
| Cdx2 | 0.11177 | -1.4995 | 5081 | |
| Olfr887 | 0.11183 | -0.98036 | 5082 | |
| Olfr328 | 0.11187 | -0.51682 | 5083 | |
| Usp39 | 0.11189 | -0.54349 | 5084 | |
| Cc2d1b | 0.11199 | -0.23698 | 5085 | |
| Cybrd1 | 0.11203 | -0.7664 | 5086 | |
| Tnnc2 | 0.11204 | -0.77339 | 5087 | |
| Sytl2 | 0.11207 | -0.49924 | 5088 | |
| Olfr1537 | 0.11216 | -0.76092 | 5089 | |
| Olfr646 | 0.11218 | -0.49891 | 5090 | |
| Selrc1 | 0.11219 | -1.7096 | 5091 | |
| Klhl23 | 0.11222 | -1.7101 | 5092 | |
| Ptpn11 | 0.11222 | -1.7101 | 5093 | |
| Emx1 | 0.11222 | -0.6459 | 5094 | |
| Maz | 0.11223 | -0.59279 | 5095 | |
| Ndufs2 | 0.11225 | -1.689 | 5096 | |
| Pdcd5 | 0.11228 | -1.7082 | 5097 | |
| Sil1 | 0.11229 | -1.4163 | 5098 | |
| Ndufaf4 | 0.11232 | -1.5166 | 5099 | |
| Prl2b1 | 0.11233 | 0.35948 | 5100 | |
| Olfm4 | 0.11235 | -1.3254 | 5101 | |
| Defa26 | 0.11236 | -1.0886 | 5102 | |
| Cnrip1 | 0.11237 | -1.7073 | 5103 | |
| Vmn2r51 | 0.11237 | -1.3301 | 5104 | |
| Olfr201 | 0.11241 | -0.33454 | 5105 | |
| Hc | 0.11245 | -0.18186 | 5106 | |
| Mbip | 0.11247 | -0.74631 | 5107 | |
| Pstk | 0.11247 | -1.708 | 5108 | |
| Elp4 | 0.1125 | 0.2735 | 5109 | |
| Itih5 | 0.11253 | -0.53709 | 5110 | |
| Adk | 0.11254 | -0.44484 | 5111 | |
| Cml5 | 0.11264 | -0.36758 | 5112 | |
| mmu-mir-760 | 0.11272 | -0.36464 | 5113 | |
| Arl1 | 0.11274 | -1.7036 | 5114 | |
| Gstt4 | 0.11288 | -0.14716 | 5115 | |
| AA415398 | 0.11292 | -0.55615 | 5116 | |
| Gpr183 | 0.11296 | -0.18871 | 5117 | |
| Rbbp7 | 0.113 | -0.78019 | 5118 | |
| Gm13305 | 0.11301 | -1.186 | 5119 | |
| Hist1h4n | 0.11301 | -2.5257 | 5120 | |
| Casp1 | 0.11302 | -1.7042 | 5121 | |
| Wfdc10 | 0.11304 | -0.73023 | 5122 | |
| Tmem67 | 0.11305 | -1.6985 | 5123 | |
| Trpt1 | 0.11307 | -1.446 | 5124 | |
| Tbx18 | 0.11309 | -1.6452 | 5125 | |
| Xpo5 | 0.11313 | -1.3277 | 5126 | |
| mmu-mir-7081 | 0.11315 | -0.80094 | 5127 | |
| 1600014K23Rik | 0.11318 | -1.7018 | 5128 | |
| Fam195b | 0.11323 | -0.50685 | 5129 | |
| Olfr843 | 0.11323 | -0.41496 | 5130 | |
| Ipo7 | 0.11328 | -0.43815 | 5131 | |
| 2310057N15Rik | 0.1133 | -0.50097 | 5132 | |
| Slc7a6 | 0.11337 | -1.6513 | 5133 | |
| 1700042B14Rik | 0.11338 | -0.14425 | 5134 | |
| Zfp93 | 0.11339 | -0.65257 | 5135 | |
| Cdca7l | 0.11339 | -1.703 | 5136 | |
| Hmmr | 0.11342 | -0.35571 | 5137 | |
| Commd9 | 0.11344 | -1.7023 | 5138 | |
| Ccr10 | 0.11345 | -1.7028 | 5139 | |
| Extl1 | 0.11351 | -1.2358 | 5140 | |
| Chchd10 | 0.11351 | -1.5615 | 5141 | |
| Gucy2c | 0.11355 | 0.40799 | 5142 | |
| C78339 | 0.11359 | -0.32245 | 5143 | |
| 4930469G21Rik | 0.11364 | -0.54725 | 5144 | |
| mmu-mir-7227 | 0.11365 | -0.87098 | 5145 | |
| Rag1 | 0.11367 | -0.62272 | 5146 | |
| Impad1 | 0.1137 | -0.89208 | 5147 | |
| Vmn1r2 | 0.11371 | -1.1134 | 5148 | |
| Adam6a | 0.11376 | 0.10285 | 5149 | |
| Psmb9 | 0.11377 | -1.7003 | 5150 | |
| Tex26 | 0.1138 | -0.15582 | 5151 | |
| Defb9 | 0.1138 | -0.32287 | 5152 | |
| Krtap13 | 0.11381 | -0.51584 | 5153 | |
| Jph2 | 0.11384 | -0.94584 | 5154 | |
| Lrat | 0.11385 | -0.79275 | 5155 | |
| Rhog | 0.11388 | -0.55124 | 5156 | |
| Sgpp2 | 0.11388 | -0.13082 | 5157 | |
| Col6a3 | 0.11389 | -0.74848 | 5158 | |
| Tma7 | 0.1139 | -0.76021 | 5159 | |
| Sergef | 0.11392 | -1.4569 | 5160 | |
| Fpr-rs4 | 0.11397 | -0.091147 | 5161 | |
| Scrt1 | 0.11397 | -0.62071 | 5162 | |
| Spi1 | 0.11399 | -1.6995 | 5163 | |
| Itga4 | 0.11406 | -1.5149 | 5164 | |
| Fsip1 | 0.11409 | -1.6168 | 5165 | |
| Msx3 | 0.11409 | 0.0046294 | 5166 | |
| Sema6a | 0.11414 | -1.4218 | 5167 | |
| Blk | 0.11418 | -0.48854 | 5168 | |
| Ubqln1 | 0.11421 | -1.6987 | 5169 | |
| Tle2 | 0.11425 | -1.6958 | 5170 | |
| Trim34b | 0.11428 | -2.5144 | 5171 | |
| Dapp1 | 0.11429 | -0.74967 | 5172 | |
| Zfp879 | 0.1143 | -1.6969 | 5173 | |
| Dtl | 0.11432 | -1.5846 | 5174 | |
| Olfr464 | 0.11435 | -0.86563 | 5175 | |
| Cdc37 | 0.11437 | 0.6313 | 5176 | |
| Casc1 | 0.11437 | -0.58765 | 5177 | |
| Alg2 | 0.11437 | -1.2803 | 5178 | |
| Vash1 | 0.11437 | -1.2042 | 5179 | |
| Clec4a3 | 0.1144 | -0.652 | 5180 | |
| Sptbn1 | 0.11445 | -1.0783 | 5181 | |
| Fam115c | 0.11448 | -1.6947 | 5182 | |
| Cers6 | 0.11453 | -1.6963 | 5183 | |
| 2410004P03Rik | 0.11454 | -1.4893 | 5184 | |
| Olfr536 | 0.11462 | -1.6961 | 5185 | |
| Gng13 | 0.11464 | -0.85554 | 5186 | |
| Lefty2 | 0.11466 | -1.0775 | 5187 | |
| Pet100 | 0.11468 | -1.2495 | 5188 | |
| Wdr25 | 0.11469 | -1.6949 | 5189 | |
| Aldh8a1 | 0.11472 | -1.4699 | 5190 | |
| Gga2 | 0.11475 | -1.2194 | 5191 | |
| Sept5 | 0.11479 | -0.89692 | 5192 | |
| Arf3 | 0.11485 | -0.49292 | 5193 | |
| Diap1 | 0.11487 | -1.2747 | 5194 | |
| Nudt22 | 0.11489 | -0.55661 | 5195 | |
| Apoc2 | 0.1149 | -0.60367 | 5196 | |
| Gan | 0.11492 | -1.6867 | 5197 | |
| Nkd1 | 0.11496 | -1.4881 | 5198 | |
| Cfb | 0.11502 | -0.71683 | 5199 | |
| Ptchd1 | 0.11507 | -1.2344 | 5200 | |
| Lgmn | 0.1151 | -0.80716 | 5201 | |
| Zfp616 | 0.11513 | -1.5229 | 5202 | |
| Lgr4 | 0.11516 | -0.48878 | 5203 | |
| Pomc | 0.11518 | -0.67787 | 5204 | |
| Cga | 0.11525 | -0.33495 | 5205 | |
| Rictor | 0.11526 | -0.82234 | 5206 | |
| Olfr19 | 0.11529 | -1.5941 | 5207 | |
| Vps16 | 0.11539 | -1.253 | 5208 | |
| Olfr310 | 0.11539 | 0.65581 | 5209 | |
| Spen | 0.11546 | -0.27935 | 5210 | |
| Olfr1414 | 0.11546 | 0.077004 | 5211 | |
| Cyp2c44 | 0.11547 | -1.6841 | 5212 | |
| Gap43 | 0.11548 | -1.4079 | 5213 | |
| Rorc | 0.1155 | -0.11066 | 5214 | |
| Tcaim | 0.1155 | -1.6899 | 5215 | |
| Maf | 0.11552 | -0.7608 | 5216 | |
| Ccnl2 | 0.11559 | -1.6743 | 5217 | |
| Csk | 0.11559 | -1.0935 | 5218 | |
| Sec31a | 0.11561 | -0.81409 | 5219 | |
| Tpo | 0.11563 | -0.66132 | 5220 | |
| Gimap7 | 0.11565 | -0.812 | 5221 | |
| Gpcpd1 | 0.11571 | -1.0329 | 5222 | |
| Cytip | 0.11571 | -0.34647 | 5223 | |
| Faah | 0.11575 | -0.69578 | 5224 | |
| Rps27a | 0.11583 | -1.4586 | 5225 | |
| Rnf113a1 | 0.11583 | -0.95088 | 5226 | |
| Gpx1 | 0.11593 | -1.6382 | 5227 | |
| mmu-mir-3963 | 0.11596 | -0.16949 | 5228 | |
| Nup62 | 0.11598 | -1.6841 | 5229 | |
| Stxbp5l | 0.116 | -0.566 | 5230 | |
| Chd8 | 0.11604 | -1.0578 | 5231 | |
| Ube2j2 | 0.11605 | -1.1834 | 5232 | |
| Gm11569 | 0.11607 | -2.4835 | 5233 | |
| Fgf7 | 0.11608 | -0.49555 | 5234 | |
| Olfr107 | 0.11609 | -1.6854 | 5235 | |
| Pcnxl3 | 0.11612 | -1.6822 | 5236 | |
| Ano8 | 0.11613 | -0.56682 | 5237 | |
| Gm3259 | 0.11617 | -1.2465 | 5238 | |
| Ap3m1 | 0.11617 | -1.6095 | 5239 | |
| Gm16445 | 0.11618 | -0.75561 | 5240 | |
| Ankrd26 | 0.11623 | -0.65681 | 5241 | |
| Rnd1 | 0.11627 | -0.59699 | 5242 | |
| mmu-mir-124-1 | 0.11628 | -1.0291 | 5243 | |
| Olfr157 | 0.11632 | -0.81931 | 5244 | |
| Usp25 | 0.11635 | -1.6815 | 5245 | |
| Slc35a2 | 0.11636 | -1.1073 | 5246 | |
| Olfr745 | 0.11644 | -1.6721 | 5247 | |
| Ccdc103 | 0.11645 | -1.1229 | 5248 | |
| Tssk4 | 0.11647 | -1.6803 | 5249 | |
| Akap9 | 0.11648 | -0.90458 | 5250 | |
| Naa30 | 0.11652 | -1.6788 | 5251 | |
| Zfp697 | 0.11653 | 0.31594 | 5252 | |
| Pgam1 | 0.11657 | -0.067868 | 5253 | |
| Zc3h18 | 0.11661 | -0.80429 | 5254 | |
| Gnb5 | 0.11665 | -0.80065 | 5255 | |
| Tgfbr1 | 0.1167 | -1.414 | 5256 | |
| Olfr115 | 0.11671 | -1.6762 | 5257 | |
| Olfr1440 | 0.11681 | -1.676 | 5258 | |
| Prrc2c | 0.11684 | -1.4743 | 5259 | |
| Trim32 | 0.11685 | -0.57057 | 5260 | |
| Aox3 | 0.11689 | -0.46806 | 5261 | |
| Llph | 0.11694 | -1.6673 | 5262 | |
| Vps4b | 0.11699 | -1.3204 | 5263 | |
| Ereg | 0.117 | -1.6788 | 5264 | |
| Ankrd52 | 0.11705 | -1.4018 | 5265 | |
| Hemgn | 0.11713 | -0.6342 | 5266 | |
| Hiatl1 | 0.11721 | -0.48899 | 5267 | |
| Gm10921 | 0.11726 | -1.6207 | 5268 | |
| Kctd5 | 0.11731 | -1.6721 | 5269 | |
| Iqch | 0.11732 | -0.6783 | 5270 | |
| Sptlc3 | 0.11734 | -0.49535 | 5271 | |
| Olfr1415 | 0.11736 | -1.2142 | 5272 | |
| Olfr195 | 0.11741 | -0.36248 | 5273 | |
| Fam21 | 0.11743 | -1.6773 | 5274 | |
| Pja1 | 0.11745 | -1.0976 | 5275 | |
| Ralgapa2 | 0.11746 | -2.2455 | 5276 | |
| 1700013G24Rik | 0.11749 | -0.58052 | 5277 | |
| Pde7b | 0.1175 | -1.6727 | 5278 | |
| Grb7 | 0.11751 | -0.8012 | 5279 | |
| Gcnt3 | 0.11753 | 1.246 | 5280 | |
| H2-Ke2 | 0.11757 | -1.3749 | 5281 | |
| Rfx6 | 0.1176 | -1.51 | 5282 | |
| Ubl3 | 0.1176 | -0.46733 | 5283 | |
| Fndc3a | 0.1177 | -0.93863 | 5284 | |
| Nsd1 | 0.11771 | -1.4644 | 5285 | |
| Zmynd10 | 0.11774 | -1.6681 | 5286 | |
| Olfm3 | 0.11777 | -0.14723 | 5287 | |
| Ripk3 | 0.11778 | -1.4048 | 5288 | |
| Nsun2 | 0.1178 | -0.63293 | 5289 | |
| Ankrd46 | 0.1178 | -0.74689 | 5290 | |
| Hecw1 | 0.11783 | -1.182 | 5291 | |
| mmu-mir-382 | 0.11783 | -0.87529 | 5292 | |
| Vmn2r124 | 0.11787 | 0.1833 | 5293 | |
| Tmem170b | 0.11789 | -1.3964 | 5294 | |
| 0610010K14Rik | 0.11791 | -1.0619 | 5295 | |
| Pdlim5 | 0.11795 | -1.0021 | 5296 | |
| BC048507 | 0.11799 | 0.28237 | 5297 | |
| B3gat1 | 0.11802 | -0.5142 | 5298 | |
| 9430015G10Rik | 0.11804 | -0.47368 | 5299 | |
| Uchl4 | 0.11807 | -1.6736 | 5300 | |
| Lyzl6 | 0.11808 | 0.089867 | 5301 | |
| Mmd | 0.11809 | -1.6721 | 5302 | |
| Efhc2 | 0.11815 | -1.6708 | 5303 | |
| Nop10 | 0.11816 | -0.8394 | 5304 | |
| Gm5431 | 0.11818 | 0.32531 | 5305 | |
| Ccnyl1 | 0.11819 | -0.68855 | 5306 | |
| Nupl1 | 0.1182 | -0.8139 | 5307 | |
| Sult3a1 | 0.11822 | -1.1771 | 5308 | |
| Gdap1 | 0.11823 | -0.92479 | 5309 | |
| Sestd1 | 0.11824 | -0.12849 | 5310 | |
| Fn3k | 0.11824 | -1.6711 | 5311 | |
| Cryga | 0.11826 | -1.6715 | 5312 | |
| Gm11487 | 0.11831 | -1.4855 | 5313 | |
| Olfr1090 | 0.11831 | -0.75922 | 5314 | |
| Hist1h2an | 0.11836 | -1.2954 | 5315 | |
| Marf1 | 0.11841 | 0.95989 | 5316 | |
| Ttc39d | 0.11842 | -1.6582 | 5317 | |
| Zfp687 | 0.11844 | -1.6555 | 5318 | |
| Awat1 | 0.11845 | -0.45729 | 5319 | |
| H2-Ab1 | 0.11849 | -0.96737 | 5320 | |
| Folr1 | 0.11851 | -0.6218 | 5321 | |
| Plek2 | 0.11858 | -1.3222 | 5322 | |
| 9930021J03Rik | 0.1186 | -0.4863 | 5323 | |
| Zeb2os | 0.11864 | -0.81305 | 5324 | |
| Abca17 | 0.11866 | -1.6623 | 5325 | |
| Rapgef6 | 0.11866 | -0.012454 | 5326 | |
| Lphn2 | 0.11866 | -0.51969 | 5327 | |
| Lrrd1 | 0.11875 | 0.47626 | 5328 | |
| Nudt19 | 0.11883 | -0.25617 | 5329 | |
| Zfp345 | 0.11884 | -1.5827 | 5330 | |
| Olfr1156 | 0.11885 | -0.82776 | 5331 | |
| Hao1 | 0.11886 | -0.28209 | 5332 | |
| Gm2030 | 0.11888 | -0.081492 | 5333 | |
| Oxa1l | 0.11888 | -1.0117 | 5334 | |
| Tor1b | 0.11889 | 0.080447 | 5335 | |
| Acta1 | 0.11899 | -1.0499 | 5336 | |
| Sntg2 | 0.119 | 0.26078 | 5337 | |
| mmu-mir-697 | 0.11901 | -1.2165 | 5338 | |
| Zfp503 | 0.11907 | -1.6647 | 5339 | |
| Brca2 | 0.11908 | -0.76332 | 5340 | |
| mmu-mir-7210 | 0.11914 | -1.1358 | 5341 | |
| Cebpe | 0.11916 | -1.1344 | 5342 | |
| Pex14 | 0.11918 | -0.99223 | 5343 | |
| Trpm2 | 0.11919 | -0.64625 | 5344 | |
| Tfb1m | 0.11927 | -1.6624 | 5345 | |
| Fam89b | 0.11929 | -0.27829 | 5346 | |
| BC029214 | 0.11932 | -1.5392 | 5347 | |
| Samd4b | 0.11937 | -1.0409 | 5348 | |
| Fgf16 | 0.11939 | -0.35149 | 5349 | |
| Tpbpb | 0.11939 | -0.2004 | 5350 | |
| Psd4 | 0.11941 | -1.6534 | 5351 | |
| mmu-mir-1949 | 0.11942 | -0.91209 | 5352 | |
| Olfr1047 | 0.11943 | -0.77426 | 5353 | |
| Olfr127 | 0.11946 | -1.6612 | 5354 | |
| Cd3d | 0.11956 | -0.56449 | 5355 | |
| Olfr128 | 0.11958 | -1.661 | 5356 | |
| Sppl2c | 0.11963 | -0.43437 | 5357 | |
| Smg5 | 0.11963 | -1.6587 | 5358 | |
| Vmn2r47 | 0.11966 | -2.1959 | 5359 | |
| mmu-mir-6999 | 0.11967 | -1.2496 | 5360 | |
| Fmo5 | 0.11969 | -1.6442 | 5361 | |
| Nusap1 | 0.11971 | -0.71088 | 5362 | |
| Yipf4 | 0.11973 | -0.21782 | 5363 | |
| Mtch2 | 0.11973 | -0.53176 | 5364 | |
| Rpl10a | 0.11973 | -0.34047 | 5365 | |
| Itpa | 0.11973 | -1.0451 | 5366 | |
| 1700016K19Rik | 0.11974 | -0.45412 | 5367 | |
| Fam83d | 0.11978 | -1.6606 | 5368 | |
| Nfe2 | 0.11987 | -1.5542 | 5369 | |
| Aifm1 | 0.11995 | -1.6597 | 5370 | |
| Itgb5 | 0.11998 | -0.77193 | 5371 | |
| mmu-mir-501 | 0.11999 | -0.56051 | 5372 | |
| mmu-mir-669n | 0.12004 | -1.2551 | 5373 | |
| Pcdhb19 | 0.12005 | -0.45838 | 5374 | |
| Gabrp | 0.12006 | -1.6444 | 5375 | |
| Lims1 | 0.12009 | -0.5839 | 5376 | |
| mmu-mir-122 | 0.12013 | -0.94689 | 5377 | |
| Olfr741 | 0.12032 | -1.6549 | 5378 | |
| Cers3 | 0.12038 | -1.4845 | 5379 | |
| Gm7030 | 0.12038 | -0.4079 | 5380 | |
| Vmn1r211 | 0.12038 | -1.629 | 5381 | |
| Olfr1019 | 0.1204 | -0.89164 | 5382 | |
| Tsc1 | 0.12041 | -0.88585 | 5383 | |
| Reln | 0.12042 | -0.59163 | 5384 | |
| 4930505A04Rik | 0.12043 | -1.6527 | 5385 | |
| Iqcf3 | 0.12046 | -0.54897 | 5386 | |
| mmu-mir-1902 | 0.12046 | -0.89863 | 5387 | |
| Etv4 | 0.12054 | -1.6568 | 5388 | |
| Prdm5 | 0.12055 | -0.45837 | 5389 | |
| Rln3 | 0.12056 | -0.44305 | 5390 | |
| Marcksl1 | 0.12059 | -0.59664 | 5391 | |
| Gm20594 | 0.12063 | 1.061 | 5392 | |
| Dmp1 | 0.12073 | -0.98486 | 5393 | |
| Tctn3 | 0.12074 | -1.6566 | 5394 | |
| Krtap9-1 | 0.12077 | -1.1521 | 5395 | |
| Gpr151 | 0.12078 | -0.7656 | 5396 | |
| Sema3c | 0.12079 | -0.058768 | 5397 | |
| E230019M04Rik | 0.12085 | -1.3768 | 5398 | |
| Orc3 | 0.12086 | -1.0416 | 5399 | |
| Myl12a | 0.12086 | -0.99092 | 5400 | |
| Ppp2r5b | 0.12096 | -0.25233 | 5401 | |
| mmu-mir-6976 | 0.12097 | -0.90416 | 5402 | |
| Mmp7 | 0.12104 | -0.12192 | 5403 | |
| Dnah8 | 0.12105 | -0.87602 | 5404 | |
| Olfr733 | 0.12108 | -0.46693 | 5405 | |
| Cdh19 | 0.12114 | -0.53423 | 5406 | |
| Zfp58 | 0.12117 | -0.44999 | 5407 | |
| Dbp | 0.12117 | -0.67195 | 5408 | |
| mmu-mir-6395 | 0.12124 | -0.73107 | 5409 | |
| 4933408B17Rik | 0.12124 | -1.1873 | 5410 | |
| Pbsn | 0.12124 | -0.46455 | 5411 | |
| A630007B06Rik | 0.12125 | -0.25234 | 5412 | |
| Olfr303 | 0.1213 | -1.5014 | 5413 | |
| Sp5 | 0.12132 | -0.25423 | 5414 | |
| Rsc1a1 | 0.12132 | 0.59751 | 5415 | |
| Psmd13 | 0.12135 | -0.50813 | 5416 | |
| Atp6v1h | 0.12138 | -0.52381 | 5417 | |
| Tmem126b | 0.1214 | -0.48119 | 5418 | |
| Tceanc | 0.12143 | -1.0626 | 5419 | |
| Spryd7 | 0.12144 | -0.47001 | 5420 | |
| Rabepk | 0.12146 | -0.52682 | 5421 | |
| Slc5a4a | 0.1215 | -0.46598 | 5422 | |
| Lin28b | 0.1215 | -0.49723 | 5423 | |
| Trmt61a | 0.1215 | -0.79907 | 5424 | |
| Rhox2d | 0.12151 | -1.5865 | 5425 | |
| Lalba | 0.12156 | -1.6519 | 5426 | |
| Slc6a20b | 0.12157 | -0.69957 | 5427 | |
| Lrrc49 | 0.12162 | -1.5093 | 5428 | |
| Mapkap1 | 0.12164 | -1.65 | 5429 | |
| Pde1b | 0.12175 | -0.52763 | 5430 | |
| Zfp451 | 0.12178 | -0.44516 | 5431 | |
| Pde7a | 0.12178 | 0.47105 | 5432 | |
| Mid1 | 0.12178 | -0.10783 | 5433 | |
| Ankrd17 | 0.1218 | -1.225 | 5434 | |
| Hmgb4 | 0.12181 | -0.75024 | 5435 | |
| Chst2 | 0.12184 | -0.71464 | 5436 | |
| Stag2 | 0.12185 | -0.43196 | 5437 | |
| Palmd | 0.12188 | -0.47467 | 5438 | |
| Map1a | 0.12192 | -1.2883 | 5439 | |
| Pdcl2 | 0.12196 | -1.6324 | 5440 | |
| Ccpg1 | 0.12198 | -0.58597 | 5441 | |
| Btf3l4 | 0.122 | -0.88402 | 5442 | |
| Tlr1 | 0.12201 | -0.44662 | 5443 | |
| Gm5916 | 0.12203 | -1.3855 | 5444 | |
| Sf3b1 | 0.12205 | -0.99067 | 5445 | |
| Baiap2l2 | 0.12209 | -1.6485 | 5446 | |
| Stk24 | 0.12209 | -0.59931 | 5447 | |
| Pkd2 | 0.1221 | -0.98447 | 5448 | |
| Mrgprf | 0.12214 | -1.6484 | 5449 | |
| Zfp873 | 0.12217 | -1.1728 | 5450 | |
| B020031M17Rik | 0.12217 | 0.076352 | 5451 | |
| Atp6v1g3 | 0.12228 | -0.32169 | 5452 | |
| Gm5901 | 0.12232 | -0.43535 | 5453 | |
| Twf1 | 0.12232 | -1.6185 | 5454 | |
| Olfr378 | 0.12234 | -0.46762 | 5455 | |
| Hif1a | 0.12236 | -0.18461 | 5456 | |
| Kcnj5 | 0.12237 | -0.65848 | 5457 | |
| Afg3l2 | 0.1224 | -1.6465 | 5458 | |
| mmu-mir-466q | 0.12242 | -2.4124 | 5459 | |
| Myb | 0.12242 | -0.2164 | 5460 | |
| Sh3glb1 | 0.12243 | -1.6237 | 5461 | |
| Aoc2 | 0.12252 | -0.53966 | 5462 | |
| Stt3b | 0.12252 | -1.0054 | 5463 | |
| Gnpat | 0.12254 | -1.6384 | 5464 | |
| Ston2 | 0.12258 | -0.78774 | 5465 | |
| Pex11b | 0.12261 | -0.37983 | 5466 | |
| Ccl12 | 0.12263 | -0.96737 | 5467 | |
| Gpr174 | 0.12272 | -0.62709 | 5468 | |
| mmu-mir-28b | 0.12274 | -0.72274 | 5469 | |
| Rpl24 | 0.1228 | -0.8019 | 5470 | |
| Tmem41b | 0.1228 | 1.6661 | 5471 | |
| Hist1h2bh | 0.1228 | 0.73268 | 5472 | |
| Prr24 | 0.12282 | -0.26284 | 5473 | |
| Olfr361 | 0.12289 | -1.6407 | 5474 | |
| Ncor1 | 0.1229 | -1.1755 | 5475 | |
| Lca5 | 0.12292 | -0.4481 | 5476 | |
| Actr5 | 0.12293 | -1.488 | 5477 | |
| Bri3bp | 0.12296 | -1.5586 | 5478 | |
| Alyref2 | 0.123 | -1.2237 | 5479 | |
| Cyp21a1 | 0.12302 | -1.639 | 5480 | |
| Nat2 | 0.12307 | -0.034468 | 5481 | |
| Gga1 | 0.12308 | -0.68927 | 5482 | |
| Morc4 | 0.12309 | -0.53757 | 5483 | |
| Rpl36 | 0.1231 | -0.08938 | 5484 | |
| Ttc30a2 | 0.12314 | -1.6425 | 5485 | |
| H2-K1 | 0.12319 | -1.6203 | 5486 | |
| Ybx1 | 0.1232 | -1.292 | 5487 | |
| Timm44 | 0.1232 | -0.57832 | 5488 | |
| Rela | 0.12323 | -0.82788 | 5489 | |
| Dennd6b | 0.12326 | 1.1624 | 5490 | |
| Gipc3 | 0.12328 | -1.6371 | 5491 | |
| Topaz1 | 0.12334 | 0.73504 | 5492 | |
| Klk1b24 | 0.12341 | -0.55223 | 5493 | |
| B3gnt7 | 0.12342 | -0.52967 | 5494 | |
| Mical1 | 0.12353 | -1.6377 | 5495 | |
| Taar1 | 0.12355 | -0.64189 | 5496 | |
| Arhgap5 | 0.1236 | -0.80864 | 5497 | |
| Gm12888 | 0.12361 | -0.45002 | 5498 | |
| Pnpla2 | 0.12367 | -0.23319 | 5499 | |
| Clstn1 | 0.12371 | -0.011144 | 5500 | |
| 9530053A07Rik | 0.12375 | -1.6359 | 5501 | |
| Zfp936 | 0.12378 | -1.5603 | 5502 | |
| Phlda1 | 0.1238 | -0.45609 | 5503 | |
| Zbtb42 | 0.12381 | -0.45185 | 5504 | |
| Rhbdd3 | 0.12384 | -1.0366 | 5505 | |
| Iqgap3 | 0.12388 | -1.2236 | 5506 | |
| Hist2h2be | 0.12389 | -0.35834 | 5507 | |
| Map7d1 | 0.12392 | -0.68468 | 5508 | |
| 1110008J03Rik | 0.12393 | -0.45552 | 5509 | |
| H3f3a | 0.12393 | -1.6366 | 5510 | |
| Esp8 | 0.12396 | -0.68407 | 5511 | |
| Padi6 | 0.12397 | -1.6351 | 5512 | |
| Gpr97 | 0.12401 | -0.66992 | 5513 | |
| Pm20d2 | 0.12409 | -1.6363 | 5514 | |
| Prkcd | 0.12409 | 0.53891 | 5515 | |
| Chmp7 | 0.12413 | -1.5686 | 5516 | |
| mmu-mir-669m-2 | 0.12417 | -1.2057 | 5517 | |
| 3110009E18Rik | 0.12419 | -0.30122 | 5518 | |
| Ubp1 | 0.12419 | -1.3619 | 5519 | |
| Dlx4 | 0.12421 | -0.23865 | 5520 | |
| Farsa | 0.12426 | -1.3777 | 5521 | |
| Gm20604 | 0.12428 | -2.4008 | 5522 | |
| Col6a2 | 0.1243 | 0.85937 | 5523 | |
| Lrrc28 | 0.12432 | -1.6357 | 5524 | |
| Inpp4a | 0.12433 | -1.635 | 5525 | |
| Pkdcc | 0.12434 | -0.57353 | 5526 | |
| Adcy3 | 0.12437 | -1.577 | 5527 | |
| Zufsp | 0.1244 | -1.0789 | 5528 | |
| Sgcz | 0.12444 | -1.6289 | 5529 | |
| Dap | 0.12446 | -0.3413 | 5530 | |
| Olfr444 | 0.12446 | -0.48193 | 5531 | |
| Rassf3 | 0.12446 | -0.12923 | 5532 | |
| Gp1bb | 0.12448 | -1.6351 | 5533 | |
| Olfr231 | 0.12451 | -1.1339 | 5534 | |
| Adcy6 | 0.12453 | -1.6282 | 5535 | |
| Akip1 | 0.12455 | -0.45295 | 5536 | |
| Rnft2 | 0.12457 | -0.31081 | 5537 | |
| Gm15091 | 0.12459 | -0.32413 | 5538 | |
| Pdhx | 0.12459 | -0.021443 | 5539 | |
| Pcdh19 | 0.12465 | -1.6325 | 5540 | |
| Stx4a | 0.12465 | -0.85513 | 5541 | |
| Cbx5 | 0.12467 | -0.57939 | 5542 | |
| Sdr39u1 | 0.12468 | -0.64093 | 5543 | |
| Fam63b | 0.12471 | -1.6315 | 5544 | |
| Adam10 | 0.12471 | 0.17774 | 5545 | |
| Setd3 | 0.12473 | -0.73758 | 5546 | |
| Hmx3 | 0.12474 | -0.66839 | 5547 | |
| Zfp609 | 0.12475 | -1.2076 | 5548 | |
| Osbpl2 | 0.12483 | -0.66517 | 5549 | |
| Clec2i | 0.12483 | -0.4306 | 5550 | |
| Ppid | 0.12487 | -1.2018 | 5551 | |
| Zkscan2 | 0.12491 | -0.43723 | 5552 | |
| Cox15 | 0.12495 | -0.56522 | 5553 | |
| Galt | 0.12497 | -0.61973 | 5554 | |
| Ahctf1 | 0.12502 | -1.6195 | 5555 | |
| mmu-mir-599 | 0.12503 | 0.14252 | 5556 | |
| Ppp2ca | 0.12505 | -0.86098 | 5557 | |
| Vmn1r104 | 0.12505 | 0.41414 | 5558 | |
| Gfi1b | 0.12508 | -0.37803 | 5559 | |
| Nmrk2 | 0.12513 | -1.0632 | 5560 | |
| Plekhg3 | 0.12525 | -0.029495 | 5561 | |
| Rccd1 | 0.1253 | -1.0409 | 5562 | |
| Mrps12 | 0.12532 | -1.3423 | 5563 | |
| Fxyd7 | 0.12536 | -1.6285 | 5564 | |
| 1700016C15Rik | 0.12538 | -1.63 | 5565 | |
| Frrs1 | 0.12538 | -0.13986 | 5566 | |
| Ubtd1 | 0.12542 | -0.80884 | 5567 | |
| Wisp1 | 0.12544 | -1.6255 | 5568 | |
| Cluap1 | 0.12546 | -0.49427 | 5569 | |
| Asb8 | 0.12547 | -1.6308 | 5570 | |
| Reg4 | 0.12552 | -0.092628 | 5571 | |
| mmu-mir-7035 | 0.12554 | -0.22655 | 5572 | |
| Bloc1s5 | 0.12557 | 0.36172 | 5573 | |
| Ctsb | 0.12557 | -1.6292 | 5574 | |
| mmu-mir-133c | 0.1256 | -1.4677 | 5575 | |
| Cetn2 | 0.12563 | -1.6287 | 5576 | |
| Pdcd6ip | 0.12571 | -0.70282 | 5577 | |
| 2700081O15Rik | 0.12587 | -1.6289 | 5578 | |
| Rasgrp2 | 0.1259 | 0.63044 | 5579 | |
| Dlst | 0.12598 | -1.6239 | 5580 | |
| Vmn2r3 | 0.12601 | 0.35369 | 5581 | |
| Olfr683 | 0.12607 | -0.67869 | 5582 | |
| Tdpoz1 | 0.12621 | 0.92082 | 5583 | |
| Vmn1r81 | 0.12625 | 1.4996 | 5584 | |
| Kazald1 | 0.12634 | -0.59194 | 5585 | |
| Atg2b | 0.12646 | -0.78449 | 5586 | |
| Cd80 | 0.12647 | -1.5997 | 5587 | |
| Gm514 | 0.1265 | 0.51623 | 5588 | |
| Sall3 | 0.12656 | -0.80289 | 5589 | |
| Myog | 0.12671 | -0.9021 | 5590 | |
| Ip6k2 | 0.12684 | 0.021397 | 5591 | |
| Anapc7 | 0.12684 | -1.6242 | 5592 | |
| Smyd4 | 0.12691 | -1.5021 | 5593 | |
| Pcdha6 | 0.12693 | -1.641 | 5594 | |
| Ddx28 | 0.12704 | -0.76373 | 5595 | |
| mmu-mir-467e | 0.12705 | -1.3785 | 5596 | |
| Ntmt1 | 0.12717 | 0.31641 | 5597 | |
| Fam194a | 0.12725 | -0.71934 | 5598 | |
| Nphs1 | 0.12725 | -1.6226 | 5599 | |
| Glyctk | 0.12729 | -1.6161 | 5600 | |
| Ildr1 | 0.12732 | -0.59586 | 5601 | |
| Bckdha | 0.1274 | -1.2409 | 5602 | |
| Rap2c | 0.12749 | -1.6215 | 5603 | |
| Copg2 | 0.12754 | -1.2349 | 5604 | |
| Itga1 | 0.12754 | -0.99157 | 5605 | |
| Phykpl | 0.12755 | -1.5033 | 5606 | |
| Spata31d1c | 0.12767 | 1.2597 | 5607 | |
| Cmtm6 | 0.1277 | -1.6148 | 5608 | |
| Vmn1r135 | 0.12782 | -1.9572 | 5609 | |
| Pcyox1 | 0.12783 | -0.66324 | 5610 | |
| BC022687 | 0.12811 | -1.6179 | 5611 | |
| Urm1 | 0.12814 | -1.6135 | 5612 | |
| Rasgrf2 | 0.12817 | -1.6176 | 5613 | |
| mmu-mir-3090 | 0.12826 | -0.56118 | 5614 | |
| Pdx1 | 0.12827 | -0.35389 | 5615 | |
| Pold1 | 0.12829 | -0.7512 | 5616 | |
| Usp9y | 0.12829 | 1.9771 | 5617 | |
| Trim12a | 0.12829 | -1.1197 | 5618 | |
| Popdc2 | 0.12833 | -1.6175 | 5619 | |
| Lrrc6 | 0.12836 | -1.6139 | 5620 | |
| Hsd3b3 | 0.12838 | -1.6156 | 5621 | |
| Olfr525 | 0.12842 | -1.4741 | 5622 | |
| Flrt3 | 0.12852 | -1.0566 | 5623 | |
| Cypt3 | 0.12853 | -1.6147 | 5624 | |
| Crtap | 0.12857 | -1.6082 | 5625 | |
| Rhox4g | 0.12865 | -1.9431 | 5626 | |
| Acap3 | 0.12866 | -0.0896 | 5627 | |
| Arid4b | 0.12875 | -0.24295 | 5628 | |
| Gm17019 | 0.12879 | -1.0033 | 5629 | |
| Nxph2 | 0.12882 | -1.6152 | 5630 | |
| Vmn2r56 | 0.12883 | -1.1106 | 5631 | |
| Hrasls | 0.12883 | -1.1342 | 5632 | |
| Olfr90 | 0.12884 | -1.613 | 5633 | |
| Cd47 | 0.12896 | -1.5761 | 5634 | |
| Caprin1 | 0.12896 | -1.6145 | 5635 | |
| Fech | 0.129 | -1.0253 | 5636 | |
| Gm5414 | 0.129 | -1.6146 | 5637 | |
| Pla2g4d | 0.12912 | -0.024918 | 5638 | |
| Lilra5 | 0.1292 | -1.599 | 5639 | |
| 1700067K01Rik | 0.1292 | 0.047258 | 5640 | |
| Scgb2b3 | 0.12927 | 1.1282 | 5641 | |
| Lima1 | 0.12928 | -1.6099 | 5642 | |
| Tmem18 | 0.12933 | -0.1997 | 5643 | |
| Lrg1 | 0.12934 | -1.6125 | 5644 | |
| Ifnab | 0.12938 | -1.6123 | 5645 | |
| Tmem203 | 0.12941 | -1.6127 | 5646 | |
| Ubxn2a | 0.1295 | 1.9461 | 5647 | |
| Tekt2 | 0.12954 | -1.6109 | 5648 | |
| Hsd17b12 | 0.12963 | -1.596 | 5649 | |
| Olfr1043 | 0.12965 | -1.6119 | 5650 | |
| Ppm1e | 0.12966 | -0.093412 | 5651 | |
| Tbl2 | 0.1297 | -0.73238 | 5652 | |
| Fam154b | 0.12971 | -1.6102 | 5653 | |
| Gimap3 | 0.12974 | 1.0513 | 5654 | |
| Ergic2 | 0.12976 | -0.3472 | 5655 | |
| Lpcat2b | 0.1298 | -0.95044 | 5656 | |
| Olfr1246 | 0.12981 | -0.60972 | 5657 | |
| Ero1l | 0.12987 | -0.54719 | 5658 | |
| Hcst | 0.1299 | -0.525 | 5659 | |
| Gm5635 | 0.12993 | -1.6105 | 5660 | |
| Haus5 | 0.12995 | -1.6106 | 5661 | |
| Fbxo31 | 0.12999 | 0.93653 | 5662 | |
| Gm10922 | 0.13003 | -2.3386 | 5663 | |
| Grem2 | 0.13008 | -1.2003 | 5664 | |
| Sde2 | 0.13012 | 0.24122 | 5665 | |
| Tap1 | 0.13013 | -1.1197 | 5666 | |
| Gria3 | 0.13016 | -0.67513 | 5667 | |
| mmu-mir-6927 | 0.13018 | -0.95696 | 5668 | |
| Ak6 | 0.13022 | -1.1143 | 5669 | |
| Pdgfrl | 0.13022 | -1.1281 | 5670 | |
| Tnnc1 | 0.13028 | 0.50569 | 5671 | |
| Nudcd2 | 0.13031 | -1.606 | 5672 | |
| Ankrd13c | 0.13033 | -0.73144 | 5673 | |
| Olfr1373 | 0.13041 | 0.043044 | 5674 | |
| Slurp1 | 0.13043 | -0.98429 | 5675 | |
| Map3k6 | 0.13045 | 0.30845 | 5676 | |
| Gsdmc3 | 0.13047 | -1.2827 | 5677 | |
| Ubap2 | 0.13049 | -0.7838 | 5678 | |
| Antxrl | 0.13051 | -1.607 | 5679 | |
| Foxi3 | 0.13054 | -1.4176 | 5680 | |
| Hspa13 | 0.13055 | -1.6077 | 5681 | |
| Hist1h4j | 0.13066 | -1.0916 | 5682 | |
| 1700008F21Rik | 0.1307 | -1.4474 | 5683 | |
| Pnmal1 | 0.1308 | -1.6038 | 5684 | |
| Olfr829 | 0.13084 | -1.6063 | 5685 | |
| Limch1 | 0.1309 | -1.6049 | 5686 | |
| Nploc4 | 0.13098 | -1.5242 | 5687 | |
| Lrrc69 | 0.13103 | -0.21772 | 5688 | |
| Ppp1r9b | 0.1311 | -1.604 | 5689 | |
| Pcdhb10 | 0.13115 | 0.0089203 | 5690 | |
| 4833420G17Rik | 0.13118 | -1.5698 | 5691 | |
| Zfp607 | 0.13124 | -1.1293 | 5692 | |
| Alb | 0.13128 | 0.92665 | 5693 | |
| BC030336 | 0.13132 | -1.6027 | 5694 | |
| Enpp7 | 0.13132 | 3.2603 | 5695 | |
| Asic2 | 0.1314 | -0.17596 | 5696 | |
| Stx19 | 0.13153 | 0.80759 | 5697 | |
| Tmsb10 | 0.13153 | -1.5952 | 5698 | |
| Fam214a | 0.13161 | -1.4769 | 5699 | |
| Cep350 | 0.13162 | -1.6006 | 5700 | |
| Cnot7 | 0.13162 | -1.6006 | 5701 | |
| Gm572 | 0.13172 | -1.6015 | 5702 | |
| Rps9 | 0.13173 | -0.25069 | 5703 | |
| Ccdc68 | 0.13177 | -1.6019 | 5704 | |
| Clca6 | 0.13186 | -0.030155 | 5705 | |
| F9 | 0.13188 | -0.66859 | 5706 | |
| 1700018F24Rik | 0.13196 | -1.6008 | 5707 | |
| BC018242 | 0.13201 | -1.3155 | 5708 | |
| mmu-mir-1958 | 0.13202 | -1.5948 | 5709 | |
| Sec13 | 0.13202 | -0.72465 | 5710 | |
| Senp5 | 0.13202 | -0.88753 | 5711 | |
| Olfr1080 | 0.13202 | 1.4952 | 5712 | |
| mmu-mir-877 | 0.13225 | -0.69499 | 5713 | |
| Olfr1487 | 0.13231 | 0.33131 | 5714 | |
| Trip11 | 0.13236 | -0.069576 | 5715 | |
| Ntpcr | 0.13242 | -1.5977 | 5716 | |
| Mpp6 | 0.13248 | -0.7213 | 5717 | |
| Nkapl | 0.1325 | -1.5954 | 5718 | |
| mmu-mir-6918 | 0.1327 | -0.89332 | 5719 | |
| Olfr1257 | 0.1327 | -0.18009 | 5720 | |
| Gsdmc | 0.13274 | -1.5967 | 5721 | |
| Brinp3 | 0.13278 | -1.2937 | 5722 | |
| Mettl9 | 0.13286 | -1.5654 | 5723 | |
| Epyc | 0.13287 | -1.5857 | 5724 | |
| Olfr1396 | 0.13298 | -1.5068 | 5725 | |
| mmu-mir-6541 | 0.13299 | -0.76872 | 5726 | |
| Ctdsp2 | 0.13302 | -1.5965 | 5727 | |
| Mkl2 | 0.13304 | -1.1753 | 5728 | |
| Ids | 0.13304 | -1.0067 | 5729 | |
| Gm826 | 0.13305 | -1.1718 | 5730 | |
| Alkbh3 | 0.1331 | -0.76477 | 5731 | |
| Cdh1 | 0.1332 | -0.87807 | 5732 | |
| Ippk | 0.13323 | -0.86253 | 5733 | |
| Olfr703 | 0.1333 | -1.5939 | 5734 | |
| Trim36 | 0.13333 | -1.0384 | 5735 | |
| Igfn1 | 0.13333 | -1.5924 | 5736 | |
| Olfr478 | 0.13335 | -1.4663 | 5737 | |
| mmu-mir-669c | 0.13336 | -1.1266 | 5738 | |
| Znrf2 | 0.13339 | 0.10245 | 5739 | |
| Osbpl9 | 0.13343 | -0.76397 | 5740 | |
| Rbm31y | 0.13353 | -1.2873 | 5741 | |
| Thap1 | 0.13356 | -1.0385 | 5742 | |
| Runx1t1 | 0.13364 | 0.17722 | 5743 | |
| BB287469 | 0.13366 | -1.5845 | 5744 | |
| mmu-mir-30b | 0.1337 | 0.092272 | 5745 | |
| Creb3l2 | 0.1338 | 0.82778 | 5746 | |
| Hes1 | 0.1339 | -1.5914 | 5747 | |
| Calm5 | 0.13392 | -0.61675 | 5748 | |
| Krt72 | 0.13393 | -1.0195 | 5749 | |
| Fstl5 | 0.13401 | 0.063207 | 5750 | |
| Slc34a3 | 0.13401 | -1.586 | 5751 | |
| Dpp10 | 0.13405 | -1.4639 | 5752 | |
| Acbd5 | 0.13405 | -0.14239 | 5753 | |
| Arhgap25 | 0.13409 | -1.1318 | 5754 | |
| Caps2 | 0.13413 | -0.29678 | 5755 | |
| Rab2b | 0.13414 | -1.3611 | 5756 | |
| Unk | 0.13422 | -1.2756 | 5757 | |
| mmu-mir-7680 | 0.13423 | -0.59538 | 5758 | |
| Parvb | 0.13426 | 0.45955 | 5759 | |
| Olfr654 | 0.13427 | -1.5891 | 5760 | |
| Inf2 | 0.1343 | -0.29021 | 5761 | |
| Syap1 | 0.13437 | -1.5789 | 5762 | |
| Slc1a5 | 0.13439 | -1.5866 | 5763 | |
| Pias2 | 0.13442 | -0.20929 | 5764 | |
| Pcdhgb5 | 0.13443 | -1.8993 | 5765 | |
| Fam222a | 0.13446 | -0.32169 | 5766 | |
| Prl7d1 | 0.13447 | 0.93424 | 5767 | |
| Gm3402 | 0.13449 | -1.7124 | 5768 | |
| mmu-mir-3069 | 0.13463 | 1.6538 | 5769 | |
| Olfr366 | 0.13464 | -0.39714 | 5770 | |
| Grpel1 | 0.13473 | -1.3034 | 5771 | |
| Rnf223 | 0.13477 | -1.5841 | 5772 | |
| Asf1b | 0.135 | -1.5811 | 5773 | |
| Rinl | 0.13511 | -0.35573 | 5774 | |
| Mybbp1a | 0.13511 | -0.91173 | 5775 | |
| Sae1 | 0.13511 | -1.5799 | 5776 | |
| Mcts2 | 0.13516 | -1.5492 | 5777 | |
| Tead2 | 0.13517 | -1.2433 | 5778 | |
| Wfdc15b | 0.13522 | -1.5833 | 5779 | |
| 1700001P01Rik | 0.13526 | -1.5838 | 5780 | |
| Chfr | 0.1353 | -1.1995 | 5781 | |
| Olfr1219 | 0.13531 | -0.2412 | 5782 | |
| Cxxc4 | 0.1355 | -1.5757 | 5783 | |
| Duox1 | 0.13554 | -0.081766 | 5784 | |
| Puf60 | 0.13556 | -1.5812 | 5785 | |
| Zmat5 | 0.13566 | -1.5797 | 5786 | |
| Gm5294 | 0.13568 | -0.88048 | 5787 | |
| Prl7a2 | 0.13573 | -1.5819 | 5788 | |
| Rhebl1 | 0.13582 | -1.5792 | 5789 | |
| Apol10a | 0.13583 | -0.50846 | 5790 | |
| Shkbp1 | 0.13589 | -1.5804 | 5791 | |
| Aqp4 | 0.13592 | -1.423 | 5792 | |
| Calm1 | 0.13594 | -1.5762 | 5793 | |
| Rnf2 | 0.13622 | -0.038162 | 5794 | |
| mmu-mir-5136 | 0.13626 | -0.45391 | 5795 | |
| Olfr1501 | 0.13628 | -1.573 | 5796 | |
| C1qa | 0.13635 | -1.2957 | 5797 | |
| Trf | 0.13635 | -1.0952 | 5798 | |
| Olfr237-ps1 | 0.13637 | -1.5687 | 5799 | |
| Txndc9 | 0.13645 | 0.25045 | 5800 | |
| Lamp2 | 0.13645 | -0.47322 | 5801 | |
| 1700003E16Rik | 0.13649 | -1.5744 | 5802 | |
| Vsig1 | 0.13653 | -1.1576 | 5803 | |
| Plcb2 | 0.13657 | 0.43477 | 5804 | |
| Spaca3 | 0.13661 | -0.20975 | 5805 | |
| Ccnjl | 0.13665 | -1.5623 | 5806 | |
| Ssmem1 | 0.13665 | -0.82971 | 5807 | |
| Olfr922 | 0.1367 | -0.54752 | 5808 | |
| Pbx4 | 0.13678 | -1.576 | 5809 | |
| Esp6-esp5 | 0.13683 | -1.8771 | 5810 | |
| Nms | 0.13686 | -0.26083 | 5811 | |
| Msto1 | 0.13692 | -1.559 | 5812 | |
| Rnf24 | 0.13694 | 0.53248 | 5813 | |
| Ucp2 | 0.13698 | -0.4046 | 5814 | |
| Atp5e | 0.13703 | -0.066179 | 5815 | |
| Olfr1044 | 0.13706 | -0.0029729 | 5816 | |
| Clk1 | 0.13707 | -1.3262 | 5817 | |
| Lpin2 | 0.13715 | -0.3007 | 5818 | |
| Opn1sw | 0.1372 | -1.5737 | 5819 | |
| 1110001J03Rik | 0.13722 | -1.567 | 5820 | |
| Ccnb1 | 0.13725 | -0.98976 | 5821 | |
| Serbp1 | 0.13732 | -1.5697 | 5822 | |
| Mki67 | 0.13738 | -1.5728 | 5823 | |
| mmu-mir-3106 | 0.13739 | -1.1298 | 5824 | |
| Abcc2 | 0.1374 | 0.11603 | 5825 | |
| Rbm28 | 0.13745 | -1.5721 | 5826 | |
| Cend1 | 0.13746 | -0.8604 | 5827 | |
| Uqcc1 | 0.13752 | 0.70747 | 5828 | |
| Npas2 | 0.13754 | -1.5717 | 5829 | |
| Synb | 0.13756 | -1.5716 | 5830 | |
| Atoh1 | 0.13764 | -0.60247 | 5831 | |
| Ino80b | 0.13766 | -1.5711 | 5832 | |
| Cecr5 | 0.13775 | -1.569 | 5833 | |
| Ntf3 | 0.13779 | -1.5698 | 5834 | |
| Dvl3 | 0.13789 | -1.0962 | 5835 | |
| Slc9a3r2 | 0.13789 | -1.0483 | 5836 | |
| Ttc5 | 0.13797 | 0.025283 | 5837 | |
| Gcnt2 | 0.13808 | -1.5682 | 5838 | |
| Glt28d2 | 0.1381 | -0.90473 | 5839 | |
| Clint1 | 0.13814 | 0.76549 | 5840 | |
| Efr3a | 0.1384 | -1.5673 | 5841 | |
| Gabrg3 | 0.1384 | -1.5673 | 5842 | |
| mmu-mir-3072 | 0.13846 | 0.13088 | 5843 | |
| Zbtb32 | 0.1386 | -1.5658 | 5844 | |
| Ubl5 | 0.13861 | -0.96538 | 5845 | |
| Sec61g | 0.13861 | 0.51266 | 5846 | |
| Gm16432 | 0.13861 | 0.2094 | 5847 | |
| Gpatch3 | 0.13867 | -0.58273 | 5848 | |
| Ly86 | 0.13877 | -1.4938 | 5849 | |
| Flcn | 0.13878 | -0.24836 | 5850 | |
| Sord | 0.13883 | -0.81589 | 5851 | |
| Slc37a1 | 0.13893 | -0.56759 | 5852 | |
| Dnajc11 | 0.13899 | -1.539 | 5853 | |
| mmu-mir-8096 | 0.13906 | -0.80411 | 5854 | |
| Pcyt2 | 0.13911 | -0.89344 | 5855 | |
| Slc9a9 | 0.13911 | -0.57598 | 5856 | |
| Abhd13 | 0.13917 | -1.2484 | 5857 | |
| Eif1a | 0.13925 | -0.70882 | 5858 | |
| Rock1 | 0.13935 | -1.4836 | 5859 | |
| Dld | 0.13942 | 0.030113 | 5860 | |
| mmu-mir-688 | 0.1395 | -1.053 | 5861 | |
| Nudt16l1 | 0.13954 | -1.2276 | 5862 | |
| Sntn | 0.13962 | -1.362 | 5863 | |
| Efna3 | 0.13968 | -1.5558 | 5864 | |
| Galnt10 | 0.13971 | 1.0466 | 5865 | |
| Tctex1d1 | 0.13991 | -0.5398 | 5866 | |
| 1810055G02Rik | 0.13999 | -0.75351 | 5867 | |
| Ccdc11 | 0.14001 | -1.5589 | 5868 | |
| Tmem134 | 0.14005 | -1.5583 | 5869 | |
| Zfp72 | 0.14012 | -0.87476 | 5870 | |
| 1110058L19Rik | 0.14013 | -1.5566 | 5871 | |
| Epha7 | 0.14016 | 0.036339 | 5872 | |
| Tgfb1 | 0.14022 | 0.63241 | 5873 | |
| Coa3 | 0.14022 | -1.3185 | 5874 | |
| Trrap | 0.14028 | -1.5468 | 5875 | |
| Mcm3ap | 0.14032 | -0.19197 | 5876 | |
| mmu-mir-7676-2 | 0.14036 | -1.2538 | 5877 | |
| Sf3b3 | 0.14036 | -0.86096 | 5878 | |
| Adam4 | 0.1404 | -0.43185 | 5879 | |
| Olfr715 | 0.14044 | -1.5302 | 5880 | |
| Trit1 | 0.14047 | -1.3724 | 5881 | |
| Zfp213 | 0.1405 | -1.5527 | 5882 | |
| Actl10 | 0.14053 | 0.45069 | 5883 | |
| Prkag3 | 0.14065 | -1.4059 | 5884 | |
| Rhox6 | 0.14069 | -1.5544 | 5885 | |
| mmu-mir-669a-1 | 0.14076 | -2.2247 | 5886 | |
| Tacr1 | 0.14076 | -1.5555 | 5887 | |
| Stat5a | 0.14086 | -0.39606 | 5888 | |
| Olfr485 | 0.14093 | -0.19346 | 5889 | |
| mmu-mir-411 | 0.14093 | -1.1948 | 5890 | |
| Csrnp1 | 0.14098 | 1.0613 | 5891 | |
| Zfp317 | 0.1411 | -0.41785 | 5892 | |
| Olfr345 | 0.14111 | -1.5511 | 5893 | |
| Etfa | 0.14115 | -0.82977 | 5894 | |
| Olfr549 | 0.14116 | -1.5528 | 5895 | |
| Tesc | 0.14119 | -1.5534 | 5896 | |
| Tnk2 | 0.14122 | -1.5468 | 5897 | |
| Cts7 | 0.14128 | -1.5526 | 5898 | |
| Ubac1 | 0.14128 | -1.5216 | 5899 | |
| Decr1 | 0.14131 | -0.94958 | 5900 | |
| Myo10 | 0.14132 | -1.5494 | 5901 | |
| mmu-mir-7226 | 0.14133 | -1.2918 | 5902 | |
| Pcolce | 0.14142 | -0.54729 | 5903 | |
| Chek2 | 0.14145 | -0.58319 | 5904 | |
| Slc35g2 | 0.14145 | -1.1861 | 5905 | |
| Mylk4 | 0.1415 | -1.3766 | 5906 | |
| Olfr594 | 0.14152 | -0.57834 | 5907 | |
| 3110079O15Rik | 0.14153 | -1.5508 | 5908 | |
| 1810010H24Rik | 0.14155 | -1.548 | 5909 | |
| Ywhaz | 0.14156 | 0.36418 | 5910 | |
| Cenpq | 0.14162 | -1.4888 | 5911 | |
| Zfp429 | 0.14164 | -0.5526 | 5912 | |
| mmu-mir-7008 | 0.14166 | -0.85773 | 5913 | |
| Sel1l2 | 0.14167 | -1.0591 | 5914 | |
| Mrpl18 | 0.1417 | -1.5388 | 5915 | |
| H2-DMa | 0.1418 | -1.5347 | 5916 | |
| Ccdc9 | 0.14193 | -1.5466 | 5917 | |
| Sucla2 | 0.14201 | -1.5479 | 5918 | |
| Hspa5 | 0.14211 | -0.71893 | 5919 | |
| mmu-mir-1191b | 0.14211 | -0.1025 | 5920 | |
| Vmn1r227 | 0.14211 | -1.4036 | 5921 | |
| Rnps1 | 0.14211 | -1.1134 | 5922 | |
| Tead1 | 0.14216 | -1.0261 | 5923 | |
| 0610009O20Rik | 0.14219 | -0.14712 | 5924 | |
| Rsph6a | 0.14244 | -1.0858 | 5925 | |
| Lcn9 | 0.14264 | -1.5436 | 5926 | |
| Mrpl30 | 0.14265 | -1.1908 | 5927 | |
| Gnas | 0.14272 | -1.5419 | 5928 | |
| mmu-mir-6403 | 0.14274 | -0.18107 | 5929 | |
| Itgb6 | 0.14275 | 0.62223 | 5930 | |
| Mndal | 0.14275 | -0.55333 | 5931 | |
| 1700056E22Rik | 0.14277 | -1.5438 | 5932 | |
| Slc26a6 | 0.14279 | 0.33705 | 5933 | |
| Hspa9 | 0.14281 | -1.5016 | 5934 | |
| 9-Mar | 0.14287 | -1.5408 | 5935 | |
| Lpar1 | 0.14289 | 0.12055 | 5936 | |
| Zfp462 | 0.1429 | -1.5431 | 5937 | |
| Ctrl | 0.14312 | -0.33606 | 5938 | |
| Ehmt2 | 0.14318 | 0.66805 | 5939 | |
| Rsu1 | 0.14323 | -1.5357 | 5940 | |
| Vmn1r84 | 0.14324 | -1.5304 | 5941 | |
| Car8 | 0.14328 | -1.5416 | 5942 | |
| Olfr457 | 0.14345 | -0.90525 | 5943 | |
| Olfr1206 | 0.14346 | -1.3827 | 5944 | |
| AI118078 | 0.14349 | 2.0855 | 5945 | |
| Cdx4 | 0.14353 | -0.084103 | 5946 | |
| Hist1h1a | 0.14355 | -0.23629 | 5947 | |
| Olfr59 | 0.14357 | -0.84265 | 5948 | |
| mmu-mir-421 | 0.1436 | -0.75273 | 5949 | |
| Fcer1g | 0.14361 | -0.9581 | 5950 | |
| Gm5128 | 0.14362 | -1.4222 | 5951 | |
| Ndp | 0.14363 | -0.67893 | 5952 | |
| Plet1 | 0.14374 | -1.092 | 5953 | |
| mmu-mir-7010 | 0.14377 | -0.49704 | 5954 | |
| Psg20 | 0.14377 | -1.16 | 5955 | |
| Sirt7 | 0.14379 | -1.5371 | 5956 | |
| Pla2g3 | 0.14386 | -0.57029 | 5957 | |
| Cdh23 | 0.14394 | 0.6241 | 5958 | |
| Aldh9a1 | 0.14396 | -1.3896 | 5959 | |
| Pla1a | 0.14401 | -1.5039 | 5960 | |
| Rerg | 0.14403 | -1.5366 | 5961 | |
| Timmdc1 | 0.14424 | -1.5344 | 5962 | |
| Gad1 | 0.14427 | -0.23475 | 5963 | |
| Abhd8 | 0.14431 | 0.15353 | 5964 | |
| Pxdc1 | 0.14443 | -0.11587 | 5965 | |
| Fam107b | 0.14447 | -1.2198 | 5966 | |
| Vmn1r207-ps | 0.14451 | -0.22648 | 5967 | |
| Brwd3 | 0.14461 | -1.5336 | 5968 | |
| Stx17 | 0.14471 | -1.5272 | 5969 | |
| Cirbp | 0.14476 | -1.5334 | 5970 | |
| Slc35e3 | 0.14476 | 1.2309 | 5971 | |
| Zfp385c | 0.14482 | -1.4053 | 5972 | |
| Ppp1ca | 0.14492 | -0.71382 | 5973 | |
| Nrsn2 | 0.14495 | -0.53272 | 5974 | |
| Krt5 | 0.14513 | 0.15117 | 5975 | |
| Nfu1 | 0.14517 | -1.5172 | 5976 | |
| Ikzf5 | 0.14525 | -1.031 | 5977 | |
| Olfr1502 | 0.14525 | -1.4835 | 5978 | |
| Larp1 | 0.14529 | -0.57979 | 5979 | |
| Slc25a20 | 0.14537 | -0.71824 | 5980 | |
| Ywhah | 0.14542 | -0.27111 | 5981 | |
| Lyz1 | 0.14542 | -1.0879 | 5982 | |
| Zdhhc20 | 0.14544 | -0.97551 | 5983 | |
| Slc50a1 | 0.14563 | -1.5287 | 5984 | |
| Abca8a | 0.14565 | -0.42231 | 5985 | |
| Gm14393 | 0.14566 | -1.0493 | 5986 | |
| Fam212b | 0.1457 | -0.53012 | 5987 | |
| Olfr395 | 0.14575 | -1.5071 | 5988 | |
| Slc6a13 | 0.14579 | -1.1659 | 5989 | |
| Mlf1 | 0.14584 | -1.5274 | 5990 | |
| Utp3 | 0.14585 | -0.54335 | 5991 | |
| Trp53bp2 | 0.14587 | -1.5278 | 5992 | |
| Nek2 | 0.14599 | 0.48144 | 5993 | |
| Dhodh | 0.14602 | -1.5277 | 5994 | |
| Mrpl43 | 0.14622 | -1.5198 | 5995 | |
| Olfr98 | 0.14625 | -0.20934 | 5996 | |
| 4933430I17Rik | 0.14646 | -1.5259 | 5997 | |
| Ces1d | 0.14648 | -0.96685 | 5998 | |
| Zfp382 | 0.14658 | -0.6337 | 5999 | |
| Kif13a | 0.14658 | -0.92789 | 6000 | |
| Wnt9b | 0.14658 | -1.5241 | 6001 | |
| Cyp2d12 | 0.14667 | -0.22147 | 6002 | |
| Vmn2r118 | 0.14669 | -0.080338 | 6003 | |
| Fermt1 | 0.14672 | -1.5201 | 6004 | |
| Rnaseh1 | 0.14673 | -0.075598 | 6005 | |
| Irak4 | 0.14673 | -1.5245 | 6006 | |
| Rpl10l | 0.14677 | -0.73351 | 6007 | |
| Ophn1 | 0.14678 | -0.15862 | 6008 | |
| Ppp3cb | 0.14679 | -0.12016 | 6009 | |
| Olfr748 | 0.14683 | -1.5174 | 6010 | |
| mmu-mir-7661 | 0.14687 | -0.83527 | 6011 | |
| Dsp | 0.14688 | -1.5243 | 6012 | |
| Vmn1r194 | 0.14689 | 1.1812 | 6013 | |
| Mcur1 | 0.14693 | -0.38622 | 6014 | |
| Pcdhgb8 | 0.14693 | -1.0198 | 6015 | |
| Rassf1 | 0.14697 | -0.1495 | 6016 | |
| Olfr159 | 0.14707 | -1.2411 | 6017 | |
| Gm21950 | 0.14709 | -0.47857 | 6018 | |
| Gtpbp8 | 0.1471 | -1.2264 | 6019 | |
| Wfdc6a | 0.14713 | -0.90216 | 6020 | |
| Eml3 | 0.14715 | -1.1474 | 6021 | |
| Prl8a8 | 0.14717 | -1.508 | 6022 | |
| Gpsm1 | 0.14722 | -1.5034 | 6023 | |
| Def8 | 0.14726 | -0.40307 | 6024 | |
| mmu-mir-5126 | 0.1473 | -1.3118 | 6025 | |
| Olfr192 | 0.1473 | -0.10348 | 6026 | |
| Ulk4 | 0.1473 | -1.5198 | 6027 | |
| Ascl3 | 0.14734 | -0.25058 | 6028 | |
| Asprv1 | 0.14734 | 0.65435 | 6029 | |
| Tpx2 | 0.14738 | -1.3305 | 6030 | |
| Syne3 | 0.14739 | -1.0196 | 6031 | |
| Olfr837 | 0.14742 | -0.34641 | 6032 | |
| AU019823 | 0.14765 | -0.52408 | 6033 | |
| Nr2f2 | 0.1477 | -1.5189 | 6034 | |
| Pth | 0.14771 | -0.082647 | 6035 | |
| Dirc2 | 0.14779 | -1.5011 | 6036 | |
| mmu-mir-146b | 0.14784 | -0.97522 | 6037 | |
| Ppat | 0.14794 | -1.2428 | 6038 | |
| Zbtb48 | 0.14795 | -0.43425 | 6039 | |
| Gm10436 | 0.14804 | 3.3383 | 6040 | |
| Dpp8 | 0.14804 | -0.62748 | 6041 | |
| mmu-mir-5622 | 0.14805 | 0.44474 | 6042 | |
| Tbc1d16 | 0.14828 | -1.153 | 6043 | |
| Faf1 | 0.14829 | -1.5176 | 6044 | |
| Crim1 | 0.14838 | -0.68187 | 6045 | |
| Esrrb | 0.14842 | -1.516 | 6046 | |
| Ube2u | 0.14844 | -0.067559 | 6047 | |
| Crym | 0.14849 | -1.4175 | 6048 | |
| Magix | 0.14856 | -0.58219 | 6049 | |
| Oosp1 | 0.14857 | -0.17224 | 6050 | |
| Bhmt | 0.14861 | 0.74284 | 6051 | |
| Enpp3 | 0.14867 | -0.062382 | 6052 | |
| Bmp8b | 0.14876 | -1.5154 | 6053 | |
| 4933434E20Rik | 0.14877 | -0.40582 | 6054 | |
| Rexo1 | 0.14881 | -0.51149 | 6055 | |
| 8030462N17Rik | 0.14882 | -1.2928 | 6056 | |
| Rmdn3 | 0.14885 | -1.515 | 6057 | |
| Tcf20 | 0.14885 | 1.5719 | 6058 | |
| Jakmip1 | 0.14889 | 0.78294 | 6059 | |
| Tmem68 | 0.14891 | -1.5138 | 6060 | |
| Lamtor5 | 0.14903 | -1.5076 | 6061 | |
| Acox3 | 0.14906 | -1.5144 | 6062 | |
| Olfr1389 | 0.14909 | -0.1251 | 6063 | |
| Sult2b1 | 0.1491 | 0.1213 | 6064 | |
| Tmem14c | 0.14914 | -0.098397 | 6065 | |
| Hcrt | 0.14914 | -1.5069 | 6066 | |
| Tat | 0.14918 | 0.12708 | 6067 | |
| Plcd3 | 0.14919 | -1.4807 | 6068 | |
| Klhl7 | 0.14926 | -1.5102 | 6069 | |
| Ltv1 | 0.1493 | -0.81908 | 6070 | |
| Capns1 | 0.14934 | 0.54026 | 6071 | |
| Vmn1r44 | 0.14939 | -0.057302 | 6072 | |
| Prmt7 | 0.14944 | -0.91957 | 6073 | |
| Mff | 0.14946 | -0.54707 | 6074 | |
| Olfr794 | 0.14947 | 0.13555 | 6075 | |
| Pdss1 | 0.14949 | -0.2892 | 6076 | |
| Oasl2 | 0.14955 | -1.3298 | 6077 | |
| Olfr39 | 0.14963 | 0.13404 | 6078 | |
| Olfr1351 | 0.14984 | -1.5111 | 6079 | |
| mmu-mir-7648 | 0.14986 | -1.1857 | 6080 | |
| 1700034O15Rik | 0.14991 | -1.5058 | 6081 | |
| Tcf12 | 0.14993 | -0.22321 | 6082 | |
| Zbtb43 | 0.15 | -0.3872 | 6083 | |
| Txnl4a | 0.15014 | -0.042167 | 6084 | |
| Uri1 | 0.15014 | -1.2805 | 6085 | |
| 1810030O07Rik | 0.15014 | -0.83032 | 6086 | |
| Capza1 | 0.15015 | -1.495 | 6087 | |
| Slc43a2 | 0.15055 | -1.5079 | 6088 | |
| Cd33 | 0.1506 | -1.5046 | 6089 | |
| Serpine3 | 0.15061 | 0.07327 | 6090 | |
| Huwe1 | 0.15065 | 0.28183 | 6091 | |
| Dgat1 | 0.15069 | 0.26642 | 6092 | |
| Grap | 0.15077 | -0.36167 | 6093 | |
| Adam7 | 0.15081 | -1.1542 | 6094 | |
| Srek1 | 0.15083 | 0.2938 | 6095 | |
| Mctp1 | 0.15083 | -0.52758 | 6096 | |
| mmu-mir-6914 | 0.15092 | -0.72158 | 6097 | |
| Arap2 | 0.15092 | -0.85858 | 6098 | |
| Utp14b | 0.151 | 0.19203 | 6099 | |
| 1700049G17Rik | 0.151 | 1.4139 | 6100 | |
| Chchd3 | 0.15106 | -0.8308 | 6101 | |
| Jade3 | 0.15108 | -1.5056 | 6102 | |
| Gm561 | 0.1511 | 0.67168 | 6103 | |
| Olfr1420 | 0.15113 | -1.227 | 6104 | |
| Ift20 | 0.15116 | -1.5045 | 6105 | |
| Ddi1 | 0.15118 | -0.79639 | 6106 | |
| Samd12 | 0.15119 | -1.5054 | 6107 | |
| Slc22a27 | 0.15122 | -0.18284 | 6108 | |
| Fev | 0.15124 | -0.77742 | 6109 | |
| mmu-mir-1957a | 0.15126 | -0.41361 | 6110 | |
| Cacybp | 0.1513 | 0.052066 | 6111 | |
| Gm17359 | 0.15135 | -0.58707 | 6112 | |
| Gm732 | 0.15147 | -1.0516 | 6113 | |
| Sema4b | 0.1515 | -1.4472 | 6114 | |
| Atp6v1c1 | 0.15157 | -0.87399 | 6115 | |
| Josd1 | 0.15162 | -0.67602 | 6116 | |
| mmu-mir-6937 | 0.15165 | -0.1938 | 6117 | |
| Acaa1b | 0.1517 | -1.1334 | 6118 | |
| Wdr89 | 0.15185 | -0.24895 | 6119 | |
| Col4a1 | 0.15185 | 0.51565 | 6120 | |
| Ncdn | 0.15187 | -0.70489 | 6121 | |
| Dph2 | 0.15189 | -1.5023 | 6122 | |
| mmu-mir-219a-2 | 0.15189 | -0.76959 | 6123 | |
| BC005537 | 0.15192 | -0.10939 | 6124 | |
| E2f2 | 0.15194 | -0.96845 | 6125 | |
| Cdc42se1 | 0.15196 | 1.1401 | 6126 | |
| Mrvi1 | 0.152 | 0.49802 | 6127 | |
| Pdk4 | 0.15204 | -0.44703 | 6128 | |
| Slc6a2 | 0.15216 | -0.73144 | 6129 | |
| Ppm1n | 0.15216 | -0.6562 | 6130 | |
| Fam155a | 0.15229 | -1.4818 | 6131 | |
| mmu-mir-7075 | 0.1523 | -1.082 | 6132 | |
| 4930474N05Rik | 0.15232 | -0.49597 | 6133 | |
| Tmem50b | 0.15236 | -1.4033 | 6134 | |
| Psmb7 | 0.15237 | -0.86663 | 6135 | |
| Map2k4 | 0.15253 | -0.68952 | 6136 | |
| Tcf7 | 0.15253 | -1.4383 | 6137 | |
| Iqcf1 | 0.15257 | -0.45665 | 6138 | |
| mmu-mir-690 | 0.15264 | -1.2498 | 6139 | |
| Zfp358 | 0.15273 | -1.0486 | 6140 | |
| Syt3 | 0.15277 | -0.38713 | 6141 | |
| Lats2 | 0.15277 | -1.4971 | 6142 | |
| Smurf2 | 0.15281 | -0.82833 | 6143 | |
| Acta2 | 0.15293 | -1.199 | 6144 | |
| Vrk3 | 0.15299 | -1.496 | 6145 | |
| Klhl22 | 0.15307 | -0.82464 | 6146 | |
| mmu-mir-466i | 0.1531 | 0.54972 | 6147 | |
| Suv420h2 | 0.15314 | 0.74815 | 6148 | |
| Dffa | 0.15321 | -0.71563 | 6149 | |
| BC005561 | 0.15326 | -0.073575 | 6150 | |
| Slc6a5 | 0.1533 | -0.058127 | 6151 | |
| Zfp654 | 0.15334 | -0.21529 | 6152 | |
| 1700001J03Rik | 0.15338 | 0.083207 | 6153 | |
| Gprasp1 | 0.15338 | -1.4955 | 6154 | |
| Actr6 | 0.15341 | -1.4957 | 6155 | |
| Cpeb2 | 0.15342 | -0.36433 | 6156 | |
| Socs3 | 0.15346 | -0.71684 | 6157 | |
| Olfr281 | 0.15351 | -1.4902 | 6158 | |
| Hectd3 | 0.15363 | -0.35204 | 6159 | |
| Gm5111 | 0.15363 | -0.1381 | 6160 | |
| Adam34 | 0.15367 | 0.25606 | 6161 | |
| Zfp558 | 0.15371 | 0.1946 | 6162 | |
| Hspa12b | 0.15375 | -0.38935 | 6163 | |
| Wnt2 | 0.15379 | 0.82813 | 6164 | |
| Gc | 0.15383 | -0.90084 | 6165 | |
| Lat | 0.15388 | -0.59017 | 6166 | |
| Bag1 | 0.15399 | -0.022227 | 6167 | |
| Olfr1168 | 0.15399 | -1.1827 | 6168 | |
| Rad1 | 0.15424 | -1.1242 | 6169 | |
| 2310061I04Rik | 0.15428 | -0.42753 | 6170 | |
| 4632434I11Rik | 0.15434 | -0.88665 | 6171 | |
| Pigq | 0.15444 | -1.4661 | 6172 | |
| mmu-mir-674 | 0.15449 | -0.93052 | 6173 | |
| Casp8ap2 | 0.15453 | -1.4872 | 6174 | |
| Gm20747 | 0.15455 | -0.50255 | 6175 | |
| Ppp2r2c | 0.15462 | -1.4893 | 6176 | |
| Dlx5 | 0.15468 | -1.4827 | 6177 | |
| Wnk1 | 0.15473 | -0.2813 | 6178 | |
| Pold3 | 0.15473 | -1.4193 | 6179 | |
| Eif4a3 | 0.15473 | -1.2803 | 6180 | |
| Rsl1d1 | 0.15473 | -0.51438 | 6181 | |
| Sin3b | 0.1548 | -0.20355 | 6182 | |
| Ncam2 | 0.15491 | -0.1127 | 6183 | |
| Mllt3 | 0.15497 | -1.4878 | 6184 | |
| Fcrls | 0.15499 | -1.0237 | 6185 | |
| Wap | 0.15501 | -1.4878 | 6186 | |
| Lig3 | 0.15512 | -0.19025 | 6187 | |
| Olfr484 | 0.15516 | -0.30665 | 6188 | |
| Pi4ka | 0.15526 | -0.41419 | 6189 | |
| Spdyb | 0.1553 | -0.78176 | 6190 | |
| Usp44 | 0.15546 | -1.4346 | 6191 | |
| Fabp4 | 0.15547 | -0.25414 | 6192 | |
| Acad12 | 0.1555 | -1.391 | 6193 | |
| Noxo1 | 0.15566 | -0.25802 | 6194 | |
| mmu-mir-203 | 0.15567 | -0.16105 | 6195 | |
| Impdh1 | 0.15569 | -1.4831 | 6196 | |
| Rap1gap2 | 0.15571 | -1.4839 | 6197 | |
| Ptpro | 0.15574 | -0.47788 | 6198 | |
| Dock10 | 0.15578 | -0.20876 | 6199 | |
| Dcaf15 | 0.15579 | -1.4824 | 6200 | |
| Tprn | 0.15581 | -1.4851 | 6201 | |
| Vmn1r205 | 0.15583 | 0.49507 | 6202 | |
| Fancg | 0.15586 | -1.4829 | 6203 | |
| Olfr1042 | 0.15587 | -1.1131 | 6204 | |
| Cftr | 0.15591 | -0.053445 | 6205 | |
| Mrgprb1 | 0.15596 | -1.4831 | 6206 | |
| Wnt9a | 0.15597 | -0.79267 | 6207 | |
| mmu-mir-6958 | 0.15603 | -0.38482 | 6208 | |
| Lrrc2 | 0.15606 | -1.484 | 6209 | |
| Qser1 | 0.15607 | 0.10136 | 6210 | |
| Glud1 | 0.1561 | -0.13862 | 6211 | |
| Wdr96 | 0.15611 | -1.3829 | 6212 | |
| Ctps | 0.15619 | 0.048912 | 6213 | |
| Mad2l2 | 0.15627 | -1.4782 | 6214 | |
| Ccdc84 | 0.15631 | -0.27658 | 6215 | |
| Dpp9 | 0.15634 | -1.4821 | 6216 | |
| mmu-mir-6946 | 0.15634 | -1.4107 | 6217 | |
| Gpr152 | 0.15635 | -1.087 | 6218 | |
| Traf3ip2 | 0.15639 | -0.72448 | 6219 | |
| 1700015F17Rik | 0.15643 | 0.089238 | 6220 | |
| 4922501C03Rik | 0.15647 | -1.4193 | 6221 | |
| Oog4 | 0.15649 | -1.4807 | 6222 | |
| Olfr911-ps1 | 0.15652 | -0.36507 | 6223 | |
| Ephx4 | 0.15664 | -0.08155 | 6224 | |
| Praf2 | 0.15669 | -1.4803 | 6225 | |
| Elmo1 | 0.15678 | -1.4762 | 6226 | |
| Vmn1r26 | 0.1568 | 0.20019 | 6227 | |
| Tm2d1 | 0.15684 | -0.01997 | 6228 | |
| Snx15 | 0.15686 | -1.4715 | 6229 | |
| Nkrf | 0.15688 | 0.43333 | 6230 | |
| Gm14308 | 0.15689 | -1.8712 | 6231 | |
| Slk | 0.15694 | -0.71078 | 6232 | |
| Cwc25 | 0.15697 | -1.1748 | 6233 | |
| Yipf5 | 0.157 | 0.26327 | 6234 | |
| Vmn2r61 | 0.15703 | -1.4756 | 6235 | |
| Prr22 | 0.15704 | 0.28164 | 6236 | |
| Itch | 0.15708 | -0.0042431 | 6237 | |
| Eid3 | 0.15709 | -1.4721 | 6238 | |
| Irx2 | 0.15713 | -0.49097 | 6239 | |
| Olfr799 | 0.15719 | -0.36816 | 6240 | |
| Rpl28 | 0.15719 | -1.4779 | 6241 | |
| P2ry14 | 0.15725 | -0.45922 | 6242 | |
| Dnajc18 | 0.15729 | -1.426 | 6243 | |
| Ccdc88a | 0.15729 | -1.2663 | 6244 | |
| Sycp3 | 0.15736 | -1.459 | 6245 | |
| 1110008L16Rik | 0.15737 | -1.4053 | 6246 | |
| Inpp5f | 0.15743 | -1.3788 | 6247 | |
| Trub2 | 0.15743 | -0.89582 | 6248 | |
| Vmn1r173 | 0.15753 | -1.4769 | 6249 | |
| E2f8 | 0.15769 | -1.4743 | 6250 | |
| Trim60 | 0.15778 | -0.52725 | 6251 | |
| Fgf10 | 0.15781 | -1.1966 | 6252 | |
| Zfp871 | 0.1579 | -0.1784 | 6253 | |
| Vmn2r1 | 0.15798 | 1.0333 | 6254 | |
| mmu-mir-7662 | 0.15804 | -1.1869 | 6255 | |
| Fetub | 0.15805 | -1.4749 | 6256 | |
| Atp7b | 0.15808 | -1.4745 | 6257 | |
| Gm11544 | 0.1581 | -1.4745 | 6258 | |
| Samd14 | 0.15813 | -0.87176 | 6259 | |
| Vmn1r24 | 0.15815 | -0.80262 | 6260 | |
| Vmn2r11 | 0.15822 | -0.8801 | 6261 | |
| 1500009L16Rik | 0.15823 | -1.7872 | 6262 | |
| Zfp959 | 0.15829 | -0.044838 | 6263 | |
| Olfr772 | 0.15834 | -1.4731 | 6264 | |
| mmu-mir-466b-1 | 0.15834 | 1.0459 | 6265 | |
| mmu-mir-7664 | 0.15839 | -0.82345 | 6266 | |
| Zfp612 | 0.15844 | -1.4732 | 6267 | |
| Nfkb2 | 0.15863 | -1.4659 | 6268 | |
| Creb5 | 0.15865 | -1.472 | 6269 | |
| mmu-mir-219b | 0.15875 | -1.3736 | 6270 | |
| Plat | 0.15877 | -1.466 | 6271 | |
| mmu-mir-3544 | 0.1588 | -0.69991 | 6272 | |
| Klhl26 | 0.15882 | -0.60153 | 6273 | |
| Lpo | 0.15891 | -1.4709 | 6274 | |
| mmu-mir-142 | 0.15894 | -1.9921 | 6275 | |
| Olfr390 | 0.15895 | -0.89798 | 6276 | |
| 4932429P05Rik | 0.15899 | 0.92723 | 6277 | |
| Cstb | 0.15903 | 0.12646 | 6278 | |
| Tmem159 | 0.15906 | -0.30246 | 6279 | |
| Wdr44 | 0.15907 | -0.069086 | 6280 | |
| Cacng6 | 0.15911 | -0.23304 | 6281 | |
| Nol12 | 0.15911 | -1.1879 | 6282 | |
| G2e3 | 0.15915 | -0.92787 | 6283 | |
| Mterfd3 | 0.15922 | -0.010083 | 6284 | |
| 1700012B09Rik | 0.15923 | -1.4688 | 6285 | |
| AY358078 | 0.15925 | -1.3437 | 6286 | |
| Inadl | 0.1594 | -0.085332 | 6287 | |
| Eno4 | 0.15942 | -1.4692 | 6288 | |
| Gm15127 | 0.15944 | -1.0283 | 6289 | |
| Stac3 | 0.15948 | -1.3314 | 6290 | |
| 2310036O22Rik | 0.15952 | -0.97561 | 6291 | |
| Arhgap42 | 0.15958 | 0.45571 | 6292 | |
| Tas2r134 | 0.15964 | -0.037646 | 6293 | |
| Helz2 | 0.15968 | 0.015507 | 6294 | |
| Ankrd12 | 0.15969 | -1.4633 | 6295 | |
| mmu-mir-3068 | 0.15978 | -1.0582 | 6296 | |
| Cpm | 0.1598 | 0.016303 | 6297 | |
| Acsm1 | 0.15987 | -1.383 | 6298 | |
| Gsta2 | 0.15988 | -2.0435 | 6299 | |
| Atp5k | 0.16001 | 0.045797 | 6300 | |
| Sass6 | 0.16009 | -0.067824 | 6301 | |
| Prdx4 | 0.16011 | -1.4608 | 6302 | |
| Thumpd1 | 0.16017 | -0.93279 | 6303 | |
| Adap2 | 0.16017 | -1.464 | 6304 | |
| Amigo1 | 0.16021 | -1.4602 | 6305 | |
| Trp53 | 0.16025 | -0.1806 | 6306 | |
| Fam169a | 0.16033 | 0.16971 | 6307 | |
| Fh1 | 0.16037 | -0.45268 | 6308 | |
| Cmtm1 | 0.16037 | -1.4649 | 6309 | |
| Hbb-b1 | 0.16042 | -1.4595 | 6310 | |
| Dnph1 | 0.16047 | -0.96966 | 6311 | |
| Gas2l3 | 0.16047 | -1.3353 | 6312 | |
| Scpep1 | 0.16047 | 0.28317 | 6313 | |
| Olfr970 | 0.16047 | 1.346 | 6314 | |
| Cpne9 | 0.16057 | -1.4612 | 6315 | |
| Mettl15 | 0.16065 | -0.99627 | 6316 | |
| Zcchc18 | 0.16087 | -1.4625 | 6317 | |
| Pax8 | 0.16094 | -0.66796 | 6318 | |
| Gstp1 | 0.16095 | -1.4594 | 6319 | |
| Ndel1 | 0.16097 | -1.4569 | 6320 | |
| Arhgap20 | 0.161 | -1.4578 | 6321 | |
| Olfr702 | 0.16106 | 0.34063 | 6322 | |
| Ifitm5 | 0.16109 | -1.2337 | 6323 | |
| Pgpep1 | 0.1611 | -0.27885 | 6324 | |
| A2m | 0.16118 | -0.90493 | 6325 | |
| Kdm6a | 0.16122 | -1.4583 | 6326 | |
| Kif26b | 0.16126 | -0.081123 | 6327 | |
| Gmeb1 | 0.1613 | -1.0822 | 6328 | |
| Nudt17 | 0.16134 | -1.161 | 6329 | |
| Ybx2 | 0.16138 | -1.0915 | 6330 | |
| 4933403O08Rik | 0.16142 | -1.4601 | 6331 | |
| Pbld2 | 0.16142 | 0.38222 | 6332 | |
| Prpf18 | 0.16144 | -1.1776 | 6333 | |
| Ccng2 | 0.16145 | -0.39335 | 6334 | |
| Gm4559 | 0.16154 | -0.40098 | 6335 | |
| Vmn1r175 | 0.16158 | -1.1667 | 6336 | |
| Nup153 | 0.16167 | -0.83174 | 6337 | |
| Vmn1r94 | 0.16167 | -2.0302 | 6338 | |
| mmu-mir-1904 | 0.16178 | -1.3184 | 6339 | |
| Asb1 | 0.16182 | -0.72916 | 6340 | |
| Il1rap | 0.16183 | -1.0763 | 6341 | |
| BC094916 | 0.16185 | 1.5703 | 6342 | |
| Ptma | 0.16187 | -1.4572 | 6343 | |
| Ttf2 | 0.1619 | -1.4579 | 6344 | |
| Efnb2 | 0.16191 | -0.58089 | 6345 | |
| Pla2g4e | 0.16195 | -0.14503 | 6346 | |
| Ythdc1 | 0.16197 | -1.4441 | 6347 | |
| Vmn2r99 | 0.16213 | 0.095703 | 6348 | |
| Tnrc18 | 0.16216 | -1.456 | 6349 | |
| Tmem27 | 0.16221 | 0.4759 | 6350 | |
| Gpr137 | 0.16235 | -0.85505 | 6351 | |
| Lrrc27 | 0.16237 | -1.4525 | 6352 | |
| Ddx58 | 0.1624 | -0.41882 | 6353 | |
| Mettl22 | 0.16242 | -1.4485 | 6354 | |
| Rgs2 | 0.16244 | -1.4551 | 6355 | |
| Ccdc136 | 0.16248 | -0.73723 | 6356 | |
| Gm6406 | 0.16249 | -0.86576 | 6357 | |
| Plekha6 | 0.16258 | -0.13249 | 6358 | |
| Creb1 | 0.16268 | -0.1629 | 6359 | |
| Sowaha | 0.1627 | -1.4474 | 6360 | |
| Fancm | 0.16279 | -0.49091 | 6361 | |
| Phyhd1 | 0.1628 | 0.82115 | 6362 | |
| Wasf1 | 0.1628 | -0.87777 | 6363 | |
| Lmtk3 | 0.16289 | -1.4524 | 6364 | |
| Tdpoz3 | 0.16292 | -1.0404 | 6365 | |
| mmu-mir-3076 | 0.16297 | -1.1499 | 6366 | |
| mmu-mir-7118 | 0.1631 | -0.86738 | 6367 | |
| Ap1m1 | 0.16313 | -0.34893 | 6368 | |
| mmu-mir-1843b | 0.16315 | -1.0336 | 6369 | |
| Sgpl1 | 0.16318 | -1.4513 | 6370 | |
| Tdrd6 | 0.16329 | -0.33765 | 6371 | |
| Ube2dnl1 | 0.16336 | -1.4484 | 6372 | |
| Wdr43 | 0.16336 | 1.3579 | 6373 | |
| Vmn2r4 | 0.16336 | -0.74923 | 6374 | |
| Mettl6 | 0.16336 | 0.25463 | 6375 | |
| Gak | 0.16336 | -0.8864 | 6376 | |
| Chit1 | 0.16339 | -1.4519 | 6377 | |
| Ccr5 | 0.16341 | -1.4519 | 6378 | |
| Nhsl1 | 0.16355 | -1.4491 | 6379 | |
| Ly6k | 0.16357 | -1.4512 | 6380 | |
| Abcd2 | 0.16372 | -1.4432 | 6381 | |
| Apod | 0.16376 | -1.4479 | 6382 | |
| Evc2 | 0.16383 | -1.4491 | 6383 | |
| Armc5 | 0.16391 | -1.0932 | 6384 | |
| Golga1 | 0.16391 | -0.47242 | 6385 | |
| Olfr1510 | 0.16397 | -1.4492 | 6386 | |
| Wt1 | 0.16397 | -0.23361 | 6387 | |
| Dapk2 | 0.16398 | -1.4474 | 6388 | |
| Pim1 | 0.16405 | -0.70995 | 6389 | |
| Polr2e | 0.16409 | -0.19509 | 6390 | |
| Olfr675 | 0.16413 | -1.3073 | 6391 | |
| Olfr141 | 0.16421 | 0.79873 | 6392 | |
| Asic3 | 0.16421 | 0.65294 | 6393 | |
| Krt2 | 0.16424 | -1.3394 | 6394 | |
| Poldip2 | 0.16429 | -0.39322 | 6395 | |
| Olr1 | 0.16431 | -0.60307 | 6396 | |
| Olfr834 | 0.16433 | -1.0512 | 6397 | |
| Tspan13 | 0.16437 | -1.4448 | 6398 | |
| Cbx1 | 0.16442 | -1.4267 | 6399 | |
| Adat3 | 0.16446 | 1.159 | 6400 | |
| BC051019 | 0.16449 | -1.4422 | 6401 | |
| mmu-mir-7013 | 0.16456 | -0.70825 | 6402 | |
| Ptger1 | 0.16456 | -1.439 | 6403 | |
| Zcchc11 | 0.16462 | 0.22425 | 6404 | |
| Hk1 | 0.16463 | -0.28005 | 6405 | |
| 2010315B03Rik | 0.16466 | -1.4368 | 6406 | |
| Snx4 | 0.16468 | 0.32602 | 6407 | |
| Hells | 0.16474 | 0.12314 | 6408 | |
| Tpp2 | 0.16474 | -1.4403 | 6409 | |
| Psmd7 | 0.16489 | -1.3953 | 6410 | |
| Gnai1 | 0.1649 | -1.2425 | 6411 | |
| Ly6g6d | 0.16494 | -0.067059 | 6412 | |
| Ankrd28 | 0.16501 | -1.2337 | 6413 | |
| Ei24 | 0.16502 | 0.14484 | 6414 | |
| Otogl | 0.16506 | -0.93811 | 6415 | |
| Zfp282 | 0.16518 | -0.3448 | 6416 | |
| mmu-mir-7027 | 0.1652 | -1.0465 | 6417 | |
| Osbpl1a | 0.16525 | -0.30765 | 6418 | |
| Ifitm3 | 0.16526 | -0.90502 | 6419 | |
| Skiv2l2 | 0.1653 | -0.68068 | 6420 | |
| Krtap24-1 | 0.16537 | -0.56831 | 6421 | |
| Azi2 | 0.16543 | -0.34328 | 6422 | |
| Klf7 | 0.16546 | 0.20715 | 6423 | |
| mmu-mir-6382 | 0.16549 | 0.96841 | 6424 | |
| Smim9 | 0.16549 | -1.3972 | 6425 | |
| Ccdc51 | 0.1655 | -1.0802 | 6426 | |
| Rala | 0.16555 | -0.11225 | 6427 | |
| Spink6 | 0.16561 | 0.4222 | 6428 | |
| Tktl2 | 0.16561 | -0.24902 | 6429 | |
| Gpalpp1 | 0.16565 | -0.47403 | 6430 | |
| Olfr297 | 0.16583 | -0.79569 | 6431 | |
| Mrpl46 | 0.16587 | -0.74512 | 6432 | |
| Vmn1r46 | 0.16591 | -0.047337 | 6433 | |
| mmu-mir-5626 | 0.16595 | -0.88429 | 6434 | |
| mmu-mir-26a-1 | 0.16597 | -1.0212 | 6435 | |
| Ccbl2 | 0.16606 | -1.2069 | 6436 | |
| Kdm1a | 0.16607 | -1.3282 | 6437 | |
| Thrap3 | 0.16613 | -1.4406 | 6438 | |
| AI314180 | 0.16615 | 0.68524 | 6439 | |
| Olfr700 | 0.16639 | -0.51085 | 6440 | |
| 4933411K20Rik | 0.16642 | -1.431 | 6441 | |
| Gm5346 | 0.16644 | -1.4383 | 6442 | |
| Olfr663 | 0.16647 | 0.57515 | 6443 | |
| Ywhaq | 0.16647 | 1.6338 | 6444 | |
| Il23a | 0.16653 | -0.49408 | 6445 | |
| Cox8b | 0.16661 | -1.4381 | 6446 | |
| Cfi | 0.16671 | -1.4362 | 6447 | |
| Erich2 | 0.1668 | -1.327 | 6448 | |
| Otub1 | 0.16684 | -0.58389 | 6449 | |
| Gm1987 | 0.16692 | -1.9965 | 6450 | |
| Anks1 | 0.16706 | -0.96197 | 6451 | |
| Olfr1093 | 0.16706 | -1.4364 | 6452 | |
| Pgpep1l | 0.16712 | -0.36057 | 6453 | |
| Bcl2l12 | 0.16712 | -0.2983 | 6454 | |
| Tomm7 | 0.16716 | 0.095797 | 6455 | |
| Gcc1 | 0.1672 | -0.14824 | 6456 | |
| Taf7l | 0.16724 | 0.020154 | 6457 | |
| Olfr606 | 0.16724 | -1.3482 | 6458 | |
| Tcirg1 | 0.16726 | -1.4339 | 6459 | |
| 4930504O13Rik | 0.16728 | -1.431 | 6460 | |
| 4930430D24Rik | 0.16731 | -0.62264 | 6461 | |
| Ostm1 | 0.16732 | 0.91135 | 6462 | |
| Olfr1277 | 0.16732 | -1.0623 | 6463 | |
| 2410131K14Rik | 0.1674 | -1.1194 | 6464 | |
| Ubxn2b | 0.16744 | -1.3911 | 6465 | |
| Rps19bp1 | 0.16744 | -0.59664 | 6466 | |
| Spg21 | 0.16749 | -1.4335 | 6467 | |
| Fbxw2 | 0.16752 | 2.2681 | 6468 | |
| Zfp219 | 0.16756 | -1.4311 | 6469 | |
| mmu-mir-3094 | 0.16756 | -1.2667 | 6470 | |
| Oog1 | 0.16758 | -0.38817 | 6471 | |
| C2cd4b | 0.16758 | -1.4324 | 6472 | |
| Gdpd1 | 0.16764 | -0.66093 | 6473 | |
| Kcne3 | 0.16768 | -1.4315 | 6474 | |
| Rimkla | 0.16768 | -1.3948 | 6475 | |
| Klf1 | 0.1677 | -1.4332 | 6476 | |
| Ptk2 | 0.16772 | -0.31064 | 6477 | |
| Vmn1r225 | 0.16779 | -1.4278 | 6478 | |
| Car9 | 0.16792 | -1.2114 | 6479 | |
| F2 | 0.16796 | -0.85186 | 6480 | |
| Tmco3 | 0.16808 | -0.22778 | 6481 | |
| Avpr2 | 0.1681 | -1.3578 | 6482 | |
| Smndc1 | 0.16816 | 0.44738 | 6483 | |
| mmu-mir-1928 | 0.1682 | -0.7908 | 6484 | |
| Ap1b1 | 0.16821 | -1.0162 | 6485 | |
| mmu-mir-300 | 0.16831 | -0.90915 | 6486 | |
| Mchr1 | 0.16835 | -1.4292 | 6487 | |
| Stt3a | 0.16845 | -1.4292 | 6488 | |
| Tal2 | 0.16849 | -0.88482 | 6489 | |
| Olfr791 | 0.16857 | -1.4287 | 6490 | |
| mmu-mir-1962 | 0.16859 | -0.19379 | 6491 | |
| Zfp41 | 0.16861 | -0.4717 | 6492 | |
| Ttbk1 | 0.16864 | -1.4252 | 6493 | |
| mmu-mir-136 | 0.1687 | -1.3262 | 6494 | |
| Grb2 | 0.16871 | -1.0686 | 6495 | |
| Il18bp | 0.16879 | -1.4267 | 6496 | |
| mmu-mir-687 | 0.16883 | -0.91006 | 6497 | |
| Slco5a1 | 0.16884 | -1.4279 | 6498 | |
| 5430419D17Rik | 0.16887 | -0.82597 | 6499 | |
| 1300017J02Rik | 0.16889 | -0.77251 | 6500 | |
| Acot4 | 0.16893 | -1.0081 | 6501 | |
| Fbxw8 | 0.16895 | -1.4271 | 6502 | |
| Lmcd1 | 0.16895 | -1.4271 | 6503 | |
| Ebi3 | 0.16901 | -1.0734 | 6504 | |
| Cnn1 | 0.16903 | -1.4255 | 6505 | |
| Gstm5 | 0.16912 | -1.4194 | 6506 | |
| Mbnl3 | 0.16913 | 0.6737 | 6507 | |
| Slc25a42 | 0.16921 | -0.28959 | 6508 | |
| Upp1 | 0.16925 | 0.50471 | 6509 | |
| Reg3d | 0.16929 | -1.0563 | 6510 | |
| Nsun3 | 0.1694 | -0.84681 | 6511 | |
| mmu-mir-218-1 | 0.1694 | -0.72204 | 6512 | |
| Krtap4-8 | 0.16944 | -1.4246 | 6513 | |
| Dcun1d3 | 0.16949 | -0.22225 | 6514 | |
| mmu-mir-698 | 0.16951 | -0.84561 | 6515 | |
| Ptbp1 | 0.16957 | 0.1303 | 6516 | |
| mmu-mir-7673 | 0.16961 | -0.70702 | 6517 | |
| Ankfy1 | 0.16965 | -0.83611 | 6518 | |
| Avil | 0.1697 | -1.2634 | 6519 | |
| Lin7c | 0.16973 | 0.10417 | 6520 | |
| Vmn2r101 | 0.16979 | -0.71582 | 6521 | |
| Tmem35 | 0.16981 | -0.78231 | 6522 | |
| Afap1l1 | 0.16984 | -1.4175 | 6523 | |
| mmu-mir-1963 | 0.16993 | -0.16696 | 6524 | |
| Zfp846 | 0.16993 | -0.61913 | 6525 | |
| Mmel1 | 0.16997 | -0.16761 | 6526 | |
| Nr2e1 | 0.16998 | -1.4166 | 6527 | |
| Fam183b | 0.17006 | -0.28581 | 6528 | |
| Cd40 | 0.1701 | -0.30055 | 6529 | |
| Mettl4 | 0.1701 | -1.0394 | 6530 | |
| mmu-mir-1964 | 0.17012 | -0.82523 | 6531 | |
| Opalin | 0.17014 | -0.039224 | 6532 | |
| mmu-mir-718 | 0.17016 | -0.94256 | 6533 | |
| Stx2 | 0.17022 | -0.69457 | 6534 | |
| mmu-mir-669g | 0.17025 | -0.8328 | 6535 | |
| Sox9 | 0.17026 | -0.60121 | 6536 | |
| Crat | 0.1703 | -1.4191 | 6537 | |
| Iqce | 0.17032 | -1.4169 | 6538 | |
| Zcchc3 | 0.17039 | -1.4088 | 6539 | |
| Polr1d | 0.1704 | -0.70585 | 6540 | |
| Klk1b9 | 0.1704 | -0.91467 | 6541 | |
| Cept1 | 0.1704 | -0.063134 | 6542 | |
| mmu-mir-100 | 0.1704 | -0.78461 | 6543 | |
| Gpr88 | 0.17042 | -0.39762 | 6544 | |
| Spdl1 | 0.17046 | -0.34395 | 6545 | |
| Mdh1b | 0.17051 | -1.4198 | 6546 | |
| mmu-mir-6420 | 0.17063 | -0.86655 | 6547 | |
| Pcdhgb4 | 0.17067 | -0.50423 | 6548 | |
| Slco1b2 | 0.1707 | -0.018818 | 6549 | |
| mmu-mir-6938 | 0.17073 | -0.90438 | 6550 | |
| mmu-mir-6385 | 0.17076 | -1.2212 | 6551 | |
| Nptx1 | 0.17082 | 1.4501 | 6552 | |
| Adra1d | 0.17092 | -1.4183 | 6553 | |
| Spag1 | 0.17092 | -1.4183 | 6554 | |
| Trappc6b | 0.17102 | -0.96205 | 6555 | |
| Clec11a | 0.17107 | -1.4173 | 6556 | |
| Ace | 0.1711 | 1.7065 | 6557 | |
| Rnf180 | 0.17114 | -0.82383 | 6558 | |
| mmu-mir-5616 | 0.17117 | -1.0204 | 6559 | |
| Hyls1 | 0.17122 | -1.4163 | 6560 | |
| Jund | 0.17123 | -1.4153 | 6561 | |
| Polr2b | 0.17124 | -0.32001 | 6562 | |
| Mylk | 0.17136 | -1.4127 | 6563 | |
| Rnf165 | 0.17136 | -0.59378 | 6564 | |
| Trim16 | 0.17141 | -1.266 | 6565 | |
| Arl16 | 0.17153 | -1.4151 | 6566 | |
| Vdac1 | 0.17154 | -1.0428 | 6567 | |
| mmu-mir-6951 | 0.17156 | -0.4865 | 6568 | |
| Atrn | 0.17173 | -1.0091 | 6569 | |
| Fezf2 | 0.17176 | -1.3924 | 6570 | |
| Phf23 | 0.17178 | -0.89945 | 6571 | |
| Tbx22 | 0.1718 | -1.3768 | 6572 | |
| Actb | 0.1718 | -0.36907 | 6573 | |
| Defb39 | 0.1718 | -0.208 | 6574 | |
| Olfr402 | 0.1718 | -0.50297 | 6575 | |
| Zfp644 | 0.17187 | -1.414 | 6576 | |
| Klhl35 | 0.1719 | -0.24162 | 6577 | |
| Fbxw28 | 0.17194 | -0.09794 | 6578 | |
| Ttll3 | 0.17196 | -1.4095 | 6579 | |
| Acnat1 | 0.17198 | -0.79039 | 6580 | |
| Mtap | 0.17201 | -1.3714 | 6581 | |
| mmu-mir-7030 | 0.17204 | 0.53555 | 6582 | |
| Olfr1496 | 0.1721 | -0.15202 | 6583 | |
| Sprr2e | 0.1722 | -0.25688 | 6584 | |
| mmu-mir-24-2 | 0.17226 | 0.60236 | 6585 | |
| Rps7 | 0.1723 | 0.11319 | 6586 | |
| Rfwd3 | 0.17231 | -1.3948 | 6587 | |
| Mapk8ip3 | 0.17238 | -1.3851 | 6588 | |
| Rqcd1 | 0.17244 | -1.0275 | 6589 | |
| mmu-mir-743a | 0.17251 | -0.56869 | 6590 | |
| Dst | 0.17272 | -1.4077 | 6591 | |
| Sult2a1 | 0.17273 | -0.72826 | 6592 | |
| Zpld1 | 0.17273 | -1.195 | 6593 | |
| Trmt10a | 0.17281 | -1.3173 | 6594 | |
| mmu-mir-490 | 0.17282 | -1.0428 | 6595 | |
| Spint1 | 0.17291 | -1.0645 | 6596 | |
| Egr4 | 0.17299 | -0.12708 | 6597 | |
| Kif12 | 0.173 | -1.4008 | 6598 | |
| Comtd1 | 0.17303 | 0.15393 | 6599 | |
| Defb11 | 0.17314 | -1.4093 | 6600 | |
| mmu-mir-187 | 0.17314 | 0.95179 | 6601 | |
| Scgb2b26 | 0.1732 | -1.393 | 6602 | |
| mmu-mir-7065 | 0.17324 | -0.68364 | 6603 | |
| Slc6a9 | 0.17328 | -0.91479 | 6604 | |
| Samd5 | 0.1733 | -1.4083 | 6605 | |
| 4921539E11Rik | 0.17332 | -1.4083 | 6606 | |
| Cox5b | 0.1735 | -1.4071 | 6607 | |
| 1700020N15Rik | 0.17363 | -0.1383 | 6608 | |
| Vwa9 | 0.17363 | 1.2676 | 6609 | |
| Supt6 | 0.17363 | -0.52328 | 6610 | |
| Tubb1 | 0.17367 | -1.3951 | 6611 | |
| Chrna5 | 0.17385 | -0.36074 | 6612 | |
| Ms4a7 | 0.17388 | -1.4059 | 6613 | |
| Mrpl16 | 0.17398 | -1.4052 | 6614 | |
| Idi1 | 0.17399 | -1.2714 | 6615 | |
| Pon3 | 0.17403 | 1.4441 | 6616 | |
| mmu-mir-692-1 | 0.17404 | -0.99022 | 6617 | |
| Lrrc66 | 0.17411 | 0.31264 | 6618 | |
| Chpt1 | 0.17412 | -1.4005 | 6619 | |
| Nnat | 0.17419 | -0.45681 | 6620 | |
| Stag1 | 0.17421 | -0.72902 | 6621 | |
| 6330416G13Rik | 0.17422 | -1.1081 | 6622 | |
| Vps4a | 0.17431 | -1.2497 | 6623 | |
| Nfatc2ip | 0.17439 | -0.08737 | 6624 | |
| Vgll4 | 0.17442 | -0.98773 | 6625 | |
| Amdhd2 | 0.17443 | -0.18848 | 6626 | |
| Ubfd1 | 0.17447 | -0.94437 | 6627 | |
| Eif3j1 | 0.17451 | -1.1136 | 6628 | |
| Olfr814 | 0.17453 | -1.3962 | 6629 | |
| Olfr1137 | 0.17459 | -0.28259 | 6630 | |
| Mtmr9 | 0.17463 | -1.2453 | 6631 | |
| S100a11 | 0.17463 | -1.4016 | 6632 | |
| Olfr1223 | 0.17473 | -0.91297 | 6633 | |
| Mier1 | 0.17473 | -0.44923 | 6634 | |
| Abhd5 | 0.17478 | -0.27093 | 6635 | |
| Bpifb1 | 0.17479 | -0.41467 | 6636 | |
| Hsd17b7 | 0.17485 | -0.91383 | 6637 | |
| Gabbr1 | 0.17487 | -0.53325 | 6638 | |
| Swi5 | 0.17488 | -1.4015 | 6639 | |
| Rex2 | 0.1749 | -0.46089 | 6640 | |
| Olfr170 | 0.17491 | -0.87182 | 6641 | |
| Wapal | 0.17492 | -1.4017 | 6642 | |
| Afm | 0.17499 | -0.40335 | 6643 | |
| Vmn2r29 | 0.17503 | -0.82155 | 6644 | |
| Olfr652 | 0.17506 | -1.4009 | 6645 | |
| Uqcc2 | 0.17508 | -1.3995 | 6646 | |
| Bcl2a1a | 0.1751 | -1.4004 | 6647 | |
| Myh4 | 0.17511 | -0.9498 | 6648 | |
| Calcb | 0.17513 | -0.50446 | 6649 | |
| Magi1 | 0.17519 | 2.2156 | 6650 | |
| Art3 | 0.17527 | -1.3114 | 6651 | |
| Tmem230 | 0.17531 | 0.97599 | 6652 | |
| Rad51d | 0.17537 | 0.98874 | 6653 | |
| Plekhs1 | 0.17551 | -0.46915 | 6654 | |
| Gm13826 | 0.17551 | 1.8398 | 6655 | |
| Dnaaf1 | 0.17552 | -1.3975 | 6656 | |
| Olfr873 | 0.17554 | -0.50327 | 6657 | |
| Fam71f1 | 0.17559 | 3.0292 | 6658 | |
| Pex19 | 0.17559 | -0.24356 | 6659 | |
| Mdm2 | 0.17562 | -1.396 | 6660 | |
| mmu-mir-592 | 0.17564 | -0.99713 | 6661 | |
| Fbxw10 | 0.17565 | -1.3981 | 6662 | |
| Cox10 | 0.17571 | -1.2593 | 6663 | |
| mmu-mir-6982 | 0.17577 | 0.018116 | 6664 | |
| Itpk1 | 0.17581 | -0.25207 | 6665 | |
| mmu-mir-181a-2 | 0.17582 | -0.67982 | 6666 | |
| Arsk | 0.17587 | -0.24072 | 6667 | |
| Med4 | 0.17589 | -1.3971 | 6668 | |
| mmu-mir-376a | 0.17591 | -0.74288 | 6669 | |
| Slc16a11 | 0.17599 | -1.3973 | 6670 | |
| Tmem165 | 0.17599 | 0.091234 | 6671 | |
| Tubb2b | 0.17601 | -1.3917 | 6672 | |
| Trhde | 0.17608 | -1.397 | 6673 | |
| Ckap4 | 0.17609 | -0.90226 | 6674 | |
| Lingo4 | 0.17611 | -1.1697 | 6675 | |
| Mmachc | 0.17631 | -1.0306 | 6676 | |
| 11-Mar | 0.17633 | -1.3475 | 6677 | |
| Prn | 0.17634 | -1.9186 | 6678 | |
| Scyl3 | 0.17634 | -0.38159 | 6679 | |
| Cyp2b23 | 0.17635 | 0.12351 | 6680 | |
| Htra1 | 0.17638 | -1.3918 | 6681 | |
| Prl | 0.17639 | -0.88116 | 6682 | |
| Uqcr10 | 0.17649 | -1.3946 | 6683 | |
| Gm14391 | 0.17654 | -1.8281 | 6684 | |
| Cnnm4 | 0.17655 | 0.43078 | 6685 | |
| mmu-mir-7009 | 0.17659 | 0.015085 | 6686 | |
| Gpr139 | 0.17661 | -1.3945 | 6687 | |
| Krt86 | 0.17666 | -1.3928 | 6688 | |
| Amy1 | 0.17667 | -0.54709 | 6689 | |
| S100pbp | 0.17671 | -1.2039 | 6690 | |
| Itm2c | 0.17672 | -1.2066 | 6691 | |
| Rbmx | 0.17675 | -0.80458 | 6692 | |
| Trp53inp2 | 0.17679 | -0.1826 | 6693 | |
| Six4 | 0.17681 | -1.3885 | 6694 | |
| 2610301B20Rik | 0.17683 | -1.0491 | 6695 | |
| 9230104L09Rik | 0.17684 | -1.3913 | 6696 | |
| Npc2 | 0.17687 | -0.88391 | 6697 | |
| 5730409E04Rik | 0.17691 | -0.48348 | 6698 | |
| mmu-mir-669m-1 | 0.17693 | -0.19298 | 6699 | |
| Adcy5 | 0.17695 | -0.98306 | 6700 | |
| Ly6g6c | 0.17699 | 0.49114 | 6701 | |
| Nlgn1 | 0.17703 | -0.51201 | 6702 | |
| Hoxa11 | 0.17711 | -0.83119 | 6703 | |
| Syce1l | 0.17723 | -0.83851 | 6704 | |
| Zkscan16 | 0.17725 | -0.52368 | 6705 | |
| Clec4n | 0.17727 | -1.3897 | 6706 | |
| Pag1 | 0.17729 | -0.82278 | 6707 | |
| mmu-mir-759 | 0.17731 | -1.1471 | 6708 | |
| Inpp5e | 0.17732 | -1.3907 | 6709 | |
| Olfr1058 | 0.17737 | 0.32997 | 6710 | |
| Golph3 | 0.1774 | -1.3915 | 6711 | |
| Ppic | 0.17747 | 0.067351 | 6712 | |
| 2010015L04Rik | 0.17773 | -0.77533 | 6713 | |
| Pdpr | 0.17773 | -0.78733 | 6714 | |
| Hist1h3c | 0.17773 | -0.49823 | 6715 | |
| Tnfsf8 | 0.17777 | -1.3869 | 6716 | |
| Rxrg | 0.17783 | -1.3903 | 6717 | |
| Cstf2 | 0.17786 | -1.3902 | 6718 | |
| A330050F15Rik | 0.17791 | 0.59368 | 6719 | |
| Picalm | 0.17799 | -1.1655 | 6720 | |
| Olfr259 | 0.17803 | -0.49029 | 6721 | |
| Bin1 | 0.17804 | -1.2776 | 6722 | |
| Ghdc | 0.17806 | -1.3862 | 6723 | |
| Otp | 0.17807 | 0.67054 | 6724 | |
| Csrnp3 | 0.17811 | -0.70388 | 6725 | |
| Wdr90 | 0.17819 | -1.3876 | 6726 | |
| Phospho1 | 0.17826 | -1.3883 | 6727 | |
| Zfp81 | 0.17827 | -0.03511 | 6728 | |
| Rasgrp1 | 0.17833 | -0.24559 | 6729 | |
| Cdkn2aipnl | 0.17835 | -0.09231 | 6730 | |
| Stau1 | 0.1784 | -1.3786 | 6731 | |
| Kcnk5 | 0.17844 | -1.3875 | 6732 | |
| Myl10 | 0.17847 | 1.376 | 6733 | |
| Prss54 | 0.17849 | -1.3867 | 6734 | |
| Ascl4 | 0.17851 | -0.66373 | 6735 | |
| A530099J19Rik | 0.17855 | -0.10652 | 6736 | |
| Cox4i2 | 0.17865 | -0.15507 | 6737 | |
| Card14 | 0.17867 | -1.3869 | 6738 | |
| Zcchc8 | 0.17871 | -0.89335 | 6739 | |
| Nlk | 0.17871 | -1.3853 | 6740 | |
| Cdca5 | 0.17874 | -1.0133 | 6741 | |
| Gm12794 | 0.17891 | 0.0055509 | 6742 | |
| Rdh10 | 0.17897 | -1.386 | 6743 | |
| Slc19a3 | 0.17904 | -1.3797 | 6744 | |
| Fastkd2 | 0.17906 | -1.3804 | 6745 | |
| Myrip | 0.17908 | -0.15272 | 6746 | |
| Ndufb5 | 0.17915 | -1.3719 | 6747 | |
| Tmem8 | 0.17919 | -0.36882 | 6748 | |
| Zfp26 | 0.17919 | -0.70861 | 6749 | |
| Arhgap28 | 0.17923 | 0.12896 | 6750 | |
| mmu-mir-344c | 0.17923 | -0.94287 | 6751 | |
| Gm12886 | 0.17927 | 0.17737 | 6752 | |
| Ung | 0.17931 | -1.3753 | 6753 | |
| Plek | 0.17935 | -1.3768 | 6754 | |
| Muc6 | 0.17939 | -1.3798 | 6755 | |
| Ndrg2 | 0.17946 | -0.18304 | 6756 | |
| Cnr1 | 0.17947 | -1.3816 | 6757 | |
| Eif4a1 | 0.17953 | -1.1397 | 6758 | |
| Lrp4 | 0.17965 | -1.2307 | 6759 | |
| Slc22a28 | 0.1797 | -1.3776 | 6760 | |
| Olfr813 | 0.1799 | -0.85538 | 6761 | |
| Epha10 | 0.1799 | -0.20604 | 6762 | |
| Pabpc4 | 0.17992 | -0.76997 | 6763 | |
| Sh3bp1 | 0.18004 | -1.3757 | 6764 | |
| Tspan9 | 0.1801 | 0.71685 | 6765 | |
| Golga3 | 0.18014 | -1.3813 | 6766 | |
| Msl3l2 | 0.18014 | -1.0585 | 6767 | |
| Sspo | 0.18017 | -1.1184 | 6768 | |
| Reep6 | 0.18019 | -1.3813 | 6769 | |
| Nalcn | 0.18022 | -0.4253 | 6770 | |
| mmu-mir-200a | 0.18023 | -1.0109 | 6771 | |
| Iqsec3 | 0.18028 | -1.2331 | 6772 | |
| Cyp2d9 | 0.18036 | -0.32328 | 6773 | |
| Itga8 | 0.18036 | -0.75548 | 6774 | |
| Olfr787 | 0.18041 | -0.73742 | 6775 | |
| mmu-mir-691 | 0.18041 | -0.57899 | 6776 | |
| mmu-mir-6970 | 0.18041 | 0.10777 | 6777 | |
| Aurka | 0.18046 | -1.1459 | 6778 | |
| Cox8c | 0.1805 | -1.3778 | 6779 | |
| mmu-mir-6381 | 0.18052 | -1.2733 | 6780 | |
| Jrkl | 0.18054 | -0.5434 | 6781 | |
| Emc6 | 0.18058 | -0.71005 | 6782 | |
| Ly6g5b | 0.18062 | 0.17152 | 6783 | |
| Fam25c | 0.18062 | -0.050813 | 6784 | |
| Slc8b1 | 0.18064 | -0.33257 | 6785 | |
| Pick1 | 0.18074 | -0.6958 | 6786 | |
| Kcnj6 | 0.18086 | -0.26284 | 6787 | |
| mmu-mir-8106 | 0.18087 | -0.94187 | 6788 | |
| St3gal6 | 0.18093 | -1.3773 | 6789 | |
| Gata3 | 0.18097 | -1.3777 | 6790 | |
| Enpp1 | 0.181 | -0.98285 | 6791 | |
| Akr1c13 | 0.18104 | -1.1963 | 6792 | |
| Grk1 | 0.18106 | -1.3693 | 6793 | |
| Pcdh17 | 0.18107 | -1.3689 | 6794 | |
| mmu-mir-199a-2 | 0.18109 | -0.96218 | 6795 | |
| Rdm1 | 0.18109 | -0.14715 | 6796 | |
| mmu-mir-3073b | 0.18115 | -1.2424 | 6797 | |
| Slc23a3 | 0.18117 | -1.3688 | 6798 | |
| Afg3l1 | 0.18122 | 0.58053 | 6799 | |
| Tas1r1 | 0.18126 | -0.4641 | 6800 | |
| Ext2 | 0.18138 | -0.29628 | 6801 | |
| Mtl5 | 0.18144 | -0.20315 | 6802 | |
| Med7 | 0.18144 | -0.046075 | 6803 | |
| Dcaf7 | 0.18149 | -0.60727 | 6804 | |
| Csn1s2b | 0.18155 | -0.068326 | 6805 | |
| Gapvd1 | 0.18165 | -0.71444 | 6806 | |
| Olfr609 | 0.1817 | -0.24478 | 6807 | |
| Zfp790 | 0.18181 | -1.2108 | 6808 | |
| Olfr1113 | 0.18182 | 1.2036 | 6809 | |
| Vmn1r237 | 0.1819 | 0.11924 | 6810 | |
| Exoc4 | 0.18194 | -0.77588 | 6811 | |
| Lrfn4 | 0.18201 | -1.294 | 6812 | |
| Greb1l | 0.18206 | -1.3726 | 6813 | |
| Tmem182 | 0.18206 | 0.6069 | 6814 | |
| D2hgdh | 0.18217 | -1.373 | 6815 | |
| Rbp1 | 0.18219 | -0.74751 | 6816 | |
| Zbtb8b | 0.18222 | 0.57098 | 6817 | |
| Galc | 0.18223 | -0.80651 | 6818 | |
| Cfp | 0.18233 | -0.69285 | 6819 | |
| Supt3 | 0.18235 | -1.3671 | 6820 | |
| Gykl1 | 0.18237 | -0.3297 | 6821 | |
| Eva1a | 0.18244 | -0.40485 | 6822 | |
| Hook3 | 0.18245 | -1.0397 | 6823 | |
| Zfp97 | 0.18251 | -0.98344 | 6824 | |
| Notch3 | 0.18253 | -1.1657 | 6825 | |
| Tex19.1 | 0.18265 | 0.42706 | 6826 | |
| Il22ra2 | 0.1827 | -0.029815 | 6827 | |
| Sult2a2 | 0.18271 | 0.17987 | 6828 | |
| Ror1 | 0.18271 | 0.16201 | 6829 | |
| Ndufa6 | 0.18279 | -1.3708 | 6830 | |
| Lipt1 | 0.18291 | -1.1671 | 6831 | |
| 1600002K03Rik | 0.18293 | -1.2971 | 6832 | |
| mmu-mir-6371 | 0.18298 | -0.45198 | 6833 | |
| Egflam | 0.18301 | -0.27637 | 6834 | |
| Unc93a | 0.18303 | 0.016385 | 6835 | |
| Dync1li2 | 0.18306 | -0.64706 | 6836 | |
| Rhox8 | 0.18309 | -0.1926 | 6837 | |
| Vstm2a | 0.18313 | -0.34108 | 6838 | |
| mmu-mir-6378 | 0.18315 | 0.55847 | 6839 | |
| Ucn2 | 0.18321 | 1.4118 | 6840 | |
| Slc9a8 | 0.18325 | -0.10538 | 6841 | |
| Lasp1 | 0.18326 | -1.3351 | 6842 | |
| Mtcp1 | 0.18333 | -0.27411 | 6843 | |
| mmu-mir-3473c | 0.18333 | -0.29867 | 6844 | |
| Exosc2 | 0.18337 | -0.53428 | 6845 | |
| Sympk | 0.1834 | -1.3647 | 6846 | |
| Gm6460 | 0.18342 | -1.6608 | 6847 | |
| Glp2r | 0.18345 | -0.37306 | 6848 | |
| Lce1e | 0.18369 | 1.0207 | 6849 | |
| 2610018G03Rik | 0.18373 | -1.3627 | 6850 | |
| Gm21949 | 0.18373 | 3.0952 | 6851 | |
| mmu-mir-1a-2 | 0.18375 | -1.5827 | 6852 | |
| Comt | 0.18377 | -0.15726 | 6853 | |
| Rmi2 | 0.18378 | -1.3637 | 6854 | |
| Gvin1 | 0.1838 | -1.2311 | 6855 | |
| Gpbar1 | 0.18381 | -0.17938 | 6856 | |
| Dmxl2 | 0.18382 | -1.3656 | 6857 | |
| Cdc25a | 0.18389 | -0.19881 | 6858 | |
| Gng12 | 0.18394 | -1.3658 | 6859 | |
| 6430573F11Rik | 0.18401 | 0.6326 | 6860 | |
| Vmn2r77 | 0.18402 | -1.3665 | 6861 | |
| Fam71f2 | 0.18407 | -0.15685 | 6862 | |
| Ceacam11 | 0.18412 | 0.48707 | 6863 | |
| Mfsd8 | 0.18418 | -1.1981 | 6864 | |
| Ces1c | 0.1842 | -0.22524 | 6865 | |
| Itln1 | 0.18422 | -0.52124 | 6866 | |
| Hace1 | 0.18428 | -0.053031 | 6867 | |
| Dmwd | 0.18432 | -1.3634 | 6868 | |
| Glrx5 | 0.1844 | -0.28773 | 6869 | |
| Tulp3 | 0.18443 | -1.3549 | 6870 | |
| Cdc42ep1 | 0.1845 | -1.3626 | 6871 | |
| Hist1h2bm | 0.1845 | -1.4778 | 6872 | |
| 6330403A02Rik | 0.18454 | -1.3594 | 6873 | |
| Olfr1306 | 0.18456 | 0.75955 | 6874 | |
| Hhex | 0.1846 | -0.72791 | 6875 | |
| mmu-mir-7057 | 0.18462 | -0.91147 | 6876 | |
| Tas2r124 | 0.18462 | -1.3606 | 6877 | |
| Hrg | 0.18464 | 0.093408 | 6878 | |
| Rab40b | 0.18468 | -1.3632 | 6879 | |
| Atl2 | 0.18471 | -1.3575 | 6880 | |
| Xylt2 | 0.18474 | -1.3601 | 6881 | |
| Olfr122 | 0.18476 | -0.64016 | 6882 | |
| Pga5 | 0.18479 | -1.362 | 6883 | |
| mmu-mir-344b | 0.18481 | -1.2752 | 6884 | |
| mmu-mir-465d | 0.18485 | -1.1596 | 6885 | |
| Ankra2 | 0.18489 | -1.3564 | 6886 | |
| Grlf1 | 0.1849 | -0.96214 | 6887 | |
| Gpr75 | 0.18496 | -0.99299 | 6888 | |
| Alcam | 0.18503 | -1.1227 | 6889 | |
| Asna1 | 0.18507 | -1.3192 | 6890 | |
| Magea3 | 0.18508 | -0.47818 | 6891 | |
| Rai2 | 0.18516 | 0.05316 | 6892 | |
| Tmem14a | 0.18516 | -1.3609 | 6893 | |
| Zfp775 | 0.18518 | -1.3529 | 6894 | |
| Ccny | 0.1852 | -1.2965 | 6895 | |
| Gpr116 | 0.18523 | -1.3604 | 6896 | |
| Trim44 | 0.18528 | -0.20157 | 6897 | |
| Ssxb2 | 0.18528 | -1.358 | 6898 | |
| Olfr434 | 0.18536 | -0.38492 | 6899 | |
| Emp2 | 0.1854 | -0.50592 | 6900 | |
| Cabp5 | 0.18544 | -0.81856 | 6901 | |
| Sclt1 | 0.18545 | -0.60699 | 6902 | |
| Hinfp | 0.18552 | -1.3595 | 6903 | |
| Chrac1 | 0.18555 | -0.94934 | 6904 | |
| Hoxc6 | 0.18567 | -1.3536 | 6905 | |
| Map3k14 | 0.18572 | -1.3575 | 6906 | |
| Grpel2 | 0.18579 | -0.10025 | 6907 | |
| Megf9 | 0.18583 | 0.30462 | 6908 | |
| Olfr1062 | 0.18585 | -1.3491 | 6909 | |
| Olfr1286 | 0.1859 | -1.2129 | 6910 | |
| Tm9sf4 | 0.18596 | -0.44806 | 6911 | |
| Map2k2 | 0.18607 | -1.3536 | 6912 | |
| Fam189b | 0.18619 | -1.2459 | 6913 | |
| Gm13306 | 0.18638 | 0.21971 | 6914 | |
| mmu-mir-1947 | 0.18638 | 0.29653 | 6915 | |
| Sprr2f | 0.18645 | -1.3431 | 6916 | |
| Spink7 | 0.18649 | -1.3556 | 6917 | |
| Tas2r140 | 0.18659 | -0.45207 | 6918 | |
| Utp20 | 0.1866 | -1.3423 | 6919 | |
| 2310042E22Rik | 0.1866 | -1.3423 | 6920 | |
| Cst9 | 0.18664 | -1.3554 | 6921 | |
| 4930506M07Rik | 0.18664 | -0.41419 | 6922 | |
| mmu-let-7j | 0.18665 | -0.89415 | 6923 | |
| Gm10228 | 0.18666 | -1.781 | 6924 | |
| Net1 | 0.18667 | 1.75 | 6925 | |
| Scyl2 | 0.1867 | -1.0505 | 6926 | |
| Isl1 | 0.18676 | -0.97122 | 6927 | |
| 4933406M09Rik | 0.18679 | -1.348 | 6928 | |
| Ebf2 | 0.1869 | -0.56883 | 6929 | |
| Atp9b | 0.18698 | -1.1897 | 6930 | |
| Krtap6-2 | 0.18701 | -0.67833 | 6931 | |
| Il3 | 0.1871 | -1.3291 | 6932 | |
| Cyp2d34 | 0.18714 | -1.1148 | 6933 | |
| Aes | 0.18718 | 1.6695 | 6934 | |
| Fads1 | 0.18722 | -0.82633 | 6935 | |
| Oit3 | 0.18726 | -0.90283 | 6936 | |
| Usp14 | 0.18732 | -0.11496 | 6937 | |
| Mcidas | 0.18736 | -0.33653 | 6938 | |
| Apex1 | 0.18741 | -1.3504 | 6939 | |
| Pbxip1 | 0.18747 | -1.3501 | 6940 | |
| Ccr3 | 0.1875 | -0.89887 | 6941 | |
| Olfr64 | 0.18758 | -0.15843 | 6942 | |
| Rftn2 | 0.18766 | -0.45312 | 6943 | |
| Notch1 | 0.18767 | -0.1339 | 6944 | |
| mmu-mir-470 | 0.18768 | -0.080019 | 6945 | |
| Clec1a | 0.1877 | -0.12352 | 6946 | |
| St3gal3 | 0.18783 | -0.96912 | 6947 | |
| Olfr1453 | 0.18795 | -0.35899 | 6948 | |
| Tmprss7 | 0.18795 | 0.75468 | 6949 | |
| Syce1 | 0.18802 | -1.3482 | 6950 | |
| Mtmr6 | 0.18805 | -1.3474 | 6951 | |
| Prkar1b | 0.18807 | -1.3413 | 6952 | |
| Cnih1 | 0.18825 | -1.2262 | 6953 | |
| Camk1g | 0.18827 | -1.3406 | 6954 | |
| Gm5936 | 0.18829 | -0.48524 | 6955 | |
| Smurf1 | 0.18831 | -1.3459 | 6956 | |
| Chst11 | 0.18836 | -1.3146 | 6957 | |
| Ckmt2 | 0.18838 | -1.3167 | 6958 | |
| Trim46 | 0.18841 | 0.78265 | 6959 | |
| Vmn2r68 | 0.18845 | 3.4005 | 6960 | |
| Fosb | 0.18851 | -0.94777 | 6961 | |
| Xylb | 0.18857 | -0.66434 | 6962 | |
| Olfr1354 | 0.18869 | -0.59968 | 6963 | |
| Masp1 | 0.18871 | -1.2486 | 6964 | |
| Mrps23 | 0.18875 | -1.3437 | 6965 | |
| Dnaja2 | 0.18875 | -0.13209 | 6966 | |
| Vmn1r20 | 0.18883 | -0.99237 | 6967 | |
| Tns3 | 0.18893 | -0.87849 | 6968 | |
| Farp2 | 0.18902 | -0.77005 | 6969 | |
| Alg9 | 0.18917 | -1.3416 | 6970 | |
| Arhgap44 | 0.18935 | -0.51235 | 6971 | |
| Pcdhb5 | 0.18937 | -0.21331 | 6972 | |
| Atp6ap1l | 0.1894 | -0.49697 | 6973 | |
| Setbp1 | 0.18944 | -1.3275 | 6974 | |
| Olfr553 | 0.18944 | -1.3427 | 6975 | |
| Nme6 | 0.18952 | 0.61465 | 6976 | |
| Inca1 | 0.18959 | -0.33574 | 6977 | |
| Hist1h4k | 0.18964 | -1.3393 | 6978 | |
| Aatf | 0.18968 | -0.29606 | 6979 | |
| Aff3 | 0.1897 | -1.2567 | 6980 | |
| Ctsll3 | 0.18971 | -0.19806 | 6981 | |
| Gm960 | 0.18973 | -1.1296 | 6982 | |
| Nqo1 | 0.18974 | 0.050754 | 6983 | |
| Ints2 | 0.18979 | -0.97432 | 6984 | |
| Asz1 | 0.18988 | -1.2351 | 6985 | |
| Sgca | 0.18991 | 0.12031 | 6986 | |
| mmu-mir-28c | 0.19006 | 0.077482 | 6987 | |
| Dnajc5b | 0.19007 | -0.1165 | 6988 | |
| Itih2 | 0.19008 | -0.67235 | 6989 | |
| Cdkn3 | 0.19013 | -1.3395 | 6990 | |
| 1700013H16Rik | 0.19013 | -0.24937 | 6991 | |
| Qtrtd1 | 0.19013 | -0.46053 | 6992 | |
| Timp4 | 0.19015 | -1.3312 | 6993 | |
| Zfp930 | 0.19021 | -1.3358 | 6994 | |
| AU021092 | 0.19023 | -1.3364 | 6995 | |
| Gpr128 | 0.19027 | 1.3379 | 6996 | |
| Lsm7 | 0.1903 | -1.2776 | 6997 | |
| Pramef25 | 0.19031 | -0.46856 | 6998 | |
| Ifnar1 | 0.19035 | 0.49666 | 6999 | |
| Ptgis | 0.19047 | -0.5965 | 7000 | |
| Bbs5 | 0.19047 | -1.3384 | 7001 | |
| Derl3 | 0.1905 | -1.3218 | 7002 | |
| AA987161 | 0.19052 | -1.3361 | 7003 | |
| Pbx3 | 0.19055 | 0.32358 | 7004 | |
| 1700123K08Rik | 0.19059 | -0.39956 | 7005 | |
| Uvrag | 0.19059 | -1.3307 | 7006 | |
| Hbp1 | 0.19061 | -1.337 | 7007 | |
| Zfp36l2 | 0.19065 | -0.99548 | 7008 | |
| Lrp8 | 0.19069 | -1.1081 | 7009 | |
| Sfrs18 | 0.19072 | -0.14061 | 7010 | |
| Olfr1417 | 0.1908 | -0.61589 | 7011 | |
| Gosr1 | 0.19084 | -0.72094 | 7012 | |
| Oas1h | 0.19092 | -1.3372 | 7013 | |
| Mrpl51 | 0.19094 | -1.335 | 7014 | |
| Wnk3 | 0.19098 | 0.17539 | 7015 | |
| Ceacam20 | 0.19102 | 0.15522 | 7016 | |
| Rell2 | 0.19105 | -0.15548 | 7017 | |
| Ptpn2 | 0.19108 | 0.083612 | 7018 | |
| mmu-mir-7684 | 0.1911 | -0.48253 | 7019 | |
| Ccnc | 0.19111 | -0.23455 | 7020 | |
| Nlrc4 | 0.19113 | -1.3364 | 7021 | |
| Olfr1333 | 0.19114 | -1.1976 | 7022 | |
| Hoxc9 | 0.19125 | -0.022287 | 7023 | |
| Gm15292 | 0.19127 | -1.46 | 7024 | |
| Pcdhgc5 | 0.19127 | -0.94242 | 7025 | |
| Tmem110 | 0.1913 | -0.80541 | 7026 | |
| Vmn2r82 | 0.19134 | 1.262 | 7027 | |
| Ash2l | 0.19136 | -1.2762 | 7028 | |
| Gnat2 | 0.19144 | -1.2474 | 7029 | |
| Lmnb1 | 0.19149 | 0.18637 | 7030 | |
| Gm4894 | 0.19155 | -0.94554 | 7031 | |
| Gripap1 | 0.19161 | -0.37191 | 7032 | |
| mmu-mir-700 | 0.19165 | -0.92519 | 7033 | |
| Zic5 | 0.19173 | -1.3291 | 7034 | |
| Plekhg2 | 0.19173 | -0.89789 | 7035 | |
| mmu-mir-3113 | 0.19173 | -0.7439 | 7036 | |
| Etfb | 0.19177 | -0.66109 | 7037 | |
| mmu-mir-7067 | 0.19182 | -0.96653 | 7038 | |
| Slc39a2 | 0.19192 | -1.3338 | 7039 | |
| Clcc1 | 0.19197 | 0.1481 | 7040 | |
| Dnajc6 | 0.19201 | -1.2497 | 7041 | |
| Cldn20 | 0.19203 | -1.3323 | 7042 | |
| Iigp1 | 0.19209 | -0.61443 | 7043 | |
| Padi1 | 0.19215 | -1.3259 | 7044 | |
| Thbs1 | 0.19221 | -1.3324 | 7045 | |
| Taar7b | 0.19223 | -1.3312 | 7046 | |
| Lgi2 | 0.19224 | -1.1081 | 7047 | |
| Gngt2 | 0.19228 | 0.3582 | 7048 | |
| Ppp1r3g | 0.19238 | -0.48454 | 7049 | |
| Zfp46 | 0.19238 | -0.82157 | 7050 | |
| Nphp4 | 0.19248 | -1.0893 | 7051 | |
| Rabgef1 | 0.19258 | -1.1285 | 7052 | |
| mmu-mir-467c | 0.1926 | -0.73434 | 7053 | |
| Rnf145 | 0.19261 | -0.49443 | 7054 | |
| Col8a1 | 0.19272 | -1.0791 | 7055 | |
| Lactb2 | 0.19272 | 1.4336 | 7056 | |
| Hnrnpdl | 0.19282 | -1.2725 | 7057 | |
| Prps1l1 | 0.19296 | -0.97391 | 7058 | |
| Grxcr2 | 0.19299 | 0.039104 | 7059 | |
| Gm4841 | 0.193 | -1.0505 | 7060 | |
| Dap3 | 0.19303 | -0.68543 | 7061 | |
| Ankrd36 | 0.19309 | -1.3029 | 7062 | |
| Sept9 | 0.19311 | -0.74662 | 7063 | |
| Mpped2 | 0.19311 | -0.81931 | 7064 | |
| Bzrap1 | 0.19313 | -1.3234 | 7065 | |
| Rsl24d1 | 0.19321 | -0.80688 | 7066 | |
| Sftpd | 0.19329 | -0.87847 | 7067 | |
| Adrm1 | 0.19331 | 0.61813 | 7068 | |
| Appl2 | 0.19331 | -1.3281 | 7069 | |
| Sh3rf2 | 0.19333 | -1.3271 | 7070 | |
| Tmem161a | 0.19347 | -0.56067 | 7071 | |
| Dnm3 | 0.19351 | -0.73252 | 7072 | |
| Tfpt | 0.19355 | 1.1314 | 7073 | |
| Pcdh11x | 0.1937 | 0.63903 | 7074 | |
| Sdad1 | 0.19371 | -1.5386 | 7075 | |
| Sars | 0.19371 | -1.5386 | 7076 | |
| Atp8b1 | 0.19376 | -0.31215 | 7077 | |
| Vmn2r15 | 0.19379 | -1.0362 | 7078 | |
| mmu-mir-7578 | 0.19393 | -0.86615 | 7079 | |
| Olfr31 | 0.19399 | -0.51114 | 7080 | |
| Gramd1c | 0.19404 | -0.029271 | 7081 | |
| Map4 | 0.1941 | -0.055671 | 7082 | |
| Adora3 | 0.19412 | -1.3243 | 7083 | |
| mmu-mir-3085 | 0.19431 | -0.85095 | 7084 | |
| Fgb | 0.1944 | -1.2117 | 7085 | |
| Acrbp | 0.19443 | -1.3242 | 7086 | |
| Olfr1290 | 0.19447 | 0.88659 | 7087 | |
| Birc6 | 0.19453 | -0.60936 | 7088 | |
| Acot5 | 0.19457 | 0.82615 | 7089 | |
| 1200014J11Rik | 0.19459 | -1.3215 | 7090 | |
| Mepe | 0.19465 | -1.3218 | 7091 | |
| Acot3 | 0.19479 | -1.1616 | 7092 | |
| Tst | 0.19481 | -1.3139 | 7093 | |
| Sftpc | 0.19487 | -1.3208 | 7094 | |
| Olfr1272 | 0.19493 | 0.25632 | 7095 | |
| Fam83b | 0.19495 | -0.55025 | 7096 | |
| mmu-mir-379 | 0.195 | -0.56743 | 7097 | |
| Ralyl | 0.19508 | -1.3225 | 7098 | |
| Abcb4 | 0.19513 | -1.321 | 7099 | |
| Gpr1 | 0.19518 | -1.3139 | 7100 | |
| Neil2 | 0.19523 | -1.3192 | 7101 | |
| mmu-mir-1932 | 0.19528 | -0.19276 | 7102 | |
| Commd6 | 0.19531 | -1.3219 | 7103 | |
| Ehd4 | 0.1954 | -1.1221 | 7104 | |
| Zic2 | 0.19545 | -0.55636 | 7105 | |
| Dpep3 | 0.19547 | -1.3118 | 7106 | |
| 2310034C09Rik | 0.19548 | 0.53341 | 7107 | |
| Nuggc | 0.19555 | -1.2959 | 7108 | |
| Trim21 | 0.19556 | 0.25692 | 7109 | |
| Nos3 | 0.1956 | -0.51245 | 7110 | |
| Gm6531 | 0.19565 | 0.61902 | 7111 | |
| Fbxw26 | 0.19579 | -1.3203 | 7112 | |
| Wdr93 | 0.19589 | 0.68167 | 7113 | |
| Arhgap39 | 0.19595 | -0.46988 | 7114 | |
| mmu-mir-8095 | 0.19595 | -0.82949 | 7115 | |
| Nup133 | 0.19597 | -0.90933 | 7116 | |
| Olfr1241 | 0.19601 | -1.3186 | 7117 | |
| Obp1a | 0.19606 | -1.3168 | 7118 | |
| mmu-mir-8120 | 0.19611 | -0.44455 | 7119 | |
| mmu-mir-7217 | 0.19611 | -1.1769 | 7120 | |
| Stac | 0.19615 | 0.12652 | 7121 | |
| Tnfrsf1b | 0.19619 | 0.22226 | 7122 | |
| Gm4952 | 0.19621 | -0.9364 | 7123 | |
| Olfr1249 | 0.19634 | -0.66271 | 7124 | |
| Hey1 | 0.1964 | -1.314 | 7125 | |
| Agpat2 | 0.1964 | -1.314 | 7126 | |
| Grk5 | 0.19642 | 0.16925 | 7127 | |
| Lman2l | 0.19646 | 1.215 | 7128 | |
| Olfr725 | 0.19654 | 0.085962 | 7129 | |
| Cep78 | 0.19658 | 0.88674 | 7130 | |
| Rwdd3 | 0.1966 | -0.66102 | 7131 | |
| Serpina1b | 0.19662 | -1.1116 | 7132 | |
| Ntrk3 | 0.19673 | -0.47297 | 7133 | |
| mmu-mir-3075 | 0.19674 | -0.81856 | 7134 | |
| Atxn10 | 0.1968 | -0.30311 | 7135 | |
| Tmx3 | 0.19684 | -1.3005 | 7136 | |
| Pipox | 0.19697 | 1.1418 | 7137 | |
| Aga | 0.19706 | -1.3104 | 7138 | |
| Phf20l1 | 0.19708 | -0.54984 | 7139 | |
| Cbr2 | 0.1971 | -0.83748 | 7140 | |
| Brat1 | 0.19712 | -1.3155 | 7141 | |
| Glt8d1 | 0.19715 | -1.3152 | 7142 | |
| Rad51c | 0.19718 | -1.3136 | 7143 | |
| C2 | 0.19723 | -0.6536 | 7144 | |
| Spin4 | 0.19725 | 1.0153 | 7145 | |
| Ppan | 0.19725 | -1.3021 | 7146 | |
| 4933416I08Rik | 0.19728 | -1.1945 | 7147 | |
| Crb2 | 0.19734 | -1.0674 | 7148 | |
| Tdo2 | 0.19737 | -0.25568 | 7149 | |
| Bpifb9b | 0.19738 | -1.3123 | 7150 | |
| Uqcrc1 | 0.1974 | -1.3129 | 7151 | |
| Gpr123 | 0.19742 | -1.307 | 7152 | |
| Hsd17b14 | 0.19748 | -0.31405 | 7153 | |
| Cpxm2 | 0.19752 | -0.49945 | 7154 | |
| Taok3 | 0.19756 | -0.43462 | 7155 | |
| mmu-mir-181b-2 | 0.1976 | -1.3114 | 7156 | |
| Samhd1 | 0.19764 | -1.3091 | 7157 | |
| Mc4r | 0.19768 | 0.51094 | 7158 | |
| Mt2 | 0.19775 | -1.3094 | 7159 | |
| Adam26a | 0.19784 | -0.049472 | 7160 | |
| Vmn1r159 | 0.19784 | -1.0608 | 7161 | |
| Gm684 | 0.1979 | -0.28238 | 7162 | |
| Clca4 | 0.19799 | -0.23442 | 7163 | |
| Pex2 | 0.19807 | -1.184 | 7164 | |
| Zfp384 | 0.19811 | -0.095395 | 7165 | |
| Zfp804b | 0.19816 | -1.3114 | 7166 | |
| Supt5 | 0.19819 | -0.38678 | 7167 | |
| Peg3 | 0.19823 | -1.2993 | 7168 | |
| Art2a-ps | 0.19829 | -1.2085 | 7169 | |
| Cdc123 | 0.19837 | -0.74646 | 7170 | |
| Mageb1 | 0.19843 | -0.1827 | 7171 | |
| Ccrn4l | 0.19847 | -1.308 | 7172 | |
| Olfr1471 | 0.19854 | 0.093788 | 7173 | |
| Dhh | 0.19858 | 0.11741 | 7174 | |
| Sf1 | 0.19862 | 0.016119 | 7175 | |
| Rab26 | 0.1987 | 0.41679 | 7176 | |
| mmu-mir-7236 | 0.19871 | -1.0466 | 7177 | |
| Olfr1264 | 0.19874 | -1.1371 | 7178 | |
| Gtpbp1 | 0.19882 | 0.47164 | 7179 | |
| mmu-mir-3079 | 0.19887 | -0.75589 | 7180 | |
| Zfp459 | 0.1989 | -0.61242 | 7181 | |
| Trim38 | 0.19894 | -0.50018 | 7182 | |
| Zfp958 | 0.19901 | -1.3071 | 7183 | |
| Usp29 | 0.19909 | -1.2995 | 7184 | |
| Mmp14 | 0.19913 | 1.1525 | 7185 | |
| Vmn2r112 | 0.19941 | -0.682 | 7186 | |
| Ttc8 | 0.19948 | -1.304 | 7187 | |
| Olfr884 | 0.19949 | 0.26474 | 7188 | |
| Vmn2r107 | 0.19954 | -0.48822 | 7189 | |
| H2afj | 0.1996 | -0.35521 | 7190 | |
| Fnd3c2 | 0.19963 | -1.303 | 7191 | |
| Ttc39c | 0.19964 | -0.23589 | 7192 | |
| Susd5 | 0.19965 | -1.2069 | 7193 | |
| Hist1h2ai | 0.19967 | -1.2777 | 7194 | |
| Gm10220 | 0.19971 | 0.28759 | 7195 | |
| mmu-mir-204 | 0.19971 | -0.85851 | 7196 | |
| Wfdc11 | 0.19976 | 0.16302 | 7197 | |
| Slfn1 | 0.1998 | -0.91249 | 7198 | |
| Gm13308 | 0.19984 | 1.164 | 7199 | |
| Heph | 0.19996 | -0.36907 | 7200 | |
| Tmem150a | 0.19998 | -1.2973 | 7201 | |
| Gzmk | 0.2 | -0.064636 | 7202 | |
| Pik3r2 | 0.20028 | -0.59149 | 7203 | |
| Krt15 | 0.20031 | -0.90207 | 7204 | |
| Ssx2ip | 0.20031 | -1.3031 | 7205 | |
| Nab2 | 0.20035 | -1.0916 | 7206 | |
| Plekha3 | 0.20036 | -1.261 | 7207 | |
| Dynlt3 | 0.20039 | -0.39478 | 7208 | |
| Nacad | 0.20043 | -0.60352 | 7209 | |
| 4933402N22Rik | 0.20049 | -1.2996 | 7210 | |
| Snph | 0.20051 | -0.12135 | 7211 | |
| Tbc1d12 | 0.20059 | -0.16872 | 7212 | |
| Tnfaip1 | 0.20072 | -1.298 | 7213 | |
| Rhox2b | 0.20075 | -1.7611 | 7214 | |
| Zbtb2 | 0.20077 | -1.2662 | 7215 | |
| mmu-mir-496b | 0.20079 | -0.33625 | 7216 | |
| Zfp248 | 0.20083 | -1.0896 | 7217 | |
| Tmem74 | 0.20088 | -0.14896 | 7218 | |
| Thap2 | 0.2009 | 0.23783 | 7219 | |
| Zfp109 | 0.20097 | -1.2967 | 7220 | |
| Vmn2r79 | 0.20106 | -1.2904 | 7221 | |
| Smim14 | 0.20114 | -0.49263 | 7222 | |
| Kif6 | 0.20123 | -1.2947 | 7223 | |
| Hk3 | 0.20129 | 0.69057 | 7224 | |
| mmu-mir-384 | 0.20133 | -0.5489 | 7225 | |
| mmu-mir-99a | 0.20138 | -0.58767 | 7226 | |
| Rab11fip2 | 0.2014 | -1.2241 | 7227 | |
| Slc35e1 | 0.20151 | -0.90755 | 7228 | |
| Slc39a7 | 0.2016 | -0.47232 | 7229 | |
| Cwc22 | 0.2016 | -0.92363 | 7230 | |
| Glod5 | 0.2016 | -0.50437 | 7231 | |
| Nbeal1 | 0.2016 | -0.54706 | 7232 | |
| Tomm5 | 0.2016 | -0.18709 | 7233 | |
| Usp43 | 0.20166 | -1.263 | 7234 | |
| mmu-mir-190b | 0.20167 | -0.75344 | 7235 | |
| Dnase1l1 | 0.20172 | -1.296 | 7236 | |
| Astl | 0.20181 | -0.81823 | 7237 | |
| Gm933 | 0.20184 | -0.12146 | 7238 | |
| Hfe | 0.20189 | -0.92127 | 7239 | |
| Cxcr4 | 0.20196 | -0.60786 | 7240 | |
| Gm6890 | 0.202 | -0.81694 | 7241 | |
| Nmb | 0.20204 | -1.1554 | 7242 | |
| Galnt6 | 0.20207 | -0.19228 | 7243 | |
| Fbl | 0.20212 | -1.2931 | 7244 | |
| 2310009B15Rik | 0.20223 | -0.84274 | 7245 | |
| Dctn1 | 0.20227 | -1.1782 | 7246 | |
| Ssr4 | 0.20232 | -0.96197 | 7247 | |
| mmu-mir-1224 | 0.20234 | -0.74609 | 7248 | |
| Gm362 | 0.20239 | -0.029061 | 7249 | |
| Gm3376 | 0.20242 | -0.63244 | 7250 | |
| Dlc1 | 0.20243 | 2.2345 | 7251 | |
| Nlrp10 | 0.20245 | -1.2943 | 7252 | |
| mmu-mir-7b | 0.20252 | -0.79521 | 7253 | |
| Fam181a | 0.20254 | -1.2909 | 7254 | |
| Trappc3l | 0.20262 | 0.71589 | 7255 | |
| Mertk | 0.20266 | 0.072199 | 7256 | |
| Selt | 0.20266 | -1.2927 | 7257 | |
| Chn1 | 0.2027 | -1.2919 | 7258 | |
| mmu-mir-7115 | 0.20273 | -0.56441 | 7259 | |
| Ccdc166 | 0.20274 | -1.1836 | 7260 | |
| mmu-mir-466b-3 | 0.20291 | -1.4023 | 7261 | |
| Adam6b | 0.20295 | -0.49721 | 7262 | |
| mmu-mir-3475 | 0.20302 | -0.88838 | 7263 | |
| 0610010B08Rik | 0.20305 | -0.12323 | 7264 | |
| Nrxn1 | 0.20309 | -0.77323 | 7265 | |
| Klra9 | 0.20313 | -1.0194 | 7266 | |
| Gm2042 | 0.2032 | -1.2853 | 7267 | |
| mmu-mir-29b-1 | 0.20321 | -0.98875 | 7268 | |
| Ndst2 | 0.20321 | -1.2913 | 7269 | |
| Stab1 | 0.20325 | 0.45457 | 7270 | |
| Vps45 | 0.20329 | -1.2913 | 7271 | |
| Kcnn1 | 0.20333 | -0.041245 | 7272 | |
| Slc17a8 | 0.20338 | -1.2862 | 7273 | |
| Vmn2r60 | 0.2034 | -1.2671 | 7274 | |
| Olfr1016 | 0.20346 | -0.39501 | 7275 | |
| Nedd4 | 0.20348 | -0.59189 | 7276 | |
| Nckipsd | 0.2035 | -1.2795 | 7277 | |
| Spink4 | 0.20351 | -0.71786 | 7278 | |
| Gm5382 | 0.20352 | -1.1412 | 7279 | |
| Gtpbp6 | 0.20353 | -1.2812 | 7280 | |
| Tmem25 | 0.20355 | -0.67594 | 7281 | |
| Diap2 | 0.20356 | 0.9319 | 7282 | |
| mmu-mir-5119 | 0.20358 | -0.45737 | 7283 | |
| Trafd1 | 0.2036 | -1.11 | 7284 | |
| Dusp11 | 0.20372 | 0.42227 | 7285 | |
| Zfp433 | 0.20376 | -1.2873 | 7286 | |
| A430078G23Rik | 0.20378 | -0.13762 | 7287 | |
| Ncln | 0.20383 | 1.285 | 7288 | |
| Cited1 | 0.20389 | -1.2833 | 7289 | |
| Slc43a1 | 0.20403 | 0.17225 | 7290 | |
| Med12l | 0.20409 | 0.13593 | 7291 | |
| Olfr809 | 0.20415 | -1.0899 | 7292 | |
| Zfp354a | 0.20415 | -1.265 | 7293 | |
| Impa1 | 0.20419 | -0.55453 | 7294 | |
| Ubtfl1 | 0.20421 | -1.2888 | 7295 | |
| Vps51 | 0.20426 | -0.4517 | 7296 | |
| Ear12 | 0.2043 | 0.12676 | 7297 | |
| Fgf20 | 0.20442 | -0.35585 | 7298 | |
| Metap1d | 0.20443 | -1.1627 | 7299 | |
| Helq | 0.20445 | -1.2838 | 7300 | |
| mmu-mir-879 | 0.20447 | -0.48512 | 7301 | |
| Ift122 | 0.20449 | -1.2791 | 7302 | |
| B9d1 | 0.20451 | -0.18621 | 7303 | |
| Mesp1 | 0.20453 | -1.2827 | 7304 | |
| Slc24a2 | 0.20457 | -1.2838 | 7305 | |
| Olfr1378 | 0.20458 | 1.6583 | 7306 | |
| Ormdl1 | 0.20458 | -1.1741 | 7307 | |
| Pcdhga12 | 0.20469 | -0.62938 | 7308 | |
| Afp | 0.20472 | -1.281 | 7309 | |
| BC048403 | 0.20473 | -0.023418 | 7310 | |
| Atp2c2 | 0.20473 | -0.73273 | 7311 | |
| Chic1 | 0.20481 | -1.2867 | 7312 | |
| mmu-mir-6355 | 0.20483 | -0.56086 | 7313 | |
| Fam19a1 | 0.20493 | -0.10015 | 7314 | |
| Olfr355 | 0.20501 | -0.82849 | 7315 | |
| Fbxo6 | 0.20502 | -1.0139 | 7316 | |
| Mterf1b | 0.20507 | -1.2851 | 7317 | |
| Gpr153 | 0.20511 | -0.49649 | 7318 | |
| Mdga2 | 0.20512 | 0.61649 | 7319 | |
| F830016B08Rik | 0.20532 | -0.11542 | 7320 | |
| Gna14 | 0.20536 | -0.68603 | 7321 | |
| Vps54 | 0.20537 | -1.2176 | 7322 | |
| Usp6nl | 0.20539 | -0.9371 | 7323 | |
| Mypn | 0.2054 | 0.51165 | 7324 | |
| Olfr1317 | 0.20555 | -0.014044 | 7325 | |
| Klhl4 | 0.20559 | -0.83385 | 7326 | |
| Timm8a2 | 0.20564 | -0.75442 | 7327 | |
| Caskin2 | 0.20567 | -1.269 | 7328 | |
| Cyp2j5 | 0.20569 | -1.2833 | 7329 | |
| Cps1 | 0.20573 | 1.2982 | 7330 | |
| Dusp7 | 0.20574 | -1.2779 | 7331 | |
| B3gat3 | 0.20574 | -0.77657 | 7332 | |
| Osbpl8 | 0.20577 | -1.2802 | 7333 | |
| Sap30l | 0.20579 | -0.36863 | 7334 | |
| St7 | 0.20587 | -0.51945 | 7335 | |
| Fabp9 | 0.20594 | -1.2142 | 7336 | |
| Zxdb | 0.20597 | -0.59337 | 7337 | |
| 1700020A23Rik | 0.20609 | -1.2797 | 7338 | |
| Top3a | 0.2061 | -1.0028 | 7339 | |
| Vmn1r185 | 0.20611 | -0.73583 | 7340 | |
| Vmn1r87 | 0.20622 | 0.16321 | 7341 | |
| Serpina1c | 0.20633 | -0.97405 | 7342 | |
| Znrf1 | 0.20637 | -0.70852 | 7343 | |
| Fbrsl1 | 0.20645 | 0.071521 | 7344 | |
| 4933402P03Rik | 0.20645 | -0.60804 | 7345 | |
| Csn1s2a | 0.20648 | -1.2791 | 7346 | |
| Tep1 | 0.20654 | -1.2791 | 7347 | |
| Rasgrf1 | 0.20656 | -1.2792 | 7348 | |
| Plcg1 | 0.20658 | -0.72524 | 7349 | |
| Hist1h2be | 0.20659 | -1.4624 | 7350 | |
| Vmn1r186 | 0.20662 | -1.2795 | 7351 | |
| Fam219b | 0.20665 | 0.77321 | 7352 | |
| mmu-mir-3966 | 0.20665 | -1.7131 | 7353 | |
| Sumo2 | 0.20669 | -0.69972 | 7354 | |
| mmu-mir-3474 | 0.20672 | -0.64153 | 7355 | |
| Rnf215 | 0.20673 | -1.2789 | 7356 | |
| Krtap19-9b | 0.20675 | -1.2794 | 7357 | |
| Pisd | 0.20676 | 0.46272 | 7358 | |
| Olfr870 | 0.2068 | -0.19514 | 7359 | |
| Sema3e | 0.20692 | 0.072396 | 7360 | |
| Gin1 | 0.207 | -0.76199 | 7361 | |
| 9930104L06Rik | 0.20704 | -0.57487 | 7362 | |
| Arel1 | 0.20715 | 1.0769 | 7363 | |
| Cd83 | 0.20719 | 0.25213 | 7364 | |
| mmu-mir-6908 | 0.20722 | -1.0057 | 7365 | |
| Slc5a4b | 0.20723 | 0.37428 | 7366 | |
| Camsap3 | 0.20724 | -1.2773 | 7367 | |
| Tpgs1 | 0.20727 | -1.2764 | 7368 | |
| Cdadc1 | 0.20729 | -1.2687 | 7369 | |
| mmu-mir-7678 | 0.20737 | -0.76996 | 7370 | |
| Cep57 | 0.20739 | -0.48213 | 7371 | |
| D730048I06Rik | 0.20744 | -1.0121 | 7372 | |
| Caskin1 | 0.20748 | -1.2689 | 7373 | |
| Pafah1b3 | 0.2075 | -1.2668 | 7374 | |
| Trpv2 | 0.20754 | -0.12033 | 7375 | |
| Prkdc | 0.20766 | -0.74069 | 7376 | |
| mmu-mir-3535 | 0.20769 | -0.67308 | 7377 | |
| Zbtb44 | 0.2077 | 1.0268 | 7378 | |
| Vtcn1 | 0.20774 | -0.69553 | 7379 | |
| Mthfs | 0.20778 | -0.51965 | 7380 | |
| Hoxb3 | 0.20782 | -0.83069 | 7381 | |
| Insig2 | 0.20786 | -0.041914 | 7382 | |
| AY761184 | 0.20786 | -0.55575 | 7383 | |
| Hist1h2bl | 0.20787 | -1.7156 | 7384 | |
| Olfr1282 | 0.2079 | -0.57277 | 7385 | |
| Col5a2 | 0.20793 | -0.47421 | 7386 | |
| 0610012H03Rik | 0.20795 | -1.2662 | 7387 | |
| Mroh4 | 0.20799 | -0.8245 | 7388 | |
| mmu-mir-3092 | 0.20802 | 0.15604 | 7389 | |
| Klf8 | 0.20805 | 0.44388 | 7390 | |
| Epb4.1l3 | 0.20813 | -0.45431 | 7391 | |
| Psap | 0.20814 | -1.2665 | 7392 | |
| Zbtb26 | 0.20818 | -0.99887 | 7393 | |
| Fam102a | 0.20825 | -0.27992 | 7394 | |
| Man1a | 0.20828 | -1.2726 | 7395 | |
| Timm8b | 0.20828 | -0.68594 | 7396 | |
| Cdk4 | 0.20836 | 0.4954 | 7397 | |
| Sik3 | 0.2084 | -0.35849 | 7398 | |
| Pcdh15 | 0.20848 | -1.271 | 7399 | |
| Fabp1 | 0.20852 | -1.2712 | 7400 | |
| Rabl5 | 0.20854 | -1.272 | 7401 | |
| Fbn2 | 0.20863 | -1.2659 | 7402 | |
| Fbxw15 | 0.20867 | -0.8273 | 7403 | |
| Gria1 | 0.20869 | -1.2666 | 7404 | |
| Entpd7 | 0.20871 | -1.2708 | 7405 | |
| mmu-mir-1247 | 0.20873 | -0.4645 | 7406 | |
| Ubc | 0.20875 | 0.0013397 | 7407 | |
| Ppp3cc | 0.20877 | -1.2709 | 7408 | |
| Pus1 | 0.20879 | -0.10324 | 7409 | |
| Ccdc92 | 0.20883 | -0.66284 | 7410 | |
| Nptn | 0.20884 | -0.52382 | 7411 | |
| Pard6a | 0.20887 | -0.71283 | 7412 | |
| Ube2m | 0.20897 | -1.2668 | 7413 | |
| Slc39a1 | 0.20898 | -0.62362 | 7414 | |
| Cntf | 0.20899 | -0.1142 | 7415 | |
| Fnip1 | 0.20906 | -0.2004 | 7416 | |
| Zfp12 | 0.20907 | -1.2647 | 7417 | |
| Nrip1 | 0.20918 | -0.24368 | 7418 | |
| Serpina3c | 0.20922 | 0.3055 | 7419 | |
| Ssb | 0.20926 | -0.41359 | 7420 | |
| Sebox | 0.20932 | 0.15841 | 7421 | |
| Olfr747 | 0.20941 | -0.70194 | 7422 | |
| Gm14139 | 0.20945 | -1.2644 | 7423 | |
| Sike1 | 0.20947 | -1.2621 | 7424 | |
| Prdm9 | 0.20957 | 0.3122 | 7425 | |
| Ddt | 0.20962 | -1.2629 | 7426 | |
| Rabgap1 | 0.20964 | -0.85018 | 7427 | |
| Muc5ac | 0.20965 | -0.070669 | 7428 | |
| Chmp3 | 0.20965 | -0.031971 | 7429 | |
| Ccne1 | 0.20966 | -0.15185 | 7430 | |
| Cilp | 0.20967 | -1.1552 | 7431 | |
| mmu-mir-7235 | 0.20969 | -1.1768 | 7432 | |
| Gmip | 0.20972 | -0.56714 | 7433 | |
| Pygl | 0.20984 | 0.57611 | 7434 | |
| Fbf1 | 0.2099 | -1.1608 | 7435 | |
| Rcan3 | 0.20992 | -0.49828 | 7436 | |
| Olfr1499 | 0.21 | -0.97974 | 7437 | |
| Ankhd1 | 0.21004 | 0.31521 | 7438 | |
| Stk25 | 0.21007 | -0.12845 | 7439 | |
| Nme1 | 0.21011 | -1.2607 | 7440 | |
| Ssh3 | 0.21011 | -0.24588 | 7441 | |
| Hps1 | 0.21013 | -1.2626 | 7442 | |
| 1700001F09Rik | 0.21015 | -0.53833 | 7443 | |
| 9930012K11Rik | 0.21019 | -0.4605 | 7444 | |
| Anxa6 | 0.21022 | -0.96845 | 7445 | |
| Gclm | 0.21023 | 0.27772 | 7446 | |
| mmu-mir-540 | 0.21026 | -0.66636 | 7447 | |
| Morn4 | 0.21026 | -0.19886 | 7448 | |
| Tcp11l2 | 0.21027 | -0.26441 | 7449 | |
| mmu-mir-7658 | 0.21028 | -0.86959 | 7450 | |
| Mafk | 0.21031 | 0.78584 | 7451 | |
| Ppfibp2 | 0.21034 | -1.2303 | 7452 | |
| Tctn1 | 0.21035 | -0.76213 | 7453 | |
| Rnaseh2c | 0.21036 | -1.2559 | 7454 | |
| Kxd1 | 0.21051 | -0.60678 | 7455 | |
| mmu-mir-466g | 0.21061 | -0.54419 | 7456 | |
| Cyb5r4 | 0.21062 | -1.256 | 7457 | |
| Stxbp2 | 0.21069 | -1.2641 | 7458 | |
| mmu-mir-3104 | 0.21069 | -0.044699 | 7459 | |
| Vmn1r238 | 0.2107 | -0.98075 | 7460 | |
| Gm5725 | 0.21081 | -1.6913 | 7461 | |
| Rrm1 | 0.21095 | -0.96538 | 7462 | |
| Clstn3 | 0.21104 | -1.2544 | 7463 | |
| Hoga1 | 0.21111 | -1.2604 | 7464 | |
| Pla2g2c | 0.21115 | -0.47972 | 7465 | |
| mmu-mir-6351 | 0.21135 | -1.1636 | 7466 | |
| Agmo | 0.21149 | -0.96892 | 7467 | |
| Gsk3b | 0.21151 | -1.2608 | 7468 | |
| Serpinb11 | 0.21155 | -1.2481 | 7469 | |
| P4htm | 0.21163 | 1.0993 | 7470 | |
| Rab3d | 0.21171 | -0.14534 | 7471 | |
| Npc1 | 0.21174 | -0.96538 | 7472 | |
| Aadat | 0.21178 | -1.2599 | 7473 | |
| B4galt3 | 0.21178 | 0.22702 | 7474 | |
| Pygo1 | 0.21184 | -1.0178 | 7475 | |
| St6gal1 | 0.21186 | -1.1574 | 7476 | |
| Dcaf10 | 0.21186 | -1.0236 | 7477 | |
| Nrsn1 | 0.2119 | 0.84119 | 7478 | |
| Bhlhb9 | 0.21198 | 0.29171 | 7479 | |
| Pcdh18 | 0.21213 | 0.17475 | 7480 | |
| Olfr332 | 0.21214 | -1.2531 | 7481 | |
| Zbtb5 | 0.21217 | -0.23211 | 7482 | |
| Ppp1r10 | 0.21225 | -1.1812 | 7483 | |
| Olfr26 | 0.21229 | -0.9692 | 7484 | |
| Pmepa1 | 0.21233 | 1.1635 | 7485 | |
| Olfr410 | 0.21239 | 0.12471 | 7486 | |
| Ppm1b | 0.21239 | -0.72615 | 7487 | |
| Siva1 | 0.21252 | -0.78443 | 7488 | |
| Lnp | 0.21256 | 0.37368 | 7489 | |
| Olfr1037 | 0.21258 | -0.60114 | 7490 | |
| Snx9 | 0.2126 | -0.35289 | 7491 | |
| Plp2 | 0.21263 | -1.1343 | 7492 | |
| 4933421I07Rik | 0.21269 | -1.2558 | 7493 | |
| Olfr860 | 0.21273 | -1.1473 | 7494 | |
| Il18 | 0.21276 | -0.74636 | 7495 | |
| Wisp2 | 0.21279 | -1.2522 | 7496 | |
| Ubxn11 | 0.21279 | -0.83645 | 7497 | |
| Ets1 | 0.21282 | -0.50223 | 7498 | |
| mmu-mir-135b | 0.21289 | -0.77802 | 7499 | |
| Krt20 | 0.21292 | -1.2375 | 7500 | |
| Dvl1 | 0.21294 | -1.2555 | 7501 | |
| Kat6a | 0.21295 | 0.38076 | 7502 | |
| Ceacam1 | 0.21303 | -0.085003 | 7503 | |
| 1110032A03Rik | 0.21307 | -0.51392 | 7504 | |
| Fbxw17 | 0.21311 | -1.2549 | 7505 | |
| Lce1h | 0.21318 | -0.50216 | 7506 | |
| Ripply3 | 0.21322 | -1.0531 | 7507 | |
| mmu-mir-192 | 0.21325 | -0.17336 | 7508 | |
| mmu-mir-3063 | 0.21331 | 0.031771 | 7509 | |
| Herc2 | 0.21332 | -1.137 | 7510 | |
| Foxi1 | 0.21341 | 1.4214 | 7511 | |
| Scnm1 | 0.21347 | -1.2485 | 7512 | |
| Tubgcp5 | 0.21357 | -1.2379 | 7513 | |
| Ager | 0.21361 | -0.48045 | 7514 | |
| Cnga1 | 0.21365 | 1.0785 | 7515 | |
| Hif3a | 0.21372 | -0.17818 | 7516 | |
| Tmem141 | 0.21376 | 0.45853 | 7517 | |
| mmu-mir-30c-2 | 0.21378 | -0.67807 | 7518 | |
| Prmt10 | 0.2138 | -0.06976 | 7519 | |
| Smc1b | 0.21381 | -0.73802 | 7520 | |
| Nlgn2 | 0.21385 | -0.5785 | 7521 | |
| Mmp17 | 0.21388 | 1.5321 | 7522 | |
| Dhx15 | 0.21388 | -1.2523 | 7523 | |
| Ccng1 | 0.21392 | -0.63916 | 7524 | |
| Zfp663 | 0.21393 | -1.252 | 7525 | |
| 6330408A02Rik | 0.21396 | 0.30731 | 7526 | |
| Dync1h1 | 0.214 | -0.23613 | 7527 | |
| Gpr101 | 0.21404 | -1.2496 | 7528 | |
| Stip1 | 0.21407 | 0.077681 | 7529 | |
| Bbs9 | 0.21418 | -0.87009 | 7530 | |
| Cdkn1b | 0.21423 | -0.37951 | 7531 | |
| mmu-mir-149 | 0.21424 | 0.20773 | 7532 | |
| Spata6 | 0.21435 | 0.38817 | 7533 | |
| Nqo2 | 0.21436 | -1.2502 | 7534 | |
| Ppargc1a | 0.21436 | -0.4046 | 7535 | |
| Chrm2 | 0.21438 | -0.23333 | 7536 | |
| Olfr461 | 0.21439 | -0.33541 | 7537 | |
| Fam167b | 0.21442 | 0.90414 | 7538 | |
| Spt1 | 0.21442 | -1.2478 | 7539 | |
| Slc30a4 | 0.21458 | -1.2442 | 7540 | |
| Arv1 | 0.21458 | 0.85384 | 7541 | |
| Nkain4 | 0.21479 | -0.73375 | 7542 | |
| R3hdm4 | 0.21481 | -1.1498 | 7543 | |
| Cyp1b1 | 0.21494 | -0.071688 | 7544 | |
| Baz2b | 0.21495 | 0.1089 | 7545 | |
| Adora1 | 0.21496 | -0.54683 | 7546 | |
| C87436 | 0.215 | -0.66775 | 7547 | |
| Dus1l | 0.21502 | -1.2455 | 7548 | |
| Mbd5 | 0.21504 | -0.26532 | 7549 | |
| Ackr4 | 0.21508 | 0.52401 | 7550 | |
| Cyp4a29-ps | 0.21516 | 0.44923 | 7551 | |
| Pet2 | 0.21526 | -0.66924 | 7552 | |
| Lrrc24 | 0.21527 | -0.78127 | 7553 | |
| Adcy7 | 0.21531 | -0.007682 | 7554 | |
| mmu-mir-7052 | 0.21536 | -0.17594 | 7555 | |
| Gm7120 | 0.21536 | -1.2455 | 7556 | |
| Tmem177 | 0.21539 | -0.62061 | 7557 | |
| Rab4a | 0.2154 | -1.2394 | 7558 | |
| Grrp1 | 0.21555 | -1.0046 | 7559 | |
| Cenph | 0.21555 | -1.2405 | 7560 | |
| Tas1r2 | 0.21557 | -1.2429 | 7561 | |
| Hypk | 0.21558 | -0.38975 | 7562 | |
| Veph1 | 0.21559 | -0.41465 | 7563 | |
| Slc16a4 | 0.21562 | -0.46107 | 7564 | |
| Ubald1 | 0.21567 | -1.171 | 7565 | |
| Raet1d | 0.21573 | -1.6626 | 7566 | |
| Gatsl3 | 0.21573 | -1.2442 | 7567 | |
| Fam170a | 0.21576 | -0.14617 | 7568 | |
| Hoxd11 | 0.21581 | -1.2434 | 7569 | |
| Adipor1 | 0.21582 | 1.2896 | 7570 | |
| Fst | 0.21586 | -0.45719 | 7571 | |
| Tusc2 | 0.21595 | -1.2006 | 7572 | |
| Mettl18 | 0.21603 | -1.2442 | 7573 | |
| Dph7 | 0.21609 | -0.85256 | 7574 | |
| Kcnk10 | 0.21613 | -1.0781 | 7575 | |
| Ddx39b | 0.21616 | -0.89544 | 7576 | |
| Naaladl1 | 0.21617 | 0.69127 | 7577 | |
| Kif1c | 0.2162 | -1.061 | 7578 | |
| Chml | 0.21622 | -1.2326 | 7579 | |
| Mmp28 | 0.21624 | -0.83398 | 7580 | |
| Cyp2ab1 | 0.21628 | -0.69898 | 7581 | |
| Olfr61 | 0.2163 | -1.2407 | 7582 | |
| Akr1c12 | 0.21636 | 0.35622 | 7583 | |
| Tmem191c | 0.2164 | -0.12941 | 7584 | |
| C2cd4c | 0.2164 | -1.2372 | 7585 | |
| Fer1l5 | 0.21643 | -0.445 | 7586 | |
| Grm1 | 0.21644 | -0.8596 | 7587 | |
| Ubiad1 | 0.21647 | -1.2352 | 7588 | |
| Ankrd34b | 0.21647 | -1.1792 | 7589 | |
| Poli | 0.21649 | -0.26461 | 7590 | |
| Lipi | 0.21651 | 0.97187 | 7591 | |
| Fkbp14 | 0.21652 | -1.2413 | 7592 | |
| Bnipl | 0.21655 | -0.29547 | 7593 | |
| Vmn1r65 | 0.21655 | -0.098472 | 7594 | |
| Ppp1r3f | 0.2166 | -1.2315 | 7595 | |
| 4930447C04Rik | 0.21666 | -1.2415 | 7596 | |
| Klk11 | 0.21687 | -1.2005 | 7597 | |
| Klf15 | 0.2169 | -0.93062 | 7598 | |
| Derl1 | 0.21699 | -1.2028 | 7599 | |
| Tmem183a | 0.21702 | 0.51806 | 7600 | |
| Gzmm | 0.21705 | -0.56309 | 7601 | |
| Vac14 | 0.21709 | -0.64764 | 7602 | |
| Eif4g1 | 0.21717 | -1.1902 | 7603 | |
| Il1b | 0.21721 | 1.2085 | 7604 | |
| Zfp62 | 0.21722 | -1.1284 | 7605 | |
| mmu-mir-6411 | 0.21725 | -0.27103 | 7606 | |
| Trmt11 | 0.21725 | -0.98124 | 7607 | |
| Prkab1 | 0.21727 | -0.92136 | 7608 | |
| Usp11 | 0.21727 | -1.2391 | 7609 | |
| Mpp3 | 0.21741 | -0.9675 | 7610 | |
| Dnal4 | 0.2175 | -1.2373 | 7611 | |
| Ranbp9 | 0.21752 | -0.4057 | 7612 | |
| Olfr1352 | 0.2176 | 0.47298 | 7613 | |
| mmu-mir-3058 | 0.2176 | -0.55553 | 7614 | |
| 1700016D06Rik | 0.21762 | -0.84336 | 7615 | |
| Dgke | 0.21763 | -1.2219 | 7616 | |
| Serpinb1b | 0.21767 | -1.1694 | 7617 | |
| Csad | 0.21771 | -1.2357 | 7618 | |
| Slx4 | 0.21775 | -0.81592 | 7619 | |
| Krtap16-3 | 0.21783 | 1.7012 | 7620 | |
| mmu-mir-101c | 0.21783 | -1.2369 | 7621 | |
| Memo1 | 0.21787 | -1.2332 | 7622 | |
| Esx1 | 0.21792 | -1.2342 | 7623 | |
| Magohb | 0.21798 | -0.40667 | 7624 | |
| mmu-mir-468 | 0.218 | -0.89235 | 7625 | |
| Pon1 | 0.218 | -0.65054 | 7626 | |
| Suco | 0.21802 | 0.53134 | 7627 | |
| Gcat | 0.21806 | -1.2328 | 7628 | |
| mmu-mir-467h | 0.2181 | 0.0040863 | 7629 | |
| Otud7a | 0.21824 | -1.2353 | 7630 | |
| Litaf | 0.21827 | -1.2318 | 7631 | |
| Cdkl1 | 0.21831 | -1.2239 | 7632 | |
| Slc29a2 | 0.2184 | -1.1225 | 7633 | |
| Sprr4 | 0.21841 | 0.087757 | 7634 | |
| Dydc1 | 0.2185 | -1.2319 | 7635 | |
| Olfr153 | 0.21856 | 0.11109 | 7636 | |
| Defa22 | 0.21856 | -0.6242 | 7637 | |
| Bend3 | 0.21863 | -0.60022 | 7638 | |
| Tstd3 | 0.21871 | -0.041227 | 7639 | |
| Esco1 | 0.21875 | -0.20297 | 7640 | |
| Lrrtm2 | 0.21883 | -0.020521 | 7641 | |
| Cth | 0.21891 | -0.38483 | 7642 | |
| mmu-mir-3083 | 0.21894 | -0.57501 | 7643 | |
| Ranbp2 | 0.21896 | -1.0995 | 7644 | |
| Tro | 0.21899 | 1.229 | 7645 | |
| Armc10 | 0.21903 | -0.91315 | 7646 | |
| Nppc | 0.21904 | -0.029129 | 7647 | |
| Whsc1 | 0.21906 | -0.42609 | 7648 | |
| Tbc1d20 | 0.21912 | -0.79533 | 7649 | |
| Reep1 | 0.21915 | -1.2311 | 7650 | |
| mmu-mir-432 | 0.21917 | -0.35786 | 7651 | |
| Hs3st2 | 0.21917 | -0.51919 | 7652 | |
| Ilk | 0.21918 | -0.90643 | 7653 | |
| Zfp786 | 0.21923 | -0.89291 | 7654 | |
| Akr1e1 | 0.21927 | -1.2304 | 7655 | |
| Lrit3 | 0.2193 | 0.15363 | 7656 | |
| Hnrnpa0 | 0.21934 | -1.2311 | 7657 | |
| Rpain | 0.21937 | -0.72127 | 7658 | |
| Wfdc3 | 0.21937 | -0.69455 | 7659 | |
| Tmem5 | 0.2194 | -1.231 | 7660 | |
| Mtf1 | 0.21944 | -0.50932 | 7661 | |
| Brinp1 | 0.21946 | -1.2252 | 7662 | |
| Zfx | 0.21948 | -1.2256 | 7663 | |
| Olfr828 | 0.21953 | -0.32456 | 7664 | |
| Arid3b | 0.21954 | -1.0058 | 7665 | |
| Baz1a | 0.21957 | -0.66159 | 7666 | |
| Xpot | 0.2196 | -0.94739 | 7667 | |
| Glycam1 | 0.21963 | -1.2271 | 7668 | |
| Hsd3b2 | 0.21965 | -1.2294 | 7669 | |
| Dysf | 0.21974 | 0.0078277 | 7670 | |
| Ypel1 | 0.21974 | 1.3967 | 7671 | |
| Atp5g2 | 0.2198 | 0.44164 | 7672 | |
| Tsen34 | 0.21984 | -0.73136 | 7673 | |
| Wbp5 | 0.21987 | -0.86378 | 7674 | |
| Itfg3 | 0.2199 | -0.89003 | 7675 | |
| Olfr1120 | 0.21992 | -1.2273 | 7676 | |
| Opn4 | 0.21996 | -1.1196 | 7677 | |
| 2610034M16Rik | 0.21999 | -1.2241 | 7678 | |
| Gm20877 | 0.22003 | -0.50036 | 7679 | |
| Eif2b3 | 0.22011 | -0.64229 | 7680 | |
| Aoc1 | 0.22016 | 0.33356 | 7681 | |
| Lzts2 | 0.22032 | -1.2266 | 7682 | |
| Col6a5 | 0.22041 | -0.61093 | 7683 | |
| Mylip | 0.22045 | 0.42932 | 7684 | |
| Rnf144a | 0.22049 | -1.0026 | 7685 | |
| Btk | 0.22058 | -1.2272 | 7686 | |
| Tgfb1i1 | 0.22059 | -0.51783 | 7687 | |
| mmu-mir-6376 | 0.22061 | -0.36436 | 7688 | |
| 7420461P10Rik | 0.22061 | 0.86904 | 7689 | |
| Spata18 | 0.22071 | -1.2269 | 7690 | |
| Htr1d | 0.22073 | -1.2144 | 7691 | |
| mmu-mir-151 | 0.22074 | -1.1356 | 7692 | |
| Tceb3 | 0.2208 | -0.26783 | 7693 | |
| Hbegf | 0.22081 | -1.2218 | 7694 | |
| Eif3b | 0.22084 | 0.97585 | 7695 | |
| Map7 | 0.22086 | -0.62942 | 7696 | |
| Slc6a12 | 0.22096 | -1.2214 | 7697 | |
| Gm5535 | 0.22101 | -1.2226 | 7698 | |
| Chmp6 | 0.22117 | 0.35891 | 7699 | |
| Bap1 | 0.22125 | -1.2248 | 7700 | |
| Rprd1a | 0.2213 | -1.2242 | 7701 | |
| mmu-mir-15a | 0.22137 | 0.26591 | 7702 | |
| Zfp937 | 0.2214 | -1.2211 | 7703 | |
| Zfp954 | 0.22145 | -0.14943 | 7704 | |
| Samm50 | 0.2215 | -1.2223 | 7705 | |
| Rbfa | 0.22171 | -1.2236 | 7706 | |
| Exoc2 | 0.22171 | -1.2236 | 7707 | |
| Eps8l2 | 0.22172 | 1.1505 | 7708 | |
| Esp1 | 0.22184 | -0.67741 | 7709 | |
| Wdr45 | 0.22192 | 0.93959 | 7710 | |
| Mdm1 | 0.22194 | -0.69629 | 7711 | |
| Olfr76 | 0.22199 | -0.45253 | 7712 | |
| mmu-mir-1906-2 | 0.222 | -1.1582 | 7713 | |
| Alkbh6 | 0.22203 | -0.34118 | 7714 | |
| Dag1 | 0.22204 | -1.206 | 7715 | |
| B230118H07Rik | 0.22208 | -1.2179 | 7716 | |
| Odf3l2 | 0.22211 | -1.0148 | 7717 | |
| Olfr1495 | 0.22216 | -1.2219 | 7718 | |
| Plce1 | 0.22227 | -1.2179 | 7719 | |
| Grn | 0.22229 | -1.2192 | 7720 | |
| Hrh1 | 0.2223 | 0.60169 | 7721 | |
| Tas2r116 | 0.22233 | -1.2201 | 7722 | |
| Lrpap1 | 0.22238 | -0.94286 | 7723 | |
| S100a9 | 0.22245 | -1.2189 | 7724 | |
| Slc13a1 | 0.22248 | -1.2165 | 7725 | |
| Robo2 | 0.22249 | 1.2932 | 7726 | |
| mmu-mir-6980 | 0.22259 | -0.774 | 7727 | |
| Popdc3 | 0.22262 | -0.12355 | 7728 | |
| Slc5a7 | 0.22274 | -0.62053 | 7729 | |
| Nup85 | 0.22275 | -0.40293 | 7730 | |
| Gm5595 | 0.22284 | -0.29988 | 7731 | |
| Cmss1 | 0.22288 | 0.23651 | 7732 | |
| Xlr5b | 0.22289 | -1.6187 | 7733 | |
| Pla2g4c | 0.22299 | -0.29455 | 7734 | |
| Wnt7a | 0.223 | -1.2166 | 7735 | |
| Olfr69 | 0.22314 | -1.2167 | 7736 | |
| Hyal4 | 0.22326 | 0.24929 | 7737 | |
| Best1 | 0.22326 | 0.29516 | 7738 | |
| Htr5b | 0.22327 | -1.2039 | 7739 | |
| Rnf187 | 0.22335 | -1.2129 | 7740 | |
| Slc22a30 | 0.22342 | 1.8346 | 7741 | |
| Tnfaip2 | 0.22355 | -1.207 | 7742 | |
| Slc26a7 | 0.22357 | 0.33954 | 7743 | |
| Ube2d2a | 0.2236 | -1.0481 | 7744 | |
| Stard5 | 0.22362 | -1.2132 | 7745 | |
| Fyttd1 | 0.22372 | -1.2155 | 7746 | |
| Postn | 0.2238 | -0.21038 | 7747 | |
| Hist1h3i | 0.2238 | -0.5936 | 7748 | |
| Olfr452 | 0.22384 | -0.15306 | 7749 | |
| Mcpt4 | 0.22388 | 1.9249 | 7750 | |
| Bbc3 | 0.22389 | -1.2065 | 7751 | |
| Ifitm7 | 0.22392 | -0.34543 | 7752 | |
| Olfr830 | 0.22403 | -0.86355 | 7753 | |
| Cstad | 0.22404 | -1.214 | 7754 | |
| Gm5797 | 0.22407 | -0.98062 | 7755 | |
| Iltifb | 0.22409 | -1.2127 | 7756 | |
| Olfr513 | 0.22411 | -1.2114 | 7757 | |
| Lyrm7 | 0.22411 | 1.0024 | 7758 | |
| Psd2 | 0.22422 | -1.2143 | 7759 | |
| Mageb5 | 0.22446 | -0.69236 | 7760 | |
| Gm5168 | 0.22449 | -0.58584 | 7761 | |
| Ino80d | 0.22455 | -1.016 | 7762 | |
| Ces3a | 0.22461 | 0.0058987 | 7763 | |
| Atp6v0e2 | 0.22465 | -1.0451 | 7764 | |
| Nlrp9a | 0.22465 | -0.34377 | 7765 | |
| Patz1 | 0.22468 | -0.27503 | 7766 | |
| Cd160 | 0.22472 | 0.99407 | 7767 | |
| Lrrtm4 | 0.22476 | -0.7966 | 7768 | |
| Gm4791 | 0.2248 | -1.2112 | 7769 | |
| Cyp2b13 | 0.22482 | -0.98947 | 7770 | |
| Rgs18 | 0.22489 | -0.53642 | 7771 | |
| Fam65b | 0.225 | -1.2077 | 7772 | |
| Vmn2r16 | 0.22505 | -1.2087 | 7773 | |
| mmu-mir-331 | 0.22507 | -0.88044 | 7774 | |
| 1700029F12Rik | 0.22511 | -1.1963 | 7775 | |
| Nov | 0.22521 | -0.048245 | 7776 | |
| Cox16 | 0.22526 | -1.1771 | 7777 | |
| Dido1 | 0.22529 | -0.93087 | 7778 | |
| Rxfp3 | 0.22534 | -0.38361 | 7779 | |
| Trappc10 | 0.22535 | -1.2044 | 7780 | |
| Bnip1 | 0.22538 | -0.23351 | 7781 | |
| Chad | 0.22538 | -0.8128 | 7782 | |
| Osbpl11 | 0.22549 | 0.22707 | 7783 | |
| Pcp4 | 0.2255 | -1.2007 | 7784 | |
| Park2 | 0.22552 | -0.26729 | 7785 | |
| Pcdha5 | 0.22553 | -0.82518 | 7786 | |
| Gjb2 | 0.22554 | -1.2057 | 7787 | |
| Insc | 0.22563 | -0.94701 | 7788 | |
| Mettl24 | 0.22568 | -0.26743 | 7789 | |
| Tet1 | 0.22571 | -0.53378 | 7790 | |
| Nefh | 0.22573 | -0.56068 | 7791 | |
| Olfr1111 | 0.22575 | -1.2075 | 7792 | |
| Jmy | 0.2258 | 0.11049 | 7793 | |
| Marveld1 | 0.22589 | -1.1955 | 7794 | |
| Mief1 | 0.22591 | -0.36508 | 7795 | |
| Oas1f | 0.22594 | -1.202 | 7796 | |
| Senp7 | 0.22599 | -0.65062 | 7797 | |
| Hormad2 | 0.22604 | -0.17376 | 7798 | |
| Nol4 | 0.22606 | -1.2064 | 7799 | |
| 4933412E24Rik | 0.22607 | -0.11201 | 7800 | |
| Tnni1 | 0.22608 | -1.202 | 7801 | |
| Naip6 | 0.22614 | 0.12541 | 7802 | |
| Olfr983 | 0.22615 | -1.2042 | 7803 | |
| Gm13102 | 0.22615 | -0.49245 | 7804 | |
| mmu-mir-29c | 0.22627 | -0.7482 | 7805 | |
| Eci2 | 0.22633 | -1.1978 | 7806 | |
| Olfr1178 | 0.22637 | -0.39772 | 7807 | |
| Cyp11b1 | 0.2264 | -1.197 | 7808 | |
| Rilpl1 | 0.22649 | 1.7715 | 7809 | |
| Strc | 0.22653 | -0.086845 | 7810 | |
| mmu-mir-214 | 0.22653 | -0.36919 | 7811 | |
| Nomo1 | 0.22664 | -1.0807 | 7812 | |
| Col9a3 | 0.22666 | -1.2042 | 7813 | |
| Rpa3 | 0.22672 | -1.2041 | 7814 | |
| Scd3 | 0.2268 | 1.1756 | 7815 | |
| Smyd1 | 0.2268 | -0.37712 | 7816 | |
| Olfr460 | 0.22685 | -0.22997 | 7817 | |
| Senp3 | 0.22691 | 0.94147 | 7818 | |
| Pfn2 | 0.22695 | -0.0093076 | 7819 | |
| Tbx1 | 0.22698 | -0.95846 | 7820 | |
| Eva1c | 0.22707 | 0.017397 | 7821 | |
| 4931406B18Rik | 0.22707 | -1.1934 | 7822 | |
| mmu-mir-6998 | 0.22707 | -0.69052 | 7823 | |
| Ephx2 | 0.2271 | -0.51224 | 7824 | |
| Tsks | 0.22722 | -0.25235 | 7825 | |
| Sall4 | 0.22725 | -1.1965 | 7826 | |
| Prss46 | 0.22733 | -0.22445 | 7827 | |
| Cgrrf1 | 0.22734 | -0.86837 | 7828 | |
| Nlrc3 | 0.22739 | -0.43628 | 7829 | |
| Ap1ar | 0.22744 | -1.1986 | 7830 | |
| Nr1h3 | 0.22745 | 1.3879 | 7831 | |
| Iffo1 | 0.22749 | -1.1955 | 7832 | |
| Syncrip | 0.22762 | -1.1999 | 7833 | |
| Ang6 | 0.22764 | -0.72265 | 7834 | |
| mmu-mir-6909 | 0.22766 | -0.81208 | 7835 | |
| Zfp664 | 0.22768 | -0.22375 | 7836 | |
| Nepn | 0.22779 | 0.29964 | 7837 | |
| Erlec1 | 0.22787 | -1.1997 | 7838 | |
| Cfl1 | 0.22795 | -1.191 | 7839 | |
| Katnbl1 | 0.22798 | -0.9945 | 7840 | |
| Nat1 | 0.22808 | -0.67427 | 7841 | |
| Adck4 | 0.2281 | 1.2349 | 7842 | |
| Hsdl1 | 0.2281 | -1.1942 | 7843 | |
| Fes | 0.22822 | -1.1974 | 7844 | |
| Cbll1 | 0.22829 | -0.84755 | 7845 | |
| Cox6c | 0.22855 | -1.1896 | 7846 | |
| Nudt8 | 0.22857 | -0.81794 | 7847 | |
| Cnnm1 | 0.22862 | -0.2268 | 7848 | |
| Lsamp | 0.22869 | -0.34753 | 7849 | |
| Stil | 0.22871 | -1.1909 | 7850 | |
| 1700129C05Rik | 0.22873 | -1.1897 | 7851 | |
| 6430548M08Rik | 0.22875 | -0.036205 | 7852 | |
| Tssk5 | 0.22883 | -0.47938 | 7853 | |
| Uqcrh | 0.22892 | -1.1566 | 7854 | |
| Dnah5 | 0.22894 | 0.5117 | 7855 | |
| mmu-mir-741 | 0.22897 | -0.7689 | 7856 | |
| Tspan1 | 0.22902 | -0.56156 | 7857 | |
| Dhfr | 0.22908 | -1.0588 | 7858 | |
| Olfr853 | 0.22912 | -1.1901 | 7859 | |
| Pdia4 | 0.22917 | -0.0043563 | 7860 | |
| Fbxo22 | 0.22922 | -1.1888 | 7861 | |
| Slc38a11 | 0.22925 | -0.91658 | 7862 | |
| Tor2a | 0.22927 | -0.17634 | 7863 | |
| Rdh9 | 0.22929 | 0.037098 | 7864 | |
| Nim1 | 0.22932 | 0.19898 | 7865 | |
| mmu-mir-6397 | 0.22944 | -0.40122 | 7866 | |
| Ldlrad4 | 0.22948 | -0.95926 | 7867 | |
| Dxo | 0.22956 | -1.1922 | 7868 | |
| Ces3b | 0.22959 | -0.08194 | 7869 | |
| Krtap1-5 | 0.22963 | 0.71534 | 7870 | |
| 1700055N04Rik | 0.22967 | 0.55514 | 7871 | |
| Ankrd23 | 0.22971 | -0.1041 | 7872 | |
| Fndc8 | 0.22976 | -1.1851 | 7873 | |
| Mrpl28 | 0.2299 | -1.1851 | 7874 | |
| Kctd14 | 0.2299 | -0.42062 | 7875 | |
| Gdi1 | 0.22991 | -0.37986 | 7876 | |
| Olfr154 | 0.22997 | 0.19203 | 7877 | |
| Pacs2 | 0.22999 | -1.1892 | 7878 | |
| Simc1 | 0.23001 | -0.089998 | 7879 | |
| Snai1 | 0.23003 | -1.1832 | 7880 | |
| Dbf4 | 0.23005 | -0.57944 | 7881 | |
| Adarb1 | 0.23009 | 0.50647 | 7882 | |
| BC147527 | 0.23015 | -1.1886 | 7883 | |
| Prex1 | 0.23032 | 0.074554 | 7884 | |
| P2ry1 | 0.23042 | -1.0671 | 7885 | |
| Zfp346 | 0.23044 | -1.1825 | 7886 | |
| Uchl1 | 0.23047 | -0.59062 | 7887 | |
| Htr3a | 0.23051 | -1.1855 | 7888 | |
| Edem3 | 0.23051 | -0.14402 | 7889 | |
| Bricd5 | 0.23057 | 0.8899 | 7890 | |
| Upk1a | 0.2307 | -0.91849 | 7891 | |
| Nthl1 | 0.2307 | -0.72311 | 7892 | |
| 1700011I03Rik | 0.23074 | -1.1211 | 7893 | |
| Pcsk2 | 0.23075 | -1.1779 | 7894 | |
| Fbxw4 | 0.23083 | -1.1809 | 7895 | |
| Vmn2r34 | 0.23086 | -0.91248 | 7896 | |
| Rnf13 | 0.23091 | -1.1727 | 7897 | |
| Stk39 | 0.23093 | 0.16594 | 7898 | |
| mmu-mir-7687 | 0.23095 | -0.47061 | 7899 | |
| Trdn | 0.23097 | -0.81524 | 7900 | |
| Gm14511 | 0.23101 | -1.1868 | 7901 | |
| mmu-mir-1900 | 0.23103 | -0.72695 | 7902 | |
| Tmpo | 0.23108 | -0.13223 | 7903 | |
| Zmiz1 | 0.23109 | -1.0408 | 7904 | |
| Anp32a | 0.23114 | -0.67007 | 7905 | |
| 4833423E24Rik | 0.23116 | -0.7739 | 7906 | |
| Olfr955 | 0.2312 | -0.39362 | 7907 | |
| 1700024P16Rik | 0.23122 | -1.181 | 7908 | |
| Pcdhb22 | 0.23131 | -1.1725 | 7909 | |
| mmu-mir-7213 | 0.23132 | -0.45415 | 7910 | |
| Sik1 | 0.23135 | 0.075847 | 7911 | |
| Krt76 | 0.23136 | -1.1798 | 7912 | |
| Klrc1 | 0.2314 | -1.1853 | 7913 | |
| Gm4787 | 0.23143 | 1.4244 | 7914 | |
| Uck2 | 0.23146 | -1.179 | 7915 | |
| Tm4sf1 | 0.23147 | 1.908 | 7916 | |
| Tfap2c | 0.23154 | -1.1728 | 7917 | |
| Syt13 | 0.23154 | 0.6556 | 7918 | |
| Batf | 0.23155 | -1.185 | 7919 | |
| Mfng | 0.23158 | -0.49774 | 7920 | |
| Map3k4 | 0.23165 | -1.1755 | 7921 | |
| Ptp4a1 | 0.23166 | 2.4536 | 7922 | |
| Olfr229 | 0.23166 | -0.29554 | 7923 | |
| Pabpn1l | 0.23185 | 0.21194 | 7924 | |
| Ring1 | 0.23189 | -0.00029913 | 7925 | |
| mmu-mir-6481 | 0.2319 | -0.36708 | 7926 | |
| 2310002J15Rik | 0.23198 | -1.0633 | 7927 | |
| Ptpn23 | 0.23203 | -1.1564 | 7928 | |
| Acot10 | 0.23206 | -1.183 | 7929 | |
| Amd1 | 0.23212 | -0.99838 | 7930 | |
| Mtus2 | 0.23218 | -1.1763 | 7931 | |
| Zfp661 | 0.23219 | 0.3273 | 7932 | |
| mmu-mir-6338 | 0.23222 | -0.69019 | 7933 | |
| Zbbx | 0.23224 | -0.8563 | 7934 | |
| mmu-mir-135a-2 | 0.23224 | 0.16137 | 7935 | |
| Depdc1b | 0.23227 | 1.0555 | 7936 | |
| Cyp2c68 | 0.23231 | -0.042944 | 7937 | |
| Cdk20 | 0.23236 | -0.14446 | 7938 | |
| Oaz2 | 0.2324 | -0.89857 | 7939 | |
| Fam71a | 0.23242 | -0.80831 | 7940 | |
| mmu-mir-6389 | 0.23249 | -0.47056 | 7941 | |
| Zscan18 | 0.2325 | -0.33617 | 7942 | |
| mmu-mir-466o | 0.2325 | -1.1718 | 7943 | |
| Cd244 | 0.23253 | -1.1077 | 7944 | |
| Tenc1 | 0.23259 | -1.1821 | 7945 | |
| Pdzrn4 | 0.23261 | -0.55767 | 7946 | |
| Olfr1368 | 0.23267 | -0.99314 | 7947 | |
| Sv2a | 0.23269 | 0.63831 | 7948 | |
| Fam131c | 0.23272 | -0.13571 | 7949 | |
| Olfr1443 | 0.23273 | -1.1509 | 7950 | |
| Gm14725 | 0.2329 | 0.17983 | 7951 | |
| Efcab1 | 0.2329 | -0.90704 | 7952 | |
| H2-M10.2 | 0.23294 | -1.1806 | 7953 | |
| Zfp735 | 0.23294 | -1.1806 | 7954 | |
| Tnfrsf18 | 0.23305 | -1.0203 | 7955 | |
| Hip1 | 0.23312 | -1.1762 | 7956 | |
| Lce1f | 0.23318 | -1.179 | 7957 | |
| Trp73 | 0.23322 | -1.1303 | 7958 | |
| Ctxn3 | 0.23326 | -1.1672 | 7959 | |
| Lgi4 | 0.2333 | -1.176 | 7960 | |
| Mob3c | 0.23339 | -1.1768 | 7961 | |
| Gyg | 0.23341 | 0.65303 | 7962 | |
| Foxl2 | 0.23345 | 3.4026 | 7963 | |
| Hmga1-rs1 | 0.23351 | -1.1746 | 7964 | |
| mmu-mir-206 | 0.23351 | 0.57292 | 7965 | |
| Gipr | 0.23353 | -1.1732 | 7966 | |
| AI597468 | 0.23356 | 0.38467 | 7967 | |
| Ruvbl2 | 0.23359 | -1.0199 | 7968 | |
| Cdhr2 | 0.23364 | -0.98411 | 7969 | |
| Fubp3 | 0.23365 | -1.1767 | 7970 | |
| Mettl25 | 0.23372 | -0.18584 | 7971 | |
| Mroh7 | 0.23373 | -1.1683 | 7972 | |
| Gpr182 | 0.23379 | 0.68809 | 7973 | |
| Olfr954 | 0.23383 | -0.92279 | 7974 | |
| Tmem82 | 0.23385 | -1.1697 | 7975 | |
| Prosc | 0.23387 | -0.69808 | 7976 | |
| Chp1 | 0.23388 | -1.1744 | 7977 | |
| Cyp2j11 | 0.23396 | -0.84943 | 7978 | |
| 7-Mar | 0.23402 | -0.25679 | 7979 | |
| Dph5 | 0.23404 | -1.1778 | 7980 | |
| Opa3 | 0.2341 | -0.67172 | 7981 | |
| Nudt2 | 0.23412 | -0.75291 | 7982 | |
| Trpm5 | 0.23414 | -0.95703 | 7983 | |
| Snx24 | 0.23424 | -1.0322 | 7984 | |
| Arhgap9 | 0.23428 | -0.075978 | 7985 | |
| Traf7 | 0.2343 | -1.1758 | 7986 | |
| Ndst1 | 0.2344 | -1.1762 | 7987 | |
| Prol1 | 0.23442 | 0.98131 | 7988 | |
| Rpl18 | 0.23442 | -0.56198 | 7989 | |
| Slc12a5 | 0.23445 | -1.1133 | 7990 | |
| Klhl2 | 0.23483 | -0.43499 | 7991 | |
| Dlat | 0.23486 | 0.32116 | 7992 | |
| Hdhd3 | 0.23488 | -1.1752 | 7993 | |
| Ifnk | 0.23495 | -0.23043 | 7994 | |
| H2-T24 | 0.23498 | -0.1127 | 7995 | |
| Gm13119 | 0.23516 | 0.77066 | 7996 | |
| Hhipl1 | 0.23518 | -1.1692 | 7997 | |
| Grcc10 | 0.23526 | -1.1737 | 7998 | |
| Mxra7 | 0.23528 | 2.6874 | 7999 | |
| Gal | 0.23532 | -0.9895 | 8000 | |
| Ik | 0.23535 | -0.24569 | 8001 | |
| Poln | 0.23539 | -0.51255 | 8002 | |
| Neurod2 | 0.23544 | -1.1674 | 8003 | |
| mmu-mir-466b-5 | 0.23548 | -0.81613 | 8004 | |
| Thg1l | 0.23551 | 0.2156 | 8005 | |
| B3gnt9 | 0.23553 | -0.5496 | 8006 | |
| Map3k1 | 0.23554 | -0.78693 | 8007 | |
| Smim5 | 0.23557 | -0.47266 | 8008 | |
| Mfsd7c | 0.23565 | -1.1723 | 8009 | |
| Coq7 | 0.23567 | -1.1601 | 8010 | |
| Tbx4 | 0.2357 | 0.68633 | 8011 | |
| mmu-mir-6902 | 0.23575 | 0.27023 | 8012 | |
| mmu-mir-5617 | 0.23585 | -0.7931 | 8013 | |
| Ttc9 | 0.23585 | -0.97904 | 8014 | |
| Uba3 | 0.23589 | 0.13369 | 8015 | |
| Ksr2 | 0.23589 | -0.18727 | 8016 | |
| Rhag | 0.23593 | -0.97012 | 8017 | |
| Fpgt | 0.23597 | -0.62871 | 8018 | |
| Vmn2r32 | 0.236 | -0.9404 | 8019 | |
| Zdhhc16 | 0.23604 | -0.39033 | 8020 | |
| Guk1 | 0.23606 | -0.68954 | 8021 | |
| mmu-mir-694 | 0.23619 | -0.51715 | 8022 | |
| Sptlc2 | 0.23622 | -1.1705 | 8023 | |
| Jph4 | 0.23634 | -0.33008 | 8024 | |
| mmu-mir-376c | 0.23636 | -0.8403 | 8025 | |
| mmu-mir-378a | 0.23637 | -0.76677 | 8026 | |
| Hs6st2 | 0.23638 | -0.34187 | 8027 | |
| Spef1 | 0.23638 | 0.21154 | 8028 | |
| Xirp2 | 0.23644 | -1.1692 | 8029 | |
| Olfr502 | 0.23646 | -0.495 | 8030 | |
| Clcn3 | 0.2365 | -1.1002 | 8031 | |
| Copz2 | 0.23652 | -1.1673 | 8032 | |
| Alox15 | 0.23653 | 1.8566 | 8033 | |
| Gbp2b | 0.23663 | -1.0374 | 8034 | |
| Tpcn2 | 0.23667 | -0.81696 | 8035 | |
| Klf10 | 0.23671 | -0.70981 | 8036 | |
| Osbpl7 | 0.23675 | -1.1572 | 8037 | |
| Gstm4 | 0.23676 | -0.41852 | 8038 | |
| H2-M9 | 0.23677 | -0.26198 | 8039 | |
| Gja5 | 0.23688 | 1.2465 | 8040 | |
| Olfr1330 | 0.23697 | -0.22216 | 8041 | |
| Galk1 | 0.23701 | -1.1573 | 8042 | |
| Hsh2d | 0.23705 | -1.0511 | 8043 | |
| 2610528J11Rik | 0.23705 | -0.50563 | 8044 | |
| Eno2 | 0.23707 | -1.1671 | 8045 | |
| Sycp2 | 0.23714 | 0.73605 | 8046 | |
| Ttll10 | 0.23718 | 0.40286 | 8047 | |
| Lrrc63 | 0.23725 | -0.90343 | 8048 | |
| Olfr1341 | 0.23726 | -0.37387 | 8049 | |
| Phlda2 | 0.23732 | -0.78789 | 8050 | |
| Olfr450 | 0.23736 | -1.1668 | 8051 | |
| Mrpl19 | 0.23743 | 0.18848 | 8052 | |
| Cldnd2 | 0.23744 | -1.1649 | 8053 | |
| Kcnk12 | 0.23746 | -0.28029 | 8054 | |
| Kcnma1 | 0.2375 | -1.1665 | 8055 | |
| mmu-mir-1930 | 0.23754 | -0.65858 | 8056 | |
| Glt25d1 | 0.23756 | -0.23647 | 8057 | |
| Klhl10 | 0.23758 | -1.1618 | 8058 | |
| Msantd4 | 0.23764 | -1.156 | 8059 | |
| Llgl2 | 0.23774 | -1.1548 | 8060 | |
| Rpl36al | 0.23775 | -0.24705 | 8061 | |
| Pigx | 0.23779 | -0.6417 | 8062 | |
| AF067061 | 0.23786 | -1.1623 | 8063 | |
| Cfdp1 | 0.23786 | -1.1348 | 8064 | |
| Suz12 | 0.2379 | 0.66042 | 8065 | |
| mmu-mir-7000 | 0.2379 | -0.6552 | 8066 | |
| Sepn1 | 0.23792 | -0.86828 | 8067 | |
| Ccdc155 | 0.23803 | -1.1626 | 8068 | |
| Olfr1231 | 0.23809 | 0.28878 | 8069 | |
| Tbc1d25 | 0.23809 | -0.51557 | 8070 | |
| Aqp7 | 0.23813 | -1.0105 | 8071 | |
| Armc1 | 0.23817 | -0.69602 | 8072 | |
| Zbtb4 | 0.23819 | -1.163 | 8073 | |
| Gpr55 | 0.23832 | -0.40573 | 8074 | |
| mmu-mir-5114 | 0.23832 | -0.79846 | 8075 | |
| Foxk2 | 0.23833 | -1.1636 | 8076 | |
| Evi2a | 0.23855 | -0.27309 | 8077 | |
| Sort1 | 0.23858 | -1.1343 | 8078 | |
| Olfr578 | 0.23866 | -0.82089 | 8079 | |
| Amer1 | 0.2387 | -1.1446 | 8080 | |
| Prkrir | 0.23878 | -1.1625 | 8081 | |
| Tnfrsf1a | 0.2388 | -0.64734 | 8082 | |
| Atf1 | 0.23882 | -1.1567 | 8083 | |
| Tada3 | 0.23884 | -1.1531 | 8084 | |
| Tbc1d31 | 0.23886 | -1.1569 | 8085 | |
| Olfr1388 | 0.2389 | -0.48223 | 8086 | |
| Olfr812 | 0.23892 | -1.1614 | 8087 | |
| Prl3c1 | 0.23893 | 0.40964 | 8088 | |
| Fam179a | 0.23894 | -0.6616 | 8089 | |
| Prrt4 | 0.23896 | -1.1585 | 8090 | |
| Acrv1 | 0.23896 | 0.68426 | 8091 | |
| Ttll5 | 0.239 | 0.66626 | 8092 | |
| Gad2 | 0.23904 | -1.1474 | 8093 | |
| Abi1 | 0.23908 | -0.9094 | 8094 | |
| Abca13 | 0.23912 | 0.55189 | 8095 | |
| Ppapdc1b | 0.23913 | -1.162 | 8096 | |
| Lipg | 0.23915 | 0.41746 | 8097 | |
| Atp1b4 | 0.23918 | -0.60117 | 8098 | |
| Pcsk1n | 0.23928 | -1.1564 | 8099 | |
| Rmnd1 | 0.23934 | -0.2091 | 8100 | |
| Nudt16 | 0.23936 | -1.152 | 8101 | |
| mmu-mir-5621 | 0.23936 | 0.38945 | 8102 | |
| Txlna | 0.23942 | -0.63875 | 8103 | |
| Slc6a18 | 0.23942 | -1.1568 | 8104 | |
| Maged1 | 0.23948 | 0.39022 | 8105 | |
| Zc3h12c | 0.23953 | -0.32437 | 8106 | |
| Dnase1l3 | 0.23957 | 2.6448 | 8107 | |
| Gm14430 | 0.23958 | -0.81393 | 8108 | |
| Stmn2 | 0.23959 | -0.32762 | 8109 | |
| Fam26d | 0.23961 | 0.2329 | 8110 | |
| Pkp4 | 0.23961 | -1.1443 | 8111 | |
| Got2 | 0.23964 | -0.95323 | 8112 | |
| Fndc3b | 0.23969 | -1.1528 | 8113 | |
| Zcchc17 | 0.23972 | -0.79864 | 8114 | |
| Olfr821 | 0.23973 | -1.1552 | 8115 | |
| Cdh24 | 0.23983 | -0.83702 | 8116 | |
| Nr1h5 | 0.23984 | -0.87273 | 8117 | |
| Ormdl3 | 0.23987 | -1.1545 | 8118 | |
| Abca5 | 0.23987 | -0.56071 | 8119 | |
| Smad3 | 0.23989 | -1.1581 | 8120 | |
| Mc2r | 0.23991 | 0.037254 | 8121 | |
| Fam57a | 0.23995 | -0.89828 | 8122 | |
| Slc35f5 | 0.24002 | -1.159 | 8123 | |
| Pcx | 0.24014 | -1.1575 | 8124 | |
| Tppp2 | 0.24017 | -1.158 | 8125 | |
| Mmp16 | 0.24024 | -1.153 | 8126 | |
| Siah1b | 0.24026 | -1.0295 | 8127 | |
| Pcdha9 | 0.24033 | -1.2345 | 8128 | |
| Olfr881 | 0.24037 | -1.0556 | 8129 | |
| Cxcr3 | 0.2404 | -0.83647 | 8130 | |
| Abcb6 | 0.24042 | -1.1579 | 8131 | |
| Gm906 | 0.24046 | 0.92274 | 8132 | |
| Olfr1051 | 0.24049 | -1.1579 | 8133 | |
| Cpd | 0.24054 | -1.1556 | 8134 | |
| Olfr491 | 0.24056 | 0.61673 | 8135 | |
| Ric8b | 0.2406 | -1.1505 | 8136 | |
| mmu-mir-3064 | 0.24065 | -0.63291 | 8137 | |
| Akt1 | 0.24071 | -1.0663 | 8138 | |
| Pcdhgb7 | 0.24072 | -0.89627 | 8139 | |
| Vmn1r88 | 0.24076 | -0.74814 | 8140 | |
| Ccdc113 | 0.24076 | -0.66234 | 8141 | |
| Ttc23 | 0.2408 | -0.70704 | 8142 | |
| Vmn1r74 | 0.24086 | -0.80725 | 8143 | |
| Zfp131 | 0.24086 | 1.4256 | 8144 | |
| Hist1h2bc | 0.2409 | -1.1503 | 8145 | |
| Fam160b1 | 0.24096 | -0.1423 | 8146 | |
| Fam168a | 0.24098 | -0.19085 | 8147 | |
| Tas2r126 | 0.24105 | -1.0635 | 8148 | |
| Gramd2 | 0.24112 | -1.1555 | 8149 | |
| Aff1 | 0.24114 | -1.1507 | 8150 | |
| Lypd6b | 0.24117 | -1.155 | 8151 | |
| Tmem225 | 0.24119 | -0.1167 | 8152 | |
| Olfr611 | 0.24123 | -0.87956 | 8153 | |
| Wrnip1 | 0.24124 | -0.45325 | 8154 | |
| Gm996 | 0.24125 | -1.1106 | 8155 | |
| Cml1 | 0.24131 | -0.78478 | 8156 | |
| Brpf3 | 0.24132 | -1.1546 | 8157 | |
| mmu-mir-6392 | 0.24134 | -0.31857 | 8158 | |
| Maml3 | 0.24143 | -1.0736 | 8159 | |
| Ccdc80 | 0.24147 | -0.51242 | 8160 | |
| Pcdhga2 | 0.24148 | -0.66688 | 8161 | |
| Col6a4 | 0.2415 | 0.098018 | 8162 | |
| Vmn2r72 | 0.24154 | -0.18683 | 8163 | |
| Helb | 0.24165 | -1.0051 | 8164 | |
| Papolg | 0.2417 | -1.1538 | 8165 | |
| mmu-mir-6393 | 0.24171 | -0.36496 | 8166 | |
| Gm19345 | 0.24175 | -1.1448 | 8167 | |
| Padi4 | 0.24181 | -0.87975 | 8168 | |
| Olfr574 | 0.24193 | -1.1105 | 8169 | |
| Ganab | 0.24203 | 0.37172 | 8170 | |
| Folr2 | 0.24209 | -0.70557 | 8171 | |
| Stx16 | 0.24218 | -0.71489 | 8172 | |
| Sult2a6 | 0.24222 | -0.52474 | 8173 | |
| Gm806 | 0.24226 | -0.56258 | 8174 | |
| Vps52 | 0.2423 | -0.28317 | 8175 | |
| mmu-mir-6973a | 0.24234 | -0.68978 | 8176 | |
| Tmem86b | 0.24252 | -0.87887 | 8177 | |
| Ccr7 | 0.24252 | -0.89351 | 8178 | |
| Esp15 | 0.2426 | -1.1504 | 8179 | |
| Casp9 | 0.24282 | -0.62584 | 8180 | |
| Mak | 0.24284 | -0.7586 | 8181 | |
| Vmn1r158 | 0.24287 | -0.85924 | 8182 | |
| Pcdhac2 | 0.24288 | -0.63255 | 8183 | |
| Cd163l1 | 0.24296 | -0.57351 | 8184 | |
| Slc10a6 | 0.24307 | -0.69301 | 8185 | |
| Clec14a | 0.24307 | 0.90906 | 8186 | |
| Gm15284 | 0.24317 | -1.5105 | 8187 | |
| Irx5 | 0.24322 | -0.033695 | 8188 | |
| Samd1 | 0.24328 | -1.1155 | 8189 | |
| Zfp207 | 0.2433 | -1.1372 | 8190 | |
| Prdx2 | 0.24332 | -0.77479 | 8191 | |
| Tmem106b | 0.24332 | -0.030825 | 8192 | |
| Tinag | 0.24338 | -1.1454 | 8193 | |
| Enkd1 | 0.24339 | -0.052809 | 8194 | |
| Psmd12 | 0.24347 | -0.38017 | 8195 | |
| Zfp869 | 0.24355 | -0.49463 | 8196 | |
| Dram2 | 0.24358 | -0.69562 | 8197 | |
| E330034G19Rik | 0.24363 | -1.1166 | 8198 | |
| Scaf4 | 0.24366 | -0.43401 | 8199 | |
| Bst1 | 0.2437 | -0.36388 | 8200 | |
| Ttc18 | 0.24373 | -0.56465 | 8201 | |
| Tm4sf20 | 0.24377 | -0.6437 | 8202 | |
| Zdhhc6 | 0.24381 | -1.1454 | 8203 | |
| Ttc39a | 0.24391 | -0.17895 | 8204 | |
| AY761185 | 0.244 | 0.31648 | 8205 | |
| Pik3r3 | 0.24403 | -1.1401 | 8206 | |
| Chd1l | 0.24404 | -0.50808 | 8207 | |
| Map9 | 0.24407 | -1.1335 | 8208 | |
| Krt33a | 0.24409 | -1.0698 | 8209 | |
| Ccdc74a | 0.24411 | -0.52028 | 8210 | |
| Map1lc3b | 0.24415 | -0.065125 | 8211 | |
| Gtse1 | 0.24419 | -1.137 | 8212 | |
| Ccdc79 | 0.24421 | -1.1448 | 8213 | |
| Cep170 | 0.24426 | 1.1714 | 8214 | |
| Spns1 | 0.24428 | -1.1441 | 8215 | |
| Fcgr3 | 0.2443 | 1.2679 | 8216 | |
| Abcf2 | 0.24438 | -0.22549 | 8217 | |
| Lpin1 | 0.24441 | -1.0324 | 8218 | |
| Aars | 0.24453 | -0.56391 | 8219 | |
| Sorbs3 | 0.24463 | -1.1369 | 8220 | |
| Prok2 | 0.24468 | -0.54246 | 8221 | |
| Dzank1 | 0.24471 | -1.1402 | 8222 | |
| Pamr1 | 0.24473 | -1.1418 | 8223 | |
| BC003331 | 0.24476 | -1.143 | 8224 | |
| Il22ra1 | 0.24479 | -0.99458 | 8225 | |
| Ap2a2 | 0.24479 | -0.17925 | 8226 | |
| Adh1 | 0.24482 | -1.1412 | 8227 | |
| Casc3 | 0.24487 | -0.6362 | 8228 | |
| Tlr12 | 0.24491 | -0.33249 | 8229 | |
| Cox7a1 | 0.24493 | -0.48588 | 8230 | |
| Dynap | 0.24496 | -0.9273 | 8231 | |
| Mcrs1 | 0.24498 | -1.139 | 8232 | |
| 1700054O13Rik | 0.24506 | 0.28826 | 8233 | |
| Cyp2a12 | 0.24509 | 0.071926 | 8234 | |
| Atp7a | 0.24517 | -0.64868 | 8235 | |
| Pmp2 | 0.24524 | -0.42207 | 8236 | |
| Gm5087 | 0.24526 | -1.1382 | 8237 | |
| Prr15l | 0.24528 | -0.20784 | 8238 | |
| Rcor2 | 0.24538 | -1.1334 | 8239 | |
| Gm2382 | 0.2454 | -0.65786 | 8240 | |
| Pknox1 | 0.24543 | 0.81589 | 8241 | |
| Iqub | 0.24546 | -0.18458 | 8242 | |
| Zkscan8 | 0.24547 | -0.86516 | 8243 | |
| Cers1 | 0.24562 | -0.82025 | 8244 | |
| Gm17660 | 0.24566 | -0.85808 | 8245 | |
| Erf | 0.24568 | -0.50504 | 8246 | |
| Hspa1a | 0.24574 | -1.1348 | 8247 | |
| Cts8 | 0.24581 | -0.37141 | 8248 | |
| Nab1 | 0.24582 | -0.084144 | 8249 | |
| Olfr1307 | 0.24588 | -1.1323 | 8250 | |
| Olfr467 | 0.2461 | -1.1337 | 8251 | |
| Nupl2 | 0.24611 | -0.84535 | 8252 | |
| Hnrnpa1 | 0.24617 | -0.27709 | 8253 | |
| Phf11c | 0.24623 | -1.0367 | 8254 | |
| Slc6a17 | 0.24638 | -0.49759 | 8255 | |
| mmu-mir-6928 | 0.2464 | -0.21955 | 8256 | |
| Pnpla6 | 0.24645 | -0.5478 | 8257 | |
| Arl13b | 0.24648 | -1.1292 | 8258 | |
| Shisa4 | 0.24649 | 0.22458 | 8259 | |
| Vps37d | 0.24656 | -1.13 | 8260 | |
| Npff | 0.24656 | -0.061696 | 8261 | |
| Olfr495 | 0.2466 | -1.0893 | 8262 | |
| Gm4981 | 0.24668 | -0.22629 | 8263 | |
| Serpinb9f | 0.24668 | 0.19417 | 8264 | |
| Chmp4c | 0.2467 | -1.0919 | 8265 | |
| Slc17a9 | 0.24672 | -1.1291 | 8266 | |
| Aire | 0.24678 | -1.1351 | 8267 | |
| Kctd1 | 0.24678 | -1.1351 | 8268 | |
| Cgnl1 | 0.24683 | -0.32736 | 8269 | |
| Ttn | 0.247 | -0.99562 | 8270 | |
| Olfr94 | 0.24702 | -1.1301 | 8271 | |
| Npepps | 0.24704 | -0.65514 | 8272 | |
| Tex264 | 0.24704 | 0.49412 | 8273 | |
| Tmem19 | 0.24721 | -0.32641 | 8274 | |
| Zfp738 | 0.24721 | -1.1348 | 8275 | |
| Siglec15 | 0.24736 | -1.1329 | 8276 | |
| Cfhr1 | 0.24736 | 0.15454 | 8277 | |
| 1700026L06Rik | 0.24739 | 0.74365 | 8278 | |
| Defb22 | 0.24742 | -1.1201 | 8279 | |
| mmu-mir-6348 | 0.24742 | -0.97529 | 8280 | |
| Olfr372 | 0.24754 | -0.64292 | 8281 | |
| Stk30 | 0.24754 | 0.61586 | 8282 | |
| Muc13 | 0.24758 | 0.75114 | 8283 | |
| Smpd4 | 0.24762 | 1.0652 | 8284 | |
| Atp5o | 0.24766 | -0.22696 | 8285 | |
| Krt81 | 0.24769 | -1.002 | 8286 | |
| Olfr720 | 0.24771 | 0.41717 | 8287 | |
| Ap4m1 | 0.24773 | -0.25952 | 8288 | |
| Ppp2r2d | 0.24777 | -0.28014 | 8289 | |
| Sigirr | 0.24781 | 0.19165 | 8290 | |
| Mamdc4 | 0.24783 | -1.1266 | 8291 | |
| Olfr187 | 0.24787 | -1.078 | 8292 | |
| Zrsr2 | 0.24788 | -0.26856 | 8293 | |
| Olfr55 | 0.248 | -0.86206 | 8294 | |
| Vmn1r62 | 0.248 | -1.4818 | 8295 | |
| Eri1 | 0.24805 | -1.0601 | 8296 | |
| Apol9b | 0.24809 | 0.25915 | 8297 | |
| Olfr1193 | 0.24811 | -0.021314 | 8298 | |
| Wnt7b | 0.24825 | -1.1274 | 8299 | |
| Diexf | 0.24826 | 0.46184 | 8300 | |
| Fam120c | 0.24845 | -0.20974 | 8301 | |
| mmu-mir-684-2 | 0.24848 | -0.61211 | 8302 | |
| Ggps1 | 0.24849 | -0.2436 | 8303 | |
| Bbs10 | 0.24849 | -1.1263 | 8304 | |
| Rfc1 | 0.24853 | -0.64824 | 8305 | |
| Gpr22 | 0.24856 | 0.43246 | 8306 | |
| Immp1l | 0.24861 | -1.0648 | 8307 | |
| Csta | 0.24863 | -1.1135 | 8308 | |
| Capn6 | 0.24867 | 0.062529 | 8309 | |
| C1d | 0.24869 | -0.22263 | 8310 | |
| Vmn1r203 | 0.24875 | -1.1146 | 8311 | |
| Fmn1 | 0.24877 | -0.66887 | 8312 | |
| Gm9573 | 0.24877 | -0.8274 | 8313 | |
| Nhp2 | 0.24881 | -1.1266 | 8314 | |
| C87499 | 0.24882 | 1.4135 | 8315 | |
| mmu-mir-802 | 0.24886 | -0.097616 | 8316 | |
| Vsx2 | 0.24889 | -0.065018 | 8317 | |
| Ccdc134 | 0.2489 | -0.38898 | 8318 | |
| BC051142 | 0.24897 | -0.96015 | 8319 | |
| Bcl10 | 0.24901 | -0.7177 | 8320 | |
| Ndufa13 | 0.24904 | -1.1277 | 8321 | |
| Zfp865 | 0.24905 | 0.30209 | 8322 | |
| Actrt1 | 0.24919 | -0.34682 | 8323 | |
| mmu-mir-7025 | 0.24927 | -0.72587 | 8324 | |
| Nol6 | 0.24935 | -0.73552 | 8325 | |
| Rbl2 | 0.24939 | -0.17586 | 8326 | |
| Gpr137b | 0.24943 | 0.50808 | 8327 | |
| Stx8 | 0.24946 | -0.91718 | 8328 | |
| 7530416G11Rik | 0.24961 | -1.1199 | 8329 | |
| Olfr992 | 0.24973 | 0.56841 | 8330 | |
| Pigp | 0.24973 | -1.1192 | 8331 | |
| Pih1d3 | 0.2498 | -0.36827 | 8332 | |
| Ugt3a2 | 0.24991 | -0.32732 | 8333 | |
| mmu-mir-5104 | 0.24997 | -0.93253 | 8334 | |
| Npffr2 | 0.25005 | -0.30477 | 8335 | |
| Fbln7 | 0.2501 | -1.1247 | 8336 | |
| mmu-mir-6399 | 0.25013 | -0.094143 | 8337 | |
| Gm21708 | 0.25021 | -0.50966 | 8338 | |
| Phf8 | 0.25021 | 0.26326 | 8339 | |
| Aqr | 0.25021 | -0.2218 | 8340 | |
| Zdhhc3 | 0.25031 | -1.1122 | 8341 | |
| Wdr63 | 0.25033 | -0.42877 | 8342 | |
| B930041F14Rik | 0.25035 | -1.1151 | 8343 | |
| Ankmy1 | 0.25039 | -0.78326 | 8344 | |
| Mboat7 | 0.25049 | -1.1206 | 8345 | |
| Cped1 | 0.25054 | -1.096 | 8346 | |
| C1qtnf7 | 0.25059 | -0.22609 | 8347 | |
| Zfp934 | 0.25059 | -0.099488 | 8348 | |
| Ptpla | 0.25069 | -1.1205 | 8349 | |
| Nipal3 | 0.2507 | 0.37939 | 8350 | |
| Sec24c | 0.25074 | -1.0429 | 8351 | |
| Ncald | 0.25075 | -1.1217 | 8352 | |
| Mroh5 | 0.25081 | -0.74684 | 8353 | |
| Olfr417 | 0.25082 | -0.74617 | 8354 | |
| Brcc3 | 0.25085 | 0.93779 | 8355 | |
| Rab11fip4 | 0.25088 | -1.1221 | 8356 | |
| Lag3 | 0.251 | -0.52478 | 8357 | |
| 1110004F10Rik | 0.25103 | -1.1205 | 8358 | |
| Slc16a7 | 0.25105 | -1.0695 | 8359 | |
| Krt1 | 0.25115 | -0.1496 | 8360 | |
| Pcdhb15 | 0.25117 | -0.87679 | 8361 | |
| Rps26 | 0.25119 | -0.56198 | 8362 | |
| Zfp831 | 0.25119 | -1.1139 | 8363 | |
| Ldlrap1 | 0.25121 | -0.30544 | 8364 | |
| mmu-mir-181d | 0.25122 | -0.50707 | 8365 | |
| Kifc2 | 0.25138 | -1.0898 | 8366 | |
| Dnm2 | 0.25139 | -1.1201 | 8367 | |
| Adamtsl3 | 0.25149 | -0.11055 | 8368 | |
| Scamp5 | 0.25151 | -1.1143 | 8369 | |
| Alkbh4 | 0.25157 | 0.67808 | 8370 | |
| Tm2d3 | 0.25158 | -1.1103 | 8371 | |
| Olfr743 | 0.2516 | -0.12811 | 8372 | |
| Ctf2 | 0.25162 | -1.1149 | 8373 | |
| Vmn1r78 | 0.25168 | -0.4669 | 8374 | |
| Nuak2 | 0.25172 | 0.03756 | 8375 | |
| Spag5 | 0.25179 | 0.3915 | 8376 | |
| 4930578C19Rik | 0.2518 | -1.1128 | 8377 | |
| AK010878 | 0.25184 | -1.1142 | 8378 | |
| Sirt4 | 0.25187 | 0.042948 | 8379 | |
| Magee1 | 0.25187 | -1.1114 | 8380 | |
| Ccdc50 | 0.25191 | -1.116 | 8381 | |
| Srpk1 | 0.25194 | 0.44296 | 8382 | |
| Ryr2 | 0.25204 | -1.1184 | 8383 | |
| Ehd2 | 0.25205 | -0.072109 | 8384 | |
| Vdr | 0.25208 | -1.1071 | 8385 | |
| Magea4 | 0.25209 | 1.4383 | 8386 | |
| Chst15 | 0.25212 | -1.1177 | 8387 | |
| 9230110F15Rik | 0.25217 | -0.65972 | 8388 | |
| Dppa4 | 0.25218 | -0.28902 | 8389 | |
| Ube2l6 | 0.2522 | -0.58965 | 8390 | |
| Gm15097 | 0.25221 | -1.2574 | 8391 | |
| Rnf122 | 0.25224 | 0.34388 | 8392 | |
| Hmga1 | 0.25228 | 0.05788 | 8393 | |
| 1700061G19Rik | 0.2523 | -0.55936 | 8394 | |
| D8Ertd82e | 0.25232 | -0.77093 | 8395 | |
| Echs1 | 0.25239 | -0.79568 | 8396 | |
| Ppbp | 0.25246 | -1.1167 | 8397 | |
| E230008N13Rik | 0.25247 | -0.51815 | 8398 | |
| Zhx1 | 0.25248 | -1.1102 | 8399 | |
| Eea1 | 0.2525 | -1.1099 | 8400 | |
| Epb4.2 | 0.2525 | -0.89317 | 8401 | |
| F12 | 0.25254 | -1.0488 | 8402 | |
| Pdss2 | 0.25268 | -0.99868 | 8403 | |
| Slc17a1 | 0.2527 | -0.76722 | 8404 | |
| Irgc1 | 0.25288 | -1.1086 | 8405 | |
| Nasp | 0.25292 | -0.73345 | 8406 | |
| Eif4e1b | 0.25292 | -1.1031 | 8407 | |
| mmu-mir-5113 | 0.25301 | -0.50413 | 8408 | |
| Cst11 | 0.25302 | -1.1013 | 8409 | |
| Gm7244 | 0.2531 | 0.30228 | 8410 | |
| mmu-mir-6920 | 0.25323 | -0.96394 | 8411 | |
| Tgtp1 | 0.25327 | -1.1134 | 8412 | |
| Chst8 | 0.25327 | 1.8258 | 8413 | |
| Cdk5rap1 | 0.25337 | 0.068278 | 8414 | |
| Tnni2 | 0.2534 | -0.37089 | 8415 | |
| Ttyh1 | 0.25342 | -1.1013 | 8416 | |
| Ep300 | 0.25344 | -0.56625 | 8417 | |
| Fxr2 | 0.25344 | -1.1147 | 8418 | |
| Slc35f1 | 0.2535 | -1.0425 | 8419 | |
| Ppil1 | 0.25355 | -0.92955 | 8420 | |
| Tmem109 | 0.25359 | -0.55734 | 8421 | |
| Dtwd1 | 0.25363 | -0.21958 | 8422 | |
| Cacnb3 | 0.25368 | -1.113 | 8423 | |
| Prkaa2 | 0.2537 | 0.12819 | 8424 | |
| Vmn1r1 | 0.2537 | -1.1045 | 8425 | |
| Gstm2 | 0.25375 | -0.5275 | 8426 | |
| Olfr642 | 0.25384 | -0.85548 | 8427 | |
| Bcas3 | 0.25387 | -1.1094 | 8428 | |
| 2810006K23Rik | 0.25395 | -1.1108 | 8429 | |
| Nhej1 | 0.25413 | -1.113 | 8430 | |
| Fam160b2 | 0.25425 | -0.88922 | 8431 | |
| mmu-mir-702 | 0.25425 | -0.81458 | 8432 | |
| Esp31 | 0.25426 | -0.023815 | 8433 | |
| Arhgef40 | 0.25433 | -0.24947 | 8434 | |
| Defb43 | 0.25445 | -1.1031 | 8435 | |
| Olfr432 | 0.25448 | -1.1085 | 8436 | |
| Mrps22 | 0.25448 | -1.1085 | 8437 | |
| Dsg4 | 0.25449 | -0.91386 | 8438 | |
| Olfr1248 | 0.25456 | 0.065099 | 8439 | |
| Rs1 | 0.25483 | -0.71514 | 8440 | |
| Rbm4b | 0.25485 | -0.96476 | 8441 | |
| Pramel7 | 0.25486 | -0.052117 | 8442 | |
| Bhlhe40 | 0.25489 | -1.1095 | 8443 | |
| Ccdc162 | 0.2549 | -0.43805 | 8444 | |
| Oog3 | 0.25493 | -1.11 | 8445 | |
| Tnpo1 | 0.25498 | -0.84384 | 8446 | |
| Ten1 | 0.25501 | -1.0782 | 8447 | |
| Phf13 | 0.25503 | -1.1059 | 8448 | |
| Olfr1513 | 0.25509 | -0.53817 | 8449 | |
| Rhox11 | 0.25516 | 0.057081 | 8450 | |
| mmu-mir-29a | 0.25518 | -0.39865 | 8451 | |
| Tas2r119 | 0.25519 | -1.0974 | 8452 | |
| Hid1 | 0.25531 | -1.0994 | 8453 | |
| Plk2 | 0.25535 | -0.56271 | 8454 | |
| Srp72 | 0.25547 | -1.0501 | 8455 | |
| Pgam2 | 0.25549 | -0.62482 | 8456 | |
| Slc25a33 | 0.2555 | 0.40834 | 8457 | |
| mmu-mir-6912 | 0.25552 | -0.35221 | 8458 | |
| mmu-mir-293 | 0.25556 | -0.74976 | 8459 | |
| Slc5a8 | 0.25557 | -0.94506 | 8460 | |
| Tril | 0.25569 | -0.86348 | 8461 | |
| Dguok | 0.25572 | -1.0944 | 8462 | |
| Il12rb1 | 0.25578 | -0.70945 | 8463 | |
| Cwh43 | 0.2558 | -1.1053 | 8464 | |
| Nat8l | 0.25584 | 0.39549 | 8465 | |
| Ogfrl1 | 0.25587 | -1.1046 | 8466 | |
| Arfip2 | 0.2559 | -0.69148 | 8467 | |
| Gnb1l | 0.25617 | -0.8455 | 8468 | |
| H2-T9 | 0.25618 | -0.47154 | 8469 | |
| Hsf2bp | 0.25625 | -0.06976 | 8470 | |
| mmu-mir-7077 | 0.25632 | 0.92036 | 8471 | |
| mmu-mir-6977 | 0.25639 | 0.44668 | 8472 | |
| Pip5k1b | 0.2564 | -0.097318 | 8473 | |
| Dgcr2 | 0.25644 | -1.093 | 8474 | |
| Kcnh5 | 0.2565 | -1.1033 | 8475 | |
| Vamp4 | 0.25656 | -0.9857 | 8476 | |
| Zfp619 | 0.2566 | -1.1044 | 8477 | |
| Upk2 | 0.25666 | 0.76161 | 8478 | |
| Gabarapl1 | 0.25666 | -0.1356 | 8479 | |
| Olfr847 | 0.25668 | -0.36425 | 8480 | |
| Apobec2 | 0.25672 | -1.1044 | 8481 | |
| Cfhr2 | 0.25673 | 1.0039 | 8482 | |
| Dusp23 | 0.25681 | 1.4799 | 8483 | |
| Ccdc159 | 0.25691 | -1.1019 | 8484 | |
| mmu-mir-21a | 0.25691 | -0.86764 | 8485 | |
| 2310057M21Rik | 0.25693 | -1.1013 | 8486 | |
| Myoc | 0.25705 | -0.41971 | 8487 | |
| Chil6 | 0.25705 | -0.93673 | 8488 | |
| Nfe2l2 | 0.25709 | -0.98223 | 8489 | |
| Casc5 | 0.25712 | -1.0986 | 8490 | |
| Tatdn1 | 0.25722 | -0.30083 | 8491 | |
| Igf2 | 0.25725 | -1.0939 | 8492 | |
| Armc8 | 0.25739 | -0.93664 | 8493 | |
| Spib | 0.2574 | -0.22615 | 8494 | |
| Mael | 0.25744 | -0.46602 | 8495 | |
| mmu-mir-7011 | 0.25748 | -0.30416 | 8496 | |
| Foxa3 | 0.25752 | 0.0005872 | 8497 | |
| Tmem91 | 0.25752 | -1.0983 | 8498 | |
| Hoxd13 | 0.25761 | -1.0951 | 8499 | |
| Olfr859 | 0.25769 | -1.0994 | 8500 | |
| Olfr1250 | 0.25778 | 0.10617 | 8501 | |
| Nfya | 0.25781 | -0.83094 | 8502 | |
| Cep72 | 0.25781 | -0.45273 | 8503 | |
| Kpna7 | 0.25785 | -0.63531 | 8504 | |
| Fbxo39 | 0.25787 | -1.1012 | 8505 | |
| Ercc8 | 0.25789 | 0.24 | 8506 | |
| Cldn1 | 0.25793 | 1.2602 | 8507 | |
| Thrb | 0.258 | -1.0747 | 8508 | |
| Kpna4 | 0.258 | -0.25281 | 8509 | |
| Fstl4 | 0.25804 | 0.16136 | 8510 | |
| Atf5 | 0.25826 | 0.00124 | 8511 | |
| Efhd1 | 0.2583 | -1.0913 | 8512 | |
| Fxn | 0.2583 | -0.87062 | 8513 | |
| mmu-mir-5046 | 0.25834 | -0.75411 | 8514 | |
| Epn2 | 0.25834 | -0.81953 | 8515 | |
| Scx | 0.25834 | -1.0973 | 8516 | |
| Bai2 | 0.25836 | -1.0959 | 8517 | |
| Gm8817 | 0.25839 | 0.71079 | 8518 | |
| Ifnar2 | 0.25839 | 1.2769 | 8519 | |
| Cd63 | 0.25845 | 0.83921 | 8520 | |
| Camk2b | 0.25852 | -1.0944 | 8521 | |
| Dpp3 | 0.25856 | -1.0881 | 8522 | |
| Pde4d | 0.25867 | -1.0941 | 8523 | |
| Cdkn2b | 0.25871 | -0.77923 | 8524 | |
| Kif5a | 0.25871 | -1.0976 | 8525 | |
| Apoa2 | 0.25876 | -0.86267 | 8526 | |
| Ccdc144b | 0.25878 | 0.037605 | 8527 | |
| Gm6370 | 0.25892 | -1.4355 | 8528 | |
| Wbscr17 | 0.25895 | -1.084 | 8529 | |
| Tas2r109 | 0.25895 | -0.41991 | 8530 | |
| Lipe | 0.25901 | 0.048736 | 8531 | |
| Ppp1r14b | 0.25905 | -0.45443 | 8532 | |
| Gabrd | 0.25905 | -1.0906 | 8533 | |
| Glrx2 | 0.25908 | 0.8843 | 8534 | |
| Ube2d2b | 0.25912 | -0.8643 | 8535 | |
| Snrpn | 0.25915 | -0.60137 | 8536 | |
| Gp5 | 0.25916 | 0.68478 | 8537 | |
| Uba6 | 0.25919 | -1.095 | 8538 | |
| Rbm22 | 0.25919 | -0.96538 | 8539 | |
| Fbxw22 | 0.25923 | -0.60479 | 8540 | |
| Cyb561a3 | 0.25923 | 0.12988 | 8541 | |
| Snhg11 | 0.25927 | -0.66247 | 8542 | |
| mmu-mir-8118 | 0.25943 | -0.67578 | 8543 | |
| Dgki | 0.25945 | -1.0782 | 8544 | |
| Ifna7 | 0.25953 | 0.25007 | 8545 | |
| Cav2 | 0.25957 | -1.0945 | 8546 | |
| Olfr73 | 0.2596 | -1.0715 | 8547 | |
| Tgm7 | 0.25969 | -1.091 | 8548 | |
| S100a16 | 0.25983 | -0.27642 | 8549 | |
| Plgrkt | 0.25986 | -0.053458 | 8550 | |
| Mettl7a3 | 0.25992 | 1.0771 | 8551 | |
| Capn9 | 0.25994 | 1.1794 | 8552 | |
| Tlx1 | 0.25998 | 1.2143 | 8553 | |
| Nemf | 0.26002 | -0.62106 | 8554 | |
| Kmt2b | 0.26009 | -0.57124 | 8555 | |
| T | 0.26016 | 0.61283 | 8556 | |
| Gmcl1l | 0.26017 | -0.59105 | 8557 | |
| AI597479 | 0.2602 | 0.69498 | 8558 | |
| mmu-mir-3099 | 0.26023 | -0.18205 | 8559 | |
| Magea5 | 0.26027 | 2.0485 | 8560 | |
| Dgkz | 0.26028 | -1.0838 | 8561 | |
| Coil | 0.26042 | -0.77775 | 8562 | |
| Rtn4 | 0.26042 | -0.4203 | 8563 | |
| Cyb5d1 | 0.26045 | -1.0897 | 8564 | |
| Chtop | 0.26052 | -0.56657 | 8565 | |
| Irx6 | 0.26053 | 0.57135 | 8566 | |
| mmu-mir-7040 | 0.26062 | -0.70919 | 8567 | |
| Grm6 | 0.2607 | -1.0908 | 8568 | |
| mmu-mir-497b | 0.26073 | -0.76268 | 8569 | |
| Ppp2r2a | 0.2608 | -0.43263 | 8570 | |
| Olfr736 | 0.26081 | -0.86633 | 8571 | |
| Palb2 | 0.26083 | -0.022697 | 8572 | |
| Dgkb | 0.26083 | 0.12532 | 8573 | |
| Arid5b | 0.26091 | -0.13689 | 8574 | |
| Fgf6 | 0.26096 | -0.49322 | 8575 | |
| Inhbc | 0.26101 | -1.0856 | 8576 | |
| Gm3604 | 0.26111 | -0.16503 | 8577 | |
| Mcoln3 | 0.26115 | -1.0825 | 8578 | |
| Nkx2-6 | 0.2612 | -0.47651 | 8579 | |
| Olfr1419 | 0.26124 | 0.43983 | 8580 | |
| Uhrf2 | 0.26135 | -1.0833 | 8581 | |
| Zfp30 | 0.26138 | -0.59299 | 8582 | |
| Usb1 | 0.2614 | -0.71459 | 8583 | |
| Pfkfb2 | 0.26147 | -0.09008 | 8584 | |
| Srp68 | 0.2615 | -1.0572 | 8585 | |
| Osgepl1 | 0.26167 | -0.091021 | 8586 | |
| Ehf | 0.26176 | 1.008 | 8587 | |
| Il1rapl1 | 0.2618 | -0.9676 | 8588 | |
| Exosc3 | 0.26184 | -1.088 | 8589 | |
| Fam19a2 | 0.26186 | -0.65975 | 8590 | |
| Csnk1g2 | 0.26188 | -1.0662 | 8591 | |
| Zfp870 | 0.26195 | 0.84812 | 8592 | |
| Catsperg2 | 0.26199 | 0.18898 | 8593 | |
| Serpinb3a | 0.2621 | -0.93217 | 8594 | |
| Rtel1 | 0.26213 | -1.0868 | 8595 | |
| mmu-mir-7117 | 0.26222 | -0.59562 | 8596 | |
| Kif21a | 0.26233 | -1.0858 | 8597 | |
| Gm5771 | 0.26236 | 0.42832 | 8598 | |
| Kif1a | 0.26239 | 0.86156 | 8599 | |
| Tbc1d13 | 0.26241 | -1.0864 | 8600 | |
| Rims4 | 0.26247 | -0.37705 | 8601 | |
| Nrap | 0.26265 | 0.2984 | 8602 | |
| Btbd6 | 0.26265 | -0.58525 | 8603 | |
| Agap2 | 0.26274 | -1.0701 | 8604 | |
| Tmem209 | 0.26282 | -0.71469 | 8605 | |
| Gldn | 0.26284 | -0.35571 | 8606 | |
| Cd200r3 | 0.26288 | -1.0802 | 8607 | |
| Trpa1 | 0.26288 | -1.0802 | 8608 | |
| Esrp1 | 0.26288 | -1.0802 | 8609 | |
| Nipsnap3b | 0.26291 | -0.011762 | 8610 | |
| Apoa1 | 0.26292 | -0.52773 | 8611 | |
| Ankrd7 | 0.26296 | -0.24489 | 8612 | |
| mmu-mir-291b | 0.26298 | -0.63649 | 8613 | |
| Mup1 | 0.26298 | -1.0847 | 8614 | |
| Scamp1 | 0.26306 | -0.61685 | 8615 | |
| Olfr958 | 0.26308 | -0.65512 | 8616 | |
| Itga11 | 0.2631 | -1.0149 | 8617 | |
| Olfr224 | 0.26316 | -1.0822 | 8618 | |
| Rnasel | 0.26325 | -0.12408 | 8619 | |
| Sipa1l2 | 0.26343 | -0.22245 | 8620 | |
| Ctnna1 | 0.26347 | -0.37347 | 8621 | |
| Prl3d2 | 0.26362 | -1.0747 | 8622 | |
| Brdt | 0.26369 | -0.57799 | 8623 | |
| Rnf167 | 0.26377 | -0.71083 | 8624 | |
| Ufc1 | 0.26395 | 0.073804 | 8625 | |
| Cdcp1 | 0.26395 | -0.34877 | 8626 | |
| Fam135b | 0.26399 | -0.42202 | 8627 | |
| Nlrp6 | 0.264 | -0.98655 | 8628 | |
| mmu-mir-23a | 0.264 | -0.49386 | 8629 | |
| Olfr376 | 0.26414 | 0.68657 | 8630 | |
| Sarm1 | 0.26418 | 1.436 | 8631 | |
| Abhd17a | 0.26418 | -1.0663 | 8632 | |
| Ranbp3l | 0.26421 | -0.20773 | 8633 | |
| Wdr60 | 0.26428 | -1.069 | 8634 | |
| Scn1a | 0.26436 | -1.0604 | 8635 | |
| Olfr287 | 0.26442 | 0.40255 | 8636 | |
| Ankrd13a | 0.26451 | 0.012793 | 8637 | |
| Pdzk1ip1 | 0.26454 | -0.15799 | 8638 | |
| Omt2b | 0.26458 | -1.0266 | 8639 | |
| Srgap3 | 0.26459 | -0.63765 | 8640 | |
| Olfr694 | 0.26463 | -1.0778 | 8641 | |
| Olfr1489 | 0.26471 | 2.5269 | 8642 | |
| Ckap2l | 0.26483 | -0.9412 | 8643 | |
| Cdon | 0.26484 | -0.32466 | 8644 | |
| Bglap2 | 0.26486 | -1.0722 | 8645 | |
| Lrba | 0.26492 | 0.10346 | 8646 | |
| Actn2 | 0.26493 | -0.84422 | 8647 | |
| Ror2 | 0.26495 | -1.065 | 8648 | |
| Zdhhc4 | 0.26499 | -0.010711 | 8649 | |
| Fbln1 | 0.26503 | -0.072854 | 8650 | |
| mmu-mir-6409 | 0.26508 | -0.95297 | 8651 | |
| Olfr893 | 0.26508 | -1.0769 | 8652 | |
| Dnah1 | 0.26518 | 0.48977 | 8653 | |
| Srm | 0.2652 | -1.0721 | 8654 | |
| Itsn2 | 0.26529 | -0.89125 | 8655 | |
| Vmn2r108 | 0.2653 | -1.0718 | 8656 | |
| Dpy19l4 | 0.26536 | 1.458 | 8657 | |
| A230046K03Rik | 0.26544 | -1.073 | 8658 | |
| Gyltl1b | 0.26547 | -0.26893 | 8659 | |
| Arl14 | 0.2655 | -1.0726 | 8660 | |
| Ampd2 | 0.26551 | -0.23452 | 8661 | |
| Pvalb | 0.26554 | -1.0626 | 8662 | |
| Tcstv1 | 0.26555 | 0.95992 | 8663 | |
| Gbp3 | 0.26559 | -0.78107 | 8664 | |
| Tsta3 | 0.26563 | -1.0737 | 8665 | |
| Hist1h2ah | 0.26566 | -0.12413 | 8666 | |
| Rhox3a | 0.26579 | -1.0623 | 8667 | |
| Kir3dl2 | 0.26581 | -1.0749 | 8668 | |
| Nipal1 | 0.26585 | -1.073 | 8669 | |
| Hoxb4 | 0.26587 | -1.064 | 8670 | |
| 4930519G04Rik | 0.26596 | -0.46476 | 8671 | |
| Abcb5 | 0.26597 | -0.74929 | 8672 | |
| Rassf6 | 0.26603 | -0.79157 | 8673 | |
| mmu-mir-7654 | 0.26611 | -0.75473 | 8674 | |
| Olfr597 | 0.26613 | -1.07 | 8675 | |
| Slc6a15 | 0.26614 | -0.8649 | 8676 | |
| Serpinb9b | 0.26619 | -1.0659 | 8677 | |
| Olfr1466 | 0.26621 | -1.0735 | 8678 | |
| Gcnt4 | 0.26624 | -0.93655 | 8679 | |
| Prop1 | 0.26626 | -0.52715 | 8680 | |
| mmu-mir-199a-1 | 0.26636 | -0.051106 | 8681 | |
| Trim58 | 0.26637 | -0.81148 | 8682 | |
| Ablim2 | 0.26646 | -1.0679 | 8683 | |
| Aplf | 0.26657 | -1.0686 | 8684 | |
| Krt23 | 0.2666 | -1.0685 | 8685 | |
| Ripk4 | 0.26672 | -1.0714 | 8686 | |
| Alg10b | 0.26674 | -1.0565 | 8687 | |
| Smn1 | 0.26681 | -0.87353 | 8688 | |
| Cadm2 | 0.26683 | -1.0678 | 8689 | |
| Car13 | 0.26686 | 0.020343 | 8690 | |
| Tbcel | 0.26698 | -1.0571 | 8691 | |
| Jmjd6 | 0.26699 | -0.19516 | 8692 | |
| 2210016L21Rik | 0.26706 | -1.0657 | 8693 | |
| Terf2ip | 0.2671 | -0.4146 | 8694 | |
| mmu-mir-6343 | 0.26711 | -0.81168 | 8695 | |
| Them5 | 0.26721 | -0.74479 | 8696 | |
| Dock6 | 0.26728 | -1.0668 | 8697 | |
| Hmgcl | 0.26733 | 1.301 | 8698 | |
| Lrdd | 0.26736 | -1.0645 | 8699 | |
| Ccdc152 | 0.26736 | -0.097146 | 8700 | |
| Tnfrsf11a | 0.26738 | -1.0667 | 8701 | |
| Slc14a1 | 0.26744 | 0.092005 | 8702 | |
| Spata2 | 0.26746 | -0.58795 | 8703 | |
| Gm765 | 0.26747 | -0.2899 | 8704 | |
| Olfr1437 | 0.26754 | -0.64618 | 8705 | |
| mmu-mir-673 | 0.26771 | -0.83561 | 8706 | |
| Prrxl1 | 0.26772 | -1.0593 | 8707 | |
| Hyal6 | 0.26785 | -1.0664 | 8708 | |
| Kdm4d | 0.26789 | -1.0212 | 8709 | |
| G3bp2 | 0.26799 | -0.60783 | 8710 | |
| Pex6 | 0.26801 | -1.0641 | 8711 | |
| Dnah17 | 0.26803 | -1.0528 | 8712 | |
| Ints6 | 0.2683 | -1.0217 | 8713 | |
| Vsig8 | 0.26837 | -1.0624 | 8714 | |
| Nfat5 | 0.26843 | -0.64625 | 8715 | |
| Hoxa1 | 0.26851 | 0.0071809 | 8716 | |
| Mxd1 | 0.26852 | -0.6741 | 8717 | |
| 1700102P08Rik | 0.26854 | -0.40232 | 8718 | |
| D330045A20Rik | 0.26858 | 0.27156 | 8719 | |
| mmu-mir-7671 | 0.26859 | 0.37583 | 8720 | |
| Maff | 0.26865 | -1.0599 | 8721 | |
| Tph2 | 0.26884 | 1.7819 | 8722 | |
| Mical3 | 0.2691 | 0.95488 | 8723 | |
| mmu-mir-6398 | 0.2691 | -0.69038 | 8724 | |
| Mme | 0.26915 | -1.0598 | 8725 | |
| Atf4 | 0.26922 | -1.0619 | 8726 | |
| Neurog1 | 0.26925 | 0.271 | 8727 | |
| Ndnl2 | 0.26947 | -1.0043 | 8728 | |
| Tcf7l2 | 0.26948 | -0.70344 | 8729 | |
| Agpat5 | 0.26951 | -0.14267 | 8730 | |
| G6pc3 | 0.26952 | -0.11921 | 8731 | |
| Itk | 0.26954 | -0.26547 | 8732 | |
| Olfr1152 | 0.26956 | -1.0573 | 8733 | |
| Rarres2 | 0.26958 | -0.57426 | 8734 | |
| Zwilch | 0.26969 | -0.13358 | 8735 | |
| Csdc2 | 0.2697 | -1.0596 | 8736 | |
| Slc39a10 | 0.26975 | -1.0631 | 8737 | |
| Olfr308 | 0.26983 | -0.94832 | 8738 | |
| Lsg1 | 0.26993 | -0.54632 | 8739 | |
| Med25 | 0.26993 | -1.0438 | 8740 | |
| Prkar1a | 0.26997 | -1.0555 | 8741 | |
| Olfr462 | 0.26999 | -1.0581 | 8742 | |
| Gdpgp1 | 0.27002 | -0.14102 | 8743 | |
| Lgals12 | 0.2701 | -1.0561 | 8744 | |
| Gm14378 | 0.2701 | -0.58812 | 8745 | |
| Defb1 | 0.27024 | -0.42272 | 8746 | |
| Myf6 | 0.27026 | -1.059 | 8747 | |
| Olfr930 | 0.27043 | -0.24458 | 8748 | |
| Cd276 | 0.27047 | 0.95828 | 8749 | |
| Lman1l | 0.27054 | 0.038375 | 8750 | |
| N4bp2 | 0.27059 | -1.0061 | 8751 | |
| Olfr77 | 0.27067 | 0.26274 | 8752 | |
| Zfp87 | 0.27067 | -0.14798 | 8753 | |
| Dlx6 | 0.27079 | -0.43417 | 8754 | |
| Mcf2 | 0.2708 | -0.12325 | 8755 | |
| Olfr441 | 0.27081 | -1.0608 | 8756 | |
| Abcc4 | 0.27083 | 0.037495 | 8757 | |
| Adamts20 | 0.27087 | 0.24724 | 8758 | |
| Parg | 0.27093 | 0.059624 | 8759 | |
| Olfr10 | 0.27096 | -0.69773 | 8760 | |
| Tcea2 | 0.27113 | -0.67455 | 8761 | |
| Mphosph6 | 0.27116 | -1.0416 | 8762 | |
| Vdac2 | 0.27119 | -1.0564 | 8763 | |
| Ubald2 | 0.27124 | 0.10014 | 8764 | |
| Aspa | 0.27126 | -1.0567 | 8765 | |
| Gmcl1 | 0.27128 | 0.19739 | 8766 | |
| Galr3 | 0.27128 | -0.97486 | 8767 | |
| Olfr671 | 0.2713 | -0.32814 | 8768 | |
| Olfr982 | 0.27139 | -1.0508 | 8769 | |
| Rgs13 | 0.27139 | -0.89304 | 8770 | |
| Pax2 | 0.27149 | -1.0551 | 8771 | |
| Gdf5 | 0.27151 | -0.7371 | 8772 | |
| Olfr60 | 0.27153 | -0.096445 | 8773 | |
| Pfkfb3 | 0.27157 | -1.0465 | 8774 | |
| Srpk2 | 0.27161 | -1.0464 | 8775 | |
| Zfp318 | 0.27165 | -1.0584 | 8776 | |
| Uqcrq | 0.27168 | -0.16643 | 8777 | |
| Adi1 | 0.27171 | -1.0444 | 8778 | |
| Rdh5 | 0.27172 | 0.088449 | 8779 | |
| Olfr351 | 0.27179 | 0.28982 | 8780 | |
| Macf1 | 0.27187 | 0.37458 | 8781 | |
| Top1mt | 0.27196 | 1.4138 | 8782 | |
| Gpr64 | 0.27201 | 1.0152 | 8783 | |
| Col4a3 | 0.27212 | 0.23355 | 8784 | |
| Rfk | 0.27212 | -0.89175 | 8785 | |
| mmu-mir-6350 | 0.27219 | -0.39208 | 8786 | |
| Inpp5b | 0.27231 | -1.0388 | 8787 | |
| Stk3 | 0.27237 | -1.0564 | 8788 | |
| Figf | 0.27238 | -0.15415 | 8789 | |
| Gm15816 | 0.27241 | -1.0529 | 8790 | |
| 1810024B03Rik | 0.27251 | -0.52773 | 8791 | |
| Klhl33 | 0.27253 | 0.99259 | 8792 | |
| Gtf3c5 | 0.27256 | -1.0556 | 8793 | |
| Olfr1052 | 0.27258 | -1.0445 | 8794 | |
| mmu-mir-466f-2 | 0.27265 | -0.96695 | 8795 | |
| Psip1 | 0.27266 | -1.0546 | 8796 | |
| Vps41 | 0.27268 | -1.0489 | 8797 | |
| Olfr322 | 0.27281 | 0.65321 | 8798 | |
| Rp1 | 0.27283 | -1.0531 | 8799 | |
| Cxxc5 | 0.27283 | -1.0531 | 8800 | |
| Klhl24 | 0.27286 | -0.87453 | 8801 | |
| Cnot2 | 0.27286 | -0.72999 | 8802 | |
| Olfr430 | 0.27288 | -1.0532 | 8803 | |
| Arid1b | 0.2729 | -0.33157 | 8804 | |
| Men1 | 0.27297 | -0.43698 | 8805 | |
| AI987944 | 0.27304 | 0.74312 | 8806 | |
| Teddm1 | 0.27319 | -0.999 | 8807 | |
| Lrig1 | 0.27323 | 0.72164 | 8808 | |
| Gm3286 | 0.27323 | -1.3606 | 8809 | |
| Spry4 | 0.27325 | -0.87579 | 8810 | |
| Enah | 0.2734 | -1.0453 | 8811 | |
| Vmn2r14 | 0.27343 | -1.0509 | 8812 | |
| Mctp2 | 0.27348 | -1.0402 | 8813 | |
| Kcnrg | 0.27351 | -1.0516 | 8814 | |
| mmu-mir-8100 | 0.27352 | 0.35652 | 8815 | |
| Nod1 | 0.27355 | -1.051 | 8816 | |
| Gjd4 | 0.2736 | 0.93827 | 8817 | |
| Sdf2l1 | 0.2736 | -0.67383 | 8818 | |
| mmu-mir-503 | 0.27363 | -0.63047 | 8819 | |
| Fam43a | 0.27367 | 0.42994 | 8820 | |
| Sgms1 | 0.27373 | -1.0332 | 8821 | |
| Vmn1r36 | 0.27378 | -0.84656 | 8822 | |
| mmu-mir-217 | 0.27384 | -0.57224 | 8823 | |
| Cxcl3 | 0.27389 | -1.0482 | 8824 | |
| Mapk8ip2 | 0.27401 | -0.071382 | 8825 | |
| Ccdc137 | 0.27403 | -1.0377 | 8826 | |
| Smad5 | 0.27415 | 0.21921 | 8827 | |
| Asb7 | 0.27417 | -1.0489 | 8828 | |
| Olfr1135 | 0.27422 | -0.27049 | 8829 | |
| Unkl | 0.27424 | -1.0446 | 8830 | |
| C4a | 0.27426 | -0.54708 | 8831 | |
| Fxyd2 | 0.27428 | -0.79115 | 8832 | |
| Skint2 | 0.27438 | -1.0468 | 8833 | |
| Bcar1 | 0.27447 | -1.0318 | 8834 | |
| Wsb2 | 0.27453 | -0.33672 | 8835 | |
| Cdh7 | 0.27455 | 1.3754 | 8836 | |
| Tescl | 0.27456 | -1.0415 | 8837 | |
| Mum1 | 0.27459 | -1.0328 | 8838 | |
| Slc7a5 | 0.27463 | -0.90092 | 8839 | |
| Ndrg1 | 0.27466 | 0.45088 | 8840 | |
| Cdip1 | 0.2747 | 0.35596 | 8841 | |
| Rint1 | 0.27475 | -1.0433 | 8842 | |
| Tox2 | 0.27485 | 0.034908 | 8843 | |
| Taf6l | 0.27488 | -0.67557 | 8844 | |
| Dnajc22 | 0.2749 | -1.0481 | 8845 | |
| Oxct1 | 0.27492 | -1.0064 | 8846 | |
| Tmem192 | 0.27492 | -0.41939 | 8847 | |
| Kcnn4 | 0.27496 | -0.91575 | 8848 | |
| Rnf148 | 0.27496 | -0.097269 | 8849 | |
| Olfr1024 | 0.275 | -1.0477 | 8850 | |
| E230025N22Rik | 0.27502 | -1.04 | 8851 | |
| Abca15 | 0.27504 | -0.89928 | 8852 | |
| Ptrh1 | 0.27506 | -1.0397 | 8853 | |
| Krt83 | 0.2751 | -1.033 | 8854 | |
| Slc26a11 | 0.27518 | -0.13504 | 8855 | |
| Rchy1 | 0.27518 | -1.0439 | 8856 | |
| Urgcp | 0.27522 | -1.0475 | 8857 | |
| Clec2d | 0.27525 | -1.041 | 8858 | |
| Snx10 | 0.27525 | -0.14108 | 8859 | |
| Gng3 | 0.27531 | -0.075335 | 8860 | |
| Agpat9 | 0.27554 | -0.93255 | 8861 | |
| Ppp2r5c | 0.27558 | -0.80043 | 8862 | |
| Olfr690 | 0.27562 | 0.055288 | 8863 | |
| Aqp5 | 0.27569 | 1.2013 | 8864 | |
| Mettl14 | 0.27576 | -0.45631 | 8865 | |
| Ebf1 | 0.2758 | -0.54165 | 8866 | |
| mmu-mir-195a | 0.2759 | -0.81082 | 8867 | |
| Olfr894 | 0.27593 | -0.075277 | 8868 | |
| Olfr133 | 0.27595 | -0.80886 | 8869 | |
| mmu-mir-496a | 0.27598 | -0.53537 | 8870 | |
| Foxp3 | 0.27607 | -0.045022 | 8871 | |
| Klhl34 | 0.27617 | -0.9012 | 8872 | |
| Klk7 | 0.27619 | -1.0376 | 8873 | |
| Olfr684 | 0.2762 | -0.62109 | 8874 | |
| Nceh1 | 0.27621 | -1.0362 | 8875 | |
| Kmt2d | 0.27624 | -0.99376 | 8876 | |
| mmu-mir-8098 | 0.27624 | -0.69175 | 8877 | |
| Bcmo1 | 0.27636 | -0.32679 | 8878 | |
| Desi2 | 0.27642 | -1.0404 | 8879 | |
| mmu-mir-7049 | 0.27649 | -0.86788 | 8880 | |
| Tmem206 | 0.27661 | -1.0296 | 8881 | |
| 4930402H24Rik | 0.27661 | -0.73485 | 8882 | |
| Zfp174 | 0.27663 | -0.30666 | 8883 | |
| Ogdh | 0.27665 | -1.0414 | 8884 | |
| B3galt6 | 0.27668 | -0.085908 | 8885 | |
| Tab1 | 0.27672 | -0.76981 | 8886 | |
| Prame | 0.27677 | -1.0405 | 8887 | |
| Smad7 | 0.27679 | -1.0316 | 8888 | |
| Tex36 | 0.27686 | 0.67627 | 8889 | |
| Klhl12 | 0.27694 | -1.042 | 8890 | |
| BC068281 | 0.27699 | 0.57202 | 8891 | |
| Akap10 | 0.277 | -1.0373 | 8892 | |
| Olfr695 | 0.27705 | -0.14621 | 8893 | |
| Dcaf8 | 0.27712 | 0.50214 | 8894 | |
| Phf20 | 0.27712 | -0.23419 | 8895 | |
| Crbn | 0.27719 | -0.67412 | 8896 | |
| mmu-mir-6359 | 0.27723 | -0.82237 | 8897 | |
| Hdgf | 0.27725 | -1.0409 | 8898 | |
| Ribc1 | 0.2773 | 0.59329 | 8899 | |
| Ak7 | 0.27738 | -0.44871 | 8900 | |
| Olfr1263 | 0.27743 | 2.4301 | 8901 | |
| Msh4 | 0.27747 | -0.1029 | 8902 | |
| Olfr1283 | 0.27751 | -0.99771 | 8903 | |
| 1700029P11Rik | 0.27752 | -0.72687 | 8904 | |
| Nhlrc4 | 0.27753 | -1.031 | 8905 | |
| Tln2 | 0.27756 | -0.12349 | 8906 | |
| Trim50 | 0.27758 | -1.0323 | 8907 | |
| Hsp90b1 | 0.2776 | -0.42704 | 8908 | |
| Serpina3k | 0.27763 | -1.0399 | 8909 | |
| Zhx2 | 0.27763 | -0.4092 | 8910 | |
| Ica1l | 0.27766 | -1.0314 | 8911 | |
| Pkd2l2 | 0.27767 | -0.62507 | 8912 | |
| Rapgefl1 | 0.27768 | -1.0306 | 8913 | |
| Hist1h2ab | 0.27773 | 0.73005 | 8914 | |
| Sgcb | 0.27776 | -1.0231 | 8915 | |
| Ptges3 | 0.27788 | -0.81212 | 8916 | |
| Scrg1 | 0.27789 | 0.58924 | 8917 | |
| Ern2 | 0.27793 | -0.40928 | 8918 | |
| Edar | 0.27801 | -1.0342 | 8919 | |
| Pydc4 | 0.27804 | 1.3213 | 8920 | |
| Spag7 | 0.27805 | -1.0383 | 8921 | |
| Gm6251 | 0.27813 | 0.24264 | 8922 | |
| Ppp3ca | 0.27818 | -0.09794 | 8923 | |
| LOC100504608 | 0.27822 | -1.0228 | 8924 | |
| Arhgef39 | 0.27826 | -0.40003 | 8925 | |
| Galnt9 | 0.27836 | -1.0226 | 8926 | |
| Pdcd2 | 0.27837 | 0.28522 | 8927 | |
| Bend5 | 0.27838 | -1.036 | 8928 | |
| mmu-mir-7232 | 0.27846 | -0.70723 | 8929 | |
| mmu-mir-6540 | 0.27848 | -0.32964 | 8930 | |
| Vmn2r58 | 0.27848 | -0.61892 | 8931 | |
| Pth2 | 0.27851 | -0.83272 | 8932 | |
| Ngp | 0.27854 | -1.0336 | 8933 | |
| Unc5d | 0.27855 | 0.86069 | 8934 | |
| Elovl2 | 0.27857 | -1.035 | 8935 | |
| Gdpd2 | 0.27862 | -0.01742 | 8936 | |
| Actr1a | 0.2787 | -0.36601 | 8937 | |
| Slc38a5 | 0.2787 | -1.0363 | 8938 | |
| Trim40 | 0.27873 | -0.93608 | 8939 | |
| Ube2j1 | 0.27881 | -0.90432 | 8940 | |
| Htr1a | 0.27885 | -0.77389 | 8941 | |
| Grsf1 | 0.27888 | 1.0879 | 8942 | |
| Hoxb8 | 0.27892 | 0.22604 | 8943 | |
| Sh2d2a | 0.27892 | -0.21962 | 8944 | |
| Selo | 0.27895 | 0.53803 | 8945 | |
| Yrdc | 0.27908 | -0.024508 | 8946 | |
| Trim24 | 0.2791 | -0.00052964 | 8947 | |
| Ush2a | 0.27913 | -1.0274 | 8948 | |
| Efna5 | 0.27917 | -0.46027 | 8949 | |
| Olfr644 | 0.27921 | -1.0293 | 8950 | |
| Sec22a | 0.27932 | -0.18189 | 8951 | |
| Gsdma3 | 0.27933 | -0.76332 | 8952 | |
| Fbxo7 | 0.27939 | -0.64482 | 8953 | |
| Ccdc115 | 0.27943 | -0.44152 | 8954 | |
| Rufy3 | 0.27943 | -0.36728 | 8955 | |
| Fam3b | 0.2795 | -1.0338 | 8956 | |
| Rbp4 | 0.27957 | -0.2733 | 8957 | |
| Pdlim2 | 0.27961 | -0.45755 | 8958 | |
| Gpatch11 | 0.27962 | -0.19179 | 8959 | |
| Gna12 | 0.2797 | -1.0245 | 8960 | |
| Hsd3b5 | 0.27977 | -1.0336 | 8961 | |
| Fra10ac1 | 0.27977 | -1.0336 | 8962 | |
| Lcn5 | 0.27979 | 0.2016 | 8963 | |
| Cplx4 | 0.27985 | -1.0199 | 8964 | |
| Trim67 | 0.27995 | -1.015 | 8965 | |
| Vmn1r208 | 0.27998 | 2.0241 | 8966 | |
| Prl8a2 | 0.28003 | -1.0295 | 8967 | |
| Slco6b1 | 0.28005 | -1.0235 | 8968 | |
| Cox20 | 0.28007 | -0.85515 | 8969 | |
| Sdsl | 0.28012 | -0.99214 | 8970 | |
| Vmn2r65 | 0.28016 | -1.0328 | 8971 | |
| Cyb5b | 0.28016 | -0.5296 | 8972 | |
| Bclaf1 | 0.28018 | -0.99594 | 8973 | |
| Sh3bp2 | 0.2802 | -0.70093 | 8974 | |
| Slfn8 | 0.28022 | -1.0324 | 8975 | |
| Nln | 0.28028 | -1.0294 | 8976 | |
| Slc39a8 | 0.28031 | -0.30472 | 8977 | |
| Cyp4f37 | 0.28031 | -1.0266 | 8978 | |
| Fam216b | 0.28034 | -0.71151 | 8979 | |
| Camk2d | 0.28042 | 0.15142 | 8980 | |
| Galntl5 | 0.28047 | 1.321 | 8981 | |
| Olfr406 | 0.28053 | -1.0212 | 8982 | |
| Nxt2 | 0.28057 | -1.0253 | 8983 | |
| Cngb3 | 0.28067 | -0.18447 | 8984 | |
| mmu-mir-290b | 0.28068 | -1.3319 | 8985 | |
| Setdb2 | 0.28072 | -0.57998 | 8986 | |
| Fmr1 | 0.28074 | -1.0006 | 8987 | |
| Ceacam3 | 0.28076 | -1.0308 | 8988 | |
| Ptprs | 0.28078 | 0.54413 | 8989 | |
| Slc9a2 | 0.2808 | -0.63157 | 8990 | |
| Vhl | 0.28085 | -0.76929 | 8991 | |
| Cct6a | 0.28089 | 0.35753 | 8992 | |
| Trhr2 | 0.2809 | -1.0311 | 8993 | |
| Ptpn1 | 0.28096 | -1.0303 | 8994 | |
| Als2cr12 | 0.28098 | -0.87848 | 8995 | |
| Sytl4 | 0.281 | -0.42105 | 8996 | |
| mmu-mir-532 | 0.28104 | -0.16833 | 8997 | |
| Mfsd11 | 0.28111 | -1.0245 | 8998 | |
| A830080D01Rik | 0.28111 | 1.4245 | 8999 | |
| Chst14 | 0.28115 | -1.0062 | 9000 | |
| mmu-mir-145a | 0.28123 | -0.40138 | 9001 | |
| Med12 | 0.28132 | -1.0246 | 9002 | |
| Daw1 | 0.28138 | -0.66271 | 9003 | |
| Rundc1 | 0.28144 | -0.27993 | 9004 | |
| Fto | 0.28147 | -1.0255 | 9005 | |
| Msrb2 | 0.28149 | -0.65785 | 9006 | |
| Recql | 0.2815 | -0.77512 | 9007 | |
| Srms | 0.28152 | -1.0271 | 9008 | |
| Birc3 | 0.28158 | -1.0294 | 9009 | |
| Zfp296 | 0.28165 | -1.0094 | 9010 | |
| Exph5 | 0.28167 | -1.014 | 9011 | |
| Cntn6 | 0.28171 | -0.35887 | 9012 | |
| Krt75 | 0.28173 | 0.078696 | 9013 | |
| Snrpb2 | 0.2818 | -0.74413 | 9014 | |
| Cabp2 | 0.28183 | -1.0215 | 9015 | |
| Bik | 0.28194 | -1.018 | 9016 | |
| Olfr360 | 0.28202 | -0.31601 | 9017 | |
| Muc2 | 0.28202 | -0.29803 | 9018 | |
| Cd48 | 0.28204 | -1.0157 | 9019 | |
| Fam124b | 0.28206 | 0.1214 | 9020 | |
| Acin1 | 0.28208 | -0.38173 | 9021 | |
| Card6 | 0.28214 | -1.0276 | 9022 | |
| Pxmp4 | 0.28216 | -1.0031 | 9023 | |
| Txndc2 | 0.28217 | -0.050244 | 9024 | |
| mmu-mir-711 | 0.28225 | -0.65128 | 9025 | |
| Sgsm1 | 0.28227 | -1.0225 | 9026 | |
| Tpd52 | 0.28228 | -0.2763 | 9027 | |
| Ankrd13d | 0.28231 | -0.83872 | 9028 | |
| Hdgfl1 | 0.28233 | -1.0203 | 9029 | |
| Rab4b | 0.28235 | 0.15815 | 9030 | |
| Sp9 | 0.28239 | -1.0262 | 9031 | |
| Frrs1l | 0.28242 | -0.85568 | 9032 | |
| Dcx | 0.28243 | -1.0208 | 9033 | |
| Mut | 0.28253 | -0.58747 | 9034 | |
| Btg2 | 0.28254 | -0.46423 | 9035 | |
| St5 | 0.28258 | -1.0214 | 9036 | |
| Tdg | 0.28267 | -1.019 | 9037 | |
| Poll | 0.28268 | -0.75089 | 9038 | |
| Limd2 | 0.28274 | -1.0201 | 9039 | |
| Sox5 | 0.28277 | -0.34749 | 9040 | |
| Olfr199 | 0.28292 | -1.0212 | 9041 | |
| mmu-mir-378b | 0.28296 | -0.91406 | 9042 | |
| Gzmg | 0.28297 | -0.78025 | 9043 | |
| Tmco6 | 0.28308 | -0.98855 | 9044 | |
| Islr2 | 0.28319 | 0.12832 | 9045 | |
| Srsf7 | 0.2832 | -1.0213 | 9046 | |
| Gm2696 | 0.28328 | -1.0224 | 9047 | |
| Ms4a18 | 0.28333 | -1.0186 | 9048 | |
| Rtl1 | 0.28334 | -0.49702 | 9049 | |
| Ces2c | 0.28341 | -0.35093 | 9050 | |
| Naa35 | 0.28348 | -1.012 | 9051 | |
| Cox4i1 | 0.28355 | -0.12082 | 9052 | |
| Ndc80 | 0.28359 | 0.37569 | 9053 | |
| Gli2 | 0.28362 | -1.0121 | 9054 | |
| Dlgap5 | 0.28363 | 0.35498 | 9055 | |
| Samd4 | 0.2837 | -1.0188 | 9056 | |
| Fmo6 | 0.28372 | -0.84483 | 9057 | |
| Vmn1r8 | 0.28388 | 0.31572 | 9058 | |
| Cyp4a12b | 0.28392 | 0.13867 | 9059 | |
| Irx4 | 0.28393 | -0.73903 | 9060 | |
| Ptprn | 0.28403 | -0.23773 | 9061 | |
| Srrm3 | 0.28405 | -1.0137 | 9062 | |
| Stmn1 | 0.2841 | 0.21801 | 9063 | |
| Rrp8 | 0.28414 | 1.1847 | 9064 | |
| Mllt1 | 0.28418 | -1.0204 | 9065 | |
| Gtf2i | 0.28426 | -1.0015 | 9066 | |
| Ing1 | 0.28428 | -0.55858 | 9067 | |
| Necab2 | 0.2843 | -1.0124 | 9068 | |
| Gpr142 | 0.28436 | -0.3377 | 9069 | |
| Lhfpl2 | 0.28436 | -0.41701 | 9070 | |
| 2610528A11Rik | 0.28449 | -1.0173 | 9071 | |
| Kif3b | 0.2845 | -0.67183 | 9072 | |
| Ins1 | 0.28451 | -1.0139 | 9073 | |
| Olfr67 | 0.28453 | -0.029869 | 9074 | |
| Cryab | 0.28461 | -0.83104 | 9075 | |
| mmu-mir-6407 | 0.28462 | -0.45649 | 9076 | |
| Tgm1 | 0.28467 | -1.0065 | 9077 | |
| Krt8 | 0.28482 | -0.99476 | 9078 | |
| Crygc | 0.28483 | -0.37276 | 9079 | |
| Proz | 0.28488 | -0.89618 | 9080 | |
| mmu-mir-8099-1 | 0.28501 | -0.52466 | 9081 | |
| Zfp750 | 0.28505 | -0.88655 | 9082 | |
| Zfp768 | 0.28513 | -1.0036 | 9083 | |
| Stra8 | 0.28518 | 0.6949 | 9084 | |
| Gm12695 | 0.28525 | 0.31643 | 9085 | |
| Gm10639 | 0.28538 | -0.32627 | 9086 | |
| Fam102b | 0.2854 | -0.64129 | 9087 | |
| Qpct | 0.28541 | 0.081533 | 9088 | |
| Slc47a2 | 0.28546 | -0.14928 | 9089 | |
| Prl2c1 | 0.28559 | -0.094061 | 9090 | |
| mmu-mir-1251 | 0.2856 | -0.55254 | 9091 | |
| Rap1a | 0.28563 | -0.63382 | 9092 | |
| Lrp3 | 0.28563 | -1.0113 | 9093 | |
| mmu-mir-28a | 0.28581 | 0.17059 | 9094 | |
| Apoc1 | 0.28583 | -0.8049 | 9095 | |
| Olfr283 | 0.28584 | -0.97124 | 9096 | |
| Nr4a3 | 0.28592 | -0.21966 | 9097 | |
| Bri3 | 0.28594 | -1.009 | 9098 | |
| Rassf9 | 0.28615 | -1.0113 | 9099 | |
| Apol7c | 0.28618 | -0.21816 | 9100 | |
| Uevld | 0.28618 | -1.0109 | 9101 | |
| A430089I19Rik | 0.28627 | -1.0132 | 9102 | |
| Mib2 | 0.2863 | -0.99232 | 9103 | |
| Slc46a3 | 0.28632 | -1.0036 | 9104 | |
| 2810459M11Rik | 0.28636 | -1.0123 | 9105 | |
| Tmed7 | 0.28644 | -0.15474 | 9106 | |
| Olfr330 | 0.28647 | -0.37585 | 9107 | |
| Serpinb2 | 0.28658 | -0.096652 | 9108 | |
| Vmn1r149 | 0.2866 | -1.3052 | 9109 | |
| Barhl1 | 0.28661 | -0.45129 | 9110 | |
| Ppp1r9a | 0.28663 | -0.44306 | 9111 | |
| Cnnm3 | 0.28667 | -1.0125 | 9112 | |
| Slc24a4 | 0.28671 | -1.0022 | 9113 | |
| Pwwp2b | 0.28687 | -0.21293 | 9114 | |
| Ptges3l | 0.28688 | -0.39815 | 9115 | |
| Napg | 0.2869 | 0.14248 | 9116 | |
| Polr2i | 0.28692 | -0.96538 | 9117 | |
| Fgfbp3 | 0.28694 | -0.13612 | 9118 | |
| Gabrb2 | 0.28694 | -1.0095 | 9119 | |
| Mgarp | 0.28697 | -0.99868 | 9120 | |
| Rb1 | 0.28702 | -1.0045 | 9121 | |
| Slc7a13 | 0.28705 | 0.84122 | 9122 | |
| Fam196a | 0.28712 | -0.95093 | 9123 | |
| Gng11 | 0.28721 | -1.0002 | 9124 | |
| Defa25 | 0.28724 | -0.18423 | 9125 | |
| Slc35f2 | 0.28727 | 0.67 | 9126 | |
| Tbc1d2 | 0.28728 | -1.0117 | 9127 | |
| Paip1 | 0.28737 | 1.633 | 9128 | |
| Gm13769 | 0.28752 | -0.95183 | 9129 | |
| Olfr657 | 0.28756 | -1.011 | 9130 | |
| Tns4 | 0.28763 | -0.99417 | 9131 | |
| Klf2 | 0.28763 | -0.97862 | 9132 | |
| 1700122O11Rik | 0.28766 | -0.46528 | 9133 | |
| Zfp809 | 0.28773 | -0.74097 | 9134 | |
| mmu-mir-5130 | 0.28775 | -0.80984 | 9135 | |
| Epas1 | 0.28781 | 0.019542 | 9136 | |
| Fam104a | 0.28785 | -0.21598 | 9137 | |
| 1700006A11Rik | 0.28788 | -0.09681 | 9138 | |
| Fam188a | 0.28792 | -1.0047 | 9139 | |
| Tsnax | 0.28792 | -0.34146 | 9140 | |
| Echdc2 | 0.28796 | -0.45887 | 9141 | |
| Pin1rt1 | 0.28801 | -0.64773 | 9142 | |
| Olfr474 | 0.28804 | -1.004 | 9143 | |
| l7Rn6 | 0.28806 | -0.88423 | 9144 | |
| Cript | 0.28813 | -0.50788 | 9145 | |
| Polr3gl | 0.28814 | -0.28448 | 9146 | |
| Dennd2c | 0.28817 | -0.64863 | 9147 | |
| Olfr178 | 0.28819 | -0.86413 | 9148 | |
| Msantd3 | 0.28835 | -1.0061 | 9149 | |
| Tifab | 0.28835 | -0.36726 | 9150 | |
| Hdhd2 | 0.28844 | -1.0075 | 9151 | |
| Lap3 | 0.28855 | 0.65593 | 9152 | |
| Rasl10b | 0.28861 | -1.0012 | 9153 | |
| Try4 | 0.28868 | -0.62352 | 9154 | |
| Sulf1 | 0.28872 | 0.48931 | 9155 | |
| Vmn1r15 | 0.28877 | -0.24724 | 9156 | |
| Lsm2 | 0.28877 | -0.87632 | 9157 | |
| 4930526D03Rik | 0.28888 | -0.99447 | 9158 | |
| Plcl2 | 0.2889 | -0.44833 | 9159 | |
| Arl9 | 0.28908 | -0.45813 | 9160 | |
| Bag2 | 0.28913 | -0.01244 | 9161 | |
| Polr2k | 0.28919 | -0.76874 | 9162 | |
| Uck1 | 0.28922 | -0.50722 | 9163 | |
| Tmem139 | 0.28923 | -0.93839 | 9164 | |
| 0610007P14Rik | 0.28928 | -1.0045 | 9165 | |
| Gpr37l1 | 0.28932 | -0.87235 | 9166 | |
| Zfp651 | 0.28941 | -0.044179 | 9167 | |
| Krtap31-1 | 0.28946 | -1.0053 | 9168 | |
| G6pd2 | 0.28946 | -0.13863 | 9169 | |
| Map1s | 0.28948 | -0.81595 | 9170 | |
| Syngap1 | 0.28953 | -0.75177 | 9171 | |
| Usp17la | 0.28957 | -0.99707 | 9172 | |
| Trim69 | 0.28959 | -0.62829 | 9173 | |
| Gnai3 | 0.28961 | -0.77763 | 9174 | |
| Pik3c2b | 0.28962 | 1.3498 | 9175 | |
| Fzd1 | 0.28963 | -0.98848 | 9176 | |
| Commd2 | 0.28966 | -0.26809 | 9177 | |
| Aip | 0.28969 | -1.0038 | 9178 | |
| Mycs | 0.28973 | 0.38613 | 9179 | |
| Olfr984 | 0.28986 | -0.91083 | 9180 | |
| Cdkl2 | 0.28988 | -0.97406 | 9181 | |
| Zfp758 | 0.28996 | -0.41916 | 9182 | |
| Gm5464 | 0.29002 | -0.19018 | 9183 | |
| Spic | 0.29008 | 0.1762 | 9184 | |
| 9930111J21Rik1 | 0.29017 | -0.40318 | 9185 | |
| Dnajc15 | 0.29024 | -0.61171 | 9186 | |
| Tmem232 | 0.29025 | -0.99465 | 9187 | |
| Olfr190 | 0.29031 | -0.26006 | 9188 | |
| Slc30a9 | 0.29032 | -0.98877 | 9189 | |
| Erich1 | 0.29036 | -0.99016 | 9190 | |
| Nat9 | 0.29042 | -0.32544 | 9191 | |
| Tgif1 | 0.29042 | -0.99946 | 9192 | |
| Ccdc163 | 0.29044 | -0.9958 | 9193 | |
| Sec23b | 0.29053 | -0.8845 | 9194 | |
| Zfp467 | 0.29053 | -0.45235 | 9195 | |
| Parp8 | 0.29057 | -1.001 | 9196 | |
| Tmco2 | 0.29071 | -0.99189 | 9197 | |
| Als2 | 0.29078 | -0.29676 | 9198 | |
| Rtn4rl1 | 0.2908 | -0.99833 | 9199 | |
| Car4 | 0.29085 | -0.19306 | 9200 | |
| Dock8 | 0.29088 | -0.55992 | 9201 | |
| mmu-mir-598 | 0.29089 | -0.18576 | 9202 | |
| Antxr1 | 0.2909 | -0.99525 | 9203 | |
| Napepld | 0.29092 | -0.99415 | 9204 | |
| mmu-mir-7089 | 0.29101 | -0.67015 | 9205 | |
| F5 | 0.29104 | -0.99817 | 9206 | |
| Tyw3 | 0.29113 | -0.99754 | 9207 | |
| Gm4907 | 0.29118 | -0.41572 | 9208 | |
| Efcab6 | 0.29118 | -0.99943 | 9209 | |
| Fxyd3 | 0.29122 | 0.71724 | 9210 | |
| Obox5 | 0.29129 | 0.19924 | 9211 | |
| Raly | 0.2913 | -0.20541 | 9212 | |
| AW554918 | 0.29134 | 0.032272 | 9213 | |
| Fen1 | 0.29139 | -0.99593 | 9214 | |
| 1700016H13Rik | 0.29142 | -0.99818 | 9215 | |
| Mfap4 | 0.29147 | -0.028916 | 9216 | |
| Taf4b | 0.29149 | -0.61077 | 9217 | |
| Il12a | 0.29165 | -0.99529 | 9218 | |
| Klra6 | 0.29166 | -1.2838 | 9219 | |
| mmu-mir-1896 | 0.29166 | -0.88931 | 9220 | |
| 8430427H17Rik | 0.29174 | -0.99071 | 9221 | |
| Taar8a | 0.29183 | -0.83363 | 9222 | |
| Mc5r | 0.29197 | -0.99652 | 9223 | |
| Gramd1a | 0.29205 | -0.47368 | 9224 | |
| Stac2 | 0.29208 | -0.86974 | 9225 | |
| Mcm8 | 0.29212 | 0.1409 | 9226 | |
| Ublcp1 | 0.29223 | 0.48801 | 9227 | |
| Cdr2 | 0.29225 | -0.98725 | 9228 | |
| Olfr191 | 0.29227 | -0.9476 | 9229 | |
| Krt4 | 0.29237 | -0.95966 | 9230 | |
| Taf15 | 0.29237 | -0.82014 | 9231 | |
| Asic1 | 0.29241 | -0.94329 | 9232 | |
| mmu-mir-666 | 0.29244 | -0.26311 | 9233 | |
| Fancc | 0.29253 | -0.82395 | 9234 | |
| Zfp955b | 0.29255 | 0.45935 | 9235 | |
| Tmem143 | 0.29266 | 0.0015042 | 9236 | |
| Slu7 | 0.29277 | -0.83666 | 9237 | |
| Gpr98 | 0.2928 | -0.25617 | 9238 | |
| Ifitm2 | 0.29295 | -0.48442 | 9239 | |
| Hcn3 | 0.29297 | -0.73781 | 9240 | |
| Stard8 | 0.29299 | -0.6172 | 9241 | |
| Pten | 0.29299 | -0.97986 | 9242 | |
| mmu-mir-668 | 0.293 | -0.7566 | 9243 | |
| Vmn1r215 | 0.29302 | -0.98782 | 9244 | |
| Olfr781 | 0.29306 | 0.109 | 9245 | |
| Cog1 | 0.2931 | 0.029876 | 9246 | |
| Il21r | 0.29313 | -0.99125 | 9247 | |
| Mpo | 0.29318 | -0.89116 | 9248 | |
| mmu-mir-7088 | 0.29326 | -0.062311 | 9249 | |
| Wnt16 | 0.29329 | -0.97383 | 9250 | |
| Pllp | 0.29335 | -0.98203 | 9251 | |
| Eppin | 0.2934 | -0.98411 | 9252 | |
| Top3b | 0.29342 | -0.51237 | 9253 | |
| Sox18 | 0.29346 | -0.76691 | 9254 | |
| Col1a2 | 0.29364 | -0.98954 | 9255 | |
| mmu-mir-6955 | 0.29365 | 0.48333 | 9256 | |
| Slc35d1 | 0.29367 | -0.53066 | 9257 | |
| Fam150b | 0.29375 | -0.66066 | 9258 | |
| Kif20b | 0.29381 | -0.98433 | 9259 | |
| Nhlh1 | 0.29383 | -0.96554 | 9260 | |
| Cd44 | 0.29389 | -0.62048 | 9261 | |
| Gm7102 | 0.29391 | -0.71635 | 9262 | |
| Slc2a6 | 0.29396 | -0.98716 | 9263 | |
| Fbxo40 | 0.29418 | 0.15474 | 9264 | |
| Ifi47 | 0.29421 | -0.94844 | 9265 | |
| Trim27 | 0.29422 | 0.62633 | 9266 | |
| Psmc3ip | 0.29423 | -0.98463 | 9267 | |
| Aldh6a1 | 0.29429 | -0.39769 | 9268 | |
| Pawr | 0.29433 | -0.98155 | 9269 | |
| Olfr1336 | 0.29435 | -0.98354 | 9270 | |
| Nptx2 | 0.29436 | -0.11817 | 9271 | |
| Armc6 | 0.29437 | -0.97621 | 9272 | |
| Hnf4g | 0.29443 | -0.67161 | 9273 | |
| Higd1b | 0.2945 | -0.65851 | 9274 | |
| Prss50 | 0.29458 | 0.72447 | 9275 | |
| Nlrp4e | 0.29461 | -0.64337 | 9276 | |
| Adamts9 | 0.29467 | -0.10251 | 9277 | |
| Fezf1 | 0.29468 | 0.85615 | 9278 | |
| Dennd4c | 0.29476 | -0.19049 | 9279 | |
| Cldn19 | 0.29486 | -0.98595 | 9280 | |
| Vmn1r122 | 0.29488 | -1.2558 | 9281 | |
| 8430419L09Rik | 0.2949 | 0.41839 | 9282 | |
| 1600014C23Rik | 0.29492 | -0.97079 | 9283 | |
| mmu-mir-6769b | 0.29497 | -0.010422 | 9284 | |
| Shisa2 | 0.29501 | -0.29989 | 9285 | |
| Msh5 | 0.29502 | -0.0051683 | 9286 | |
| mmu-mir-495 | 0.29509 | -0.32867 | 9287 | |
| Fabp12 | 0.29515 | -0.93699 | 9288 | |
| Coq10b | 0.29519 | 0.057143 | 9289 | |
| Slc39a3 | 0.29523 | -0.021629 | 9290 | |
| Sept10 | 0.29528 | 0.93227 | 9291 | |
| Srsf9 | 0.2953 | -0.98234 | 9292 | |
| Phf1 | 0.29537 | 0.10643 | 9293 | |
| Myo5c | 0.29538 | -0.98557 | 9294 | |
| Olfr1258 | 0.29542 | -0.8636 | 9295 | |
| Olfr521 | 0.29542 | 0.25487 | 9296 | |
| Gm4861 | 0.29546 | -0.98191 | 9297 | |
| Myl6b | 0.29551 | -0.9867 | 9298 | |
| Olfr58 | 0.29553 | -0.8458 | 9299 | |
| Mov10 | 0.29556 | -0.144 | 9300 | |
| Klhl13 | 0.29559 | -0.34905 | 9301 | |
| Pira6 | 0.29562 | -0.91382 | 9302 | |
| Atxn3 | 0.29573 | -0.98696 | 9303 | |
| Zfp513 | 0.29573 | -0.063364 | 9304 | |
| Cldn6 | 0.29577 | -0.55364 | 9305 | |
| Mt4 | 0.29584 | -0.78412 | 9306 | |
| mmu-mir-7045 | 0.29584 | -0.38248 | 9307 | |
| Lrrc72 | 0.29586 | -0.97351 | 9308 | |
| Htatsf1 | 0.29591 | -0.55242 | 9309 | |
| Nodal | 0.29595 | -0.17815 | 9310 | |
| Prkg1 | 0.29599 | -0.98161 | 9311 | |
| mmu-mir-7238 | 0.29617 | -0.50929 | 9312 | |
| Map2 | 0.2962 | -0.98133 | 9313 | |
| Zfp593 | 0.29625 | -0.98546 | 9314 | |
| Nr0b1 | 0.29629 | -0.98587 | 9315 | |
| Perp | 0.29641 | -0.28905 | 9316 | |
| Larp1b | 0.29649 | -0.97833 | 9317 | |
| Ogfod2 | 0.29656 | 0.19116 | 9318 | |
| Bbs2 | 0.29659 | -0.97193 | 9319 | |
| S100a8 | 0.2966 | -0.9756 | 9320 | |
| Hist1h3e | 0.29666 | -0.34094 | 9321 | |
| Olig1 | 0.2967 | 0.14898 | 9322 | |
| Cpne3 | 0.2967 | -0.97502 | 9323 | |
| Cphx1 | 0.29674 | 0.43705 | 9324 | |
| Lpcat4 | 0.29677 | -0.46733 | 9325 | |
| Srd5a3 | 0.29682 | -0.96588 | 9326 | |
| Atf7ip2 | 0.29682 | -0.95231 | 9327 | |
| 2510009E07Rik | 0.29687 | -0.14187 | 9328 | |
| Zfp821 | 0.29688 | -0.053662 | 9329 | |
| Pkd1 | 0.29689 | -0.98288 | 9330 | |
| Fam172a | 0.29693 | -0.98438 | 9331 | |
| Mzt1 | 0.29694 | 0.34999 | 9332 | |
| Cage1 | 0.29702 | -0.98197 | 9333 | |
| Mdga1 | 0.29703 | -0.24554 | 9334 | |
| 1600016N20Rik | 0.29713 | 0.56408 | 9335 | |
| Xlr5a | 0.29731 | -0.43692 | 9336 | |
| Abcb1b | 0.29735 | 0.55477 | 9337 | |
| Slc51b | 0.29738 | -0.35978 | 9338 | |
| Boll | 0.29744 | -0.97113 | 9339 | |
| Spag11a | 0.29746 | -0.97434 | 9340 | |
| Plekhg4 | 0.29749 | 0.35024 | 9341 | |
| Anxa8 | 0.2975 | -0.046642 | 9342 | |
| Gpt | 0.29754 | -0.23472 | 9343 | |
| Vmn2r43 | 0.29755 | -0.85684 | 9344 | |
| Mrgpra3 | 0.29771 | -0.92978 | 9345 | |
| Xlr4b | 0.29773 | -1.2514 | 9346 | |
| Cd4 | 0.29773 | -0.17811 | 9347 | |
| BC024978 | 0.29782 | -0.093593 | 9348 | |
| mmu-mir-3969 | 0.2979 | 0.45797 | 9349 | |
| Rhox10 | 0.29794 | -0.9809 | 9350 | |
| Pgm2l1 | 0.29794 | -0.9809 | 9351 | |
| Smgc | 0.29794 | -0.41261 | 9352 | |
| Angptl2 | 0.29794 | 0.67812 | 9353 | |
| Sulf2 | 0.29807 | -0.97805 | 9354 | |
| Nrbp2 | 0.29807 | -0.97805 | 9355 | |
| Serinc5 | 0.29815 | -0.97758 | 9356 | |
| Vmn2r114 | 0.29815 | -0.97758 | 9357 | |
| Daxx | 0.29818 | -0.16578 | 9358 | |
| Tcf23 | 0.29819 | -0.03662 | 9359 | |
| Vmn2r45 | 0.29822 | -0.83834 | 9360 | |
| Serpinf1 | 0.29825 | -0.95051 | 9361 | |
| mmu-mir-3097 | 0.29827 | 0.078704 | 9362 | |
| Eif3l | 0.29838 | -0.97637 | 9363 | |
| Pik3r4 | 0.2985 | -0.0079089 | 9364 | |
| Spata33 | 0.29851 | -0.2742 | 9365 | |
| F3 | 0.29853 | -0.68007 | 9366 | |
| Susd3 | 0.29854 | -0.94902 | 9367 | |
| 1700018B08Rik | 0.29859 | -0.89993 | 9368 | |
| Scgb1b29 | 0.29859 | -0.82132 | 9369 | |
| Col4a2 | 0.29864 | -0.97967 | 9370 | |
| Nkx2-4 | 0.2987 | 0.71777 | 9371 | |
| Gjc1 | 0.2987 | 0.35656 | 9372 | |
| Oacyl | 0.29879 | -0.46686 | 9373 | |
| Nxpe5 | 0.29887 | -0.9737 | 9374 | |
| Mapkbp1 | 0.29893 | -0.97891 | 9375 | |
| Hamp | 0.29899 | -0.89956 | 9376 | |
| Krtap22-2 | 0.29901 | -0.90704 | 9377 | |
| Mettl21c | 0.29904 | -0.14469 | 9378 | |
| Tdpoz2 | 0.29906 | -0.45456 | 9379 | |
| mmu-mir-350 | 0.29907 | -0.35311 | 9380 | |
| Hdgfrp3 | 0.2991 | -0.9691 | 9381 | |
| Wrap73 | 0.29918 | 0.86108 | 9382 | |
| Pkd1l2 | 0.2992 | -0.94303 | 9383 | |
| Gpr173 | 0.29922 | -0.32001 | 9384 | |
| Rnf181 | 0.29923 | -0.45743 | 9385 | |
| Phf14 | 0.29929 | -0.96356 | 9386 | |
| Syvn1 | 0.29939 | -0.96971 | 9387 | |
| Olfr875 | 0.29946 | -0.9707 | 9388 | |
| Dock5 | 0.29947 | -0.016283 | 9389 | |
| Adamdec1 | 0.29954 | -0.82075 | 9390 | |
| Arhgef15 | 0.29958 | 0.63836 | 9391 | |
| Trim12c | 0.2996 | -0.55489 | 9392 | |
| Gne | 0.29963 | -0.17501 | 9393 | |
| Hbq1a | 0.29965 | 0.51173 | 9394 | |
| Syn3 | 0.2997 | -0.70363 | 9395 | |
| Gpr15 | 0.29973 | -0.86804 | 9396 | |
| Hpcal1 | 0.29975 | -0.96551 | 9397 | |
| Lmod3 | 0.29979 | -0.9692 | 9398 | |
| Mgat4a | 0.29979 | -0.25623 | 9399 | |
| Olfr1500 | 0.29984 | -0.66983 | 9400 | |
| H2-Ob | 0.29987 | -0.49446 | 9401 | |
| Pcca | 0.2999 | -0.73666 | 9402 | |
| Cldn13 | 0.29994 | -0.30835 | 9403 | |
| Hebp2 | 0.29996 | -0.47573 | 9404 | |
| Chrna1 | 0.30003 | -0.96811 | 9405 | |
| Ino80c | 0.30004 | -0.42881 | 9406 | |
| Ccsap | 0.30006 | -0.97145 | 9407 | |
| Olfr393 | 0.30006 | -0.97145 | 9408 | |
| Krt24 | 0.3001 | -0.068388 | 9409 | |
| Enthd2 | 0.30024 | -0.97485 | 9410 | |
| Pdxdc1 | 0.30028 | 0.35048 | 9411 | |
| Ccdc94 | 0.30033 | -0.14826 | 9412 | |
| Ap3b2 | 0.3004 | 0.93704 | 9413 | |
| Zc3h13 | 0.30045 | -0.42962 | 9414 | |
| Gnaz | 0.30055 | -0.47126 | 9415 | |
| Adh4 | 0.30061 | -0.34421 | 9416 | |
| Sparc | 0.30062 | -0.23227 | 9417 | |
| Tac4 | 0.30064 | -0.96649 | 9418 | |
| Olfr71 | 0.30069 | 0.50539 | 9419 | |
| Zswim4 | 0.30073 | 0.78365 | 9420 | |
| Celsr3 | 0.3008 | -0.95921 | 9421 | |
| 9130401M01Rik | 0.30082 | 0.62393 | 9422 | |
| Tmcc2 | 0.30091 | 0.88356 | 9423 | |
| Gcm2 | 0.30098 | 0.26094 | 9424 | |
| Gata5 | 0.30105 | -0.77102 | 9425 | |
| Traf3ip3 | 0.30106 | -0.75276 | 9426 | |
| Xrra1 | 0.30112 | -0.96654 | 9427 | |
| Gm14325 | 0.30114 | -0.97211 | 9428 | |
| Bmp2 | 0.30118 | -0.96819 | 9429 | |
| mmu-mir-484 | 0.30128 | -0.66396 | 9430 | |
| Vmn2r7 | 0.30129 | -0.8797 | 9431 | |
| Aamdc | 0.30136 | -0.97159 | 9432 | |
| Olfr124 | 0.30136 | -0.93319 | 9433 | |
| Arrdc4 | 0.30148 | -0.38205 | 9434 | |
| Cenpj | 0.30152 | -0.95285 | 9435 | |
| mmu-mir-19b-1 | 0.30157 | -0.38656 | 9436 | |
| mmu-mir-22 | 0.30165 | -0.1666 | 9437 | |
| mmu-mir-8091 | 0.30165 | -0.75133 | 9438 | |
| Hist2h2aa1 | 0.30173 | -0.93742 | 9439 | |
| Olfr576 | 0.30176 | -0.96753 | 9440 | |
| Psmb5 | 0.30187 | -0.9679 | 9441 | |
| Cacna2d2 | 0.30191 | 0.63971 | 9442 | |
| mmu-mir-6905 | 0.30202 | -0.65624 | 9443 | |
| Nit2 | 0.30202 | -0.36298 | 9444 | |
| Ablim1 | 0.30205 | -0.20663 | 9445 | |
| Cwc27 | 0.30207 | -0.96855 | 9446 | |
| Trak2 | 0.3021 | -0.95766 | 9447 | |
| Atpaf1 | 0.3022 | 1.3467 | 9448 | |
| mmu-mir-6944 | 0.30223 | -0.80721 | 9449 | |
| Amfr | 0.30223 | -0.26371 | 9450 | |
| Slc15a5 | 0.30224 | -0.033477 | 9451 | |
| Ndufb9 | 0.30227 | -0.88327 | 9452 | |
| Ptprn2 | 0.3023 | -0.94849 | 9453 | |
| Bloc1s6 | 0.30239 | -0.031806 | 9454 | |
| Nagk | 0.30245 | -0.91171 | 9455 | |
| 1700023F06Rik | 0.3025 | -0.72447 | 9456 | |
| Rgs7 | 0.3025 | -0.58736 | 9457 | |
| Rars2 | 0.30251 | -0.96681 | 9458 | |
| Nit1 | 0.30264 | -0.96209 | 9459 | |
| Vwa5b1 | 0.30266 | -0.43805 | 9460 | |
| Kif3c | 0.30267 | -0.44394 | 9461 | |
| Pacrg | 0.3027 | -0.85427 | 9462 | |
| Piga | 0.30272 | -0.96198 | 9463 | |
| Pcdhb4 | 0.30273 | -0.52223 | 9464 | |
| Snd1 | 0.30274 | -0.96648 | 9465 | |
| Olfr1211 | 0.30277 | -0.58757 | 9466 | |
| Dnajb2 | 0.30277 | 0.2339 | 9467 | |
| Tnfaip8l1 | 0.30279 | -0.63225 | 9468 | |
| Efcab12 | 0.3028 | 1.7546 | 9469 | |
| Rit2 | 0.30289 | -0.95602 | 9470 | |
| Atxn7 | 0.30298 | -0.78252 | 9471 | |
| St6galnac6 | 0.30302 | 0.083207 | 9472 | |
| Rspo1 | 0.30302 | -0.94347 | 9473 | |
| Samt4 | 0.30312 | -0.96647 | 9474 | |
| Dnase2b | 0.30319 | -0.9583 | 9475 | |
| Bud13 | 0.30321 | -0.61641 | 9476 | |
| Olfr1325 | 0.30331 | -0.29393 | 9477 | |
| Gpr157 | 0.30331 | -0.42154 | 9478 | |
| Ifrd1 | 0.30338 | -0.32552 | 9479 | |
| 1810011O10Rik | 0.30338 | -0.84653 | 9480 | |
| Gm13124 | 0.30341 | -0.87829 | 9481 | |
| Lysmd2 | 0.30346 | -0.95108 | 9482 | |
| Prdm10 | 0.30347 | 0.016461 | 9483 | |
| Gkn1 | 0.3035 | -0.96336 | 9484 | |
| Olfr735 | 0.30352 | 0.085236 | 9485 | |
| Fam83g | 0.30355 | -0.95919 | 9486 | |
| Fam198a | 0.30356 | 0.66004 | 9487 | |
| Olfr299 | 0.30359 | -0.74992 | 9488 | |
| Arrdc5 | 0.30363 | -0.73428 | 9489 | |
| Olfr878 | 0.30365 | -0.962 | 9490 | |
| Dok2 | 0.30367 | -0.95948 | 9491 | |
| Fam122b | 0.30372 | -0.95739 | 9492 | |
| Fads6 | 0.30375 | -0.69184 | 9493 | |
| Nnt | 0.30375 | 0.1456 | 9494 | |
| Fnip2 | 0.30395 | -0.88373 | 9495 | |
| Uty | 0.30412 | -0.93449 | 9496 | |
| Bahcc1 | 0.30418 | -0.8752 | 9497 | |
| 9530003J23Rik | 0.30424 | -0.96201 | 9498 | |
| Olfr311 | 0.30427 | -0.82623 | 9499 | |
| Olfr164 | 0.30431 | -0.80742 | 9500 | |
| Jdp2 | 0.30434 | -0.080418 | 9501 | |
| mmu-mir-105 | 0.30439 | -0.63465 | 9502 | |
| Herc6 | 0.30448 | -0.15081 | 9503 | |
| Pepd | 0.30452 | -0.94286 | 9504 | |
| Magi3 | 0.30456 | -0.96202 | 9505 | |
| Gatm | 0.30467 | -0.95164 | 9506 | |
| Baiap2 | 0.3047 | -0.22455 | 9507 | |
| AK129341 | 0.30477 | 0.067769 | 9508 | |
| 1700026D08Rik | 0.30481 | -0.33579 | 9509 | |
| Ngef | 0.30502 | -0.26183 | 9510 | |
| Efemp1 | 0.30506 | -0.049943 | 9511 | |
| mmu-mir-1953 | 0.30507 | -0.13698 | 9512 | |
| Treml2 | 0.30507 | -0.85853 | 9513 | |
| Psg23 | 0.30515 | -0.42355 | 9514 | |
| Fbxo27 | 0.30526 | -0.93824 | 9515 | |
| Rock2 | 0.30527 | 0.46936 | 9516 | |
| Siah1a | 0.3054 | -0.10695 | 9517 | |
| Kcnk1 | 0.30547 | -0.59169 | 9518 | |
| Scfd1 | 0.30552 | 2.167 | 9519 | |
| mmu-mir-455 | 0.30577 | -0.66977 | 9520 | |
| Odf1 | 0.30581 | -0.27777 | 9521 | |
| Il6ra | 0.30585 | -0.94621 | 9522 | |
| Egfbp2 | 0.30595 | 1.347 | 9523 | |
| Olfr1350 | 0.306 | -0.93939 | 9524 | |
| Olfr1450 | 0.30602 | -0.32364 | 9525 | |
| Atp2a3 | 0.30606 | -0.94373 | 9526 | |
| 1100001G20Rik | 0.30611 | -0.95743 | 9527 | |
| mmu-mir-5108 | 0.30619 | 0.32802 | 9528 | |
| Nox1 | 0.3062 | -0.62556 | 9529 | |
| Mtmr1 | 0.3062 | -0.95544 | 9530 | |
| Kcnmb1 | 0.3062 | -0.95544 | 9531 | |
| Il2rg | 0.30625 | 0.019876 | 9532 | |
| Dusp28 | 0.30627 | -0.94257 | 9533 | |
| Slc12a4 | 0.30631 | -0.75535 | 9534 | |
| 1700065D16Rik | 0.30632 | -0.95267 | 9535 | |
| Pcdhb6 | 0.30638 | -0.9518 | 9536 | |
| Uhmk1 | 0.30641 | 0.44776 | 9537 | |
| mmu-mir-10b | 0.30643 | -0.36582 | 9538 | |
| Guca1a | 0.30671 | -0.9536 | 9539 | |
| Csnk1d | 0.30673 | -0.26329 | 9540 | |
| Scfd2 | 0.30678 | -0.1794 | 9541 | |
| Parp11 | 0.30682 | -0.27848 | 9542 | |
| Tmprss11a | 0.30687 | -0.86723 | 9543 | |
| mmu-mir-6997 | 0.30689 | 0.92849 | 9544 | |
| Rhox1 | 0.30689 | -0.90458 | 9545 | |
| Rnf34 | 0.30695 | -0.52958 | 9546 | |
| Stx11 | 0.307 | -0.85411 | 9547 | |
| Psmc6 | 0.30705 | 0.12905 | 9548 | |
| Pde1c | 0.30718 | -0.95 | 9549 | |
| Orc2 | 0.30729 | -0.79927 | 9550 | |
| Tpd52l2 | 0.3073 | 0.5875 | 9551 | |
| Vmn2r12 | 0.30735 | -0.94427 | 9552 | |
| Shisa3 | 0.30738 | -0.026543 | 9553 | |
| Cyp51 | 0.30741 | -0.53084 | 9554 | |
| Snap47 | 0.30754 | -0.93213 | 9555 | |
| S100a5 | 0.30757 | -0.75832 | 9556 | |
| Wnt10b | 0.3077 | 0.14157 | 9557 | |
| Sox10 | 0.30773 | -0.34335 | 9558 | |
| Kctd6 | 0.30778 | -0.94583 | 9559 | |
| Ggta1 | 0.3078 | -0.66675 | 9560 | |
| Olfr1040 | 0.3078 | -0.34647 | 9561 | |
| Mgat1 | 0.30787 | -0.86163 | 9562 | |
| Acap1 | 0.30793 | -0.94906 | 9563 | |
| Cplx1 | 0.30795 | -0.93645 | 9564 | |
| Olfr641 | 0.30801 | -0.94668 | 9565 | |
| Fam114a2 | 0.30802 | -0.079045 | 9566 | |
| Traf3 | 0.30803 | -0.72266 | 9567 | |
| Ulk3 | 0.3081 | -0.93448 | 9568 | |
| Zbtb7b | 0.30812 | 0.11283 | 9569 | |
| Glb1l2 | 0.30814 | -0.94623 | 9570 | |
| Siglech | 0.30816 | -0.93756 | 9571 | |
| Pex7 | 0.30824 | -0.63912 | 9572 | |
| mmu-mir-16-2 | 0.30825 | -0.89227 | 9573 | |
| Tns1 | 0.3083 | -0.49234 | 9574 | |
| Akap12 | 0.30834 | 0.024439 | 9575 | |
| Nabp1 | 0.30837 | -0.46095 | 9576 | |
| Ces1a | 0.30844 | -0.94576 | 9577 | |
| Fsd2 | 0.30844 | -0.86717 | 9578 | |
| Calcoco1 | 0.30846 | -0.94866 | 9579 | |
| Mob2 | 0.30854 | -0.16327 | 9580 | |
| Naa16 | 0.30855 | -0.27918 | 9581 | |
| Medag | 0.30859 | -0.72342 | 9582 | |
| Gm9125 | 0.30862 | -0.15225 | 9583 | |
| mmu-mir-92a-1 | 0.30868 | -0.77479 | 9584 | |
| 4930524B15Rik | 0.30868 | -0.27549 | 9585 | |
| Olfr820 | 0.30868 | -0.94485 | 9586 | |
| Slmo1 | 0.30869 | 1.4893 | 9587 | |
| Ocstamp | 0.30877 | -0.93534 | 9588 | |
| Gtf2ird2 | 0.3088 | -0.47009 | 9589 | |
| Vcpkmt | 0.30884 | 0.028278 | 9590 | |
| Lypla1 | 0.30884 | -0.93346 | 9591 | |
| Kat6b | 0.30888 | -0.94446 | 9592 | |
| Eno1 | 0.30907 | -0.94659 | 9593 | |
| Gm14295 | 0.30912 | -0.38361 | 9594 | |
| Olfr453 | 0.30918 | -0.47226 | 9595 | |
| Zbtb11 | 0.30926 | 0.32904 | 9596 | |
| mmu-mir-20a | 0.30928 | -0.81326 | 9597 | |
| Ndufa5 | 0.3093 | -0.92264 | 9598 | |
| Spint4 | 0.3093 | -0.46446 | 9599 | |
| Pifo | 0.30933 | -0.71508 | 9600 | |
| Npr1 | 0.30936 | -0.94135 | 9601 | |
| Olfr995 | 0.30936 | -0.94135 | 9602 | |
| 4931406C07Rik | 0.3094 | -0.94712 | 9603 | |
| Bicd2 | 0.30945 | -0.24938 | 9604 | |
| Cyp24a1 | 0.30948 | 1.0656 | 9605 | |
| D5Ertd579e | 0.30954 | -0.93472 | 9606 | |
| Defb42 | 0.30955 | -0.22113 | 9607 | |
| Rrbp1 | 0.30969 | -0.93096 | 9608 | |
| Mplkip | 0.30969 | -0.22849 | 9609 | |
| 4-Mar | 0.30971 | -0.93335 | 9610 | |
| Sdhaf1 | 0.30972 | -0.50664 | 9611 | |
| Itgb1bp1 | 0.30976 | -0.63265 | 9612 | |
| Cyb5d2 | 0.30983 | 1.3375 | 9613 | |
| Prps1l3 | 0.30994 | -0.94272 | 9614 | |
| Emc1 | 0.30996 | -0.93003 | 9615 | |
| Acvr2a | 0.30997 | 0.39545 | 9616 | |
| Mycbp2 | 0.31007 | -0.94516 | 9617 | |
| Usp10 | 0.31008 | -0.24918 | 9618 | |
| Brinp2 | 0.31018 | -0.22561 | 9619 | |
| Limk2 | 0.31024 | -0.93317 | 9620 | |
| Nudt18 | 0.31026 | -0.91693 | 9621 | |
| Usp19 | 0.31029 | -0.35258 | 9622 | |
| Tbccd1 | 0.31033 | -0.45972 | 9623 | |
| Setd6 | 0.31037 | -0.91671 | 9624 | |
| Htra4 | 0.31045 | -0.93651 | 9625 | |
| Uimc1 | 0.31047 | -0.35885 | 9626 | |
| Gpx4 | 0.31054 | -0.58008 | 9627 | |
| Hps3 | 0.31061 | -0.85834 | 9628 | |
| Riok3 | 0.31068 | -0.38507 | 9629 | |
| Pacrgl | 0.31068 | -0.9345 | 9630 | |
| Olfr924 | 0.31071 | -0.94303 | 9631 | |
| Slc41a1 | 0.31079 | -0.082343 | 9632 | |
| mmu-mir-551b | 0.31081 | -0.49852 | 9633 | |
| N4bp3 | 0.31081 | -0.569 | 9634 | |
| Serf1 | 0.3109 | 1.3228 | 9635 | |
| Ahcy | 0.311 | -0.3831 | 9636 | |
| Klhl6 | 0.31104 | 1.1202 | 9637 | |
| mmu-mir-6236 | 0.31111 | -0.92113 | 9638 | |
| Zfp781 | 0.31111 | -0.91473 | 9639 | |
| Ttc1 | 0.31111 | -0.33765 | 9640 | |
| Gys2 | 0.31128 | -0.93568 | 9641 | |
| Hcls1 | 0.31136 | 0.2174 | 9642 | |
| S1pr3 | 0.31139 | -0.93397 | 9643 | |
| Map1b | 0.31145 | -0.2706 | 9644 | |
| Mbl2 | 0.31146 | -0.63457 | 9645 | |
| Pbx2 | 0.31147 | -0.92283 | 9646 | |
| Fjx1 | 0.3115 | -0.63481 | 9647 | |
| Cox8a | 0.31156 | -0.93659 | 9648 | |
| Krt78 | 0.3116 | -0.92749 | 9649 | |
| Uros | 0.31164 | -0.93503 | 9650 | |
| Vapa | 0.3117 | -0.16648 | 9651 | |
| Scn8a | 0.31175 | 0.65327 | 9652 | |
| Gm17677 | 0.31181 | -0.9366 | 9653 | |
| Palm3 | 0.31182 | 0.15302 | 9654 | |
| Rab5c | 0.31187 | -0.84161 | 9655 | |
| Olfr214 | 0.31189 | 0.042885 | 9656 | |
| mmu-mir-467g | 0.3119 | -0.75076 | 9657 | |
| Zfp316 | 0.31193 | 0.20647 | 9658 | |
| Gmfb | 0.31205 | -0.92828 | 9659 | |
| Cars | 0.31209 | -0.93217 | 9660 | |
| H2-T3 | 0.31211 | -0.92766 | 9661 | |
| Pms1 | 0.31213 | -0.60571 | 9662 | |
| Ccdc47 | 0.31215 | -0.93665 | 9663 | |
| Hibch | 0.31221 | 1.4968 | 9664 | |
| Icos | 0.31222 | -0.93666 | 9665 | |
| Smtnl2 | 0.31226 | 0.89448 | 9666 | |
| Uqcr11 | 0.31232 | -0.67403 | 9667 | |
| Chga | 0.31232 | -0.93668 | 9668 | |
| Cog5 | 0.31235 | -0.64611 | 9669 | |
| Slc2a12 | 0.31239 | 0.072802 | 9670 | |
| Ano2 | 0.31245 | -0.91691 | 9671 | |
| Trpc3 | 0.31247 | -0.87568 | 9672 | |
| mmu-mir-6915 | 0.31249 | -0.46929 | 9673 | |
| Psen2 | 0.31253 | -0.35584 | 9674 | |
| Tas2r107 | 0.31258 | -0.92051 | 9675 | |
| Olfr196 | 0.3126 | 0.41476 | 9676 | |
| 4930522H14Rik | 0.31262 | -0.9193 | 9677 | |
| Syt7 | 0.31264 | 0.60021 | 9678 | |
| Sec23ip | 0.31276 | -0.93125 | 9679 | |
| Olfr692 | 0.31281 | -0.92514 | 9680 | |
| Dstyk | 0.31285 | 0.42184 | 9681 | |
| Csf3r | 0.31288 | -0.35292 | 9682 | |
| Ift81 | 0.31297 | -0.14616 | 9683 | |
| Xaf1 | 0.313 | -0.92579 | 9684 | |
| Lrrc15 | 0.31303 | 1.8581 | 9685 | |
| Snai2 | 0.31313 | -0.9371 | 9686 | |
| Fads3 | 0.31315 | -0.93706 | 9687 | |
| Rmi1 | 0.3132 | -0.031534 | 9688 | |
| Olfr116 | 0.31334 | 0.47135 | 9689 | |
| Pde3b | 0.31336 | -0.92741 | 9690 | |
| mmu-mir-767 | 0.3134 | -0.57138 | 9691 | |
| Caprin2 | 0.31341 | 1.0892 | 9692 | |
| Dock4 | 0.31345 | -0.31852 | 9693 | |
| Rpl41 | 0.31349 | -0.088409 | 9694 | |
| Ank3 | 0.31356 | 0.5764 | 9695 | |
| mmu-mir-6238 | 0.31356 | -0.5939 | 9696 | |
| Relb | 0.31359 | 0.5274 | 9697 | |
| Tbx10 | 0.31362 | -0.91129 | 9698 | |
| Pcmtd2 | 0.31366 | -0.80175 | 9699 | |
| Taok1 | 0.3137 | -0.92623 | 9700 | |
| Cdkn1c | 0.31373 | -0.92537 | 9701 | |
| Gtdc1 | 0.31377 | -0.89783 | 9702 | |
| Pdha1 | 0.3138 | -0.55534 | 9703 | |
| 1700001L19Rik | 0.31384 | -0.48335 | 9704 | |
| Plac1 | 0.31405 | -0.91474 | 9705 | |
| Cacng5 | 0.31405 | -0.62733 | 9706 | |
| Gpr4 | 0.31409 | -0.92681 | 9707 | |
| mmu-mir-216a | 0.31414 | -0.44315 | 9708 | |
| Rasgrp3 | 0.31416 | -0.50556 | 9709 | |
| Stradb | 0.31423 | 0.37443 | 9710 | |
| Mrgpra4 | 0.3143 | -0.92294 | 9711 | |
| 9430023L20Rik | 0.31444 | -0.87187 | 9712 | |
| AU022252 | 0.31451 | -0.29 | 9713 | |
| Cercam | 0.31458 | -0.89753 | 9714 | |
| Tmem86a | 0.31465 | -0.92014 | 9715 | |
| Fam189a2 | 0.31468 | -0.69422 | 9716 | |
| Kiss1 | 0.31471 | -0.90156 | 9717 | |
| Nod2 | 0.31473 | -0.91395 | 9718 | |
| Gulp1 | 0.3148 | -0.58757 | 9719 | |
| Vmn1r236 | 0.31481 | -0.92988 | 9720 | |
| H2-M11 | 0.31488 | -0.1025 | 9721 | |
| Gareml | 0.31497 | 0.66277 | 9722 | |
| Ptpn7 | 0.31503 | -0.9201 | 9723 | |
| 2210408I21Rik | 0.31504 | -0.054746 | 9724 | |
| 3110047P20Rik | 0.31507 | -0.57798 | 9725 | |
| Frmd3 | 0.31509 | -0.80885 | 9726 | |
| Wfdc9 | 0.31511 | -0.65318 | 9727 | |
| mmu-mir-191 | 0.31517 | -0.53427 | 9728 | |
| Klk1b1 | 0.31517 | -0.92351 | 9729 | |
| Poteg | 0.31522 | -0.59004 | 9730 | |
| Chpf2 | 0.31533 | -0.29417 | 9731 | |
| Csf1r | 0.31536 | 0.66247 | 9732 | |
| Gsg2 | 0.31539 | -0.93027 | 9733 | |
| Mlana | 0.31552 | -0.9218 | 9734 | |
| Olfr1098 | 0.31552 | -0.0030888 | 9735 | |
| Fat4 | 0.31552 | -0.19726 | 9736 | |
| Olfr221 | 0.31558 | -0.32024 | 9737 | |
| Lrrc8e | 0.31561 | 1.0379 | 9738 | |
| Anp32e | 0.31567 | -0.57662 | 9739 | |
| Atp11a | 0.31569 | -0.92226 | 9740 | |
| Zfat | 0.31573 | -0.92545 | 9741 | |
| Tmem178 | 0.31576 | -0.71824 | 9742 | |
| Vmn1r216 | 0.31579 | 0.57624 | 9743 | |
| Ric3 | 0.31582 | 0.94055 | 9744 | |
| Zbtb41 | 0.31586 | -0.23286 | 9745 | |
| Syce3 | 0.31588 | -0.9154 | 9746 | |
| Pkdrej | 0.31589 | 0.72517 | 9747 | |
| Noto | 0.31609 | -0.92787 | 9748 | |
| Rab3b | 0.31611 | -0.91337 | 9749 | |
| Rbm44 | 0.31616 | -0.7338 | 9750 | |
| Aldh18a1 | 0.3162 | -0.92198 | 9751 | |
| Akr1d1 | 0.31624 | -0.92623 | 9752 | |
| Fbxo21 | 0.31632 | -0.49602 | 9753 | |
| Stap2 | 0.31645 | -0.35956 | 9754 | |
| Bet1 | 0.31648 | -0.92738 | 9755 | |
| Olfr726 | 0.31651 | -0.34164 | 9756 | |
| Cadm1 | 0.31654 | -0.85785 | 9757 | |
| Mfsd5 | 0.31656 | -0.91748 | 9758 | |
| mmu-mir-6347 | 0.31656 | -0.35988 | 9759 | |
| Tecr | 0.31656 | -0.12719 | 9760 | |
| Hilpda | 0.3166 | 0.88618 | 9761 | |
| Pex16 | 0.31662 | -0.7763 | 9762 | |
| Trappc12 | 0.31667 | -0.91107 | 9763 | |
| Pitpnm1 | 0.31669 | -0.90812 | 9764 | |
| Rbms1 | 0.3167 | 0.18836 | 9765 | |
| Arl10 | 0.31677 | -0.29233 | 9766 | |
| Gdf10 | 0.31688 | -0.30573 | 9767 | |
| Ccdc42b | 0.31695 | 1.192 | 9768 | |
| 1700024G13Rik | 0.31701 | -0.68403 | 9769 | |
| Cyp2j8 | 0.31702 | -0.23482 | 9770 | |
| BC030476 | 0.31705 | -0.92102 | 9771 | |
| Glb1 | 0.3172 | -0.8257 | 9772 | |
| A1bg | 0.31727 | 0.35597 | 9773 | |
| 1700017N19Rik | 0.3173 | -0.80383 | 9774 | |
| Acsm2 | 0.31733 | -0.90316 | 9775 | |
| Scn10a | 0.31737 | 0.24476 | 9776 | |
| Rpl22 | 0.31745 | 0.090722 | 9777 | |
| Asb16 | 0.31748 | -0.4287 | 9778 | |
| Pitpnc1 | 0.3175 | -0.070806 | 9779 | |
| Naalad2 | 0.31754 | -0.83976 | 9780 | |
| Vmn2r89 | 0.31755 | 1.8884 | 9781 | |
| Ppp1r1c | 0.31759 | -0.91031 | 9782 | |
| Amer2 | 0.31759 | 0.43706 | 9783 | |
| Dtwd2 | 0.31763 | -0.50608 | 9784 | |
| Plxnd1 | 0.31765 | -0.90014 | 9785 | |
| 4930538K18Rik | 0.31766 | 0.72687 | 9786 | |
| Sh3pxd2a | 0.31769 | -0.37716 | 9787 | |
| Acsf3 | 0.31769 | -0.19154 | 9788 | |
| Cd1d2 | 0.31776 | -0.91309 | 9789 | |
| Armcx4 | 0.3178 | -0.14313 | 9790 | |
| Ccdc121 | 0.3178 | -0.90156 | 9791 | |
| Bco2 | 0.31782 | -0.91587 | 9792 | |
| Cep57l1 | 0.31784 | -0.91957 | 9793 | |
| Pla2g10 | 0.3179 | -0.77282 | 9794 | |
| Scnn1g | 0.31794 | 1.119 | 9795 | |
| Ltbp3 | 0.31797 | 0.13207 | 9796 | |
| Mybpc2 | 0.31799 | -0.90105 | 9797 | |
| Pwp1 | 0.31801 | -0.19523 | 9798 | |
| Scgb2b19 | 0.31811 | -0.91963 | 9799 | |
| Supt16 | 0.31815 | 0.55042 | 9800 | |
| Resp18 | 0.31822 | -0.6358 | 9801 | |
| Ube2z | 0.31831 | -0.90962 | 9802 | |
| Cutal | 0.31833 | 0.84476 | 9803 | |
| Adamts17 | 0.31842 | -0.91233 | 9804 | |
| Smim20 | 0.31844 | -0.62397 | 9805 | |
| Krtap6-5 | 0.31847 | -0.75658 | 9806 | |
| Bmp15 | 0.31854 | -0.089098 | 9807 | |
| Gbe1 | 0.31861 | -0.87346 | 9808 | |
| Strbp | 0.31861 | -0.91291 | 9809 | |
| A530064D06Rik | 0.31868 | 0.20337 | 9810 | |
| Mthfd1l | 0.31875 | -0.13113 | 9811 | |
| mmu-mir-6363 | 0.31884 | 0.21346 | 9812 | |
| Sult4a1 | 0.31893 | -0.39618 | 9813 | |
| Nrp1 | 0.31895 | -0.91858 | 9814 | |
| Tubg2 | 0.31896 | -0.14008 | 9815 | |
| Ap5m1 | 0.31907 | -0.064615 | 9816 | |
| Ppapdc1a | 0.3191 | -0.91791 | 9817 | |
| Bhmt2 | 0.31917 | -0.91366 | 9818 | |
| Olfr70 | 0.31919 | -0.86406 | 9819 | |
| Tuba8 | 0.31924 | -0.21355 | 9820 | |
| Wdyhv1 | 0.31928 | 2.108 | 9821 | |
| Ythdf1 | 0.3193 | -0.90011 | 9822 | |
| mmu-mir-8097 | 0.31934 | 0.67069 | 9823 | |
| Gnb2l1 | 0.31935 | -0.91717 | 9824 | |
| Psme3 | 0.31938 | -0.40938 | 9825 | |
| Msra | 0.31946 | -0.91818 | 9826 | |
| Cd74 | 0.31949 | -0.90462 | 9827 | |
| mmu-mir-6377 | 0.31949 | 0.49402 | 9828 | |
| Slc20a1 | 0.31959 | -0.22408 | 9829 | |
| Katna1 | 0.31963 | -0.91137 | 9830 | |
| Dkk3 | 0.31963 | -0.53599 | 9831 | |
| Htr4 | 0.3197 | -0.91547 | 9832 | |
| Ogfod1 | 0.31974 | 0.092785 | 9833 | |
| Ccdc32 | 0.3198 | -0.49156 | 9834 | |
| Vmn2r52 | 0.31981 | 0.54305 | 9835 | |
| Tox4 | 0.31983 | -0.91418 | 9836 | |
| Esp18 | 0.31987 | -0.8801 | 9837 | |
| Cox7b | 0.31991 | -0.42817 | 9838 | |
| Bin3 | 0.32002 | -0.15675 | 9839 | |
| Lhpp | 0.32004 | -0.90872 | 9840 | |
| Olfr1079 | 0.32009 | -0.90253 | 9841 | |
| Pex12 | 0.32019 | -0.91538 | 9842 | |
| Ccdc141 | 0.32023 | 1.075 | 9843 | |
| Tmem101 | 0.32028 | -0.38318 | 9844 | |
| Tpm2 | 0.3203 | 1.42 | 9845 | |
| Grem1 | 0.32037 | -0.3138 | 9846 | |
| Dspp | 0.32044 | -0.50674 | 9847 | |
| Hgfac | 0.32047 | -0.91141 | 9848 | |
| Fahd2a | 0.32063 | -0.36869 | 9849 | |
| Tmem40 | 0.32071 | -0.90263 | 9850 | |
| Pgbd1 | 0.32076 | 0.025448 | 9851 | |
| mmu-mir-5624 | 0.32078 | -0.91484 | 9852 | |
| H2-Ke6 | 0.32079 | 0.24232 | 9853 | |
| Eml4 | 0.32083 | 0.31607 | 9854 | |
| Rora | 0.32094 | -0.91253 | 9855 | |
| Pithd1 | 0.32094 | -0.91253 | 9856 | |
| Emc7 | 0.321 | -0.16589 | 9857 | |
| Adamts12 | 0.32107 | 0.48922 | 9858 | |
| H1foo | 0.32114 | 0.11928 | 9859 | |
| Asb13 | 0.32118 | 0.62399 | 9860 | |
| Dhrs9 | 0.32121 | 0.29019 | 9861 | |
| Olfr56 | 0.32124 | -0.91119 | 9862 | |
| Trhr | 0.32125 | -0.34555 | 9863 | |
| Spdya | 0.32131 | -0.018013 | 9864 | |
| Nme5 | 0.32132 | -0.045402 | 9865 | |
| Vmn2r35 | 0.32143 | -0.92884 | 9866 | |
| Col12a1 | 0.3215 | 1.1297 | 9867 | |
| 2310044G17Rik | 0.32154 | -0.82788 | 9868 | |
| Krtap5-3 | 0.32157 | 0.22846 | 9869 | |
| Vmn2r80 | 0.32167 | 1.6625 | 9870 | |
| Ttll2 | 0.32178 | -0.15637 | 9871 | |
| Klhl31 | 0.32188 | -0.040791 | 9872 | |
| Ndufa1 | 0.32188 | -0.90391 | 9873 | |
| Fnbp1 | 0.32191 | -0.90389 | 9874 | |
| Izumo1 | 0.32199 | -0.59769 | 9875 | |
| Efcc1 | 0.32201 | -0.894 | 9876 | |
| Creb3l1 | 0.32202 | -0.70647 | 9877 | |
| Lars | 0.32206 | -0.91101 | 9878 | |
| Asb11 | 0.32207 | 0.49373 | 9879 | |
| Nek8 | 0.32211 | -0.91025 | 9880 | |
| St6galnac5 | 0.32211 | -0.91025 | 9881 | |
| Vtn | 0.32221 | -0.51145 | 9882 | |
| Zfp637 | 0.32223 | -0.9059 | 9883 | |
| Gpr33 | 0.32225 | -0.34268 | 9884 | |
| Tmem190 | 0.32227 | 0.21562 | 9885 | |
| Plekhn1 | 0.32231 | -0.81472 | 9886 | |
| Rhox2g | 0.32235 | -0.90989 | 9887 | |
| Tank | 0.32235 | -0.90989 | 9888 | |
| Med9 | 0.3224 | -0.75458 | 9889 | |
| Dmrtc1b | 0.32244 | -0.1488 | 9890 | |
| Tpst2 | 0.32251 | -0.63956 | 9891 | |
| mmu-mir-3089 | 0.32259 | -0.90913 | 9892 | |
| Pars2 | 0.32261 | -0.58355 | 9893 | |
| Cd1d1 | 0.32262 | -0.36301 | 9894 | |
| Olfr901 | 0.32265 | -0.84004 | 9895 | |
| Asb10 | 0.32269 | 0.24257 | 9896 | |
| BC107364 | 0.32276 | 0.21265 | 9897 | |
| Ccdc181 | 0.32283 | -0.89967 | 9898 | |
| 2010111I01Rik | 0.32285 | -0.78409 | 9899 | |
| Fga | 0.32287 | -0.89641 | 9900 | |
| Fam76a | 0.3229 | 0.057126 | 9901 | |
| Hint2 | 0.32296 | -0.90292 | 9902 | |
| Gm1587 | 0.32298 | -0.90068 | 9903 | |
| Phc2 | 0.32306 | -0.79709 | 9904 | |
| Celf4 | 0.32308 | -0.31159 | 9905 | |
| Tbc1d14 | 0.32309 | -0.47529 | 9906 | |
| Ubap2l | 0.32313 | 0.32901 | 9907 | |
| Zfp850 | 0.32315 | -0.82806 | 9908 | |
| Vgf | 0.32318 | -0.55333 | 9909 | |
| Zfp120 | 0.32326 | -0.73257 | 9910 | |
| Canx | 0.32328 | -0.9051 | 9911 | |
| Mbd2 | 0.3233 | -0.065823 | 9912 | |
| Tmed2 | 0.32332 | -0.89468 | 9913 | |
| Rbm4 | 0.32336 | -0.67825 | 9914 | |
| Kif15 | 0.32339 | -0.89869 | 9915 | |
| Exoc8 | 0.32345 | -0.89647 | 9916 | |
| Taar8b | 0.32357 | -0.289 | 9917 | |
| Bloc1s4 | 0.3236 | -0.33063 | 9918 | |
| Pqbp1 | 0.32364 | 0.65661 | 9919 | |
| Commd10 | 0.32372 | -0.8113 | 9920 | |
| Olfr1121 | 0.32377 | -0.90526 | 9921 | |
| 2010107G12Rik | 0.32379 | -0.89714 | 9922 | |
| Tpte | 0.3239 | -0.50925 | 9923 | |
| Fam96b | 0.32405 | -0.89746 | 9924 | |
| Lcmt2 | 0.32407 | -0.89034 | 9925 | |
| Eif4ebp1 | 0.32413 | 0.19185 | 9926 | |
| Zc3h3 | 0.32414 | -0.904 | 9927 | |
| Ick | 0.3243 | 0.76487 | 9928 | |
| Cldn22 | 0.32434 | 0.50112 | 9929 | |
| Gstk1 | 0.32437 | -0.90563 | 9930 | |
| Arl4d | 0.32448 | 0.95399 | 9931 | |
| Ctif | 0.32454 | -0.89018 | 9932 | |
| Kctd16 | 0.32458 | 0.94411 | 9933 | |
| Fam220a | 0.32458 | 1.0169 | 9934 | |
| Rc3h1 | 0.32469 | -0.89738 | 9935 | |
| En1 | 0.32474 | -0.89973 | 9936 | |
| Rsad1 | 0.32479 | -0.30621 | 9937 | |
| Hsbp1 | 0.32486 | -0.53228 | 9938 | |
| Ndufb2 | 0.32489 | -0.89295 | 9939 | |
| Catsper3 | 0.32495 | -0.12726 | 9940 | |
| Vwa8 | 0.32497 | -0.89043 | 9941 | |
| Gorasp2 | 0.32499 | -0.11005 | 9942 | |
| Dgat2 | 0.325 | 0.28556 | 9943 | |
| Zfp110 | 0.32502 | -0.18284 | 9944 | |
| Lrrc38 | 0.32504 | -0.61459 | 9945 | |
| Tbce | 0.32514 | -0.71724 | 9946 | |
| Arhgef1 | 0.32514 | -0.48729 | 9947 | |
| Klra8 | 0.32516 | -1.1242 | 9948 | |
| Fhit | 0.32519 | -0.39603 | 9949 | |
| Alx1 | 0.32525 | -0.86491 | 9950 | |
| Fibin | 0.32528 | 1.2443 | 9951 | |
| 4921501E09Rik | 0.32532 | 0.57387 | 9952 | |
| Wasf3 | 0.32539 | 0.35588 | 9953 | |
| Olfr44 | 0.32546 | 0.19636 | 9954 | |
| Zfml | 0.32547 | -0.90343 | 9955 | |
| mmu-mir-466c-2 | 0.32548 | -0.53669 | 9956 | |
| Ltn1 | 0.32553 | -0.74085 | 9957 | |
| Tyk2 | 0.32554 | -0.743 | 9958 | |
| Cib1 | 0.32557 | -0.89154 | 9959 | |
| Fam124a | 0.3256 | -0.84148 | 9960 | |
| Btnl9 | 0.32563 | -0.12841 | 9961 | |
| Cacna1i | 0.32577 | -0.65 | 9962 | |
| Atp5j | 0.32581 | -0.09654 | 9963 | |
| Rasal3 | 0.32591 | -0.32024 | 9964 | |
| Mlec | 0.32595 | -0.12168 | 9965 | |
| Rab20 | 0.32612 | 0.73149 | 9966 | |
| Rfpl4 | 0.32618 | -0.28999 | 9967 | |
| Rnf186 | 0.32619 | 1.5067 | 9968 | |
| Tcl1b1 | 0.32624 | -0.88904 | 9969 | |
| Mthfd1 | 0.32626 | -0.26314 | 9970 | |
| Dnajc16 | 0.32628 | -0.88122 | 9971 | |
| Acot2 | 0.32633 | 0.52721 | 9972 | |
| Mcph1 | 0.32639 | -0.29026 | 9973 | |
| Pdp1 | 0.32642 | -0.75488 | 9974 | |
| E130308A19Rik | 0.32644 | -0.77972 | 9975 | |
| Vmn1r58 | 0.32646 | -0.89406 | 9976 | |
| D930015E06Rik | 0.32665 | -0.08938 | 9977 | |
| Vmn2r85 | 0.32667 | -0.89931 | 9978 | |
| mmu-mir-15b | 0.32673 | -0.30791 | 9979 | |
| Tmem233 | 0.32675 | -0.15226 | 9980 | |
| Cyr61 | 0.32676 | -0.65009 | 9981 | |
| Arhgap26 | 0.32679 | -0.45698 | 9982 | |
| Msmp | 0.32689 | -0.46697 | 9983 | |
| Prkar2a | 0.32693 | -0.16136 | 9984 | |
| Gng5 | 0.32696 | -0.32982 | 9985 | |
| Rybp | 0.32697 | -0.89583 | 9986 | |
| 9030617O03Rik | 0.32706 | -0.26528 | 9987 | |
| Pom121 | 0.32707 | 0.48456 | 9988 | |
| Efcab5 | 0.3271 | -0.88333 | 9989 | |
| Adprm | 0.32714 | -0.19853 | 9990 | |
| Ifi202b | 0.32714 | -0.82491 | 9991 | |
| Eif2s3y | 0.32721 | 0.15066 | 9992 | |
| Nps | 0.32728 | 0.14876 | 9993 | |
| Skint5 | 0.32731 | -0.52667 | 9994 | |
| Sp7 | 0.32734 | -0.88196 | 9995 | |
| 4930407I10Rik | 0.32735 | 0.066586 | 9996 | |
| Npc1l1 | 0.32738 | -0.84835 | 9997 | |
| Gabra5 | 0.32738 | -0.57941 | 9998 | |
| Kbtbd12 | 0.3274 | -0.8786 | 9999 | |
| Flnc | 0.32742 | -0.88696 | 10000 | |
| Agap1 | 0.32749 | -0.88009 | 10001 | |
| Vmn2r20 | 0.32756 | -0.12549 | 10002 | |
| Casp6 | 0.3276 | -0.87557 | 10003 | |
| 2810428I15Rik | 0.32763 | -0.47154 | 10004 | |
| Wfikkn2 | 0.3277 | -0.88525 | 10005 | |
| Slamf9 | 0.32778 | -0.033766 | 10006 | |
| Camsap1 | 0.3278 | -0.89647 | 10007 | |
| Gm11554 | 0.3279 | -0.8633 | 10008 | |
| Nlrp4g | 0.32791 | -0.0006934 | 10009 | |
| Olfr732 | 0.32792 | -0.88145 | 10010 | |
| Amz1 | 0.32798 | -0.87344 | 10011 | |
| Lonrf3 | 0.32805 | 0.16889 | 10012 | |
| Vmn1r77 | 0.32805 | -0.80688 | 10013 | |
| Ranbp6 | 0.32812 | -0.3086 | 10014 | |
| Nucb2 | 0.32813 | -0.25946 | 10015 | |
| Letmd1 | 0.3282 | -0.7287 | 10016 | |
| Olfr1504 | 0.32822 | -0.46539 | 10017 | |
| Ankrd34a | 0.32829 | 0.45792 | 10018 | |
| Zscan10 | 0.32849 | -0.89164 | 10019 | |
| Ubl4b | 0.3285 | 1.0142 | 10020 | |
| Ddi2 | 0.3286 | 0.57781 | 10021 | |
| Olfr1275 | 0.32864 | -0.83761 | 10022 | |
| Hadh | 0.32866 | -0.88175 | 10023 | |
| U2surp | 0.32871 | -0.44074 | 10024 | |
| Fam187a | 0.32874 | -0.88529 | 10025 | |
| Brd1 | 0.32884 | -0.87958 | 10026 | |
| Srgn | 0.32888 | -0.53125 | 10027 | |
| Cyth1 | 0.32902 | -0.053134 | 10028 | |
| Zdhhc21 | 0.32913 | 0.16264 | 10029 | |
| Rbfox1 | 0.3293 | 0.75545 | 10030 | |
| Ndufb3 | 0.32934 | -0.88844 | 10031 | |
| Aptx | 0.32948 | 0.090919 | 10032 | |
| Fam65a | 0.32953 | -0.88917 | 10033 | |
| Mmp10 | 0.32955 | -0.77745 | 10034 | |
| Nup54 | 0.32962 | -0.8342 | 10035 | |
| Gpr45 | 0.32965 | -0.89252 | 10036 | |
| Zfp963 | 0.32972 | 0.48058 | 10037 | |
| Olfr1061 | 0.32976 | 0.54946 | 10038 | |
| 0610037L13Rik | 0.32979 | -0.68168 | 10039 | |
| Ankrd2 | 0.32983 | -0.73501 | 10040 | |
| Mroh2b | 0.3299 | -0.49461 | 10041 | |
| Cldn3 | 0.32996 | -0.43988 | 10042 | |
| BC030867 | 0.32996 | -0.030696 | 10043 | |
| Rabl3 | 0.32998 | -0.16166 | 10044 | |
| Slco1a1 | 0.33 | 0.0019963 | 10045 | |
| mmu-mir-17 | 0.33005 | -0.4623 | 10046 | |
| Ogdhl | 0.33007 | -0.5306 | 10047 | |
| C87414 | 0.33012 | 0.53341 | 10048 | |
| Irx1 | 0.33017 | -0.60762 | 10049 | |
| Clec16a | 0.3302 | -0.88327 | 10050 | |
| Rbms2 | 0.33024 | -0.88131 | 10051 | |
| Pdzd4 | 0.33026 | -0.87868 | 10052 | |
| Gm14685 | 0.33028 | -0.73689 | 10053 | |
| Sbk2 | 0.33031 | 0.97656 | 10054 | |
| Fan1 | 0.33035 | -0.89056 | 10055 | |
| Nkx1-2 | 0.33039 | -0.87729 | 10056 | |
| Serpind1 | 0.33049 | 0.14467 | 10057 | |
| Klhl42 | 0.3305 | -0.73252 | 10058 | |
| Auts2 | 0.33056 | -0.82182 | 10059 | |
| Abcd3 | 0.33066 | -0.45545 | 10060 | |
| Mefv | 0.3307 | -0.87803 | 10061 | |
| Tfap2e | 0.33072 | -0.88373 | 10062 | |
| Olfr677 | 0.33077 | 1.0744 | 10063 | |
| 2700094K13Rik | 0.3308 | -0.0099926 | 10064 | |
| Grik4 | 0.33083 | -0.87536 | 10065 | |
| 4930550C14Rik | 0.33085 | -0.63541 | 10066 | |
| Mro | 0.33092 | -0.8844 | 10067 | |
| 4833403I15Rik | 0.33092 | -0.8844 | 10068 | |
| Pus7l | 0.33094 | 0.20933 | 10069 | |
| Pramef12 | 0.33095 | -0.87379 | 10070 | |
| Exoc6 | 0.33099 | -0.88128 | 10071 | |
| Bbip1 | 0.33101 | -0.24441 | 10072 | |
| Nsmce4a | 0.33106 | -0.67713 | 10073 | |
| Paxip1 | 0.33115 | -0.78231 | 10074 | |
| Pde4dip | 0.33117 | -0.87779 | 10075 | |
| Slc5a11 | 0.33122 | -0.24663 | 10076 | |
| Tulp4 | 0.33125 | 0.26439 | 10077 | |
| Prpf8 | 0.33131 | -0.88391 | 10078 | |
| Unc13a | 0.33141 | -0.01598 | 10079 | |
| Myoz1 | 0.33141 | 0.095173 | 10080 | |
| mmu-mir-8110 | 0.33141 | -0.070638 | 10081 | |
| Atp1b1 | 0.33148 | -0.58406 | 10082 | |
| Brsk1 | 0.33148 | -0.88304 | 10083 | |
| Ktn1 | 0.3315 | 1.6445 | 10084 | |
| mmu-mir-3071 | 0.33153 | -0.65138 | 10085 | |
| Olfr1418 | 0.3316 | 0.14582 | 10086 | |
| mmu-mir-465b-1 | 0.33164 | -1.1042 | 10087 | |
| Prrg2 | 0.33167 | -0.52033 | 10088 | |
| Tex40 | 0.33169 | -0.88448 | 10089 | |
| Ccr2 | 0.33171 | -0.028302 | 10090 | |
| mmu-mir-5124b | 0.33172 | -1.1048 | 10091 | |
| Ctxn1 | 0.33175 | -0.85822 | 10092 | |
| Olfr874 | 0.33178 | 0.15447 | 10093 | |
| Stfa1 | 0.33182 | -0.87596 | 10094 | |
| Higd2a | 0.33186 | -0.20273 | 10095 | |
| Prss38 | 0.33193 | -0.88002 | 10096 | |
| Lhfpl5 | 0.33195 | -0.87122 | 10097 | |
| Ctnnd1 | 0.33195 | -0.32938 | 10098 | |
| Nr2c2 | 0.33198 | -0.15571 | 10099 | |
| Lims2 | 0.332 | -0.87266 | 10100 | |
| Yipf3 | 0.33202 | -0.65981 | 10101 | |
| Fbxw19 | 0.33205 | -0.40428 | 10102 | |
| Actn4 | 0.3321 | -0.18652 | 10103 | |
| Ndufaf1 | 0.33214 | -0.87343 | 10104 | |
| Rcvrn | 0.33216 | -0.2662 | 10105 | |
| Arhgef10l | 0.33218 | -0.58784 | 10106 | |
| Elmo3 | 0.33223 | -0.88481 | 10107 | |
| Txnrd3 | 0.33226 | -0.2005 | 10108 | |
| Rhof | 0.33235 | -0.87346 | 10109 | |
| Nrn1 | 0.33235 | 0.1258 | 10110 | |
| Asrgl1 | 0.3324 | -0.44524 | 10111 | |
| Slc26a9 | 0.33244 | -0.026266 | 10112 | |
| Raver1 | 0.33245 | -0.69987 | 10113 | |
| Ddx43 | 0.33252 | 0.38652 | 10114 | |
| Cep170b | 0.33257 | -0.87759 | 10115 | |
| Ift57 | 0.33262 | -0.46206 | 10116 | |
| Gpr12 | 0.33266 | -0.87786 | 10117 | |
| Epn1 | 0.3327 | -0.86932 | 10118 | |
| Prelp | 0.33289 | 1.7131 | 10119 | |
| Son | 0.33293 | -0.46596 | 10120 | |
| Flnb | 0.33296 | -0.88282 | 10121 | |
| Plscr3 | 0.333 | -0.87596 | 10122 | |
| Gpr50 | 0.33303 | 0.34106 | 10123 | |
| Gnb1 | 0.33311 | -0.46119 | 10124 | |
| 9030624J02Rik | 0.33313 | -0.0063759 | 10125 | |
| Socs1 | 0.33313 | -0.46119 | 10126 | |
| Olfr20 | 0.33317 | -0.23418 | 10127 | |
| 4933425L06Rik | 0.3332 | -0.84147 | 10128 | |
| Ccdc62 | 0.33334 | 0.022523 | 10129 | |
| Spata9 | 0.33337 | 0.1633 | 10130 | |
| Knop1 | 0.33341 | -0.86977 | 10131 | |
| Wbp4 | 0.33344 | -0.22011 | 10132 | |
| Aimp1 | 0.33348 | -0.86212 | 10133 | |
| Ankrd6 | 0.33351 | -0.62443 | 10134 | |
| Ces2f | 0.33351 | 0.11121 | 10135 | |
| Etnk2 | 0.33354 | -0.87206 | 10136 | |
| Ttc30a1 | 0.33358 | -0.042655 | 10137 | |
| mmu-mir-7080 | 0.3336 | 0.25176 | 10138 | |
| Serac1 | 0.33365 | 1.8141 | 10139 | |
| Mesdc2 | 0.33374 | -0.87587 | 10140 | |
| Defb18 | 0.33377 | -0.77305 | 10141 | |
| Pde10a | 0.33379 | -0.32568 | 10142 | |
| Olfr638 | 0.33383 | 0.13471 | 10143 | |
| Hist1h3d | 0.334 | 0.94007 | 10144 | |
| Pde9a | 0.3341 | 0.9314 | 10145 | |
| Ezr | 0.33421 | -0.73879 | 10146 | |
| Tctex1d2 | 0.33424 | -0.86976 | 10147 | |
| Upb1 | 0.33424 | 0.43711 | 10148 | |
| Cmtm5 | 0.33431 | 1.0323 | 10149 | |
| Cacfd1 | 0.33432 | -0.8685 | 10150 | |
| Btg4 | 0.33437 | -0.86504 | 10151 | |
| Rpe65 | 0.33438 | 0.2716 | 10152 | |
| Cd209b | 0.33441 | -0.38017 | 10153 | |
| Creld1 | 0.33443 | -0.87799 | 10154 | |
| Kcnip3 | 0.33445 | -0.8665 | 10155 | |
| BC048644 | 0.33448 | -0.86988 | 10156 | |
| Gtf2h1 | 0.33452 | -0.87224 | 10157 | |
| Vmn1r119 | 0.33454 | 0.42759 | 10158 | |
| Olfr384 | 0.33462 | 0.38806 | 10159 | |
| Kdelr1 | 0.33475 | -0.86524 | 10160 | |
| Zwint | 0.33485 | -0.8643 | 10161 | |
| Mtbp | 0.33492 | -0.87772 | 10162 | |
| Psmd2 | 0.33492 | -0.87772 | 10163 | |
| Snap91 | 0.33495 | -0.85936 | 10164 | |
| Nid2 | 0.33499 | -0.86474 | 10165 | |
| Tmem256 | 0.3351 | -0.60757 | 10166 | |
| Wnt11 | 0.33512 | -0.87722 | 10167 | |
| Calm2 | 0.33514 | -0.86784 | 10168 | |
| Tnfsf4 | 0.33514 | -0.86691 | 10169 | |
| Mfap2 | 0.33516 | -0.86603 | 10170 | |
| Frmpd1 | 0.33521 | -0.8709 | 10171 | |
| Prcc | 0.33525 | -0.3762 | 10172 | |
| Ckmt1 | 0.33529 | -0.86087 | 10173 | |
| Mapk6 | 0.33532 | -0.86423 | 10174 | |
| Aldh1l2 | 0.33534 | -0.87417 | 10175 | |
| mmu-mir-582 | 0.33541 | -0.081535 | 10176 | |
| Gm12 | 0.33545 | -0.86065 | 10177 | |
| Abcb8 | 0.33549 | -0.50225 | 10178 | |
| Vmn1r204 | 0.33553 | -0.634 | 10179 | |
| Myeov2 | 0.33555 | -0.85768 | 10180 | |
| Gm5483 | 0.33562 | -0.87538 | 10181 | |
| F11r | 0.33564 | -0.56006 | 10182 | |
| Kel | 0.33566 | 0.3154 | 10183 | |
| Immp2l | 0.33571 | -0.8593 | 10184 | |
| Olfr926 | 0.3358 | -0.8636 | 10185 | |
| B4galt4 | 0.33587 | 0.078349 | 10186 | |
| Arr3 | 0.33599 | -0.089969 | 10187 | |
| Fhl4 | 0.33599 | -0.81801 | 10188 | |
| Zfp160 | 0.33603 | -0.68315 | 10189 | |
| Chst12 | 0.33605 | -0.86176 | 10190 | |
| Tmem43 | 0.33611 | -0.57351 | 10191 | |
| Nudt3 | 0.33612 | -0.85682 | 10192 | |
| Tspyl4 | 0.33615 | 0.39191 | 10193 | |
| Otud1 | 0.33629 | -0.85422 | 10194 | |
| Guca2b | 0.33633 | -0.85707 | 10195 | |
| Pde11a | 0.33634 | -0.19328 | 10196 | |
| Tmem132a | 0.33635 | -0.40629 | 10197 | |
| Olfr203 | 0.3364 | -0.86511 | 10198 | |
| Mfsd12 | 0.33648 | -0.86573 | 10199 | |
| Gcfc2 | 0.33655 | -0.16283 | 10200 | |
| Olfr130 | 0.33657 | -0.41167 | 10201 | |
| Rgsl1 | 0.3366 | 0.30967 | 10202 | |
| Uchl3 | 0.33663 | -0.40055 | 10203 | |
| Mcpt9 | 0.33668 | -0.60427 | 10204 | |
| Ifi27 | 0.3367 | -0.11533 | 10205 | |
| Zfp946 | 0.33673 | -0.87062 | 10206 | |
| mmu-mir-380 | 0.33677 | -0.44789 | 10207 | |
| mmu-mir-1190 | 0.33681 | -0.35524 | 10208 | |
| 3300002I08Rik | 0.33683 | -0.85703 | 10209 | |
| Tubgcp3 | 0.33684 | -0.60128 | 10210 | |
| Tmem126a | 0.33687 | -0.86929 | 10211 | |
| Dcun1d2 | 0.3369 | -0.86572 | 10212 | |
| Prkca | 0.33695 | 0.22958 | 10213 | |
| Hcn4 | 0.33708 | 0.45274 | 10214 | |
| 5730508B09Rik | 0.33711 | -0.27871 | 10215 | |
| Card11 | 0.33713 | -0.85462 | 10216 | |
| Pde3a | 0.33715 | -0.0823 | 10217 | |
| Mlxipl | 0.33718 | -0.86273 | 10218 | |
| Txn2 | 0.33726 | 1.7744 | 10219 | |
| Dnajc24 | 0.33729 | -0.83731 | 10220 | |
| Zkscan14 | 0.33733 | -0.096512 | 10221 | |
| Hemt1 | 0.3374 | -0.8674 | 10222 | |
| Iqcb1 | 0.33743 | 0.006241 | 10223 | |
| Trim10 | 0.33746 | 0.18388 | 10224 | |
| Zfp541 | 0.33757 | -0.85595 | 10225 | |
| Vmn1r66 | 0.33757 | 1.0156 | 10226 | |
| Zfp648 | 0.33763 | -0.85987 | 10227 | |
| Olfr1454 | 0.33767 | -0.86967 | 10228 | |
| Usp17le | 0.3377 | -0.85755 | 10229 | |
| Dnmbp | 0.33774 | 0.34853 | 10230 | |
| Slc25a48 | 0.33776 | -0.75478 | 10231 | |
| Atp8a2 | 0.33778 | -0.76419 | 10232 | |
| Ank2 | 0.3378 | -0.86146 | 10233 | |
| Fam175a | 0.33781 | -0.74741 | 10234 | |
| C130074G19Rik | 0.33785 | -0.86032 | 10235 | |
| Ramp3 | 0.33792 | -0.85636 | 10236 | |
| Cyp4f18 | 0.33792 | -0.85636 | 10237 | |
| Pura | 0.33796 | -0.85508 | 10238 | |
| Smarcd1 | 0.33802 | -0.61718 | 10239 | |
| Rbms3 | 0.33805 | 0.47557 | 10240 | |
| Zc3hav1l | 0.33816 | -0.7703 | 10241 | |
| Abl1 | 0.33828 | -0.67128 | 10242 | |
| Dlgap1 | 0.33829 | 2.4113 | 10243 | |
| Ak5 | 0.33838 | -0.86534 | 10244 | |
| Yap1 | 0.3384 | -0.80092 | 10245 | |
| Ms4a5 | 0.33856 | -0.86448 | 10246 | |
| Rnf11 | 0.33858 | -0.85936 | 10247 | |
| Tmc5 | 0.33861 | -0.70794 | 10248 | |
| Tmc7 | 0.33865 | -0.85601 | 10249 | |
| Arf5 | 0.33869 | -0.39929 | 10250 | |
| Lyrm1 | 0.33871 | -0.72681 | 10251 | |
| Olfr1218 | 0.33874 | 0.58576 | 10252 | |
| Sox14 | 0.33878 | 0.3898 | 10253 | |
| Tspan17 | 0.33878 | -0.85852 | 10254 | |
| BC055324 | 0.33881 | -0.59971 | 10255 | |
| Fam19a4 | 0.33887 | -0.86131 | 10256 | |
| Dnajb12 | 0.33893 | -0.84992 | 10257 | |
| mmu-mir-6904 | 0.33896 | -0.16329 | 10258 | |
| Tmem215 | 0.33899 | -0.42299 | 10259 | |
| Zc3h15 | 0.33905 | -0.087907 | 10260 | |
| Ccdc142 | 0.33908 | -0.33337 | 10261 | |
| Olfr223 | 0.33909 | -0.27409 | 10262 | |
| Trank1 | 0.33911 | -0.85201 | 10263 | |
| Lst1 | 0.3392 | -0.86652 | 10264 | |
| Olfr132 | 0.33926 | 1.0529 | 10265 | |
| Casq1 | 0.33933 | -0.57487 | 10266 | |
| Ube2k | 0.33939 | -0.85697 | 10267 | |
| Gm6696 | 0.33942 | -1.0705 | 10268 | |
| Vstm4 | 0.33943 | -0.62397 | 10269 | |
| Nat6 | 0.33947 | 0.022545 | 10270 | |
| Pik3r5 | 0.33954 | -0.83645 | 10271 | |
| Gm12250 | 0.33963 | -0.8078 | 10272 | |
| Dear1 | 0.33968 | -0.85424 | 10273 | |
| Wif1 | 0.33968 | -0.85424 | 10274 | |
| Tmem207 | 0.33978 | -0.8509 | 10275 | |
| Camta1 | 0.33988 | -0.81335 | 10276 | |
| Dek | 0.33989 | -0.28182 | 10277 | |
| Dpm2 | 0.33994 | -0.85427 | 10278 | |
| Gm6040 | 0.34002 | -0.46007 | 10279 | |
| Tmem145 | 0.34006 | -0.85913 | 10280 | |
| 4930548H24Rik | 0.3401 | -0.86329 | 10281 | |
| Baalc | 0.34015 | -0.86413 | 10282 | |
| Dnajc1 | 0.34019 | -0.66211 | 10283 | |
| C1qtnf6 | 0.34019 | 1.3213 | 10284 | |
| Sncb | 0.34021 | -0.85222 | 10285 | |
| Sh3pxd2b | 0.34023 | 0.032905 | 10286 | |
| Tnfaip8 | 0.34036 | -0.84444 | 10287 | |
| Olfr103 | 0.34046 | -0.84877 | 10288 | |
| Snx29 | 0.34049 | -0.40503 | 10289 | |
| Olfr65 | 0.34049 | 0.052411 | 10290 | |
| Nhlrc1 | 0.34054 | -0.019473 | 10291 | |
| mmu-mir-290a | 0.34061 | 0.40025 | 10292 | |
| Tdrkh | 0.34066 | 0.20493 | 10293 | |
| Rgmb | 0.34071 | 0.60221 | 10294 | |
| mmu-mir-7036 | 0.34077 | 0.075456 | 10295 | |
| Pnma3 | 0.34078 | -0.47742 | 10296 | |
| Stab2 | 0.34081 | 1.7307 | 10297 | |
| Bnip3 | 0.34084 | -0.82536 | 10298 | |
| Slc24a1 | 0.34088 | -0.51099 | 10299 | |
| Mill2 | 0.34091 | -0.85158 | 10300 | |
| 2310047M10Rik | 0.34093 | -0.85376 | 10301 | |
| Mum1l1 | 0.34095 | -0.5765 | 10302 | |
| Nudt7 | 0.34097 | -0.86007 | 10303 | |
| Rnf185 | 0.34099 | 0.077877 | 10304 | |
| Pla2g15 | 0.34104 | -0.15046 | 10305 | |
| Fam96a | 0.34109 | -0.77535 | 10306 | |
| 4930451G09Rik | 0.34114 | -0.56286 | 10307 | |
| Fgf1 | 0.34114 | -0.45798 | 10308 | |
| Hist1h3f | 0.34119 | -0.49174 | 10309 | |
| Cyp4f13 | 0.34125 | -0.3866 | 10310 | |
| Mrpl2 | 0.34133 | -0.67718 | 10311 | |
| Muc1 | 0.34136 | -0.8525 | 10312 | |
| Ano4 | 0.3414 | -0.83966 | 10313 | |
| Rab12 | 0.34143 | -0.86025 | 10314 | |
| Ism1 | 0.34147 | -0.096532 | 10315 | |
| Ccl27a | 0.34154 | 1.5945 | 10316 | |
| Pde2a | 0.34156 | -0.85339 | 10317 | |
| Pkd1l3 | 0.3416 | -0.55822 | 10318 | |
| Wdr26 | 0.34168 | -0.78359 | 10319 | |
| Olfr951 | 0.34171 | 0.18094 | 10320 | |
| Glt8d2 | 0.34178 | -0.22525 | 10321 | |
| Sertad2 | 0.34182 | -0.72033 | 10322 | |
| Sntb2 | 0.34186 | -0.85455 | 10323 | |
| Epha5 | 0.34188 | -0.49001 | 10324 | |
| Smr3a | 0.34192 | 0.10422 | 10325 | |
| Fdxr | 0.34193 | -0.74542 | 10326 | |
| Slc30a6 | 0.34195 | -0.22754 | 10327 | |
| D8Ertd738e | 0.34198 | -0.84999 | 10328 | |
| Tet3 | 0.34198 | -0.84999 | 10329 | |
| Flt1 | 0.3421 | -0.8375 | 10330 | |
| Vil1 | 0.34216 | -0.78259 | 10331 | |
| Mbtd1 | 0.34223 | 1.4989 | 10332 | |
| Ms4a6b | 0.34227 | -0.84808 | 10333 | |
| Lgals8 | 0.34229 | -0.85461 | 10334 | |
| Esyt3 | 0.3423 | 1.143 | 10335 | |
| Klre1 | 0.34236 | -0.85602 | 10336 | |
| Rab39 | 0.34238 | -0.69405 | 10337 | |
| Slc27a1 | 0.34243 | -0.74837 | 10338 | |
| Tmem194b | 0.34245 | -0.84023 | 10339 | |
| Chp2 | 0.34247 | -0.84051 | 10340 | |
| D3Ertd751e | 0.34249 | -0.50847 | 10341 | |
| Usp22 | 0.34257 | 0.60634 | 10342 | |
| Adh6a | 0.3426 | -0.83729 | 10343 | |
| Olfr1238 | 0.34266 | -0.85729 | 10344 | |
| Zbtb18 | 0.34271 | -0.4031 | 10345 | |
| Ces1b | 0.34274 | -0.8179 | 10346 | |
| Foxr2 | 0.34274 | -0.79918 | 10347 | |
| mmu-mir-3473e | 0.34277 | -0.7419 | 10348 | |
| Xkr5 | 0.34282 | -0.84001 | 10349 | |
| Plod2 | 0.34285 | -0.8287 | 10350 | |
| Gpt2 | 0.34288 | -0.29762 | 10351 | |
| Unc93b1 | 0.34292 | -0.43618 | 10352 | |
| Dpyd | 0.34292 | -0.073088 | 10353 | |
| Dusp21 | 0.34298 | 0.889 | 10354 | |
| Ppp1r15b | 0.34306 | -0.18716 | 10355 | |
| Gp9 | 0.3431 | -0.19823 | 10356 | |
| Xpo7 | 0.34317 | -0.69289 | 10357 | |
| Defb4 | 0.34319 | -0.84621 | 10358 | |
| Sec11c | 0.34329 | -0.82441 | 10359 | |
| Endov | 0.34333 | 0.28201 | 10360 | |
| Cdk8 | 0.34352 | -0.85384 | 10361 | |
| Olfr871 | 0.3436 | -0.85395 | 10362 | |
| Rab25 | 0.34364 | 0.10067 | 10363 | |
| Lonrf2 | 0.34374 | 0.71396 | 10364 | |
| Tbxa2r | 0.34377 | -0.84396 | 10365 | |
| Uvssa | 0.34384 | -0.84424 | 10366 | |
| Gm21319 | 0.34395 | -0.49873 | 10367 | |
| Mov10l1 | 0.34398 | 0.48796 | 10368 | |
| Fgf23 | 0.34399 | -0.84085 | 10369 | |
| Sptb | 0.34405 | -0.015103 | 10370 | |
| Prr3 | 0.34408 | -0.17356 | 10371 | |
| Urad | 0.34414 | -0.84343 | 10372 | |
| Fam58b | 0.34419 | -0.74714 | 10373 | |
| Mpp2 | 0.34427 | -0.81969 | 10374 | |
| Tceal7 | 0.34429 | 1.072 | 10375 | |
| Patl2 | 0.34436 | -0.0052041 | 10376 | |
| Manba | 0.34436 | -0.39042 | 10377 | |
| mmu-mir-667 | 0.34438 | -0.59858 | 10378 | |
| Olfr93 | 0.34443 | -0.59806 | 10379 | |
| 1700021K19Rik | 0.3445 | 0.33412 | 10380 | |
| Eny2 | 0.34457 | -0.1511 | 10381 | |
| mmu-mir-5122 | 0.3446 | -0.41878 | 10382 | |
| Atr | 0.34467 | -0.41258 | 10383 | |
| Riiad1 | 0.34478 | -0.84885 | 10384 | |
| Hsd3b7 | 0.34478 | -0.84885 | 10385 | |
| mmu-mir-684-1 | 0.34489 | -0.7901 | 10386 | |
| Ccdc60 | 0.3449 | -0.76269 | 10387 | |
| Rab5a | 0.34493 | -0.84674 | 10388 | |
| Pbrm1 | 0.34497 | -0.84176 | 10389 | |
| Ndor1 | 0.34498 | 0.9453 | 10390 | |
| Aox4 | 0.34505 | 0.46941 | 10391 | |
| Bpi | 0.34506 | -0.84939 | 10392 | |
| Acnat2 | 0.3451 | -0.82818 | 10393 | |
| Olfr15 | 0.34514 | -0.83462 | 10394 | |
| Ralbp1 | 0.34516 | -0.83996 | 10395 | |
| Fam69a | 0.34523 | -0.63633 | 10396 | |
| Gm773 | 0.34527 | -0.84265 | 10397 | |
| Krt17 | 0.3453 | -0.841 | 10398 | |
| Olfr688 | 0.3453 | -0.31871 | 10399 | |
| Gnrhr | 0.34539 | -0.46866 | 10400 | |
| 0610031J06Rik | 0.34547 | -0.58688 | 10401 | |
| Fam69b | 0.34549 | -0.82155 | 10402 | |
| Mvb12b | 0.34551 | -0.0091693 | 10403 | |
| mmu-mir-7053 | 0.34553 | -0.64842 | 10404 | |
| Dok6 | 0.34556 | -0.60231 | 10405 | |
| Npbwr1 | 0.34559 | -0.83936 | 10406 | |
| Cbln1 | 0.3456 | 0.18468 | 10407 | |
| Rdh11 | 0.34563 | -0.29989 | 10408 | |
| Hrk | 0.34566 | -0.81882 | 10409 | |
| Rpl3l | 0.34569 | -0.83574 | 10410 | |
| Trak1 | 0.3458 | -0.68857 | 10411 | |
| 9430020K01Rik | 0.34585 | -0.21335 | 10412 | |
| Olfr968 | 0.3459 | -0.65019 | 10413 | |
| Dhdh | 0.34593 | -0.81978 | 10414 | |
| Fkbp8 | 0.34597 | -0.75436 | 10415 | |
| Nfatc2 | 0.34604 | -0.59191 | 10416 | |
| Cd300lh | 0.34608 | 0.79278 | 10417 | |
| Snapc5 | 0.34611 | 0.14801 | 10418 | |
| mmu-mir-1960 | 0.34612 | -0.11107 | 10419 | |
| Ecm2 | 0.34625 | -0.63287 | 10420 | |
| Hnrnph2 | 0.34627 | -0.58769 | 10421 | |
| Aldh3b2 | 0.34634 | -0.82736 | 10422 | |
| Hand1 | 0.34644 | -0.84019 | 10423 | |
| Olfr552 | 0.34645 | -0.69384 | 10424 | |
| mmu-let-7f-1 | 0.34648 | 0.77879 | 10425 | |
| Gopc | 0.34651 | -0.8318 | 10426 | |
| Dtx2 | 0.34654 | -0.27567 | 10427 | |
| Wfdc6b | 0.34656 | -0.38472 | 10428 | |
| 4933433C11Rik | 0.34657 | 0.091487 | 10429 | |
| Krt36 | 0.34657 | -0.42889 | 10430 | |
| Polg2 | 0.34664 | -0.83649 | 10431 | |
| Ucp1 | 0.34673 | -0.5067 | 10432 | |
| Cpeb3 | 0.34673 | -0.83555 | 10433 | |
| Pdilt | 0.34683 | -0.55317 | 10434 | |
| Serinc3 | 0.34688 | -0.26503 | 10435 | |
| Suv39h1 | 0.3469 | 1.5816 | 10436 | |
| Sostdc1 | 0.34693 | 0.21176 | 10437 | |
| Krtap19-4 | 0.347 | -0.1018 | 10438 | |
| Isyna1 | 0.34703 | 0.35981 | 10439 | |
| Sec14l3 | 0.3471 | -0.68915 | 10440 | |
| Klhdc4 | 0.34712 | -0.22955 | 10441 | |
| Lcat | 0.34712 | -0.6091 | 10442 | |
| Ppara | 0.34731 | 0.27712 | 10443 | |
| Olfr23 | 0.34731 | -0.47236 | 10444 | |
| Zfp94 | 0.34731 | -0.83745 | 10445 | |
| Wdpcp | 0.34748 | 0.92322 | 10446 | |
| Aff4 | 0.34751 | -0.47488 | 10447 | |
| Pif1 | 0.34758 | -0.31406 | 10448 | |
| Ctsg | 0.34765 | -0.84299 | 10449 | |
| Itpka | 0.34765 | 1.0402 | 10450 | |
| Gpbp1l1 | 0.34776 | -0.19997 | 10451 | |
| Xkr6 | 0.34778 | -0.8288 | 10452 | |
| Olfr466 | 0.34789 | -0.8267 | 10453 | |
| Ttc3 | 0.34789 | -0.36189 | 10454 | |
| Scgb2b15 | 0.34793 | -0.5707 | 10455 | |
| Uox | 0.34793 | -0.8301 | 10456 | |
| Apobec4 | 0.34795 | -0.81759 | 10457 | |
| Amotl1 | 0.34797 | -0.81469 | 10458 | |
| Tbk1 | 0.34799 | 0.84845 | 10459 | |
| Dlec1 | 0.348 | -0.82807 | 10460 | |
| Pi4k2a | 0.34803 | -0.81495 | 10461 | |
| mmu-mir-6370 | 0.34807 | -0.05407 | 10462 | |
| Hspb7 | 0.3481 | -0.44878 | 10463 | |
| Rpl39l | 0.3481 | -0.66507 | 10464 | |
| Cmtm2a | 0.34816 | 0.63691 | 10465 | |
| I830077J02Rik | 0.34819 | -0.64753 | 10466 | |
| Saal1 | 0.34821 | -0.82238 | 10467 | |
| Hpd | 0.34827 | 0.5785 | 10468 | |
| Tbc1d5 | 0.3483 | -0.52223 | 10469 | |
| A930018P22Rik | 0.3483 | -0.16837 | 10470 | |
| Try5 | 0.34834 | -0.20711 | 10471 | |
| Vps8 | 0.3484 | 0.12352 | 10472 | |
| Rpia | 0.34849 | -0.82787 | 10473 | |
| Nr1i3 | 0.34849 | -0.82787 | 10474 | |
| Tph1 | 0.34854 | -0.65679 | 10475 | |
| Zfp128 | 0.34865 | -0.82157 | 10476 | |
| Best3 | 0.34878 | -0.35503 | 10477 | |
| mmu-mir-297b | 0.34881 | 0.24258 | 10478 | |
| Abcb7 | 0.34882 | -0.79755 | 10479 | |
| Dennd3 | 0.34888 | 0.35909 | 10480 | |
| Gm4297 | 0.34891 | -0.74083 | 10481 | |
| Xpo4 | 0.34892 | 0.56509 | 10482 | |
| Clock | 0.34899 | 0.7296 | 10483 | |
| Fignl2 | 0.34902 | -0.039651 | 10484 | |
| Gstt3 | 0.34905 | 1.8108 | 10485 | |
| Slc38a1 | 0.34909 | -0.70307 | 10486 | |
| Thtpa | 0.34909 | -0.10258 | 10487 | |
| Barx1 | 0.34935 | -0.82491 | 10488 | |
| Rpl15 | 0.34948 | -0.56198 | 10489 | |
| Vcpip1 | 0.34951 | -0.83375 | 10490 | |
| Ldb2 | 0.34957 | -0.82553 | 10491 | |
| Gna13 | 0.34959 | -0.65437 | 10492 | |
| Gsdmd | 0.34967 | 0.47724 | 10493 | |
| Ndufa10 | 0.34974 | -0.82199 | 10494 | |
| Arl11 | 0.34976 | -0.82417 | 10495 | |
| Pcdh7 | 0.34985 | -0.82197 | 10496 | |
| Ppp3r1 | 0.34986 | 0.16503 | 10497 | |
| Tcf21 | 0.34991 | -0.3155 | 10498 | |
| Ift172 | 0.34993 | -0.8308 | 10499 | |
| mmu-mir-5132 | 0.34995 | -0.27399 | 10500 | |
| Olfr1184 | 0.34996 | -0.78231 | 10501 | |
| Olfr358 | 0.35 | -0.82318 | 10502 | |
| Oma1 | 0.35001 | 0.23333 | 10503 | |
| Fam169b | 0.35008 | -0.48594 | 10504 | |
| Tceal5 | 0.35013 | -0.82909 | 10505 | |
| Spata1 | 0.35022 | -0.34524 | 10506 | |
| Tnp2 | 0.35032 | 0.25018 | 10507 | |
| Sik2 | 0.35035 | -0.33075 | 10508 | |
| Trpm6 | 0.35049 | -0.83021 | 10509 | |
| Tra2a | 0.35052 | -0.60457 | 10510 | |
| Rsf1 | 0.35062 | -0.83168 | 10511 | |
| Trabd2b | 0.35072 | -0.56679 | 10512 | |
| Ahsa1 | 0.35086 | -0.15707 | 10513 | |
| Sdr42e1 | 0.35088 | -0.82017 | 10514 | |
| Tmem100 | 0.3509 | -0.8166 | 10515 | |
| Atcay | 0.35103 | -0.82341 | 10516 | |
| 4930468A15Rik | 0.35109 | -0.82261 | 10517 | |
| Cnppd1 | 0.35112 | -0.57498 | 10518 | |
| Upp2 | 0.35121 | 0.11698 | 10519 | |
| Klhl30 | 0.35122 | -0.10937 | 10520 | |
| Smco1 | 0.35124 | -0.22335 | 10521 | |
| Olfr1309 | 0.35125 | -0.032852 | 10522 | |
| Slc31a2 | 0.35131 | -0.7915 | 10523 | |
| Npy6r | 0.35133 | -0.82622 | 10524 | |
| Anapc2 | 0.35138 | -0.82961 | 10525 | |
| Gpr87 | 0.35155 | -0.34856 | 10526 | |
| Kctd8 | 0.35158 | -0.18005 | 10527 | |
| Tbc1d9 | 0.3516 | -0.83212 | 10528 | |
| Ap1s1 | 0.35161 | -0.20062 | 10529 | |
| Cxcr2 | 0.35171 | -0.12513 | 10530 | |
| Coro7 | 0.35172 | 0.0045379 | 10531 | |
| Slc36a3 | 0.35192 | -0.82141 | 10532 | |
| Cspg4 | 0.35202 | 0.068318 | 10533 | |
| Lyst | 0.35206 | -0.76361 | 10534 | |
| Gpx2 | 0.35213 | -0.36939 | 10535 | |
| Bdh2 | 0.35223 | -0.60395 | 10536 | |
| Tmem26 | 0.3523 | 0.27179 | 10537 | |
| Agtpbp1 | 0.35235 | 0.87627 | 10538 | |
| Tmem38b | 0.35235 | 0.34348 | 10539 | |
| Cts6 | 0.35238 | -0.82574 | 10540 | |
| Ido2 | 0.3524 | -0.5761 | 10541 | |
| Mup3 | 0.35249 | -0.76636 | 10542 | |
| Hprt | 0.35254 | -0.56419 | 10543 | |
| Acadsb | 0.35256 | -0.3531 | 10544 | |
| Gm20815 | 0.35257 | -0.96724 | 10545 | |
| Ccnb1ip1 | 0.35267 | -0.82149 | 10546 | |
| Gm1045 | 0.35269 | -0.81728 | 10547 | |
| mmu-mir-7239 | 0.35277 | -0.2309 | 10548 | |
| Dip2b | 0.35281 | -0.82546 | 10549 | |
| Itgb3bp | 0.35291 | -0.82406 | 10550 | |
| Atxn1 | 0.35291 | 0.64342 | 10551 | |
| Sufu | 0.35293 | -0.17098 | 10552 | |
| Gabrq | 0.35305 | 0.74569 | 10553 | |
| mmu-mir-3109 | 0.35308 | -0.61395 | 10554 | |
| D17H6S53E | 0.35317 | 0.261 | 10555 | |
| Banf2 | 0.35319 | -0.80482 | 10556 | |
| Ildr2 | 0.35326 | -0.3016 | 10557 | |
| Fgd2 | 0.35334 | -0.82034 | 10558 | |
| Tsacc | 0.35337 | -0.81638 | 10559 | |
| Prmt2 | 0.35342 | -0.46083 | 10560 | |
| Thada | 0.35342 | -0.82068 | 10561 | |
| Fastk | 0.35345 | -0.54706 | 10562 | |
| Fem1a | 0.35345 | 0.45878 | 10563 | |
| Lnpep | 0.35349 | 0.60211 | 10564 | |
| Vwa5a | 0.3535 | -0.81556 | 10565 | |
| Dusp22 | 0.35352 | -0.36469 | 10566 | |
| Ipmk | 0.35368 | -0.17508 | 10567 | |
| Gng2 | 0.35372 | -0.71023 | 10568 | |
| Hist1h2ao | 0.35373 | -0.99559 | 10569 | |
| mmu-mir-193b | 0.35373 | -0.6211 | 10570 | |
| Gm4788 | 0.35379 | -0.22704 | 10571 | |
| Xrn2 | 0.35387 | -0.81556 | 10572 | |
| Nlrp9c | 0.35389 | -0.82555 | 10573 | |
| Rnf43 | 0.35391 | -0.45801 | 10574 | |
| Slc39a12 | 0.354 | 0.14056 | 10575 | |
| Irx3 | 0.35407 | -0.14246 | 10576 | |
| mmu-mir-7220 | 0.35434 | -0.34652 | 10577 | |
| mmu-mir-3070b | 0.35435 | -0.81796 | 10578 | |
| Sh2d5 | 0.35437 | 1.7063 | 10579 | |
| Trpc7 | 0.35439 | -0.7681 | 10580 | |
| Il11 | 0.35456 | 0.35922 | 10581 | |
| Mmp9 | 0.35457 | -0.82271 | 10582 | |
| Olfr63 | 0.35464 | -0.43484 | 10583 | |
| Arid2 | 0.35468 | -0.81341 | 10584 | |
| Slc4a7 | 0.35476 | -0.20698 | 10585 | |
| Tmed11 | 0.35479 | -0.81686 | 10586 | |
| Nphp3 | 0.35485 | -0.064698 | 10587 | |
| Tuba3b | 0.35488 | -0.81244 | 10588 | |
| Appbp2 | 0.35495 | -0.40454 | 10589 | |
| 4922502D21Rik | 0.35506 | -0.41307 | 10590 | |
| mmu-mir-199b | 0.35506 | -0.81923 | 10591 | |
| Gls | 0.35522 | -0.46194 | 10592 | |
| Nlrp9b | 0.35526 | 0.48312 | 10593 | |
| Tmco5 | 0.35532 | -0.81298 | 10594 | |
| Padi3 | 0.35532 | 0.0024222 | 10595 | |
| Msh2 | 0.35538 | -0.55568 | 10596 | |
| Xrcc2 | 0.35542 | -0.46974 | 10597 | |
| Ccl6 | 0.35546 | -0.10677 | 10598 | |
| Fsd1l | 0.35549 | 0.35717 | 10599 | |
| Klk1 | 0.35553 | 0.91085 | 10600 | |
| Zbtb40 | 0.35553 | -0.82078 | 10601 | |
| Xpnpep2 | 0.35555 | -0.81404 | 10602 | |
| Six6 | 0.35556 | -0.64051 | 10603 | |
| Arhgap24 | 0.3556 | 0.086562 | 10604 | |
| Lrmp | 0.35577 | -0.26449 | 10605 | |
| Prss39 | 0.35579 | -0.80854 | 10606 | |
| mmu-mir-703 | 0.35581 | 0.32725 | 10607 | |
| Errfi1 | 0.3559 | -0.46807 | 10608 | |
| Slc35a4 | 0.35594 | 1.162 | 10609 | |
| Prkg2 | 0.356 | 2.7431 | 10610 | |
| mmu-mir-134 | 0.35613 | -0.50924 | 10611 | |
| Dctn3 | 0.35617 | -0.8162 | 10612 | |
| Gm1123 | 0.35617 | 0.25174 | 10613 | |
| Ndst3 | 0.35619 | -0.50578 | 10614 | |
| Slc27a2 | 0.35621 | 0.49245 | 10615 | |
| Scg3 | 0.35623 | -0.81972 | 10616 | |
| Shcbp1l | 0.35631 | 0.64071 | 10617 | |
| Itgb7 | 0.35634 | 0.34488 | 10618 | |
| Dennd1a | 0.35636 | -0.72009 | 10619 | |
| Snx21 | 0.35638 | 2.5053 | 10620 | |
| Trim56 | 0.35645 | -0.81734 | 10621 | |
| Sgms2 | 0.35654 | -0.6981 | 10622 | |
| Gm10665 | 0.35661 | -0.49152 | 10623 | |
| mmu-mir-6715 | 0.35661 | 0.15862 | 10624 | |
| Ndfip1 | 0.35665 | 0.45803 | 10625 | |
| Bak1 | 0.35672 | 0.51303 | 10626 | |
| Tbl1xr1 | 0.35674 | -0.80015 | 10627 | |
| Cldn17 | 0.35682 | -0.50268 | 10628 | |
| Fam150a | 0.35682 | -0.79105 | 10629 | |
| Cdc42bpg | 0.35687 | -0.79981 | 10630 | |
| Gh | 0.35695 | -0.57686 | 10631 | |
| Tex15 | 0.35698 | -0.80433 | 10632 | |
| Bend6 | 0.35702 | -0.7955 | 10633 | |
| Atg4d | 0.35704 | -0.81888 | 10634 | |
| Ccdc88c | 0.35708 | -0.81534 | 10635 | |
| Tmem98 | 0.35721 | -0.8169 | 10636 | |
| Zfp398 | 0.35724 | -0.38623 | 10637 | |
| Pcdhgc4 | 0.35724 | 0.58795 | 10638 | |
| Cryba2 | 0.35735 | -0.60646 | 10639 | |
| mmu-mir-880 | 0.3574 | -0.63687 | 10640 | |
| Ctla2b | 0.35743 | 0.36876 | 10641 | |
| Vmn1r172 | 0.35748 | -0.79598 | 10642 | |
| Car5a | 0.35753 | -0.80034 | 10643 | |
| Dnajc28 | 0.35761 | -0.80526 | 10644 | |
| Smim13 | 0.35766 | -0.71363 | 10645 | |
| Psg21 | 0.35773 | 0.7691 | 10646 | |
| Ctag2 | 0.35774 | -0.79865 | 10647 | |
| Pgls | 0.35777 | -0.80831 | 10648 | |
| Tmem196 | 0.35777 | 1.4015 | 10649 | |
| Tjp3 | 0.35787 | -0.54412 | 10650 | |
| Bola3 | 0.358 | -0.70285 | 10651 | |
| Igsf23 | 0.35825 | -0.80391 | 10652 | |
| Blvrb | 0.35828 | -0.81179 | 10653 | |
| Oas1b | 0.35828 | -0.81179 | 10654 | |
| 1700001K19Rik | 0.35831 | 0.060108 | 10655 | |
| Arhgef19 | 0.35831 | -0.80299 | 10656 | |
| mmu-mir-412 | 0.3584 | 1.5896 | 10657 | |
| Pafah2 | 0.3584 | -0.80742 | 10658 | |
| Tmem237 | 0.35847 | -0.018342 | 10659 | |
| Mpg | 0.35851 | 0.40056 | 10660 | |
| Tapbpl | 0.35853 | -0.81009 | 10661 | |
| mmu-mir-6899 | 0.35862 | 0.68481 | 10662 | |
| Hcn2 | 0.35875 | 0.59288 | 10663 | |
| Tpi1 | 0.3588 | -0.80534 | 10664 | |
| Fitm1 | 0.35885 | -0.80772 | 10665 | |
| Cib2 | 0.35891 | -0.80371 | 10666 | |
| Fam221a | 0.35893 | -0.7902 | 10667 | |
| Paxbp1 | 0.35899 | -0.38067 | 10668 | |
| Ddc | 0.35899 | -0.80203 | 10669 | |
| mmu-mir-7001 | 0.35901 | -0.57377 | 10670 | |
| Clec4a1 | 0.35908 | -0.81003 | 10671 | |
| Mpc2 | 0.35914 | -0.81281 | 10672 | |
| Gm20823 | 0.35922 | -0.98852 | 10673 | |
| Olfr342 | 0.35926 | -0.58388 | 10674 | |
| Pdp2 | 0.3593 | -0.79227 | 10675 | |
| Mrpl1 | 0.35931 | 2.523 | 10676 | |
| Cox7b2 | 0.35936 | -0.038387 | 10677 | |
| Rundc3a | 0.35939 | -0.79787 | 10678 | |
| mmu-mir-3087 | 0.3594 | 0.21402 | 10679 | |
| Olfr559 | 0.35943 | 0.081582 | 10680 | |
| Tsr3 | 0.35946 | -0.38638 | 10681 | |
| Alkbh1 | 0.35956 | -0.64496 | 10682 | |
| Vps72 | 0.35965 | -0.46479 | 10683 | |
| Calcrl | 0.35965 | 0.073649 | 10684 | |
| Aplnr | 0.35967 | -0.7927 | 10685 | |
| Supt20 | 0.3597 | -0.79765 | 10686 | |
| mmu-mir-140 | 0.3597 | -0.4021 | 10687 | |
| Olfr722 | 0.35971 | 0.10827 | 10688 | |
| Irf3 | 0.35976 | 0.54396 | 10689 | |
| 4931429I11Rik | 0.35978 | -0.12059 | 10690 | |
| Lmod1 | 0.35985 | -0.69554 | 10691 | |
| Olfr220 | 0.3599 | -0.39327 | 10692 | |
| Slc44a5 | 0.35996 | -0.79835 | 10693 | |
| Mllt11 | 0.35998 | -0.14508 | 10694 | |
| Rce1 | 0.36003 | -0.48392 | 10695 | |
| Vmn1r56 | 0.36007 | -0.20792 | 10696 | |
| BC048502 | 0.36009 | -0.78775 | 10697 | |
| mmu-mir-721 | 0.36016 | -0.59121 | 10698 | |
| Klra21 | 0.3602 | 0.24833 | 10699 | |
| Vamp1 | 0.36022 | -0.78435 | 10700 | |
| Itga2b | 0.36024 | -0.61945 | 10701 | |
| Mup6 | 0.36029 | -0.25432 | 10702 | |
| Serpinb9d | 0.36034 | -0.71667 | 10703 | |
| Pzp | 0.36034 | 0.52158 | 10704 | |
| Lrrc1 | 0.3604 | -0.80908 | 10705 | |
| Hsd17b4 | 0.36044 | -0.78854 | 10706 | |
| mmu-mir-2137 | 0.36059 | -0.48891 | 10707 | |
| Zbtb21 | 0.36064 | -0.33589 | 10708 | |
| Olfr1195 | 0.36068 | 0.2061 | 10709 | |
| Olfr1245 | 0.36068 | -0.54956 | 10710 | |
| Ier3ip1 | 0.36071 | -0.80127 | 10711 | |
| Klc4 | 0.36098 | -0.093182 | 10712 | |
| Olfr1427 | 0.36106 | -0.38957 | 10713 | |
| Ascl2 | 0.36106 | -0.72273 | 10714 | |
| Orc1 | 0.36121 | -0.79116 | 10715 | |
| Zdhhc2 | 0.36128 | -0.13656 | 10716 | |
| Thsd1 | 0.36128 | 1.2846 | 10717 | |
| Maea | 0.36134 | -0.62289 | 10718 | |
| Olfr175-ps1 | 0.36139 | -0.80686 | 10719 | |
| Ptk6 | 0.36143 | -0.76144 | 10720 | |
| Cd69 | 0.3615 | -0.70434 | 10721 | |
| Pglyrp3 | 0.36155 | -0.74448 | 10722 | |
| Gm3336 | 0.36158 | -0.10656 | 10723 | |
| Maneal | 0.36176 | -0.46857 | 10724 | |
| Pdzd11 | 0.36176 | -0.64307 | 10725 | |
| Mboat4 | 0.36178 | -0.21109 | 10726 | |
| L2hgdh | 0.36182 | 1.1031 | 10727 | |
| Olfr1462 | 0.36189 | -0.64658 | 10728 | |
| Mea1 | 0.36191 | -0.79438 | 10729 | |
| mmu-mir-6925 | 0.36192 | 1.0483 | 10730 | |
| Mageb18 | 0.36196 | -0.029132 | 10731 | |
| Olfr362 | 0.36198 | -0.79971 | 10732 | |
| Mroh6 | 0.362 | -0.27072 | 10733 | |
| Fam159b | 0.36206 | -0.27575 | 10734 | |
| Vat1l | 0.36206 | 0.60485 | 10735 | |
| Abracl | 0.36207 | -0.43091 | 10736 | |
| Uap1 | 0.36216 | -0.059909 | 10737 | |
| Slc1a1 | 0.36218 | -0.7925 | 10738 | |
| Gm11128 | 0.3622 | 1.4937 | 10739 | |
| Crls1 | 0.3622 | -0.07308 | 10740 | |
| Eif4g2 | 0.36224 | -0.36724 | 10741 | |
| Lrrc39 | 0.36226 | -0.44192 | 10742 | |
| Sparcl1 | 0.3623 | -0.08779 | 10743 | |
| Prss45 | 0.36231 | -0.8012 | 10744 | |
| Wdr81 | 0.36236 | -0.68351 | 10745 | |
| Fadd | 0.3624 | 1.7995 | 10746 | |
| Rnf115 | 0.36242 | -0.41538 | 10747 | |
| Polr3h | 0.36252 | 0.25413 | 10748 | |
| Atp2c1 | 0.36258 | -0.3643 | 10749 | |
| Kiss1r | 0.36259 | -0.78498 | 10750 | |
| Lmbrd2 | 0.36263 | -0.30127 | 10751 | |
| Vmn2r92 | 0.36264 | -0.79256 | 10752 | |
| Acd | 0.36267 | 0.10654 | 10753 | |
| Olfr705 | 0.36288 | -0.47054 | 10754 | |
| Gsta4 | 0.3629 | 0.48872 | 10755 | |
| Sh3bgrl | 0.36292 | -0.79807 | 10756 | |
| Olfr1161 | 0.36297 | -0.3091 | 10757 | |
| Casp8 | 0.36307 | -0.61724 | 10758 | |
| Cpsf1 | 0.36312 | -0.78534 | 10759 | |
| Bmp1 | 0.36317 | -0.36164 | 10760 | |
| Olfr1444 | 0.36321 | -0.18605 | 10761 | |
| mmu-mir-7233 | 0.36322 | -0.56252 | 10762 | |
| Pcf11 | 0.36332 | -0.66955 | 10763 | |
| Lipt2 | 0.36334 | -0.63604 | 10764 | |
| Trappc9 | 0.36337 | -0.048852 | 10765 | |
| Lamc3 | 0.36347 | -0.78577 | 10766 | |
| Tbc1d7 | 0.36348 | 0.23815 | 10767 | |
| Mgat5 | 0.3635 | -0.31465 | 10768 | |
| Gpr52 | 0.36352 | -0.78107 | 10769 | |
| Chrdl1 | 0.36354 | 0.71357 | 10770 | |
| Agfg1 | 0.36356 | -0.7953 | 10771 | |
| Pak6 | 0.36358 | 0.68605 | 10772 | |
| Clec12a | 0.36368 | -0.15596 | 10773 | |
| Bod1l | 0.36371 | 0.77349 | 10774 | |
| Sepw1 | 0.36375 | -0.46216 | 10775 | |
| Aldh4a1 | 0.36376 | -0.2771 | 10776 | |
| Olfr251 | 0.36378 | -0.11183 | 10777 | |
| Vmn2r50 | 0.36378 | -0.7968 | 10778 | |
| Aasdh | 0.36381 | -0.47715 | 10779 | |
| Trim28 | 0.36383 | -0.5861 | 10780 | |
| Gorasp1 | 0.36388 | 1.1324 | 10781 | |
| Hnrnpab | 0.36394 | -0.79127 | 10782 | |
| Olfr1508 | 0.36398 | -0.44885 | 10783 | |
| Slc25a25 | 0.364 | -0.77876 | 10784 | |
| Hdac11 | 0.36408 | -0.25791 | 10785 | |
| mmu-mir-3967 | 0.36418 | 0.5103 | 10786 | |
| Nos1 | 0.36419 | -0.79008 | 10787 | |
| Iffo2 | 0.36428 | -0.79205 | 10788 | |
| Olfr575 | 0.36428 | 0.82197 | 10789 | |
| Tirap | 0.36438 | 1.3334 | 10790 | |
| Olfr472 | 0.36442 | -0.25715 | 10791 | |
| Atg10 | 0.36461 | -0.78743 | 10792 | |
| Abr | 0.3647 | -0.57565 | 10793 | |
| Fance | 0.36471 | -0.77244 | 10794 | |
| B4galt2 | 0.36475 | -0.43984 | 10795 | |
| Kcng4 | 0.36477 | -0.79282 | 10796 | |
| Adra2c | 0.36484 | -0.55218 | 10797 | |
| Lmo4 | 0.36486 | -0.45048 | 10798 | |
| Nnmt | 0.36489 | -0.0678 | 10799 | |
| Plcxd1 | 0.36492 | 0.34118 | 10800 | |
| Scara5 | 0.36495 | -0.71569 | 10801 | |
| Prl3d1 | 0.36506 | -0.58232 | 10802 | |
| Ppp1r13b | 0.36509 | 1.1182 | 10803 | |
| Trappc2l | 0.36529 | -0.3725 | 10804 | |
| Anxa11 | 0.36534 | -0.40109 | 10805 | |
| Mettl1 | 0.36536 | -0.74973 | 10806 | |
| Ldoc1 | 0.36539 | 1.2662 | 10807 | |
| Nbea | 0.36543 | 0.21103 | 10808 | |
| Rab30 | 0.36544 | -0.78848 | 10809 | |
| Emb | 0.36546 | 0.034511 | 10810 | |
| Calr3 | 0.36548 | -0.77599 | 10811 | |
| Ctc1 | 0.36549 | -0.7233 | 10812 | |
| BC089597 | 0.36552 | -0.78789 | 10813 | |
| Apln | 0.36554 | -0.2695 | 10814 | |
| Tas2r131 | 0.36556 | -0.79747 | 10815 | |
| Lrrc29 | 0.36566 | 1.4347 | 10816 | |
| Icam2 | 0.36581 | -0.4775 | 10817 | |
| Ugt3a1 | 0.36592 | -0.78332 | 10818 | |
| mmu-mir-7068 | 0.36594 | 0.29045 | 10819 | |
| Htr1f | 0.36602 | -0.78904 | 10820 | |
| Kif2c | 0.36603 | -0.079754 | 10821 | |
| Olfr392 | 0.36607 | -0.63024 | 10822 | |
| Stk11 | 0.3661 | 1.292 | 10823 | |
| Hesx1 | 0.3662 | 1.4982 | 10824 | |
| Efna2 | 0.3662 | -0.78292 | 10825 | |
| Gm14920 | 0.36625 | -0.66401 | 10826 | |
| Hmgcs2 | 0.36627 | -0.78667 | 10827 | |
| Akr1cl | 0.36629 | -0.78627 | 10828 | |
| Krtap8-1 | 0.36631 | -0.79547 | 10829 | |
| Smdt1 | 0.36638 | -0.13637 | 10830 | |
| Fcgbp | 0.3665 | 0.72776 | 10831 | |
| Gal3st1 | 0.36654 | -0.429 | 10832 | |
| Bsx | 0.36655 | -0.77245 | 10833 | |
| 3110043O21Rik | 0.36662 | -0.78506 | 10834 | |
| Zfp273 | 0.3667 | 2.0138 | 10835 | |
| Siglece | 0.36674 | -0.7255 | 10836 | |
| Vmn2r117 | 0.36684 | -0.79315 | 10837 | |
| Trpv5 | 0.36687 | 0.097301 | 10838 | |
| Ctrc | 0.36691 | 0.25866 | 10839 | |
| Mettl7b | 0.36694 | -0.6418 | 10840 | |
| Ninj2 | 0.36695 | -0.2342 | 10841 | |
| Hs3st3a1 | 0.36702 | -0.78231 | 10842 | |
| Olfr301 | 0.36706 | -0.78352 | 10843 | |
| Zfp13 | 0.36708 | -0.77377 | 10844 | |
| mmu-mir-7653 | 0.36709 | -0.36681 | 10845 | |
| Slc36a1 | 0.36709 | -0.25436 | 10846 | |
| Dmrtc1c2 | 0.36711 | -0.69093 | 10847 | |
| Olfr1392 | 0.36715 | -0.7416 | 10848 | |
| Abhd1 | 0.36721 | -0.43254 | 10849 | |
| Rhox2f | 0.36724 | 0.18498 | 10850 | |
| Lrrfip1 | 0.36726 | -0.66398 | 10851 | |
| Pou2f3 | 0.36733 | -0.77313 | 10852 | |
| Neurod4 | 0.36737 | -0.47229 | 10853 | |
| Arhgef7 | 0.36741 | -0.79076 | 10854 | |
| Pikfyve | 0.36744 | -0.28556 | 10855 | |
| Txndc8 | 0.36749 | 0.50206 | 10856 | |
| Dhx29 | 0.36749 | 0.13818 | 10857 | |
| Gm715 | 0.3675 | -0.77415 | 10858 | |
| Akap3 | 0.36764 | -0.32032 | 10859 | |
| Cyyr1 | 0.36768 | -0.14932 | 10860 | |
| Hspa12a | 0.36784 | -0.33567 | 10861 | |
| mmu-mir-449c | 0.36788 | -0.32983 | 10862 | |
| Spatc1 | 0.36792 | -0.7646 | 10863 | |
| Skint7 | 0.36798 | 1.5199 | 10864 | |
| Olfr596 | 0.36799 | -0.78599 | 10865 | |
| Gm4884 | 0.36801 | -0.77743 | 10866 | |
| Ercc5 | 0.36801 | -0.26011 | 10867 | |
| Nmt2 | 0.36805 | -0.7711 | 10868 | |
| Fam43b | 0.36811 | 0.82334 | 10869 | |
| Ttr | 0.36816 | -0.77382 | 10870 | |
| Krt34 | 0.36821 | -0.73483 | 10871 | |
| Zfp777 | 0.36825 | -0.38292 | 10872 | |
| Rb1cc1 | 0.3683 | -0.77493 | 10873 | |
| mmu-mir-743b | 0.36832 | -0.78937 | 10874 | |
| Ppm1g | 0.36834 | -0.50282 | 10875 | |
| Arl4c | 0.36838 | 0.21171 | 10876 | |
| Ube2o | 0.36841 | -0.7637 | 10877 | |
| Ros1 | 0.36848 | 1.1807 | 10878 | |
| Neurl1b | 0.36857 | -0.78556 | 10879 | |
| Dlx2 | 0.36862 | 0.19904 | 10880 | |
| Olfr96 | 0.36863 | -0.77272 | 10881 | |
| Sub1 | 0.36865 | -0.7588 | 10882 | |
| Cep76 | 0.36874 | -0.4664 | 10883 | |
| Mucl1 | 0.36882 | -0.78186 | 10884 | |
| Nxn | 0.36886 | -0.77942 | 10885 | |
| Nos1ap | 0.36886 | -0.29614 | 10886 | |
| Meis1 | 0.36891 | -0.53121 | 10887 | |
| Siglecg | 0.36897 | 0.33971 | 10888 | |
| Gpam | 0.36897 | -0.50507 | 10889 | |
| Pdcd10 | 0.36903 | -0.6128 | 10890 | |
| Pcbd2 | 0.36904 | -0.76889 | 10891 | |
| Sav1 | 0.36907 | -0.067009 | 10892 | |
| Efcab11 | 0.36908 | -0.68282 | 10893 | |
| Rfesd | 0.36916 | -0.77373 | 10894 | |
| Agap3 | 0.3692 | -0.77113 | 10895 | |
| Trim14 | 0.36922 | -0.00049262 | 10896 | |
| Fndc7 | 0.36925 | 0.63909 | 10897 | |
| Gm10662 | 0.36927 | -0.78404 | 10898 | |
| Trnp1 | 0.36938 | -0.77116 | 10899 | |
| Ammecr1l | 0.36939 | 0.21602 | 10900 | |
| Mup17 | 0.36941 | -0.94355 | 10901 | |
| Scarb2 | 0.36951 | -0.76309 | 10902 | |
| Lrrc4 | 0.36953 | -0.49042 | 10903 | |
| Chrdl2 | 0.36957 | -0.7679 | 10904 | |
| Nck2 | 0.36962 | -0.06706 | 10905 | |
| Igfl3 | 0.36969 | 0.16403 | 10906 | |
| Zfp366 | 0.36977 | -0.45542 | 10907 | |
| Wwc1 | 0.36982 | -0.76919 | 10908 | |
| Arfgap1 | 0.36984 | -0.72234 | 10909 | |
| Eapp | 0.36997 | -0.76924 | 10910 | |
| Pi4k2b | 0.37006 | -0.77134 | 10911 | |
| Spag6 | 0.37017 | 0.66492 | 10912 | |
| Chac2 | 0.37019 | -0.77056 | 10913 | |
| Ms4a8a | 0.37029 | -0.44717 | 10914 | |
| Ccdc108 | 0.37031 | -0.77102 | 10915 | |
| Gm5886 | 0.37039 | 0.030692 | 10916 | |
| Fam227a | 0.37042 | 0.70349 | 10917 | |
| Cacul1 | 0.37046 | -0.7681 | 10918 | |
| Map4k1 | 0.37052 | -0.76893 | 10919 | |
| Trp53bp1 | 0.37054 | -0.084618 | 10920 | |
| Npsr1 | 0.37059 | 0.10951 | 10921 | |
| Gpc6 | 0.37074 | -0.32291 | 10922 | |
| Rtn2 | 0.37085 | -0.7659 | 10923 | |
| Aimp2 | 0.37086 | -0.65247 | 10924 | |
| Dnttip1 | 0.37088 | -0.77148 | 10925 | |
| Phkg2 | 0.37092 | 0.74119 | 10926 | |
| Arl6ip1 | 0.37094 | -0.19798 | 10927 | |
| Tmie | 0.37096 | -0.41158 | 10928 | |
| Dpep1 | 0.37099 | 0.68979 | 10929 | |
| Ikbke | 0.37109 | -0.56456 | 10930 | |
| Luc7l | 0.37112 | -0.76802 | 10931 | |
| mmu-mir-511 | 0.37114 | -0.44611 | 10932 | |
| Pik3c2a | 0.37118 | -0.76103 | 10933 | |
| mmu-mir-212 | 0.37119 | -0.61777 | 10934 | |
| Fchsd1 | 0.37122 | 0.52966 | 10935 | |
| Fam221b | 0.37125 | -0.76475 | 10936 | |
| Pitpna | 0.37132 | -0.66925 | 10937 | |
| Snurf | 0.37134 | -0.75773 | 10938 | |
| Zfp874b | 0.37141 | -0.77188 | 10939 | |
| Amph | 0.3716 | -0.12779 | 10940 | |
| D10Jhu81e | 0.37165 | -0.77201 | 10941 | |
| Ep400 | 0.37167 | -0.75479 | 10942 | |
| Ppp1r42 | 0.37176 | -0.75683 | 10943 | |
| Dcbld1 | 0.37179 | 1.4355 | 10944 | |
| Fos | 0.37183 | -0.40393 | 10945 | |
| Cox6a2 | 0.37186 | 0.23757 | 10946 | |
| Cbr4 | 0.37189 | -0.44106 | 10947 | |
| Gip | 0.37191 | -0.73214 | 10948 | |
| Ttll6 | 0.37194 | -0.7781 | 10949 | |
| Traf1 | 0.37196 | -0.26708 | 10950 | |
| Pigf | 0.37202 | -0.77554 | 10951 | |
| Syt10 | 0.37203 | 0.070417 | 10952 | |
| Cpne1 | 0.37211 | -0.51328 | 10953 | |
| Adamts2 | 0.37216 | -0.77836 | 10954 | |
| Itgav | 0.37216 | -0.52073 | 10955 | |
| Acp6 | 0.37223 | 0.25046 | 10956 | |
| Afap1l2 | 0.37233 | -0.76127 | 10957 | |
| Bloc1s2 | 0.37238 | -0.76437 | 10958 | |
| Olfr1276 | 0.3724 | -0.77146 | 10959 | |
| Phospho2 | 0.37241 | -0.36064 | 10960 | |
| Epc2 | 0.37242 | -0.76957 | 10961 | |
| Gm7157 | 0.37244 | -0.7552 | 10962 | |
| Prnp | 0.37266 | 0.0052113 | 10963 | |
| Vps37a | 0.37273 | -0.5797 | 10964 | |
| Olfr1122 | 0.37277 | -0.77668 | 10965 | |
| Tnks | 0.37286 | -0.37059 | 10966 | |
| Kdm5b | 0.37293 | -0.73268 | 10967 | |
| Lemd2 | 0.37301 | -0.75999 | 10968 | |
| Nagpa | 0.37303 | 1.0787 | 10969 | |
| Rtp4 | 0.37306 | -0.55733 | 10970 | |
| Cox19 | 0.37308 | -0.74745 | 10971 | |
| Ccdc138 | 0.37313 | -0.64121 | 10972 | |
| Cntn3 | 0.37316 | -0.48148 | 10973 | |
| Gm20172 | 0.37319 | 0.14314 | 10974 | |
| Gabrb1 | 0.37319 | -0.76728 | 10975 | |
| Daglb | 0.37323 | -0.4342 | 10976 | |
| Igsf3 | 0.37326 | 1.6158 | 10977 | |
| C920025E04Rik | 0.37326 | -0.70781 | 10978 | |
| 4932414N04Rik | 0.37329 | 0.24131 | 10979 | |
| Gpr81 | 0.37339 | -0.56873 | 10980 | |
| Stard10 | 0.37346 | -0.75878 | 10981 | |
| Zfp819 | 0.37359 | -0.2945 | 10982 | |
| Hic2 | 0.37361 | -0.76222 | 10983 | |
| Luzp1 | 0.37366 | -0.5119 | 10984 | |
| Slc16a5 | 0.37375 | -0.74658 | 10985 | |
| mmu-mir-1894 | 0.37375 | -0.27685 | 10986 | |
| Nebl | 0.37377 | -0.75854 | 10987 | |
| Iqcd | 0.37384 | -0.10352 | 10988 | |
| Lman2 | 0.37396 | 1.2728 | 10989 | |
| mmu-mir-6516 | 0.374 | -0.48201 | 10990 | |
| Slc23a2 | 0.37403 | -0.61373 | 10991 | |
| Papd7 | 0.37408 | -0.77185 | 10992 | |
| Tmem17 | 0.37423 | -0.73924 | 10993 | |
| Rbm33 | 0.37429 | 1.2203 | 10994 | |
| Cav1 | 0.3743 | -0.74483 | 10995 | |
| Twist2 | 0.37439 | -0.76094 | 10996 | |
| Timp1 | 0.37439 | 0.72251 | 10997 | |
| Adamts13 | 0.37445 | -0.76771 | 10998 | |
| Commd1 | 0.3745 | -0.65593 | 10999 | |
| 9230110C19Rik | 0.37453 | 0.82378 | 11000 | |
| 4931440F15Rik | 0.37454 | -0.74909 | 11001 | |
| Anxa7 | 0.37459 | 0.27817 | 11002 | |
| Lsm6 | 0.37466 | -0.066026 | 11003 | |
| Gdf2 | 0.37476 | 0.54832 | 11004 | |
| Celf1 | 0.37479 | -0.75569 | 11005 | |
| C1qtnf4 | 0.37479 | 0.35575 | 11006 | |
| P2rx2 | 0.37503 | -0.46848 | 11007 | |
| Disc1 | 0.37503 | -0.75076 | 11008 | |
| Mbnl2 | 0.37506 | -0.55338 | 11009 | |
| Dhx30 | 0.37514 | -0.74996 | 11010 | |
| Elf2 | 0.37516 | -0.75618 | 11011 | |
| Zmynd8 | 0.37519 | -0.36629 | 11012 | |
| Ipo5 | 0.37521 | -0.76172 | 11013 | |
| mmu-mir-7242 | 0.37527 | -0.47632 | 11014 | |
| Aqp3 | 0.37536 | -0.32657 | 11015 | |
| Mrpl14 | 0.37539 | 0.40867 | 11016 | |
| Olfr583 | 0.37541 | -0.76321 | 11017 | |
| Gast | 0.37544 | -0.76092 | 11018 | |
| Apoe | 0.37546 | -0.21335 | 11019 | |
| Lysmd4 | 0.37547 | -0.68441 | 11020 | |
| Pogz | 0.37552 | -0.46364 | 11021 | |
| Sema6d | 0.37566 | -0.38703 | 11022 | |
| Slc29a1 | 0.37572 | -0.53697 | 11023 | |
| Fam227b | 0.37574 | -0.76125 | 11024 | |
| Tmem125 | 0.37576 | 0.03607 | 11025 | |
| mmu-mir-125b-1 | 0.37584 | -0.33756 | 11026 | |
| Usp53 | 0.37586 | -0.76509 | 11027 | |
| Ptch2 | 0.37594 | -0.74822 | 11028 | |
| Rab9 | 0.37596 | 1.1852 | 11029 | |
| Gm10267 | 0.37597 | -0.76669 | 11030 | |
| Olfr420 | 0.37599 | 0.32351 | 11031 | |
| Olfr1472 | 0.37605 | -0.7649 | 11032 | |
| Fbxw9 | 0.37606 | 0.17876 | 11033 | |
| Slc1a7 | 0.37607 | -0.73632 | 11034 | |
| Lrrn3 | 0.37611 | -0.72606 | 11035 | |
| Txndc17 | 0.37612 | -0.16491 | 11036 | |
| Il19 | 0.37614 | -0.75695 | 11037 | |
| Ap5s1 | 0.37616 | -0.7549 | 11038 | |
| Bpgm | 0.37626 | 0.24034 | 11039 | |
| Gm10471 | 0.37629 | -0.3616 | 11040 | |
| Olfr51 | 0.37629 | -0.7608 | 11041 | |
| Relt | 0.37639 | 0.76658 | 11042 | |
| Clec18a | 0.37642 | -0.72654 | 11043 | |
| Klhl41 | 0.37645 | -0.74589 | 11044 | |
| Creb3l3 | 0.37645 | -0.31192 | 11045 | |
| mmu-mir-1931 | 0.37648 | 0.41052 | 11046 | |
| 1700067P10Rik | 0.37651 | -0.16521 | 11047 | |
| Eda | 0.37652 | -0.078035 | 11048 | |
| Mrpl9 | 0.37653 | -0.73336 | 11049 | |
| Rrs1 | 0.3766 | -0.16973 | 11050 | |
| Eif1ax | 0.37664 | -0.76444 | 11051 | |
| Vmn2r8 | 0.37665 | 0.35839 | 11052 | |
| Lgals9 | 0.37669 | -0.27662 | 11053 | |
| Olfr658 | 0.37672 | -0.7509 | 11054 | |
| Olfr716 | 0.37682 | 0.59041 | 11055 | |
| Pcbp3 | 0.37691 | -0.11248 | 11056 | |
| Olfr1448 | 0.37702 | -0.68685 | 11057 | |
| Gm12789 | 0.37706 | -0.75589 | 11058 | |
| Kit | 0.37709 | -0.73357 | 11059 | |
| Vmn1r200 | 0.37712 | -0.24588 | 11060 | |
| Wipi1 | 0.37715 | -0.64469 | 11061 | |
| Abcb10 | 0.3772 | -0.76293 | 11062 | |
| Adprh | 0.37722 | -0.74668 | 11063 | |
| Dleu7 | 0.37724 | -0.74843 | 11064 | |
| Ppard | 0.37728 | -0.38183 | 11065 | |
| BC100451 | 0.37732 | 2.7622 | 11066 | |
| Lbp | 0.37735 | -0.72979 | 11067 | |
| Selenbp2 | 0.37738 | 1.1718 | 11068 | |
| Kif17 | 0.3774 | -0.53608 | 11069 | |
| Cyp4b1 | 0.37745 | 0.52979 | 11070 | |
| Caln1 | 0.37749 | -0.75467 | 11071 | |
| Wdfy1 | 0.37755 | -0.45822 | 11072 | |
| Slc37a2 | 0.37762 | -0.75325 | 11073 | |
| Ubqln2 | 0.37765 | -0.080374 | 11074 | |
| Lactb | 0.37768 | -0.74589 | 11075 | |
| Timm50 | 0.37773 | -0.70285 | 11076 | |
| Taldo1 | 0.37778 | -0.60644 | 11077 | |
| Fam199x | 0.37782 | 0.79838 | 11078 | |
| Esam | 0.37784 | -0.73884 | 11079 | |
| Olfr212 | 0.37786 | -0.75442 | 11080 | |
| Agrn | 0.37788 | -0.32101 | 11081 | |
| Gnpnat1 | 0.37791 | -0.75775 | 11082 | |
| Eqtn | 0.37798 | 0.39416 | 11083 | |
| 2610034B18Rik | 0.37802 | -0.75078 | 11084 | |
| Krt222 | 0.37804 | -0.74987 | 11085 | |
| Rgs3 | 0.37805 | -0.71668 | 11086 | |
| Mex3a | 0.37806 | -0.73961 | 11087 | |
| Olfr889 | 0.37808 | -0.4309 | 11088 | |
| Pdgfrb | 0.37811 | -0.15955 | 11089 | |
| Kcnj14 | 0.37811 | 1.6937 | 11090 | |
| Hoxa5 | 0.37815 | 1.105 | 11091 | |
| Ssh2 | 0.37826 | -0.73222 | 11092 | |
| Vmn1r124 | 0.37828 | -0.72312 | 11093 | |
| Klk1b22 | 0.37833 | -0.73314 | 11094 | |
| Dbnl | 0.37841 | 0.42179 | 11095 | |
| Gpatch2l | 0.37842 | -0.17495 | 11096 | |
| Crp | 0.37846 | -0.74011 | 11097 | |
| H2-M3 | 0.37851 | 0.8598 | 11098 | |
| Calm4 | 0.37854 | -0.73987 | 11099 | |
| Klhl17 | 0.37855 | 0.89468 | 11100 | |
| Baat | 0.37857 | -0.72188 | 11101 | |
| Mprip | 0.37866 | -0.74412 | 11102 | |
| Nudt14 | 0.37868 | 0.49398 | 11103 | |
| Cntrob | 0.37868 | 0.076184 | 11104 | |
| Vti1b | 0.37874 | -0.161 | 11105 | |
| 1700001O22Rik | 0.37878 | 0.19446 | 11106 | |
| Nxph3 | 0.37879 | -0.7436 | 11107 | |
| Fuom | 0.37881 | 0.55335 | 11108 | |
| Klf11 | 0.37881 | -0.74492 | 11109 | |
| Rbm20 | 0.37886 | -0.3269 | 11110 | |
| Enox1 | 0.37886 | -0.66851 | 11111 | |
| Hoxd3 | 0.37888 | -0.69255 | 11112 | |
| Slc41a2 | 0.37892 | -0.4031 | 11113 | |
| Upk3bl | 0.37898 | -0.28285 | 11114 | |
| Micalcl | 0.37904 | 0.068178 | 11115 | |
| Serpinb6e | 0.37915 | -0.73903 | 11116 | |
| Vsnl1 | 0.37917 | -0.51853 | 11117 | |
| Fosl2 | 0.37924 | -0.68055 | 11118 | |
| Tradd | 0.37931 | 0.42558 | 11119 | |
| Nr5a2 | 0.37944 | 0.11758 | 11120 | |
| Olfr510 | 0.37952 | 1.2903 | 11121 | |
| Surf4 | 0.37957 | -0.36451 | 11122 | |
| Kdm2b | 0.37957 | 0.28681 | 11123 | |
| Mdfic | 0.37959 | -0.73405 | 11124 | |
| Clec9a | 0.37967 | -0.2621 | 11125 | |
| Ppp1r3d | 0.37972 | -0.24538 | 11126 | |
| Lix1l | 0.37974 | 0.73241 | 11127 | |
| Olfr851 | 0.3799 | 0.71007 | 11128 | |
| Lrrc48 | 0.37994 | 1.5061 | 11129 | |
| Gm595 | 0.37994 | -0.74051 | 11130 | |
| Ccdc13 | 0.37997 | -0.33567 | 11131 | |
| 2310042D19Rik | 0.37997 | -0.52206 | 11132 | |
| Hddc2 | 0.38 | -0.56812 | 11133 | |
| Ccdc27 | 0.38003 | -0.74992 | 11134 | |
| Cd81 | 0.38004 | -0.50176 | 11135 | |
| Ldlrad1 | 0.38007 | -0.41096 | 11136 | |
| Rnf103 | 0.3801 | 1.3224 | 11137 | |
| Mmp8 | 0.38012 | -0.75439 | 11138 | |
| Rbm12 | 0.38015 | -0.74837 | 11139 | |
| Copb1 | 0.38015 | -0.74837 | 11140 | |
| Mocos | 0.3802 | 0.11867 | 11141 | |
| Lyve1 | 0.38023 | -0.61764 | 11142 | |
| Matn2 | 0.38026 | -0.60432 | 11143 | |
| Mrgprx2 | 0.3803 | -0.74445 | 11144 | |
| Nsdhl | 0.38037 | -0.74599 | 11145 | |
| Fgd4 | 0.38039 | -0.59307 | 11146 | |
| Tmem9b | 0.38045 | 0.35094 | 11147 | |
| Msh6 | 0.3805 | 0.62202 | 11148 | |
| Ppme1 | 0.38055 | -0.74764 | 11149 | |
| AA467197 | 0.38057 | 0.15812 | 11150 | |
| Chordc1 | 0.38062 | -0.73758 | 11151 | |
| Akap8l | 0.38063 | 0.11711 | 11152 | |
| Gm4736 | 0.38066 | 0.38689 | 11153 | |
| Tmbim4 | 0.38072 | -0.75421 | 11154 | |
| Ctss | 0.38073 | -0.33701 | 11155 | |
| Plcl1 | 0.38076 | -0.72809 | 11156 | |
| Eya1 | 0.38079 | -0.72949 | 11157 | |
| Mllt6 | 0.3808 | 0.66578 | 11158 | |
| Fbxo25 | 0.38086 | 0.45579 | 11159 | |
| Nme7 | 0.3809 | -0.31502 | 11160 | |
| Pcdha3 | 0.3809 | -0.72976 | 11161 | |
| Akap5 | 0.38093 | -0.16659 | 11162 | |
| Vmn1r13 | 0.38096 | 0.15501 | 11163 | |
| Scamp4 | 0.381 | -0.60924 | 11164 | |
| Synm | 0.38103 | -0.59129 | 11165 | |
| Dppa3 | 0.38106 | -0.71893 | 11166 | |
| Vip | 0.38111 | -0.73682 | 11167 | |
| Cxcl17 | 0.38129 | -0.081254 | 11168 | |
| 1110059G10Rik | 0.3813 | -0.60867 | 11169 | |
| Pdxp | 0.38134 | -0.75273 | 11170 | |
| Atp1a4 | 0.38136 | 0.28748 | 11171 | |
| Zfp605 | 0.38141 | -0.094893 | 11172 | |
| Dpep2 | 0.38145 | -0.50328 | 11173 | |
| Armcx2 | 0.38154 | -0.4371 | 11174 | |
| Olfr119 | 0.38156 | -0.71809 | 11175 | |
| Smarca4 | 0.38159 | -0.16482 | 11176 | |
| Fbxl12 | 0.38162 | -0.021505 | 11177 | |
| Inhba | 0.38165 | -0.72578 | 11178 | |
| Klk1b21 | 0.38167 | 0.14834 | 11179 | |
| mmu-mir-5615-2 | 0.38174 | -0.90212 | 11180 | |
| Iqgap2 | 0.38177 | -0.55212 | 11181 | |
| Olfr800 | 0.38179 | -0.40031 | 11182 | |
| Abcb9 | 0.38184 | -0.75096 | 11183 | |
| Zfp652 | 0.38185 | 2.0086 | 11184 | |
| Ryk | 0.38187 | -0.73009 | 11185 | |
| 2810474O19Rik | 0.3819 | -0.72045 | 11186 | |
| mmu-mir-8119 | 0.38191 | -0.53037 | 11187 | |
| Plxdc2 | 0.38192 | -0.32909 | 11188 | |
| Hmgcr | 0.38203 | -0.73816 | 11189 | |
| 1700029H14Rik | 0.38205 | -0.73215 | 11190 | |
| Piwil2 | 0.38205 | -0.65857 | 11191 | |
| Hamp2 | 0.38207 | -0.38511 | 11192 | |
| Gm128 | 0.38215 | 0.52239 | 11193 | |
| Olfr912 | 0.38223 | -0.17288 | 11194 | |
| Iqca | 0.38227 | -0.73606 | 11195 | |
| Phf3 | 0.38234 | -0.7358 | 11196 | |
| Vmn1r29 | 0.38235 | 0.85691 | 11197 | |
| Nmu | 0.38242 | 1.3298 | 11198 | |
| Ccdc41 | 0.38246 | -0.73612 | 11199 | |
| Btnl6 | 0.38257 | -0.74407 | 11200 | |
| Adck2 | 0.38257 | -0.74407 | 11201 | |
| B4galt1 | 0.38258 | -0.40395 | 11202 | |
| Pnrc1 | 0.38263 | -0.58836 | 11203 | |
| Stk16 | 0.38272 | -0.38821 | 11204 | |
| Mettl10 | 0.38275 | 0.48449 | 11205 | |
| Hivep1 | 0.3828 | -0.68587 | 11206 | |
| St8sia5 | 0.38284 | 1.1475 | 11207 | |
| Msc | 0.38285 | -0.43443 | 11208 | |
| Adam12 | 0.38294 | -0.74043 | 11209 | |
| Nap1l5 | 0.38326 | -0.73276 | 11210 | |
| Tshr | 0.38338 | -0.73984 | 11211 | |
| P2ry10 | 0.3834 | -0.73118 | 11212 | |
| Clec2f | 0.38349 | -0.73198 | 11213 | |
| Slc38a2 | 0.38354 | -0.73286 | 11214 | |
| Abtb2 | 0.38356 | -0.72223 | 11215 | |
| Zfhx4 | 0.3836 | -0.42387 | 11216 | |
| Prps2 | 0.38363 | -0.72076 | 11217 | |
| Fpr-rs6 | 0.38364 | 0.063489 | 11218 | |
| Spata17 | 0.38367 | 1.4222 | 11219 | |
| Dtx4 | 0.38369 | -0.011347 | 11220 | |
| Lce1k | 0.38376 | -0.30649 | 11221 | |
| Fbxo32 | 0.38377 | 0.4075 | 11222 | |
| Zar1 | 0.38385 | -0.72529 | 11223 | |
| Scamp2 | 0.3839 | -0.19969 | 11224 | |
| Eml5 | 0.38392 | -0.73696 | 11225 | |
| Dlk1 | 0.38393 | -0.18623 | 11226 | |
| Oas1g | 0.38394 | -0.74535 | 11227 | |
| Zfp945 | 0.38396 | -0.72634 | 11228 | |
| Tssk6 | 0.38397 | -0.52583 | 11229 | |
| Atl1 | 0.384 | 1.5197 | 11230 | |
| Serpinb6c | 0.38405 | 0.2662 | 11231 | |
| Des | 0.38407 | -0.72559 | 11232 | |
| Inppl1 | 0.38415 | -0.72909 | 11233 | |
| Pcnx | 0.3842 | -0.55575 | 11234 | |
| Frmpd4 | 0.3842 | -0.0076382 | 11235 | |
| Sdk1 | 0.38426 | 0.8783 | 11236 | |
| Pet117 | 0.38432 | -0.72691 | 11237 | |
| Ghrl | 0.38444 | 0.27173 | 11238 | |
| 5330417C22Rik | 0.38453 | -0.098379 | 11239 | |
| Dclre1a | 0.38456 | -0.70146 | 11240 | |
| Gm4925 | 0.3846 | -0.23735 | 11241 | |
| Tnrc6a | 0.38466 | -0.62111 | 11242 | |
| Slc13a4 | 0.38467 | -0.73276 | 11243 | |
| Gm5803 | 0.38472 | -0.67083 | 11244 | |
| Galnt15 | 0.38476 | -0.10769 | 11245 | |
| Ces1e | 0.38485 | 0.039304 | 11246 | |
| Nrl | 0.38489 | -0.73254 | 11247 | |
| Fam117a | 0.38497 | -0.55188 | 11248 | |
| Olfr112 | 0.38497 | -0.72776 | 11249 | |
| Plcxd3 | 0.385 | -0.7351 | 11250 | |
| Gtf2f1 | 0.38504 | -0.73872 | 11251 | |
| Tmem29 | 0.38522 | -0.31674 | 11252 | |
| Aplp2 | 0.38523 | -0.70125 | 11253 | |
| Jun | 0.38535 | 1.7908 | 11254 | |
| Pcdhga9 | 0.38535 | -0.23274 | 11255 | |
| Dnajc5g | 0.38536 | -0.72822 | 11256 | |
| Btbd3 | 0.38539 | -0.72428 | 11257 | |
| Zfp516 | 0.38539 | -0.72428 | 11258 | |
| Ssxb1 | 0.3854 | 0.30535 | 11259 | |
| Mrgprb4 | 0.38551 | -0.38543 | 11260 | |
| Alox5 | 0.38554 | -0.66373 | 11261 | |
| Olfr1018 | 0.38561 | -0.30013 | 11262 | |
| 1700019N19Rik | 0.38569 | -0.72142 | 11263 | |
| Nr2c1 | 0.38571 | 0.12569 | 11264 | |
| Olfr173 | 0.38574 | -0.21087 | 11265 | |
| Eltd1 | 0.3858 | -0.73852 | 11266 | |
| Cd84 | 0.38581 | 1.1518 | 11267 | |
| Zfp74 | 0.38583 | -0.7298 | 11268 | |
| Lrrc57 | 0.38589 | -0.6394 | 11269 | |
| Dock11 | 0.38594 | -0.73168 | 11270 | |
| Duoxa2 | 0.38594 | -0.35266 | 11271 | |
| Stk31 | 0.386 | -0.73121 | 11272 | |
| Micall1 | 0.38605 | -0.71463 | 11273 | |
| Dnali1 | 0.38607 | -0.71609 | 11274 | |
| Hs3st1 | 0.38614 | 0.51028 | 11275 | |
| Arhgef5 | 0.38618 | -0.566 | 11276 | |
| Sec61a1 | 0.38624 | -0.1226 | 11277 | |
| Sv2b | 0.38625 | -0.035919 | 11278 | |
| H13 | 0.38627 | -0.57687 | 11279 | |
| Dph1 | 0.38627 | -0.72125 | 11280 | |
| Slc22a17 | 0.38631 | -0.33793 | 11281 | |
| Sema6b | 0.38645 | -0.7142 | 11282 | |
| Ctnnal1 | 0.38653 | -0.70263 | 11283 | |
| Aldoart2 | 0.38656 | -0.71716 | 11284 | |
| Prr19 | 0.38676 | -0.33608 | 11285 | |
| Olfr615 | 0.38678 | -0.59323 | 11286 | |
| Tgfb2 | 0.38684 | 0.15133 | 11287 | |
| Zmat3 | 0.38684 | 0.80627 | 11288 | |
| Obp2a | 0.38689 | -0.51529 | 11289 | |
| Olfr913 | 0.38693 | -0.73912 | 11290 | |
| Olfr257 | 0.387 | -0.68939 | 11291 | |
| Emc4 | 0.38709 | -0.2563 | 11292 | |
| Slc5a12 | 0.38722 | -0.71109 | 11293 | |
| 9930013L23Rik | 0.38722 | -0.71109 | 11294 | |
| Olfr1344 | 0.38726 | -0.66177 | 11295 | |
| Bpifb6 | 0.38729 | -0.73106 | 11296 | |
| Ccdc154 | 0.38734 | -0.71246 | 11297 | |
| mmu-mir-365-1 | 0.38735 | 0.52827 | 11298 | |
| Esp16 | 0.38736 | -0.71653 | 11299 | |
| Pomgnt1 | 0.38743 | -0.72064 | 11300 | |
| Zfp52 | 0.38747 | -0.71755 | 11301 | |
| Ccno | 0.38752 | -0.7118 | 11302 | |
| Cs | 0.38752 | 0.16075 | 11303 | |
| D630003M21Rik | 0.38754 | -0.72393 | 11304 | |
| Cyp27a1 | 0.38756 | -0.71809 | 11305 | |
| Meox1 | 0.38758 | -0.72102 | 11306 | |
| Cpq | 0.38765 | -0.12738 | 11307 | |
| BC051665 | 0.38767 | -0.49801 | 11308 | |
| Zfp811 | 0.38769 | -0.34069 | 11309 | |
| Ak1 | 0.38771 | 0.50924 | 11310 | |
| Nedd4l | 0.38776 | -0.60132 | 11311 | |
| Cdh15 | 0.38781 | -0.44365 | 11312 | |
| Prl3a1 | 0.38785 | -0.72408 | 11313 | |
| Gm1993 | 0.38785 | -0.79575 | 11314 | |
| Olfr916 | 0.3879 | 0.062937 | 11315 | |
| Gm1564 | 0.388 | -0.15156 | 11316 | |
| Fhod1 | 0.38804 | 0.039068 | 11317 | |
| Adam21 | 0.38812 | -0.72657 | 11318 | |
| 9030025P20Rik | 0.38829 | 0.5592 | 11319 | |
| Ascc3 | 0.38839 | -0.71881 | 11320 | |
| Olfr476 | 0.38849 | -0.71002 | 11321 | |
| Apobr | 0.38854 | -0.53558 | 11322 | |
| Wdr16 | 0.3886 | -0.44329 | 11323 | |
| Ttc7 | 0.38861 | -0.73369 | 11324 | |
| Nlgn3 | 0.38863 | -0.53023 | 11325 | |
| Clec4f | 0.3887 | 0.18282 | 11326 | |
| Ifi27l2b | 0.38873 | 1.728 | 11327 | |
| Dync1i1 | 0.38874 | -0.72041 | 11328 | |
| Olfr1252 | 0.38876 | -0.69859 | 11329 | |
| Vmn2r98 | 0.3888 | 0.021685 | 11330 | |
| Dnah2 | 0.38881 | -0.73016 | 11331 | |
| mmu-mir-6953 | 0.38886 | -0.45749 | 11332 | |
| Cdh4 | 0.3889 | -0.22161 | 11333 | |
| Fkbp7 | 0.38892 | -0.71493 | 11334 | |
| Clca5 | 0.38898 | 0.91844 | 11335 | |
| Duoxa1 | 0.38906 | -0.36823 | 11336 | |
| C2cd4d | 0.38911 | 0.045674 | 11337 | |
| Mks1 | 0.38921 | -0.71498 | 11338 | |
| Syndig1l | 0.38922 | -0.44316 | 11339 | |
| Vwde | 0.38926 | -0.01638 | 11340 | |
| Nrxn2 | 0.38929 | -0.28957 | 11341 | |
| Pdzd9 | 0.38929 | -0.60799 | 11342 | |
| Mcl1 | 0.38932 | -0.081456 | 11343 | |
| Lmna | 0.38934 | -0.72626 | 11344 | |
| St8sia1 | 0.38936 | -0.65653 | 11345 | |
| Hpca | 0.38942 | -0.030958 | 11346 | |
| Havcr2 | 0.38943 | -0.72726 | 11347 | |
| Lias | 0.38955 | 0.24052 | 11348 | |
| Tgif2 | 0.38956 | -0.4117 | 11349 | |
| Ibtk | 0.38968 | 1.3641 | 11350 | |
| Nop56 | 0.38974 | -0.52229 | 11351 | |
| Ago4 | 0.38978 | -0.71149 | 11352 | |
| Tppp | 0.38981 | -0.47839 | 11353 | |
| Slc16a6 | 0.38986 | -0.091137 | 11354 | |
| Defb13 | 0.38987 | -0.73238 | 11355 | |
| Ccr4 | 0.38998 | -0.71225 | 11356 | |
| Jade1 | 0.39001 | -0.059944 | 11357 | |
| Mitd1 | 0.39004 | -0.71934 | 11358 | |
| Gm904 | 0.39011 | -0.72584 | 11359 | |
| Sephs2 | 0.39011 | -0.72584 | 11360 | |
| Cdc20b | 0.39017 | 0.43773 | 11361 | |
| Mospd1 | 0.39021 | -0.48469 | 11362 | |
| mmu-mir-124-2 | 0.3903 | 0.69561 | 11363 | |
| Etv2 | 0.39036 | -0.70555 | 11364 | |
| Pafah1b2 | 0.39037 | 0.013517 | 11365 | |
| Cer1 | 0.39039 | -0.71912 | 11366 | |
| Zfp191 | 0.39043 | -0.37729 | 11367 | |
| Efhb | 0.39045 | -0.71357 | 11368 | |
| Olfr1039 | 0.39052 | -0.72991 | 11369 | |
| Olfr520 | 0.39057 | -0.10172 | 11370 | |
| Crybb3 | 0.39063 | -0.7199 | 11371 | |
| Drd2 | 0.39063 | -0.10599 | 11372 | |
| Defb25 | 0.39083 | 0.042183 | 11373 | |
| Strn3 | 0.39085 | -0.6656 | 11374 | |
| S100a7a | 0.39099 | -0.71831 | 11375 | |
| Hip1r | 0.39102 | 1.7118 | 11376 | |
| Gm3776 | 0.39115 | -0.28472 | 11377 | |
| Timm21 | 0.39118 | -0.5038 | 11378 | |
| Fgfr1op2 | 0.39122 | -0.49921 | 11379 | |
| Ccdc149 | 0.39129 | -0.054313 | 11380 | |
| Gm10024 | 0.39132 | -0.72077 | 11381 | |
| mmu-mir-8104 | 0.39132 | -0.38294 | 11382 | |
| 4921507P07Rik | 0.39133 | -0.58403 | 11383 | |
| Ptprj | 0.39143 | -0.44161 | 11384 | |
| Olfr1297 | 0.39146 | 1.8098 | 11385 | |
| Cldn10 | 0.39158 | -0.28445 | 11386 | |
| Slc12a1 | 0.39158 | -0.66625 | 11387 | |
| Glra3 | 0.39162 | -0.72332 | 11388 | |
| Kcp | 0.39178 | -0.10669 | 11389 | |
| Olfr1158 | 0.39181 | 0.28877 | 11390 | |
| Loxhd1 | 0.39184 | 0.040412 | 11391 | |
| Mrgprb2 | 0.39187 | 0.43923 | 11392 | |
| Apc2 | 0.39191 | 0.013459 | 11393 | |
| Prm3 | 0.39201 | -0.72109 | 11394 | |
| Vmn1r48 | 0.3921 | 0.13048 | 11395 | |
| Odf2 | 0.39212 | -0.30904 | 11396 | |
| Hrh3 | 0.39213 | 0.12261 | 11397 | |
| Slc35f4 | 0.39223 | -0.72143 | 11398 | |
| 2310007B03Rik | 0.39227 | -0.71631 | 11399 | |
| mmu-mir-1298 | 0.39227 | 2.2534 | 11400 | |
| Ttc9b | 0.3923 | -0.19457 | 11401 | |
| Proser2 | 0.39236 | -0.37964 | 11402 | |
| Ano7 | 0.39239 | -0.57378 | 11403 | |
| Gm17455 | 0.3924 | 2.0831 | 11404 | |
| Edem1 | 0.39243 | -0.39079 | 11405 | |
| Hyal1 | 0.39243 | -0.71873 | 11406 | |
| mmu-mir-7663 | 0.39246 | 0.63201 | 11407 | |
| Gm15056 | 0.39248 | -0.70511 | 11408 | |
| Rhbdf2 | 0.39261 | -0.69487 | 11409 | |
| Chst1 | 0.39262 | 0.31283 | 11410 | |
| Sypl2 | 0.39266 | -0.16527 | 11411 | |
| Il17rb | 0.39272 | -0.0081071 | 11412 | |
| Fcho1 | 0.39272 | -0.18455 | 11413 | |
| 1110025L11Rik | 0.39279 | 0.25108 | 11414 | |
| Tmem74b | 0.39288 | -0.7135 | 11415 | |
| Olfr591 | 0.39299 | -0.38362 | 11416 | |
| Cep95 | 0.39302 | -0.17071 | 11417 | |
| Abcg5 | 0.39311 | -0.45594 | 11418 | |
| Xpo1 | 0.39316 | 0.052231 | 11419 | |
| Zfp942 | 0.39319 | -0.65314 | 11420 | |
| Thsd4 | 0.39321 | 1.6081 | 11421 | |
| Ass1 | 0.39328 | -0.38361 | 11422 | |
| Kif22 | 0.39344 | -0.70466 | 11423 | |
| Vwa1 | 0.39344 | -0.34329 | 11424 | |
| Ptgds | 0.39355 | -0.70897 | 11425 | |
| Asgr1 | 0.39357 | 1.3034 | 11426 | |
| Prrt1 | 0.39358 | -0.72045 | 11427 | |
| Rbm41 | 0.39368 | -0.7127 | 11428 | |
| Clrn3 | 0.3937 | 0.31316 | 11429 | |
| Olfr1416 | 0.39375 | -0.53849 | 11430 | |
| Vax1 | 0.39382 | -0.6204 | 11431 | |
| Robo1 | 0.39384 | -0.7177 | 11432 | |
| Zfp617 | 0.39395 | -0.4968 | 11433 | |
| Pla2g1b | 0.39402 | -0.69539 | 11434 | |
| Cyp2f2 | 0.39403 | 1.7403 | 11435 | |
| Prrc2a | 0.39412 | 0.15172 | 11436 | |
| Pitpnm2 | 0.39417 | -0.66905 | 11437 | |
| Lrig2 | 0.39424 | -0.62986 | 11438 | |
| Ucma | 0.39435 | -0.69619 | 11439 | |
| Hist1h4i | 0.39435 | 0.030525 | 11440 | |
| Mybpc3 | 0.39445 | 0.22433 | 11441 | |
| Mtfmt | 0.39451 | -0.71775 | 11442 | |
| Clrn2 | 0.39453 | -0.69706 | 11443 | |
| Deb1 | 0.39471 | -0.71581 | 11444 | |
| Ttc22 | 0.39471 | 1.4396 | 11445 | |
| Ccdc175 | 0.39478 | -0.71961 | 11446 | |
| Tex9 | 0.3948 | -0.69402 | 11447 | |
| Fam71e1 | 0.39481 | -0.056136 | 11448 | |
| Sepp1 | 0.39484 | -0.27857 | 11449 | |
| Esyt2 | 0.39484 | -0.69799 | 11450 | |
| Gm4871 | 0.39494 | 0.52237 | 11451 | |
| Tex10 | 0.39497 | -0.63472 | 11452 | |
| Cant1 | 0.3952 | -0.6275 | 11453 | |
| Gprc5a | 0.39522 | -0.62482 | 11454 | |
| Mbd1 | 0.39525 | 0.27035 | 11455 | |
| Ankrd37 | 0.3953 | -0.31657 | 11456 | |
| Hoxb6 | 0.3954 | -0.53496 | 11457 | |
| C230052I12Rik | 0.39543 | -0.68913 | 11458 | |
| Hook1 | 0.39553 | 1.1812 | 11459 | |
| Klhdc10 | 0.39553 | -0.69657 | 11460 | |
| Cd52 | 0.39558 | -0.69609 | 11461 | |
| mmu-mir-33 | 0.39558 | -0.55743 | 11462 | |
| Prh1 | 0.3956 | -0.69586 | 11463 | |
| Spidr | 0.39562 | 0.38134 | 11464 | |
| Fgf9 | 0.39575 | 0.30787 | 11465 | |
| Nme3 | 0.39591 | -0.69235 | 11466 | |
| Golgb1 | 0.39592 | -0.68197 | 11467 | |
| Prdx5 | 0.39605 | 0.60139 | 11468 | |
| Tmem106a | 0.39629 | 0.083029 | 11469 | |
| Olfr54 | 0.39629 | 0.17495 | 11470 | |
| 2010001E11Rik | 0.39632 | -0.69253 | 11471 | |
| Vav1 | 0.3964 | 1.2228 | 11472 | |
| Egfl6 | 0.39656 | -0.69236 | 11473 | |
| Myf5 | 0.39658 | -0.2756 | 11474 | |
| Phldb3 | 0.3966 | 0.66268 | 11475 | |
| Nyap1 | 0.39661 | -0.6947 | 11476 | |
| Cebpz | 0.39663 | -0.70716 | 11477 | |
| Serpinb9 | 0.39665 | -0.71485 | 11478 | |
| Cast | 0.39667 | -0.70488 | 11479 | |
| Gnl3l | 0.39675 | -0.70782 | 11480 | |
| Slc25a12 | 0.39675 | -0.70782 | 11481 | |
| Cst6 | 0.39676 | -0.11494 | 11482 | |
| Gpr180 | 0.39717 | 2.4894 | 11483 | |
| Rbm17 | 0.39719 | -0.71078 | 11484 | |
| Brpf1 | 0.39721 | -0.54712 | 11485 | |
| Inhbe | 0.39723 | -0.70264 | 11486 | |
| Cabin1 | 0.3973 | 0.97242 | 11487 | |
| Ago3 | 0.39734 | -0.68424 | 11488 | |
| Krtap4-1 | 0.39738 | -0.064765 | 11489 | |
| Fbxw21 | 0.3974 | -0.69776 | 11490 | |
| Pla2g16 | 0.39743 | -0.70966 | 11491 | |
| Tnik | 0.39746 | -0.69149 | 11492 | |
| Megf11 | 0.39746 | -0.15025 | 11493 | |
| Ifna5 | 0.39751 | 0.45245 | 11494 | |
| Mtf2 | 0.39752 | -0.70636 | 11495 | |
| Tas2r121 | 0.39754 | 0.041223 | 11496 | |
| Chrng | 0.39767 | -0.39416 | 11497 | |
| Igsf1 | 0.3977 | 0.49774 | 11498 | |
| Rnpep | 0.39772 | -0.68999 | 11499 | |
| Ythdf2 | 0.3978 | 0.53343 | 11500 | |
| Cldn25 | 0.39783 | -0.71081 | 11501 | |
| Kif9 | 0.39799 | -0.071433 | 11502 | |
| Nos2 | 0.39801 | -0.6951 | 11503 | |
| Saa4 | 0.39804 | 0.14441 | 11504 | |
| Apmap | 0.39807 | -0.70035 | 11505 | |
| Bmi1 | 0.39812 | -0.47009 | 11506 | |
| Nedd9 | 0.39812 | -0.26237 | 11507 | |
| Fam20b | 0.39815 | -0.69753 | 11508 | |
| Lonp2 | 0.39819 | -0.15589 | 11509 | |
| Hras | 0.39819 | -0.68975 | 11510 | |
| Zfp575 | 0.39821 | -0.53006 | 11511 | |
| Cdh16 | 0.39822 | 0.37567 | 11512 | |
| Acy3 | 0.39826 | -0.24898 | 11513 | |
| Zfp677 | 0.39829 | -0.27437 | 11514 | |
| Ing5 | 0.39841 | -0.6977 | 11515 | |
| Gatad2a | 0.39857 | -0.095594 | 11516 | |
| mmu-mir-1258 | 0.39866 | -0.048542 | 11517 | |
| Dusp8 | 0.39871 | -0.68568 | 11518 | |
| Adipoq | 0.39873 | -0.67446 | 11519 | |
| Pdzd3 | 0.39875 | -0.68651 | 11520 | |
| Cdcp2 | 0.39877 | -0.68223 | 11521 | |
| Rbm19 | 0.39881 | 0.11669 | 11522 | |
| Tmc3 | 0.39884 | 0.23262 | 11523 | |
| Mvb12a | 0.39886 | -0.7059 | 11524 | |
| Rrn3 | 0.39902 | -0.70569 | 11525 | |
| Lrrc3b | 0.39903 | -0.39112 | 11526 | |
| Tmem211 | 0.39908 | -0.70241 | 11527 | |
| Snx7 | 0.39915 | 0.37715 | 11528 | |
| Pnma1 | 0.39921 | -0.69168 | 11529 | |
| mmu-mir-539 | 0.39924 | 0.56656 | 11530 | |
| Olfr1324 | 0.39926 | -0.67493 | 11531 | |
| Espn | 0.39932 | -0.6934 | 11532 | |
| Dopey2 | 0.39932 | -0.2473 | 11533 | |
| Bid | 0.39937 | -0.69188 | 11534 | |
| Chdh | 0.39942 | -0.010954 | 11535 | |
| Arpc1b | 0.39945 | -0.705 | 11536 | |
| Irs3 | 0.39949 | -0.68665 | 11537 | |
| Cabp4 | 0.39951 | -0.52151 | 11538 | |
| D630039A03Rik | 0.39952 | -0.48641 | 11539 | |
| Slc25a53 | 0.39967 | -0.33485 | 11540 | |
| mmu-mir-7669 | 0.39968 | -0.3432 | 11541 | |
| Lyzl4 | 0.39975 | -0.69019 | 11542 | |
| Mup13 | 0.39978 | -0.80327 | 11543 | |
| Ehd1 | 0.39991 | -0.68203 | 11544 | |
| Neu1 | 0.39993 | -0.69186 | 11545 | |
| 2610002M06Rik | 0.39997 | -0.69535 | 11546 | |
| Sspn | 0.40004 | -0.39756 | 11547 | |
| Olfr329-ps | 0.40013 | -0.69338 | 11548 | |
| 1700037C18Rik | 0.40016 | -0.68229 | 11549 | |
| Tpm1 | 0.4002 | -0.54699 | 11550 | |
| Wrap53 | 0.40025 | -0.29448 | 11551 | |
| Foxp1 | 0.40029 | -0.67996 | 11552 | |
| Prr23a | 0.4003 | -0.67182 | 11553 | |
| F2rl3 | 0.40034 | -0.6402 | 11554 | |
| Rhox3f | 0.40038 | -0.43254 | 11555 | |
| Defb7 | 0.40039 | 1.3426 | 11556 | |
| Med13 | 0.4004 | -0.60896 | 11557 | |
| Slfn3 | 0.40049 | -0.59703 | 11558 | |
| mmu-mir-344h-1 | 0.40052 | -0.46703 | 11559 | |
| Bbs1 | 0.40056 | -0.3813 | 11560 | |
| Hint1 | 0.40059 | -0.44186 | 11561 | |
| Dbndd2 | 0.4006 | -0.24167 | 11562 | |
| Xbp1 | 0.40065 | -0.16768 | 11563 | |
| Samt3 | 0.4007 | -0.68232 | 11564 | |
| Dmtf1 | 0.40072 | 0.88034 | 11565 | |
| Lrp2bp | 0.40075 | 0.758 | 11566 | |
| Olfr1253 | 0.40078 | 0.20636 | 11567 | |
| Olfr108 | 0.4008 | -0.70268 | 11568 | |
| Marco | 0.40081 | 0.49919 | 11569 | |
| Fam110a | 0.40083 | -0.68623 | 11570 | |
| 1700094D03Rik | 0.40087 | -0.69369 | 11571 | |
| Dtna | 0.40088 | 0.20564 | 11572 | |
| Suclg2 | 0.40089 | -0.69044 | 11573 | |
| Map3k3 | 0.40092 | -0.69699 | 11574 | |
| Rcan2 | 0.40094 | 0.010373 | 11575 | |
| Cplx3 | 0.40104 | -0.12319 | 11576 | |
| Rnf25 | 0.40107 | 0.19219 | 11577 | |
| Wnt5b | 0.4011 | -0.69031 | 11578 | |
| Itga9 | 0.4011 | -0.16049 | 11579 | |
| Gm11435 | 0.40124 | -0.69179 | 11580 | |
| Wdr86 | 0.40127 | -0.34036 | 11581 | |
| Mgrn1 | 0.4013 | -0.42281 | 11582 | |
| Lcn11 | 0.40134 | -0.36103 | 11583 | |
| Leap2 | 0.40139 | -0.36623 | 11584 | |
| Actc1 | 0.40145 | -0.67775 | 11585 | |
| Otos | 0.40149 | 0.46398 | 11586 | |
| Zfp266 | 0.40156 | 0.45946 | 11587 | |
| A230050P20Rik | 0.40165 | 0.50253 | 11588 | |
| Ube2ql1 | 0.40165 | -0.032476 | 11589 | |
| Enho | 0.4017 | -0.69279 | 11590 | |
| Eif4ebp3 | 0.40175 | -0.27309 | 11591 | |
| Ghitm | 0.40177 | -0.65226 | 11592 | |
| Fancl | 0.40181 | -0.69813 | 11593 | |
| Gpsm3 | 0.40183 | -0.67845 | 11594 | |
| Prr7 | 0.40185 | 0.10543 | 11595 | |
| Cd28 | 0.40191 | -0.2682 | 11596 | |
| Ocel1 | 0.40195 | 0.82868 | 11597 | |
| P4hb | 0.40201 | 0.24501 | 11598 | |
| Rwdd2b | 0.40201 | -0.68815 | 11599 | |
| Plxna2 | 0.40204 | -0.025189 | 11600 | |
| Ripply2 | 0.40208 | -0.67755 | 11601 | |
| Mta3 | 0.40211 | -0.099609 | 11602 | |
| Pcgf2 | 0.40214 | 0.080445 | 11603 | |
| Myo1g | 0.40217 | -0.17087 | 11604 | |
| Klrb1b | 0.40233 | 0.68285 | 11605 | |
| Xylt1 | 0.40237 | 0.010526 | 11606 | |
| Thy1 | 0.40239 | -0.67867 | 11607 | |
| Cd24a | 0.40255 | -0.68494 | 11608 | |
| Rab1 | 0.40262 | 2.5028 | 11609 | |
| 4930430A15Rik | 0.40271 | -0.69419 | 11610 | |
| 4921509C19Rik | 0.40271 | -0.093229 | 11611 | |
| Rufy1 | 0.40272 | -0.47322 | 11612 | |
| Zp3 | 0.40285 | 0.056881 | 11613 | |
| Olfr1102 | 0.40286 | -0.69923 | 11614 | |
| Gem | 0.40295 | 0.3887 | 11615 | |
| Spata32 | 0.40297 | -0.6737 | 11616 | |
| Ikbkb | 0.40302 | -0.52143 | 11617 | |
| Vmn2r23 | 0.40309 | -0.68661 | 11618 | |
| Nxph1 | 0.40317 | -0.09483 | 11619 | |
| Tmem65 | 0.40324 | 0.58785 | 11620 | |
| Olfml2a | 0.40324 | -0.68786 | 11621 | |
| Ilkap | 0.40327 | -0.69163 | 11622 | |
| Mlc1 | 0.40329 | -0.5116 | 11623 | |
| Jag1 | 0.40337 | 0.81625 | 11624 | |
| Ulk1 | 0.40338 | -0.65665 | 11625 | |
| Sds | 0.40343 | -0.097541 | 11626 | |
| Dlgap2 | 0.40349 | -0.67723 | 11627 | |
| Arhgef18 | 0.40356 | -0.68453 | 11628 | |
| Myoz3 | 0.4036 | -0.68678 | 11629 | |
| Hunk | 0.40362 | 0.99917 | 11630 | |
| Krt6b | 0.40369 | -0.69451 | 11631 | |
| Zfp111 | 0.40375 | 0.053661 | 11632 | |
| Klra10 | 0.40376 | -0.67784 | 11633 | |
| Nova2 | 0.40378 | -0.33679 | 11634 | |
| Sorcs2 | 0.40385 | -0.66381 | 11635 | |
| Magea10 | 0.40395 | -0.63906 | 11636 | |
| mmu-mir-6539 | 0.40407 | 0.32742 | 11637 | |
| Eif4h | 0.40408 | 1.1916 | 11638 | |
| Cyp4a14 | 0.40409 | -0.6739 | 11639 | |
| Efhc1 | 0.40421 | -0.66343 | 11640 | |
| Slfn5 | 0.40424 | -0.26544 | 11641 | |
| Trim34a | 0.40425 | -0.69299 | 11642 | |
| Fignl1 | 0.40427 | -0.21516 | 11643 | |
| Adgb | 0.40427 | -0.68578 | 11644 | |
| Igfbp7 | 0.40437 | -0.46954 | 11645 | |
| Adam26b | 0.40445 | -0.69161 | 11646 | |
| Piezo2 | 0.40446 | 0.34401 | 11647 | |
| Fgf17 | 0.40449 | -0.26092 | 11648 | |
| Abhd10 | 0.4045 | -0.58784 | 11649 | |
| Omt2a | 0.40456 | -0.50044 | 11650 | |
| Hyal3 | 0.40459 | -0.33495 | 11651 | |
| Csmd3 | 0.40461 | -0.62225 | 11652 | |
| Gzmb | 0.40466 | 0.53412 | 11653 | |
| Car3 | 0.4047 | -0.6746 | 11654 | |
| Rbm12b2 | 0.40474 | -0.69187 | 11655 | |
| Knstrn | 0.40476 | -0.68144 | 11656 | |
| mmu-mir-665 | 0.40478 | -0.38234 | 11657 | |
| Krt35 | 0.40488 | -0.45131 | 11658 | |
| Ube2a | 0.4049 | -0.68991 | 11659 | |
| Rergl | 0.40497 | -0.69189 | 11660 | |
| Tmeff1 | 0.40498 | -0.19706 | 11661 | |
| B3gnt6 | 0.40499 | -0.67915 | 11662 | |
| Nudc | 0.40501 | -0.23593 | 11663 | |
| Cyp20a1 | 0.40507 | 0.29003 | 11664 | |
| Ccdc172 | 0.40512 | -0.69197 | 11665 | |
| Sun1 | 0.40514 | -0.45903 | 11666 | |
| Zfp706 | 0.40523 | -0.48391 | 11667 | |
| Clca3 | 0.40532 | -0.67079 | 11668 | |
| H2-Q10 | 0.40538 | 0.92174 | 11669 | |
| Paics | 0.40544 | -0.46766 | 11670 | |
| Rgs1 | 0.40548 | -0.68493 | 11671 | |
| Lmbr1l | 0.40552 | 0.57772 | 11672 | |
| Senp8 | 0.40555 | -0.6895 | 11673 | |
| Cyp4a10 | 0.40566 | -0.68582 | 11674 | |
| Adamtsl4 | 0.40573 | -0.27372 | 11675 | |
| Krt6a | 0.40577 | -0.69095 | 11676 | |
| Slc25a43 | 0.4062 | -0.65137 | 11677 | |
| Cyp3a11 | 0.40623 | -0.67346 | 11678 | |
| Smok3a | 0.40623 | 0.97771 | 11679 | |
| Pramef8 | 0.40638 | -0.66447 | 11680 | |
| Ppef2 | 0.40649 | 0.11661 | 11681 | |
| Olfr324 | 0.40652 | 0.35257 | 11682 | |
| Cpt1a | 0.40661 | -0.68384 | 11683 | |
| N4bp1 | 0.40662 | -0.28911 | 11684 | |
| Pdcl | 0.40671 | -0.027738 | 11685 | |
| Fam101a | 0.40675 | -0.31391 | 11686 | |
| Pcdha7 | 0.40675 | -0.76356 | 11687 | |
| Tor1aip2 | 0.40678 | -0.23728 | 11688 | |
| Shc1 | 0.40691 | -0.052338 | 11689 | |
| Tsc22d1 | 0.40696 | -0.43065 | 11690 | |
| Pmm2 | 0.40698 | -0.67782 | 11691 | |
| Gm609 | 0.407 | 0.47136 | 11692 | |
| Adam2 | 0.40704 | -0.67681 | 11693 | |
| Sema4g | 0.40712 | 0.10892 | 11694 | |
| Mau2 | 0.40712 | -0.67212 | 11695 | |
| Ascc1 | 0.40714 | -0.60775 | 11696 | |
| Olfr623 | 0.40716 | -0.2175 | 11697 | |
| Spink13 | 0.40726 | -0.47989 | 11698 | |
| Timd4 | 0.40731 | -0.66868 | 11699 | |
| Pla2g6 | 0.40731 | -0.64731 | 11700 | |
| Esp23 | 0.40731 | -0.18655 | 11701 | |
| Kpna1 | 0.40744 | -0.49469 | 11702 | |
| Olfr1240 | 0.40749 | 1.6604 | 11703 | |
| Got1 | 0.40755 | 0.94467 | 11704 | |
| Olfr815 | 0.40759 | -0.6794 | 11705 | |
| Frem1 | 0.40761 | 0.058814 | 11706 | |
| Cluh | 0.40762 | -0.67216 | 11707 | |
| Esrp2 | 0.40765 | 0.41974 | 11708 | |
| Plekhb2 | 0.40765 | -0.65527 | 11709 | |
| 2610002J02Rik | 0.40768 | -0.56507 | 11710 | |
| Fshb | 0.40772 | -0.66178 | 11711 | |
| Lipc | 0.40774 | 0.53598 | 11712 | |
| Mmp3 | 0.40781 | -0.279 | 11713 | |
| Prr5l | 0.4079 | -0.65014 | 11714 | |
| Dab1 | 0.40806 | 0.90967 | 11715 | |
| Svs1 | 0.4081 | -0.67201 | 11716 | |
| Als2cr11 | 0.40813 | 0.5578 | 11717 | |
| Creb3l4 | 0.40816 | 0.93227 | 11718 | |
| Sat2 | 0.40826 | 0.25793 | 11719 | |
| Tnfsf13b | 0.40828 | -0.66987 | 11720 | |
| Hdac1 | 0.4083 | -0.67197 | 11721 | |
| Rgs11 | 0.40832 | 0.60913 | 11722 | |
| Gdpd4 | 0.40848 | -0.67833 | 11723 | |
| 3110002H16Rik | 0.40855 | -0.34731 | 11724 | |
| Tia1 | 0.40855 | -0.6794 | 11725 | |
| Elk3 | 0.40858 | -0.037719 | 11726 | |
| Cpne4 | 0.40862 | -0.22465 | 11727 | |
| Ebpl | 0.40864 | -0.66896 | 11728 | |
| Zbtb45 | 0.40869 | -0.65453 | 11729 | |
| Lce1j | 0.40873 | -0.66964 | 11730 | |
| Ccdc34 | 0.40875 | -0.48658 | 11731 | |
| Atg4b | 0.40887 | 0.76175 | 11732 | |
| Mipol1 | 0.40893 | -0.67895 | 11733 | |
| R3hdml | 0.40899 | 0.85215 | 11734 | |
| Clpp | 0.409 | -0.6638 | 11735 | |
| Fam159a | 0.40903 | 0.20449 | 11736 | |
| Usp3 | 0.40906 | 0.84519 | 11737 | |
| Slc22a22 | 0.40912 | 0.24779 | 11738 | |
| Mysm1 | 0.40913 | -0.68148 | 11739 | |
| Mpzl1 | 0.4092 | -0.66123 | 11740 | |
| Acap2 | 0.40922 | -0.68068 | 11741 | |
| Brf2 | 0.40928 | 1.4401 | 11742 | |
| Ephx1 | 0.40931 | -0.59751 | 11743 | |
| Nrip3 | 0.40934 | -0.66543 | 11744 | |
| Cep63 | 0.40938 | -0.45646 | 11745 | |
| Tacc2 | 0.40943 | 0.5676 | 11746 | |
| Ncoa4 | 0.40945 | -0.47784 | 11747 | |
| S100a13 | 0.40947 | 0.63681 | 11748 | |
| Gng8 | 0.40948 | -0.68197 | 11749 | |
| Vmn1r47 | 0.40951 | 0.14922 | 11750 | |
| Prdx6b | 0.40951 | -0.66948 | 11751 | |
| Vmn1r195 | 0.40963 | -0.41549 | 11752 | |
| Sncg | 0.40967 | -0.66007 | 11753 | |
| mmu-mir-770 | 0.40973 | 0.21801 | 11754 | |
| Ccdc104 | 0.40974 | -0.44646 | 11755 | |
| Loxl1 | 0.40983 | 0.69489 | 11756 | |
| Tc2n | 0.40983 | -0.6749 | 11757 | |
| Elovl4 | 0.40986 | -0.66936 | 11758 | |
| Mkks | 0.40987 | -0.027256 | 11759 | |
| Lenep | 0.40999 | -0.63278 | 11760 | |
| Reg3b | 0.40999 | -0.54455 | 11761 | |
| Vmn2r49 | 0.41004 | -0.78892 | 11762 | |
| Wnk2 | 0.41019 | -0.55367 | 11763 | |
| 4930432K21Rik | 0.41019 | 0.14358 | 11764 | |
| Hmox2 | 0.41021 | -0.67848 | 11765 | |
| Rpgr | 0.41024 | -0.42626 | 11766 | |
| Hbb-b2 | 0.41031 | 0.25183 | 11767 | |
| Arl5a | 0.41037 | 0.91176 | 11768 | |
| Stk10 | 0.41039 | -0.62893 | 11769 | |
| Bmx | 0.41041 | -0.10495 | 11770 | |
| Mcfd2 | 0.41052 | -0.65631 | 11771 | |
| Asphd1 | 0.41055 | -0.65291 | 11772 | |
| Coq6 | 0.41059 | -0.60522 | 11773 | |
| Tmem72 | 0.41059 | -0.32898 | 11774 | |
| Vps13c | 0.41061 | -0.10872 | 11775 | |
| Olfr969 | 0.41066 | -0.67153 | 11776 | |
| Prokr1 | 0.41077 | -0.40419 | 11777 | |
| Kctd9 | 0.41079 | 0.59263 | 11778 | |
| Xxylt1 | 0.41088 | 0.15302 | 11779 | |
| 4833424O15Rik | 0.41091 | -0.65977 | 11780 | |
| Sftpa1 | 0.41094 | -0.66625 | 11781 | |
| Tnni3k | 0.41101 | -0.42933 | 11782 | |
| Vmn1r202 | 0.41106 | -0.36264 | 11783 | |
| Dpy30 | 0.41108 | -0.66284 | 11784 | |
| Olfr648 | 0.41111 | 1.9741 | 11785 | |
| Leng8 | 0.4112 | -0.26893 | 11786 | |
| Gm11545 | 0.41122 | -0.26323 | 11787 | |
| D930020B18Rik | 0.41124 | -0.6527 | 11788 | |
| Rasl11a | 0.4113 | 1.4192 | 11789 | |
| Nol8 | 0.41131 | -0.67762 | 11790 | |
| Gpr25 | 0.41133 | -0.64892 | 11791 | |
| Serpina10 | 0.41149 | -0.65786 | 11792 | |
| Acbd4 | 0.41151 | -0.65761 | 11793 | |
| Gm101 | 0.41157 | -0.037063 | 11794 | |
| mmu-mir-193a | 0.41157 | 0.93876 | 11795 | |
| Tcf19 | 0.41165 | -0.15052 | 11796 | |
| Fgf3 | 0.41171 | -0.64601 | 11797 | |
| Met | 0.41171 | 0.11478 | 11798 | |
| Zfp653 | 0.41176 | -0.39276 | 11799 | |
| Rbbp9 | 0.41187 | 0.046097 | 11800 | |
| BC117090 | 0.41192 | -0.65588 | 11801 | |
| Tlr5 | 0.41194 | -0.3985 | 11802 | |
| Iapp | 0.41198 | -0.65962 | 11803 | |
| Bcl2l15 | 0.41207 | -0.6396 | 11804 | |
| mmu-mir-7037 | 0.41209 | -0.3852 | 11805 | |
| Cyp2d10 | 0.4121 | 0.16034 | 11806 | |
| AA792892 | 0.41216 | -0.38347 | 11807 | |
| Pear1 | 0.41219 | -0.18999 | 11808 | |
| Gxylt2 | 0.41225 | -0.64957 | 11809 | |
| Cd38 | 0.41226 | -0.53586 | 11810 | |
| Il17re | 0.41227 | -0.66379 | 11811 | |
| Ocln | 0.41232 | -0.65293 | 11812 | |
| Plxdc1 | 0.41245 | 0.13494 | 11813 | |
| Rsph1 | 0.41251 | -0.2744 | 11814 | |
| Tacc1 | 0.41254 | -0.2682 | 11815 | |
| Prr5 | 0.41261 | -0.30639 | 11816 | |
| Nfe2l3 | 0.41261 | -0.64783 | 11817 | |
| Glipr2 | 0.41266 | -0.66841 | 11818 | |
| Olfr994 | 0.41266 | -0.66841 | 11819 | |
| Arhgap27 | 0.41284 | -0.65242 | 11820 | |
| Ankrd61 | 0.41286 | -0.65424 | 11821 | |
| Vmn2r28 | 0.41302 | 0.046611 | 11822 | |
| mmu-mir-6989 | 0.41304 | 0.60198 | 11823 | |
| Pld6 | 0.41328 | 0.02728 | 11824 | |
| Gm13277 | 0.41334 | -0.7348 | 11825 | |
| Itga10 | 0.41335 | -0.54857 | 11826 | |
| Cask | 0.41337 | 0.20725 | 11827 | |
| Ano1 | 0.41342 | -0.63903 | 11828 | |
| S100a3 | 0.41357 | -0.4614 | 11829 | |
| Ccdc171 | 0.41363 | 0.35874 | 11830 | |
| Ndufs7 | 0.41366 | -0.49512 | 11831 | |
| Gm13043 | 0.41368 | -0.51996 | 11832 | |
| G6b | 0.41372 | 0.10112 | 11833 | |
| Crybg3 | 0.41376 | -0.65543 | 11834 | |
| 4930563E22Rik | 0.41385 | -0.66698 | 11835 | |
| Erlin2 | 0.41392 | -0.03594 | 11836 | |
| Scrn1 | 0.41394 | -0.64962 | 11837 | |
| Mmrn2 | 0.41411 | -0.13887 | 11838 | |
| Ankrd53 | 0.41414 | -0.53721 | 11839 | |
| mmu-mir-133a-2 | 0.41416 | -0.28543 | 11840 | |
| Fam3c | 0.41417 | -0.30504 | 11841 | |
| Phactr3 | 0.41418 | -0.64373 | 11842 | |
| Cux2 | 0.4142 | -0.23543 | 11843 | |
| mmu-mir-6936 | 0.41422 | -0.10391 | 11844 | |
| Olfr1338 | 0.41423 | -0.50131 | 11845 | |
| Phldb1 | 0.41433 | 1.4892 | 11846 | |
| Olfr1406 | 0.41434 | -0.64765 | 11847 | |
| Oxr1 | 0.41438 | -0.66193 | 11848 | |
| Lamp5 | 0.41443 | -0.65361 | 11849 | |
| Ddx5 | 0.41452 | -0.18923 | 11850 | |
| Hoxa10 | 0.41454 | -0.66641 | 11851 | |
| Pecr | 0.41459 | 0.54324 | 11852 | |
| Ube2g1 | 0.41459 | -0.619 | 11853 | |
| Lemd3 | 0.41462 | 1.3496 | 11854 | |
| Sp140 | 0.41462 | -0.7662 | 11855 | |
| Sh3rf3 | 0.41468 | -0.64506 | 11856 | |
| Aldh3b1 | 0.41468 | -0.41717 | 11857 | |
| Pld4 | 0.41474 | -0.65033 | 11858 | |
| Nlrp14 | 0.41474 | -0.38545 | 11859 | |
| Slco2b1 | 0.41478 | -0.24038 | 11860 | |
| Unc79 | 0.41486 | -0.6049 | 11861 | |
| Odf4 | 0.4149 | -0.65831 | 11862 | |
| Gpr141 | 0.41497 | -0.093994 | 11863 | |
| Esyt1 | 0.41503 | 0.28829 | 11864 | |
| Doc2b | 0.41504 | -0.65786 | 11865 | |
| Klhdc3 | 0.41509 | 0.33515 | 11866 | |
| C77370 | 0.41513 | -0.64895 | 11867 | |
| Olfr1461 | 0.41519 | -0.64584 | 11868 | |
| Guf1 | 0.41522 | 0.74599 | 11869 | |
| Rnmtl1 | 0.41529 | -0.57547 | 11870 | |
| Ceacam15 | 0.41531 | -0.32221 | 11871 | |
| Cpsf7 | 0.41532 | 1.6681 | 11872 | |
| Dnah11 | 0.41538 | -0.18944 | 11873 | |
| Il17d | 0.4154 | -0.63895 | 11874 | |
| BC051628 | 0.41541 | -0.5116 | 11875 | |
| Rtca | 0.41542 | -0.45037 | 11876 | |
| Kif7 | 0.41553 | -0.65286 | 11877 | |
| Numbl | 0.41554 | 0.95728 | 11878 | |
| Abcd4 | 0.41558 | -0.61386 | 11879 | |
| Sarnp | 0.41564 | 0.90941 | 11880 | |
| Fam109a | 0.41567 | 0.40759 | 11881 | |
| Slc16a14 | 0.41571 | -0.63128 | 11882 | |
| Tmem80 | 0.41573 | -0.63941 | 11883 | |
| Lao1 | 0.41575 | -0.16216 | 11884 | |
| mmu-mir-7070 | 0.41584 | 1.2383 | 11885 | |
| Dnajc19 | 0.41589 | -0.66633 | 11886 | |
| Grp | 0.41591 | -0.65279 | 11887 | |
| Syne1 | 0.41594 | -0.41247 | 11888 | |
| Fgf11 | 0.41594 | 0.88451 | 11889 | |
| Hba-a1 | 0.41596 | -0.6602 | 11890 | |
| Phtf1 | 0.41598 | -0.46379 | 11891 | |
| Kremen2 | 0.41601 | -0.63979 | 11892 | |
| Olfr550 | 0.41605 | 0.32052 | 11893 | |
| Izumo4 | 0.41611 | 0.74078 | 11894 | |
| Glra1 | 0.41611 | -0.65658 | 11895 | |
| Ssxb9 | 0.41615 | -0.8226 | 11896 | |
| Ints12 | 0.41627 | 0.92018 | 11897 | |
| Smtnl1 | 0.41638 | -0.66172 | 11898 | |
| Ccdc126 | 0.41645 | -0.49649 | 11899 | |
| 8030423J24Rik | 0.41649 | 0.54173 | 11900 | |
| Olfr169 | 0.41661 | 0.47767 | 11901 | |
| Krtap3-1 | 0.41663 | -0.6402 | 11902 | |
| Gas2l1 | 0.41669 | -0.44528 | 11903 | |
| Uqcrfs1 | 0.41672 | -0.63072 | 11904 | |
| Ppl | 0.41679 | -0.65492 | 11905 | |
| Noa1 | 0.41691 | 0.038702 | 11906 | |
| Rpgrip1l | 0.41692 | -0.075886 | 11907 | |
| mmu-mir-574 | 0.41694 | -0.33633 | 11908 | |
| Slc17a4 | 0.41706 | -0.6457 | 11909 | |
| Vmn1r50 | 0.41707 | -0.05452 | 11910 | |
| Secisbp2 | 0.41715 | 0.32537 | 11911 | |
| Dennd5b | 0.41715 | -0.16279 | 11912 | |
| Sgk2 | 0.41719 | -0.64831 | 11913 | |
| Ccser1 | 0.41735 | -0.41366 | 11914 | |
| Myh7 | 0.41737 | -0.64309 | 11915 | |
| Trdmt1 | 0.41749 | -0.64625 | 11916 | |
| Cisd3 | 0.41778 | -0.65498 | 11917 | |
| Tnfrsf12a | 0.41787 | -0.63171 | 11918 | |
| Slc6a6 | 0.41797 | -0.64542 | 11919 | |
| Cyth2 | 0.41808 | -0.64049 | 11920 | |
| Ncoa6 | 0.4182 | -0.36376 | 11921 | |
| Zc3h11a | 0.41821 | -0.006621 | 11922 | |
| Cyb5r3 | 0.41829 | -0.62766 | 11923 | |
| Nrp | 0.4183 | 0.087321 | 11924 | |
| Fam46d | 0.41834 | -0.17775 | 11925 | |
| Xcr1 | 0.41834 | -0.61544 | 11926 | |
| Prg2 | 0.41836 | -0.65088 | 11927 | |
| Gal3st2 | 0.41838 | -0.6279 | 11928 | |
| Rpusd1 | 0.41843 | 0.60409 | 11929 | |
| Impg2 | 0.41853 | -0.64951 | 11930 | |
| Agxt2 | 0.41862 | -0.19856 | 11931 | |
| Fam210b | 0.41863 | -0.63402 | 11932 | |
| Ntan1 | 0.4187 | -0.61693 | 11933 | |
| Olfr1054 | 0.41874 | -0.64775 | 11934 | |
| Abcc6 | 0.41875 | 0.556 | 11935 | |
| 2610318N02Rik | 0.41877 | -0.57504 | 11936 | |
| Styk1 | 0.41883 | -0.52324 | 11937 | |
| Fam53a | 0.41887 | -0.048152 | 11938 | |
| Kif27 | 0.41889 | -0.6437 | 11939 | |
| Map2k7 | 0.41892 | -0.36071 | 11940 | |
| Cep110 | 0.419 | 0.46767 | 11941 | |
| Mapre3 | 0.41901 | -0.65888 | 11942 | |
| Gfra4 | 0.4191 | 0.063272 | 11943 | |
| Prkaa1 | 0.41919 | -0.13376 | 11944 | |
| Kansl3 | 0.41922 | -0.63937 | 11945 | |
| Sptlc1 | 0.41924 | -0.31447 | 11946 | |
| Eras | 0.41926 | -0.62398 | 11947 | |
| Ankrd29 | 0.41932 | 0.2844 | 11948 | |
| Prss22 | 0.41935 | -0.53612 | 11949 | |
| Slc25a38 | 0.41937 | -0.62481 | 11950 | |
| Ntn3 | 0.41938 | 0.2288 | 11951 | |
| Prkx | 0.41941 | 0.015619 | 11952 | |
| Trpv3 | 0.41951 | -0.63126 | 11953 | |
| Arpp21 | 0.41955 | -0.62595 | 11954 | |
| Cep68 | 0.4196 | -0.64545 | 11955 | |
| Ttll8 | 0.41973 | 1.3413 | 11956 | |
| Il10ra | 0.41973 | -0.647 | 11957 | |
| Skap2 | 0.41978 | -0.65019 | 11958 | |
| Gm14137 | 0.41979 | 0.88401 | 11959 | |
| Fat2 | 0.41983 | -0.27176 | 11960 | |
| 1700022P22Rik | 0.41985 | -0.63692 | 11961 | |
| 4930453H23Rik | 0.41989 | -0.37713 | 11962 | |
| Clcn4-2 | 0.41991 | -0.62461 | 11963 | |
| Ptprcap | 0.41994 | -0.6311 | 11964 | |
| Olfr1032 | 0.41998 | 1.0204 | 11965 | |
| Carkd | 0.42005 | 0.16334 | 11966 | |
| Tfcp2 | 0.42009 | -0.54201 | 11967 | |
| Olfr1431 | 0.42039 | -0.54948 | 11968 | |
| Aurkaip1 | 0.42043 | 0.16478 | 11969 | |
| Dnmt3b | 0.42046 | 0.34815 | 11970 | |
| Vmn1r201 | 0.42063 | -0.62757 | 11971 | |
| Neurl1a | 0.42067 | -0.63318 | 11972 | |
| Gdf9 | 0.4207 | -0.39299 | 11973 | |
| Pip5k1a | 0.42071 | 1.5073 | 11974 | |
| Jmjd7 | 0.42075 | -0.6198 | 11975 | |
| Lnx1 | 0.42081 | 0.18453 | 11976 | |
| mmu-mir-6374 | 0.42081 | 0.24732 | 11977 | |
| Ahsa2 | 0.42083 | -0.64357 | 11978 | |
| Zc3hc1 | 0.42093 | -0.65471 | 11979 | |
| Cdc42ep3 | 0.42093 | -0.54698 | 11980 | |
| Eif2ak2 | 0.42096 | -0.64712 | 11981 | |
| Atoh8 | 0.42096 | 1.2204 | 11982 | |
| Olfr126 | 0.421 | 1.2143 | 11983 | |
| Ahr | 0.42103 | 0.6299 | 11984 | |
| Apol6 | 0.42103 | -0.63665 | 11985 | |
| Bglap3 | 0.42106 | -0.64014 | 11986 | |
| Pigyl | 0.42115 | -0.045665 | 11987 | |
| Gzme | 0.42125 | -0.29263 | 11988 | |
| Lrch1 | 0.42131 | -0.63176 | 11989 | |
| Fgfrl1 | 0.42131 | 1.2853 | 11990 | |
| Olfr1221 | 0.42134 | -0.24365 | 11991 | |
| Cypt15 | 0.42138 | -0.63531 | 11992 | |
| Tm4sf19 | 0.42144 | 1.4088 | 11993 | |
| mmu-mir-7086 | 0.42153 | -0.1358 | 11994 | |
| Calml4 | 0.42166 | 1.023 | 11995 | |
| Mtx2 | 0.42171 | -0.3264 | 11996 | |
| Khdrbs3 | 0.42176 | -0.63957 | 11997 | |
| Hapln1 | 0.42178 | -0.12827 | 11998 | |
| Nfkbia | 0.4218 | -0.48226 | 11999 | |
| Cd34 | 0.42185 | 0.13743 | 12000 | |
| Ezh2 | 0.42188 | -0.20439 | 12001 | |
| D430042O09Rik | 0.42194 | 0.073659 | 12002 | |
| 2210010C04Rik | 0.42207 | -0.40463 | 12003 | |
| Tmem132c | 0.4221 | -0.59457 | 12004 | |
| mmu-mir-196b | 0.42212 | -0.49785 | 12005 | |
| Hbb-bt | 0.42216 | -0.22131 | 12006 | |
| Defb3 | 0.42228 | -0.54255 | 12007 | |
| Kcng1 | 0.42232 | -0.17732 | 12008 | |
| Galnt2 | 0.42239 | -0.60985 | 12009 | |
| Vmn2r106 | 0.42243 | -0.65112 | 12010 | |
| Mafa | 0.42246 | -0.62552 | 12011 | |
| 9430016H08Rik | 0.42256 | -0.63226 | 12012 | |
| Olfr543 | 0.42261 | -0.27964 | 12013 | |
| Olfr1347 | 0.42264 | -0.62358 | 12014 | |
| Slc28a2 | 0.42264 | -0.17222 | 12015 | |
| Gtpbp2 | 0.42266 | -0.64452 | 12016 | |
| Yod1 | 0.42277 | -0.41837 | 12017 | |
| Kng2 | 0.42279 | -0.63243 | 12018 | |
| Lrch4 | 0.42286 | -0.048217 | 12019 | |
| Raet1c | 0.42291 | -0.58483 | 12020 | |
| Vmn1r223 | 0.42292 | 1.5658 | 12021 | |
| Mapre1 | 0.42298 | -0.64668 | 12022 | |
| Ap3s2 | 0.42302 | -0.64032 | 12023 | |
| Acer2 | 0.42307 | -0.096855 | 12024 | |
| Gm8994 | 0.42308 | -0.46111 | 12025 | |
| Slc35b2 | 0.42309 | -0.64911 | 12026 | |
| Ube2e3 | 0.42316 | -0.64764 | 12027 | |
| Gm14151 | 0.42321 | -0.51407 | 12028 | |
| mmu-mir-7682 | 0.42325 | -0.70284 | 12029 | |
| Cyp3a16 | 0.42329 | -0.53038 | 12030 | |
| Pole4 | 0.42336 | -0.63189 | 12031 | |
| Serpina3a | 0.42338 | -0.54794 | 12032 | |
| Kcnk4 | 0.42345 | -0.42921 | 12033 | |
| Slc7a4 | 0.42352 | -0.62356 | 12034 | |
| Kcng3 | 0.42354 | -0.62183 | 12035 | |
| Slc12a3 | 0.42355 | 0.56555 | 12036 | |
| mmu-mir-345 | 0.42365 | 0.36144 | 12037 | |
| Gper1 | 0.42365 | -0.58589 | 12038 | |
| Kcnk18 | 0.42371 | 0.1514 | 12039 | |
| Rassf4 | 0.42376 | -0.63604 | 12040 | |
| Olfr1002 | 0.42379 | -0.63883 | 12041 | |
| Zdhhc24 | 0.42381 | -0.21352 | 12042 | |
| mmu-mir-1912 | 0.42392 | -0.59321 | 12043 | |
| Mamld1 | 0.42393 | -0.62018 | 12044 | |
| Dzip1l | 0.42398 | -0.53274 | 12045 | |
| Padi2 | 0.42401 | -0.64027 | 12046 | |
| Cacng7 | 0.42403 | -0.62579 | 12047 | |
| Myh7b | 0.42406 | -0.058896 | 12048 | |
| Tmsb15b2 | 0.42406 | -0.65166 | 12049 | |
| Olfr624 | 0.42408 | -0.54166 | 12050 | |
| Gadd45a | 0.4241 | -0.64292 | 12051 | |
| Rrp1b | 0.42412 | 1.3098 | 12052 | |
| Nid1 | 0.42428 | -0.62279 | 12053 | |
| Olfr1157 | 0.42433 | -0.63804 | 12054 | |
| Blzf1 | 0.42439 | -0.57064 | 12055 | |
| Gramd3 | 0.4244 | 0.27814 | 12056 | |
| Ptpdc1 | 0.42444 | -0.47218 | 12057 | |
| Olfr649 | 0.42448 | -0.62696 | 12058 | |
| Ece2 | 0.42451 | -0.6285 | 12059 | |
| Slc17a5 | 0.42453 | 0.20185 | 12060 | |
| Gm6583 | 0.42455 | -0.012467 | 12061 | |
| BC017643 | 0.42467 | 0.81051 | 12062 | |
| Taf4a | 0.42469 | -0.63862 | 12063 | |
| B3gnt3 | 0.42472 | -0.52223 | 12064 | |
| Srsf4 | 0.42475 | -0.61624 | 12065 | |
| Tmem56 | 0.42481 | -0.59341 | 12066 | |
| Tlr6 | 0.42482 | -0.33744 | 12067 | |
| Zim1 | 0.42488 | -0.5645 | 12068 | |
| Slc13a3 | 0.42491 | -0.62441 | 12069 | |
| Atg16l2 | 0.42498 | -0.61908 | 12070 | |
| Sh2d1a | 0.42502 | -0.60533 | 12071 | |
| Npm2 | 0.42505 | -0.61807 | 12072 | |
| Zer1 | 0.42507 | -0.37715 | 12073 | |
| Csmd1 | 0.42511 | -0.62223 | 12074 | |
| Lyrm2 | 0.42525 | -0.17811 | 12075 | |
| Tk2 | 0.4253 | -0.31522 | 12076 | |
| Atf2 | 0.42534 | -0.3401 | 12077 | |
| Cyp2c40 | 0.42541 | 0.88281 | 12078 | |
| Smg8 | 0.42545 | -0.63145 | 12079 | |
| mmu-mir-679 | 0.42546 | 0.31253 | 12080 | |
| Gm3696 | 0.42547 | -0.716 | 12081 | |
| Myl4 | 0.42552 | -0.63398 | 12082 | |
| Slc4a10 | 0.42554 | -0.14149 | 12083 | |
| mmu-mir-7228 | 0.42556 | -0.59446 | 12084 | |
| Tmem161b | 0.42562 | -0.33696 | 12085 | |
| Xcl1 | 0.42575 | -0.62791 | 12086 | |
| Sema3f | 0.42579 | -0.063205 | 12087 | |
| Zfp428 | 0.42579 | -0.62574 | 12088 | |
| Cass4 | 0.42597 | -0.6279 | 12089 | |
| Ppap2a | 0.42604 | -0.61166 | 12090 | |
| Smyd3 | 0.42604 | -0.3423 | 12091 | |
| Haghl | 0.42626 | -0.60255 | 12092 | |
| Ranbp3 | 0.42632 | 0.68548 | 12093 | |
| Myt1 | 0.42638 | -0.61508 | 12094 | |
| Cldn18 | 0.42642 | 0.21744 | 12095 | |
| Sh3bp5l | 0.42645 | -0.085062 | 12096 | |
| Olfr774 | 0.42647 | -0.35659 | 12097 | |
| Olfr1056 | 0.4265 | -0.12469 | 12098 | |
| Bdh1 | 0.42657 | -0.61877 | 12099 | |
| Zmat2 | 0.42661 | 0.25884 | 12100 | |
| Kmt2c | 0.42665 | -0.61134 | 12101 | |
| Lpar3 | 0.42667 | 0.31863 | 12102 | |
| Tlr3 | 0.42673 | -0.37802 | 12103 | |
| St18 | 0.42674 | -0.60364 | 12104 | |
| Mtm1 | 0.42685 | -0.6023 | 12105 | |
| Tshb | 0.42692 | -0.63534 | 12106 | |
| Zfp408 | 0.42695 | -0.61293 | 12107 | |
| Mcee | 0.42699 | -0.61501 | 12108 | |
| Trat1 | 0.42713 | -0.61964 | 12109 | |
| Crmp1 | 0.42717 | -0.60212 | 12110 | |
| Lor | 0.42719 | -0.54815 | 12111 | |
| Egr3 | 0.42721 | -0.6057 | 12112 | |
| Cmpk1 | 0.42739 | -0.47172 | 12113 | |
| Ptges | 0.42741 | -0.63382 | 12114 | |
| Chd1 | 0.42748 | -0.61649 | 12115 | |
| Syt2 | 0.4275 | -0.29588 | 12116 | |
| Fbxl18 | 0.42752 | 0.40444 | 12117 | |
| Bzw2 | 0.42755 | -0.056721 | 12118 | |
| Zfp35 | 0.42755 | -0.087066 | 12119 | |
| Skint6 | 0.42758 | -0.096219 | 12120 | |
| Timp2 | 0.42768 | -0.51027 | 12121 | |
| Utrn | 0.4277 | -0.41236 | 12122 | |
| Olfr617 | 0.42775 | -0.62439 | 12123 | |
| Alad | 0.4278 | 0.70371 | 12124 | |
| Nr3c2 | 0.42782 | -0.62234 | 12125 | |
| Slamf6 | 0.42786 | 0.39812 | 12126 | |
| Cyb5r2 | 0.42789 | -0.13489 | 12127 | |
| Sppl2b | 0.42789 | -0.1479 | 12128 | |
| Tmem248 | 0.42792 | -0.34562 | 12129 | |
| Dcc | 0.42796 | 0.94483 | 12130 | |
| Olfr1094 | 0.42798 | -0.62872 | 12131 | |
| Pinlyp | 0.42816 | -0.61132 | 12132 | |
| Zmiz2 | 0.42818 | 0.18852 | 12133 | |
| Cpne7 | 0.42823 | -0.36468 | 12134 | |
| Tiam1 | 0.42824 | 0.81467 | 12135 | |
| Rai14 | 0.42825 | -0.56588 | 12136 | |
| Cep152 | 0.42836 | -0.61459 | 12137 | |
| Trappc8 | 0.42846 | -0.10053 | 12138 | |
| Cnksr1 | 0.42849 | -0.51836 | 12139 | |
| Slc8a3 | 0.42852 | -0.52508 | 12140 | |
| Tmem63b | 0.42874 | -0.42847 | 12141 | |
| 1810065E05Rik | 0.42877 | -0.46848 | 12142 | |
| Galnt18 | 0.4288 | 0.2846 | 12143 | |
| Acox1 | 0.42881 | -0.62123 | 12144 | |
| Aldh5a1 | 0.42883 | -0.61637 | 12145 | |
| Lhfp | 0.42893 | 0.58045 | 12146 | |
| Fzd4 | 0.42895 | -0.16386 | 12147 | |
| Batf3 | 0.42899 | -0.49841 | 12148 | |
| Cyth3 | 0.42902 | -0.0041492 | 12149 | |
| Nr1d1 | 0.42912 | -0.50431 | 12150 | |
| Csn1s1 | 0.42914 | -0.17729 | 12151 | |
| Cd163 | 0.42918 | 0.76981 | 12152 | |
| AI467606 | 0.42922 | -0.61164 | 12153 | |
| Cmtr2 | 0.42924 | -0.62505 | 12154 | |
| Khsrp | 0.42926 | -0.35743 | 12155 | |
| Ccnb2 | 0.42927 | -0.42326 | 12156 | |
| Tmem127 | 0.4293 | 0.1204 | 12157 | |
| Sp100 | 0.4294 | 0.82832 | 12158 | |
| Hsbp1l1 | 0.42944 | 0.41541 | 12159 | |
| C4b | 0.42951 | -0.61646 | 12160 | |
| Fut11 | 0.42956 | -0.59431 | 12161 | |
| Pla2g12b | 0.42959 | 0.11075 | 12162 | |
| Rasl2-9 | 0.42974 | -0.60658 | 12163 | |
| Aak1 | 0.42976 | 0.37133 | 12164 | |
| Plcb3 | 0.42976 | 0.52693 | 12165 | |
| Axl | 0.42978 | -0.59157 | 12166 | |
| Ccdc97 | 0.4298 | -0.1973 | 12167 | |
| Zscan26 | 0.42983 | -0.60741 | 12168 | |
| Kcnk9 | 0.42985 | -0.42448 | 12169 | |
| Pnpo | 0.42985 | 0.075179 | 12170 | |
| Capn3 | 0.42985 | -0.37137 | 12171 | |
| Fam166b | 0.42993 | -0.2935 | 12172 | |
| Bmp5 | 0.42998 | -0.59653 | 12173 | |
| Mettl7a1 | 0.43001 | -0.62446 | 12174 | |
| Vmn1r18 | 0.43001 | 1.5162 | 12175 | |
| 4933427G17Rik | 0.43005 | 0.64169 | 12176 | |
| Cpox | 0.43009 | 0.36801 | 12177 | |
| Prss23 | 0.43018 | 0.59228 | 12178 | |
| mmu-mir-139 | 0.43018 | 0.71424 | 12179 | |
| Mapre2 | 0.43019 | -0.62106 | 12180 | |
| Zfp668 | 0.43023 | -0.49839 | 12181 | |
| Ccdc109b | 0.43024 | -0.1254 | 12182 | |
| Olfr248 | 0.43026 | -0.58957 | 12183 | |
| Satb1 | 0.43027 | 0.11121 | 12184 | |
| Wnt10a | 0.43028 | -0.59674 | 12185 | |
| Enox2 | 0.4303 | -0.62032 | 12186 | |
| mmu-mir-669a-2 | 0.43039 | -0.56269 | 12187 | |
| Tas1r3 | 0.4305 | -0.28405 | 12188 | |
| Msrb3 | 0.43052 | 0.04543 | 12189 | |
| Rbm26 | 0.43053 | -0.60924 | 12190 | |
| Lcn12 | 0.43058 | -0.62006 | 12191 | |
| mmu-mir-1188 | 0.43062 | 0.49885 | 12192 | |
| Cdc25c | 0.43064 | -0.47943 | 12193 | |
| Ddx19b | 0.43077 | 0.50532 | 12194 | |
| Cideb | 0.43081 | -0.19877 | 12195 | |
| Pi15 | 0.43086 | -0.62451 | 12196 | |
| Prrx1 | 0.4309 | -0.52368 | 12197 | |
| Rnase10 | 0.43096 | 0.20823 | 12198 | |
| Tmem132e | 0.43102 | -0.098335 | 12199 | |
| Gpr160 | 0.43106 | 0.57329 | 12200 | |
| 4930596D02Rik | 0.43107 | -0.60966 | 12201 | |
| Obox6 | 0.43109 | -0.4319 | 12202 | |
| Gramd1b | 0.43111 | -0.59893 | 12203 | |
| 4930529M08Rik | 0.43127 | -0.40485 | 12204 | |
| Lrit2 | 0.43132 | -0.61181 | 12205 | |
| Vangl1 | 0.43141 | -0.47093 | 12206 | |
| mmu-mir-3081 | 0.43141 | 0.18084 | 12207 | |
| Actbl2 | 0.43146 | -0.16141 | 12208 | |
| Tgfa | 0.4315 | -0.61472 | 12209 | |
| Ptchd3 | 0.43152 | -0.60307 | 12210 | |
| Ppp1r15a | 0.4316 | 0.058825 | 12211 | |
| Pdlim7 | 0.43161 | -0.60924 | 12212 | |
| 1700084C01Rik | 0.43163 | -0.62143 | 12213 | |
| Steap1 | 0.43168 | -0.60739 | 12214 | |
| Pirt | 0.43171 | -0.47456 | 12215 | |
| Psmb11 | 0.43172 | -0.59893 | 12216 | |
| B9d2 | 0.43176 | 0.0059035 | 12217 | |
| mmu-mir-124-3 | 0.43177 | -0.56456 | 12218 | |
| Vmn1r76 | 0.4319 | -0.43575 | 12219 | |
| Rit1 | 0.43193 | -0.015635 | 12220 | |
| Krt39 | 0.43195 | -0.46302 | 12221 | |
| mmu-mir-6362 | 0.43201 | 0.11959 | 12222 | |
| Hapln4 | 0.43202 | -0.60043 | 12223 | |
| Tstd1 | 0.4321 | -0.19147 | 12224 | |
| Zfp872 | 0.43215 | -0.5815 | 12225 | |
| Trabd | 0.43215 | -0.61477 | 12226 | |
| mmu-mir-7243 | 0.43223 | 0.39139 | 12227 | |
| Glod4 | 0.4323 | -0.33024 | 12228 | |
| Usp20 | 0.43231 | -0.61067 | 12229 | |
| Lpcat2 | 0.43246 | -0.60978 | 12230 | |
| Ldb3 | 0.43249 | -0.59564 | 12231 | |
| Pigw | 0.43249 | -0.35301 | 12232 | |
| Arl5b | 0.43258 | -0.59748 | 12233 | |
| Aoc3 | 0.43271 | -0.61516 | 12234 | |
| Dna2 | 0.43276 | -0.57232 | 12235 | |
| Foxq1 | 0.43278 | -0.58543 | 12236 | |
| Proca1 | 0.43283 | -0.50566 | 12237 | |
| 4930451I11Rik | 0.4329 | -0.62257 | 12238 | |
| Fbxl14 | 0.43293 | 0.97782 | 12239 | |
| Prl2c3 | 0.43295 | -0.6822 | 12240 | |
| Xrcc6bp1 | 0.43296 | 0.89949 | 12241 | |
| Mad1l1 | 0.43303 | -0.53508 | 12242 | |
| Card9 | 0.43316 | 0.14708 | 12243 | |
| Edil3 | 0.43335 | -0.61797 | 12244 | |
| Slc25a39 | 0.43344 | -0.59538 | 12245 | |
| Calu | 0.43344 | 1.0447 | 12246 | |
| Csde1 | 0.43353 | -0.621 | 12247 | |
| Tmem45a | 0.43355 | -0.60986 | 12248 | |
| Isx | 0.43355 | 1.6585 | 12249 | |
| Zmym3 | 0.43358 | -0.54369 | 12250 | |
| Sephs1 | 0.43362 | -0.6083 | 12251 | |
| Ttc24 | 0.43366 | -0.61933 | 12252 | |
| Trp53i13 | 0.4338 | -0.59245 | 12253 | |
| Pknox2 | 0.43389 | -0.36596 | 12254 | |
| Zfp941 | 0.43393 | 0.68143 | 12255 | |
| Ubox5 | 0.43396 | -0.3203 | 12256 | |
| Clec2g | 0.43402 | 0.51905 | 12257 | |
| Hsf3 | 0.43405 | -0.42099 | 12258 | |
| Trio | 0.43414 | 0.63932 | 12259 | |
| Yy1 | 0.43423 | -0.60565 | 12260 | |
| Hcfc2 | 0.43426 | -0.60197 | 12261 | |
| Fam110c | 0.43436 | -0.46188 | 12262 | |
| Pgap3 | 0.43439 | -0.59917 | 12263 | |
| Klk1b4 | 0.43441 | -0.60031 | 12264 | |
| Bcl2 | 0.43443 | -0.61545 | 12265 | |
| Abcc10 | 0.43452 | 0.45615 | 12266 | |
| Catsper4 | 0.43458 | 0.0048042 | 12267 | |
| Gm216 | 0.43459 | -0.44042 | 12268 | |
| Mon1a | 0.43461 | 0.10461 | 12269 | |
| Olfr479 | 0.43464 | -0.071145 | 12270 | |
| Dbil5 | 0.43466 | -0.6131 | 12271 | |
| Mapk10 | 0.43474 | 0.47837 | 12272 | |
| Gpr176 | 0.43475 | -0.38472 | 12273 | |
| Cela2a | 0.43479 | -0.55201 | 12274 | |
| Tmem47 | 0.43483 | -0.093954 | 12275 | |
| Rgs7bp | 0.43484 | -0.61645 | 12276 | |
| Henmt1 | 0.43488 | -0.59983 | 12277 | |
| Tex38 | 0.435 | 1.131 | 12278 | |
| Plk5 | 0.43502 | -0.59437 | 12279 | |
| Mrgprh | 0.43505 | -0.46945 | 12280 | |
| Tubb4a | 0.43511 | -0.59464 | 12281 | |
| Nav1 | 0.43542 | -0.59484 | 12282 | |
| Gimap4 | 0.43547 | -0.59734 | 12283 | |
| Hist1h2ae | 0.43554 | -0.60801 | 12284 | |
| Gm17727 | 0.43558 | -0.5879 | 12285 | |
| Pank4 | 0.43563 | -0.59149 | 12286 | |
| Ldhal6b | 0.43567 | -0.07284 | 12287 | |
| Pcgf1 | 0.43571 | -0.59468 | 12288 | |
| Selenbp1 | 0.43579 | -0.29279 | 12289 | |
| Actg1 | 0.43581 | -0.34015 | 12290 | |
| Zfp473 | 0.43583 | -0.59529 | 12291 | |
| Nyap2 | 0.43585 | -0.59934 | 12292 | |
| Setd1b | 0.43589 | -0.40028 | 12293 | |
| Prdm11 | 0.43603 | 1.4 | 12294 | |
| Hmbs | 0.43607 | 0.34003 | 12295 | |
| Pdgfra | 0.43626 | -0.10222 | 12296 | |
| Tekt4 | 0.4364 | -0.58687 | 12297 | |
| Cldn4 | 0.43642 | -0.60561 | 12298 | |
| Lurap1l | 0.43644 | -0.59394 | 12299 | |
| Nr6a1 | 0.43655 | -0.30109 | 12300 | |
| Irf7 | 0.4366 | -0.50477 | 12301 | |
| Camp | 0.43666 | -0.60243 | 12302 | |
| Cacna1d | 0.43683 | -0.59246 | 12303 | |
| Fut8 | 0.43689 | -0.59136 | 12304 | |
| Pla2g2d | 0.43691 | -0.32831 | 12305 | |
| Scg5 | 0.43697 | -0.00039495 | 12306 | |
| Esp5 | 0.43707 | -0.29169 | 12307 | |
| Olfr566 | 0.43712 | -0.51169 | 12308 | |
| Th | 0.43718 | -0.59551 | 12309 | |
| Cdh12 | 0.43719 | -0.19084 | 12310 | |
| Eme1 | 0.43721 | -0.60927 | 12311 | |
| Ace2 | 0.43725 | -0.57476 | 12312 | |
| Golga7b | 0.43728 | -0.60709 | 12313 | |
| Gpr84 | 0.43737 | -0.57735 | 12314 | |
| Ccdc85a | 0.43738 | 0.33972 | 12315 | |
| Xrcc4 | 0.43741 | -0.59803 | 12316 | |
| Apof | 0.43744 | -0.37826 | 12317 | |
| Rfx1 | 0.43753 | -0.22319 | 12318 | |
| Mc3r | 0.43755 | -0.58552 | 12319 | |
| Tmem130 | 0.43762 | -0.61041 | 12320 | |
| Olfr1234 | 0.43764 | -0.59349 | 12321 | |
| Rnase9 | 0.43766 | -0.59579 | 12322 | |
| Clcn1 | 0.43767 | -0.017088 | 12323 | |
| Speg | 0.43778 | -0.010275 | 12324 | |
| Camkk2 | 0.43781 | -0.55953 | 12325 | |
| Fam149a | 0.43784 | -0.03018 | 12326 | |
| Dmrta1 | 0.43787 | -0.58906 | 12327 | |
| Papd4 | 0.43787 | 0.79535 | 12328 | |
| Naa40 | 0.43796 | -0.59947 | 12329 | |
| Ppef1 | 0.43797 | -0.15177 | 12330 | |
| Tgfbrap1 | 0.438 | -0.59482 | 12331 | |
| Bcl2a1c | 0.43806 | -0.60731 | 12332 | |
| Tas2r136 | 0.43806 | -0.60731 | 12333 | |
| Dbx2 | 0.43809 | -0.44151 | 12334 | |
| Cabyr | 0.43814 | -0.59867 | 12335 | |
| Mrap2 | 0.43816 | -0.5848 | 12336 | |
| Sidt1 | 0.43818 | 0.071979 | 12337 | |
| Ifi35 | 0.4382 | -0.21105 | 12338 | |
| 9430038I01Rik | 0.43827 | -0.56368 | 12339 | |
| Gcm1 | 0.43829 | -0.60108 | 12340 | |
| Tspan4 | 0.43832 | -0.5091 | 12341 | |
| Acot11 | 0.43834 | 0.27008 | 12342 | |
| Slc1a3 | 0.43834 | 0.3958 | 12343 | |
| Wdr70 | 0.43834 | -0.23918 | 12344 | |
| Bace1 | 0.43838 | -0.59711 | 12345 | |
| Lin7a | 0.43843 | -0.48906 | 12346 | |
| Ogfod3 | 0.43844 | -0.60457 | 12347 | |
| Hist1h1t | 0.43849 | 0.25274 | 12348 | |
| Sln | 0.43855 | -0.35615 | 12349 | |
| Pmm1 | 0.43861 | -0.56721 | 12350 | |
| Fam131b | 0.43862 | 0.65796 | 12351 | |
| Ppp1r16a | 0.43863 | -0.52111 | 12352 | |
| 4930404N11Rik | 0.43865 | 0.38733 | 12353 | |
| Tubd1 | 0.43867 | -0.59597 | 12354 | |
| Akap8 | 0.43868 | 1.1539 | 12355 | |
| mmu-mir-367 | 0.4387 | -0.42201 | 12356 | |
| Insrr | 0.43875 | -0.58745 | 12357 | |
| Cpt1b | 0.43881 | -0.57859 | 12358 | |
| Krt19 | 0.43884 | -0.5656 | 12359 | |
| Cks2 | 0.43886 | -0.57633 | 12360 | |
| Gm20854 | 0.43896 | 1.0486 | 12361 | |
| Mmp2 | 0.43898 | -0.59129 | 12362 | |
| Uncx | 0.43899 | 0.21343 | 12363 | |
| Cdk5 | 0.43902 | 0.34471 | 12364 | |
| Luzp4 | 0.43905 | -0.66532 | 12365 | |
| Tex33 | 0.43911 | -0.59653 | 12366 | |
| Epo | 0.43918 | -0.59049 | 12367 | |
| Myo7b | 0.43919 | 2.1189 | 12368 | |
| Lgi3 | 0.43922 | -0.58699 | 12369 | |
| Apbb3 | 0.43924 | -0.54151 | 12370 | |
| Zfp184 | 0.43931 | -0.22666 | 12371 | |
| Ctla2a | 0.4394 | -0.59422 | 12372 | |
| Spryd3 | 0.43942 | -0.15791 | 12373 | |
| Tbx6 | 0.43945 | 0.0068389 | 12374 | |
| Ahrr | 0.43951 | 0.13187 | 12375 | |
| Tsc2 | 0.43956 | -0.57752 | 12376 | |
| Jam3 | 0.43958 | 1.1798 | 12377 | |
| Atg4a | 0.43958 | -0.58775 | 12378 | |
| Ppp1r1a | 0.43967 | -0.48519 | 12379 | |
| Cdh2 | 0.43968 | -0.065264 | 12380 | |
| Rmnd5a | 0.4397 | -0.58404 | 12381 | |
| Usp12 | 0.43972 | -0.6028 | 12382 | |
| Ece1 | 0.43976 | -0.57434 | 12383 | |
| Lrp5 | 0.43992 | -0.39793 | 12384 | |
| Zfp524 | 0.43994 | -0.40513 | 12385 | |
| Cdc42ep4 | 0.43995 | -0.2547 | 12386 | |
| mmu-mir-7028 | 0.43995 | -0.29547 | 12387 | |
| Nipsnap3a | 0.44004 | 0.57038 | 12388 | |
| Pcbp2 | 0.44007 | 0.24789 | 12389 | |
| Angptl7 | 0.44019 | 0.37532 | 12390 | |
| Col4a5 | 0.44022 | -0.58073 | 12391 | |
| Elavl3 | 0.44024 | -0.43362 | 12392 | |
| Maged2 | 0.44028 | -0.34096 | 12393 | |
| Slc2a8 | 0.44029 | 0.20316 | 12394 | |
| 1700029I15Rik | 0.44034 | -0.58067 | 12395 | |
| Prdx3 | 0.44035 | -0.37506 | 12396 | |
| Crisp4 | 0.44037 | -0.16938 | 12397 | |
| Cyp2d26 | 0.44044 | 0.62847 | 12398 | |
| Snx2 | 0.44047 | 0.85323 | 12399 | |
| Dusp2 | 0.44057 | 0.39486 | 12400 | |
| Enoph1 | 0.44061 | 0.86703 | 12401 | |
| Tmed10 | 0.44061 | -0.59853 | 12402 | |
| Stk11ip | 0.44061 | -0.59853 | 12403 | |
| Cops7a | 0.44075 | 2.0629 | 12404 | |
| Gm13011 | 0.44078 | -0.56373 | 12405 | |
| Zfp566 | 0.44083 | 0.011429 | 12406 | |
| Bcar3 | 0.44085 | -0.59683 | 12407 | |
| Foxp2 | 0.44089 | -0.58846 | 12408 | |
| Htr2b | 0.44091 | -0.033205 | 12409 | |
| Kif3a | 0.44094 | 0.39221 | 12410 | |
| Ctsc | 0.441 | -0.5927 | 12411 | |
| D6Wsu163e | 0.44108 | -0.58316 | 12412 | |
| Ttbk2 | 0.44112 | -0.26963 | 12413 | |
| Ubqln3 | 0.44115 | -0.53776 | 12414 | |
| Tmco4 | 0.44126 | -0.56317 | 12415 | |
| Krt73 | 0.44128 | -0.54648 | 12416 | |
| Lyzl1 | 0.4413 | -0.17275 | 12417 | |
| Pde4a | 0.44132 | -0.57744 | 12418 | |
| mmu-mir-182 | 0.4414 | 0.8537 | 12419 | |
| Eya3 | 0.44157 | -0.58832 | 12420 | |
| Sgcd | 0.44165 | -0.32794 | 12421 | |
| Map6d1 | 0.44166 | -0.53393 | 12422 | |
| Gprin2 | 0.44177 | -0.12074 | 12423 | |
| Lin37 | 0.44186 | 0.64068 | 12424 | |
| Hn1l | 0.44195 | -0.095904 | 12425 | |
| Gphb5 | 0.44199 | 0.057259 | 12426 | |
| Olfr981 | 0.44201 | -0.57789 | 12427 | |
| Krtap21-1 | 0.44201 | -0.57789 | 12428 | |
| Appl1 | 0.44203 | -0.068149 | 12429 | |
| Pdlim1 | 0.44211 | -0.36877 | 12430 | |
| Ifnlr1 | 0.4422 | -0.29298 | 12431 | |
| Oas2 | 0.44221 | -0.57676 | 12432 | |
| Olfr1265 | 0.44226 | -0.53526 | 12433 | |
| H2-Eb2 | 0.44232 | -0.58464 | 12434 | |
| Nags | 0.44236 | -0.60052 | 12435 | |
| mmu-mir-434 | 0.44237 | -0.4447 | 12436 | |
| mmu-mir-205 | 0.44243 | -0.13769 | 12437 | |
| Kdelc2 | 0.44243 | -0.40519 | 12438 | |
| Dgcr6 | 0.44252 | -0.57753 | 12439 | |
| Vwf | 0.44257 | 0.088315 | 12440 | |
| Cyfip2 | 0.44266 | -0.599 | 12441 | |
| Fam86 | 0.44275 | -0.53011 | 12442 | |
| Foxk1 | 0.44279 | 0.55253 | 12443 | |
| mmu-mir-5106 | 0.44283 | -0.1927 | 12444 | |
| Rnf135 | 0.44286 | -0.58255 | 12445 | |
| Rasgrp4 | 0.44291 | 0.46099 | 12446 | |
| Chd6 | 0.44302 | -0.59221 | 12447 | |
| Ly6g5c | 0.44311 | -0.10739 | 12448 | |
| Ccm2 | 0.44316 | -0.51508 | 12449 | |
| Fxyd5 | 0.44319 | 1.1423 | 12450 | |
| 5830473C10Rik | 0.44334 | 0.76285 | 12451 | |
| Ap5b1 | 0.44341 | -0.58709 | 12452 | |
| Slc30a1 | 0.44343 | 0.43561 | 12453 | |
| Klrb1c | 0.44347 | -0.56772 | 12454 | |
| Ehhadh | 0.4435 | -0.57956 | 12455 | |
| Bsnd | 0.44353 | -0.53131 | 12456 | |
| Xrcc6 | 0.44356 | -0.57887 | 12457 | |
| Scamp3 | 0.44359 | -0.58287 | 12458 | |
| Cxcr6 | 0.44359 | 1.1997 | 12459 | |
| Bet1l | 0.44361 | -0.56638 | 12460 | |
| Nes | 0.44365 | -0.57069 | 12461 | |
| Fut2 | 0.44377 | -0.083006 | 12462 | |
| Tmc1 | 0.44378 | -0.58359 | 12463 | |
| Cnih2 | 0.44378 | -0.58359 | 12464 | |
| Abhd12 | 0.44391 | 0.050616 | 12465 | |
| Mal | 0.444 | -0.25169 | 12466 | |
| Map3k15 | 0.44408 | -0.20635 | 12467 | |
| Pde1a | 0.44408 | -0.55416 | 12468 | |
| Phf21a | 0.44414 | -0.41275 | 12469 | |
| Art5 | 0.44417 | -0.33092 | 12470 | |
| Trim35 | 0.44418 | -0.58761 | 12471 | |
| Tmem208 | 0.44424 | -0.5718 | 12472 | |
| Olfr1273-ps | 0.44439 | 0.47769 | 12473 | |
| Ndufa12 | 0.44442 | -0.55869 | 12474 | |
| Endou | 0.44448 | -0.076454 | 12475 | |
| Olfr666 | 0.44454 | -0.58836 | 12476 | |
| Vamp8 | 0.44454 | 2.0467 | 12477 | |
| Cyp4v3 | 0.44461 | -0.5762 | 12478 | |
| mmu-mir-6341 | 0.44461 | -0.23521 | 12479 | |
| Gdf11 | 0.44463 | -0.29899 | 12480 | |
| Olfr344 | 0.44465 | -0.41778 | 12481 | |
| Otud6b | 0.4447 | -0.34065 | 12482 | |
| Kif24 | 0.44473 | -0.08439 | 12483 | |
| Nap1l4 | 0.4448 | -0.097518 | 12484 | |
| Aarsd1 | 0.44481 | -0.5748 | 12485 | |
| Txnrd2 | 0.44488 | -0.15115 | 12486 | |
| Zfp455 | 0.44491 | 0.20733 | 12487 | |
| Gna15 | 0.44497 | 0.18016 | 12488 | |
| Rhox3c | 0.445 | -0.17284 | 12489 | |
| 1700012A03Rik | 0.44508 | -0.047224 | 12490 | |
| Pde6h | 0.4451 | -0.57811 | 12491 | |
| Ms4a10 | 0.44519 | 0.10223 | 12492 | |
| Plekha5 | 0.44525 | 0.068061 | 12493 | |
| Olfr318 | 0.4453 | -0.59472 | 12494 | |
| Klrd1 | 0.4454 | -0.23793 | 12495 | |
| Igfbp4 | 0.44542 | -0.56085 | 12496 | |
| Tmem55b | 0.44551 | -0.57817 | 12497 | |
| Zc3h12b | 0.44556 | -0.37639 | 12498 | |
| Gpr110 | 0.44558 | -0.52069 | 12499 | |
| Dsg2 | 0.4456 | -0.58492 | 12500 | |
| Csnk1g1 | 0.44562 | 0.73666 | 12501 | |
| Tmem8b | 0.44562 | -0.49126 | 12502 | |
| mmu-mir-681 | 0.44562 | -0.1158 | 12503 | |
| Prdm6 | 0.44565 | -0.58183 | 12504 | |
| Acn9 | 0.44565 | 0.085252 | 12505 | |
| Slc35e2 | 0.44571 | -0.069022 | 12506 | |
| mmu-mir-8108 | 0.44574 | -0.018635 | 12507 | |
| Dnah7b | 0.4458 | 0.30014 | 12508 | |
| Apbb1 | 0.44583 | -0.57913 | 12509 | |
| Olfr91 | 0.44583 | 0.55081 | 12510 | |
| Vmn1r35 | 0.44585 | -0.39451 | 12511 | |
| Zeb1 | 0.44599 | -0.57576 | 12512 | |
| Rhbdd1 | 0.44611 | -0.068739 | 12513 | |
| Slain1 | 0.44617 | 1.5579 | 12514 | |
| Gm1322 | 0.44628 | -0.59271 | 12515 | |
| Gm8765 | 0.44634 | -0.083433 | 12516 | |
| Mul1 | 0.44639 | -0.45632 | 12517 | |
| Fbxw14 | 0.44644 | -0.59133 | 12518 | |
| Arsb | 0.44648 | -0.56574 | 12519 | |
| Sirt2 | 0.44655 | 0.67642 | 12520 | |
| Fam174b | 0.44667 | -0.43151 | 12521 | |
| Rbbp8 | 0.44669 | -0.58257 | 12522 | |
| Aig1 | 0.44678 | -0.56882 | 12523 | |
| Cpa1 | 0.44678 | 0.12187 | 12524 | |
| Wdr37 | 0.44682 | -0.16186 | 12525 | |
| Arl6ip4 | 0.44685 | -0.12227 | 12526 | |
| Gata6 | 0.44687 | -0.5843 | 12527 | |
| Cypt7 | 0.44695 | -0.70695 | 12528 | |
| Mrpl50 | 0.44697 | -0.30113 | 12529 | |
| Dot1l | 0.447 | 1.0729 | 12530 | |
| Gm13718 | 0.447 | -0.56694 | 12531 | |
| Tsga8 | 0.44705 | -0.56957 | 12532 | |
| Pak1 | 0.44709 | 1.8028 | 12533 | |
| Otx1 | 0.4471 | -0.36762 | 12534 | |
| Uggt1 | 0.44712 | -0.57113 | 12535 | |
| Slmap | 0.44716 | -0.12796 | 12536 | |
| Cd226 | 0.44718 | -0.1215 | 12537 | |
| Hs6st1 | 0.44721 | -0.41629 | 12538 | |
| Ccndbp1 | 0.44738 | -0.5774 | 12539 | |
| Ces4a | 0.44743 | -0.034547 | 12540 | |
| Cadps | 0.44749 | -0.58733 | 12541 | |
| Fktn | 0.44753 | -0.074432 | 12542 | |
| Gucd1 | 0.44762 | -0.57312 | 12543 | |
| Ssr1 | 0.44764 | -0.55711 | 12544 | |
| Cobll1 | 0.44768 | -0.54888 | 12545 | |
| Arhgef3 | 0.44777 | 0.067176 | 12546 | |
| Bcat1 | 0.44782 | -0.58795 | 12547 | |
| Gm11757 | 0.44783 | -0.87455 | 12548 | |
| 1700074P13Rik | 0.44787 | -0.57763 | 12549 | |
| Coa5 | 0.44787 | 0.21203 | 12550 | |
| Clstn2 | 0.44789 | -0.51275 | 12551 | |
| Map7d2 | 0.44792 | -0.45242 | 12552 | |
| Dmpk | 0.44796 | -0.39239 | 12553 | |
| BC005764 | 0.44798 | -0.4791 | 12554 | |
| Ssbp3 | 0.44803 | -0.3846 | 12555 | |
| Tspyl3 | 0.44807 | -0.55971 | 12556 | |
| mmu-mir-6964 | 0.44808 | -0.30262 | 12557 | |
| Fryl | 0.44813 | -0.28739 | 12558 | |
| Slc26a2 | 0.44815 | -0.56587 | 12559 | |
| Nisch | 0.44821 | 0.085153 | 12560 | |
| Ncan | 0.44824 | -0.56498 | 12561 | |
| Tdh | 0.44826 | -0.55309 | 12562 | |
| Tldc1 | 0.44832 | 0.83612 | 12563 | |
| AW549877 | 0.44836 | -0.5818 | 12564 | |
| Mybpc1 | 0.44839 | -0.5726 | 12565 | |
| Rnf146 | 0.44839 | 0.25949 | 12566 | |
| Hey2 | 0.44843 | -0.21993 | 12567 | |
| Prickle3 | 0.44848 | -0.56676 | 12568 | |
| Cd72 | 0.4485 | 1.0891 | 12569 | |
| Atp2a1 | 0.44854 | -0.56893 | 12570 | |
| Ryr1 | 0.44861 | -0.55974 | 12571 | |
| Rangrf | 0.44862 | 0.052861 | 12572 | |
| Mapk7 | 0.44864 | -0.4973 | 12573 | |
| Serpine1 | 0.44879 | -0.56718 | 12574 | |
| Aph1a | 0.44882 | -0.10828 | 12575 | |
| Bmyc | 0.44884 | 0.0017747 | 12576 | |
| mmu-mir-6340 | 0.44885 | 0.026407 | 12577 | |
| Atad5 | 0.44905 | -0.1574 | 12578 | |
| 1700020D05Rik | 0.44908 | -0.57941 | 12579 | |
| Tbc1d22b | 0.44908 | -0.44226 | 12580 | |
| C6 | 0.44919 | -0.30221 | 12581 | |
| C1ra | 0.44919 | 1.1777 | 12582 | |
| Map3k11 | 0.44932 | -0.54836 | 12583 | |
| Meox2 | 0.44933 | 0.61189 | 12584 | |
| Nupr1 | 0.44936 | -0.21883 | 12585 | |
| Tmem221 | 0.44936 | -0.25595 | 12586 | |
| mmu-mir-6356 | 0.4494 | -0.49958 | 12587 | |
| Cyp2j12 | 0.4494 | -0.44042 | 12588 | |
| Tsn | 0.44941 | -0.56251 | 12589 | |
| Emc10 | 0.44954 | 0.084789 | 12590 | |
| Olfr313 | 0.44954 | -0.56089 | 12591 | |
| Pls1 | 0.44963 | -0.43564 | 12592 | |
| Zfp710 | 0.44964 | -0.57586 | 12593 | |
| Tas2r123 | 0.44964 | -0.57586 | 12594 | |
| Tmem116 | 0.44966 | -0.29321 | 12595 | |
| Draxin | 0.44968 | -0.55397 | 12596 | |
| Tmem37 | 0.44975 | 0.28563 | 12597 | |
| Peak1 | 0.44977 | -0.56136 | 12598 | |
| Tshz2 | 0.44981 | -0.54777 | 12599 | |
| Rad9b | 0.44982 | -0.086837 | 12600 | |
| Olfr272 | 0.44985 | -0.1645 | 12601 | |
| Olfr571 | 0.44997 | -0.32813 | 12602 | |
| Olfr978 | 0.45007 | -0.57046 | 12603 | |
| Grin3a | 0.45007 | -0.57046 | 12604 | |
| Lrp11 | 0.45011 | -0.57603 | 12605 | |
| Jrk | 0.45015 | 0.38421 | 12606 | |
| Vmn1r234 | 0.45015 | -0.20667 | 12607 | |
| Tyw1 | 0.45018 | -0.018688 | 12608 | |
| Nckap1l | 0.45019 | -0.58041 | 12609 | |
| Fam134a | 0.45024 | 0.69292 | 12610 | |
| Cd5l | 0.45032 | 0.13699 | 12611 | |
| Pigv | 0.45038 | -0.578 | 12612 | |
| Shfm1 | 0.4504 | -0.41524 | 12613 | |
| Ganc | 0.45043 | -0.56249 | 12614 | |
| Fpr3 | 0.45043 | -0.29679 | 12615 | |
| Pcdh10 | 0.45045 | -0.55679 | 12616 | |
| Trim13 | 0.45046 | -0.55773 | 12617 | |
| A230065H16Rik | 0.45055 | 1.1207 | 12618 | |
| Tcerg1l | 0.45061 | -0.43793 | 12619 | |
| Nup35 | 0.45064 | 0.054601 | 12620 | |
| 2700097O09Rik | 0.45067 | 0.22142 | 12621 | |
| Lyg2 | 0.45067 | -0.55222 | 12622 | |
| Ifna2 | 0.45073 | 1.5299 | 12623 | |
| Cct7 | 0.45105 | -0.52818 | 12624 | |
| Sf3a2 | 0.45105 | -0.5135 | 12625 | |
| Mrgpra2a | 0.45111 | -0.2334 | 12626 | |
| mmu-mir-5110 | 0.45111 | 0.0056811 | 12627 | |
| BC049352 | 0.45117 | -0.56067 | 12628 | |
| 4930558K02Rik | 0.45122 | -0.55038 | 12629 | |
| Lamtor4 | 0.45137 | -0.24431 | 12630 | |
| Epdr1 | 0.45138 | -0.53549 | 12631 | |
| A630033H20Rik | 0.4514 | 0.44095 | 12632 | |
| Bcorl1 | 0.45142 | -0.55963 | 12633 | |
| Actr3b | 0.45143 | 0.16311 | 12634 | |
| Usp26 | 0.45149 | -0.47852 | 12635 | |
| Ppp1r3a | 0.45158 | -0.55006 | 12636 | |
| Lysmd3 | 0.45168 | -0.56809 | 12637 | |
| Vmn1r91 | 0.45181 | -0.5781 | 12638 | |
| Prss51 | 0.45188 | -0.45749 | 12639 | |
| Tspan18 | 0.45201 | 0.37924 | 12640 | |
| Ccdc157 | 0.45206 | -0.55362 | 12641 | |
| Rgs5 | 0.45217 | -0.39448 | 12642 | |
| Zdhhc15 | 0.4522 | 0.42141 | 12643 | |
| 1700066M21Rik | 0.45233 | -0.57503 | 12644 | |
| Mrgprg | 0.45241 | 0.0072324 | 12645 | |
| Pacsin2 | 0.45249 | 0.16328 | 12646 | |
| Syt9 | 0.45249 | 0.18625 | 12647 | |
| Vit | 0.45249 | -0.54666 | 12648 | |
| mmu-mir-6408 | 0.45251 | -0.5675 | 12649 | |
| Ctsm | 0.45256 | -0.21828 | 12650 | |
| Gck | 0.45264 | -0.36654 | 12651 | |
| Slco4c1 | 0.45265 | -0.24275 | 12652 | |
| Mtmr14 | 0.45269 | -0.54733 | 12653 | |
| Lacc1 | 0.45271 | -0.32726 | 12654 | |
| Phldb2 | 0.45276 | -0.55806 | 12655 | |
| Htatip2 | 0.45277 | 0.11517 | 12656 | |
| Olfr628 | 0.45282 | -0.23967 | 12657 | |
| 6430550D23Rik | 0.45282 | -0.56101 | 12658 | |
| Ankrd9 | 0.45287 | 0.65985 | 12659 | |
| Sfta2 | 0.4529 | -0.23909 | 12660 | |
| Olfr661 | 0.45296 | -0.57021 | 12661 | |
| Ttl | 0.45305 | -0.019265 | 12662 | |
| Nsmf | 0.45308 | 0.27315 | 12663 | |
| Kif1b | 0.45312 | -0.17949 | 12664 | |
| Gimap8 | 0.45314 | -0.15404 | 12665 | |
| Xndr-trpc2 | 0.45324 | -0.54811 | 12666 | |
| Grm7 | 0.45326 | -0.30933 | 12667 | |
| Ngdn | 0.45329 | 0.317 | 12668 | |
| Surf2 | 0.4533 | -0.4195 | 12669 | |
| Tmem45b | 0.45333 | -0.37888 | 12670 | |
| Hoxd10 | 0.45347 | -0.50597 | 12671 | |
| Slc3a1 | 0.45355 | -0.21703 | 12672 | |
| Defb41 | 0.45367 | -0.56559 | 12673 | |
| S100g | 0.45371 | -0.54967 | 12674 | |
| Limd1 | 0.45373 | -0.56498 | 12675 | |
| Zfp352 | 0.4538 | -0.55734 | 12676 | |
| Fam189a1 | 0.45392 | -0.28715 | 12677 | |
| U2af1l4 | 0.45403 | -0.16192 | 12678 | |
| mmu-mir-7211 | 0.45404 | -0.56235 | 12679 | |
| Spag17 | 0.45405 | -0.56944 | 12680 | |
| Agpat3 | 0.45407 | -0.53225 | 12681 | |
| Slc22a5 | 0.4541 | -0.3627 | 12682 | |
| Olfr1426 | 0.4542 | 0.18284 | 12683 | |
| Mif | 0.45421 | -0.54743 | 12684 | |
| Rab33b | 0.45423 | -0.54687 | 12685 | |
| Gnal | 0.45432 | -0.55888 | 12686 | |
| Ppp1r12b | 0.45435 | -0.52766 | 12687 | |
| Athl1 | 0.45449 | 0.52857 | 12688 | |
| Lum | 0.45453 | -0.55206 | 12689 | |
| BC003965 | 0.45454 | -0.50273 | 12690 | |
| Grin1os | 0.45464 | -0.22235 | 12691 | |
| Hoxa6 | 0.45465 | 0.7808 | 12692 | |
| Cbx3 | 0.45469 | -0.56091 | 12693 | |
| Usp54 | 0.45472 | -0.37438 | 12694 | |
| Accsl | 0.45481 | -0.54787 | 12695 | |
| Bfar | 0.45481 | -0.54787 | 12696 | |
| Pcdh20 | 0.45484 | -0.12679 | 12697 | |
| Dkk2 | 0.45487 | -0.55685 | 12698 | |
| Mllt4 | 0.45496 | -0.56141 | 12699 | |
| Lrrn4cl | 0.455 | -0.53917 | 12700 | |
| Clvs2 | 0.45503 | -0.41909 | 12701 | |
| Olfr161 | 0.45503 | -0.56456 | 12702 | |
| Polk | 0.45506 | 0.58588 | 12703 | |
| Casp4 | 0.45508 | -0.56233 | 12704 | |
| Nt5c3 | 0.45513 | -0.41203 | 12705 | |
| Zdhhc13 | 0.45515 | -0.56337 | 12706 | |
| Il13 | 0.45515 | -0.56337 | 12707 | |
| Mansc1 | 0.45518 | -0.43674 | 12708 | |
| Ferd3l | 0.45523 | -0.27411 | 12709 | |
| Tmprss11bnl | 0.45526 | -0.57033 | 12710 | |
| Rbm43 | 0.45532 | -0.56891 | 12711 | |
| Wdr35 | 0.45533 | 0.059523 | 12712 | |
| Afap1 | 0.45541 | -0.31968 | 12713 | |
| Pgr | 0.45542 | -0.12675 | 12714 | |
| Ube2v2 | 0.45543 | -0.55451 | 12715 | |
| Olfr30 | 0.45551 | 0.42569 | 12716 | |
| Msgn1 | 0.45553 | -0.56115 | 12717 | |
| Olfr1305 | 0.45554 | -0.49961 | 12718 | |
| Olfr701 | 0.45555 | -0.42617 | 12719 | |
| Ctnnb1 | 0.4556 | 0.22575 | 12720 | |
| Myh13 | 0.45563 | 0.98591 | 12721 | |
| Olfr429 | 0.45566 | -0.55516 | 12722 | |
| Olfr181 | 0.45578 | 0.074959 | 12723 | |
| Lifr | 0.45584 | -0.34977 | 12724 | |
| Mre11a | 0.45592 | 1.0852 | 12725 | |
| Olfr53 | 0.45603 | -0.085802 | 12726 | |
| Tspyl2 | 0.45606 | 0.28701 | 12727 | |
| Prrt2 | 0.45607 | -0.54827 | 12728 | |
| Atad1 | 0.45609 | -0.53649 | 12729 | |
| mmu-mir-5124a | 0.4561 | -0.37069 | 12730 | |
| Pla2g4a | 0.45619 | -0.46091 | 12731 | |
| Glipr1 | 0.45623 | -0.54507 | 12732 | |
| Spag11b | 0.45634 | -0.090439 | 12733 | |
| Ldlrad2 | 0.45636 | -0.54201 | 12734 | |
| Pkp1 | 0.45638 | 1.0001 | 12735 | |
| Recql5 | 0.45641 | -0.12102 | 12736 | |
| Sap130 | 0.45643 | -0.55374 | 12737 | |
| Pard3b | 0.45653 | -0.56465 | 12738 | |
| Tbc1d10c | 0.45657 | 0.33508 | 12739 | |
| Tcte1 | 0.45661 | -0.37701 | 12740 | |
| Dda1 | 0.45663 | 0.59955 | 12741 | |
| Olfr348 | 0.45666 | -0.082724 | 12742 | |
| Sipa1l1 | 0.45669 | -0.25948 | 12743 | |
| Dcst1 | 0.45675 | -0.01746 | 12744 | |
| Tmem108 | 0.45684 | -0.54045 | 12745 | |
| Nmrk1 | 0.45686 | -0.4088 | 12746 | |
| Kif19a | 0.45692 | -0.078198 | 12747 | |
| Gm4340 | 0.45693 | -0.10262 | 12748 | |
| Ddhd2 | 0.45705 | -0.54913 | 12749 | |
| Arfip1 | 0.45706 | -0.47323 | 12750 | |
| Tnfrsf10b | 0.45707 | -0.4306 | 12751 | |
| Myo16 | 0.45709 | -0.52969 | 12752 | |
| Setd8 | 0.4572 | -0.47218 | 12753 | |
| Fgd6 | 0.45723 | -0.53261 | 12754 | |
| Cnga4 | 0.45732 | -0.47799 | 12755 | |
| Rnaseh2b | 0.45733 | 0.49299 | 12756 | |
| Slc35c1 | 0.4575 | -0.532 | 12757 | |
| Qrich2 | 0.45754 | -0.54247 | 12758 | |
| Smim6 | 0.45764 | -0.53755 | 12759 | |
| mmu-mir-654 | 0.45777 | 0.52888 | 12760 | |
| Pvrl1 | 0.45777 | -0.53998 | 12761 | |
| Zfp599 | 0.4578 | 0.53168 | 12762 | |
| Slc2a3 | 0.45784 | -0.55066 | 12763 | |
| Spock2 | 0.45784 | 0.12424 | 12764 | |
| Tbl1x | 0.45786 | -0.54132 | 12765 | |
| Kdelr2 | 0.45791 | -0.33719 | 12766 | |
| Muc5b | 0.45794 | 0.3648 | 12767 | |
| Slc25a27 | 0.458 | 0.37839 | 12768 | |
| Itga5 | 0.458 | -0.35801 | 12769 | |
| Tcp11 | 0.45802 | -0.53424 | 12770 | |
| Cdan1 | 0.45809 | 2.4147 | 12771 | |
| Nsun6 | 0.45815 | 0.33091 | 12772 | |
| Ccdc120 | 0.45818 | -0.43031 | 12773 | |
| Rhobtb2 | 0.45827 | -0.52976 | 12774 | |
| Avpr1b | 0.45832 | -0.53131 | 12775 | |
| Lypd5 | 0.45833 | 0.11935 | 12776 | |
| E430025E21Rik | 0.45838 | -0.55643 | 12777 | |
| mmu-mir-297c | 0.45839 | 1.1042 | 12778 | |
| Cdk5rap2 | 0.45845 | 0.56232 | 12779 | |
| Eri2 | 0.45851 | 0.055622 | 12780 | |
| Tex21 | 0.45857 | 2.089 | 12781 | |
| Spata22 | 0.45859 | -0.53292 | 12782 | |
| Cdx1 | 0.45863 | -0.26337 | 12783 | |
| Emc8 | 0.45866 | -0.51776 | 12784 | |
| Pcdh12 | 0.45868 | -0.5288 | 12785 | |
| Olfr92 | 0.4587 | -0.43759 | 12786 | |
| Tef | 0.45878 | -0.52891 | 12787 | |
| Rnf150 | 0.45881 | -0.33081 | 12788 | |
| Fam60a | 0.45884 | 0.045408 | 12789 | |
| Suds3 | 0.45888 | -0.55002 | 12790 | |
| Fam171a2 | 0.45893 | -0.548 | 12791 | |
| Gphn | 0.45902 | -0.3097 | 12792 | |
| Ube2c | 0.45905 | -0.20953 | 12793 | |
| Olfr519 | 0.45908 | 0.53345 | 12794 | |
| Tsga10 | 0.45911 | -0.10589 | 12795 | |
| Il17a | 0.45917 | 0.809 | 12796 | |
| Lrit1 | 0.4592 | -0.25764 | 12797 | |
| Isl2 | 0.45922 | -0.54044 | 12798 | |
| Vmn2r122 | 0.45924 | 1.6374 | 12799 | |
| Tas2r106 | 0.45925 | -0.51203 | 12800 | |
| Slc25a13 | 0.45931 | -0.55685 | 12801 | |
| Nxph4 | 0.45938 | -0.29137 | 12802 | |
| Fam211b | 0.4594 | -0.10646 | 12803 | |
| Fat3 | 0.45952 | -0.53797 | 12804 | |
| Myl12b | 0.45963 | -0.30892 | 12805 | |
| mmu-mir-1893 | 0.45963 | -0.46169 | 12806 | |
| Fam101b | 0.45968 | -0.51448 | 12807 | |
| Gm438 | 0.4597 | -0.13743 | 12808 | |
| Pdlim3 | 0.45975 | 0.29941 | 12809 | |
| Tmem52 | 0.45981 | -0.12654 | 12810 | |
| 1700021F05Rik | 0.45988 | -0.3033 | 12811 | |
| Adamts16 | 0.4599 | -0.30957 | 12812 | |
| Syn1 | 0.45993 | -0.52625 | 12813 | |
| Adam15 | 0.46004 | -0.5335 | 12814 | |
| Sult2a7 | 0.46018 | -0.54329 | 12815 | |
| Saysd1 | 0.4602 | -0.52757 | 12816 | |
| mmu-mir-99b | 0.46022 | 0.58416 | 12817 | |
| Nr1d2 | 0.46025 | -0.19123 | 12818 | |
| mmu-mir-3067 | 0.46027 | 0.38516 | 12819 | |
| Tesk1 | 0.46035 | -0.29371 | 12820 | |
| Dpp7 | 0.46038 | -0.48561 | 12821 | |
| Tubb6 | 0.46043 | -0.44413 | 12822 | |
| Zfp598 | 0.46047 | -0.21879 | 12823 | |
| Rhox12 | 0.46062 | 0.70951 | 12824 | |
| Ap4e1 | 0.46065 | -0.52927 | 12825 | |
| Spaca7 | 0.46072 | -0.53056 | 12826 | |
| Wdr53 | 0.46077 | -0.53666 | 12827 | |
| Olfr895 | 0.46087 | 0.40248 | 12828 | |
| Gpnmb | 0.46093 | -0.48833 | 12829 | |
| Plac8l1 | 0.46114 | 2.0287 | 12830 | |
| mmu-mir-615 | 0.46116 | -0.6093 | 12831 | |
| Cdh18 | 0.46117 | 0.45624 | 12832 | |
| Cbl | 0.4612 | -0.51452 | 12833 | |
| Sct | 0.46124 | -0.52803 | 12834 | |
| Abhd4 | 0.46127 | 0.75882 | 12835 | |
| Fras1 | 0.46129 | -0.51908 | 12836 | |
| Pdcd4 | 0.46131 | -0.53849 | 12837 | |
| Cklf | 0.46133 | -0.43042 | 12838 | |
| Crtac1 | 0.46135 | -0.40146 | 12839 | |
| Ces2e | 0.46141 | -0.54716 | 12840 | |
| Frem3 | 0.46145 | -0.54539 | 12841 | |
| Dip2c | 0.46152 | -0.5204 | 12842 | |
| Ppp1r13l | 0.46161 | -0.52432 | 12843 | |
| Rab14 | 0.46163 | -0.52288 | 12844 | |
| Svs4 | 0.46165 | 0.15504 | 12845 | |
| Hspbap1 | 0.4617 | -0.53364 | 12846 | |
| Il3ra | 0.46171 | -0.44541 | 12847 | |
| Ppp1r36 | 0.46173 | -0.54926 | 12848 | |
| Serpini2 | 0.46174 | -0.11314 | 12849 | |
| Akt3 | 0.46179 | -0.51697 | 12850 | |
| Sult2a4 | 0.46188 | -0.54645 | 12851 | |
| Galnt4 | 0.46192 | 0.29408 | 12852 | |
| Zkscan4 | 0.46192 | -0.52849 | 12853 | |
| Ampd1 | 0.46197 | -0.26605 | 12854 | |
| Gm5077 | 0.46204 | 1.4681 | 12855 | |
| Gm2022 | 0.46207 | 0.85786 | 12856 | |
| Alms1 | 0.46215 | -0.52296 | 12857 | |
| Cox17 | 0.46217 | -0.24562 | 12858 | |
| Olfr1163 | 0.46225 | 1.5405 | 12859 | |
| Olfr156 | 0.46232 | -0.52459 | 12860 | |
| Kmt2e | 0.4624 | 0.76918 | 12861 | |
| Chrnd | 0.46252 | 0.85253 | 12862 | |
| Ccdc85c | 0.46256 | -0.54113 | 12863 | |
| mmu-mir-5129 | 0.46262 | -0.30292 | 12864 | |
| Ngfr | 0.4627 | -0.52424 | 12865 | |
| 1700034J05Rik | 0.46274 | -0.25709 | 12866 | |
| Gsx2 | 0.4629 | -0.52301 | 12867 | |
| Htt | 0.46292 | -0.55106 | 12868 | |
| Spert | 0.463 | 0.51539 | 12869 | |
| mmu-mir-409 | 0.46305 | -0.38423 | 12870 | |
| Nifk | 0.46317 | -0.53647 | 12871 | |
| Scube1 | 0.46324 | 0.53972 | 12872 | |
| Ipo8 | 0.46326 | -0.51862 | 12873 | |
| Man1b1 | 0.46327 | -0.2352 | 12874 | |
| Dusp4 | 0.46329 | -0.52895 | 12875 | |
| Chrm5 | 0.46331 | -0.0029494 | 12876 | |
| S1pr5 | 0.46336 | 0.99675 | 12877 | |
| Gm364 | 0.4634 | -0.53726 | 12878 | |
| Tubb4b | 0.46342 | 0.46346 | 12879 | |
| Nkx2-3 | 0.46345 | -0.50709 | 12880 | |
| Zbtb12 | 0.46348 | -0.39753 | 12881 | |
| Nop16 | 0.46351 | -0.53973 | 12882 | |
| Fam71e2 | 0.46354 | -0.4229 | 12883 | |
| Dmbx1 | 0.46365 | -0.54499 | 12884 | |
| Hist1h3g | 0.46367 | -0.53518 | 12885 | |
| Dusp19 | 0.46369 | -0.53181 | 12886 | |
| Sltm | 0.46372 | 0.61905 | 12887 | |
| Prss27 | 0.46383 | -0.51891 | 12888 | |
| Chadl | 0.46387 | 0.35332 | 12889 | |
| Paqr5 | 0.4639 | 0.24028 | 12890 | |
| Itga7 | 0.46393 | -0.32287 | 12891 | |
| A430005L14Rik | 0.46394 | -0.5075 | 12892 | |
| Tmem169 | 0.46396 | -0.084004 | 12893 | |
| Rnase4 | 0.46397 | -0.524 | 12894 | |
| Ly6c2 | 0.46405 | -0.31575 | 12895 | |
| Lrrc26 | 0.46406 | -0.54752 | 12896 | |
| Kifap3 | 0.46408 | -0.54278 | 12897 | |
| Krt31 | 0.46408 | -0.12151 | 12898 | |
| S1pr2 | 0.4641 | -0.517 | 12899 | |
| Nr4a1 | 0.46411 | 0.28495 | 12900 | |
| Csl | 0.46413 | -0.51362 | 12901 | |
| Olfr488 | 0.46415 | -0.52826 | 12902 | |
| Olfr952 | 0.46417 | -0.031063 | 12903 | |
| Krt13 | 0.46424 | -0.4113 | 12904 | |
| Slc22a6 | 0.46429 | -0.51426 | 12905 | |
| Rxrb | 0.46439 | 0.24557 | 12906 | |
| mmu-mir-150 | 0.46439 | -0.20522 | 12907 | |
| Sit1 | 0.46442 | -0.024225 | 12908 | |
| Slc35b4 | 0.46442 | -0.53494 | 12909 | |
| Smco3 | 0.46447 | -0.53673 | 12910 | |
| Adap1 | 0.46456 | -0.41368 | 12911 | |
| Vmn1r42 | 0.46458 | -0.33023 | 12912 | |
| Cdhr3 | 0.4646 | -0.2074 | 12913 | |
| Myom2 | 0.4646 | -0.52145 | 12914 | |
| Loxl3 | 0.46463 | 1.6985 | 12915 | |
| Tssk3 | 0.46465 | -0.52963 | 12916 | |
| Tram1l1 | 0.46466 | 0.98931 | 12917 | |
| Nat14 | 0.46469 | 0.37834 | 12918 | |
| Igdcc4 | 0.46476 | -0.51744 | 12919 | |
| mmu-mir-7092 | 0.4649 | -0.53536 | 12920 | |
| 1700012P22Rik | 0.46491 | 0.69029 | 12921 | |
| Ltbp2 | 0.46499 | 0.86827 | 12922 | |
| Ttc38 | 0.46508 | -0.40387 | 12923 | |
| Ipo4 | 0.46519 | -0.52848 | 12924 | |
| Kctd19 | 0.46524 | -0.49384 | 12925 | |
| Mecom | 0.46531 | -0.51282 | 12926 | |
| Lysmd1 | 0.46538 | -0.29767 | 12927 | |
| Syne2 | 0.4654 | -0.53775 | 12928 | |
| Scarf1 | 0.46544 | -0.5107 | 12929 | |
| Adra1a | 0.46553 | -0.089537 | 12930 | |
| Slc37a3 | 0.46556 | -0.52902 | 12931 | |
| Krtap31-2 | 0.46558 | -0.51693 | 12932 | |
| Slc11a2 | 0.46559 | 0.27128 | 12933 | |
| Magea1 | 0.46567 | 0.85799 | 12934 | |
| Mfsd6l | 0.46574 | -0.41455 | 12935 | |
| Olfr750 | 0.46578 | 0.79833 | 12936 | |
| Ubl4a | 0.46583 | -0.50615 | 12937 | |
| Zfp760 | 0.46585 | -0.22605 | 12938 | |
| Grid2 | 0.46591 | -0.32885 | 12939 | |
| Ccdc66 | 0.46597 | 0.34453 | 12940 | |
| Klk15 | 0.46599 | -0.49766 | 12941 | |
| Tnip1 | 0.46601 | -0.53711 | 12942 | |
| Klhl36 | 0.46605 | 0.69888 | 12943 | |
| Tepp | 0.46612 | -0.51235 | 12944 | |
| Arhgap31 | 0.46618 | -0.034467 | 12945 | |
| Yae1d1 | 0.46621 | -0.38328 | 12946 | |
| Clip3 | 0.46624 | -0.46306 | 12947 | |
| Olfr242 | 0.4663 | 0.10723 | 12948 | |
| Cdk10 | 0.46636 | -0.52306 | 12949 | |
| 4930444G20Rik | 0.46636 | 0.5701 | 12950 | |
| Slc38a6 | 0.46649 | -0.52339 | 12951 | |
| Mapk4 | 0.46669 | -0.50009 | 12952 | |
| Cd209g | 0.46672 | 0.28126 | 12953 | |
| Zfp280b | 0.46674 | -0.50813 | 12954 | |
| Snap29 | 0.46676 | -0.49175 | 12955 | |
| Jam2 | 0.46681 | 1.1965 | 12956 | |
| Ambra1 | 0.46685 | -0.51435 | 12957 | |
| Rnf19a | 0.46687 | 0.34719 | 12958 | |
| Sypl | 0.46696 | -0.53605 | 12959 | |
| Champ1 | 0.46699 | -0.43869 | 12960 | |
| Oxgr1 | 0.46706 | -0.52293 | 12961 | |
| Npr2 | 0.46711 | -0.42687 | 12962 | |
| Itprip | 0.46712 | -0.45094 | 12963 | |
| Oas3 | 0.46714 | -0.28577 | 12964 | |
| mmu-mir-6903 | 0.46715 | 0.0090447 | 12965 | |
| BC037034 | 0.46719 | -0.51437 | 12966 | |
| Kctd21 | 0.4672 | 0.74608 | 12967 | |
| Gdf1 | 0.46721 | -0.50615 | 12968 | |
| Slc10a7 | 0.46724 | -0.5022 | 12969 | |
| Trim43a | 0.46726 | -0.056136 | 12970 | |
| Agt | 0.46732 | -0.090988 | 12971 | |
| Mgat4b | 0.46735 | -0.49709 | 12972 | |
| Dbh | 0.46746 | -0.52305 | 12973 | |
| Cyp2j9 | 0.46747 | -0.072181 | 12974 | |
| Parvg | 0.4675 | 0.72217 | 12975 | |
| Atp4a | 0.46753 | -0.12189 | 12976 | |
| Svip | 0.46762 | -0.11141 | 12977 | |
| Gm7694 | 0.46762 | -0.50936 | 12978 | |
| Vmn1r40 | 0.46765 | -0.50047 | 12979 | |
| Smpd3 | 0.46767 | -0.52811 | 12980 | |
| Galp | 0.46768 | -0.1041 | 12981 | |
| V1ra8 | 0.46769 | -0.50405 | 12982 | |
| Atic | 0.46773 | -0.52491 | 12983 | |
| Il1rapl2 | 0.46785 | -0.51314 | 12984 | |
| Atf6b | 0.46786 | 0.58965 | 12985 | |
| Fpgs | 0.4679 | 0.29443 | 12986 | |
| Cd8a | 0.46794 | -0.13015 | 12987 | |
| Dact2 | 0.46803 | -0.49193 | 12988 | |
| Ech1 | 0.46806 | -0.5143 | 12989 | |
| Lrfn1 | 0.46823 | 0.50348 | 12990 | |
| Olfr1153 | 0.46828 | -0.038697 | 12991 | |
| Fxyd1 | 0.46831 | 0.27439 | 12992 | |
| Ppfia2 | 0.46846 | -0.51763 | 12993 | |
| Oas1a | 0.46853 | -0.51982 | 12994 | |
| Smad2 | 0.46858 | 0.13713 | 12995 | |
| Slc22a20 | 0.46862 | -0.51199 | 12996 | |
| Smarcc2 | 0.46871 | -0.44923 | 12997 | |
| Dixdc1 | 0.46876 | -0.51336 | 12998 | |
| Krtap5-1 | 0.46881 | -0.50521 | 12999 | |
| Pcdh1 | 0.4689 | -0.51771 | 13000 | |
| Itga6 | 0.46899 | -0.53355 | 13001 | |
| mmu-mir-2861 | 0.46901 | -0.58301 | 13002 | |
| Cylc2 | 0.46903 | -0.46058 | 13003 | |
| Slc8a2 | 0.46905 | -0.16148 | 13004 | |
| Gid8 | 0.46908 | -0.28472 | 13005 | |
| Map2k1 | 0.4691 | 1.0039 | 13006 | |
| Cops8 | 0.4691 | -0.38654 | 13007 | |
| Dgka | 0.46915 | -0.17908 | 13008 | |
| Gpr108 | 0.46923 | -0.52381 | 13009 | |
| Sh3bgr | 0.46926 | -0.5303 | 13010 | |
| Rhou | 0.46929 | 0.57978 | 13011 | |
| Adra2b | 0.46929 | -0.53284 | 13012 | |
| Pcsk6 | 0.46932 | 0.99405 | 13013 | |
| Gm10318 | 0.46938 | 0.21929 | 13014 | |
| Ptafr | 0.46941 | -0.52612 | 13015 | |
| Naa50 | 0.46941 | -0.18485 | 13016 | |
| Poc1b | 0.46944 | -0.5054 | 13017 | |
| Sectm1b | 0.46969 | -0.50537 | 13018 | |
| Cstf2t | 0.4698 | -0.5036 | 13019 | |
| Ifi30 | 0.46986 | -0.0071318 | 13020 | |
| Sox7 | 0.46987 | -0.48975 | 13021 | |
| Tmem44 | 0.4699 | -0.49937 | 13022 | |
| Mcoln2 | 0.46992 | -0.0091436 | 13023 | |
| Kprp | 0.47003 | -0.49854 | 13024 | |
| Ugt2b1 | 0.47004 | -0.29991 | 13025 | |
| Exd1 | 0.47007 | -0.21706 | 13026 | |
| Olfr1362 | 0.47008 | -0.50498 | 13027 | |
| Olfr850 | 0.4701 | -0.50517 | 13028 | |
| Col15a1 | 0.47015 | -0.50858 | 13029 | |
| Foxh1 | 0.47016 | -0.024677 | 13030 | |
| Cd320 | 0.47017 | -0.50555 | 13031 | |
| Nt5c1b | 0.47019 | -0.51132 | 13032 | |
| Duox2 | 0.47024 | -0.51059 | 13033 | |
| 4930571K23Rik | 0.4703 | -0.49909 | 13034 | |
| Olfr888 | 0.47031 | 0.39487 | 13035 | |
| Chd9 | 0.47034 | -0.50711 | 13036 | |
| 6430571L13Rik | 0.47042 | -0.49775 | 13037 | |
| Cxcl10 | 0.47044 | 0.55237 | 13038 | |
| Gm10334 | 0.47049 | 1.2334 | 13039 | |
| Sctr | 0.4705 | -0.5119 | 13040 | |
| mmu-mir-294 | 0.47059 | -0.024935 | 13041 | |
| Mtdh | 0.47061 | 0.69911 | 13042 | |
| mmu-mir-93 | 0.47063 | -0.24638 | 13043 | |
| Lrrc10 | 0.47063 | 0.21469 | 13044 | |
| Ehbp1l1 | 0.47067 | -0.48786 | 13045 | |
| Dchs1 | 0.47068 | 0.46918 | 13046 | |
| Cabs1 | 0.47079 | -0.53044 | 13047 | |
| Pf4 | 0.47083 | -0.49823 | 13048 | |
| Cstl1 | 0.47087 | -0.52573 | 13049 | |
| Podn | 0.4709 | -0.50998 | 13050 | |
| Npy5r | 0.4709 | 0.56732 | 13051 | |
| Phf11d | 0.47097 | -0.50674 | 13052 | |
| Gm4763 | 0.47105 | 0.48273 | 13053 | |
| Ak8 | 0.47111 | -0.40565 | 13054 | |
| Cep135 | 0.47112 | -0.51354 | 13055 | |
| Dnaja1 | 0.47117 | -0.51325 | 13056 | |
| Cd82 | 0.47121 | -0.49299 | 13057 | |
| Prdm13 | 0.47124 | -0.48436 | 13058 | |
| Sc5d | 0.47126 | 0.99783 | 13059 | |
| Itgae | 0.4713 | 1.4395 | 13060 | |
| Ebf4 | 0.4713 | 0.42037 | 13061 | |
| Plvap | 0.47133 | -0.4904 | 13062 | |
| Pdia3 | 0.47144 | -0.52193 | 13063 | |
| BC030500 | 0.47153 | 0.30739 | 13064 | |
| Trmt1 | 0.47153 | -0.17841 | 13065 | |
| Bmpr1b | 0.47156 | -0.021953 | 13066 | |
| Atp5g3 | 0.4716 | -0.047764 | 13067 | |
| Tbrg1 | 0.47165 | -0.022321 | 13068 | |
| Treh | 0.47171 | 0.07453 | 13069 | |
| Gm4858 | 0.47174 | -0.45112 | 13070 | |
| Elovl3 | 0.47177 | -0.38198 | 13071 | |
| mmu-mir-7005 | 0.47178 | 0.59579 | 13072 | |
| Hmgn3 | 0.47191 | 0.090065 | 13073 | |
| Fut4 | 0.47193 | -0.51383 | 13074 | |
| Klhdc8a | 0.472 | 0.36378 | 13075 | |
| Lats1 | 0.47203 | -0.43378 | 13076 | |
| Kctd13 | 0.47203 | 1.9106 | 13077 | |
| Fam122a | 0.47206 | -0.058295 | 13078 | |
| Gcsam | 0.47217 | 0.14819 | 13079 | |
| Il1r1 | 0.47227 | -0.33853 | 13080 | |
| AI837181 | 0.4723 | -0.25914 | 13081 | |
| Acbd7 | 0.47233 | -0.39803 | 13082 | |
| Dach1 | 0.47236 | 1.8368 | 13083 | |
| Emilin3 | 0.4724 | -0.51484 | 13084 | |
| Fam19a3 | 0.47242 | -0.41594 | 13085 | |
| Tle1 | 0.47246 | -0.52655 | 13086 | |
| Stoml2 | 0.47248 | 0.37653 | 13087 | |
| Ghrhr | 0.47251 | -0.23854 | 13088 | |
| Il4i1 | 0.47251 | 0.47961 | 13089 | |
| Olfr338 | 0.47253 | -0.48949 | 13090 | |
| Sigmar1 | 0.47254 | 0.26653 | 13091 | |
| Rap1gds1 | 0.47255 | -0.49474 | 13092 | |
| mmu-mir-1249 | 0.47264 | 0.14478 | 13093 | |
| Asb14 | 0.47265 | -0.20856 | 13094 | |
| Pola2 | 0.47267 | -0.42538 | 13095 | |
| Sh3glb2 | 0.47273 | -0.46194 | 13096 | |
| 4930444P10Rik | 0.47274 | -0.077035 | 13097 | |
| Ythdf3 | 0.4728 | -0.49493 | 13098 | |
| Rgl3 | 0.47285 | -0.4924 | 13099 | |
| Zfand3 | 0.47287 | 0.2736 | 13100 | |
| Zfp36 | 0.47292 | -0.41914 | 13101 | |
| Tbx3 | 0.47296 | -0.30924 | 13102 | |
| H60c | 0.47301 | -0.055689 | 13103 | |
| Blmh | 0.47303 | -0.37491 | 13104 | |
| Arrdc3 | 0.47308 | -0.21991 | 13105 | |
| Vmn1r218 | 0.4731 | -0.45326 | 13106 | |
| Plekha8 | 0.4732 | -0.49924 | 13107 | |
| Olfr125 | 0.47326 | -0.49777 | 13108 | |
| Cradd | 0.47328 | -0.28273 | 13109 | |
| Zfp438 | 0.47332 | -0.50672 | 13110 | |
| Npm3 | 0.47332 | -0.50672 | 13111 | |
| Dydc2 | 0.47335 | -0.012492 | 13112 | |
| mmu-mir-1892 | 0.47358 | 0.18986 | 13113 | |
| Mast4 | 0.47361 | -0.45755 | 13114 | |
| Cnn2 | 0.47367 | 0.045422 | 13115 | |
| P2ry12 | 0.47367 | -0.49551 | 13116 | |
| Wdr27 | 0.47373 | 0.037797 | 13117 | |
| Snapc4 | 0.47383 | 0.33251 | 13118 | |
| Hapln3 | 0.47387 | 0.44127 | 13119 | |
| Cat | 0.4739 | 0.7399 | 13120 | |
| Olfr742 | 0.47394 | -0.51758 | 13121 | |
| C1s | 0.47395 | 0.21319 | 13122 | |
| Ate1 | 0.47407 | 0.52038 | 13123 | |
| Drg1 | 0.47412 | -0.42324 | 13124 | |
| Prkaca | 0.47414 | -0.26239 | 13125 | |
| Defa24 | 0.47423 | -0.32936 | 13126 | |
| Gtpbp3 | 0.47426 | -0.48789 | 13127 | |
| Pnp2 | 0.4743 | -0.5003 | 13128 | |
| Pvrl2 | 0.47434 | -0.50721 | 13129 | |
| Plec | 0.47434 | -0.50721 | 13130 | |
| Olfr486 | 0.47436 | -0.30744 | 13131 | |
| Slc12a7 | 0.47446 | -0.25587 | 13132 | |
| Zfp92 | 0.47449 | -0.47477 | 13133 | |
| Myh15 | 0.47455 | -0.5218 | 13134 | |
| C2cd5 | 0.47458 | -0.4267 | 13135 | |
| Cyp2j13 | 0.47465 | -0.49257 | 13136 | |
| Vmn1r21 | 0.4747 | -0.50268 | 13137 | |
| Dntt | 0.47474 | -0.50027 | 13138 | |
| Abca12 | 0.47476 | -0.48479 | 13139 | |
| Eif2d | 0.47476 | 0.64358 | 13140 | |
| Cables1 | 0.4748 | -0.50271 | 13141 | |
| Morf4l2 | 0.47488 | 0.22894 | 13142 | |
| Cog7 | 0.475 | 0.77467 | 13143 | |
| Hyal2 | 0.47501 | -0.5016 | 13144 | |
| Neu4 | 0.47503 | 0.22045 | 13145 | |
| Hdac7 | 0.47503 | -0.5003 | 13146 | |
| Dsg3 | 0.47509 | -0.21451 | 13147 | |
| St6galnac1 | 0.47512 | -0.40571 | 13148 | |
| Slc25a32 | 0.47515 | -0.51022 | 13149 | |
| Zadh2 | 0.47524 | -0.48464 | 13150 | |
| Krtap7-1 | 0.47532 | -0.51506 | 13151 | |
| Psg18 | 0.47532 | -0.51506 | 13152 | |
| Nipa1 | 0.47542 | 0.04837 | 13153 | |
| Cnr2 | 0.47551 | -0.46324 | 13154 | |
| Htr1b | 0.47552 | -0.4287 | 13155 | |
| Slc17a6 | 0.47555 | -0.48665 | 13156 | |
| Kng1 | 0.47578 | -0.41171 | 13157 | |
| Gabra4 | 0.47579 | 0.031681 | 13158 | |
| Tac2 | 0.47585 | -0.4953 | 13159 | |
| Gpr35 | 0.47598 | 0.82006 | 13160 | |
| Dlg1 | 0.47601 | -0.49509 | 13161 | |
| Zbtb14 | 0.47639 | 0.065848 | 13162 | |
| Rnf121 | 0.47642 | -0.51046 | 13163 | |
| Morn1 | 0.47645 | -0.48852 | 13164 | |
| Jarid2 | 0.47649 | -0.26163 | 13165 | |
| Exog | 0.47651 | -0.47733 | 13166 | |
| Rhpn2 | 0.47653 | -0.49021 | 13167 | |
| Gm2799 | 0.47653 | -0.43689 | 13168 | |
| Socs4 | 0.47657 | 0.59114 | 13169 | |
| Hnf1a | 0.4766 | -0.49601 | 13170 | |
| mmu-mir-7023 | 0.47673 | -0.15299 | 13171 | |
| Slc25a44 | 0.47681 | -0.50208 | 13172 | |
| Sec31b | 0.47681 | 0.25099 | 13173 | |
| Lrrc73 | 0.47683 | -0.092876 | 13174 | |
| Vmn1r6 | 0.47685 | -0.46364 | 13175 | |
| Radil | 0.47687 | 0.25664 | 13176 | |
| Trim29 | 0.47692 | -0.06108 | 13177 | |
| Ppm1f | 0.47701 | 1.0762 | 13178 | |
| Nprl3 | 0.47706 | -0.48785 | 13179 | |
| Trim2 | 0.4772 | 0.6703 | 13180 | |
| Fbxo2 | 0.47721 | -0.48888 | 13181 | |
| Hdac2 | 0.47731 | -0.43143 | 13182 | |
| Dpysl3 | 0.47739 | -0.48532 | 13183 | |
| Ints3 | 0.47742 | -0.50153 | 13184 | |
| Il4 | 0.47746 | -0.48369 | 13185 | |
| Gas8 | 0.47749 | -0.49551 | 13186 | |
| Nr4a2 | 0.47751 | -0.50252 | 13187 | |
| Sertad3 | 0.47752 | 0.48129 | 13188 | |
| Pde6a | 0.47753 | -0.50344 | 13189 | |
| mmu-mir-3061 | 0.47763 | -0.47472 | 13190 | |
| 2200002D01Rik | 0.47765 | -0.38344 | 13191 | |
| Cox14 | 0.47769 | 0.10793 | 13192 | |
| Syt11 | 0.47787 | -0.47587 | 13193 | |
| Ethe1 | 0.4781 | -0.23345 | 13194 | |
| Olfr1288 | 0.47812 | -0.49058 | 13195 | |
| mmu-mir-7a-1 | 0.47814 | -0.479 | 13196 | |
| Gprc5b | 0.47815 | -0.4921 | 13197 | |
| Tmem198b | 0.47816 | 0.9018 | 13198 | |
| Bdkrb2 | 0.47824 | -0.41715 | 13199 | |
| Ptp4a2 | 0.47826 | -0.48382 | 13200 | |
| Epn3 | 0.47835 | -0.47565 | 13201 | |
| Ltk | 0.47852 | 0.4404 | 13202 | |
| Vmn2r5 | 0.47853 | -0.48429 | 13203 | |
| Clec4g | 0.47855 | -0.44795 | 13204 | |
| Kank2 | 0.47856 | -0.46806 | 13205 | |
| Bdp1 | 0.47858 | -0.37317 | 13206 | |
| Ppap2b | 0.4786 | -0.478 | 13207 | |
| Pappa | 0.47861 | -0.26071 | 13208 | |
| Pus10 | 0.47865 | -0.25477 | 13209 | |
| Gpr113 | 0.4787 | 0.023416 | 13210 | |
| Sept12 | 0.47887 | -0.48524 | 13211 | |
| Zfp708 | 0.47892 | -0.35359 | 13212 | |
| Gm9758 | 0.47899 | -0.35765 | 13213 | |
| St6gal2 | 0.47902 | -0.016637 | 13214 | |
| Arhgap8 | 0.47926 | -0.47527 | 13215 | |
| Soat1 | 0.47931 | -0.14213 | 13216 | |
| Pramef17 | 0.47935 | -0.4885 | 13217 | |
| Igdcc3 | 0.47937 | 0.25785 | 13218 | |
| Hmgb2 | 0.47949 | -0.49752 | 13219 | |
| Pigc | 0.47949 | 2.5723 | 13220 | |
| Dhrsx | 0.47951 | -0.46482 | 13221 | |
| Adam23 | 0.47956 | -0.48675 | 13222 | |
| D230025D16Rik | 0.47962 | 1.241 | 13223 | |
| Ndufb6 | 0.47965 | -0.48414 | 13224 | |
| Lamb3 | 0.4797 | -0.058506 | 13225 | |
| Jph1 | 0.47973 | 0.21934 | 13226 | |
| Olfr1013 | 0.47979 | 0.11452 | 13227 | |
| Grhl3 | 0.47981 | -0.41616 | 13228 | |
| Ltbp4 | 0.47988 | -0.46926 | 13229 | |
| Lmtk2 | 0.4799 | 0.1989 | 13230 | |
| Olfr1213 | 0.47991 | -0.4949 | 13231 | |
| Mlycd | 0.47995 | -0.25574 | 13232 | |
| 5830411N06Rik | 0.47999 | 0.15111 | 13233 | |
| Slc22a7 | 0.48008 | -0.49349 | 13234 | |
| Cenpw | 0.48017 | 0.4209 | 13235 | |
| Dgcr8 | 0.48022 | -0.45837 | 13236 | |
| Nufip1 | 0.48031 | -0.48954 | 13237 | |
| Ell2 | 0.48038 | -0.46354 | 13238 | |
| mmu-mir-5107 | 0.48039 | -0.14128 | 13239 | |
| Serpina1d | 0.4804 | -0.33274 | 13240 | |
| Pnliprp1 | 0.48042 | -0.46815 | 13241 | |
| Hlcs | 0.48044 | -0.41111 | 13242 | |
| Tcl1 | 0.48046 | 0.68084 | 13243 | |
| Acmsd | 0.48049 | -0.48627 | 13244 | |
| Pkd2l1 | 0.48051 | -0.48758 | 13245 | |
| Slc7a7 | 0.48058 | 1.4909 | 13246 | |
| Ang4 | 0.48061 | -0.45686 | 13247 | |
| Crybb2 | 0.48063 | -0.494 | 13248 | |
| Acoxl | 0.48065 | -0.49629 | 13249 | |
| Zbed3 | 0.48067 | -0.45202 | 13250 | |
| Lrrc7 | 0.48073 | 0.55383 | 13251 | |
| Olfr904 | 0.48079 | 2.4452 | 13252 | |
| 4930513O06Rik | 0.48081 | -0.48932 | 13253 | |
| Tnfaip8l2 | 0.48087 | 0.36096 | 13254 | |
| Sox13 | 0.4809 | 0.31129 | 13255 | |
| Pex10 | 0.48092 | -0.23239 | 13256 | |
| Bcl7a | 0.48097 | -0.45747 | 13257 | |
| Nkx3-2 | 0.48099 | -0.46562 | 13258 | |
| Med21 | 0.48105 | 0.0096221 | 13259 | |
| Rdh8 | 0.48114 | -0.50569 | 13260 | |
| Ppp1r18 | 0.48117 | -0.48845 | 13261 | |
| S100a14 | 0.48119 | -0.47286 | 13262 | |
| Zfp628 | 0.48126 | 1.4979 | 13263 | |
| Gm614 | 0.48129 | 0.29814 | 13264 | |
| Furin | 0.48131 | -0.47664 | 13265 | |
| Eepd1 | 0.48134 | 0.36304 | 13266 | |
| Bag5 | 0.48138 | -0.4599 | 13267 | |
| Ostf1 | 0.48143 | 2.3234 | 13268 | |
| BC023829 | 0.48152 | -0.071181 | 13269 | |
| Nudt9 | 0.48154 | -0.035037 | 13270 | |
| Paip2 | 0.48156 | -0.097048 | 13271 | |
| Tmem132b | 0.48158 | 0.53644 | 13272 | |
| Serpinb7 | 0.4816 | -0.46118 | 13273 | |
| Hes5 | 0.48168 | 0.62283 | 13274 | |
| Rab3gap2 | 0.48174 | -0.48177 | 13275 | |
| Klrb1 | 0.48176 | -0.45131 | 13276 | |
| Orai2 | 0.48178 | -0.25619 | 13277 | |
| Stard7 | 0.48188 | -0.48273 | 13278 | |
| Cep290 | 0.48198 | -0.48503 | 13279 | |
| Ubn2 | 0.48198 | -0.48503 | 13280 | |
| Pcdha12 | 0.48199 | -0.0063592 | 13281 | |
| Abcf1 | 0.48205 | 0.73064 | 13282 | |
| Apol7e | 0.48219 | -0.48048 | 13283 | |
| mmu-mir-323 | 0.48227 | -0.29898 | 13284 | |
| Usp28 | 0.48233 | -0.4613 | 13285 | |
| Gas2 | 0.48238 | -0.46312 | 13286 | |
| Zkscan3 | 0.48242 | -0.48041 | 13287 | |
| Vwa5b2 | 0.48245 | -0.47073 | 13288 | |
| Vmn1r16 | 0.48246 | 1.5185 | 13289 | |
| Pot1b | 0.48247 | -0.47088 | 13290 | |
| Amy2b | 0.48249 | -0.22182 | 13291 | |
| F8a | 0.48263 | -0.481 | 13292 | |
| Zfp773 | 0.48265 | -0.31891 | 13293 | |
| Dedd2 | 0.48267 | -0.34512 | 13294 | |
| Ak3 | 0.48269 | 0.11351 | 13295 | |
| Sumf2 | 0.48274 | -0.43011 | 13296 | |
| Galnt7 | 0.48275 | -0.39934 | 13297 | |
| Rnf214 | 0.48279 | -0.47157 | 13298 | |
| Col8a2 | 0.48283 | -0.47785 | 13299 | |
| Akap6 | 0.48285 | -0.4812 | 13300 | |
| Serpini1 | 0.48287 | -0.37241 | 13301 | |
| Tub | 0.48299 | 0.30431 | 13302 | |
| Gba | 0.48302 | 0.1973 | 13303 | |
| Kbtbd11 | 0.48308 | -0.45392 | 13304 | |
| F2rl1 | 0.48313 | 0.49391 | 13305 | |
| Pkib | 0.48316 | 0.82394 | 13306 | |
| Gfod2 | 0.48319 | -0.2967 | 13307 | |
| Olfr656 | 0.48322 | -0.47608 | 13308 | |
| Lsmd1 | 0.48322 | 0.8546 | 13309 | |
| Gnao1 | 0.48329 | -0.46498 | 13310 | |
| Batf2 | 0.48338 | -0.47461 | 13311 | |
| Mtfp1 | 0.48343 | 0.5823 | 13312 | |
| Dtnb | 0.48345 | -0.47218 | 13313 | |
| Kbtbd2 | 0.48356 | -0.48836 | 13314 | |
| Rasa1 | 0.48357 | 0.98046 | 13315 | |
| Hfe2 | 0.48361 | -0.42376 | 13316 | |
| Rpap3 | 0.4837 | -0.47522 | 13317 | |
| Clk4 | 0.4841 | -0.25571 | 13318 | |
| Soat2 | 0.48413 | 1.5432 | 13319 | |
| Lamc1 | 0.48428 | 0.81658 | 13320 | |
| mmu-mir-7060 | 0.48432 | -0.27898 | 13321 | |
| Slc19a2 | 0.48438 | -0.37932 | 13322 | |
| C1qb | 0.48441 | -0.48193 | 13323 | |
| Npy1r | 0.48445 | -0.47299 | 13324 | |
| Nfyb | 0.48446 | -0.56198 | 13325 | |
| Mep1b | 0.48447 | -0.47509 | 13326 | |
| Olfr1301 | 0.48452 | -0.47363 | 13327 | |
| Mpz | 0.4846 | -0.11438 | 13328 | |
| mmu-mir-6916 | 0.48463 | -0.43882 | 13329 | |
| Atf3 | 0.48474 | -0.47139 | 13330 | |
| Gpr19 | 0.48479 | -0.47203 | 13331 | |
| C8a | 0.48481 | -0.26267 | 13332 | |
| Fam132a | 0.48486 | -0.46638 | 13333 | |
| Defb14 | 0.48486 | -0.19258 | 13334 | |
| Wdr54 | 0.48492 | 0.25606 | 13335 | |
| Asap3 | 0.48503 | -0.47932 | 13336 | |
| Fgfbp1 | 0.48503 | -0.47932 | 13337 | |
| Timm8a1 | 0.48513 | -0.38434 | 13338 | |
| Syt14 | 0.48518 | 0.23894 | 13339 | |
| Vmn1r83 | 0.48535 | -0.48155 | 13340 | |
| Tmem223 | 0.48547 | -0.45614 | 13341 | |
| mmu-mir-342 | 0.48548 | 0.86435 | 13342 | |
| Neurl4 | 0.48553 | 0.20589 | 13343 | |
| Flg2 | 0.48561 | -0.44097 | 13344 | |
| Trove2 | 0.48565 | -0.14175 | 13345 | |
| Tmod4 | 0.48568 | -0.46401 | 13346 | |
| Dnajc12 | 0.48571 | -0.35368 | 13347 | |
| Rhoa | 0.48574 | -0.34524 | 13348 | |
| Ubr2 | 0.48589 | -0.016272 | 13349 | |
| Fbn1 | 0.48599 | -0.45006 | 13350 | |
| Mvk | 0.48608 | -0.464 | 13351 | |
| Olfr305 | 0.48609 | 0.61922 | 13352 | |
| Mlip | 0.48612 | 1.7602 | 13353 | |
| Dcdc2a | 0.48615 | 1.6028 | 13354 | |
| Gpr156 | 0.48622 | -0.44103 | 13355 | |
| Fgf21 | 0.48624 | 1.9045 | 13356 | |
| Sox3 | 0.48631 | -0.46347 | 13357 | |
| Smad9 | 0.48635 | 0.050809 | 13358 | |
| Ddx27 | 0.4864 | -0.47791 | 13359 | |
| Ccdc19 | 0.48647 | 0.17379 | 13360 | |
| Tmem199 | 0.48654 | -0.44784 | 13361 | |
| Pqlc2 | 0.48663 | -0.47667 | 13362 | |
| Slc22a14 | 0.48667 | 0.61398 | 13363 | |
| Pex3 | 0.4867 | -0.43568 | 13364 | |
| Rpp38 | 0.48685 | 0.40509 | 13365 | |
| Olfr1134 | 0.48688 | -0.12243 | 13366 | |
| Agrp | 0.48692 | -0.46528 | 13367 | |
| mmu-mir-486 | 0.48696 | -0.11808 | 13368 | |
| Hmgn2 | 0.48697 | 0.052018 | 13369 | |
| Zfp799 | 0.48705 | -0.17128 | 13370 | |
| 1110065P20Rik | 0.48708 | 0.14479 | 13371 | |
| Zan | 0.48715 | -0.47025 | 13372 | |
| Olfr554 | 0.48722 | -0.17949 | 13373 | |
| Traf2 | 0.48732 | 0.014248 | 13374 | |
| Ovca2 | 0.48745 | -0.23848 | 13375 | |
| Proc | 0.48758 | -0.062915 | 13376 | |
| Pou4f2 | 0.48769 | 0.20455 | 13377 | |
| Cr2 | 0.48775 | -0.47742 | 13378 | |
| Gabra2 | 0.48788 | -0.44827 | 13379 | |
| Gramd4 | 0.48793 | -0.080169 | 13380 | |
| Atxn7l2 | 0.48795 | -0.47573 | 13381 | |
| Fam73b | 0.488 | -0.45237 | 13382 | |
| mmu-mir-1198 | 0.488 | -0.05657 | 13383 | |
| Pnliprp2 | 0.48802 | -0.44642 | 13384 | |
| Misp | 0.48804 | -0.45388 | 13385 | |
| Sin3a | 0.48806 | -0.45486 | 13386 | |
| Ntn1 | 0.48814 | -0.45781 | 13387 | |
| Ncam1 | 0.48818 | -0.46965 | 13388 | |
| Uggt2 | 0.48823 | -0.47365 | 13389 | |
| Ncf4 | 0.48826 | 1.0743 | 13390 | |
| Efcab14 | 0.48834 | -0.14074 | 13391 | |
| Chil3 | 0.48836 | -0.32885 | 13392 | |
| Zfp703 | 0.48838 | -0.44211 | 13393 | |
| Vmn1r213 | 0.48841 | -0.46493 | 13394 | |
| Myd88 | 0.48843 | -0.46252 | 13395 | |
| Slco4a1 | 0.48854 | 0.53869 | 13396 | |
| Otop1 | 0.48857 | 0.26065 | 13397 | |
| Pdyn | 0.4886 | -0.15966 | 13398 | |
| Tcf25 | 0.48863 | -0.46128 | 13399 | |
| Msx1 | 0.48868 | -0.29413 | 13400 | |
| Lrrc71 | 0.48869 | -0.4687 | 13401 | |
| Cldn7 | 0.48874 | -0.19681 | 13402 | |
| Sirt6 | 0.48879 | -0.065577 | 13403 | |
| Vmn2r94 | 0.48886 | -0.29098 | 13404 | |
| Olfr1229 | 0.48889 | -0.45701 | 13405 | |
| 2310061N02Rik | 0.48895 | -0.034762 | 13406 | |
| Slc8a1 | 0.48897 | -0.35232 | 13407 | |
| LOC100040786 | 0.48898 | -0.457 | 13408 | |
| Iqsec2 | 0.48904 | -0.46998 | 13409 | |
| Dip2a | 0.48921 | -0.16578 | 13410 | |
| Unc5c | 0.48929 | -0.23833 | 13411 | |
| Tbx20 | 0.48931 | 0.78419 | 13412 | |
| Pom121l12 | 0.4894 | -0.40434 | 13413 | |
| Rad18 | 0.4895 | -0.44463 | 13414 | |
| Nans | 0.48959 | 0.047418 | 13415 | |
| Pou4f1 | 0.48962 | 1.0943 | 13416 | |
| Nono | 0.48967 | -0.44788 | 13417 | |
| Skint3 | 0.48979 | 0.24039 | 13418 | |
| Olfr877 | 0.48984 | -0.47861 | 13419 | |
| mmu-mir-7026 | 0.48993 | 0.22223 | 13420 | |
| mmu-mir-6379 | 0.48993 | -0.47648 | 13421 | |
| Krt16 | 0.49 | -0.46034 | 13422 | |
| Tmem63a | 0.49002 | -0.30623 | 13423 | |
| Rnf208 | 0.49009 | -0.43498 | 13424 | |
| mmu-mir-184 | 0.4901 | 0.027642 | 13425 | |
| Myh6 | 0.49011 | -0.40404 | 13426 | |
| Vmn1r192 | 0.49012 | -0.19586 | 13427 | |
| Gpr161 | 0.49023 | -0.46565 | 13428 | |
| Abi2 | 0.49028 | -0.17654 | 13429 | |
| Epha1 | 0.49029 | -0.44153 | 13430 | |
| Oprk1 | 0.49032 | -0.45416 | 13431 | |
| Celf6 | 0.49035 | -0.46807 | 13432 | |
| Olfr1451 | 0.4904 | -0.47276 | 13433 | |
| Vmn1r221 | 0.4904 | -0.47276 | 13434 | |
| Sema4c | 0.49041 | -0.40889 | 13435 | |
| Lrrc36 | 0.49045 | -0.44409 | 13436 | |
| Klhl25 | 0.49052 | -0.12722 | 13437 | |
| Sccpdh | 0.49054 | -0.44573 | 13438 | |
| Gng10 | 0.4906 | -0.45934 | 13439 | |
| Ptprq | 0.49066 | -0.007453 | 13440 | |
| Ly6e | 0.49075 | -0.45738 | 13441 | |
| Grm3 | 0.49077 | -0.44135 | 13442 | |
| Cacnb1 | 0.49081 | -0.33221 | 13443 | |
| F2rl2 | 0.49083 | -0.45468 | 13444 | |
| mmu-mir-7675 | 0.49084 | -0.84859 | 13445 | |
| Bst2 | 0.49086 | -0.45656 | 13446 | |
| Aff2 | 0.49086 | -0.26042 | 13447 | |
| Bspry | 0.49089 | -0.47125 | 13448 | |
| Lamc2 | 0.49091 | -0.45713 | 13449 | |
| Pcdh9 | 0.49093 | -0.46813 | 13450 | |
| Glis1 | 0.49098 | 2.1973 | 13451 | |
| Olfr1173 | 0.49111 | -0.032099 | 13452 | |
| Zzef1 | 0.4912 | -0.45771 | 13453 | |
| Tmem260 | 0.49123 | -0.26586 | 13454 | |
| Kcnu1 | 0.49127 | 0.24742 | 13455 | |
| Cacng3 | 0.49132 | -0.4312 | 13456 | |
| Lmln | 0.49148 | -0.45273 | 13457 | |
| Metrnl | 0.4915 | -0.47463 | 13458 | |
| Kap | 0.4915 | 0.21518 | 13459 | |
| Slc34a1 | 0.49157 | -0.095163 | 13460 | |
| Ppp1r3c | 0.49164 | -0.39603 | 13461 | |
| Cyp2g1 | 0.49168 | -0.34921 | 13462 | |
| Grin2c | 0.4917 | -0.43491 | 13463 | |
| Dazl | 0.49172 | 0.11386 | 13464 | |
| Carm1 | 0.49178 | -0.45325 | 13465 | |
| Prtg | 0.49186 | -0.47668 | 13466 | |
| 4921524L21Rik | 0.49191 | -0.43729 | 13467 | |
| Zc3h12d | 0.492 | 0.0082408 | 13468 | |
| Ydjc | 0.49208 | 0.23267 | 13469 | |
| Olfr1087 | 0.49214 | -0.45602 | 13470 | |
| Ush1c | 0.49223 | -0.43409 | 13471 | |
| Ccdc6 | 0.49233 | -0.026943 | 13472 | |
| mmu-mir-701 | 0.49236 | -0.65569 | 13473 | |
| Tjap1 | 0.49246 | -0.36685 | 13474 | |
| Plag1 | 0.49248 | -0.3361 | 13475 | |
| Olfr139 | 0.49257 | -0.017147 | 13476 | |
| B4galt6 | 0.49257 | 0.62246 | 13477 | |
| Edn3 | 0.49259 | -0.30952 | 13478 | |
| Calb1 | 0.49266 | -0.46608 | 13479 | |
| Pp2d1 | 0.49271 | -0.35639 | 13480 | |
| Snapin | 0.49277 | -0.43545 | 13481 | |
| Pan3 | 0.49278 | -0.09109 | 13482 | |
| Mgll | 0.49286 | -0.081838 | 13483 | |
| Srr | 0.49291 | -0.2605 | 13484 | |
| Grm2 | 0.49292 | 0.26779 | 13485 | |
| Nfkb1 | 0.49298 | -0.44502 | 13486 | |
| Anpep | 0.49305 | -0.35434 | 13487 | |
| Fhdc1 | 0.49312 | -0.47349 | 13488 | |
| Rbm11 | 0.49316 | -0.18446 | 13489 | |
| Rhov | 0.49321 | -0.43707 | 13490 | |
| Tmed6 | 0.49321 | -0.35359 | 13491 | |
| Porcn | 0.49327 | 0.052659 | 13492 | |
| Polh | 0.49328 | -0.44833 | 13493 | |
| Prrg3 | 0.4933 | -0.3148 | 13494 | |
| Armcx3 | 0.4933 | -0.25115 | 13495 | |
| Agpat4 | 0.49333 | -0.12311 | 13496 | |
| Syde2 | 0.49335 | -0.45099 | 13497 | |
| Lce3b | 0.49338 | -0.13539 | 13498 | |
| Gabra3 | 0.49339 | -0.46766 | 13499 | |
| Nkiras1 | 0.49359 | 0.36389 | 13500 | |
| Mamdc2 | 0.49368 | -0.4301 | 13501 | |
| Dyrk4 | 0.4937 | 0.042965 | 13502 | |
| Olfr739 | 0.49373 | 0.0043186 | 13503 | |
| Zswim1 | 0.4938 | -0.34701 | 13504 | |
| Hivep2 | 0.49384 | -0.40262 | 13505 | |
| Lck | 0.49385 | -0.17832 | 13506 | |
| B230216G23Rik | 0.49396 | 0.66096 | 13507 | |
| Igsf11 | 0.494 | -0.43279 | 13508 | |
| Tmed1 | 0.49404 | 0.13983 | 13509 | |
| Gm8882 | 0.49411 | -0.031063 | 13510 | |
| 1700113H08Rik | 0.49417 | 1.0472 | 13511 | |
| Klhdc7b | 0.49425 | -0.3363 | 13512 | |
| Spz1 | 0.49429 | -0.45585 | 13513 | |
| Zfp212 | 0.49429 | -0.45585 | 13514 | |
| Myct1 | 0.49433 | -0.46539 | 13515 | |
| Zfp740 | 0.4944 | 1.5097 | 13516 | |
| Mina | 0.49441 | -0.053394 | 13517 | |
| mmu-mir-6913 | 0.49446 | 0.24906 | 13518 | |
| Snrpa1 | 0.49447 | -0.56198 | 13519 | |
| Eid2b | 0.49448 | -0.44187 | 13520 | |
| Hist1h2ak | 0.4945 | -0.081216 | 13521 | |
| Ctsa | 0.49451 | -0.19302 | 13522 | |
| Srsf10 | 0.49453 | -0.44862 | 13523 | |
| Rnft1 | 0.49454 | 0.87679 | 13524 | |
| Focad | 0.49457 | 1.5662 | 13525 | |
| Trim30d | 0.49466 | 0.33519 | 13526 | |
| Tnxb | 0.49475 | -0.44828 | 13527 | |
| Rgs12 | 0.4948 | 1.0155 | 13528 | |
| Spint3 | 0.49482 | -0.45172 | 13529 | |
| Apoc4 | 0.49489 | 0.58555 | 13530 | |
| Extl2 | 0.49489 | -0.44586 | 13531 | |
| Neurl3 | 0.49497 | 0.29924 | 13532 | |
| Ldhc | 0.49503 | 0.3375 | 13533 | |
| Rps6ka6 | 0.49514 | -0.43177 | 13534 | |
| Gm15080 | 0.49523 | -0.45285 | 13535 | |
| Mtmr10 | 0.49535 | 0.27667 | 13536 | |
| BC021614 | 0.49551 | -0.02411 | 13537 | |
| Ifnl3 | 0.49553 | -0.37656 | 13538 | |
| Gm11564 | 0.49555 | -0.30533 | 13539 | |
| Psd | 0.49557 | -0.44507 | 13540 | |
| Dnajc9 | 0.49563 | -0.45639 | 13541 | |
| Tecpr1 | 0.49569 | -0.32696 | 13542 | |
| 1700017B05Rik | 0.49575 | -0.033099 | 13543 | |
| Bicc1 | 0.49587 | -0.42767 | 13544 | |
| Il12b | 0.49591 | -0.42881 | 13545 | |
| Serpinb6b | 0.49593 | 0.48035 | 13546 | |
| Mmd2 | 0.49594 | -0.43266 | 13547 | |
| Csrp2bp | 0.496 | -0.34886 | 13548 | |
| Srl | 0.49605 | -0.45813 | 13549 | |
| Tmem11 | 0.49616 | 0.074391 | 13550 | |
| Cyp4a30b-ps | 0.49621 | -0.11862 | 13551 | |
| Apoa5 | 0.49622 | -0.42558 | 13552 | |
| Blcap | 0.49624 | -0.20668 | 13553 | |
| Hgsnat | 0.49632 | 0.53743 | 13554 | |
| Pfn3 | 0.49635 | -0.42492 | 13555 | |
| Ripk2 | 0.49636 | 0.27636 | 13556 | |
| Creg1 | 0.49644 | -0.35191 | 13557 | |
| Tmem234 | 0.49646 | -0.42827 | 13558 | |
| Cst7 | 0.4965 | 1.2851 | 13559 | |
| Cdk5r2 | 0.49651 | -0.42976 | 13560 | |
| Pitx1 | 0.49653 | -0.4338 | 13561 | |
| 4933411K16Rik | 0.49656 | 0.81599 | 13562 | |
| Prkar2b | 0.49659 | 0.49062 | 13563 | |
| Thrsp | 0.4966 | -0.28173 | 13564 | |
| Cdkn2a | 0.49662 | 1.3074 | 13565 | |
| Klk4 | 0.49662 | -0.29392 | 13566 | |
| Alpk3 | 0.49671 | -0.43586 | 13567 | |
| Gm4847 | 0.49676 | -0.44814 | 13568 | |
| Cadm4 | 0.49683 | -0.29657 | 13569 | |
| Cyb561 | 0.49685 | -0.43986 | 13570 | |
| Nck1 | 0.49687 | -0.43824 | 13571 | |
| Rbm15 | 0.49692 | 1.5805 | 13572 | |
| Ppcdc | 0.49702 | -0.43882 | 13573 | |
| Clybl | 0.49714 | -0.43131 | 13574 | |
| Mmp13 | 0.49719 | 0.40461 | 13575 | |
| Olfr959 | 0.49731 | -0.39636 | 13576 | |
| Nt5e | 0.49737 | 0.74753 | 13577 | |
| Dsg1c | 0.49737 | -0.45813 | 13578 | |
| Mapk8 | 0.49744 | -0.34139 | 13579 | |
| Mall | 0.49748 | 0.15652 | 13580 | |
| Themis | 0.49751 | -0.45662 | 13581 | |
| Trim75 | 0.49753 | -0.42868 | 13582 | |
| Klk1b27 | 0.49755 | -0.20902 | 13583 | |
| Park7 | 0.49758 | -0.32266 | 13584 | |
| Tmem158 | 0.4976 | -0.25967 | 13585 | |
| Sel1l3 | 0.49767 | -0.11648 | 13586 | |
| Ermap | 0.49768 | 0.71497 | 13587 | |
| Vmn2r70 | 0.49769 | -0.32936 | 13588 | |
| Pcgf3 | 0.49777 | 2.6286 | 13589 | |
| Homer2 | 0.49783 | 0.18931 | 13590 | |
| Lrrc19 | 0.49788 | -0.2026 | 13591 | |
| Gm1553 | 0.49791 | -0.44762 | 13592 | |
| Plekhg6 | 0.49794 | -0.43352 | 13593 | |
| 4833439L19Rik | 0.49801 | 0.0713 | 13594 | |
| Nr3c1 | 0.49804 | -0.42053 | 13595 | |
| Kank3 | 0.4981 | -0.4352 | 13596 | |
| Sh3bgrl2 | 0.49815 | -0.43817 | 13597 | |
| Usp47 | 0.49817 | -0.43003 | 13598 | |
| Spats2l | 0.49819 | -0.44852 | 13599 | |
| Fmnl2 | 0.49826 | -0.11687 | 13600 | |
| 1810062G17Rik | 0.4983 | -0.45256 | 13601 | |
| Arsi | 0.49832 | 0.48576 | 13602 | |
| G6pdx | 0.49833 | -0.44687 | 13603 | |
| Zbtb25 | 0.49835 | -0.35383 | 13604 | |
| Ctdp1 | 0.49837 | -0.054036 | 13605 | |
| Tmem9 | 0.49841 | -0.44242 | 13606 | |
| Bcor | 0.49846 | 1.0386 | 13607 | |
| Cnot1 | 0.49849 | 0.27896 | 13608 | |
| G6pc2 | 0.49855 | -0.10949 | 13609 | |
| Hspa8 | 0.4986 | 0.21839 | 13610 | |
| Tpp1 | 0.49862 | -0.40293 | 13611 | |
| Morn3 | 0.49871 | -0.44829 | 13612 | |
| Olfr1328 | 0.49875 | 1.5232 | 13613 | |
| Tas2r110 | 0.49878 | -0.43129 | 13614 | |
| Pparg | 0.4988 | 0.89122 | 13615 | |
| Prickle1 | 0.49885 | -0.40346 | 13616 | |
| Sec16a | 0.49894 | -0.032168 | 13617 | |
| Mkrn3 | 0.49899 | -0.44214 | 13618 | |
| Nudt15 | 0.49905 | 0.074195 | 13619 | |
| Sfn | 0.49908 | -0.21222 | 13620 | |
| Neo1 | 0.49912 | -0.185 | 13621 | |
| P4ha1 | 0.49912 | -0.45089 | 13622 | |
| Abcg2 | 0.49915 | -0.43129 | 13623 | |
| Sesn2 | 0.49917 | -0.43053 | 13624 | |
| Rbmxl2 | 0.49921 | -0.083625 | 13625 | |
| Cyp4f39 | 0.49921 | -0.43154 | 13626 | |
| Ly6h | 0.49926 | -0.42672 | 13627 | |
| Serhl | 0.49932 | -0.24076 | 13628 | |
| Fbxl13 | 0.49944 | -0.43869 | 13629 | |
| 1110032F04Rik | 0.49952 | -0.4251 | 13630 | |
| Olfr1509 | 0.49956 | -0.42861 | 13631 | |
| 1700109H08Rik | 0.49958 | -0.44 | 13632 | |
| Akap14 | 0.4996 | -0.41848 | 13633 | |
| Fer1l4 | 0.49962 | -0.2505 | 13634 | |
| mmu-mir-181a-1 | 0.49963 | 0.09048 | 13635 | |
| Irak1 | 0.49964 | 1.5918 | 13636 | |
| Arhgap23 | 0.49966 | 0.10713 | 13637 | |
| Pik3cb | 0.49972 | -0.43934 | 13638 | |
| Epha4 | 0.49975 | -0.016151 | 13639 | |
| Zfp286 | 0.49976 | -0.43133 | 13640 | |
| Skint4 | 0.49979 | -0.25266 | 13641 | |
| Nanog | 0.49979 | -0.28943 | 13642 | |
| Mixl1 | 0.49985 | -0.14222 | 13643 | |
| Slc29a3 | 0.49987 | 0.07585 | 13644 | |
| Mbp | 0.49987 | -0.11787 | 13645 | |
| Zdhhc8 | 0.49992 | 0.59969 | 13646 | |
| Hrsp12 | 0.49997 | -0.43869 | 13647 | |
| Ube2d1 | 0.50007 | -0.26376 | 13648 | |
| Capn15 | 0.50015 | 1.3959 | 13649 | |
| Kdm5c | 0.50018 | 1.0632 | 13650 | |
| Iqsec1 | 0.50021 | 0.028473 | 13651 | |
| Nfrkb | 0.50027 | 0.70511 | 13652 | |
| Rnase1 | 0.5003 | -0.0072569 | 13653 | |
| Olfr1254 | 0.50032 | -0.39808 | 13654 | |
| Ltb4r1 | 0.50052 | -0.35592 | 13655 | |
| Rnls | 0.50081 | 0.46657 | 13656 | |
| Sdc3 | 0.50091 | 0.16848 | 13657 | |
| Mif4gd | 0.50095 | 0.24797 | 13658 | |
| Hist1h4b | 0.50098 | 0.28638 | 13659 | |
| mmu-mir-494 | 0.50108 | 0.28436 | 13660 | |
| mmu-mir-449b | 0.50115 | -0.22634 | 13661 | |
| mmu-mir-133b | 0.50119 | 0.21328 | 13662 | |
| Spem1 | 0.5013 | 0.12505 | 13663 | |
| Igsf9b | 0.50156 | -0.38924 | 13664 | |
| Spata4 | 0.50167 | 0.78667 | 13665 | |
| Thsd7a | 0.5017 | -0.36056 | 13666 | |
| Ptprb | 0.50173 | 1.7694 | 13667 | |
| Tas2r135 | 0.50181 | 0.88201 | 13668 | |
| 3110052M02Rik | 0.50196 | 0.4656 | 13669 | |
| Rhox2h | 0.50221 | 0.20359 | 13670 | |
| Hyal5 | 0.50226 | 0.47135 | 13671 | |
| Olfr746 | 0.50236 | 0.34191 | 13672 | |
| B020004J07Rik | 0.50273 | 0.19082 | 13673 | |
| Pmfbp1 | 0.50284 | -0.22135 | 13674 | |
| Atp5g1 | 0.5029 | -0.12258 | 13675 | |
| Olfr483 | 0.50293 | -0.13082 | 13676 | |
| Agpat6 | 0.50298 | 0.11295 | 13677 | |
| G3bp1 | 0.50304 | 0.84861 | 13678 | |
| mmu-mir-3066 | 0.50317 | -0.093577 | 13679 | |
| Ceacam9 | 0.50318 | -0.37633 | 13680 | |
| Sh3d19 | 0.50324 | -0.2484 | 13681 | |
| Txk | 0.5033 | 0.73771 | 13682 | |
| Keg1 | 0.5035 | -0.32343 | 13683 | |
| Olfr1133 | 0.50356 | -0.11297 | 13684 | |
| Olfr3 | 0.5036 | 0.11452 | 13685 | |
| Prrc2b | 0.50364 | 0.3561 | 13686 | |
| Hecw2 | 0.50378 | 1.0818 | 13687 | |
| Tnfrsf25 | 0.5039 | -0.35711 | 13688 | |
| Pcdhb21 | 0.5041 | 0.013919 | 13689 | |
| mmu-mir-483 | 0.50422 | -0.49521 | 13690 | |
| Psme1 | 0.50435 | 0.15437 | 13691 | |
| Arl2bp | 0.50444 | 1.5916 | 13692 | |
| Lect1 | 0.50464 | -0.28953 | 13693 | |
| Slc16a13 | 0.50472 | -0.20201 | 13694 | |
| Usp2 | 0.50481 | 0.2746 | 13695 | |
| Prpsap2 | 0.50484 | 0.85585 | 13696 | |
| Lcn8 | 0.5049 | 0.158 | 13697 | |
| Zcchc7 | 0.5051 | -0.33436 | 13698 | |
| Mapkapk3 | 0.50517 | 1.3783 | 13699 | |
| Srsf5 | 0.50521 | 0.52152 | 13700 | |
| Tanc2 | 0.50539 | 0.10328 | 13701 | |
| Zfp957 | 0.50554 | 0.063749 | 13702 | |
| Aplp1 | 0.50566 | -0.30228 | 13703 | |
| Rabep1 | 0.50589 | -0.37907 | 13704 | |
| Taar7a | 0.50592 | 0.35464 | 13705 | |
| Mmp23 | 0.50595 | 0.26356 | 13706 | |
| Stmn4 | 0.50601 | -0.23505 | 13707 | |
| Gm15800 | 0.50638 | 0.7182 | 13708 | |
| 2300005B03Rik | 0.50672 | 0.15976 | 13709 | |
| Zic4 | 0.50675 | -0.27884 | 13710 | |
| E430018J23Rik | 0.50683 | -0.12367 | 13711 | |
| 4922501L14Rik | 0.50697 | -0.091178 | 13712 | |
| Pcsk5 | 0.507 | 0.76012 | 13713 | |
| mmu-mir-6898 | 0.50714 | 0.22563 | 13714 | |
| Smco4 | 0.50717 | -0.23997 | 13715 | |
| Fam26e | 0.5072 | 0.049867 | 13716 | |
| Notum | 0.50728 | 0.41642 | 13717 | |
| Col9a1 | 0.50746 | 1.2638 | 13718 | |
| mmu-mir-292 | 0.50752 | -0.32085 | 13719 | |
| Mmp1a | 0.50761 | 0.4149 | 13720 | |
| Ifi44 | 0.50765 | 0.71101 | 13721 | |
| Fkbp15 | 0.5077 | 0.76133 | 13722 | |
| H2-Q7 | 0.50778 | 0.029611 | 13723 | |
| Gm5134 | 0.50799 | 0.0058556 | 13724 | |
| Ncor2 | 0.50804 | 0.66076 | 13725 | |
| Lce6a | 0.50804 | -0.13197 | 13726 | |
| Vmn1r28 | 0.50808 | -0.36507 | 13727 | |
| Wdr78 | 0.50818 | 0.58042 | 13728 | |
| Foxred1 | 0.50822 | 1.0438 | 13729 | |
| Ptk2b | 0.50833 | 0.35559 | 13730 | |
| Olfr1278 | 0.50842 | -0.034656 | 13731 | |
| Nyx | 0.50865 | 1.1111 | 13732 | |
| Rassf2 | 0.50884 | 0.033159 | 13733 | |
| Gm5934 | 0.50893 | 0.20159 | 13734 | |
| Brd4 | 0.50921 | 1.6388 | 13735 | |
| Panx2 | 0.50933 | -0.32375 | 13736 | |
| mmu-mir-6963 | 0.50974 | -0.30687 | 13737 | |
| Gm14743 | 0.50981 | 0.96884 | 13738 | |
| Ttll9 | 0.51006 | -0.030713 | 13739 | |
| mmu-mir-8109 | 0.51064 | -0.13888 | 13740 | |
| Laptm5 | 0.51066 | 0.18209 | 13741 | |
| Slc39a14 | 0.51073 | -0.32074 | 13742 | |
| Avpr1a | 0.51119 | 0.033718 | 13743 | |
| Tmem88 | 0.51142 | -0.2449 | 13744 | |
| Prdm1 | 0.51145 | 0.7065 | 13745 | |
| Psmd1 | 0.51148 | -0.19298 | 13746 | |
| Mxd3 | 0.51153 | 0.15954 | 13747 | |
| Gkap1 | 0.51163 | 0.49149 | 13748 | |
| Fam212a | 0.51167 | 0.058356 | 13749 | |
| Prkcsh | 0.5117 | 0.53991 | 13750 | |
| Fgf8 | 0.51173 | -0.23423 | 13751 | |
| Olfr1057 | 0.51196 | -0.047166 | 13752 | |
| Ccdc105 | 0.51227 | 0.066093 | 13753 | |
| Phkb | 0.51243 | 0.14937 | 13754 | |
| Sned1 | 0.51252 | -0.020521 | 13755 | |
| Gm3285 | 0.51255 | 0.16327 | 13756 | |
| 2810007J24Rik | 0.51258 | 0.3535 | 13757 | |
| Ankk1 | 0.51266 | -0.21104 | 13758 | |
| Eif4enif1 | 0.51289 | 0.31415 | 13759 | |
| Zfand4 | 0.51308 | 0.40893 | 13760 | |
| Map6 | 0.51311 | -0.38509 | 13761 | |
| Gm4177 | 0.51325 | 0.86468 | 13762 | |
| Scg2 | 0.51332 | 0.29711 | 13763 | |
| mmu-mir-3059 | 0.51332 | 0.37417 | 13764 | |
| Abca4 | 0.51339 | 0.27674 | 13765 | |
| mmu-mir-3095 | 0.51364 | 0.42837 | 13766 | |
| Tdrd7 | 0.51365 | 0.085003 | 13767 | |
| 4930502E18Rik | 0.5137 | -0.31333 | 13768 | |
| Sbk3 | 0.51373 | -0.26482 | 13769 | |
| Acot8 | 0.5141 | 1.1342 | 13770 | |
| mmu-mir-491 | 0.51413 | -0.63205 | 13771 | |
| Penk | 0.51421 | 0.43889 | 13772 | |
| Sacm1l | 0.51424 | 0.23212 | 13773 | |
| Nr0b2 | 0.51446 | 0.73443 | 13774 | |
| Adamts6 | 0.51452 | 0.13261 | 13775 | |
| Rnf138 | 0.51457 | 0.29143 | 13776 | |
| Sdhb | 0.5146 | 0.082347 | 13777 | |
| Tarbp2 | 0.51466 | 1.4301 | 13778 | |
| 1110059E24Rik | 0.51473 | 0.497 | 13779 | |
| Ppp1r35 | 0.51473 | 1.2123 | 13780 | |
| Lce3d | 0.51484 | -0.70866 | 13781 | |
| Gm10697 | 0.51486 | 1.3015 | 13782 | |
| Gdpd5 | 0.51488 | -0.36943 | 13783 | |
| Smek2 | 0.51501 | 0.20493 | 13784 | |
| Itgad | 0.51519 | 1.0632 | 13785 | |
| mmu-mir-7015 | 0.5152 | -0.38855 | 13786 | |
| Olfr385 | 0.51533 | 2.7524 | 13787 | |
| Irf9 | 0.51553 | 0.22576 | 13788 | |
| Spdef | 0.51556 | 0.90901 | 13789 | |
| Tex19.2 | 0.51559 | -0.37571 | 13790 | |
| 4930430F08Rik | 0.51575 | -0.034337 | 13791 | |
| Zscan21 | 0.51578 | 1.5341 | 13792 | |
| mmu-mir-375 | 0.51588 | -0.049125 | 13793 | |
| Usp38 | 0.51592 | 0.55888 | 13794 | |
| mmu-mir-6418 | 0.51593 | -0.15141 | 13795 | |
| Alox12e | 0.51615 | -0.10369 | 13796 | |
| mmu-mir-343 | 0.51616 | -0.38639 | 13797 | |
| Nudt6 | 0.5162 | 1.4063 | 13798 | |
| Rab27a | 0.51644 | 0.49863 | 13799 | |
| Hnrnph1 | 0.51665 | 0.007144 | 13800 | |
| Igfbpl1 | 0.51674 | -0.20111 | 13801 | |
| Cntn1 | 0.51679 | 0.8956 | 13802 | |
| Fxyd4 | 0.51682 | 0.015093 | 13803 | |
| Tmem89 | 0.51702 | 0.29901 | 13804 | |
| Pigk | 0.51718 | -0.11581 | 13805 | |
| Gm16405 | 0.51721 | 0.054277 | 13806 | |
| Tectb | 0.51727 | 0.35774 | 13807 | |
| Ptpn22 | 0.51732 | 0.43807 | 13808 | |
| CK137956 | 0.51735 | 0.66027 | 13809 | |
| Ly6g6e | 0.51752 | 0.050919 | 13810 | |
| Ptov1 | 0.51763 | -0.12289 | 13811 | |
| Rgs6 | 0.51791 | -0.2545 | 13812 | |
| Rpp25l | 0.518 | 0.47516 | 13813 | |
| 4930525M21Rik | 0.51811 | 1.2058 | 13814 | |
| Ccdc64 | 0.5185 | 0.49165 | 13815 | |
| Zfp60 | 0.51853 | 0.90102 | 13816 | |
| Rfx2 | 0.51867 | 0.38026 | 13817 | |
| Acss1 | 0.5187 | 0.52348 | 13818 | |
| Olfr965 | 0.51875 | 0.50339 | 13819 | |
| Slc9a5 | 0.51885 | 1.0883 | 13820 | |
| Hspb11 | 0.51895 | 0.36358 | 13821 | |
| Pspc1 | 0.51895 | 0.16996 | 13822 | |
| Phlpp1 | 0.51906 | -0.23957 | 13823 | |
| Mrgpra6 | 0.51911 | 1.35 | 13824 | |
| Leng9 | 0.51925 | 0.10387 | 13825 | |
| Cyp4x1 | 0.51931 | -0.16328 | 13826 | |
| Naif1 | 0.51939 | 0.46349 | 13827 | |
| Rab3ip | 0.51962 | -0.15181 | 13828 | |
| Bhlhe41 | 0.51981 | 0.24566 | 13829 | |
| mmu-mir-7069 | 0.52004 | -0.50629 | 13830 | |
| Gadd45b | 0.52023 | 0.068096 | 13831 | |
| Rnf151 | 0.52026 | -0.29406 | 13832 | |
| Nptxr | 0.52029 | 0.58754 | 13833 | |
| Sox17 | 0.52031 | 0.05898 | 13834 | |
| Olfr131 | 0.52037 | -0.35281 | 13835 | |
| Gm694 | 0.52043 | -0.03759 | 13836 | |
| 2310011J03Rik | 0.52051 | 0.40314 | 13837 | |
| Olfr9 | 0.52076 | 0.93316 | 13838 | |
| 1700021F07Rik | 0.52084 | -0.16557 | 13839 | |
| Crip3 | 0.52107 | 0.16642 | 13840 | |
| Mtfr1l | 0.52112 | 0.17021 | 13841 | |
| Trim68 | 0.52132 | 1.4995 | 13842 | |
| Fblim1 | 0.52154 | 1.2682 | 13843 | |
| Gm5640 | 0.5216 | -0.22917 | 13844 | |
| Tmem176a | 0.52168 | -0.29678 | 13845 | |
| Il2ra | 0.52174 | 1.1787 | 13846 | |
| Clic4 | 0.52185 | -0.22813 | 13847 | |
| Pdpn | 0.52188 | -0.27946 | 13848 | |
| Rnf219 | 0.5219 | 0.65766 | 13849 | |
| Gm12253 | 0.52196 | 0.36672 | 13850 | |
| Serpinb8 | 0.52226 | 0.072776 | 13851 | |
| Olfr270 | 0.5224 | 0.68167 | 13852 | |
| Arnt2 | 0.52243 | 0.048387 | 13853 | |
| Zfp658 | 0.52246 | 0.62365 | 13854 | |
| Serpinb3d | 0.52253 | 0.077536 | 13855 | |
| Rif1 | 0.52271 | -0.42343 | 13856 | |
| Smyd5 | 0.52274 | -0.37334 | 13857 | |
| Oat | 0.52288 | -0.24805 | 13858 | |
| mmu-mir-6943 | 0.52295 | -0.36454 | 13859 | |
| Ccer1 | 0.52296 | 0.056154 | 13860 | |
| Lama5 | 0.52307 | 0.0037662 | 13861 | |
| Klhdc8b | 0.5231 | 0.27607 | 13862 | |
| Slc17a3 | 0.52324 | 0.42883 | 13863 | |
| Mcm10 | 0.52329 | 0.6044 | 13864 | |
| Gm5132 | 0.52339 | 2.018 | 13865 | |
| Hist1h2af | 0.52343 | 1.16 | 13866 | |
| Sptan1 | 0.52349 | -0.28674 | 13867 | |
| Xpa | 0.52353 | 0.14834 | 13868 | |
| Pcyt1b | 0.52357 | 1.0166 | 13869 | |
| Pramef6 | 0.5236 | 0.20865 | 13870 | |
| Cxcl11 | 0.52382 | -0.24432 | 13871 | |
| Gm14851 | 0.52403 | 0.36675 | 13872 | |
| 9630033F20Rik | 0.52425 | 0.11604 | 13873 | |
| Sorbs1 | 0.52429 | 0.46691 | 13874 | |
| Leo1 | 0.52438 | 0.11893 | 13875 | |
| Myl3 | 0.5244 | 0.12595 | 13876 | |
| Olfr862 | 0.52446 | 0.48881 | 13877 | |
| mmu-mir-211 | 0.52454 | 0.46499 | 13878 | |
| Gcc2 | 0.52474 | -0.043938 | 13879 | |
| Optc | 0.52493 | 0.23778 | 13880 | |
| Josd2 | 0.52496 | -0.1336 | 13881 | |
| Gp6 | 0.52507 | -0.11064 | 13882 | |
| Galr2 | 0.5251 | -0.15055 | 13883 | |
| Jsrp1 | 0.52515 | 0.014097 | 13884 | |
| Bpnt1 | 0.52554 | 0.72416 | 13885 | |
| mmu-mir-1968 | 0.52617 | -0.045692 | 13886 | |
| Bach1 | 0.52623 | -0.049012 | 13887 | |
| mmu-mir-692-2 | 0.52628 | -0.33129 | 13888 | |
| Slc10a2 | 0.52637 | 0.40729 | 13889 | |
| Prickle2 | 0.52654 | 0.6889 | 13890 | |
| Nubpl | 0.52663 | 0.34984 | 13891 | |
| Olfr1170 | 0.52673 | 0.21197 | 13892 | |
| Srebf2 | 0.52677 | 0.63135 | 13893 | |
| B430306N03Rik | 0.52684 | 0.52054 | 13894 | |
| 2310079G19Rik | 0.52692 | -0.36566 | 13895 | |
| Taar7d | 0.52698 | -0.11581 | 13896 | |
| Fbxo41 | 0.52745 | 1.548 | 13897 | |
| Cldn23 | 0.52756 | 0.35349 | 13898 | |
| Dcaf11 | 0.52764 | 1.0306 | 13899 | |
| Pam | 0.52771 | -0.32909 | 13900 | |
| Olfr986 | 0.52778 | 1.3722 | 13901 | |
| Bmp3 | 0.52792 | 0.34899 | 13902 | |
| mmu-mir-669a-12 | 0.52803 | -0.32865 | 13903 | |
| Myoz2 | 0.52819 | 0.38128 | 13904 | |
| Olfr740 | 0.52844 | 2.2597 | 13905 | |
| Sncaip | 0.52852 | 0.51297 | 13906 | |
| Ankrd35 | 0.52872 | -0.37786 | 13907 | |
| Lphn1 | 0.52877 | 0.64179 | 13908 | |
| Efcab2 | 0.5288 | 0.78021 | 13909 | |
| AI429214 | 0.52883 | -0.27023 | 13910 | |
| Btbd1 | 0.52899 | 1.4914 | 13911 | |
| Gm2863 | 0.529 | -0.31604 | 13912 | |
| Olfr123 | 0.52921 | 0.63876 | 13913 | |
| Bgn | 0.52924 | -0.40706 | 13914 | |
| Akna | 0.52927 | 0.60937 | 13915 | |
| mmu-let-7a-1 | 0.52934 | 0.58444 | 13916 | |
| mmu-mir-125a | 0.52946 | -0.081821 | 13917 | |
| Cryba1 | 0.5296 | 0.099506 | 13918 | |
| mmu-mir-7685 | 0.52963 | 1.0317 | 13919 | |
| mmu-mir-207 | 0.52975 | -0.54139 | 13920 | |
| Phip | 0.52985 | -0.30919 | 13921 | |
| mmu-mir-433 | 0.52987 | -0.22196 | 13922 | |
| Erp44 | 0.52997 | -0.23511 | 13923 | |
| Dlg3 | 0.53002 | -0.18764 | 13924 | |
| Nav3 | 0.53018 | 0.39062 | 13925 | |
| Olfr448 | 0.53026 | 0.49374 | 13926 | |
| Tdrd1 | 0.53031 | 0.95006 | 13927 | |
| mmu-mir-26b | 0.53036 | 0.16397 | 13928 | |
| Crygs | 0.53056 | -0.39334 | 13929 | |
| mmu-mir-7051 | 0.53059 | 0.15789 | 13930 | |
| Rbm5 | 0.53078 | 0.36276 | 13931 | |
| Sst | 0.53081 | -0.08191 | 13932 | |
| Ccdc107 | 0.53084 | -0.13163 | 13933 | |
| Cypt10 | 0.53092 | 0.51058 | 13934 | |
| Tmod3 | 0.53101 | 1.1154 | 13935 | |
| Gjc3 | 0.53114 | 0.0068434 | 13936 | |
| Adrb3 | 0.53128 | -0.41971 | 13937 | |
| Cdh8 | 0.5313 | -0.090411 | 13938 | |
| Krtcap2 | 0.5315 | -0.20087 | 13939 | |
| Aacs | 0.53159 | 0.46192 | 13940 | |
| Man2b1 | 0.53167 | -0.32596 | 13941 | |
| Tmem222 | 0.5318 | -0.27465 | 13942 | |
| Gpd2 | 0.53182 | 0.86711 | 13943 | |
| Ccdc102a | 0.53188 | 0.44937 | 13944 | |
| Gjb5 | 0.53191 | -0.21761 | 13945 | |
| Trem2 | 0.53196 | 1.826 | 13946 | |
| Myo1b | 0.53199 | -0.0092279 | 13947 | |
| Olfr936 | 0.53213 | 0.10817 | 13948 | |
| Cbx4 | 0.53215 | -0.406 | 13949 | |
| Clnk | 0.53221 | -0.0091848 | 13950 | |
| Yif1b | 0.53224 | 0.26997 | 13951 | |
| Cd200r2 | 0.53226 | 0.12562 | 13952 | |
| Gcsh | 0.53232 | 1.6745 | 13953 | |
| Lzts3 | 0.53243 | 1.9667 | 13954 | |
| Gprasp2 | 0.5327 | 0.95875 | 13955 | |
| Phgr1 | 0.53273 | 0.90671 | 13956 | |
| Pnmal2 | 0.53317 | 0.67734 | 13957 | |
| Trim37 | 0.53322 | 1.2806 | 13958 | |
| Prm1 | 0.53332 | 1.0265 | 13959 | |
| Det1 | 0.53341 | -0.40695 | 13960 | |
| Ceacam10 | 0.53347 | 0.14731 | 13961 | |
| Syde1 | 0.5335 | 0.21719 | 13962 | |
| Mtnr1a | 0.5338 | -0.35505 | 13963 | |
| Lep | 0.53382 | 0.72729 | 13964 | |
| Olfr1066 | 0.53385 | -0.083593 | 13965 | |
| mmu-mir-7087 | 0.53389 | -0.10628 | 13966 | |
| mmu-mir-6969 | 0.5343 | 1.7684 | 13967 | |
| Gm1661 | 0.53443 | -0.20333 | 13968 | |
| Znrf3 | 0.53451 | -0.082933 | 13969 | |
| Lancl1 | 0.53456 | 0.20033 | 13970 | |
| Olfr1006 | 0.53473 | -0.22537 | 13971 | |
| Rem1 | 0.53495 | 0.23468 | 13972 | |
| mmu-mir-7032 | 0.53499 | 0.031758 | 13973 | |
| Wipi2 | 0.5356 | 0.36034 | 13974 | |
| Prg3 | 0.53563 | 0.49157 | 13975 | |
| mmu-mir-1955 | 0.53569 | 0.42265 | 13976 | |
| Mdh2 | 0.53587 | 0.32188 | 13977 | |
| mmu-mir-30e | 0.53594 | 0.46809 | 13978 | |
| Tas2r137 | 0.53609 | 0.47022 | 13979 | |
| Rnf14 | 0.53624 | -0.40173 | 13980 | |
| Slc41a3 | 0.53641 | 0.30176 | 13981 | |
| Qrich1 | 0.53645 | -0.15079 | 13982 | |
| mmu-mir-653 | 0.5365 | -0.054594 | 13983 | |
| Irf2bp1 | 0.53653 | 1.6854 | 13984 | |
| mmu-mir-10a | 0.53689 | 0.049335 | 13985 | |
| Eci3 | 0.53696 | -0.40706 | 13986 | |
| Gm7257 | 0.53709 | 0.27958 | 13987 | |
| Slc17a7 | 0.53754 | -0.18415 | 13988 | |
| Nkx6-1 | 0.53756 | 1.8872 | 13989 | |
| 4933406J08Rik | 0.53765 | 0.30733 | 13990 | |
| Ggt7 | 0.53769 | 0.93607 | 13991 | |
| Cckar | 0.53769 | -0.0048663 | 13992 | |
| Retn | 0.53773 | -0.38635 | 13993 | |
| Gpr34 | 0.53797 | 0.95564 | 13994 | |
| Ptprf | 0.53805 | 0.43089 | 13995 | |
| Rnf44 | 0.53819 | -0.053608 | 13996 | |
| Gfpt1 | 0.53841 | 0.19045 | 13997 | |
| Lrrc23 | 0.53854 | 0.45903 | 13998 | |
| Morn5 | 0.53857 | 0.33747 | 13999 | |
| Dtx1 | 0.53862 | 0.1532 | 14000 | |
| Serpina3g | 0.53865 | -0.41781 | 14001 | |
| Zfyve28 | 0.53883 | 0.21972 | 14002 | |
| Lcn6 | 0.53894 | -0.45023 | 14003 | |
| BC053393 | 0.53906 | -0.32366 | 14004 | |
| mmu-mir-346 | 0.53909 | 0.12821 | 14005 | |
| Cmtm3 | 0.53921 | 2.8416 | 14006 | |
| Papola | 0.53944 | -0.21896 | 14007 | |
| Samd11 | 0.53966 | 1.0168 | 14008 | |
| Med13l | 0.53981 | 0.39041 | 14009 | |
| Fbxl22 | 0.53993 | -0.092083 | 14010 | |
| Rgn | 0.53997 | 0.67353 | 14011 | |
| Olfr734 | 0.54004 | 0.076593 | 14012 | |
| Fam174a | 0.54028 | 0.17058 | 14013 | |
| Selm | 0.54031 | 0.4285 | 14014 | |
| mmu-mir-2139 | 0.54038 | -0.22431 | 14015 | |
| Slc18a2 | 0.54052 | 0.015645 | 14016 | |
| Slain2 | 0.54063 | -0.16098 | 14017 | |
| Zfp277 | 0.54067 | 0.49615 | 14018 | |
| Btnl2 | 0.54071 | 0.54969 | 14019 | |
| Ppif | 0.54088 | -0.26303 | 14020 | |
| Rel | 0.54099 | 0.43928 | 14021 | |
| Clec2h | 0.54109 | 0.025901 | 14022 | |
| Nsg1 | 0.54115 | 0.1306 | 14023 | |
| Nol7 | 0.54139 | 0.77919 | 14024 | |
| Kcna3 | 0.54145 | 0.51496 | 14025 | |
| Ikzf2 | 0.54157 | -0.30053 | 14026 | |
| Slc22a3 | 0.5418 | -0.18683 | 14027 | |
| Spry1 | 0.54182 | -0.25505 | 14028 | |
| mmu-mir-7003 | 0.54185 | 0.92338 | 14029 | |
| Atad2b | 0.54185 | 0.60789 | 14030 | |
| Hmgn1 | 0.54196 | 0.47524 | 14031 | |
| Foxd3 | 0.54199 | 0.57881 | 14032 | |
| Tmx4 | 0.54212 | -0.14766 | 14033 | |
| Olfr655 | 0.5422 | 0.41959 | 14034 | |
| Agtr1b | 0.54226 | 1.9518 | 14035 | |
| Nbeal2 | 0.54234 | 0.70148 | 14036 | |
| Ubtd2 | 0.54245 | -0.074212 | 14037 | |
| Inpp5k | 0.54253 | 0.53986 | 14038 | |
| Col18a1 | 0.54255 | 0.022368 | 14039 | |
| Ccl21b | 0.54257 | -0.20323 | 14040 | |
| Zdhhc14 | 0.54299 | 2.2499 | 14041 | |
| Ctnna2 | 0.54301 | 0.49618 | 14042 | |
| mmu-mir-208b | 0.54306 | -0.28685 | 14043 | |
| Srrm4 | 0.54315 | 1.4573 | 14044 | |
| Slc18a1 | 0.5432 | 0.1846 | 14045 | |
| Mug1 | 0.54326 | 1.3056 | 14046 | |
| mmu-mir-3964 | 0.54347 | 0.25568 | 14047 | |
| Bpifa1 | 0.54354 | 0.26601 | 14048 | |
| Zfp933 | 0.54354 | -0.37386 | 14049 | |
| Mmp19 | 0.54374 | 0.16359 | 14050 | |
| Chil1 | 0.5438 | -0.30536 | 14051 | |
| Apbb1ip | 0.54396 | 0.1384 | 14052 | |
| Olfr1128 | 0.54403 | 0.14495 | 14053 | |
| Aldh3a1 | 0.54407 | 0.34134 | 14054 | |
| Saa1 | 0.54409 | -0.13766 | 14055 | |
| Kcnj2 | 0.5442 | -0.40417 | 14056 | |
| Ccnl1 | 0.54444 | 0.21584 | 14057 | |
| mmu-mir-6402 | 0.5445 | 0.047404 | 14058 | |
| Tomt | 0.5445 | 0.15337 | 14059 | |
| Leprotl1 | 0.54482 | 0.02197 | 14060 | |
| Lrrn1 | 0.54502 | -0.33484 | 14061 | |
| Krtap16-1 | 0.5451 | 0.43028 | 14062 | |
| Sbpl | 0.54517 | 0.85399 | 14063 | |
| BC055111 | 0.54522 | -0.23993 | 14064 | |
| 1700057G04Rik | 0.54525 | 0.13233 | 14065 | |
| Plekha7 | 0.54528 | -0.40361 | 14066 | |
| Dhrs13 | 0.54531 | 0.71268 | 14067 | |
| Tacstd2 | 0.54539 | 0.13235 | 14068 | |
| Rspo2 | 0.54541 | 0.041789 | 14069 | |
| Spta1 | 0.54544 | 0.035499 | 14070 | |
| Vmn1r210 | 0.54552 | 0.86103 | 14071 | |
| Cts3 | 0.54568 | -0.27317 | 14072 | |
| mmu-mir-365-2 | 0.54569 | 1.2496 | 14073 | |
| Jag2 | 0.54579 | -0.092437 | 14074 | |
| Ccdc65 | 0.54584 | 0.011331 | 14075 | |
| Hdac8 | 0.54592 | 0.016705 | 14076 | |
| Olfr557 | 0.54595 | 0.55715 | 14077 | |
| Qprt | 0.54598 | -0.33121 | 14078 | |
| Prss34 | 0.54603 | -0.084996 | 14079 | |
| Lmx1a | 0.54642 | -0.19751 | 14080 | |
| Brwd1 | 0.54642 | -0.038495 | 14081 | |
| Eri3 | 0.54657 | -0.31415 | 14082 | |
| Map3k9 | 0.54673 | -0.26605 | 14083 | |
| Harbi1 | 0.54676 | -0.047963 | 14084 | |
| Zxdc | 0.54684 | 1.1449 | 14085 | |
| Prl3d3 | 0.54688 | 0.54049 | 14086 | |
| Trim15 | 0.54713 | -0.17822 | 14087 | |
| mmu-mir-216b | 0.54715 | -0.42844 | 14088 | |
| Cpne6 | 0.54719 | 1.4467 | 14089 | |
| Rest | 0.54737 | 1.2548 | 14090 | |
| Klrb1a | 0.54743 | 0.56449 | 14091 | |
| Zfp592 | 0.54762 | 1.2329 | 14092 | |
| Spaca1 | 0.54764 | 0.24779 | 14093 | |
| Nlrp1b | 0.54775 | 1.062 | 14094 | |
| Pik3cg | 0.54788 | -0.068378 | 14095 | |
| Ireb2 | 0.54791 | 0.66509 | 14096 | |
| Lmf2 | 0.54799 | 1.1188 | 14097 | |
| Gpr171 | 0.54829 | 0.75347 | 14098 | |
| Mt3 | 0.54834 | 0.0045625 | 14099 | |
| Igfbp5 | 0.5485 | 0.14083 | 14100 | |
| Spata13 | 0.54855 | 1.0756 | 14101 | |
| Cep120 | 0.54861 | -0.4234 | 14102 | |
| Gm1979 | 0.54883 | -0.24972 | 14103 | |
| Msrb1 | 0.54885 | 0.72066 | 14104 | |
| Smim11 | 0.54902 | -0.39331 | 14105 | |
| Ormdl2 | 0.54902 | -0.37568 | 14106 | |
| Sox4 | 0.54918 | 0.025934 | 14107 | |
| Mia2 | 0.54918 | 0.81776 | 14108 | |
| Zfp386 | 0.54922 | 0.27745 | 14109 | |
| Moxd1 | 0.54952 | 1.5083 | 14110 | |
| Whrn | 0.54965 | -0.27421 | 14111 | |
| mmu-mir-505 | 0.5497 | -0.46929 | 14112 | |
| Mvp | 0.54978 | -0.40705 | 14113 | |
| 1500015O10Rik | 0.54984 | -0.16919 | 14114 | |
| Mtmr7 | 0.55011 | -0.23444 | 14115 | |
| Hs1bp3 | 0.55061 | -0.12582 | 14116 | |
| Apoh | 0.55067 | -0.26022 | 14117 | |
| Scarf2 | 0.55072 | 0.19524 | 14118 | |
| 2010002M12Rik | 0.55088 | -0.14309 | 14119 | |
| Ralgps2 | 0.55096 | 0.78057 | 14120 | |
| Kcna7 | 0.55099 | 0.25845 | 14121 | |
| mmu-mir-1899 | 0.55107 | 0.91899 | 14122 | |
| Eps15 | 0.55147 | 0.00011397 | 14123 | |
| mmu-mir-145b | 0.55165 | 0.12232 | 14124 | |
| Gsta3 | 0.55168 | 0.33258 | 14125 | |
| Cacng4 | 0.55176 | -0.19705 | 14126 | |
| Cyp2e1 | 0.55179 | 0.14753 | 14127 | |
| Bfsp1 | 0.55201 | 0.20694 | 14128 | |
| Slc2a7 | 0.55205 | -0.35747 | 14129 | |
| Zfp622 | 0.55232 | -0.42704 | 14130 | |
| Cxcl16 | 0.5524 | -0.064841 | 14131 | |
| Hmcn1 | 0.55243 | 0.61423 | 14132 | |
| Ypel4 | 0.55251 | 0.33546 | 14133 | |
| Abcg1 | 0.55256 | 0.018231 | 14134 | |
| Car12 | 0.55261 | -0.34857 | 14135 | |
| 1190003K10Rik | 0.55264 | 1.2712 | 14136 | |
| Paqr8 | 0.55291 | 0.39391 | 14137 | |
| mmu-mir-322 | 0.55297 | -0.32237 | 14138 | |
| Ggnbp1 | 0.55305 | 1.3405 | 14139 | |
| Tmem63c | 0.55312 | -0.093502 | 14140 | |
| mmu-mir-7224 | 0.55317 | -0.31047 | 14141 | |
| Ofd1 | 0.55317 | -0.34518 | 14142 | |
| Ms4a15 | 0.55323 | 0.20793 | 14143 | |
| Fndc1 | 0.55325 | 0.72461 | 14144 | |
| Frs3 | 0.55328 | -0.035156 | 14145 | |
| Pnn | 0.55331 | 1.4217 | 14146 | |
| mmu-mir-210 | 0.55333 | 0.02306 | 14147 | |
| Eaf1 | 0.55333 | 2.1339 | 14148 | |
| mmu-mir-7063 | 0.5536 | -0.21379 | 14149 | |
| Hhatl | 0.55368 | -0.26365 | 14150 | |
| Mitf | 0.55381 | 0.24434 | 14151 | |
| Psenen | 0.55402 | 0.28596 | 14152 | |
| 1700025F22Rik | 0.55408 | -0.43872 | 14153 | |
| Ltb | 0.55424 | -0.029869 | 14154 | |
| Vangl2 | 0.55434 | 0.13403 | 14155 | |
| Atp13a3 | 0.55439 | 0.72902 | 14156 | |
| Acpl2 | 0.55445 | 0.5118 | 14157 | |
| Nme2 | 0.55453 | -0.11514 | 14158 | |
| Fastkd3 | 0.55477 | -0.21023 | 14159 | |
| Mccc2 | 0.55487 | -0.20688 | 14160 | |
| Tmem202 | 0.55511 | 2.9573 | 14161 | |
| Milr1 | 0.55522 | 1.1586 | 14162 | |
| Gm5148 | 0.55543 | -0.31766 | 14163 | |
| Olfr599 | 0.5556 | -0.35779 | 14164 | |
| Setd1a | 0.5556 | 0.91346 | 14165 | |
| Klhl32 | 0.55564 | -0.33439 | 14166 | |
| Plekha2 | 0.55569 | -0.076536 | 14167 | |
| BC017158 | 0.55572 | 0.98428 | 14168 | |
| N4bp2l1 | 0.55575 | 0.67496 | 14169 | |
| Rragd | 0.55599 | 1.3488 | 14170 | |
| Ttc21a | 0.55604 | 0.50828 | 14171 | |
| Mavs | 0.55607 | 0.051739 | 14172 | |
| Scel | 0.5562 | 0.41853 | 14173 | |
| Bok | 0.55633 | -0.063299 | 14174 | |
| Ifi44l | 0.55637 | 2.1604 | 14175 | |
| Slc35c2 | 0.55637 | -0.15563 | 14176 | |
| mmu-mir-1843a | 0.55638 | -0.04444 | 14177 | |
| 2610044O15Rik8 | 0.55644 | 0.3873 | 14178 | |
| Cntnap5b | 0.55662 | -0.27261 | 14179 | |
| Ticam2 | 0.5567 | -0.35513 | 14180 | |
| Cpa4 | 0.55673 | -0.25725 | 14181 | |
| Stfa2l1 | 0.55691 | 0.060332 | 14182 | |
| Nipsnap1 | 0.55697 | -0.010457 | 14183 | |
| mmu-mir-298 | 0.55706 | -0.22097 | 14184 | |
| Rab3c | 0.55712 | 0.80211 | 14185 | |
| Akr1c6 | 0.55734 | 0.082773 | 14186 | |
| Ndufb4 | 0.55747 | 0.20765 | 14187 | |
| Them6 | 0.55755 | 0.067638 | 14188 | |
| Cep44 | 0.55759 | -0.096573 | 14189 | |
| Pak2 | 0.55765 | 0.23656 | 14190 | |
| Ppp1r7 | 0.55771 | -0.065582 | 14191 | |
| Lhx4 | 0.55792 | -0.37843 | 14192 | |
| Cyth4 | 0.55822 | 0.92893 | 14193 | |
| LOC100038947 | 0.55831 | 0.37288 | 14194 | |
| Klra18 | 0.5585 | 0.86209 | 14195 | |
| mmu-mir-6419 | 0.55862 | -0.42433 | 14196 | |
| Zfp867 | 0.55863 | 0.25227 | 14197 | |
| Itpkb | 0.55874 | -0.12119 | 14198 | |
| Smoc2 | 0.55895 | 0.13762 | 14199 | |
| Chodl | 0.55903 | 0.81354 | 14200 | |
| Ttc21b | 0.5591 | 0.15391 | 14201 | |
| 4933411G11Rik | 0.55916 | 0.68819 | 14202 | |
| Gm5615 | 0.55942 | 0.43363 | 14203 | |
| Gm14744 | 0.55945 | 0.59796 | 14204 | |
| Gucy1b3 | 0.55947 | -0.34903 | 14205 | |
| mmu-mir-883b | 0.5595 | 0.30857 | 14206 | |
| Avl9 | 0.55953 | 0.25881 | 14207 | |
| F13b | 0.55976 | -0.37031 | 14208 | |
| Rara | 0.55982 | 0.25854 | 14209 | |
| Itgbl1 | 0.55984 | -0.024947 | 14210 | |
| 4930557A04Rik | 0.55987 | -0.053563 | 14211 | |
| Phlda3 | 0.5599 | 0.16202 | 14212 | |
| E330017A01Rik | 0.56 | 0.55796 | 14213 | |
| Tex29 | 0.56008 | 0.5758 | 14214 | |
| Hist2h4 | 0.56018 | 0.33677 | 14215 | |
| Ptprt | 0.56025 | 0.41197 | 14216 | |
| Coq3 | 0.56058 | 0.45883 | 14217 | |
| Cd164l2 | 0.56074 | 0.30979 | 14218 | |
| mmu-mir-5619 | 0.56088 | 0.68354 | 14219 | |
| Olfr1176 | 0.56096 | -0.095144 | 14220 | |
| mmu-mir-6923 | 0.56098 | -0.071817 | 14221 | |
| Pde6g | 0.56103 | 0.3411 | 14222 | |
| mmu-mir-7686 | 0.56111 | -0.033337 | 14223 | |
| Zfp746 | 0.56124 | 0.17976 | 14224 | |
| Prss57 | 0.56132 | -0.080898 | 14225 | |
| Eif2ak3 | 0.5615 | 0.15504 | 14226 | |
| Mpv17 | 0.56155 | 1.6646 | 14227 | |
| Timm13 | 0.56158 | 1.4895 | 14228 | |
| Ap1m2 | 0.56168 | -0.034265 | 14229 | |
| Fut10 | 0.56171 | 1.6431 | 14230 | |
| Trh | 0.56174 | 0.058495 | 14231 | |
| Gm17689 | 0.56178 | -0.4146 | 14232 | |
| mmu-mir-299a | 0.56188 | -0.25836 | 14233 | |
| Zar1l | 0.56192 | -0.31474 | 14234 | |
| Usp49 | 0.56221 | -0.025347 | 14235 | |
| Ppargc1b | 0.56223 | 1.0769 | 14236 | |
| Tnnt2 | 0.56234 | -0.21407 | 14237 | |
| Fkbp11 | 0.56237 | 0.49644 | 14238 | |
| Lypd2 | 0.56255 | 0.14696 | 14239 | |
| Pcdhgb1 | 0.56256 | -0.16876 | 14240 | |
| Vmn1r120 | 0.5626 | -0.20195 | 14241 | |
| Csf1 | 0.56273 | 1.2246 | 14242 | |
| Rab17 | 0.56301 | 1.0916 | 14243 | |
| Lpcat3 | 0.56305 | 1.7778 | 14244 | |
| Arid3c | 0.56318 | -0.32917 | 14245 | |
| Sdr9c7 | 0.56323 | 1.6636 | 14246 | |
| Kcnj12 | 0.56331 | -0.39969 | 14247 | |
| Slc25a10 | 0.56355 | 0.75643 | 14248 | |
| A630095N17Rik | 0.5637 | -0.26193 | 14249 | |
| Tbxas1 | 0.56373 | 0.022518 | 14250 | |
| Acot13 | 0.56386 | -0.1644 | 14251 | |
| Dnajc14 | 0.56391 | 1.6607 | 14252 | |
| Gcg | 0.56407 | 0.10341 | 14253 | |
| Myadm | 0.5643 | 0.58289 | 14254 | |
| Serpinb9g | 0.56438 | 0.23164 | 14255 | |
| Klc3 | 0.56444 | 1.3323 | 14256 | |
| Prl7a1 | 0.56457 | -0.010915 | 14257 | |
| Hs3st4 | 0.56459 | 0.79627 | 14258 | |
| Lrrc17 | 0.56467 | 0.077879 | 14259 | |
| Socs6 | 0.5647 | 0.19163 | 14260 | |
| Colgalt2 | 0.56475 | 0.13187 | 14261 | |
| Gm20767 | 0.56483 | -0.067849 | 14262 | |
| Ifi203 | 0.56485 | -0.11314 | 14263 | |
| Frat1 | 0.56493 | -0.25397 | 14264 | |
| Ost4 | 0.56504 | 1.6378 | 14265 | |
| Pou6f1 | 0.56504 | 0.12291 | 14266 | |
| Rabgap1l | 0.56509 | 0.05117 | 14267 | |
| Krt26 | 0.56527 | 0.49244 | 14268 | |
| Lypd4 | 0.56538 | 0.68655 | 14269 | |
| Zfpm1 | 0.56543 | 0.21687 | 14270 | |
| Taar7e | 0.56548 | 0.61524 | 14271 | |
| Mapk9 | 0.56559 | 0.19916 | 14272 | |
| Pramel3 | 0.56561 | -0.034337 | 14273 | |
| Murc | 0.56572 | 0.029702 | 14274 | |
| Mab21l3 | 0.56577 | 0.57464 | 14275 | |
| Zdhhc23 | 0.56579 | -0.30993 | 14276 | |
| Olfr46 | 0.56603 | 0.20547 | 14277 | |
| Ccl8 | 0.56606 | 0.067089 | 14278 | |
| Ap2a1 | 0.56613 | 0.22714 | 14279 | |
| Clec4a2 | 0.56624 | 0.62564 | 14280 | |
| Zfp446 | 0.56632 | 0.45881 | 14281 | |
| Pcdhb17 | 0.56647 | 0.21787 | 14282 | |
| Frat2 | 0.56658 | -0.11215 | 14283 | |
| Hcrtr1 | 0.56671 | 0.55585 | 14284 | |
| Zbtb39 | 0.56682 | -0.14952 | 14285 | |
| 4933405L10Rik | 0.56694 | 0.068454 | 14286 | |
| mmu-mir-7048 | 0.56701 | 0.0065861 | 14287 | |
| Kcnh1 | 0.56702 | 0.23254 | 14288 | |
| Dzip3 | 0.5671 | 0.21227 | 14289 | |
| Fam110b | 0.56723 | 1.4332 | 14290 | |
| Olfr339 | 0.56731 | 0.16979 | 14291 | |
| Olfr1331 | 0.56733 | -0.10895 | 14292 | |
| Pla2r1 | 0.56744 | 1.1092 | 14293 | |
| Eif2ak1 | 0.56749 | 0.030704 | 14294 | |
| Olfr584 | 0.56751 | 0.73573 | 14295 | |
| Hoxd9 | 0.56754 | -0.3728 | 14296 | |
| Rab28 | 0.56767 | 1.1939 | 14297 | |
| Olfr1329 | 0.56785 | 0.36476 | 14298 | |
| Kcnk7 | 0.56788 | -0.17398 | 14299 | |
| Nkx6-3 | 0.56822 | 3.084 | 14300 | |
| Tfeb | 0.56829 | 0.091694 | 14301 | |
| Zranb3 | 0.56832 | 0.30051 | 14302 | |
| Pcid2 | 0.56866 | 0.95385 | 14303 | |
| Tnrc6c | 0.56876 | -0.24239 | 14304 | |
| Ccz1 | 0.56909 | 0.33961 | 14305 | |
| Ccr8 | 0.5692 | 1.5254 | 14306 | |
| Slfnl1 | 0.56923 | 0.20745 | 14307 | |
| Tchh | 0.56939 | -0.38019 | 14308 | |
| Olfr101 | 0.56942 | 0.49193 | 14309 | |
| Zfp759 | 0.56965 | 1.1803 | 14310 | |
| Ppp1r14d | 0.5697 | -0.1216 | 14311 | |
| Olfr449 | 0.56972 | -0.3689 | 14312 | |
| BC024139 | 0.56975 | 0.97239 | 14313 | |
| Tmem167 | 0.56988 | 0.42195 | 14314 | |
| Fermt3 | 0.5699 | -0.30877 | 14315 | |
| St8sia4 | 0.56998 | 1.1365 | 14316 | |
| Vmn1r80 | 0.57015 | -0.010702 | 14317 | |
| Gsto1 | 0.57024 | -0.075626 | 14318 | |
| Hs3st6 | 0.57032 | 1.0581 | 14319 | |
| mmu-mir-6931 | 0.57042 | -0.58017 | 14320 | |
| mmu-mir-301b | 0.57062 | -0.28276 | 14321 | |
| Smok2b | 0.57066 | 0.51915 | 14322 | |
| Tbc1d32 | 0.57073 | 0.55101 | 14323 | |
| Calhm1 | 0.57073 | 0.44797 | 14324 | |
| mmu-mir-467b | 0.57076 | -0.13397 | 14325 | |
| Tnr | 0.57079 | 0.68988 | 14326 | |
| St8sia6 | 0.57097 | -0.10369 | 14327 | |
| Gm11563 | 0.57099 | -0.21217 | 14328 | |
| Ttll11 | 0.57112 | -0.10525 | 14329 | |
| LOC100502896 | 0.57114 | -0.24894 | 14330 | |
| Cox7a2 | 0.5713 | 0.69131 | 14331 | |
| Trim9 | 0.57133 | -0.27309 | 14332 | |
| mmu-mir-3547 | 0.57138 | -0.44532 | 14333 | |
| mmu-mir-154 | 0.57143 | 0.046334 | 14334 | |
| Tmem168 | 0.57164 | 0.07652 | 14335 | |
| Mettl3 | 0.57172 | 0.94566 | 14336 | |
| mmu-mir-7039 | 0.57174 | 0.57533 | 14337 | |
| Tcf15 | 0.57208 | -0.014558 | 14338 | |
| G0s2 | 0.57236 | 1.7527 | 14339 | |
| Pfkfb4 | 0.57254 | -0.21367 | 14340 | |
| Pappa2 | 0.5727 | -0.19633 | 14341 | |
| mmu-mir-7225 | 0.5727 | 1.8065 | 14342 | |
| Gpc3 | 0.57273 | 0.13514 | 14343 | |
| Dusp27 | 0.57275 | -0.12518 | 14344 | |
| Tmod2 | 0.57278 | 0.097152 | 14345 | |
| Scgb1b24 | 0.5728 | 1.1324 | 14346 | |
| Ppm1a | 0.57298 | 1.2762 | 14347 | |
| Gprin3 | 0.57314 | 0.031986 | 14348 | |
| Trim65 | 0.57337 | 0.21951 | 14349 | |
| Bad | 0.5735 | -0.21106 | 14350 | |
| Ffar2 | 0.57356 | 0.043133 | 14351 | |
| Carf | 0.57364 | 0.36485 | 14352 | |
| Sirpb1a | 0.57373 | 0.40214 | 14353 | |
| Oxtr | 0.57394 | 0.19933 | 14354 | |
| Capn1 | 0.57396 | 1.0137 | 14355 | |
| Fam229b | 0.57402 | 1.8981 | 14356 | |
| mmu-mir-1199 | 0.57411 | 1.4137 | 14357 | |
| Il17rc | 0.57412 | 0.13626 | 14358 | |
| Cml3 | 0.57425 | 0.41016 | 14359 | |
| Smg1 | 0.57432 | -0.30573 | 14360 | |
| Depdc1a | 0.57435 | 0.22457 | 14361 | |
| Snrk | 0.57448 | 0.13932 | 14362 | |
| Igfbp3 | 0.5745 | 1.6618 | 14363 | |
| Olfr608 | 0.57458 | 0.38799 | 14364 | |
| Sohlh2 | 0.57466 | 0.33013 | 14365 | |
| Pnpla5 | 0.57489 | -0.30222 | 14366 | |
| mmu-mir-8102 | 0.57491 | -0.20159 | 14367 | |
| Gja10 | 0.57494 | 0.63485 | 14368 | |
| Olfr1337 | 0.57505 | 0.31695 | 14369 | |
| Vmn1r113 | 0.57507 | 0.14609 | 14370 | |
| Tigit | 0.5751 | 0.57617 | 14371 | |
| Fgf18 | 0.57534 | -0.087315 | 14372 | |
| Zfp260 | 0.57534 | 0.37898 | 14373 | |
| Mrgprb5 | 0.57546 | 0.22463 | 14374 | |
| Ccdc114 | 0.57556 | 0.91992 | 14375 | |
| Gm129 | 0.57579 | 0.050468 | 14376 | |
| Id4 | 0.576 | 0.25665 | 14377 | |
| Rufy2 | 0.57605 | 0.6947 | 14378 | |
| Col13a1 | 0.57607 | -0.043014 | 14379 | |
| Kctd17 | 0.57615 | 1.2461 | 14380 | |
| Lce3e | 0.5762 | -0.076956 | 14381 | |
| Slc35a5 | 0.57628 | 1.9165 | 14382 | |
| mmu-mir-5120 | 0.57629 | -0.37322 | 14383 | |
| Olfr494 | 0.57646 | 0.29447 | 14384 | |
| Gjb6 | 0.57668 | 0.24411 | 14385 | |
| 2410089E03Rik | 0.57692 | -0.10048 | 14386 | |
| Pitrm1 | 0.5771 | 0.13757 | 14387 | |
| Ly6f | 0.57718 | -0.13635 | 14388 | |
| Gm13103 | 0.57725 | -0.14677 | 14389 | |
| Tubal3 | 0.57732 | 0.96214 | 14390 | |
| Atp6v0a2 | 0.57739 | -0.28165 | 14391 | |
| mmu-mir-6412 | 0.57749 | -0.15583 | 14392 | |
| Steap4 | 0.57764 | 0.18981 | 14393 | |
| H2-M1 | 0.57772 | 0.058117 | 14394 | |
| Stk40 | 0.578 | -0.34466 | 14395 | |
| Adam9 | 0.57807 | -0.0021779 | 14396 | |
| Cdca2 | 0.57818 | -0.15039 | 14397 | |
| Card10 | 0.57833 | 0.12093 | 14398 | |
| Defa5 | 0.57834 | -0.10246 | 14399 | |
| Prok1 | 0.57836 | 1.3666 | 14400 | |
| Scgb2b24 | 0.57838 | 0.66508 | 14401 | |
| Grpr | 0.57853 | 0.85593 | 14402 | |
| AW209491 | 0.57864 | 0.0077449 | 14403 | |
| Ggn | 0.57866 | 0.086291 | 14404 | |
| Ugt2b5 | 0.57869 | -0.13594 | 14405 | |
| mmu-mir-8112 | 0.57884 | -0.27474 | 14406 | |
| mmu-mir-1969 | 0.57899 | 0.31213 | 14407 | |
| Polq | 0.57907 | 0.22445 | 14408 | |
| Hepacam | 0.5794 | 0.47548 | 14409 | |
| Pole2 | 0.57943 | 0.2041 | 14410 | |
| B4galt7 | 0.57945 | 1.3461 | 14411 | |
| Adam22 | 0.57956 | 0.029316 | 14412 | |
| Odf2l | 0.57961 | 0.11745 | 14413 | |
| Sh3kbp1 | 0.57963 | -0.36441 | 14414 | |
| Grhl2 | 0.57971 | 0.015864 | 14415 | |
| 6330409D20Rik | 0.57981 | 0.078369 | 14416 | |
| Surf1 | 0.57985 | -0.15668 | 14417 | |
| Nat8 | 0.58013 | 0.11976 | 14418 | |
| Rnd2 | 0.58025 | -0.14817 | 14419 | |
| Iqcf6 | 0.5803 | 0.36895 | 14420 | |
| Shisa5 | 0.58032 | 0.15947 | 14421 | |
| Lin54 | 0.58043 | -0.33891 | 14422 | |
| Gm2012 | 0.5805 | -0.071488 | 14423 | |
| Serpinf2 | 0.58053 | -0.20439 | 14424 | |
| mmu-mir-201 | 0.58081 | 0.64914 | 14425 | |
| Plin2 | 0.58082 | -0.36659 | 14426 | |
| Fcrla | 0.58101 | 0.68627 | 14427 | |
| Zfp770 | 0.58155 | 0.078699 | 14428 | |
| Plch1 | 0.58157 | -0.10855 | 14429 | |
| Olig3 | 0.58165 | 0.021505 | 14430 | |
| Ttc32 | 0.58193 | 0.20117 | 14431 | |
| AI413582 | 0.58216 | -0.29423 | 14432 | |
| Rhox9 | 0.58221 | 0.028461 | 14433 | |
| mmu-mir-707 | 0.58228 | 0.096382 | 14434 | |
| Fmo2 | 0.58236 | 0.47225 | 14435 | |
| Cdc42se2 | 0.58249 | -0.40363 | 14436 | |
| Glt6d1 | 0.58264 | -0.45666 | 14437 | |
| Cln8 | 0.58293 | 0.60055 | 14438 | |
| Slc25a5 | 0.58305 | 1.3584 | 14439 | |
| mmu-mir-7004 | 0.5831 | 0.37407 | 14440 | |
| Wdr83 | 0.58312 | 0.057858 | 14441 | |
| Scml2 | 0.58324 | 0.19141 | 14442 | |
| Hnf4a | 0.58329 | 0.076616 | 14443 | |
| Neil3 | 0.58339 | 0.54239 | 14444 | |
| Mos | 0.58339 | -0.0032629 | 14445 | |
| Peli2 | 0.58358 | 0.86805 | 14446 | |
| Atxn7l3 | 0.58366 | -0.28804 | 14447 | |
| Mkrn2 | 0.58371 | 0.23511 | 14448 | |
| Tex261 | 0.58383 | 0.7474 | 14449 | |
| Etv1 | 0.58388 | -0.23183 | 14450 | |
| Itgb2 | 0.58391 | 0.70878 | 14451 | |
| mmu-mir-1898 | 0.58395 | 0.065696 | 14452 | |
| Zfp280d | 0.58399 | -0.4088 | 14453 | |
| mmu-mir-1929 | 0.58403 | 0.39405 | 14454 | |
| Faim | 0.58408 | 0.20394 | 14455 | |
| Tmem39a | 0.58419 | 1.5367 | 14456 | |
| mmu-mir-7076 | 0.58432 | -0.17582 | 14457 | |
| Amtn | 0.58459 | 0.53413 | 14458 | |
| mmu-mir-7676-1 | 0.58461 | 0.025733 | 14459 | |
| Vwc2 | 0.5848 | 0.42064 | 14460 | |
| Ndufaf3 | 0.585 | -0.19683 | 14461 | |
| P4ha3 | 0.58523 | 1.2849 | 14462 | |
| Vim | 0.58525 | 0.17938 | 14463 | |
| Bcl2a1b | 0.58535 | 0.65294 | 14464 | |
| Zdhhc19 | 0.58538 | -0.39649 | 14465 | |
| Gm5591 | 0.58556 | 0.67108 | 14466 | |
| Glb1l3 | 0.58563 | 0.48991 | 14467 | |
| Usp30 | 0.58573 | -0.06317 | 14468 | |
| mmu-mir-3057 | 0.5858 | 0.055753 | 14469 | |
| Rasgef1a | 0.58599 | -0.16113 | 14470 | |
| Tfcp2l1 | 0.58606 | 0.67749 | 14471 | |
| Actl7b | 0.58609 | 0.56008 | 14472 | |
| Tyrp1 | 0.58614 | 0.25316 | 14473 | |
| Zfp536 | 0.58642 | -0.12118 | 14474 | |
| Gsdmc2 | 0.58659 | 1.5098 | 14475 | |
| Tmem119 | 0.58664 | 0.71519 | 14476 | |
| Mep1a | 0.58677 | -0.26426 | 14477 | |
| Foxb1 | 0.5868 | 0.85391 | 14478 | |
| Mrc2 | 0.58685 | -0.15884 | 14479 | |
| Ctr9 | 0.5869 | -0.27968 | 14480 | |
| Acbd3 | 0.58695 | 0.1083 | 14481 | |
| Klk8 | 0.58697 | 0.17886 | 14482 | |
| Slc22a2 | 0.58712 | 2.8781 | 14483 | |
| Pcsk9 | 0.5874 | -0.30144 | 14484 | |
| Sox15 | 0.58743 | -0.16784 | 14485 | |
| Idh2 | 0.5875 | -0.0032528 | 14486 | |
| Pbld1 | 0.58763 | 1.0191 | 14487 | |
| Dpysl5 | 0.5877 | 0.43046 | 14488 | |
| Rnaset2b | 0.58773 | -0.038474 | 14489 | |
| Yipf6 | 0.58775 | 0.92398 | 14490 | |
| D11Wsu47e | 0.58783 | 1.3174 | 14491 | |
| Bscl2 | 0.58796 | -0.034891 | 14492 | |
| Gm4498 | 0.58818 | 2.199 | 14493 | |
| mmu-mir-3965 | 0.58819 | -0.4552 | 14494 | |
| Efnb1 | 0.58821 | 1.6667 | 14495 | |
| Hap1 | 0.58832 | 0.0012146 | 14496 | |
| Olfr729 | 0.58838 | -0.37571 | 14497 | |
| Cr1l | 0.58841 | 0.61171 | 14498 | |
| Prnd | 0.58854 | 0.36339 | 14499 | |
| Fzd9 | 0.58856 | 0.20259 | 14500 | |
| Spink2 | 0.58886 | 0.54675 | 14501 | |
| Armcx5 | 0.58898 | 0.014877 | 14502 | |
| Cntnap2 | 0.58904 | 0.45715 | 14503 | |
| Slc5a9 | 0.58909 | 0.27929 | 14504 | |
| mmu-mir-429 | 0.58911 | 0.15307 | 14505 | |
| Hivep3 | 0.58911 | -0.29317 | 14506 | |
| Klf4 | 0.58916 | 0.28973 | 14507 | |
| Hspb8 | 0.58927 | -0.26221 | 14508 | |
| Olfr503 | 0.58932 | 1.2773 | 14509 | |
| Plg | 0.58959 | 0.63781 | 14510 | |
| mmu-mir-1897 | 0.5897 | 0.13684 | 14511 | |
| mmu-mir-425 | 0.58973 | -0.5451 | 14512 | |
| Rab31 | 0.58982 | -0.18888 | 14513 | |
| Csn2 | 0.58987 | 0.66672 | 14514 | |
| St7l | 0.58997 | 0.8861 | 14515 | |
| Snx12 | 0.59003 | 0.037363 | 14516 | |
| mmu-mir-295 | 0.59003 | -0.20376 | 14517 | |
| Cisd1 | 0.59012 | -0.068419 | 14518 | |
| Cd300ld | 0.59022 | -0.11281 | 14519 | |
| Aspscr1 | 0.5904 | -0.26906 | 14520 | |
| Dhrs7b | 0.59047 | 1.0612 | 14521 | |
| Hdac6 | 0.59055 | 0.38065 | 14522 | |
| Emp3 | 0.59061 | 0.13311 | 14523 | |
| Vpreb2 | 0.59089 | 1.4772 | 14524 | |
| Timm17b | 0.59089 | 0.44523 | 14525 | |
| Mzb1 | 0.59117 | 0.32918 | 14526 | |
| mmu-mir-466h | 0.59118 | -0.052471 | 14527 | |
| 2610524H06Rik | 0.59132 | 0.63781 | 14528 | |
| Fundc1 | 0.59147 | 1.0378 | 14529 | |
| Gm11780 | 0.5918 | 1.1201 | 14530 | |
| Thnsl2 | 0.592 | 1.0611 | 14531 | |
| D3Ertd254e | 0.59218 | 1.4161 | 14532 | |
| Ttll1 | 0.5922 | 0.93371 | 14533 | |
| Olfr738 | 0.59223 | 0.76704 | 14534 | |
| Ispd | 0.59228 | -0.0748 | 14535 | |
| Arhgef25 | 0.59243 | 0.36432 | 14536 | |
| Adam5 | 0.59255 | -0.20472 | 14537 | |
| Shoc2 | 0.59265 | 0.095934 | 14538 | |
| mmu-mir-6345 | 0.59276 | -0.23451 | 14539 | |
| Dsc2 | 0.59294 | 0.97537 | 14540 | |
| Vmn1r168 | 0.59323 | 1.8898 | 14541 | |
| BC031181 | 0.59334 | 1.3683 | 14542 | |
| Sc4mol | 0.59353 | 0.069279 | 14543 | |
| Palm2 | 0.59355 | 0.69473 | 14544 | |
| Aoah | 0.59368 | 0.34458 | 14545 | |
| Tas2r117 | 0.5937 | 0.70072 | 14546 | |
| Tmem140 | 0.59373 | 0.1549 | 14547 | |
| Clec2e | 0.5938 | -0.1391 | 14548 | |
| Eng | 0.59385 | 0.039251 | 14549 | |
| Olfr1323 | 0.5939 | -0.10732 | 14550 | |
| Vmn1r197 | 0.5941 | 0.19623 | 14551 | |
| Vnn1 | 0.59437 | 0.0095226 | 14552 | |
| Tbc1d8b | 0.5945 | 0.72278 | 14553 | |
| Cxcl9 | 0.59462 | 0.90551 | 14554 | |
| Atp10b | 0.59469 | 0.063507 | 14555 | |
| Amn | 0.59474 | 0.58895 | 14556 | |
| Pias1 | 0.59487 | 1.2247 | 14557 | |
| Znrd1as | 0.59502 | 0.85483 | 14558 | |
| Fmo1 | 0.59515 | -0.38687 | 14559 | |
| mmu-mir-7221 | 0.59518 | 0.016834 | 14560 | |
| Zfp319 | 0.59521 | 0.52923 | 14561 | |
| Vmn1r70 | 0.59521 | 0.073439 | 14562 | |
| Sprr3 | 0.59535 | 0.015976 | 14563 | |
| Nek6 | 0.59537 | -0.42682 | 14564 | |
| Lrriq4 | 0.59545 | -0.38451 | 14565 | |
| Col20a1 | 0.59547 | 0.75132 | 14566 | |
| Necab3 | 0.5955 | 0.20041 | 14567 | |
| Hba-x | 0.59552 | 0.063658 | 14568 | |
| Ssr3 | 0.59561 | 0.11925 | 14569 | |
| Zfp1 | 0.59561 | 0.26661 | 14570 | |
| Rsph3b | 0.59577 | 0.033288 | 14571 | |
| mmu-mir-544 | 0.59599 | -0.14477 | 14572 | |
| Sult1a1 | 0.59604 | 0.70893 | 14573 | |
| Dnajc27 | 0.5963 | 0.6055 | 14574 | |
| Cbr3 | 0.59649 | 1.3953 | 14575 | |
| Acvr1c | 0.59684 | -0.29677 | 14576 | |
| Chst13 | 0.59689 | 0.82641 | 14577 | |
| Pla2g4b | 0.59691 | -0.05641 | 14578 | |
| mmu-mir-9-1 | 0.59693 | 0.55857 | 14579 | |
| Ccdc8 | 0.59716 | 0.31639 | 14580 | |
| Prss16 | 0.59724 | -0.32466 | 14581 | |
| Ndufs8 | 0.59736 | -0.36615 | 14582 | |
| Hils1 | 0.59739 | -0.35185 | 14583 | |
| Ttc16 | 0.59743 | -0.16986 | 14584 | |
| Ang3 | 0.59762 | 1.1153 | 14585 | |
| Lmx1b | 0.59762 | -0.2127 | 14586 | |
| Pex1 | 0.59766 | 0.27443 | 14587 | |
| Sptbn2 | 0.59773 | 0.47903 | 14588 | |
| Tle3 | 0.59778 | 1.2231 | 14589 | |
| Ankzf1 | 0.59783 | 0.89864 | 14590 | |
| Ankrd32 | 0.59798 | -0.0060305 | 14591 | |
| Pitx2 | 0.59813 | -0.34717 | 14592 | |
| Podnl1 | 0.59815 | 0.42828 | 14593 | |
| Trim42 | 0.59823 | 0.12492 | 14594 | |
| Mybph | 0.59828 | 0.44169 | 14595 | |
| Ctla4 | 0.5986 | -0.33787 | 14596 | |
| Slc34a2 | 0.59872 | 0.12849 | 14597 | |
| Chn2 | 0.59882 | -0.36691 | 14598 | |
| Fbxo24 | 0.5989 | 0.062931 | 14599 | |
| H2afy | 0.59895 | -0.18002 | 14600 | |
| Nkd2 | 0.59905 | 0.57793 | 14601 | |
| Ptpn12 | 0.59907 | 0.99726 | 14602 | |
| L3hypdh | 0.5991 | -0.29697 | 14603 | |
| mmu-mir-5100 | 0.59911 | -0.47652 | 14604 | |
| Zfp229 | 0.59912 | 0.76729 | 14605 | |
| Lonrf1 | 0.59927 | 1.9924 | 14606 | |
| Gprin1 | 0.59934 | -0.14075 | 14607 | |
| Cdk2ap2 | 0.59937 | -0.12617 | 14608 | |
| Spata19 | 0.59947 | 1.1724 | 14609 | |
| Krtap6-1 | 0.59947 | -0.421 | 14610 | |
| Spint5 | 0.59954 | 0.49145 | 14611 | |
| Tmigd1 | 0.59969 | 0.52137 | 14612 | |
| Nt5c | 0.59986 | -0.099689 | 14613 | |
| Nexn | 0.59989 | 0.10797 | 14614 | |
| Bcl6b | 0.59994 | -0.2304 | 14615 | |
| Drd3 | 0.60003 | -0.12169 | 14616 | |
| B020004C17Rik | 0.60011 | -0.32015 | 14617 | |
| Tdp1 | 0.60018 | -0.15663 | 14618 | |
| Chil4 | 0.60033 | -0.38694 | 14619 | |
| Neil1 | 0.60041 | -0.37295 | 14620 | |
| Ndufs6 | 0.60065 | -0.42982 | 14621 | |
| Gar1 | 0.60079 | 2.7831 | 14622 | |
| 1700028K03Rik | 0.6009 | -0.25475 | 14623 | |
| mmu-mir-129-2 | 0.60097 | 0.54207 | 14624 | |
| Arfgef1 | 0.601 | 0.22739 | 14625 | |
| Lhfpl1 | 0.60127 | -0.13004 | 14626 | |
| Clec10a | 0.60137 | 1.2368 | 14627 | |
| Arhgap6 | 0.60147 | 0.87201 | 14628 | |
| Ermp1 | 0.60149 | 0.25858 | 14629 | |
| Moap1 | 0.60152 | 1.5338 | 14630 | |
| Prorsd1 | 0.60156 | -0.17224 | 14631 | |
| Acsm4 | 0.60165 | 0.96117 | 14632 | |
| Rbbp5 | 0.60176 | 0.94014 | 14633 | |
| Cox18 | 0.60184 | 1.0328 | 14634 | |
| Pigm | 0.60195 | 0.0027064 | 14635 | |
| Sgsm2 | 0.60198 | 0.44436 | 14636 | |
| Rbm45 | 0.60208 | 0.34103 | 14637 | |
| E030018B13Rik | 0.60216 | 1.1267 | 14638 | |
| mmu-mir-31 | 0.60218 | -0.45286 | 14639 | |
| Tie1 | 0.60221 | -0.11501 | 14640 | |
| Mgmt | 0.60233 | 0.97736 | 14641 | |
| Tenm1 | 0.60235 | 0.67837 | 14642 | |
| Med16 | 0.60238 | 0.39763 | 14643 | |
| Pex11a | 0.60243 | 0.10413 | 14644 | |
| Zhx3 | 0.60282 | -0.35384 | 14645 | |
| Slc20a2 | 0.60304 | 1.1687 | 14646 | |
| Olfr612 | 0.60312 | 0.34339 | 14647 | |
| Gchfr | 0.60319 | -0.27419 | 14648 | |
| Nhlrc3 | 0.60346 | -0.32427 | 14649 | |
| Hiat1 | 0.60358 | 1.0958 | 14650 | |
| Golga7 | 0.60361 | -0.36664 | 14651 | |
| Ccdc54 | 0.60368 | 0.64508 | 14652 | |
| C1rb | 0.6037 | -0.27291 | 14653 | |
| Rad50 | 0.60375 | 0.3548 | 14654 | |
| Gadl1 | 0.60378 | 2.4809 | 14655 | |
| Olfr167 | 0.60402 | 0.4078 | 14656 | |
| Ephb1 | 0.60412 | -0.29653 | 14657 | |
| Wfdc18 | 0.6042 | -0.2195 | 14658 | |
| Rcl1 | 0.60429 | -0.15177 | 14659 | |
| Tspear | 0.60432 | 1.2825 | 14660 | |
| Mfsd6 | 0.60451 | -0.27352 | 14661 | |
| Bcas1 | 0.60459 | -0.16654 | 14662 | |
| 4922505E12Rik | 0.60461 | 1.6348 | 14663 | |
| 2810417H13Rik | 0.60474 | 0.55407 | 14664 | |
| Vmn1r217 | 0.60481 | 0.45301 | 14665 | |
| Pea15a | 0.60481 | 0.0933 | 14666 | |
| Ankrd44 | 0.60493 | -0.080397 | 14667 | |
| Olfr312 | 0.60503 | 0.72318 | 14668 | |
| Sema5a | 0.60508 | 0.31048 | 14669 | |
| Olfr1469 | 0.60513 | 0.4211 | 14670 | |
| 2900011O08Rik | 0.60527 | 0.54349 | 14671 | |
| 2010109A12Rik | 0.6053 | 0.26988 | 14672 | |
| Vav2 | 0.60542 | -0.3928 | 14673 | |
| Ppp2r3c | 0.60549 | -0.067897 | 14674 | |
| Uba1y | 0.60556 | -0.26967 | 14675 | |
| mmu-mir-467f | 0.60559 | 0.010198 | 14676 | |
| Hook2 | 0.60561 | -0.038467 | 14677 | |
| Tyr | 0.60574 | 0.23159 | 14678 | |
| Ssh1 | 0.60576 | 2.4235 | 14679 | |
| mmu-mir-3102 | 0.60589 | 0.17056 | 14680 | |
| Pyy | 0.60601 | 0.94786 | 14681 | |
| Tff2 | 0.60608 | -0.040911 | 14682 | |
| Tmed4 | 0.60614 | 0.51325 | 14683 | |
| Tbx5 | 0.6062 | 0.44281 | 14684 | |
| mmu-mir-6985 | 0.60621 | -0.21971 | 14685 | |
| Armcx6 | 0.60629 | -0.32706 | 14686 | |
| Pkhd1 | 0.60633 | 0.071109 | 14687 | |
| P2rx5 | 0.6065 | -0.26459 | 14688 | |
| Nuak1 | 0.60662 | -0.39494 | 14689 | |
| AF366264 | 0.60664 | 0.49009 | 14690 | |
| Esp34 | 0.60669 | 0.46841 | 14691 | |
| mmu-mir-500 | 0.60688 | -0.23071 | 14692 | |
| Stub1 | 0.60699 | 0.62458 | 14693 | |
| Ptcd2 | 0.60704 | 1.7035 | 14694 | |
| mmu-mir-329 | 0.60709 | -0.070989 | 14695 | |
| Myo9b | 0.60726 | 1.2016 | 14696 | |
| Stxbp5 | 0.60733 | 0.82638 | 14697 | |
| Clvs1 | 0.60745 | -0.2299 | 14698 | |
| Mid2 | 0.6075 | 1.9196 | 14699 | |
| Tvp23b | 0.60762 | 0.98929 | 14700 | |
| mmu-mir-344-1 | 0.60763 | -0.17745 | 14701 | |
| Shank2 | 0.60767 | 0.20454 | 14702 | |
| Gm5169 | 0.60771 | -0.22323 | 14703 | |
| Galk2 | 0.60777 | 1.6181 | 14704 | |
| Gpr63 | 0.60779 | 1.3395 | 14705 | |
| Ubash3a | 0.60782 | 0.1598 | 14706 | |
| mmu-mir-6907 | 0.6079 | 0.1699 | 14707 | |
| Kdm4b | 0.60791 | 0.010782 | 14708 | |
| Slc14a2 | 0.60806 | -0.16168 | 14709 | |
| Immt | 0.60808 | 0.14723 | 14710 | |
| Rhbdl1 | 0.60823 | 0.085472 | 14711 | |
| Cyp3a13 | 0.6083 | 1.2347 | 14712 | |
| Otud7b | 0.60843 | -0.39452 | 14713 | |
| mmu-mir-6538 | 0.60857 | 0.17678 | 14714 | |
| Atp6v1e2 | 0.60862 | -0.11159 | 14715 | |
| Olfr1148 | 0.60864 | 0.76805 | 14716 | |
| Cela3b | 0.60877 | -0.34862 | 14717 | |
| D17Wsu92e | 0.60884 | 0.28539 | 14718 | |
| Kdm2a | 0.60889 | 1.5128 | 14719 | |
| mmu-mir-6911 | 0.60889 | 0.89601 | 14720 | |
| Olfr1449 | 0.60896 | 1.0485 | 14721 | |
| Tnfsf12Tnfsf13 | 0.60918 | 1.2548 | 14722 | |
| Ccl25 | 0.60928 | 0.61171 | 14723 | |
| Olfr967 | 0.6093 | 0.091823 | 14724 | |
| Zfp943 | 0.60939 | -0.39685 | 14725 | |
| Chia1 | 0.60939 | 0.47646 | 14726 | |
| Wdr47 | 0.60944 | -0.10836 | 14727 | |
| Metrn | 0.60955 | 1.3942 | 14728 | |
| Tas2r103 | 0.60959 | 0.36389 | 14729 | |
| Dctpp1 | 0.60969 | 0.050535 | 14730 | |
| Uts2 | 0.60981 | 0.82314 | 14731 | |
| mmu-mir-148a | 0.60998 | -0.13511 | 14732 | |
| Epm2a | 0.61027 | 1.9585 | 14733 | |
| Krcc1 | 0.61035 | 1.9568 | 14734 | |
| Usp21 | 0.61044 | 0.16325 | 14735 | |
| Camkk1 | 0.61054 | -0.038967 | 14736 | |
| Gpr61 | 0.61071 | 0.073214 | 14737 | |
| mmu-mir-7223 | 0.61075 | 0.085218 | 14738 | |
| Il10rb | 0.61084 | 0.60148 | 14739 | |
| Ehmt1 | 0.61088 | 1.0121 | 14740 | |
| mmu-mir-6910 | 0.6109 | -0.3866 | 14741 | |
| Plekhb1 | 0.61093 | -0.37157 | 14742 | |
| Brms1 | 0.61103 | 0.69581 | 14743 | |
| Vash2 | 0.61112 | 1.3126 | 14744 | |
| Nicn1 | 0.61122 | 0.68359 | 14745 | |
| Atg3 | 0.61124 | -0.1488 | 14746 | |
| Prrc1 | 0.61127 | 0.97466 | 14747 | |
| Tac1 | 0.61148 | 1.1649 | 14748 | |
| Hnrnpr | 0.61161 | 0.092757 | 14749 | |
| Gm8369 | 0.61169 | 0.069568 | 14750 | |
| Cdkn1a | 0.61183 | -0.056992 | 14751 | |
| Desi1 | 0.61197 | 0.15173 | 14752 | |
| Acsm3 | 0.61217 | 0.44538 | 14753 | |
| Ivl | 0.61229 | -0.23043 | 14754 | |
| Olfr689 | 0.61238 | -0.085172 | 14755 | |
| Gbp5 | 0.61241 | 0.57329 | 14756 | |
| Pramel1 | 0.61248 | 0.85009 | 14757 | |
| Sycn | 0.61265 | 0.22399 | 14758 | |
| Defa-rs7 | 0.6127 | 0.47502 | 14759 | |
| Arl3 | 0.61279 | -0.22321 | 14760 | |
| mmu-mir-5618 | 0.6128 | -0.077772 | 14761 | |
| Dlgap4 | 0.61301 | -0.11003 | 14762 | |
| mmu-mir-34c | 0.61305 | 0.11276 | 14763 | |
| Psma8 | 0.61325 | 0.17227 | 14764 | |
| Kcnj10 | 0.61337 | -0.29279 | 14765 | |
| mmu-mir-6361 | 0.61342 | 0.68659 | 14766 | |
| Vmn2r13 | 0.61346 | 1.3238 | 14767 | |
| Vmn1r25 | 0.6135 | 0.10143 | 14768 | |
| Abhd6 | 0.61354 | 0.68823 | 14769 | |
| Mrm1 | 0.61362 | 0.094916 | 14770 | |
| Ffar1 | 0.61366 | -0.29668 | 14771 | |
| Spata2l | 0.61376 | 0.10868 | 14772 | |
| Txndc11 | 0.61398 | -0.27356 | 14773 | |
| Wnt8b | 0.61403 | -0.29996 | 14774 | |
| Hist1h2aa | 0.61405 | 0.13903 | 14775 | |
| Ahcyl1 | 0.61407 | -0.37721 | 14776 | |
| Mknk1 | 0.61422 | 1.1089 | 14777 | |
| Map3k13 | 0.61429 | 0.6723 | 14778 | |
| Dgkk | 0.61434 | -0.18169 | 14779 | |
| Ssxb5 | 0.61445 | -0.2461 | 14780 | |
| Esp6 | 0.61464 | -0.34592 | 14781 | |
| Pstpip2 | 0.61494 | -0.38718 | 14782 | |
| Lrrfip2 | 0.61497 | -0.093593 | 14783 | |
| Tmprss11f | 0.61512 | 0.39016 | 14784 | |
| Zdhhc18 | 0.61518 | 0.83774 | 14785 | |
| Ambp | 0.61526 | 0.42958 | 14786 | |
| Cml2 | 0.61532 | -0.031287 | 14787 | |
| Cyp4a32 | 0.61532 | 0.061116 | 14788 | |
| Sssca1 | 0.61535 | 0.21508 | 14789 | |
| Zc3h12a | 0.61547 | 0.56604 | 14790 | |
| mmu-mir-670 | 0.61557 | -0.30131 | 14791 | |
| Pcdhga5 | 0.61565 | -0.27983 | 14792 | |
| Lin52 | 0.61571 | -0.20682 | 14793 | |
| Bag6 | 0.61605 | -0.29605 | 14794 | |
| Larp6 | 0.6161 | -0.17209 | 14795 | |
| Gpr39 | 0.61641 | -0.079163 | 14796 | |
| 2310022A10Rik | 0.61653 | -0.35664 | 14797 | |
| Lhb | 0.61665 | 0.033134 | 14798 | |
| Fam166a | 0.61667 | -0.34551 | 14799 | |
| Orm3 | 0.6167 | 0.34154 | 14800 | |
| Gm3258 | 0.61672 | -0.028226 | 14801 | |
| Dmd | 0.61701 | 0.38249 | 14802 | |
| Pigb | 0.61718 | -0.28115 | 14803 | |
| Vmn2r111 | 0.61723 | -0.24412 | 14804 | |
| mmu-mir-6394 | 0.61729 | -0.28579 | 14805 | |
| Tatdn3 | 0.61732 | -0.18936 | 14806 | |
| Nin | 0.61736 | -0.037311 | 14807 | |
| Defb19 | 0.61736 | -0.2935 | 14808 | |
| mmu-mir-7020 | 0.61736 | 0.40754 | 14809 | |
| Tnks2 | 0.61739 | 0.18831 | 14810 | |
| Tagln3 | 0.61744 | -0.24477 | 14811 | |
| Ctbp2 | 0.61761 | 1.03 | 14812 | |
| mmu-mir-6959 | 0.61771 | 0.37426 | 14813 | |
| Slc35b3 | 0.61775 | 0.082981 | 14814 | |
| Mageb2 | 0.61777 | 0.40765 | 14815 | |
| Prss32 | 0.61778 | -0.0013107 | 14816 | |
| Mtif3 | 0.61785 | -0.17704 | 14817 | |
| mmu-mir-453 | 0.61791 | 0.218 | 14818 | |
| Dck | 0.61799 | 0.3743 | 14819 | |
| Cndp2 | 0.61847 | -0.25697 | 14820 | |
| Wdr19 | 0.6185 | 1.3647 | 14821 | |
| Gm17384 | 0.61862 | -0.11026 | 14822 | |
| Bcl7b | 0.61869 | 0.37046 | 14823 | |
| Psmf1 | 0.61871 | -0.36401 | 14824 | |
| Rbm24 | 0.61874 | -0.32231 | 14825 | |
| Gltp | 0.61907 | -0.19782 | 14826 | |
| Ccar1 | 0.61917 | 1.3122 | 14827 | |
| Lhx5 | 0.61921 | 0.50123 | 14828 | |
| Gatsl2 | 0.61931 | 0.051294 | 14829 | |
| Pla2g2e | 0.61938 | -0.12988 | 14830 | |
| Creb3 | 0.61953 | -0.087115 | 14831 | |
| Cir1 | 0.61957 | 0.14248 | 14832 | |
| Ahcyl2 | 0.61965 | -0.11764 | 14833 | |
| Htr6 | 0.61972 | -0.076165 | 14834 | |
| Fam136a | 0.61976 | 0.61861 | 14835 | |
| mmu-mir-6947 | 0.61987 | 0.63894 | 14836 | |
| Cygb | 0.61993 | -0.27451 | 14837 | |
| Il2rb | 0.62002 | -0.40796 | 14838 | |
| Sorbs2 | 0.62005 | -0.32698 | 14839 | |
| Kcnh6 | 0.6201 | -0.29448 | 14840 | |
| Hrh2 | 0.62022 | 0.27834 | 14841 | |
| E130309D14Rik | 0.62024 | 0.13682 | 14842 | |
| Aggf1 | 0.62029 | -0.21388 | 14843 | |
| Olfr32 | 0.62031 | 0.18734 | 14844 | |
| Il6 | 0.62036 | 0.96696 | 14845 | |
| Zfp418 | 0.62043 | 0.20322 | 14846 | |
| Gp49a | 0.62048 | -0.24586 | 14847 | |
| mmu-mir-6237 | 0.62056 | -0.2019 | 14848 | |
| Capn12 | 0.62089 | 0.94259 | 14849 | |
| Nox4 | 0.62093 | -0.11016 | 14850 | |
| Wscd2 | 0.62098 | 0.58437 | 14851 | |
| Rsbn1l | 0.62101 | -0.033903 | 14852 | |
| Vwa7 | 0.62105 | 0.29777 | 14853 | |
| Olfr782 | 0.62108 | -0.12842 | 14854 | |
| Plagl1 | 0.62122 | -0.33811 | 14855 | |
| Idh1 | 0.62134 | 0.34021 | 14856 | |
| B3galt2 | 0.62136 | 0.36702 | 14857 | |
| Dfna5 | 0.62139 | 0.10306 | 14858 | |
| Grina | 0.62143 | -0.29564 | 14859 | |
| Kcnc1 | 0.62162 | -0.25623 | 14860 | |
| Tceal3 | 0.62174 | 0.95878 | 14861 | |
| Inpp5d | 0.62182 | 1.9559 | 14862 | |
| Gas2l2 | 0.6219 | 0.62503 | 14863 | |
| Trap1 | 0.62193 | 0.43445 | 14864 | |
| Clec2l | 0.62198 | -0.27664 | 14865 | |
| Ccdc82 | 0.62215 | 0.39335 | 14866 | |
| Nprl2 | 0.62217 | 0.68672 | 14867 | |
| Bpifa3 | 0.62221 | -0.084327 | 14868 | |
| Rbpms | 0.62224 | 0.22653 | 14869 | |
| Phactr4 | 0.62234 | 0.038751 | 14870 | |
| Lrp6 | 0.62239 | 1.2205 | 14871 | |
| Anxa13 | 0.62265 | -0.44182 | 14872 | |
| Ren1 | 0.6227 | 1.0117 | 14873 | |
| Nmral1 | 0.62274 | -0.13628 | 14874 | |
| Taf3 | 0.62277 | -0.21104 | 14875 | |
| Slc22a23 | 0.62279 | 0.0056109 | 14876 | |
| Mbd3l2 | 0.62281 | 0.10059 | 14877 | |
| Dyx1c1 | 0.62303 | -0.11584 | 14878 | |
| Gstt2 | 0.62308 | 0.48968 | 14879 | |
| Mss51 | 0.6231 | 0.50164 | 14880 | |
| Sec14l2 | 0.62312 | -0.047652 | 14881 | |
| Krtap5-4 | 0.62317 | 0.52721 | 14882 | |
| Faf2 | 0.62329 | 0.24252 | 14883 | |
| Olfr117 | 0.62336 | 0.0092721 | 14884 | |
| Ppp1r14c | 0.62341 | 0.41939 | 14885 | |
| Olfr803 | 0.62341 | 1.2695 | 14886 | |
| Zfp655 | 0.62357 | 0.78177 | 14887 | |
| Olfr211 | 0.62362 | -0.0048314 | 14888 | |
| Ccdc14 | 0.62367 | -0.03649 | 14889 | |
| Pcdhga11 | 0.62381 | 0.10145 | 14890 | |
| Chrnb2 | 0.62384 | 0.44081 | 14891 | |
| Krtap20-2 | 0.62387 | 0.70157 | 14892 | |
| Ccdc169 | 0.62387 | 0.17797 | 14893 | |
| Gja8 | 0.62393 | 0.7802 | 14894 | |
| Coro1b | 0.62395 | 0.61285 | 14895 | |
| Dhrs11 | 0.62405 | 0.072317 | 14896 | |
| Pnldc1 | 0.62407 | -0.14243 | 14897 | |
| Olfr1162 | 0.62433 | 0.0271 | 14898 | |
| Hrct1 | 0.62438 | 0.64225 | 14899 | |
| Cyhr1 | 0.6244 | -0.20248 | 14900 | |
| Olfr771 | 0.62452 | 2.2558 | 14901 | |
| mmu-mir-669d-2 | 0.62455 | 0.17049 | 14902 | |
| Vmn2r26 | 0.62466 | 0.5182 | 14903 | |
| 4930486L24Rik | 0.62476 | 0.15769 | 14904 | |
| Tnfsf14 | 0.62485 | 0.10983 | 14905 | |
| Papss1 | 0.62488 | 0.017639 | 14906 | |
| Lbr | 0.625 | 0.00084234 | 14907 | |
| AI607873 | 0.62513 | 0.72273 | 14908 | |
| Nhs | 0.62516 | -0.0877 | 14909 | |
| Snx33 | 0.6254 | 1.1036 | 14910 | |
| mmu-mir-132 | 0.62543 | 0.17256 | 14911 | |
| Lrpprc | 0.62552 | -0.43611 | 14912 | |
| Med28 | 0.62559 | 0.97323 | 14913 | |
| Gm20822 | 0.62563 | 1.2515 | 14914 | |
| Olfr564 | 0.62582 | -0.18255 | 14915 | |
| 4933434I20Rik | 0.62613 | -0.3487 | 14916 | |
| Bnip2 | 0.62621 | -0.39493 | 14917 | |
| Crebzf | 0.62639 | -0.087151 | 14918 | |
| Sdccag3 | 0.62644 | -0.23471 | 14919 | |
| Matn4 | 0.62654 | -0.20543 | 14920 | |
| Acy1 | 0.62658 | -0.27772 | 14921 | |
| Itpr1 | 0.6266 | 0.472 | 14922 | |
| Igf2bp2 | 0.62686 | 0.93536 | 14923 | |
| Fam160a1 | 0.62689 | 0.78535 | 14924 | |
| Ensa | 0.62697 | 0.043237 | 14925 | |
| Micu1 | 0.627 | -0.28701 | 14926 | |
| mmu-mir-6404 | 0.62706 | -0.35373 | 14927 | |
| Zak | 0.6271 | 0.54611 | 14928 | |
| Nfia | 0.62715 | 1.2153 | 14929 | |
| Vmn2r100 | 0.62719 | -0.41836 | 14930 | |
| mmu-mir-23b | 0.62721 | -0.066865 | 14931 | |
| Oscp1 | 0.62722 | 0.8603 | 14932 | |
| Dlg4 | 0.62724 | 0.97705 | 14933 | |
| Nmur2 | 0.62726 | 0.027839 | 14934 | |
| Fbxw7 | 0.62729 | -0.14033 | 14935 | |
| Itm2a | 0.62762 | 0.6202 | 14936 | |
| mmu-mir-383 | 0.62762 | -0.1427 | 14937 | |
| Pex26 | 0.62774 | -0.26369 | 14938 | |
| mmu-mir-7046 | 0.62795 | 0.53693 | 14939 | |
| Gla | 0.62799 | -0.041504 | 14940 | |
| Olfr319 | 0.62808 | -0.42783 | 14941 | |
| Nkg7 | 0.62808 | 0.44765 | 14942 | |
| mmu-mir-883a | 0.62823 | -0.51331 | 14943 | |
| Ubac2 | 0.62828 | -0.059207 | 14944 | |
| Golt1a | 0.62846 | 0.49265 | 14945 | |
| Olfr1164 | 0.62854 | -0.31249 | 14946 | |
| Vgll2 | 0.62858 | 0.30413 | 14947 | |
| Defb38 | 0.62884 | 1.4828 | 14948 | |
| Daam2 | 0.62886 | -0.42862 | 14949 | |
| Plau | 0.62924 | 1.4306 | 14950 | |
| Sema4d | 0.6294 | -0.051887 | 14951 | |
| Cd274 | 0.62959 | 0.37045 | 14952 | |
| mmu-mir-186 | 0.6296 | 0.73775 | 14953 | |
| Donson | 0.62984 | 0.027029 | 14954 | |
| Brsk2 | 0.63011 | 0.56904 | 14955 | |
| Tmem69 | 0.63013 | 0.76652 | 14956 | |
| Myom1 | 0.63025 | 0.15109 | 14957 | |
| Ccp110 | 0.6303 | -0.31647 | 14958 | |
| Slc7a2 | 0.63043 | 0.090771 | 14959 | |
| Srgap2 | 0.63051 | 0.10513 | 14960 | |
| Sv2c | 0.63067 | -0.38162 | 14961 | |
| Timm17a | 0.6307 | 0.9463 | 14962 | |
| Mrpl33 | 0.63075 | 0.13799 | 14963 | |
| mmu-mir-338 | 0.63084 | 0.25498 | 14964 | |
| Olfr1348 | 0.63088 | 0.57603 | 14965 | |
| Mbl1 | 0.63091 | 0.69983 | 14966 | |
| Uts2r | 0.63103 | -0.32752 | 14967 | |
| Hexa | 0.63109 | 0.34666 | 14968 | |
| Olfr819 | 0.63114 | 0.97312 | 14969 | |
| Shisa9 | 0.63139 | 0.61904 | 14970 | |
| Hagh | 0.63147 | 0.28445 | 14971 | |
| Tslp | 0.63151 | 0.99963 | 14972 | |
| Ar | 0.63161 | -0.046642 | 14973 | |
| Speer4d | 0.63182 | 0.71681 | 14974 | |
| Lrp2 | 0.63191 | -0.13973 | 14975 | |
| Gcgr | 0.63198 | 0.10805 | 14976 | |
| Fcgr4 | 0.63201 | 0.27056 | 14977 | |
| Clhc1 | 0.63203 | 1.0378 | 14978 | |
| Kctd11 | 0.6321 | -0.030261 | 14979 | |
| Aldh1a3 | 0.63219 | 1.2448 | 14980 | |
| Phc1 | 0.63224 | 0.06054 | 14981 | |
| Olfr1465 | 0.63231 | 0.15769 | 14982 | |
| Gm7073 | 0.63238 | 0.77545 | 14983 | |
| Abcc3 | 0.63245 | 0.6108 | 14984 | |
| Mtss1l | 0.63254 | 0.085082 | 14985 | |
| Mr1 | 0.63261 | 0.070029 | 14986 | |
| Thnsl1 | 0.63264 | 0.28838 | 14987 | |
| Mcoln1 | 0.63287 | 1.5443 | 14988 | |
| mmu-mir-195b | 0.63287 | -0.46822 | 14989 | |
| D3Bwg0562e | 0.63289 | 1.0734 | 14990 | |
| Kdm5d | 0.63292 | 0.053194 | 14991 | |
| Abhd3 | 0.6331 | 0.31551 | 14992 | |
| 1810043G02Rik | 0.63313 | 0.83892 | 14993 | |
| Zfp574 | 0.63315 | 0.11929 | 14994 | |
| Olfr1370 | 0.6332 | 0.0075758 | 14995 | |
| Foxj1 | 0.63338 | 0.22685 | 14996 | |
| Dhrs4 | 0.63341 | -0.028985 | 14997 | |
| mmu-mir-6364 | 0.63342 | -0.036496 | 14998 | |
| Eomes | 0.63345 | 1.0263 | 14999 | |
| Ciapin1 | 0.63358 | 0.10325 | 15000 | |
| Frmd4b | 0.63362 | -0.13324 | 15001 | |
| Hspa4l | 0.63364 | 0.33291 | 15002 | |
| Phf11b | 0.63369 | 0.55579 | 15003 | |
| Wdr17 | 0.63373 | 1.9314 | 15004 | |
| Olfr493 | 0.63376 | 0.0068759 | 15005 | |
| Kcnt2 | 0.63388 | -0.35926 | 15006 | |
| Ifit2 | 0.63397 | 1.7013 | 15007 | |
| Slc13a2 | 0.63399 | 0.24799 | 15008 | |
| Xpo6 | 0.63419 | -0.17054 | 15009 | |
| Lpgat1 | 0.63427 | 0.71656 | 15010 | |
| Rundc3b | 0.63464 | -0.11688 | 15011 | |
| Dock1 | 0.63476 | -0.42728 | 15012 | |
| Cpb1 | 0.63485 | 0.41434 | 15013 | |
| Zbp1 | 0.63492 | -0.097993 | 15014 | |
| Plekhm1 | 0.635 | -0.40506 | 15015 | |
| Zdhhc1 | 0.63515 | 0.45701 | 15016 | |
| Mreg | 0.63538 | 1.9467 | 15017 | |
| Fam219a | 0.63558 | -0.37602 | 15018 | |
| Col5a1 | 0.63564 | 0.30083 | 15019 | |
| Atp6v1c2 | 0.63571 | -0.36277 | 15020 | |
| Ptbp2 | 0.63578 | 0.56386 | 15021 | |
| Mfge8 | 0.63584 | -0.016975 | 15022 | |
| Cwf19l2 | 0.63599 | 1.3574 | 15023 | |
| Bcat2 | 0.63601 | -0.081492 | 15024 | |
| mmu-mir-764 | 0.6361 | 0.14863 | 15025 | |
| Vmn1r71 | 0.63615 | 0.32091 | 15026 | |
| Trpc6 | 0.63627 | -0.11235 | 15027 | |
| Fbxo43 | 0.63636 | 0.88812 | 15028 | |
| Col7a1 | 0.63659 | 0.25651 | 15029 | |
| Elavl4 | 0.63671 | 0.50036 | 15030 | |
| Csgalnact2 | 0.63675 | 0.94312 | 15031 | |
| 3-Mar | 0.6368 | 2.4362 | 15032 | |
| Rbfox2 | 0.63685 | 0.67751 | 15033 | |
| 4933417A18Rik | 0.63696 | -0.20145 | 15034 | |
| Hsd17b13 | 0.637 | -0.095487 | 15035 | |
| Itfg1 | 0.63703 | -0.056667 | 15036 | |
| Btg1 | 0.63705 | 2.1909 | 15037 | |
| Aqp9 | 0.63719 | -0.1974 | 15038 | |
| Gab2 | 0.63726 | -0.15244 | 15039 | |
| Olfml2b | 0.6374 | 1.0537 | 15040 | |
| Sytl5 | 0.63749 | -0.4067 | 15041 | |
| Eps8l1 | 0.63754 | -0.36439 | 15042 | |
| Cutc | 0.63763 | -0.23483 | 15043 | |
| Sec23a | 0.63766 | 0.76034 | 15044 | |
| Taar5 | 0.63769 | 0.10205 | 15045 | |
| Foxd4 | 0.63769 | 0.18971 | 15046 | |
| Dcdc2b | 0.63775 | 0.26409 | 15047 | |
| Olfr38 | 0.63777 | -0.30287 | 15048 | |
| Mbnl1 | 0.63782 | 1.0046 | 15049 | |
| Amotl2 | 0.63784 | 1.5618 | 15050 | |
| Ap1s3 | 0.63791 | 0.60116 | 15051 | |
| Mthfd2 | 0.63791 | 0.019039 | 15052 | |
| Sbno2 | 0.638 | 0.71489 | 15053 | |
| Esp36 | 0.63804 | 0.14732 | 15054 | |
| Dse | 0.63807 | -0.042068 | 15055 | |
| Gm13177 | 0.63821 | 0.96882 | 15056 | |
| Prss2 | 0.63837 | -0.23064 | 15057 | |
| Bag4 | 0.6384 | 0.2312 | 15058 | |
| mmu-mir-1934 | 0.63841 | -0.36564 | 15059 | |
| Hs3st5 | 0.63847 | 2.2838 | 15060 | |
| Spink5 | 0.63851 | 0.026601 | 15061 | |
| Gm17252 | 0.63856 | -0.16377 | 15062 | |
| 2410012M07Rik | 0.63858 | -0.31533 | 15063 | |
| Thap11 | 0.63881 | 0.48249 | 15064 | |
| Olfr262 | 0.63889 | -0.11026 | 15065 | |
| Chtf8 | 0.639 | 0.46191 | 15066 | |
| Aldoc | 0.639 | 1.3495 | 15067 | |
| Ccdc17 | 0.63909 | 0.75236 | 15068 | |
| Olfr295 | 0.6392 | -0.052478 | 15069 | |
| Sec14l4 | 0.63937 | 0.069113 | 15070 | |
| Ap1g1 | 0.63953 | 0.5962 | 15071 | |
| Cd6 | 0.63955 | 0.51231 | 15072 | |
| mmu-mir-1936 | 0.63964 | 0.032268 | 15073 | |
| Cel | 0.63968 | 0.59139 | 15074 | |
| mmu-mir-142b | 0.63969 | 0.043389 | 15075 | |
| H2-M5 | 0.63978 | -0.34046 | 15076 | |
| E030030I06Rik | 0.63983 | 0.69524 | 15077 | |
| mmu-mir-6973b | 0.63983 | 0.29953 | 15078 | |
| Nxnl2 | 0.63985 | 1.6147 | 15079 | |
| mmu-mir-509 | 0.63986 | 0.29347 | 15080 | |
| Olfr1233 | 0.6399 | -0.30285 | 15081 | |
| Galr1 | 0.63996 | 0.03293 | 15082 | |
| Ttyh2 | 0.64001 | 1.0156 | 15083 | |
| Arpc1a | 0.6402 | 0.018713 | 15084 | |
| Rab19 | 0.64022 | -0.20477 | 15085 | |
| 4930415F15Rik | 0.64033 | -0.42492 | 15086 | |
| Tbx19 | 0.64052 | 0.008406 | 15087 | |
| Parp16 | 0.64056 | 0.53561 | 15088 | |
| Apoc3 | 0.64075 | -0.2896 | 15089 | |
| Bcl3 | 0.64086 | -0.25203 | 15090 | |
| mmu-mir-27a | 0.64089 | -0.41019 | 15091 | |
| Pon2 | 0.64116 | 0.16689 | 15092 | |
| Vmn2r109 | 0.64118 | -0.39813 | 15093 | |
| Igsf9 | 0.64123 | -0.080942 | 15094 | |
| Zfp276 | 0.64125 | 0.053997 | 15095 | |
| Klhdc7a | 0.64132 | -0.40893 | 15096 | |
| Olfr1230 | 0.64135 | -0.18766 | 15097 | |
| Nsun7 | 0.64151 | 0.098018 | 15098 | |
| Echdc1 | 0.64154 | -0.16301 | 15099 | |
| Kcnip4 | 0.64154 | 0.37863 | 15100 | |
| Olfr207 | 0.6416 | -0.0079165 | 15101 | |
| Fbxo9 | 0.64187 | 0.64879 | 15102 | |
| Nrbf2 | 0.64197 | -0.0070657 | 15103 | |
| Nfatc1 | 0.64219 | -0.28862 | 15104 | |
| AF251705 | 0.64226 | 0.89801 | 15105 | |
| Pou4f3 | 0.64229 | -0.1978 | 15106 | |
| Olfr1424 | 0.64232 | 0.62095 | 15107 | |
| Nrn1l | 0.64232 | -0.022988 | 15108 | |
| 4933402J07Rik | 0.64236 | -0.41601 | 15109 | |
| Fdx1 | 0.6424 | 0.11904 | 15110 | |
| Mdm4 | 0.64247 | -0.30223 | 15111 | |
| Ggt6 | 0.64249 | 0.13508 | 15112 | |
| mmu-mir-7082 | 0.6425 | -0.031564 | 15113 | |
| Ly75 | 0.64252 | -0.18383 | 15114 | |
| 1700080E11Rik | 0.64265 | 2.2732 | 15115 | |
| Pcdhga8 | 0.64266 | 0.10628 | 15116 | |
| Adamts18 | 0.64268 | 1.1268 | 15117 | |
| Dclk2 | 0.64276 | 1.4273 | 15118 | |
| Kcne4 | 0.64297 | 0.15734 | 15119 | |
| Mpl | 0.64304 | 1.1453 | 15120 | |
| Tada2b | 0.64307 | 1.1689 | 15121 | |
| mmu-mir-6368 | 0.64307 | -0.24542 | 15122 | |
| Znrf4 | 0.64313 | -0.23216 | 15123 | |
| mmu-mir-8116 | 0.64315 | -0.26103 | 15124 | |
| Ptgdr | 0.64318 | -0.31548 | 15125 | |
| mmu-mir-190a | 0.64363 | 0.98513 | 15126 | |
| Gm15881 | 0.64368 | 1.319 | 15127 | |
| Ankrd39 | 0.64373 | 0.063612 | 15128 | |
| Mmp11 | 0.64376 | 1.9276 | 15129 | |
| B630005N14Rik | 0.64396 | 0.85527 | 15130 | |
| Gm12171 | 0.64407 | 0.78939 | 15131 | |
| Tpcn1 | 0.6441 | 1.993 | 15132 | |
| Pdcd1lg2 | 0.64413 | -0.38671 | 15133 | |
| Slc45a2 | 0.64421 | -0.29656 | 15134 | |
| mmu-mir-6991 | 0.64425 | -0.0025855 | 15135 | |
| Dcaf4 | 0.6443 | -0.37692 | 15136 | |
| 1110057K04Rik | 0.64439 | 0.44 | 15137 | |
| Lrcol1 | 0.64442 | -0.2299 | 15138 | |
| Ikzf4 | 0.64446 | 1.2351 | 15139 | |
| Colq | 0.64448 | 1.1817 | 15140 | |
| Rnf112 | 0.64455 | 0.10297 | 15141 | |
| Il34 | 0.64463 | 0.87243 | 15142 | |
| A330070K13Rik | 0.64487 | 0.32462 | 15143 | |
| Aldh2 | 0.64494 | 2.0329 | 15144 | |
| 2300009A05Rik | 0.64499 | 1.4697 | 15145 | |
| Gpd1 | 0.64501 | 0.3394 | 15146 | |
| Cryzl1 | 0.64503 | 0.3007 | 15147 | |
| Cdr2l | 0.64505 | -0.13575 | 15148 | |
| Cdpf1 | 0.64515 | 0.098395 | 15149 | |
| Il15ra | 0.64535 | -0.13215 | 15150 | |
| Hoxc13 | 0.6454 | -0.10471 | 15151 | |
| Vmn1r103 | 0.64542 | 0.15602 | 15152 | |
| Chd7 | 0.64547 | 2.0116 | 15153 | |
| Rptor | 0.64549 | 0.46401 | 15154 | |
| mmu-mir-493 | 0.6455 | 0.11598 | 15155 | |
| Rasef | 0.64558 | 0.76046 | 15156 | |
| Sfrp2 | 0.64566 | -0.16632 | 15157 | |
| Trim30b | 0.64583 | -0.20198 | 15158 | |
| Tmem258 | 0.64589 | -0.44199 | 15159 | |
| 1700007B14Rik | 0.64589 | 0.17841 | 15160 | |
| Ddit4l | 0.64597 | 1.0978 | 15161 | |
| Dnajc3 | 0.64606 | -0.091918 | 15162 | |
| Try10 | 0.6461 | -0.1304 | 15163 | |
| Psmg1 | 0.64648 | 0.48433 | 15164 | |
| Tspan2 | 0.64663 | 0.25135 | 15165 | |
| mmu-mir-215 | 0.64667 | -0.26041 | 15166 | |
| Wdr6 | 0.64694 | 0.75747 | 15167 | |
| LOC100503280 | 0.64704 | 0.7516 | 15168 | |
| Opn3 | 0.64708 | 0.65747 | 15169 | |
| Tm4sf5 | 0.64713 | 0.72021 | 15170 | |
| Lama1 | 0.64744 | 0.35184 | 15171 | |
| Slc2a9 | 0.64754 | 0.52596 | 15172 | |
| Olfr389 | 0.64772 | 2.4427 | 15173 | |
| Mstn | 0.64776 | -0.012387 | 15174 | |
| Cyp4f14 | 0.64783 | 0.22571 | 15175 | |
| Fbxo42 | 0.6479 | -0.20451 | 15176 | |
| Slc36a2 | 0.64798 | -0.15757 | 15177 | |
| Tekt3 | 0.64806 | 0.55316 | 15178 | |
| Lpp | 0.64813 | 0.14102 | 15179 | |
| Heyl | 0.64824 | 0.36312 | 15180 | |
| Sept11 | 0.64831 | 0.30822 | 15181 | |
| Cand2 | 0.64833 | 0.1625 | 15182 | |
| Ccdc101 | 0.64844 | 0.53297 | 15183 | |
| Doc2g | 0.64849 | 0.27063 | 15184 | |
| Gm13152 | 0.64856 | 0.39928 | 15185 | |
| Stat5b | 0.64858 | -0.35487 | 15186 | |
| Nudt1 | 0.64874 | -0.21089 | 15187 | |
| Olfr1085 | 0.64883 | 3.3768 | 15188 | |
| Kcnh4 | 0.64885 | 2.3754 | 15189 | |
| Fam120a | 0.64887 | 0.81888 | 15190 | |
| L3mbtl1 | 0.6491 | -0.13681 | 15191 | |
| Aebp2 | 0.64912 | -0.28857 | 15192 | |
| Cyp1a1 | 0.64921 | 1.8336 | 15193 | |
| Rasgef1b | 0.64935 | 0.42903 | 15194 | |
| Vmn1r191 | 0.64944 | 0.090056 | 15195 | |
| Acsl6 | 0.64958 | 0.073938 | 15196 | |
| Lrrc14 | 0.6496 | -0.035384 | 15197 | |
| mmu-mir-804 | 0.64961 | 0.24629 | 15198 | |
| Lrig3 | 0.64978 | -0.27169 | 15199 | |
| Hal | 0.64982 | 0.19043 | 15200 | |
| Agxt | 0.65012 | 3.1527 | 15201 | |
| Olfr845 | 0.65014 | 0.18248 | 15202 | |
| mmu-mir-3962 | 0.65067 | 0.15824 | 15203 | |
| mmu-mir-7215 | 0.65077 | 0.18616 | 15204 | |
| Unc119b | 0.6508 | 0.029212 | 15205 | |
| 9530068E07Rik | 0.65089 | -0.22945 | 15206 | |
| 1700031F05Rik | 0.65093 | 1.2628 | 15207 | |
| Ifnz | 0.65098 | 0.59566 | 15208 | |
| Rp1l1 | 0.65111 | 1.993 | 15209 | |
| Rorb | 0.65118 | -0.41046 | 15210 | |
| Sult5a1 | 0.65127 | 0.24189 | 15211 | |
| mmu-mir-448 | 0.65134 | 0.64229 | 15212 | |
| Rragb | 0.65141 | 0.11532 | 15213 | |
| Dcaf12 | 0.65143 | 0.62281 | 15214 | |
| Nmnat3 | 0.65147 | -0.19553 | 15215 | |
| Iah1 | 0.65161 | 0.91685 | 15216 | |
| 1700008I05Rik | 0.65164 | -0.058383 | 15217 | |
| Egfl7 | 0.65181 | 0.31549 | 15218 | |
| Osbpl5 | 0.65186 | 0.33342 | 15219 | |
| Vmn2r103 | 0.65188 | 0.41133 | 15220 | |
| Gm4461 | 0.65192 | 0.51881 | 15221 | |
| BC030499 | 0.65196 | 0.16205 | 15222 | |
| Spata31d1d | 0.65206 | -0.18352 | 15223 | |
| Rrh | 0.65208 | 0.98742 | 15224 | |
| Tlr11 | 0.65225 | -0.25621 | 15225 | |
| Bnip3l | 0.65225 | 0.34079 | 15226 | |
| H2-Q1 | 0.65233 | -0.30408 | 15227 | |
| Nfix | 0.65235 | 0.53394 | 15228 | |
| Smarcad1 | 0.65237 | 0.32403 | 15229 | |
| Cyp2b19 | 0.65253 | 0.12452 | 15230 | |
| Erg | 0.6526 | -0.27128 | 15231 | |
| Cckbr | 0.65276 | -0.30917 | 15232 | |
| Zfp292 | 0.65278 | 0.24596 | 15233 | |
| Rin3 | 0.65287 | 0.48878 | 15234 | |
| Ginm1 | 0.65303 | 0.47888 | 15235 | |
| Srrt | 0.65318 | 0.6212 | 15236 | |
| Med19 | 0.65321 | -0.072916 | 15237 | |
| Gm15386 | 0.65337 | -0.18092 | 15238 | |
| Klrg2 | 0.65345 | 0.0304 | 15239 | |
| Tbx2 | 0.65357 | 1.0166 | 15240 | |
| Plin1 | 0.6537 | 1.4934 | 15241 | |
| Clec4b1 | 0.65374 | 1.1467 | 15242 | |
| Ampd3 | 0.65381 | -0.13028 | 15243 | |
| Srf | 0.65399 | -0.38035 | 15244 | |
| Asmt | 0.65406 | -0.3502 | 15245 | |
| Atl3 | 0.65422 | 0.22707 | 15246 | |
| Vstm2b | 0.65424 | 0.029316 | 15247 | |
| Tmed5 | 0.65428 | -0.27355 | 15248 | |
| Chd5 | 0.65433 | 0.052139 | 15249 | |
| Oasl1 | 0.65437 | 0.16331 | 15250 | |
| mmu-mir-669d | 0.65441 | -0.25253 | 15251 | |
| Calb2 | 0.65452 | 0.52569 | 15252 | |
| Fhl1 | 0.65455 | 0.20532 | 15253 | |
| Klhdc9 | 0.65464 | 1.1162 | 15254 | |
| Pcdhga3 | 0.65466 | 0.31377 | 15255 | |
| Mapk1ip1 | 0.65478 | 0.079635 | 15256 | |
| Becn1 | 0.65482 | -0.046014 | 15257 | |
| Cd200r1 | 0.65496 | 0.67047 | 15258 | |
| Pgf | 0.655 | -0.036622 | 15259 | |
| Txndc12 | 0.65502 | -0.0098084 | 15260 | |
| Unc45b | 0.65513 | 0.55681 | 15261 | |
| Jade2 | 0.65525 | 0.774 | 15262 | |
| Pter | 0.6554 | 0.28174 | 15263 | |
| Dcaf17 | 0.65543 | 0.070392 | 15264 | |
| Flii | 0.65545 | 0.74173 | 15265 | |
| Rasd1 | 0.65547 | 0.46099 | 15266 | |
| Kcna4 | 0.65553 | -0.29962 | 15267 | |
| Ksr1 | 0.65556 | 1.8667 | 15268 | |
| Tgds | 0.65574 | 1.169 | 15269 | |
| Lama3 | 0.65583 | 1.7307 | 15270 | |
| Fbxl19 | 0.65596 | 2.0189 | 15271 | |
| Ramp2 | 0.65627 | 0.13047 | 15272 | |
| Irf2bpl | 0.65634 | 0.42772 | 15273 | |
| Nmt1 | 0.65654 | 0.28021 | 15274 | |
| Ccl24 | 0.65669 | 1.0643 | 15275 | |
| Prss29 | 0.65674 | 0.34993 | 15276 | |
| Atp9a | 0.65682 | 0.42631 | 15277 | |
| Egf | 0.65712 | -0.13674 | 15278 | |
| Olfr1012 | 0.65743 | -0.035022 | 15279 | |
| Olfr593 | 0.65747 | 0.23056 | 15280 | |
| Vmn1r22 | 0.6576 | -0.4432 | 15281 | |
| mmu-mir-7047 | 0.65767 | 0.61764 | 15282 | |
| Olfr1202 | 0.65767 | 1.3963 | 15283 | |
| Ube2b | 0.65775 | 1.3107 | 15284 | |
| Chuk | 0.65799 | -0.032201 | 15285 | |
| Itgal | 0.65804 | 1.1328 | 15286 | |
| Usf1 | 0.65806 | -0.043512 | 15287 | |
| Atad2 | 0.65828 | 0.57209 | 15288 | |
| mmu-mir-101b | 0.65835 | -0.29643 | 15289 | |
| Fbxo48 | 0.65866 | 0.093661 | 15290 | |
| Rfx3 | 0.65868 | 0.052139 | 15291 | |
| Ficd | 0.6587 | 0.49484 | 15292 | |
| Herc3 | 0.65877 | 0.80364 | 15293 | |
| Wdr52 | 0.65884 | 0.22904 | 15294 | |
| Zfp114 | 0.65885 | -0.45443 | 15295 | |
| Ppfia3 | 0.6589 | 1.366 | 15296 | |
| Dhx32 | 0.65906 | 0.63105 | 15297 | |
| Hexim2 | 0.65908 | 0.079369 | 15298 | |
| Tpra1 | 0.65915 | 1.0288 | 15299 | |
| Tmsb15b1 | 0.65919 | -0.32135 | 15300 | |
| Clmn | 0.65926 | 2.7617 | 15301 | |
| Gm14124 | 0.65945 | -0.32601 | 15302 | |
| St3gal1 | 0.6595 | -0.29015 | 15303 | |
| Apcdd1 | 0.65957 | 0.66712 | 15304 | |
| Cngb1 | 0.6596 | 0.65078 | 15305 | |
| Sdr16c5 | 0.65986 | 1.721 | 15306 | |
| Aloxe3 | 0.65988 | -0.27754 | 15307 | |
| mmu-mir-194-2 | 0.65988 | -0.41628 | 15308 | |
| 0610040J01Rik | 0.65992 | 1.6812 | 15309 | |
| Atp13a1 | 0.66004 | -0.37491 | 15310 | |
| Gm3404 | 0.66017 | 0.14436 | 15311 | |
| mmu-mir-467a-2 | 0.66018 | 0.14983 | 15312 | |
| Pkn1 | 0.66021 | 0.66606 | 15313 | |
| Gm7092 | 0.66023 | 1.3855 | 15314 | |
| Dnajc7 | 0.6603 | -0.055431 | 15315 | |
| Olfr1387 | 0.66055 | 0.29545 | 15316 | |
| mmu-mir-713 | 0.66083 | 0.33854 | 15317 | |
| Mxd4 | 0.66112 | 1.5185 | 15318 | |
| Rnf128 | 0.66114 | -0.15128 | 15319 | |
| Midn | 0.66116 | 0.23971 | 15320 | |
| 2310002L09Rik | 0.6613 | 0.73746 | 15321 | |
| Ms4a4d | 0.66134 | -0.38635 | 15322 | |
| Sema7a | 0.66136 | 0.92951 | 15323 | |
| Klra19 | 0.66149 | 1.0818 | 15324 | |
| Tecta | 0.66172 | -0.39674 | 15325 | |
| Olfr24 | 0.66177 | -0.27302 | 15326 | |
| She | 0.66185 | 0.37184 | 15327 | |
| Chst4 | 0.66189 | -0.35521 | 15328 | |
| Lair1 | 0.66194 | -0.29956 | 15329 | |
| Phyh | 0.66198 | -0.10885 | 15330 | |
| mmu-mir-6996 | 0.66218 | -0.39214 | 15331 | |
| Smagp | 0.66218 | 0.090071 | 15332 | |
| Olfr1311 | 0.66223 | 0.57411 | 15333 | |
| Vmn1r228 | 0.66236 | 0.32284 | 15334 | |
| Sectm1a | 0.66276 | 1.2774 | 15335 | |
| Gpr155 | 0.66279 | 1.1995 | 15336 | |
| Tram2 | 0.66282 | 1.6226 | 15337 | |
| Piwil4 | 0.66293 | -0.41582 | 15338 | |
| Ppp1r17 | 0.66293 | 0.36109 | 15339 | |
| Ddx3y | 0.663 | 0.065179 | 15340 | |
| Pycrl | 0.66304 | -0.24724 | 15341 | |
| Siglec1 | 0.66322 | -0.12851 | 15342 | |
| Clic5 | 0.66339 | 0.2616 | 15343 | |
| Olfr1228 | 0.6635 | 0.59836 | 15344 | |
| Vmn2r81 | 0.66375 | 0.42771 | 15345 | |
| Macrod2 | 0.66377 | 0.13718 | 15346 | |
| Wnt8a | 0.66394 | -0.21388 | 15347 | |
| Colec11 | 0.66414 | -0.26235 | 15348 | |
| Foxj3 | 0.66418 | 1.4797 | 15349 | |
| Gpr146 | 0.66434 | -0.4025 | 15350 | |
| Kif5b | 0.66441 | 0.64194 | 15351 | |
| Ctsj | 0.66444 | -0.33376 | 15352 | |
| Bbs12 | 0.66449 | 0.76257 | 15353 | |
| Prdm16 | 0.6646 | 0.12159 | 15354 | |
| Mrpl13 | 0.66469 | 0.505 | 15355 | |
| Gpa33 | 0.66471 | -0.25691 | 15356 | |
| Slfn4 | 0.66491 | 1.0392 | 15357 | |
| Gm9268 | 0.66493 | 0.44023 | 15358 | |
| Slc52a2 | 0.66498 | 0.32364 | 15359 | |
| Otc | 0.665 | 0.3475 | 15360 | |
| Susd1 | 0.66509 | 0.14577 | 15361 | |
| Ptgr2 | 0.66515 | 0.26349 | 15362 | |
| Tmem241 | 0.66518 | 1.7572 | 15363 | |
| Mbd3l1 | 0.66533 | 0.35326 | 15364 | |
| Micu3 | 0.66535 | 1.2852 | 15365 | |
| Olfr1205 | 0.66539 | 1.1641 | 15366 | |
| Apip | 0.66553 | 0.11154 | 15367 | |
| Rhox4a | 0.66557 | 0.55542 | 15368 | |
| Olfr1084 | 0.66564 | 0.57285 | 15369 | |
| Rnf41 | 0.66566 | -0.3057 | 15370 | |
| Guca1b | 0.66572 | 1.1528 | 15371 | |
| Ptrh2 | 0.66579 | 0.1804 | 15372 | |
| Leprel1 | 0.66581 | 0.71043 | 15373 | |
| Adamts4 | 0.66586 | -0.15316 | 15374 | |
| Cd68 | 0.66592 | 0.020331 | 15375 | |
| Aim1 | 0.66598 | 3.1163 | 15376 | |
| Sqrdl | 0.66605 | -0.25798 | 15377 | |
| Gml | 0.66613 | -0.2358 | 15378 | |
| Creld2 | 0.66616 | -0.33229 | 15379 | |
| Ndrg4 | 0.66624 | 0.26503 | 15380 | |
| mmu-mir-676 | 0.66624 | 0.25184 | 15381 | |
| mmu-mir-8105 | 0.66625 | 0.66491 | 15382 | |
| 2200002J24Rik | 0.66636 | -0.41029 | 15383 | |
| Coq5 | 0.6664 | -0.14582 | 15384 | |
| Ap2s1 | 0.66649 | -0.023317 | 15385 | |
| Khdc1b | 0.66651 | 0.15243 | 15386 | |
| Spink11 | 0.66671 | 0.94032 | 15387 | |
| Gm13128 | 0.66673 | 0.63315 | 15388 | |
| Olfr1208 | 0.66675 | 1.3188 | 15389 | |
| Enpp4 | 0.66686 | -0.26926 | 15390 | |
| Cd300lg | 0.66688 | 0.26545 | 15391 | |
| Pias3 | 0.66696 | -0.22002 | 15392 | |
| Vgll1 | 0.66712 | 0.23231 | 15393 | |
| Ppp2r3a | 0.66723 | -0.14668 | 15394 | |
| mmu-mir-3620 | 0.66727 | -0.51844 | 15395 | |
| Ccm2l | 0.66738 | 1.122 | 15396 | |
| Tigd3 | 0.66743 | -0.10766 | 15397 | |
| Adam1a | 0.66761 | 0.18927 | 15398 | |
| Gpr111 | 0.66767 | 1.3286 | 15399 | |
| Dll3 | 0.66769 | 0.98777 | 15400 | |
| Rai1 | 0.66774 | -0.11973 | 15401 | |
| Olfr908 | 0.66785 | 0.75707 | 15402 | |
| Xlr3c | 0.66791 | -0.09394 | 15403 | |
| Otop3 | 0.66795 | -0.06773 | 15404 | |
| Mrps17 | 0.66798 | -0.067665 | 15405 | |
| Qk | 0.66811 | 0.0040536 | 15406 | |
| Pdik1l | 0.66821 | 0.49365 | 15407 | |
| Cfd | 0.66826 | 0.56191 | 15408 | |
| Loxl4 | 0.66828 | -0.28057 | 15409 | |
| Chd2 | 0.6683 | 1.3002 | 15410 | |
| Tmem218 | 0.66843 | -0.17077 | 15411 | |
| Wfdc15a | 0.66862 | -0.32877 | 15412 | |
| Hoxc12 | 0.6687 | 1.4992 | 15413 | |
| Ovch2 | 0.66876 | 0.99787 | 15414 | |
| C1qtnf2 | 0.66881 | 0.86326 | 15415 | |
| Pkia | 0.66885 | 3.2502 | 15416 | |
| Laptm4a | 0.66887 | 0.60615 | 15417 | |
| Gm11236 | 0.6689 | 0.17265 | 15418 | |
| Heca | 0.66894 | 0.1999 | 15419 | |
| Me2 | 0.66902 | 0.33155 | 15420 | |
| Klrg1 | 0.66913 | -0.2125 | 15421 | |
| Olfr987 | 0.66915 | -0.05392 | 15422 | |
| Ntm | 0.66926 | 0.57449 | 15423 | |
| Slc25a16 | 0.66939 | -0.22204 | 15424 | |
| mmu-mir-7237 | 0.6694 | 0.46622 | 15425 | |
| Olfr691 | 0.66941 | -0.0032695 | 15426 | |
| Adm | 0.6695 | 0.0062252 | 15427 | |
| Ptch1 | 0.66963 | -0.067009 | 15428 | |
| Dcakd | 0.66972 | 0.51624 | 15429 | |
| mmu-mir-7014 | 0.66988 | 0.51967 | 15430 | |
| Oxnad1 | 0.66999 | 0.65505 | 15431 | |
| Fam134b | 0.67015 | -0.019019 | 15432 | |
| Rims1 | 0.67024 | 1.6311 | 15433 | |
| Rbpjl | 0.67037 | 0.23486 | 15434 | |
| Trpv4 | 0.6707 | -0.19235 | 15435 | |
| mmu-mir-6919 | 0.67072 | -0.34581 | 15436 | |
| Lyplal1 | 0.67085 | 0.24024 | 15437 | |
| Poldip3 | 0.67087 | 0.01454 | 15438 | |
| mmu-mir-7689 | 0.67097 | 0.3149 | 15439 | |
| Ttc19 | 0.67098 | 1.4573 | 15440 | |
| Pax7 | 0.67102 | 0.64664 | 15441 | |
| Slc51a | 0.67104 | -0.26496 | 15442 | |
| Serpina5 | 0.67109 | -0.044538 | 15443 | |
| Ccpg1os | 0.67113 | 0.26763 | 15444 | |
| Dcps | 0.67123 | -0.42842 | 15445 | |
| Lamb1 | 0.67126 | -0.37716 | 15446 | |
| Ppapdc2 | 0.67137 | 0.43694 | 15447 | |
| Il1f10 | 0.67143 | -0.089237 | 15448 | |
| Spsb4 | 0.67159 | -0.049924 | 15449 | |
| 1700092M07Rik | 0.6718 | -0.21849 | 15450 | |
| Upk3b | 0.67182 | -0.078353 | 15451 | |
| Acpt | 0.67185 | -0.34864 | 15452 | |
| Ddx31 | 0.67202 | -0.40826 | 15453 | |
| Slitrk3 | 0.67213 | 0.31216 | 15454 | |
| 4930564D02Rik | 0.67219 | 0.13438 | 15455 | |
| Cnst | 0.67221 | -0.2557 | 15456 | |
| Svs3a | 0.67224 | -0.0088406 | 15457 | |
| Rep15 | 0.67226 | -0.014461 | 15458 | |
| H2afv | 0.67232 | -0.27451 | 15459 | |
| Htr2c | 0.67243 | 0.78998 | 15460 | |
| Eppk1 | 0.67261 | -0.27996 | 15461 | |
| Dab2 | 0.67267 | -0.18716 | 15462 | |
| Pi16 | 0.67271 | 0.032786 | 15463 | |
| Paip2b | 0.67273 | 0.13717 | 15464 | |
| Zfp748 | 0.67278 | 1.2144 | 15465 | |
| Nif3l1 | 0.67297 | 0.56457 | 15466 | |
| Pcdhb1 | 0.67299 | 1.0026 | 15467 | |
| Dusp13 | 0.6731 | 0.53672 | 15468 | |
| Mrpl48 | 0.67319 | 0.76604 | 15469 | |
| Matn1 | 0.67321 | 1.0341 | 15470 | |
| Adora2a | 0.67345 | 0.025687 | 15471 | |
| Kctd12b | 0.67353 | 0.29072 | 15472 | |
| Mob3a | 0.67356 | 0.13064 | 15473 | |
| Osbp | 0.67362 | 0.037727 | 15474 | |
| mmu-mir-6335 | 0.67383 | 0.95332 | 15475 | |
| Hpx | 0.67386 | -0.17591 | 15476 | |
| Fzr1 | 0.67395 | -0.28348 | 15477 | |
| Ndufaf6 | 0.67431 | 1.2493 | 15478 | |
| Ssc5d | 0.67435 | 0.56831 | 15479 | |
| Pdzd2 | 0.67437 | -0.20906 | 15480 | |
| Gm8787 | 0.67439 | 0.22243 | 15481 | |
| mmu-mir-3110 | 0.67443 | -0.4405 | 15482 | |
| Zc3h7b | 0.67455 | 0.52392 | 15483 | |
| Lrtm1 | 0.67457 | 0.18424 | 15484 | |
| Olfr1247 | 0.6747 | 0.95928 | 15485 | |
| Olfr1031 | 0.67477 | 0.080373 | 15486 | |
| mmu-mir-744 | 0.67478 | -0.092483 | 15487 | |
| Olfr724 | 0.6748 | 0.55185 | 15488 | |
| Gfod1 | 0.67483 | 1.1248 | 15489 | |
| Prkcdbp | 0.67485 | 1.6948 | 15490 | |
| Ear5 | 0.67493 | 0.083254 | 15491 | |
| Fam45a | 0.67507 | -0.41056 | 15492 | |
| Zfp28 | 0.67507 | 1.759 | 15493 | |
| Zcchc14 | 0.67513 | 0.42946 | 15494 | |
| Olfr1132 | 0.67536 | 0.14593 | 15495 | |
| P2rx1 | 0.67543 | 0.1176 | 15496 | |
| Bahd1 | 0.67549 | 1.1822 | 15497 | |
| mmu-mir-101a | 0.6755 | 0.12356 | 15498 | |
| Pcp4l1 | 0.67559 | 0.22167 | 15499 | |
| Gimap1 | 0.67562 | -0.1231 | 15500 | |
| Prlr | 0.67584 | 0.010495 | 15501 | |
| Klhl9 | 0.67588 | 0.33391 | 15502 | |
| Ppdpf | 0.67596 | -0.4323 | 15503 | |
| Rdh19 | 0.67606 | -0.21082 | 15504 | |
| Olfr1026 | 0.67629 | 0.99528 | 15505 | |
| Adcy10 | 0.67633 | 0.23658 | 15506 | |
| P2ry2 | 0.67637 | -0.26217 | 15507 | |
| Xpnpep1 | 0.67644 | -0.14305 | 15508 | |
| Ccdc3 | 0.67647 | 0.34145 | 15509 | |
| Dnaic2 | 0.67672 | 0.29084 | 15510 | |
| Zscan2 | 0.67675 | -0.33341 | 15511 | |
| Grip2 | 0.67684 | 0.11541 | 15512 | |
| Tle6 | 0.67689 | 0.15151 | 15513 | |
| Il18r1 | 0.67696 | -0.39821 | 15514 | |
| 4931429L15Rik | 0.67699 | 0.21324 | 15515 | |
| mmu-mir-2136 | 0.67703 | 0.05026 | 15516 | |
| Allc | 0.67706 | -0.24421 | 15517 | |
| Akr1b7 | 0.6771 | 1.1388 | 15518 | |
| Rassf5 | 0.67723 | 0.41271 | 15519 | |
| Fam129c | 0.67728 | -0.27204 | 15520 | |
| Spats1 | 0.67732 | 0.013304 | 15521 | |
| mmu-mir-6939 | 0.67735 | -0.051054 | 15522 | |
| Olfr370 | 0.6774 | 0.093318 | 15523 | |
| Ninj1 | 0.67747 | 0.89508 | 15524 | |
| Dhcr24 | 0.67755 | -0.3146 | 15525 | |
| Zcchc4 | 0.67768 | 0.051243 | 15526 | |
| Kcnab1 | 0.67775 | -0.40058 | 15527 | |
| mmu-mir-677 | 0.67779 | -0.060285 | 15528 | |
| Gm13280 | 0.67783 | 0.52325 | 15529 | |
| Eif5a2 | 0.67785 | -0.18911 | 15530 | |
| Ap1g2 | 0.67787 | 1.0514 | 15531 | |
| 1700001C19Rik | 0.67813 | 0.30288 | 15532 | |
| mmu-mir-344g | 0.67827 | -0.3319 | 15533 | |
| 4921511H03Rik | 0.67843 | 0.49313 | 15534 | |
| Tvp23a | 0.67847 | 0.15113 | 15535 | |
| Zbtb22 | 0.67851 | -0.24094 | 15536 | |
| Krtap4-6 | 0.67873 | 0.29109 | 15537 | |
| Gnpda2 | 0.67877 | 0.24328 | 15538 | |
| 2410124H12Rik | 0.67881 | -0.010381 | 15539 | |
| Klhdc2 | 0.67883 | 0.71024 | 15540 | |
| 1700080O16Rik | 0.67886 | 0.13572 | 15541 | |
| Gja6 | 0.67896 | 1.1076 | 15542 | |
| Gm3409 | 0.67939 | -0.34795 | 15543 | |
| Plcg2 | 0.6795 | 0.28638 | 15544 | |
| Fam214b | 0.67952 | -0.317 | 15545 | |
| Taar9 | 0.67957 | 0.65088 | 15546 | |
| Cdc42bpb | 0.6796 | 2.662 | 15547 | |
| 5830418K08Rik | 0.67963 | 0.1156 | 15548 | |
| Adprhl1 | 0.67971 | -0.36248 | 15549 | |
| Ndufv2 | 0.68008 | 0.047767 | 15550 | |
| Olfr1145 | 0.6802 | -0.090601 | 15551 | |
| Gsn | 0.68028 | -0.24574 | 15552 | |
| Zfp84 | 0.68039 | 0.28098 | 15553 | |
| Ikbip | 0.68041 | 1.1324 | 15554 | |
| Olfr1393 | 0.68047 | -0.38046 | 15555 | |
| mmu-mir-144 | 0.68056 | -0.42156 | 15556 | |
| Mafb | 0.68062 | -0.33355 | 15557 | |
| Rassf7 | 0.68071 | 0.21697 | 15558 | |
| Pxk | 0.68073 | 0.47829 | 15559 | |
| BC061237 | 0.68078 | 0.76728 | 15560 | |
| Lrrc59 | 0.68086 | 1.4362 | 15561 | |
| Atmin | 0.68099 | -0.11026 | 15562 | |
| Tmem175 | 0.68101 | 0.16617 | 15563 | |
| mmu-mir-7002 | 0.68101 | -0.29662 | 15564 | |
| mmu-mir-3098 | 0.68107 | -0.20352 | 15565 | |
| Psme2 | 0.68113 | 2.4079 | 15566 | |
| Kcnc4 | 0.68124 | 0.46443 | 15567 | |
| BC021891 | 0.6813 | 0.66545 | 15568 | |
| Gpld1 | 0.68139 | 0.050279 | 15569 | |
| Rmnd5b | 0.68142 | 0.021015 | 15570 | |
| Hsdl2 | 0.68145 | -0.062321 | 15571 | |
| Gm15319 | 0.68147 | 0.19491 | 15572 | |
| Anks6 | 0.68166 | 0.18009 | 15573 | |
| Fam107a | 0.68172 | -0.38255 | 15574 | |
| Aldh16a1 | 0.68183 | -0.25559 | 15575 | |
| Bmpr2 | 0.68189 | 0.31839 | 15576 | |
| Rhbdd2 | 0.68189 | -0.18582 | 15577 | |
| Cnot4 | 0.68192 | 0.041955 | 15578 | |
| mmu-mir-6405 | 0.68198 | 0.58916 | 15579 | |
| Alpk2 | 0.68198 | 0.34052 | 15580 | |
| Olfr1395 | 0.68205 | 0.087056 | 15581 | |
| Trap1a | 0.68207 | 1.3859 | 15582 | |
| Taf9b | 0.68226 | 1.2131 | 15583 | |
| Ppp1r3b | 0.6823 | 1.5714 | 15584 | |
| Sac3d1 | 0.68234 | -0.35775 | 15585 | |
| Olfr213 | 0.68286 | 0.065649 | 15586 | |
| Larp4 | 0.68289 | 0.070387 | 15587 | |
| Olfr1197 | 0.68294 | 0.30047 | 15588 | |
| Dqx1 | 0.68296 | -0.41617 | 15589 | |
| Olfr600 | 0.68299 | 0.62537 | 15590 | |
| Il11ra1 | 0.68305 | -0.43186 | 15591 | |
| Gm815 | 0.68306 | -0.42578 | 15592 | |
| Ccl22 | 0.68315 | 0.55148 | 15593 | |
| Endog | 0.68318 | 0.76907 | 15594 | |
| Cd40lg | 0.68323 | -0.28942 | 15595 | |
| Pigz | 0.68334 | 0.49831 | 15596 | |
| Ddx26b | 0.68349 | 0.34102 | 15597 | |
| Bpifa2 | 0.68378 | 0.76538 | 15598 | |
| Epgn | 0.68381 | -0.11847 | 15599 | |
| Olfr1242 | 0.68385 | 0.52274 | 15600 | |
| Arhgef38 | 0.68389 | 0.42189 | 15601 | |
| Zc2hc1b | 0.68401 | 1.2314 | 15602 | |
| Pou2f2 | 0.6841 | 1.3658 | 15603 | |
| Rnf5 | 0.68412 | 0.85888 | 15604 | |
| Gstm6 | 0.68414 | 0.36317 | 15605 | |
| Ankmy2 | 0.68427 | 1.6667 | 15606 | |
| Rhobtb1 | 0.68446 | 0.4904 | 15607 | |
| St3gal5 | 0.68448 | 0.78901 | 15608 | |
| Lcorl | 0.68456 | 1.0206 | 15609 | |
| mmu-mir-874 | 0.68478 | -0.13569 | 15610 | |
| Anapc16 | 0.68488 | 0.88182 | 15611 | |
| Ms4a4c | 0.68494 | 0.41633 | 15612 | |
| Wdr62 | 0.68509 | -0.14465 | 15613 | |
| Pth1r | 0.68517 | -0.06223 | 15614 | |
| Arl6ip6 | 0.68528 | -0.26705 | 15615 | |
| Spatc1l | 0.6853 | 0.0075081 | 15616 | |
| E330009J07Rik | 0.68564 | 0.72737 | 15617 | |
| Bcan | 0.6857 | 0.83213 | 15618 | |
| Crhbp | 0.68578 | 0.73691 | 15619 | |
| Gab1 | 0.68581 | 0.33661 | 15620 | |
| Sstr5 | 0.68587 | 0.45618 | 15621 | |
| Hsd11b1 | 0.68589 | 0.27423 | 15622 | |
| Xndr | 0.68597 | 0.11696 | 15623 | |
| Grm4 | 0.686 | -0.0067781 | 15624 | |
| Tlr4 | 0.68604 | 0.16146 | 15625 | |
| F8 | 0.68606 | 0.23019 | 15626 | |
| Mecr | 0.68614 | 1.2662 | 15627 | |
| mmu-mir-302d | 0.68614 | -0.39461 | 15628 | |
| mmu-mir-18b | 0.68619 | -0.35126 | 15629 | |
| Zfyve27 | 0.68623 | 0.55449 | 15630 | |
| Vps36 | 0.68629 | 0.33811 | 15631 | |
| Atp1a3 | 0.68639 | 0.29285 | 15632 | |
| Sgsm3 | 0.68646 | -0.26227 | 15633 | |
| Lhx8 | 0.6865 | -0.18243 | 15634 | |
| Crtc2 | 0.6866 | -0.051753 | 15635 | |
| Hao2 | 0.68667 | 0.11666 | 15636 | |
| Zp2 | 0.68671 | -0.21743 | 15637 | |
| Gm3701 | 0.68675 | 0.24653 | 15638 | |
| Gzmn | 0.68675 | 0.2552 | 15639 | |
| Armc4 | 0.68677 | -0.14301 | 15640 | |
| Olfr890 | 0.68686 | 0.44023 | 15641 | |
| Pcdhga10 | 0.68691 | -0.32199 | 15642 | |
| Prss37 | 0.68696 | 1.1501 | 15643 | |
| Gm5142 | 0.68715 | -0.033825 | 15644 | |
| Gbp9 | 0.68726 | 1.468 | 15645 | |
| Hhat | 0.68728 | -0.095043 | 15646 | |
| Nt5c1a | 0.6874 | 0.24914 | 15647 | |
| Tmem57 | 0.68753 | 0.76446 | 15648 | |
| Mark2 | 0.6876 | 0.20218 | 15649 | |
| Smim3 | 0.68771 | 0.52452 | 15650 | |
| Olfr16 | 0.68787 | 0.92373 | 15651 | |
| Zfp367 | 0.6879 | 0.69062 | 15652 | |
| Gm13272 | 0.68799 | 0.88608 | 15653 | |
| 4930590J08Rik | 0.68802 | -0.0070458 | 15654 | |
| Pmel | 0.68802 | -0.31558 | 15655 | |
| Acat1 | 0.68828 | 0.093114 | 15656 | |
| Lat2 | 0.68837 | 0.10054 | 15657 | |
| Spaca4 | 0.6884 | 0.40612 | 15658 | |
| Alk | 0.6884 | -0.12932 | 15659 | |
| Csnk1e | 0.68845 | 0.011308 | 15660 | |
| Il20ra | 0.6885 | -0.01794 | 15661 | |
| Apoa4 | 0.68866 | 0.20247 | 15662 | |
| Tspo2 | 0.68872 | 0.36771 | 15663 | |
| Arpc5 | 0.68876 | 1.9294 | 15664 | |
| Klk6 | 0.68884 | 0.78297 | 15665 | |
| Zfp874a | 0.68893 | 0.054839 | 15666 | |
| Dpys | 0.68896 | 0.7776 | 15667 | |
| Fis1 | 0.68896 | 0.36803 | 15668 | |
| Pax5 | 0.6891 | 0.28981 | 15669 | |
| Crocc | 0.68918 | 0.55221 | 15670 | |
| Arhgap36 | 0.68926 | 1.9619 | 15671 | |
| mmu-mir-7646 | 0.68928 | -0.44906 | 15672 | |
| Ptprz1 | 0.68928 | 0.11799 | 15673 | |
| S1pr1 | 0.68939 | 1.3648 | 15674 | |
| Zfp647 | 0.68941 | 0.78639 | 15675 | |
| Parn | 0.68954 | -0.13895 | 15676 | |
| Arhgef12 | 0.68989 | 0.91683 | 15677 | |
| 1110012L19Rik | 0.68991 | 1.0826 | 15678 | |
| Fam73a | 0.68999 | 0.38051 | 15679 | |
| Skida1 | 0.69006 | -0.1179 | 15680 | |
| Gzmd | 0.6902 | 1.3937 | 15681 | |
| Fbxo34 | 0.69033 | 0.47459 | 15682 | |
| Gm15070 | 0.69045 | -0.2255 | 15683 | |
| mmu-mir-695 | 0.6905 | 0.95895 | 15684 | |
| Zfp236 | 0.6906 | 0.35868 | 15685 | |
| Gtf2h4 | 0.69062 | 0.061341 | 15686 | |
| mmu-mir-6954 | 0.69065 | 0.36197 | 15687 | |
| 4933416C03Rik | 0.69066 | -0.37982 | 15688 | |
| Vcan | 0.6907 | 0.25056 | 15689 | |
| Gm16532 | 0.69074 | 0.27877 | 15690 | |
| Fam187b | 0.69079 | -0.018194 | 15691 | |
| Mogs | 0.6911 | 0.49898 | 15692 | |
| Lss | 0.69126 | 1.4199 | 15693 | |
| Prss44 | 0.69133 | 0.20596 | 15694 | |
| Plekhh1 | 0.69137 | 1.3084 | 15695 | |
| Slc39a5 | 0.69144 | -0.060727 | 15696 | |
| Tas2r113 | 0.69144 | -0.30263 | 15697 | |
| mmu-mir-5709 | 0.69147 | -0.032109 | 15698 | |
| C130060K24Rik | 0.69151 | -0.19838 | 15699 | |
| Spata31 | 0.69153 | 0.97735 | 15700 | |
| Timp3 | 0.69158 | -0.28609 | 15701 | |
| Pik3ip1 | 0.6916 | 0.97655 | 15702 | |
| Cuta | 0.69164 | 0.39623 | 15703 | |
| Cntnap4 | 0.69168 | 0.18507 | 15704 | |
| Coro1c | 0.69172 | 0.41196 | 15705 | |
| Crb3 | 0.69176 | 0.0051496 | 15706 | |
| S1pr4 | 0.69185 | 1.5807 | 15707 | |
| Wdr34 | 0.69193 | 0.38642 | 15708 | |
| Dnah9 | 0.692 | 0.0041091 | 15709 | |
| Slc35d3 | 0.69245 | 1.1632 | 15710 | |
| Neb | 0.69259 | 0.15706 | 15711 | |
| Hyi | 0.6927 | 0.98903 | 15712 | |
| Gaa | 0.69276 | -0.094374 | 15713 | |
| Isoc2a | 0.69305 | -0.33038 | 15714 | |
| Pld1 | 0.69311 | 0.043196 | 15715 | |
| Mad2l1bp | 0.69313 | -0.14891 | 15716 | |
| C5ar1 | 0.69324 | 0.61073 | 15717 | |
| Swap70 | 0.69336 | 0.27002 | 15718 | |
| Pnpla8 | 0.69339 | -0.10331 | 15719 | |
| Oog2 | 0.69355 | 0.74809 | 15720 | |
| Olfr808 | 0.69357 | 1.3459 | 15721 | |
| Neurog2 | 0.69365 | -0.12623 | 15722 | |
| Pts | 0.69371 | 1.6899 | 15723 | |
| Fam162a | 0.69386 | 0.72709 | 15724 | |
| Zfp518b | 0.69408 | -0.35369 | 15725 | |
| Pnmt | 0.69412 | 0.28991 | 15726 | |
| Ascl1 | 0.69417 | 1.0609 | 15727 | |
| Acadvl | 0.69421 | -0.41791 | 15728 | |
| Lctl | 0.69423 | 0.090929 | 15729 | |
| Fam188b | 0.69425 | -0.17385 | 15730 | |
| Plekhf1 | 0.69433 | 1.2485 | 15731 | |
| Pagr1a | 0.69443 | -0.20305 | 15732 | |
| Wdr65 | 0.6945 | -0.29791 | 15733 | |
| Rprml | 0.69456 | 1.1412 | 15734 | |
| Madd | 0.6946 | 0.91371 | 15735 | |
| Abtb1 | 0.69468 | -0.34905 | 15736 | |
| Gpha2 | 0.69479 | 0.30595 | 15737 | |
| Trex1 | 0.69494 | -0.061637 | 15738 | |
| Slc9c1 | 0.69497 | 2.2655 | 15739 | |
| Sh2d3c | 0.69518 | 0.66595 | 15740 | |
| Mageb16 | 0.69532 | -0.13752 | 15741 | |
| Cdh13 | 0.69542 | 0.70636 | 15742 | |
| Gm15140 | 0.69552 | 0.24259 | 15743 | |
| Tmem167b | 0.69569 | 0.0523 | 15744 | |
| Wnk4 | 0.69577 | -0.41795 | 15745 | |
| Dpf3 | 0.69601 | -0.02324 | 15746 | |
| Krt27 | 0.69604 | 0.5437 | 15747 | |
| Snx11 | 0.69622 | -0.41572 | 15748 | |
| Nxf2 | 0.69626 | 1.6334 | 15749 | |
| Khdrbs1 | 0.69631 | 0.20444 | 15750 | |
| Atp1b2 | 0.69633 | 0.097931 | 15751 | |
| Serpina3n | 0.69635 | 0.97712 | 15752 | |
| Ecel1 | 0.69637 | 1.8379 | 15753 | |
| Igf2bp3 | 0.69645 | -0.2133 | 15754 | |
| Meis2 | 0.6966 | 0.89124 | 15755 | |
| Ffar4 | 0.69662 | -0.18854 | 15756 | |
| Themis2 | 0.69668 | -0.41494 | 15757 | |
| Htr5a | 0.6968 | -0.039066 | 15758 | |
| Col6a1 | 0.69689 | 0.54377 | 15759 | |
| Depdc5 | 0.69697 | 0.85844 | 15760 | |
| Shisa7 | 0.69703 | 0.88596 | 15761 | |
| Fam13b | 0.69711 | -0.040133 | 15762 | |
| Pnpla7 | 0.69713 | 0.0813 | 15763 | |
| Aida | 0.69715 | -0.076017 | 15764 | |
| Olfr1198 | 0.69719 | 1.5581 | 15765 | |
| Stmnd1 | 0.69721 | 1.063 | 15766 | |
| Tmem235 | 0.69725 | 0.35271 | 15767 | |
| Rsad2 | 0.69736 | 0.12231 | 15768 | |
| Kcnmb4 | 0.69752 | 1.1603 | 15769 | |
| Magt1 | 0.69773 | -0.37137 | 15770 | |
| Rnf6 | 0.69785 | 0.20411 | 15771 | |
| Rsl1 | 0.69801 | 0.42139 | 15772 | |
| Celf3 | 0.69803 | -0.38351 | 15773 | |
| Mfsd9 | 0.69805 | 0.90139 | 15774 | |
| mmu-mir-8113 | 0.6981 | -0.085902 | 15775 | |
| Lgr6 | 0.69818 | 0.20814 | 15776 | |
| Tnn | 0.6982 | -0.10027 | 15777 | |
| Itih1 | 0.69838 | 0.64672 | 15778 | |
| Csnk1a1 | 0.69855 | 2.1767 | 15779 | |
| Hsd17b1 | 0.69859 | -0.41676 | 15780 | |
| Aqp2 | 0.69861 | 0.41188 | 15781 | |
| Akr1c21 | 0.69895 | 0.062677 | 15782 | |
| Tas2r129 | 0.699 | -0.38758 | 15783 | |
| Acan | 0.69904 | 0.48375 | 15784 | |
| Itgb1bp2 | 0.69908 | 0.18307 | 15785 | |
| Gm6772 | 0.6991 | 1.569 | 15786 | |
| Trip13 | 0.69914 | 0.078174 | 15787 | |
| Pclo | 0.69928 | 0.033996 | 15788 | |
| Rreb1 | 0.69932 | -0.089871 | 15789 | |
| Gltscr1l | 0.69949 | -0.28317 | 15790 | |
| Gpr133 | 0.69955 | -0.17296 | 15791 | |
| Mcc | 0.69969 | -0.26875 | 15792 | |
| Olfr114 | 0.69971 | 1.3326 | 15793 | |
| Gm6121 | 0.69975 | 0.54611 | 15794 | |
| Ugt2a1 | 0.69981 | 0.34376 | 15795 | |
| Tgif2lx1 | 0.69983 | 0.2512 | 15796 | |
| Olfr74 | 0.69985 | 0.63508 | 15797 | |
| Zfp334 | 0.70002 | 0.55211 | 15798 | |
| Gm6164 | 0.70007 | 0.69069 | 15799 | |
| Svs5 | 0.70009 | 0.35443 | 15800 | |
| Ccdc53 | 0.70012 | -0.355 | 15801 | |
| Rasal2 | 0.70016 | -0.2236 | 15802 | |
| Kcnh7 | 0.70018 | -0.047344 | 15803 | |
| Slc6a14 | 0.70034 | 0.82466 | 15804 | |
| Uhrf1bp1l | 0.70036 | -0.31977 | 15805 | |
| Rilpl2 | 0.70039 | -0.13047 | 15806 | |
| Wdr41 | 0.70047 | 0.32928 | 15807 | |
| Tdgf1 | 0.70051 | 0.16028 | 15808 | |
| Smim18 | 0.70055 | -0.1871 | 15809 | |
| Idh3b | 0.70059 | 1.6025 | 15810 | |
| Agps | 0.70061 | 0.16636 | 15811 | |
| Ccdc71 | 0.70069 | 0.01005 | 15812 | |
| Med30 | 0.70104 | 0.23383 | 15813 | |
| Rgs9bp | 0.70108 | 0.21627 | 15814 | |
| Vps53 | 0.70118 | -0.37724 | 15815 | |
| Slc25a30 | 0.70122 | 0.027736 | 15816 | |
| Galnt12 | 0.70126 | 0.41166 | 15817 | |
| Gpr158 | 0.70128 | 0.38659 | 15818 | |
| Selk | 0.7013 | 0.32971 | 15819 | |
| St6galnac4 | 0.70142 | -0.35065 | 15820 | |
| Gimap6 | 0.70148 | 0.743 | 15821 | |
| Lgals1 | 0.70162 | 0.40248 | 15822 | |
| Manf | 0.70171 | -0.34914 | 15823 | |
| Sim2 | 0.70178 | 0.47282 | 15824 | |
| Acot6 | 0.70191 | 0.56019 | 15825 | |
| Tinagl1 | 0.70199 | -0.024947 | 15826 | |
| Entpd6 | 0.70209 | 0.27405 | 15827 | |
| Igflr1 | 0.7024 | -0.25219 | 15828 | |
| Snn | 0.70242 | -0.1933 | 15829 | |
| Nfkbib | 0.70252 | -0.2272 | 15830 | |
| Chic2 | 0.70262 | -0.31018 | 15831 | |
| Katnal1 | 0.70264 | 1.0929 | 15832 | |
| Nlrp4f | 0.70267 | 0.26601 | 15833 | |
| Olfr373 | 0.70272 | 2.0317 | 15834 | |
| Coro6 | 0.7029 | -0.40155 | 15835 | |
| Gdf15 | 0.70303 | 0.51237 | 15836 | |
| mmu-mir-705 | 0.70307 | 0.48104 | 15837 | |
| 5430401F13Rik | 0.70325 | 0.91587 | 15838 | |
| Trim39 | 0.70329 | 0.68284 | 15839 | |
| Insl5 | 0.70333 | -0.29082 | 15840 | |
| Olfr1377 | 0.70335 | 0.37685 | 15841 | |
| Lzic | 0.70337 | 0.70146 | 15842 | |
| Tmem185b | 0.70353 | 1.3078 | 15843 | |
| Tas2r104 | 0.70371 | 0.45321 | 15844 | |
| Ube2f | 0.70381 | -0.33756 | 15845 | |
| Fam180a | 0.70388 | -0.034883 | 15846 | |
| Shprh | 0.70396 | 0.57662 | 15847 | |
| Gpsm2 | 0.70412 | 1.1848 | 15848 | |
| Agr3 | 0.70418 | 0.90916 | 15849 | |
| Slc4a4 | 0.70428 | -0.41287 | 15850 | |
| Paqr3 | 0.7043 | 0.0073748 | 15851 | |
| Mettl21a | 0.70432 | 0.86531 | 15852 | |
| Ube2s | 0.70434 | 1.2849 | 15853 | |
| Dscr3 | 0.7044 | -0.16598 | 15854 | |
| Tdrd9 | 0.70444 | 0.28008 | 15855 | |
| Nrarp | 0.70448 | 0.024473 | 15856 | |
| Ppp2cb | 0.70459 | 2.6552 | 15857 | |
| Ppp4r1 | 0.70459 | -0.40325 | 15858 | |
| Olfr414 | 0.70474 | 0.83104 | 15859 | |
| Elmod3 | 0.70478 | 0.50604 | 15860 | |
| 6330403K07Rik | 0.70493 | 1.0735 | 15861 | |
| Kazn | 0.70507 | -0.32909 | 15862 | |
| Olfr640 | 0.70517 | -0.11274 | 15863 | |
| Vmn1r171 | 0.70529 | -0.17628 | 15864 | |
| Olfr1009 | 0.70531 | 0.44922 | 15865 | |
| Stox1 | 0.70533 | 0.14683 | 15866 | |
| Olfr50 | 0.70567 | 0.76079 | 15867 | |
| Ndn | 0.70589 | 0.40337 | 15868 | |
| Coq4 | 0.70601 | 0.050855 | 15869 | |
| mmu-mir-143 | 0.70609 | 0.1861 | 15870 | |
| Stx1a | 0.70615 | -0.30926 | 15871 | |
| Pycr2 | 0.70622 | 1.1757 | 15872 | |
| Git1 | 0.70626 | -0.025614 | 15873 | |
| Capn11 | 0.7064 | -0.13463 | 15874 | |
| Prox2 | 0.70662 | 0.6736 | 15875 | |
| Gdap2 | 0.7067 | -0.24688 | 15876 | |
| Lfng | 0.70682 | -0.0050332 | 15877 | |
| Icam5 | 0.70688 | 0.094931 | 15878 | |
| Rab7l1 | 0.70699 | 0.71256 | 15879 | |
| Gna11 | 0.70704 | -0.13176 | 15880 | |
| Ercc6l | 0.70708 | -0.20906 | 15881 | |
| Rab44 | 0.7071 | 0.72533 | 15882 | |
| Dsc1 | 0.70736 | 0.89535 | 15883 | |
| Abca7 | 0.70742 | 0.83435 | 15884 | |
| Olfr976 | 0.70744 | 1.0797 | 15885 | |
| Serpinh1 | 0.70754 | 0.086104 | 15886 | |
| P4ha2 | 0.70764 | 0.21443 | 15887 | |
| Isoc2b | 0.70776 | 0.45424 | 15888 | |
| Ggct | 0.70778 | 0.045412 | 15889 | |
| Skint9 | 0.70782 | 1.8744 | 15890 | |
| Slc6a1 | 0.70784 | -0.25623 | 15891 | |
| Krtap4-13 | 0.70797 | 0.81177 | 15892 | |
| Atrx | 0.708 | 1.3951 | 15893 | |
| Lrrc8d | 0.70804 | 0.62388 | 15894 | |
| Dmrtc1c1 | 0.70808 | 0.56356 | 15895 | |
| Calm3 | 0.70814 | 0.51062 | 15896 | |
| Tgm5 | 0.7082 | 0.85227 | 15897 | |
| Nkpd1 | 0.70822 | -0.2481 | 15898 | |
| Sbk1 | 0.70828 | -0.39184 | 15899 | |
| Src | 0.7083 | 0.90484 | 15900 | |
| Scara3 | 0.70832 | -0.094196 | 15901 | |
| Aifm3 | 0.70834 | 0.72837 | 15902 | |
| Atg2a | 0.70846 | 0.37613 | 15903 | |
| Olfr530 | 0.70848 | 0.27701 | 15904 | |
| Sult1c2 | 0.7085 | 0.55602 | 15905 | |
| Clcn6 | 0.7086 | 0.82392 | 15906 | |
| Lamp1 | 0.7087 | -0.015528 | 15907 | |
| Nelfe | 0.70882 | -0.42991 | 15908 | |
| Ms4a4b | 0.70888 | 0.12674 | 15909 | |
| Slc35f3 | 0.709 | -0.22129 | 15910 | |
| Por | 0.70906 | 0.40823 | 15911 | |
| Dmxl1 | 0.7092 | -0.37727 | 15912 | |
| Gm11710 | 0.70958 | 0.096905 | 15913 | |
| Endod1 | 0.70958 | 0.497 | 15914 | |
| Sirt5 | 0.70964 | 0.1153 | 15915 | |
| 4930550L24Rik | 0.7097 | 0.71052 | 15916 | |
| Olfr806 | 0.7098 | -0.17432 | 15917 | |
| Khk | 0.70984 | -0.30965 | 15918 | |
| Gpc2 | 0.70994 | 2.8378 | 15919 | |
| Adrbk2 | 0.70998 | 0.66248 | 15920 | |
| Etv3 | 0.71005 | 0.90505 | 15921 | |
| Gstcd | 0.71005 | -0.35639 | 15922 | |
| Jmjd8 | 0.71014 | -0.1524 | 15923 | |
| 1700034F02Rik | 0.7102 | -0.013866 | 15924 | |
| Abhd2 | 0.71036 | 0.094913 | 15925 | |
| Odf3l1 | 0.71042 | 0.10349 | 15926 | |
| mmu-mir-466a | 0.71049 | 0.2783 | 15927 | |
| Fgd5 | 0.7107 | -0.089246 | 15928 | |
| Rad51ap2 | 0.71073 | 0.42795 | 15929 | |
| Spast | 0.71076 | -0.36925 | 15930 | |
| Fcrl5 | 0.7109 | 0.045879 | 15931 | |
| Hba-a2 | 0.7109 | 0.28803 | 15932 | |
| Rprm | 0.71098 | 0.12598 | 15933 | |
| Fscn3 | 0.71122 | -0.38685 | 15934 | |
| Mex3c | 0.71127 | 1.4506 | 15935 | |
| 4930449I24Rik | 0.71137 | 0.58252 | 15936 | |
| Rfwd2 | 0.71143 | 0.13579 | 15937 | |
| Acad8 | 0.71145 | 0.22156 | 15938 | |
| Asah2 | 0.71153 | 0.93227 | 15939 | |
| Fbxl7 | 0.71155 | -0.0051182 | 15940 | |
| Slc5a3 | 0.71163 | -0.21865 | 15941 | |
| Tada2a | 0.71175 | 0.81507 | 15942 | |
| Poc1a | 0.71185 | 0.78122 | 15943 | |
| Ceacam16 | 0.71189 | -0.3782 | 15944 | |
| Qdpr | 0.71191 | -0.027064 | 15945 | |
| Gjd2 | 0.71215 | -0.25398 | 15946 | |
| Pate4 | 0.71225 | -0.27089 | 15947 | |
| Gm16501 | 0.71227 | -0.082849 | 15948 | |
| Serpina7 | 0.71229 | 2.4863 | 15949 | |
| Rgs20 | 0.71239 | 0.8666 | 15950 | |
| Gm5726 | 0.7124 | 0.29574 | 15951 | |
| Tmed9 | 0.71245 | -0.020821 | 15952 | |
| Pank1 | 0.71257 | 0.5515 | 15953 | |
| Pcdhga7 | 0.71266 | 0.542 | 15954 | |
| Pramel6 | 0.71267 | 1.4401 | 15955 | |
| Zfp157 | 0.7127 | -0.066924 | 15956 | |
| Kcnmb3 | 0.71272 | 0.089932 | 15957 | |
| Limk1 | 0.71274 | 0.66488 | 15958 | |
| Gbp6 | 0.71286 | -0.034287 | 15959 | |
| Ngb | 0.7129 | 0.34841 | 15960 | |
| Mfi2 | 0.71292 | -0.013505 | 15961 | |
| Syt12 | 0.71307 | 1.1098 | 15962 | |
| Cyb561d1 | 0.71312 | -0.37772 | 15963 | |
| Peli1 | 0.71316 | 0.21419 | 15964 | |
| Tmem189 | 0.7132 | -0.34742 | 15965 | |
| Olfr43 | 0.7132 | 0.1117 | 15966 | |
| Upk1b | 0.71324 | -0.17704 | 15967 | |
| Astn2 | 0.71326 | 1.1012 | 15968 | |
| Btg3 | 0.7133 | 0.064775 | 15969 | |
| Ap5z1 | 0.71337 | -0.32794 | 15970 | |
| Atp6v0c | 0.71337 | -0.25336 | 15971 | |
| Pdlim4 | 0.7134 | 0.43442 | 15972 | |
| Col25a1 | 0.71352 | 0.43414 | 15973 | |
| Cmtm4 | 0.71356 | 0.97339 | 15974 | |
| Aadacl2 | 0.71363 | 0.68039 | 15975 | |
| mmu-mir-1971 | 0.71369 | 0.49799 | 15976 | |
| Vta1 | 0.71373 | 0.048334 | 15977 | |
| Pacs1 | 0.71377 | -0.023472 | 15978 | |
| 2310057J18Rik | 0.71389 | -0.25956 | 15979 | |
| Cidec | 0.71411 | 0.29428 | 15980 | |
| Shc3 | 0.71414 | 0.026388 | 15981 | |
| Slc4a1ap | 0.71427 | -0.41613 | 15982 | |
| Tyro3 | 0.71429 | 0.47822 | 15983 | |
| Onecut3 | 0.71439 | -0.031822 | 15984 | |
| Enkur | 0.71441 | 0.15182 | 15985 | |
| Rnf19b | 0.71454 | 0.5081 | 15986 | |
| Magi2 | 0.71462 | 1.9408 | 15987 | |
| Pcdhb3 | 0.71513 | 0.037267 | 15988 | |
| Zfp691 | 0.71521 | 0.8022 | 15989 | |
| Frmd6 | 0.7154 | 0.76894 | 15990 | |
| Fam49a | 0.71545 | 0.43406 | 15991 | |
| Olfr1243 | 0.71553 | 0.31984 | 15992 | |
| Cxx1c | 0.71557 | 0.50076 | 15993 | |
| Gbp8 | 0.71565 | -0.45129 | 15994 | |
| Add2 | 0.71567 | -0.11626 | 15995 | |
| Emc2 | 0.71575 | 2.2929 | 15996 | |
| mmu-mir-3103 | 0.7158 | -0.14428 | 15997 | |
| Fzd2 | 0.71586 | -0.19731 | 15998 | |
| Olfr1494 | 0.71588 | 0.41659 | 15999 | |
| Olfr8 | 0.71592 | 0.31787 | 16000 | |
| Tmem254a | 0.71598 | -0.24626 | 16001 | |
| Gm4141 | 0.71621 | -0.22181 | 16002 | |
| Marcks | 0.71626 | -0.11822 | 16003 | |
| Rrnad1 | 0.71635 | 0.32748 | 16004 | |
| Abhd12b | 0.71639 | -0.22184 | 16005 | |
| Zfp804a | 0.71645 | -0.41623 | 16006 | |
| Zfp395 | 0.71649 | -0.06315 | 16007 | |
| Umodl1 | 0.71675 | -0.057818 | 16008 | |
| Lbx1 | 0.71689 | -0.42022 | 16009 | |
| Entpd8 | 0.71694 | 0.85197 | 16010 | |
| Clec1b | 0.71708 | 0.81466 | 16011 | |
| Large | 0.71724 | 1.0205 | 16012 | |
| Hemk1 | 0.71726 | 1.5945 | 16013 | |
| Dpcr1 | 0.7173 | -0.20106 | 16014 | |
| Zfp784 | 0.71749 | -0.17868 | 16015 | |
| Hectd2 | 0.71754 | 0.95131 | 16016 | |
| Lcor | 0.71787 | -0.0073086 | 16017 | |
| Pcyox1l | 0.71792 | -0.0015269 | 16018 | |
| H2-M10.6 | 0.71796 | 0.4708 | 16019 | |
| Cebpd | 0.71806 | 0.19599 | 16020 | |
| Olfr134 | 0.71818 | 0.2738 | 16021 | |
| Rhox5 | 0.7182 | 0.97022 | 16022 | |
| mmu-mir-7a-2 | 0.71837 | 0.60116 | 16023 | |
| Otol1 | 0.71867 | -0.025538 | 16024 | |
| Optn | 0.71886 | -0.070902 | 16025 | |
| Pold4 | 0.7189 | 1.9073 | 16026 | |
| Krtap19-5 | 0.71912 | -0.35459 | 16027 | |
| Grik1 | 0.71936 | -0.25081 | 16028 | |
| Nfic | 0.71949 | 0.18396 | 16029 | |
| Il17ra | 0.71955 | -0.28084 | 16030 | |
| Jagn1 | 0.71957 | -0.13116 | 16031 | |
| Grip1 | 0.71958 | 0.90178 | 16032 | |
| Kcnq1 | 0.7196 | 1.963 | 16033 | |
| Exosc10 | 0.71966 | 1.027 | 16034 | |
| Zswim8 | 0.71972 | 0.60189 | 16035 | |
| Tead3 | 0.71982 | -0.099157 | 16036 | |
| Olfr1402 | 0.71986 | -0.3062 | 16037 | |
| Alkbh7 | 0.71988 | 0.76949 | 16038 | |
| mmu-mir-489 | 0.71994 | 0.13695 | 16039 | |
| Onecut1 | 0.71996 | 0.90443 | 16040 | |
| Nradd | 0.72 | -0.082381 | 16041 | |
| Sema3g | 0.72027 | -0.0047852 | 16042 | |
| Galnt14 | 0.72029 | 1.7359 | 16043 | |
| Rad54b | 0.72031 | 0.71338 | 16044 | |
| Arfgap3 | 0.72036 | -0.055305 | 16045 | |
| Insm2 | 0.72042 | 0.46446 | 16046 | |
| Gpr135 | 0.72046 | 1.6394 | 16047 | |
| mmu-mir-6900 | 0.72057 | 2.0359 | 16048 | |
| Plac9a | 0.72062 | -0.24046 | 16049 | |
| Fam46c | 0.72064 | 0.55 | 16050 | |
| Ptpn20 | 0.72068 | 0.75305 | 16051 | |
| Artn | 0.72075 | 0.52139 | 16052 | |
| Golt1b | 0.72078 | 0.36219 | 16053 | |
| Mdh1 | 0.72082 | 0.67213 | 16054 | |
| Nanos1 | 0.72089 | 0.032436 | 16055 | |
| Mcat | 0.72095 | 0.0602 | 16056 | |
| Socs5 | 0.72099 | 0.35853 | 16057 | |
| Dhrs7c | 0.72101 | 0.063303 | 16058 | |
| Dmrtc1a | 0.72112 | 0.011402 | 16059 | |
| Glp1r | 0.72118 | -0.12116 | 16060 | |
| Dio2 | 0.72126 | -0.39502 | 16061 | |
| Irgm1 | 0.7213 | 0.68215 | 16062 | |
| Spink3 | 0.72136 | 0.11564 | 16063 | |
| Acat2 | 0.72139 | 0.51758 | 16064 | |
| Asf1a | 0.72149 | 0.52636 | 16065 | |
| Pgr15l | 0.72152 | 0.011281 | 16066 | |
| Use1 | 0.72159 | -0.21947 | 16067 | |
| Zfp385b | 0.72169 | 0.16817 | 16068 | |
| Ppp1r27 | 0.72186 | 0.27699 | 16069 | |
| Olfr1154 | 0.7219 | 1.3793 | 16070 | |
| Tmem164 | 0.72195 | 0.6217 | 16071 | |
| Slc45a4 | 0.72202 | -0.093903 | 16072 | |
| Pspn | 0.72203 | -0.21672 | 16073 | |
| Nfe2l1 | 0.72215 | 0.31264 | 16074 | |
| Gm4301 | 0.72224 | 0.53417 | 16075 | |
| Olfr463 | 0.72225 | 0.49026 | 16076 | |
| Insm1 | 0.72227 | -0.21764 | 16077 | |
| Slc30a8 | 0.72233 | 0.25387 | 16078 | |
| Olfr706 | 0.72242 | 0.87127 | 16079 | |
| Zbtb7c | 0.72244 | 0.4851 | 16080 | |
| B3gnt8 | 0.72258 | 1.5959 | 16081 | |
| Clec5a | 0.72262 | 0.54855 | 16082 | |
| Olfr458 | 0.72281 | 1.3777 | 16083 | |
| Atp8b2 | 0.72291 | 0.12778 | 16084 | |
| Tspan32 | 0.72314 | 0.3433 | 16085 | |
| Vmn1r219 | 0.72318 | 0.031459 | 16086 | |
| Klrc2 | 0.72329 | -0.18509 | 16087 | |
| Prl2c2 | 0.72365 | -0.19719 | 16088 | |
| Dnajb5 | 0.72368 | 0.60359 | 16089 | |
| Dach2 | 0.72374 | 0.14702 | 16090 | |
| Tomm40l | 0.72397 | 1.7226 | 16091 | |
| Lingo1 | 0.7243 | 1.1279 | 16092 | |
| Taar3 | 0.72435 | 0.17742 | 16093 | |
| Zfp101 | 0.72439 | 0.87741 | 16094 | |
| Srp19 | 0.72462 | 1.3128 | 16095 | |
| Dnajc30 | 0.72474 | 0.84494 | 16096 | |
| Uaca | 0.72484 | 1.2818 | 16097 | |
| Cmpk2 | 0.72487 | -0.28926 | 16098 | |
| Klhl8 | 0.72493 | 2.5424 | 16099 | |
| Smap2 | 0.72499 | -0.42067 | 16100 | |
| Man2c1 | 0.72501 | -0.044179 | 16101 | |
| Angptl6 | 0.72505 | 0.32411 | 16102 | |
| Fam229a | 0.72519 | 0.03616 | 16103 | |
| Kcnj16 | 0.72519 | -0.23317 | 16104 | |
| Ccl26 | 0.72524 | -0.17177 | 16105 | |
| Mia3 | 0.72547 | -0.27968 | 16106 | |
| Tmem255a | 0.7258 | -0.23274 | 16107 | |
| Sbf1 | 0.72584 | 0.12903 | 16108 | |
| Skor1 | 0.72587 | 0.45339 | 16109 | |
| Slc25a18 | 0.72599 | -0.30657 | 16110 | |
| Shc2 | 0.72605 | 0.41198 | 16111 | |
| Cldn12 | 0.72607 | 1.0743 | 16112 | |
| Uxs1 | 0.72611 | 0.78546 | 16113 | |
| Dytn | 0.72616 | 0.95314 | 16114 | |
| Hhla1 | 0.72626 | 0.27394 | 16115 | |
| Atp13a2 | 0.72634 | -0.04603 | 16116 | |
| Gusb | 0.72643 | 1.7813 | 16117 | |
| Olfr1390 | 0.72658 | 0.00018829 | 16118 | |
| Olfr676 | 0.72662 | -0.04303 | 16119 | |
| Tfip11 | 0.72664 | -0.15746 | 16120 | |
| 1700012B07Rik | 0.72668 | 0.1271 | 16121 | |
| Nudt10 | 0.72674 | 0.59462 | 16122 | |
| Dpt | 0.72691 | 1.5936 | 16123 | |
| Wfdc17 | 0.72698 | -0.20093 | 16124 | |
| Traf6 | 0.72712 | 0.02652 | 16125 | |
| Extl3 | 0.72716 | -0.016662 | 16126 | |
| Mtrf1 | 0.72735 | -0.36507 | 16127 | |
| Dpp4 | 0.72737 | 0.033557 | 16128 | |
| Vmn1r116 | 0.72751 | 0.48755 | 16129 | |
| Ifngr1 | 0.72768 | 0.96325 | 16130 | |
| Vmn2r21 | 0.72784 | 0.47854 | 16131 | |
| Olfr6 | 0.72787 | -0.088155 | 16132 | |
| Sash3 | 0.72789 | 0.20763 | 16133 | |
| Hist1h1e | 0.72812 | 0.40982 | 16134 | |
| Ubxn6 | 0.72813 | -0.15412 | 16135 | |
| Taf1a | 0.72815 | -0.14541 | 16136 | |
| Tspyl5 | 0.72817 | 0.82405 | 16137 | |
| Mtrr | 0.72833 | 1.036 | 16138 | |
| Tex24 | 0.72834 | -0.032762 | 16139 | |
| Pglyrp2 | 0.72838 | -0.0069898 | 16140 | |
| Tmem8c | 0.72844 | 1.717 | 16141 | |
| Crct1 | 0.72848 | 0.0036814 | 16142 | |
| Cnot8 | 0.72858 | 0.99259 | 16143 | |
| Ecsit | 0.72861 | -0.17526 | 16144 | |
| Zfp36l1 | 0.72863 | 2.7139 | 16145 | |
| Vmn2r19 | 0.72865 | -0.19545 | 16146 | |
| Lrp1 | 0.72869 | -0.0014288 | 16147 | |
| Capn10 | 0.72869 | -0.37077 | 16148 | |
| Ankrd40 | 0.72884 | -0.39186 | 16149 | |
| Scgb2b27 | 0.72886 | -0.3717 | 16150 | |
| Zmynd12 | 0.72894 | 0.54452 | 16151 | |
| Gm1110 | 0.7292 | 0.60837 | 16152 | |
| Cplx2 | 0.72938 | -0.054847 | 16153 | |
| Fam193b | 0.72949 | 0.85846 | 16154 | |
| Slc26a5 | 0.72968 | 2.8727 | 16155 | |
| Ak4 | 0.72972 | 1.0406 | 16156 | |
| Tbc1d15 | 0.72979 | 0.60721 | 16157 | |
| Aqp11 | 0.72987 | 0.5437 | 16158 | |
| Siah3 | 0.72989 | -0.029992 | 16159 | |
| Tssc4 | 0.72998 | 0.9786 | 16160 | |
| mmu-let-7c-1 | 0.72998 | 0.82676 | 16161 | |
| Raet1a | 0.73 | 1.3428 | 16162 | |
| Ccdc88b | 0.73006 | 0.82681 | 16163 | |
| Ctnnbl1 | 0.73012 | -0.28071 | 16164 | |
| Negr1 | 0.73019 | -0.29677 | 16165 | |
| mmu-mir-3112 | 0.73031 | 1.3177 | 16166 | |
| Tnfrsf11b | 0.73034 | 0.10281 | 16167 | |
| Col16a1 | 0.73038 | 0.89809 | 16168 | |
| Acot12 | 0.73044 | 0.79424 | 16169 | |
| Cdkn2d | 0.73067 | -0.17161 | 16170 | |
| Psd3 | 0.73071 | 0.57381 | 16171 | |
| mmu-mir-299b | 0.73071 | -0.22561 | 16172 | |
| Ssxb10 | 0.73074 | 0.12975 | 16173 | |
| Qrfpr | 0.73086 | 1.82 | 16174 | |
| Olfr777 | 0.7309 | 0.19611 | 16175 | |
| Fam228b | 0.73094 | 0.90169 | 16176 | |
| Gata4 | 0.73097 | 0.38574 | 16177 | |
| Il17c | 0.73108 | 0.096075 | 16178 | |
| Tmem59 | 0.7311 | 0.397 | 16179 | |
| Lrrtm3 | 0.73114 | -0.18959 | 16180 | |
| Olfr975 | 0.7312 | 0.27047 | 16181 | |
| Zfp180 | 0.73122 | 0.78909 | 16182 | |
| Ctnnbip1 | 0.73139 | 0.72494 | 16183 | |
| Fth1 | 0.73141 | 0.0098895 | 16184 | |
| Mthfsd | 0.73158 | 0.73177 | 16185 | |
| Vmn1r9 | 0.73163 | -0.1086 | 16186 | |
| Olfr365 | 0.73171 | 0.03413 | 16187 | |
| Rnf126 | 0.73173 | -0.11773 | 16188 | |
| Pank3 | 0.73184 | 0.98403 | 16189 | |
| 1810009A15Rik | 0.73186 | 1.1647 | 16190 | |
| Slc25a34 | 0.73196 | -0.053162 | 16191 | |
| Scrib | 0.73198 | 0.43369 | 16192 | |
| Elmod2 | 0.73203 | -0.14728 | 16193 | |
| Mtss1 | 0.73205 | 0.25037 | 16194 | |
| Zfp42 | 0.73218 | -0.070522 | 16195 | |
| Nipal4 | 0.73241 | 0.17606 | 16196 | |
| Prdx6 | 0.73249 | -0.18237 | 16197 | |
| mmu-mir-106a | 0.73275 | 0.33836 | 16198 | |
| Vmn2r120 | 0.73281 | 0.12001 | 16199 | |
| Insr | 0.73292 | -0.35529 | 16200 | |
| Psmb8 | 0.73307 | -0.027303 | 16201 | |
| Thbs3 | 0.73311 | 0.19909 | 16202 | |
| Ranbp17 | 0.73324 | 1.4921 | 16203 | |
| Ccdc33 | 0.73338 | 0.24813 | 16204 | |
| Ssfa2 | 0.73338 | 0.41173 | 16205 | |
| Pbk | 0.7335 | 0.44324 | 16206 | |
| mmu-mir-3101 | 0.73358 | 0.60023 | 16207 | |
| LOC666331 | 0.73362 | -0.11692 | 16208 | |
| Sele | 0.73367 | 0.016334 | 16209 | |
| Plch2 | 0.73384 | 0.4954 | 16210 | |
| Cald1 | 0.734 | 0.17852 | 16211 | |
| Tube1 | 0.73405 | -0.085802 | 16212 | |
| Mt1 | 0.73415 | 0.01367 | 16213 | |
| Zdhhc5 | 0.73426 | 1.3136 | 16214 | |
| Itgax | 0.73433 | -0.10806 | 16215 | |
| 1700011A15Rik | 0.73447 | -0.082684 | 16216 | |
| Sfrp5 | 0.73454 | 0.2713 | 16217 | |
| Lpl | 0.73458 | -0.32949 | 16218 | |
| Dnase1 | 0.7346 | -0.11329 | 16219 | |
| Sh3bp4 | 0.73465 | 0.23719 | 16220 | |
| En2 | 0.73469 | -0.019777 | 16221 | |
| Clcn5 | 0.73477 | 0.99012 | 16222 | |
| Sapcd1 | 0.73484 | -0.030673 | 16223 | |
| N28178 | 0.73486 | 0.42082 | 16224 | |
| mmu-mir-126a | 0.73488 | 0.56364 | 16225 | |
| Nbl1 | 0.73492 | 0.41863 | 16226 | |
| mmu-mir-678 | 0.73495 | 0.29389 | 16227 | |
| Aph1c | 0.73501 | 0.46444 | 16228 | |
| Lsr | 0.73505 | 0.0087047 | 16229 | |
| E2f1 | 0.73509 | 0.60244 | 16230 | |
| Snx31 | 0.73512 | 0.29115 | 16231 | |
| Mfsd7a | 0.7352 | 0.70523 | 16232 | |
| Vmn1r51 | 0.73526 | -0.067209 | 16233 | |
| Gucy1a3 | 0.73526 | -0.21009 | 16234 | |
| Kbtbd7 | 0.73529 | 0.55013 | 16235 | |
| Sbsn | 0.73535 | 0.58486 | 16236 | |
| Eed | 0.7355 | -0.40541 | 16237 | |
| 4931414P19Rik | 0.73556 | 0.14393 | 16238 | |
| mmu-mir-499 | 0.73566 | -0.11428 | 16239 | |
| 4930426D05Rik | 0.73584 | 0.4069 | 16240 | |
| mmu-mir-3105 | 0.73585 | 0.5645 | 16241 | |
| Zfp39 | 0.73587 | 0.4922 | 16242 | |
| Klk9 | 0.73597 | 0.80119 | 16243 | |
| Rhox2a | 0.73602 | 0.074744 | 16244 | |
| Arsj | 0.73606 | 1.1221 | 16245 | |
| Ebf3 | 0.7361 | 0.96724 | 16246 | |
| Ltbp1 | 0.73615 | -0.363 | 16247 | |
| Neurog3 | 0.73617 | -0.32246 | 16248 | |
| Lrrc46 | 0.73632 | -0.41077 | 16249 | |
| Arntl2 | 0.7364 | 0.083646 | 16250 | |
| mmu-let-7b | 0.73652 | 0.29462 | 16251 | |
| Gpr126 | 0.73653 | -0.33256 | 16252 | |
| 1600002H07Rik | 0.73657 | -0.39345 | 16253 | |
| Synj2 | 0.73664 | 0.20163 | 16254 | |
| Stfa3 | 0.7367 | 2.5516 | 16255 | |
| Hist3h2ba | 0.73675 | 0.55662 | 16256 | |
| Plaur | 0.73677 | -0.1588 | 16257 | |
| Ppm1j | 0.73681 | 1.1872 | 16258 | |
| Pcdhb14 | 0.73689 | 0.48517 | 16259 | |
| Gpr82 | 0.73699 | -0.37361 | 16260 | |
| Olfr972 | 0.73703 | 0.43007 | 16261 | |
| Pomt2 | 0.73705 | -0.15861 | 16262 | |
| Mb21d2 | 0.7372 | 0.85504 | 16263 | |
| Gm11541 | 0.73728 | 0.99682 | 16264 | |
| Krt9 | 0.73739 | 0.19983 | 16265 | |
| Cmtm2b | 0.73743 | -0.27611 | 16266 | |
| Rnd3 | 0.73746 | 0.60658 | 16267 | |
| Cttnbp2 | 0.73748 | -0.1408 | 16268 | |
| Maml1 | 0.7375 | -0.012924 | 16269 | |
| Ifng | 0.73752 | -0.34871 | 16270 | |
| Itgb2l | 0.73754 | 0.49299 | 16271 | |
| Asxl3 | 0.73756 | 0.32716 | 16272 | |
| Mterf1a | 0.73767 | -0.30778 | 16273 | |
| mmu-mir-363 | 0.73775 | 0.0087609 | 16274 | |
| Brms1l | 0.73782 | -0.35303 | 16275 | |
| Ncstn | 0.73792 | -0.031917 | 16276 | |
| Zdhhc12 | 0.73795 | 0.8232 | 16277 | |
| Plxna4 | 0.73798 | 0.13315 | 16278 | |
| Foxd2 | 0.73798 | 0.13839 | 16279 | |
| 5830415F09Rik | 0.73802 | 0.067252 | 16280 | |
| Lrp12 | 0.73804 | 0.80326 | 16281 | |
| Fkbp4 | 0.7381 | 0.1306 | 16282 | |
| Klf5 | 0.73817 | -0.013926 | 16283 | |
| Gm14446 | 0.73828 | 0.38536 | 16284 | |
| Olfr1143 | 0.73843 | -0.065448 | 16285 | |
| Klhl28 | 0.73847 | 0.11846 | 16286 | |
| Slc26a3 | 0.73851 | 0.1812 | 16287 | |
| Tmem70 | 0.73853 | 1.3652 | 16288 | |
| Zranb2 | 0.73857 | 0.48175 | 16289 | |
| Sqle | 0.7386 | 2.1741 | 16290 | |
| Neurod6 | 0.73865 | 0.3718 | 16291 | |
| Shmt1 | 0.73879 | 0.15091 | 16292 | |
| Tshz3 | 0.73886 | -0.2306 | 16293 | |
| Zfp442 | 0.73888 | 0.65545 | 16294 | |
| Cpne8 | 0.7389 | 0.48729 | 16295 | |
| 9330182O14Rik | 0.73897 | -0.38428 | 16296 | |
| Olfr1511 | 0.73897 | 0.5747 | 16297 | |
| Slc25a29 | 0.73906 | 0.93622 | 16298 | |
| Gm8298 | 0.73931 | 0.30111 | 16299 | |
| mmu-mir-98 | 0.73932 | 0.1025 | 16300 | |
| Cypt1 | 0.73936 | -0.092721 | 16301 | |
| Pdcd1 | 0.7394 | 0.0048766 | 16302 | |
| mmu-mir-335 | 0.73943 | 2.1677 | 16303 | |
| Vmn1r170 | 0.73945 | -0.43801 | 16304 | |
| Irs2 | 0.73955 | -0.026182 | 16305 | |
| Evi5 | 0.7397 | -0.21869 | 16306 | |
| Gch1 | 0.73977 | -0.25355 | 16307 | |
| Cd97 | 0.73983 | 1.3468 | 16308 | |
| Gzmf | 0.73988 | 0.29261 | 16309 | |
| Slc25a41 | 0.73994 | 0.97723 | 16310 | |
| Myh8 | 0.73997 | 0.33442 | 16311 | |
| Uap1l1 | 0.74009 | 0.097254 | 16312 | |
| Six5 | 0.74021 | 0.66267 | 16313 | |
| Fahd1 | 0.7403 | 0.28811 | 16314 | |
| Trim33 | 0.74045 | 0.6748 | 16315 | |
| Mboat2 | 0.74062 | 0.70295 | 16316 | |
| Rom1 | 0.74071 | 1.867 | 16317 | |
| Dusp3 | 0.74073 | 0.09172 | 16318 | |
| Dis3l2 | 0.74078 | -0.13028 | 16319 | |
| Olfr1045 | 0.74078 | 2.171 | 16320 | |
| Olfr49 | 0.74109 | -0.12089 | 16321 | |
| Fbxw20 | 0.74112 | 0.17591 | 16322 | |
| Rps6kl1 | 0.7412 | 0.24094 | 16323 | |
| Micu2 | 0.74133 | 0.89951 | 16324 | |
| Fam53c | 0.74142 | 0.13916 | 16325 | |
| Kcnj9 | 0.74145 | 1.0055 | 16326 | |
| mmu-mir-547 | 0.74161 | 0.17363 | 16327 | |
| Chrna6 | 0.74164 | 1.2516 | 16328 | |
| Spa17 | 0.74172 | 0.72051 | 16329 | |
| Cyp2c54 | 0.74172 | 0.54448 | 16330 | |
| Egr1 | 0.74181 | 1.0635 | 16331 | |
| Smco2 | 0.74182 | 0.77514 | 16332 | |
| Drd4 | 0.74192 | -0.25462 | 16333 | |
| Corin | 0.74194 | 0.63905 | 16334 | |
| Ephb2 | 0.74197 | -0.028737 | 16335 | |
| Thap4 | 0.74199 | 0.11876 | 16336 | |
| Slitrk5 | 0.74203 | -0.35897 | 16337 | |
| Fam46a | 0.74209 | 1.1931 | 16338 | |
| Il20 | 0.74214 | 0.088257 | 16339 | |
| Tor4a | 0.74221 | -0.27015 | 16340 | |
| Matn3 | 0.74234 | 0.2428 | 16341 | |
| B4galnt1 | 0.74245 | 0.62563 | 16342 | |
| Gp2 | 0.74249 | -0.068638 | 16343 | |
| Gm11596 | 0.74252 | 0.34197 | 16344 | |
| Lgals6 | 0.74254 | 1.4655 | 16345 | |
| Adam17 | 0.74256 | -0.32416 | 16346 | |
| Adcy1 | 0.74258 | -0.2853 | 16347 | |
| Olfr160 | 0.74262 | -0.19277 | 16348 | |
| Dcbld2 | 0.74263 | 0.15197 | 16349 | |
| Cpa2 | 0.74271 | 0.9957 | 16350 | |
| Prpf39 | 0.74293 | 0.37729 | 16351 | |
| Scaf11 | 0.74306 | 0.051608 | 16352 | |
| Ppp1r1b | 0.74308 | -0.28242 | 16353 | |
| Olfr1034 | 0.74309 | 0.041421 | 16354 | |
| Cbx8 | 0.74319 | 0.098374 | 16355 | |
| Usp8 | 0.74343 | 1.2099 | 16356 | |
| Ston1 | 0.74344 | -0.31043 | 16357 | |
| Defb35 | 0.74363 | -0.31506 | 16358 | |
| Gtf2ird1 | 0.7437 | -0.4209 | 16359 | |
| Gm10058 | 0.7439 | -0.15998 | 16360 | |
| Tax1bp1 | 0.74405 | -0.39641 | 16361 | |
| Gm19668 | 0.74429 | 0.13548 | 16362 | |
| Rin1 | 0.74431 | 0.13552 | 16363 | |
| Snx19 | 0.74441 | 1.1719 | 16364 | |
| Diablo | 0.74445 | -0.04051 | 16365 | |
| 1110051M20Rik | 0.74455 | 0.4074 | 16366 | |
| Cpsf4 | 0.74466 | 0.4982 | 16367 | |
| Ptbp3 | 0.74467 | -0.37402 | 16368 | |
| Asb5 | 0.74471 | 0.31628 | 16369 | |
| Ltf | 0.74475 | 0.55224 | 16370 | |
| Hoxa2 | 0.7448 | 0.52881 | 16371 | |
| 4930563D23Rik | 0.74488 | 0.090936 | 16372 | |
| mmu-mir-223 | 0.74498 | 2.3209 | 16373 | |
| Hadhb | 0.745 | 2.1673 | 16374 | |
| Ripply1 | 0.74506 | 0.40331 | 16375 | |
| Tbc1d9b | 0.74519 | 0.55489 | 16376 | |
| Trpv1 | 0.74524 | -0.046924 | 16377 | |
| Ntn5 | 0.74526 | 0.068397 | 16378 | |
| Pou3f1 | 0.74531 | 0.95256 | 16379 | |
| Slc7a15 | 0.74531 | -0.2329 | 16380 | |
| Hoxc10 | 0.74535 | -0.112 | 16381 | |
| 2610507B11Rik | 0.74552 | 0.73904 | 16382 | |
| Olfr1299 | 0.74564 | 0.097863 | 16383 | |
| Gm4980 | 0.74566 | 1.2665 | 16384 | |
| Tcfl5 | 0.74571 | -0.25879 | 16385 | |
| Vmn1r52 | 0.74574 | 0.60361 | 16386 | |
| Sos1 | 0.74606 | 0.10868 | 16387 | |
| Prmt8 | 0.74614 | -0.3889 | 16388 | |
| Gss | 0.74615 | 0.048336 | 16389 | |
| Actg2 | 0.74617 | 0.45885 | 16390 | |
| Exosc5 | 0.7462 | -0.36092 | 16391 | |
| Tnfsf15 | 0.74626 | 1.1803 | 16392 | |
| Olfr917 | 0.7463 | 0.25939 | 16393 | |
| 1700019B03Rik | 0.74634 | 0.074082 | 16394 | |
| Olfr146 | 0.74636 | 1.9744 | 16395 | |
| Tulp2 | 0.74643 | -0.067665 | 16396 | |
| Sppl3 | 0.74646 | -0.39154 | 16397 | |
| Olfr1408 | 0.74656 | 0.25404 | 16398 | |
| Zfp689 | 0.74657 | -0.20008 | 16399 | |
| Gzma | 0.74667 | 0.75463 | 16400 | |
| Scgn | 0.74672 | 0.31589 | 16401 | |
| Cybb | 0.74674 | 0.041147 | 16402 | |
| Wdr31 | 0.74676 | 0.31491 | 16403 | |
| Ube2w | 0.74681 | 1.1152 | 16404 | |
| Dcxr | 0.74683 | -0.1456 | 16405 | |
| Il13ra2 | 0.74685 | 0.80806 | 16406 | |
| Ppp1r16b | 0.74697 | -0.33099 | 16407 | |
| BC061212 | 0.74708 | 0.3794 | 16408 | |
| mmu-mir-6965 | 0.74735 | 0.59972 | 16409 | |
| Sdcbp | 0.74736 | 1.4169 | 16410 | |
| Atp11b | 0.74744 | 3.4605 | 16411 | |
| Dcun1d4 | 0.74744 | -0.33597 | 16412 | |
| Clasp1 | 0.74748 | 0.28784 | 16413 | |
| Trpm8 | 0.74752 | -0.21866 | 16414 | |
| Mesp2 | 0.74756 | -0.30385 | 16415 | |
| Krt12 | 0.7476 | -0.21774 | 16416 | |
| AI462493 | 0.74768 | 0.77162 | 16417 | |
| Vmn1r11 | 0.7477 | 1.5512 | 16418 | |
| Csf2rb2 | 0.74774 | 0.030113 | 16419 | |
| Olfr1356 | 0.74785 | 0.74679 | 16420 | |
| Phkg1 | 0.74788 | -0.23551 | 16421 | |
| Cap2 | 0.74799 | -0.095724 | 16422 | |
| Cidea | 0.7481 | -0.37376 | 16423 | |
| Chrne | 0.74828 | -0.41648 | 16424 | |
| Esr2 | 0.74832 | 0.10244 | 16425 | |
| Kcnf1 | 0.74845 | 0.10347 | 16426 | |
| Yipf1 | 0.7485 | 0.34875 | 16427 | |
| 2310050C09Rik | 0.74868 | 0.10536 | 16428 | |
| Grid2ip | 0.74874 | 0.46424 | 16429 | |
| Pla2g12a | 0.74886 | 1.3374 | 16430 | |
| Me3 | 0.74897 | -0.16196 | 16431 | |
| Hist1h1d | 0.74905 | 0.16381 | 16432 | |
| Olfr165 | 0.74919 | -0.096114 | 16433 | |
| Efr3b | 0.74921 | 0.33101 | 16434 | |
| mmu-mir-219c | 0.74922 | 0.40887 | 16435 | |
| Pxmp2 | 0.74927 | -0.35216 | 16436 | |
| Vmn1r12 | 0.74941 | 0.076182 | 16437 | |
| Mef2d | 0.7495 | 1.0455 | 16438 | |
| Btbd8 | 0.7497 | -0.38508 | 16439 | |
| Slit2 | 0.74973 | 0.75688 | 16440 | |
| Kcnb1 | 0.74983 | 1.0158 | 16441 | |
| mmu-mir-669b | 0.74987 | 0.37785 | 16442 | |
| Vamp3 | 0.75004 | -0.068419 | 16443 | |
| A830010M20Rik | 0.75011 | -0.20888 | 16444 | |
| Col11a2 | 0.75015 | 0.077473 | 16445 | |
| Purg | 0.75018 | 0.12604 | 16446 | |
| Gpx5 | 0.75024 | 0.15362 | 16447 | |
| Rad17 | 0.75027 | 0.69938 | 16448 | |
| Pou3f3 | 0.75029 | -0.07919 | 16449 | |
| Tmprss13 | 0.75031 | 0.562 | 16450 | |
| Galnt16 | 0.75038 | 0.28168 | 16451 | |
| Calcoco2 | 0.75046 | 0.53649 | 16452 | |
| Sprtn | 0.75058 | -0.33329 | 16453 | |
| H2afy2 | 0.75064 | 0.16258 | 16454 | |
| Adam25 | 0.75065 | 0.22582 | 16455 | |
| Ubxn10 | 0.75087 | 1.0247 | 16456 | |
| Edaradd | 0.75089 | 0.30635 | 16457 | |
| Lhx9 | 0.75091 | -0.13116 | 16458 | |
| Ndnf | 0.75109 | -0.28027 | 16459 | |
| Serpinb3b | 0.75118 | 0.4867 | 16460 | |
| Mpc1 | 0.75119 | -0.35795 | 16461 | |
| Pdia6 | 0.75123 | 0.25657 | 16462 | |
| Olfr1099 | 0.75127 | 0.046215 | 16463 | |
| Oit1 | 0.75157 | 0.29143 | 16464 | |
| Zfp532 | 0.75159 | 0.86463 | 16465 | |
| Prkch | 0.75166 | 0.12935 | 16466 | |
| Olfr796 | 0.75173 | -0.03879 | 16467 | |
| Samd3 | 0.75177 | 0.62318 | 16468 | |
| Itga3 | 0.7519 | 0.65064 | 16469 | |
| Ebna1bp2 | 0.75192 | -0.34781 | 16470 | |
| Aftph | 0.75216 | 0.19203 | 16471 | |
| Scgb3a1 | 0.75242 | 0.48948 | 16472 | |
| Prkcg | 0.75245 | 0.1367 | 16473 | |
| Gm4745 | 0.7526 | 0.9496 | 16474 | |
| Mpeg1 | 0.75261 | -0.12801 | 16475 | |
| Ifitm10 | 0.75267 | 0.35761 | 16476 | |
| mmu-mir-6933 | 0.7527 | 0.65241 | 16477 | |
| Olfm1 | 0.75274 | -0.0041111 | 16478 | |
| Cd55 | 0.75277 | 1.6434 | 16479 | |
| Fam170b | 0.75288 | -0.2436 | 16480 | |
| Olfr346 | 0.7529 | 0.87949 | 16481 | |
| Smim15 | 0.75294 | 1.238 | 16482 | |
| 4930523C07Rik | 0.75299 | 1.484 | 16483 | |
| Adpgk | 0.75303 | 1.0883 | 16484 | |
| Ctsq | 0.75319 | 0.532 | 16485 | |
| Trmt44 | 0.75322 | -0.37687 | 16486 | |
| BC004004 | 0.75333 | 0.31072 | 16487 | |
| Olfr398 | 0.75342 | 0.50245 | 16488 | |
| Msh3 | 0.75346 | 0.089958 | 16489 | |
| Smox | 0.75363 | 0.31501 | 16490 | |
| Tnfrsf17 | 0.75369 | 0.18768 | 16491 | |
| Fbxo10 | 0.75376 | 0.10361 | 16492 | |
| Gm13125 | 0.75378 | -0.1788 | 16493 | |
| Mapk3 | 0.75381 | 0.65896 | 16494 | |
| Krtap4-9 | 0.75405 | -0.30136 | 16495 | |
| Prkacb | 0.75408 | 0.60679 | 16496 | |
| Mb21d1 | 0.75416 | -0.40571 | 16497 | |
| Bcdin3d | 0.75422 | 0.63943 | 16498 | |
| P2rx6 | 0.75424 | 0.22822 | 16499 | |
| Olfr421-ps1 | 0.75426 | 0.027676 | 16500 | |
| Nktr | 0.75431 | -0.062577 | 16501 | |
| Kifc1 | 0.75437 | 0.1156 | 16502 | |
| C1ql3 | 0.75456 | 0.054414 | 16503 | |
| Slc27a6 | 0.7546 | 0.17595 | 16504 | |
| Snrpc | 0.75462 | 0.096995 | 16505 | |
| mmu-mir-6342 | 0.75463 | 0.39866 | 16506 | |
| Mybphl | 0.75463 | 0.19071 | 16507 | |
| Ankrd10 | 0.75465 | 0.045421 | 16508 | |
| Robo4 | 0.75488 | 0.94419 | 16509 | |
| Kbtbd3 | 0.7549 | 0.42084 | 16510 | |
| Abat | 0.75513 | -0.23674 | 16511 | |
| Fmo9 | 0.75515 | -0.25313 | 16512 | |
| Gucy2g | 0.75519 | 0.086045 | 16513 | |
| Nap1l2 | 0.75537 | 0.91 | 16514 | |
| Insig1 | 0.75538 | 1.5256 | 16515 | |
| Thpo | 0.75544 | -0.16104 | 16516 | |
| Xab2 | 0.75564 | -0.3303 | 16517 | |
| Klra33 | 0.75567 | 1.2238 | 16518 | |
| Rab32 | 0.75578 | 0.697 | 16519 | |
| Nkx2-2 | 0.75583 | 0.38941 | 16520 | |
| Adssl1 | 0.75585 | 0.021505 | 16521 | |
| Aen | 0.75593 | -0.27316 | 16522 | |
| Olfr1216 | 0.75601 | -0.19418 | 16523 | |
| Dok7 | 0.75602 | 0.063489 | 16524 | |
| Zfp579 | 0.75611 | 0.05947 | 16525 | |
| Rnf125 | 0.75622 | 0.26835 | 16526 | |
| Fcho2 | 0.75625 | -0.31791 | 16527 | |
| Olfr1335 | 0.75625 | -0.21469 | 16528 | |
| Capg | 0.75633 | 1.1807 | 16529 | |
| Zdhhc25 | 0.75634 | -0.31988 | 16530 | |
| Erbb2ip | 0.75638 | 1.3826 | 16531 | |
| Tuba1c | 0.75641 | 0.20159 | 16532 | |
| Cntnap5c | 0.75643 | -0.085436 | 16533 | |
| Zfp217 | 0.75646 | -0.39549 | 16534 | |
| C3 | 0.75649 | 0.27583 | 16535 | |
| Olfr166 | 0.75659 | -0.14228 | 16536 | |
| AA414768 | 0.75668 | -0.36704 | 16537 | |
| Olfr350 | 0.7567 | 0.48636 | 16538 | |
| Ddah2 | 0.75673 | 1.1975 | 16539 | |
| A4gnt | 0.75679 | 1.9151 | 16540 | |
| Ret | 0.75682 | 0.68433 | 16541 | |
| Plscr2 | 0.757 | -0.094636 | 16542 | |
| Pkp3 | 0.75708 | 0.067055 | 16543 | |
| H2-DMb1 | 0.75727 | -0.25986 | 16544 | |
| Olfr1225 | 0.7573 | 0.47323 | 16545 | |
| Asic5 | 0.75732 | -0.42674 | 16546 | |
| Zfp563 | 0.75738 | 0.2432 | 16547 | |
| Rgs17 | 0.75747 | 0.13551 | 16548 | |
| Igj | 0.7575 | 1.3226 | 16549 | |
| Mnd1 | 0.75751 | 0.71827 | 16550 | |
| Cpvl | 0.75762 | 0.12636 | 16551 | |
| Npw | 0.75764 | -0.35386 | 16552 | |
| Olfr1048 | 0.75773 | -0.010443 | 16553 | |
| Vmn1r19 | 0.75787 | 0.24734 | 16554 | |
| Stk38l | 0.75789 | 0.13558 | 16555 | |
| Krtdap | 0.75789 | 0.32881 | 16556 | |
| Shroom4 | 0.75794 | 1.7069 | 16557 | |
| Gm5347 | 0.75796 | -0.29634 | 16558 | |
| Rell1 | 0.75799 | -0.2462 | 16559 | |
| Paqr7 | 0.75801 | 0.25189 | 16560 | |
| Tff1 | 0.75803 | -0.20907 | 16561 | |
| Tas2r102 | 0.75806 | 0.50465 | 16562 | |
| Olfr802 | 0.75813 | 1.6451 | 16563 | |
| Rapgef5 | 0.75822 | -0.01603 | 16564 | |
| Tex35 | 0.75824 | -0.023391 | 16565 | |
| Slc7a14 | 0.75827 | 0.24458 | 16566 | |
| Vpreb1 | 0.7583 | 1.0276 | 16567 | |
| Prrt3 | 0.75836 | -0.2041 | 16568 | |
| Prss52 | 0.75845 | 0.4816 | 16569 | |
| Lgr5 | 0.75847 | 0.54554 | 16570 | |
| Gpr56 | 0.75864 | 0.46461 | 16571 | |
| Stk35 | 0.75877 | 0.2548 | 16572 | |
| Bphl | 0.75886 | 0.18013 | 16573 | |
| Pnoc | 0.75889 | 1.1386 | 16574 | |
| Vmn2r42 | 0.75912 | 0.6508 | 16575 | |
| Pth2r | 0.75912 | -0.15148 | 16576 | |
| Rnf17 | 0.75933 | 0.97417 | 16577 | |
| Rab13 | 0.75937 | 0.1185 | 16578 | |
| Rbbp6 | 0.75948 | 1.8944 | 16579 | |
| Lgals4 | 0.75954 | -0.27482 | 16580 | |
| Akr1c19 | 0.75961 | -0.35571 | 16581 | |
| Cdk5r1 | 0.75975 | 1.0075 | 16582 | |
| Gm5820 | 0.75977 | -0.24447 | 16583 | |
| Dbx1 | 0.7599 | 0.26953 | 16584 | |
| Myocd | 0.75993 | 1.5542 | 16585 | |
| Igfals | 0.75997 | 0.59817 | 16586 | |
| Zdhhc17 | 0.76002 | 0.26236 | 16587 | |
| Zfyve26 | 0.76005 | -0.2569 | 16588 | |
| Spire2 | 0.76009 | -0.16591 | 16589 | |
| Dcaf12l1 | 0.76012 | -0.35575 | 16590 | |
| Car15 | 0.76023 | 0.35305 | 16591 | |
| mmu-mir-302c | 0.76024 | 1.388 | 16592 | |
| Olfr849 | 0.76026 | 0.042635 | 16593 | |
| Zfp639 | 0.76033 | -0.40513 | 16594 | |
| Casp2 | 0.76042 | 1.3233 | 16595 | |
| Mblac1 | 0.76047 | -0.32366 | 16596 | |
| Arid4a | 0.76079 | 0.88133 | 16597 | |
| Vmn1r4 | 0.76087 | -0.35669 | 16598 | |
| Vamp2 | 0.761 | 0.052791 | 16599 | |
| Dus4l | 0.76104 | 1.4153 | 16600 | |
| Btn2a2 | 0.76105 | 0.70754 | 16601 | |
| Fam228a | 0.76111 | -0.38528 | 16602 | |
| Akr1c18 | 0.76112 | 0.23628 | 16603 | |
| Cacnb4 | 0.76114 | 0.16321 | 16604 | |
| Rbm46 | 0.76132 | 0.83862 | 16605 | |
| Dcaf6 | 0.76139 | 0.14658 | 16606 | |
| Zfp866 | 0.76146 | 0.077568 | 16607 | |
| Map2k3 | 0.76149 | 0.32544 | 16608 | |
| Cnbd2 | 0.76168 | 1.7171 | 16609 | |
| Olfr1095 | 0.76177 | -0.28717 | 16610 | |
| Gabrb3 | 0.76182 | 0.78161 | 16611 | |
| Abca1 | 0.76182 | 0.22045 | 16612 | |
| Pus7 | 0.76189 | -0.035139 | 16613 | |
| Krt71 | 0.76191 | 1.0586 | 16614 | |
| Prl8a9 | 0.76196 | -0.12016 | 16615 | |
| Hspa14 | 0.76202 | 0.6112 | 16616 | |
| Zcrb1 | 0.76205 | 0.68421 | 16617 | |
| Tcerg1 | 0.76207 | 0.50072 | 16618 | |
| Olfr1413 | 0.76209 | 0.59526 | 16619 | |
| Cspg5 | 0.76228 | 0.83763 | 16620 | |
| 1700030K09Rik | 0.76233 | 0.25979 | 16621 | |
| Plxnc1 | 0.76242 | -0.40389 | 16622 | |
| Zic1 | 0.76252 | 0.55744 | 16623 | |
| Tmprss5 | 0.76254 | -0.31037 | 16624 | |
| Spin2c | 0.76258 | 0.74488 | 16625 | |
| Mphosph9 | 0.76263 | 0.83469 | 16626 | |
| Gm11562 | 0.76266 | 0.50336 | 16627 | |
| Dazap2 | 0.7627 | 0.12259 | 16628 | |
| Ptgfrn | 0.76275 | -0.098407 | 16629 | |
| Gprc6a | 0.76278 | 1.0098 | 16630 | |
| Raet1b | 0.76284 | 1.4455 | 16631 | |
| Olfr100 | 0.76296 | 0.45686 | 16632 | |
| AF529169 | 0.763 | -0.38027 | 16633 | |
| Myzap | 0.76301 | 0.077221 | 16634 | |
| Rax | 0.7631 | -0.35337 | 16635 | |
| Uhrf1bp1 | 0.76312 | 0.71027 | 16636 | |
| H2afx | 0.76326 | -0.39809 | 16637 | |
| BC048562 | 0.76337 | 0.33626 | 16638 | |
| Coro2b | 0.76348 | 0.36218 | 16639 | |
| Dera | 0.76374 | 0.41526 | 16640 | |
| Ggt1 | 0.76378 | 0.65556 | 16641 | |
| Plxna3 | 0.76379 | 0.098119 | 16642 | |
| Myl9 | 0.7639 | 0.21571 | 16643 | |
| Barx2 | 0.76399 | 0.72814 | 16644 | |
| Hsf2 | 0.764 | -0.22958 | 16645 | |
| Chat | 0.76404 | 1.4768 | 16646 | |
| Tbc1d10a | 0.76418 | -0.27031 | 16647 | |
| Col4a4 | 0.76425 | 0.30931 | 16648 | |
| Lim2 | 0.76444 | 0.1545 | 16649 | |
| Cdv3 | 0.76447 | -0.2854 | 16650 | |
| Vav3 | 0.76451 | -0.12904 | 16651 | |
| Olfr33 | 0.76456 | 0.015467 | 16652 | |
| Tspan14 | 0.76475 | 0.15463 | 16653 | |
| Apbb2 | 0.76478 | 0.6164 | 16654 | |
| Olfr1436 | 0.76484 | 0.59526 | 16655 | |
| Vmn1r181 | 0.76491 | -0.10281 | 16656 | |
| Zmynd19 | 0.765 | 0.05386 | 16657 | |
| Gng7 | 0.76505 | 0.15566 | 16658 | |
| Fbxl8 | 0.76513 | 0.16884 | 16659 | |
| Cav3 | 0.76524 | -0.11553 | 16660 | |
| Olfr826 | 0.7653 | 0.85519 | 16661 | |
| Foxe1 | 0.76532 | -0.23537 | 16662 | |
| Lekr1 | 0.76537 | 0.37569 | 16663 | |
| 4921530D09Rik | 0.76543 | 0.43194 | 16664 | |
| Mfsd2b | 0.76551 | 0.11209 | 16665 | |
| Ank1 | 0.7656 | -0.28409 | 16666 | |
| Zfp422 | 0.76562 | 0.5831 | 16667 | |
| Slc22a21 | 0.7657 | 0.21683 | 16668 | |
| Il33 | 0.76579 | -0.058778 | 16669 | |
| C86695 | 0.76581 | 0.50537 | 16670 | |
| Usp32 | 0.76596 | 1.0496 | 16671 | |
| Cd7 | 0.76598 | 0.36595 | 16672 | |
| 2300002M23Rik | 0.76601 | 0.45564 | 16673 | |
| Tmem150b | 0.76607 | 0.040573 | 16674 | |
| Cntn2 | 0.76608 | 0.27095 | 16675 | |
| Gm12597 | 0.7661 | 0.17471 | 16676 | |
| Ccdc77 | 0.76615 | 1.0896 | 16677 | |
| Edf1 | 0.7662 | 1.0509 | 16678 | |
| Hipk3 | 0.76626 | -0.38645 | 16679 | |
| Tmc8 | 0.76627 | 0.41754 | 16680 | |
| Gm19402 | 0.76629 | 1.0096 | 16681 | |
| Rarres1 | 0.76633 | 0.16021 | 16682 | |
| Manea | 0.76636 | 0.99205 | 16683 | |
| Fig4 | 0.76638 | 0.83366 | 16684 | |
| Klhl40 | 0.76645 | 0.47004 | 16685 | |
| Smarcc1 | 0.76646 | -0.34176 | 16686 | |
| Cmbl | 0.76648 | -0.042912 | 16687 | |
| Foxc1 | 0.76652 | 0.22518 | 16688 | |
| Tas2r115 | 0.7666 | 1.11 | 16689 | |
| Stambpl1 | 0.76674 | 2.1103 | 16690 | |
| Olfr698 | 0.76676 | -0.10794 | 16691 | |
| Pdzd8 | 0.76677 | -0.38444 | 16692 | |
| Parl | 0.76679 | -0.27143 | 16693 | |
| Slc5a6 | 0.76689 | -0.17704 | 16694 | |
| Zbtb20 | 0.76707 | 0.19867 | 16695 | |
| Hr | 0.7672 | 0.25232 | 16696 | |
| Rbl1 | 0.76722 | 0.30556 | 16697 | |
| Rph3a | 0.76726 | 0.09827 | 16698 | |
| Bpifb2 | 0.76729 | -0.1586 | 16699 | |
| 1700037H04Rik | 0.76731 | 0.63322 | 16700 | |
| Apol9a | 0.76739 | 0.021989 | 16701 | |
| Olfr570 | 0.76741 | 0.32713 | 16702 | |
| Hspb1 | 0.7675 | 0.2814 | 16703 | |
| Lpxn | 0.76755 | -0.20275 | 16704 | |
| Smchd1 | 0.76757 | 0.48896 | 16705 | |
| Glyatl3 | 0.76782 | -0.14648 | 16706 | |
| Olfr1261 | 0.76784 | 0.25828 | 16707 | |
| Pusl1 | 0.76793 | 0.36371 | 16708 | |
| Lypd3 | 0.76796 | -0.24043 | 16709 | |
| Prss35 | 0.76805 | 0.31854 | 16710 | |
| Gabpb2 | 0.76819 | -0.098846 | 16711 | |
| Zc3h8 | 0.76824 | -0.0094406 | 16712 | |
| Phactr2 | 0.76825 | 0.93925 | 16713 | |
| Chrm3 | 0.76827 | 0.080638 | 16714 | |
| Mtmr12 | 0.76834 | -0.0086711 | 16715 | |
| Gsg1l | 0.76839 | 0.19859 | 16716 | |
| Zfy2 | 0.76848 | 1.2578 | 16717 | |
| Ube2e1 | 0.76855 | 0.05726 | 16718 | |
| Cnp | 0.76875 | 0.39352 | 16719 | |
| H2-Bl | 0.76887 | 0.1353 | 16720 | |
| Slc35g3 | 0.76889 | 1.1532 | 16721 | |
| 1810019J16Rik | 0.76894 | 0.065641 | 16722 | |
| Vmn2r76 | 0.76897 | 0.61156 | 16723 | |
| Rassf10 | 0.76903 | 0.30573 | 16724 | |
| Slc9a7 | 0.76908 | -0.34619 | 16725 | |
| Tnfrsf22 | 0.76908 | -0.19293 | 16726 | |
| Synj1 | 0.76913 | 0.72171 | 16727 | |
| Olfr487 | 0.76915 | -0.24689 | 16728 | |
| Lamp3 | 0.76929 | 0.5293 | 16729 | |
| Cyp2c70 | 0.76937 | 0.38353 | 16730 | |
| Gbp11 | 0.76942 | 0.10998 | 16731 | |
| Trem1 | 0.76947 | 1.4802 | 16732 | |
| Nfkbil1 | 0.76952 | 0.34841 | 16733 | |
| Crcp | 0.76969 | 1.1914 | 16734 | |
| Pkig | 0.76974 | -0.22559 | 16735 | |
| Col26a1 | 0.76981 | -0.031407 | 16736 | |
| Hoxb5 | 0.76985 | -0.2109 | 16737 | |
| Impg1 | 0.77002 | -0.1422 | 16738 | |
| Actrt3 | 0.77004 | 0.13586 | 16739 | |
| Galnt5 | 0.77005 | 0.10903 | 16740 | |
| Saa3 | 0.77009 | -0.24081 | 16741 | |
| Gm12887 | 0.77012 | -0.039563 | 16742 | |
| Tmem38a | 0.77021 | -0.091676 | 16743 | |
| Pctp | 0.77027 | -0.025934 | 16744 | |
| Rtn4r | 0.77029 | -0.35201 | 16745 | |
| Ctrb1 | 0.77034 | -0.32671 | 16746 | |
| Ccl4 | 0.77079 | 0.95616 | 16747 | |
| Tox3 | 0.77091 | 0.32213 | 16748 | |
| 1190002N15Rik | 0.77096 | 0.013303 | 16749 | |
| mmu-mir-324 | 0.77108 | 1.0635 | 16750 | |
| Olfr524 | 0.77108 | -0.050159 | 16751 | |
| C1ql4 | 0.77114 | -0.15105 | 16752 | |
| Tmem246 | 0.77123 | 0.07892 | 16753 | |
| Lrrn2 | 0.77126 | -0.095132 | 16754 | |
| Gm410 | 0.7713 | 0.16136 | 16755 | |
| Myo1e | 0.77133 | 1.9029 | 16756 | |
| Bpifb9a | 0.77154 | 1.4198 | 16757 | |
| Dio3 | 0.7716 | 1.2899 | 16758 | |
| Csrnp2 | 0.77167 | 0.29289 | 16759 | |
| Taar4 | 0.77169 | -0.34187 | 16760 | |
| Slc25a23 | 0.77184 | 0.65568 | 16761 | |
| Ctsf | 0.77186 | 0.41507 | 16762 | |
| Loh12cr1 | 0.77188 | 0.032774 | 16763 | |
| Ppp1r12c | 0.77201 | -0.11908 | 16764 | |
| Slc9a3r1 | 0.77213 | -0.25236 | 16765 | |
| Tmppe | 0.77218 | -0.22549 | 16766 | |
| Eef2k | 0.7722 | 0.059589 | 16767 | |
| Dnajb7 | 0.77233 | -0.28435 | 16768 | |
| Dhx57 | 0.77235 | 0.70396 | 16769 | |
| Olfr183 | 0.77245 | -0.10076 | 16770 | |
| Best2 | 0.77247 | -0.38528 | 16771 | |
| Lcp1 | 0.77249 | 0.3138 | 16772 | |
| Elovl5 | 0.7725 | 0.32713 | 16773 | |
| Gls2 | 0.77252 | 0.50287 | 16774 | |
| Gm20806 | 0.77258 | 0.35073 | 16775 | |
| Kbtbd8 | 0.77262 | 1.3477 | 16776 | |
| Celf5 | 0.77282 | 0.5832 | 16777 | |
| Agbl3 | 0.77285 | -0.17837 | 16778 | |
| Sbp | 0.77285 | 0.65212 | 16779 | |
| Gm2933 | 0.77288 | 0.47983 | 16780 | |
| Cldn8 | 0.77291 | -0.23739 | 16781 | |
| 4930567H17Rik | 0.77333 | 0.30497 | 16782 | |
| BC053749 | 0.77336 | 0.28433 | 16783 | |
| Vmn1r38 | 0.77338 | 0.17271 | 16784 | |
| Prss30 | 0.77342 | 0.67969 | 16785 | |
| Zscan4f | 0.77344 | 0.4119 | 16786 | |
| Papl | 0.77355 | 1.4118 | 16787 | |
| Ptprk | 0.77369 | 2.8014 | 16788 | |
| Srcin1 | 0.77377 | -0.26437 | 16789 | |
| Evx2 | 0.77384 | -0.11523 | 16790 | |
| Ndufa8 | 0.77386 | -0.16392 | 16791 | |
| Ceacam14 | 0.77393 | -0.41606 | 16792 | |
| Emid1 | 0.77408 | 0.15751 | 16793 | |
| Arhgap12 | 0.77409 | -0.16474 | 16794 | |
| Olfr551 | 0.77422 | -0.072491 | 16795 | |
| Bhlhe22 | 0.7743 | 0.182 | 16796 | |
| Wnt3a | 0.7745 | -0.02015 | 16797 | |
| Vmn2r24 | 0.77452 | 0.75795 | 16798 | |
| Ntn4 | 0.77453 | -0.04911 | 16799 | |
| Shank1 | 0.77462 | 0.38824 | 16800 | |
| Olfr294 | 0.77463 | 0.47098 | 16801 | |
| Fam32a | 0.77487 | 2.726 | 16802 | |
| 2410127L17Rik | 0.77503 | 0.13261 | 16803 | |
| Rnf152 | 0.77507 | 0.029597 | 16804 | |
| Galntl6 | 0.77509 | 0.93845 | 16805 | |
| Chsy3 | 0.77516 | 0.37679 | 16806 | |
| Mlph | 0.7752 | 0.31799 | 16807 | |
| Scn3a | 0.77527 | 0.02559 | 16808 | |
| Ccl27b | 0.77532 | 0.21732 | 16809 | |
| Oas1e | 0.77536 | 0.035252 | 16810 | |
| Dnajc13 | 0.77548 | -0.41673 | 16811 | |
| Ifnb1 | 0.77563 | 0.67292 | 16812 | |
| Sox1 | 0.77569 | -0.11948 | 16813 | |
| 2810408A11Rik | 0.7758 | -0.17507 | 16814 | |
| Col24a1 | 0.77585 | 0.042147 | 16815 | |
| Krt10 | 0.77591 | 0.30123 | 16816 | |
| Inpp5a | 0.776 | 0.54567 | 16817 | |
| Gm11744 | 0.77601 | 0.25684 | 16818 | |
| Otor | 0.77603 | 1.3145 | 16819 | |
| Olfr433 | 0.77606 | -0.32368 | 16820 | |
| Ptdss2 | 0.7761 | 0.95596 | 16821 | |
| Mog | 0.77611 | 0.27842 | 16822 | |
| Exoc1 | 0.77618 | 1.5322 | 16823 | |
| Fbxo8 | 0.7763 | 1.0911 | 16824 | |
| Set | 0.7764 | 0.28766 | 16825 | |
| 4930402F06Rik | 0.77648 | 0.20228 | 16826 | |
| Zfp704 | 0.77657 | -0.26287 | 16827 | |
| Gm11437 | 0.77679 | -0.30378 | 16828 | |
| Grap2 | 0.7768 | -0.20115 | 16829 | |
| Dnajc10 | 0.77682 | 0.92755 | 16830 | |
| Acyp1 | 0.77684 | 0.095511 | 16831 | |
| Olfr822 | 0.77694 | -0.020677 | 16832 | |
| Xrcc5 | 0.77699 | 0.31774 | 16833 | |
| mmu-mir-5123 | 0.77707 | 0.39334 | 16834 | |
| Igf1 | 0.77714 | 0.66388 | 16835 | |
| Sftpb | 0.77719 | 0.15454 | 16836 | |
| Kat2a | 0.77734 | 0.71223 | 16837 | |
| Nxpe4 | 0.77735 | 0.81363 | 16838 | |
| Mark4 | 0.77749 | -0.22934 | 16839 | |
| Olfr1322 | 0.77752 | -0.42385 | 16840 | |
| Tmem186 | 0.77767 | 0.078677 | 16841 | |
| Minos1 | 0.77769 | -0.42411 | 16842 | |
| Stk17b | 0.7777 | 0.36285 | 16843 | |
| Chsy1 | 0.77774 | 0.29402 | 16844 | |
| Dnm1 | 0.77782 | 0.41324 | 16845 | |
| Ly9 | 0.77787 | -0.17669 | 16846 | |
| Ssbp2 | 0.77798 | -0.39414 | 16847 | |
| Rcbtb2 | 0.77807 | 0.62386 | 16848 | |
| Tgoln1 | 0.77816 | 0.27555 | 16849 | |
| Tbc1d23 | 0.77817 | 0.086988 | 16850 | |
| Prkag1 | 0.77822 | 0.95419 | 16851 | |
| Pom121l2 | 0.77824 | 1.0967 | 16852 | |
| Ift140 | 0.77827 | -0.22838 | 16853 | |
| Gde1 | 0.77832 | 0.26577 | 16854 | |
| Gm11938 | 0.77841 | -0.40518 | 16855 | |
| Tfap4 | 0.7785 | 2.467 | 16856 | |
| Gnptg | 0.77872 | 0.056533 | 16857 | |
| Dcpp1 | 0.77889 | -0.039624 | 16858 | |
| Kat7 | 0.77894 | 0.52365 | 16859 | |
| mmu-mir-467d | 0.77898 | 0.52652 | 16860 | |
| Yaf2 | 0.77899 | -0.18229 | 16861 | |
| Evx1 | 0.77902 | 0.61209 | 16862 | |
| mmu-mir-6932 | 0.77904 | 1.0341 | 16863 | |
| Acvr1 | 0.77919 | -0.13106 | 16864 | |
| Sla | 0.77925 | -0.08696 | 16865 | |
| Siglec5 | 0.77929 | -0.10826 | 16866 | |
| mmu-mir-128-2 | 0.7793 | 0.66822 | 16867 | |
| Tagap1 | 0.7793 | 0.8004 | 16868 | |
| Vti1a | 0.77932 | 0.84877 | 16869 | |
| Fiz1 | 0.77935 | 0.56955 | 16870 | |
| Faim3 | 0.77937 | -0.22094 | 16871 | |
| Morc2b | 0.77939 | 0.56138 | 16872 | |
| Acad10 | 0.77945 | 1.2419 | 16873 | |
| Zfp106 | 0.77952 | 0.0243 | 16874 | |
| Olfr943 | 0.77955 | -0.10641 | 16875 | |
| Tnfsf10 | 0.77957 | 0.28898 | 16876 | |
| Mef2a | 0.77964 | 0.33782 | 16877 | |
| Atrnl1 | 0.77969 | 0.65417 | 16878 | |
| Tmc2 | 0.7797 | 1.0855 | 16879 | |
| Vwa3a | 0.77992 | -0.40983 | 16880 | |
| Gcnt1 | 0.78007 | 0.92536 | 16881 | |
| Ccdc87 | 0.78015 | 0.086462 | 16882 | |
| Dhx58 | 0.78018 | 0.059613 | 16883 | |
| Insl3 | 0.78023 | -0.33067 | 16884 | |
| Exoc3l | 0.78033 | 0.42139 | 16885 | |
| Edc4 | 0.78035 | 0.1671 | 16886 | |
| Clk3 | 0.78065 | 2.2384 | 16887 | |
| Gm1140 | 0.7807 | 0.51566 | 16888 | |
| Pdgfb | 0.78073 | 0.10022 | 16889 | |
| Tmem106c | 0.78075 | 0.49176 | 16890 | |
| Mup11 | 0.78075 | 0.58562 | 16891 | |
| Dstn | 0.78078 | -0.35882 | 16892 | |
| Farp1 | 0.78085 | -0.11839 | 16893 | |
| Ttll4 | 0.78101 | 0.86788 | 16894 | |
| Tmtc4 | 0.78103 | 1.3641 | 16895 | |
| Chmp2b | 0.78105 | 0.93326 | 16896 | |
| Rhod | 0.78118 | 1.5387 | 16897 | |
| Prpsap1 | 0.78119 | -0.40467 | 16898 | |
| Pcbd1 | 0.78123 | 0.7477 | 16899 | |
| Alas2 | 0.78127 | 1.3682 | 16900 | |
| Folh1 | 0.78129 | -0.15692 | 16901 | |
| Orm2 | 0.78138 | -0.030228 | 16902 | |
| Kcna5 | 0.78146 | 0.031858 | 16903 | |
| Brd2 | 0.78149 | -0.049354 | 16904 | |
| Srsf11 | 0.78151 | -0.15618 | 16905 | |
| Obsl1 | 0.78164 | 1.2546 | 16906 | |
| AI317395 | 0.78171 | 0.63259 | 16907 | |
| Herpud1 | 0.78174 | 0.2828 | 16908 | |
| Kcnd2 | 0.78179 | -0.40661 | 16909 | |
| Clec3b | 0.78184 | 0.3634 | 16910 | |
| Figla | 0.78187 | 0.67854 | 16911 | |
| Cbfb | 0.7819 | 0.85199 | 16912 | |
| Syne4 | 0.78197 | 0.65826 | 16913 | |
| Mrps18c | 0.78205 | 0.8877 | 16914 | |
| Ctsz | 0.78209 | -0.40568 | 16915 | |
| Rcn3 | 0.7821 | 0.3394 | 16916 | |
| Akr1c14 | 0.78225 | -0.0429 | 16917 | |
| Sh2d4b | 0.78233 | 0.16401 | 16918 | |
| Gpc4 | 0.78237 | 0.67919 | 16919 | |
| Accs | 0.78248 | -0.15308 | 16920 | |
| Prss33 | 0.78255 | 1.7878 | 16921 | |
| Egln3 | 0.78256 | 0.45644 | 16922 | |
| Gsap | 0.78261 | -0.040945 | 16923 | |
| Chst9 | 0.78265 | 0.7809 | 16924 | |
| Tnfsf18 | 0.78272 | 0.1799 | 16925 | |
| Zfp40 | 0.78276 | 0.094842 | 16926 | |
| Clrn1 | 0.78289 | 0.33034 | 16927 | |
| Cdh20 | 0.78292 | 0.84623 | 16928 | |
| E2f5 | 0.78297 | 0.51683 | 16929 | |
| Cdca7 | 0.78302 | -0.40506 | 16930 | |
| Pip4k2c | 0.78311 | 0.08108 | 16931 | |
| Ube2v1 | 0.78312 | -0.40653 | 16932 | |
| Foxred2 | 0.78314 | -0.082097 | 16933 | |
| Ranbp10 | 0.7832 | 0.22678 | 16934 | |
| Rere | 0.78323 | 0.014271 | 16935 | |
| Scd1 | 0.78335 | 0.51083 | 16936 | |
| Zfpl1 | 0.78338 | 0.36541 | 16937 | |
| Gm6289 | 0.78352 | 0.092542 | 16938 | |
| Lrtm2 | 0.7836 | 0.059097 | 16939 | |
| Acsl5 | 0.78366 | -0.29347 | 16940 | |
| Spry3 | 0.78383 | 0.66287 | 16941 | |
| 1700009N14Rik | 0.78384 | 0.59263 | 16942 | |
| Olfr140 | 0.78388 | 0.08641 | 16943 | |
| 2310067B10Rik | 0.78389 | -0.30577 | 16944 | |
| Kpna3 | 0.78391 | 0.53162 | 16945 | |
| Kdm1b | 0.78416 | 0.99089 | 16946 | |
| Olfr605 | 0.78417 | 0.63523 | 16947 | |
| Fam162b | 0.78419 | 0.42405 | 16948 | |
| Sh2d7 | 0.78424 | 0.88664 | 16949 | |
| Mmgt1 | 0.78425 | -0.056526 | 16950 | |
| Ly6a | 0.78427 | 0.71219 | 16951 | |
| Myo7a | 0.78432 | 1.8482 | 16952 | |
| mmu-mir-1982 | 0.78435 | 1.6509 | 16953 | |
| mmu-mir-1668 | 0.78439 | 0.16069 | 16954 | |
| Cyp46a1 | 0.78447 | 0.51717 | 16955 | |
| Inip | 0.78453 | 0.34579 | 16956 | |
| Ndrg3 | 0.78456 | -0.048311 | 16957 | |
| Mdfi | 0.78466 | 0.035531 | 16958 | |
| Casp3 | 0.78471 | -0.13111 | 16959 | |
| Homer3 | 0.78474 | 0.76599 | 16960 | |
| Gspt2 | 0.78476 | -0.4217 | 16961 | |
| Ptdss1 | 0.78479 | 0.29799 | 16962 | |
| Pla2g2a | 0.78483 | 0.64065 | 16963 | |
| Tmem238 | 0.78514 | -0.2081 | 16964 | |
| Macc1 | 0.78525 | 0.47529 | 16965 | |
| Slc6a20a | 0.7853 | 0.69425 | 16966 | |
| 3830406C13Rik | 0.78532 | -0.32529 | 16967 | |
| Kirrel3 | 0.78533 | -0.19183 | 16968 | |
| Zfp715 | 0.78535 | 0.61184 | 16969 | |
| Fip1l1 | 0.78538 | 1.4227 | 16970 | |
| Runx2 | 0.78548 | -0.34593 | 16971 | |
| Cd180 | 0.78551 | 0.25774 | 16972 | |
| Olfr1023 | 0.78565 | -0.025873 | 16973 | |
| Gm11565 | 0.78568 | 0.8121 | 16974 | |
| Ptchd4 | 0.78569 | 0.96874 | 16975 | |
| Defa17 | 0.78574 | 0.54183 | 16976 | |
| Olfr773 | 0.7859 | 0.24717 | 16977 | |
| Calhm2 | 0.78605 | 0.45498 | 16978 | |
| Zfp458 | 0.78623 | 1.1537 | 16979 | |
| Cep128 | 0.78639 | 0.15787 | 16980 | |
| Ubd | 0.78641 | -0.049019 | 16981 | |
| Nek5 | 0.78648 | 0.80702 | 16982 | |
| Cuzd1 | 0.7866 | -0.378 | 16983 | |
| Piwil1 | 0.78668 | -0.26868 | 16984 | |
| Kcng2 | 0.78672 | 1.1722 | 16985 | |
| Apba2 | 0.78703 | 0.41221 | 16986 | |
| Gpr31b | 0.78706 | 0.16098 | 16987 | |
| Mup20 | 0.78709 | -0.27754 | 16988 | |
| Pofut2 | 0.78714 | 1.291 | 16989 | |
| Neu3 | 0.78717 | 0.18789 | 16990 | |
| Defb48 | 0.78728 | 0.30269 | 16991 | |
| Rbak | 0.78743 | -0.38007 | 16992 | |
| Wfdc16 | 0.78748 | 0.85938 | 16993 | |
| Evpl | 0.78754 | -0.0069831 | 16994 | |
| mmu-mir-32 | 0.78756 | 0.7027 | 16995 | |
| Sowahb | 0.78764 | -0.10542 | 16996 | |
| Sgta | 0.78766 | 1.9665 | 16997 | |
| Gm5071 | 0.78769 | -0.31198 | 16998 | |
| Opcml | 0.78772 | -0.38365 | 16999 | |
| Pcdha2 | 0.78774 | 0.17041 | 17000 | |
| Ptpn9 | 0.78778 | 1.0313 | 17001 | |
| Tceal8 | 0.78782 | 0.42812 | 17002 | |
| Cry2 | 0.78785 | 0.1345 | 17003 | |
| Snx3 | 0.78788 | -0.16192 | 17004 | |
| Ptprr | 0.78805 | 0.075969 | 17005 | |
| Alpl | 0.78813 | 0.72894 | 17006 | |
| Fgf5 | 0.78843 | 0.11116 | 17007 | |
| Il4ra | 0.78852 | -0.20807 | 17008 | |
| Lrfn3 | 0.78861 | 0.16357 | 17009 | |
| Slc25a54 | 0.78871 | -0.25389 | 17010 | |
| Slc12a2 | 0.78874 | 0.10647 | 17011 | |
| Tmem33 | 0.78879 | -0.34306 | 17012 | |
| Prodh2 | 0.78884 | 0.22188 | 17013 | |
| Helt | 0.78889 | -0.28121 | 17014 | |
| Mest | 0.78889 | 0.56139 | 17015 | |
| AF067063 | 0.789 | 0.19524 | 17016 | |
| Tmem243 | 0.78911 | -0.34509 | 17017 | |
| Gpr125 | 0.78916 | 0.19766 | 17018 | |
| Malsu1 | 0.78923 | 0.32876 | 17019 | |
| Eif5a | 0.78927 | -0.33848 | 17020 | |
| Hspa1l | 0.7893 | 0.18056 | 17021 | |
| mmu-mir-710 | 0.78934 | 0.38011 | 17022 | |
| Prr11 | 0.78946 | 1.014 | 17023 | |
| mmu-mir-3074-1 | 0.78949 | -0.17472 | 17024 | |
| Cacng1 | 0.78955 | 1.624 | 17025 | |
| Shroom2 | 0.78956 | 0.56139 | 17026 | |
| Epc1 | 0.78987 | 0.2576 | 17027 | |
| Ehd3 | 0.78989 | -0.051956 | 17028 | |
| Aspm | 0.78993 | 0.45319 | 17029 | |
| Zfp36l3 | 0.78997 | 0.63834 | 17030 | |
| Cd101 | 0.79001 | 0.62982 | 17031 | |
| Mcm9 | 0.79005 | 0.25767 | 17032 | |
| Wwp2 | 0.79006 | 0.42986 | 17033 | |
| Rps27l | 0.79012 | 0.12891 | 17034 | |
| Cd209e | 0.79016 | 0.73698 | 17035 | |
| Pcgf5 | 0.79018 | 0.77341 | 17036 | |
| Anxa5 | 0.79027 | -0.20795 | 17037 | |
| Capn8 | 0.79034 | 0.086239 | 17038 | |
| mmu-mir-185 | 0.79034 | 0.42174 | 17039 | |
| Svs6 | 0.79038 | -0.13365 | 17040 | |
| Rims3 | 0.79042 | 0.084915 | 17041 | |
| mmu-let-7g | 0.79043 | 1.0364 | 17042 | |
| Cep192 | 0.79046 | 0.23194 | 17043 | |
| Ndufs4 | 0.79058 | 0.29102 | 17044 | |
| Olfr1020 | 0.79066 | -0.25009 | 17045 | |
| Ednra | 0.7908 | 0.12944 | 17046 | |
| Nfkbid | 0.79088 | 0.23943 | 17047 | |
| Cyp4a12a | 0.79099 | -0.33563 | 17048 | |
| Nlrp4b | 0.79101 | 0.83258 | 17049 | |
| Nell2 | 0.79104 | -0.18965 | 17050 | |
| Tm6sf1 | 0.79114 | -0.13093 | 17051 | |
| Bsph2 | 0.7913 | -0.3935 | 17052 | |
| Tsx | 0.79135 | -0.042125 | 17053 | |
| Zfp3 | 0.79151 | 0.87914 | 17054 | |
| Pln | 0.79165 | 0.38573 | 17055 | |
| Psmd10 | 0.79174 | 0.80588 | 17056 | |
| Hspb3 | 0.79176 | -0.29268 | 17057 | |
| Sh3yl1 | 0.79187 | 0.016558 | 17058 | |
| Vamp5 | 0.79195 | 1.4299 | 17059 | |
| Snx25 | 0.79199 | 0.27767 | 17060 | |
| Timm10b | 0.79213 | -0.079522 | 17061 | |
| Ankrd63 | 0.79226 | 0.014453 | 17062 | |
| Cebpg | 0.79229 | 0.5337 | 17063 | |
| Nkain2 | 0.79234 | -0.1337 | 17064 | |
| Ccdc89 | 0.79243 | 0.98595 | 17065 | |
| Hp | 0.79255 | 0.03782 | 17066 | |
| Mmaa | 0.79258 | -0.089998 | 17067 | |
| Gm16515 | 0.7927 | -0.13441 | 17068 | |
| Zkscan5 | 0.79274 | -0.40056 | 17069 | |
| Sfxn5 | 0.79277 | 0.25603 | 17070 | |
| Jazf1 | 0.79288 | 1.1967 | 17071 | |
| Irs1 | 0.79296 | 0.042081 | 17072 | |
| Gm590 | 0.79302 | 0.68757 | 17073 | |
| mmu-mir-106b | 0.79309 | 0.99765 | 17074 | |
| Acvr1b | 0.79313 | -0.16552 | 17075 | |
| Sohlh1 | 0.79317 | 0.51506 | 17076 | |
| Atn1 | 0.79321 | 0.51984 | 17077 | |
| Ccl11 | 0.79321 | 1.2444 | 17078 | |
| Tgtp2 | 0.79321 | -0.19592 | 17079 | |
| Helz | 0.79323 | 0.02655 | 17080 | |
| Treml1 | 0.79326 | 0.71225 | 17081 | |
| Lyg1 | 0.79329 | 0.12867 | 17082 | |
| Spp1 | 0.79331 | -0.39451 | 17083 | |
| Tmem151b | 0.79337 | -0.15487 | 17084 | |
| Olfr266 | 0.79347 | 0.93845 | 17085 | |
| Arnt | 0.79358 | -0.28901 | 17086 | |
| Hpcal4 | 0.79361 | 1.2338 | 17087 | |
| Srebf1 | 0.79369 | 0.057996 | 17088 | |
| Cbln3 | 0.79371 | -0.14937 | 17089 | |
| Tgm2 | 0.79372 | 0.59705 | 17090 | |
| H2-Q2 | 0.79375 | 0.34094 | 17091 | |
| Ndufs3 | 0.79379 | 1.6086 | 17092 | |
| Ankrd1 | 0.79383 | 0.31958 | 17093 | |
| Tnf | 0.79387 | 0.2921 | 17094 | |
| Dvl2 | 0.79389 | 0.89751 | 17095 | |
| Tifa | 0.79393 | -0.34769 | 17096 | |
| Sec22c | 0.7941 | 0.92529 | 17097 | |
| Gsto2 | 0.79412 | -0.0079081 | 17098 | |
| Tlcd1 | 0.79418 | -0.38588 | 17099 | |
| Gm5082 | 0.79422 | 0.67656 | 17100 | |
| Fsbp | 0.79428 | 0.42873 | 17101 | |
| 9430069I07Rik | 0.79439 | 0.065543 | 17102 | |
| Pum1 | 0.79442 | 0.9329 | 17103 | |
| Phox2a | 0.79453 | 0.54093 | 17104 | |
| A630001G21Rik | 0.79459 | 0.30539 | 17105 | |
| Mpped1 | 0.79461 | 0.3726 | 17106 | |
| Anxa4 | 0.79463 | -0.26427 | 17107 | |
| Nudt12 | 0.79467 | 1.1134 | 17108 | |
| Col1a1 | 0.79475 | -0.40093 | 17109 | |
| E2f3 | 0.79486 | -0.0071875 | 17110 | |
| Sec24a | 0.79499 | -0.32235 | 17111 | |
| Gm8909 | 0.79501 | 0.94413 | 17112 | |
| Krtap5-2 | 0.79509 | -0.33954 | 17113 | |
| Cby1 | 0.79513 | -0.22471 | 17114 | |
| Cdk18 | 0.79517 | -0.14969 | 17115 | |
| mmu-mir-337 | 0.7953 | 0.21867 | 17116 | |
| Mab21l2 | 0.79545 | -0.16252 | 17117 | |
| Pydc3 | 0.79547 | -0.058021 | 17118 | |
| Atg14 | 0.79548 | 0.12147 | 17119 | |
| Onecut2 | 0.79551 | -0.048852 | 17120 | |
| Nell1 | 0.79553 | 1.2634 | 17121 | |
| Olfr533 | 0.79569 | -0.41606 | 17122 | |
| Cdc14a | 0.7957 | 0.65149 | 17123 | |
| Tnfrsf4 | 0.79583 | -0.28992 | 17124 | |
| Tmem42 | 0.79589 | -0.12952 | 17125 | |
| Atp13a4 | 0.79599 | 0.16945 | 17126 | |
| Slc48a1 | 0.796 | -0.40706 | 17127 | |
| Pcgf6 | 0.79622 | 1.0153 | 17128 | |
| Rab5b | 0.79627 | 0.70563 | 17129 | |
| Cd200 | 0.79641 | 0.38426 | 17130 | |
| Dtnbp1 | 0.79643 | 0.13218 | 17131 | |
| Miox | 0.79651 | 0.00069031 | 17132 | |
| Msx2 | 0.79651 | 0.31109 | 17133 | |
| Slc30a2 | 0.79673 | -0.044036 | 17134 | |
| Insl6 | 0.79676 | -0.057132 | 17135 | |
| Pank2 | 0.79679 | 0.82219 | 17136 | |
| Scp2d1 | 0.79687 | 0.56051 | 17137 | |
| Arhgef37 | 0.7969 | -0.04482 | 17138 | |
| Mex3b | 0.79692 | -0.052531 | 17139 | |
| Acvrl1 | 0.79693 | 0.033906 | 17140 | |
| mmu-mir-6375 | 0.79694 | 0.3762 | 17141 | |
| Srrm2 | 0.79698 | -0.096511 | 17142 | |
| Sstr3 | 0.79701 | -0.27759 | 17143 | |
| Mtpn | 0.79709 | 0.43609 | 17144 | |
| Krba1 | 0.79723 | 0.48291 | 17145 | |
| Cenpv | 0.79736 | -0.12781 | 17146 | |
| Vmn2r83 | 0.79741 | -0.40473 | 17147 | |
| Ccnd3 | 0.79747 | -0.22299 | 17148 | |
| Tagln2 | 0.79754 | 0.53372 | 17149 | |
| Shmt2 | 0.7978 | -0.38181 | 17150 | |
| Pcolce2 | 0.79781 | -0.24807 | 17151 | |
| Cldn24 | 0.79784 | -0.3182 | 17152 | |
| Adcy4 | 0.79792 | -0.30837 | 17153 | |
| Tmem213 | 0.79794 | -0.19809 | 17154 | |
| Pcdha10 | 0.79797 | -0.19775 | 17155 | |
| Krtap12-1 | 0.79803 | -0.23939 | 17156 | |
| mmu-mir-21c | 0.79821 | -0.23768 | 17157 | |
| Steap2 | 0.79821 | -0.18105 | 17158 | |
| Iqcc | 0.79836 | 1.0476 | 17159 | |
| Ccdc132 | 0.79839 | 1.169 | 17160 | |
| Krt32 | 0.79847 | 0.497 | 17161 | |
| Zfp91 | 0.7985 | 0.2707 | 17162 | |
| Tes | 0.79858 | 0.15988 | 17163 | |
| Gm4922 | 0.79878 | -0.37981 | 17164 | |
| Etnk1 | 0.79916 | -0.19987 | 17165 | |
| 1600015I10Rik | 0.79917 | 0.43031 | 17166 | |
| Plin3 | 0.79939 | 1.0304 | 17167 | |
| Lce1g | 0.79946 | 0.080411 | 17168 | |
| Ddx52 | 0.79953 | 0.54317 | 17169 | |
| Rab11b | 0.79956 | 0.41196 | 17170 | |
| Atraid | 0.79959 | 0.37225 | 17171 | |
| Chrnb4 | 0.79962 | 2.0109 | 17172 | |
| AI836003 | 0.79964 | 0.47288 | 17173 | |
| Nrip2 | 0.79976 | 1.0903 | 17174 | |
| Tas2r139 | 0.79979 | 0.22434 | 17175 | |
| Polb | 0.79984 | 0.072864 | 17176 | |
| Lce1m | 0.79995 | -0.012912 | 17177 | |
| Olfr1199 | 0.79998 | 0.012118 | 17178 | |
| mmu-mir-7012 | 0.79998 | 0.74961 | 17179 | |
| Synrg | 0.80006 | 1.5203 | 17180 | |
| Naaa | 0.80011 | -0.44009 | 17181 | |
| Gm382 | 0.80012 | 0.0016257 | 17182 | |
| Sdf2 | 0.80017 | 0.31792 | 17183 | |
| Zkscan17 | 0.80025 | 0.41327 | 17184 | |
| Ccdc39 | 0.80028 | 0.053449 | 17185 | |
| Wbscr28 | 0.80036 | -0.15475 | 17186 | |
| Zfp949 | 0.8004 | -0.32568 | 17187 | |
| Gm3264 | 0.80049 | 0.59595 | 17188 | |
| Grk4 | 0.8006 | 0.32573 | 17189 | |
| Olfr356 | 0.80062 | -0.060201 | 17190 | |
| Lta | 0.80064 | 0.10455 | 17191 | |
| Nudt11 | 0.80068 | 1.5916 | 17192 | |
| Khdrbs2 | 0.80078 | 0.89152 | 17193 | |
| Slc5a5 | 0.80085 | 0.14633 | 17194 | |
| Mpv17l2 | 0.80087 | -0.38651 | 17195 | |
| Olfr1129 | 0.8009 | 0.46822 | 17196 | |
| Kcnh3 | 0.80095 | 0.24154 | 17197 | |
| Kctd2 | 0.80107 | 0.13048 | 17198 | |
| Kif18a | 0.80112 | 1.385 | 17199 | |
| Rab15 | 0.80114 | 0.16744 | 17200 | |
| Rbm42 | 0.80118 | -0.34541 | 17201 | |
| Pnp | 0.80119 | -0.22182 | 17202 | |
| Tusc5 | 0.80124 | -0.33522 | 17203 | |
| Gm166 | 0.80152 | 0.92574 | 17204 | |
| Kcnip2 | 0.80158 | -0.18467 | 17205 | |
| Mtrf1l | 0.80161 | -0.4053 | 17206 | |
| Plscr4 | 0.80163 | 0.77214 | 17207 | |
| Cyp4a31 | 0.8018 | 0.65409 | 17208 | |
| Cmya5 | 0.80186 | -0.34356 | 17209 | |
| Bcam | 0.8019 | 2.1547 | 17210 | |
| B3galt5 | 0.80192 | 0.84888 | 17211 | |
| Eif2ak4 | 0.80197 | 0.5789 | 17212 | |
| Dusp14 | 0.802 | 0.084349 | 17213 | |
| Tmub2 | 0.80202 | 0.034419 | 17214 | |
| Gstm7 | 0.80205 | -0.3219 | 17215 | |
| Ceacam18 | 0.80212 | -0.29356 | 17216 | |
| Aar2 | 0.80217 | 0.2339 | 17217 | |
| Olfr1474 | 0.80245 | 0.04172 | 17218 | |
| Nt5dc2 | 0.80246 | 1.0693 | 17219 | |
| Vwc2l | 0.80251 | -0.18887 | 17220 | |
| mmu-mir-7019 | 0.80255 | 0.46001 | 17221 | |
| Tfg | 0.80262 | -0.10841 | 17222 | |
| AU018091 | 0.80263 | 0.21971 | 17223 | |
| Olfr357 | 0.80274 | 0.27231 | 17224 | |
| Degs2 | 0.80278 | 1.7089 | 17225 | |
| St3gal4 | 0.80289 | -0.22689 | 17226 | |
| Cwc15 | 0.80303 | 1.531 | 17227 | |
| Dnajb3 | 0.8031 | 0.43561 | 17228 | |
| Pbp2 | 0.80316 | 0.4896 | 17229 | |
| Fam114a1 | 0.80323 | -0.0095795 | 17230 | |
| Fam207a | 0.80328 | 0.26581 | 17231 | |
| Cbx2 | 0.80331 | 1.0291 | 17232 | |
| Ppfia4 | 0.80333 | -0.38928 | 17233 | |
| Cacna2d1 | 0.80347 | 1.0702 | 17234 | |
| Adam19 | 0.80348 | -0.077518 | 17235 | |
| Olfr827 | 0.80353 | 0.3569 | 17236 | |
| Pygo2 | 0.80357 | 1.3224 | 17237 | |
| Fasl | 0.80363 | 2.7606 | 17238 | |
| C1ql1 | 0.8037 | 0.60908 | 17239 | |
| Krtap3-2 | 0.80376 | 0.69547 | 17240 | |
| Arhgef10 | 0.8038 | 0.074906 | 17241 | |
| Ifna14 | 0.804 | -0.2871 | 17242 | |
| Frg1 | 0.80403 | -0.015714 | 17243 | |
| Hmbox1 | 0.80406 | 1.0077 | 17244 | |
| Ppp2r4 | 0.80408 | -0.36369 | 17245 | |
| Mast3 | 0.8041 | 1.9964 | 17246 | |
| Rtn3 | 0.80433 | -0.047418 | 17247 | |
| Ash1l | 0.80442 | 0.24153 | 17248 | |
| Ankrd33b | 0.80454 | -0.29105 | 17249 | |
| Fhl2 | 0.80456 | 0.87738 | 17250 | |
| Olig2 | 0.80459 | 0.4644 | 17251 | |
| Dcaf12l2 | 0.80469 | 0.27484 | 17252 | |
| Wdr38 | 0.80474 | 0.019405 | 17253 | |
| Myl7 | 0.80482 | 1.1729 | 17254 | |
| Emd | 0.80489 | -0.099535 | 17255 | |
| Rps6ka3 | 0.80495 | 0.15556 | 17256 | |
| mmu-mir-6981 | 0.80498 | 0.23167 | 17257 | |
| Cnksr2 | 0.80498 | 0.37195 | 17258 | |
| Cyp2w1 | 0.80503 | -0.10318 | 17259 | |
| Rsph4a | 0.80509 | -0.28589 | 17260 | |
| Olfr622 | 0.80512 | 1.7306 | 17261 | |
| B3galnt1 | 0.8052 | -0.2109 | 17262 | |
| Olfr1404 | 0.80534 | 0.14813 | 17263 | |
| Cabp1 | 0.80535 | -0.12089 | 17264 | |
| Ss18 | 0.80543 | 1.2305 | 17265 | |
| Gm13040 | 0.80555 | -0.25167 | 17266 | |
| Hydin | 0.80557 | 0.35449 | 17267 | |
| Il24 | 0.80563 | 0.81217 | 17268 | |
| AU041133 | 0.80564 | 0.080851 | 17269 | |
| Trpc2 | 0.80573 | 1.7621 | 17270 | |
| Nhlrc2 | 0.80583 | 0.37897 | 17271 | |
| Kcnj8 | 0.80584 | 1.3799 | 17272 | |
| Kank1 | 0.80588 | 0.46792 | 17273 | |
| Trim71 | 0.8059 | 0.41669 | 17274 | |
| Cnfn | 0.80613 | 0.34196 | 17275 | |
| A330021E22Rik | 0.80618 | 0.17066 | 17276 | |
| Nek11 | 0.80627 | 0.19146 | 17277 | |
| F10 | 0.80634 | 0.3529 | 17278 | |
| Ccdc124 | 0.80639 | 0.049721 | 17279 | |
| Olfr5 | 0.80643 | 0.4221 | 17280 | |
| Cmklr1 | 0.8065 | -0.16449 | 17281 | |
| Ccdc151 | 0.80654 | -0.19191 | 17282 | |
| BC089491 | 0.80656 | 0.055433 | 17283 | |
| Sstr4 | 0.8066 | -0.22508 | 17284 | |
| mmu-mir-1967 | 0.80662 | 0.973 | 17285 | |
| Stk32a | 0.80669 | 1.2681 | 17286 | |
| Gm5868 | 0.80675 | 0.76913 | 17287 | |
| Bend7 | 0.807 | 0.12374 | 17288 | |
| Fbxl21 | 0.80704 | -0.37461 | 17289 | |
| Rab36 | 0.80717 | 0.62276 | 17290 | |
| Fam98a | 0.80718 | -0.37604 | 17291 | |
| Fancd2os | 0.80744 | 0.35683 | 17292 | |
| Lypd8 | 0.8075 | -0.11252 | 17293 | |
| Col3a1 | 0.80756 | 3.2955 | 17294 | |
| mmu-mir-6978 | 0.80758 | 0.40486 | 17295 | |
| Slc16a3 | 0.8076 | -0.096113 | 17296 | |
| Spg7 | 0.80782 | 0.083892 | 17297 | |
| Ano3 | 0.80785 | 0.52174 | 17298 | |
| 1700012L04Rik | 0.80795 | -0.13078 | 17299 | |
| Lace1 | 0.808 | 1.0673 | 17300 | |
| mmu-mir-7059 | 0.80801 | 0.09839 | 17301 | |
| Arhgap18 | 0.80806 | 0.18284 | 17302 | |
| Ctsr | 0.80811 | 0.22226 | 17303 | |
| Aadacl3 | 0.80815 | 0.029951 | 17304 | |
| Tab2 | 0.80821 | 1.9971 | 17305 | |
| Brs3 | 0.80824 | -0.4053 | 17306 | |
| Atxn7l1 | 0.80827 | 0.96584 | 17307 | |
| mmu-mir-127 | 0.80829 | 0.7152 | 17308 | |
| Prss28 | 0.80832 | -0.11906 | 17309 | |
| Gif | 0.80838 | 0.13366 | 17310 | |
| Jakmip3 | 0.80839 | -0.10629 | 17311 | |
| Jakmip2 | 0.80845 | 0.73921 | 17312 | |
| Wfs1 | 0.80859 | -0.28339 | 17313 | |
| Gm13084 | 0.80872 | 1.022 | 17314 | |
| Iba57 | 0.80876 | -0.40771 | 17315 | |
| Xdh | 0.80879 | -0.035455 | 17316 | |
| Ankrd13b | 0.8088 | -0.084821 | 17317 | |
| Chpf | 0.80885 | -0.31594 | 17318 | |
| Npffr1 | 0.80888 | -0.077157 | 17319 | |
| Olfr1298 | 0.80893 | 0.030113 | 17320 | |
| Ccr1l1 | 0.80897 | -0.089709 | 17321 | |
| 4932443I19Rik | 0.80907 | 0.2277 | 17322 | |
| Rasa3 | 0.80909 | 0.97019 | 17323 | |
| Pfkm | 0.8091 | 0.37436 | 17324 | |
| Tmcc1 | 0.80925 | 0.15611 | 17325 | |
| Iqck | 0.80931 | -0.32593 | 17326 | |
| H2-Ea-ps | 0.80939 | 0.6164 | 17327 | |
| Kptn | 0.80953 | 0.29689 | 17328 | |
| Nmnat2 | 0.80965 | 0.23382 | 17329 | |
| Aldh3a2 | 0.8098 | 0.015762 | 17330 | |
| Yif1a | 0.80984 | -0.34018 | 17331 | |
| Coq10a | 0.80992 | 0.55734 | 17332 | |
| Zc3h6 | 0.80996 | 0.30872 | 17333 | |
| Yipf7 | 0.81004 | 0.73528 | 17334 | |
| Tmem121 | 0.81008 | -0.23709 | 17335 | |
| Olfr1366 | 0.8101 | -0.1686 | 17336 | |
| Klra2 | 0.81011 | -0.085689 | 17337 | |
| Mien1 | 0.81037 | 0.040053 | 17338 | |
| Gnl1 | 0.81044 | 1.1311 | 17339 | |
| Slc15a3 | 0.81046 | -0.17017 | 17340 | |
| Ssna1 | 0.81049 | 0.44706 | 17341 | |
| Ptpra | 0.81073 | 0.15722 | 17342 | |
| Rtdr1 | 0.81076 | 0.67106 | 17343 | |
| Tex12 | 0.8108 | -0.14315 | 17344 | |
| Tm7sf3 | 0.81092 | 0.053401 | 17345 | |
| Lsm14b | 0.81113 | 0.79734 | 17346 | |
| Cabp7 | 0.81113 | 0.75352 | 17347 | |
| Fbxo17 | 0.81116 | 0.70278 | 17348 | |
| Slc16a2 | 0.81121 | 0.15721 | 17349 | |
| Tspan11 | 0.81128 | 1.2583 | 17350 | |
| Tspo | 0.81145 | -0.36248 | 17351 | |
| Ube2e2 | 0.81148 | 0.94739 | 17352 | |
| Nckap5 | 0.81149 | 0.60377 | 17353 | |
| Gnb2 | 0.81151 | 0.76786 | 17354 | |
| Ctso | 0.81157 | 0.12499 | 17355 | |
| Marc2 | 0.81164 | 0.0096842 | 17356 | |
| Kpna6 | 0.81166 | 0.56673 | 17357 | |
| Mocs2 | 0.81173 | -0.0096882 | 17358 | |
| Il23r | 0.81178 | 0.30062 | 17359 | |
| Cdh22 | 0.81185 | 0.62458 | 17360 | |
| Olfr1346 | 0.81187 | -0.19682 | 17361 | |
| Gys1 | 0.81193 | 0.88322 | 17362 | |
| Sesn3 | 0.81208 | 1.183 | 17363 | |
| Kidins220 | 0.81228 | 0.79711 | 17364 | |
| Ncf1 | 0.8124 | 0.48376 | 17365 | |
| Mpi | 0.81253 | 0.091986 | 17366 | |
| Mmp15 | 0.81257 | 0.011385 | 17367 | |
| Rbm34 | 0.81258 | 0.9678 | 17368 | |
| Trib2 | 0.81266 | 0.72061 | 17369 | |
| Man1c1 | 0.81274 | -0.10663 | 17370 | |
| Tmod1 | 0.81278 | 0.48825 | 17371 | |
| Fbxo11 | 0.81288 | 0.09188 | 17372 | |
| Cylc1 | 0.8129 | 1.3642 | 17373 | |
| Lins | 0.8131 | -0.1508 | 17374 | |
| Gm13271 | 0.81316 | -0.014525 | 17375 | |
| Gm5741 | 0.81327 | 0.4735 | 17376 | |
| Lrrn4 | 0.81334 | 0.7 | 17377 | |
| Alpk1 | 0.81337 | 1.6294 | 17378 | |
| 1700019O17Rik | 0.81345 | -0.0098296 | 17379 | |
| Tbpl2 | 0.81346 | 1.4069 | 17380 | |
| Otud4 | 0.81354 | 0.067099 | 17381 | |
| Cherp | 0.81355 | 0.18975 | 17382 | |
| Ctnna3 | 0.81359 | -0.091448 | 17383 | |
| Prl4a1 | 0.81364 | 0.55084 | 17384 | |
| Hspa1b | 0.81373 | 0.29741 | 17385 | |
| Slc24a3 | 0.81379 | -0.34348 | 17386 | |
| Areg | 0.8139 | 0.95459 | 17387 | |
| Rabac1 | 0.8141 | 0.30156 | 17388 | |
| Plp1 | 0.81413 | -0.030477 | 17389 | |
| Ccdc70 | 0.81417 | 1.5681 | 17390 | |
| Bola1 | 0.81419 | 0.12166 | 17391 | |
| Magel2 | 0.81429 | 0.087594 | 17392 | |
| Mrpl40 | 0.81431 | 0.55335 | 17393 | |
| Cartpt | 0.81439 | -0.045865 | 17394 | |
| Utf1 | 0.81443 | -0.3934 | 17395 | |
| Sod3 | 0.81444 | -0.097323 | 17396 | |
| Pfkp | 0.81451 | 0.61596 | 17397 | |
| Sacs | 0.81463 | 1.8039 | 17398 | |
| Dolpp1 | 0.81468 | 0.3479 | 17399 | |
| mmu-mir-6367 | 0.81474 | 0.14507 | 17400 | |
| Ppp2r5d | 0.81477 | 0.68552 | 17401 | |
| Isoc1 | 0.81478 | 0.22221 | 17402 | |
| Auh | 0.81481 | 0.71043 | 17403 | |
| Gm7849 | 0.81484 | 1.1211 | 17404 | |
| Ankrd16 | 0.81488 | 0.79404 | 17405 | |
| Mfsd10 | 0.81497 | 0.43766 | 17406 | |
| Ip6k3 | 0.81499 | 0.11324 | 17407 | |
| Gm884 | 0.815 | 0.10973 | 17408 | |
| Slc6a3 | 0.81505 | 0.49068 | 17409 | |
| Fgl2 | 0.81506 | -0.002707 | 17410 | |
| Cdkl3 | 0.81511 | 1.0615 | 17411 | |
| Kctd20 | 0.81518 | -0.35326 | 17412 | |
| Prkag2 | 0.81519 | 0.1957 | 17413 | |
| Olfr304 | 0.81527 | 0.46442 | 17414 | |
| Arhgef16 | 0.8153 | 0.45616 | 17415 | |
| 5730507C01Rik | 0.81531 | 0.40763 | 17416 | |
| Mettl11b | 0.81534 | 0.23139 | 17417 | |
| Rltpr | 0.81537 | 0.98056 | 17418 | |
| Osm | 0.81555 | 0.2125 | 17419 | |
| Dzip1 | 0.81564 | 0.011759 | 17420 | |
| Rusc1 | 0.81575 | 1.1243 | 17421 | |
| 1600014C10Rik | 0.81584 | 1.3545 | 17422 | |
| Ywhae | 0.81599 | -0.21047 | 17423 | |
| Fgf22 | 0.81603 | 0.7052 | 17424 | |
| Foxi2 | 0.81605 | -0.29512 | 17425 | |
| Asic4 | 0.81606 | 0.18621 | 17426 | |
| Gm4477 | 0.81615 | 0.080104 | 17427 | |
| Mccc1 | 0.81618 | 1.8621 | 17428 | |
| Hepacam2 | 0.81624 | 1.3245 | 17429 | |
| Mef2c | 0.81626 | 0.0060087 | 17430 | |
| Psen1 | 0.81628 | 0.093276 | 17431 | |
| Rab3a | 0.8163 | 0.15297 | 17432 | |
| Smarcal1 | 0.8164 | 0.33749 | 17433 | |
| Habp2 | 0.81662 | -0.27342 | 17434 | |
| Tacr3 | 0.81668 | -0.41446 | 17435 | |
| Cecr6 | 0.8167 | 0.98311 | 17436 | |
| Srsf3 | 0.81677 | 0.026836 | 17437 | |
| Kctd3 | 0.8168 | 0.48945 | 17438 | |
| N6amt2 | 0.81695 | -0.046642 | 17439 | |
| Arhgef6 | 0.81698 | 1.138 | 17440 | |
| Hist1h4c | 0.81699 | -0.072065 | 17441 | |
| Psmd9 | 0.81708 | 0.092194 | 17442 | |
| Sorcs3 | 0.81709 | 1.1785 | 17443 | |
| Arfgap2 | 0.81711 | -0.2977 | 17444 | |
| A430107P09Rik | 0.81713 | 0.24734 | 17445 | |
| Inpp1 | 0.81715 | -0.3873 | 17446 | |
| Sult1c1 | 0.81717 | 0.79187 | 17447 | |
| Hcfc1r1 | 0.81729 | 0.4807 | 17448 | |
| Rap2b | 0.81734 | 0.42896 | 17449 | |
| Gm4846 | 0.81742 | -0.0040432 | 17450 | |
| Triqk | 0.81769 | -0.090029 | 17451 | |
| Hltf | 0.81772 | 0.19845 | 17452 | |
| Arg1 | 0.81778 | -0.35459 | 17453 | |
| mmu-mir-3078 | 0.81787 | 1.104 | 17454 | |
| Npas1 | 0.81788 | 0.20752 | 17455 | |
| Olfr1428 | 0.81799 | -0.23089 | 17456 | |
| AW551984 | 0.81806 | 1.1545 | 17457 | |
| Gm9994 | 0.81809 | -0.4342 | 17458 | |
| Zmat4 | 0.81811 | -0.11426 | 17459 | |
| Rfx8 | 0.81813 | 1.2484 | 17460 | |
| Lrwd1 | 0.81816 | -0.33366 | 17461 | |
| Gm6927 | 0.81829 | -0.013393 | 17462 | |
| Cenpf | 0.81832 | -0.25326 | 17463 | |
| Il17f | 0.81841 | 0.065276 | 17464 | |
| Pck2 | 0.81851 | 0.016226 | 17465 | |
| 9830001H06Rik | 0.81857 | 0.50151 | 17466 | |
| Ccdc81 | 0.81858 | 0.47863 | 17467 | |
| P2ry4 | 0.81862 | -0.32447 | 17468 | |
| Cilp2 | 0.81867 | 0.14464 | 17469 | |
| Hoxa7 | 0.81869 | 0.39597 | 17470 | |
| Cox7c | 0.81872 | -0.37421 | 17471 | |
| Cdkn2aip | 0.81879 | -0.22422 | 17472 | |
| Ss18l1 | 0.81889 | 0.48552 | 17473 | |
| Rdh7 | 0.81893 | 0.87076 | 17474 | |
| Vapb | 0.81895 | 0.60893 | 17475 | |
| Plaa | 0.81904 | -0.062823 | 17476 | |
| Olfr898 | 0.81906 | -0.035836 | 17477 | |
| Wibg | 0.81908 | -0.034577 | 17478 | |
| Mgam | 0.81909 | 0.19718 | 17479 | |
| Prss53 | 0.81911 | 0.076711 | 17480 | |
| Rxfp4 | 0.81912 | 0.56172 | 17481 | |
| Ggact | 0.81917 | -0.31168 | 17482 | |
| Iqcg | 0.81918 | 0.88012 | 17483 | |
| Uroc1 | 0.81933 | 0.23468 | 17484 | |
| 7420426K07Rik | 0.81934 | -0.41239 | 17485 | |
| Bcl6 | 0.81939 | 1.7904 | 17486 | |
| Kcnh8 | 0.81947 | -0.21958 | 17487 | |
| Cxcr5 | 0.81957 | 1.9449 | 17488 | |
| Pvr | 0.81995 | -0.23453 | 17489 | |
| Ephx3 | 0.82008 | 0.0022659 | 17490 | |
| Rhoq | 0.82017 | 2.2855 | 17491 | |
| Med27 | 0.82021 | -0.32136 | 17492 | |
| Prune2 | 0.82024 | -0.25604 | 17493 | |
| Aard | 0.82029 | 0.13685 | 17494 | |
| Fbxl17 | 0.82037 | 0.097999 | 17495 | |
| Cyp2c37 | 0.8204 | -0.13615 | 17496 | |
| Ang | 0.82046 | -0.28691 | 17497 | |
| Olfr1359 | 0.82052 | 0.078793 | 17498 | |
| Lrrc8c | 0.82065 | 2.0513 | 17499 | |
| Mpp4 | 0.82068 | 0.74387 | 17500 | |
| Cep250 | 0.82078 | -0.27763 | 17501 | |
| Gzf1 | 0.82081 | 0.76704 | 17502 | |
| C030006K11Rik | 0.82091 | -0.2048 | 17503 | |
| Clic6 | 0.82094 | -0.38569 | 17504 | |
| Megf8 | 0.82097 | 0.74397 | 17505 | |
| Gm13178 | 0.82102 | -0.13965 | 17506 | |
| Adss | 0.82108 | -0.16111 | 17507 | |
| Pax6 | 0.82115 | 1.63 | 17508 | |
| Gm10439 | 0.82123 | -0.19699 | 17509 | |
| Vmn1r82 | 0.82127 | 1.59 | 17510 | |
| Ttyh3 | 0.82129 | 0.24993 | 17511 | |
| Micall2 | 0.82134 | -0.36348 | 17512 | |
| Hacl1 | 0.82136 | -0.088764 | 17513 | |
| Trip6 | 0.82142 | 0.11677 | 17514 | |
| E130309F12Rik | 0.82142 | -0.25917 | 17515 | |
| Zfp263 | 0.82146 | 2.4644 | 17516 | |
| Olfr934 | 0.8215 | 0.19062 | 17517 | |
| Cyp11a1 | 0.82154 | -0.20792 | 17518 | |
| Gsc | 0.82157 | 0.96813 | 17519 | |
| Snx22 | 0.82163 | -0.38167 | 17520 | |
| Mycn | 0.82177 | -0.38873 | 17521 | |
| Ly6g6f | 0.8218 | 1.909 | 17522 | |
| Hcn1 | 0.82182 | 0.15266 | 17523 | |
| Shox2 | 0.82185 | 0.5049 | 17524 | |
| Defa4 | 0.8219 | 0.32082 | 17525 | |
| Zfp362 | 0.82194 | -0.30867 | 17526 | |
| mmu-mir-466k | 0.82201 | 0.28527 | 17527 | |
| Cct8l1 | 0.82202 | 0.79334 | 17528 | |
| Myo6 | 0.82206 | 0.25105 | 17529 | |
| Map4k3 | 0.82212 | 0.1153 | 17530 | |
| Clip1 | 0.82215 | -0.2896 | 17531 | |
| Nutm1 | 0.82221 | 0.48994 | 17532 | |
| Vmn1r157 | 0.82226 | 0.48591 | 17533 | |
| Nxt1 | 0.82239 | 0.83628 | 17534 | |
| Xirp1 | 0.82251 | 0.34668 | 17535 | |
| Ceacam19 | 0.82259 | 0.7284 | 17536 | |
| Vmn1r196 | 0.82268 | 0.44283 | 17537 | |
| Gpr162 | 0.8227 | 1.9943 | 17538 | |
| Capza3 | 0.82274 | -0.41245 | 17539 | |
| Olfr354 | 0.82278 | -0.21119 | 17540 | |
| Tmem200a | 0.82281 | 0.81402 | 17541 | |
| Smpd2 | 0.82283 | 0.059244 | 17542 | |
| Islr | 0.82285 | 0.24225 | 17543 | |
| Glce | 0.82288 | -0.12757 | 17544 | |
| Krt82 | 0.82295 | -0.19827 | 17545 | |
| Zfp788 | 0.82298 | 0.89444 | 17546 | |
| Kcnk13 | 0.823 | 0.95707 | 17547 | |
| M6pr | 0.82304 | 0.25558 | 17548 | |
| Pip4k2a | 0.82306 | -0.33274 | 17549 | |
| mmu-let-7k | 0.82309 | 1.2229 | 17550 | |
| Zfp454 | 0.8231 | 0.4976 | 17551 | |
| Zcchc2 | 0.82316 | 0.24034 | 17552 | |
| Rcn1 | 0.82321 | 0.46202 | 17553 | |
| Itgb8 | 0.82328 | 0.33642 | 17554 | |
| Acad9 | 0.82333 | 0.16285 | 17555 | |
| Rhbdl2 | 0.8234 | 0.70665 | 17556 | |
| Cxcl5 | 0.82344 | -0.42645 | 17557 | |
| Efcab3 | 0.82346 | -0.15433 | 17558 | |
| Arl5c | 0.82351 | 2.0921 | 17559 | |
| Epsti1 | 0.82357 | 0.34054 | 17560 | |
| Fndc4 | 0.82371 | 1.2761 | 17561 | |
| Tbc1d10b | 0.82381 | -0.11297 | 17562 | |
| Olfr411 | 0.82383 | 0.39986 | 17563 | |
| Krt14 | 0.82384 | -0.39097 | 17564 | |
| Ccnj | 0.82413 | 0.17478 | 17565 | |
| Gypc | 0.82421 | 0.30385 | 17566 | |
| Ets2 | 0.82426 | -0.087882 | 17567 | |
| Olfr353 | 0.82433 | -0.32732 | 17568 | |
| Erbb2 | 0.82441 | 0.33324 | 17569 | |
| Mbd3 | 0.82443 | 0.21751 | 17570 | |
| Doxl2 | 0.82456 | 0.22802 | 17571 | |
| Esp24 | 0.82461 | 0.10909 | 17572 | |
| Fam105b | 0.82467 | -0.060874 | 17573 | |
| Magea6 | 0.82475 | 0.39418 | 17574 | |
| Fert2 | 0.82475 | 0.19965 | 17575 | |
| Dll1 | 0.82477 | -0.32177 | 17576 | |
| Defa20 | 0.82479 | 0.94812 | 17577 | |
| Hapln2 | 0.82487 | 0.28422 | 17578 | |
| Snca | 0.82498 | 0.046026 | 17579 | |
| mmu-mir-742 | 0.82503 | -0.09799 | 17580 | |
| Tomm20l | 0.82504 | -0.42946 | 17581 | |
| Ankrd22 | 0.82508 | 0.46893 | 17582 | |
| Rgs22 | 0.82513 | 0.45715 | 17583 | |
| Tfap2a | 0.82514 | 0.014229 | 17584 | |
| Sprr2d | 0.82517 | 0.61289 | 17585 | |
| Homer1 | 0.82518 | -0.12583 | 17586 | |
| Rnase11 | 0.82525 | -0.1665 | 17587 | |
| Klk10 | 0.82527 | -0.027209 | 17588 | |
| Ide | 0.82528 | 1.3306 | 17589 | |
| Ovol2 | 0.82531 | 0.12793 | 17590 | |
| Tcstv3 | 0.82538 | -0.087432 | 17591 | |
| Zfp944 | 0.8254 | 1.4687 | 17592 | |
| Nkx2-5 | 0.82544 | 0.82225 | 17593 | |
| Lig4 | 0.8255 | 0.23243 | 17594 | |
| Ropn1 | 0.82551 | -0.39793 | 17595 | |
| Evc | 0.82552 | 0.13848 | 17596 | |
| Lypd1 | 0.82561 | 1.0963 | 17597 | |
| Pdgfa | 0.82562 | -0.33377 | 17598 | |
| Fam72a | 0.82579 | -0.36301 | 17599 | |
| Mlh3 | 0.82587 | -0.054962 | 17600 | |
| Tmem163 | 0.82597 | 0.5813 | 17601 | |
| Cd93 | 0.82598 | 0.045614 | 17602 | |
| Kdr | 0.82606 | 0.5658 | 17603 | |
| Olfr835 | 0.82608 | 0.1762 | 17604 | |
| Atp8a1 | 0.82609 | 0.74896 | 17605 | |
| Calr4 | 0.82614 | 0.25485 | 17606 | |
| Gm6484 | 0.82616 | -0.26772 | 17607 | |
| Thop1 | 0.82625 | 0.26315 | 17608 | |
| Epor | 0.82629 | 1.7115 | 17609 | |
| Muc20 | 0.82639 | 0.90252 | 17610 | |
| Tmem180 | 0.82642 | 0.5356 | 17611 | |
| Gltpd1 | 0.82645 | -0.1642 | 17612 | |
| Spats2 | 0.82651 | 0.64987 | 17613 | |
| Med18 | 0.82662 | 0.038702 | 17614 | |
| Pik3c2g | 0.82665 | -0.19494 | 17615 | |
| Sbf2 | 0.82667 | -0.026543 | 17616 | |
| Nup37 | 0.82673 | 0.80523 | 17617 | |
| Rsg1 | 0.82681 | 0.16737 | 17618 | |
| mmu-mir-7231 | 0.82682 | 0.94989 | 17619 | |
| Tgfb3 | 0.82683 | -0.051336 | 17620 | |
| Nfib | 0.82684 | 1.0409 | 17621 | |
| C5ar2 | 0.8269 | 0.15717 | 17622 | |
| Gm867 | 0.82693 | -0.073361 | 17623 | |
| Cnpy1 | 0.82696 | 0.042924 | 17624 | |
| Rsbn1 | 0.82697 | -0.26809 | 17625 | |
| Stc1 | 0.82714 | 0.48617 | 17626 | |
| Oxct2b | 0.82715 | 1.0063 | 17627 | |
| Tpsb2 | 0.82717 | -0.35593 | 17628 | |
| Prr14 | 0.82724 | 0.51626 | 17629 | |
| Zfp369 | 0.82732 | 0.46392 | 17630 | |
| Lbx2 | 0.82738 | 0.48928 | 17631 | |
| Acsf2 | 0.82742 | 0.70514 | 17632 | |
| Eif4b | 0.82748 | -0.086306 | 17633 | |
| Ino80 | 0.82749 | 0.5131 | 17634 | |
| Vegfa | 0.82756 | 1.5074 | 17635 | |
| Hopx | 0.8277 | -0.18069 | 17636 | |
| Ppap2c | 0.82777 | -0.24189 | 17637 | |
| Olfr317 | 0.82779 | 0.73003 | 17638 | |
| Olfr1357 | 0.82782 | 0.22077 | 17639 | |
| Rhox3g | 0.82786 | 0.57861 | 17640 | |
| Ninl | 0.82791 | -0.10533 | 17641 | |
| Pck1 | 0.82794 | 0.14153 | 17642 | |
| Wdsub1 | 0.82804 | 1.5427 | 17643 | |
| Lcn10 | 0.82806 | -0.20843 | 17644 | |
| Apopt1 | 0.82811 | 0.41432 | 17645 | |
| Lnx2 | 0.82815 | -0.37501 | 17646 | |
| Sowahd | 0.8282 | 0.40995 | 17647 | |
| Fastkd1 | 0.82823 | 0.14606 | 17648 | |
| Sept3 | 0.82827 | -0.10493 | 17649 | |
| Thbs2 | 0.82831 | 1.6703 | 17650 | |
| Adamtsl1 | 0.82832 | -0.26183 | 17651 | |
| Tespa1 | 0.82838 | -0.071413 | 17652 | |
| Ifitm6 | 0.82841 | 0.62989 | 17653 | |
| Zcchc16 | 0.82846 | 0.18215 | 17654 | |
| Herc4 | 0.82851 | -0.10519 | 17655 | |
| Fbp1 | 0.82852 | 1.104 | 17656 | |
| Lphn3 | 0.82853 | -0.33976 | 17657 | |
| Tuba3a | 0.82859 | -0.030825 | 17658 | |
| Sh3gl3 | 0.82869 | -0.19411 | 17659 | |
| Cyb561d2 | 0.82871 | -0.25642 | 17660 | |
| Zfp119b | 0.82873 | 0.19537 | 17661 | |
| Olfr1294 | 0.82875 | 0.38906 | 17662 | |
| Zfp146 | 0.82887 | 0.66838 | 17663 | |
| Socs7 | 0.82891 | 0.77828 | 17664 | |
| Dnah7a | 0.82896 | -0.13763 | 17665 | |
| Lelp1 | 0.829 | 0.73039 | 17666 | |
| Cyp27b1 | 0.82913 | 0.38642 | 17667 | |
| mmu-mir-3084-1 | 0.82915 | -0.34199 | 17668 | |
| Casq2 | 0.82917 | -0.022366 | 17669 | |
| Cep89 | 0.8292 | -0.20579 | 17670 | |
| Ccnt1 | 0.82922 | 0.84055 | 17671 | |
| 2410076I21Rik | 0.82924 | -0.19111 | 17672 | |
| Kpna2 | 0.82925 | -0.032081 | 17673 | |
| Sh3tc2 | 0.82929 | 0.22773 | 17674 | |
| R3hdm1 | 0.82931 | 0.55909 | 17675 | |
| Dcpp3 | 0.82942 | -0.13069 | 17676 | |
| H2-Eb1 | 0.8295 | 1.0894 | 17677 | |
| 4932411E22Rik | 0.82953 | 0.67382 | 17678 | |
| Gsk3a | 0.82968 | -0.12478 | 17679 | |
| Asah1 | 0.82977 | -0.22304 | 17680 | |
| Sh2d4a | 0.8298 | -0.35271 | 17681 | |
| Vipr2 | 0.82984 | -0.01698 | 17682 | |
| mmu-mir-19b-2 | 0.82988 | 0.016705 | 17683 | |
| Mslnl | 0.83004 | 0.60267 | 17684 | |
| Cpn2 | 0.83008 | -0.26103 | 17685 | |
| Prpf4b | 0.83016 | 1.0676 | 17686 | |
| Adh5 | 0.83017 | 0.46866 | 17687 | |
| Lpar6 | 0.83019 | 1.1535 | 17688 | |
| Spns3 | 0.83027 | 0.98403 | 17689 | |
| Rab3gap1 | 0.83028 | 0.50968 | 17690 | |
| mmu-mir-451b | 0.83039 | 0.44718 | 17691 | |
| Gypa | 0.8304 | 0.89578 | 17692 | |
| Rfx5 | 0.83042 | 0.26616 | 17693 | |
| 4933402N03Rik | 0.83048 | -0.34555 | 17694 | |
| Olfr541 | 0.83051 | 0.03747 | 17695 | |
| Zfp951 | 0.83055 | 0.57358 | 17696 | |
| Rab35 | 0.83056 | 0.19381 | 17697 | |
| Col27a1 | 0.83058 | 1.0395 | 17698 | |
| Btd | 0.83059 | 1.1262 | 17699 | |
| Gcdh | 0.83062 | 0.24636 | 17700 | |
| Plekhg5 | 0.83063 | 0.11685 | 17701 | |
| Olfr620 | 0.83066 | -0.43871 | 17702 | |
| Olfr1104 | 0.83067 | 0.14397 | 17703 | |
| Ptpn13 | 0.83069 | 2.2614 | 17704 | |
| Cmah | 0.8307 | 1.543 | 17705 | |
| Defb34 | 0.83073 | -0.35955 | 17706 | |
| Unc50 | 0.83076 | 2.1282 | 17707 | |
| Kcnj13 | 0.83087 | -0.0023287 | 17708 | |
| Gpr62 | 0.83088 | -0.13026 | 17709 | |
| Cldn5 | 0.8309 | 0.5039 | 17710 | |
| 2010300C02Rik | 0.83094 | 0.73086 | 17711 | |
| Scgb2b7 | 0.83104 | 0.51569 | 17712 | |
| 0610030E20Rik | 0.83112 | 0.30583 | 17713 | |
| Gucy2d | 0.83113 | -0.18191 | 17714 | |
| Mapkapk5 | 0.83122 | 0.90698 | 17715 | |
| Top2b | 0.83127 | 0.31822 | 17716 | |
| Adal | 0.83133 | 0.079864 | 17717 | |
| Plekho2 | 0.83134 | -0.39862 | 17718 | |
| Pmch | 0.8314 | 0.2729 | 17719 | |
| Aspdh | 0.83147 | -0.1285 | 17720 | |
| Tstd2 | 0.83152 | 0.035911 | 17721 | |
| Rnf207 | 0.83155 | 0.88463 | 17722 | |
| Prl3b1 | 0.83157 | 0.38116 | 17723 | |
| Gm14461 | 0.83161 | 0.37098 | 17724 | |
| Clcnkb | 0.83162 | 0.53601 | 17725 | |
| Rag2 | 0.8317 | 0.635 | 17726 | |
| Tprgl | 0.83179 | 0.25329 | 17727 | |
| Lce1i | 0.8318 | 1.2868 | 17728 | |
| Angpt2 | 0.83181 | 0.17416 | 17729 | |
| Gpr150 | 0.83184 | 0.018735 | 17730 | |
| Galns | 0.83201 | 0.13636 | 17731 | |
| Vmn2r88 | 0.83208 | 0.48416 | 17732 | |
| P2rx3 | 0.83213 | 0.92314 | 17733 | |
| Serinc4 | 0.83215 | 0.36741 | 17734 | |
| Clpx | 0.8322 | 0.0088048 | 17735 | |
| Mtfr1 | 0.83222 | 0.65369 | 17736 | |
| Mapk11 | 0.83236 | -0.070323 | 17737 | |
| Mturn | 0.83238 | 0.38418 | 17738 | |
| Slc25a15 | 0.83251 | 0.66432 | 17739 | |
| Tmem30c | 0.83261 | -0.34225 | 17740 | |
| Tkt | 0.83264 | 0.78726 | 17741 | |
| Med23 | 0.8327 | 2.0343 | 17742 | |
| Gm11567 | 0.83275 | 0.67911 | 17743 | |
| Snx27 | 0.83275 | 0.070006 | 17744 | |
| Cyp4f15 | 0.83283 | 0.0013565 | 17745 | |
| Aspg | 0.83284 | -0.21424 | 17746 | |
| Crygb | 0.83294 | 0.82086 | 17747 | |
| Rhoj | 0.83308 | 0.10461 | 17748 | |
| Ccdc90b | 0.83323 | 0.34615 | 17749 | |
| Sh3bgrl3 | 0.83339 | 0.1532 | 17750 | |
| Mpp7 | 0.83345 | 1.6253 | 17751 | |
| Slc28a1 | 0.83358 | 1.316 | 17752 | |
| Snrnp40 | 0.83369 | 0.56695 | 17753 | |
| Fam171b | 0.83387 | 0.13972 | 17754 | |
| Defb50 | 0.83394 | 0.055613 | 17755 | |
| Aqp1 | 0.834 | 0.91363 | 17756 | |
| Sim1 | 0.83404 | -0.28435 | 17757 | |
| Gm3238 | 0.83408 | 0.70451 | 17758 | |
| Olfr1463 | 0.83409 | -0.31198 | 17759 | |
| 1700015E13Rik | 0.83412 | 0.028883 | 17760 | |
| Tmem150c | 0.83413 | 0.30323 | 17761 | |
| Olfr507 | 0.83415 | 0.59313 | 17762 | |
| Wdr20rt | 0.83419 | -0.17304 | 17763 | |
| Pbdc1 | 0.83434 | 0.19141 | 17764 | |
| Purb | 0.83435 | -0.32441 | 17765 | |
| Herc1 | 0.83438 | 0.11127 | 17766 | |
| Itgam | 0.83443 | 0.91078 | 17767 | |
| L3mbtl3 | 0.83445 | 0.60227 | 17768 | |
| Nr1i2 | 0.83446 | -0.13903 | 17769 | |
| Ifitm1 | 0.83452 | 0.5856 | 17770 | |
| Trim52 | 0.83456 | 0.23155 | 17771 | |
| Lilrb4 | 0.83457 | -0.24155 | 17772 | |
| Pdia5 | 0.83466 | 1.2686 | 17773 | |
| Apeh | 0.83475 | 0.06347 | 17774 | |
| Vmn2r39 | 0.83476 | 0.57271 | 17775 | |
| St3gal2 | 0.83477 | 0.035124 | 17776 | |
| Fmn2 | 0.83486 | 0.015607 | 17777 | |
| Bach2 | 0.83487 | -0.41968 | 17778 | |
| Bod1 | 0.83489 | 0.45781 | 17779 | |
| Scn11a | 0.8349 | 0.29415 | 17780 | |
| Zfp787 | 0.83492 | 0.62894 | 17781 | |
| Smu1 | 0.83495 | -0.56198 | 17782 | |
| Foxo4 | 0.83501 | 0.31667 | 17783 | |
| Wdfy3 | 0.83504 | 0.96102 | 17784 | |
| Mkrn1 | 0.83505 | 1.2827 | 17785 | |
| Fam57b | 0.83507 | 0.24718 | 17786 | |
| Olfr651 | 0.83518 | -0.38344 | 17787 | |
| Tenm4 | 0.8352 | -0.022416 | 17788 | |
| Rbp7 | 0.83522 | -0.27127 | 17789 | |
| Tfap2b | 0.83527 | 0.18124 | 17790 | |
| Crlf2 | 0.83535 | 0.36071 | 17791 | |
| Nkiras2 | 0.83535 | -0.054849 | 17792 | |
| Crygd | 0.83542 | 0.342 | 17793 | |
| Tekt1 | 0.83556 | -0.056166 | 17794 | |
| Lgals3bp | 0.8357 | 1.1753 | 17795 | |
| Zfp68 | 0.83571 | -0.14601 | 17796 | |
| C1qtnf9 | 0.83575 | -0.23402 | 17797 | |
| Vmn1r226 | 0.83576 | 0.64049 | 17798 | |
| Tsc22d3 | 0.83584 | 0.70325 | 17799 | |
| Zfp113 | 0.83586 | 0.78907 | 17800 | |
| AV320801 | 0.83589 | 0.18702 | 17801 | |
| Cep19 | 0.83591 | 0.005153 | 17802 | |
| Tcf7l1 | 0.83595 | 0.67095 | 17803 | |
| Sema3d | 0.83597 | -0.2494 | 17804 | |
| 2310039H08Rik | 0.836 | 0.39735 | 17805 | |
| Bcl2l14 | 0.83601 | 1.1104 | 17806 | |
| Apba3 | 0.83615 | 0.28256 | 17807 | |
| Ms4a3 | 0.83616 | -0.085421 | 17808 | |
| Dok5 | 0.83617 | 0.4053 | 17809 | |
| Stox2 | 0.83623 | 1.321 | 17810 | |
| Olfr419 | 0.83627 | 0.10799 | 17811 | |
| Chm | 0.8363 | -0.081293 | 17812 | |
| Synpo2 | 0.83632 | 1.282 | 17813 | |
| Nhsl2 | 0.83636 | 0.72637 | 17814 | |
| Abhd16b | 0.83639 | 0.15444 | 17815 | |
| B4galnt3 | 0.83655 | 0.45623 | 17816 | |
| Amacr | 0.83657 | 0.17688 | 17817 | |
| Cryl1 | 0.83658 | 0.70691 | 17818 | |
| Ido1 | 0.83661 | 0.020503 | 17819 | |
| Svs3b | 0.83664 | -0.21464 | 17820 | |
| Usp17lc | 0.83669 | 0.078053 | 17821 | |
| Slco1c1 | 0.8367 | 0.034735 | 17822 | |
| Efhd2 | 0.83676 | 1.3013 | 17823 | |
| 5033411D12Rik | 0.83691 | -0.30586 | 17824 | |
| 5031410I06Rik | 0.83693 | 0.87139 | 17825 | |
| Mon1b | 0.83693 | -0.24171 | 17826 | |
| Nipa2 | 0.83695 | -0.10399 | 17827 | |
| Prpf40b | 0.83696 | 0.077856 | 17828 | |
| Pld3 | 0.837 | 0.28843 | 17829 | |
| Lynx1 | 0.83714 | -0.40043 | 17830 | |
| Tbc1d1 | 0.83719 | -0.09564 | 17831 | |
| Pde5a | 0.83725 | -0.12472 | 17832 | |
| Zfp9 | 0.83729 | -0.045517 | 17833 | |
| Lime1 | 0.83731 | 0.083053 | 17834 | |
| Tmem154 | 0.83733 | 0.56819 | 17835 | |
| Gm5460 | 0.83737 | 1.5328 | 17836 | |
| Lrrc47 | 0.83746 | 0.57316 | 17837 | |
| Mmp1b | 0.83757 | 1.5814 | 17838 | |
| Serpina3m | 0.83759 | -0.43973 | 17839 | |
| mmu-mir-1942 | 0.83762 | 0.29062 | 17840 | |
| Serpina11 | 0.83764 | -0.29403 | 17841 | |
| Rnf38 | 0.83765 | -0.042548 | 17842 | |
| Bckdhb | 0.83774 | -0.00024637 | 17843 | |
| 3010026O09Rik | 0.83775 | -0.40339 | 17844 | |
| Trerf1 | 0.83778 | 0.085433 | 17845 | |
| Olfr1410 | 0.83788 | 0.51745 | 17846 | |
| Acaa1a | 0.83791 | 0.86905 | 17847 | |
| 4930447A16Rik | 0.83807 | 1.2789 | 17848 | |
| Triml1 | 0.83813 | 0.62298 | 17849 | |
| Adck5 | 0.83821 | 0.31523 | 17850 | |
| Ezh1 | 0.83822 | -0.33041 | 17851 | |
| Aox2 | 0.83826 | 0.79595 | 17852 | |
| Traf4 | 0.83828 | -0.08636 | 17853 | |
| Tmem88b | 0.83829 | 0.10079 | 17854 | |
| Scai | 0.83836 | -0.19537 | 17855 | |
| Anxa9 | 0.83838 | -0.24273 | 17856 | |
| Acadm | 0.83841 | 0.92306 | 17857 | |
| Fscb | 0.83849 | -0.26918 | 17858 | |
| Spink14 | 0.83853 | 0.66057 | 17859 | |
| Olfr1342 | 0.83856 | 0.42246 | 17860 | |
| Keap1 | 0.8386 | -0.37784 | 17861 | |
| Gpm6b | 0.83861 | 0.61122 | 17862 | |
| Wdr5b | 0.83865 | 0.62556 | 17863 | |
| Egln1 | 0.83869 | 0.60969 | 17864 | |
| Ccdc176 | 0.83876 | 0.27063 | 17865 | |
| Sat1 | 0.83886 | 0.57905 | 17866 | |
| Rab11a | 0.83887 | 0.059259 | 17867 | |
| Lancl2 | 0.8389 | 0.61828 | 17868 | |
| Dync2li1 | 0.83891 | 0.54715 | 17869 | |
| Ankfn1 | 0.83895 | 0.30603 | 17870 | |
| Aldh1b1 | 0.83896 | 0.526 | 17871 | |
| Zfr2 | 0.83899 | 0.033399 | 17872 | |
| Olfr291 | 0.839 | 0.17781 | 17873 | |
| Tmprss11d | 0.83903 | -0.3819 | 17874 | |
| Unc119 | 0.83907 | -0.005323 | 17875 | |
| Gpr119 | 0.83911 | -0.3819 | 17876 | |
| Lsm12 | 0.83923 | 0.28663 | 17877 | |
| 2410015M20Rik | 0.83933 | 0.52867 | 17878 | |
| Sytl3 | 0.83949 | 0.02197 | 17879 | |
| Olfr714 | 0.83954 | -0.089841 | 17880 | |
| Rasgef1c | 0.83956 | 1.0518 | 17881 | |
| Nfkbiz | 0.83965 | 0.19803 | 17882 | |
| Cadm3 | 0.83969 | 2.7102 | 17883 | |
| 1700006E09Rik | 0.8397 | 0.60797 | 17884 | |
| Lepr | 0.83972 | 0.037817 | 17885 | |
| Kbtbd4 | 0.83974 | 0.12222 | 17886 | |
| Hrasls5 | 0.8398 | -0.13937 | 17887 | |
| Cldn14 | 0.83981 | 0.72272 | 17888 | |
| Thyn1 | 0.83987 | -0.3969 | 17889 | |
| Col6a6 | 0.83989 | 1.5554 | 17890 | |
| Rab38 | 0.84004 | 1.6287 | 17891 | |
| Stag3 | 0.84012 | 1.105 | 17892 | |
| Id3 | 0.84017 | 1.1599 | 17893 | |
| Mpv17l | 0.84017 | -0.30469 | 17894 | |
| Fam78a | 0.84019 | 0.98266 | 17895 | |
| Ccdc150 | 0.84027 | -0.29795 | 17896 | |
| Dnmt3a | 0.84028 | -0.34139 | 17897 | |
| Lyl1 | 0.84029 | -0.088564 | 17898 | |
| Usf2 | 0.84031 | -0.32331 | 17899 | |
| Wscd1 | 0.84041 | 0.39704 | 17900 | |
| Btbd17 | 0.84046 | -0.26113 | 17901 | |
| Dlgap3 | 0.84054 | -0.0853 | 17902 | |
| Spink10 | 0.84055 | -0.27365 | 17903 | |
| Stom | 0.84067 | 2.8866 | 17904 | |
| Sdha | 0.84068 | 0.64223 | 17905 | |
| Fam163b | 0.84071 | -0.30122 | 17906 | |
| Pacsin3 | 0.84087 | 0.43677 | 17907 | |
| Dennd2d | 0.84092 | 0.59016 | 17908 | |
| Myo1a | 0.8411 | 1.0755 | 17909 | |
| Ldhd | 0.84111 | 0.95164 | 17910 | |
| Rsrc1 | 0.84112 | 1.1038 | 17911 | |
| Dpysl4 | 0.84115 | -0.32033 | 17912 | |
| Fggy | 0.84116 | 0.43132 | 17913 | |
| mmu-mir-200c | 0.84128 | -0.049835 | 17914 | |
| Tspan12 | 0.84135 | 0.52266 | 17915 | |
| Msmb | 0.8415 | 0.92021 | 17916 | |
| Zfp148 | 0.84152 | -0.09104 | 17917 | |
| Cyp2u1 | 0.84163 | 0.48652 | 17918 | |
| 0610009B22Rik | 0.8417 | -0.42481 | 17919 | |
| Vmn1r224 | 0.8418 | 0.32729 | 17920 | |
| Gm13276 | 0.84181 | 0.80053 | 17921 | |
| Tomm6 | 0.84182 | 0.13377 | 17922 | |
| 3110062M04Rik | 0.8419 | -0.094928 | 17923 | |
| Spag4 | 0.84192 | 0.064454 | 17924 | |
| Sox30 | 0.84207 | -0.058682 | 17925 | |
| Prss36 | 0.8421 | 0.21492 | 17926 | |
| Vrtn | 0.84214 | -0.38446 | 17927 | |
| Pigl | 0.84223 | 0.044693 | 17928 | |
| Rgs19 | 0.84224 | 2.0998 | 17929 | |
| Plekhm3 | 0.84226 | -0.2831 | 17930 | |
| Tspan15 | 0.84235 | 0.17581 | 17931 | |
| Sfxn3 | 0.84236 | 0.51514 | 17932 | |
| 1190007I07Rik | 0.8424 | 0.11907 | 17933 | |
| Dkk4 | 0.84246 | 1.5993 | 17934 | |
| Krtap1-3 | 0.84248 | 0.49454 | 17935 | |
| Rnase13 | 0.84253 | 0.40857 | 17936 | |
| Prkci | 0.84255 | -0.055071 | 17937 | |
| Yy2 | 0.84256 | -0.26843 | 17938 | |
| Sucnr1 | 0.84257 | -0.013527 | 17939 | |
| Srrd | 0.84264 | -0.3823 | 17940 | |
| Hnrnpd | 0.84266 | -0.18091 | 17941 | |
| Gucy2e | 0.84269 | 0.016682 | 17942 | |
| Smlr1 | 0.84271 | 0.67838 | 17943 | |
| Rac3 | 0.84272 | 0.011426 | 17944 | |
| Rhot2 | 0.84275 | -0.28868 | 17945 | |
| Elf4 | 0.84276 | 0.061641 | 17946 | |
| Zfp597 | 0.84277 | 0.11815 | 17947 | |
| Pot1a | 0.84279 | 0.058903 | 17948 | |
| Dnaaf2 | 0.84287 | 0.37522 | 17949 | |
| Olfr1302 | 0.84289 | -0.34206 | 17950 | |
| Idi2 | 0.84291 | 0.4932 | 17951 | |
| Gm10767 | 0.84293 | -0.40787 | 17952 | |
| Gm10100 | 0.84295 | -0.15113 | 17953 | |
| Fpr-rs3 | 0.84297 | -0.052396 | 17954 | |
| Olfr250 | 0.8431 | 0.25445 | 17955 | |
| Ncoa1 | 0.8432 | 0.74378 | 17956 | |
| Hddc3 | 0.84329 | 0.89989 | 17957 | |
| BC026585 | 0.8433 | -0.099788 | 17958 | |
| Chka | 0.84337 | -0.27674 | 17959 | |
| Golim4 | 0.84349 | -0.23551 | 17960 | |
| Ywhab | 0.84351 | 0.1622 | 17961 | |
| Nap1l1 | 0.84354 | -0.15306 | 17962 | |
| mmu-mir-1943 | 0.84356 | 0.84898 | 17963 | |
| Irf2bp2 | 0.84357 | -0.40438 | 17964 | |
| Rpap1 | 0.84358 | 0.35707 | 17965 | |
| mmu-mir-16-1 | 0.84359 | -0.39603 | 17966 | |
| Etnppl | 0.84359 | -0.11281 | 17967 | |
| Pabpc1l | 0.84362 | 0.024107 | 17968 | |
| Ushbp1 | 0.84367 | 0.055912 | 17969 | |
| Coq9 | 0.84369 | 0.28062 | 17970 | |
| 1700088E04Rik | 0.84377 | -0.19867 | 17971 | |
| Qrfp | 0.84388 | -0.28739 | 17972 | |
| Hps6 | 0.84395 | 1.3582 | 17973 | |
| Prrg4 | 0.84396 | 0.036994 | 17974 | |
| Basp1 | 0.84398 | 1.5712 | 17975 | |
| Olfr1512 | 0.84399 | -0.39388 | 17976 | |
| Fam192a | 0.84403 | -0.38167 | 17977 | |
| Trappc6a | 0.84404 | -0.047044 | 17978 | |
| Pgm1 | 0.84411 | 0.6156 | 17979 | |
| Ptprh | 0.84415 | 0.70337 | 17980 | |
| Xk | 0.84419 | 0.86475 | 17981 | |
| Cdh5 | 0.84424 | 0.0075461 | 17982 | |
| Odf3b | 0.84432 | 1.1082 | 17983 | |
| Gpr21 | 0.84433 | 0.21609 | 17984 | |
| Plekhh3 | 0.84437 | 0.47178 | 17985 | |
| mmu-mir-6406 | 0.84449 | -0.29797 | 17986 | |
| Col14a1 | 0.84452 | 2.0185 | 17987 | |
| Ifna9 | 0.84453 | -0.38474 | 17988 | |
| Gpihbp1 | 0.84458 | -0.11967 | 17989 | |
| Sez6l2 | 0.84471 | 0.94576 | 17990 | |
| Scgb1b20 | 0.84474 | 0.87312 | 17991 | |
| 4930591A17Rik | 0.84477 | 0.60738 | 17992 | |
| Acsl3 | 0.84478 | 1.4564 | 17993 | |
| 1810009J06Rik | 0.84482 | -0.15162 | 17994 | |
| Reg1 | 0.84483 | 0.48996 | 17995 | |
| 4930562C15Rik | 0.84495 | 0.071182 | 17996 | |
| Vldlr | 0.84498 | 0.73177 | 17997 | |
| Aco1 | 0.84503 | 0.18663 | 17998 | |
| Lsp1 | 0.8451 | -0.3427 | 17999 | |
| Adam3 | 0.84512 | 1.4958 | 18000 | |
| mmu-mir-6897 | 0.84513 | -0.216 | 18001 | |
| Rasd2 | 0.84523 | 0.7584 | 18002 | |
| Notch2 | 0.84524 | -0.23311 | 18003 | |
| Dclk3 | 0.84549 | -0.33796 | 18004 | |
| Sall1 | 0.8455 | 0.87728 | 18005 | |
| Pgbd5 | 0.84552 | 0.87926 | 18006 | |
| 9330182L06Rik | 0.84555 | -0.3935 | 18007 | |
| Prps1 | 0.84563 | 1.223 | 18008 | |
| Dnaaf3 | 0.84583 | -0.12831 | 18009 | |
| Esp38 | 0.84588 | 0.13025 | 18010 | |
| Urah | 0.8459 | 0.69349 | 18011 | |
| Efcab9 | 0.84592 | -0.16556 | 18012 | |
| Vmn1r27 | 0.84593 | -0.040017 | 18013 | |
| Fa2h | 0.84602 | 0.39569 | 18014 | |
| Olfr382 | 0.8462 | 0.63426 | 18015 | |
| Dus3l | 0.84626 | -0.28005 | 18016 | |
| Map4k4 | 0.84631 | 0.96533 | 18017 | |
| Apcs | 0.84633 | -0.17209 | 18018 | |
| Sdc2 | 0.84637 | 0.22107 | 18019 | |
| 2700029M09Rik | 0.84639 | 0.85554 | 18020 | |
| Sh2b3 | 0.84647 | 0.72008 | 18021 | |
| Map1lc3a | 0.84651 | -0.004347 | 18022 | |
| Ada | 0.84664 | 1.3779 | 18023 | |
| mmu-mir-6952 | 0.84664 | 0.13116 | 18024 | |
| 5031414D18Rik | 0.8467 | -0.35785 | 18025 | |
| Lpar5 | 0.84673 | 0.35645 | 18026 | |
| Gm14322 | 0.84675 | 0.78901 | 18027 | |
| Hvcn1 | 0.8468 | -0.23337 | 18028 | |
| Mdc1 | 0.84697 | -0.086757 | 18029 | |
| Ppt2 | 0.84698 | -0.10378 | 18030 | |
| Tmc6 | 0.84707 | 1.2546 | 18031 | |
| Mllt10 | 0.84712 | -0.38446 | 18032 | |
| Galnt3 | 0.84717 | 1.1841 | 18033 | |
| Krt79 | 0.84723 | 0.65574 | 18034 | |
| Mgst1 | 0.84728 | 0.975 | 18035 | |
| Gid4 | 0.84739 | -0.21268 | 18036 | |
| Rtkn | 0.84741 | 0.041254 | 18037 | |
| Gcn1l1 | 0.8475 | 0.91695 | 18038 | |
| Ces2b | 0.84751 | 0.35286 | 18039 | |
| Olfr323 | 0.84755 | -0.033312 | 18040 | |
| Ltbr | 0.84761 | 0.43847 | 18041 | |
| Esm1 | 0.84763 | -0.27689 | 18042 | |
| Clip2 | 0.84788 | -0.10591 | 18043 | |
| Ssbp4 | 0.84793 | 0.24998 | 18044 | |
| Mycl | 0.84794 | 2.3772 | 18045 | |
| Pros1 | 0.84797 | -0.37995 | 18046 | |
| 1700009P17Rik | 0.84809 | 0.96941 | 18047 | |
| Tmprss11e | 0.84811 | 0.58177 | 18048 | |
| 4930404A10Rik | 0.84826 | 0.82657 | 18049 | |
| Chaf1a | 0.84836 | 1.3312 | 18050 | |
| Rwdd2a | 0.8484 | 1.1321 | 18051 | |
| Gja3 | 0.84854 | 1.7933 | 18052 | |
| Sorl1 | 0.84856 | 0.098112 | 18053 | |
| mmu-mir-297a-3 | 0.84865 | 1.8429 | 18054 | |
| Olfr854 | 0.84867 | -0.2355 | 18055 | |
| Arhgap19 | 0.84872 | -0.31522 | 18056 | |
| 9530002B09Rik | 0.84873 | 0.55818 | 18057 | |
| Shroom3 | 0.84879 | -0.0093549 | 18058 | |
| Prl6a1 | 0.8489 | 1.9899 | 18059 | |
| Gm20826 | 0.84894 | 0.33994 | 18060 | |
| Nlrp4c | 0.84897 | 1.0606 | 18061 | |
| Psca | 0.84905 | 0.68796 | 18062 | |
| Taf6 | 0.84908 | 0.76045 | 18063 | |
| Cort | 0.84909 | -0.42015 | 18064 | |
| Myh10 | 0.8491 | 0.071652 | 18065 | |
| Rcor1 | 0.84917 | -0.38623 | 18066 | |
| 3110001I22Rik | 0.84921 | 0.6693 | 18067 | |
| Renbp | 0.8493 | -0.38361 | 18068 | |
| Vmn1r32 | 0.84931 | -0.32684 | 18069 | |
| Kifc3 | 0.84934 | 0.61252 | 18070 | |
| mmu-mir-706 | 0.84945 | 0.31771 | 18071 | |
| Tmem66 | 0.84953 | -0.31764 | 18072 | |
| Rbp2 | 0.84954 | -0.31094 | 18073 | |
| 9130409I23Rik | 0.84968 | 0.41757 | 18074 | |
| Map10 | 0.8498 | -0.12846 | 18075 | |
| Sec14l5 | 0.84988 | 1.6879 | 18076 | |
| Zfp839 | 0.84989 | -0.28203 | 18077 | |
| Pdxk | 0.84995 | 0.21933 | 18078 | |
| Glg1 | 0.84998 | 0.94247 | 18079 | |
| Mfsd7b | 0.84999 | 0.53708 | 18080 | |
| Ttc34 | 0.85019 | -0.15168 | 18081 | |
| Prmt3 | 0.8502 | 0.8665 | 18082 | |
| 2900026A02Rik | 0.8503 | 0.68139 | 18083 | |
| AU018829 | 0.85033 | 0.33659 | 18084 | |
| Gm11568 | 0.85045 | 2.4872 | 18085 | |
| Catsper1 | 0.85047 | 0.36446 | 18086 | |
| Tmem60 | 0.85049 | 0.7236 | 18087 | |
| mmu-mir-761 | 0.8505 | 0.16779 | 18088 | |
| Fv1 | 0.85051 | 0.13908 | 18089 | |
| Mkx | 0.85061 | 0.24964 | 18090 | |
| Gpc5 | 0.85062 | -0.31701 | 18091 | |
| Scn2a1 | 0.85063 | 0.81776 | 18092 | |
| Apobec3 | 0.85067 | 0.43403 | 18093 | |
| Arg2 | 0.85069 | 0.29067 | 18094 | |
| Gm15085 | 0.85073 | 1.1965 | 18095 | |
| Tial1 | 0.85075 | 0.36153 | 18096 | |
| 4921530L21Rik | 0.8508 | 0.19669 | 18097 | |
| Lrrc4b | 0.85081 | -0.084067 | 18098 | |
| Slc9a3 | 0.85086 | -0.3388 | 18099 | |
| Kcnk6 | 0.85088 | 0.11396 | 18100 | |
| Zfp707 | 0.85103 | 0.9939 | 18101 | |
| Nmi | 0.85104 | 1.209 | 18102 | |
| Hist1h1b | 0.85112 | 0.11932 | 18103 | |
| Arhgap40 | 0.85126 | 0.017988 | 18104 | |
| 1700010B08Rik | 0.8513 | 0.20525 | 18105 | |
| Unc13b | 0.85141 | 0.062246 | 18106 | |
| Eaf2 | 0.85143 | 2.3677 | 18107 | |
| Ftsj1 | 0.85144 | -0.15866 | 18108 | |
| Mansc4 | 0.8515 | -0.090253 | 18109 | |
| Zfand2a | 0.85154 | 0.011331 | 18110 | |
| H6pd | 0.85161 | 1.0021 | 18111 | |
| Gm13275 | 0.85163 | 0.34022 | 18112 | |
| Rtn1 | 0.85182 | -0.13812 | 18113 | |
| Vmn2r121 | 0.85187 | -0.050391 | 18114 | |
| Cln6 | 0.85191 | 0.21009 | 18115 | |
| Dnmt3l | 0.85194 | 0.48766 | 18116 | |
| Abcc5 | 0.85204 | 0.57129 | 18117 | |
| Nrk | 0.85208 | 0.23629 | 18118 | |
| Cldn15 | 0.85218 | 0.27528 | 18119 | |
| Aim1l | 0.8522 | 0.071168 | 18120 | |
| Slfn2 | 0.85222 | 0.45058 | 18121 | |
| Gpaa1 | 0.85237 | 0.59341 | 18122 | |
| mmu-mir-9-2 | 0.85239 | 0.20908 | 18123 | |
| Slc25a46 | 0.85243 | 0.73087 | 18124 | |
| 3632451O06Rik | 0.85245 | 0.10245 | 18125 | |
| Arxes1 | 0.85246 | -0.2813 | 18126 | |
| Cym | 0.85256 | 0.94245 | 18127 | |
| Zp1 | 0.85259 | 0.14439 | 18128 | |
| Hic1 | 0.85261 | -0.33061 | 18129 | |
| Faxc | 0.85266 | 0.081514 | 18130 | |
| Ubash3b | 0.85276 | -0.046476 | 18131 | |
| Bpifb3 | 0.85283 | 1.2512 | 18132 | |
| Olfr568 | 0.85284 | 0.77116 | 18133 | |
| Rgs14 | 0.85287 | -0.36064 | 18134 | |
| Olfr427 | 0.85287 | 0.85614 | 18135 | |
| Abl2 | 0.853 | 0.28903 | 18136 | |
| Crabp2 | 0.85312 | -0.18146 | 18137 | |
| Arfgef2 | 0.85313 | -0.27745 | 18138 | |
| Ppia | 0.85318 | 0.22565 | 18139 | |
| Tmprss9 | 0.85333 | -0.37944 | 18140 | |
| Ltc4s | 0.85336 | -0.19636 | 18141 | |
| 1110007C09Rik | 0.85337 | -0.037848 | 18142 | |
| Cwf19l1 | 0.85349 | 0.014217 | 18143 | |
| Crtc3 | 0.85354 | -0.36976 | 18144 | |
| Ccdc23 | 0.85357 | 0.76695 | 18145 | |
| Bbs4 | 0.85359 | 0.82957 | 18146 | |
| Tpsg1 | 0.85366 | 1.2907 | 18147 | |
| Zfp940 | 0.85374 | 0.6688 | 18148 | |
| Zfp251 | 0.8539 | 0.16187 | 18149 | |
| Amer3 | 0.85394 | 0.14088 | 18150 | |
| Cyp2c65 | 0.8541 | 0.028873 | 18151 | |
| Elovl6 | 0.85423 | -0.038941 | 18152 | |
| Olfr872 | 0.85424 | 0.037605 | 18153 | |
| Tbc1d4 | 0.85427 | 0.0021208 | 18154 | |
| Zfp456 | 0.85433 | -0.20715 | 18155 | |
| Scnn1a | 0.85434 | 0.62733 | 18156 | |
| Plekhf2 | 0.85439 | 0.087855 | 18157 | |
| Ddit4 | 0.85443 | 0.113 | 18158 | |
| Emilin1 | 0.8545 | 0.12424 | 18159 | |
| 2510002D24Rik | 0.85455 | 0.020662 | 18160 | |
| Plekhh2 | 0.85458 | 0.798 | 18161 | |
| mmu-mir-467a-3 | 0.85467 | 1.2942 | 18162 | |
| Egfl8 | 0.85481 | -0.38471 | 18163 | |
| Wfdc1 | 0.85482 | 0.024868 | 18164 | |
| 1700017D01Rik | 0.85485 | -0.32905 | 18165 | |
| Prr15 | 0.85487 | 0.43282 | 18166 | |
| Olfr1381 | 0.85488 | 0.1006 | 18167 | |
| Kcna1 | 0.855 | 1.2548 | 18168 | |
| Btn1a1 | 0.85504 | -0.035417 | 18169 | |
| Olfr490 | 0.85505 | -0.0061398 | 18170 | |
| Slc30a5 | 0.85512 | -0.2028 | 18171 | |
| Rab33a | 0.85516 | -0.11016 | 18172 | |
| Gata2 | 0.85518 | 0.22523 | 18173 | |
| Hdac5 | 0.85525 | 0.053564 | 18174 | |
| Ppp1r21 | 0.85527 | 0.13809 | 18175 | |
| Myo9a | 0.85533 | 0.11061 | 18176 | |
| Gpd1l | 0.8554 | 0.64738 | 18177 | |
| Ncoa5 | 0.85553 | 0.11364 | 18178 | |
| Olfr532 | 0.85555 | 0.070553 | 18179 | |
| Galm | 0.85561 | 0.57264 | 18180 | |
| Olfr435 | 0.85564 | -0.093545 | 18181 | |
| Unc13c | 0.85568 | -0.30543 | 18182 | |
| Tnfrsf13c | 0.85569 | 2.1562 | 18183 | |
| Tuba4a | 0.85574 | 0.65164 | 18184 | |
| Exoc6b | 0.85581 | 1.0408 | 18185 | |
| Klrk1 | 0.85586 | -0.34163 | 18186 | |
| Zcchc10 | 0.85589 | -0.087348 | 18187 | |
| Irf5 | 0.85595 | 0.63216 | 18188 | |
| Ttll7 | 0.85596 | 1.4411 | 18189 | |
| Lef1 | 0.856 | 0.037883 | 18190 | |
| Tnfsf12 | 0.85602 | 0.84672 | 18191 | |
| Hnrnpll | 0.85623 | -0.23924 | 18192 | |
| 1700020L24Rik | 0.85628 | -0.32213 | 18193 | |
| Tmem247 | 0.85629 | 0.59838 | 18194 | |
| Trex2 | 0.8563 | 0.73653 | 18195 | |
| Strn | 0.85631 | 0.90946 | 18196 | |
| Fam134c | 0.85649 | -0.15027 | 18197 | |
| Olfr668 | 0.8565 | -0.16016 | 18198 | |
| Serpina3i | 0.85661 | 0.39952 | 18199 | |
| Pgm2 | 0.85669 | -0.094983 | 18200 | |
| Tex13a | 0.85685 | 0.41947 | 18201 | |
| Syp | 0.85701 | -0.39891 | 18202 | |
| Ribc2 | 0.85704 | 0.12135 | 18203 | |
| Ppig | 0.85705 | 1.5291 | 18204 | |
| Ajap1 | 0.85707 | 0.58653 | 18205 | |
| Ntsr2 | 0.8571 | 0.11882 | 18206 | |
| B4galnt2 | 0.85715 | 0.39488 | 18207 | |
| Dbndd1 | 0.85718 | 0.028924 | 18208 | |
| Susd4 | 0.85724 | -0.066638 | 18209 | |
| Tjp1 | 0.85726 | -0.09456 | 18210 | |
| Aknad1 | 0.85737 | 0.28249 | 18211 | |
| Myom3 | 0.85738 | -0.13641 | 18212 | |
| Olfr1367 | 0.85745 | -0.39976 | 18213 | |
| Sdf4 | 0.85748 | 0.52241 | 18214 | |
| mmu-mir-6945 | 0.85751 | 0.67034 | 18215 | |
| Uprt | 0.85752 | 0.98851 | 18216 | |
| Gm20736 | 0.85753 | 0.76957 | 18217 | |
| Pald1 | 0.85763 | 1.0706 | 18218 | |
| Pask | 0.85765 | 0.95282 | 18219 | |
| Col10a1 | 0.85766 | 0.051744 | 18220 | |
| Cyp3a57 | 0.8577 | 0.68086 | 18221 | |
| Ube4b | 0.85783 | -0.33852 | 18222 | |
| Eps8l3 | 0.85784 | -0.38647 | 18223 | |
| Etfdh | 0.85789 | 0.53767 | 18224 | |
| Olfr168 | 0.85791 | -0.12809 | 18225 | |
| Syngr1 | 0.85805 | 0.22943 | 18226 | |
| Vmn1r59 | 0.85818 | -0.20172 | 18227 | |
| Foxd1 | 0.85823 | 0.27134 | 18228 | |
| Vmn1r55 | 0.85825 | 0.43819 | 18229 | |
| Nrcam | 0.85828 | -0.41897 | 18230 | |
| Cisd2 | 0.85829 | -0.29456 | 18231 | |
| Cblb | 0.85835 | 0.98272 | 18232 | |
| Fbxl3 | 0.85836 | 0.22045 | 18233 | |
| Olfr1086 | 0.85838 | 0.22812 | 18234 | |
| Dgcr14 | 0.85845 | 0.55229 | 18235 | |
| Fam50b | 0.85848 | 0.78925 | 18236 | |
| Sgsh | 0.85848 | 0.70943 | 18237 | |
| Egfem1 | 0.85854 | 0.41946 | 18238 | |
| Sox21 | 0.85856 | -0.2589 | 18239 | |
| Edn1 | 0.8586 | 0.33367 | 18240 | |
| Slc9b1 | 0.85862 | -0.23963 | 18241 | |
| mmu-mir-466p | 0.85865 | 0.21791 | 18242 | |
| mmu-mir-152 | 0.85873 | -0.29108 | 18243 | |
| Tmprss12 | 0.85882 | 1.0167 | 18244 | |
| Arl13a | 0.85882 | -0.033434 | 18245 | |
| Sycp1 | 0.85885 | 0.10386 | 18246 | |
| Taz | 0.85912 | 0.22826 | 18247 | |
| Gm13889 | 0.85913 | 0.81323 | 18248 | |
| Drap1 | 0.85914 | -0.10146 | 18249 | |
| Camta2 | 0.85919 | 0.4313 | 18250 | |
| Akap17b | 0.8592 | 0.0061534 | 18251 | |
| Zfp488 | 0.85924 | 0.59213 | 18252 | |
| Azi1 | 0.85925 | -0.019086 | 18253 | |
| Hmox1 | 0.85927 | 1.9045 | 18254 | |
| Myo5b | 0.85929 | 0.27953 | 18255 | |
| Msantd1 | 0.85938 | -0.1254 | 18256 | |
| Abcc1 | 0.85954 | 0.046379 | 18257 | |
| Dscaml1 | 0.85963 | 0.037936 | 18258 | |
| Cap1 | 0.85967 | 3.1215 | 18259 | |
| Klf9 | 0.85972 | 2.287 | 18260 | |
| Tlk2 | 0.85978 | 1.0015 | 18261 | |
| Ooep | 0.85995 | -0.41394 | 18262 | |
| Gabrr1 | 0.85998 | 1.152 | 18263 | |
| mmu-mir-7073 | 0.85999 | 0.84798 | 18264 | |
| Dmgdh | 0.86 | 0.55917 | 18265 | |
| Ssu2 | 0.86006 | 0.60011 | 18266 | |
| Ptgs2 | 0.86013 | 0.64558 | 18267 | |
| Duxbl1 | 0.86015 | 0.61235 | 18268 | |
| Snrpa | 0.86017 | 0.12921 | 18269 | |
| Slc2a13 | 0.86019 | 0.57893 | 18270 | |
| Vkorc1l1 | 0.86022 | -0.33237 | 18271 | |
| Atxn1l | 0.86028 | -0.19132 | 18272 | |
| Capza2 | 0.86035 | 0.11219 | 18273 | |
| Zbtb6 | 0.86037 | 0.23014 | 18274 | |
| Olfr228 | 0.8604 | 0.017274 | 18275 | |
| Serpina1a | 0.86041 | 0.34884 | 18276 | |
| Pim2 | 0.86049 | 0.91552 | 18277 | |
| mmu-mir-301a | 0.86054 | 0.58866 | 18278 | |
| Cyp39a1 | 0.86058 | 0.0071681 | 18279 | |
| Ubl4 | 0.86062 | -0.12495 | 18280 | |
| Nsg2 | 0.86065 | 0.31842 | 18281 | |
| Kcnk15 | 0.86073 | -0.42806 | 18282 | |
| Numb | 0.86085 | -0.049363 | 18283 | |
| Spag8 | 0.86099 | 0.031644 | 18284 | |
| AI464131 | 0.86103 | 0.78669 | 18285 | |
| Zfp37 | 0.86121 | -0.31526 | 18286 | |
| Myo1c | 0.86125 | 0.84635 | 18287 | |
| Mink1 | 0.86126 | -0.18727 | 18288 | |
| Hoxa3 | 0.86128 | -0.076098 | 18289 | |
| Ell | 0.86131 | -0.36348 | 18290 | |
| Kdm6b | 0.86132 | 0.23785 | 18291 | |
| Olfr618 | 0.86134 | 0.41677 | 18292 | |
| Pou3f4 | 0.86137 | 0.51734 | 18293 | |
| B3galtl | 0.86138 | 1.0986 | 18294 | |
| Ms4a6d | 0.8614 | -0.32814 | 18295 | |
| Ddn | 0.86141 | -0.069198 | 18296 | |
| Gipc1 | 0.86145 | 0.94112 | 18297 | |
| C8b | 0.86147 | 0.92498 | 18298 | |
| Zfyve19 | 0.86152 | -0.26881 | 18299 | |
| Wnt6 | 0.86156 | -0.26038 | 18300 | |
| Cd8b1 | 0.86158 | 0.46635 | 18301 | |
| BC035044 | 0.86163 | 0.96846 | 18302 | |
| Tysnd1 | 0.86165 | 0.28383 | 18303 | |
| Gba2 | 0.86174 | 1.4138 | 18304 | |
| Fam65c | 0.86175 | -0.089413 | 18305 | |
| Ccdc96 | 0.86176 | -0.2914 | 18306 | |
| Prdm14 | 0.86182 | -0.10205 | 18307 | |
| Kcnn2 | 0.86187 | 0.18023 | 18308 | |
| Pja2 | 0.8619 | 0.40309 | 18309 | |
| Ugdh | 0.86196 | -0.35425 | 18310 | |
| Pemt | 0.86199 | -0.18942 | 18311 | |
| Pcdha4-g | 0.86205 | 1.8366 | 18312 | |
| mmu-mir-7085 | 0.86206 | -0.14612 | 18313 | |
| Zfp64 | 0.86207 | -0.25164 | 18314 | |
| 4930544G11Rik | 0.8621 | 0.024564 | 18315 | |
| Irak1bp1 | 0.86216 | 0.12167 | 18316 | |
| Snai3 | 0.86221 | -0.29545 | 18317 | |
| Vstm5 | 0.86227 | -0.19065 | 18318 | |
| Cnga2 | 0.8623 | 0.50498 | 18319 | |
| Csf3 | 0.86231 | 0.050665 | 18320 | |
| Fam211a | 0.86233 | 1.2323 | 18321 | |
| Smoc1 | 0.86236 | -0.054685 | 18322 | |
| Ttc7b | 0.86238 | 0.053597 | 18323 | |
| Unc13d | 0.86239 | 1.6038 | 18324 | |
| Cck | 0.86241 | -0.27924 | 18325 | |
| Sipa1 | 0.86243 | -0.20093 | 18326 | |
| Slc46a1 | 0.86258 | 0.60009 | 18327 | |
| H2-M2 | 0.86259 | -0.21982 | 18328 | |
| Lcn4 | 0.8626 | 0.317 | 18329 | |
| Cmas | 0.86266 | 0.37775 | 18330 | |
| 1700029J07Rik | 0.86269 | -0.1398 | 18331 | |
| Pde6c | 0.8627 | 0.26767 | 18332 | |
| Kremen1 | 0.86277 | 0.27693 | 18333 | |
| Aaas | 0.86278 | 0.0052092 | 18334 | |
| Fam193a | 0.86279 | 0.85729 | 18335 | |
| Crb1 | 0.86281 | 0.026909 | 18336 | |
| Ptgir | 0.86282 | -0.045402 | 18337 | |
| Filip1 | 0.86285 | 0.32585 | 18338 | |
| Cbs | 0.86295 | -0.12611 | 18339 | |
| Lrrc16b | 0.86299 | -0.11339 | 18340 | |
| Dnd1 | 0.86301 | 0.5939 | 18341 | |
| Nlrp1a | 0.86313 | 0.2458 | 18342 | |
| Kcnh2 | 0.86322 | -0.046949 | 18343 | |
| Evl | 0.86323 | -0.20189 | 18344 | |
| mmu-mir-208a | 0.86324 | 0.72528 | 18345 | |
| Tfe3 | 0.86324 | 1.0397 | 18346 | |
| Kcnq2 | 0.86327 | 0.72561 | 18347 | |
| Cpn1 | 0.86329 | -0.088881 | 18348 | |
| Gm4984 | 0.86334 | 0.2087 | 18349 | |
| mmu-mir-7222 | 0.86348 | 0.39692 | 18350 | |
| Pcdhga6 | 0.86351 | 2.1096 | 18351 | |
| Lrch2 | 0.86351 | 0.40436 | 18352 | |
| Spint2 | 0.86353 | -0.28408 | 18353 | |
| Sphkap | 0.86357 | -0.25466 | 18354 | |
| Smim7 | 0.86359 | 1.0006 | 18355 | |
| Mlxip | 0.86366 | 1.5577 | 18356 | |
| mmu-mir-7054 | 0.86374 | 0.301 | 18357 | |
| Mylk2 | 0.86391 | 0.53896 | 18358 | |
| Jup | 0.86392 | 0.10645 | 18359 | |
| Lpcat1 | 0.864 | 0.13489 | 18360 | |
| Twsg1 | 0.86403 | -0.066654 | 18361 | |
| Araf | 0.86409 | 1.1983 | 18362 | |
| Samd9l | 0.86411 | 0.44179 | 18363 | |
| Tax1bp3 | 0.86415 | 1.1049 | 18364 | |
| Olfr1118 | 0.86418 | -0.1301 | 18365 | |
| Ppm1l | 0.86419 | 0.30315 | 18366 | |
| Olfr1442 | 0.86424 | 1.0271 | 18367 | |
| Atg9b | 0.86429 | 0.67895 | 18368 | |
| Fbxl2 | 0.86433 | 0.24568 | 18369 | |
| Tpbpa | 0.86435 | 0.028497 | 18370 | |
| mmu-mir-3080 | 0.86437 | 0.066996 | 18371 | |
| Ddx50 | 0.86442 | 0.40857 | 18372 | |
| Tcf24 | 0.86443 | 1.8698 | 18373 | |
| Ccl9 | 0.86445 | 2.4838 | 18374 | |
| mmu-mir-8114 | 0.86454 | 0.49618 | 18375 | |
| Ttll13 | 0.86455 | -0.074152 | 18376 | |
| Pramel5 | 0.86457 | -0.39308 | 18377 | |
| Syt1 | 0.86459 | -0.43173 | 18378 | |
| Apom | 0.8646 | 1.023 | 18379 | |
| C1ql2 | 0.86464 | -0.077957 | 18380 | |
| Syk | 0.86478 | 1.1759 | 18381 | |
| Xrcc1 | 0.86479 | -0.22323 | 18382 | |
| Ppapdc3 | 0.86483 | 0.50726 | 18383 | |
| Pdcd6 | 0.8649 | 0.49867 | 18384 | |
| Gm9112 | 0.86517 | -0.019247 | 18385 | |
| 1700028P14Rik | 0.86518 | 0.0045504 | 18386 | |
| Sash1 | 0.86523 | -0.078057 | 18387 | |
| Slc1a2 | 0.86531 | -0.36799 | 18388 | |
| Vmn1r187 | 0.86536 | 0.32469 | 18389 | |
| Foxa2 | 0.86541 | -0.43243 | 18390 | |
| Nampt | 0.86543 | 0.2763 | 18391 | |
| Msantd2 | 0.86548 | 0.0076904 | 18392 | |
| Prss40 | 0.86551 | 0.23243 | 18393 | |
| Podxl | 0.86552 | 0.22528 | 18394 | |
| Hoxa9 | 0.86558 | -0.33989 | 18395 | |
| Spry2 | 0.86561 | -0.38446 | 18396 | |
| Slirp | 0.86562 | 0.58582 | 18397 | |
| Rarb | 0.86567 | -0.10966 | 18398 | |
| AB124611 | 0.8657 | -0.27829 | 18399 | |
| Cdr1 | 0.86573 | 0.14749 | 18400 | |
| Tollip | 0.86575 | 0.061486 | 18401 | |
| Snx8 | 0.86582 | 0.033679 | 18402 | |
| Cfl2 | 0.86589 | -0.2142 | 18403 | |
| Sult1e1 | 0.86592 | 1.1388 | 18404 | |
| Arhgef4 | 0.86593 | -0.061889 | 18405 | |
| Olfr678 | 0.86606 | -0.1989 | 18406 | |
| Cd59b | 0.86607 | 0.71982 | 18407 | |
| Klra5 | 0.86613 | 0.2258 | 18408 | |
| Med1 | 0.86614 | -0.25386 | 18409 | |
| Vmn2r123 | 0.86616 | -0.1888 | 18410 | |
| mmu-mir-3473g | 0.86619 | -0.043042 | 18411 | |
| Olfr1030 | 0.8662 | 0.27485 | 18412 | |
| Eps8 | 0.86624 | 1.284 | 18413 | |
| Map3k2 | 0.86628 | 0.40236 | 18414 | |
| Scube3 | 0.8663 | -0.31682 | 18415 | |
| Esrra | 0.86632 | 0.23259 | 18416 | |
| Krt42 | 0.86635 | 0.10234 | 18417 | |
| Wdr8 | 0.86639 | 0.28856 | 18418 | |
| Fgd1 | 0.86643 | -0.38677 | 18419 | |
| Xkr8 | 0.86661 | -0.32603 | 18420 | |
| Bmp4 | 0.86668 | -0.20093 | 18421 | |
| Hsd3b6 | 0.8667 | 0.24064 | 18422 | |
| Elmod1 | 0.86675 | 0.35507 | 18423 | |
| Smarca1 | 0.8668 | 0.28943 | 18424 | |
| F13a1 | 0.86686 | 0.28888 | 18425 | |
| mmu-mir-103-2 | 0.86702 | 0.77795 | 18426 | |
| Hlx | 0.86712 | 0.90833 | 18427 | |
| Mmab | 0.86714 | 0.41977 | 18428 | |
| Pex11g | 0.86739 | 0.054503 | 18429 | |
| Eif3e | 0.8674 | 1.9464 | 18430 | |
| Scrt2 | 0.86744 | -0.11653 | 18431 | |
| Commd5 | 0.86745 | -0.34617 | 18432 | |
| Abca8b | 0.86746 | -0.12182 | 18433 | |
| Gfap | 0.86747 | 0.10116 | 18434 | |
| Slc11a1 | 0.86762 | 1.0417 | 18435 | |
| Sun5 | 0.86764 | 1.0791 | 18436 | |
| Sval2 | 0.86765 | 0.55836 | 18437 | |
| Foxn2 | 0.86769 | -0.43247 | 18438 | |
| Pou1f1 | 0.86772 | -0.26206 | 18439 | |
| Fam217b | 0.86778 | 0.1851 | 18440 | |
| Rwdd1 | 0.86782 | 0.81546 | 18441 | |
| Ppp1r14a | 0.86783 | 0.74958 | 18442 | |
| Tktl1 | 0.86785 | -0.040773 | 18443 | |
| Mblac2 | 0.86788 | 2.3617 | 18444 | |
| Usp51 | 0.86792 | -0.017677 | 18445 | |
| Nosip | 0.86805 | 0.1868 | 18446 | |
| Krt7 | 0.86807 | -0.1593 | 18447 | |
| Serpinb6d | 0.86809 | 0.81368 | 18448 | |
| Rlf | 0.86811 | -0.014361 | 18449 | |
| Hist2h2ab | 0.86816 | -0.31174 | 18450 | |
| Glis3 | 0.86821 | -0.047918 | 18451 | |
| Ocm | 0.86825 | 0.19838 | 18452 | |
| Dynlt1a | 0.8684 | 0.075855 | 18453 | |
| Gm21677 | 0.8685 | 0.47585 | 18454 | |
| Vmn2r66 | 0.86852 | -0.28407 | 18455 | |
| Aadac | 0.86855 | -0.015366 | 18456 | |
| mmu-mir-1956 | 0.86855 | 0.49605 | 18457 | |
| Mogat1 | 0.86856 | 1.6979 | 18458 | |
| Thoc6 | 0.86861 | -0.017454 | 18459 | |
| Tgfbi | 0.86863 | -0.27289 | 18460 | |
| Esp3 | 0.86867 | -0.09466 | 18461 | |
| Treml4 | 0.86871 | 0.084575 | 18462 | |
| Rabl6 | 0.86876 | 0.0062035 | 18463 | |
| Acr | 0.86878 | 1.6523 | 18464 | |
| Fgf14 | 0.86882 | 0.57487 | 18465 | |
| Slitrk6 | 0.86883 | -0.44615 | 18466 | |
| Fzd10 | 0.86885 | 0.22728 | 18467 | |
| Olfr527 | 0.86896 | 0.47068 | 18468 | |
| Scgb1b30 | 0.86902 | 0.30088 | 18469 | |
| Rnf220 | 0.86905 | 1.2205 | 18470 | |
| Actrt2 | 0.86907 | 0.26926 | 18471 | |
| Zfp523 | 0.86917 | -0.055499 | 18472 | |
| Pak3 | 0.86929 | -0.42367 | 18473 | |
| Gm5108 | 0.86932 | 0.068768 | 18474 | |
| Crabp1 | 0.86943 | 0.1511 | 18475 | |
| Hist1h4f | 0.86946 | 0.1734 | 18476 | |
| Ift46 | 0.86949 | -0.24613 | 18477 | |
| Disp2 | 0.8695 | -0.33253 | 18478 | |
| Klhl11 | 0.86952 | -0.33666 | 18479 | |
| Map3k8 | 0.86953 | -0.41101 | 18480 | |
| Npepl1 | 0.86958 | -0.008687 | 18481 | |
| Ppp4r4 | 0.86962 | -0.25525 | 18482 | |
| mmu-mir-6917 | 0.86964 | 0.24276 | 18483 | |
| Ercc1 | 0.86966 | 0.2978 | 18484 | |
| Pramel4 | 0.86974 | 1.3023 | 18485 | |
| Foxc2 | 0.8698 | 0.24823 | 18486 | |
| mmu-mir-671 | 0.86992 | 0.35372 | 18487 | |
| Ckm | 0.87006 | 0.2977 | 18488 | |
| Mxi1 | 0.87011 | -0.046778 | 18489 | |
| C2cd4a | 0.87022 | -0.3435 | 18490 | |
| Olfr685 | 0.87024 | 0.77705 | 18491 | |
| Tmem160 | 0.87026 | -0.076512 | 18492 | |
| Olfr1445 | 0.87027 | 0.57719 | 18493 | |
| Gm20765 | 0.87034 | -0.031743 | 18494 | |
| Slc39a4 | 0.87043 | 0.1697 | 18495 | |
| Galnt11 | 0.87046 | 0.47995 | 18496 | |
| Ankrd60 | 0.87049 | 0.50871 | 18497 | |
| Cdh6 | 0.87059 | 0.43709 | 18498 | |
| Rbm10 | 0.8706 | 0.38073 | 18499 | |
| Znhit1 | 0.87067 | 0.95848 | 18500 | |
| C4bp | 0.87071 | -0.10788 | 18501 | |
| Fmnl3 | 0.87082 | 0.63995 | 18502 | |
| Etv6 | 0.87085 | 0.84581 | 18503 | |
| Ctbp1 | 0.87086 | 0.19357 | 18504 | |
| Vmn1r54 | 0.8709 | 0.73054 | 18505 | |
| Ifi205 | 0.87092 | -0.34526 | 18506 | |
| Mup21 | 0.87095 | 0.57104 | 18507 | |
| Sprr1b | 0.87098 | 0.48268 | 18508 | |
| Os9 | 0.87104 | 2.8764 | 18509 | |
| Abcc12 | 0.8711 | 0.93309 | 18510 | |
| Rnf111 | 0.87111 | 0.56771 | 18511 | |
| Sqstm1 | 0.87114 | 1.1093 | 18512 | |
| Gal3st4 | 0.87118 | -0.37163 | 18513 | |
| Entpd3 | 0.87119 | -0.23764 | 18514 | |
| Tgfbr2 | 0.87124 | 0.36849 | 18515 | |
| Olfr225 | 0.87127 | 0.65398 | 18516 | |
| 4921536K21Rik | 0.87134 | -0.0025493 | 18517 | |
| Mthfd2l | 0.8714 | 0.63861 | 18518 | |
| Abcf3 | 0.87144 | 0.05031 | 18519 | |
| Pthlh | 0.87147 | 0.066579 | 18520 | |
| Sh2d1b2 | 0.87148 | -0.034869 | 18521 | |
| Naga | 0.87154 | -0.41538 | 18522 | |
| Pex5 | 0.87159 | 0.81406 | 18523 | |
| Vmn1r14 | 0.87168 | 0.75002 | 18524 | |
| Smek1 | 0.87172 | 0.44215 | 18525 | |
| Gm436 | 0.87184 | 0.10208 | 18526 | |
| Olfr172 | 0.87185 | 1.326 | 18527 | |
| Kcnj11 | 0.87191 | -0.19539 | 18528 | |
| Olfr945 | 0.87193 | 1.8681 | 18529 | |
| Zfp938 | 0.87194 | -0.22225 | 18530 | |
| Wtip | 0.87201 | -0.21389 | 18531 | |
| Osr1 | 0.87202 | 0.13557 | 18532 | |
| Gm766 | 0.87207 | -0.090476 | 18533 | |
| Trem3 | 0.8722 | 0.79308 | 18534 | |
| Reg2 | 0.8723 | 0.031874 | 18535 | |
| Xlr3b | 0.87238 | 0.90811 | 18536 | |
| Fam161a | 0.87238 | 1.5485 | 18537 | |
| Fbxo38 | 0.8724 | 0.39575 | 18538 | |
| Acads | 0.87242 | -0.32744 | 18539 | |
| Trub1 | 0.87247 | 0.41863 | 18540 | |
| Etl4 | 0.87257 | -0.059524 | 18541 | |
| Mical2 | 0.87258 | 0.83056 | 18542 | |
| 6030419C18Rik | 0.87259 | 0.24626 | 18543 | |
| Vmn1r33 | 0.87264 | 1.3649 | 18544 | |
| Slc9a1 | 0.87265 | -0.07485 | 18545 | |
| Olfr418-ps1 | 0.87266 | 0.63487 | 18546 | |
| Omp | 0.87267 | 0.072 | 18547 | |
| Bnc1 | 0.87272 | -0.08108 | 18548 | |
| Cntnap3 | 0.87272 | -0.039259 | 18549 | |
| Grifin | 0.87279 | 0.92049 | 18550 | |
| Actl9 | 0.87282 | 0.46549 | 18551 | |
| Tmem131 | 0.87284 | 0.070469 | 18552 | |
| Alkbh5 | 0.87285 | 0.27693 | 18553 | |
| Nr2f6 | 0.87304 | 0.77516 | 18554 | |
| Cdc14b | 0.87306 | 0.7642 | 18555 | |
| Gm9 | 0.87307 | 1.7158 | 18556 | |
| Wnt1 | 0.8731 | -0.29391 | 18557 | |
| Slc44a4 | 0.87312 | 0.22654 | 18558 | |
| Flad1 | 0.87313 | 0.19343 | 18559 | |
| Mark1 | 0.87314 | 0.046519 | 18560 | |
| Adarb2 | 0.87315 | -0.19194 | 18561 | |
| Lmo1 | 0.87323 | 1.6288 | 18562 | |
| Eml2 | 0.87333 | 1.8081 | 18563 | |
| Vwa2 | 0.87334 | -0.065586 | 18564 | |
| Tlr9 | 0.87336 | 0.15564 | 18565 | |
| Abhd17c | 0.87339 | 0.22917 | 18566 | |
| Adam30 | 0.87344 | 0.21743 | 18567 | |
| Prkd2 | 0.87346 | 0.98916 | 18568 | |
| Myo15 | 0.87349 | -0.030207 | 18569 | |
| Ugt2b36 | 0.8735 | 0.92862 | 18570 | |
| Fam163a | 0.87353 | 0.015552 | 18571 | |
| D1Ertd622e | 0.87356 | -0.39057 | 18572 | |
| Vmn1r174 | 0.87365 | -0.092373 | 18573 | |
| Ftcd | 0.87367 | 2.0326 | 18574 | |
| Olfr572 | 0.8737 | -0.10404 | 18575 | |
| Shank3 | 0.87382 | 0.47322 | 18576 | |
| Olfr62 | 0.87386 | -0.37171 | 18577 | |
| Elf1 | 0.87388 | 0.61892 | 18578 | |
| Mfsd4 | 0.87394 | -0.34925 | 18579 | |
| Hspg2 | 0.87402 | 0.067089 | 18580 | |
| 2310014L17Rik | 0.87414 | 0.83923 | 18581 | |
| Pfkfb1 | 0.87422 | 0.29854 | 18582 | |
| Ppp2r2b | 0.87429 | 0.11295 | 18583 | |
| Fgf12 | 0.87438 | 0.032264 | 18584 | |
| Slc6a8 | 0.87444 | -0.18929 | 18585 | |
| Arhgap32 | 0.87445 | -0.27918 | 18586 | |
| Braf | 0.87462 | 0.46952 | 18587 | |
| Gm5728 | 0.87471 | 0.28197 | 18588 | |
| Asb2 | 0.87476 | 0.82054 | 18589 | |
| Fam129b | 0.87489 | 0.12827 | 18590 | |
| Mark3 | 0.87501 | 0.23821 | 18591 | |
| Adam18 | 0.87511 | -0.12148 | 18592 | |
| Shc4 | 0.87527 | 0.5127 | 18593 | |
| Rtp1 | 0.87531 | 1.0218 | 18594 | |
| Igfbp6 | 0.87536 | 0.12996 | 18595 | |
| Fam213b | 0.87543 | 0.42386 | 18596 | |
| Fbxw13 | 0.87545 | -0.32807 | 18597 | |
| Mta1 | 0.87546 | 0.38325 | 18598 | |
| Gdf6 | 0.87548 | -0.06597 | 18599 | |
| Atg16l1 | 0.87555 | 0.010543 | 18600 | |
| Cchcr1 | 0.87571 | 0.098292 | 18601 | |
| Cyp2c38 | 0.87583 | 0.6272 | 18602 | |
| Crispld1 | 0.87585 | -0.37716 | 18603 | |
| Pih1d1 | 0.87587 | 0.067895 | 18604 | |
| Sfrp4 | 0.87587 | 0.23407 | 18605 | |
| 4931440L10Rik | 0.87591 | 0.97759 | 18606 | |
| Adam11 | 0.87595 | 0.64643 | 18607 | |
| Rnf114 | 0.87596 | 0.2767 | 18608 | |
| Klra23 | 0.87597 | 0.95036 | 18609 | |
| Itgb3 | 0.87599 | -0.12835 | 18610 | |
| Pnck | 0.87603 | -0.20948 | 18611 | |
| C330007P06Rik | 0.87613 | 1.4454 | 18612 | |
| Man2b2 | 0.87623 | -0.23576 | 18613 | |
| Med24 | 0.87625 | -0.2956 | 18614 | |
| Rbfox3 | 0.87631 | 0.91115 | 18615 | |
| Kcne1 | 0.87633 | 0.42804 | 18616 | |
| Pyroxd2 | 0.87634 | 0.033341 | 18617 | |
| Muc4 | 0.87656 | 0.062498 | 18618 | |
| Atp13a5 | 0.8767 | -0.32303 | 18619 | |
| mmu-mir-6941 | 0.87674 | -0.042741 | 18620 | |
| Rfng | 0.87687 | 0.19164 | 18621 | |
| Nlrp12 | 0.87689 | -0.21065 | 18622 | |
| mmu-mir-3970 | 0.8769 | 0.57105 | 18623 | |
| Pwwp2a | 0.87692 | 0.010744 | 18624 | |
| Dcn | 0.87694 | 0.64969 | 18625 | |
| G630090E17Rik | 0.87695 | 0.57544 | 18626 | |
| Asb15 | 0.87698 | -0.30894 | 18627 | |
| Srpx | 0.8771 | 0.22714 | 18628 | |
| Etaa1 | 0.87711 | -0.23891 | 18629 | |
| mmu-mir-133a-1 | 0.87717 | 1.0279 | 18630 | |
| Tmem52b | 0.87717 | 0.33659 | 18631 | |
| Pik3r6 | 0.87718 | 0.32439 | 18632 | |
| Spag16 | 0.87722 | 0.62733 | 18633 | |
| Gm12429 | 0.87723 | 1.9502 | 18634 | |
| Smarcd3 | 0.87725 | -0.12089 | 18635 | |
| Sft2d2 | 0.8773 | 0.37456 | 18636 | |
| Suox | 0.87731 | -0.39628 | 18637 | |
| Zfp518a | 0.87737 | -0.014902 | 18638 | |
| mmu-mir-3073a | 0.8774 | 0.13815 | 18639 | |
| Ephb4 | 0.87744 | -0.17667 | 18640 | |
| Scn9a | 0.87746 | -0.38441 | 18641 | |
| Nkx3-1 | 0.87751 | 0.91671 | 18642 | |
| Ear6 | 0.87753 | 0.29038 | 18643 | |
| Zap70 | 0.87759 | -0.41731 | 18644 | |
| Ostn | 0.87763 | 0.18558 | 18645 | |
| Muc19 | 0.87766 | 0.60045 | 18646 | |
| Abca14 | 0.87767 | 0.50619 | 18647 | |
| Cad | 0.87773 | 0.0079567 | 18648 | |
| I830012O16Rik | 0.87774 | 0.070032 | 18649 | |
| Plcb1 | 0.8778 | 0.041327 | 18650 | |
| Sbspon | 0.87785 | 1.5997 | 18651 | |
| Prdm8 | 0.87786 | 0.15274 | 18652 | |
| mmu-mir-652 | 0.87789 | 0.39739 | 18653 | |
| Klhl38 | 0.87794 | 0.50968 | 18654 | |
| Epha6 | 0.87796 | 0.25719 | 18655 | |
| Fmnl1 | 0.87806 | 0.14863 | 18656 | |
| Esrrg | 0.87806 | 0.27229 | 18657 | |
| Trmt1l | 0.87808 | -0.21049 | 18658 | |
| Myrf | 0.87809 | -0.14159 | 18659 | |
| Zswim5 | 0.87813 | 1.4198 | 18660 | |
| Stam | 0.87815 | 0.20766 | 18661 | |
| Htr7 | 0.87819 | 0.33638 | 18662 | |
| Dcstamp | 0.87828 | -0.058659 | 18663 | |
| 4932411N23Rik | 0.87829 | -0.18649 | 18664 | |
| Ypel2 | 0.87842 | -0.31596 | 18665 | |
| Slc32a1 | 0.87847 | 0.10408 | 18666 | |
| Asl | 0.87849 | 1.1864 | 18667 | |
| Nt5c3b | 0.8785 | 0.58988 | 18668 | |
| Cxadr | 0.87851 | 0.58045 | 18669 | |
| Zfp692 | 0.87852 | 0.45293 | 18670 | |
| Fscn1 | 0.87857 | 1.8444 | 18671 | |
| 4931409K22Rik | 0.87859 | -0.12531 | 18672 | |
| Tas2r108 | 0.87862 | 1.5916 | 18673 | |
| Comp | 0.87865 | -0.075974 | 18674 | |
| Prr16 | 0.87869 | 0.72285 | 18675 | |
| Epb4.1l5 | 0.8787 | 1.0587 | 18676 | |
| 4931406P16Rik | 0.87871 | 0.91431 | 18677 | |
| Nrm | 0.87872 | 1.4097 | 18678 | |
| Zscan25 | 0.87876 | 0.091776 | 18679 | |
| Alas1 | 0.87883 | -0.022162 | 18680 | |
| Zmynd15 | 0.87885 | 0.23029 | 18681 | |
| St8sia2 | 0.87886 | -0.10454 | 18682 | |
| Il17rd | 0.8789 | 0.12641 | 18683 | |
| Olfr921 | 0.87892 | 1.2873 | 18684 | |
| Fbxo36 | 0.87896 | 0.92108 | 18685 | |
| Slc22a16 | 0.87902 | 1.7286 | 18686 | |
| Khnyn | 0.87907 | 0.58094 | 18687 | |
| Dgat2l6 | 0.87908 | -0.36514 | 18688 | |
| Psme2b | 0.8791 | 0.18738 | 18689 | |
| Bmpr1a | 0.87911 | 0.039025 | 18690 | |
| Adrbk1 | 0.87926 | 0.66149 | 18691 | |
| Pcdhgb2 | 0.87939 | 0.77822 | 18692 | |
| Nr1h4 | 0.87947 | 0.24755 | 18693 | |
| Abca6 | 0.87948 | 0.17291 | 18694 | |
| Tigd4 | 0.87951 | -0.15399 | 18695 | |
| Tecrl | 0.87952 | 1.1378 | 18696 | |
| Mrps5 | 0.87956 | 1.2201 | 18697 | |
| Hoxc8 | 0.87959 | 0.84777 | 18698 | |
| Gnaq | 0.87961 | -0.12119 | 18699 | |
| Fut7 | 0.8799 | 0.55926 | 18700 | |
| Cyp2c67 | 0.87993 | -0.38929 | 18701 | |
| Tnfaip8l3 | 0.87995 | -0.023891 | 18702 | |
| Car6 | 0.87996 | 0.51803 | 18703 | |
| Fam131a | 0.87999 | -0.080354 | 18704 | |
| Ifne | 0.8801 | 0.02196 | 18705 | |
| Rab11fip3 | 0.88011 | 0.41613 | 18706 | |
| Olfr1411 | 0.88016 | 1.0038 | 18707 | |
| Cthrc1 | 0.88029 | -0.078459 | 18708 | |
| Kif21b | 0.8803 | -0.28717 | 18709 | |
| Meaf6 | 0.88031 | -0.059842 | 18710 | |
| Frk | 0.88032 | -0.30652 | 18711 | |
| Rab22a | 0.88045 | 0.80499 | 18712 | |
| Nlrp3 | 0.88053 | 0.25147 | 18713 | |
| Fam129a | 0.88054 | 0.00719 | 18714 | |
| Prss56 | 0.88059 | -0.22719 | 18715 | |
| Stk38 | 0.88063 | 0.29628 | 18716 | |
| Gabre | 0.88064 | 0.15016 | 18717 | |
| Epx | 0.8808 | 0.47934 | 18718 | |
| Atp10d | 0.88091 | 1.1654 | 18719 | |
| mmu-mir-465c-1 | 0.88094 | 0.15772 | 18720 | |
| Sema3a | 0.88096 | 0.18574 | 18721 | |
| Rad23a | 0.88098 | 0.15226 | 18722 | |
| Dusp9 | 0.88102 | 0.43588 | 18723 | |
| Slc22a19 | 0.88108 | -0.34735 | 18724 | |
| Glipr1l1 | 0.88109 | 0.44893 | 18725 | |
| Zfp512 | 0.88115 | 0.18886 | 18726 | |
| mmu-mir-7672 | 0.88117 | 0.56917 | 18727 | |
| Sfmbt2 | 0.8812 | 0.74011 | 18728 | |
| Zbtb49 | 0.88123 | 0.25562 | 18729 | |
| Bsg | 0.88126 | 0.52282 | 18730 | |
| Trmt10b | 0.88129 | -0.23788 | 18731 | |
| Zranb1 | 0.88139 | 0.28006 | 18732 | |
| Ccdc148 | 0.88142 | 0.10351 | 18733 | |
| Acox2 | 0.88144 | 0.5141 | 18734 | |
| Hdac9 | 0.8815 | -0.039105 | 18735 | |
| Amigo2 | 0.88169 | -0.039802 | 18736 | |
| Prpmp5 | 0.88174 | 0.53882 | 18737 | |
| Aatk | 0.88175 | -0.097039 | 18738 | |
| Oxsm | 0.88176 | 0.10008 | 18739 | |
| Il5 | 0.88192 | -0.18191 | 18740 | |
| Scn2b | 0.88195 | 0.05956 | 18741 | |
| Elac1 | 0.88198 | 0.25084 | 18742 | |
| Lrsam1 | 0.88215 | 0.40447 | 18743 | |
| Xkrx | 0.8822 | 0.066012 | 18744 | |
| Il12rb2 | 0.88222 | -0.080967 | 18745 | |
| Cdhr1 | 0.88227 | 0.28923 | 18746 | |
| Fkbp1a | 0.88229 | 1.0956 | 18747 | |
| Ankrd66 | 0.88231 | 1.2765 | 18748 | |
| Flt4 | 0.88234 | 0.22497 | 18749 | |
| Dpy19l2 | 0.88249 | 2.1782 | 18750 | |
| Adora2b | 0.88253 | 0.46644 | 18751 | |
| Ces1g | 0.8826 | 1.371 | 18752 | |
| Il16 | 0.88265 | 0.33925 | 18753 | |
| Masp2 | 0.88268 | 0.17246 | 18754 | |
| Olfr1433 | 0.88277 | 0.27874 | 18755 | |
| Ibsp | 0.88279 | 0.8159 | 18756 | |
| Olfr686 | 0.88281 | 0.66372 | 18757 | |
| AU022751 | 0.88286 | 0.49835 | 18758 | |
| Olfr273 | 0.88288 | -0.13771 | 18759 | |
| Sec63 | 0.88289 | -0.25896 | 18760 | |
| Kcne1l | 0.88304 | 1.0113 | 18761 | |
| Nme9 | 0.88306 | 0.44183 | 18762 | |
| Gxylt1 | 0.88309 | 0.58131 | 18763 | |
| Arid3a | 0.88312 | 1.0599 | 18764 | |
| Arhgap21 | 0.88322 | 0.51362 | 18765 | |
| Inha | 0.8833 | 0.33689 | 18766 | |
| Olfr470 | 0.88334 | -0.11826 | 18767 | |
| C530008M17Rik | 0.88335 | 0.38523 | 18768 | |
| Tbc1d21 | 0.88336 | 1.7887 | 18769 | |
| Dhx38 | 0.88341 | -0.37687 | 18770 | |
| Slc39a13 | 0.88346 | 0.19217 | 18771 | |
| Nlrc5 | 0.88349 | 0.028714 | 18772 | |
| Mnda | 0.88352 | 0.59226 | 18773 | |
| Sept4 | 0.88361 | -0.30578 | 18774 | |
| Taar7f | 0.88363 | 0.81282 | 18775 | |
| Tldc2 | 0.88364 | 0.92043 | 18776 | |
| Trim45 | 0.88369 | 0.099572 | 18777 | |
| Tex22 | 0.88374 | 2.1121 | 18778 | |
| Whsc1l1 | 0.88384 | 1.0384 | 18779 | |
| 8430408G22Rik | 0.88386 | 0.21381 | 18780 | |
| Atat1 | 0.88401 | 0.65147 | 18781 | |
| 6030458C11Rik | 0.88403 | 0.47161 | 18782 | |
| Tas2r122 | 0.88404 | 0.23111 | 18783 | |
| 1700019D03Rik | 0.88406 | -0.15923 | 18784 | |
| Fcamr | 0.8841 | 0.68353 | 18785 | |
| Olfr78 | 0.88411 | 0.35504 | 18786 | |
| Fam160a2 | 0.88414 | 0.51238 | 18787 | |
| Map2k6 | 0.88416 | 0.29917 | 18788 | |
| Nwd1 | 0.88417 | -0.036051 | 18789 | |
| Vmn2r2 | 0.88418 | 1.1992 | 18790 | |
| Il9r | 0.88425 | -0.1162 | 18791 | |
| Kdm5a | 0.88429 | 1.0397 | 18792 | |
| Cblc | 0.8843 | -0.066143 | 18793 | |
| Vmn1r180 | 0.88433 | 0.73914 | 18794 | |
| Ttc13 | 0.88434 | -0.079814 | 18795 | |
| Olfr446 | 0.88439 | 0.69158 | 18796 | |
| Ahsg | 0.88446 | 0.04196 | 18797 | |
| Cebpa | 0.88448 | -0.038788 | 18798 | |
| Zfp119a | 0.88453 | -0.017024 | 18799 | |
| Tmed3 | 0.88454 | 0.23498 | 18800 | |
| D19Bwg1357e | 0.88459 | 1.3192 | 18801 | |
| Plagl2 | 0.88459 | 0.27512 | 18802 | |
| Cd2 | 0.88461 | 1.1115 | 18803 | |
| Slc16a1 | 0.88467 | -0.19763 | 18804 | |
| Pdcd7 | 0.88471 | 0.81976 | 18805 | |
| 2410004B18Rik | 0.8848 | 0.58709 | 18806 | |
| Mpnd | 0.885 | 0.23964 | 18807 | |
| Vmn1r128 | 0.88506 | 0.59505 | 18808 | |
| Dync1li1 | 0.88508 | 0.25486 | 18809 | |
| Klri2 | 0.8851 | 1.9717 | 18810 | |
| mmu-mir-871 | 0.8851 | 0.066081 | 18811 | |
| Slc4a11 | 0.88515 | -0.36664 | 18812 | |
| H2-DMb2 | 0.88521 | 3.5879 | 18813 | |
| Sgtb | 0.88522 | 0.008018 | 18814 | |
| Ntf5 | 0.88529 | 0.7858 | 18815 | |
| Spire1 | 0.88532 | -0.26208 | 18816 | |
| Mttp | 0.88546 | 0.35042 | 18817 | |
| Mid1ip1 | 0.88553 | 0.46833 | 18818 | |
| Tbc1d2b | 0.88554 | 0.51329 | 18819 | |
| Scgb1b2 | 0.88556 | 0.060287 | 18820 | |
| 1700007G11Rik | 0.88557 | 0.97273 | 18821 | |
| Tecpr2 | 0.88559 | 0.30505 | 18822 | |
| Pou5f1 | 0.8856 | 0.81679 | 18823 | |
| Vmn2r104 | 0.88561 | 1.4795 | 18824 | |
| Ntrk1 | 0.88562 | 0.35962 | 18825 | |
| 4921506M07Rik | 0.88565 | 0.60939 | 18826 | |
| Usp17lb | 0.88569 | 0.078699 | 18827 | |
| Cdc42ep2 | 0.8857 | 0.22102 | 18828 | |
| Lect2 | 0.88571 | 0.65857 | 18829 | |
| Ccr6 | 0.88572 | -0.28219 | 18830 | |
| Zfpm2 | 0.88579 | 0.36732 | 18831 | |
| Gstm3 | 0.88584 | -0.021178 | 18832 | |
| Strada | 0.88584 | 1.2126 | 18833 | |
| Ski | 0.88586 | -0.097083 | 18834 | |
| Olfr1239 | 0.88594 | 0.052141 | 18835 | |
| Rcan1 | 0.88595 | -0.30692 | 18836 | |
| Alox12 | 0.88597 | 0.12204 | 18837 | |
| Hp1bp3 | 0.88611 | 0.61352 | 18838 | |
| Mfn1 | 0.88615 | 2.0497 | 18839 | |
| Spata31d1b | 0.88616 | 0.39966 | 18840 | |
| Tob2 | 0.8862 | -0.091856 | 18841 | |
| Mrgprb8 | 0.88627 | 0.42614 | 18842 | |
| Olfr761 | 0.88629 | 0.072678 | 18843 | |
| Ncs1 | 0.88631 | 1.1843 | 18844 | |
| Palld | 0.88632 | 2.4191 | 18845 | |
| Reps1 | 0.88637 | 0.44123 | 18846 | |
| Dcp1a | 0.88638 | -0.18565 | 18847 | |
| Obox1 | 0.8864 | 0.6835 | 18848 | |
| Nkain1 | 0.88641 | -0.063445 | 18849 | |
| Tuft1 | 0.88645 | 0.72054 | 18850 | |
| Crebrf | 0.8865 | 1.364 | 18851 | |
| Fam179b | 0.88653 | 0.43779 | 18852 | |
| Gk5 | 0.8867 | 0.099216 | 18853 | |
| Otud3 | 0.88672 | -0.24693 | 18854 | |
| Dapl1 | 0.88674 | 0.50916 | 18855 | |
| Fndc5 | 0.88677 | -0.31044 | 18856 | |
| Gtf2h5 | 0.88682 | 1.6844 | 18857 | |
| Olfr1262 | 0.88685 | -0.27767 | 18858 | |
| Ifrd2 | 0.8869 | -0.32256 | 18859 | |
| Cers4 | 0.88691 | -0.34888 | 18860 | |
| Pcmt1 | 0.88693 | 0.86983 | 18861 | |
| Eogt | 0.88695 | 0.50503 | 18862 | |
| Grasp | 0.88707 | 0.40936 | 18863 | |
| Syt5 | 0.88714 | -0.17104 | 18864 | |
| Zfp947 | 0.88724 | 1.034 | 18865 | |
| Defb15 | 0.88726 | 0.67911 | 18866 | |
| Olfr1201 | 0.8873 | -0.27004 | 18867 | |
| Ptprc | 0.88746 | -0.32518 | 18868 | |
| Grm8 | 0.88748 | 0.21818 | 18869 | |
| Olfr561 | 0.88749 | 0.55272 | 18870 | |
| Shbg | 0.88756 | -0.0025806 | 18871 | |
| Rpusd2 | 0.88763 | -0.11724 | 18872 | |
| Btnl4 | 0.88766 | 1.083 | 18873 | |
| Tead4 | 0.88769 | 0.58386 | 18874 | |
| Dexi | 0.88771 | 0.28528 | 18875 | |
| Spata20 | 0.88773 | 0.52489 | 18876 | |
| Myh2 | 0.88782 | 0.74147 | 18877 | |
| Glyr1 | 0.88788 | -0.24671 | 18878 | |
| H2afb3-ps | 0.88789 | -0.23703 | 18879 | |
| 0610011F06Rik | 0.88792 | 1.2413 | 18880 | |
| Hpn | 0.88799 | 1.1895 | 18881 | |
| Ube3b | 0.88801 | 0.32771 | 18882 | |
| Tmem144 | 0.88803 | 0.80441 | 18883 | |
| Olfr371 | 0.88811 | 0.30679 | 18884 | |
| Camk2a | 0.88813 | -0.15262 | 18885 | |
| Napsa | 0.88817 | 0.33521 | 18886 | |
| Smpdl3b | 0.8882 | 0.42215 | 18887 | |
| Ambn | 0.88824 | 0.5349 | 18888 | |
| 3110021N24Rik | 0.88827 | -0.061438 | 18889 | |
| Frmd5 | 0.88829 | 0.29137 | 18890 | |
| Pou3f2 | 0.88838 | 1.3671 | 18891 | |
| Srek1ip1 | 0.88843 | -0.35256 | 18892 | |
| Pccb | 0.88844 | 0.79437 | 18893 | |
| Zswim2 | 0.88849 | 0.47125 | 18894 | |
| Plekhm2 | 0.8885 | -0.26959 | 18895 | |
| Fam151b | 0.88862 | 1.5024 | 18896 | |
| Sox12 | 0.88863 | -0.31873 | 18897 | |
| Cct6b | 0.88866 | 0.38305 | 18898 | |
| Spsb3 | 0.88873 | 0.88368 | 18899 | |
| Rab39b | 0.88879 | -0.059484 | 18900 | |
| Mab21l1 | 0.8888 | 1.0621 | 18901 | |
| Slc15a1 | 0.88881 | -0.29605 | 18902 | |
| mmu-mir-882 | 0.88884 | -0.15535 | 18903 | |
| Naip1 | 0.88889 | 0.78125 | 18904 | |
| Rbp3 | 0.88892 | 0.69721 | 18905 | |
| Fez1 | 0.88893 | -0.17019 | 18906 | |
| Irf4 | 0.88894 | -0.044229 | 18907 | |
| M1ap | 0.88899 | 0.24986 | 18908 | |
| Klhdc1 | 0.8891 | 0.80493 | 18909 | |
| Spopl | 0.88916 | 1.5549 | 18910 | |
| Trpm3 | 0.88917 | 0.21586 | 18911 | |
| Nefm | 0.88921 | 0.14676 | 18912 | |
| Mpzl3 | 0.88926 | 0.15935 | 18913 | |
| Cerkl | 0.88934 | -0.07588 | 18914 | |
| Lrfn2 | 0.88937 | 0.98693 | 18915 | |
| Pcdha11 | 0.88944 | 0.90245 | 18916 | |
| Parp10 | 0.88954 | 0.59374 | 18917 | |
| Apaf1 | 0.88955 | -0.14133 | 18918 | |
| Fxr1 | 0.88959 | 0.12199 | 18919 | |
| Ttc29 | 0.88961 | 1.8001 | 18920 | |
| Kdm7a | 0.88966 | 0.21112 | 18921 | |
| Rnf7 | 0.88997 | 0.54779 | 18922 | |
| Pabpc4l | 0.88999 | 0.020154 | 18923 | |
| Pirb | 0.89001 | 0.65962 | 18924 | |
| Ifit1 | 0.89005 | -0.24876 | 18925 | |
| Slc18b1 | 0.89025 | 0.028009 | 18926 | |
| Spg11 | 0.89026 | 1.0939 | 18927 | |
| Sval3 | 0.89034 | 0.12474 | 18928 | |
| Panx1 | 0.89043 | 1.2767 | 18929 | |
| Sms | 0.89051 | 0.030422 | 18930 | |
| Ntng2 | 0.89052 | 0.5302 | 18931 | |
| Zfp948 | 0.89057 | 0.7837 | 18932 | |
| Tnfsf9 | 0.89062 | 0.59905 | 18933 | |
| Trim63 | 0.89068 | 0.23759 | 18934 | |
| Iqcf4 | 0.89069 | 0.51512 | 18935 | |
| 4932418E24Rik | 0.89072 | -0.21824 | 18936 | |
| Dct | 0.89073 | -0.17985 | 18937 | |
| Suv420h1 | 0.89077 | 0.31346 | 18938 | |
| Pnma5 | 0.89083 | -0.17339 | 18939 | |
| 1810037I17Rik | 0.89085 | -0.081734 | 18940 | |
| Serpinc1 | 0.89089 | 0.40641 | 18941 | |
| Was | 0.89091 | 0.63032 | 18942 | |
| 9230112D13Rik | 0.89092 | 0.46999 | 18943 | |
| Klkb1 | 0.89094 | 0.28423 | 18944 | |
| H2-T10 | 0.89098 | -0.024252 | 18945 | |
| Ptplad1 | 0.89104 | -0.061087 | 18946 | |
| Hoxc5 | 0.89109 | 0.14309 | 18947 | |
| mmu-mir-7674 | 0.8911 | -0.11512 | 18948 | |
| Kcmf1 | 0.89112 | 0.18004 | 18949 | |
| Neurl2 | 0.89116 | 1.0653 | 18950 | |
| Csrp1 | 0.89127 | -0.069172 | 18951 | |
| Prodh | 0.89131 | 0.63061 | 18952 | |
| E2f7 | 0.89135 | -0.23603 | 18953 | |
| Phyhipl | 0.89136 | 0.19967 | 18954 | |
| Cd300a | 0.89137 | 0.15863 | 18955 | |
| Fam53b | 0.89146 | 2.596 | 18956 | |
| Fpr1 | 0.89153 | -0.027158 | 18957 | |
| Max | 0.89156 | 1.2261 | 18958 | |
| D7Ertd443e | 0.89159 | -0.17145 | 18959 | |
| Olfr1015 | 0.89161 | -0.21633 | 18960 | |
| Ctgf | 0.89163 | -0.0027238 | 18961 | |
| Dner | 0.89168 | 0.48224 | 18962 | |
| Rnaset2a | 0.89169 | -0.25317 | 18963 | |
| Col4a3bp | 0.89172 | 0.025348 | 18964 | |
| Tnfrsf19 | 0.89173 | 0.053523 | 18965 | |
| Il1rl2 | 0.89178 | 0.80491 | 18966 | |
| Elfn2 | 0.89187 | 0.093097 | 18967 | |
| Tsnaxip1 | 0.89193 | 0.20572 | 18968 | |
| Lyar | 0.892 | 1.3131 | 18969 | |
| Atp11c | 0.89203 | -0.30449 | 18970 | |
| Aldh1l1 | 0.89204 | -0.30749 | 18971 | |
| Olfr979 | 0.89207 | -0.17017 | 18972 | |
| Tnfrsf9 | 0.8922 | 0.66313 | 18973 | |
| Phka1 | 0.89221 | 0.46041 | 18974 | |
| Capsl | 0.89225 | 0.60275 | 18975 | |
| Col17a1 | 0.89231 | -0.071437 | 18976 | |
| Ptchd2 | 0.89243 | 0.16981 | 18977 | |
| mmu-mir-6344 | 0.89251 | 1.509 | 18978 | |
| Trim43b | 0.89254 | -0.05656 | 18979 | |
| Elane | 0.89257 | 1.8566 | 18980 | |
| Olfr1131 | 0.89259 | 0.12116 | 18981 | |
| 6030468B19Rik | 0.89262 | 0.045503 | 18982 | |
| Vps18 | 0.89265 | 1.9781 | 18983 | |
| Nostrin | 0.89266 | 0.14706 | 18984 | |
| Creg2 | 0.89269 | 1.0078 | 18985 | |
| Pip5k1c | 0.89276 | 1.0433 | 18986 | |
| Ube2q2 | 0.89281 | 0.63528 | 18987 | |
| Krtap17-1 | 0.89282 | -0.022792 | 18988 | |
| Trip12 | 0.89308 | 0.269 | 18989 | |
| Mtr | 0.89311 | -0.16341 | 18990 | |
| Rps10 | 0.89317 | -0.28249 | 18991 | |
| Zfp629 | 0.89327 | 0.46637 | 18992 | |
| Ppy | 0.89333 | -0.29997 | 18993 | |
| Smarca2 | 0.89337 | 0.1337 | 18994 | |
| Abhd16a | 0.89344 | -0.32272 | 18995 | |
| Ccdc57 | 0.89347 | 2.2198 | 18996 | |
| Gal3st3 | 0.8935 | 0.39625 | 18997 | |
| Gm1965 | 0.89351 | 0.33452 | 18998 | |
| Spred3 | 0.89352 | 1.0384 | 18999 | |
| Tmbim1 | 0.8936 | 0.59407 | 19000 | |
| Olfr18 | 0.89362 | -0.21138 | 19001 | |
| Slitrk1 | 0.89365 | -0.1665 | 19002 | |
| Dcpp2 | 0.89372 | 0.8841 | 19003 | |
| Rap1b | 0.89376 | -0.10877 | 19004 | |
| Gatad1 | 0.89379 | 0.89421 | 19005 | |
| Pcsk4 | 0.8938 | 0.8054 | 19006 | |
| Neu2 | 0.89382 | 2.764 | 19007 | |
| Tas2r130 | 0.89397 | 0.037949 | 19008 | |
| Cab39 | 0.89398 | 0.57266 | 19009 | |
| Thbs4 | 0.89403 | 2.7424 | 19010 | |
| Spred2 | 0.89404 | 0.40824 | 19011 | |
| Junb | 0.89407 | 0.0086889 | 19012 | |
| Abhd15 | 0.89409 | 0.14199 | 19013 | |
| Pigu | 0.89412 | -0.069956 | 19014 | |
| Nhlh2 | 0.89414 | -0.24318 | 19015 | |
| Olfr455 | 0.89416 | 0.60788 | 19016 | |
| Kcna10 | 0.89418 | 0.89737 | 19017 | |
| Olfr1532-ps1 | 0.89421 | 0.81049 | 19018 | |
| C130079G13Rik | 0.89422 | 0.39933 | 19019 | |
| Ltb4r2 | 0.89425 | 0.49962 | 19020 | |
| Idh3g | 0.89428 | 0.17193 | 19021 | |
| Ccr1 | 0.89432 | 0.85301 | 19022 | |
| Soga2 | 0.89438 | 0.89864 | 19023 | |
| Ptrf | 0.89439 | 0.5137 | 19024 | |
| Kcnk3 | 0.89449 | 1.8869 | 19025 | |
| Clcn2 | 0.89453 | 0.18417 | 19026 | |
| mmu-mir-1192 | 0.89454 | 0.83703 | 19027 | |
| Tagap | 0.89456 | -0.29568 | 19028 | |
| Olfr197 | 0.89458 | 0.46366 | 19029 | |
| Ramp1 | 0.89463 | 1.1342 | 19030 | |
| Olfr147 | 0.89465 | -0.15324 | 19031 | |
| mmu-mir-325 | 0.8947 | 0.3052 | 19032 | |
| Zfr | 0.89474 | -0.27753 | 19033 | |
| Olfr110 | 0.89481 | 0.45847 | 19034 | |
| Olfr1274-ps | 0.89484 | 0.58416 | 19035 | |
| mmu-mir-344d-2 | 0.89485 | -0.09192 | 19036 | |
| Ier5 | 0.89488 | 0.11045 | 19037 | |
| Cbx7 | 0.89496 | 0.097327 | 19038 | |
| Olfr1320 | 0.895 | 0.1621 | 19039 | |
| Ucp3 | 0.89507 | 1.6846 | 19040 | |
| Fzd7 | 0.89508 | 1.0162 | 19041 | |
| Lurap1 | 0.89515 | 0.56998 | 19042 | |
| Opn5 | 0.89521 | 0.31462 | 19043 | |
| Fzd3 | 0.89524 | 1.2362 | 19044 | |
| Fcrl1 | 0.89525 | 0.59598 | 19045 | |
| Ipcef1 | 0.89531 | 1.2972 | 19046 | |
| Slc25a17 | 0.89543 | 0.498 | 19047 | |
| Golm1 | 0.89544 | 0.90664 | 19048 | |
| Hoxd12 | 0.89545 | 0.11262 | 19049 | |
| Ccdc37 | 0.89547 | 0.40924 | 19050 | |
| Mpst | 0.89552 | 0.10957 | 19051 | |
| Spink12 | 0.89558 | 2.0353 | 19052 | |
| Magea8 | 0.89566 | 0.098221 | 19053 | |
| Pard6g | 0.89573 | 0.1559 | 19054 | |
| Nav2 | 0.89574 | 0.26341 | 19055 | |
| Lrrc43 | 0.89576 | 1.7531 | 19056 | |
| Dhrs3 | 0.89577 | 0.35893 | 19057 | |
| Bcl9 | 0.89582 | -0.23034 | 19058 | |
| Pcmtd1 | 0.89583 | -0.00051811 | 19059 | |
| Clcn7 | 0.89587 | -0.1098 | 19060 | |
| Olfr286 | 0.89591 | 0.34054 | 19061 | |
| Cd9 | 0.89618 | -0.20334 | 19062 | |
| Defb20 | 0.89623 | -0.17939 | 19063 | |
| Tmem205 | 0.89625 | 0.24412 | 19064 | |
| Dnajb14 | 0.89639 | 1.0046 | 19065 | |
| Olfr1141 | 0.89643 | 1.1617 | 19066 | |
| Serpinb9c | 0.8965 | 0.18797 | 19067 | |
| Cyp2a22 | 0.89653 | 0.13525 | 19068 | |
| Tmem62 | 0.89658 | 0.13557 | 19069 | |
| Hsd11b2 | 0.89668 | 1.4233 | 19070 | |
| Btnl10 | 0.8967 | 0.34131 | 19071 | |
| Whamm | 0.8969 | 0.51323 | 19072 | |
| Sap30 | 0.89691 | 0.02682 | 19073 | |
| Rnf26 | 0.89705 | -0.042513 | 19074 | |
| Zfp414 | 0.89711 | 0.73578 | 19075 | |
| Mamstr | 0.89712 | 0.11963 | 19076 | |
| Pira2 | 0.89716 | 0.026021 | 19077 | |
| Cnnm2 | 0.89723 | 0.044058 | 19078 | |
| Gpbp1 | 0.89725 | 0.16262 | 19079 | |
| Gmfg | 0.89727 | 0.97063 | 19080 | |
| Lrrc18 | 0.89728 | 0.047019 | 19081 | |
| Pcdhb8 | 0.89729 | 0.23174 | 19082 | |
| Defb29 | 0.89733 | -0.17003 | 19083 | |
| Abra | 0.89736 | 1.1216 | 19084 | |
| Ccdc135 | 0.89739 | 0.41601 | 19085 | |
| Rnf39 | 0.89743 | 0.38307 | 19086 | |
| mmu-mir-6979 | 0.89748 | 0.8472 | 19087 | |
| Fbxo44 | 0.89749 | -0.17534 | 19088 | |
| Ina | 0.89757 | 0.41533 | 19089 | |
| Sft2d3 | 0.89761 | -0.21591 | 19090 | |
| Fkbp10 | 0.89766 | 0.28695 | 19091 | |
| Scnn1b | 0.89781 | 1.2197 | 19092 | |
| Zfp182 | 0.89787 | -0.078256 | 19093 | |
| Usp42 | 0.89791 | 0.59751 | 19094 | |
| Doc2a | 0.89792 | 2.1237 | 19095 | |
| Ago1 | 0.89799 | 0.15391 | 19096 | |
| Clec4d | 0.89801 | -0.15079 | 19097 | |
| Paox | 0.89805 | 1.0078 | 19098 | |
| Sprr2k | 0.89808 | 1.6393 | 19099 | |
| Glb1l | 0.8981 | 0.66679 | 19100 | |
| Zmynd11 | 0.89811 | 0.018693 | 19101 | |
| Aldh1a1 | 0.89815 | -0.17222 | 19102 | |
| Dynll2 | 0.89818 | -0.19907 | 19103 | |
| Cxcl15 | 0.8982 | 0.07403 | 19104 | |
| Pcdhga1 | 0.8983 | 1.9824 | 19105 | |
| Cd200r4 | 0.89831 | 0.17212 | 19106 | |
| Smcr8 | 0.89833 | 0.87632 | 19107 | |
| Nlrp4a | 0.8984 | 0.018947 | 19108 | |
| Chrm4 | 0.89847 | 0.018779 | 19109 | |
| Rxra | 0.8985 | 1.4542 | 19110 | |
| Gm11127 | 0.89857 | 0.73459 | 19111 | |
| Tmem174 | 0.89861 | 0.62238 | 19112 | |
| Grhpr | 0.89864 | 0.58319 | 19113 | |
| Ccl7 | 0.89867 | 0.01907 | 19114 | |
| Slfn14 | 0.89869 | 1.6331 | 19115 | |
| Lhx3 | 0.8987 | 0.10835 | 19116 | |
| Slc45a3 | 0.89873 | 0.10331 | 19117 | |
| Dnah6 | 0.89874 | 1.1615 | 19118 | |
| Mmp24 | 0.89877 | 0.81187 | 19119 | |
| Ttc25 | 0.8988 | 0.2201 | 19120 | |
| Tatdn2 | 0.89884 | 0.44196 | 19121 | |
| B3galt4 | 0.89888 | 0.062539 | 19122 | |
| Gbgt1 | 0.89891 | 0.14792 | 19123 | |
| Ndufaf7 | 0.89895 | 1.3277 | 19124 | |
| Rab43 | 0.89896 | 0.089477 | 19125 | |
| Ces2g | 0.89898 | 0.041769 | 19126 | |
| Gle1 | 0.89901 | 0.53894 | 19127 | |
| Gm14085 | 0.89908 | 0.021028 | 19128 | |
| Intu | 0.89912 | 0.72499 | 19129 | |
| Brip1 | 0.8992 | -0.24255 | 19130 | |
| Six3 | 0.89921 | 0.11364 | 19131 | |
| Ano10 | 0.89922 | 0.54722 | 19132 | |
| Lgals7 | 0.89923 | 0.26385 | 19133 | |
| Gpx7 | 0.89925 | 0.65479 | 19134 | |
| V1rd18 | 0.89927 | -0.16697 | 19135 | |
| 4930435E12Rik | 0.89928 | 0.59563 | 19136 | |
| Sdc1 | 0.89932 | 0.2287 | 19137 | |
| Mcpt2 | 0.89941 | 0.010047 | 19138 | |
| Ush1g | 0.89942 | 0.33356 | 19139 | |
| Oxct2a | 0.89947 | 0.66889 | 19140 | |
| Zfp90 | 0.89948 | -0.11015 | 19141 | |
| Yeats2 | 0.8995 | 0.24263 | 19142 | |
| Suclg1 | 0.89959 | 0.31128 | 19143 | |
| Tmem252 | 0.8996 | 0.75685 | 19144 | |
| Rlbp1 | 0.89963 | 1.3777 | 19145 | |
| Cxcl14 | 0.89964 | -0.14502 | 19146 | |
| Satl1 | 0.89965 | 1.1515 | 19147 | |
| Enpp6 | 0.89967 | 1.0905 | 19148 | |
| Mip | 0.89981 | 0.91869 | 19149 | |
| Exd2 | 0.89994 | 0.00241 | 19150 | |
| Agbl5 | 0.89995 | 0.46772 | 19151 | |
| Amy2a2 | 0.89999 | 0.48912 | 19152 | |
| Med29 | 0.90004 | -0.16242 | 19153 | |
| mmu-mir-183 | 0.90005 | -0.018818 | 19154 | |
| Gm2002 | 0.90012 | 0.77443 | 19155 | |
| mmu-mir-6383 | 0.90013 | 0.68702 | 19156 | |
| mmu-mir-148b | 0.90014 | 0.72302 | 19157 | |
| Zfp287 | 0.90016 | 0.54243 | 19158 | |
| Zmym6 | 0.90029 | 0.72364 | 19159 | |
| Olfr807 | 0.90033 | 1.1642 | 19160 | |
| mmu-mir-876 | 0.90037 | -0.15765 | 19161 | |
| 1700040L02Rik | 0.90039 | 0.61805 | 19162 | |
| Sh3rf1 | 0.90047 | 1.143 | 19163 | |
| Scoc | 0.9005 | 1.1894 | 19164 | |
| Trnau1ap | 0.90055 | 0.2758 | 19165 | |
| Gm813 | 0.90056 | 1.4876 | 19166 | |
| Vmn2r69 | 0.90065 | 0.034146 | 19167 | |
| Prl7c1 | 0.90067 | 0.56427 | 19168 | |
| Trcg1 | 0.90072 | 0.024511 | 19169 | |
| Gtf3c2 | 0.90074 | 0.19484 | 19170 | |
| Nkx6-2 | 0.90092 | -0.062924 | 19171 | |
| Fkbp9 | 0.90093 | 1.5485 | 19172 | |
| Strip2 | 0.90094 | 0.74041 | 19173 | |
| Thap3 | 0.901 | 0.27047 | 19174 | |
| Gsx1 | 0.90103 | 0.054605 | 19175 | |
| Kctd12 | 0.90108 | 0.68963 | 19176 | |
| Trim41 | 0.9011 | 0.20852 | 19177 | |
| Smtn | 0.90112 | 0.40211 | 19178 | |
| Cdk7 | 0.90113 | 0.89824 | 19179 | |
| Baiap3 | 0.90114 | -0.080208 | 19180 | |
| Grin2d | 0.90115 | 0.068965 | 19181 | |
| Zfp771 | 0.90115 | -0.024795 | 19182 | |
| Stx3 | 0.90118 | -0.18448 | 19183 | |
| Ccr9 | 0.90125 | 0.87102 | 19184 | |
| Mgp | 0.90126 | 0.23766 | 19185 | |
| Ptpre | 0.90133 | -0.076165 | 19186 | |
| Psph | 0.90137 | 1.1632 | 19187 | |
| Fbxw5 | 0.90138 | 0.074977 | 19188 | |
| Pttg1 | 0.90139 | 1.4557 | 19189 | |
| Zfp329 | 0.90144 | -0.10714 | 19190 | |
| Repin1 | 0.90155 | 0.10105 | 19191 | |
| Nt5m | 0.90159 | 0.52944 | 19192 | |
| Slc16a9 | 0.90161 | 0.51207 | 19193 | |
| Anks3 | 0.90163 | 0.41085 | 19194 | |
| Mgat5b | 0.90164 | 0.42489 | 19195 | |
| Il1f5 | 0.90175 | 0.28995 | 19196 | |
| D10Bwg1379e | 0.90177 | 1.0591 | 19197 | |
| Bpifb4 | 0.90194 | -0.15349 | 19198 | |
| Lrrc8b | 0.90199 | 1.4541 | 19199 | |
| Lmf1 | 0.90201 | 0.2319 | 19200 | |
| Adam8 | 0.90202 | -0.27393 | 19201 | |
| Chst10 | 0.90207 | -0.15167 | 19202 | |
| Jph3 | 0.90211 | 0.1759 | 19203 | |
| mmu-mir-6995 | 0.90211 | 0.44225 | 19204 | |
| Rspo4 | 0.90211 | 0.4795 | 19205 | |
| Specc1l | 0.90218 | 2.0685 | 19206 | |
| Sh3gl2 | 0.90226 | 2.4256 | 19207 | |
| Igbp1b | 0.90228 | 0.18868 | 19208 | |
| Far1 | 0.90237 | 0.24917 | 19209 | |
| Ces2h | 0.9024 | 1.6902 | 19210 | |
| Chrna3 | 0.9024 | -0.21102 | 19211 | |
| 4933409G03Rik | 0.90248 | 0.06591 | 19212 | |
| A430033K04Rik | 0.90253 | 2.6844 | 19213 | |
| Qsox1 | 0.90254 | 0.76276 | 19214 | |
| mmu-mir-450a-2 | 0.90259 | 0.34718 | 19215 | |
| Bmp2k | 0.90261 | 0.24372 | 19216 | |
| Nup210l | 0.90266 | -0.080454 | 19217 | |
| Tfr2 | 0.90268 | 0.89839 | 19218 | |
| Phactr1 | 0.9027 | 0.013798 | 19219 | |
| Def6 | 0.90273 | -0.19929 | 19220 | |
| Mal2 | 0.90278 | 0.035147 | 19221 | |
| Pigo | 0.90281 | -0.19633 | 19222 | |
| Exo5 | 0.90283 | 0.22371 | 19223 | |
| Hnrnpa3 | 0.90284 | 0.35411 | 19224 | |
| Slc23a1 | 0.90285 | 1.0316 | 19225 | |
| Ankrd24 | 0.90288 | 0.25406 | 19226 | |
| Vmn2r22 | 0.90295 | 0.15512 | 19227 | |
| Steap3 | 0.90299 | 0.10152 | 19228 | |
| mmu-mir-7036b | 0.903 | 0.20783 | 19229 | |
| Myl2 | 0.90302 | 0.20578 | 19230 | |
| Itm2b | 0.90303 | 0.9566 | 19231 | |
| Ccdc147 | 0.90305 | -0.17774 | 19232 | |
| Ikzf3 | 0.90309 | 0.20598 | 19233 | |
| Zcchc6 | 0.90311 | 0.0996 | 19234 | |
| Spo11 | 0.90323 | 1.9723 | 19235 | |
| Fancb | 0.90325 | -0.047496 | 19236 | |
| Chst5 | 0.90331 | 1.8104 | 19237 | |
| Tmem120b | 0.90332 | 0.23538 | 19238 | |
| Vmn1r209 | 0.90333 | 0.031808 | 19239 | |
| Fam149b | 0.90345 | -0.23046 | 19240 | |
| Olfr288 | 0.90348 | 0.54738 | 19241 | |
| Zfp763 | 0.9036 | -0.21468 | 19242 | |
| Hnrnpul1 | 0.90362 | -0.17016 | 19243 | |
| 2410016O06Rik | 0.90369 | 0.99309 | 19244 | |
| Kalrn | 0.90373 | 0.66432 | 19245 | |
| Igsf5 | 0.90377 | 0.14496 | 19246 | |
| Actn1 | 0.90378 | -0.20012 | 19247 | |
| Olfr137 | 0.9038 | 0.8726 | 19248 | |
| Cst3 | 0.90383 | 0.078166 | 19249 | |
| Ppp5c | 0.90399 | 0.23659 | 19250 | |
| Fasn | 0.90402 | 0.056212 | 19251 | |
| Akap11 | 0.90405 | 0.20546 | 19252 | |
| Nrde2 | 0.90406 | 1.4212 | 19253 | |
| mmu-mir-1938 | 0.90406 | 0.38131 | 19254 | |
| Hoxd4 | 0.90411 | 0.64013 | 19255 | |
| Ihh | 0.90415 | 0.14437 | 19256 | |
| Serf2 | 0.90416 | 0.14266 | 19257 | |
| Acsm5 | 0.90417 | 1.0944 | 19258 | |
| Hephl1 | 0.90421 | 0.58022 | 19259 | |
| Lmo7 | 0.90423 | 2.268 | 19260 | |
| Cacna1e | 0.90431 | -0.03187 | 19261 | |
| Nmur1 | 0.90433 | 0.59633 | 19262 | |
| Sorcs1 | 0.90438 | 0.93796 | 19263 | |
| Gm5415 | 0.90445 | -0.14841 | 19264 | |
| Pygm | 0.90446 | -0.045098 | 19265 | |
| Trim6 | 0.90453 | 2.6245 | 19266 | |
| Fam20a | 0.90463 | 0.22599 | 19267 | |
| Trim8 | 0.9047 | 0.43516 | 19268 | |
| Golph3l | 0.90471 | 0.92342 | 19269 | |
| 1700013F07Rik | 0.90472 | 0.99447 | 19270 | |
| Olfr619 | 0.90473 | 0.754 | 19271 | |
| Zcchc5 | 0.90481 | 0.50909 | 19272 | |
| Plcd1 | 0.90482 | 0.068502 | 19273 | |
| Myot | 0.90486 | 1.764 | 19274 | |
| Gm136 | 0.90487 | 0.31122 | 19275 | |
| Ccdc130 | 0.90488 | 2.5806 | 19276 | |
| BC100530 | 0.90491 | -0.030673 | 19277 | |
| Fam184a | 0.90495 | 0.86768 | 19278 | |
| Sirpa | 0.90502 | 0.042029 | 19279 | |
| Traf5 | 0.90502 | 0.39417 | 19280 | |
| Olfr998 | 0.90506 | 0.46608 | 19281 | |
| mmu-mir-7022 | 0.90506 | 1.9757 | 19282 | |
| Gm6377 | 0.90507 | 0.094716 | 19283 | |
| Olfr577 | 0.90514 | 0.35048 | 19284 | |
| Gpr27 | 0.90518 | 0.39311 | 19285 | |
| Olfr1107 | 0.90525 | 1.3005 | 19286 | |
| 5830403L16Rik | 0.90531 | 0.86205 | 19287 | |
| Tmem198 | 0.90532 | 0.23706 | 19288 | |
| Pcnxl2 | 0.90541 | 0.11934 | 19289 | |
| Cntn4 | 0.90544 | 0.84401 | 19290 | |
| Agmat | 0.90554 | 0.14584 | 19291 | |
| Ccdc36 | 0.90557 | 0.40648 | 19292 | |
| Dok1 | 0.90564 | 1.1628 | 19293 | |
| Fam184b | 0.90565 | 0.26915 | 19294 | |
| Itga2 | 0.90567 | 0.48261 | 19295 | |
| Tmem151a | 0.9058 | -0.17288 | 19296 | |
| Stra6 | 0.90591 | 1.9628 | 19297 | |
| Hsph1 | 0.90601 | 0.43273 | 19298 | |
| Tango2 | 0.90605 | 0.9772 | 19299 | |
| Olfr477 | 0.90606 | 0.043437 | 19300 | |
| Vkorc1 | 0.9061 | 0.53817 | 19301 | |
| Ddrgk1 | 0.90619 | 0.81458 | 19302 | |
| Tmx1 | 0.90624 | 0.20131 | 19303 | |
| Gabrr3 | 0.90627 | 0.028147 | 19304 | |
| Grhl1 | 0.9063 | -0.12387 | 19305 | |
| Mylpf | 0.90634 | 0.52222 | 19306 | |
| Adra1b | 0.90634 | 0.2272 | 19307 | |
| Tmem219 | 0.90641 | 0.49934 | 19308 | |
| Chchd5 | 0.90646 | -0.20914 | 19309 | |
| Slc35a3 | 0.90654 | 0.92446 | 19310 | |
| Dpp6 | 0.90655 | 1.1243 | 19311 | |
| Tmem176b | 0.90656 | 1.1731 | 19312 | |
| Cyld | 0.90657 | 0.1864 | 19313 | |
| Zfp810 | 0.90659 | 0.41964 | 19314 | |
| Tmem55a | 0.90666 | -0.22665 | 19315 | |
| Six2 | 0.90668 | 0.18341 | 19316 | |
| 2310030G06Rik | 0.9067 | -0.19023 | 19317 | |
| Fmo4 | 0.90672 | -0.012757 | 19318 | |
| Mrc1 | 0.90673 | 1.7929 | 19319 | |
| Gm12169 | 0.90675 | 0.39335 | 19320 | |
| Itsn1 | 0.90676 | 0.65606 | 19321 | |
| Gns | 0.90682 | 0.12758 | 19322 | |
| Ddx11 | 0.90688 | 0.63266 | 19323 | |
| Cdc42bpa | 0.90693 | 0.53373 | 19324 | |
| Fkbp1b | 0.90701 | -0.018431 | 19325 | |
| 4931428F04Rik | 0.90702 | 0.074102 | 19326 | |
| Baz2a | 0.90705 | 0.044214 | 19327 | |
| Capn2 | 0.90707 | 0.61194 | 19328 | |
| Slc25a4 | 0.90709 | 1.368 | 19329 | |
| Abcd1 | 0.9071 | -0.032081 | 19330 | |
| Cib3 | 0.90715 | 0.75603 | 19331 | |
| Ado | 0.90719 | 0.24906 | 19332 | |
| Cyp11b2 | 0.9072 | 0.38567 | 19333 | |
| 4930402K13Rik | 0.90722 | 1.0638 | 19334 | |
| Gm6537 | 0.90723 | 0.11826 | 19335 | |
| Slc31a1 | 0.90726 | -0.065212 | 19336 | |
| Plcb4 | 0.90727 | 0.79175 | 19337 | |
| Tsga13 | 0.90733 | 0.66005 | 19338 | |
| Kcns2 | 0.90737 | 0.42618 | 19339 | |
| Vmn2r10 | 0.90739 | 0.86447 | 19340 | |
| Clcf1 | 0.90742 | -0.053138 | 19341 | |
| Flt3l | 0.90743 | 1.0096 | 19342 | |
| Sesn1 | 0.90744 | 0.031196 | 19343 | |
| Gm5141 | 0.90746 | 0.10004 | 19344 | |
| Ccdc178 | 0.90752 | 2.2184 | 19345 | |
| Rnf166 | 0.90753 | 0.12385 | 19346 | |
| Adrb1 | 0.90776 | -0.059 | 19347 | |
| Dyrk3 | 0.90777 | 0.5733 | 19348 | |
| Krt28 | 0.90779 | 0.072919 | 19349 | |
| Ttc12 | 0.90791 | 0.063749 | 19350 | |
| Slc18a3 | 0.90791 | -0.081003 | 19351 | |
| Olfr632 | 0.90808 | 0.81109 | 19352 | |
| Ndufa7 | 0.9081 | 0.68706 | 19353 | |
| Srcrb4d | 0.90814 | 1.3258 | 19354 | |
| Rnpepl1 | 0.90818 | 0.64995 | 19355 | |
| mmu-mir-7024 | 0.90823 | 0.053262 | 19356 | |
| Dgkh | 0.90833 | -0.15586 | 19357 | |
| Olfr1138 | 0.90839 | -0.11269 | 19358 | |
| Coro1a | 0.9084 | -0.0010383 | 19359 | |
| Tmem231 | 0.90847 | 0.19077 | 19360 | |
| Tlx3 | 0.90852 | 0.16391 | 19361 | |
| Gm4906 | 0.90864 | 1.6774 | 19362 | |
| Prm2 | 0.90867 | 0.58266 | 19363 | |
| Ncoa3 | 0.90871 | -0.0051927 | 19364 | |
| Ddo | 0.90881 | 0.47994 | 19365 | |
| Plxnb2 | 0.90883 | 0.010985 | 19366 | |
| Hnrnpm | 0.90893 | 0.60956 | 19367 | |
| Scin | 0.90899 | 0.21792 | 19368 | |
| Msr1 | 0.909 | 0.80689 | 19369 | |
| Samd10 | 0.90901 | 0.55243 | 19370 | |
| Ube2r2 | 0.90909 | 0.62273 | 19371 | |
| Ptges2 | 0.90909 | 0.30671 | 19372 | |
| Adck1 | 0.90919 | 0.045214 | 19373 | |
| Thsd7b | 0.90923 | 0.29841 | 19374 | |
| Hbb-bs | 0.90924 | 0.066348 | 19375 | |
| Malt1 | 0.90925 | -0.031208 | 19376 | |
| Arx | 0.90939 | 1.4944 | 19377 | |
| Crisp1 | 0.90968 | 1.5933 | 19378 | |
| Cds1 | 0.90971 | 0.22015 | 19379 | |
| Jmjd1c | 0.90977 | 0.50871 | 19380 | |
| Vasn | 0.90982 | -0.084346 | 19381 | |
| Rnase12 | 0.90999 | 0.47477 | 19382 | |
| Nol3 | 0.91003 | -0.18795 | 19383 | |
| Olfr1313 | 0.91007 | 1.2913 | 19384 | |
| Kdm4a | 0.9101 | 0.29187 | 19385 | |
| 2300003K06Rik | 0.91017 | -0.12408 | 19386 | |
| Olfr1394 | 0.91022 | 0.96657 | 19387 | |
| Cyp26a1 | 0.91024 | 0.65449 | 19388 | |
| Kifc5b | 0.91029 | -0.080978 | 19389 | |
| Dlg5 | 0.91033 | 0.35124 | 19390 | |
| mmu-mir-6415 | 0.91038 | 0.93693 | 19391 | |
| Ctcfl | 0.91043 | 1.6625 | 19392 | |
| Clu | 0.91051 | 0.2203 | 19393 | |
| Arhgef33 | 0.91056 | 0.3801 | 19394 | |
| Zfp383 | 0.91061 | 0.15003 | 19395 | |
| Vopp1 | 0.91062 | -0.026431 | 19396 | |
| Pax9 | 0.91067 | -0.15032 | 19397 | |
| Kcnk16 | 0.91072 | 0.65456 | 19398 | |
| mmu-mir-467a-1 | 0.91075 | 0.85681 | 19399 | |
| Ccdc117 | 0.91082 | -0.014968 | 19400 | |
| Taf13 | 0.91086 | 0.085684 | 19401 | |
| Gm4951 | 0.91092 | -0.036784 | 19402 | |
| Retsat | 0.91097 | 0.17805 | 19403 | |
| A430105I19Rik | 0.911 | 0.54546 | 19404 | |
| Ell3 | 0.91101 | 0.074152 | 19405 | |
| Mzf1 | 0.91102 | 1.2143 | 19406 | |
| Plxna1 | 0.9111 | 0.66518 | 19407 | |
| Zkscan6 | 0.91112 | 0.266 | 19408 | |
| AY074887 | 0.91125 | 1.0853 | 19409 | |
| Phf2 | 0.91145 | 0.94741 | 19410 | |
| Arrdc2 | 0.91146 | 0.32308 | 19411 | |
| Tmem128 | 0.91147 | -0.09582 | 19412 | |
| Mustn1 | 0.91149 | 0.015278 | 19413 | |
| Olfr1484 | 0.91152 | -0.09298 | 19414 | |
| Olfr535 | 0.91163 | 0.27008 | 19415 | |
| Gjc2 | 0.91166 | 0.44102 | 19416 | |
| Actl7a | 0.91173 | 0.65851 | 19417 | |
| Klf13 | 0.91174 | 0.57664 | 19418 | |
| mmu-mir-873b | 0.91176 | -0.37669 | 19419 | |
| Tmbim7 | 0.91183 | 0.6794 | 19420 | |
| Usp31 | 0.91185 | 1.1156 | 19421 | |
| Olfr1217 | 0.91187 | 0.17345 | 19422 | |
| Marveld2 | 0.91189 | 1.6242 | 19423 | |
| H2-T22 | 0.9119 | 0.38721 | 19424 | |
| 4933422H20Rik | 0.91193 | -0.13635 | 19425 | |
| Enpp2 | 0.91199 | 0.88829 | 19426 | |
| Rbm48 | 0.91201 | 0.91783 | 19427 | |
| Mob3b | 0.91207 | 0.57865 | 19428 | |
| Fam209 | 0.91211 | 0.97394 | 19429 | |
| Serinc2 | 0.91237 | 1.9686 | 19430 | |
| Trim17 | 0.91238 | 0.81187 | 19431 | |
| Papss2 | 0.9124 | 0.18452 | 19432 | |
| Nobox | 0.91242 | 0.04678 | 19433 | |
| Olfr1491 | 0.91244 | 1.7995 | 19434 | |
| Bmp7 | 0.91249 | 0.10148 | 19435 | |
| Abcc9 | 0.91251 | 0.29372 | 19436 | |
| Gm15293 | 0.91258 | 0.47057 | 19437 | |
| Idnk | 0.91261 | -0.071333 | 19438 | |
| Shcbp1 | 0.91265 | 0.12736 | 19439 | |
| Ngfrap1 | 0.91266 | 0.84313 | 19440 | |
| Garem | 0.91268 | 0.28707 | 19441 | |
| Entpd5 | 0.91277 | -0.17036 | 19442 | |
| Ccbl1 | 0.91279 | 3.0384 | 19443 | |
| Kmt2a | 0.91282 | 1.1516 | 19444 | |
| H2-Aa | 0.91285 | 1.7954 | 19445 | |
| Cacng8 | 0.91286 | 0.30208 | 19446 | |
| mmu-mir-6921 | 0.91288 | -0.13057 | 19447 | |
| Nfam1 | 0.91288 | 1.367 | 19448 | |
| Ctps2 | 0.91291 | 0.087401 | 19449 | |
| 5730559C18Rik | 0.91292 | 2.3093 | 19450 | |
| Osbpl3 | 0.91295 | 0.10743 | 19451 | |
| Tnnt1 | 0.91297 | 0.35717 | 19452 | |
| Grik5 | 0.91298 | 0.76748 | 19453 | |
| Atp6v1b2 | 0.913 | -0.2181 | 19454 | |
| Smg7 | 0.9131 | -0.18545 | 19455 | |
| Stap1 | 0.91314 | 0.067103 | 19456 | |
| Zbtb46 | 0.91316 | -0.014561 | 19457 | |
| mmu-mir-5623 | 0.91318 | 1.3066 | 19458 | |
| Slc40a1 | 0.9132 | 1.0873 | 19459 | |
| mmu-mir-6417 | 0.91322 | -0.038549 | 19460 | |
| Ctsw | 0.91325 | 0.63656 | 19461 | |
| Cers2 | 0.91328 | 3.3683 | 19462 | |
| Olfr1053 | 0.91349 | 0.48486 | 19463 | |
| Spryd4 | 0.9135 | 0.40757 | 19464 | |
| Gcnt7 | 0.91354 | 0.45414 | 19465 | |
| Sprr2i | 0.91369 | 0.087679 | 19466 | |
| Hdc | 0.91369 | 0.0079264 | 19467 | |
| Grxcr1 | 0.91375 | 0.11981 | 19468 | |
| P2rx4 | 0.91377 | 0.81611 | 19469 | |
| Fuz | 0.9138 | 2.0129 | 19470 | |
| BC080695 | 0.91382 | -0.15869 | 19471 | |
| Parp2 | 0.91383 | 0.28788 | 19472 | |
| Ntrk2 | 0.91385 | 2.1776 | 19473 | |
| Cyb5rl | 0.91388 | 2.3565 | 19474 | |
| C1qc | 0.9139 | 0.51362 | 19475 | |
| Man2a1 | 0.91391 | 2.2994 | 19476 | |
| Rabl2 | 0.91396 | 0.12669 | 19477 | |
| Capn13 | 0.91399 | 0.16549 | 19478 | |
| Lipn | 0.91401 | 0.33925 | 19479 | |
| mmu-mir-188 | 0.91402 | 0.406 | 19480 | |
| Otof | 0.91409 | 0.94976 | 19481 | |
| Arpp19 | 0.91414 | 0.5975 | 19482 | |
| Olfr1490 | 0.91429 | 0.0013966 | 19483 | |
| 1500012F01Rik | 0.91432 | 0.38114 | 19484 | |
| Ms4a2 | 0.91434 | 2.2567 | 19485 | |
| Zfp618 | 0.91444 | 0.035848 | 19486 | |
| Sox2 | 0.91453 | 0.63813 | 19487 | |
| B3galt1 | 0.91469 | 0.22511 | 19488 | |
| Map2k5 | 0.91472 | 0.13569 | 19489 | |
| Taf5l | 0.91474 | 0.014647 | 19490 | |
| Heatr5a | 0.91483 | 0.46725 | 19491 | |
| Ctdspl | 0.91488 | 0.14982 | 19492 | |
| Thap7 | 0.91492 | 0.023942 | 19493 | |
| Stk19 | 0.91503 | 0.79402 | 19494 | |
| Eva1b | 0.91504 | 0.094177 | 19495 | |
| Taar2 | 0.91505 | 0.064087 | 19496 | |
| Fgf4 | 0.91516 | 0.99405 | 19497 | |
| Rnf141 | 0.91525 | -0.01505 | 19498 | |
| Cacna1b | 0.91526 | 1.5134 | 19499 | |
| Fads2 | 0.9153 | 0.15934 | 19500 | |
| Olfr629 | 0.91533 | -0.0014288 | 19501 | |
| AI661453 | 0.91536 | 0.30203 | 19502 | |
| Gm973 | 0.91541 | -0.109 | 19503 | |
| Phf10 | 0.91547 | 0.019351 | 19504 | |
| Wdtc1 | 0.91548 | 0.23897 | 19505 | |
| mmu-mir-5710 | 0.91548 | 0.75551 | 19506 | |
| Slbp | 0.91553 | 0.084405 | 19507 | |
| Aven | 0.91557 | 0.16821 | 19508 | |
| Ear11 | 0.91568 | 0.53024 | 19509 | |
| Serpinb1c | 0.91576 | 0.069173 | 19510 | |
| Emilin2 | 0.91579 | 2.2866 | 19511 | |
| Twist1 | 0.91582 | -0.116 | 19512 | |
| Asb9 | 0.91583 | 0.35671 | 19513 | |
| Dnal1 | 0.91584 | 0.037652 | 19514 | |
| Gm1673 | 0.91589 | 1.0332 | 19515 | |
| Tas2r114 | 0.91592 | 0.26368 | 19516 | |
| Ccdc12 | 0.91594 | 0.16824 | 19517 | |
| Cadps2 | 0.91596 | 0.12723 | 19518 | |
| Cxcl13 | 0.91596 | 1.0052 | 19519 | |
| Actr3 | 0.91601 | -0.14177 | 19520 | |
| Krtap4-16 | 0.91602 | 1.481 | 19521 | |
| Lgalsl | 0.91603 | 2.3673 | 19522 | |
| A130010J15Rik | 0.91607 | -0.10869 | 19523 | |
| Gpat2 | 0.91613 | 0.086986 | 19524 | |
| Plekhj1 | 0.91616 | 2.0399 | 19525 | |
| Gm4832 | 0.91621 | 0.77037 | 19526 | |
| Prkd3 | 0.91623 | -0.032099 | 19527 | |
| Tmem64 | 0.91625 | 0.15815 | 19528 | |
| Flywch1 | 0.9163 | 0.025983 | 19529 | |
| Inpp5j | 0.91646 | 0.56082 | 19530 | |
| Tmem204 | 0.91648 | 0.12707 | 19531 | |
| Zfhx2 | 0.91654 | -0.080367 | 19532 | |
| Colec12 | 0.9166 | 0.10439 | 19533 | |
| 4931417E11Rik | 0.91663 | 1.5624 | 19534 | |
| Snx20 | 0.91665 | 0.33267 | 19535 | |
| Vasp | 0.91667 | 0.65292 | 19536 | |
| Olfr284 | 0.91669 | 0.092273 | 19537 | |
| Tlr2 | 0.9167 | 0.18348 | 19538 | |
| Lrrc14b | 0.91674 | 0.349 | 19539 | |
| Nphs2 | 0.91677 | 0.76167 | 19540 | |
| mmu-mir-504 | 0.91679 | 1.1099 | 19541 | |
| Fam98c | 0.91681 | -0.066529 | 19542 | |
| Noxred1 | 0.91683 | 0.5843 | 19543 | |
| mmu-mir-25 | 0.91688 | -0.040231 | 19544 | |
| Tlk1 | 0.91698 | 1.4777 | 19545 | |
| Spon2 | 0.91699 | 0.67743 | 19546 | |
| Yipf2 | 0.917 | 0.68212 | 19547 | |
| 4930579G24Rik | 0.91704 | 0.2082 | 19548 | |
| mmu-mir-194-1 | 0.91708 | 0.26158 | 19549 | |
| Gm6878 | 0.9171 | 0.80031 | 19550 | |
| Sez6 | 0.91719 | 0.012943 | 19551 | |
| Nrg2 | 0.91721 | 0.78319 | 19552 | |
| Dlx3 | 0.91725 | 0.33093 | 19553 | |
| Olfr730 | 0.91726 | -0.053061 | 19554 | |
| Pax4 | 0.91727 | -0.12796 | 19555 | |
| Sox11 | 0.91729 | -0.017838 | 19556 | |
| Anks1b | 0.91732 | 0.35539 | 19557 | |
| Cuedc1 | 0.91741 | -0.073118 | 19558 | |
| Agpat1 | 0.91744 | 2.0619 | 19559 | |
| Crtam | 0.91746 | 0.66413 | 19560 | |
| Fam186b | 0.9176 | 1.5893 | 19561 | |
| Olfr1457 | 0.91761 | 0.64162 | 19562 | |
| Usp17ld | 0.91772 | 0.34358 | 19563 | |
| Tbx21 | 0.91773 | 0.098836 | 19564 | |
| Sft2d1 | 0.91779 | 0.15051 | 19565 | |
| Zbtb10 | 0.91782 | 1.2536 | 19566 | |
| Cdkn2c | 0.91786 | 0.64321 | 19567 | |
| AI593442 | 0.91787 | 0.053357 | 19568 | |
| Apoa1bp | 0.91788 | 0.0077431 | 19569 | |
| Ovol3 | 0.9179 | 0.080299 | 19570 | |
| Sugp2 | 0.91804 | -0.072106 | 19571 | |
| Gm20809 | 0.91806 | 0.3773 | 19572 | |
| Bche | 0.91807 | 0.34477 | 19573 | |
| Igsf21 | 0.9181 | 0.76915 | 19574 | |
| Ifna6 | 0.91813 | 0.655 | 19575 | |
| Pou2af1 | 0.91815 | 1.6835 | 19576 | |
| Mrgprx1 | 0.91815 | 0.040056 | 19577 | |
| Rasal1 | 0.91825 | 0.6997 | 19578 | |
| Cma1 | 0.91826 | 1.3494 | 19579 | |
| Meig1 | 0.91846 | 0.42227 | 19580 | |
| Adad2 | 0.91861 | 0.51482 | 19581 | |
| Mef2b | 0.91863 | 0.87744 | 19582 | |
| Cdh3 | 0.91869 | 0.49648 | 19583 | |
| AI846148 | 0.91877 | -0.023252 | 19584 | |
| Strn4 | 0.91878 | 2.3307 | 19585 | |
| Bai1 | 0.91885 | 0.04596 | 19586 | |
| Olfr1209 | 0.91886 | 0.52683 | 19587 | |
| mmu-mir-762 | 0.91894 | 0.27515 | 19588 | |
| Retnlb | 0.91895 | 0.63556 | 19589 | |
| Olfr374 | 0.91903 | -0.05508 | 19590 | |
| mmu-mir-1a-1 | 0.91903 | 1.0361 | 19591 | |
| Klk12 | 0.91904 | 0.91511 | 19592 | |
| Lrrc32 | 0.91907 | 0.35242 | 19593 | |
| Zg16 | 0.91912 | 0.71197 | 19594 | |
| Ugt1a10 | 0.91927 | 0.12133 | 19595 | |
| Stpg1 | 0.91931 | 0.38051 | 19596 | |
| Cst13 | 0.91939 | 0.22297 | 19597 | |
| Mettl20 | 0.9194 | 0.33936 | 19598 | |
| Gpx6 | 0.91943 | 0.40681 | 19599 | |
| mmu-mir-3572 | 0.91945 | 1.1952 | 19600 | |
| Mettl5 | 0.91949 | 0.32044 | 19601 | |
| Clca1 | 0.91956 | 0.61442 | 19602 | |
| Ssr2 | 0.91958 | 0.84463 | 19603 | |
| Tas2r138 | 0.91964 | 0.21161 | 19604 | |
| Hmgn5 | 0.9197 | 0.073302 | 19605 | |
| H2afb1 | 0.91977 | 0.0014808 | 19606 | |
| Fem1c | 0.91979 | 0.2255 | 19607 | |
| Klra12 | 0.9198 | 0.17038 | 19608 | |
| Dusp5 | 0.91983 | 2.105 | 19609 | |
| mmu-mir-3070a | 0.91986 | 0.33019 | 19610 | |
| Vps13b | 0.91988 | 1.2126 | 19611 | |
| Lax1 | 0.91992 | -0.029949 | 19612 | |
| Vmn1r206 | 0.92004 | 0.1102 | 19613 | |
| Olfr545 | 0.92007 | 1.774 | 19614 | |
| Pcdhb11 | 0.9201 | 0.31322 | 19615 | |
| Eml1 | 0.92015 | 0.76954 | 19616 | |
| Snap25 | 0.92016 | 0.38752 | 19617 | |
| Parp3 | 0.92017 | 0.74201 | 19618 | |
| Cep112 | 0.92018 | 0.3734 | 19619 | |
| Fgfr3 | 0.92024 | 0.76077 | 19620 | |
| Usp1 | 0.92026 | 0.21821 | 19621 | |
| Gm1527 | 0.92028 | 0.45599 | 19622 | |
| Slc22a12 | 0.92029 | -0.079498 | 19623 | |
| Jmjd4 | 0.92035 | 0.24203 | 19624 | |
| Mppe1 | 0.92043 | 1.0783 | 19625 | |
| Foxl1 | 0.92045 | -0.07318 | 19626 | |
| Olfr938 | 0.92047 | 0.26751 | 19627 | |
| Kcns1 | 0.92049 | 0.44173 | 19628 | |
| Rbbp8nl | 0.9206 | -0.11824 | 19629 | |
| Pygb | 0.92063 | 1.0994 | 19630 | |
| Pah | 0.92064 | 0.22118 | 19631 | |
| Pvrl4 | 0.92065 | 0.0013127 | 19632 | |
| Cxx1a | 0.92078 | 0.22926 | 19633 | |
| Scn4a | 0.9208 | 0.46476 | 19634 | |
| 4430402I18Rik | 0.92094 | 1.4093 | 19635 | |
| Pou6f2 | 0.92097 | 0.047528 | 19636 | |
| Adnp2 | 0.921 | 0.39196 | 19637 | |
| Pcdhb16 | 0.921 | 0.15711 | 19638 | |
| Nox3 | 0.92105 | -0.091832 | 19639 | |
| mmu-mir-449a | 0.92105 | 0.0040333 | 19640 | |
| Glis2 | 0.92115 | 0.14716 | 19641 | |
| Asxl2 | 0.92118 | 1.8043 | 19642 | |
| Ttll12 | 0.92122 | 0.79658 | 19643 | |
| mmu-mir-6949 | 0.92123 | 0.31489 | 19644 | |
| Gm5617 | 0.92125 | 1.5787 | 19645 | |
| Tbc1d8 | 0.9213 | -0.071958 | 19646 | |
| Erbb4 | 0.92131 | 0.08407 | 19647 | |
| Vps13a | 0.92132 | 0.22502 | 19648 | |
| Pde6b | 0.92136 | 0.42689 | 19649 | |
| Man2a2 | 0.92145 | 0.27614 | 19650 | |
| Olfr426 | 0.92149 | 0.30894 | 19651 | |
| Pdf | 0.92156 | 0.44544 | 19652 | |
| Zfp275 | 0.92157 | 0.50112 | 19653 | |
| 4930564C03Rik | 0.92157 | 0.51204 | 19654 | |
| Mgat2 | 0.92161 | 0.69975 | 19655 | |
| Ypel3 | 0.92164 | 0.94905 | 19656 | |
| Avpi1 | 0.92165 | 0.54529 | 19657 | |
| Cd22 | 0.92166 | 0.85274 | 19658 | |
| Phox2b | 0.92173 | 0.0065533 | 19659 | |
| Emc9 | 0.92174 | 0.068584 | 19660 | |
| Foxf2 | 0.92181 | 0.41187 | 19661 | |
| Chrna9 | 0.92185 | 0.5961 | 19662 | |
| Scn5a | 0.92189 | 0.8027 | 19663 | |
| N4bp2l2 | 0.92194 | 0.37727 | 19664 | |
| Epha8 | 0.92204 | 0.039949 | 19665 | |
| mmu-mir-7034 | 0.92206 | 1.139 | 19666 | |
| mmu-mir-6896 | 0.9221 | 0.10396 | 19667 | |
| Soga3 | 0.92211 | 0.56478 | 19668 | |
| Ifna11 | 0.92216 | 0.65519 | 19669 | |
| mmu-mir-7006 | 0.92224 | -0.10295 | 19670 | |
| Olfr2 | 0.92225 | 0.15041 | 19671 | |
| Prss58 | 0.9223 | 0.38903 | 19672 | |
| Bglap | 0.92231 | 0.20699 | 19673 | |
| Vps9d1 | 0.92235 | 0.085941 | 19674 | |
| Wwp1 | 0.92244 | 1.2553 | 19675 | |
| Id1 | 0.92258 | 0.34354 | 19676 | |
| Naa11 | 0.9227 | 0.12569 | 19677 | |
| Chkb | 0.92274 | 0.061342 | 19678 | |
| Slamf8 | 0.92275 | 1.7482 | 19679 | |
| Stmn3 | 0.92281 | 0.43437 | 19680 | |
| Hoxb2 | 0.92282 | 0.11272 | 19681 | |
| Egr2 | 0.92282 | 0.43681 | 19682 | |
| Olfr544 | 0.92292 | 0.89435 | 19683 | |
| Kera | 0.92298 | 1.6216 | 19684 | |
| Phex | 0.92301 | 2.2557 | 19685 | |
| Ch25h | 0.92306 | 0.011784 | 19686 | |
| Rap2a | 0.92307 | 0.87623 | 19687 | |
| 2310007L24Rik | 0.92315 | 0.52007 | 19688 | |
| Akt2 | 0.92316 | 1.2458 | 19689 | |
| Crnn | 0.92324 | 0.59182 | 19690 | |
| Zbtb7a | 0.92328 | 0.34103 | 19691 | |
| Zfp423 | 0.92334 | 0.43895 | 19692 | |
| Reep2 | 0.92341 | 0.24416 | 19693 | |
| Lpar2 | 0.92345 | -0.020641 | 19694 | |
| Pilrb1 | 0.92348 | 0.17362 | 19695 | |
| Foxo6 | 0.92353 | 0.91765 | 19696 | |
| Arntl | 0.92356 | 0.67036 | 19697 | |
| Haao | 0.9236 | 0.27732 | 19698 | |
| Fbxo33 | 0.92361 | 1.238 | 19699 | |
| Smpd5 | 0.92365 | 1.2231 | 19700 | |
| Chrd | 0.92368 | 0.043163 | 19701 | |
| Galnt1 | 0.92378 | 0.73559 | 19702 | |
| BC021785 | 0.92379 | 0.70525 | 19703 | |
| Olfr215 | 0.9238 | 0.79988 | 19704 | |
| Olfr401 | 0.92397 | 1.145 | 19705 | |
| Lca5l | 0.92398 | 0.6462 | 19706 | |
| Olfr480 | 0.92404 | 0.067919 | 19707 | |
| Prdx1 | 0.92408 | 1.0105 | 19708 | |
| Bex1 | 0.92414 | 0.33362 | 19709 | |
| Plbd2 | 0.92415 | 0.51305 | 19710 | |
| Got1l1 | 0.92417 | 0.010217 | 19711 | |
| Gm10256 | 0.92419 | 1.3985 | 19712 | |
| Dcdc2c | 0.9242 | 0.77889 | 19713 | |
| Il27ra | 0.92422 | 0.295 | 19714 | |
| Ndufaf2 | 0.92428 | 0.010587 | 19715 | |
| Olfr801 | 0.92429 | 0.095358 | 19716 | |
| Ear14 | 0.9243 | 0.55112 | 19717 | |
| Dyrk1b | 0.92433 | 0.17986 | 19718 | |
| Tsc22d4 | 0.92441 | 0.3161 | 19719 | |
| mmu-mir-341 | 0.92451 | 0.44976 | 19720 | |
| Rab40c | 0.92473 | 0.39806 | 19721 | |
| Atp4b | 0.92475 | 1.5243 | 19722 | |
| Fam173a | 0.92476 | 1.2198 | 19723 | |
| Zcchc12 | 0.92487 | 0.22938 | 19724 | |
| Slc12a6 | 0.92488 | -0.018813 | 19725 | |
| mmu-mir-7021 | 0.92488 | 0.99959 | 19726 | |
| Mn1 | 0.92493 | -0.043283 | 19727 | |
| Wfdc5 | 0.92494 | 0.53851 | 19728 | |
| Ctns | 0.92497 | 1.4745 | 19729 | |
| Nlrx1 | 0.9251 | 0.37491 | 19730 | |
| Dusp6 | 0.92512 | 0.2504 | 19731 | |
| Tmem170 | 0.92517 | 0.4303 | 19732 | |
| Lcn2 | 0.92519 | 0.573 | 19733 | |
| Cbx6 | 0.92523 | 1.1413 | 19734 | |
| Smyd2 | 0.92527 | -0.041479 | 19735 | |
| Kitl | 0.92527 | 0.73119 | 19736 | |
| Map4k2 | 0.92529 | 0.54249 | 19737 | |
| Zfp410 | 0.92531 | 1.367 | 19738 | |
| Mrrf | 0.92532 | 0.1708 | 19739 | |
| Cd19 | 0.92539 | 1.0061 | 19740 | |
| Zc3h4 | 0.92547 | 0.052142 | 19741 | |
| Armc12 | 0.92548 | 0.63779 | 19742 | |
| Rps6kc1 | 0.92553 | 0.070166 | 19743 | |
| Hectd1 | 0.92554 | 0.2002 | 19744 | |
| 9130008F23Rik | 0.92556 | 0.20667 | 19745 | |
| Chst7 | 0.92565 | 0.50551 | 19746 | |
| Crygn | 0.92568 | 1.1404 | 19747 | |
| Ftmt | 0.92568 | 1.3248 | 19748 | |
| Snta1 | 0.92572 | 0.18017 | 19749 | |
| Ogg1 | 0.92576 | 0.23358 | 19750 | |
| Vmn2r84 | 0.92585 | 1.2046 | 19751 | |
| Sall2 | 0.92586 | 0.7137 | 19752 | |
| Krt33b | 0.92588 | 0.52532 | 19753 | |
| Pld5 | 0.92591 | 0.17979 | 19754 | |
| Prlhr | 0.92603 | 1.8333 | 19755 | |
| Stx1b | 0.92609 | 0.89205 | 19756 | |
| Npl | 0.92623 | 0.40659 | 19757 | |
| Olfr1270 | 0.92623 | 0.010543 | 19758 | |
| Glt1d1 | 0.92632 | 0.11537 | 19759 | |
| Cyp7a1 | 0.92634 | 0.89639 | 19760 | |
| Cdh10 | 0.92639 | 0.33061 | 19761 | |
| Paqr9 | 0.92644 | 0.13669 | 19762 | |
| Ifnl2 | 0.92646 | 0.058116 | 19763 | |
| Dscam | 0.92651 | 0.81643 | 19764 | |
| Arid1a | 0.92656 | 1.9207 | 19765 | |
| Parpbp | 0.9266 | 0.0025366 | 19766 | |
| Hes6 | 0.92664 | 0.71225 | 19767 | |
| Gm11595 | 0.92673 | 0.15273 | 19768 | |
| Rbmy | 0.92673 | 1.3016 | 19769 | |
| Cdca4 | 0.92676 | 0.45418 | 19770 | |
| Pqlc1 | 0.92679 | -0.031326 | 19771 | |
| Tfap2d | 0.92684 | 0.59498 | 19772 | |
| Clic1 | 0.92689 | 0.47345 | 19773 | |
| Ccser2 | 0.92696 | 0.19343 | 19774 | |
| Tmem200c | 0.92696 | 1.1087 | 19775 | |
| Rec8 | 0.92699 | 0.9619 | 19776 | |
| Tmem253 | 0.92701 | 0.54558 | 19777 | |
| mmu-mir-181c | 0.92704 | 0.58526 | 19778 | |
| Asap1 | 0.92704 | -0.031203 | 19779 | |
| Stra13 | 0.92708 | 0.14379 | 19780 | |
| Noxa1 | 0.92708 | 0.28849 | 19781 | |
| mmu-mir-1945 | 0.9271 | 0.26188 | 19782 | |
| Zfp105 | 0.92717 | 0.16509 | 19783 | |
| mmu-mir-5131 | 0.92722 | 0.021041 | 19784 | |
| Rcsd1 | 0.92725 | 0.1156 | 19785 | |
| Olfr1186 | 0.92729 | 1.3215 | 19786 | |
| Clec4b2 | 0.92741 | 0.25336 | 19787 | |
| Prkd1 | 0.92742 | 0.28993 | 19788 | |
| Cul4b | 0.92747 | 0.65747 | 19789 | |
| Prss21 | 0.92753 | 0.96583 | 19790 | |
| Crh | 0.92757 | -0.011852 | 19791 | |
| 1600012H06Rik | 0.9276 | 0.14337 | 19792 | |
| Sprr2b | 0.92762 | 0.62671 | 19793 | |
| Svil | 0.92767 | 0.27504 | 19794 | |
| Rgs8 | 0.92769 | 0.67367 | 19795 | |
| Eid2 | 0.92771 | 1.4199 | 19796 | |
| Il1f6 | 0.92779 | -0.020151 | 19797 | |
| Rhox7 | 0.9278 | 1.07 | 19798 | |
| Itpripl2 | 0.92788 | 0.39137 | 19799 | |
| A3galt2 | 0.92793 | 0.3538 | 19800 | |
| 1700022I11Rik | 0.92797 | 0.17299 | 19801 | |
| Llgl1 | 0.92799 | 0.45144 | 19802 | |
| Fam126b | 0.92803 | 0.74522 | 19803 | |
| Bloc1s3 | 0.92806 | 0.84833 | 19804 | |
| Agtr2 | 0.92835 | -0.0014293 | 19805 | |
| Hmg20a | 0.92835 | 0.26924 | 19806 | |
| Serpina12 | 0.92843 | 0.95319 | 19807 | |
| Pnpla1 | 0.92845 | 0.3278 | 19808 | |
| Aif1 | 0.92847 | -0.063679 | 19809 | |
| Cd14 | 0.92851 | 0.11681 | 19810 | |
| mmu-mir-1905 | 0.92857 | 0.21589 | 19811 | |
| Mapk13 | 0.92857 | 0.090594 | 19812 | |
| Tmbim6 | 0.92859 | 0.17528 | 19813 | |
| Pcsk1 | 0.92863 | 0.57257 | 19814 | |
| Msn | 0.92868 | 0.57614 | 19815 | |
| Cggbp1 | 0.92868 | 1.4134 | 19816 | |
| Zfp608 | 0.92882 | 0.19779 | 19817 | |
| Zcchc24 | 0.92886 | 1.1581 | 19818 | |
| Atpif1 | 0.92888 | 0.76253 | 19819 | |
| Fam47e | 0.9289 | 0.072606 | 19820 | |
| Arhgef9 | 0.92897 | 0.14007 | 19821 | |
| mmu-mir-6975 | 0.92897 | -0.14799 | 19822 | |
| Mrfap1 | 0.92898 | 1.3472 | 19823 | |
| Tmem97 | 0.92902 | 0.21818 | 19824 | |
| Zfp568 | 0.92904 | 0.92476 | 19825 | |
| Papd5 | 0.92908 | 0.30946 | 19826 | |
| mmu-mir-7681 | 0.92917 | -0.069037 | 19827 | |
| Pomk | 0.92919 | 0.50086 | 19828 | |
| Bhlha9 | 0.9293 | 0.64663 | 19829 | |
| Mfsd3 | 0.92934 | 0.13716 | 19830 | |
| Adamtsl5 | 0.92935 | 0.10103 | 19831 | |
| Ccdc28b | 0.92948 | 0.48751 | 19832 | |
| Foxo3 | 0.9295 | 0.35211 | 19833 | |
| Folr4 | 0.92951 | 0.32888 | 19834 | |
| Exoc3l4 | 0.92955 | 0.21805 | 19835 | |
| Prom1 | 0.9296 | 0.73941 | 19836 | |
| Txlng | 0.92964 | 0.68234 | 19837 | |
| BC027072 | 0.92967 | 1.001 | 19838 | |
| T2 | 0.92968 | 0.34422 | 19839 | |
| Slamf1 | 0.92976 | 0.72623 | 19840 | |
| H2-M10.3 | 0.92979 | 0.67439 | 19841 | |
| 15-Sep | 0.92994 | -0.016419 | 19842 | |
| Wnt2b | 0.93021 | 0.061591 | 19843 | |
| App | 0.9303 | 0.94881 | 19844 | |
| Ppcs | 0.93032 | 0.12508 | 19845 | |
| Nkx2-9 | 0.93033 | 0.36151 | 19846 | |
| Ufsp2 | 0.93034 | 0.1901 | 19847 | |
| Cxcl2 | 0.93035 | 0.14491 | 19848 | |
| Car10 | 0.93036 | 0.40546 | 19849 | |
| Ppip5k1 | 0.93037 | 0.7875 | 19850 | |
| Tspan10 | 0.9304 | 0.59161 | 19851 | |
| Irak3 | 0.93042 | 0.071848 | 19852 | |
| Scly | 0.93047 | 0.67117 | 19853 | |
| Sema5b | 0.9305 | 0.11377 | 19854 | |
| 1110038F14Rik | 0.93052 | 0.17315 | 19855 | |
| Osmr | 0.93058 | -0.016825 | 19856 | |
| Unc80 | 0.9306 | 0.29163 | 19857 | |
| Tex28 | 0.93061 | 1.2382 | 19858 | |
| Rgs16 | 0.93062 | 0.27772 | 19859 | |
| Pcdha8 | 0.93064 | 0.89153 | 19860 | |
| mmu-mir-683-1 | 0.93069 | 0.40199 | 19861 | |
| Slc25a1 | 0.93069 | 0.42762 | 19862 | |
| Rc3h2 | 0.9307 | 0.28174 | 19863 | |
| Olfr805 | 0.93077 | 0.51979 | 19864 | |
| H2-Q6 | 0.93086 | 0.66841 | 19865 | |
| Vmo1 | 0.93088 | 0.4729 | 19866 | |
| Dhtkd1 | 0.93092 | 0.063336 | 19867 | |
| Ocrl | 0.93111 | 0.056626 | 19868 | |
| Psma3 | 0.93111 | 0.1057 | 19869 | |
| Olfr1089 | 0.93121 | 0.28008 | 19870 | |
| Zfp961 | 0.93123 | 0.20581 | 19871 | |
| Cers5 | 0.93124 | 0.27589 | 19872 | |
| Dsel | 0.93128 | 0.78472 | 19873 | |
| Marveld3 | 0.93129 | 0.86852 | 19874 | |
| Macrod1 | 0.9313 | 0.52399 | 19875 | |
| Garnl3 | 0.93131 | 0.35993 | 19876 | |
| Rps6kb2 | 0.93135 | 0.70991 | 19877 | |
| Get4 | 0.9314 | 1.2129 | 19878 | |
| Pla2g7 | 0.93141 | 1.0133 | 19879 | |
| Slc10a4 | 0.93142 | 0.70215 | 19880 | |
| Pxt1 | 0.93143 | 0.0903 | 19881 | |
| Gpr3 | 0.93153 | 0.60253 | 19882 | |
| Hnrnph3 | 0.93153 | 0.49116 | 19883 | |
| Mafg | 0.93156 | 0.22325 | 19884 | |
| Olfr282 | 0.93156 | 0.3903 | 19885 | |
| Pycr1 | 0.93159 | 1.1405 | 19886 | |
| Il18rap | 0.93168 | 1.1838 | 19887 | |
| Defb12 | 0.93169 | 0.072372 | 19888 | |
| mmu-mir-7219 | 0.93171 | -0.012466 | 19889 | |
| Hykk | 0.93172 | 0.20401 | 19890 | |
| 4932438H23Rik | 0.93179 | 1.9929 | 19891 | |
| mmu-mir-6948 | 0.93183 | 0.51484 | 19892 | |
| Pdha2 | 0.93185 | 0.36135 | 19893 | |
| Mospd3 | 0.93188 | 0.5496 | 19894 | |
| Ap3s1 | 0.93191 | 0.7597 | 19895 | |
| mmu-mir-296 | 0.93193 | 0.38968 | 19896 | |
| mmu-mir-3968 | 0.93193 | 1.3385 | 19897 | |
| Fibcd1 | 0.93196 | 0.089875 | 19898 | |
| Rdh16 | 0.93197 | 1.3208 | 19899 | |
| Spred1 | 0.93199 | 0.16591 | 19900 | |
| mmu-mir-7056 | 0.93203 | -0.18436 | 19901 | |
| Gria2 | 0.93206 | 0.42229 | 19902 | |
| Naip7 | 0.9321 | 0.59886 | 19903 | |
| Slc38a4 | 0.93211 | 0.52511 | 19904 | |
| BC068157 | 0.93221 | 0.045713 | 19905 | |
| Ulbp1 | 0.93222 | 0.39984 | 19906 | |
| Gm20816 | 0.93226 | 1.3941 | 19907 | |
| Cdsn | 0.93234 | 0.37509 | 19908 | |
| H2-Q4 | 0.93241 | 0.33397 | 19909 | |
| Gm13083 | 0.93246 | 1.0543 | 19910 | |
| Ap3m2 | 0.93247 | 0.21695 | 19911 | |
| Olfr481 | 0.93253 | 1.937 | 19912 | |
| Skint10 | 0.93266 | 1.6185 | 19913 | |
| Cd300c | 0.93267 | 0.12187 | 19914 | |
| Pianp | 0.93269 | 1.7491 | 19915 | |
| Spp2 | 0.9327 | 0.84787 | 19916 | |
| Stx7 | 0.93273 | 0.32181 | 19917 | |
| E130311K13Rik | 0.93281 | 0.63422 | 19918 | |
| Gm1604b | 0.93301 | 0.035499 | 19919 | |
| 1700093K21Rik | 0.93309 | 0.30644 | 19920 | |
| Kdelc1 | 0.93312 | 1.2578 | 19921 | |
| Fabp6 | 0.93314 | 0.06252 | 19922 | |
| Megf6 | 0.93317 | 0.34509 | 19923 | |
| Sost | 0.93319 | 0.34032 | 19924 | |
| BC061194 | 0.93322 | 0.12126 | 19925 | |
| Crhr1 | 0.93324 | 0.06339 | 19926 | |
| Col2a1 | 0.93341 | 0.24138 | 19927 | |
| Filip1l | 0.93344 | 0.11772 | 19928 | |
| Tdpoz4 | 0.93352 | 0.10803 | 19929 | |
| Rgr | 0.93353 | 0.67981 | 19930 | |
| Nmbr | 0.93362 | 0.13869 | 19931 | |
| 4833427G06Rik | 0.93362 | 0.69621 | 19932 | |
| 1110017D15Rik | 0.93363 | 0.2366 | 19933 | |
| Spaca6 | 0.93364 | 0.044542 | 19934 | |
| mmu-mir-6400 | 0.9337 | -0.11647 | 19935 | |
| Pcbp1 | 0.93373 | 1.5924 | 19936 | |
| Pik3ap1 | 0.93375 | 0.41908 | 19937 | |
| Cdk16 | 0.93384 | 0.87017 | 19938 | |
| Zpbp2 | 0.93388 | 0.83067 | 19939 | |
| Ttc30b | 0.93391 | 0.96948 | 19940 | |
| Ccdc71l | 0.93393 | 0.76388 | 19941 | |
| Phf6 | 0.93396 | 0.24513 | 19942 | |
| Gdap1l1 | 0.93397 | 0.10342 | 19943 | |
| Gm1966 | 0.93399 | 0.81616 | 19944 | |
| Mlf2 | 0.93399 | 1.0394 | 19945 | |
| Efcab7 | 0.93408 | 0.36344 | 19946 | |
| Actr1b | 0.93416 | 0.42171 | 19947 | |
| mmu-mir-6387 | 0.93416 | 0.80947 | 19948 | |
| Fbxl16 | 0.93417 | 0.076739 | 19949 | |
| mmu-mir-6972 | 0.93427 | 0.36801 | 19950 | |
| Olfr176 | 0.93432 | 0.68473 | 19951 | |
| Grin3b | 0.93448 | 1.7803 | 19952 | |
| Cd109 | 0.9345 | 1.2761 | 19953 | |
| Matk | 0.9346 | 0.74375 | 19954 | |
| Eif2a | 0.9346 | 0.49373 | 19955 | |
| Scn7a | 0.93465 | 0.68845 | 19956 | |
| Nbas | 0.93465 | 0.28317 | 19957 | |
| Orai3 | 0.93474 | 0.10968 | 19958 | |
| Thumpd2 | 0.9348 | 0.055043 | 19959 | |
| mmu-mir-6940 | 0.93482 | 0.50441 | 19960 | |
| Gm3417 | 0.93485 | 0.16996 | 19961 | |
| Klk14 | 0.93491 | 0.54863 | 19962 | |
| 4930432M17Rik | 0.93494 | 0.6925 | 19963 | |
| mmu-mir-6968 | 0.93496 | -0.16559 | 19964 | |
| Chchd1 | 0.93498 | 0.23411 | 19965 | |
| Crhr2 | 0.935 | 0.74497 | 19966 | |
| Tfpi2 | 0.93501 | 3.125 | 19967 | |
| Pgp | 0.93502 | 2.6531 | 19968 | |
| Rfxap | 0.93503 | 0.74374 | 19969 | |
| Xkr7 | 0.93512 | 0.66929 | 19970 | |
| Slc38a9 | 0.93522 | 0.21369 | 19971 | |
| Gda | 0.93524 | 0.23669 | 19972 | |
| Rimbp2 | 0.93529 | 0.26401 | 19973 | |
| Caly | 0.93532 | 0.85504 | 19974 | |
| Ppm1h | 0.93537 | 0.59282 | 19975 | |
| Gfral | 0.93538 | 0.14067 | 19976 | |
| Mat2b | 0.93539 | 0.4029 | 19977 | |
| F7 | 0.93543 | 0.87253 | 19978 | |
| Cdc42ep5 | 0.93553 | 0.22124 | 19979 | |
| Npnt | 0.93558 | 0.34363 | 19980 | |
| Phpt1 | 0.93562 | 0.63327 | 19981 | |
| Olfr17 | 0.93569 | 0.1633 | 19982 | |
| 2310035C23Rik | 0.9357 | 0.75593 | 19983 | |
| Nf2 | 0.93571 | 0.71162 | 19984 | |
| Pgm5 | 0.93572 | 0.068006 | 19985 | |
| Slc4a8 | 0.93579 | 0.86482 | 19986 | |
| Dcaf5 | 0.93581 | 0.51011 | 19987 | |
| Cdk19 | 0.93583 | 0.28838 | 19988 | |
| Trim26 | 0.93585 | 0.51945 | 19989 | |
| Ern1 | 0.93586 | 0.055377 | 19990 | |
| Mfrp | 0.93587 | 0.025081 | 19991 | |
| Cntnap5a | 0.93597 | 0.53099 | 19992 | |
| Pdzrn3 | 0.936 | 0.56142 | 19993 | |
| Tmem104 | 0.93601 | 0.23631 | 19994 | |
| Fam151a | 0.93602 | 0.77704 | 19995 | |
| Zfp235 | 0.93606 | 0.92329 | 19996 | |
| Ifltd1 | 0.93611 | 0.45667 | 19997 | |
| Nenf | 0.93613 | 0.69428 | 19998 | |
| Serpina3j | 0.93616 | 0.19566 | 19999 | |
| Mgat4c | 0.93616 | 1.1435 | 20000 | |
| Patl1 | 0.93629 | 0.29227 | 20001 | |
| Dusp1 | 0.9363 | 0.16564 | 20002 | |
| Ccdc170 | 0.93631 | 0.87643 | 20003 | |
| Fsd1 | 0.93633 | 0.050844 | 20004 | |
| H2-L | 0.93638 | 3.3729 | 20005 | |
| Olfr1130 | 0.9364 | 0.4017 | 20006 | |
| Opn1mw | 0.93641 | 1.0856 | 20007 | |
| Aldh1a7 | 0.93647 | 0.42887 | 20008 | |
| Zfp882 | 0.93658 | 0.57991 | 20009 | |
| 4930568D16Rik | 0.93665 | 1.9809 | 20010 | |
| Gm525 | 0.93669 | 0.20465 | 20011 | |
| Babam1 | 0.93669 | 0.26212 | 20012 | |
| Pdia2 | 0.9367 | 1.1111 | 20013 | |
| Gadd45g | 0.93671 | 0.81942 | 20014 | |
| Cd3e | 0.93675 | 0.45548 | 20015 | |
| Cdhr5 | 0.93676 | 0.51638 | 20016 | |
| Tmsb4x | 0.93677 | 0.77854 | 20017 | |
| Fuca1 | 0.93679 | 0.031459 | 20018 | |
| Adamts8 | 0.93686 | 1.2859 | 20019 | |
| Cnih4 | 0.9369 | 0.43151 | 20020 | |
| Bcap29 | 0.93713 | 0.44641 | 20021 | |
| Pdgfd | 0.93713 | 0.31382 | 20022 | |
| Tbr1 | 0.93718 | 2.2735 | 20023 | |
| Fam89a | 0.93721 | 0.11311 | 20024 | |
| Pitpnm3 | 0.93728 | 0.2163 | 20025 | |
| Rtp2 | 0.9373 | 0.025847 | 20026 | |
| Sult6b1 | 0.93731 | 0.10193 | 20027 | |
| Npy4r | 0.93733 | 0.11821 | 20028 | |
| Fam118a | 0.93738 | 0.8939 | 20029 | |
| 2010107G23Rik | 0.93739 | 0.70532 | 20030 | |
| Dhx34 | 0.93752 | 0.17124 | 20031 | |
| Olfr1423 | 0.93754 | 0.12405 | 20032 | |
| Sdhaf2 | 0.93755 | 0.027733 | 20033 | |
| Serp1 | 0.93761 | 0.40383 | 20034 | |
| mmu-mir-330 | 0.93761 | 0.15281 | 20035 | |
| mmu-mir-297a-4 | 0.93765 | 0.64676 | 20036 | |
| Vmn1r112 | 0.93766 | 1.724 | 20037 | |
| Tmem201 | 0.93774 | 1.1539 | 20038 | |
| Lpin3 | 0.93776 | 0.23292 | 20039 | |
| Naip2 | 0.93777 | 0.53908 | 20040 | |
| Vmn1r100 | 0.93777 | 1.5403 | 20041 | |
| Prokr2 | 0.93783 | 0.48875 | 20042 | |
| Ralgps1 | 0.93786 | 0.25421 | 20043 | |
| Tmem120a | 0.93789 | 0.42043 | 20044 | |
| Spr | 0.93791 | 0.11221 | 20045 | |
| Slx1b | 0.93795 | 0.25527 | 20046 | |
| Mxra8 | 0.93803 | 0.51834 | 20047 | |
| Ldhb | 0.93811 | 0.56478 | 20048 | |
| Mtnr1b | 0.93815 | 0.98395 | 20049 | |
| Spesp1 | 0.93821 | 0.36126 | 20050 | |
| Map3k10 | 0.93829 | 0.23688 | 20051 | |
| Vsig10 | 0.93843 | 0.078699 | 20052 | |
| Bbs7 | 0.93844 | 0.19191 | 20053 | |
| Dusp18 | 0.93848 | 1.5898 | 20054 | |
| Gm7534 | 0.93848 | 1.2298 | 20055 | |
| Suv39h2 | 0.9385 | 0.85885 | 20056 | |
| Cypt12 | 0.93853 | 0.36502 | 20057 | |
| Krt18 | 0.93853 | 0.73431 | 20058 | |
| Olfr1279 | 0.93858 | 1.5543 | 20059 | |
| Psg16 | 0.93861 | 1.2015 | 20060 | |
| Gm9992 | 0.93866 | 0.5743 | 20061 | |
| Nrg1 | 0.93868 | 0.58623 | 20062 | |
| Gfra1 | 0.93873 | 1.1117 | 20063 | |
| Olfr723 | 0.93875 | 0.17886 | 20064 | |
| Spag9 | 0.93877 | 1.4447 | 20065 | |
| Ttc9c | 0.9388 | 0.33465 | 20066 | |
| Sar1b | 0.93885 | 0.21914 | 20067 | |
| Pias4 | 0.93893 | 1.1183 | 20068 | |
| Prdm2 | 0.93899 | 0.38383 | 20069 | |
| Nek3 | 0.93901 | 0.18513 | 20070 | |
| mmu-mir-374b | 0.93902 | 1.1706 | 20071 | |
| Vmn1r31 | 0.93903 | 0.70847 | 20072 | |
| Olfr1105 | 0.93907 | 0.2345 | 20073 | |
| Bend4 | 0.93908 | 0.32909 | 20074 | |
| Gfm2 | 0.93916 | 0.5074 | 20075 | |
| F830045P16Rik | 0.93918 | 0.30414 | 20076 | |
| Arsg | 0.93922 | 0.17612 | 20077 | |
| Disp1 | 0.93925 | 0.33624 | 20078 | |
| Nog | 0.93926 | 0.14819 | 20079 | |
| Anxa2 | 0.93929 | 0.27624 | 20080 | |
| Adam24 | 0.9393 | 0.84546 | 20081 | |
| Triml2 | 0.93934 | 0.053932 | 20082 | |
| Fuk | 0.93938 | 0.57884 | 20083 | |
| Krtap11-1 | 0.93939 | 1.0216 | 20084 | |
| Nek1 | 0.93944 | 1.2922 | 20085 | |
| Sema4a | 0.93945 | 0.35458 | 20086 | |
| Atoh7 | 0.93946 | 0.2566 | 20087 | |
| Kif5c | 0.93949 | 0.46419 | 20088 | |
| Rhcg | 0.93954 | 0.22261 | 20089 | |
| Ucn | 0.93967 | 0.36666 | 20090 | |
| mmu-mir-378c | 0.93972 | -0.077287 | 20091 | |
| mmu-mir-7660 | 0.93974 | 0.037049 | 20092 | |
| Gpr68 | 0.93976 | 0.25911 | 20093 | |
| Taok2 | 0.93981 | 1.0868 | 20094 | |
| Odam | 0.93982 | 1.7575 | 20095 | |
| Nabp2 | 0.93983 | 0.20075 | 20096 | |
| Olfr974 | 0.93993 | 0.15999 | 20097 | |
| Vmn2r93 | 0.93995 | 0.5904 | 20098 | |
| Xrcc3 | 0.94002 | 0.24279 | 20099 | |
| Ciz1 | 0.94015 | 0.90392 | 20100 | |
| Akt1s1 | 0.94016 | 0.063063 | 20101 | |
| Mgat3 | 0.94023 | 0.33854 | 20102 | |
| Slx | 0.94025 | 1.2815 | 20103 | |
| Ankrd33 | 0.94025 | 0.51103 | 20104 | |
| Cetn3 | 0.94032 | 1.0225 | 20105 | |
| Crlf1 | 0.94039 | 1.0128 | 20106 | |
| 1700025G04Rik | 0.94041 | 0.38108 | 20107 | |
| Bnc2 | 0.94048 | 1.9426 | 20108 | |
| Ccdc30 | 0.9405 | 0.21916 | 20109 | |
| Tnks1bp1 | 0.94052 | 0.4604 | 20110 | |
| Flrt1 | 0.94053 | 0.42964 | 20111 | |
| Wiz | 0.94056 | 0.15459 | 20112 | |
| Atp5s | 0.94059 | 0.11745 | 20113 | |
| Obfc1 | 0.94061 | 1.5468 | 20114 | |
| Cdnf | 0.94066 | 0.7721 | 20115 | |
| Htra3 | 0.94066 | 0.17842 | 20116 | |
| 4921504E06Rik | 0.94069 | 0.54901 | 20117 | |
| A530016L24Rik | 0.94074 | 0.7141 | 20118 | |
| Vimp | 0.94079 | 0.20286 | 20119 | |
| Manbal | 0.94081 | 0.74036 | 20120 | |
| Akap1 | 0.94088 | 0.066552 | 20121 | |
| Mycbp | 0.94095 | 0.9954 | 20122 | |
| Sod2 | 0.94096 | 0.72052 | 20123 | |
| Mrps25 | 0.94097 | 0.067617 | 20124 | |
| Leprel2 | 0.94099 | 0.080798 | 20125 | |
| Deaf1 | 0.94101 | 0.40563 | 20126 | |
| Tlr13 | 0.94103 | 1.86 | 20127 | |
| Cyp3a41a | 0.94103 | 0.68784 | 20128 | |
| Nap1l3 | 0.9411 | 0.95317 | 20129 | |
| Ankle1 | 0.94111 | 0.85178 | 20130 | |
| Abcg4 | 0.94122 | 0.62473 | 20131 | |
| Dnaja4 | 0.94129 | 0.56517 | 20132 | |
| Hoxb13 | 0.94133 | 0.16557 | 20133 | |
| Egfr | 0.94134 | 1.1891 | 20134 | |
| Shd | 0.9414 | 1.7081 | 20135 | |
| Izumo2 | 0.94144 | 0.78626 | 20136 | |
| Tm9sf1 | 0.94145 | 1.5326 | 20137 | |
| Trappc13 | 0.9415 | 0.70429 | 20138 | |
| mmu-mir-219a-1 | 0.94154 | -0.051484 | 20139 | |
| Paqr6 | 0.94168 | 2.9004 | 20140 | |
| Pcdhb13 | 0.94171 | 0.097863 | 20141 | |
| Cmip | 0.94172 | 1.8426 | 20142 | |
| Slfn9 | 0.94185 | 0.082775 | 20143 | |
| Cd302 | 0.94189 | 0.17752 | 20144 | |
| Gm5136 | 0.94192 | 1.9054 | 20145 | |
| Eif3j2 | 0.94193 | 1.0199 | 20146 | |
| Olfr603 | 0.94198 | 1.29 | 20147 | |
| Ofcc1 | 0.94204 | 0.60609 | 20148 | |
| Vmn2r40 | 0.94208 | 0.71539 | 20149 | |
| Npb | 0.94208 | 0.45851 | 20150 | |
| Pde4c | 0.94218 | 1.2882 | 20151 | |
| Ctf1 | 0.94226 | 0.16418 | 20152 | |
| Rbm6 | 0.94231 | 2.1855 | 20153 | |
| Ccdc43 | 0.94232 | 0.76481 | 20154 | |
| Rilp | 0.94234 | 0.16527 | 20155 | |
| Map3k19 | 0.94238 | 0.82494 | 20156 | |
| Brd7 | 0.9424 | 0.6102 | 20157 | |
| Cd248 | 0.94247 | 1.5087 | 20158 | |
| Tet2 | 0.94255 | 0.78964 | 20159 | |
| Tmem229b | 0.94255 | 0.16925 | 20160 | |
| Pebp4 | 0.94257 | 0.40747 | 20161 | |
| Stc2 | 0.94259 | 0.29279 | 20162 | |
| Ubxn8 | 0.94261 | 0.1882 | 20163 | |
| Rpp25 | 0.94263 | 1.7879 | 20164 | |
| Vps33b | 0.94264 | 0.67833 | 20165 | |
| C8g | 0.94271 | 0.69429 | 20166 | |
| Nek7 | 0.94274 | 0.14552 | 20167 | |
| Gpr115 | 0.94277 | 0.68089 | 20168 | |
| Ccdc153 | 0.94282 | 0.85596 | 20169 | |
| Tgm6 | 0.94283 | 1.6329 | 20170 | |
| Kctd4 | 0.94289 | 1.1443 | 20171 | |
| Rfx7 | 0.9429 | 0.21162 | 20172 | |
| St14 | 0.94291 | 0.30725 | 20173 | |
| Lrriq3 | 0.94296 | 0.87257 | 20174 | |
| Prkce | 0.94311 | 0.23884 | 20175 | |
| Ccdc25 | 0.94317 | 0.47488 | 20176 | |
| Rbm18 | 0.94319 | 0.44434 | 20177 | |
| Sp110 | 0.94328 | 0.17272 | 20178 | |
| Pcdhb12 | 0.94334 | 0.28629 | 20179 | |
| Iyd | 0.9434 | 0.38459 | 20180 | |
| Osgin1 | 0.94341 | 0.89801 | 20181 | |
| Gpatch2 | 0.94343 | 0.90197 | 20182 | |
| Esr1 | 0.94345 | 0.75001 | 20183 | |
| 1700065I17Rik | 0.94346 | 0.055482 | 20184 | |
| Katnb1 | 0.9435 | 0.20799 | 20185 | |
| 4930578I06Rik | 0.94351 | 0.18385 | 20186 | |
| Ces2a | 0.94355 | 0.89807 | 20187 | |
| Sval1 | 0.94359 | 0.29516 | 20188 | |
| Rnf144b | 0.94362 | 0.64079 | 20189 | |
| Ube2g2 | 0.94364 | 0.34328 | 20190 | |
| Upk3a | 0.94371 | 0.23701 | 20191 | |
| Ecscr | 0.94372 | 0.88915 | 20192 | |
| Vmn1r23 | 0.94374 | 0.59038 | 20193 | |
| Hsd17b2 | 0.94376 | 0.16984 | 20194 | |
| B3gat2 | 0.94391 | 0.20227 | 20195 | |
| Pim3 | 0.94393 | 2.1393 | 20196 | |
| Nudt13 | 0.94398 | 0.15173 | 20197 | |
| Mcpt8 | 0.94406 | 0.1679 | 20198 | |
| Emr4 | 0.94414 | 0.72617 | 20199 | |
| Sphk2 | 0.94421 | 0.26902 | 20200 | |
| Hmg20b | 0.94423 | 0.31123 | 20201 | |
| Awat2 | 0.94428 | 0.32342 | 20202 | |
| Defb26 | 0.94443 | 0.72023 | 20203 | |
| Zfp449 | 0.94444 | 0.26794 | 20204 | |
| Exo1 | 0.94452 | 0.23719 | 20205 | |
| Apon | 0.94452 | 0.12991 | 20206 | |
| Usp48 | 0.94454 | 1.7034 | 20207 | |
| Flywch2 | 0.94455 | 0.14391 | 20208 | |
| Abca3 | 0.94457 | 1.2058 | 20209 | |
| Zfp354b | 0.94462 | 0.65206 | 20210 | |
| Sidt2 | 0.94465 | 0.22146 | 20211 | |
| 4930503L19Rik | 0.94472 | 2.2338 | 20212 | |
| Dmrta2 | 0.94475 | 1.0573 | 20213 | |
| Slx4ip | 0.94479 | 2.1296 | 20214 | |
| Pgk2 | 0.94481 | 0.89072 | 20215 | |
| Slc4a3 | 0.94482 | 1.1939 | 20216 | |
| Tomm34 | 0.94483 | 0.10711 | 20217 | |
| Rdh13 | 0.94484 | 1.8334 | 20218 | |
| AU015228 | 0.9449 | 1.4083 | 20219 | |
| Stk32b | 0.94491 | 1.6553 | 20220 | |
| Egln2 | 0.94495 | 0.42701 | 20221 | |
| Csf2rb | 0.94501 | 0.16639 | 20222 | |
| D16Ertd472e | 0.94502 | 0.59667 | 20223 | |
| Klk1b8 | 0.94504 | 1.6612 | 20224 | |
| Slamf7 | 0.94506 | 1.2387 | 20225 | |
| Ccdc91 | 0.94508 | 1.5264 | 20226 | |
| Fam47c | 0.94508 | 0.41902 | 20227 | |
| Kcnd1 | 0.94515 | 0.085332 | 20228 | |
| Il27 | 0.9452 | 2.7551 | 20229 | |
| Flot2 | 0.94524 | 0.76403 | 20230 | |
| Zfp51 | 0.94525 | 1.2442 | 20231 | |
| Asb17 | 0.94527 | 0.088307 | 20232 | |
| Olfr1183 | 0.94529 | 0.47277 | 20233 | |
| Mast2 | 0.94533 | 0.5118 | 20234 | |
| Slc10a1 | 0.94538 | 0.31806 | 20235 | |
| Armc2 | 0.94543 | 1.702 | 20236 | |
| mmu-mir-6957 | 0.94545 | 0.061284 | 20237 | |
| Ptrhd1 | 0.94548 | 1.3904 | 20238 | |
| Tnp1 | 0.94553 | 0.46036 | 20239 | |
| Tmem216 | 0.94554 | 0.30989 | 20240 | |
| mmu-mir-6950 | 0.94555 | 0.29461 | 20241 | |
| Cux1 | 0.94555 | 0.43693 | 20242 | |
| Gstp2 | 0.94556 | 0.20603 | 20243 | |
| Lamb2 | 0.94558 | 0.85889 | 20244 | |
| 1700010I14Rik | 0.9456 | 0.51384 | 20245 | |
| Gps2 | 0.94577 | 0.40839 | 20246 | |
| Dak | 0.94582 | 0.77445 | 20247 | |
| mmu-mir-218-2 | 0.94607 | 0.51894 | 20248 | |
| Ptk7 | 0.94608 | 0.82724 | 20249 | |
| Tyrobp | 0.94612 | 0.74922 | 20250 | |
| 6-Mar | 0.94616 | 0.45437 | 20251 | |
| Slc25a31 | 0.9462 | 1.5484 | 20252 | |
| Trp63 | 0.94628 | 0.95715 | 20253 | |
| Nrxn3 | 0.9465 | 1.1349 | 20254 | |
| Olfr1204 | 0.94654 | 0.14965 | 20255 | |
| Idua | 0.94656 | 1.5115 | 20256 | |
| Sowahc | 0.9466 | 0.30499 | 20257 | |
| Pdcd2l | 0.9467 | 0.083311 | 20258 | |
| Liph | 0.94671 | 1.7753 | 20259 | |
| Cda | 0.94676 | 0.67213 | 20260 | |
| Gpank1 | 0.9468 | 0.28232 | 20261 | |
| Map4k5 | 0.94685 | 0.43409 | 20262 | |
| Akap2 | 0.94688 | 0.21096 | 20263 | |
| Adcy9 | 0.94694 | 0.090556 | 20264 | |
| Gm3706 | 0.94694 | 1.4812 | 20265 | |
| Runx3 | 0.94698 | 0.2652 | 20266 | |
| Scand1 | 0.94699 | 0.074339 | 20267 | |
| Gm13286 | 0.947 | 0.23395 | 20268 | |
| Olfr152 | 0.94701 | 0.82655 | 20269 | |
| Hmx1 | 0.94708 | 0.61032 | 20270 | |
| C1rl | 0.94719 | 1.1297 | 20271 | |
| Trpm4 | 0.94721 | 0.71318 | 20272 | |
| Mapkapk2 | 0.94724 | 0.27186 | 20273 | |
| Acsl1 | 0.94726 | 0.91893 | 20274 | |
| Cul4a | 0.94728 | 0.96373 | 20275 | |
| Arid5a | 0.94734 | 0.41473 | 20276 | |
| Fam19a5 | 0.94735 | 1.0074 | 20277 | |
| Olfr1287 | 0.94737 | 0.31272 | 20278 | |
| Reps2 | 0.94737 | 0.4096 | 20279 | |
| Eif4g3 | 0.94743 | 0.83435 | 20280 | |
| Art1 | 0.94746 | 0.51676 | 20281 | |
| Fgf2 | 0.94747 | 1.0124 | 20282 | |
| Lrrc16a | 0.94748 | 1.843 | 20283 | |
| Asnsd1 | 0.94751 | 1.4346 | 20284 | |
| Ctsl | 0.94755 | 0.94145 | 20285 | |
| Slco1a6 | 0.94762 | 0.89588 | 20286 | |
| Cdk17 | 0.94764 | 0.15656 | 20287 | |
| mmu-mir-7666 | 0.94769 | 0.93926 | 20288 | |
| mmu-mir-6994 | 0.94771 | 0.42982 | 20289 | |
| Mroh1 | 0.94774 | 1.7315 | 20290 | |
| Aldh1a2 | 0.94777 | 0.35342 | 20291 | |
| Hbb-bh2 | 0.94781 | 1.943 | 20292 | |
| Rtn4rl2 | 0.94785 | 0.46783 | 20293 | |
| Stambp | 0.94793 | 1.2568 | 20294 | |
| Pcif1 | 0.94795 | 2.6363 | 20295 | |
| Gldc | 0.948 | 0.76885 | 20296 | |
| Angel1 | 0.94819 | 1.5304 | 20297 | |
| Pde6d | 0.94825 | 0.51002 | 20298 | |
| Vmn2r110 | 0.94831 | 0.61686 | 20299 | |
| Gnb4 | 0.94833 | 0.20083 | 20300 | |
| Boc | 0.94833 | 0.57481 | 20301 | |
| Tmem81 | 0.94837 | 0.72962 | 20302 | |
| Clcnka | 0.9484 | 0.94541 | 20303 | |
| Pgam5 | 0.94841 | 1.3939 | 20304 | |
| Abcb1a | 0.94851 | 0.2819 | 20305 | |
| Ppfia1 | 0.94852 | 1.5762 | 20306 | |
| mmu-mir-8090 | 0.94854 | 0.496 | 20307 | |
| Rad21l | 0.94856 | 0.88234 | 20308 | |
| mmu-mir-7683 | 0.94858 | 0.8385 | 20309 | |
| Mst1r | 0.94863 | 1.5498 | 20310 | |
| Tm6sf2 | 0.94864 | 0.18177 | 20311 | |
| Sez6l | 0.94867 | 1.5047 | 20312 | |
| Phyhip | 0.94887 | 0.40255 | 20313 | |
| mmu-mir-130c | 0.94887 | 1.3731 | 20314 | |
| Kctd15 | 0.94889 | 2.7834 | 20315 | |
| Olfr1022 | 0.94905 | 0.22061 | 20316 | |
| Cystm1 | 0.9491 | 0.53879 | 20317 | |
| Fbxl4 | 0.94917 | 0.37566 | 20318 | |
| Foxe3 | 0.9492 | 0.53369 | 20319 | |
| AI747448 | 0.94921 | 0.31351 | 20320 | |
| 4933415F23Rik | 0.94927 | 1.0024 | 20321 | |
| Ufsp1 | 0.94933 | 0.12587 | 20322 | |
| Olfr610 | 0.9494 | 0.79974 | 20323 | |
| Cdo1 | 0.94942 | 0.67674 | 20324 | |
| Ankrd55 | 0.94948 | 0.66742 | 20325 | |
| 9330159F19Rik | 0.94953 | 0.13748 | 20326 | |
| Amdhd1 | 0.94954 | 0.52885 | 20327 | |
| Klf14 | 0.94955 | 0.3724 | 20328 | |
| Necap2 | 0.94965 | 0.2487 | 20329 | |
| Hirip3 | 0.94967 | 0.5021 | 20330 | |
| Fam20c | 0.94967 | 0.80971 | 20331 | |
| Ccdc42 | 0.94971 | 1.0243 | 20332 | |
| 2610008E11Rik | 0.94977 | 1.1618 | 20333 | |
| Olfr1349 | 0.94978 | 0.26421 | 20334 | |
| Pla2g4f | 0.94984 | 0.77892 | 20335 | |
| Slc7a9 | 0.94986 | 0.18394 | 20336 | |
| Rogdi | 0.94991 | 0.55947 | 20337 | |
| Gmpr2 | 0.94995 | 0.48479 | 20338 | |
| Plekha1 | 0.95006 | 0.68062 | 20339 | |
| Pde4b | 0.9501 | 1.899 | 20340 | |
| mmu-mir-6239 | 0.9501 | 0.6906 | 20341 | |
| Smg9 | 0.95014 | 1.0944 | 20342 | |
| Il20rb | 0.95014 | 0.11573 | 20343 | |
| Cul7 | 0.95015 | 0.43966 | 20344 | |
| Tmem28 | 0.95019 | 0.23988 | 20345 | |
| Defb6 | 0.95021 | 0.25959 | 20346 | |
| Angel2 | 0.95023 | 0.77863 | 20347 | |
| Gm5544 | 0.95025 | 0.20984 | 20348 | |
| Mtx3 | 0.95029 | 0.52185 | 20349 | |
| mmu-mir-3100 | 0.95033 | 0.92316 | 20350 | |
| mmu-mir-7229 | 0.95046 | 0.65384 | 20351 | |
| Gsr | 0.95049 | 1.1498 | 20352 | |
| Irg1 | 0.95056 | 0.32251 | 20353 | |
| Gabrg1 | 0.95058 | 2.6898 | 20354 | |
| Dact1 | 0.95058 | 0.52028 | 20355 | |
| Mobp | 0.95059 | 0.38624 | 20356 | |
| Tal1 | 0.95062 | 0.12978 | 20357 | |
| Bmper | 0.95063 | 0.70372 | 20358 | |
| Zdhhc11 | 0.95068 | 0.27826 | 20359 | |
| Lrrc52 | 0.95076 | 0.74363 | 20360 | |
| Trpc4 | 0.95081 | 0.15051 | 20361 | |
| Rnf123 | 0.951 | 2.8508 | 20362 | |
| Hs6st3 | 0.95104 | 0.65349 | 20363 | |
| 1700071K01Rik | 0.95104 | 1.4713 | 20364 | |
| mmu-mir-7216 | 0.95107 | -0.01849 | 20365 | |
| Gm10681 | 0.9511 | 0.78418 | 20366 | |
| mmu-mir-196a-2 | 0.95116 | 1.5006 | 20367 | |
| Prss12 | 0.95119 | 0.65878 | 20368 | |
| Cd209d | 0.95129 | 1.2701 | 20369 | |
| Olfr1255 | 0.9513 | 0.1527 | 20370 | |
| Me1 | 0.95136 | 0.25558 | 20371 | |
| Tpk1 | 0.95144 | 0.40848 | 20372 | |
| Nfx1 | 0.95149 | 0.7225 | 20373 | |
| Fut1 | 0.9515 | 0.88323 | 20374 | |
| Pbx1 | 0.95158 | 1.5322 | 20375 | |
| Tenm3 | 0.95159 | 0.62141 | 20376 | |
| Otud6a | 0.9516 | 0.57628 | 20377 | |
| Pxn | 0.95164 | 1.3346 | 20378 | |
| Pcdha1 | 0.95164 | 2.0187 | 20379 | |
| Hadha | 0.95175 | 0.79058 | 20380 | |
| Gmeb2 | 0.95181 | 1.2262 | 20381 | |
| Gpr6 | 0.95188 | 0.36617 | 20382 | |
| Ccar2 | 0.95202 | 0.96345 | 20383 | |
| Krtap4-7 | 0.95205 | 0.17779 | 20384 | |
| Klc2 | 0.95211 | 0.65053 | 20385 | |
| Vcam1 | 0.95212 | 0.92662 | 20386 | |
| Bmp8a | 0.95215 | 0.73399 | 20387 | |
| Nfkbie | 0.95223 | 0.58506 | 20388 | |
| 1810013L24Rik | 0.9523 | 1.3117 | 20389 | |
| Tbx15 | 0.95233 | 0.59402 | 20390 | |
| Pitpnb | 0.95234 | 0.98206 | 20391 | |
| Slc25a37 | 0.9524 | 0.56138 | 20392 | |
| Rxfp1 | 0.95241 | 0.78304 | 20393 | |
| Ankdd1b | 0.95252 | 0.35698 | 20394 | |
| Rgs9 | 0.95259 | 2.5039 | 20395 | |
| Eno3 | 0.9526 | 0.42906 | 20396 | |
| Hipk2 | 0.95263 | 0.30783 | 20397 | |
| Cyp2t4 | 0.95266 | 1.6279 | 20398 | |
| Lce1a2 | 0.9527 | 0.92648 | 20399 | |
| Pomt1 | 0.95273 | 0.72493 | 20400 | |
| Zbtb1 | 0.95276 | 0.52262 | 20401 | |
| Ptpru | 0.95286 | 0.69472 | 20402 | |
| 1700110M21Rik | 0.95288 | 1.1994 | 20403 | |
| mmu-mir-202 | 0.95296 | 0.43348 | 20404 | |
| Kcnq5 | 0.95298 | 0.1251 | 20405 | |
| Kcnip1 | 0.95313 | 0.27367 | 20406 | |
| Cyp2a5 | 0.95315 | 0.39156 | 20407 | |
| Ugt2b37 | 0.95318 | 0.74665 | 20408 | |
| Nat3 | 0.9532 | 1.2151 | 20409 | |
| Dmkn | 0.95321 | 1.8387 | 20410 | |
| Nr2f1 | 0.95323 | 0.5601 | 20411 | |
| 10-Mar | 0.95327 | 0.84378 | 20412 | |
| Slc4a5 | 0.95328 | 1.0131 | 20413 | |
| Kcne2 | 0.9533 | 2.1852 | 20414 | |
| Dppa2 | 0.95333 | 1.1226 | 20415 | |
| Lsmem1 | 0.95339 | 0.79597 | 20416 | |
| Rln1 | 0.95339 | 0.74681 | 20417 | |
| Olfr1115 | 0.9534 | 0.67822 | 20418 | |
| Slpi | 0.95341 | 0.33571 | 20419 | |
| Acss3 | 0.95349 | 1.5737 | 20420 | |
| Olfr1000 | 0.95351 | 1.0245 | 20421 | |
| Gtf3c6 | 0.9536 | 1.4025 | 20422 | |
| Gm14548 | 0.95361 | 1.1627 | 20423 | |
| Gm6588 | 0.95373 | 0.89231 | 20424 | |
| Vnn3 | 0.95375 | 0.14957 | 20425 | |
| mmu-mir-7093 | 0.95378 | 0.32837 | 20426 | |
| Rffl | 0.95386 | 0.80794 | 20427 | |
| Tymp | 0.95395 | 1.0538 | 20428 | |
| Gorab | 0.95425 | 1.3282 | 20429 | |
| Tdrp | 0.95428 | 0.4932 | 20430 | |
| 1700008A04Rik | 0.95431 | 0.12814 | 20431 | |
| 1700011L22Rik | 0.95433 | 0.26801 | 20432 | |
| Zfp688 | 0.95438 | 0.25934 | 20433 | |
| Aco2 | 0.9544 | 0.52855 | 20434 | |
| Hspbp1 | 0.9544 | 0.29996 | 20435 | |
| Psors1c2 | 0.9545 | 1.1088 | 20436 | |
| Glul | 0.95461 | 0.15192 | 20437 | |
| Gsdma2 | 0.95464 | 0.3231 | 20438 | |
| Fry | 0.95465 | 1.1259 | 20439 | |
| Shisa6 | 0.95473 | 0.17393 | 20440 | |
| Mup5 | 0.95476 | 0.45513 | 20441 | |
| Fam195a | 0.9548 | 1.8333 | 20442 | |
| Pigr | 0.95484 | 0.9002 | 20443 | |
| Plekho1 | 0.95488 | 0.9119 | 20444 | |
| Emr1 | 0.95488 | 0.65784 | 20445 | |
| Cpxm1 | 0.9549 | 0.70222 | 20446 | |
| Dnajb11 | 0.95491 | 1.6018 | 20447 | |
| Stard4 | 0.95492 | 0.80958 | 20448 | |
| Ccl20 | 0.95498 | 0.47238 | 20449 | |
| Enc1 | 0.955 | 1.0492 | 20450 | |
| 3425401B19Rik | 0.95501 | 0.63693 | 20451 | |
| mmu-mir-7031 | 0.95502 | 0.96235 | 20452 | |
| Slc25a47 | 0.95504 | 0.15627 | 20453 | |
| Slc30a10 | 0.95506 | 0.80908 | 20454 | |
| Plk3 | 0.95509 | 1.8623 | 20455 | |
| Plod3 | 0.95511 | 0.45122 | 20456 | |
| Mageh1 | 0.95517 | 0.2949 | 20457 | |
| Gm5531 | 0.95522 | 1.7124 | 20458 | |
| Dhcr7 | 0.95526 | 0.7856 | 20459 | |
| Emx2 | 0.9554 | 0.37904 | 20460 | |
| Vegfb | 0.95553 | 0.21209 | 20461 | |
| Pradc1 | 0.95561 | 0.52063 | 20462 | |
| Dok3 | 0.95564 | 0.34298 | 20463 | |
| Ccdc160 | 0.95573 | 1.7594 | 20464 | |
| Bex6 | 0.95575 | 0.5049 | 20465 | |
| Sirt1 | 0.9559 | 0.38481 | 20466 | |
| Vmn1r43 | 0.95597 | 0.55715 | 20467 | |
| Gm156 | 0.95603 | 0.75828 | 20468 | |
| Tmem178b | 0.95615 | 0.332 | 20469 | |
| mmu-mir-431 | 0.95616 | 0.66336 | 20470 | |
| Zfp553 | 0.95624 | 0.2798 | 20471 | |
| Olfr1214 | 0.95627 | 0.29208 | 20472 | |
| Evi2a-evi2b | 0.95628 | 0.37396 | 20473 | |
| 1810011H11Rik | 0.95631 | 0.3408 | 20474 | |
| Speer4c | 0.95631 | 1.5198 | 20475 | |
| Ptger4 | 0.95633 | 0.81026 | 20476 | |
| Dlx1 | 0.9564 | 0.33544 | 20477 | |
| Zfp956 | 0.95643 | 1.9044 | 20478 | |
| Hsp90ab1 | 0.95647 | 0.90839 | 20479 | |
| D19Ertd737e | 0.95649 | 0.68731 | 20480 | |
| Layn | 0.95653 | 0.65813 | 20481 | |
| B4galnt4 | 0.95664 | 1.1737 | 20482 | |
| Scrn3 | 0.95664 | 0.41493 | 20483 | |
| Cd247 | 0.95667 | 0.36682 | 20484 | |
| Retnlg | 0.95668 | 0.3476 | 20485 | |
| Gm20738 | 0.95671 | 0.61961 | 20486 | |
| Vmn1r231 | 0.95673 | 0.72363 | 20487 | |
| mmu-mir-758 | 0.95682 | 0.42004 | 20488 | |
| Scaf8 | 0.95689 | 2.0559 | 20489 | |
| Gm6614 | 0.95691 | 0.50788 | 20490 | |
| H2-M10.4 | 0.95691 | 1.7793 | 20491 | |
| Fabp5 | 0.95692 | 0.24061 | 20492 | |
| D1Pas1 | 0.95702 | 0.82887 | 20493 | |
| Nacc2 | 0.95705 | 0.26928 | 20494 | |
| 4930539E08Rik | 0.95707 | 1.1013 | 20495 | |
| Fkbp3 | 0.9571 | 1.3468 | 20496 | |
| 1700007K09Rik | 0.95711 | 0.3174 | 20497 | |
| Hipk4 | 0.95717 | 1.3903 | 20498 | |
| Tk1 | 0.95722 | 0.64957 | 20499 | |
| Trim61 | 0.95725 | 0.98631 | 20500 | |
| Ppp1r2 | 0.95734 | 0.48877 | 20501 | |
| Tssk2 | 0.95735 | 0.60014 | 20502 | |
| Sstr1 | 0.95741 | 1.2035 | 20503 | |
| Zbtb16 | 0.95742 | 0.91421 | 20504 | |
| Eme2 | 0.95754 | 0.82778 | 20505 | |
| Sp4 | 0.9576 | 0.88872 | 20506 | |
| Ccdc146 | 0.95763 | 0.15812 | 20507 | |
| Evi2b | 0.95765 | 0.44617 | 20508 | |
| Gltscr1 | 0.95766 | 0.69991 | 20509 | |
| Tnnt3 | 0.95768 | 0.54226 | 20510 | |
| A830018L16Rik | 0.95773 | 0.66842 | 20511 | |
| Clip4 | 0.95778 | 0.17724 | 20512 | |
| Pnma2 | 0.95779 | 0.9965 | 20513 | |
| Cpne2 | 0.95795 | 1.5551 | 20514 | |
| Mynn | 0.95799 | 0.29802 | 20515 | |
| Slc5a1 | 0.95808 | 0.15832 | 20516 | |
| Per3 | 0.9581 | 1.01 | 20517 | |
| Bsdc1 | 0.95823 | 0.25296 | 20518 | |
| Psg27 | 0.95824 | 0.63468 | 20519 | |
| Ptn | 0.9583 | 1.1122 | 20520 | |
| Arhgap30 | 0.95831 | 0.84561 | 20521 | |
| Hs3st3b1 | 0.95843 | 0.21852 | 20522 | |
| Capn5 | 0.95849 | 0.59391 | 20523 | |
| Gnmt | 0.95854 | 0.39523 | 20524 | |
| Drd5 | 0.95855 | 0.55564 | 20525 | |
| Gjb3 | 0.95855 | 0.55646 | 20526 | |
| Psapl1 | 0.95874 | 0.40085 | 20527 | |
| Tssc1 | 0.95877 | 0.31195 | 20528 | |
| Tnc | 0.95878 | 0.56298 | 20529 | |
| Olfr1 | 0.95888 | 1.4615 | 20530 | |
| Foxo1 | 0.9589 | 0.26246 | 20531 | |
| Elovl7 | 0.95892 | 0.6245 | 20532 | |
| mmu-mir-6346 | 0.95896 | 0.41062 | 20533 | |
| Has3 | 0.95908 | 0.99469 | 20534 | |
| Cpeb1 | 0.95909 | 0.43049 | 20535 | |
| Rap1gap | 0.95914 | 1.23 | 20536 | |
| Luc7l2 | 0.95916 | 1.0164 | 20537 | |
| Tmem217 | 0.95917 | 2.4776 | 20538 | |
| Ociad2 | 0.9592 | 0.75399 | 20539 | |
| Fam84b | 0.95926 | 0.37314 | 20540 | |
| Wnt5a | 0.95933 | 1.2491 | 20541 | |
| Pard3 | 0.95945 | 1.0286 | 20542 | |
| Dtd2 | 0.95946 | 0.63296 | 20543 | |
| Proser1 | 0.95955 | 0.28099 | 20544 | |
| Ccs | 0.95955 | 0.32998 | 20545 | |
| Prap1 | 0.95959 | 0.37996 | 20546 | |
| Mri1 | 0.9596 | 2.2257 | 20547 | |
| Nek10 | 0.9596 | 0.60531 | 20548 | |
| Setd4 | 0.95963 | 0.3417 | 20549 | |
| Fanca | 0.95966 | 1.5828 | 20550 | |
| Golga5 | 0.95976 | 1.9506 | 20551 | |
| Tpd52l1 | 0.95977 | 1.1101 | 20552 | |
| Tspan31 | 0.95978 | 0.36811 | 20553 | |
| Mag | 0.95981 | 1.7592 | 20554 | |
| Cep41 | 0.95983 | 0.88007 | 20555 | |
| Tcf4 | 0.95988 | 0.40912 | 20556 | |
| Tmem87a | 0.95989 | 1.2483 | 20557 | |
| Trim47 | 0.9599 | 0.2492 | 20558 | |
| Uckl1 | 0.95994 | 1.0926 | 20559 | |
| Cyp2c50 | 0.95996 | 0.50141 | 20560 | |
| Itpkc | 0.96006 | 0.40884 | 20561 | |
| Olfr1232 | 0.96008 | 1.8134 | 20562 | |
| Safb2 | 0.96018 | 1.1102 | 20563 | |
| Zyx | 0.96019 | 0.69749 | 20564 | |
| Kdm8 | 0.9602 | 0.95054 | 20565 | |
| E2f4 | 0.96029 | 1.0254 | 20566 | |
| Dennd2a | 0.96034 | 0.9151 | 20567 | |
| Pram1 | 0.96038 | 1.3782 | 20568 | |
| Ifna12 | 0.9604 | 1.788 | 20569 | |
| Oxld1 | 0.96042 | 2.094 | 20570 | |
| Spop | 0.96042 | 1.1535 | 20571 | |
| Ptcra | 0.96046 | 0.69756 | 20572 | |
| mmu-mir-3960 | 0.96056 | 0.52602 | 20573 | |
| Stx6 | 0.96058 | 1.9401 | 20574 | |
| Clps | 0.96073 | 0.30246 | 20575 | |
| Zswim7 | 0.96078 | 0.553 | 20576 | |
| Rhpn1 | 0.96079 | 0.72539 | 20577 | |
| Fli1 | 0.96085 | 1.1449 | 20578 | |
| Apol11a | 0.96086 | 0.21825 | 20579 | |
| Hmx2 | 0.96096 | 1.92 | 20580 | |
| Entpd2 | 0.96097 | 0.50697 | 20581 | |
| Katnal2 | 0.96098 | 1.0729 | 20582 | |
| Ttc23l | 0.96102 | 1.0536 | 20583 | |
| 2510049J12Rik | 0.96106 | 0.32284 | 20584 | |
| Dnajc25 | 0.96114 | 0.22448 | 20585 | |
| Chrm1 | 0.96115 | 1.5232 | 20586 | |
| Zfp493 | 0.96126 | 1.7909 | 20587 | |
| Trim3 | 0.96128 | 1.2683 | 20588 | |
| Cst10 | 0.96135 | 0.55102 | 20589 | |
| Pilra | 0.96144 | 0.64236 | 20590 | |
| Ajuba | 0.9615 | 1.1662 | 20591 | |
| Moxd2 | 0.96151 | 1.4031 | 20592 | |
| Olfr763 | 0.96152 | 0.73667 | 20593 | |
| Slco3a1 | 0.96156 | 0.89751 | 20594 | |
| Chmp1b | 0.96184 | 0.46461 | 20595 | |
| Gm14305 | 0.96188 | 1.8368 | 20596 | |
| Carns1 | 0.96215 | 0.69619 | 20597 | |
| Tcte2 | 0.96222 | 0.80254 | 20598 | |
| Cdc34 | 0.96235 | 0.57129 | 20599 | |
| Blvra | 0.96243 | 1.4291 | 20600 | |
| Commd7 | 0.96254 | 0.37411 | 20601 | |
| Irak2 | 0.96254 | 2.0466 | 20602 | |
| Cep85l | 0.96257 | 1.1716 | 20603 | |
| Oaz3 | 0.96258 | 0.65817 | 20604 | |
| Sec24d | 0.96267 | 0.34214 | 20605 | |
| Ano6 | 0.96269 | 0.93681 | 20606 | |
| Prom2 | 0.96272 | 1.7629 | 20607 | |
| Rbm7 | 0.96276 | 0.32437 | 20608 | |
| Scgb1b27 | 0.96277 | 1.9194 | 20609 | |
| Hoxa4 | 0.96284 | 0.8588 | 20610 | |
| Grin2a | 0.96285 | 0.5435 | 20611 | |
| Gnat3 | 0.96287 | 2.8911 | 20612 | |
| Ces1f | 0.96291 | 1.1407 | 20613 | |
| Fbxl6 | 0.96296 | 0.39736 | 20614 | |
| Zfp169 | 0.96297 | 1.1033 | 20615 | |
| Napb | 0.96298 | 2.3738 | 20616 | |
| mmu-mir-542 | 0.96298 | 1.4233 | 20617 | |
| 1700047I17Rik2 | 0.96303 | 0.35707 | 20618 | |
| Zfp189 | 0.96307 | 0.774 | 20619 | |
| Fam46b | 0.9631 | 1.9755 | 20620 | |
| Tmem214 | 0.96311 | 0.57319 | 20621 | |
| Umod | 0.96313 | 0.72504 | 20622 | |
| Mfap3l | 0.96319 | 0.86442 | 20623 | |
| Fabp3 | 0.9634 | 0.30931 | 20624 | |
| Prox1 | 0.96343 | 0.41241 | 20625 | |
| Zfp108 | 0.96349 | 1.7841 | 20626 | |
| Gpr124 | 0.96361 | 0.60784 | 20627 | |
| Gjd3 | 0.96387 | 0.2847 | 20628 | |
| Sprr2a1 | 0.96389 | 1.0818 | 20629 | |
| mmu-mir-5135 | 0.96394 | 0.55679 | 20630 | |
| Wfdc8 | 0.96397 | 0.32835 | 20631 | |
| Olfml1 | 0.96398 | 1.0865 | 20632 | |
| Zfp61 | 0.96399 | 1.0024 | 20633 | |
| Nrg3 | 0.96404 | 0.7837 | 20634 | |
| C2cd2l | 0.96413 | 0.25994 | 20635 | |
| Runx1 | 0.96419 | 0.78678 | 20636 | |
| Apold1 | 0.96422 | 0.84186 | 20637 | |
| Efs | 0.96427 | 1.0325 | 20638 | |
| Pcdhga4 | 0.96431 | 1.042 | 20639 | |
| Selp | 0.96431 | 1.5738 | 20640 | |
| Gabra1 | 0.96433 | 0.19216 | 20641 | |
| Lactbl1 | 0.96436 | 0.98484 | 20642 | |
| Mcf2l | 0.9644 | 1.1718 | 20643 | |
| Nadsyn1 | 0.96449 | 1.6544 | 20644 | |
| mmu-mir-6924 | 0.96458 | -0.051773 | 20645 | |
| Tada1 | 0.96459 | 1.2399 | 20646 | |
| Ms4a1 | 0.96464 | 0.92201 | 20647 | |
| Fgd3 | 0.96466 | 1.34 | 20648 | |
| Sh3gl1 | 0.96467 | 0.27332 | 20649 | |
| Kcns3 | 0.96475 | 0.41186 | 20650 | |
| Gm5592 | 0.96478 | 0.55898 | 20651 | |
| Megf10 | 0.96479 | 0.93679 | 20652 | |
| Zfp672 | 0.96488 | 0.44876 | 20653 | |
| Dupd1 | 0.96495 | 0.20379 | 20654 | |
| Styxl1 | 0.96496 | 1.8741 | 20655 | |
| Gpr114 | 0.96501 | 0.67509 | 20656 | |
| Foxb2 | 0.96507 | 1.1039 | 20657 | |
| Skint8 | 0.96513 | 1.183 | 20658 | |
| Csnka2ip | 0.96514 | 1.125 | 20659 | |
| Kcnv2 | 0.9652 | 1.1078 | 20660 | |
| mmu-mir-450b | 0.96525 | 1.4815 | 20661 | |
| Rnf169 | 0.96526 | 1.6125 | 20662 | |
| Xpc | 0.96527 | 1.156 | 20663 | |
| Grb10 | 0.96536 | 0.84752 | 20664 | |
| Acad11 | 0.96542 | 1.2966 | 20665 | |
| Wnt4 | 0.96556 | 0.6521 | 20666 | |
| Olfr1459 | 0.96566 | 1.6523 | 20667 | |
| Pqlc3 | 0.96569 | 0.35935 | 20668 | |
| Prss42 | 0.96573 | 0.44268 | 20669 | |
| Zfp526 | 0.96575 | 0.93395 | 20670 | |
| Dus2 | 0.96575 | 1.8922 | 20671 | |
| Nme4 | 0.96589 | 0.77313 | 20672 | |
| Grin2b | 0.96592 | 0.28871 | 20673 | |
| Spinkl | 0.96594 | 1.0949 | 20674 | |
| Slitrk4 | 0.96611 | 0.36555 | 20675 | |
| Spata16 | 0.96612 | 1.6127 | 20676 | |
| Sell | 0.96618 | 0.76409 | 20677 | |
| 2310033P09Rik | 0.96623 | 0.70864 | 20678 | |
| Slc15a2 | 0.96629 | 1.4842 | 20679 | |
| Tmem39b | 0.9663 | 0.32509 | 20680 | |
| Kcnab3 | 0.96632 | 1.1172 | 20681 | |
| Cd209a | 0.96633 | 0.33336 | 20682 | |
| Myt1l | 0.96634 | 0.86244 | 20683 | |
| Abca16 | 0.9664 | 0.57755 | 20684 | |
| Pvrl3 | 0.96648 | 1.2318 | 20685 | |
| Gpr83 | 0.96655 | 1.3049 | 20686 | |
| Mpp5 | 0.96659 | 0.73425 | 20687 | |
| Tgif2lx2 | 0.9666 | 1.9193 | 20688 | |
| Gas7 | 0.96667 | 0.33148 | 20689 | |
| Fgr | 0.9667 | 0.23252 | 20690 | |
| Slc25a28 | 0.96671 | 0.54047 | 20691 | |
| Rtp3 | 0.96672 | 0.50605 | 20692 | |
| Efemp2 | 0.96683 | 0.23381 | 20693 | |
| Pfas | 0.96688 | 0.77558 | 20694 | |
| Flrt2 | 0.96695 | 0.29293 | 20695 | |
| Slc6a19 | 0.96697 | 0.91389 | 20696 | |
| 1700034E13Rik | 0.96698 | 1.0878 | 20697 | |
| mmu-mir-107 | 0.96706 | 0.91162 | 20698 | |
| Paqr4 | 0.96724 | 0.77525 | 20699 | |
| Pdhb | 0.96728 | 0.61314 | 20700 | |
| mmu-mir-381 | 0.96731 | 0.48111 | 20701 | |
| Dkkl1 | 0.96739 | 1.6616 | 20702 | |
| Frzb | 0.96744 | 1.0375 | 20703 | |
| Diras1 | 0.96744 | 0.9736 | 20704 | |
| Heg1 | 0.96747 | 0.80269 | 20705 | |
| Npdc1 | 0.9676 | 0.42266 | 20706 | |
| Crygf | 0.96765 | 0.72837 | 20707 | |
| Tmem220 | 0.96766 | 1.0518 | 20708 | |
| Gfra2 | 0.96771 | 0.82089 | 20709 | |
| Kbtbd13 | 0.96785 | 0.66662 | 20710 | |
| Mkl1 | 0.96787 | 0.61204 | 20711 | |
| Fuca2 | 0.96795 | 0.86654 | 20712 | |
| Fam161b | 0.968 | 0.89504 | 20713 | |
| Tmem102 | 0.96814 | 1.2159 | 20714 | |
| C330006A16Rik | 0.96816 | 0.54262 | 20715 | |
| Olfr1446 | 0.96825 | 0.46893 | 20716 | |
| Fancd2 | 0.96827 | 0.91045 | 20717 | |
| Ldb1 | 0.96828 | 0.25948 | 20718 | |
| Myo1h | 0.9683 | 1.098 | 20719 | |
| Cep164 | 0.96835 | 1.0893 | 20720 | |
| Pebp1 | 0.96836 | 1.2647 | 20721 | |
| Sel1l | 0.96841 | 0.48335 | 20722 | |
| Fkbp6 | 0.96845 | 2.2331 | 20723 | |
| Szrd1 | 0.9685 | 0.70842 | 20724 | |
| Zfp444 | 0.96852 | 1.7369 | 20725 | |
| P2rx7 | 0.96865 | 1.4764 | 20726 | |
| Hdlbp | 0.96865 | 0.32416 | 20727 | |
| Pex13 | 0.96871 | 0.44888 | 20728 | |
| Acer1 | 0.96872 | 0.41452 | 20729 | |
| Oard1 | 0.96874 | 0.60218 | 20730 | |
| Adamtsl2 | 0.96875 | 2.1465 | 20731 | |
| Olfr469 | 0.96878 | 0.86932 | 20732 | |
| Arih2 | 0.9688 | 0.94114 | 20733 | |
| 5430421N21Rik | 0.96888 | 0.28519 | 20734 | |
| 2510003E04Rik | 0.96892 | 1.1776 | 20735 | |
| Wnt3 | 0.96896 | 0.36709 | 20736 | |
| Sept6 | 0.96903 | 0.41242 | 20737 | |
| Stbd1 | 0.96908 | 0.93222 | 20738 | |
| Rsph3a | 0.96913 | 1.0003 | 20739 | |
| Ptprm | 0.96923 | 1.2632 | 20740 | |
| Cdyl | 0.96924 | 0.54239 | 20741 | |
| Trp53rk | 0.96925 | 0.82189 | 20742 | |
| Prr9 | 0.96932 | 2.2702 | 20743 | |
| Zswim6 | 0.96933 | 1.512 | 20744 | |
| Rufy4 | 0.96936 | 0.87069 | 20745 | |
| Cyp26b1 | 0.96946 | 0.63538 | 20746 | |
| Kcnn3 | 0.96948 | 1.1754 | 20747 | |
| mmu-mir-485 | 0.96952 | 0.45407 | 20748 | |
| Arhgap33 | 0.96959 | 0.50139 | 20749 | |
| Zfp641 | 0.96963 | 0.84328 | 20750 | |
| Mier3 | 0.96963 | 0.2505 | 20751 | |
| Lrrk1 | 0.96972 | 1.6691 | 20752 | |
| Ttpal | 0.96973 | 0.6735 | 20753 | |
| BC048679 | 0.96979 | 0.72809 | 20754 | |
| Tcam1 | 0.96983 | 0.7989 | 20755 | |
| Zfp82 | 0.96984 | 0.53448 | 20756 | |
| Glo1 | 0.96987 | 0.80947 | 20757 | |
| Clptm1l | 0.96994 | 1.5359 | 20758 | |
| Bdkrb1 | 0.97005 | 0.59663 | 20759 | |
| Mknk2 | 0.97006 | 1.3413 | 20760 | |
| Alkbh2 | 0.97008 | 0.48509 | 20761 | |
| Adamts19 | 0.97009 | 0.45034 | 20762 | |
| Cys1 | 0.97012 | 0.42903 | 20763 | |
| Nynrin | 0.97015 | 1.5417 | 20764 | |
| Sdk2 | 0.97015 | 0.29372 | 20765 | |
| Olfr391-ps | 0.97023 | 1.3805 | 20766 | |
| Satb2 | 0.97024 | 1.8275 | 20767 | |
| Irgq | 0.97025 | 0.45515 | 20768 | |
| Wdfy2 | 0.97028 | 0.44785 | 20769 | |
| Tspyl1 | 0.9703 | 0.45466 | 20770 | |
| Yes1 | 0.97031 | 0.92685 | 20771 | |
| Defb5 | 0.97033 | 1.482 | 20772 | |
| Cc2d2a | 0.97037 | 0.68799 | 20773 | |
| Krt40 | 0.97038 | 1.4927 | 20774 | |
| Esd | 0.97051 | 0.97647 | 20775 | |
| Usp9x | 0.97061 | 0.88443 | 20776 | |
| Chrna4 | 0.97063 | 0.42655 | 20777 | |
| Oaf | 0.97071 | 0.42144 | 20778 | |
| Dok4 | 0.97074 | 2.3647 | 20779 | |
| 3110035E14Rik | 0.97075 | 0.80321 | 20780 | |
| St6galnac3 | 0.97082 | 0.56013 | 20781 | |
| Emc3 | 0.9709 | 1.1145 | 20782 | |
| Tmem236 | 0.97094 | 1.0451 | 20783 | |
| Tesk2 | 0.97094 | 0.60635 | 20784 | |
| Kcnt1 | 0.97096 | 1.1447 | 20785 | |
| Mill1 | 0.97097 | 0.97651 | 20786 | |
| Rsph9 | 0.971 | 0.39419 | 20787 | |
| Snrnp27 | 0.97101 | 0.65932 | 20788 | |
| Capn7 | 0.97111 | 1.3819 | 20789 | |
| Hrc | 0.97117 | 1.4097 | 20790 | |
| Sdccag8 | 0.97127 | 0.66445 | 20791 | |
| Tmem212 | 0.97131 | 1.766 | 20792 | |
| 2810408M09Rik | 0.97131 | 0.70267 | 20793 | |
| Olfr314 | 0.97134 | 0.53208 | 20794 | |
| Vmn1r61 | 0.97136 | 0.81653 | 20795 | |
| C2cd2 | 0.97138 | 1.7257 | 20796 | |
| Dnase1l2 | 0.9714 | 0.79385 | 20797 | |
| Hibadh | 0.97143 | 1.7474 | 20798 | |
| Erc1 | 0.97158 | 0.32555 | 20799 | |
| Cdc37l1 | 0.97159 | 1.5572 | 20800 | |
| 4930547C10Rik | 0.9716 | 1.8099 | 20801 | |
| Asxl1 | 0.97165 | 0.42716 | 20802 | |
| Swsap1 | 0.97174 | 0.31945 | 20803 | |
| Gbx2 | 0.97179 | 1.1352 | 20804 | |
| Topors | 0.97199 | 0.6136 | 20805 | |
| Far2 | 0.97202 | 0.74878 | 20806 | |
| Zmat1 | 0.97203 | 0.3675 | 20807 | |
| Rnf4 | 0.97206 | 0.96044 | 20808 | |
| Setd7 | 0.97238 | 1.0438 | 20809 | |
| Smarcd2 | 0.97239 | 1.1012 | 20810 | |
| Cst12 | 0.9724 | 0.44197 | 20811 | |
| Ffar3 | 0.97242 | 0.64955 | 20812 | |
| Mapk8ip1 | 0.97242 | 1.51 | 20813 | |
| 2510039O18Rik | 0.97245 | 0.57314 | 20814 | |
| Syf2 | 0.97249 | 1.606 | 20815 | |
| Rdh12 | 0.97262 | 0.65 | 20816 | |
| Tmem229a | 0.97266 | 0.42689 | 20817 | |
| Pak7 | 0.97269 | 0.70773 | 20818 | |
| Usp45 | 0.9727 | 1.0762 | 20819 | |
| Cypt14 | 0.97271 | 0.31514 | 20820 | |
| Gm13078 | 0.97287 | 0.2862 | 20821 | |
| Fam196b | 0.97289 | 0.70589 | 20822 | |
| Has1 | 0.97289 | 1.0382 | 20823 | |
| Wisp3 | 0.9729 | 1.4813 | 20824 | |
| Fhl3 | 0.97291 | 1.0705 | 20825 | |
| Cdh9 | 0.97294 | 0.63289 | 20826 | |
| Slc35e4 | 0.97295 | 1.861 | 20827 | |
| Cpne5 | 0.97299 | 1.3965 | 20828 | |
| Mgl2 | 0.97299 | 0.4068 | 20829 | |
| Mtfr2 | 0.97302 | 0.77561 | 20830 | |
| Unc5a | 0.97303 | 1.2645 | 20831 | |
| Hrh4 | 0.97305 | 0.60133 | 20832 | |
| Ndufa4l2 | 0.97305 | 0.48666 | 20833 | |
| Gmnc | 0.9731 | 0.823 | 20834 | |
| Arl14epl | 0.97317 | 1.4802 | 20835 | |
| Lgsn | 0.97323 | 0.4365 | 20836 | |
| Slc22a13 | 0.97328 | 0.56934 | 20837 | |
| Fam117b | 0.9733 | 0.84678 | 20838 | |
| Ogfr | 0.97332 | 1.1132 | 20839 | |
| Smr2 | 0.97335 | 0.95685 | 20840 | |
| Gli3 | 0.97339 | 0.68749 | 20841 | |
| Gm608 | 0.97341 | 1.4849 | 20842 | |
| Hist1h2bk | 0.97341 | 4.0888 | 20843 | |
| Mgme1 | 0.97343 | 0.91465 | 20844 | |
| 1700066B19Rik | 0.97345 | 1.9373 | 20845 | |
| Lyrm9 | 0.97349 | 1.0676 | 20846 | |
| Plcxd2 | 0.9735 | 0.78764 | 20847 | |
| Olfr1196 | 0.97353 | 0.97624 | 20848 | |
| 1110034G24Rik | 0.97354 | 0.61465 | 20849 | |
| Sfxn2 | 0.97366 | 2.3448 | 20850 | |
| Gnrh1 | 0.97368 | 0.89267 | 20851 | |
| Gpr37 | 0.9738 | 0.44755 | 20852 | |
| mmu-let-7d | 0.97381 | 0.64855 | 20853 | |
| Ufd1l | 0.97387 | 0.82098 | 20854 | |
| Ncmap | 0.97389 | 0.64582 | 20855 | |
| Pced1a | 0.97397 | 0.75335 | 20856 | |
| Olfr681 | 0.97416 | 0.5908 | 20857 | |
| Lemd1 | 0.97416 | 0.96197 | 20858 | |
| Clec4e | 0.97421 | 0.7041 | 20859 | |
| Safb | 0.97437 | 1.3026 | 20860 | |
| Obscn | 0.97438 | 1.0848 | 20861 | |
| Smim22 | 0.97439 | 0.65757 | 20862 | |
| Ache | 0.97445 | 1.2712 | 20863 | |
| Plb1 | 0.97448 | 0.48647 | 20864 | |
| Senp1 | 0.9745 | 1.2183 | 20865 | |
| Olfr1412 | 0.97454 | 0.56534 | 20866 | |
| Rev1 | 0.97456 | 0.85893 | 20867 | |
| Npas4 | 0.97458 | 0.63699 | 20868 | |
| Fstl3 | 0.97458 | 0.44803 | 20869 | |
| Tusc3 | 0.97464 | 0.83279 | 20870 | |
| Elk1 | 0.97466 | 0.71763 | 20871 | |
| Arhgef28 | 0.97471 | 0.63033 | 20872 | |
| Prg4 | 0.97476 | 1.0537 | 20873 | |
| Aqp6 | 0.97482 | 0.46786 | 20874 | |
| Mypop | 0.9749 | 0.7087 | 20875 | |
| Cryba4 | 0.9749 | 0.40708 | 20876 | |
| Zfp560 | 0.97496 | 0.57202 | 20877 | |
| Nfil3 | 0.97501 | 1.1133 | 20878 | |
| 4921511M17Rik | 0.97502 | 0.88375 | 20879 | |
| Irf2 | 0.97511 | 0.45441 | 20880 | |
| Olfr1535 | 0.97522 | 0.72079 | 20881 | |
| Usp4 | 0.97524 | 0.82698 | 20882 | |
| Klhl3 | 0.97526 | 0.47153 | 20883 | |
| mmu-mir-6396 | 0.97526 | 0.75444 | 20884 | |
| Rmdn1 | 0.97529 | 1.6704 | 20885 | |
| Zfp646 | 0.97532 | 0.38233 | 20886 | |
| Cbln2 | 0.97535 | 0.50908 | 20887 | |
| Ttc39b | 0.97539 | 0.97629 | 20888 | |
| Gm6760 | 0.97542 | 0.63893 | 20889 | |
| Fam24a | 0.97544 | 0.89929 | 20890 | |
| Klhl29 | 0.97551 | 1.332 | 20891 | |
| Tnfaip6 | 0.97552 | 0.33097 | 20892 | |
| Lmo3 | 0.97554 | 1.0613 | 20893 | |
| Olfr693 | 0.97556 | 1.9753 | 20894 | |
| Il22 | 0.97556 | 0.5225 | 20895 | |
| mmu-mir-3082 | 0.9756 | 0.45415 | 20896 | |
| Igfbp2 | 0.97567 | 0.52812 | 20897 | |
| Hoxb1 | 0.97578 | 0.67724 | 20898 | |
| Stxbp1 | 0.9758 | 1.5277 | 20899 | |
| Mrps36 | 0.97581 | 0.71119 | 20900 | |
| Mmp25 | 0.97589 | 2.1304 | 20901 | |
| Elk4 | 0.97594 | 1.5064 | 20902 | |
| Myg1 | 0.97602 | 0.62436 | 20903 | |
| Mettl17 | 0.97603 | 1.3071 | 20904 | |
| Itgb4 | 0.97604 | 0.80056 | 20905 | |
| Maf1 | 0.97612 | 0.84394 | 20906 | |
| 1700039E15Rik | 0.97614 | 2.1904 | 20907 | |
| E030002O03Rik | 0.97616 | 1.6975 | 20908 | |
| Slc6a7 | 0.97626 | 1.1482 | 20909 | |
| mmu-mir-1933 | 0.97626 | 1.1037 | 20910 | |
| Rho | 0.97627 | 0.7017 | 20911 | |
| Cep97 | 0.97629 | 1.1858 | 20912 | |
| S100a1 | 0.9763 | 1.0997 | 20913 | |
| Catsperb | 0.9764 | 1.4284 | 20914 | |
| Akr7a5 | 0.97642 | 0.48983 | 20915 | |
| Slc44a1 | 0.97644 | 1.1404 | 20916 | |
| Spsb1 | 0.97647 | 0.74363 | 20917 | |
| Cdk2ap1 | 0.9765 | 1.0176 | 20918 | |
| Usp15 | 0.9765 | 1.173 | 20919 | |
| H2bfm | 0.97653 | 1.124 | 20920 | |
| Rras | 0.97658 | 1.887 | 20921 | |
| Lztr1 | 0.9766 | 0.92096 | 20922 | |
| Tusc1 | 0.9767 | 1.2704 | 20923 | |
| Ccnd2 | 0.97671 | 0.94355 | 20924 | |
| H2-D1 | 0.97689 | 1.2764 | 20925 | |
| Olfr699 | 0.97694 | 0.53813 | 20926 | |
| Bag3 | 0.97697 | 1.3989 | 20927 | |
| Spsb2 | 0.97702 | 1.0776 | 20928 | |
| Trim31 | 0.97706 | 0.63873 | 20929 | |
| Olfr1308 | 0.97714 | 0.89488 | 20930 | |
| Pcbp4 | 0.97716 | 1.8252 | 20931 | |
| 2310022B05Rik | 0.97717 | 1.9582 | 20932 | |
| Fgf13 | 0.97718 | 0.65152 | 20933 | |
| Cldn2 | 0.97721 | 1.3973 | 20934 | |
| Thbd | 0.97727 | 2.5576 | 20935 | |
| Rdh14 | 0.97728 | 3.1147 | 20936 | |
| 3830417A13Rik | 0.97733 | 0.92969 | 20937 | |
| Rabep2 | 0.97734 | 0.75076 | 20938 | |
| Olfr1179 | 0.97742 | 1.4936 | 20939 | |
| 4930451C15Rik | 0.97756 | 0.90275 | 20940 | |
| Fndc9 | 0.97763 | 1.1162 | 20941 | |
| Smad4 | 0.97767 | 1.4168 | 20942 | |
| Lzts1 | 0.97779 | 0.73118 | 20943 | |
| Drp2 | 0.97782 | 0.93037 | 20944 | |
| Rgl1 | 0.97783 | 0.31466 | 20945 | |
| Rpgrip1 | 0.97786 | 2.4093 | 20946 | |
| Golga4 | 0.97789 | 2.1017 | 20947 | |
| Hpgds | 0.9779 | 1.1188 | 20948 | |
| Klb | 0.97802 | 1.7997 | 20949 | |
| Degs1 | 0.97803 | 0.50973 | 20950 | |
| Amigo3 | 0.97804 | 0.53375 | 20951 | |
| Mdk | 0.97809 | 0.48743 | 20952 | |
| Dusp16 | 0.97827 | 0.54088 | 20953 | |
| mmu-mir-6384 | 0.97832 | 0.84906 | 20954 | |
| Gm1647 | 0.97833 | 1.7869 | 20955 | |
| Tnfsf11 | 0.9784 | 1.1898 | 20956 | |
| Fshr | 0.97842 | 1.407 | 20957 | |
| mmu-mir-5625 | 0.97851 | 1.5284 | 20958 | |
| Mei1 | 0.97852 | 1.5369 | 20959 | |
| Olfr993 | 0.97874 | 0.83875 | 20960 | |
| Kdm4c | 0.97882 | 1.0197 | 20961 | |
| Ighmbp2 | 0.97886 | 0.74716 | 20962 | |
| Aipl1 | 0.97887 | 0.58159 | 20963 | |
| Hkdc1 | 0.97887 | 0.64932 | 20964 | |
| Olfr935 | 0.97888 | 2.1115 | 20965 | |
| Slc22a1 | 0.97891 | 1.8821 | 20966 | |
| Ict1 | 0.97892 | 1.0391 | 20967 | |
| mmu-mir-344i | 0.97893 | 1.1142 | 20968 | |
| Ghr | 0.97895 | 1.7307 | 20969 | |
| Ssxb6 | 0.979 | 1.6476 | 20970 | |
| Prima1 | 0.97902 | 0.45809 | 20971 | |
| Ckb | 0.97905 | 1.7732 | 20972 | |
| 2610020H08Rik | 0.97907 | 1.117 | 20973 | |
| Zfp868 | 0.97908 | 0.55585 | 20974 | |
| Olfr1055 | 0.97909 | 1.957 | 20975 | |
| Camkmt | 0.97916 | 1.1368 | 20976 | |
| mmu-mir-465a | 0.97917 | 2.4515 | 20977 | |
| mmu-mir-6360 | 0.97919 | 0.98814 | 20978 | |
| Mageb4 | 0.97923 | 1.489 | 20979 | |
| Fam120b | 0.97927 | 0.95494 | 20980 | |
| Wfikkn1 | 0.97928 | 1.5491 | 20981 | |
| Olfr713 | 0.97933 | 1.2853 | 20982 | |
| Skiv2l | 0.97942 | 0.46669 | 20983 | |
| Olfr560 | 0.97947 | 0.46769 | 20984 | |
| Tmem30b | 0.97949 | 1.0896 | 20985 | |
| Gm14420 | 0.97955 | 0.96094 | 20986 | |
| Edem2 | 0.97955 | 0.62808 | 20987 | |
| mmu-mir-378d | 0.97957 | 0.9585 | 20988 | |
| Rusc2 | 0.9796 | 0.44881 | 20989 | |
| Zswim3 | 0.97974 | 1.5805 | 20990 | |
| LOC102308570 | 0.97987 | 2.2347 | 20991 | |
| Arhgdig | 0.97992 | 1.7239 | 20992 | |
| Npr3 | 0.97992 | 1.2092 | 20993 | |
| Rab8a | 0.97994 | 0.6835 | 20994 | |
| Rgcc | 0.97996 | 1.433 | 20995 | |
| Synpo2l | 0.97998 | 1.7282 | 20996 | |
| Mpdz | 0.98004 | 0.77974 | 20997 | |
| Lama4 | 0.98005 | 0.50143 | 20998 | |
| Sis | 0.98011 | 0.98867 | 20999 | |
| Dtx3 | 0.98014 | 0.98956 | 21000 | |
| Arrb2 | 0.98014 | 1.0447 | 21001 | |
| 8030411F24Rik | 0.9802 | 1.6664 | 21002 | |
| Cacna1g | 0.98023 | 0.63111 | 21003 | |
| Nup62cl | 0.98024 | 2.1191 | 21004 | |
| Neto2 | 0.98025 | 0.98336 | 21005 | |
| Rtbdn | 0.98027 | 0.98887 | 21006 | |
| Cldn9 | 0.98032 | 1.0098 | 21007 | |
| Agl | 0.98035 | 0.98782 | 21008 | |
| Fat1 | 0.98036 | 2.0286 | 21009 | |
| Alkbh8 | 0.98039 | 1.2342 | 21010 | |
| Gli1 | 0.98049 | 0.39351 | 21011 | |
| B3galnt2 | 0.98052 | 1.3748 | 21012 | |
| Ddx6 | 0.98057 | 2.3168 | 21013 | |
| mmu-mir-96 | 0.98065 | 0.77053 | 21014 | |
| Pitx3 | 0.98068 | 1.0717 | 21015 | |
| Prb1 | 0.98069 | 0.44306 | 21016 | |
| Scgb3a2 | 0.98069 | 0.71493 | 21017 | |
| Lrrc3 | 0.9807 | 0.62648 | 21018 | |
| Syce2 | 0.98071 | 0.90988 | 21019 | |
| mmu-mir-6354 | 0.98073 | 0.34078 | 21020 | |
| Cldn16 | 0.98075 | 1.429 | 21021 | |
| Kank4 | 0.98079 | 0.95523 | 21022 | |
| Scube2 | 0.98088 | 0.64453 | 21023 | |
| mmu-mir-1981 | 0.98104 | 1.127 | 21024 | |
| Tcea3 | 0.98105 | 1.0665 | 21025 | |
| Stk32c | 0.98108 | 0.48793 | 21026 | |
| Tacr2 | 0.98112 | 2.5849 | 21027 | |
| L1td1 | 0.98114 | 1.3276 | 21028 | |
| Olfr135 | 0.98114 | 1.3843 | 21029 | |
| Lypd6 | 0.98115 | 1.0129 | 21030 | |
| Inmt | 0.98118 | 1.0231 | 21031 | |
| Olfr555 | 0.98119 | 1.2464 | 21032 | |
| Bcap31 | 0.98121 | 1.4854 | 21033 | |
| Olfr508 | 0.98126 | 0.55532 | 21034 | |
| Ero1lb | 0.98127 | 0.46015 | 21035 | |
| Tex11 | 0.98133 | 3.0699 | 21036 | |
| Zfp326 | 0.98138 | 0.95482 | 21037 | |
| Peli3 | 0.9814 | 2.0395 | 21038 | |
| Pmp22 | 0.98141 | 0.96798 | 21039 | |
| Med6 | 0.98143 | 1.3092 | 21040 | |
| Foxs1 | 0.98143 | 0.92472 | 21041 | |
| Olfr1385 | 0.98143 | 0.64265 | 21042 | |
| Usp50 | 0.98164 | 1.7123 | 21043 | |
| Msi1 | 0.98165 | 1.3887 | 21044 | |
| Mgst3 | 0.98171 | 0.8731 | 21045 | |
| Olfr1100 | 0.98172 | 3.0467 | 21046 | |
| Fam35a | 0.98178 | 1.4003 | 21047 | |
| 1700015G11Rik | 0.98178 | 0.77329 | 21048 | |
| Smim19 | 0.9818 | 0.90224 | 21049 | |
| Gulo | 0.98181 | 1.2462 | 21050 | |
| Scarb1 | 0.9819 | 1.474 | 21051 | |
| Dbn1 | 0.98194 | 0.7285 | 21052 | |
| Urod | 0.98196 | 0.92607 | 21053 | |
| Tbrg4 | 0.98201 | 0.6422 | 21054 | |
| mmu-mir-6983 | 0.98202 | 0.43992 | 21055 | |
| Olfr47 | 0.98212 | 1.1554 | 21056 | |
| Il1rl1 | 0.98214 | 0.43457 | 21057 | |
| Mapt | 0.9822 | 0.58586 | 21058 | |
| Zfp7 | 0.98221 | 1.6985 | 21059 | |
| Dennd4b | 0.98222 | 0.4584 | 21060 | |
| H1fx | 0.98226 | 0.60097 | 21061 | |
| Clec12b | 0.98229 | 0.41912 | 21062 | |
| Klra15 | 0.98237 | 0.58488 | 21063 | |
| mmu-mir-377 | 0.98239 | 0.80758 | 21064 | |
| Tmub1 | 0.98243 | 1.8825 | 21065 | |
| Fzd8 | 0.98261 | 0.86977 | 21066 | |
| Spon1 | 0.98265 | 1.7072 | 21067 | |
| Sytl1 | 0.98273 | 1.6243 | 21068 | |
| Gmpr | 0.98274 | 1.3835 | 21069 | |
| C1qtnf1 | 0.98275 | 1.5753 | 21070 | |
| Slc16a12 | 0.98278 | 0.63375 | 21071 | |
| Ddr1 | 0.98285 | 1.1934 | 21072 | |
| Gale | 0.98287 | 1.053 | 21073 | |
| Cntd1 | 0.98287 | 0.89264 | 21074 | |
| Olfr876 | 0.98289 | 0.59532 | 21075 | |
| Olfr149 | 0.98292 | 1.1456 | 21076 | |
| Mlx | 0.98301 | 1.8683 | 21077 | |
| Poglut1 | 0.98313 | 1.5592 | 21078 | |
| Olfr1310 | 0.98318 | 0.87982 | 21079 | |
| Ctsk | 0.98319 | 0.38676 | 21080 | |
| Slc7a3 | 0.98337 | 1.9882 | 21081 | |
| 3110082I17Rik | 0.98344 | 1.0943 | 21082 | |
| Nxpe3 | 0.98352 | 1.4495 | 21083 | |
| Gm4070 | 0.98353 | 1.8822 | 21084 | |
| A730017C20Rik | 0.98354 | 1.4179 | 21085 | |
| Gje1 | 0.98359 | 0.66872 | 21086 | |
| Ovol1 | 0.98359 | 1.1073 | 21087 | |
| Oprd1 | 0.98365 | 0.61964 | 21088 | |
| Gm10229 | 0.98373 | 2.5676 | 21089 | |
| Npvf | 0.98373 | 1.1086 | 21090 | |
| Vegfc | 0.9838 | 0.60231 | 21091 | |
| mmu-mir-546 | 0.98384 | 1.2958 | 21092 | |
| Colec10 | 0.98387 | 1.2045 | 21093 | |
| Rab11fip1 | 0.98391 | 1.2233 | 21094 | |
| Scrn2 | 0.98392 | 1.0994 | 21095 | |
| Lingo3 | 0.98398 | 1.0582 | 21096 | |
| Ppp1r32 | 0.98399 | 0.82428 | 21097 | |
| Tgfbr3 | 0.984 | 1.7869 | 21098 | |
| Apol7b | 0.98402 | 0.90028 | 21099 | |
| 2010003K11Rik | 0.98405 | 0.89551 | 21100 | |
| Tmc4 | 0.98406 | 1.3384 | 21101 | |
| Elovl1 | 0.98407 | 1.3037 | 21102 | |
| Foxa1 | 0.98411 | 2.0718 | 21103 | |
| Mrgpra1 | 0.9842 | 0.84895 | 21104 | |
| BC005624 | 0.98428 | 2.1417 | 21105 | |
| Igtp | 0.98434 | 1.0382 | 21106 | |
| Ubqlnl | 0.9844 | 1.2788 | 21107 | |
| Lrp1b | 0.98445 | 0.95777 | 21108 | |
| Olfr1353 | 0.98447 | 2.3358 | 21109 | |
| Cdh26 | 0.98447 | 0.9001 | 21110 | |
| Ap4s1 | 0.98449 | 1.3464 | 21111 | |
| Ccl21a | 0.98451 | 0.9018 | 21112 | |
| mmu-mir-3062 | 0.98456 | 0.92239 | 21113 | |
| 3110040N11Rik | 0.9846 | 0.65405 | 21114 | |
| Raver2 | 0.98465 | 0.87537 | 21115 | |
| Mc1r | 0.98466 | 1.2284 | 21116 | |
| Speer4f | 0.98469 | 0.48251 | 21117 | |
| Cln3 | 0.98473 | 0.62857 | 21118 | |
| Eif4ebp2 | 0.98478 | 1.8256 | 21119 | |
| Pglyrp4 | 0.98483 | 0.62392 | 21120 | |
| Olfr937 | 0.98483 | 1.0787 | 21121 | |
| Gm14475 | 0.9849 | 2.1943 | 21122 | |
| Pex5l | 0.98493 | 1.3521 | 21123 | |
| Fam181b | 0.98494 | 0.56194 | 21124 | |
| Klc1 | 0.98508 | 0.93923 | 21125 | |
| Higd1c | 0.98508 | 0.69585 | 21126 | |
| Clk2 | 0.98515 | 0.97233 | 21127 | |
| A930009A15Rik | 0.98517 | 1.3561 | 21128 | |
| Olfr948 | 0.9852 | 1.1934 | 21129 | |
| Pm20d1 | 0.98533 | 1.4079 | 21130 | |
| Mei4 | 0.98535 | 1.2076 | 21131 | |
| Smarce1 | 0.98536 | 1.2069 | 21132 | |
| Krtap9-3 | 0.98536 | 1.255 | 21133 | |
| Icam4 | 0.98538 | 1.0496 | 21134 | |
| Robo3 | 0.98548 | 1.0291 | 21135 | |
| mmu-mir-568 | 0.98549 | 0.77232 | 21136 | |
| 4933427D14Rik | 0.98552 | 1.7996 | 21137 | |
| Fbxw24 | 0.98559 | 1.0711 | 21138 | |
| Orm1 | 0.98566 | 0.43376 | 21139 | |
| Tab3 | 0.98573 | 0.49662 | 21140 | |
| Fhod3 | 0.9858 | 1.3766 | 21141 | |
| Pla2g2f | 0.98582 | 0.68327 | 21142 | |
| Enpep | 0.98584 | 0.8904 | 21143 | |
| Pgd | 0.98588 | 0.82242 | 21144 | |
| Wdr11 | 0.98589 | 0.78479 | 21145 | |
| Pms2 | 0.9859 | 0.8994 | 21146 | |
| Meis3 | 0.98593 | 0.554 | 21147 | |
| Olfr298 | 0.98597 | 2.4652 | 21148 | |
| Dnajb8 | 0.98598 | 1.0007 | 21149 | |
| Slc45a1 | 0.98598 | 0.49322 | 21150 | |
| Lefty1 | 0.98604 | 1.8919 | 21151 | |
| Ccdc158 | 0.98604 | 0.45366 | 21152 | |
| Gkn3 | 0.98605 | 0.54301 | 21153 | |
| 2310005G13Rik | 0.98605 | 0.97517 | 21154 | |
| Msln | 0.98607 | 0.74375 | 21155 | |
| Tas2r105 | 0.9861 | 2.1508 | 21156 | |
| Bmp10 | 0.98613 | 1.1855 | 21157 | |
| Mier2 | 0.98613 | 1.4054 | 21158 | |
| Pds5a | 0.98615 | 1.0662 | 21159 | |
| Cd207 | 0.98624 | 1.6016 | 21160 | |
| Ube2dnl2 | 0.98642 | 0.44774 | 21161 | |
| S100z | 0.98644 | 0.92916 | 21162 | |
| Ptpn18 | 0.98654 | 1.3296 | 21163 | |
| Ccdc61 | 0.98658 | 0.94688 | 21164 | |
| Kcnd3 | 0.98661 | 1.3503 | 21165 | |
| 4930595M18Rik | 0.98663 | 0.55862 | 21166 | |
| Dusp10 | 0.98671 | 1.3355 | 21167 | |
| Rbks | 0.98675 | 1.3155 | 21168 | |
| Olfr1226 | 0.98675 | 0.59314 | 21169 | |
| 8-Mar | 0.98681 | 0.60237 | 21170 | |
| Il25 | 0.98684 | 0.6891 | 21171 | |
| Rnf168 | 0.98695 | 0.59543 | 21172 | |
| Cerk | 0.98696 | 0.57015 | 21173 | |
| Pign | 0.98699 | 1.3894 | 21174 | |
| Tmem181a | 0.98709 | 1.7292 | 21175 | |
| Smcp | 0.9871 | 1.3 | 21176 | |
| Sumo3 | 0.98714 | 1.6425 | 21177 | |
| Grik3 | 0.98715 | 0.70632 | 21178 | |
| Syt17 | 0.98722 | 1.7783 | 21179 | |
| Tlx2 | 0.98723 | 0.84321 | 21180 | |
| Bin2 | 0.98729 | 1.2713 | 21181 | |
| 2210016F16Rik | 0.9873 | 1.1799 | 21182 | |
| Tspan5 | 0.9873 | 1.5119 | 21183 | |
| Tmem200b | 0.98735 | 1.3909 | 21184 | |
| mmu-mir-6401 | 0.98742 | 1.2547 | 21185 | |
| 1700123O20Rik | 0.98742 | 1.2376 | 21186 | |
| Git2 | 0.98743 | 1.1547 | 21187 | |
| Fcgrt | 0.98748 | 1.8942 | 21188 | |
| 2410137M14Rik | 0.98754 | 1.5563 | 21189 | |
| Olfr988 | 0.98757 | 0.84059 | 21190 | |
| Mocs1 | 0.98758 | 0.86095 | 21191 | |
| Fam49b | 0.9876 | 1.2669 | 21192 | |
| Add1 | 0.98762 | 0.7408 | 21193 | |
| Akap7 | 0.98764 | 1.0129 | 21194 | |
| Crtc1 | 0.98771 | 0.52274 | 21195 | |
| Ccdc177 | 0.98776 | 0.63937 | 21196 | |
| Ralb | 0.98794 | 0.93459 | 21197 | |
| Lbh | 0.98796 | 0.78856 | 21198 | |
| Adam29 | 0.98798 | 0.86553 | 21199 | |
| Tmem136 | 0.98799 | 0.82448 | 21200 | |
| Adamts5 | 0.988 | 0.94812 | 21201 | |
| mmu-mir-6244 | 0.98803 | 1.1119 | 21202 | |
| Smpdl3a | 0.98807 | 1.2269 | 21203 | |
| 1700024P04Rik | 0.98809 | 0.49464 | 21204 | |
| Scaf1 | 0.98821 | 3.0159 | 21205 | |
| Prx | 0.98825 | 1.1416 | 21206 | |
| Vipas39 | 0.98829 | 0.90585 | 21207 | |
| Olfr539 | 0.98831 | 0.55018 | 21208 | |
| Alppl2 | 0.98842 | 0.72862 | 21209 | |
| Otop2 | 0.98843 | 1.8231 | 21210 | |
| 4632428N05Rik | 0.98845 | 1.2524 | 21211 | |
| Tulp1 | 0.98851 | 1.7495 | 21212 | |
| Zic3 | 0.98852 | 0.69904 | 21213 | |
| Hira | 0.98853 | 1.2309 | 21214 | |
| Zfp202 | 0.9886 | 0.84162 | 21215 | |
| Slc39a9 | 0.98861 | 1.8741 | 21216 | |
| Zbtb9 | 0.98862 | 0.94752 | 21217 | |
| Trp53tg5 | 0.98863 | 1.5144 | 21218 | |
| Tmem184b | 0.98864 | 0.52354 | 21219 | |
| Dgkq | 0.98864 | 1.358 | 21220 | |
| 2010107E04Rik | 0.98868 | 0.50952 | 21221 | |
| Gm7714 | 0.98869 | 0.699 | 21222 | |
| Tspan3 | 0.98875 | 1.9387 | 21223 | |
| Sgpp1 | 0.98883 | 2.7459 | 21224 | |
| Tas2r118 | 0.98885 | 1.0732 | 21225 | |
| Ppp6r1 | 0.98887 | 1.1028 | 21226 | |
| Leprel4 | 0.98891 | 0.92908 | 21227 | |
| Gfi1 | 0.98892 | 1.5585 | 21228 | |
| Fam13c | 0.98894 | 1.5883 | 21229 | |
| Olfr1014 | 0.989 | 1.003 | 21230 | |
| 4930415L06Rik | 0.98903 | 0.71221 | 21231 | |
| Lmo2 | 0.9891 | 0.73463 | 21232 | |
| Pdap1 | 0.98913 | 1.1392 | 21233 | |
| Cox7a2l | 0.98917 | 1.6709 | 21234 | |
| Ppwd1 | 0.9893 | 2.7485 | 21235 | |
| Kcnmb2 | 0.98933 | 1.4762 | 21236 | |
| Frem2 | 0.98937 | 1.1234 | 21237 | |
| Olfr1475 | 0.98949 | 0.56863 | 21238 | |
| Prune | 0.98952 | 2.2546 | 21239 | |
| Slc19a1 | 0.98962 | 0.55748 | 21240 | |
| Gm7325 | 0.98962 | 0.82833 | 21241 | |
| Vmn1r60 | 0.98963 | 1.0822 | 21242 | |
| Aqp12 | 0.98968 | 0.96651 | 21243 | |
| Zscan22 | 0.98972 | 0.59124 | 21244 | |
| Atg4c | 0.98974 | 1.0937 | 21245 | |
| mmu-mir-6993 | 0.98977 | 1.06 | 21246 | |
| 1110008P14Rik | 0.98977 | 1.0449 | 21247 | |
| Coprs | 0.98981 | 0.54449 | 21248 | |
| Cbfa2t3 | 0.98985 | 1.8207 | 21249 | |
| Cdyl2 | 0.98994 | 0.68019 | 21250 | |
| Piezo1 | 0.98995 | 0.72227 | 21251 | |
| Cyba | 0.98996 | 1.6606 | 21252 | |
| Gabbr2 | 0.99005 | 0.99123 | 21253 | |
| Btnl1 | 0.99013 | 0.84106 | 21254 | |
| Ap3d1 | 0.99022 | 1.56 | 21255 | |
| Phf21b | 0.99022 | 0.96158 | 21256 | |
| Samd7 | 0.99025 | 1.1541 | 21257 | |
| Tdp2 | 0.99028 | 2.0789 | 21258 | |
| D17Wsu104e | 0.99038 | 0.9542 | 21259 | |
| Upf3a | 0.99044 | 0.9246 | 21260 | |
| Aldoart1 | 0.99045 | 2.8638 | 21261 | |
| Abcc8 | 0.99055 | 0.70584 | 21262 | |
| Tagln | 0.99062 | 1.8373 | 21263 | |
| Rftn1 | 0.99064 | 0.64576 | 21264 | |
| Plxnb1 | 0.99079 | 2.1119 | 21265 | |
| Nadk2 | 0.9908 | 1.256 | 21266 | |
| Cited2 | 0.99081 | 2.0507 | 21267 | |
| Klhl18 | 0.99082 | 0.75914 | 21268 | |
| Ccdc129 | 0.99087 | 0.94961 | 21269 | |
| Lox | 0.99088 | 0.98284 | 21270 | |
| Olfr1391 | 0.99088 | 0.68262 | 21271 | |
| Gpr165 | 0.99089 | 0.93748 | 21272 | |
| Cysltr2 | 0.99103 | 1.5752 | 21273 | |
| Smok2a | 0.9912 | 1.0075 | 21274 | |
| Cyp2c66 | 0.99121 | 1.1786 | 21275 | |
| Cog6 | 0.99128 | 1.4417 | 21276 | |
| Chgb | 0.9913 | 1.7883 | 21277 | |
| Kif13b | 0.99132 | 1.3507 | 21278 | |
| mmu-mir-1903 | 0.99139 | 1.1148 | 21279 | |
| Qsox2 | 0.99148 | 1.1233 | 21280 | |
| Chid1 | 0.99156 | 1.0214 | 21281 | |
| Defb46 | 0.99156 | 0.68756 | 21282 | |
| 2210013O21Rik | 0.99159 | 1.152 | 21283 | |
| L3mbtl4 | 0.99166 | 1.8049 | 21284 | |
| Supt7l | 0.99168 | 2.4154 | 21285 | |
| Abi3 | 0.99171 | 2.214 | 21286 | |
| Tmem179 | 0.99175 | 1.0075 | 21287 | |
| Gm5114 | 0.99182 | 1.0451 | 21288 | |
| Myo5a | 0.99186 | 0.70749 | 21289 | |
| Baiap2l1 | 0.9919 | 1.1518 | 21290 | |
| Aym1 | 0.9919 | 0.96747 | 21291 | |
| Slc13a5 | 0.99192 | 2.7012 | 21292 | |
| 4930428D18Rik | 0.99199 | 0.96829 | 21293 | |
| Sertad1 | 0.99201 | 1.7765 | 21294 | |
| Sgip1 | 0.99201 | 1.2325 | 21295 | |
| Syt6 | 0.99205 | 0.69372 | 21296 | |
| Tha1 | 0.99206 | 1.5747 | 21297 | |
| Pard6b | 0.99206 | 1.2673 | 21298 | |
| Akp3 | 0.99207 | 1.2165 | 21299 | |
| Vsig2 | 0.99209 | 1.2633 | 21300 | |
| Eya2 | 0.99212 | 1.5631 | 21301 | |
| Iqgap1 | 0.99214 | 1.2543 | 21302 | |
| Efna4 | 0.99216 | 0.80289 | 21303 | |
| Kcnq4 | 0.99218 | 2.0022 | 21304 | |
| Lepre1 | 0.99221 | 1.484 | 21305 | |
| Trim62 | 0.99227 | 1.6528 | 21306 | |
| Ldoc1l | 0.99228 | 1.2429 | 21307 | |
| Zfp719 | 0.99234 | 1.0892 | 21308 | |
| Greb1 | 0.99236 | 1.335 | 21309 | |
| Dpysl2 | 0.99237 | 1.7946 | 21310 | |
| mmu-mir-669l | 0.99254 | 0.97419 | 21311 | |
| Lcn3 | 0.99255 | 0.74999 | 21312 | |
| Ube2cbp | 0.99257 | 2.0399 | 21313 | |
| Fam203a | 0.99259 | 1.2501 | 21314 | |
| Brd9 | 0.99261 | 2.749 | 21315 | |
| mmu-mir-878 | 0.99269 | 1.2143 | 21316 | |
| Magoh | 0.99274 | 1.7266 | 21317 | |
| Fbxl5 | 0.99276 | 0.98149 | 21318 | |
| Parp6 | 0.99286 | 1.0378 | 21319 | |
| Il1rn | 0.99287 | 1.8592 | 21320 | |
| Ctu1 | 0.99291 | 1.6186 | 21321 | |
| mmu-mir-291a | 0.993 | 0.69287 | 21322 | |
| Pnlip | 0.99302 | 2.0331 | 21323 | |
| Apob | 0.99303 | 1.633 | 21324 | |
| mmu-mir-6366 | 0.9931 | 1.0631 | 21325 | |
| Efcab4a | 0.9931 | 0.91111 | 21326 | |
| Ifna13 | 0.9931 | 1.9228 | 21327 | |
| Osbpl10 | 0.99314 | 0.91543 | 21328 | |
| Tmem259 | 0.99324 | 2.2075 | 21329 | |
| Drd1a | 0.99328 | 2.3843 | 21330 | |
| Rxfp2 | 0.99332 | 1.6947 | 21331 | |
| Gipc2 | 0.99334 | 0.82503 | 21332 | |
| Slc27a4 | 0.99337 | 1.353 | 21333 | |
| Prr18 | 0.99338 | 1.5111 | 21334 | |
| Nfxl1 | 0.99339 | 2.6894 | 21335 | |
| Decr2 | 0.99343 | 0.83091 | 21336 | |
| 2610305D13Rik | 0.99345 | 1.6122 | 21337 | |
| Oas1c | 0.99352 | 1.5848 | 21338 | |
| Tcf3 | 0.99363 | 2.0654 | 21339 | |
| Rpusd3 | 0.9937 | 1.8334 | 21340 | |
| Tpbg | 0.9937 | 1.5946 | 21341 | |
| Map3k5 | 0.99372 | 1.1693 | 21342 | |
| Gm266 | 0.99376 | 1.2838 | 21343 | |
| Magee2 | 0.99379 | 1.1387 | 21344 | |
| Hck | 0.99381 | 1.5279 | 21345 | |
| Stim2 | 0.99384 | 1.3508 | 21346 | |
| Prss55 | 0.99387 | 0.94242 | 21347 | |
| Pycard | 0.99406 | 0.87018 | 21348 | |
| Ldlrad3 | 0.99408 | 0.98169 | 21349 | |
| Zfp57 | 0.99409 | 1.9251 | 21350 | |
| Pdk3 | 0.99415 | 1.6199 | 21351 | |
| Syngr3 | 0.99415 | 2.0815 | 21352 | |
| Trim66 | 0.99418 | 0.772 | 21353 | |
| L1cam | 0.99419 | 1.3716 | 21354 | |
| Mogat2 | 0.99428 | 1.0325 | 21355 | |
| Camk2g | 0.99429 | 0.94211 | 21356 | |
| Mtch1 | 0.9944 | 1.5775 | 21357 | |
| Actl6b | 0.99441 | 2.3889 | 21358 | |
| mmu-mir-3961 | 0.99442 | 1.8477 | 21359 | |
| Fn3krp | 0.99449 | 0.99677 | 21360 | |
| Il9 | 0.99454 | 0.93312 | 21361 | |
| Tmem171 | 0.99465 | 2.0019 | 21362 | |
| Vmn1r129 | 0.99471 | 3.5167 | 21363 | |
| Mib1 | 0.99472 | 1.2656 | 21364 | |
| Ttc36 | 0.99481 | 0.91486 | 21365 | |
| Trim7 | 0.99491 | 0.86911 | 21366 | |
| Ppp1r37 | 0.99494 | 1.8807 | 21367 | |
| Epb4.1l4b | 0.99496 | 1.8533 | 21368 | |
| Svopl | 0.99511 | 1.2203 | 21369 | |
| Nucks1 | 0.99519 | 1.1129 | 21370 | |
| Lxn | 0.99519 | 1.8026 | 21371 | |
| 9530077C05Rik | 0.99524 | 1.6998 | 21372 | |
| Mfap5 | 0.99544 | 1.0503 | 21373 | |
| Dbpht2 | 0.99546 | 1.298 | 21374 | |
| Slc24a5 | 0.9956 | 2.0581 | 21375 | |
| Tmem255b | 0.99564 | 1.2569 | 21376 | |
| Mtus1 | 0.99568 | 2.5523 | 21377 | |
| Vmn2r53 | 0.99571 | 2.5583 | 21378 | |
| 3110057O12Rik | 0.99579 | 0.86811 | 21379 | |
| Mapk12 | 0.99585 | 1.3483 | 21380 | |
| Cc2d1a | 0.99588 | 1.7037 | 21381 | |
| Olfr307 | 0.9959 | 0.96044 | 21382 |  |
| Gm94 | 0.99593 | 1.1062 | 21383 |  |
| Catsperd | 0.99594 | 2.7334 | 21384 |  |
| Ociad1 | 0.99596 | 1.4888 | 21385 |  |
| Angptl4 | 0.99599 | 1.0565 | 21386 |  |
| mmu-mir-21b | 0.99607 | 1.2418 | 21387 |  |
| Zfp955a | 0.99625 | 1.8642 | 21388 |  |
| Zfy1 | 0.99627 | 1.0118 | 21389 |  |
| Pik3cd | 0.99628 | 0.92017 | 21390 |  |
| Csf2ra | 0.99634 | 0.91684 | 21391 |  |
| Tmem194 | 0.99641 | 1.4762 | 21392 |  |
| Ybx3 | 0.99641 | 1.3758 | 21393 |  |
| Arhgap4 | 0.99643 | 1.3674 | 21394 |  |
| Mospd2 | 0.99645 | 2.853 | 21395 |  |
| Olfr883 | 0.99653 | 3.052 | 21396 |  |
| Gucy2f | 0.99661 | 3.7548 | 21397 |  |
| Bola2 | 0.99662 | 1.0905 | 21398 |  |
| Vmac | 0.99664 | 0.94208 | 21399 |  |
| Olfr412 | 0.99667 | 1.1962 | 21400 |  |
| Pskh1 | 0.99668 | 0.84645 | 21401 |  |
| Cpt2 | 0.99674 | 0.91711 | 21402 |  |
| Txlnb | 0.99679 | 1.1878 | 21403 |  |
| Mecp2 | 0.99682 | 2.6097 | 21404 |  |
| Kirrel | 0.99686 | 1.6796 | 21405 |  |
| Asb4 | 0.99689 | 0.90853 | 21406 |  |
| Tmem87b | 0.99692 | 0.97849 | 21407 |  |
| Acadl | 0.99693 | 1.0439 | 21408 |  |
| Gm13157 | 0.99696 | 1.1238 | 21409 |  |
| Jak3 | 0.99709 | 1.4384 | 21410 |  |
| Tbl3 | 0.99713 | 2.1861 | 21411 |  |
| Olfr816 | 0.99716 | 2.5487 | 21412 |  |
| Coch | 0.99727 | 0.92969 | 21413 |  |
| Defb23 | 0.99728 | 2.6908 | 21414 |  |
| Gng4 | 0.99731 | 0.94116 | 21415 |  |
| Akr1c20 | 0.99733 | 1.2084 | 21416 |  |
| Vmn2r95 | 0.99735 | 1.2296 | 21417 |  |
| Ddx39 | 0.99737 | 1.0901 | 21418 |  |
| mmu-mir-7091 | 0.99738 | 1.029 | 21419 |  |
| Fam78b | 0.9974 | 1.5015 | 21420 |  |
| Ephb6 | 0.99743 | 1.5502 | 21421 |  |
| Tssk1 | 0.99744 | 0.96025 | 21422 |  |
| Gpr65 | 0.99744 | 1.2366 | 21423 |  |
| Gstm1 | 0.99756 | 1.0102 | 21424 |  |
| Vax2 | 0.99756 | 1.1972 | 21425 |  |
| mmu-mir-704 | 0.99759 | 1.0957 | 21426 |  |
| Ift80 | 0.9976 | 1.2273 | 21427 |  |
| Usp33 | 0.99766 | 2.0122 | 21428 |  |
| Slc2a4 | 0.99768 | 1.1646 | 21429 |  |
| Cyp2b10 | 0.99775 | 2.3925 | 21430 |  |
| Syngr2 | 0.99776 | 2.1966 | 21431 |  |
| Mras | 0.99777 | 1.0883 | 21432 |  |
| Zfp960 | 0.9978 | 2.7591 | 21433 |  |
| Psg26 | 0.99781 | 2.2437 | 21434 |  |
| mmu-mir-1197 | 0.99788 | 1.1133 | 21435 |  |
| Wdfy4 | 0.99788 | 3.4733 | 21436 |  |
| Ergic1 | 0.99794 | 1.4365 | 21437 |  |
| Tex2 | 0.99796 | 2.8295 | 21438 |  |
| Hes3 | 0.998 | 1.3013 | 21439 |  |
| Aktip | 0.998 | 1.7889 | 21440 |  |
| Olfr1010 | 0.99806 | 1.3529 | 21441 |  |
| 9830107B12Rik | 0.99808 | 2.6412 | 21442 |  |
| Mrps33 | 0.99811 | 1.1636 | 21443 |  |
| mmu-mir-221 | 0.99811 | 0.81676 | 21444 |  |
| Dnah10 | 0.99814 | 1.5721 | 21445 |  |
| Tlr8 | 0.99816 | 1.8135 | 21446 |  |
| Rbm3 | 0.9982 | 4.0727 | 21447 |  |
| Olfr1269 | 0.99822 | 1.2211 | 21448 |  |
| Oaz1 | 0.99822 | 1.3592 | 21449 |  |
| Ylpm1 | 0.99825 | 1.8157 | 21450 |  |
| Themis3 | 0.99838 | 1.1901 | 21451 |  |
| Mnx1 | 0.99843 | 1.3476 | 21452 |  |
| Fcnb | 0.99848 | 1.952 | 21453 |  |
| Olfr653 | 0.99849 | 1.521 | 21454 |  |
| Hoxa13 | 0.9985 | 1.4706 | 21455 |  |
| Ccdc78 | 0.99862 | 2.7823 | 21456 |  |
| Krtap10-4 | 0.99865 | 1.3845 | 21457 |  |
| Pabpc2 | 0.9987 | 1.4375 | 21458 |  |
| mmu-mir-6992 | 0.99873 | 1.1055 | 21459 |  |
| Dhrs7 | 0.99898 | 1.2438 | 21460 |  |
| Vmn1r57 | 0.99901 | 1.3318 | 21461 |  |
| Dtx3l | 0.99905 | 2.5155 | 21462 |  |
| mmu-mir-719 | 0.99913 | 2.1487 | 21463 |  |
| Olfr145 | 0.99916 | 2.0058 | 21464 |  |
| Abhd11 | 0.99927 | 2.105 | 21465 |  |
| Nrp2 | 0.9993 | 1.9698 | 21466 |  |
| Fam135a | 0.99931 | 2.401 | 21467 |  |
| Wfdc12 | 0.99934 | 2.4039 | 21468 |  |
| Rin2 | 0.99935 | 1.5424 | 21469 |  |
| Sfrp1 | 0.99935 | 2.1692 | 21470 |  |
| Olfr866 | 0.99939 | 1.7072 | 21471 |  |
| Reg3a | 0.99944 | 1.9326 | 21472 |  |
| Sprr1a | 0.99945 | 1.3635 | 21473 |  |
| Gpm6a | 0.99946 | 1.425 | 21474 |  |
| Acot9 | 0.99951 | 1.3622 | 21475 |  |
| Cpz | 0.99957 | 2.3106 | 21476 |  |
| Tmprss2 | 0.99963 | 1.5707 | 21477 |  |
| Hexim1 | 0.99964 | 2.3826 | 21478 |  |
| Prlh | 0.99968 | 1.6234 | 21479 |  |
| Qtrt1 | 0.99971 | 2.3766 | 21480 |  |
| Cttn | 0.99972 | 1.9088 | 21481 |  |
| Oscar | 0.99974 | 1.9019 | 21482 |  |
| Asb6 | 0.99975 | 2.6508 | 21483 |  |
| S100a4 | 0.99982 | 2.6345 | 21484 |  |
| Zdbf2 | 0.99983 | 1.6751 | 21485 |  |
